# Disrupting Mitochondrial Electron Transfer Chain Complex I Decreases Immune Checkpoints in Murine and Human Acute Myeloid Leukemic Cells

**DOI:** 10.3390/cancers13143499

**Published:** 2021-07-13

**Authors:** Raquel Luna-Yolba, Justine Marmoiton, Véronique Gigo, Xavier Marechal, Emeline Boet, Ambrine Sahal, Nathalie Alet, Ifat Abramovich, Eyal Gottlieb, Virgile Visentin, Michael R. Paillasse, Jean-Emmanuel Sarry

**Affiliations:** 1 EVOTEC, Campus Curie, 31100 Toulouse, France; Raquel.Luna-Yolba@evotec.com (R.L.-Y.); Justine.Marmoiton@evotec.com (J.M.); Veronique.Gigo@evotec.com (V.G.); Xavier.Marechal@evotec.com (X.M.); Nathalie.Alet@evotec.com (N.A.); Virgile.Visentin@evotec.com (V.V.); 2 Centre de Recherches en Cancérologie de Toulouse, Université de Toulouse, Inserm, CNRS, 31100 Toulouse, France; emeline.boet@inserm.fr (E.B.); ambrine.sahal@inserm.fr (A.S.); 3 LabEx Toucan, 31100 Toulouse, France; 4 Equipe Labellisée Ligue Nationale Contre le Cancer 2018, 31100 Toulouse, France; 5 Technion—Israel Institute of Technology, Haifa 32000, Israel; ifat.a@technion.ac.il (I.A.); e.gottlieb@technion.ac.il (E.G.)

**Keywords:** OxPHOS, Immune checkpoints, AML

## Abstract

**Simple Summary:**

Despite all of the advancements made in recent years in the treatment of acute myeloid leukemia (AML), long-term survival is achieved by only 30–40% of AML patients. Thus, new therapeutic strategies are strongly needed. This work confirms the increase in oxidative phosphorylation upon cytarabine resistance in AML murine cells, reinforcing the interest of targeting it. In addition, it identifies a new role of the first complex of the electron transport chain (ETCI) in the regulation of the immune checkpoints PD-L1 and CD39 in murine and human leukemic cells. Thus, this work opens the door to the evaluation of ETCI inhibitors in combination with immunotherapy.

**Abstract:**

Oxidative metabolism is crucial for leukemic stem cell (LSC) function and drug resistance in acute myeloid leukemia (AML). Mitochondrial metabolism also affects the immune system and therefore the anti-tumor response. The modulation of oxidative phosphorylation (OxPHOS) has emerged as a promising approach to improve the therapy outcome for AML patients. However, the effect of mitochondrial inhibitors on the immune compartment in the context of AML is yet to be explored. Immune checkpoints such as ectonucleotidase CD39 and programmed dead ligand 1 (PD-L1) have been reported to be expressed in AML and linked to chemo-resistance and a poor prognosis. In the present study, we first demonstrated that a novel selective electron transfer chain complex (ETC) I inhibitor, EVT-701, decreased the OxPHOS metabolism of murine and human cytarabine (AraC)-resistant leukemic cell lines. Furthermore, we showed that while AraC induced an immune response regulation by increasing CD39 expression and by reinforcing the interferon-γ/PD-L1 axis, EVT-701 reduced CD39 and PD-L1 expression in vitro in a panel of both murine and human AML cell lines, especially upon AraC treatment. Altogether, this work uncovers a non-canonical function of ETCI in controlling CD39 and PD-L1 immune checkpoints, thereby improving the anti-tumor response in AML.

## 1. Introduction

The overall survival in acute myeloid leukemia (AML) patients is poor due to relapses after a usually successful induction therapy [1]. This is due to residual leukemic cells after chemotherapy that regrow, resulting in a relapse [2]. Energy metabolism and redox homeostasis have emerged as hallmarks of carcinogenesis and their role in the response to chemotherapy in cancer cells is now fully acknowledged [3,4]. A large body of evidence links both cancer cell stemness and chemotherapy resistance to OxPHOS metabolism [5,6,7,8,9], rendering OxPHOS inhibitors important therapeutic agents to disrupt tumorigenesis and improve the chemotherapy outcome. Mitochondrial metabolism has been shown to be a promising target to overcome relapses in leukemia. Recent research has shown that mitochondria in AML cells surviving after chemotherapy have a higher transmembrane potential [10,11]. In B-progenitor acute lymphoblastic leukemia (B-ALL), diagnosis relapse initiating clones (dRI) transcriptionally enriched for mitochondrial metabolism are present from the diagnosis [12]. In addition, the direct or indirect inhibition of the electron transport chain (ETC) [13,14,15] or the oxidation of substrates such as fatty acids [16] have proven to be of therapeutic benefit in leukemia, synergizing with AraC or with BCL-2 inhibition [17,18]. One much less studied aspect is the effect of OxPHOS inhibitors in an immunocompetent context, especially in AML, as the vast majority of the studies use NSG mice to allow the study of human cell lines or samples. In other cancer types, metformin has been demonstrated to decrease the expression of immune checkpoint components such as PD-L1 [19], CD39 and CD73 [20] both in cancer and immunosuppressive cell populations, enabling a CD8 T cell cytotoxic response [19,21]. However, due to the pleiotropic effects of metformin, it is not clear if these effects are predominately mediated by ETCI inhibition. EVT-701 is a novel ETCI inhibitor that has shown efficacy in different solid tumor models but its effect on AML has not previously been evaluated [22,23].

In this study, we report that murine leukemic AML cell lines exposed to AraC behave similar to chemo-resistant human AML cells by upregulating OxPHOS to escape chemotherapy, making our murine model translational to the human pathology. We also show that AraC increased the PD-L1 and CD39 immune checkpoint expression at the cell surface membrane. Interestingly, the inhibition of the ETCI complex by EVT-701 decreased the levels of both immune checkpoints in vitro. Altogether, our findings show that disrupting mitochondrial metabolism through the inhibition of ETCI modulates the immune checkpoints PD-L1 and CD39 and may improve the anti-tumor response in AML.

## 2. Materials and Methods

### 2.1. Cell Culture

Leukemic cell lines were maintained in a DMEM medium containing 1 g/L of glucose, 2 mM glutamax (#35050-038), 1X NEAA (#11140-035, Gibco) and 1 mM sodium pyruvate (#11360-039), all acquired from ThermoFisher Scientific (Illkirch Graffenstaden, France) and 10% fetal bovine serum (#F7524, Sigma-Aldrich, Saint Quentin Fallavier, France). The cells were incubated at 37 °C with 5% CO_2_ and split every 3 days to maintain an exponential growth phase. The cells were purchased from the American Type Culture Collection (Rockville, MD, USA). Table 1 summarizes the clinical and mutational features of the leukemic cell lines used in this study.

### 2.2. IncuCyte Assay

The cells were seeded in triplicate in 384-well ViewPlates (#6007480, Perking Elmer, Villebon sur Yvette, France) at a density of 1000 cells/well. After an overnight, the cells were stimulated with the compounds and placed into the IncuCyte Zoom (Ozyme, Saint-Cyr-l’Ecole, France) reader until the end of the experiment (5 days after). Images were taken every 3 h. The confluence for each condition was analyzed using the masks of the IncuCyte Zoom software.

### 2.3. TMRE and MTDR Assay

The cells were seeded in fresh media 24 h prior to FACs experiments. To prepare the samples, the cells were washed in PBS and stained to detect the live cells with violet live dead staining (#L34955, Thermo Fisher). Tetramethylrhodamine, ethyl ester (TMRE) (#T669, Thermo Fisher Scientific) was then added for the characterization of the mitochondrial membrane potential and MitoTracker Deep Red FM (#M22426, Thermo Fisher Scientific) was used for the assessment of the mitochondrial mass. After incubation at 37 °C for 20 min, the cells were washed, resuspended in a FACs buffer and analyzed in a FACs Canto (BD Biosciences, Le Pont De Claix, France). FCCP (Sigma-Aldrich) was added as a control for specific mitochondrial membrane potential staining.

### 2.4. ATP Content Assay

The ATP cell content was measured using Cell Titer Glo (#G7571, Promega, Charbonnières Les Bains, France). In summary, the cells were seeded at 2000 cells/well in three replicates in 384-well ViewPlates (Perking Elmer). After an overnight, oligomycin A (Sigma-Aldrich) and sodium iodoacetate (Sigma-Aldrich) were added, either both or in combination. Following a 1 h incubation, the cell confluence was assessed with IncuCyte for further normalization and 40 μL of Cell Titer Glo reaction mix was added to each well, reaching a final volume of 80 μL. The plates were then analyzed for luminescence with an EnVision 2105 Multimode Plate Reader (Perkin Elmer). By comparing the different conditions, the global ATP and the percentages of both glycolytic and mitochondrial ATP were determined.

### 2.5. Cell Proliferation

The cell proliferation was calculated by measuring the number of viable cells with trypan blue dye and Nexcelom Bioscience Cellometer^TM^ Auto T4 (Ozyme) or staining the cells with live fixable staining (#L34955, Thermo Fisher Scientific) and adding counting beads (#C36950, Thermo Fisher Scientific) for a further analysis with a BD LSR Fortessa™ cell analyzer and FlowJo™ software (Version 10, Becton, Dickinson and Company, Ashland, OR, USA).

### 2.6. Seahorse

The cells were incubated overnight with the vehicle or with EVT-701. Following the incubation time, the cells were suspended in pre-warmed Seahorse XF media (Agilent, Les Ulis, France) containing 10 mM glucose, 2 mM glutamine and 1 mM pyruvate. A total of 10^5^ cells were seeded per well of Seahorse 96-well plates, previously coated with Cell-tak (#10317081, Thermo Fisher Scientific). The Mito Stress Test was performed with 1–3 µM oligomycin, 1–10 µM FCCP (both optimized for each cell line), 50 µM antimycin/rotenone and 50 mM 2-DG. The OCR and ECAR values were normalized to a cell confluence percentage with IncuCyte.

To assess the restoration of oxygen consumption by succinate in EVT-treated cells, 10^5^ cells were seeded per well in replicates of five. After plate centrifugation, the Seahorse medium was replaced by a pre-warmed mitochondria assay solution (MAS) medium containing 10 mM pyruvate, 4 mM ADP and 2 mM malate, prepared as described by Agilent (https://www.agilent.com/cs/library/usermanuals/public/insert-xf-pmp-reagent-web.pdf, 23 January 2020). A total of 10 mM of succinate was added to the wells where the bypass of the ETCI blockade was assessed. Plasma membrane permeabilizer (PMP, Agilent) was added just before the introduction of the plates into the Seahorse reader. Unless otherwise mentioned, all reagents were bought from Sigma-Aldrich.

### 2.7. Western Blot

The cells were lysed with a RIPAS buffer (#98065, Cell Signaling Technology, Ozyme) containing a protease inhibitor cocktail (#5872S, Cell Signaling Technology, Ozyme). The proteins were quantified by the Pierce BCA protein assay kit (#23227, Thermo Fisher Scientific) and the protein concentration for each sample was adjusted to 500 µg/mL by adding a loading buffer containing DTT or β-mercaptoethanol. Once reduced, the samples were heated to 55 °C to complete the protein denaturalization without affecting the thermosensitive band of the SDHB (ETCII) for the assessment of the ETC components or to 95 °C for 5 min for the assessment of STAT and p-Stat1. The samples were resolved in 4–20% Criterion™ TGX™ Precast Midi Protein Gels from Biorad (#5671093, Marnes-la-Coquette, France) by electrophoresis, transferred to nitrocellulose membranes and incubated with a rodent OxPHOS antibody cocktail (#ab110413) from Abcam (Amsterdam, The Netherlands) used at a dilution of 1:250, a STAT1 antibody (#9172S, Cell Signaling) used at a dilution of 1:1000, Phospho-Stat1 (Tyr701) (#9167S, Cell Signaling) used at 1:1000 or actin (#A2228, Sigma-Aldrich) diluted 1:20,000. An incubation with the secondary antibody goat anti-mouse IgG-HRP (#7076, Cell Signaling Technology, Ozyme) or anti-rabbit IgG HRP-linked antibody (#7074, Cell Signaling) and the further addition of a SuperSignal West Pico Chemiluminescent Substrate (#34580, Thermo Fisher Scientific) then revealed the protein bands in the membranes. Images were captured with Syngene PXi and quantified with GeneTools from Syngene (Thermo Fisher Scientific).

### 2.8. Data Sets and the Gene Set Enrichment Analysis (GSEA)

All publicly accessible transcriptomic data sets used in this study are listed below:

- TCGA: [24]
- GSE12417: [25]
- GSE97631: [10]
- MOLM14 treated with metformin: GSE97346.

GSEA was performed using DESEQ2 and fGSEA R packages. Gene sets were downloaded from the GSEA website (https://www.gsea-msigdb.org/gsea/msigdb/, 21 November. 2020) and the Farge_High OxPHOS signature from [10]. Volcano plots and enrichment maps were used for the visualization of the GSEA results. For each gene signature, its normalized enrichment score (NES) and false discovery rate (FDR) considering the p-adjusted values (padj) were evaluated.

### 2.9. Metabolomics

A total of four replicates of 7 × 10^5^ cells/mL were seeded in 6-well plates per experiment. The cells were stimulated with EVT-701 or the vehicle. After a 24 h incubation with the compounds, 10 µL of media were sampled for metabolic extraction while the remaining media were discarded and the cells were washed with PBS. Metabolite extractions were performed by adding 1 mL of a cold extraction mix consisting of methanol (#1060351000, Sigma-Aldrich), acetonitrile (#1000291000, Sigma-Aldrich) and water (#1153331000, Sigma-Aldrich) in a proportion of 5:3:2. All solvents were LC-MS grade. The samples were vortexed for 10 min at 4 °C and then immediately centrifuged at the maximum speed for 10 min at 4 °C. Clear supernatants were transferred to Eppendorf tubes and kept at −80 °C until shipping to the Technion—Israel Institute of Technology for an LC-MS analysis. The LC-MS metabolomics analysis was performed as described previously [26]. Briefly, a Thermo Ultimate 3000 high-performance liquid chromatography (HPLC) system coupled to a Q-Exactive Orbitrap Mass Spectrometer (Thermo Fisher Scientific) was used with a resolution of 35,000 at 200 mass/charge ratio (*m*/*z*), electrospray ionization and polarity switching mode to enable both positive and negative ions across a mass range of 67 to 1000 *m*/*z*. The HPLC setup consisted of a ZIC-pHILIC column (SeQuant; 150 mm × 2.1 mm, 5 μm; Merck) with a ZIC-pHILIC guard column (SeQuant; 20 mm × 2.1 mm). A total of 5 µL of the metabolite extracts were injected and the compounds were separated with a mobile phase gradient of 15 min, starting at 20% aqueous (20 mM ammonium carbonate adjusted to pH 0.2 with 0.1% of 25% ammonium hydroxide) and 80% organic (acetonitrile) and terminated with 20% acetonitrile. The flow rate and column temperature were maintained at 0.2 mL/min and 45 °C, respectively, for a total run time of 27 min. All metabolites were detected using mass accuracy below 5 ppm. Thermo Xcalibur was used for the data acquisition. The analyses were performed with TraceFinder 4.1 (Thermo Fisher Scientific). The peak areas of the metabolites were determined by using the exact mass of the singly charged ions. The retention time of the metabolites was predetermined on the pHILIC column by analyzing an in-house mass spectrometry metabolite library that was built by running commercially available standards. The cell number was used for data normalization.

### 2.10. Transcriptomics

The cells were seeded in 20 µL at a density of 7500 cells/well in 384-well plates (#3542, Corning, Boulogne-Billancourt, France) and treated with different doses and combinations of AraC (#PHR1787, Sigma-Aldrich, St. Louis, MO, USA), EVT-701 and IACS or DMSO for 24 h. After a 24 h incubation, the plates were centrifuged and the media were replaced by a lysis buffer containing an RNasin^®^ ribonuclease inhibitor (#2511, Promega). The plates with lysates were frozen to −80 °C and sent to EVOTEC Gottingen where RNAseq was performed using Evotec’s proprietary high-throughput platform, ScreenSeq.

Briefly, the plates were thawed and the oligo(dT)-containing oligos were distributed among the wells using an Agilent Bravo automated liquid handling system. Each oligo had a unique barcode specific for the particular well. In addition, there was a stretch of random nucleotides (unique molecular identifiers, UMIs) next to the barcode to uniquely label the original mRNA molecules. After the mRNAs in the wells were barcoded, all of the material from the wells of one plate was pooled together followed by the library preparation. The quality of libraries was controlled using BioAnalyzer. There was one library generated per plate.

The sequencing reaction was performed using a NovaSeq 6000 Illumina platform and paired-end reads covering the gene and the barcoded UMI parts. The raw data were processed to identify the genes, to demultiplex the samples from different wells based on the well barcodes and to identify all original mRNA/cDNA molecules based on the UMIs.

Subsequently, the demultiplexed files were aligned to the reference genome (Mus_musculus.GRCm38.97) using a STAR aligner and a table with counts per gene was obtained (the raw count matrix in the Supplementary Material).

The data analysis was performed with PanHunter (EVOTEC platform), DESEQ2 and fGSEA R packages.

### 2.11. NAD+/NADH Ratio Assessment

The NAD+/NADH ratio was evaluated using NAD/NADH Glo (#G9072, Promega). In summary, 4 × 10^5^ cells/mL were seeded in 96-well plates (Perking Elmer) and stimulated with different doses of EVT-701 in a final volume of 200 μL/well. After the 24 h incubation time, the NAD+/NADH was evaluated as described (https://france.promega.com/-/media/files/resources/protocols/technical-manuals/101/nad-nadh-glo-assay-protocol.pdf?la=en, 15 December 2020). Briefly, a twice-serial 2-fold dilution of the cells was performed before diluting another 2-fold with 1% DTAB to lyse the cells. From each well, 50 μL were transferred to a microtube for NAD+ detection in acidic media (25 μL of 0.4 N HCl were added) or to an empty tube for NADH detection in basic media. The samples were heated at 60 °C for 15 min, then left for 10 min at room temperature. A total of 25 μL of Trizma base were added to the acidic NAD+ sample to neutralize HCl. A total of 50 μL of Trizma-HCl were added to the basic samples containing NADH. From each tube, four replicates of 20 μL were seeded in 384-well plates (Perking Elmer). The NAD/NADH detection reagent was prepared immediately before its addition to the well in a ratio of 1:1 (20 μL/well). After the incubation time (30–40 min) at room temperature, the luminescence was read by using an EnVision 2105 Multimode Plate Reader (Perkin Elmer).

### 2.12. PD-L1 and CD39 Expression Assessment

Murine AML cell lines were seeded in fresh media 72 h prior to the FACs experiment and stimulated with murine IFN-γ at 25 ng/mL (#575306, BioLegend, Ozyme), 1 µM EVT-701, 0.03 µM (L1210) or 0.1 µM (C1498) AraC or a combination of the compounds. For human AML cell lines, the cells were seeded in fresh media 24 h prior to the FACs experiment and stimulated with human 25 ng/mL IFN-γ (#285-IF/CF, R&D Systems, Bio-Techne SAS, Noyal Châtillon sur Seiche, France), 1 µM EVT-701 or 0.3 µM AraC or a combination of the compounds. The cells were then washed in PBS and stained with violet live dead staining (#L34955, Thermo Fisher). The samples were then incubated for 30 min at 4 °C with the corresponding CD39 and PD-L1 antibody mix or the control isotypes (further detailed in Table 2). The cells were then washed and resuspended in a FACs buffer followed by an analysis on BD LSRFortessa™ (murine AML cell lines) or BD FACSCanto™ (human AML cell lines) cytometers and FlowJo™ software. For the NAD supplementation experiments, 0.5 mg/mL NAD+ (#1.24542.0005, Merk) at pH 7 was used. The median fluorescence intensity for each experiment is represented.

### 2.13. Statistical Analyses

GraphPad Prism Software version 8 (La Jolla, CA, USA) was used for the statistical analysis. The results were expressed as a mean ± SD. T-tests were used for the comparison of the two groups whereas a one-way Anova with a Tukey post-test was carried out to compare one variant in > 2 groups or a two-way Anova was used to compare more than one variant in > 2 groups. The significance is represented by stars in which * is *p* < 0.05, ** is *p* < 0.01, *** is *p* < 0.005 and **** is *p* < 0.001.

### 2.14. Graphical Summaries

Graphical schemes were generated using scientific illustration toolkits from Motifolio (Motifolio Inc., PO Box 2040, Ellicott City, MD, USA).

## 3. Results

### 3.1. OxPHOS Phenotype and Mitochondrial Gene Signatures Are Enriched in AML Patients with a Shorter Survival

**Figure 1:**
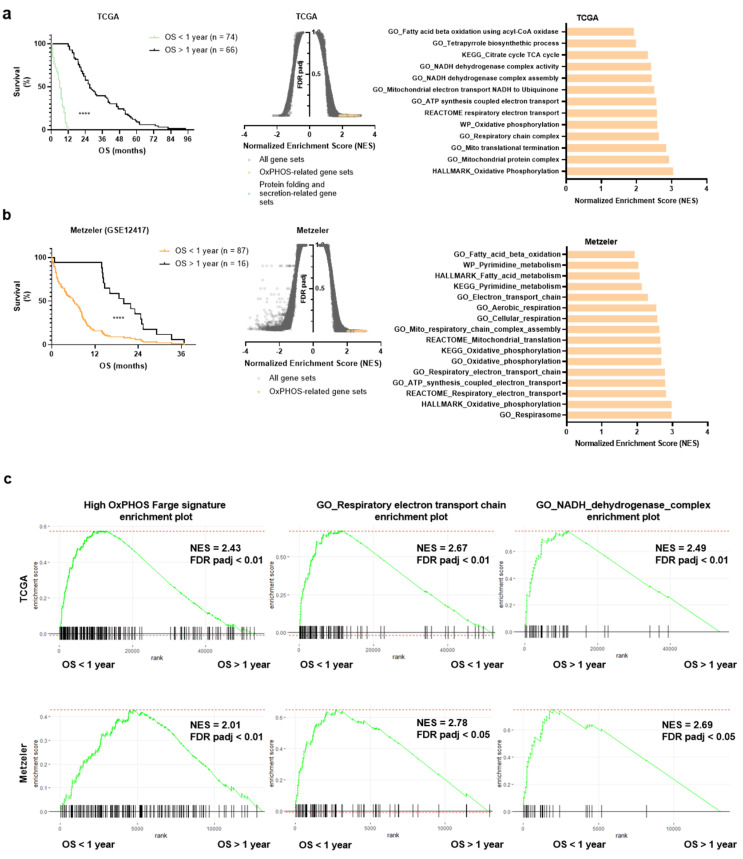
OxPHOS and mitochondrial gene expression is associated with a shorter overall survival in human AML. (**a**) Kaplan–Meier survival curves of the TCGA-AML cohort for patients with a short (OS > 1 year) or long (OS < 1 year) overall survival (**left**). Volcano plot showing GSEA results in the TCGA cohort comparing short vs. long survival (**middle**). OxPHOS-related gene sets enriched in the short survival group (FDR < 0.01) are highlighted in orange and protein folding and secretion-related gene sets are in green. On the (**right**), a plot showing TCGA short vs. long survival OxPHOS-related differentially expressed gene sets (FDR < 0.05) with their normalized enrichment scores (NES). (**b**) On the left, Kaplan–Meier survival curves of the Metzeler cohort for patients with a short (OS > 1 year) or long (OS < 1 year) overall survival. In the middle, the volcano plot showing GSEA results in the Metzeler cohort comparing short vs. long survival. OxPHOS and mitochondria-related gene sets enriched in the short vs. long survival group (FDR < 0.05) are highlighted in orange. On the right, a plot showing short vs. long survival OxPHOS and mitochondria-related differentially expressed gene sets (FDR < 0.05) with their normalized enrichment scores (NES). (**c**) Gene set enrichment plots of the Farge_High OxPHOS gene signature, the GO_respiratory electron transport chain and NADH dehydrogenase complex gene signatures in the TCGA (**left**) and in Metzeler (**right**) cohorts.

The publically available human AML data sets from TCGA-AML [24] and the Metzeler [25] cohort were analyzed to assess whether there was an association between the mitochondrial and OxPHOS gene expression with overall survival. Briefly, each cohort was divided into short or long overall survival (OS > or < to 1 year for both cohorts; Figure 1a,b) and a gene set enrichment analysis (GSEA) was performed. In both cohorts, OxPHOS- and mitochondrial metabolism-related gene signatures were enriched in the short overall survival groups of patients (Figure 1a,b). In addition, sets of genes involved in the unfolded protein response and mitochondrial stress were also enriched in short survival patients from the TCGA cohort. Interestingly, a higher expression of OxPHOS and NADH-dehydrogenase ETCI complex-related gene signatures derived from AML patients [10] was also seen in shorter survival groups of both TCGA and Metzeler cohorts (Figure 1c), suggesting an involvement of ETCI and mitochondrial metabolism as adverse prognosticators for AML patients.

### 3.2. AraC-Tolerant Murine Leukemic Cells Exhibit a High OxPHOS Phenotype also Seen in Drug-Resistant Human AML Cells

**Figure 2:**
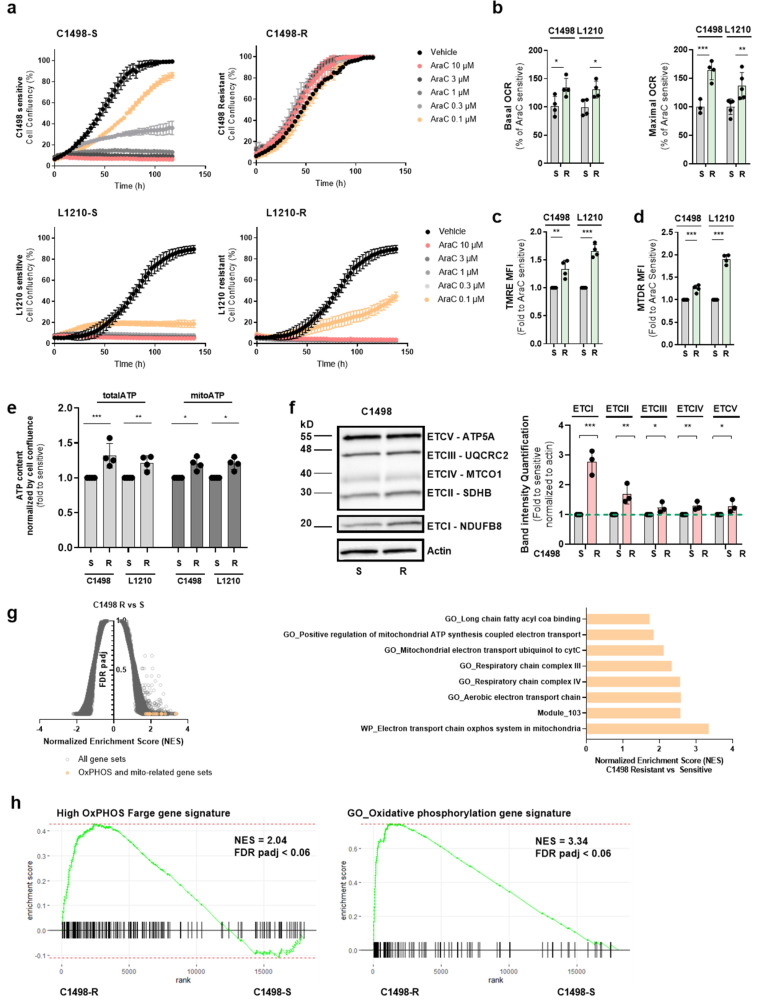
AraC-tolerant leukemic cells have more active mitochondria. (**a**) Proliferation IncuCyte assay of murine leukemic C1498 and L1210 cell lines sensitive (C1498S, L1210S) and resistant (C1498R, L1210R) to AraC. (**b**) Seahorse assessment of basal and maximal respiration in murine leukemic cells sensitive or tolerant to AraC (*n* = 4). (**c**) FACs assessment of mitochondrial membrane potential with the TMRE probe in AraC-sensitive vs. -resistant conditions for both cell lines (*n* = 4); (**d**) Mitochondrial mass assessment with MTDR (MitoTracker Deep Red) in both cell lines in the condition of sensitiveness and resistance to AraC (*n* = 4). (**e**) Total and mitochondrial ATP content measured by Cell Titer Glo in C1498 and L1210 cell lines sensitive and resistant (*n* = 4). (**f**) Protein expression levels assessed by Western blot of the ETC complexes in C1498 sensitive and resistant to AraC (*n* = 3). (**g**) Volcano plot showing the signatures enriched in AraC-resistant vs. -sensitive C1498 cells (FDR q-val < 0.05) on the left and a plot showing OxPHOS and mitochondrial-related gene sets differentially expressed in resistant vs. sensitive conditions with their normalized enrichment scores (NES). (**h**) Gene set enrichment plot of the GO oxidative phosphorylation and Farge_High OxPHOS gene signatures in resistant vs. sensitive C1498 cells. * is *p* < 0.05, ** is *p* < 0.01, *** is *p* < 0.005, ns is statistically non-significant.

As we were interested in an immunocompetent context, we assessed whether the previously described high OxPHOS phenotype of AraC-resistant human cells was also observed in murine AML cells. To this end, C1498 and L1210 murine AML cell lines were grown in the presence of AraC, whose concentration was increased up to 10 µM for C1498 and up to 0.1 µM for L1210 (Figure 2a). AraC-tolerant cells (C1498-R, L1210-R) displayed higher basal and maximal respiratory rates as well as a higher mitochondrial membrane potential, mass and ATP levels compared with AraC-sensitive cells (C1498-S, L1210-S; Figure 2b–e). Western blots revealed a higher expression of ETC complex subunits, especially ETCI, in C1498-R-resistant cells compared with C1498-S cells (Figure 2f, Supplementary Materials Figures S3 and S4) whereas no consistent changes in ETC complex subunits were observed in L1210-R cells compared with L120-S (Figure S1a, Supplementary Materials Figures S3 and S4). In addition, AraC-resistant C1498 cells also showed an enrichment in OxPHOS-related gene sets including the high OxPHOS gene signature (Figure 2g,h). Therefore, murine leukemic cells able to grow in the presence of AraC exhibited an increased OxPHOS metabolism observed in chemo-resistant human leukemic cells.

### 3.3. EVT-701 Blocks OxPHOS by Inhibiting ETCI and Induces Metabolic Compensatory Reprogramming in Human and Murine AML Cells

**Figure 3:**
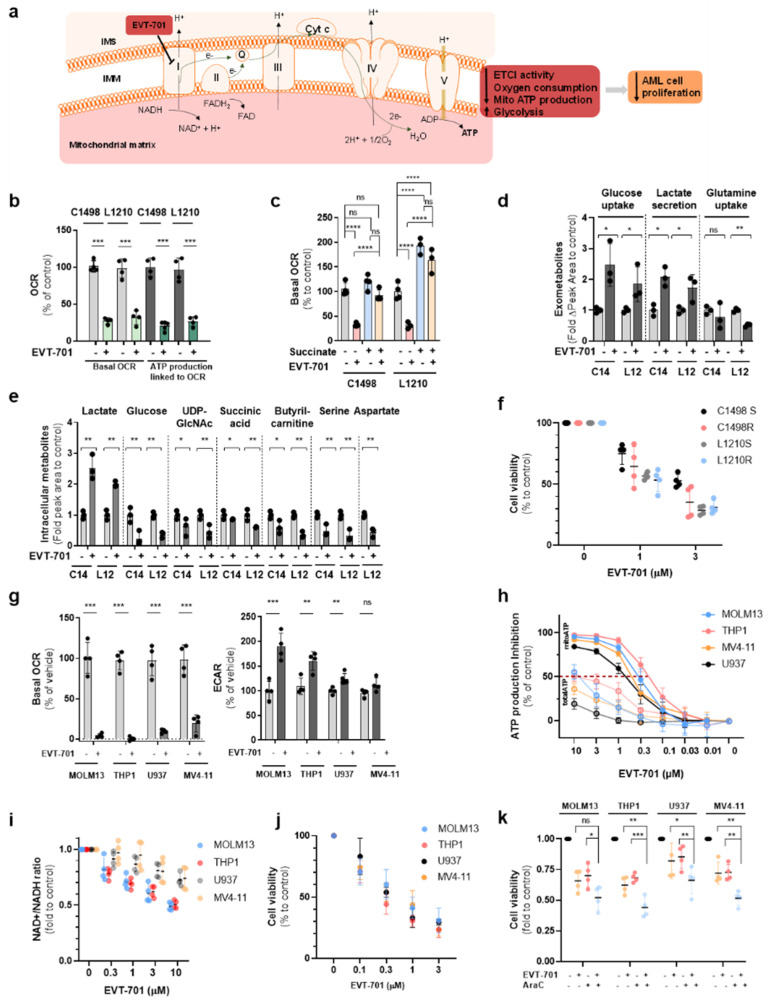
EVT-701 blocks OxPHOS by inhibiting ETCI and induces a profound metabolic compensatory reprogramming. (**a**) Schematic representation of the mode of action of EVT-701. (**b**) Effect of EVT-701 on the basal oxygen consumption rate and ATP production linked to OCR in murine AML cells, assessed by Seahorse (*n* = 4). (**c**) Effect of 10 mM succinate supplementation upon ETCI inhibition with EVT-701 in permeabilized leukemic cells (*n* = 4 C1498, *n* = 3 L1201). (**d**) Effect of EVT-701 on lactate and glucose in the exometabolome of C1498 and L1210 after 24 h incubation (*n* = 3). (**e**) Effect of EVT-701 on intracellular metabolites in C1498 and L1210 cells after 24 h incubation (*n* = 3). (**f**) Effect of EVT-701 on C1498 and L1210 proliferation (*n* = 4). (**g**) Effect of EVT-701 on oxygen consumption rate and extracellular acidification rate in human AML cell lines assessed by Seahorse (*n* = 4). (**h**) Dose-response effect of EVT-701 on total ATP and mitochondrial ATP production in human AML cell lines assessed by Cell Titer Glo (*n* = 4). (**i**) NAD+/NADH ratio change in response to EVT-701 in human AML cell lines (*n* = 4). (**j**) Dose-response effect of EVT-701 on human AML cell line proliferation (*n* = 4). (**k**) Effect of EVT-701, AraC or their combination on human AML cell line proliferation (*n* = 4). * is *p* < 0.05, ** is *p* < 0.01, *** is *p* < 0.005, **** is *p* < 0.001, ns is statistically non-significant.

As shown in solid tumors [23], EVT-701 was demonstrated here to inhibit oxygen consumption in both murine and human leukemic cell lines grown in the presence of glucose and pyruvate (Figure 3a,b). The blockade in mitochondrial respiration was bypassed in the presence of succinate, which donates electrons at the level of ETC complex II (Figure 3c), denoting that EVT-701 does not inhibit the electron transport chain beyond the ETCI complex. EVT-701 induced a shift toward a glycolytic phenotype as observed by an increase in glucose consumption and lactate production in the exometabolome of the murine leukemic cells (Figure 3d). EVT-701 did not affect the expression of ETC complexes assessed by Western blot on AML cells (Figure S1b, Supplementary Materials Figures S3 and S4). At the intracellular level, EVT-701 decreased the levels of glucose, UDP-GlcNAc, succinic acid, butyryl-carnitine, serine and aspartate and it increased the intracellular lactate in both the C1498 and L1210 cell lines (Figure 3e). EVT-701 decreased the cell proliferation as a single agent in murine leukemic cells (Figure 3f). Importantly, EVT-701 similarly affected the mitochondrial function of four human AML cell lines, decreasing OCR, the mitochondrial ATP level and the cellular NAD+/NADH ratio (Figure 3g–i) measured in the presence of glucose and pyruvate while it induced a decrease in cell viability (Figure 3j) and showed an additive effect to AraC to decrease the viability of human AML cells (Figure 3k).

### 3.4. EVT-701 Decreases the Expression of Immune Checkpoint Markers in Murine and Human Leukemic Cells

**Figure 4:**
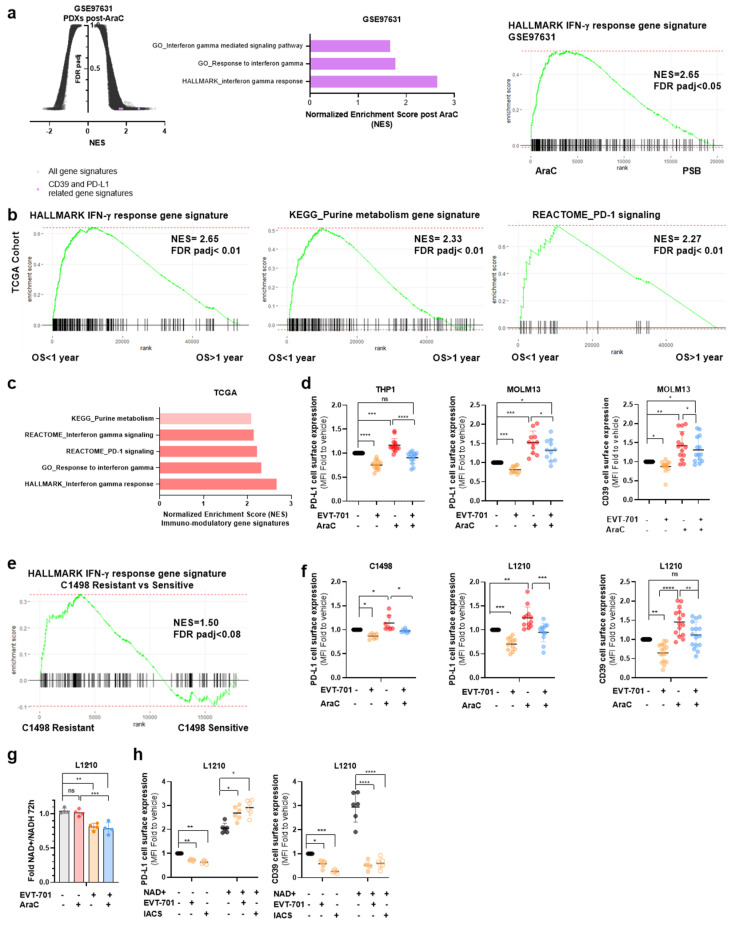
EVT-701 decreases the immune checkpoint expression to boost the immune response and increase the therapeutic benefit. (**a**) Volcano plot showing GSEA results in the GSE97631 data set containing the transcriptomic profiles of residual AML in PDXs AraC or vehicle-treated. Immune modulation-related gene signatures are in purple. In the middle, a plot showing AraC vs. PBS-treated PDXs differentially expressed gene sets with their normalized enrichment scores (NES). On the right, the enrichment plot of the hallmark Interferon-γ gene signature compatible with PD-L1 increases after AraC in vivo. (**b**) Enrichment plots of the hallmark Interferon-γ gene signature (**left**), KEGG purine metabolism-containing CD39 (**middle**) and Reactome PD-1 signaling (**right**) in the short group survival of the TCGA cohort. (**c**) Plot showing CD39- and PD-L1-related signatures in the short group of the TCGA cohort with their normalized enrichment scores (NES). (**d**) FACS assessment of the modulation of inducible PD-L1 and CD39 membrane expression on THP1 and MOLM13 cells by EVT-701, AraC or a combination of both (*n* > 10). (**e**) Enrichment plot of the hallmark Interferon-γ gene signature in C1498-resistant vs. -sensitive cell transcriptomes. (**f**) FACs assessment of the modulation of inducible and CD39 membrane expression on C1498 (*n* = 7) and L1210 (*n* > 10) by EVT-701, AraC or a combination of both at 72 h. (**g**) Effect of EVT-701, AraC or a combination of both on the NAD+/NADH ratio in L1210 at 72 h (*n* = 4). (**h**) Effect of exogenous NAD+ on the modulation of PD-L1 and CD39 membrane levels by EVT-701 or with IACS (*n* = 6). * is *p* < 0.05, ** is *p* < 0.01, *** is *p* < 0.005, **** is *p* < 0.001, ns is statistically non-significant.

GSEA showed that gene sets associated with the interferon-γ response or signaling pathways that lead to an increased expression of PD-L1 were enriched in AraC-residual AML cells (Figure 4a) and in patients with a shorter overall survival (OS; Figure 4b,c), indicating a key role of immune checkpoints in a relapse to chemotherapy in AML. Interestingly, the transcriptome of murine-resistant cells was also enriched in the interferon-γ response gene signature (Figure 4 and Supplementary Material Figure S5). To confirm this observation, we assessed CD39 and inducible PD-L1 levels by flow cytometry in murine and human leukemic cell lines treated with EVT-701, AraC or their combination. AraC increased the cell surface expression of both inducible PD-L1 and CD39 in leukemic cells while EVT-701 as single agent as well as in combination with AraC decreased these proteins in the cellular membrane, suggesting potential as an immunomodulatory agent (Figure 4d–f). Interestingly, ETCI inhibition with IACS also resulted in decreased CD39 and inducible PD-L1 membrane levels (Supplementary Figure S2c,d). In addition, CD39 mRNA was also decreased by metformin (Supplementary Figure S2f). EVT-701 as single agent and in combination with AraC decreased the cellular NAD+/NADH ratio (Figure 4g), which has recently been proposed as the driving factor of inducible PD-L1 decreased expression [27]. Indeed, media supplementation with NAD+ restored PD-L1 levels in the presence of EVT-701 or IACS while CD39 levels were not altered by exogenous NAD+ in the presence of ETCI inhibitors (Figure 4h), showing a differential modulatory effect of ETCI inhibition on the two checkpoints. In addition, while PD-L1 expression was induced by IFN-γ, CD39 was unaffected (Supplementary Figure S2a,b,e), highlighting a different regulatory mechanism for both immune checkpoints. In sum, EVT-701 may be able to decrease tumor-mediated immunosuppression by decreasing inducible PD-L1 and CD39 expression on leukemic cells.

## 4. Discussion

In summary, we have demonstrated that the OxPHOS phenotype induced by the metabolic adaptation to chemotherapy is a common mechanism in murine and human AML cells and many other forms of cancer [10,11]. In addition, we have shown that the genetic signatures of shorter survival groups within human AML cohorts are enriched in OxPHOS and mitochondrial metabolism gene sets, underscoring the involvement of mitochondria in the progression and relapse of the disease. We characterized the effect of EVT-701, a new selective ETCI inhibitor, on the metabolism in human and murine AML cell lines, characterizing the metabolic reprogramming it exerts on AML cells by inducing a shift toward glycolysis, increasing glucose consumption and lactate production while decreasing mitochondrial ATP production and the cellular NAD+/NADH ratio. The metabolic changes observed upon EVT-701 suggest an impairment of the OxPHOS function and all have been previously observed with other ETCI inhibitors. The glycolytic shift upon ETCI inhibition (Pasteur effect) has been already observed with other ETCI inhibitors such as metformin [28]. In addition, a fall in aspartate levels also occurred with IACS-01759 (hereafter IACS) [15]. This is explained by the drop in the NAD+/NADH ratio induced by ETCI inhibition, which impairs aspartate biosynthesis by interfering with the mitochondrial malate dehydrogenase (MDH2) activity [29,30]. It has been also reported in several works that de novo serine biosynthesis is impaired upon ETCI inhibition [31,32] while being still catabolized via the folate pathway [32], leading to the reduction in serine levels. The decrease in succinic acid could suggest decreased TCA activity though the levels of other TCA cycle intermediates were not consistently reduced in this study. This could be compatible with TCA replenishment through the glutamine-derived reductive carboxylation of α-KG via NADPH-dependent isocitrate dehydrogenase 1 (IDH1) to generate citrate, also observed with other ETCI inhibitors [33,34] despite the fact that EVT-701 does not stimulate glutamine uptake (Figure 3d). In addition, EVT-701 decreased the proliferation of AML cells both as a single agent and in combination with AraC. Using in vitro models, we showed that AraC increased CD39 and inducible PD-L1 at the cell surface while EVT-701 as single agent decreased the expression levels of these proteins and, when used in combination with AraC, neutralized the increase induced by the chemotherapeutic agent. In addition, we showed that the decrease of immune checkpoints is not exclusive to EVT-701 because other ETCI inhibitors such as IACS and, as reported in the literature, metformin elicited similar responses [19,20].

As NAD+ supplementation abrogated the decrease of the inducible PD-L1 expression but not that of CD39 upon ETCI inhibition and considering also that only PD-L1 is induced by IFN-γ, we propose that two different regulatory mechanisms concur to achieve the modulation of these two immune checkpoint components. It is important to remark that despite the controversy around the NAD+ uptake by mammalian cells, it has been previously reported that exogenous NAD+ can rescue cells from death upon NAD depletion induced by the nicotinamide phosphoribosyl transferase (NAMPT) inhibitor FK866 [35]. In addition, NAD+ supplementation might also rescue cells from the block in DNA repair resulting from PARP1 inactivation upon FK866 NAD depletion [36], reinforcing the assumption that NAD+ can be uptaken and used by the cells. Remarkably, MCART1/SLC25A51 has been recently identified as the NAD importer in the mitochondria of mammal cells and necessary for ETCI activity [37,38]. Interestingly, previous studies showed that exogenous NAD+ can access mammalian mitochondria and increase respiration [39]. Therefore, supplementation with NAD+ could potentially affect the intracellular NAD+/NADH ratio changes induced by ETCI inhibition in our cellular context. The proposed regulatory mechanism of PD-L1 involves ETCI-driven NAD+ as a major coordinator. NAD+ is known to be a cofactor of sirtuin 1 (SIRT1), a class III histone deacetylase involved in epigenetic control [40]. Resveratrol is a SIRT1 agonist that has been reported to stimulate ETCI activity to increase the NAD+/NADH ratio and activate SIRT1 [41]. Interestingly, several studies described that resveratrol upregulated PD-L1 expression in several cancer types including colon, breast and lung cancer [42,43,44]. One of these studies supported that PD-L1 expression was upregulated upon SIRT1 activation with resveratrol via NF-κB [43]. Remarkably, PD-L1 inducible expression has been reported to be upregulated through IFN-γ activation of the NF-κB pathway [42]. Interestingly, a recent work identified that the ETCI function is essential for IFN-γ signaling because upon the genetic or pharmacological function abrogation of exclusively the first ETC complex, IFN-γ signaling is impaired and PD-L1 levels are decreased [45]. This study suggests that the NAD+ driven by the functional ETCI is essential for IFN-γ signaling as the impaired activity of exclusively ETCI is enough to decrease the IFN- γ signaling pathway and PD-L1 expression. Interestingly, it has been recently observed that OxPHOS cells have increased PD-L1 levels [46,47].

**Figure 5:**
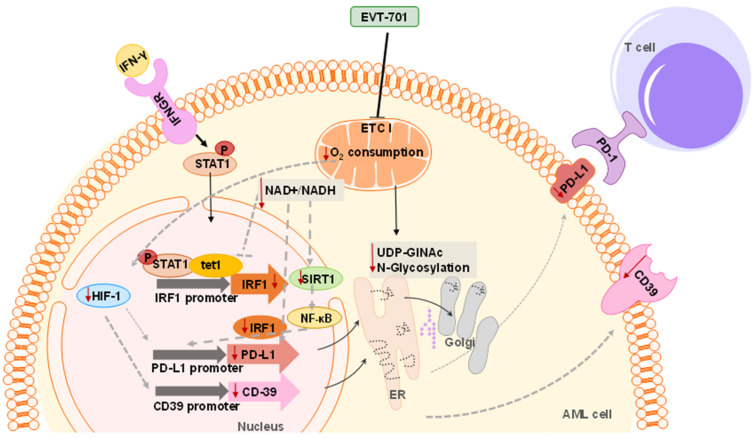
Schematic view of the proposed mechanism of action of EVT-701 as modulator of the immune checkpoints PD-L1 and CD39 membrane levels.

In addition, functional ETCI and NAD metabolisms have been shown to be involved in the epigenetic regulation of inducible PD-L1 expression [27]. In fact, this study proposed a mechanism by which NAD+ metabolism, via TCA intermediates such α-ketoglutarate (α-KG), activates TET1. TET1 then interacts with p-STAT1, previously phosphorylated and activated by JAK after IFN-γ IFNGR stimulation. They showed that the TET1-p-STAT1 interaction stabilized TET-1 and facilitated the TET1-mediated demethylation of the Irf1 promoter, which subsequently promotes the generation of IRF1 and induces PD-L1 expression [48,49,50]. The link between TET1 activity and PD-L1 expression has already been proposed in gliomas. Lower PD-L1 levels have been reported in patients bearing IDH mutations, increasing (R)-2-hydroxyglutarate (2-HG) oncometabolite production [51,52]. 2-HG is a competitive inhibitor of multiple α-KG-dependent dioxygenases, which includes the TET family of 5-methlycytosine hydroxylase [53,54] thereby explaining the loss of TET activity concomitant with IDH mutations. It has recently been reported that in response to 2-HG inhibition, the PD-L1 levels in IDH1 mutant tumors were increased to the same levels observed in WT-IDH gliomas [55]. As IDH mutations are also present in several AML patients [56,57], the same profile regarding the IDH mutational state and PD-L1 levels might also be expected although this remains to be proven. In addition, TET1 repression has also been associated with increased tumor-infiltrating immune cells in several cancer types such as basal-like breast cancer (BLBC), melanoma, ovarian, lung and thyroid cancer [58]. Considering all of the above, we propose that ETCI-NADH dehydrogenase activity regulates the level and ratios of NAD+/NADH, which is essential for SIRT1- and/or TET1-mediated PD-L1 stimulation. Recent studies showed that CD39 expression was not induced by IFN-γ [59,60] whereas it can be induced under hypoxic conditions [61,62]. ETCI inhibitors have already been reported to interfere with HIF stabilization such as IACS [15,63] and metformin [64,65,66]. EVT-701 was also developed from a screening campaign to characterize HIF-1α inhibitors [22]; therefore, we propose an impairment of hypoxic signaling by interfering with HIF-1α stabilization as the mechanism by which EVT-701 decreases CD39 expression. Importantly, EVT-701 was shown to decrease the levels of the N-glycosylation unit UDP-GlcNAc, which can impair the CD39 function and localization in the cell membrane [67], highlighting another layer of the regulation of CD39 by EVT-701. Further studies will be needed to elucidate the exact mechanism involved in the ETCI regulation of PD-L1 and CD39 (Figure 5). Future perspective research would answer the question of whether ETCI inhibition decreases immune checkpoints in patient-derived samples and, eventually, in patient-derived xenografts (PDXs).

## 5. Conclusions

In conclusion, this work describes the opposed actions of chemotherapy and ETCI inhibitors on immune system modulation, providing a non-canonical function of ETCI in the regulation of CD39 and PD-L1 and a rationale to improve the anti-tumor response in AML including combinations with immune checkpoint therapies.

## Figures and Tables

**Table 1:** Clinical and mutational information about the AML cell lines used in this study.

	FAB	Karyotype	Sex	FLT3		NPM1	IDH1	Kit	N/K
				ITD	TKD		IDH2		Ras
Human									
MOLM13	M5-AML	ins(11;9)(q23;p22p23)	M	ITD	WT	WT	WT	WT	WT
MV4-11	M5-AML	Complex	M	ITD	WT	WT	WT	WT	WT
THP-1	M5-AML	Complex	M	WT	WT	WT	WT	WT	NRAS p.G12D
U937	M5-AML	t(10;11)(p13;q14)	M	WT	WT	WT	WT	WT	WT

**Table 2:** CD39 and PD-L1 antibodies used in this study.

Antibody	Dilution	Supplier	Catalogue Number
Anti-mPD-L1-APC	1:50	Biolegend, Ozyme	#124312
APC-anti-Rat IgG2b,κ	1:50	Biolegend, Ozyme	#400611
Anti-mCD39-PE-Cy7	1:50	Biolegend, Ozyme	#143806
PE/Cyanine7-anti-Rat IgG2a,κ	1:50	Biolegend, Ozyme	#400522
Anti-hPD-L1-APC	1:100	Biolegend, Ozyme	#329708
APC-anti-Mouse IgG2b,κ	1:100	Biolegend, Ozyme	#400320
Anti-hCD39-PE-Cy7	1:100	Biolegend, Ozyme	#328212
PE/Cyanine7-anti-Mouse IgG1,κ	1:100	Biolegend, Ozyme	#400126

## Data Availability

GEO (Gene expression Omnibus, https://www.ncbi.nlm.nih.gov/geo/, 21 November 2020) accession codes of data sets used in the study are GSE12417; GSE97631; GSE97346. The transcriptomic data C1498 R vs. S are attached in Supplementary Material Figure S5_raw_and_normalized_count_matrix.

## Supplementary Materials

The following are available online at https://www.mdpi.com/article/10.3390/cancers13143499/s1, Figure S1: ETC complexes expression in murine AML cells, Figure S2: Modulation of immune checkpoints on leukemic cells, Supplementary Material S3: Western blot raw images, Supplementary Material S4: Densitometry readings of Western blot bands, Supplementary Material S5: Raw count and normalized count matrix of the transcriptomic data C1498 R vs. S.
