# Supplementary material for: Disrupting Mitochondrial Electron Transfer Chain Complex I Decreases Immune Checkpoints in Murine and Human Acute Myeloid Leukemic Cells
Source: Cancers (Basel). 2021 Jul 13;13(14):3499. doi: 10.3390/cancers13143499 (PMC8306173; doi:10.3390/cancers13143499)
Supplement: Supplementary file 1 [file cancers-1271757-supplementary.pdf › cancers-1271757-supplementary.pdf]

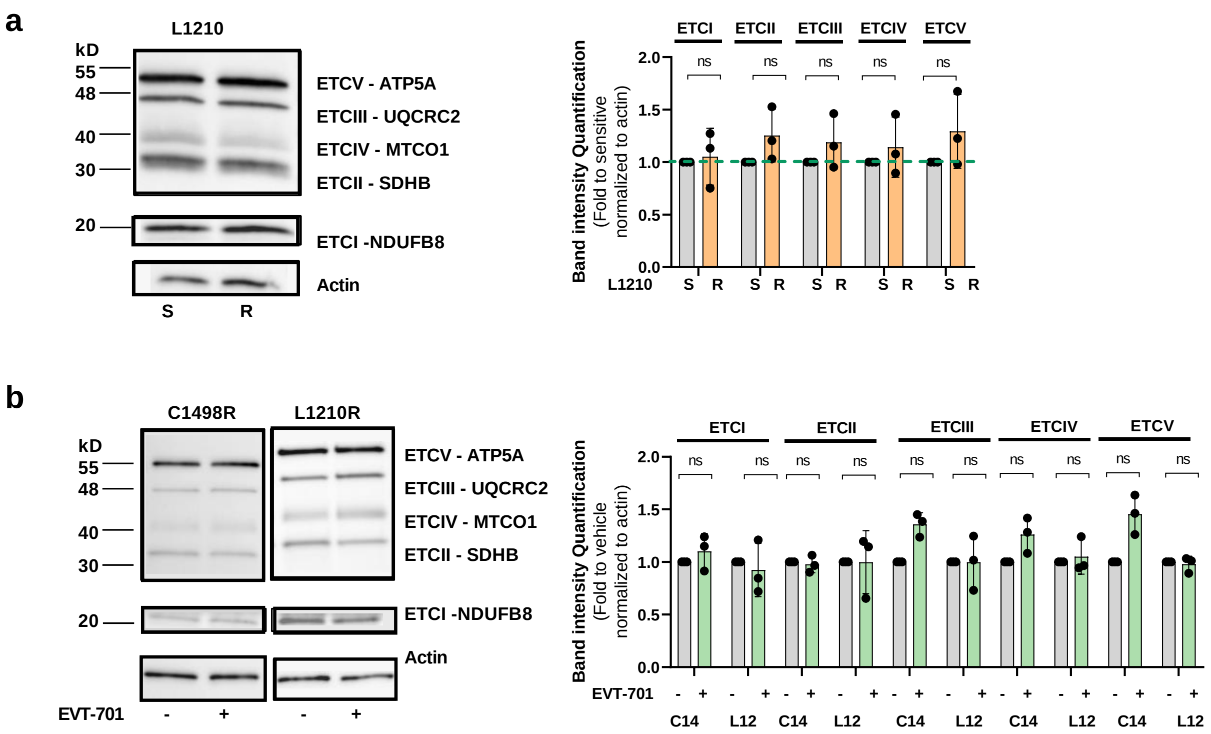

Supplementary figure S1.

a) Protein expression levels assessed by Western blot of the ETC complexes in L1210 sensitive and resistant to AraC (N=3). b) Protein expression levels assessed by Western blot of the ETC complexes in L1210 and C1498 resistant to AraC upon 72h of EVT-701 treatment (N=3).

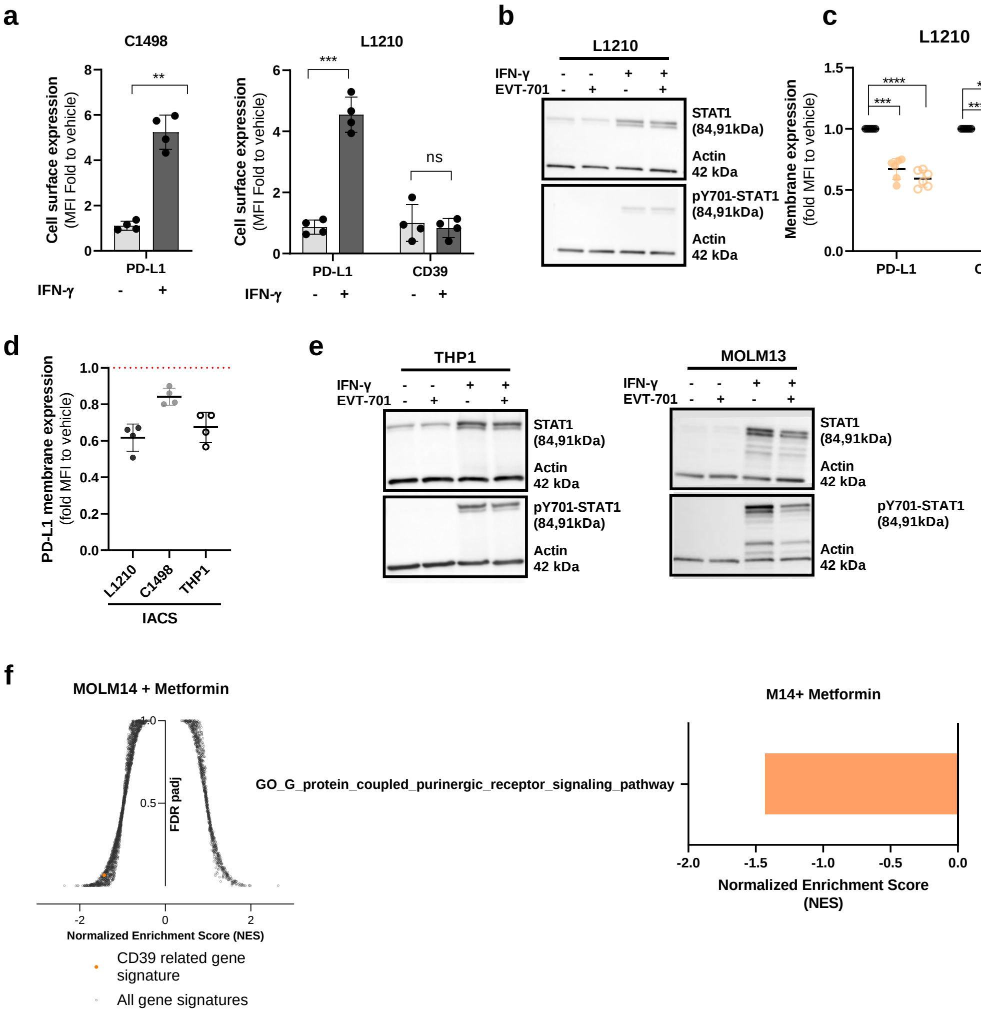

Supplementary figure S2.

a) IFN-γ effect on PD-L1 membrane expression level assessed by FACS on C1498 cells (left). IFN-γ effect on PD-L1 and CD39 membrane expression level assessed by FACS on L1210 cells (right) (N=4). b) Western blot of L1210 showing pY701-STAT1 and total STAT1 levels after 48h of in the presence or absence of IFN-γ and EVT-701 (N=3). c) Effect of IACS or EVT-701 on PD-L1 and CD39 membrane expression levels in L1210 (N=6). d) Effect of IACS on PD-L1 membrane expression level in L1210, C1498 and THP1 cells (N=4). e) Western blot of THP1 and MOLM13 cells showing pY701-STAT1 and total STAT1 levels after 24h of in the presence or absence of IFN-γ and EVT-701 (N=3). f) Volcano plot representation of GSEA of metformin effect on MOLM14 transcriptome. CD39 related gene signatures downregulated under metformin treatment (FDR< 0.05) are highlighted in orange (left). On the right, a plot showing the CD39 related signatures in MOLM14 treated with metformin compared to PBS with their normalized enrichment scores (NES).

Supplemental Materials S3. Western blots Images

WB of Figure 2f and S1a

N=1

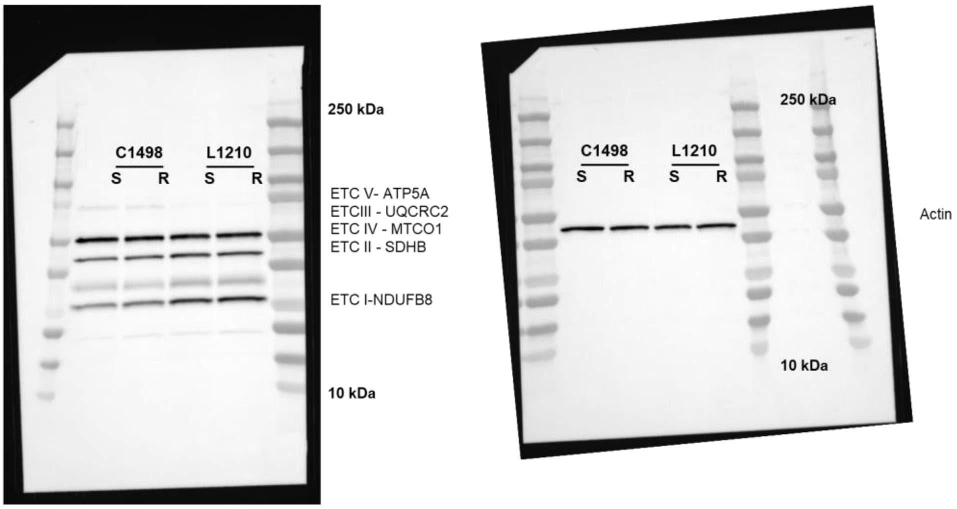

N=2

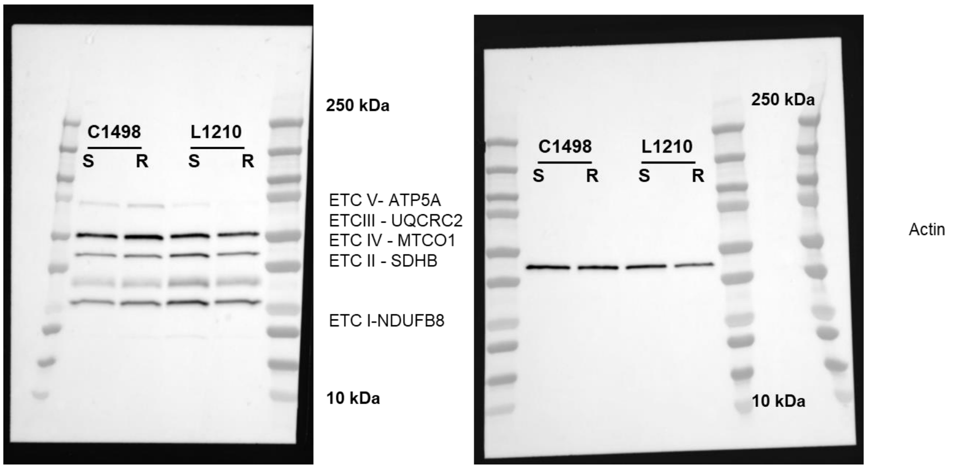

N=3

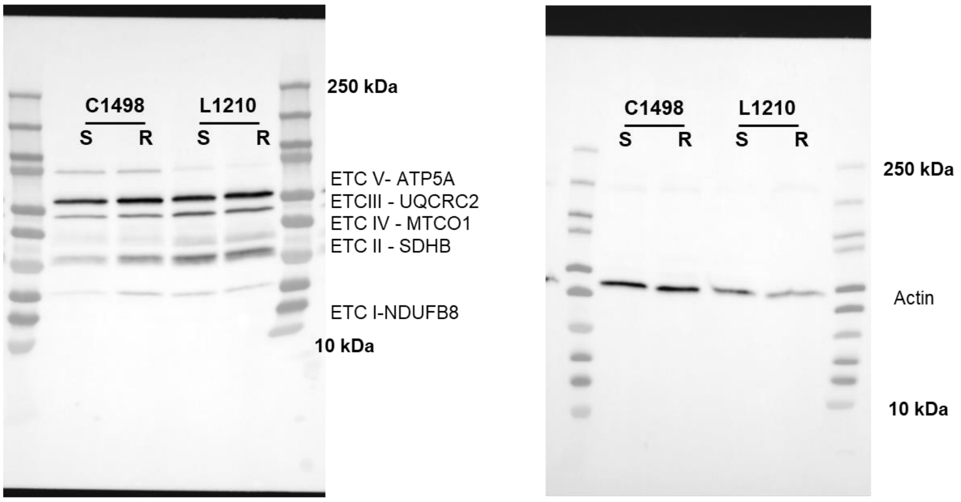

WB of Figure S1b

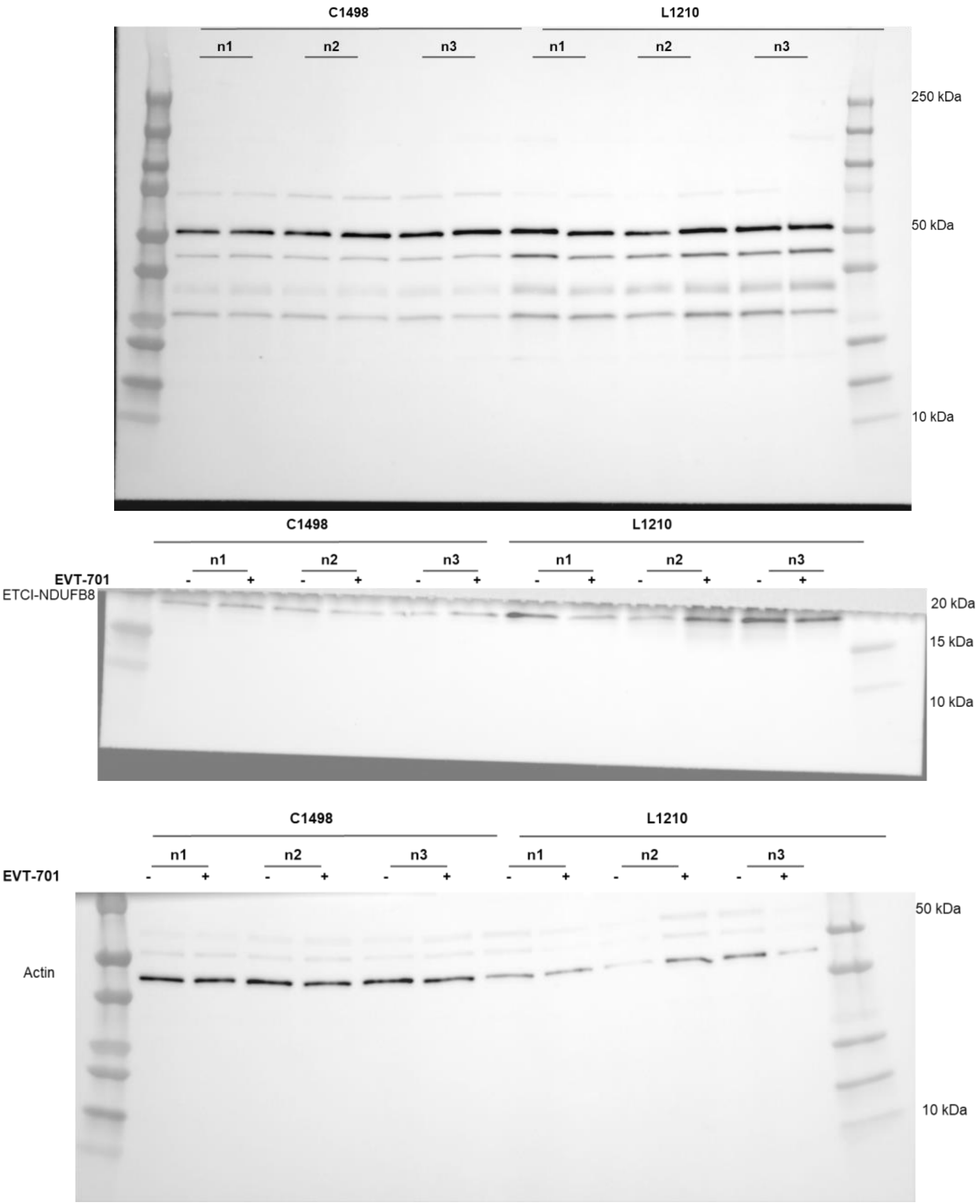

WB of Figure S2 b

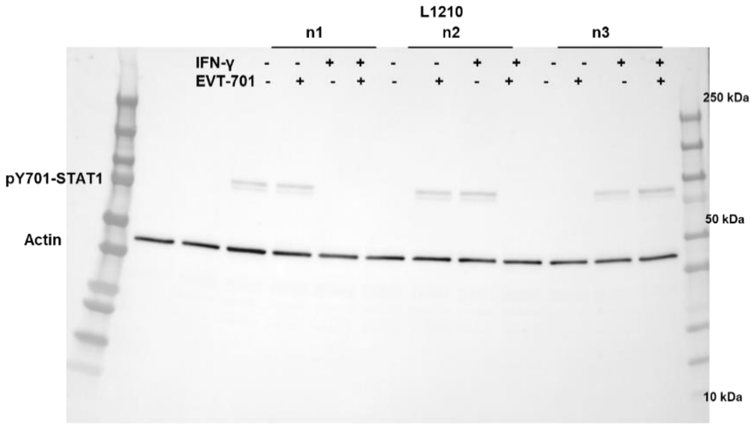

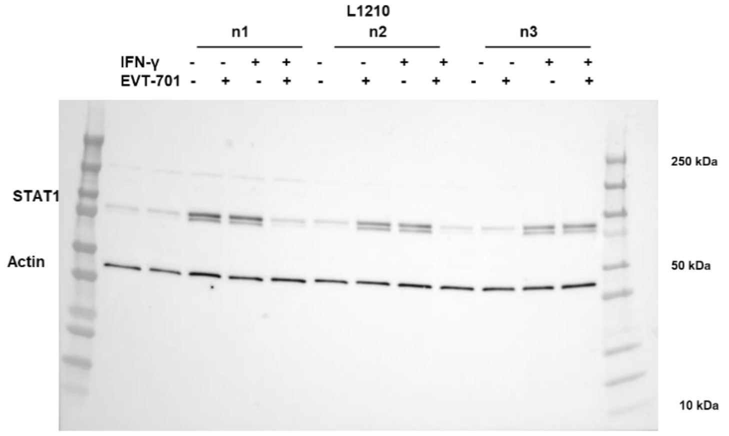

WB of Figure S2 e

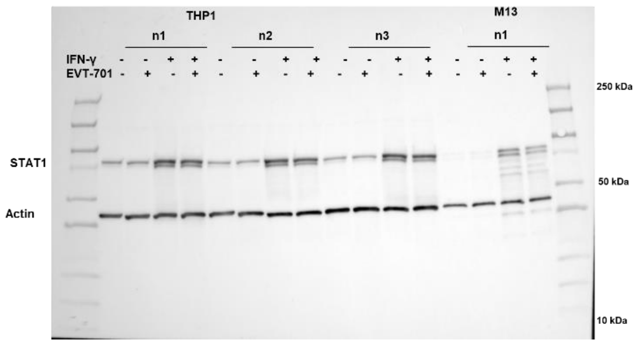

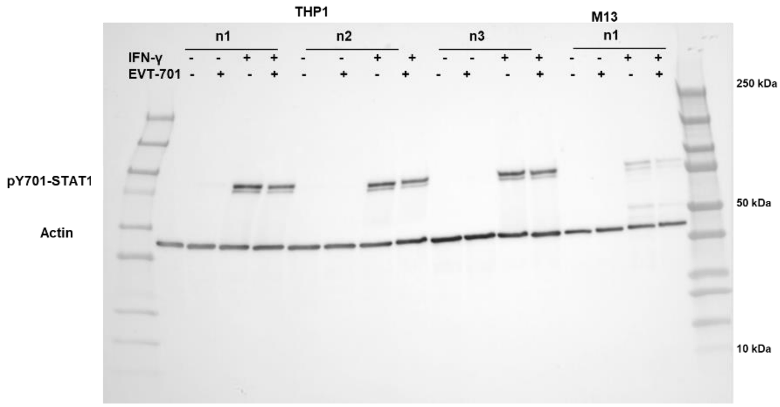

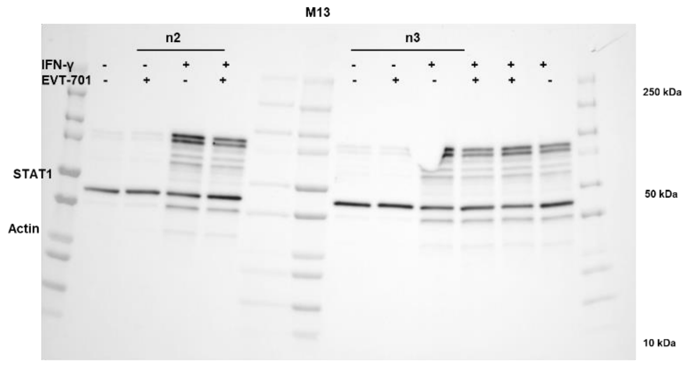

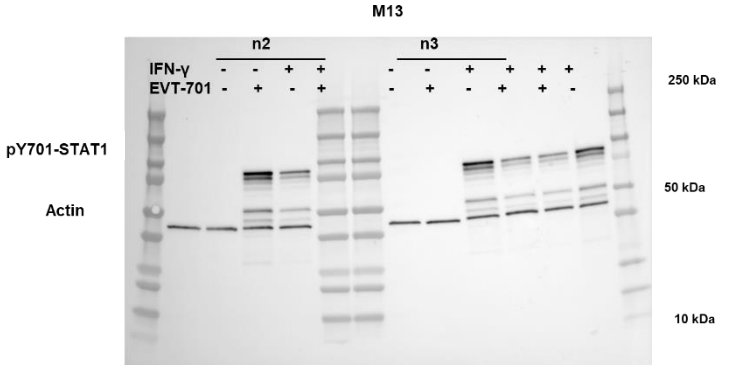

Supplementary material S4. Densiometry readings WB

R vs S

		Raw minus blank	Raw minus blank					Fold Actine							Fold R/S						
		Actin	ETCV	ETCIV	ETC III	ETC II	ETCI	ETCV	ETCIV	ETC III	ETC II	ETCI	ETCI alone	Fold actin ETCI alone	Fold RS ETCI alone	ETCV	ETCIV	ETC III	ETC II	ETCI	
n1	1	2959242	91549748	43798832	25037702	40348624	1750969	30.93689	14.80069	8.46085	13.63478	0.591695	1367393	0.462075							C1498 S
	2	2505841	85654788	45676588	23100524	49417956	3450929	34.18205	18.22805	9.218671	19.72111	1.377154	2376223	0.948274	2.052205	1.104896	1.231567	1.089568	1.446382	2.327472	C1498R
	3	2225516	83123980	59238268	37874852	71103060	3768126	37.35043	26.61777	17.01846	31.94902	1.693147	2614150	1.174626							L1210 S
	4	2518123	92209428	60000720	40844204	82946820	4829425	36.61832	23.82756	16.2201	32.93994	1.917867	3836457	1.523538	1.297041	0.980399	0.895175	0.953089	1.031016	1.132723	L1210 R
n2	5	52027858	41669121	15016657	11685267	20285647	321753	0.8009	0.288627	0.224596	0.3899	0.006184	2102256	0.040406							C1498 S
	6	45144074	54386645	18886889	14532761	27202105	798554	1.204735	0.418369	0.32192	0.602562	0.017689	4367910	0.096755	2.394547	1.504226	1.449514	1.433325	1.545428	2.860335	C1498R
	7	40806634	46274989	28770105	27670943	39283669	1352283	1.134007	0.705035	0.678099	0.962678	0.033139	6956008	0.170463							L1210 S
	8	25897954	35995489	19665665	20231109	30007701	645272	1.389897	0.759352	0.781186	1.15869	0.024916	3494853	0.134947	0.791652	1.225652	1.077042	1.152023	1.203611	0.751866	L1210 R
n3	17	60572963	9799915	1268869	3533925	2829630	418350	0.161787	0.020948	0.058342	0.046714	0.006907	24686479	0.407549							C1498 S
	18	60021155	11839857	1482120	4111953	5764975	1301643	0.197261	0.024693	0.068508	0.096049	0.021686	66147551	1.102071	2.704139	1.219266	1.178803	1.174263	2.056091	3.139978	C1498R
	19	35912499	10491029	2648236	5923176	8088197	1027627	0.292128	0.073741	0.164934	0.22522	0.028615	58339793	1.624498							L1210 S
	20	25202415	12326046	2706749	6082311	8666962	917707	0.489082	0.1074	0.241338	0.343894	0.036413	53141445	2.108585	1.297992	1.674207	1.456447	1.463246	1.526928	1.272542	L1210 R

EVT vs DMSO

		Raw vol.			Minus blank			Fold actin			Fold to vehicle		
	**Spot 1**	**9742035.00**	DMSO	C1498	7961606.00	DMSO		0.14716856	DMSO		**1**	**DMSO**	
	Spot 2	9877911.00	EVT		8097482.00	EVT		0.16917253	EVT		**1.14951537**	**EVT**	
	Spot 3	8863342.00	DMSO		7082913.00	DMSO	C1498	0.11655103	DMSO	C1498	**1**	**DMSO**	C1498
	Spot 4	6723643.00	EVT		4943214.00	EVT		0.10653494	EVT		**0.91406267**	**EVT**	
	Spot 5	5836087.00	DMSO		4055658.00	DMSO		0.06888587	DMSO		**1**	**DMSO**	
	Spot 6	5826615	EVT		4046186.00	EVT		0.08533556	EVT		**1.23879642**	**EVT**	
ETC I	Spot 7	16797492	DMSO	L1210	15017063.00	DMSO		0.73497191	DMSO		**1**	**DMSO**	
	Spot 8	10623639	EVT		8843210.00	EVT		0.52894242	EVT		**0.71967705**	**EVT**	
	Spot 9	11122353.00	DMSO		9341924.00	DMSO	L1210	1.75609582	DMSO	L1210	**1**	**DMSO**	L1210
	Spot 10	18056340	EVT		16275911.00	EVT		2.12246893	EVT		**1.20862934**	**EVT**	
	Spot 11	25432498.00	DMSO		23652069.00	DMSO		0.86670915	DMSO		**1**	**DMSO**	
	Spot 12	22759936	EVT		20979507.00	EVT		0.73360401	EVT		**0.84642467**	**EVT**	
	Spot 13	1780429.00	BLANK		0.00	BLANK			BLANK				
	Spot 1	75187280	DMSO		73033530	DMSO		1.35000899	DMSO		**1**	**DMSO**	
	Spot 2	83655200	EVT		81501450	EVT		1.7027276	EVT		**1.26127131**	**EVT**	
	Spot 3	95735664	DMSO	C1498	93581914	DMSO	C1498	1.53991277	DMSO	C1498	**1**	**DMSO**	C1498
	Spot 4	119084584	EVT		116930834	EVT		2.52006483	EVT		**1.63649843**	**EVT**	
	Spot 5	105191608	DMSO		103037858	DMSO		1.75011108	DMSO		**1**	**DMSO**	
ETC V	Spot 6	123531168	EVT		121377418	EVT		2.55989477	EVT		**1.46270417**	**EVT**	
	Spot 7	125528600	DMSO		123374850	DMSO		6.03826791	DMSO		**1**	**DMSO**	
	Spot 8	106410896	EVT		104257146	EVT		6.23597389	EVT		**1.03274217**	**EVT**	
	Spot 9	82008408	DMSO	L1210	79854658	DMSO	L1210	15.0110867	DMSO	L1210	**1**	**DMSO**	L1210
	Spot 10	118517992	EVT		116364242	EVT		15.1745416	EVT		**1.01088894**	**EVT**	
	Spot 11	119038632	DMSO		116884882	DMSO		4.28314315	DMSO		**1**	**DMSO**	
	Spot 12	111497168	EVT		109343418	EVT		3.82348213	EVT		**0.89268138**	**EVT**	
	Spot 13	14386070	DMSO		12232320	DMSO		0.22611179	DMSO		**1**	**DMSO**	
	Spot 14	15525738	EVT		13371988	EVT		0.27936746	EVT		**1.23552807**	**EVT**	
	Spot 15	15072265	DMSO	C1498	12918515	DMSO	C1498	0.21257725	DMSO	C1498	**1**	**DMSO**	C1498
	Spot 16	16467614	EVT		14313864	EVT		0.30848891	EVT		**1.45118494**	**EVT**	
	Spot 17	17446148	DMSO		15292398	DMSO		0.25974332	DMSO		**1**	**DMSO**	
ETC III	Spot 18	19217352	EVT		17063602	EVT		0.3598777	EVT		**1.38551281**	**EVT**	
	Spot 19	56697020	DMSO		54543270	DMSO		2.66948148	DMSO		**1**	**DMSO**	
	Spot 20	34768184	EVT		32614434	EVT		1.95078004	EVT		**0.73077114**	**EVT**	

	Spot 21	37470568	DMSO	L1210	35316818	DMSO	L1210	6.63885902	DMSO	L1210	**1**	**DMSO**	L1210
	Spot 22	53972412	EVT		51818662	EVT		6.75744048	EVT		**1.01786172**	**EVT**	
	Spot 23	43758312	DMSO		41604562	DMSO		1.52456239	DMSO		**1**	**DMSO**	
	Spot 24	56451680	EVT		54297930	EVT		1.89867089	EVT		**1.24538747**	**EVT**	
	Spot 25	11036570	DMSO		8882820	DMSO		0.164197	DMSO		**1**	**DMSO**	
	Spot 26	12230029	EVT		10076279	EVT		0.21051354	EVT		**1.28207903**	**EVT**	
	Spot 27	9778978	DMSO	C1498	7625228	DMSO	C1498	0.12547495	DMSO	C1498	**1**	**DMSO**	C1498
	Spot 28	8456589	EVT		6302839	EVT		0.13583725	EVT		**1.08258461**	**EVT**	
	Spot 29	10515183	DMSO		8361433	DMSO		0.14202	DMSO		**1**	**DMSO**	
ETC IV	Spot 30	11696936	EVT		9543186	EVT		0.20126933	EVT		**1.41719007**	**EVT**	
	Spot 31	29702100	DMSO		27548350	DMSO		1.34828385	DMSO		**1**	**DMSO**	
	Spot 32	23423218	EVT		21269468	EVT		1.2721991	EVT		**0.94356919**	**EVT**	
	Spot 33	23170590	DMSO	L1210	21016840	DMSO	L1210	3.95074771	DMSO	L1210	**1**	**DMSO**	L1210
	Spot 34	31423456	EVT		29269706	EVT		3.81693175	EVT		**0.96612895**	**EVT**	
	Spot 35	31067904	DMSO		28914154	DMSO		1.05953361	DMSO		**1**	**DMSO**	
	Spot 36	39755848	EVT		37602098	EVT		1.31485692	EVT		**1.24097708**	**EVT**	
	Spot 37	24171800	DMSO		22018050	DMSO		0.40699889	DMSO		**1**	**DMSO**	
	Spot 38	19738942	EVT		17585192	EVT		0.36738968	EVT		**0.90267982**	**EVT**	
	Spot 39	22057252	DMSO	C1498	19903502	DMSO	C1498	0.32751688	DMSO	C1498	**1**	**DMSO**	C1498
	Spot 40	16855192	EVT		14701442	EVT		0.31684189	EVT		**0.96740629**	**EVT**	
	Spot 41	20616448	DMSO		18462698	DMSO		0.31359127	DMSO		**1**	**DMSO**	
ETC II	Spot 42	17978148	EVT		15824398	EVT		0.33374242	EVT		**1.06425931**	**EVT**	
	Spot 43	41452144	DMSO		39298394	DMSO		1.92335984	DMSO		**1**	**DMSO**	
	Spot 44	40602360	EVT		38448610	EVT		2.29974191	EVT		**1.19568989**	**EVT**	
	Spot 45	33663320	DMSO	L1210	31509570	DMSO	L1210	5.92317216	DMSO	L1210	**1**	**DMSO**	L1210
	Spot 46	54100000	EVT		51946250	EVT		6.77407867	EVT		**1.14365723**	**EVT**	
	Spot 47	49417504	DMSO		47263754	DMSO		1.73193847	DMSO		**1**	**DMSO**	
	Spot 48	34524708	EVT		32370958	EVT		1.13193626	EVT		**0.65356609**	**EVT**	
Blank	Spot 49	2153750			0								

Actin

	**Raw vol.**									
Spot 1	55467384	DMSO		54098551	DMSO			0		0
Spot 2	49234064	EVT		47865231	EVT			0		0
Spot 3	62139752	DMSO	C1498	60770919	DMSO	C1498	C1498	0	**C1498**	0
Spot 4	47768764	EVT		46399931	EVT			0		0
Spot 5	60243872	DMSO		58875039	DMSO			0		0
Spot 6	48783836	EVT		47415003	EVT			0		0
Spot 7	21800992	DMSO		20432159	DMSO			0		0
Spot 8	18087496	EVT		16718663	EVT			0		0
Spot 9	6688545	DMSO	L1210	5319712	DMSO	L1210	L1210	0	**L1210**	0
Spot 10	9037219	EVT		7668386	EVT			0		0
Spot 11	28658344	DMSO		27289511	DMSO			0		0
Spot 12	29966696	EVT		28597863	EVT			0		0
Spot 13	1368833	BLANK			BLANK					

Supplementary material S5. Raw and normalized count matrix

Raw count matrix

ID	GeneID	C1498S_rep1	C1498S_rep2	C1498S_rep3	C1498S_rep4	C1498S_rep5	C1498S_rep6	C1498R_rep1	C1498R_rep2	C1498R_rep3	C1498R_rep4	C1498R_rep5	C1498R_rep6
EGFP	EGFP	3	1	0	2	0	2	10	9	10	18	10	8
ENSMUSG00000000001	GNAI3	14	11	6	8	20	13	99	123	71	99	90	74
ENSMUSG00000000028	CDC45	9	13	6	13	9	11	55	55	41	44	44	47
ENSMUSG00000000056	NARF	1	2	3	2	4	2	14	17	14	13	21	15
ENSMUSG00000000058	CAV2	0	0	3	0	0	2	6	2	1	6	3	4
ENSMUSG00000000078	KLF6	10	6	15	17	7	13	41	35	37	39	31	31
ENSMUSG00000000085	SCMH1	8	5	15	18	11	11	33	51	36	51	31	26
ENSMUSG00000000088	COX5A	24	28	31	27	33	23	221	216	144	183	161	170
ENSMUSG00000000093	TBX2	0	0	0	0	0	0	0	0	2	0	0	0
ENSMUSG00000000127	FER	0	1	0	0	0	0	0	0	0	0	0	0
ENSMUSG00000000131	XPO6	8	14	10	10	12	12	52	58	44	70	45	51
ENSMUSG00000000134	TFE3	0	3	1	1	2	4	19	15	7	10	4	12
ENSMUSG00000000142	AXIN2	6	6	10	4	7	5	39	34	19	40	36	33
ENSMUSG00000000148	BRAT1	2	0	3	1	2	2	11	12	12	15	11	14
ENSMUSG00000000149	GNA12	1	1	4	3	2	1	16	22	12	15	17	19
ENSMUSG00000000157	ITGB2L	0	0	0	0	0	1	0	0	0	0	0	0
ENSMUSG00000000159	IGSF5	1	2	5	4	1	3	10	15	11	9	11	8
ENSMUSG00000000167	PIH1D2	0	0	0	0	0	0	0	3	0	2	0	0
ENSMUSG00000000168	DLAT	5	10	4	10	7	2	53	49	39	56	31	46
ENSMUSG00000000171	SDHD	17	20	19	19	25	14	143	174	115	170	122	127
ENSMUSG00000000183	FGF6	0	0	0	0	0	0	0	0	0	0	0	1
ENSMUSG00000000184	CCND2	124	236	166	198	178	193	703	682	458	768	474	550
ENSMUSG00000000194	GPR107	2	5	3	2	6	2	13	15	10	21	8	10
ENSMUSG00000000215	INS2	0	0	0	0	0	0	0	2	0	0	0	0
ENSMUSG00000000223	DRP2	0	0	0	1	0	0	0	5	1	2	0	2
ENSMUSG00000000244	TSPAN32	13	18	13	11	18	15	45	66	30	56	41	59
ENSMUSG00000000253	GMPR	0	0	0	0	0	0	1	0	0	0	0	0
ENSMUSG00000000263	GLRA1	0	0	1	0	0	0	0	0	0	0	1	0
ENSMUSG00000000266	MID2	0	0	0	0	0	0	0	1	0	0	0	0
ENSMUSG00000000275	TRIM25	4	7	3	1	7	2	17	28	21	34	17	23
ENSMUSG00000000276	DGKE	1	0	4	6	4	3	9	14	7	11	8	11
ENSMUSG00000000278	SCPEP1	4	3	3	3	10	4	32	33	26	30	20	27
ENSMUSG00000000282	MNT	4	2	4	4	6	0	21	16	15	22	11	17
ENSMUSG00000000290	ITGB2	11	10	6	10	12	14	45	42	39	54	38	41
ENSMUSG00000000295	HDDC2	7	8	4	1	6	9	41	50	34	56	43	51
ENSMUSG00000000301	PEMT	1	0	0	0	0	0	3	1	1	1	1	1
ENSMUSG00000000308	CKMT1	3	3	3	0	1	1	3	5	5	3	5	1
ENSMUSG00000000325	ARVCF	0	2	4	4	5	7	25	25	14	31	21	22
ENSMUSG00000000326	COMT	103	145	134	169	148	163	337	347	299	405	288	350
ENSMUSG00000000339	RTCA	8	9	9	14	8	16	83	97	64	84	74	83
ENSMUSG00000000340	DBT	13	27	14	29	13	19	84	111	97	119	65	87
ENSMUSG00000000346	DAZAP2	6	5	25	10	20	9	95	86	59	82	70	85
ENSMUSG00000000355	MCTS1	7	7	7	11	10	12	76	94	78	108	61	54
ENSMUSG00000000374	TRAPPC10	2	2	5	5	3	2	19	21	18	30	18	24
ENSMUSG00000000378	CCM2	7	13	9	7	8	7	66	57	57	57	50	47
ENSMUSG00000000384	TBRG4	9	7	10	11	18	11	45	60	40	58	35	44
ENSMUSG00000000386	MX1	0	0	0	1	0	0	0	0	0	0	0	0
ENSMUSG00000000399	NDUFA9	6	14	15	12	11	7	56	74	68	72	48	64
ENSMUSG00000000409	LCK	4	4	3	2	6	4	12	23	15	20	11	8
ENSMUSG00000000416	CTTNBP2	0	1	1	0	0	0	1	1	2	1	0	2
ENSMUSG00000000420	GALNT1	41	46	59	95	53	47	178	187	127	181	148	117
ENSMUSG00000000439	MKRN2	4	6	11	9	3	3	26	28	21	25	24	30
ENSMUSG00000000441	RAF1	7	9	11	11	19	10	62	54	45	67	40	46
ENSMUSG00000000486	37135	4	1	4	3	2	1	20	18	11	13	16	15

ENSMUSG00000000532	ACVR1B	0	2	1	0	0	0	1	1	0	1	0	1
ENSMUSG00000000538	TOM1L2	1	1	3	4	3	2	11	7	5	20	11	14
ENSMUSG00000000552	ZFP385A	2	0	0	0	0	1	3	2	2	2	0	3
ENSMUSG00000000560	GABRA2	0	0	0	0	0	0	0	0	0	1	0	0
ENSMUSG00000000561	WDR77	6	9	5	11	13	6	72	74	53	94	55	70
ENSMUSG00000000562	ADORA3	0	0	0	0	0	0	1	0	0	0	0	0
ENSMUSG00000000563	ATP5F1	47	61	41	67	73	64	545	630	402	570	467	425
ENSMUSG00000000568	HNRNPD	27	39	37	43	44	46	274	297	218	301	180	251
ENSMUSG00000000579	DYNLT1C	0	0	0	2	0	0	0	2	0	1	0	0
ENSMUSG00000000581	C1D	6	10	7	13	10	7	39	61	33	71	26	34
ENSMUSG00000000594	GM2A	0	0	0	1	5	2	0	1	1	0	0	1
ENSMUSG00000000600	KRIT1	25	12	18	19	17	21	87	111	70	108	69	77
ENSMUSG00000000605	CLCN4	4	7	5	2	3	3	18	20	14	16	12	16
ENSMUSG00000000627	SEMA4F	3	3	4	1	1	4	6	13	8	9	3	8
ENSMUSG00000000628	HK2	12	17	17	17	9	18	69	76	56	79	37	61
ENSMUSG00000000631	MYO18A	6	5	2	4	3	5	12	13	16	17	5	18
ENSMUSG00000000673	HAAO	0	0	0	0	0	0	0	1	0	0	0	0
ENSMUSG00000000682	CD52	0	0	0	0	0	0	0	1	0	1	0	0
ENSMUSG00000000686	ABHD15	0	1	1	2	1	0	4	6	5	1	4	4
ENSMUSG00000000693	LOXL3	3	3	3	0	6	2	12	14	8	14	10	13
ENSMUSG00000000706	BTN1A1	0	1	0	0	0	0	0	0	2	0	0	0
ENSMUSG00000000708	KAT2B	6	1	6	3	4	9	23	16	11	30	17	14
ENSMUSG00000000711	RAB5B	0	0	0	0	1	1	4	12	5	4	6	7
ENSMUSG00000000738	SPG7	6	5	5	6	10	7	28	26	29	25	31	26
ENSMUSG00000000739	SULT5A1	0	0	0	0	0	0	0	0	1	0	1	0
ENSMUSG00000000740	RPL13	216	210	235	247	238	249	912	977	710	904	744	771
ENSMUSG00000000743	CHMP1A	1	0	4	1	3	4	14	19	21	19	13	26
ENSMUSG00000000751	RPA1	14	21	12	8	16	9	86	90	73	84	73	79
ENSMUSG00000000759	TUBGCP3	7	4	3	6	7	6	37	30	16	23	24	25
ENSMUSG00000000766	OPRM1	2	1	1	2	0	0	3	0	1	5	0	2
ENSMUSG00000000776	POLR3D	1	2	3	2	2	4	15	13	9	15	3	13
ENSMUSG00000000782	TCF7	51	53	47	68	59	59	323	354	252	365	255	292
ENSMUSG00000000787	DDX3X	29	27	30	33	34	33	249	253	173	307	174	182
ENSMUSG00000000791	IL12RB1	0	1	1	0	0	0	0	0	2	1	0	1
ENSMUSG00000000792	SLC5A5	0	0	0	1	1	0	0	1	1	0	0	1
ENSMUSG00000000804	USP32	0	6	6	7	3	3	21	27	15	36	21	22
ENSMUSG00000000811	TXNRD3	0	0	0	0	0	1	0	0	0	0	0	0
ENSMUSG00000000817	FASL	0	1	0	0	0	0	1	0	0	0	0	0
ENSMUSG00000000823	ZFP512B	8	10	15	15	8	16	76	62	40	50	37	55
ENSMUSG00000000826	DNAJC5	15	18	11	19	22	18	85	76	60	82	72	60
ENSMUSG00000000827	TPD52L2	6	14	10	9	15	6	68	64	52	70	45	59
ENSMUSG00000000838	FMR1	6	11	13	19	15	7	75	68	61	83	54	59
ENSMUSG00000000869	IL4	0	0	0	0	1	1	0	0	0	0	1	0
ENSMUSG00000000876	PXMP4	4	4	12	4	6	3	25	32	24	27	29	35
ENSMUSG00000000881	DLG3	0	2	0	1	0	1	8	4	7	2	3	3
ENSMUSG00000000884	GNB1L	2	1	3	0	1	0	4	8	4	10	4	8
ENSMUSG00000000889	DBH	0	0	1	0	1	0	0	1	1	0	0	2
ENSMUSG00000000902	SMARCB1	3	7	10	11	8	6	44	40	32	51	36	37
ENSMUSG00000000903	VPREB3	0	0	0	0	0	0	0	0	0	1	0	0
ENSMUSG00000000915	HIP1R	4	5	11	9	9	7	45	50	37	33	28	38
ENSMUSG00000000916	NSUN5	8	6	7	12	14	10	50	61	40	61	27	40
ENSMUSG00000000948	GM38393	0	0	0	0	0	1	1	1	2	1	0	0
ENSMUSG00000000958	SLC7A7	0	0	0	0	0	0	2	1	3	0	1	0
ENSMUSG00000000959	OXA1L	5	5	5	8	9	6	48	38	46	49	46	40
ENSMUSG00000000976	HEATR6	4	5	3	4	6	6	42	31	21	36	26	30
ENSMUSG00000001014	ICAM4	0	1	1	0	0	1	3	0	0	1	4	0
ENSMUSG00000001016	ILF2	48	45	55	44	45	37	287	267	223	305	226	243
ENSMUSG00000001017	CHTOP	9	17	12	15	12	15	64	54	54	63	57	55

ENSMUSG00000001018	SNAPIN	26	11	34	32	32	17	105	119	132	103	116	87
ENSMUSG00000001020	S100A4	5	6	7	2	3	5	11	17	13	10	14	9
ENSMUSG00000001021	S100A3	0	0	0	0	0	0	0	0	0	1	0	0
ENSMUSG00000001025	S100A6	1	0	0	0	0	0	1	2	0	2	1	0
ENSMUSG00000001027	SCN4A	0	0	0	0	0	0	0	0	1	0	0	0
ENSMUSG00000001029	ICAM2	6	1	6	4	4	5	18	19	17	20	11	20
ENSMUSG00000001052	SEC24B	6	8	5	9	20	5	64	66	42	76	56	41
ENSMUSG00000001053	N4BP3	0	1	1	1	0	1	4	1	1	1	6	1
ENSMUSG00000001054	RMND5B	1	3	2	3	4	1	9	8	8	19	3	13
ENSMUSG00000001056	NHP2	23	20	16	30	24	31	160	157	124	144	98	146
ENSMUSG00000001062	VPS9D1	2	2	2	2	1	1	10	9	3	5	4	4
ENSMUSG00000001065	ZFP276	0	0	4	3	8	2	10	12	9	16	11	18
ENSMUSG00000001082	MFSD10	3	1	6	4	2	0	3	10	7	3	5	1
ENSMUSG00000001089	LUZP1	0	1	0	0	0	2	1	3	3	3	6	5
ENSMUSG00000001098	KCTD10	5	6	3	2	1	4	26	20	12	14	13	14
ENSMUSG00000001100	POLDIP2	3	4	2	6	4	1	21	17	15	14	18	21
ENSMUSG00000001105	IFT20	7	10	15	11	8	11	46	58	40	68	48	49
ENSMUSG00000001123	LGALS9	3	1	2	2	2	0	3	6	7	5	10	12
ENSMUSG00000001127	ARAF	7	12	6	9	16	9	56	53	45	59	50	49
ENSMUSG00000001128	CFP	0	1	3	4	1	2	7	14	6	2	5	0
ENSMUSG00000001131	TIMP1	0	0	0	0	0	0	0	1	1	1	0	0
ENSMUSG00000001134	UXT	1	0	0	1	2	0	6	4	4	13	3	9
ENSMUSG00000001138	CNNM3	3	8	4	8	2	7	27	34	17	21	23	27
ENSMUSG00000001143	LMAN2L	1	0	2	2	0	1	7	15	8	8	5	5
ENSMUSG00000001150	MCM3AP	3	3	3	2	3	5	15	20	13	32	14	24
ENSMUSG00000001151	PCNT	17	24	16	14	23	19	71	75	61	96	68	54
ENSMUSG00000001156	MXD1	0	3	2	2	0	0	5	13	7	6	4	6
ENSMUSG00000001157	GMCL1	9	5	2	6	6	10	24	22	24	34	11	19
ENSMUSG00000001158	SNRNP27	13	14	8	16	10	17	78	71	49	48	49	47
ENSMUSG00000001166	OAS1C	0	1	1	1	2	0	1	4	2	3	2	0
ENSMUSG00000001173	OCRL	2	1	2	4	2	0	6	5	4	3	3	7
ENSMUSG00000001175	CALM1	192	204	216	224	244	236	849	934	615	972	652	784
ENSMUSG00000001211	AGPAT3	7	8	6	6	13	8	35	48	30	44	31	42
ENSMUSG00000001225	SLC26A3	0	0	0	0	0	0	0	1	1	1	0	0
ENSMUSG00000001227	SEMA6B	0	1	0	2	0	0	0	3	0	3	1	2
ENSMUSG00000001228	UHRF1	34	18	21	25	33	43	160	187	135	190	122	155
ENSMUSG00000001229	DPP9	7	8	5	5	3	8	29	40	24	39	20	21
ENSMUSG00000001240	RAMP2	0	0	0	0	0	0	0	0	1	0	0	0
ENSMUSG00000001247	LSR	0	0	0	1	0	0	1	0	0	2	0	0
ENSMUSG00000001248	GRAMD1A	14	18	17	14	17	22	84	86	64	86	70	62
ENSMUSG00000001249	HPN	0	0	0	0	0	0	0	1	0	0	0	0
ENSMUSG00000001280	SP1	1	3	3	5	3	4	19	23	15	25	18	18
ENSMUSG00000001281	ITGB7	10	17	20	23	14	14	78	114	78	95	72	89
ENSMUSG00000001285	MYG1	8	3	9	4	10	8	47	51	41	50	24	37
ENSMUSG00000001288	RARG	2	0	2	2	2	0	9	4	8	9	5	1
ENSMUSG00000001289	PFDN5	17	30	32	23	28	16	104	109	90	129	109	102
ENSMUSG00000001300	EFNB2	0	0	0	0	0	0	0	1	0	0	0	0
ENSMUSG00000001305	RRP15	4	6	2	7	3	3	37	41	27	32	23	41
ENSMUSG00000001313	RND2	0	0	0	0	0	0	1	0	1	0	2	0
ENSMUSG00000001323	SRR	3	6	2	0	1	4	10	10	8	18	8	12
ENSMUSG00000001333	SYNC	1	3	2	3	2	2	5	10	6	8	11	9
ENSMUSG00000001334	FNDC5	0	1	0	0	0	1	0	1	0	1	2	0
ENSMUSG00000001349	CNN1	0	0	0	0	0	0	0	0	0	0	1	0
ENSMUSG00000001366	FBXO9	3	6	4	3	3	2	22	30	19	24	23	25
ENSMUSG00000001376	VPS50	7	6	7	9	6	9	39	29	25	34	22	34
ENSMUSG00000001380	HARS	17	15	27	24	17	12	97	126	88	119	96	84
ENSMUSG00000001383	ZMAT2	17	19	20	21	23	25	97	151	94	100	92	125
ENSMUSG00000001403	UBE2C	5	13	7	10	7	10	81	52	50	52	54	58

ENSMUSG00000001415	SMG5	12	26	18	24	24	16	75	108	65	89	66	86
ENSMUSG00000001416	CCT3	35	55	55	66	56	47	307	368	302	371	303	299
ENSMUSG00000001418	GLMP	3	2	5	5	4	5	23	27	23	33	16	26
ENSMUSG00000001419	MEF2D	11	6	6	11	7	12	46	65	51	70	49	52
ENSMUSG00000001420	TMEM79	2	1	0	2	0	0	2	2	1	2	4	1
ENSMUSG00000001424	SND1	154	160	183	180	185	188	892	1019	733	929	581	835
ENSMUSG00000001436	SLC19A1	3	2	2	3	0	1	20	15	4	16	11	12
ENSMUSG00000001440	KPNB1	33	48	41	55	42	54	233	278	208	308	192	238
ENSMUSG00000001441	NPEPPS	19	25	8	23	22	15	89	79	80	109	74	69
ENSMUSG00000001445	MRPL10	0	2	3	6	6	4	34	29	25	43	20	28
ENSMUSG00000001467	CYP51	10	11	14	17	22	10	96	102	81	106	74	89
ENSMUSG00000001472	TCF25	19	39	28	23	40	34	143	156	106	152	129	127
ENSMUSG00000001482	DEF8	1	2	0	4	1	2	13	9	11	10	7	11
ENSMUSG00000001494	SOST	1	0	0	0	0	0	0	0	0	0	0	0
ENSMUSG00000001517	FOXM1	2	5	6	9	9	12	30	33	18	29	29	26
ENSMUSG00000001518	ITFG2	5	7	9	2	4	2	21	22	13	19	16	20
ENSMUSG00000001520	NRIP2	0	0	0	0	1	0	0	0	0	0	0	1
ENSMUSG00000001521	TULP3	1	6	0	6	8	4	12	7	12	10	5	4
ENSMUSG00000001524	GTF2H4	3	4	9	5	9	5	33	30	25	40	19	30
ENSMUSG00000001525	TUBB5	95	167	138	185	191	197	669	766	555	726	487	667
ENSMUSG00000001542	ELL2	3	2	3	4	4	7	37	33	32	27	28	15
ENSMUSG00000001552	JUP	0	0	1	0	0	0	0	0	0	0	0	0
ENSMUSG00000001558	KLHL10	0	0	1	0	0	0	0	0	0	0	0	0
ENSMUSG00000001569	NOM1	10	9	6	10	8	10	57	59	35	58	60	55
ENSMUSG00000001576	ERGIC1	5	11	12	12	15	8	61	81	68	79	52	65
ENSMUSG00000001583	TNK1	0	0	0	0	0	0	0	1	0	0	1	0
ENSMUSG00000001588	ACAP1	15	20	13	21	22	14	72	81	57	57	59	57
ENSMUSG00000001604	TCEA3	0	0	0	0	0	0	0	1	0	0	0	0
ENSMUSG00000001627	IFRD1	7	14	13	19	13	14	107	115	74	117	82	62
ENSMUSG00000001630	STK38L	2	4	2	4	2	0	8	14	6	12	17	14
ENSMUSG00000001632	BRPF1	15	17	11	29	15	17	61	80	57	98	55	62
ENSMUSG00000001642	AKR1B3	3	0	1	0	3	2	3	11	5	10	6	9
ENSMUSG00000001657	HOXC8	2	0	2	2	3	3	7	4	1	6	4	6
ENSMUSG00000001661	HOXC6	2	2	2	3	3	4	13	5	7	22	10	10
ENSMUSG00000001663	GSTT1	0	0	0	0	0	0	0	1	0	1	0	0
ENSMUSG00000001666	DDT	3	3	1	7	2	5	26	24	15	19	6	25
ENSMUSG00000001674	DDX18	11	12	11	25	19	16	45	50	46	69	45	55
ENSMUSG00000001687	UBL3	25	32	33	33	35	24	156	205	149	224	149	159
ENSMUSG00000001707	EEF1E1	14	23	13	16	26	21	113	146	88	131	80	95
ENSMUSG00000001729	AKT1	14	12	13	14	16	17	71	81	49	86	54	79
ENSMUSG00000001741	IL16	23	26	22	28	32	27	152	161	99	146	131	108
ENSMUSG00000001750	TCIRG1	5	4	6	4	7	5	30	30	34	40	17	22
ENSMUSG00000001751	NAGLU	0	0	0	0	0	0	0	1	1	0	0	0
ENSMUSG00000001755	PPAT	0	3	0	0	3	1	11	6	10	8	8	6
ENSMUSG00000001763	TSPAN33	0	0	1	1	0	0	3	1	1	1	0	1
ENSMUSG00000001767	CRNKL1	9	5	2	3	9	6	46	41	43	41	25	31
ENSMUSG00000001774	CHORDC1	6	9	9	11	14	13	46	58	33	56	46	53
ENSMUSG00000001783	RTCB	7	11	13	17	16	4	71	60	33	51	35	52
ENSMUSG00000001785	PWP1	3	4	7	2	6	4	31	32	30	25	23	37
ENSMUSG00000001786	FBXO7	2	2	4	3	2	1	9	20	13	16	17	12
ENSMUSG00000001794	CAPNS1	2	9	7	10	10	9	45	41	33	50	41	56
ENSMUSG00000001819	HOXD13	0	0	0	0	0	0	0	0	0	0	0	1
ENSMUSG00000001829	CLPB	6	2	4	5	6	5	20	16	19	32	14	27
ENSMUSG00000001833	39326	23	53	35	49	44	44	275	256	220	368	207	229
ENSMUSG00000001844	ZDHHC4	2	2	1	4	2	2	9	9	7	12	8	9
ENSMUSG00000001847	RAC1	22	21	19	27	27	22	121	124	121	109	83	128
ENSMUSG00000001855	NUP214	4	8	8	9	11	6	34	42	27	40	27	29
ENSMUSG00000001864	AIF1L	0	0	0	1	0	0	0	0	0	0	0	1

ENSMUSG00000001865	CPA3	38	56	40	53	56	45	291	277	229	288	239	297
ENSMUSG00000001891	UGP2	6	10	9	15	6	7	66	55	44	64	39	39
ENSMUSG00000001909	TRMT1	14	23	22	23	27	17	86	92	71	99	68	84
ENSMUSG00000001910	NACC1	3	6	5	6	6	5	37	33	20	30	12	30
ENSMUSG00000001911	NFIX	1	2	1	0	1	0	3	5	2	6	3	4
ENSMUSG00000001918	SLC1A5	34	42	50	51	40	29	204	228	150	202	162	175
ENSMUSG00000001924	UBA1	13	15	20	19	12	11	84	97	66	105	84	89
ENSMUSG00000001942	SIAE	2	4	4	3	1	3	18	14	16	17	15	13
ENSMUSG00000001946	ESAM	1	0	0	0	0	0	0	0	0	0	0	1
ENSMUSG00000001948	SPA17	1	1	3	0	1	3	8	15	4	12	11	15
ENSMUSG00000001962	FAM50A	4	7	3	3	2	5	27	34	18	27	21	17
ENSMUSG00000001964	EMD	5	7	2	2	1	3	21	43	34	38	28	31
ENSMUSG00000001983	TACO1	0	0	0	0	1	0	1	3	2	1	1	0
ENSMUSG00000001986	GRIA3	1	0	0	0	0	0	0	0	0	0	0	0
ENSMUSG00000001988	NPAS1	0	0	0	0	0	0	0	0	0	0	1	0
ENSMUSG00000001998	AP4E1	0	2	1	0	3	4	3	5	3	9	3	3
ENSMUSG00000001999	BLVRA	2	1	10	11	9	4	44	44	40	30	25	36
ENSMUSG00000002006	PDZD4	0	0	0	1	1	0	2	1	2	0	0	3
ENSMUSG00000002007	SRPK3	0	0	1	0	0	0	1	0	0	2	2	0
ENSMUSG00000002010	IDH3G	11	3	12	5	7	11	45	53	38	58	44	54
ENSMUSG00000002014	SSR4	14	27	26	29	28	29	157	172	119	176	106	146
ENSMUSG00000002015	BCAP31	10	9	5	15	11	8	61	72	56	83	63	83
ENSMUSG00000002017	FAM98A	7	3	2	4	1	4	22	29	16	23	11	20
ENSMUSG00000002028	KMT2A	48	61	36	61	57	58	158	164	127	225	118	143
ENSMUSG00000002031	IFT46	3	2	8	3	3	3	19	14	6	18	13	11
ENSMUSG00000002032	TMEM25	0	0	0	0	0	2	1	0	0	2	3	0
ENSMUSG00000002033	CD3G	103	115	116	120	95	93	629	641	501	585	437	574
ENSMUSG00000002043	TRAPPC6A	1	1	1	1	4	2	8	7	7	8	10	10
ENSMUSG00000002052	SUPT6	6	9	14	21	17	13	48	64	57	78	59	53
ENSMUSG00000002055	SPAG5	11	12	13	17	19	14	69	81	70	71	50	59
ENSMUSG00000002058	UNC119	0	2	1	3	0	0	9	5	5	9	2	3
ENSMUSG00000002059	RAB34	0	0	0	0	0	0	0	0	0	1	0	0
ENSMUSG00000002064	SDF2	0	3	1	1	3	3	17	17	11	18	16	13
ENSMUSG00000002068	CCNE1	6	4	6	7	4	4	33	25	12	23	13	17
ENSMUSG00000002083	BBC3	14	9	8	12	9	8	50	54	36	42	40	47
ENSMUSG00000002102	PSMC3	21	23	21	22	20	17	108	95	86	123	83	120
ENSMUSG00000002103	ACP2	1	3	1	4	6	7	20	14	12	46	5	14
ENSMUSG00000002104	RAPSN	4	2	2	9	3	5	16	9	4	10	10	17
ENSMUSG00000002105	SLC39A13	0	0	0	1	0	2	3	5	2	2	1	4
ENSMUSG00000002107	CELF2	104	138	143	132	129	127	530	482	394	583	398	419
ENSMUSG00000002108	NR1H3	0	0	0	0	0	0	0	0	0	0	2	1
ENSMUSG00000002109	DDB2	11	7	9	10	11	16	46	47	38	77	32	41
ENSMUSG00000002111	SPI1	0	0	0	0	0	0	0	0	1	0	0	0
ENSMUSG00000002129	SF3A1	10	15	13	12	16	10	89	82	79	83	61	61
ENSMUSG00000002147	STAT6	8	8	5	11	7	7	34	41	25	26	20	36
ENSMUSG00000002204	NAPSA	0	0	1	0	0	0	2	0	2	0	2	0
ENSMUSG00000002205	VRK3	4	3	3	3	4	3	16	19	13	19	10	16
ENSMUSG00000002210	SMG9	0	4	3	1	4	2	11	7	9	10	7	10
ENSMUSG00000002221	PAXIP1	12	9	9	10	9	9	52	45	28	58	37	38
ENSMUSG00000002222	RMND5A	26	21	19	23	19	27	123	100	69	105	77	73
ENSMUSG00000002227	MOV10	14	15	17	10	11	21	44	63	51	49	41	39
ENSMUSG00000002228	PPM1J	0	0	0	1	0	0	0	0	0	0	0	1
ENSMUSG00000002233	RHOC	0	0	0	1	0	0	2	5	0	1	0	2
ENSMUSG00000002250	PPARD	3	4	8	6	7	3	28	34	12	13	13	24
ENSMUSG00000002257	DEF6	9	10	17	21	14	18	71	76	55	72	71	64
ENSMUSG00000002274	METRN	16	13	22	21	14	15	94	90	69	90	68	71
ENSMUSG00000002279	LMF1	2	3	5	7	3	1	12	15	12	8	8	14
ENSMUSG00000002280	NARFL	4	2	5	2	2	5	17	15	15	15	19	12

ENSMUSG00000002289	ANGPTL4	5	3	4	3	4	1	21	18	20	16	18	17
ENSMUSG00000002297	DBF4	22	25	22	30	26	22	113	92	88	124	97	86
ENSMUSG00000002307	DAXX	7	15	8	8	9	8	41	55	28	55	33	49
ENSMUSG00000002308	CD320	1	3	9	6	7	4	24	20	13	26	16	20
ENSMUSG00000002319	IPO4	13	6	5	20	20	15	51	64	54	59	39	48
ENSMUSG00000002320	TM9SF1	1	0	1	0	1	3	4	7	2	6	4	3
ENSMUSG00000002325	IRF9	1	3	1	1	4	0	3	5	3	6	5	2
ENSMUSG00000002326	GMPR2	2	5	2	3	5	5	20	20	15	27	12	12
ENSMUSG00000002329	MDP1	4	4	4	6	6	5	27	33	18	21	30	22
ENSMUSG00000002332	DHRS1	1	2	2	4	3	0	12	13	4	15	9	7
ENSMUSG00000002342	TMEM161A	6	6	6	11	15	5	45	54	48	43	40	51
ENSMUSG00000002343	ARMC6	4	3	3	3	1	1	9	9	12	10	8	6
ENSMUSG00000002345	BORCS8	3	1	2	2	2	2	7	12	9	5	9	9
ENSMUSG00000002346	SLC25A42	0	1	0	0	1	1	3	6	8	7	0	4
ENSMUSG00000002365	SNX9	3	3	2	7	2	5	12	29	15	19	10	20
ENSMUSG00000002372	RANBP3	19	29	22	27	31	28	105	127	103	146	101	106
ENSMUSG00000002379	NDUFA11	14	14	15	13	25	19	101	115	101	111	86	97
ENSMUSG00000002393	NR2F6	1	2	5	5	4	2	10	10	13	18	13	14
ENSMUSG00000002395	USE1	6	5	2	6	4	11	30	42	27	37	32	39
ENSMUSG00000002396	OCEL1	0	2	2	3	1	1	4	2	4	4	6	3
ENSMUSG00000002409	DYRK1B	1	0	0	2	0	1	2	2	1	4	0	2
ENSMUSG00000002413	BRAF	15	16	27	24	13	12	60	71	64	46	65	63
ENSMUSG00000002416	NDUFB2	17	19	19	18	24	9	87	90	80	98	57	105
ENSMUSG00000002428	HLTF	6	15	11	18	19	11	73	74	55	94	56	61
ENSMUSG00000002455	PRPF6	7	8	10	8	10	5	52	58	45	52	38	57
ENSMUSG00000002458	RGS19	0	1	2	5	2	0	19	29	24	27	12	12
ENSMUSG00000002459	RGS20	0	0	0	0	0	0	0	1	0	0	0	0
ENSMUSG00000002477	SNRPD1	29	39	30	41	49	34	269	276	206	264	214	287
ENSMUSG00000002486	TCHP	0	4	2	3	4	2	18	22	11	17	13	8
ENSMUSG00000002489	TIAM1	2	9	5	9	9	7	33	20	23	24	18	28
ENSMUSG00000002496	TSC2	1	6	6	6	9	7	26	29	25	27	22	25
ENSMUSG00000002524	PUF60	12	15	9	18	22	20	87	125	78	110	97	87
ENSMUSG00000002546	GOLGA2	9	8	10	16	16	11	49	38	32	45	46	42
ENSMUSG00000002550	UCK1	3	2	4	3	1	2	19	16	14	21	14	21
ENSMUSG00000002578	IKZF4	0	2	0	1	0	0	0	0	0	0	0	0
ENSMUSG00000002580	MIEN1	0	0	3	2	0	1	0	0	0	0	1	0
ENSMUSG00000002602	AXL	1	0	2	1	3	2	5	5	2	3	3	3
ENSMUSG00000002603	TGFB1	1	1	3	4	2	2	9	8	13	9	14	10
ENSMUSG00000002608	CCDC97	5	3	6	5	5	5	20	31	23	22	15	26
ENSMUSG00000002617	ZFP40	3	5	5	7	4	4	20	20	14	33	14	25
ENSMUSG00000002625	AKAP8L	0	1	1	0	0	3	1	3	3	4	3	9
ENSMUSG00000002635	PDCD2L	9	2	11	4	6	8	39	59	38	39	33	25
ENSMUSG00000002658	GTF2F1	19	35	27	35	27	29	119	132	107	153	118	132
ENSMUSG00000002660	CLPP	13	10	10	9	18	9	59	68	57	69	51	42
ENSMUSG00000002661	ALKBH7	9	6	12	17	16	7	50	49	47	37	41	35
ENSMUSG00000002668	DENND1C	15	16	21	21	23	10	81	106	72	94	74	89
ENSMUSG00000002679	MED6	3	2	9	5	5	10	33	36	26	31	24	19
ENSMUSG00000002688	PRKD1	0	0	0	0	0	0	0	0	0	0	1	0
ENSMUSG00000002699	LCP2	10	14	10	11	7	9	63	59	38	81	40	55
ENSMUSG00000002718	CSE1L	5	21	10	10	6	5	68	68	51	82	48	47
ENSMUSG00000002728	NAA20	7	5	7	10	7	4	34	58	39	42	30	51
ENSMUSG00000002731	PRKRA	1	2	2	1	1	1	15	10	13	7	6	6
ENSMUSG00000002732	FKBP7	1	1	1	0	0	0	9	8	7	10	2	6
ENSMUSG00000002733	PLEKHA3	19	21	20	32	25	13	145	176	139	155	106	143
ENSMUSG00000002741	YKT6	5	9	3	8	6	2	19	35	25	33	20	32
ENSMUSG00000002748	BAZ1B	88	92	111	136	129	112	497	469	345	524	388	386
ENSMUSG00000002763	PEX6	13	7	8	10	7	10	31	22	38	26	31	29
ENSMUSG00000002767	MRPL2	1	7	8	5	14	11	54	71	37	52	43	46

ENSMUSG00000002768	MEA1	2	3	5	1	2	3	26	28	17	22	18	13
ENSMUSG00000002769	GNMT	0	0	0	0	0	0	0	0	2	2	0	2
ENSMUSG00000002778	KDELR1	11	16	16	17	18	15	107	105	83	87	60	81
ENSMUSG00000002781	TMEM143	1	0	0	0	1	0	2	5	1	1	0	1
ENSMUSG00000002797	GGCT	5	4	3	6	3	2	21	18	19	18	18	29
ENSMUSG00000002799	JAG2	0	0	0	0	0	1	2	1	0	1	0	2
ENSMUSG00000002803	BTBD6	1	1	1	0	2	1	2	5	2	5	3	1
ENSMUSG00000002804	NUDT14	1	0	1	1	2	0	5	2	3	1	4	3
ENSMUSG00000002812	FLII	10	9	14	18	15	9	43	57	39	47	49	55
ENSMUSG00000002814	TOP3A	1	1	1	2	4	1	10	8	6	11	7	12
ENSMUSG00000002820	ATG4D	7	10	6	8	11	7	31	20	27	32	29	30
ENSMUSG00000002825	QTRT1	5	7	8	11	3	7	39	39	32	39	32	39
ENSMUSG00000002831	PLIN4	0	0	2	0	3	2	5	3	3	2	2	0
ENSMUSG00000002833	HDGFL2	13	22	15	25	27	22	68	105	75	85	80	82
ENSMUSG00000002835	CHAF1A	16	21	19	25	26	13	100	124	90	112	87	88
ENSMUSG00000002844	ADPRH	3	3	2	3	3	2	13	14	14	18	10	17
ENSMUSG00000002845	TMEM39A	2	7	7	7	3	6	38	41	27	38	23	33
ENSMUSG00000002846	TIMMDC1	12	7	10	13	11	13	39	36	29	35	25	24
ENSMUSG00000002847	PLA1A	0	1	0	0	0	0	4	2	0	2	3	1
ENSMUSG00000002870	MCM2	20	47	35	42	30	37	235	243	164	372	194	184
ENSMUSG00000002871	TPRA1	3	5	4	3	4	4	15	20	15	25	23	17
ENSMUSG00000002881	NAB1	11	11	17	16	17	17	59	53	48	92	47	57
ENSMUSG00000002885	ADGRE5	8	7	9	7	12	13	48	42	51	43	34	53
ENSMUSG00000002897	IL17RA	12	8	13	24	12	15	59	70	43	60	40	57
ENSMUSG00000002900	LAMB1	0	0	0	0	0	0	0	0	1	0	0	0
ENSMUSG00000002908	KCNN1	0	0	0	0	0	0	0	0	0	1	0	0
ENSMUSG00000002910	ARRDC2	0	1	1	6	4	0	6	5	7	4	4	5
ENSMUSG00000002944	CD36	0	0	0	1	0	0	0	0	0	0	0	0
ENSMUSG00000002948	MAP2K7	11	4	4	5	4	7	23	32	25	31	27	28
ENSMUSG00000002949	TIMM44	8	16	16	13	12	13	66	62	51	49	58	37
ENSMUSG00000002957	AP2A2	9	7	16	6	14	9	59	74	56	65	43	58
ENSMUSG00000002963	PNKP	3	1	5	2	1	3	11	15	10	12	12	9
ENSMUSG00000002968	MED25	10	12	10	8	7	9	42	40	32	56	28	37
ENSMUSG00000002980	BCAM	0	0	0	0	0	0	0	0	1	0	0	0
ENSMUSG00000002981	CLPTM1	7	9	4	7	9	16	57	75	58	72	46	66
ENSMUSG00000002983	RELB	5	2	0	0	2	1	3	5	5	1	2	3
ENSMUSG00000002984	TOMM40	19	19	16	22	19	37	113	116	92	90	94	99
ENSMUSG00000002985	APOE	4	3	4	6	4	5	16	18	20	8	11	14
ENSMUSG00000002996	HBP1	3	8	5	5	4	8	29	22	21	29	31	22
ENSMUSG00000002997	PRKAR2B	0	1	0	2	0	2	8	3	6	8	8	4
ENSMUSG00000003031	CDKN1B	36	53	41	67	50	56	327	309	256	358	231	272
ENSMUSG00000003033	AP1M1	4	7	5	7	7	7	23	23	14	18	14	18
ENSMUSG00000003037	RAB8A	9	20	9	14	19	11	73	88	64	72	53	71
ENSMUSG00000003038	HMGN2	3	5	2	5	5	7	34	31	21	35	25	37
ENSMUSG00000003039	FAM32A	3	10	10	14	18	6	72	73	39	63	59	54
ENSMUSG00000003051	ELF3	0	0	0	0	0	0	0	0	0	0	1	0
ENSMUSG00000003062	STARD3NL	4	5	7	9	8	6	29	26	21	38	20	33
ENSMUSG00000003068	STK11	12	13	8	26	22	14	67	82	55	93	70	60
ENSMUSG00000003070	EFNA2	0	0	0	0	0	0	0	1	1	1	1	0
ENSMUSG00000003072	ATP5D	30	31	35	43	31	27	158	190	149	179	124	181
ENSMUSG00000003099	PPP5C	30	37	24	41	39	34	141	143	114	170	105	143
ENSMUSG00000003119	CDK12	17	17	20	22	27	24	89	81	57	120	74	90
ENSMUSG00000003123	LIPE	3	2	3	2	2	2	9	11	5	9	3	8
ENSMUSG00000003131	PAFAH1B2	13	7	13	17	21	19	89	94	83	96	47	85
ENSMUSG00000003134	TBC1D8	3	2	2	2	1	2	15	12	8	7	9	9
ENSMUSG00000003135	CNOT11	5	5	12	13	9	15	54	53	45	83	52	47
ENSMUSG00000003153	SLC2A3	8	7	12	16	6	12	46	59	35	50	50	47
ENSMUSG00000003154	FOXJ2	6	2	3	3	6	7	9	12	10	16	4	12

ENSMUSG00000003161	SRI	9	19	11	17	16	24	98	103	72	92	87	79
ENSMUSG00000003166	DGCR2	4	4	8	7	8	8	37	27	21	37	27	17
ENSMUSG00000003184	IRF3	4	5	5	4	6	7	25	40	25	26	25	28
ENSMUSG00000003190	BCL2L12	3	5	3	3	4	8	21	22	28	23	17	17
ENSMUSG00000003198	ZFP959	1	1	0	3	3	2	5	10	9	12	2	5
ENSMUSG00000003199	MPND	5	6	6	10	12	7	39	47	29	30	29	46
ENSMUSG00000003200	SH3GL1	3	7	6	7	7	8	31	39	21	31	25	39
ENSMUSG00000003206	EBI3	0	0	0	0	0	0	0	0	1	0	0	0
ENSMUSG00000003208	CCDC94	2	0	1	2	6	3	15	14	13	7	10	13
ENSMUSG00000003226	RANBP2	20	31	22	26	26	17	98	116	96	124	87	95
ENSMUSG00000003228	GRK5	0	1	3	1	0	0	10	4	4	7	11	2
ENSMUSG00000003233	DVL3	5	4	7	8	6	6	21	31	15	25	22	26
ENSMUSG00000003234	ABCF3	3	3	3	6	3	2	17	11	6	15	9	15
ENSMUSG00000003235	EIF2B5	6	2	4	3	10	6	39	37	21	31	23	35
ENSMUSG00000003269	CYTH2	1	5	7	3	5	2	23	24	27	32	18	25
ENSMUSG00000003271	SULT2B1	0	0	0	0	0	0	2	7	2	3	1	4
ENSMUSG00000003279	DLGAP1	0	0	1	0	0	0	0	1	0	0	0	0
ENSMUSG00000003282	PLAG1	0	0	0	0	0	0	0	0	0	2	0	0
ENSMUSG00000003299	MRPL4	4	6	2	2	10	4	27	28	20	29	24	19
ENSMUSG00000003308	KEAP1	4	8	5	10	10	13	42	42	49	48	43	42
ENSMUSG00000003309	AP1M2	1	1	1	0	1	0	5	7	7	8	4	5
ENSMUSG00000003316	GLG1	14	17	12	24	18	17	72	73	53	95	54	70
ENSMUSG00000003341	ATP8B3	0	1	0	0	0	0	0	0	0	0	0	0
ENSMUSG00000003344	BTBD2	0	0	0	1	1	1	3	3	1	0	1	1
ENSMUSG00000003345	CSNK1G2	8	5	9	6	12	5	38	40	35	54	30	32
ENSMUSG00000003346	ABHD17A	8	7	11	14	9	7	55	63	45	58	41	53
ENSMUSG00000003348	MOB3A	2	5	9	9	14	5	45	43	36	35	28	43
ENSMUSG00000003354	CCDC65	0	0	0	0	0	0	0	0	0	1	0	0
ENSMUSG00000003355	FKBP11	0	0	0	0	0	0	2	0	0	2	0	3
ENSMUSG00000003360	DDX23	41	42	38	58	55	48	175	155	181	181	129	172
ENSMUSG00000003363	PLD3	1	1	4	6	2	1	8	15	7	16	6	8
ENSMUSG00000003378	GRIK5	2	1	0	2	3	2	6	7	2	5	4	1
ENSMUSG00000003379	CD79A	0	0	0	0	0	0	1	0	0	1	1	1
ENSMUSG00000003380	RABAC1	0	7	5	5	5	4	34	37	16	30	27	23
ENSMUSG00000003382	ETV3	10	17	18	23	17	21	58	53	39	75	57	38
ENSMUSG00000003402	PRKCSH	10	7	9	10	3	10	52	69	40	47	48	63
ENSMUSG00000003410	ELAVL3	0	0	0	1	0	1	1	1	0	1	2	2
ENSMUSG00000003421	NOSIP	3	5	6	9	4	4	51	38	13	29	23	32
ENSMUSG00000003423	PIH1D1	3	0	1	2	4	3	6	10	7	14	9	10
ENSMUSG00000003429	RPS11	105	101	117	118	120	135	641	717	538	703	558	636
ENSMUSG00000003435	SUPT5	12	10	19	17	14	19	66	87	49	77	74	53
ENSMUSG00000003436	DLL3	0	0	0	0	0	0	0	2	0	0	0	0
ENSMUSG00000003437	PAF1	9	9	9	13	16	7	52	50	48	69	42	55
ENSMUSG00000003438	TIMM50	8	10	5	6	5	7	35	39	29	42	27	31
ENSMUSG00000003444	MED29	0	1	1	0	0	0	7	1	1	8	0	5
ENSMUSG00000003452	BICD1	2	5	1	7	4	3	30	23	11	37	17	19
ENSMUSG00000003458	NCSTN	3	6	8	6	4	8	29	30	27	31	21	25
ENSMUSG00000003464	PEX19	2	11	9	8	6	13	62	69	49	71	51	53
ENSMUSG00000003500	IMPDH1	6	11	10	7	5	4	26	26	34	38	22	26
ENSMUSG00000003526	PRODH	0	0	1	0	0	0	0	0	0	0	0	0
ENSMUSG00000003527	DGCR14	2	0	2	1	2	0	9	9	9	5	6	9
ENSMUSG00000003528	SLC25A1	2	6	6	6	7	4	27	17	16	20	17	26
ENSMUSG00000003531	DGCR6	0	2	1	0	2	3	27	12	11	16	9	12
ENSMUSG00000003541	IER3	0	0	0	0	0	0	0	0	0	1	0	0
ENSMUSG00000003545	FOSB	0	0	0	0	0	0	1	2	1	0	0	1
ENSMUSG00000003546	KLC4	1	1	1	2	2	1	8	5	3	11	8	14
ENSMUSG00000003549	ERCC1	1	1	1	1	2	3	7	10	8	10	8	7
ENSMUSG00000003559	AS3MT	0	0	0	0	0	0	0	0	1	0	1	0

ENSMUSG00000003573	HOMER3	0	1	1	1	0	1	2	6	2	7	4	5
ENSMUSG00000003575	CRTC1	1	0	1	1	1	1	10	7	5	11	9	11
ENSMUSG00000003581	RNF215	1	1	2	1	0	4	5	7	11	6	4	7
ENSMUSG00000003604	AVEN	6	4	2	2	2	4	21	16	8	32	14	12
ENSMUSG00000003617	CP	0	0	0	1	0	0	2	1	0	2	1	1
ENSMUSG00000003623	CROT	12	13	11	14	6	9	55	66	32	48	40	38
ENSMUSG00000003644	RPS6KA1	0	0	1	0	0	1	0	0	1	1	0	0
ENSMUSG00000003660	SNRNP200	8	11	10	10	11	15	76	85	42	67	44	54
ENSMUSG00000003662	CIAO1	5	3	3	11	9	3	32	32	38	38	24	29
ENSMUSG00000003665	HAS1	0	0	0	0	0	0	0	0	0	0	0	1
ENSMUSG00000003680	TAF6L	1	2	2	0	0	3	7	12	11	4	8	11
ENSMUSG00000003721	INSIG2	4	7	7	12	8	9	36	30	23	24	30	30
ENSMUSG00000003731	KPNA6	16	5	5	7	11	11	65	71	44	53	53	46
ENSMUSG00000003746	MAN1A	2	3	2	0	6	1	5	9	7	15	11	11
ENSMUSG00000003752	ITPKC	1	0	1	1	2	1	5	3	2	1	2	1
ENSMUSG00000003762	COQ8B	0	0	0	0	1	0	4	4	3	4	2	2
ENSMUSG00000003778	BRD8	14	33	26	41	35	29	133	124	98	143	115	115
ENSMUSG00000003779	KIF20A	1	3	5	4	8	5	27	30	24	37	20	24
ENSMUSG00000003808	FARSA	8	9	9	16	7	9	48	57	44	64	48	60
ENSMUSG00000003809	GCDH	3	2	4	3	2	4	18	16	6	19	18	11
ENSMUSG00000003810	MAST2	8	8	11	11	16	8	46	35	39	47	34	39
ENSMUSG00000003812	DNASE2A	3	3	6	8	3	4	14	12	14	12	12	16
ENSMUSG00000003813	RAD23A	11	13	10	21	13	6	42	51	37	59	36	58
ENSMUSG00000003814	CALR	73	110	93	117	106	98	584	594	468	596	434	530
ENSMUSG00000003824	SYCE2	1	1	0	0	0	0	0	0	1	2	0	1	0
ENSMUSG00000003847	NFAT5	8	12	7	11	12	11	48	34	31	53	26	31
ENSMUSG00000003848	NOB1	7	13	5	6	11	7	45	36	28	51	27	45
ENSMUSG00000003849	NQO1	0	1	0	0	2	1	1	1	4	4	0	4
ENSMUSG00000003863	PPFIA3	0	0	0	0	0	0	0	0	0	1	0	0
ENSMUSG00000003865	GYS1	8	1	1	7	5	2	9	7	6	10	7	16
ENSMUSG00000003868	RUVBL2	12	14	9	15	10	15	73	82	55	89	59	82
ENSMUSG00000003872	LIN7B	0	1	0	0	0	0	0	0	1	0	0	0
ENSMUSG00000003873	BAX	10	10	8	16	17	11	55	58	49	52	57	57
ENSMUSG00000003882	IL7R	0	0	1	1	0	0	0	0	1	1	0	0
ENSMUSG00000003923	TFAM	10	14	10	7	18	11	85	91	48	92	49	41
ENSMUSG00000003929	ZFP81	5	8	6	16	5	4	28	29	25	31	16	27
ENSMUSG00000003934	EFNB3	1	1	0	2	1	0	1	1	1	5	0	5
ENSMUSG00000003948	MMD	1	7	4	7	4	5	26	22	19	19	13	22
ENSMUSG00000003955	FAM162A	14	8	17	17	18	11	128	107	105	102	75	74
ENSMUSG00000003970	RPL8	220	270	237	301	311	262	1103	1171	898	1145	874	1113
ENSMUSG00000003974	GRM3	0	0	0	0	0	0	0	0	1	0	0	0
ENSMUSG00000004018	FANCL	4	3	4	6	9	7	25	25	26	25	16	26
ENSMUSG00000004040	STAT3	14	9	13	14	10	7	50	56	52	79	54	52
ENSMUSG00000004043	STAT5A	1	5	0	4	4	3	14	18	13	13	10	13
ENSMUSG00000004044	CAVIN1	0	0	0	0	0	0	0	0	1	0	0	0
ENSMUSG00000004054	MAP3K11	4	4	3	5	2	1	14	21	9	14	10	9
ENSMUSG00000004056	AKT2	2	2	2	4	1	1	16	18	7	20	9	17
ENSMUSG00000004069	DNAJA3	14	15	7	15	6	11	59	61	58	59	35	48
ENSMUSG00000004070	HMOX2	7	8	11	13	13	8	58	69	47	66	57	64
ENSMUSG00000004071	CDIP1	4	12	12	9	11	5	48	61	28	46	34	35
ENSMUSG00000004096	CWC15	17	26	18	26	19	21	169	168	103	149	114	141
ENSMUSG00000004099	DNMT1	40	57	51	62	53	42	218	216	175	261	208	220
ENSMUSG00000004100	PPAN	20	15	20	26	15	24	135	141	100	143	115	108
ENSMUSG00000004105	ANGPTL2	0	0	2	0	2	0	1	2	2	1	0	1
ENSMUSG00000004113	CACNA1B	0	0	0	0	1	0	0	0	4	0	1	0
ENSMUSG00000004127	TRMT10A	4	10	11	10	9	14	55	55	37	53	40	35
ENSMUSG00000004187	KIFC2	1	0	0	0	1	0	0	0	2	0	0	0
ENSMUSG00000004207	PSAP	1	0	0	0	1	1	13	17	9	22	13	11

ENSMUSG00000004221	IKBKG	2	2	0	0	0	3	7	16	7	12	9	12
ENSMUSG00000004233	WARS2	2	3	3	7	1	0	5	8	6	6	6	5
ENSMUSG00000004263	ATN1	0	4	5	8	7	1	13	12	11	16	14	12
ENSMUSG00000004264	PHB2	37	38	35	45	61	50	235	265	211	212	178	203
ENSMUSG00000004266	PTPN6	2	1	0	4	2	0	13	11	11	15	8	16
ENSMUSG00000004267	ENO2	0	1	0	1	0	0	0	4	1	3	1	2
ENSMUSG00000004268	EMG1	13	28	28	24	36	30	166	180	150	204	128	138
ENSMUSG00000004270	LPCAT3	5	7	4	8	4	3	28	16	25	24	24	25
ENSMUSG00000004285	ATP6V1F	4	2	4	5	9	9	42	42	25	50	38	37
ENSMUSG00000004317	CLCN5	3	3	3	6	3	5	8	9	10	17	5	12
ENSMUSG00000004319	CLCN3	38	54	30	57	53	53	231	235	177	249	184	196
ENSMUSG00000004347	PDE1C	0	0	0	0	0	0	1	0	0	1	0	1
ENSMUSG00000004356	UTP20	9	8	19	13	12	8	56	52	47	65	55	52
ENSMUSG00000004364	CUL3	26	25	27	32	35	34	175	147	109	166	101	120
ENSMUSG00000004371	IL11	3	4	5	2	3	2	23	14	13	20	21	20
ENSMUSG00000004393	DDX56	5	8	6	2	6	4	40	42	34	46	44	32
ENSMUSG00000004394	TMED4	5	7	7	5	8	10	31	33	34	43	32	33
ENSMUSG00000004446	BID	1	0	0	1	3	0	9	7	2	5	8	7
ENSMUSG00000004451	RALB	0	0	0	0	0	0	1	0	0	1	0	0
ENSMUSG00000004455	PPP1CC	1	0	0	1	0	0	0	0	2	4	3	4
ENSMUSG00000004460	DNAJB11	7	8	16	16	25	13	47	81	46	62	59	52
ENSMUSG00000004462	TBCCD1	5	1	3	4	3	1	31	18	11	22	17	12
ENSMUSG00000004500	ZFP324	0	1	1	2	0	2	5	11	5	8	6	5
ENSMUSG00000004508	GAB2	0	1	1	4	1	1	5	4	8	3	2	4
ENSMUSG00000004530	CORO1C	10	10	15	13	11	15	65	62	40	52	42	50
ENSMUSG00000004535	TAX1BP1	40	50	49	55	46	50	276	288	181	289	195	218
ENSMUSG00000004558	NDRG2	0	0	0	1	0	0	0	0	0	0	0	0
ENSMUSG00000004561	METTL17	1	5	4	2	6	2	14	18	14	17	18	8
ENSMUSG00000004562	ARHGEF40	1	0	0	0	1	1	1	2	2	2	2	2
ENSMUSG00000004565	PNPLA6	0	6	0	4	6	3	12	18	10	22	11	9
ENSMUSG00000004567	MCOLN1	2	1	1	1	2	1	7	8	6	7	5	5
ENSMUSG00000004568	ARHGEF18	4	3	4	6	4	1	31	11	26	27	14	15
ENSMUSG00000004591	PKN2	23	25	18	31	39	28	113	143	97	112	90	85
ENSMUSG00000004610	ETFB	0	3	3	1	1	1	0	0	0	1	0	1
ENSMUSG00000004626	STXBP2	2	0	0	3	2	4	0	1	0	1	1	0
ENSMUSG00000004630	PCP2	0	0	0	0	0	0	0	0	0	1	0	1
ENSMUSG00000004633	CHN2	9	13	12	14	21	8	50	63	53	72	39	47
ENSMUSG00000004637	WWOX	0	3	0	0	3	0	2	2	3	3	1	3
ENSMUSG00000004642	SLBP	13	21	17	13	22	18	44	51	41	65	40	42
ENSMUSG00000004661	ARID3B	1	1	1	0	1	0	4	3	1	3	5	3
ENSMUSG00000004665	CNN2	21	29	22	28	30	31	113	134	76	120	86	107
ENSMUSG00000004667	POLR2E	8	11	11	8	8	9	73	90	55	70	75	77
ENSMUSG00000004677	MYO9B	11	7	7	7	5	5	27	34	22	39	29	23
ENSMUSG00000004698	HDAC9	2	2	0	3	1	0	5	3	9	11	7	1
ENSMUSG00000004707	LY9	10	6	15	17	14	13	58	55	52	49	40	36
ENSMUSG00000004709	CD244	1	1	2	7	1	0	10	13	9	14	6	5
ENSMUSG00000004730	ADGRE1	0	0	0	0	0	0	0	0	0	2	0	0
ENSMUSG00000004748	MTFP1	2	3	3	1	1	0	15	12	10	9	11	13
ENSMUSG00000004768	RAB23	4	3	2	3	2	3	22	14	11	19	12	17
ENSMUSG00000004771	RAB11A	6	7	5	10	7	11	42	49	38	58	34	34
ENSMUSG00000004788	EIF2B2	4	4	4	3	1	5	23	25	19	21	10	13
ENSMUSG00000004789	DLST	12	14	8	9	12	8	80	82	51	68	57	65
ENSMUSG00000004798	ULK2	0	0	3	0	2	1	6	7	7	7	4	2
ENSMUSG00000004815	DGKQ	2	1	5	2	2	1	12	15	6	16	14	13
ENSMUSG00000004837	GRAP	1	0	0	0	0	1	1	3	2	2	2	0
ENSMUSG00000004843	CHMP2B	2	3	2	3	2	4	19	18	19	22	19	13
ENSMUSG00000004846	PLOD3	3	7	4	3	7	4	22	28	19	20	18	20
ENSMUSG00000004849	AP1S1	5	17	7	12	16	14	61	72	58	82	46	67

ENSMUSG00000004865	SRPK1	25	41	38	50	49	29	195	212	155	205	180	157
ENSMUSG00000004880	LBR	33	38	35	38	37	34	213	180	138	222	138	164
ENSMUSG00000004885	CRABP2	0	0	0	1	0	0	1	3	1	2	4	1
ENSMUSG00000004891	NES	9	16	14	13	11	9	53	53	51	56	43	36
ENSMUSG00000004892	BCAN	1	0	1	0	0	0	0	0	0	0	0	0
ENSMUSG00000004895	PRCC	8	16	16	14	14	7	46	64	45	61	44	53
ENSMUSG00000004896	RRNAD1	24	29	9	17	17	25	79	77	83	81	57	79
ENSMUSG00000004897	HDGF	38	59	43	62	69	49	292	325	203	300	213	247
ENSMUSG00000004929	THOP1	1	5	4	5	12	5	26	30	24	38	23	35
ENSMUSG00000004931	APBA3	0	0	0	0	0	0	3	1	1	0	1	0
ENSMUSG00000004933	MATK	0	0	0	0	0	1	0	2	0	0	0	0
ENSMUSG00000004934	PIAS4	11	15	20	14	14	17	49	67	51	62	50	51
ENSMUSG00000004936	MAP2K1	8	4	9	9	9	9	61	39	35	52	31	40
ENSMUSG00000004937	SGTA	4	2	6	9	8	6	40	26	28	39	34	38
ENSMUSG00000004939	NMRK2	0	0	0	0	0	0	0	1	0	0	0	0
ENSMUSG00000004945	TMEM242	4	2	4	6	6	8	23	32	24	42	31	24
ENSMUSG00000004947	DTX2	10	5	9	8	3	6	38	48	39	46	32	40
ENSMUSG00000004952	RASA4	0	0	0	0	0	0	1	0	0	1	0	1
ENSMUSG00000004980	HNRNPA2B1	146	185	177	220	187	195	1418	1414	945	1341	880	967
ENSMUSG00000004988	FXYD4	2	3	4	3	0	1	11	4	11	4	5	9
ENSMUSG00000004994	CCDC130	0	1	1	3	3	0	3	6	4	6	1	3
ENSMUSG00000004996	MRI1	12	5	8	8	11	13	60	54	53	65	50	44
ENSMUSG00000005034	PRKACB	75	108	72	96	81	92	546	476	378	724	398	401
ENSMUSG00000005043	SGSH	1	1	2	1	3	0	6	3	2	0	2	6
ENSMUSG00000005045	CHD5	1	0	1	0	0	0	2	2	3	0	0	3
ENSMUSG00000005054	CSTB	5	13	9	9	6	8	72	70	45	63	47	53
ENSMUSG00000005069	PEX5	0	7	8	7	9	6	18	40	26	26	30	22
ENSMUSG00000005078	JKAMP	2	4	7	9	8	5	23	21	18	34	25	22
ENSMUSG00000005087	CD44	22	23	26	20	22	20	71	83	83	100	71	99
ENSMUSG00000005102	EIF2AK4	4	4	3	3	2	4	26	34	25	36	27	24
ENSMUSG00000005103	WDR1	81	85	84	75	80	88	334	360	280	387	261	319
ENSMUSG00000005107	SLC2A9	0	0	0	0	1	0	1	1	0	1	2	0
ENSMUSG00000005125	NDRG1	0	1	0	0	0	0	0	0	1	0	0	0
ENSMUSG00000005131	4930550C14F	0	1	0	0	0	0	0	4	3	3	1	2
ENSMUSG00000005142	MAN2B1	14	36	18	31	15	22	98	94	90	97	60	85
ENSMUSG00000005148	KLF5	0	0	0	0	0	0	0	1	0	0	0	0
ENSMUSG00000005150	WDR83	0	3	6	1	1	3	10	10	7	11	4	7
ENSMUSG00000005161	PRDX2	17	17	8	21	13	15	99	116	112	108	88	108
ENSMUSG00000005198	POLR2A	7	8	20	21	15	13	66	61	59	64	58	77
ENSMUSG00000005204	SENP3	4	3	4	3	5	5	30	34	18	31	20	28
ENSMUSG00000005225	PLEKHA8	5	8	3	2	3	1	14	14	13	16	19	12
ENSMUSG00000005233	SPC25	2	1	2	5	5	4	20	24	15	25	19	21
ENSMUSG00000005237	DNAH2	0	0	1	0	0	0	0	0	0	1	0	0
ENSMUSG00000005262	UFD1	9	5	10	7	7	5	52	43	46	56	43	35
ENSMUSG00000005267	ZFP287	0	1	0	1	1	0	0	0	0	0	0	0
ENSMUSG00000005268	PRLR	0	0	0	0	0	1	0	0	0	0	0	0
ENSMUSG00000005299	LETM1	14	20	23	25	17	23	113	124	79	115	89	83
ENSMUSG00000005312	UBQLN1	11	23	13	20	22	24	91	134	62	101	75	78
ENSMUSG00000005320	FGFR4	0	0	0	0	0	0	1	0	0	1	0	1
ENSMUSG00000005338	CADM3	0	1	2	1	1	0	4	11	2	3	4	12
ENSMUSG00000005354	TXN2	6	10	7	6	12	12	48	68	37	62	37	48
ENSMUSG00000005360	SLC1A3	0	0	0	0	0	0	0	1	0	0	0	0
ENSMUSG00000005362	CRBN	2	9	8	7	7	9	60	55	62	60	32	40
ENSMUSG00000005364	IL5RA	0	0	0	0	0	0	0	0	0	0	1	0
ENSMUSG00000005370	MSH6	5	7	3	14	15	5	56	35	38	49	35	42
ENSMUSG00000005371	FBXO11	4	6	8	16	9	4	55	27	31	48	27	29
ENSMUSG00000005373	MLXIPL	0	0	0	0	0	0	1	0	0	1	1	0
ENSMUSG00000005374	TBL2	5	4	5	9	8	8	29	36	28	40	24	23

ENSMUSG00000005378	BUD23	15	13	21	21	22	25	110	134	106	109	84	115
ENSMUSG00000005410	MCM5	19	28	23	24	38	22	112	138	123	145	96	121
ENSMUSG00000005413	HMOX1	1	0	0	0	1	2	1	3	3	5	1	4
ENSMUSG00000005417	MPRIP	3	10	9	13	13	7	38	44	30	40	25	31
ENSMUSG00000005442	CIC	2	5	3	7	3	4	23	38	25	29	24	19
ENSMUSG00000005447	PAFAH1B3	4	6	5	9	11	4	26	24	30	39	18	25
ENSMUSG00000005465	IL27RA	3	4	3	5	3	1	14	13	20	7	19	13
ENSMUSG00000005469	PRKACA	12	15	12	9	6	12	75	62	62	64	44	60
ENSMUSG00000005470	ASF1B	4	11	8	7	7	5	42	40	25	44	33	48
ENSMUSG00000005481	DDX39	16	19	15	14	20	19	109	103	66	101	98	87
ENSMUSG00000005483	DNAJB1	2	6	2	4	1	4	36	26	25	45	26	36
ENSMUSG00000005493	MSH4	1	0	0	0	1	0	0	0	1	0	0	1
ENSMUSG00000005501	USP40	2	6	7	7	7	3	15	17	11	11	10	14
ENSMUSG00000005505	KBTBD4	2	3	4	3	3	2	18	20	12	19	10	12
ENSMUSG00000005506	CELF1	29	39	50	42	45	51	215	207	141	238	135	145
ENSMUSG00000005510	NDUFS3	2	3	0	2	2	6	14	15	9	22	7	12
ENSMUSG00000005514	POR	10	8	6	12	12	13	32	40	33	40	32	35
ENSMUSG00000005533	IGF1R	0	0	0	0	0	0	0	0	0	0	1	0
ENSMUSG00000005534	INSR	7	11	14	6	6	4	50	43	42	46	32	29
ENSMUSG00000005540	FCER2A	0	0	0	0	0	0	0	0	0	0	1	0
ENSMUSG00000005566	TRIM28	13	18	17	19	29	21	113	109	113	137	95	111
ENSMUSG00000005575	UBE2M	22	25	24	25	26	17	122	120	117	112	94	137
ENSMUSG00000005580	ADCY9	0	0	0	1	0	0	0	0	0	0	0	0
ENSMUSG00000005583	MEF2C	1	0	0	0	0	0	0	1	0	0	1	0
ENSMUSG00000005609	CTR9	8	19	17	20	16	14	76	72	56	94	61	65
ENSMUSG00000005610	EIF4G2	33	50	58	58	55	57	396	432	290	452	304	305
ENSMUSG00000005611	MRVI1	0	0	0	0	0	1	2	3	1	3	3	1
ENSMUSG00000005615	PCYT1A	3	1	3	1	2	1	16	17	13	14	8	10
ENSMUSG00000005621	ZFP592	1	3	4	4	1	8	18	18	10	19	11	19
ENSMUSG00000005625	PSMD4	24	22	19	30	33	20	170	172	109	194	150	168
ENSMUSG00000005628	TMOD4	0	0	0	0	0	0	2	1	0	1	0	0
ENSMUSG00000005640	INSRR	0	0	0	1	0	0	0	0	0	0	0	0
ENSMUSG00000005649	CABP5	0	0	0	0	0	0	0	0	2	0	0	1
ENSMUSG00000005656	SNX6	7	7	7	5	13	6	52	46	30	45	45	47
ENSMUSG00000005667	MTHFD2	20	26	21	23	28	17	128	109	79	120	64	114
ENSMUSG00000005674	TOMM40L	3	2	3	2	3	3	14	15	5	20	14	21
ENSMUSG00000005677	NR1I3	0	0	0	1	1	0	5	1	2	3	1	3
ENSMUSG00000005681	APOA2	0	3	1	0	2	2	4	0	1	5	3	6
ENSMUSG00000005682	PAN2	1	3	6	3	5	4	16	12	10	13	15	8
ENSMUSG00000005683	CS	14	30	23	32	24	19	129	135	118	155	101	137
ENSMUSG00000005686	AMPD3	0	0	0	0	0	0	0	1	0	0	0	0
ENSMUSG00000005687	BCAS2	21	38	32	33	28	18	223	238	179	267	164	161
ENSMUSG00000005696	SH2D1A	3	1	2	2	1	0	3	4	5	4	2	6
ENSMUSG00000005698	CTCF	23	23	21	33	32	40	157	147	127	186	124	115
ENSMUSG00000005699	PARD6A	1	2	1	0	1	1	4	7	3	5	3	7
ENSMUSG00000005705	AGRP	0	0	0	0	0	0	0	1	1	2	0	1
ENSMUSG00000005718	TFAP4	14	19	8	18	17	7	61	53	42	70	41	56
ENSMUSG00000005732	RANBP1	63	87	91	94	107	83	658	619	493	676	476	483
ENSMUSG00000005763	CD247	0	0	0	0	1	0	0	3	0	0	0	0
ENSMUSG00000005774	RFX5	0	1	1	2	3	2	15	10	11	19	16	9
ENSMUSG00000005779	PSMB4	31	54	56	50	64	54	328	356	229	297	264	264
ENSMUSG00000005802	SLC30A4	1	2	3	3	1	4	10	6	6	8	5	11
ENSMUSG00000005803	SQOR	0	0	0	0	1	0	3	1	3	4	2	2
ENSMUSG00000005804	BLOC1S6	4	6	4	5	13	3	44	46	19	39	35	44
ENSMUSG00000005813	METAP1	5	4	13	6	15	12	32	49	17	47	35	41
ENSMUSG00000005823	GPR108	5	6	2	8	5	1	16	25	14	14	18	12
ENSMUSG00000005824	TNFSF14	0	2	1	0	1	2	0	1	0	1	1	1
ENSMUSG00000005846	RSL1D1	30	53	42	45	46	32	316	321	228	361	195	208

ENSMUSG00000005871	APC	7	7	7	10	3	8	41	26	28	34	26	23
ENSMUSG00000005873	REEP5	15	19	14	14	8	17	81	79	53	73	55	69
ENSMUSG00000005881	ERGIC3	10	5	8	10	7	8	33	45	36	61	39	23
ENSMUSG00000005882	UQCC1	6	4	7	2	4	8	37	21	26	24	18	28
ENSMUSG00000005883	SPO11	0	5	2	3	0	0	10	14	11	16	10	12
ENSMUSG00000005886	NCOA2	7	2	11	7	6	6	28	38	30	33	14	20
ENSMUSG00000005893	NR2C2	3	15	10	5	5	9	37	44	29	44	34	28
ENSMUSG00000005897	NR2C1	2	0	0	3	1	1	5	15	12	11	9	6
ENSMUSG00000005899	SMPD4	6	8	14	4	7	9	36	51	38	44	28	38
ENSMUSG00000005907	PEX1	2	3	1	4	10	3	19	22	15	18	9	12
ENSMUSG00000005917	OTX1	0	0	0	0	0	0	1	0	0	0	0	0
ENSMUSG00000005936	KCTD20	3	6	3	6	7	7	32	25	23	22	28	25
ENSMUSG00000005947	ITGAE	1	1	4	0	2	0	1	3	0	1	0	0
ENSMUSG00000005949	CTNS	2	1	1	1	1	2	8	8	4	7	3	3
ENSMUSG00000005950	P2RX5	0	0	0	0	0	0	0	1	0	0	0	0
ENSMUSG00000005951	SHPK	0	0	0	1	0	0	1	0	3	1	3	0
ENSMUSG00000005952	TRPV1	0	0	0	0	0	0	0	0	0	1	0	1
ENSMUSG00000005968	TUFT1	0	1	4	4	1	1	3	8	3	7	1	3
ENSMUSG00000005973	RCN1	6	12	1	2	8	5	35	29	30	39	22	30
ENSMUSG00000005980	DNASE1	0	1	0	0	0	0	8	6	5	4	2	3
ENSMUSG00000005981	TRAP1	15	27	16	23	23	24	149	141	101	164	108	120
ENSMUSG00000005982	NAA60	2	5	5	1	2	4	27	21	19	21	14	16
ENSMUSG00000005983	1700037C18F	1	1	0	0	2	2	6	6	8	7	6	2
ENSMUSG00000005986	ANKRD13D	2	0	0	0	0	0	0	0	0	1	1	0
ENSMUSG00000006005	TPR	75	101	86	109	91	97	439	464	315	483	340	407
ENSMUSG00000006010	BC003331	6	9	14	9	13	14	38	44	41	38	33	32
ENSMUSG00000006014	PRG4	3	4	6	7	2	3	16	15	13	30	18	23
ENSMUSG00000006019	DHX34	0	2	2	2	2	2	4	6	3	6	2	5
ENSMUSG00000006021	KPTN	2	1	1	3	3	1	19	12	14	11	7	5
ENSMUSG00000006024	NAPA	4	2	6	9	10	8	33	46	35	30	36	46
ENSMUSG00000006050	SRA1	6	13	6	9	8	3	38	42	31	42	23	29
ENSMUSG00000006057	ATP5G1	5	11	16	11	16	21	96	91	76	112	63	98
ENSMUSG00000006058	SNF8	15	8	7	8	7	4	32	47	39	34	31	48
ENSMUSG00000006095	TBCB	7	9	9	12	10	4	41	46	41	64	37	39
ENSMUSG00000006127	INPP5K	2	3	1	2	2	0	11	11	6	8	10	11
ENSMUSG00000006134	CRKL	6	11	12	14	5	6	49	41	29	33	27	25
ENSMUSG00000006154	EPS8L1	0	0	0	1	0	2	6	6	6	10	2	7
ENSMUSG00000006169	CLINT1	16	14	16	13	27	13	135	149	98	132	114	122
ENSMUSG00000006179	PRSS16	0	1	3	0	0	1	7	4	2	4	2	5
ENSMUSG00000006191	CDKAL1	8	12	16	14	12	7	90	67	54	67	49	57
ENSMUSG00000006215	ZBTB17	2	3	5	2	6	7	19	17	17	17	16	20
ENSMUSG00000006262	MOB1B	2	5	2	5	5	3	18	20	27	30	21	23
ENSMUSG00000006273	ATP6V1B2	11	13	13	20	16	13	76	88	45	76	48	62
ENSMUSG00000006276	EPS15L1	3	6	4	7	4	3	31	31	30	36	29	29
ENSMUSG00000006281	TEP1	1	3	2	1	3	2	9	12	7	14	5	5
ENSMUSG00000006288	TTC5	3	16	9	8	19	10	38	42	29	34	28	46
ENSMUSG00000006289	OSGEP	1	2	4	3	5	2	28	23	22	24	16	15
ENSMUSG00000006299	AAMP	18	32	22	30	24	19	169	155	120	173	135	137
ENSMUSG00000006301	TMBIM1	2	2	2	6	7	3	27	19	12	21	24	26
ENSMUSG00000006304	ARPC2	18	43	31	67	40	34	194	190	149	215	143	164
ENSMUSG00000006307	KMT2B	5	6	4	5	2	8	19	28	17	32	24	29
ENSMUSG00000006310	ZBTB32	0	0	0	0	0	0	0	1	0	0	0	2
ENSMUSG00000006313	UPK1A	0	0	0	0	1	0	0	2	1	0	1	1
ENSMUSG00000006315	TMEM147	2	11	7	5	9	7	51	46	33	37	31	49
ENSMUSG00000006333	RPS9	111	134	134	179	159	167	979	1021	861	971	819	901
ENSMUSG00000006335	TFPT	1	4	3	3	3	2	19	20	10	10	14	23
ENSMUSG00000006342	SUSD2	0	0	0	0	1	0	0	0	0	0	0	0
ENSMUSG00000006344	GGT5	0	0	0	0	0	0	1	0	0	0	0	0

ENSMUSG00000006345	GGT1	0	0	0	1	1	0	1	1	0	0	0	2
ENSMUSG00000006356	CRIP2	0	0	0	0	1	0	1	1	1	0	0	0
ENSMUSG00000006360	CRIP1	35	44	43	55	56	34	301	316	270	291	248	304
ENSMUSG00000006362	CBFA2T3	8	10	7	18	10	12	58	38	31	56	34	46
ENSMUSG00000006369	FBLN1	0	0	0	0	0	0	0	0	0	0	1	0
ENSMUSG00000006373	PGRMC1	7	8	11	14	6	8	74	85	82	82	63	53
ENSMUSG00000006378	GCAT	5	5	3	4	8	3	32	45	29	47	39	35
ENSMUSG00000006386	TEK	0	0	0	0	0	0	1	0	0	0	0	0
ENSMUSG00000006389	MPL	0	0	0	1	0	0	1	0	0	0	0	1
ENSMUSG00000006390	ELOVL1	6	8	11	10	13	9	54	59	47	66	40	64
ENSMUSG00000006392	MED8	7	11	10	10	12	12	76	80	62	86	58	59
ENSMUSG00000006395	HYI	2	5	3	2	5	7	24	26	24	21	16	21
ENSMUSG00000006398	CDC20	25	28	36	48	45	39	191	180	157	162	137	176
ENSMUSG00000006411	NECTIN4	0	0	0	0	0	0	0	1	1	4	0	0
ENSMUSG00000006412	PFDN2	65	87	105	99	101	108	346	405	322	362	276	338
ENSMUSG00000006418	RNF114	4	6	8	13	5	11	40	41	29	41	31	29
ENSMUSG00000006423	C330007P06F	1	5	3	4	2	1	13	8	4	7	6	8
ENSMUSG00000006442	SRM	13	23	14	16	21	14	104	114	80	109	91	99
ENSMUSG00000006445	EPHA2	1	0	1	0	0	2	1	0	0	0	0	0
ENSMUSG00000006456	RBM14	0	2	4	6	4	3	26	34	28	42	17	30
ENSMUSG00000006457	ACTN3	0	0	0	1	0	0	1	0	0	0	1	0
ENSMUSG00000006463	ZDHHC24	2	1	1	0	5	1	5	5	7	5	5	4
ENSMUSG00000006464	BBS1	0	0	0	1	1	0	2	1	2	1	0	3
ENSMUSG00000006471	NDOR1	3	1	1	3	2	2	6	5	7	10	14	8
ENSMUSG00000006476	NSMF	4	7	4	6	9	8	26	35	25	31	11	36
ENSMUSG00000006488	PRL7A1	0	0	0	0	0	0	0	1	0	1	0	0
ENSMUSG00000006494	PDK1	28	32	21	23	35	24	190	204	126	174	148	134
ENSMUSG00000006498	PTBP1	18	16	21	16	19	15	117	142	97	134	112	117
ENSMUSG00000006517	MVD	4	5	5	6	11	5	43	42	27	49	25	36
ENSMUSG00000006519	CYBA	14	26	32	30	17	13	198	178	120	162	146	159
ENSMUSG00000006526	TMEM110	1	2	0	1	1	3	8	5	7	13	14	10
ENSMUSG00000006527	SFMBT1	7	9	10	11	10	8	38	36	20	37	26	29
ENSMUSG00000006567	ATP7B	0	0	0	0	0	0	0	0	0	0	0	2
ENSMUSG00000006575	RUNDC3A	1	1	3	1	3	0	2	2	5	6	5	7
ENSMUSG00000006585	CDT1	18	22	14	21	23	17	138	130	98	128	95	115
ENSMUSG00000006589	APRT	12	15	9	15	10	10	96	80	67	85	62	86
ENSMUSG00000006599	GTF2H1	12	11	14	20	25	16	98	102	81	110	77	93
ENSMUSG00000006638	ABHD1	0	2	1	1	2	3	4	5	8	7	7	4
ENSMUSG00000006641	SLC5A6	1	1	3	0	0	0	7	3	5	6	3	2
ENSMUSG00000006649	NPHS1	0	0	1	0	0	0	0	1	1	1	0	0
ENSMUSG00000006673	QRICH1	44	45	51	32	46	23	162	194	141	200	139	147
ENSMUSG00000006675	P4HTM	0	1	0	0	1	1	5	5	5	2	3	2
ENSMUSG00000006676	USP19	6	5	7	6	6	7	39	41	28	49	25	40
ENSMUSG00000006678	POLA1	7	11	6	14	11	14	52	53	43	66	40	46
ENSMUSG00000006699	CDC42	27	38	29	64	38	28	224	227	186	214	158	182
ENSMUSG00000006705	PKNOX1	3	6	3	6	7	7	19	13	11	29	8	15
ENSMUSG00000006715	GMNN	25	26	28	45	38	36	224	252	179	272	205	188
ENSMUSG00000006717	ACOT13	10	7	10	9	14	11	65	86	47	101	61	68
ENSMUSG00000006720	ZFP184	0	0	1	0	0	0	1	2	2	4	0	1
ENSMUSG00000006724	CYP27B1	0	0	0	0	0	0	0	0	1	0	0	0
ENSMUSG00000006728	CDK4	25	28	32	44	29	26	207	258	203	190	169	192
ENSMUSG00000006731	B4GALNT1	4	1	0	3	2	0	17	20	16	15	7	13
ENSMUSG00000006732	METTL1	10	7	8	7	14	7	36	56	35	37	23	42
ENSMUSG00000006736	TSPAN31	4	4	6	5	9	5	37	48	27	38	33	37
ENSMUSG00000006740	KIF5B	34	47	42	61	62	52	218	191	146	243	157	178
ENSMUSG00000006763	SAAL1	1	3	3	4	3	4	19	15	11	19	16	20
ENSMUSG00000006782	CNP	1	3	2	4	2	0	5	8	6	6	13	10
ENSMUSG00000006784	TTC25	0	0	0	1	0	0	0	0	0	2	0	0

ENSMUSG00000006800	SULF2	0	3	0	0	0	1	3	3	1	7	1	2
ENSMUSG00000006818	SOD2	15	20	18	23	13	10	128	136	112	132	116	114
ENSMUSG00000006850	TMCO6	0	0	2	1	3	1	6	6	7	5	3	3
ENSMUSG00000006906	STAMBP	2	1	2	6	4	2	19	11	12	17	19	8
ENSMUSG00000006920	EZH1	1	1	5	4	1	3	23	22	17	18	22	22
ENSMUSG00000006932	CTNNB1	12	17	17	19	14	19	168	149	102	164	109	102
ENSMUSG00000006941	EIF1B	7	9	8	15	19	12	60	60	32	55	40	55
ENSMUSG00000006998	PSMD2	8	13	16	24	19	16	87	89	70	85	70	71
ENSMUSG00000007029	VARS	73	93	83	94	102	76	352	420	308	411	304	322
ENSMUSG00000007030	VWA7	0	0	0	0	1	0	0	0	1	3	0	0
ENSMUSG00000007033	HSPA1L	0	1	0	1	0	0	0	1	0	1	1	1
ENSMUSG00000007035	MSH5	0	0	1	0	1	0	5	6	1	3	2	1
ENSMUSG00000007036	ABHD16A	6	5	3	7	6	3	22	33	20	29	27	24
ENSMUSG00000007038	NEU1	2	12	8	4	9	5	16	26	25	34	24	35
ENSMUSG00000007039	DDAH2	1	1	4	6	1	0	9	5	14	7	8	8
ENSMUSG00000007041	CLIC1	29	50	54	68	61	40	217	254	164	204	152	217
ENSMUSG00000007050	LSM2	26	45	31	56	33	41	271	229	181	314	194	231
ENSMUSG00000007080	POLE	6	11	12	8	5	7	45	52	34	51	30	39
ENSMUSG00000007122	CASQ1	0	0	0	0	0	0	0	0	0	0	1	0
ENSMUSG00000007207	STX1A	5	9	15	15	11	8	39	31	29	25	23	38
ENSMUSG00000007216	ZFP775	0	0	0	0	0	0	1	2	1	2	0	2
ENSMUSG00000007338	MRPL49	6	7	9	6	10	8	55	43	28	43	31	38
ENSMUSG00000007379	DENND2C	0	0	0	0	0	0	0	1	0	0	0	0
ENSMUSG00000007411	MARK3	18	15	22	19	17	11	72	77	62	91	65	73
ENSMUSG00000007415	GATAD1	21	24	28	24	22	22	102	110	84	110	76	71
ENSMUSG00000007458	M6PR	19	14	23	17	37	20	78	106	73	98	66	67
ENSMUSG00000007476	LRRC8A	7	9	11	11	7	14	27	23	15	43	19	30
ENSMUSG00000007480	MC5R	1	2	1	1	0	1	2	1	0	1	1	1
ENSMUSG00000007564	PPP2R1A	21	22	22	26	33	21	144	165	132	153	127	148
ENSMUSG00000007570	FANCE	2	1	0	2	4	2	14	14	10	12	11	9
ENSMUSG00000007589	TINF2	3	2	0	4	4	6	17	16	15	17	15	21
ENSMUSG00000007594	HAPLN4	1	0	0	0	0	0	0	0	0	0	0	0
ENSMUSG00000007603	DUS3L	8	11	11	10	5	8	27	54	37	37	37	43
ENSMUSG00000007610	GTPBP3	2	0	3	5	2	4	16	21	16	13	11	18
ENSMUSG00000007613	TGFBR1	8	6	8	11	2	6	29	25	12	36	12	22
ENSMUSG00000007617	HOMER1	5	6	6	5	6	2	28	31	23	23	15	18
ENSMUSG00000007646	RAD51C	0	1	1	0	1	0	2	3	3	7	4	5
ENSMUSG00000007656	ARPP19	36	70	54	69	56	55	420	418	329	438	341	384
ENSMUSG00000007659	BCL2L1	31	41	29	49	44	46	196	222	188	220	164	199
ENSMUSG00000007670	KHSRP	264	338	297	351	334	321	1255	1324	939	1279	929	1176
ENSMUSG00000007682	DIO2	0	0	0	0	0	0	0	0	0	0	0	1
ENSMUSG00000007721	CCDC124	6	3	3	4	8	10	42	50	40	47	32	59
ENSMUSG00000007739	CCT4	11	17	19	23	18	20	136	161	118	146	110	136
ENSMUSG00000007777	0610009B22F	0	0	1	1	0	1	6	5	3	6	4	7
ENSMUSG00000007812	ZFP655	2	4	0	5	6	6	22	17	16	19	12	14
ENSMUSG00000007815	RHOA	52	68	74	72	66	70	340	325	227	322	231	279
ENSMUSG00000007817	ZMIZ1	3	12	8	12	12	12	71	92	57	104	57	79
ENSMUSG00000007827	ANKRD26	43	63	51	67	47	53	273	231	173	272	185	203
ENSMUSG00000007833	ALDH16A1	1	2	9	5	3	3	36	21	16	29	28	31
ENSMUSG00000007836	HNRNPA0	92	119	139	122	151	141	714	710	515	755	545	581
ENSMUSG00000007837	PRRG2	0	0	1	0	1	1	1	8	5	4	1	5
ENSMUSG00000007850	HNRNPH1	22	27	18	38	46	37	247	248	160	304	172	198
ENSMUSG00000007867	IFT43	1	0	0	0	1	0	2	0	0	0	0	0
ENSMUSG00000007872	ID3	1	0	1	0	1	1	1	6	4	1	2	2
ENSMUSG00000007880	ARID1A	34	48	30	37	47	44	108	120	109	135	96	138
ENSMUSG00000007891	CTSD	5	10	6	10	10	5	40	38	35	23	30	39
ENSMUSG00000007892	RPLP1	125	173	174	245	202	220	1134	1165	839	1035	987	950
ENSMUSG00000007944	TTC9B	0	1	0	0	0	0	1	0	0	2	1	1

ENSMUSG00000007946	PHOX2A	1	0	7	0	1	1	2	6	1	1	0	3
ENSMUSG00000007950	ABHD8	10	10	13	15	17	16	54	60	46	68	49	66
ENSMUSG00000008036	AP2S1	10	10	15	9	12	9	64	70	60	71	58	65
ENSMUSG00000008090	FGFRL1	0	0	0	0	0	1	0	4	0	3	2	1
ENSMUSG00000008129	4930432K21F	0	0	2	2	0	1	2	5	4	1	1	2
ENSMUSG00000008136	FHL2	0	1	1	2	1	2	19	12	8	12	11	9
ENSMUSG00000008140	EMC10	5	3	5	2	4	2	17	20	10	18	13	13
ENSMUSG00000008153	CLSTN3	0	0	0	1	0	0	0	0	0	0	0	0
ENSMUSG00000008167	FBXW9	1	0	2	1	3	0	4	7	2	3	3	1
ENSMUSG00000008193	SPIB	0	0	0	0	0	0	0	1	1	0	1	0
ENSMUSG00000008200	FNBP4	23	35	26	36	33	30	169	178	127	178	122	149
ENSMUSG00000008206	CERS4	23	19	17	26	21	26	110	102	85	111	75	84
ENSMUSG00000008226	SCRN3	0	1	3	0	1	2	11	14	11	7	6	5
ENSMUSG00000008301	PHAX	9	9	8	13	13	15	56	59	44	66	52	60
ENSMUSG00000008307	1700109H08F	1	0	2	2	1	5	7	9	3	6	4	5
ENSMUSG00000008318	RELT	0	2	0	0	0	0	5	3	4	9	7	5
ENSMUSG00000008333	SNRPB2	25	46	34	38	36	35	173	188	125	202	124	145
ENSMUSG00000008348	UBC	34	66	48	44	68	55	253	264	208	298	221	243
ENSMUSG00000008373	PRPF31	10	9	13	12	14	13	59	63	43	83	43	61
ENSMUSG00000008384	SERTAD1	0	1	1	1	1	1	3	9	1	4	6	3
ENSMUSG00000008393	CARHSP1	3	2	0	0	4	1	5	11	5	8	2	7
ENSMUSG00000008398	ELK3	5	14	6	8	6	6	32	35	27	28	33	26
ENSMUSG00000008429	HERPUD2	5	8	6	8	4	7	29	33	35	45	30	23
ENSMUSG00000008435	RDH13	4	1	4	5	6	2	23	16	14	31	14	18
ENSMUSG00000008450	NUTF2	0	0	0	0	0	0	1	1	2	0	1	2
ENSMUSG00000008475	ARPC5	32	47	25	38	31	63	161	168	138	173	126	129
ENSMUSG00000008482	RNF151	0	0	2	1	0	0	0	0	1	1	0	1
ENSMUSG00000008489	ELAVL2	0	0	0	0	0	1	0	0	0	0	0	0
ENSMUSG00000008496	POU2F2	9	20	29	25	22	22	65	61	34	84	49	45
ENSMUSG00000008590	HTR3B	0	0	0	0	0	0	0	1	0	0	0	0
ENSMUSG00000008604	UBQLN4	7	12	13	14	9	7	50	63	46	55	46	48
ENSMUSG00000008668	RPS18	436	563	568	644	590	624	2588	3114	2204	2633	2256	2516
ENSMUSG00000008682	RPL10	50	57	47	68	62	65	437	465	296	466	359	385
ENSMUSG00000008683	RPS15A	130	192	169	186	169	186	1016	1012	778	1013	708	804
ENSMUSG00000008690	NCAPH2	8	10	11	20	15	13	59	61	39	54	54	53
ENSMUSG00000008730	HIPK1	11	9	15	10	15	10	76	58	25	73	47	50
ENSMUSG00000008763	MAN1A2	26	34	37	46	39	29	164	141	113	217	118	130
ENSMUSG00000008822	ACYP1	3	3	3	3	4	2	26	26	27	31	20	26
ENSMUSG00000008855	HDAC5	4	7	5	5	8	16	52	41	38	39	37	42
ENSMUSG00000008859	RALA	13	6	15	13	16	4	47	58	38	34	33	36
ENSMUSG00000008892	VDAC3	2	6	3	5	1	4	31	23	14	27	16	22
ENSMUSG00000008958	VPS72	3	4	4	2	2	4	25	36	23	40	21	27
ENSMUSG00000008976	GABPA	1	4	9	9	5	7	35	44	40	31	40	39
ENSMUSG00000008999	BMP7	5	3	6	2	4	5	19	17	10	22	16	19
ENSMUSG00000009013	DYNLL1	19	24	31	35	23	17	125	141	108	166	104	138
ENSMUSG00000009030	PDCL	4	8	6	5	12	6	35	37	30	45	25	34
ENSMUSG00000009035	TMEM184B	1	7	2	1	1	1	15	9	14	16	10	14
ENSMUSG00000009073	NF2	10	6	5	10	2	10	29	40	24	32	31	39
ENSMUSG00000009075	CABP7	0	0	1	0	0	0	1	0	4	2	1	7
ENSMUSG00000009076	ZMAT5	1	1	5	4	5	5	26	30	29	27	23	35
ENSMUSG00000009079	EWSR1	58	70	65	110	74	88	375	428	334	452	305	311
ENSMUSG00000009090	AP1B1	6	5	5	7	10	4	36	37	22	25	33	42
ENSMUSG00000009092	DERL3	0	0	1	0	0	0	2	1	1	3	1	0
ENSMUSG00000009093	GSTT4	0	0	0	0	0	1	3	4	2	3	4	2
ENSMUSG00000009108	GNAT2	0	1	0	0	0	0	1	1	1	1	1	1
ENSMUSG00000009112	BCL2L13	7	6	7	18	9	7	47	59	34	41	39	40
ENSMUSG00000009115	SPATC1L	0	0	1	0	0	0	1	0	1	0	0	1
ENSMUSG00000009145	DQX1	2	1	0	4	3	1	8	8	7	5	4	4

ENSMUSG00000009207	LNPK	19	25	24	27	30	19	98	88	62	90	57	76
ENSMUSG00000009246	TRPM5	0	0	0	0	0	0	0	0	0	1	0	0
ENSMUSG00000009248	ASCL2	0	0	0	0	0	0	0	1	1	0	0	0
ENSMUSG00000009281	RARRES2	0	0	0	0	0	0	0	1	1	0	0	0
ENSMUSG00000009291	PTTG1IP	4	1	3	2	3	3	21	18	12	16	19	15
ENSMUSG00000009292	TRPM2	4	1	3	5	3	0	13	9	3	6	7	12
ENSMUSG00000009293	UBE2G2	4	4	6	7	5	6	45	44	40	50	32	46
ENSMUSG00000009350	MPO	0	0	0	0	0	0	0	0	1	0	0	0
ENSMUSG00000009406	ELK1	1	4	4	2	1	1	12	12	5	7	10	14
ENSMUSG00000009418	NAV1	13	14	8	9	12	11	37	52	30	35	39	32
ENSMUSG00000009470	TNPO1	12	17	23	14	12	14	92	74	55	99	53	80
ENSMUSG00000009535	RNMT	11	8	6	7	10	11	68	64	35	71	52	26
ENSMUSG00000009549	SRP14	24	25	27	30	45	27	217	197	173	219	160	163
ENSMUSG00000009555	CDK9	17	17	25	23	30	23	153	171	141	162	134	163
ENSMUSG00000009563	TOR2A	7	6	7	10	9	5	42	53	52	37	53	49
ENSMUSG00000009566	FPGS	11	15	19	21	25	18	96	96	74	105	60	85
ENSMUSG00000009569	MKL2	1	1	1	3	1	0	9	6	12	11	5	10
ENSMUSG00000009575	CBX5	35	47	46	36	41	38	185	211	116	215	171	147
ENSMUSG00000009585	APOBEC3	14	28	18	16	24	18	83	90	66	87	57	69
ENSMUSG00000009596	TAF7L	0	0	0	0	0	0	0	1	0	0	0	0
ENSMUSG00000009614	SARDH	0	0	0	0	0	0	1	0	0	1	2	1
ENSMUSG00000009621	VAV2	2	9	1	7	5	8	40	27	30	28	19	36
ENSMUSG00000009630	PPP2CB	4	2	0	2	0	1	10	11	13	10	7	14
ENSMUSG00000009640	DMAP1	11	10	7	4	8	9	34	35	35	45	28	24
ENSMUSG00000009647	MCU	0	0	1	2	3	5	5	12	10	12	5	9
ENSMUSG00000009681	BCR	0	0	0	0	0	0	0	0	0	0	1	0
ENSMUSG00000009687	FXYD5	15	10	13	18	13	14	85	80	38	88	52	71
ENSMUSG00000009731	KCND1	1	1	0	1	0	0	0	0	0	1	0	0
ENSMUSG00000009733	TFCP2	2	2	0	1	1	3	12	13	12	19	9	13
ENSMUSG00000009734	POU6F2	0	0	0	0	0	0	0	0	1	2	0	0
ENSMUSG00000009739	POU6F1	0	1	0	0	0	0	0	0	0	0	0	0
ENSMUSG00000009741	UBP1	29	56	29	62	41	39	197	213	186	272	178	190
ENSMUSG00000009828	ICK	2	2	2	1	3	2	12	8	6	11	5	2
ENSMUSG00000009863	SDHB	19	30	24	18	22	19	158	146	122	142	119	119
ENSMUSG00000009894	SNAP47	3	2	2	3	3	2	11	9	8	21	4	8
ENSMUSG00000009905	KDSR	18	28	29	33	31	26	87	75	67	99	73	89
ENSMUSG00000009907	VPS4B	9	8	6	7	7	6	22	30	26	48	38	25
ENSMUSG00000009927	RPS25	190	223	257	264	270	251	1496	1558	1164	1522	1148	1324
ENSMUSG00000009995	TAZ	3	4	3	1	6	4	18	20	18	18	14	19
ENSMUSG00000010025	ALDH3A2	2	1	3	4	2	2	7	7	12	4	5	8
ENSMUSG00000010044	ZMYND10	0	0	0	0	0	0	1	0	0	0	1	1
ENSMUSG00000010045	TMEM115	0	1	1	3	2	0	14	13	9	21	7	18
ENSMUSG00000010047	HYAL2	0	1	0	1	1	0	5	6	5	5	7	7
ENSMUSG00000010048	IFRD2	4	9	6	6	7	10	56	50	45	61	40	44
ENSMUSG00000010051	HYAL1	1	0	0	1	0	0	0	3	0	1	1	2
ENSMUSG00000010054	TUSC2	2	4	4	1	6	5	19	20	12	16	15	17
ENSMUSG00000010057	NPRL2	4	0	0	1	2	2	9	7	6	11	13	9
ENSMUSG00000010066	CACNA2D2	241	281	297	357	354	363	1167	1499	1007	1419	990	1110
ENSMUSG00000010067	RASSF1	5	4	2	3	9	2	22	20	17	19	12	23
ENSMUSG00000010080	EPN3	0	0	0	0	0	0	1	1	0	0	0	0
ENSMUSG00000010095	SLC3A2	7	11	12	18	14	12	64	87	66	71	65	81
ENSMUSG00000010097	NXF1	15	13	12	8	12	14	63	72	67	66	65	67
ENSMUSG00000010110	STX5A	9	4	5	8	7	12	22	39	27	29	23	33
ENSMUSG00000010136	PIFO	1	0	0	0	1	0	0	0	0	0	0	0
ENSMUSG00000010154	SPIRE2	0	0	0	0	0	1	1	2	2	2	3	3
ENSMUSG00000010277	2610507B11F	13	12	13	15	15	14	78	82	58	91	67	92
ENSMUSG00000010290	AI597479	2	3	2	3	2	5	15	21	16	18	16	14
ENSMUSG00000010342	TEX14	2	6	0	4	3	2	12	21	13	13	9	9

ENSMUSG00000010358	IFI35	2	0	1	2	4	1	17	18	8	8	15	11
ENSMUSG00000010362	RDM1	2	1	0	0	0	1	3	1	0	1	2	3
ENSMUSG00000010376	NEDD8	15	18	16	10	21	20	137	110	95	125	105	116
ENSMUSG00000010392	GOSR1	5	4	3	7	2	7	19	22	17	24	13	23
ENSMUSG00000010406	MRPL52	14	7	12	12	14	9	74	65	43	80	50	61
ENSMUSG00000010453	KANSL3	19	14	11	17	19	14	73	79	62	67	52	64
ENSMUSG00000010492	UCKL1OS	0	0	0	2	1	2	4	5	4	4	5	2
ENSMUSG00000010517	FAF1	6	8	5	9	1	6	39	46	38	43	27	35
ENSMUSG00000010529	GM266	0	0	0	0	0	0	0	1	0	0	0	0
ENSMUSG00000010538	TSACC	2	0	0	2	0	0	2	1	2	0	2	1
ENSMUSG00000010554	METTL16	5	9	4	5	7	7	37	55	37	39	48	38
ENSMUSG00000010592	DAZL	0	0	0	0	0	0	0	0	0	0	1	0
ENSMUSG00000010607	PIGYL	4	1	3	3	3	4	18	14	20	12	15	11
ENSMUSG00000010608	RBM25	201	276	252	317	289	243	998	1122	820	1116	923	820
ENSMUSG00000010609	PSEN2	25	29	30	21	18	21	116	99	58	82	82	97
ENSMUSG00000010663	FADS1	0	2	3	2	1	2	12	12	11	14	6	11
ENSMUSG00000010751	TNFRSF22	0	0	0	0	0	0	0	1	0	0	0	0
ENSMUSG00000010755	CARS	22	17	27	38	22	20	140	132	115	143	95	117
ENSMUSG00000010825	GRID2IP	0	0	0	0	1	0	0	0	0	0	0	0
ENSMUSG00000010830	KDELR3	0	0	1	1	0	0	0	2	0	0	1	1
ENSMUSG00000010911	APIP	0	8	4	3	0	3	22	23	15	25	12	21
ENSMUSG00000010914	PDHX	3	4	8	4	4	8	39	35	35	30	32	34
ENSMUSG00000010936	VAC14	5	6	5	2	4	3	24	32	19	31	20	23
ENSMUSG00000011008	MCOLN2	7	9	10	16	11	13	71	65	55	59	51	48
ENSMUSG00000011096	AKT1S1	2	4	2	6	5	4	19	14	10	17	16	22
ENSMUSG00000011114	TBRG1	13	9	8	24	12	13	58	62	41	61	56	50
ENSMUSG00000011118	PANX3	0	0	1	0	0	0	0	0	0	0	0	0
ENSMUSG00000011148	ADSSL1	0	0	0	0	0	0	1	0	0	2	0	0
ENSMUSG00000011158	BRF1	8	9	7	6	9	9	52	49	34	39	31	28
ENSMUSG00000011171	VIPR2	0	0	0	0	0	1	0	0	1	0	1	0
ENSMUSG00000011179	ODC1	3	1	3	2	2	1	12	16	16	19	16	11
ENSMUSG00000011254	THG1L	0	4	4	3	5	4	13	12	16	8	11	7
ENSMUSG00000011256	ADAM19	0	0	0	0	0	0	0	0	1	0	0	0
ENSMUSG00000011257	PABPC4	17	27	18	26	19	24	85	106	87	110	79	99
ENSMUSG00000011267	ZFP296	0	1	0	0	0	0	0	0	0	0	0	0
ENSMUSG00000011306	SUGP1	10	4	3	6	5	4	24	44	25	17	30	32
ENSMUSG00000011350	GM5893	0	0	1	0	0	0	0	0	0	0	0	0
ENSMUSG00000011382	DHDH	0	0	0	0	0	0	1	0	0	0	0	0
ENSMUSG00000011427	ZFP790	0	1	0	1	1	1	5	3	1	2	2	5
ENSMUSG00000011463	CPB1	1	2	1	1	1	1	3	9	6	5	5	8
ENSMUSG00000011486	SLC25A41	0	0	0	0	0	0	2	0	0	0	0	0
ENSMUSG00000011658	FUZ	3	1	0	3	3	1	11	18	6	9	4	3
ENSMUSG00000011751	SPTBN4	0	0	0	0	0	0	0	0	1	1	0	0
ENSMUSG00000011752	PGAM1	0	0	1	0	0	0	4	2	2	2	1	0
ENSMUSG00000011831	EVI5	2	6	3	9	9	5	22	21	28	24	16	13
ENSMUSG00000011832	EVI5L	1	1	2	1	1	0	5	7	6	4	3	4
ENSMUSG00000011837	SNAPC2	0	2	1	0	1	2	6	6	2	4	5	8
ENSMUSG00000011877	GIT1	10	10	13	9	10	10	62	56	46	65	50	55
ENSMUSG00000011884	GLTP	3	10	7	7	7	5	45	36	20	35	22	21
ENSMUSG00000011958	BNIP2	20	16	17	23	25	26	101	102	67	97	80	73
ENSMUSG00000011960	CCNT1	34	44	28	39	21	34	105	115	90	121	84	105
ENSMUSG00000012017	SCARF2	0	0	0	0	0	0	1	0	0	0	0	0
ENSMUSG00000012076	BRMS1L	2	0	3	4	5	2	11	19	12	21	10	14
ENSMUSG00000012114	MED15	8	6	3	8	3	9	28	23	19	17	19	26
ENSMUSG00000012117	DHDDS	3	2	3	4	2	4	9	9	8	20	3	11
ENSMUSG00000012126	UBXN11	0	0	1	1	1	0	4	0	0	1	0	3
ENSMUSG00000012211	TEX22	0	0	0	0	0	0	0	0	0	1	0	0
ENSMUSG00000012296	TJAP1	6	5	5	9	7	9	28	25	28	31	28	32

ENSMUSG00000012405	RPL15	15	11	13	15	11	18	71	63	40	72	47	65
ENSMUSG00000012422	TMEM167	20	13	11	32	15	20	64	86	51	77	51	65
ENSMUSG00000012429	MPLKIP	5	5	7	8	1	9	50	38	25	39	18	28
ENSMUSG00000012443	KIF11	13	16	15	16	11	11	83	81	53	83	68	74
ENSMUSG00000012483	RPA3	40	40	40	41	53	39	348	359	255	282	285	247
ENSMUSG00000012535	TNPO3	41	52	36	64	62	52	250	280	205	316	208	239
ENSMUSG00000012609	TTLL5	0	3	3	4	1	1	3	10	5	7	11	8
ENSMUSG00000012640	ZFP715	0	2	0	1	7	1	14	11	12	10	8	11
ENSMUSG00000012777	ACP4	1	0	1	3	2	0	8	8	11	13	9	4
ENSMUSG00000012819	CDH23	0	0	0	0	0	0	0	1	0	0	0	1
ENSMUSG00000012848	RPS5	136	156	160	169	150	146	983	1061	747	970	816	905
ENSMUSG00000012889	PODNL1	2	1	0	6	2	3	6	10	8	4	4	4
ENSMUSG00000013033	ADGRL1	15	15	17	24	14	16	49	58	41	69	33	54
ENSMUSG00000013076	AMOTL1	0	1	0	0	0	0	0	0	0	0	0	0
ENSMUSG00000013089	ETV5	4	2	13	9	8	6	37	45	28	37	19	36
ENSMUSG00000013091	TMEM190	0	0	0	0	0	0	1	2	0	0	0	1
ENSMUSG00000013150	GFOD2	1	4	1	1	2	0	9	7	4	2	5	7
ENSMUSG00000013155	ENKD1	13	16	13	10	6	14	38	52	35	37	46	40
ENSMUSG00000013158	4933405L10R	0	0	0	0	0	0	1	1	0	0	0	0
ENSMUSG00000013160	ATP6V0D1	3	4	3	1	6	0	26	18	6	23	12	23
ENSMUSG00000013236	PTPRS	28	41	48	50	50	47	175	203	171	233	165	173
ENSMUSG00000013275	SLC41A1	0	2	1	1	3	1	11	7	5	12	6	17
ENSMUSG00000013415	IGF2BP1	0	0	0	0	0	0	0	1	0	0	0	0
ENSMUSG00000013418	B4GALNT2	0	0	0	1	0	0	0	0	0	0	0	0
ENSMUSG00000013419	ZFP651	0	0	2	0	1	0	1	0	2	2	1	2
ENSMUSG00000013465	NELFB	9	15	8	12	7	9	72	77	51	59	52	58
ENSMUSG00000013495	TMEM175	0	0	3	4	2	0	8	8	9	12	10	11
ENSMUSG00000013523	BCAS1	2	1	2	2	1	0	2	0	2	0	1	1
ENSMUSG00000013539	TANGO2	2	3	2	3	1	3	11	12	10	10	10	10
ENSMUSG00000013593	NDUFS2	19	19	9	23	26	14	95	119	89	137	74	112
ENSMUSG00000013611	SNX31	0	0	1	0	0	0	0	2	0	0	1	1
ENSMUSG00000013622	ATRAID	3	0	1	3	4	3	8	11	14	10	9	8
ENSMUSG00000013629	CAD	9	19	16	25	22	17	92	80	68	73	78	73
ENSMUSG00000013646	SH3BP5L	1	2	3	2	0	4	11	13	12	11	10	11
ENSMUSG00000013662	ATAD1	7	10	10	17	8	17	54	51	43	79	30	55
ENSMUSG00000013663	PTEN	51	64	67	83	57	54	364	374	269	369	294	295
ENSMUSG00000013698	PEA15A	0	1	0	3	1	0	6	6	3	5	3	4
ENSMUSG00000013701	TIMM23	0	1	1	0	0	1	3	3	3	3	3	3
ENSMUSG00000013707	TNFAIP8L2	4	3	6	2	4	3	20	29	15	6	17	20
ENSMUSG00000013736	TRNT1	10	13	14	16	18	9	94	120	93	154	98	102
ENSMUSG00000013787	EHMT2	14	17	12	24	19	15	88	119	59	93	77	93
ENSMUSG00000013822	ELOF1	4	2	4	4	4	6	28	28	27	27	21	26
ENSMUSG00000013833	MED16	1	1	0	1	0	2	7	8	9	8	7	6
ENSMUSG00000013846	ST3GAL1	1	2	2	2	0	5	7	14	9	11	12	8
ENSMUSG00000013858	TMEM259	13	13	28	17	15	21	75	86	67	78	65	80
ENSMUSG00000013878	RNF170	0	1	2	0	0	2	5	2	3	1	4	3
ENSMUSG00000013973	DEDD	8	8	7	15	11	15	57	59	36	59	34	43
ENSMUSG00000013997	NIT1	22	17	23	22	20	37	71	66	54	80	50	58
ENSMUSG00000014030	PAX5	0	0	0	0	0	1	0	1	0	0	1	1
ENSMUSG00000014039	PRDM15	3	4	3	3	1	1	8	20	11	12	12	14
ENSMUSG00000014074	RNF168	9	12	9	10	9	7	44	58	43	62	29	25
ENSMUSG00000014077	CHP1	8	9	4	6	9	5	49	56	40	48	40	57
ENSMUSG00000014158	TRPV4	0	0	0	0	0	0	0	0	0	1	0	0
ENSMUSG00000014177	TVP23B	7	13	3	13	12	4	77	62	48	95	58	77
ENSMUSG00000014195	DNAJC7	17	21	18	19	24	21	90	105	79	127	97	88
ENSMUSG00000014198	ZFP385C	0	0	0	0	0	1	1	1	0	1	0	0
ENSMUSG00000014226	CACYBP	19	31	28	47	25	32	239	251	182	271	168	176
ENSMUSG00000014232	CLUAP1	2	2	8	9	6	4	36	33	22	25	22	17

ENSMUSG00000014243	ZSWIM7	0	1	1	0	1	0	8	4	4	6	0	8
ENSMUSG00000014245	PIGL	3	3	7	7	6	9	22	23	23	35	12	28
ENSMUSG00000014294	NDUFA2	25	33	25	29	28	21	195	190	148	186	158	169
ENSMUSG00000014301	PAM16	5	8	4	9	8	7	38	47	40	40	21	49
ENSMUSG00000014303	GLIS2	1	3	7	1	4	0	26	16	10	11	9	9
ENSMUSG00000014313	COX6C	65	59	70	57	73	67	476	535	344	474	392	449
ENSMUSG00000014329	BICC1	0	1	1	0	0	0	0	0	0	0	0	0
ENSMUSG00000014349	UBE2Z	9	4	6	13	13	6	40	37	41	42	33	23
ENSMUSG00000014351	GIP	0	0	0	0	0	0	0	1	0	0	0	0
ENSMUSG00000014353	TMEM87B	2	4	4	1	8	3	16	17	16	15	8	10
ENSMUSG00000014355	ANAPC1	14	14	16	25	23	12	71	73	57	89	55	65
ENSMUSG00000014402	TSG101	3	3	2	3	7	5	21	29	20	33	18	11
ENSMUSG00000014418	HPS5	1	4	1	6	3	3	23	20	16	19	20	20
ENSMUSG00000014426	MAP3K4	12	8	8	11	8	6	62	60	49	59	48	49
ENSMUSG00000014444	PIEZO1	9	13	10	12	14	8	52	52	37	49	39	43
ENSMUSG00000014470	RNF166	1	11	6	8	11	4	33	47	37	40	32	37
ENSMUSG00000014496	ANKRD28	6	5	1	5	4	3	16	26	10	18	16	14
ENSMUSG00000014498	ANKRD52	2	0	2	1	2	4	6	6	8	11	4	6
ENSMUSG00000014503	PKD2L2	0	1	1	5	2	5	3	5	1	5	2	2
ENSMUSG00000014504	SRP19	38	36	37	37	32	50	311	355	272	342	246	270
ENSMUSG00000014547	WDFY2	3	6	3	8	3	2	21	18	12	32	15	12
ENSMUSG00000014550	RBSN	3	6	3	5	2	5	22	21	15	23	22	20
ENSMUSG00000014551	MRPS25	10	8	16	18	15	16	69	89	49	93	79	62
ENSMUSG00000014554	DGUOK	4	5	2	1	3	2	7	13	15	16	7	14
ENSMUSG00000014592	CAMTA1	4	2	4	3	7	2	32	22	17	16	19	16
ENSMUSG00000014601	STRIP1	2	2	6	4	3	6	15	15	15	16	15	23
ENSMUSG00000014606	SLC25A11	4	4	7	8	8	8	31	38	20	32	26	27
ENSMUSG00000014609	CHRNE	0	0	0	0	0	0	2	0	2	1	0	1
ENSMUSG00000014633	CMC2	5	4	11	8	5	8	27	44	22	35	17	19
ENSMUSG00000014668	CHFR	4	5	9	15	8	6	51	42	31	50	37	33
ENSMUSG00000014747	ANKRD53	0	0	0	0	0	0	1	0	0	0	0	0
ENSMUSG00000014748	TEX261	8	10	13	10	16	16	49	41	28	51	35	35
ENSMUSG00000014763	FAM120B	8	13	5	14	13	15	49	62	38	54	36	39
ENSMUSG00000014767	TBP	10	11	8	6	13	18	81	66	57	87	52	56
ENSMUSG00000014769	PSMB1	62	95	92	94	106	110	550	634	450	584	422	526
ENSMUSG00000014771	PDCD2	9	14	15	19	16	13	80	87	70	85	74	60
ENSMUSG00000014773	DLL1	7	6	8	10	6	15	35	35	19	47	19	32
ENSMUSG00000014776	NOL3	0	0	0	1	0	0	1	0	0	1	2	1
ENSMUSG00000014778	FHOD1	0	2	1	2	2	4	10	10	8	9	5	9
ENSMUSG00000014782	PLEKHG4	0	1	0	0	0	0	0	0	0	0	0	0
ENSMUSG00000014786	SLC9A5	1	1	2	1	3	0	12	14	11	18	8	13
ENSMUSG00000014791	ELMO3	0	1	0	0	1	0	3	3	3	1	1	2
ENSMUSG00000014813	STC1	3	0	2	1	1	1	0	0	0	0	0	0
ENSMUSG00000014837	4931428F04F	0	0	0	0	0	0	8	3	4	3	4	1
ENSMUSG00000014846	TPPP3	0	0	0	0	0	1	1	1	0	1	1	2
ENSMUSG00000014850	MSH3	7	10	18	15	11	14	55	62	32	63	42	36
ENSMUSG00000014852	ADAMTS13	0	0	0	0	0	1	1	1	0	1	1	0
ENSMUSG00000014856	TMEM208	8	5	5	6	8	5	54	53	37	55	41	46
ENSMUSG00000014859	E2F4	8	9	10	7	12	16	68	65	51	62	54	55
ENSMUSG00000014867	SURF4	20	29	20	23	26	27	155	148	123	168	118	152
ENSMUSG00000014873	SURF2	10	12	18	12	10	10	61	59	53	75	66	53
ENSMUSG00000014907	NAF1	8	18	10	12	18	11	82	108	59	107	70	68
ENSMUSG00000014956	PPP1CB	27	29	28	35	32	31	283	235	178	285	170	193
ENSMUSG00000014959	GORASP2	25	24	34	43	38	30	134	120	135	143	119	148
ENSMUSG00000014980	TSEN15	4	2	5	2	9	3	23	20	20	24	24	14
ENSMUSG00000015002	EFR3A	15	10	26	36	24	29	69	56	44	74	56	62
ENSMUSG00000015013	TRAPPC2L	1	3	2	6	2	3	20	15	16	20	8	17
ENSMUSG00000015016	ACSF3	2	0	2	1	1	1	3	2	2	3	1	1

ENSMUSG00000015023	DDX19A	7	14	9	7	4	17	62	46	35	73	47	37
ENSMUSG00000015027	GALNS	0	1	0	2	0	0	3	8	2	3	1	3
ENSMUSG00000015083	C8G	0	0	0	0	0	0	2	0	1	0	0	0
ENSMUSG00000015087	RABL6	45	45	48	55	65	42	238	252	178	238	197	194
ENSMUSG00000015090	PTGDS	0	0	0	0	0	0	0	0	0	1	0	0
ENSMUSG00000015092	EDF1	31	32	33	46	42	37	230	290	199	267	184	228
ENSMUSG00000015095	FBXW5	3	3	4	2	5	1	10	17	13	20	10	11
ENSMUSG00000015112	SLC25A13	7	7	8	6	7	4	42	31	31	53	25	35
ENSMUSG00000015120	UBE2I	6	1	4	2	4	7	21	15	17	16	15	8
ENSMUSG00000015126	TSR3	10	11	14	6	9	7	43	42	38	39	30	43
ENSMUSG00000015127	UNKL	5	8	8	8	7	6	24	34	20	29	30	26
ENSMUSG00000015143	ACTN1	2	1	1	0	0	1	0	0	2	1	0	2
ENSMUSG00000015149	SIRT2	2	3	6	6	3	1	12	18	18	20	21	15
ENSMUSG00000015165	HNRNPL	463	551	566	594	563	584	2221	2281	1801	2331	1704	2084
ENSMUSG00000015176	NOLC1	29	61	65	81	76	57	396	410	296	439	296	342
ENSMUSG00000015189	CASD1	2	3	5	3	4	7	17	22	9	23	10	13
ENSMUSG00000015202	MAGI1	2	2	0	1	1	0	9	12	6	6	4	3
ENSMUSG00000015214	MTMR1	3	6	1	5	1	7	23	26	16	36	17	24
ENSMUSG00000015217	HMGB3	2	12	6	5	7	7	37	38	30	34	33	29
ENSMUSG00000015222	MAP2	3	2	3	3	1	1	17	24	5	18	13	14
ENSMUSG00000015243	ABCA1	0	0	0	0	0	0	0	0	0	0	1	0
ENSMUSG00000015247	NIPSNAP3B	0	1	0	0	0	0	0	0	0	0	0	0
ENSMUSG00000015289	LAGE3	0	2	2	4	0	3	17	22	12	21	13	16
ENSMUSG00000015290	UBL4A	8	11	11	14	11	16	63	76	47	71	51	70
ENSMUSG00000015291	GDI1	6	9	10	10	5	14	57	59	41	51	45	41
ENSMUSG00000015312	GADD45B	1	2	3	2	2	1	13	10	15	8	9	10
ENSMUSG00000015316	SLAMF1	0	0	0	0	0	0	1	0	0	0	0	0
ENSMUSG00000015335	ZDHHC12	6	2	5	7	7	5	31	25	17	36	21	25
ENSMUSG00000015337	ENDOG	1	1	2	1	5	6	12	16	16	19	12	14
ENSMUSG00000015340	CYBB	0	1	0	0	0	0	0	0	0	0	1	0
ENSMUSG00000015341	GOLGA7	2	1	1	4	2	2	15	9	9	17	12	15
ENSMUSG00000015354	PCOLCE2	0	1	0	0	0	1	2	2	1	5	1	2
ENSMUSG00000015355	CD48	0	0	0	0	1	0	0	0	0	0	0	0
ENSMUSG00000015357	CLPX	3	5	7	7	3	3	26	32	25	54	23	29
ENSMUSG00000015363	TRABD	11	13	6	6	13	12	40	35	28	40	23	36
ENSMUSG00000015377	DENND6B	5	4	2	2	3	5	21	18	12	22	15	24
ENSMUSG00000015451	C4A	0	0	0	0	0	0	0	0	1	0	0	0
ENSMUSG00000015452	AGER	0	1	0	1	0	0	3	1	1	2	0	1
ENSMUSG00000015461	ATF6B	4	6	5	10	10	1	34	25	27	42	27	21
ENSMUSG00000015467	EGFL8	33	33	30	36	26	26	76	59	59	69	58	62
ENSMUSG00000015468	NOTCH4	0	0	0	0	0	0	0	0	1	1	0	0
ENSMUSG00000015474	PPT2	1	0	0	1	0	0	0	0	0	0	0	0
ENSMUSG00000015478	RNF5	7	11	7	11	11	9	43	47	35	47	37	54
ENSMUSG00000015484	FAM163A	0	0	0	0	2	1	3	1	0	1	2	3
ENSMUSG00000015488	CACFD1	3	4	9	3	5	2	24	23	23	18	15	19
ENSMUSG00000015501	HIVEP2	2	4	1	11	3	6	10	10	14	14	9	14
ENSMUSG00000015522	ARNT	7	6	10	12	13	10	68	59	40	60	28	65
ENSMUSG00000015533	ITGA2	5	13	5	12	5	8	48	42	22	35	26	32
ENSMUSG00000015536	MOCS2	3	5	8	5	7	8	41	44	16	43	31	37
ENSMUSG00000015542	NAT9	0	0	0	0	1	1	2	2	3	1	0	1
ENSMUSG00000015575	ATP6V0E	10	12	10	19	5	8	63	72	67	71	61	55
ENSMUSG00000015597	ZFP318	5	18	12	13	14	14	61	65	51	73	60	53
ENSMUSG00000015605	SRF	3	7	0	3	2	3	16	22	16	14	12	19
ENSMUSG00000015619	GATA3	101	128	110	139	149	108	467	431	348	491	313	401
ENSMUSG00000015656	HSPA8	124	132	141	180	159	166	656	773	548	728	544	623
ENSMUSG00000015659	SERAC1	0	1	0	0	0	0	2	0	1	2	1	2
ENSMUSG00000015668	PDZD11	3	0	1	2	4	4	23	29	16	22	13	18
ENSMUSG00000015671	PSMA2	30	20	21	30	39	24	219	217	160	219	179	188

ENSMUSG00000015672	MRPL32	8	15	11	17	15	16	108	94	78	105	67	84
ENSMUSG00000015697	SETDB1	9	10	9	16	10	10	63	70	64	83	65	65
ENSMUSG00000015702	ANXA9	0	0	0	0	2	1	1	3	0	1	0	2
ENSMUSG00000015709	ARNT2	0	0	1	0	0	2	3	3	3	4	4	3
ENSMUSG00000015711	PRUNE1	0	1	1	0	0	0	4	7	6	2	0	3
ENSMUSG00000015714	CERS2	10	26	9	19	23	15	76	87	76	96	60	81
ENSMUSG00000015733	CAPZA2	7	13	8	19	15	10	91	104	80	101	79	81
ENSMUSG00000015745	PLEKHO1	2	0	5	0	3	6	4	11	4	6	2	4
ENSMUSG00000015747	VPS45	3	4	2	2	2	4	12	12	8	7	8	12
ENSMUSG00000015748	PRPF3	6	14	10	11	7	14	69	58	29	74	47	65
ENSMUSG00000015749	ANP32E	27	41	32	48	36	32	230	236	150	247	173	183
ENSMUSG00000015750	APH1A	1	1	2	1	0	3	5	6	6	7	6	4
ENSMUSG00000015755	TAB2	16	19	14	36	18	28	104	130	79	110	80	78
ENSMUSG00000015757	PPIL4	15	28	24	25	28	27	137	154	107	159	103	120
ENSMUSG00000015759	CNIH1	12	10	8	14	10	8	53	64	47	55	39	53
ENSMUSG00000015776	MED22	4	4	5	7	5	5	35	35	28	36	22	32
ENSMUSG00000015790	SURF1	6	10	10	10	10	9	37	59	38	40	39	59
ENSMUSG00000015804	MED28	6	4	2	6	2	6	34	34	16	28	24	32
ENSMUSG00000015806	QDPR	8	12	5	2	11	9	47	32	37	48	36	38
ENSMUSG00000015812	GNRH1	0	0	0	0	0	0	0	1	1	3	0	1
ENSMUSG00000015837	SQSTM1	10	13	12	9	5	10	46	59	53	54	43	46
ENSMUSG00000015839	NFE2L2	7	5	18	11	17	15	89	103	76	101	62	68
ENSMUSG00000015846	RXRA	0	1	0	0	1	0	0	0	0	0	0	0
ENSMUSG00000015850	ADAMTSL4	0	0	0	0	0	0	0	0	0	0	0	2
ENSMUSG00000015869	PRPSAP1	4	10	3	7	3	10	26	15	15	21	14	15
ENSMUSG00000015879	FAM184B	0	0	0	0	0	0	1	0	0	0	1	0
ENSMUSG00000015880	NCAPG	10	20	21	28	23	17	112	143	71	145	93	79
ENSMUSG00000015882	LCORL	9	13	8	13	16	8	72	75	66	79	40	62
ENSMUSG00000015889	LTA4H	5	8	11	19	14	15	67	69	48	69	53	48
ENSMUSG00000015890	AMDHD1	0	0	0	0	0	0	1	0	1	0	0	0
ENSMUSG00000015932	DSTN	6	4	6	5	6	2	31	22	14	21	17	15
ENSMUSG00000015937	H2AFY	95	124	124	144	136	104	548	596	408	564	424	465
ENSMUSG00000015942	GTF2IRD2	1	2	3	3	2	1	8	7	4	9	6	7
ENSMUSG00000015943	BOLA1	10	4	8	15	16	5	53	60	47	54	47	50
ENSMUSG00000015947	FCGR1	4	7	1	4	6	3	13	17	10	13	15	15
ENSMUSG00000015950	NCF1	0	0	0	0	0	1	0	0	0	0	2	0
ENSMUSG00000015957	WNT11	0	0	0	0	0	0	1	0	0	0	1	0
ENSMUSG00000015961	ADSS	12	17	18	24	16	15	110	133	84	137	80	94
ENSMUSG00000015968	CACNA1D	1	4	4	4	3	2	16	32	23	33	12	20
ENSMUSG00000015970	CHDH	1	2	2	3	1	2	5	7	4	7	5	3
ENSMUSG00000015971	ACTR8	4	6	5	5	3	3	38	32	22	22	22	35
ENSMUSG00000015994	FNTA	3	7	3	5	7	6	20	27	22	36	23	23
ENSMUSG00000016018	SKIV2L2	26	24	30	34	32	32	170	164	126	180	161	153
ENSMUSG00000016087	FLI1	20	33	27	24	21	42	112	112	90	123	100	104
ENSMUSG00000016181	DIEXF	5	7	3	12	10	3	30	36	26	20	27	26
ENSMUSG00000016194	HSD11B1	0	0	1	0	0	1	4	5	2	3	3	2
ENSMUSG00000016206	H2-M3	8	10	12	16	18	11	66	64	58	88	50	58
ENSMUSG00000016252	ATP5E	20	36	38	39	18	24	196	176	161	173	129	158
ENSMUSG00000016253	NELFCD	2	4	1	1	1	3	19	5	7	24	17	13
ENSMUSG00000016256	CTSZ	1	1	2	0	6	2	12	12	9	12	7	13
ENSMUSG00000016257	PRELID3B	8	13	11	18	13	17	133	103	87	156	99	118
ENSMUSG00000016308	UBE2A	11	4	4	10	11	9	50	57	39	68	42	44
ENSMUSG00000016319	SLC25A5	23	32	19	40	38	34	239	272	196	252	167	241
ENSMUSG00000016344	PPDPF	0	0	2	3	4	0	8	13	7	13	13	12
ENSMUSG00000016356	COL20A1	0	1	0	1	0	0	2	1	6	1	5	3
ENSMUSG00000016386	MPPED2	0	0	0	2	1	0	1	1	3	2	0	2
ENSMUSG00000016409	NKAP	10	24	9	15	13	6	44	49	29	49	29	34
ENSMUSG00000016427	NDUFA1	32	38	26	32	27	26	207	245	186	192	175	206

ENSMUSG00000016477	E2F3	10	26	18	17	20	27	83	97	78	97	84	71
ENSMUSG00000016481	CR1L	6	4	6	7	5	4	14	24	18	30	15	22
ENSMUSG00000016487	PPFIBP1	2	1	3	4	2	1	15	14	10	20	22	9
ENSMUSG00000016493	CD46	1	1	2	1	0	2	6	3	2	5	5	9
ENSMUSG00000016495	PLGRKT	2	1	6	2	3	5	26	33	23	34	28	30
ENSMUSG00000016496	CD274	0	0	2	1	1	1	2	3	4	4	1	1
ENSMUSG00000016498	PDCD1LG2	0	0	2	0	3	1	6	5	5	4	3	7
ENSMUSG00000016503	GTF3A	8	4	5	7	9	9	47	48	44	51	34	39
ENSMUSG00000016510	MTIF3	1	2	2	2	2	3	13	18	14	22	10	17
ENSMUSG00000016520	LNX2	1	1	0	0	2	1	10	11	8	9	3	5
ENSMUSG00000016526	DYRK3	0	0	4	0	1	1	9	4	4	3	3	5
ENSMUSG00000016528	MAPKAPK2	12	8	9	11	12	17	68	61	57	65	60	75
ENSMUSG00000016534	LAMP2	4	3	5	1	4	4	32	25	21	24	28	18
ENSMUSG00000016541	ATXN10	4	7	12	9	7	10	45	65	41	51	41	47
ENSMUSG00000016552	FOXRED2	0	0	0	0	1	0	2	7	3	5	6	3
ENSMUSG00000016554	EIF3D	12	27	26	21	33	20	142	151	127	145	106	133
ENSMUSG00000016559	H3F3B	40	58	51	56	47	70	414	414	329	448	331	351
ENSMUSG00000016619	NUP50	4	5	12	13	12	10	58	54	61	72	53	66
ENSMUSG00000016624	PHF21B	1	2	6	4	5	3	11	12	10	18	8	14
ENSMUSG00000016626	NLRP14	0	0	0	0	0	0	1	0	0	2	0	0
ENSMUSG00000016637	IFT27	7	4	8	9	8	4	45	63	43	58	38	48
ENSMUSG00000016664	PACSIN2	2	3	5	4	6	4	31	22	25	19	18	27
ENSMUSG00000016756	CMAH	79	79	53	93	71	85	416	418	324	438	289	375
ENSMUSG00000016757	TTLL12	3	5	3	4	4	6	28	36	30	33	27	30
ENSMUSG00000016831	TOX4	6	9	7	14	9	7	51	45	36	47	50	40
ENSMUSG00000016833	MRPS18C	12	15	13	11	20	12	96	114	65	87	62	68
ENSMUSG00000016918	SULF1	0	1	0	0	0	0	6	2	2	3	1	3
ENSMUSG00000016921	SRSF6	22	29	25	28	21	28	155	157	121	172	102	128
ENSMUSG00000016933	PLCG1	2	0	3	4	5	5	36	33	32	34	20	23
ENSMUSG00000016940	KCTD2	0	2	0	0	1	2	4	5	6	4	4	5
ENSMUSG00000016946	KCTD5	4	8	8	11	5	8	49	35	31	49	35	31
ENSMUSG00000016984	ETAA1	2	6	9	9	6	12	51	58	31	33	41	29
ENSMUSG00000017009	SDC4	0	0	1	0	0	1	0	0	0	0	0	0
ENSMUSG00000017119	NBR1	3	1	6	4	3	4	26	35	22	28	26	23
ENSMUSG00000017132	CYTH1	10	10	9	9	6	8	39	33	35	33	31	29
ENSMUSG00000017146	BRCA1	5	7	7	7	6	8	34	38	33	38	20	33
ENSMUSG00000017167	CNTNAP1	0	0	0	0	0	0	0	2	2	0	0	0
ENSMUSG00000017176	NT5C3B	18	19	20	19	16	27	71	73	67	70	49	72
ENSMUSG00000017188	COA3	3	1	8	3	6	4	33	25	21	32	23	21
ENSMUSG00000017210	MED24	8	10	11	15	9	11	58	62	53	70	41	44
ENSMUSG00000017221	PSMD3	21	20	19	23	15	19	106	134	79	106	91	110
ENSMUSG00000017264	EXOSC10	11	18	20	28	18	15	82	92	66	82	63	81
ENSMUSG00000017286	GLOD4	2	3	2	3	0	2	28	24	9	33	16	19
ENSMUSG00000017288	VPS53	3	4	7	3	8	10	32	38	18	29	21	26
ENSMUSG00000017291	TAOK1	15	21	23	17	11	14	67	66	51	70	57	68
ENSMUSG00000017299	DNTTIP1	1	0	2	1	1	0	8	10	6	10	7	7
ENSMUSG00000017300	TNNC2	0	0	0	0	0	0	2	0	0	1	0	0
ENSMUSG00000017307	ACOT8	1	2	2	4	1	1	8	11	7	8	5	4
ENSMUSG00000017376	NLK	16	16	12	18	19	17	78	84	47	93	55	69
ENSMUSG00000017386	TRAF4	1	0	1	0	0	0	2	2	2	1	2	1
ENSMUSG00000017390	ALDOC	0	0	0	0	0	0	0	0	0	0	0	2
ENSMUSG00000017404	RPL19	101	114	102	149	126	121	797	844	702	734	660	742
ENSMUSG00000017405	NEK8	0	1	1	1	1	1	9	8	5	1	2	5
ENSMUSG00000017412	CACNB4	0	0	1	0	0	0	2	0	0	0	2	0
ENSMUSG00000017417	PLXDC1	0	1	0	0	0	0	0	0	0	0	0	0
ENSMUSG00000017418	ARL5B	3	4	4	8	3	2	31	24	16	35	22	17
ENSMUSG00000017421	ZFP207	27	26	30	33	24	32	188	175	99	265	124	130
ENSMUSG00000017428	PSMD11	28	36	34	27	15	22	98	101	69	111	75	88

ENSMUSG00000017453	PIPOX	0	0	0	0	0	0	0	0	1	0	1	1
ENSMUSG00000017478	ZC3H18	27	31	21	38	39	34	182	167	119	169	126	168
ENSMUSG00000017485	TOP2B	40	55	49	53	44	52	379	380	239	459	271	260
ENSMUSG00000017491	RARB	0	0	0	0	0	0	1	0	0	1	0	0
ENSMUSG00000017493	IGFBP4	0	0	0	0	0	0	0	0	1	0	0	0
ENSMUSG00000017499	CDC6	11	11	15	15	15	14	103	103	63	97	75	63
ENSMUSG00000017548	SUZ12	36	51	38	73	43	46	332	280	190	347	226	214
ENSMUSG00000017550	ATAD5	16	26	20	27	19	26	121	97	82	130	62	80
ENSMUSG00000017561	CRLF3	12	16	16	16	18	11	82	111	66	79	94	89
ENSMUSG00000017607	TNS4	0	0	0	1	0	0	0	0	0	1	0	0
ENSMUSG00000017615	TNFAIP1	10	8	7	7	7	12	27	26	15	27	11	25
ENSMUSG00000017631	ABR	4	4	5	5	5	6	33	18	19	19	13	21
ENSMUSG00000017639	RAB11FIP4	0	1	0	0	0	0	0	0	0	0	0	0
ENSMUSG00000017664	SLC35C2	3	4	1	3	4	3	12	12	8	10	9	11
ENSMUSG00000017670	ELMO2	3	1	4	3	2	2	16	15	12	7	12	14
ENSMUSG00000017677	WSB1	9	15	19	29	7	11	92	94	91	102	88	75
ENSMUSG00000017679	TTPAL	0	2	2	1	0	1	8	2	7	4	3	9
ENSMUSG00000017686	RHOT1	8	12	2	4	5	13	43	49	38	61	32	30
ENSMUSG00000017692	RHBDL3	2	4	2	3	2	1	22	19	12	10	11	15
ENSMUSG00000017697	ADA	0	1	0	1	0	0	3	1	0	0	0	0
ENSMUSG00000017707	SERINC3	17	25	24	31	23	23	162	169	107	195	115	122
ENSMUSG00000017713	THA1	1	1	2	0	0	1	4	11	4	4	6	8
ENSMUSG00000017715	PGS1	2	3	4	2	3	13	19	22	19	13	18	17
ENSMUSG00000017716	BIRC5	13	19	18	12	10	14	72	80	43	86	55	79
ENSMUSG00000017718	AFMID	0	0	0	0	0	0	0	1	0	0	1	0
ENSMUSG00000017721	PIGT	0	2	1	3	4	2	8	3	3	4	8	5
ENSMUSG00000017723	WFDC2	0	0	0	0	0	0	0	0	0	2	0	1
ENSMUSG00000017724	ETV4	0	0	0	0	0	0	0	1	0	0	0	1
ENSMUSG00000017734	DBNDD2	0	0	0	0	0	0	0	3	1	0	0	0
ENSMUSG00000017737	MMP9	0	0	1	1	0	0	0	0	2	1	1	1
ENSMUSG00000017747	GHDC	0	2	0	2	0	1	1	3	1	4	2	2
ENSMUSG00000017754	PLTP	1	0	1	0	1	0	1	3	3	4	1	3
ENSMUSG00000017756	SLC12A7	1	0	2	3	2	2	14	12	10	11	10	15
ENSMUSG00000017760	CTSA	7	1	5	5	4	4	29	24	13	31	19	20
ENSMUSG00000017764	ZSWIM1	0	0	1	1	0	0	1	3	0	1	0	2
ENSMUSG00000017765	SLC12A4	1	0	4	2	5	1	3	4	2	2	4	4
ENSMUSG00000017774	MYO1C	4	0	2	2	1	0	12	11	12	11	5	9
ENSMUSG00000017776	CRK	17	13	10	11	7	7	76	73	46	70	44	43
ENSMUSG00000017778	COX7C	42	50	43	51	64	45	367	398	271	367	275	312
ENSMUSG00000017781	PITPNA	4	8	8	11	10	3	37	41	24	44	31	30
ENSMUSG00000017801	MLX	7	7	7	8	4	4	35	32	21	24	25	34
ENSMUSG00000017802	RETREG3	3	2	5	1	2	0	8	15	10	14	9	11
ENSMUSG00000017830	DHX58	0	0	0	1	0	0	1	0	2	1	1	0
ENSMUSG00000017831	RAB5A	2	2	0	4	1	1	11	14	6	12	6	12
ENSMUSG00000017837	NKIRAS2	2	3	3	3	1	2	9	4	13	14	10	9
ENSMUSG00000017843	PPP2R5C	18	18	25	26	16	45	122	101	73	149	76	85
ENSMUSG00000017858	IFT52	0	1	2	1	0	0	3	7	4	2	4	6
ENSMUSG00000017861	MYBL2	1	0	1	0	1	1	5	5	2	5	2	0
ENSMUSG00000017897	EYA2	3	4	3	8	3	2	10	25	7	22	18	21
ENSMUSG00000017929	B4GALT5	3	12	5	3	7	9	32	22	33	39	22	57
ENSMUSG00000017999	DDX27	26	24	25	41	16	29	127	168	117	164	117	130
ENSMUSG00000018001	CYTH3	0	0	0	0	0	0	0	0	1	0	0	2
ENSMUSG00000018008	CYTH4	5	2	4	5	2	7	13	31	14	18	24	16
ENSMUSG00000018012	RAC3	0	1	1	4	1	2	5	3	3	4	3	2
ENSMUSG00000018040	RRP7A	3	5	6	4	2	3	20	35	25	20	14	23
ENSMUSG00000018042	CYB5R3	1	2	2	7	3	2	17	31	14	19	18	33
ENSMUSG00000018068	INTS2	8	11	12	12	11	8	44	78	54	55	40	35
ENSMUSG00000018076	MED13L	50	68	75	65	71	73	232	264	182	311	176	239

ENSMUSG00000018102	HIST1H2BC	18	21	19	22	17	30	225	229	158	238	184	188
ENSMUSG00000018143	MAFK	2	2	4	2	3	1	17	20	14	17	10	22
ENSMUSG00000018160	MED1	15	17	12	12	20	14	78	89	48	85	48	69
ENSMUSG00000018166	ERBB3	0	2	2	5	1	2	0	0	1	0	0	0
ENSMUSG00000018167	STARD3	4	8	7	8	5	0	13	23	25	16	21	23
ENSMUSG00000018169	MFNG	4	7	4	6	10	6	39	54	41	43	49	31
ENSMUSG00000018171	VMP1	2	3	6	10	12	5	32	40	33	31	28	31
ENSMUSG00000018189	UCHL5	12	19	18	20	10	11	115	138	98	120	87	98
ENSMUSG00000018196	GLRX2	5	10	9	12	11	6	53	50	32	55	48	47
ENSMUSG00000018199	TROVE2	2	4	4	3	5	4	18	13	10	11	17	14
ENSMUSG00000018209	STK4	10	10	15	22	21	22	94	82	65	95	59	69
ENSMUSG00000018238	GDF9	0	0	1	0	0	0	0	0	1	0	0	0
ENSMUSG00000018239	ZCCHC10	8	13	9	13	9	8	70	55	52	77	42	68
ENSMUSG00000018286	PSMB6	33	30	26	36	32	25	174	205	143	186	156	172
ENSMUSG00000018287	SPAG7	2	4	6	4	5	7	36	59	38	47	28	46
ENSMUSG00000018293	PFN1	115	122	138	144	131	94	816	817	625	799	535	740
ENSMUSG00000018322	TOMM34	4	3	4	4	10	7	22	33	18	33	23	23
ENSMUSG00000018326	YWHAB	28	38	28	50	36	36	202	191	138	200	139	161
ENSMUSG00000018334	KSR1	0	0	2	3	0	2	12	7	6	17	5	12
ENSMUSG00000018340	ANXA6	11	18	11	30	15	15	54	65	45	68	53	65
ENSMUSG00000018341	IL12RB2	1	0	0	0	0	0	0	2	0	2	1	0
ENSMUSG00000018347	ZKSCAN6	1	3	0	0	1	1	2	3	3	6	5	7
ENSMUSG00000018362	KPNA2	0	0	0	1	0	0	5	4	2	7	2	4
ENSMUSG00000018363	SMURF2	4	4	4	4	4	1	14	16	11	14	14	17
ENSMUSG00000018372	CEP95	7	4	10	8	9	3	45	37	36	50	24	38
ENSMUSG00000018377	VEZF1	15	15	13	13	9	7	60	46	42	64	42	49
ENSMUSG00000018378	CUEDC1	2	2	5	4	2	4	19	14	8	17	12	16
ENSMUSG00000018379	SRSF1	43	57	50	64	62	60	376	364	300	438	243	313
ENSMUSG00000018387	SHROOM1	0	0	0	0	0	0	0	0	0	0	0	1
ENSMUSG00000018395	KIF3A	3	1	3	5	3	3	17	19	13	29	19	13
ENSMUSG00000018398	39692	3	3	2	1	2	4	15	5	7	14	8	8
ENSMUSG00000018401	MTMR4	2	1	1	1	1	1	10	12	13	15	7	9
ENSMUSG00000018405	MRM1	3	2	0	0	2	1	3	5	3	8	6	3
ENSMUSG00000018411	MAPT	0	0	0	0	0	0	0	0	0	1	0	1
ENSMUSG00000018412	KANSL1	8	14	10	13	16	15	78	76	29	82	59	49
ENSMUSG00000018415	GID4	2	4	4	3	7	3	10	13	10	15	11	15
ENSMUSG00000018425	DHX40	4	4	6	8	7	6	41	30	19	42	19	15
ENSMUSG00000018427	YPEL2	1	0	2	1	1	1	9	7	4	5	4	9
ENSMUSG00000018428	AKAP1	6	6	5	14	5	8	47	41	33	39	28	33
ENSMUSG00000018433	NOL11	9	14	12	12	9	6	60	63	48	53	43	49
ENSMUSG00000018442	DERL2	5	5	5	5	4	7	34	23	12	26	22	20
ENSMUSG00000018446	C1QBP	10	11	21	20	16	19	161	143	98	159	113	140
ENSMUSG00000018449	RPAIN	3	2	2	1	3	2	9	17	10	10	6	13
ENSMUSG00000018451	6330403K07F	0	0	0	0	0	0	1	2	0	2	1	0
ENSMUSG00000018470	KCNAB3	0	0	0	0	0	0	0	1	0	0	0	0
ENSMUSG00000018474	CHD3	31	46	30	39	37	37	131	158	100	135	118	130
ENSMUSG00000018476	KDM6B	23	23	16	24	13	18	69	92	50	82	44	85
ENSMUSG00000018479	1700125H20F	0	0	0	0	0	0	0	1	0	0	0	0
ENSMUSG00000018481	APPBP2	13	15	7	14	9	8	59	70	37	70	38	53
ENSMUSG00000018500	ADORA2B	0	0	0	0	0	0	0	0	0	2	0	0
ENSMUSG00000018501	NCOR1	65	86	75	103	83	73	267	321	251	344	220	247
ENSMUSG00000018507	TRPV2	14	10	8	11	12	12	58	77	44	77	51	61
ENSMUSG00000018509	CENPV	7	8	11	17	8	7	54	52	46	56	48	65
ENSMUSG00000018537	PCGF2	2	5	5	1	6	3	10	9	7	18	7	19
ENSMUSG00000018541	CWC25	5	6	2	4	5	4	21	21	24	17	19	16
ENSMUSG00000018543	1700001P01F	0	0	0	0	0	0	0	0	0	1	0	1
ENSMUSG00000018547	PIP4K2B	4	5	5	6	3	1	17	25	21	26	22	19
ENSMUSG00000018548	TRIM37	1	8	5	7	6	4	25	23	10	15	11	19

ENSMUSG00000018559	CTDNEP1	9	13	17	9	11	10	37	58	50	60	35	41
ENSMUSG00000018565	ELP5	9	6	10	10	6	2	36	40	33	36	31	39
ENSMUSG00000018567	GABARAP	11	4	6	7	6	7	40	44	38	35	32	42
ENSMUSG00000018572	PHF23	4	1	5	7	4	9	35	29	39	32	21	23
ENSMUSG00000018574	ACADVL	3	4	9	5	4	6	44	39	21	29	28	38
ENSMUSG00000018581	DNAH11	4	2	1	6	1	4	13	14	7	21	12	8
ENSMUSG00000018583	G3BP1	187	218	209	225	244	199	777	905	621	984	643	647
ENSMUSG00000018585	ATOX1	5	9	6	9	11	5	77	61	46	65	51	65
ENSMUSG00000018599	MIEF2	1	3	1	0	1	3	8	6	4	9	6	6
ENSMUSG00000018648	DUSP14	0	0	0	0	0	0	1	3	3	1	0	1
ENSMUSG00000018651	TADA2A	4	5	5	5	6	4	27	24	14	33	24	25
ENSMUSG00000018654	IKZF1	38	38	40	46	42	41	190	190	142	240	145	183
ENSMUSG00000018659	PNPO	1	1	6	5	3	3	17	17	13	12	12	8
ENSMUSG00000018661	COG1	4	1	3	1	2	0	7	2	5	11	9	10
ENSMUSG00000018666	CBX1	44	53	51	76	47	82	420	466	363	451	332	406
ENSMUSG00000018669	CDK5RAP3	3	5	0	6	5	3	19	29	21	20	9	18
ENSMUSG00000018672	COPZ2	0	0	0	0	1	1	0	0	0	0	0	0
ENSMUSG00000018677	SLC25A39	6	14	7	14	12	11	70	50	52	75	40	46
ENSMUSG00000018678	SP2	5	6	7	7	6	4	17	30	19	29	17	17
ENSMUSG00000018697	AATF	8	6	7	10	8	9	31	26	32	28	25	38
ENSMUSG00000018707	DYNC1H1	7	7	10	14	17	13	45	59	42	55	49	55
ENSMUSG00000018733	PEX12	1	2	2	1	2	4	6	11	12	12	16	7
ENSMUSG00000018736	NDEL1	3	3	2	3	1	3	15	20	17	13	17	10
ENSMUSG00000018740	SLC25A35	0	0	0	0	0	0	1	0	1	0	0	0
ENSMUSG00000018750	ZBTB4	4	1	2	1	1	1	2	4	3	1	1	4
ENSMUSG00000018761	MPDU1	2	5	2	2	3	6	21	22	14	22	19	15
ENSMUSG00000018765	FXR2	7	9	10	14	5	10	52	57	46	34	48	42
ENSMUSG00000018770	ATP5G3	75	73	96	94	95	73	719	725	564	822	556	610
ENSMUSG00000018774	CD68	0	0	0	0	0	0	3	0	1	2	0	0
ENSMUSG00000018796	ACSL1	0	1	1	4	2	3	13	8	5	11	5	8
ENSMUSG00000018800	ABCA5	0	0	1	0	1	0	5	1	5	1	3	3
ENSMUSG00000018809	SMYD4	0	3	3	3	2	2	12	10	7	3	5	13
ENSMUSG00000018819	LSP1	0	0	0	0	0	0	0	0	0	1	0	0
ENSMUSG00000018820	ZFYVE27	3	4	1	2	3	4	9	16	12	17	16	19
ENSMUSG00000018821	AVPI1	1	0	0	0	0	1	1	0	1	3	4	2
ENSMUSG00000018822	SFRP5	0	0	0	0	1	0	0	0	0	0	0	0
ENSMUSG00000018830	MYH11	0	0	0	1	0	0	1	1	0	1	0	0
ENSMUSG00000018841	RAD51D	4	1	2	1	2	2	17	14	12	8	14	18
ENSMUSG00000018844	FNDC8	0	0	0	1	3	0	2	2	2	2	1	5
ENSMUSG00000018846	PANK3	2	5	7	4	8	7	47	46	35	52	34	35
ENSMUSG00000018848	RARS	11	12	8	19	12	7	103	108	71	89	63	72
ENSMUSG00000018849	WWC1	0	0	0	0	0	0	1	0	0	1	1	0
ENSMUSG00000018858	MRPL58	3	3	4	7	8	6	21	20	14	30	15	17
ENSMUSG00000018861	FDXR	3	7	5	3	3	6	19	29	13	28	14	19
ENSMUSG00000018882	MRPL45	8	6	8	12	16	13	66	58	48	58	39	57
ENSMUSG00000018899	IRF1	3	8	8	3	10	9	30	30	23	25	28	31
ENSMUSG00000018900	SLC22A5	0	0	0	0	0	1	0	3	1	1	0	1
ENSMUSG00000018906	P4HA2	0	0	0	0	0	0	2	0	0	0	0	0
ENSMUSG00000018909	ARRB1	0	0	0	0	0	0	3	5	0	1	1	0
ENSMUSG00000018916	CSF2	2	0	0	4	1	2	1	4	2	5	3	7
ENSMUSG00000018920	CXCL16	1	0	0	0	0	0	0	0	0	0	1	0
ENSMUSG00000018921	PELP1	16	7	13	17	14	12	56	82	61	85	68	71
ENSMUSG00000018923	MED11	3	1	2	4	1	2	16	19	6	13	15	18
ENSMUSG00000018924	ALOX15	0	0	0	1	0	0	0	0	0	0	0	0
ENSMUSG00000018925	HEATR9	0	0	0	0	0	0	0	1	0	0	0	0
ENSMUSG00000018931	NATD1	0	0	0	0	0	0	2	2	0	1	0	0
ENSMUSG00000018932	MAP2K3	5	6	2	7	6	6	33	28	24	30	23	16
ENSMUSG00000018965	YWHAH	7	6	5	6	4	4	32	25	21	41	29	37

ENSMUSG00000018974	SART3	13	11	12	15	20	10	40	50	42	59	43	52
ENSMUSG00000018983	E2F2	8	3	6	9	7	6	27	20	25	29	19	20
ENSMUSG00000018986	SLFN3	1	6	3	4	1	3	10	14	8	14	16	6
ENSMUSG00000018995	NARS2	1	2	1	3	0	0	8	6	9	8	6	3
ENSMUSG00000018999	SLC35B4	12	7	11	7	13	10	46	34	25	45	25	44
ENSMUSG00000019039	DALRD3	5	9	5	15	7	8	42	48	29	27	54	41
ENSMUSG00000019054	FIS1	8	19	13	13	13	5	95	88	46	74	62	71
ENSMUSG00000019055	PLOD1	0	1	0	0	0	0	4	2	3	6	2	3
ENSMUSG00000019066	RAB3D	0	0	0	0	0	0	1	0	0	0	2	0
ENSMUSG00000019080	MFSD3	2	2	2	1	2	2	8	6	3	4	1	10
ENSMUSG00000019082	SLC25A22	0	6	5	7	7	3	25	21	16	23	17	29
ENSMUSG00000019087	ATP6AP1	4	4	1	4	2	7	34	25	13	29	18	22
ENSMUSG00000019088	DNASE1L1	0	0	0	0	0	0	4	4	2	2	0	0
ENSMUSG00000019132	BC005537	63	93	83	91	72	102	334	334	286	385	234	235
ENSMUSG00000019139	ISYNA1	7	3	7	14	12	5	46	45	24	33	25	38
ENSMUSG00000019143	HARS2	2	2	3	4	5	3	19	22	22	21	21	21
ENSMUSG00000019158	TMEM160	5	8	12	14	10	13	50	75	38	59	47	46
ENSMUSG00000019173	RAB5C	6	16	7	18	14	10	52	73	47	59	49	54
ENSMUSG00000019179	MDH2	19	30	29	30	28	30	231	249	185	247	199	200
ENSMUSG00000019188	H13	16	21	16	22	26	23	128	144	110	136	84	135
ENSMUSG00000019189	RNF145	4	11	12	8	7	9	67	74	43	86	59	53
ENSMUSG00000019210	ATP6V1E1	7	2	7	6	3	9	33	46	24	46	36	36
ENSMUSG00000019214	CHTF18	17	9	8	13	14	12	57	61	42	59	45	60
ENSMUSG00000019230	LHX9	0	1	0	0	1	0	2	0	0	0	0	0
ENSMUSG00000019235	RPS6KL1	2	3	3	1	1	1	6	7	5	8	10	8
ENSMUSG00000019254	PPP1R12C	18	13	18	23	16	9	67	73	45	69	66	63
ENSMUSG00000019261	MAP1S	4	5	4	3	7	0	14	16	18	21	13	19
ENSMUSG00000019295	TMEM129	0	6	2	2	4	4	12	15	14	11	18	10
ENSMUSG00000019297	NOP9	2	4	4	2	3	2	28	27	10	16	9	20
ENSMUSG00000019302	ATP6V0A1	0	2	0	1	0	0	0	4	1	5	1	2
ENSMUSG00000019303	PSMC3IP	2	3	2	5	6	5	34	34	26	32	18	31
ENSMUSG00000019320	NOXO1	1	0	0	0	0	0	0	0	0	1	0	1
ENSMUSG00000019326	AOC3	1	0	0	0	0	0	0	0	0	0	2	0
ENSMUSG00000019338	ZFP687	20	25	30	28	24	27	86	81	69	83	57	83
ENSMUSG00000019359	GDPD2	0	0	0	0	0	1	0	0	0	0	0	0
ENSMUSG00000019362	D8ERTD738E	20	24	17	18	21	21	128	126	109	141	98	136
ENSMUSG00000019370	CALM3	74	75	94	103	97	88	273	303	241	259	236	302
ENSMUSG00000019373	COPS3	10	12	11	13	12	10	86	95	76	75	57	62
ENSMUSG00000019428	FKBP8	18	25	13	21	13	10	114	110	63	76	73	102
ENSMUSG00000019432	DDX39B	39	61	39	37	61	43	210	202	172	207	187	214
ENSMUSG00000019433	GIPC1	1	4	1	3	3	0	6	14	3	12	1	8
ENSMUSG00000019437	TLCD1	29	36	26	34	48	37	216	221	174	268	174	173
ENSMUSG00000019461	PLSCR3	0	0	0	0	2	0	2	2	0	0	1	2
ENSMUSG00000019464	PTGER1	0	1	1	0	0	1	4	4	1	3	2	6
ENSMUSG00000019467	ARHGEF25	3	0	1	5	4	8	5	6	7	4	4	6
ENSMUSG00000019470	XAB2	7	13	9	12	7	14	37	42	26	36	45	32
ENSMUSG00000019471	CDC37	8	16	12	17	17	11	80	82	65	79	68	95
ENSMUSG00000019478	RAB4A	3	1	5	6	5	4	34	38	37	52	27	34
ENSMUSG00000019487	TRIP10	1	5	6	4	4	4	17	20	16	20	16	15
ENSMUSG00000019494	COPS6	14	19	15	16	15	20	80	106	62	106	83	67
ENSMUSG00000019505	UBB	31	40	31	36	39	29	188	215	156	196	165	192
ENSMUSG00000019518	AP4M1	20	24	22	18	21	26	89	146	81	98	86	115
ENSMUSG00000019528	GYG	18	27	11	13	20	14	105	121	96	115	75	76
ENSMUSG00000019539	RCN3	0	0	1	0	0	0	0	0	0	0	0	0
ENSMUSG00000019558	SLC6A8	0	0	0	0	0	0	0	2	0	0	1	0
ENSMUSG00000019564	ARID3A	8	7	17	14	19	20	64	50	58	73	64	58
ENSMUSG00000019578	UBXN6	6	12	6	6	10	18	58	60	36	68	41	48
ENSMUSG00000019579	MYDGF	14	14	15	20	15	18	103	101	80	93	85	87

ENSMUSG00000019659	CCDC12	1	1	2	2	1	4	17	8	19	16	8	13
ENSMUSG00000019689	FMC1	16	14	21	30	28	17	131	135	122	148	106	118
ENSMUSG00000019699	AKT3	0	0	0	0	0	0	0	0	0	0	0	1
ENSMUSG00000019710	MRPL24	21	22	15	22	42	17	147	142	120	133	107	99
ENSMUSG00000019715	GLE1	7	8	12	8	11	11	46	76	53	64	53	73
ENSMUSG00000019726	LYST	0	0	1	0	0	1	6	11	5	7	6	9
ENSMUSG00000019731	SLC35E1	7	11	9	3	9	5	21	19	19	22	18	16
ENSMUSG00000019732	CALR3	0	0	0	1	0	0	1	1	0	1	0	2
ENSMUSG00000019734	TMC4	1	1	1	0	1	1	0	1	0	1	1	1
ENSMUSG00000019737	SYNE4	1	0	0	1	0	1	0	2	2	1	0	0
ENSMUSG00000019738	POLR2I	4	4	6	6	4	10	36	30	25	32	41	30
ENSMUSG00000019763	RMND1	5	1	6	3	8	1	19	16	10	22	11	12
ENSMUSG00000019767	CCDC170	0	1	0	0	0	0	0	0	0	0	0	0
ENSMUSG00000019768	ESR1	3	2	4	0	2	0	7	12	5	7	3	7
ENSMUSG00000019773	FBXO5	11	31	10	15	11	14	81	95	69	101	67	77
ENSMUSG00000019774	MTRF1L	3	7	4	10	3	8	28	39	13	19	25	20
ENSMUSG00000019775	RGS17	0	0	0	0	0	0	0	1	0	0	0	0
ENSMUSG00000019777	HDAC2	17	25	25	36	27	21	148	145	103	167	104	104
ENSMUSG00000019782	RWDD1	13	17	9	16	7	4	66	42	43	40	40	42
ENSMUSG00000019787	TRDN	0	0	0	0	0	0	0	0	1	0	0	0
ENSMUSG00000019790	STXBP5	6	14	7	12	11	9	27	37	29	33	28	32
ENSMUSG00000019791	HINT3	0	2	2	2	1	1	1	5	4	1	3	6
ENSMUSG00000019792	TRMT11	2	5	1	2	4	2	20	23	12	30	23	19
ENSMUSG00000019794	KATNA1	5	6	3	5	4	8	28	32	25	33	30	21
ENSMUSG00000019795	PCMT1	5	4	12	13	7	4	43	47	34	56	43	34
ENSMUSG00000019796	LRP11	0	0	4	1	5	2	5	6	5	4	5	8
ENSMUSG00000019797	1700021F05F	2	7	3	7	6	2	15	23	15	20	16	28
ENSMUSG00000019802	SEC63	6	14	13	19	15	8	52	58	25	57	27	48
ENSMUSG00000019804	SNX3	14	15	10	9	15	13	96	88	67	101	78	85
ENSMUSG00000019806	AIG1	1	2	3	5	4	4	15	15	12	12	7	14
ENSMUSG00000019808	ADAT2	1	0	0	0	1	0	4	1	1	2	1	0
ENSMUSG00000019809	PEX3	1	3	2	1	8	4	16	18	15	16	20	7
ENSMUSG00000019810	FUCA2	3	4	2	2	2	4	9	8	11	6	7	8
ENSMUSG00000019813	CEP57L1	5	2	0	8	6	10	28	19	20	34	24	22
ENSMUSG00000019814	LTV1	4	8	4	4	7	13	41	40	33	43	38	31
ENSMUSG00000019817	PLAGL1	79	94	123	158	120	92	501	579	493	651	405	569
ENSMUSG00000019818	CD164	8	13	16	8	13	16	57	73	41	68	41	60
ENSMUSG00000019820	UTRN	7	18	16	10	12	12	62	50	47	72	46	46
ENSMUSG00000019822	SMPD2	3	7	4	8	4	2	15	26	9	13	16	12
ENSMUSG00000019823	MICAL1	4	3	5	3	6	7	12	29	13	17	14	14
ENSMUSG00000019826	ZBTB24	0	5	2	2	1	0	11	11	11	5	1	3
ENSMUSG00000019837	GTF3C6	12	8	8	11	2	7	35	50	41	47	43	37
ENSMUSG00000019838	SLC16A10	5	6	6	11	8	8	27	37	21	39	37	29
ENSMUSG00000019841	REV3L	13	12	10	12	12	7	67	55	34	63	26	33
ENSMUSG00000019842	TRAF3IP2	4	3	3	3	1	3	8	24	17	15	15	17
ENSMUSG00000019843	FYN	9	19	11	29	25	18	74	91	65	83	72	69
ENSMUSG00000019845	TUBE1	5	2	4	5	5	2	17	16	22	22	12	18
ENSMUSG00000019849	PREP	7	14	11	22	9	18	88	111	74	90	63	76
ENSMUSG00000019850	TNFAIP3	2	1	1	0	1	0	4	4	7	6	7	7
ENSMUSG00000019851	PERP	0	0	0	0	0	0	0	0	0	0	0	1
ENSMUSG00000019852	ARFGEF3	15	13	10	11	13	13	46	44	23	41	25	37
ENSMUSG00000019854	REPS1	5	5	8	7	6	10	32	33	29	33	25	33
ENSMUSG00000019857	ASF1A	14	16	14	6	7	8	60	59	47	76	55	53
ENSMUSG00000019861	GOPC	4	5	1	4	8	3	28	42	29	48	16	32
ENSMUSG00000019863	QRSL1	0	2	3	3	6	0	9	16	7	5	7	4
ENSMUSG00000019864	RTN4IP1	1	1	1	0	1	0	1	2	5	4	2	4
ENSMUSG00000019866	CRYBG1	0	0	0	0	0	1	0	0	1	0	0	0
ENSMUSG00000019868	VTA1	4	4	4	5	7	2	28	33	28	40	33	25

ENSMUSG00000019872	SMPDL3A	3	5	1	1	1	2	17	24	11	25	16	19
ENSMUSG00000019873	REEP3	3	3	2	8	2	4	21	15	11	17	4	11
ENSMUSG00000019877	SERINC1	5	17	11	9	6	6	71	92	61	89	40	65
ENSMUSG00000019878	HSF2	35	37	28	47	42	44	246	230	174	246	158	197
ENSMUSG00000019883	ECHDC1	4	2	5	2	3	2	31	19	20	28	15	21
ENSMUSG00000019889	PTPRK	0	0	0	0	0	0	1	0	1	2	1	3
ENSMUSG00000019897	CCDC59	14	33	19	23	24	25	129	120	80	129	78	106
ENSMUSG00000019907	PPP1R12A	20	71	36	56	43	36	138	146	108	166	92	100
ENSMUSG00000019916	P4HA1	4	2	0	4	0	4	32	30	23	27	18	28
ENSMUSG00000019917	40422	10	12	6	4	4	11	40	41	32	41	36	32
ENSMUSG00000019920	LIMS1	3	7	10	11	3	14	59	56	25	75	30	36
ENSMUSG00000019923	ZWINT	3	10	5	10	7	7	58	45	39	45	50	37
ENSMUSG00000019927	UBE2D1	3	3	5	6	4	9	23	29	10	30	15	17
ENSMUSG00000019929	DCN	0	0	0	0	0	0	0	0	0	1	0	0
ENSMUSG00000019933	MRLN	0	0	0	0	0	0	0	1	0	0	0	0
ENSMUSG00000019936	EPYC	0	0	0	0	0	0	0	1	0	0	0	0
ENSMUSG00000019942	CDK1	7	9	11	18	24	20	102	99	88	113	70	86
ENSMUSG00000019943	ATP2B1	33	54	30	47	48	53	162	169	129	175	86	115
ENSMUSG00000019945	CABCOCO1	0	0	0	0	0	0	0	1	0	1	1	1
ENSMUSG00000019947	ARID5B	4	3	5	6	4	6	22	37	16	32	30	29
ENSMUSG00000019948	ACTR6	1	3	3	0	4	0	22	21	12	17	14	28
ENSMUSG00000019951	UHRF1BP1L	9	12	5	15	8	4	46	55	40	43	47	40
ENSMUSG00000019952	POC1B	12	11	14	16	11	10	49	53	46	56	48	56
ENSMUSG00000019960	DUSP6	94	107	106	116	117	100	567	581	436	561	404	474
ENSMUSG00000019961	TMPO	28	44	42	50	45	32	287	254	219	321	232	235
ENSMUSG00000019969	PSEN1	5	2	4	1	3	1	19	17	10	10	11	12
ENSMUSG00000019970	SGK1	2	1	2	2	1	1	8	7	8	15	10	14
ENSMUSG00000019971	CEP290	0	0	0	0	0	0	0	0	1	2	0	0
ENSMUSG00000019975	IKBIP	6	12	13	9	12	9	54	56	45	61	38	53
ENSMUSG00000019977	HBS1L	2	9	4	7	8	4	42	30	28	36	24	29
ENSMUSG00000019978	EPB41L2	6	6	8	5	9	3	30	34	30	40	26	23
ENSMUSG00000019979	APAF1	3	6	9	5	5	5	35	42	24	38	33	34
ENSMUSG00000019982	MYB	8	10	14	7	7	6	42	64	53	42	28	44
ENSMUSG00000019984	MED23	4	8	3	7	6	1	14	21	13	22	11	17
ENSMUSG00000019986	AHI1	3	5	4	7	2	4	17	21	16	14	9	15
ENSMUSG00000019987	ARG1	3	4	8	4	12	6	23	13	16	19	5	12
ENSMUSG00000019988	NEDD1	0	3	2	6	6	3	18	16	12	30	16	17
ENSMUSG00000019989	ENPP3	0	0	0	1	0	0	0	0	0	0	0	0
ENSMUSG00000019992	MTFR2	2	2	2	1	1	0	2	5	2	5	3	6
ENSMUSG00000019998	STX7	0	0	0	0	0	0	0	0	1	3	0	1
ENSMUSG00000020000	MOXD1	0	0	0	0	0	0	0	1	0	0	0	0
ENSMUSG00000020003	PEX7	3	4	5	6	4	6	30	30	25	29	33	31
ENSMUSG00000020007	IL20RA	3	2	0	5	1	0	13	9	7	13	5	14
ENSMUSG00000020009	IFNGR1	22	12	24	27	24	27	71	122	66	105	64	84
ENSMUSG00000020014	CFAP54	0	0	1	0	0	2	0	1	1	0	1	0
ENSMUSG00000020015	CDK17	11	15	7	8	5	12	80	58	53	99	43	53
ENSMUSG00000020017	HAL	0	0	0	0	0	0	0	0	2	2	0	1
ENSMUSG00000020018	SNRPF	43	81	70	91	84	92	360	378	304	385	272	348
ENSMUSG00000020021	FGD6	0	3	3	1	2	3	9	11	3	13	11	9
ENSMUSG00000020022	NDUFA12	22	22	22	32	18	18	115	162	127	124	112	117
ENSMUSG00000020023	TMCC3	2	1	0	0	1	0	2	1	0	0	1	2
ENSMUSG00000020024	CEP83	20	50	24	40	46	23	138	148	88	163	123	129
ENSMUSG00000020027	SOCS2	0	1	0	0	0	0	0	2	1	0	0	0
ENSMUSG00000020029	NUDT4	14	14	7	12	9	9	78	63	41	61	49	56
ENSMUSG00000020034	TCP11L2	1	2	3	4	4	0	16	19	20	24	17	13
ENSMUSG00000020037	RFX4	0	0	1	0	0	0	1	2	0	1	1	1
ENSMUSG00000020038	CRY1	3	0	1	2	0	2	9	12	7	21	10	6
ENSMUSG00000020048	HSP90B1	95	119	117	131	145	145	784	934	591	952	613	641

ENSMUSG00000020056	WASHC3	2	1	6	6	3	1	20	17	14	18	22	15
ENSMUSG00000020059	SYCP3	0	0	0	0	0	0	1	2	1	3	2	0
ENSMUSG00000020061	MYBPC1	0	0	0	0	0	0	0	1	0	0	0	0
ENSMUSG00000020063	SIRT1	1	5	3	7	5	2	17	34	31	29	20	28
ENSMUSG00000020064	HERC4	13	13	13	13	15	8	46	27	35	39	37	37
ENSMUSG00000020069	HNRNPH3	6	20	8	19	19	13	50	56	51	63	45	58
ENSMUSG00000020070	RUFY2	4	4	6	3	2	2	21	19	20	18	28	17
ENSMUSG00000020072	PBLD2	0	0	0	0	0	0	0	1	0	0	0	0
ENSMUSG00000020074	CCAR1	39	54	54	84	70	68	291	293	234	299	243	245
ENSMUSG00000020075	DDX21	28	37	32	73	50	33	258	240	164	225	164	194
ENSMUSG00000020076	DDX50	5	12	15	18	18	9	59	52	42	67	53	49
ENSMUSG00000020077	SRGN	2	8	2	6	6	3	23	29	15	42	19	15
ENSMUSG00000020078	VPS26A	8	8	10	12	13	7	58	59	47	50	40	54
ENSMUSG00000020079	SUPV3L1	7	5	5	4	1	6	23	24	22	27	23	21
ENSMUSG00000020085	AIFM2	2	0	0	0	0	0	8	8	4	7	5	4
ENSMUSG00000020086	H2AFY2	0	0	0	0	0	0	1	0	1	3	0	0
ENSMUSG00000020087	TYSND1	2	0	0	1	0	4	9	8	8	16	9	10
ENSMUSG00000020088	SAR1A	12	15	18	24	10	15	72	73	66	75	55	65
ENSMUSG00000020089	PPA1	8	9	7	12	10	14	63	63	50	68	36	48
ENSMUSG00000020090	NPFFR1	0	0	0	0	1	0	0	0	0	0	0	0
ENSMUSG00000020091	EIF4EBP2	7	14	9	8	18	11	64	61	47	51	39	58
ENSMUSG00000020092	PALD1	0	0	0	0	0	0	0	0	0	1	0	0
ENSMUSG00000020096	TBATA	0	1	1	0	1	0	0	1	0	0	1	0
ENSMUSG00000020097	SGPL1	2	7	6	4	5	4	17	13	9	22	12	17
ENSMUSG00000020100	SLC29A3	1	0	0	0	0	1	0	1	2	0	0	1
ENSMUSG00000020101	VSIR	2	1	1	3	3	3	10	5	7	14	6	11
ENSMUSG00000020105	LRIG3	0	0	0	0	0	0	0	0	0	0	1	0
ENSMUSG00000020107	ANAPC16	8	8	6	4	6	7	40	35	13	32	24	24
ENSMUSG00000020108	DDIT4	9	8	7	8	10	9	62	61	55	60	50	54
ENSMUSG00000020109	DNAJB12	10	16	9	18	16	14	65	77	46	64	46	62
ENSMUSG00000020111	MICU1	0	2	3	1	0	0	5	8	7	3	2	9
ENSMUSG00000020114	CAND1	7	12	18	13	10	13	76	83	54	66	66	58
ENSMUSG00000020115	TBK1	4	7	3	5	5	5	34	46	30	51	27	32
ENSMUSG00000020116	PNO1	12	15	11	18	16	14	90	97	72	74	47	78
ENSMUSG00000020120	PLEK	0	0	0	0	0	0	1	1	0	1	0	0
ENSMUSG00000020123	AVPR1A	0	0	0	0	0	0	1	0	0	2	0	1
ENSMUSG00000020124	USP15	9	11	10	22	19	14	82	91	64	103	64	58
ENSMUSG00000020128	VPS54	14	16	20	24	17	17	66	89	41	61	48	58
ENSMUSG00000020130	TBC1D15	8	6	11	8	11	14	48	49	38	53	39	42
ENSMUSG00000020131	PCSK4	0	2	0	1	0	0	3	6	6	4	2	4
ENSMUSG00000020132	RAB21	9	10	9	9	15	14	57	64	40	65	41	41
ENSMUSG00000020133	2310011J03R	5	3	6	2	2	4	9	16	7	12	7	12
ENSMUSG00000020134	PELI1	13	14	16	15	10	12	61	66	49	96	34	47
ENSMUSG00000020135	APC2	0	0	1	0	0	0	1	0	0	0	0	0
ENSMUSG00000020137	THAP2	2	1	4	4	5	2	25	20	21	23	10	18
ENSMUSG00000020140	LGR5	19	9	9	9	12	9	24	28	32	36	18	21
ENSMUSG00000020142	SLC1A4	1	2	2	10	4	8	23	29	16	41	18	28
ENSMUSG00000020143	DOCK2	8	10	15	19	14	16	80	85	46	79	52	62
ENSMUSG00000020149	RAB1	15	27	24	20	24	27	197	191	152	225	116	142
ENSMUSG00000020150	GAMT	0	0	0	0	0	0	0	0	0	0	0	1
ENSMUSG00000020152	ACTR2	56	74	60	90	73	71	352	379	228	397	219	278
ENSMUSG00000020153	NDUFS7	13	13	12	18	12	16	72	73	49	84	62	80
ENSMUSG00000020154	PTPRB	0	0	0	0	0	0	0	0	0	0	1	0
ENSMUSG00000020155	KCNMB1	0	4	2	5	3	0	18	10	16	22	13	7
ENSMUSG00000020156	MUM1	5	4	13	5	3	10	23	31	35	41	23	29
ENSMUSG00000020163	UQCR11	18	19	18	26	18	18	144	139	111	118	114	119
ENSMUSG00000020166	CNOT2	4	7	6	9	6	8	54	62	39	62	37	38
ENSMUSG00000020167	TCF3	18	33	29	28	24	20	114	128	93	125	81	98

ENSMUSG00000020170	FRS2	5	3	3	6	2	7	17	10	5	18	13	18
ENSMUSG00000020171	YEATS4	5	10	6	18	6	15	61	64	40	72	42	53
ENSMUSG00000020175	RAB36	0	1	0	0	0	0	1	1	0	0	0	1
ENSMUSG00000020176	GRB10	1	0	1	2	0	0	4	1	0	4	2	0
ENSMUSG00000020178	ADORA2A	2	1	3	1	0	2	8	7	7	5	12	7
ENSMUSG00000020180	SNRPD3	63	77	66	70	76	66	493	523	368	507	389	445
ENSMUSG00000020182	DDC	9	7	1	4	2	4	31	33	24	38	30	36
ENSMUSG00000020183	CPM	0	0	1	0	0	0	0	0	0	0	0	0
ENSMUSG00000020184	MDM2	7	10	5	11	12	14	56	56	51	66	49	50
ENSMUSG00000020185	E2F7	1	1	1	0	0	1	0	3	1	3	0	1
ENSMUSG00000020186	CSRP2	0	0	0	0	1	0	0	0	0	0	0	0
ENSMUSG00000020189	OSBPL8	7	10	18	11	9	8	48	47	33	54	36	36
ENSMUSG00000020190	MKNK2	18	24	23	32	29	41	127	140	110	153	130	139
ENSMUSG00000020191	SPATA48	0	0	0	0	0	0	0	1	0	1	0	2
ENSMUSG00000020193	ZPBP	1	0	0	0	0	0	0	1	1	1	0	0
ENSMUSG00000020196	CABIN1	13	9	12	11	17	9	69	76	51	82	43	43
ENSMUSG00000020198	AP3D1	11	18	9	27	28	22	65	64	70	82	72	63
ENSMUSG00000020205	PHLDA1	25	37	29	29	45	20	146	143	128	148	112	124
ENSMUSG00000020211	SF3A2	13	12	8	14	11	14	54	72	57	56	61	54
ENSMUSG00000020212	MDM1	2	3	1	3	6	6	19	23	15	34	11	19
ENSMUSG00000020216	JSRP1	0	0	0	0	0	0	0	1	0	0	0	0
ENSMUSG00000020219	TIMM13	7	16	16	13	20	12	97	83	74	101	64	91
ENSMUSG00000020220	VPS13D	0	3	3	1	3	0	16	23	12	16	13	9
ENSMUSG00000020224	LLPH	5	7	6	13	6	3	41	38	36	60	31	29
ENSMUSG00000020225	TMBIM4	2	4	3	4	4	4	27	23	14	22	8	16
ENSMUSG00000020228	HELB	4	5	10	10	3	9	35	38	24	48	33	20
ENSMUSG00000020230	PRMT2	0	0	0	0	0	1	5	2	0	2	1	0
ENSMUSG00000020231	DIP2A	1	0	2	2	1	2	6	11	10	8	3	8
ENSMUSG00000020232	HMG20B	9	10	3	10	10	11	46	40	27	47	41	35
ENSMUSG00000020234	4930404N11I	1	0	0	0	1	2	1	0	1	1	0	0
ENSMUSG00000020235	FZR1	4	4	1	6	8	7	13	15	12	24	11	13
ENSMUSG00000020238	NCLN	9	13	5	9	11	5	36	41	21	41	32	29
ENSMUSG00000020246	HCFC2	1	4	0	4	3	5	12	14	12	24	7	7
ENSMUSG00000020248	NFYB	7	2	8	6	3	6	30	40	21	33	26	29
ENSMUSG00000020250	TXNRD1	7	17	21	23	16	21	111	116	88	123	54	100
ENSMUSG00000020251	GLT8D2	0	0	0	0	1	0	0	0	1	0	0	0
ENSMUSG00000020253	PPM1M	3	4	2	2	2	5	24	23	19	19	10	10
ENSMUSG00000020255	D10WSU102E	13	9	11	12	9	7	55	46	38	61	46	41
ENSMUSG00000020256	ALDH1L2	0	0	0	1	0	1	0	1	0	0	0	1
ENSMUSG00000020257	WDR82	6	13	9	8	10	4	50	49	31	60	31	59
ENSMUSG00000020258	GLYCTK	1	0	0	1	0	0	2	0	0	1	1	2
ENSMUSG00000020260	POFUT2	5	9	10	7	4	5	19	14	22	20	23	23
ENSMUSG00000020261	SLC36A1	3	3	0	8	3	1	7	3	8	7	12	6
ENSMUSG00000020262	ADARB1	0	3	1	3	2	5	21	14	13	13	11	9
ENSMUSG00000020263	APPL2	8	7	4	7	4	8	35	34	21	45	27	24
ENSMUSG00000020265	SUMO3	13	3	13	14	11	10	44	53	40	50	33	33
ENSMUSG00000020267	HINT1	66	86	79	79	90	83	435	507	326	452	365	429
ENSMUSG00000020268	LYRM7	0	1	0	2	0	2	5	5	6	0	3	8
ENSMUSG00000020271	FBXW11	1	2	1	8	1	5	26	24	17	27	18	14
ENSMUSG00000020272	STK10	9	18	10	13	18	10	49	59	49	82	44	65
ENSMUSG00000020273	PAPOLG	1	1	2	0	4	4	15	11	11	14	7	9
ENSMUSG00000020275	REL	0	3	1	2	2	2	12	14	6	13	9	10
ENSMUSG00000020277	PFKL	7	4	15	11	8	12	60	55	27	47	29	38
ENSMUSG00000020279	IL9R	12	16	12	14	12	14	74	95	61	88	44	65
ENSMUSG00000020280	PUS10	4	11	4	6	8	3	37	31	21	42	29	20
ENSMUSG00000020283	PEX13	4	10	4	6	1	2	20	19	10	22	13	24
ENSMUSG00000020284	1810043G02I	1	1	2	2	0	1	4	7	4	6	6	7
ENSMUSG00000020286	1700093K21F	0	0	1	0	0	0	0	0	0	1	1	1

ENSMUSG00000020287	MPG	7	2	3	2	4	4	15	14	11	18	14	13
ENSMUSG00000020288	AHSA2	7	13	13	20	13	13	102	89	56	102	78	79
ENSMUSG00000020289	NPRL3	1	1	1	3	3	2	13	4	4	8	4	7
ENSMUSG00000020290	XPO1	33	41	41	44	37	49	257	239	174	246	187	216
ENSMUSG00000020297	NSG2	0	0	0	0	0	0	1	0	0	0	0	0
ENSMUSG00000020300	CPEB4	2	2	4	6	7	2	20	20	9	22	15	20
ENSMUSG00000020303	STC2	1	3	1	2	0	3	5	8	13	11	6	3
ENSMUSG00000020305	ASB3	2	1	0	2	1	2	11	11	8	24	8	2
ENSMUSG00000020307	CDC34	12	14	21	17	28	22	98	104	82	131	94	95
ENSMUSG00000020308	TPGS1	2	2	1	2	3	2	8	10	9	9	7	3
ENSMUSG00000020309	CHAC2	1	0	0	1	1	3	9	6	3	9	3	13
ENSMUSG00000020311	ERLEC1	2	6	6	4	5	3	22	27	15	25	22	25
ENSMUSG00000020315	SPTBN1	21	23	33	31	32	21	128	138	91	164	96	121
ENSMUSG00000020319	WDPCP	0	1	0	1	0	0	0	3	0	1	1	0
ENSMUSG00000020321	MDH1	26	40	35	30	42	26	239	287	208	257	179	180
ENSMUSG00000020323	PRSS57	0	0	0	0	0	0	4	6	1	2	1	1
ENSMUSG00000020325	FSTL3	0	0	0	1	0	0	0	2	2	1	1	0
ENSMUSG00000020326	CCNG1	5	5	6	4	5	8	20	22	22	31	25	22
ENSMUSG00000020327	FGF22	2	3	2	0	1	1	5	6	4	8	5	4
ENSMUSG00000020328	NUDCD2	42	33	32	53	66	41	259	305	216	276	195	176
ENSMUSG00000020329	POLRMT	1	3	1	2	6	2	22	23	19	15	14	17
ENSMUSG00000020330	HMMR	12	17	23	17	22	24	91	107	72	96	77	69
ENSMUSG00000020331	HCN2	0	0	2	1	0	0	1	5	4	2	3	4
ENSMUSG00000020333	ACSL6	0	0	0	0	1	1	0	0	0	0	0	0
ENSMUSG00000020340	CYFIP2	15	18	23	21	31	14	119	113	106	130	72	87
ENSMUSG00000020346	MGAT1	3	8	2	4	3	4	23	30	13	23	25	24
ENSMUSG00000020349	PPP2CA	13	17	15	19	12	19	88	107	72	145	66	76
ENSMUSG00000020354	SGCD	0	0	0	0	0	0	0	0	0	0	0	1
ENSMUSG00000020358	HNRNPAB	52	83	57	68	64	73	469	468	321	506	358	384
ENSMUSG00000020359	PHYKPL	12	24	17	23	17	31	92	83	68	121	78	77
ENSMUSG00000020361	HSPA4	27	45	31	50	44	40	196	253	181	272	185	200
ENSMUSG00000020362	CNOT6	16	29	16	16	28	37	139	113	87	134	88	96
ENSMUSG00000020366	MAPK9	1	6	3	4	5	2	18	30	15	25	21	14
ENSMUSG00000020368	CANX	52	75	77	86	78	83	458	457	355	519	323	358
ENSMUSG00000020372	RACK1	172	249	235	275	254	224	1505	1644	1261	1568	1183	1393
ENSMUSG00000020375	RUFY1	0	1	0	1	0	0	0	4	0	2	0	4
ENSMUSG00000020380	RAD50	18	24	32	49	42	35	119	136	83	131	90	93
ENSMUSG00000020381	MRNIP	1	1	1	1	2	0	3	9	7	9	6	1
ENSMUSG00000020385	CLK4	14	9	13	12	6	15	40	62	65	81	46	43
ENSMUSG00000020386	SAR1B	3	4	10	6	6	6	60	54	46	40	32	39
ENSMUSG00000020387	JADE2	4	0	2	4	1	1	6	2	8	8	6	10
ENSMUSG00000020388	PDLIM4	0	0	0	0	0	0	0	0	0	0	1	0
ENSMUSG00000020389	CDKL3	0	1	1	0	1	1	2	5	0	6	6	3
ENSMUSG00000020390	UBE2B	26	35	22	34	27	19	190	179	124	227	150	135
ENSMUSG00000020392	CDKN2AIPNL	6	10	8	8	12	11	61	72	41	62	46	43
ENSMUSG00000020393	KREMEN1	8	8	5	6	9	4	24	27	21	19	13	21
ENSMUSG00000020395	ITK	13	11	13	21	26	18	65	77	59	79	55	39
ENSMUSG00000020397	MED7	8	4	1	12	5	3	42	38	24	36	24	22
ENSMUSG00000020399	HAVCR2	0	0	0	0	1	0	0	0	1	1	0	0
ENSMUSG00000020400	TNIP1	1	6	3	4	3	2	18	20	11	27	11	15
ENSMUSG00000020401	FAM71B	2	1	3	1	3	4	7	10	5	14	14	6
ENSMUSG00000020402	VDAC1	32	49	36	44	34	35	242	258	225	263	206	230
ENSMUSG00000020409	SLU7	2	8	10	8	8	9	48	40	30	62	31	34
ENSMUSG00000020412	ASCC2	2	3	3	3	7	4	20	16	11	18	12	15
ENSMUSG00000020413	HUS1	2	2	1	2	3	3	14	14	14	18	9	11
ENSMUSG00000020415	PTTG1	0	1	1	0	1	2	13	12	5	11	10	6
ENSMUSG00000020420	ZFP607A	2	1	0	2	2	3	3	2	4	9	3	3
ENSMUSG00000020423	BTG2	2	0	2	0	3	1	0	0	0	0	0	0

ENSMUSG00000020424	GATSL3	0	0	0	0	0	1	1	1	1	0	1	1
ENSMUSG00000020430	PES1	2	4	4	5	6	1	15	25	18	21	22	17
ENSMUSG00000020431	ADCY1	0	3	0	1	1	1	9	3	3	4	4	1
ENSMUSG00000020432	TCN2	0	0	0	0	1	0	0	0	0	1	0	0
ENSMUSG00000020437	MYO1G	19	30	24	28	24	19	131	132	96	142	104	107
ENSMUSG00000020440	ARF5	64	80	63	77	77	84	262	311	238	322	231	304
ENSMUSG00000020441	2310033P09F	12	18	15	9	16	45	71	73	27	123	51	76
ENSMUSG00000020444	GUK1	4	5	3	3	4	2	20	25	15	19	20	24
ENSMUSG00000020447	NPC1L1	0	0	2	1	0	0	1	3	3	2	0	2
ENSMUSG00000020448	RNF185	1	1	1	3	2	1	9	12	14	9	9	10
ENSMUSG00000020451	LIMK2	1	3	4	5	5	6	21	20	20	27	25	25
ENSMUSG00000020453	PATZ1	1	5	3	5	4	4	37	39	22	45	25	24
ENSMUSG00000020454	EIF4ENIF1	11	10	7	10	15	10	52	64	38	48	49	30
ENSMUSG00000020455	TRIM11	6	0	1	5	4	2	12	13	7	21	14	9
ENSMUSG00000020456	OGDH	17	10	15	14	13	11	68	70	46	72	39	51
ENSMUSG00000020457	DRG1	5	9	11	5	10	4	30	35	27	31	34	32
ENSMUSG00000020458	RTN4	9	9	9	13	12	14	68	73	54	82	45	50
ENSMUSG00000020459	MTIF2	5	8	9	12	9	6	38	54	39	52	31	47
ENSMUSG00000020460	RPS27A	181	220	225	334	269	265	912	974	738	911	762	853
ENSMUSG00000020461	CLHC1	0	0	0	1	0	1	0	0	0	2	0	0
ENSMUSG00000020462	CFAP36	6	14	10	7	8	7	52	45	36	48	34	42
ENSMUSG00000020463	PPP4R3B	6	14	11	13	17	7	84	60	40	81	53	43
ENSMUSG00000020464	PNPT1	11	12	14	13	12	19	77	93	52	80	81	65
ENSMUSG00000020471	POLD2	8	15	11	21	18	12	50	82	50	63	55	58
ENSMUSG00000020472	ZKSCAN17	2	2	1	0	0	0	7	16	13	8	6	6
ENSMUSG00000020473	AEBP1	0	0	0	0	0	0	0	1	0	0	0	0
ENSMUSG00000020474	POLM	1	3	4	0	3	2	7	7	7	5	8	7
ENSMUSG00000020476	DBNL	5	8	2	9	7	1	36	34	36	35	23	36
ENSMUSG00000020477	MRPS24	7	8	6	9	10	13	48	50	47	39	44	52
ENSMUSG00000020482	CCDC117	3	3	3	6	4	3	34	40	27	30	21	21
ENSMUSG00000020483	DYNLL2	13	17	8	20	22	13	116	110	79	118	98	115
ENSMUSG00000020484	XBP1	4	10	4	6	8	6	32	38	24	30	31	21
ENSMUSG00000020485	SUPT4A	1	2	4	1	1	1	27	19	18	20	17	24
ENSMUSG00000020486	38231	0	0	0	1	0	0	1	1	2	1	0	3
ENSMUSG00000020491	2810021J22R	0	0	0	1	1	2	2	4	5	3	2	2
ENSMUSG00000020492	SKA2	5	16	13	14	9	12	55	61	58	63	54	47
ENSMUSG00000020493	PRR11	5	4	3	8	3	1	13	16	16	21	9	13
ENSMUSG00000020495	SMG8	1	2	2	1	1	2	8	10	7	17	10	20
ENSMUSG00000020496	RNF187	3	9	9	8	8	10	48	46	46	54	45	44
ENSMUSG00000020513	TUBD1	0	1	2	1	0	1	3	7	4	5	2	2
ENSMUSG00000020514	MRPL22	2	4	2	4	7	2	15	22	21	26	17	21
ENSMUSG00000020515	CNOT8	8	10	4	8	10	7	44	39	36	40	25	30
ENSMUSG00000020516	RPS6KB1	17	27	21	22	23	27	139	128	90	146	97	120
ENSMUSG00000020519	SAP30L	6	6	6	7	8	4	37	36	32	34	23	29
ENSMUSG00000020520	GALNT10	0	0	0	0	0	0	0	0	0	0	1	0
ENSMUSG00000020521	RNFT1	7	5	5	9	10	6	61	40	42	65	46	43
ENSMUSG00000020522	MFAP3	2	4	8	10	5	2	18	29	13	28	17	20
ENSMUSG00000020523	FAM114A2	10	9	8	9	8	7	34	37	22	52	29	32
ENSMUSG00000020525	PPM1D	5	1	2	4	5	3	16	17	12	21	11	11
ENSMUSG00000020526	ZNHIT3	3	5	6	6	7	3	33	46	21	45	27	20
ENSMUSG00000020527	MYO19	2	4	2	3	4	2	18	18	11	20	8	13
ENSMUSG00000020528	PRPSAP2	2	0	2	2	1	5	22	22	13	28	11	16
ENSMUSG00000020530	GGNBP2	32	30	24	23	35	31	142	127	93	160	102	121
ENSMUSG00000020532	ACACA	5	7	9	9	7	6	31	40	38	32	22	19
ENSMUSG00000020534	SHMT1	9	11	8	10	17	9	65	79	67	65	53	62
ENSMUSG00000020536	LLGL1	3	5	2	2	8	1	14	17	9	20	13	12
ENSMUSG00000020537	DRG2	3	2	6	2	4	4	28	28	29	24	17	19
ENSMUSG00000020538	SREBF1	6	12	15	15	11	12	77	85	69	85	50	89

ENSMUSG00000020541	TOM1L1	0	0	1	0	1	1	3	6	1	1	3	2
ENSMUSG00000020544	COX11	3	2	1	2	1	2	10	15	8	10	9	12
ENSMUSG00000020545	LRRC72	0	0	0	0	0	0	0	1	0	0	0	0
ENSMUSG00000020546	STXBP4	0	0	1	2	2	2	3	7	7	4	5	4
ENSMUSG00000020547	BZW2	37	37	55	47	48	47	248	252	181	270	209	217
ENSMUSG00000020549	ELAC2	3	1	1	2	3	1	12	12	8	18	11	11
ENSMUSG00000020561	TWISTNB	21	18	18	37	26	21	111	121	94	121	83	100
ENSMUSG00000020564	ATXN7L1	0	0	0	1	0	0	3	3	2	1	3	4
ENSMUSG00000020566	ATP6V1C2	0	0	0	0	0	0	0	0	0	1	0	0
ENSMUSG00000020570	SYPL	21	25	19	18	29	20	87	89	57	94	71	69
ENSMUSG00000020571	PDIA6	29	25	20	23	30	28	181	186	126	192	126	138
ENSMUSG00000020572	NAMPT	1	6	2	4	12	5	42	42	35	58	35	31
ENSMUSG00000020573	PIK3CG	3	5	1	3	4	6	6	13	16	23	15	9
ENSMUSG00000020576	NBAS	3	3	4	3	4	6	23	16	15	17	8	13
ENSMUSG00000020577	TSPAN13	24	32	31	38	39	36	188	233	140	262	147	178
ENSMUSG00000020580	ROCK2	25	22	11	37	28	30	94	103	74	135	83	62
ENSMUSG00000020585	LAPTM4A	9	28	18	18	16	17	83	99	77	119	70	75
ENSMUSG00000020590	SNX13	3	1	2	8	5	2	26	16	13	24	15	17
ENSMUSG00000020592	SDC1	0	1	0	0	0	0	1	0	0	0	0	0
ENSMUSG00000020593	LPIN1	2	3	5	2	7	7	9	22	17	21	9	20
ENSMUSG00000020594	PUM2	46	61	51	76	81	62	243	289	168	240	175	184
ENSMUSG00000020598	NRCAM	0	0	0	0	0	1	0	0	0	0	0	0
ENSMUSG00000020599	RGS9	0	0	0	0	0	0	0	1	0	0	0	1
ENSMUSG00000020601	TRIB2	0	1	0	0	0	0	0	1	0	1	1	0
ENSMUSG00000020604	ARSG	0	0	0	1	0	0	0	1	0	0	0	2
ENSMUSG00000020605	HS1BP3	0	0	0	0	0	0	0	0	1	1	2	1
ENSMUSG00000020608	SMC6	31	32	35	48	36	39	153	144	103	177	132	126
ENSMUSG00000020610	AMZ2	2	6	7	12	6	4	31	25	15	25	24	18
ENSMUSG00000020611	GNA13	8	5	16	5	10	13	60	53	45	46	28	38
ENSMUSG00000020612	PRKAR1A	11	16	13	19	21	15	116	100	101	114	84	76
ENSMUSG00000020617	1700012B07F	0	0	0	0	0	0	0	0	1	0	0	0
ENSMUSG00000020621	RDH14	0	3	0	2	1	3	10	10	9	9	4	5
ENSMUSG00000020623	MAP2K6	1	6	1	2	3	5	7	21	11	11	7	15
ENSMUSG00000020628	TRAPPC12	5	1	4	3	1	5	8	14	9	10	8	17
ENSMUSG00000020629	ADI1	3	1	0	4	0	5	19	18	10	21	9	11
ENSMUSG00000020630	RNASEH1	4	4	5	1	2	4	4	11	7	9	9	13
ENSMUSG00000020634	UBXN2A	7	10	2	7	9	10	24	22	23	29	19	22
ENSMUSG00000020640	ITSN2	24	14	18	19	20	22	80	76	61	111	62	63
ENSMUSG00000020642	RNF144A	4	2	1	7	3	3	12	19	13	20	11	13
ENSMUSG00000020644	ID2	3	3	9	6	3	5	30	27	24	19	25	16
ENSMUSG00000020647	NCOA1	4	9	7	6	2	5	21	7	20	22	19	9
ENSMUSG00000020648	DUS4L	2	3	3	5	2	3	22	27	22	32	23	19
ENSMUSG00000020649	RRM2	85	117	105	140	142	134	829	816	675	829	606	754
ENSMUSG00000020650	BCAP29	25	31	28	40	17	23	104	103	85	149	89	96
ENSMUSG00000020652	CENPO	1	2	2	2	3	2	18	13	12	10	21	11
ENSMUSG00000020653	KLF11	0	0	0	0	0	0	0	3	3	2	1	3
ENSMUSG00000020654	ADCY3	2	5	3	5	4	12	29	25	21	29	18	30
ENSMUSG00000020657	DNAJC27	2	2	1	1	3	0	4	5	9	9	7	8
ENSMUSG00000020658	EFR3B	2	3	0	1	2	1	3	5	5	5	3	2
ENSMUSG00000020659	CBLL1	6	5	6	6	10	6	40	35	24	34	29	25
ENSMUSG00000020660	POMC	0	1	1	1	0	1	3	6	7	4	1	3
ENSMUSG00000020661	DNMT3A	23	39	26	37	30	42	119	129	80	152	86	104
ENSMUSG00000020664	DLD	14	12	9	17	12	14	97	109	72	72	72	63
ENSMUSG00000020668	KIF3C	2	3	8	0	2	1	11	10	7	13	6	9
ENSMUSG00000020669	SH3YL1	0	0	0	0	0	0	1	4	1	1	3	2
ENSMUSG00000020671	RAB10	7	11	6	28	6	8	53	40	29	72	32	44
ENSMUSG00000020677	DDX52	25	27	18	20	21	23	120	127	86	104	86	116
ENSMUSG00000020680	TAF15	14	19	19	27	18	23	104	137	105	141	121	109

ENSMUSG00000020687	CDC27	5	9	10	6	12	8	33	45	37	37	38	36
ENSMUSG00000020689	ITGB3	0	0	0	0	0	0	0	0	0	0	0	1
ENSMUSG00000020691	METTL2	1	2	7	3	1	2	12	13	12	16	15	9
ENSMUSG00000020692	NLE1	5	8	9	7	5	8	34	49	19	39	19	33
ENSMUSG00000020694	TLK2	10	21	15	31	29	22	107	110	56	100	64	87
ENSMUSG00000020696	RFFL	1	3	2	6	7	4	23	26	8	22	17	16
ENSMUSG00000020697	LIG3	7	9	20	14	5	7	58	53	45	60	45	31
ENSMUSG00000020698	CCT6B	0	0	0	0	0	1	0	0	0	0	0	0
ENSMUSG00000020700	MAP3K3	2	2	2	4	6	5	12	10	14	17	11	15
ENSMUSG00000020705	DDX42	19	22	20	23	27	27	123	98	81	151	98	99
ENSMUSG00000020706	FTSJ3	12	15	17	9	23	14	89	68	51	82	67	47
ENSMUSG00000020707	RNF135	0	0	0	0	0	1	0	0	0	0	0	0
ENSMUSG00000020708	PSMC5	24	27	33	30	23	26	148	141	117	142	123	128
ENSMUSG00000020709	ADAP2	0	1	0	0	0	0	0	0	0	0	0	0
ENSMUSG00000020712	TCAM1	0	0	0	0	1	0	0	0	0	0	0	0
ENSMUSG00000020715	ERN1	1	2	2	2	5	3	8	6	7	13	6	11
ENSMUSG00000020716	NF1	15	13	15	23	8	17	51	67	34	86	47	72
ENSMUSG00000020717	PECAM1	7	11	7	9	8	8	29	45	32	31	30	36
ENSMUSG00000020718	POLG2	2	0	4	1	5	3	11	12	10	11	13	16
ENSMUSG00000020719	DDX5	41	62	56	61	58	60	518	508	356	652	325	375
ENSMUSG00000020720	PSMD12	19	12	18	20	31	20	140	128	113	154	122	118
ENSMUSG00000020721	HELZ	20	20	16	31	18	23	74	63	48	69	60	61
ENSMUSG00000020728	CEP112	1	2	0	0	0	1	8	8	2	8	9	5
ENSMUSG00000020732	RAB37	3	7	3	5	4	4	25	26	17	24	18	30
ENSMUSG00000020733	SLC9A3R1	12	19	12	29	18	25	119	117	101	138	90	123
ENSMUSG00000020736	NT5C	12	7	9	11	10	7	49	69	52	64	42	53
ENSMUSG00000020737	HN1	9	10	8	14	7	13	82	72	70	72	61	52
ENSMUSG00000020738	SUMO2	1	1	0	0	1	0	2	0	0	0	0	0
ENSMUSG00000020739	NUP85	19	29	27	25	42	27	124	164	125	135	122	157
ENSMUSG00000020740	GGA3	3	4	4	7	3	4	15	17	13	12	9	12
ENSMUSG00000020741	CLUH	16	13	16	10	14	15	72	68	57	71	63	52
ENSMUSG00000020743	MIF4GD	0	1	2	0	2	0	6	7	7	7	9	6
ENSMUSG00000020744	SLC25A19	1	1	0	4	3	1	13	11	12	10	6	7
ENSMUSG00000020745	PAFAH1B1	10	27	17	26	22	15	122	105	73	121	85	86
ENSMUSG00000020747	TMEM94	4	4	3	8	6	8	20	19	22	19	15	16
ENSMUSG00000020752	RECQL5	0	1	0	4	4	1	5	6	8	3	1	4
ENSMUSG00000020755	SAP30BP	2	3	2	6	2	1	23	22	15	29	10	20
ENSMUSG00000020758	ITGB4	0	2	0	0	0	3	1	2	2	5	3	2
ENSMUSG00000020766	GALK1	10	14	20	13	26	13	76	106	86	96	88	76
ENSMUSG00000020770	UNK	5	4	5	7	6	5	17	34	17	17	22	17
ENSMUSG00000020773	TRIM47	0	0	0	0	0	0	0	0	1	0	0	2
ENSMUSG00000020774	ASPA	0	0	0	0	0	0	0	0	0	1	0	0
ENSMUSG00000020775	MRPL38	3	10	6	12	10	8	46	41	42	46	37	44
ENSMUSG00000020776	FBF1	2	1	0	2	2	2	3	3	3	6	2	8
ENSMUSG00000020777	ACOX1	3	2	3	3	1	3	18	15	15	19	10	12
ENSMUSG00000020778	TEN1	9	6	4	10	6	6	37	42	22	38	22	39
ENSMUSG00000020780	SRP68	12	7	10	8	15	8	55	41	45	63	43	40
ENSMUSG00000020781	TSEN54	0	2	0	3	2	0	5	9	7	14	10	10
ENSMUSG00000020782	LLGL2	0	0	0	0	0	0	0	0	1	0	0	0
ENSMUSG00000020783	NCBP3	4	3	8	6	4	7	30	29	16	28	29	27
ENSMUSG00000020785	CAMKK1	0	0	0	0	0	2	0	0	2	1	1	1
ENSMUSG00000020787	P2RX1	4	1	2	6	2	1	15	18	9	18	8	14
ENSMUSG00000020788	ATP2A3	5	6	6	13	9	7	2	0	2	1	1	1
ENSMUSG00000020790	ANKFY1	2	3	8	6	2	2	26	22	15	27	22	18
ENSMUSG00000020792	EXOC7	0	0	2	0	0	4	6	15	13	10	8	11
ENSMUSG00000020794	UBE2G1	8	2	4	5	8	6	43	31	27	37	24	19
ENSMUSG00000020798	SPNS3	0	0	1	0	1	1	1	1	1	2	2	0
ENSMUSG00000020801	MED31	0	1	4	3	6	2	16	14	15	11	16	21

ENSMUSG00000020802	UBE2O	5	6	4	8	6	5	35	48	25	41	23	31
ENSMUSG00000020803	TXNDC17	10	5	12	9	5	15	80	62	76	82	65	59
ENSMUSG00000020807	4933427D14I	0	0	0	0	0	0	1	0	0	0	0	0
ENSMUSG00000020812	1810032O08I	1	2	1	3	0	1	8	21	15	17	17	13
ENSMUSG00000020814	MXRA7	0	0	0	0	0	0	0	0	1	0	0	0
ENSMUSG00000020817	RABEP1	18	21	17	20	28	13	94	98	62	126	75	78
ENSMUSG00000020818	MFSD11	2	3	1	1	0	0	2	1	7	9	7	3
ENSMUSG00000020821	KIF1C	2	4	2	2	6	1	17	5	7	11	6	12
ENSMUSG00000020823	SEC14L1	1	1	1	1	2	1	13	10	6	10	9	7
ENSMUSG00000020826	NOS2	0	1	0	0	0	0	0	0	0	0	0	0
ENSMUSG00000020827	MINK1	2	1	3	3	3	4	16	18	7	15	12	15
ENSMUSG00000020828	PLD2	0	0	0	1	0	0	0	0	0	0	0	0
ENSMUSG00000020829	SLC46A1	0	0	0	0	0	0	0	0	1	2	0	2
ENSMUSG00000020831	0610010K14I	0	0	0	0	0	0	0	1	0	0	0	0
ENSMUSG00000020832	ERAL1	0	1	4	3	2	5	17	11	17	18	13	19
ENSMUSG00000020834	DHRS13	1	2	1	0	2	3	10	9	3	4	5	6
ENSMUSG00000020843	TIMM22	1	7	1	3	3	0	24	14	18	14	15	20
ENSMUSG00000020844	NXN	2	2	3	3	3	1	20	17	15	23	11	9
ENSMUSG00000020846	RFLNB	0	1	0	3	5	3	10	9	2	4	3	8
ENSMUSG00000020847	RPH3AL	0	0	0	3	0	0	0	2	1	1	5	9	3
ENSMUSG00000020849	YWHAE	58	80	58	70	77	68	391	460	284	467	313	346
ENSMUSG00000020850	PRPF8	11	19	27	12	10	19	112	122	99	119	78	103
ENSMUSG00000020859	SPAG9	5	5	5	6	7	8	27	30	26	36	23	36
ENSMUSG00000020863	LUC7L3	63	92	87	128	99	98	528	591	440	631	562	507
ENSMUSG00000020864	ANKRD40	2	9	7	0	2	2	30	21	31	30	14	29
ENSMUSG00000020868	XYLT2	4	3	3	3	5	2	17	13	14	13	15	23
ENSMUSG00000020869	LRRC59	9	13	16	13	15	12	108	89	64	105	81	69
ENSMUSG00000020870	CDC34B	0	0	1	0	0	0	0	0	0	0	0	0
ENSMUSG00000020872	TAC4	0	0	0	0	0	0	0	1	1	0	0	1
ENSMUSG00000020873	SLC35B1	11	10	8	11	19	6	56	45	34	56	31	50
ENSMUSG00000020876	SNX11	1	0	2	3	0	1	6	4	4	4	2	6
ENSMUSG00000020877	SCRN2	1	1	0	1	1	2	6	6	3	8	8	1
ENSMUSG00000020878	LRRC46	0	0	0	0	1	0	0	0	0	0	1	1
ENSMUSG00000020882	CACNB1	0	0	0	0	1	0	0	1	0	0	0	0
ENSMUSG00000020883	FBXL20	6	9	8	8	10	8	21	28	23	20	21	27
ENSMUSG00000020884	ASGR1	0	0	0	0	0	0	0	1	0	0	0	0
ENSMUSG00000020886	DLG4	0	2	0	3	1	4	3	7	5	2	1	4
ENSMUSG00000020888	DVL2	4	5	13	4	1	5	31	33	17	27	21	26
ENSMUSG00000020889	NR1D1	0	0	0	1	0	0	2	1	3	6	0	3
ENSMUSG00000020891	ALOX8	0	0	0	0	1	0	1	0	0	0	0	1
ENSMUSG00000020893	PER1	3	2	4	9	2	1	29	26	16	21	15	19
ENSMUSG00000020894	VAMP2	4	1	0	6	3	1	12	10	11	16	9	14
ENSMUSG00000020895	TMEM107	0	1	0	1	1	1	4	4	3	1	3	5
ENSMUSG00000020897	AURKB	10	13	4	15	18	17	82	90	62	80	47	74
ENSMUSG00000020898	CTC1	3	2	3	3	2	4	19	20	13	17	16	14
ENSMUSG00000020899	PFAS	13	25	19	19	28	15	117	128	95	130	79	114
ENSMUSG00000020900	MYH10	0	0	1	0	0	0	1	0	0	0	0	0
ENSMUSG00000020901	PIK3R5	1	0	1	1	0	0	1	1	2	5	2	3
ENSMUSG00000020903	STX8	2	2	1	1	1	1	12	12	10	12	10	13
ENSMUSG00000020910	ADPRM	1	3	1	3	5	6	11	16	14	14	6	8
ENSMUSG00000020914	TOP2A	59	74	64	117	78	104	353	406	291	402	295	283
ENSMUSG00000020917	ACLY	23	26	13	42	23	21	156	170	137	212	125	152
ENSMUSG00000020918	KAT2A	4	2	5	3	4	9	29	38	28	36	31	41
ENSMUSG00000020919	STAT5B	8	6	8	5	3	5	44	41	30	30	15	33
ENSMUSG00000020921	TMEM101	0	3	1	0	1	2	3	3	1	0	3	2
ENSMUSG00000020922	LSM12	42	58	77	57	58	63	174	187	131	159	129	166
ENSMUSG00000020923	UBTF	30	21	46	33	39	41	192	224	157	223	135	148
ENSMUSG00000020925	CCDC43	5	10	9	4	9	8	17	29	21	42	18	19

ENSMUSG00000020928	HIGD1B	0	0	0	0	0	0	0	0	0	0	1	0
ENSMUSG00000020929	EFTUD2	13	13	6	17	14	6	77	92	58	80	71	68
ENSMUSG00000020930	CCDC103	0	0	0	0	0	0	0	0	0	0	0	1
ENSMUSG00000020932	GFAP	0	0	0	1	0	0	0	0	0	0	0	0
ENSMUSG00000020935	DCAKD	5	1	4	3	5	4	15	18	9	17	5	12
ENSMUSG00000020936	NMT1	3	7	8	3	8	7	33	39	32	32	26	29
ENSMUSG00000020937	PLCD3	0	1	0	0	0	0	1	0	0	3	0	0
ENSMUSG00000020940	1700023F06R	0	0	0	0	0	0	1	0	0	0	0	0
ENSMUSG00000020941	MAP3K14	0	0	0	0	1	3	3	5	0	6	2	1
ENSMUSG00000020946	GOSR2	1	2	3	1	5	0	9	8	8	11	4	9
ENSMUSG00000020948	KLHL28	6	3	6	3	7	4	25	13	9	20	5	10
ENSMUSG00000020949	FKBP3	32	36	41	33	38	43	299	278	223	327	193	205
ENSMUSG00000020952	SCFD1	7	12	10	14	17	17	59	90	53	93	54	55
ENSMUSG00000020954	STRN3	13	18	23	27	17	19	75	51	64	72	49	56
ENSMUSG00000020955	AP4S1	3	1	2	2	5	2	16	14	9	21	10	11
ENSMUSG00000020956	DTD2	2	8	5	11	6	2	25	34	26	23	13	33
ENSMUSG00000020961	STON2	0	0	0	1	0	0	1	0	0	0	0	0
ENSMUSG00000020962	GTF2A1	2	8	8	16	6	3	35	37	14	20	24	25
ENSMUSG00000020964	SEL1L	13	11	11	14	13	12	34	40	26	42	31	46
ENSMUSG00000020973	DNAAF2	1	2	1	0	2	1	8	8	7	13	8	6
ENSMUSG00000020974	POLE2	2	3	3	3	2	4	18	15	13	11	14	8
ENSMUSG00000020978	KLHDC2	1	7	7	3	3	2	26	20	28	29	24	27
ENSMUSG00000020982	NEMF	30	40	27	40	23	23	148	146	112	137	133	135
ENSMUSG00000020986	SEC23A	2	10	3	10	6	1	38	53	26	40	23	36
ENSMUSG00000020988	L2HGDH	1	5	2	8	6	6	45	35	31	52	27	23
ENSMUSG00000020993	TRAPPC6B	4	3	5	11	5	4	38	41	22	54	24	22
ENSMUSG00000020994	PNN	31	46	34	51	58	47	290	257	227	330	265	236
ENSMUSG00000021000	MIA2	34	38	41	57	45	37	231	207	127	246	174	145
ENSMUSG00000021003	GALC	4	3	3	3	5	4	9	12	10	18	10	13
ENSMUSG00000021007	SPATA7	1	0	4	3	2	5	11	13	7	9	5	11
ENSMUSG00000021009	PTPN21	2	1	3	1	1	4	6	11	7	11	8	5
ENSMUSG00000021010	NPAS3	0	1	0	0	0	0	0	0	0	0	0	0
ENSMUSG00000021012	ZC3H14	20	11	12	15	16	11	61	73	60	79	49	65
ENSMUSG00000021018	POLR2H	8	7	7	8	7	10	47	53	40	44	30	41
ENSMUSG00000021022	PPP2R3C	2	7	3	5	3	0	12	18	13	16	8	14
ENSMUSG00000021023	1110008L16R	1	5	0	1	3	1	12	15	13	9	12	7
ENSMUSG00000021024	PSMA6	15	29	28	33	32	30	170	177	131	181	149	137
ENSMUSG00000021025	NFKBIA	11	13	17	16	19	19	66	53	49	63	52	56
ENSMUSG00000021027	RALGAPA1	6	10	19	17	8	13	58	43	50	59	39	63
ENSMUSG00000021028	MBIP	4	8	6	11	8	3	34	38	28	40	24	28
ENSMUSG00000021032	NGB	0	0	0	0	0	0	0	0	0	1	0	0
ENSMUSG00000021033	GSTZ1	2	1	4	2	2	1	9	11	4	9	6	7
ENSMUSG00000021036	SPTLC2	7	6	7	6	9	7	35	22	28	38	20	30
ENSMUSG00000021037	AHSA1	5	2	1	4	5	3	29	45	21	37	21	26
ENSMUSG00000021038	VIPAS39	2	0	1	0	1	3	3	9	5	5	0	7
ENSMUSG00000021039	SNW1	16	21	19	22	23	23	128	132	106	115	96	98
ENSMUSG00000021040	SLIRP	22	18	28	24	26	23	144	130	118	140	107	156
ENSMUSG00000021044	ADCK1	2	0	0	3	0	2	3	6	3	4	7	8
ENSMUSG00000021048	MTHFD1	11	15	13	13	18	18	97	88	67	81	70	61
ENSMUSG00000021051	PPP2R5E	1	4	4	6	7	2	26	24	19	20	21	21
ENSMUSG00000021054	SGPP1	3	4	2	1	2	3	0	0	0	0	0	0
ENSMUSG00000021057	AKAP5	0	1	0	1	0	0	1	2	0	3	0	2
ENSMUSG00000021065	FUT8	3	0	4	3	6	2	6	12	9	10	12	6
ENSMUSG00000021068	NIN	9	7	3	4	2	5	38	33	31	38	21	33
ENSMUSG00000021071	TRIM9	0	0	0	0	0	0	0	0	1	0	1	0
ENSMUSG00000021072	TMX1	16	16	11	10	9	13	53	55	36	57	47	50
ENSMUSG00000021076	ACTR10	3	9	11	14	7	11	44	43	35	46	38	41
ENSMUSG00000021078	TOMM20L	1	1	1	1	0	0	3	5	1	2	6	3

ENSMUSG00000021079	TIMM9	8	11	13	21	9	10	70	66	37	77	52	44
ENSMUSG00000021086	CCDC175	0	0	1	1	1	0	7	3	7	6	4	3
ENSMUSG00000021094	DHRS7	4	2	6	3	9	6	27	28	26	24	15	30
ENSMUSG00000021096	PPM1A	10	8	15	13	7	11	45	48	35	37	26	35
ENSMUSG00000021102	GLRX5	7	3	6	9	1	7	51	49	30	36	44	41
ENSMUSG00000021103	MNAT1	5	6	6	12	7	11	37	39	36	36	34	30
ENSMUSG00000021108	PRKCH	7	7	10	5	8	5	25	31	20	32	25	21
ENSMUSG00000021109	HIF1A	6	6	7	12	8	6	37	39	36	67	39	32
ENSMUSG00000021111	PAPOLA	11	16	20	25	16	12	110	125	102	133	103	99
ENSMUSG00000021112	MPP5	3	8	2	6	4	10	30	32	24	35	20	24
ENSMUSG00000021113	SNAPC1	2	3	4	2	4	3	25	29	10	32	14	17
ENSMUSG00000021114	ATP6V1D	1	4	2	3	8	7	31	23	19	31	35	24
ENSMUSG00000021115	VRK1	6	11	14	10	16	16	72	84	52	62	45	69
ENSMUSG00000021116	EIF2S1	9	14	17	20	18	16	83	88	89	91	73	95
ENSMUSG00000021120	PIGH	1	2	0	0	2	1	11	6	4	18	8	4
ENSMUSG00000021123	RDH12	0	0	0	1	1	0	0	0	0	0	0	0
ENSMUSG00000021124	VTI1B	5	6	2	10	7	2	32	25	19	25	15	20
ENSMUSG00000021125	ARG2	1	1	0	0	0	0	1	1	2	1	2	2
ENSMUSG00000021127	ZFP36L1	12	25	17	20	15	23	116	109	61	126	86	106
ENSMUSG00000021130	GALNT16	1	0	0	0	0	0	0	0	1	1	1	0
ENSMUSG00000021131	ERH	0	1	2	3	2	1	7	9	4	17	14	15
ENSMUSG00000021133	SUSD6	6	4	3	8	7	5	26	33	26	38	20	20
ENSMUSG00000021134	SRSF5	21	28	32	37	42	37	206	222	162	241	140	170
ENSMUSG00000021135	SLC10A1	0	0	0	2	0	0	0	0	0	0	1	0
ENSMUSG00000021139	GM20498	1	0	1	0	1	0	2	2	1	3	1	0
ENSMUSG00000021140	PCNX	1	2	3	2	3	4	17	15	12	16	7	15
ENSMUSG00000021143	PACS2	1	2	5	2	7	2	13	15	15	11	14	10
ENSMUSG00000021144	MTA1	19	33	31	32	27	20	108	114	88	136	92	126
ENSMUSG00000021147	WDR37	4	4	5	5	4	2	28	18	18	25	19	23
ENSMUSG00000021149	GTPBP4	21	16	24	32	32	18	123	106	68	122	89	88
ENSMUSG00000021156	ZMYND11	10	15	27	27	29	24	108	109	88	99	83	100
ENSMUSG00000021171	ESYT2	39	27	27	31	45	31	110	106	94	135	73	90
ENSMUSG00000021175	CDCA7L	6	12	6	8	9	7	53	46	24	37	43	45
ENSMUSG00000021176	EFCAB11	2	2	1	1	0	0	2	1	2	5	1	0
ENSMUSG00000021177	TDP1	4	3	4	2	4	6	15	27	16	28	23	22
ENSMUSG00000021178	PSMC1	16	16	11	20	12	12	112	99	52	96	66	71
ENSMUSG00000021179	NRDE2	1	5	1	7	1	1	9	11	8	12	14	11
ENSMUSG00000021180	RPS6KA5	2	1	5	3	1	2	17	16	12	13	11	14
ENSMUSG00000021182	CCDC88C	5	8	7	7	10	9	56	42	41	51	29	45
ENSMUSG00000021185	DGLUCY	0	0	0	0	0	0	2	0	0	5	0	2
ENSMUSG00000021187	TC2N	0	1	0	0	0	0	0	0	0	0	0	0
ENSMUSG00000021188	TRIP11	14	29	14	25	11	18	70	71	50	98	44	52
ENSMUSG00000021189	ATXN3	7	4	6	7	5	4	27	17	18	20	18	13
ENSMUSG00000021190	LGMN	0	0	0	1	0	0	7	6	8	10	3	3
ENSMUSG00000021192	GOLGA5	1	1	2	2	1	3	7	12	10	9	9	6
ENSMUSG00000021193	PITRM1	2	8	7	11	11	13	53	51	47	48	33	42
ENSMUSG00000021196	PFKP	15	16	15	18	29	18	118	95	71	120	96	80
ENSMUSG00000021198	UNC79	0	2	1	1	0	1	8	3	1	7	2	4
ENSMUSG00000021200	ASB2	4	1	3	0	0	1	6	6	5	7	7	4
ENSMUSG00000021203	OTUB2	1	2	1	1	2	3	8	7	3	5	5	3
ENSMUSG00000021211	AKR1C12	0	0	0	0	0	0	0	1	0	0	0	0
ENSMUSG00000021213	AKR1C13	0	0	0	0	0	0	1	0	0	0	0	0
ENSMUSG00000021214	AKR1C18	0	0	1	0	0	0	1	0	0	0	0	0
ENSMUSG00000021215	NET1	11	13	13	10	19	15	80	70	40	72	53	47
ENSMUSG00000021216	TUBAL3	0	0	0	0	0	0	0	0	0	0	0	1
ENSMUSG00000021218	GDI2	15	26	27	23	34	28	171	139	117	159	122	117
ENSMUSG00000021219	RGS6	0	0	0	0	0	0	0	0	0	0	1	0
ENSMUSG00000021221	DPF3	0	0	0	0	0	0	1	1	0	1	2	1

ENSMUSG00000021222	DCAF4	7	3	5	7	3	6	17	33	13	24	16	19
ENSMUSG00000021224	NUMB	0	3	2	2	5	3	13	15	8	19	6	12
ENSMUSG00000021226	ACOT2	16	25	18	18	19	22	69	76	48	70	47	53
ENSMUSG00000021234	FAM161B	0	0	0	1	0	0	0	3	0	1	3	2
ENSMUSG00000021235	COQ6	1	4	2	2	1	4	15	13	7	16	4	12
ENSMUSG00000021236	ENTPD5	0	5	6	6	3	6	19	22	7	20	17	24
ENSMUSG00000021238	ALDH6A1	0	4	4	0	3	3	11	9	12	8	6	13
ENSMUSG00000021240	ABCD4	1	1	1	3	1	4	13	9	7	4	5	7
ENSMUSG00000021241	ISCA2	6	2	7	4	4	8	30	26	25	31	26	20
ENSMUSG00000021242	NPC2	6	9	9	15	8	9	52	65	37	62	38	42
ENSMUSG00000021243	FCF1	10	18	12	16	15	9	97	108	67	100	93	85
ENSMUSG00000021244	YLPM1	10	17	16	26	15	12	72	74	60	80	55	75
ENSMUSG00000021245	MLH3	0	1	0	0	2	1	1	4	1	7	3	2
ENSMUSG00000021248	TMED10	13	12	9	24	10	7	36	48	39	51	42	40
ENSMUSG00000021250	FOS	1	1	0	0	0	1	3	0	1	0	0	0
ENSMUSG00000021252	ERG28	9	10	12	7	9	9	72	64	52	69	59	66
ENSMUSG00000021254	GPATCH2L	0	2	2	0	2	0	4	9	3	8	2	13
ENSMUSG00000021257	ANGEL1	0	0	0	0	0	0	2	1	0	0	1	0
ENSMUSG00000021258	CCNK	3	4	8	5	9	7	24	32	21	26	22	26
ENSMUSG00000021259	CYP46A1	0	0	0	0	0	0	0	0	0	0	0	1
ENSMUSG00000021262	EVL	13	16	24	20	14	15	89	106	80	85	68	89
ENSMUSG00000021263	DEGS2	6	18	3	4	6	7	32	24	21	27	13	34
ENSMUSG00000021264	YY1	17	15	19	21	24	26	127	162	96	168	111	107
ENSMUSG00000021265	SLC25A29	1	0	0	0	1	0	3	1	1	2	2	2
ENSMUSG00000021266	WARS	2	7	3	4	7	9	26	23	17	33	28	27
ENSMUSG00000021270	HSP90AA1	63	74	63	86	76	97	340	319	268	407	264	273
ENSMUSG00000021271	ZFP839	1	0	0	2	1	2	11	6	6	5	3	2
ENSMUSG00000021273	FDFT1	2	3	8	3	1	3	27	31	22	32	23	33
ENSMUSG00000021275	TECPR2	1	0	0	2	2	3	8	7	6	7	9	9
ENSMUSG00000021276	CINP	3	6	2	9	1	5	32	38	25	37	39	27
ENSMUSG00000021277	TRAF3	8	8	5	5	6	6	19	20	12	21	10	26
ENSMUSG00000021278	AMN	0	1	0	0	0	0	0	0	1	0	0	0
ENSMUSG00000021282	EIF5	37	52	55	56	55	47	300	322	223	338	217	263
ENSMUSG00000021285	PPP1R13B	0	2	1	3	3	2	10	12	3	10	10	8
ENSMUSG00000021286	ZFYVE21	1	2	1	0	0	1	9	12	5	9	6	5
ENSMUSG00000021287	XRCC3	2	1	2	2	2	2	13	14	12	7	6	8
ENSMUSG00000021288	KLC1	8	11	3	8	5	6	25	30	22	36	31	23
ENSMUSG00000021290	2010107E04F	24	30	30	29	26	34	229	224	183	205	194	195
ENSMUSG00000021298	GPR132	1	5	1	4	1	3	7	9	5	10	5	7
ENSMUSG00000021301	HECW1	0	0	0	1	0	0	0	0	0	0	0	0
ENSMUSG00000021302	GGPS1	3	1	4	9	2	5	17	23	21	16	12	25
ENSMUSG00000021311	MTR	1	4	4	2	4	4	18	33	12	23	14	26
ENSMUSG00000021313	RYR2	0	0	0	1	0	0	0	0	0	0	0	0
ENSMUSG00000021314	AMPH	1	0	1	1	1	2	0	3	3	5	3	3
ENSMUSG00000021322	AOAH	3	1	0	0	0	2	2	6	3	2	4	2
ENSMUSG00000021326	TRIM27	13	13	10	11	9	8	64	64	55	70	48	50
ENSMUSG00000021327	ZKSCAN3	10	13	8	13	16	12	68	87	74	76	63	74
ENSMUSG00000021338	CARMIL1	1	8	5	2	4	2	8	15	5	14	8	6
ENSMUSG00000021339	MRS2	11	13	7	13	12	9	35	30	30	33	35	24
ENSMUSG00000021340	GPLD1	0	1	0	0	1	0	1	0	1	0	1	1
ENSMUSG00000021356	IRF4	1	0	0	0	0	0	0	0	0	0	1	0
ENSMUSG00000021357	EXOC2	1	1	5	5	3	5	20	15	16	28	12	19
ENSMUSG00000021359	TFAP2A	0	0	1	1	0	0	2	1	0	0	0	0
ENSMUSG00000021360	GCNT2	1	1	4	2	3	0	13	15	6	14	14	9
ENSMUSG00000021361	TMEM14C	12	10	6	10	8	10	56	52	42	39	34	39
ENSMUSG00000021365	NEDD9	8	6	3	2	6	8	19	25	21	27	14	19
ENSMUSG00000021366	HIVEP1	5	5	8	5	3	3	13	13	15	22	15	16
ENSMUSG00000021368	TBC1D7	0	0	0	1	2	2	4	6	2	5	1	7

ENSMUSG00000021371	MCUR1	2	1	2	2	3	4	12	11	7	7	11	10
ENSMUSG00000021373	CAP2	0	0	0	1	0	0	3	5	2	1	2	4
ENSMUSG00000021374	NUP153	1	5	4	7	8	5	25	42	34	34	24	27
ENSMUSG00000021375	KIF13A	0	2	0	1	2	1	14	17	7	8	4	8
ENSMUSG00000021376	TPMT	1	1	1	1	0	1	8	3	2	4	3	8
ENSMUSG00000021377	DEK	78	142	113	175	108	118	463	438	335	506	363	371
ENSMUSG00000021384	SUSD3	1	0	0	1	0	0	0	1	0	0	0	1
ENSMUSG00000021385	IPPK	5	7	4	5	4	5	47	40	24	33	20	28
ENSMUSG00000021388	ASPN	0	0	1	1	0	1	1	1	0	0	0	0
ENSMUSG00000021390	OGN	0	0	1	0	0	0	0	0	0	0	0	0
ENSMUSG00000021391	CENPP	4	6	2	9	3	3	28	27	21	33	16	17
ENSMUSG00000021392	NOL8	14	20	17	29	19	26	99	98	81	116	82	81
ENSMUSG00000021395	SPIN1	4	13	9	8	9	11	53	38	45	52	42	35
ENSMUSG00000021396	NXNL2	0	0	0	1	0	0	0	0	1	0	0	0
ENSMUSG00000021400	WRNIP1	2	6	4	9	8	9	53	58	37	47	21	32
ENSMUSG00000021408	RIPK1	7	6	9	8	2	4	15	24	23	28	17	33
ENSMUSG00000021413	PRPF4B	38	67	42	60	62	67	343	315	210	426	263	271
ENSMUSG00000021414	FAM217A	1	0	0	0	0	0	0	0	0	1	0	0
ENSMUSG00000021417	ECI2	4	7	7	8	2	10	26	31	27	35	25	21
ENSMUSG00000021418	RPP40	3	5	4	5	4	5	20	20	19	25	18	19
ENSMUSG00000021420	FARS2	2	1	1	1	1	0	9	8	5	7	5	8
ENSMUSG00000021427	SSR1	55	71	62	58	54	70	242	253	162	210	169	182
ENSMUSG00000021428	RIOK1	16	15	15	19	20	14	81	79	50	87	55	77
ENSMUSG00000021431	SNRNP48	34	37	52	51	56	37	170	170	155	250	142	151
ENSMUSG00000021432	SLC35B3	1	4	4	2	2	1	19	30	13	28	14	17
ENSMUSG00000021448	SHC3	0	0	0	0	0	0	0	0	0	1	0	0
ENSMUSG00000021451	SEMA4D	6	10	8	12	9	4	30	47	37	63	36	37
ENSMUSG00000021453	GADD45G	8	0	3	2	10	4	24	19	14	23	17	26
ENSMUSG00000021458	2010111I01R	0	2	3	1	0	1	5	7	2	5	4	3
ENSMUSG00000021460	AUH	16	9	15	3	12	11	49	42	35	52	38	41
ENSMUSG00000021461	FANCC	2	0	1	2	2	1	6	11	8	10	6	7
ENSMUSG00000021466	PTCH1	7	10	10	22	15	11	63	63	54	55	54	58
ENSMUSG00000021468	SPTLC1	1	2	2	0	4	1	6	5	2	5	3	3
ENSMUSG00000021470	ERCC6L2	3	5	3	7	13	10	25	35	14	32	19	20
ENSMUSG00000021474	SFXN1	30	44	43	40	39	41	198	164	136	191	132	168
ENSMUSG00000021476	HABP4	1	1	2	0	4	0	8	7	8	13	4	6
ENSMUSG00000021481	ZFP346	7	6	4	6	2	4	21	23	14	17	14	21
ENSMUSG00000021482	AAED1	5	10	5	5	15	7	21	40	25	27	22	22
ENSMUSG00000021484	LMAN2	22	32	27	29	35	37	145	147	127	170	132	122
ENSMUSG00000021485	MXD3	2	4	5	7	8	7	20	25	15	17	13	20
ENSMUSG00000021486	PRELID1	14	10	18	25	20	16	95	102	64	97	67	77
ENSMUSG00000021488	NSD1	19	32	15	35	27	21	74	61	28	89	47	63
ENSMUSG00000021493	PDLIM7	2	4	4	2	5	5	15	12	8	19	12	12
ENSMUSG00000021494	DDX41	6	9	8	9	10	4	43	55	31	68	26	32
ENSMUSG00000021495	FAM193B	13	21	21	14	13	21	46	64	46	65	50	48
ENSMUSG00000021496	PCBD2	1	1	0	1	5	4	22	20	13	17	8	17
ENSMUSG00000021497	TXNDC15	6	4	9	5	7	5	32	24	23	24	14	26
ENSMUSG00000021499	CATSPER3	0	0	0	0	0	0	1	0	0	0	0	0
ENSMUSG00000021500	DDX46	41	59	30	44	40	38	225	206	178	200	170	182
ENSMUSG00000021501	CAML	3	2	0	11	4	1	16	17	15	15	11	14
ENSMUSG00000021504	B4GALT7	2	10	3	0	3	5	27	32	27	22	24	23
ENSMUSG00000021510	ZFP729A	3	0	0	6	1	1	8	8	1	11	9	10
ENSMUSG00000021514	ZFP369	3	3	3	4	3	2	20	16	14	15	12	17
ENSMUSG00000021518	PTDSS1	4	3	3	5	6	0	6	13	12	17	11	19
ENSMUSG00000021519	MTERF3	2	1	8	1	4	1	7	18	15	18	24	11
ENSMUSG00000021520	UQCRB	22	24	21	27	36	16	215	221	148	189	176	184
ENSMUSG00000021532	FASTKD3	1	2	2	1	4	3	24	21	17	26	25	18
ENSMUSG00000021537	CETN3	21	19	28	28	21	27	186	167	120	174	131	122

ENSMUSG00000021540	SMAD5	3	5	5	6	6	8	21	19	15	17	14	19
ENSMUSG00000021546	HNRNPK	114	167	130	177	194	141	1074	1195	846	1044	817	946
ENSMUSG00000021548	CCNH	6	7	2	15	9	5	51	58	43	56	34	30
ENSMUSG00000021549	RASA1	5	9	12	15	16	16	61	49	51	64	32	44
ENSMUSG00000021550	2210016F16F	4	2	3	7	3	5	20	32	14	23	22	24
ENSMUSG00000021555	NAA35	9	13	9	17	14	14	38	63	40	70	56	55
ENSMUSG00000021556	GOLM1	14	12	12	13	10	8	55	56	54	58	35	44
ENSMUSG00000021557	AGTPBP1	4	12	7	9	9	7	49	37	20	41	39	26
ENSMUSG00000021565	SLC6A19	9	4	17	14	14	13	69	112	65	98	59	71
ENSMUSG00000021567	NKD2	0	0	1	0	0	0	0	0	0	0	0	0
ENSMUSG00000021569	TRIP13	8	7	5	2	5	7	21	27	28	27	31	25
ENSMUSG00000021572	CEP72	0	2	1	0	0	2	11	5	10	12	6	5
ENSMUSG00000021573	TPPP	0	0	0	0	0	0	0	1	0	0	2	0
ENSMUSG00000021575	AHRR	0	0	0	0	0	0	0	1	0	0	0	1
ENSMUSG00000021576	PDCD6	9	13	16	10	15	13	62	86	53	91	66	61
ENSMUSG00000021577	SDHA	6	15	8	14	13	18	83	70	59	70	49	71
ENSMUSG00000021578	CCDC127	10	18	10	14	11	15	64	80	40	52	52	70
ENSMUSG00000021579	LRRC14B	0	1	1	1	0	1	4	3	3	1	4	2
ENSMUSG00000021583	ERAP1	2	8	8	14	3	13	64	50	53	65	44	50
ENSMUSG00000021585	CAST	17	17	14	19	17	29	94	83	75	117	71	64
ENSMUSG00000021589	RHOBTB3	0	1	1	0	0	0	3	4	5	3	7	3
ENSMUSG00000021591	GLRX	1	0	0	0	1	0	2	6	3	6	6	4
ENSMUSG00000021592	ARSK	1	8	1	5	1	1	9	9	7	11	5	3
ENSMUSG00000021594	SRD5A1	2	2	0	3	0	2	3	3	2	4	3	5
ENSMUSG00000021595	NSUN2	15	16	20	23	18	16	130	109	105	111	94	98
ENSMUSG00000021596	MCTP1	6	5	11	3	2	7	18	32	23	38	18	29
ENSMUSG00000021597	SLF1	7	13	8	12	10	13	34	35	23	45	30	29
ENSMUSG00000021598	MED10	3	5	2	5	5	7	39	37	32	42	29	26
ENSMUSG00000021606	NDUFS6	22	20	19	21	23	21	158	190	134	162	146	141
ENSMUSG00000021607	MRPL36	9	3	6	4	4	6	39	45	27	44	36	34
ENSMUSG00000021608	LPCAT1	5	8	10	10	6	6	38	58	33	41	40	30
ENSMUSG00000021610	CLPTM1L	11	9	13	13	13	7	48	53	42	63	50	48
ENSMUSG00000021611	TERT	2	4	1	4	2	1	14	10	3	11	6	11
ENSMUSG00000021614	VCAN	0	0	0	0	1	0	1	2	1	0	1	5
ENSMUSG00000021615	XRCC4	1	0	1	1	1	1	6	5	3	8	3	6
ENSMUSG00000021619	ATG10	2	1	1	2	0	1	3	4	5	2	1	1
ENSMUSG00000021621	ZCCHC9	8	1	6	7	4	5	27	25	25	42	24	29
ENSMUSG00000021629	SLC30A5	3	7	6	7	10	5	41	53	43	59	35	40
ENSMUSG00000021635	RAD17	1	7	4	6	5	6	29	41	24	27	37	30
ENSMUSG00000021636	MARVELD2	2	2	1	2	1	0	9	6	8	7	9	10
ENSMUSG00000021638	OCLN	0	0	0	0	2	0	0	0	0	0	0	1
ENSMUSG00000021639	GTF2H2	5	3	7	11	5	11	32	39	34	46	24	25
ENSMUSG00000021643	SERF1	8	7	5	12	12	9	100	63	47	76	54	70
ENSMUSG00000021645	SMN1	11	8	8	11	6	7	53	73	37	61	49	42
ENSMUSG00000021646	MCCC2	3	3	3	1	4	3	24	25	15	20	15	14
ENSMUSG00000021647	CARTPT	1	0	0	0	0	0	0	0	2	0	1	0
ENSMUSG00000021650	PTCD2	0	2	5	3	3	5	12	18	15	21	12	11
ENSMUSG00000021660	BTF3	1	1	1	1	1	1	29	30	18	24	17	29
ENSMUSG00000021661	ANKRA2	1	3	1	1	5	2	18	14	9	14	10	11
ENSMUSG00000021665	HEXB	1	3	1	5	7	3	13	13	10	14	12	8
ENSMUSG00000021666	GFM2	5	4	2	3	6	3	17	26	14	17	13	17
ENSMUSG00000021668	POLK	3	3	0	2	3	4	22	15	23	23	15	14
ENSMUSG00000021669	COL4A3BP	3	5	8	6	10	5	25	36	22	32	19	25
ENSMUSG00000021670	HMGCR	43	55	53	61	59	48	326	348	270	367	243	255
ENSMUSG00000021671	POC5	0	2	3	2	2	1	17	15	15	19	18	16
ENSMUSG00000021675	F2RL2	0	0	0	0	0	0	0	0	1	0	0	0
ENSMUSG00000021676	IQGAP2	14	17	11	22	21	18	85	98	42	90	66	58
ENSMUSG00000021680	CRHBP	0	0	0	0	1	0	2	3	1	1	0	0

ENSMUSG00000021681	AGGF1	24	15	25	29	20	18	74	84	56	94	61	76
ENSMUSG00000021684	PDE8B	0	0	0	1	0	0	1	2	0	1	0	0
ENSMUSG00000021686	AP3B1	6	10	10	15	13	11	60	56	42	73	47	48
ENSMUSG00000021687	SCAMP1	1	3	7	8	7	6	28	25	25	33	18	22
ENSMUSG00000021690	JMY	7	5	6	14	11	13	35	39	31	34	30	26
ENSMUSG00000021692	DIMT1	2	5	3	1	3	2	18	20	20	21	12	11
ENSMUSG00000021693	KIF2A	17	21	24	24	16	18	105	113	72	92	92	78
ENSMUSG00000021694	ERCC8	0	2	0	2	3	1	8	9	5	2	5	11
ENSMUSG00000021696	ELOVL7	0	0	0	0	1	0	0	0	1	1	0	1
ENSMUSG00000021697	DEPDC1B	1	0	0	0	0	0	2	3	0	3	1	1
ENSMUSG00000021699	PDE4D	1	2	2	6	0	5	8	9	7	11	5	5
ENSMUSG00000021702	THBS4	0	0	0	0	0	0	0	1	0	0	0	0
ENSMUSG00000021703	SERINC5	0	0	0	0	0	0	1	0	0	0	0	0
ENSMUSG00000021704	MTX3	3	0	0	2	3	1	5	9	8	6	6	7
ENSMUSG00000021706	ZFYVE16	0	0	2	0	0	4	0	10	5	16	10	3
ENSMUSG00000021707	DHFR	24	25	29	33	27	30	147	183	123	162	112	135
ENSMUSG00000021709	ERBIN	14	15	10	10	14	10	82	68	38	83	53	49
ENSMUSG00000021710	NLN	2	4	2	3	4	6	23	31	13	25	21	20
ENSMUSG00000021711	TRAPPC13	8	5	6	11	8	5	70	67	41	76	52	45
ENSMUSG00000021712	TRIM23	3	5	8	7	3	4	33	33	22	27	25	22
ENSMUSG00000021713	PPWD1	43	37	41	38	31	42	175	190	149	204	150	198
ENSMUSG00000021714	CENPK	16	13	14	22	17	23	88	93	64	81	60	54
ENSMUSG00000021715	CWC27	3	8	6	5	2	5	38	36	30	35	19	30
ENSMUSG00000021716	SREK1IP1	18	33	21	34	26	31	92	66	50	97	58	61
ENSMUSG00000021718	4933425L06R	0	0	0	0	0	1	2	1	0	2	1	1
ENSMUSG00000021725	PARP8	1	4	5	4	7	6	15	24	12	12	10	6
ENSMUSG00000021728	EMB	16	15	21	32	32	20	92	128	88	123	89	72
ENSMUSG00000021730	HCN1	0	0	0	0	0	0	0	1	1	0	0	0
ENSMUSG00000021731	MRPS30	4	7	4	9	5	7	35	41	33	42	31	27
ENSMUSG00000021733	SLC4A7	6	7	5	12	12	8	38	43	34	37	27	13
ENSMUSG00000021737	PSMD6	6	19	15	12	19	16	98	99	79	103	65	80
ENSMUSG00000021738	ATXN7	4	4	2	11	2	0	17	17	27	22	11	13
ENSMUSG00000021745	PTPRG	0	0	0	0	0	0	0	0	1	0	0	0
ENSMUSG00000021748	PDHB	18	10	17	18	17	17	101	97	78	102	71	83
ENSMUSG00000021752	KCTD6	1	2	0	1	1	0	7	7	8	7	4	5
ENSMUSG00000021754	MAP3K1	29	17	25	36	30	30	85	84	78	107	80	87
ENSMUSG00000021756	IL6ST	2	6	3	3	7	5	19	22	13	28	11	13
ENSMUSG00000021759	PLPP1	5	14	9	8	9	6	34	41	28	32	28	31
ENSMUSG00000021764	NDUFS4	7	19	12	17	17	17	117	107	74	107	74	79
ENSMUSG00000021767	KAT6B	32	40	41	52	45	50	245	243	157	277	146	212
ENSMUSG00000021770	SAMD8	20	20	24	26	24	14	63	65	46	73	65	61
ENSMUSG00000021771	VDAC2	27	34	21	33	36	29	236	259	187	280	206	185
ENSMUSG00000021772	NKIRAS1	1	7	4	6	6	3	30	27	23	23	31	23
ENSMUSG00000021773	COMTD1	8	1	4	7	7	3	40	40	29	44	45	41
ENSMUSG00000021774	UBE2E1	15	10	18	14	10	4	112	104	70	108	79	85
ENSMUSG00000021775	NR1D2	1	1	1	3	2	1	5	15	3	5	8	11
ENSMUSG00000021779	THRB	0	1	0	0	0	0	1	0	1	2	0	1
ENSMUSG00000021785	NGLY1	4	4	3	3	5	1	8	13	10	15	9	13
ENSMUSG00000021786	OXSM	1	4	1	3	1	2	11	5	4	9	7	10
ENSMUSG00000021792	FAM213A	1	0	0	0	0	0	0	0	0	0	0	0
ENSMUSG00000021794	GLUD1	8	12	11	16	15	7	74	80	59	73	55	65
ENSMUSG00000021806	NID2	0	0	0	0	0	0	2	1	1	1	0	1
ENSMUSG00000021807	RTRAF	15	21	12	24	11	16	104	111	64	92	89	89
ENSMUSG00000021809	NUDT13	2	1	2	5	2	1	12	18	11	11	8	20
ENSMUSG00000021810	ECD	4	9	7	10	8	6	61	48	46	71	35	45
ENSMUSG00000021811	DNAJC9	12	22	9	22	14	16	119	123	91	118	101	102
ENSMUSG00000021814	ANXA7	18	18	20	15	24	20	75	79	77	94	73	87
ENSMUSG00000021815	MSS51	0	1	1	3	1	0	4	7	7	8	1	11

ENSMUSG00000021816	PPP3CB	13	12	9	15	16	15	55	73	55	76	47	52
ENSMUSG00000021819	ZSWIM8	5	5	4	7	3	4	22	27	13	31	27	32
ENSMUSG00000021820	CAMK2G	26	33	29	37	17	23	113	138	89	136	77	84
ENSMUSG00000021823	VCL	1	1	2	2	5	0	10	16	16	11	9	14
ENSMUSG00000021824	AP3M1	5	1	5	8	5	4	23	42	22	28	25	17
ENSMUSG00000021830	TXNDC16	4	5	4	2	8	4	35	25	22	28	22	20
ENSMUSG00000021831	ERO1L	13	14	18	21	19	12	73	78	44	90	56	67
ENSMUSG00000021832	PSMC6	35	35	32	52	44	44	302	333	232	330	231	206
ENSMUSG00000021840	MAPK1IP1L	9	12	14	16	8	13	56	62	52	72	48	46
ENSMUSG00000021843	KTN1	41	65	59	60	54	52	197	199	169	264	153	146
ENSMUSG00000021846	PELI2	0	0	0	0	0	0	0	1	0	0	0	0
ENSMUSG00000021852	SLC35F4	0	1	0	0	0	0	0	0	0	0	0	0
ENSMUSG00000021868	PPIF	7	8	11	9	10	5	42	50	39	36	29	38
ENSMUSG00000021870	SLMAP	8	13	16	20	12	16	65	83	53	80	37	44
ENSMUSG00000021876	RNASE4	2	2	1	0	0	0	1	0	0	1	0	1
ENSMUSG00000021877	ARF4	12	20	11	33	21	17	128	127	96	133	85	113
ENSMUSG00000021879	DNAH12	0	0	0	1	0	0	0	1	0	0	0	0
ENSMUSG00000021884	HACL1	0	1	1	0	0	0	1	1	2	1	1	1
ENSMUSG00000021886	GPR65	17	20	16	21	22	18	55	69	44	62	48	54
ENSMUSG00000021890	EAF1	2	4	2	3	6	5	35	34	28	46	21	26
ENSMUSG00000021891	METTL6	7	4	11	5	11	5	37	51	35	46	37	44
ENSMUSG00000021892	SH3BP5	0	0	0	0	0	0	4	3	3	3	1	4
ENSMUSG00000021893	CAPN7	5	8	9	9	8	11	39	45	29	42	32	28
ENSMUSG00000021895	ARHGEF3	4	3	6	8	5	5	27	27	28	30	15	14
ENSMUSG00000021900	BTD	1	0	0	1	0	2	4	5	5	4	6	4
ENSMUSG00000021901	BAP1	8	10	8	15	15	14	62	61	44	56	50	52
ENSMUSG00000021902	PHF7	2	4	2	1	1	1	4	8	4	3	1	6
ENSMUSG00000021905	DPH3	5	6	7	3	5	8	32	40	21	35	34	23
ENSMUSG00000021906	OXNAD1	0	6	1	2	2	3	12	10	5	15	12	12
ENSMUSG00000021910	NISCH	10	18	22	23	21	23	104	102	64	113	65	86
ENSMUSG00000021911	PARG	5	4	6	8	6	4	28	47	27	33	24	24
ENSMUSG00000021913	OGDHL	0	0	1	0	2	0	3	3	3	2	2	3
ENSMUSG00000021916	GLT8D1	0	0	4	2	3	3	4	3	2	6	5	5
ENSMUSG00000021917	SPCS1	11	6	11	10	5	9	55	49	27	36	30	31
ENSMUSG00000021918	NEK4	2	1	1	2	0	1	6	9	6	5	7	8
ENSMUSG00000021928	EBPL	2	2	2	0	3	4	7	13	10	13	14	14
ENSMUSG00000021929	KPNA3	5	10	6	10	12	15	48	56	51	76	48	40
ENSMUSG00000021930	SPRYD7	5	7	1	4	4	1	33	23	29	33	26	19
ENSMUSG00000021932	RNASEH2B	3	6	7	11	10	9	61	54	49	62	47	49
ENSMUSG00000021936	MAPK8	3	0	2	2	1	6	23	15	20	29	17	23
ENSMUSG00000021938	PSPC1	13	19	14	13	19	19	99	79	71	107	58	90
ENSMUSG00000021939	CTSB	4	5	2	4	4	4	24	30	23	28	20	24
ENSMUSG00000021945	ZMYM2	13	12	12	7	8	13	70	51	37	61	59	46
ENSMUSG00000021947	CRYL1	5	6	7	4	5	4	22	41	23	33	27	23
ENSMUSG00000021948	PRKCD	2	2	2	5	4	4	8	15	10	15	10	17
ENSMUSG00000021951	EEF1AKMT1	1	1	2	1	0	0	6	9	4	4	5	10
ENSMUSG00000021952	XPO4	10	17	12	12	14	15	52	61	41	68	44	45
ENSMUSG00000021957	TKT	35	55	54	60	68	67	350	405	291	385	265	358
ENSMUSG00000021958	PINX1	0	3	0	2	2	1	20	17	23	23	16	23
ENSMUSG00000021959	LATS2	2	2	1	0	1	1	7	3	5	8	6	6
ENSMUSG00000021962	DCP1A	1	2	7	2	5	3	20	21	9	29	20	23
ENSMUSG00000021963	SAP18	6	4	5	4	3	6	27	25	15	24	29	19
ENSMUSG00000021965	SKA3	2	3	7	4	6	4	25	23	21	29	17	17
ENSMUSG00000021967	MRPL57	16	13	9	14	16	15	109	101	72	86	77	64
ENSMUSG00000021969	ZDHHC20	24	26	30	41	45	32	123	100	90	131	97	104
ENSMUSG00000021972	HMBOX1	4	8	3	9	2	6	27	23	11	22	19	11
ENSMUSG00000021973	MICU2	5	3	10	13	5	5	29	46	24	39	25	45
ENSMUSG00000021975	INTS9	0	2	3	2	2	4	6	6	11	12	4	11

ENSMUSG00000021978	EXTL3	4	1	4	5	1	1	15	11	4	12	12	21
ENSMUSG00000021981	CAB39L	2	3	5	13	4	3	38	37	33	35	23	19
ENSMUSG00000021982	CDADC1	6	10	4	10	11	4	42	41	31	35	31	25
ENSMUSG00000021983	ATP8A2	22	33	26	26	33	40	124	140	120	162	112	115
ENSMUSG00000021987	MTMR6	4	5	5	9	10	10	41	35	34	38	39	35
ENSMUSG00000021990	SPATA13	2	6	1	4	5	5	16	17	13	26	14	11
ENSMUSG00000021993	MIPEP	0	3	0	3	2	2	7	22	15	16	4	15
ENSMUSG00000021996	ESD	4	7	7	7	8	7	42	44	39	52	37	47
ENSMUSG00000021998	LCP1	28	24	27	30	26	37	178	164	105	202	118	129
ENSMUSG00000022000	ZC3H13	12	9	10	14	19	12	57	65	32	61	42	50
ENSMUSG00000022003	SLC25A30	2	2	3	3	2	0	6	15	7	14	8	10
ENSMUSG00000022008	GPALPP1	2	1	1	0	1	3	12	12	7	8	8	9
ENSMUSG00000022009	NUFIP1	4	3	1	2	2	2	9	14	9	12	13	13
ENSMUSG00000022012	ENOX1	0	0	0	0	0	1	3	0	0	1	1	1
ENSMUSG00000022013	DNAJC15	44	32	34	34	34	44	341	331	275	348	307	284
ENSMUSG00000022014	EPSTI1	36	40	39	51	53	40	362	328	244	380	280	277
ENSMUSG00000022015	TNFSF11	12	15	14	13	11	10	75	75	51	93	62	58
ENSMUSG00000022016	AKAP11	8	8	5	10	6	6	29	38	33	38	25	27
ENSMUSG00000022018	RGCC	6	6	7	8	5	8	47	43	34	47	36	37
ENSMUSG00000022019	TDRD3	9	5	10	10	8	9	32	39	32	42	32	27
ENSMUSG00000022020	NAA16	21	27	24	22	28	21	111	146	89	140	106	120
ENSMUSG00000022021	DIAPH3	10	16	16	22	15	21	77	77	67	73	48	51
ENSMUSG00000022022	MTRF1	2	1	0	0	0	1	9	7	6	13	9	7
ENSMUSG00000022023	WBP4	7	11	12	9	23	14	63	60	41	84	53	54
ENSMUSG00000022024	SUGT1	11	28	18	15	21	11	97	102	70	99	76	75
ENSMUSG00000022031	ELP3	2	2	1	4	2	3	15	15	19	15	9	12
ENSMUSG00000022033	PBK	4	5	7	3	7	4	27	25	16	37	18	25
ENSMUSG00000022034	ESCO2	21	20	19	20	19	16	60	73	49	61	46	58
ENSMUSG00000022035	CCDC25	1	9	3	12	3	12	31	20	23	20	11	23
ENSMUSG00000022039	ADAM2	0	0	0	0	0	0	0	2	0	0	0	0
ENSMUSG00000022040	EPHX2	0	0	0	0	1	0	0	0	0	0	0	0
ENSMUSG00000022043	TRIM35	3	2	4	5	8	1	18	23	11	13	11	14
ENSMUSG00000022044	STMN4	0	0	0	0	2	0	1	1	1	0	0	1
ENSMUSG00000022048	DPYSL2	8	12	7	23	12	11	41	60	35	51	41	41
ENSMUSG00000022051	BNIP3L	0	3	1	2	4	4	15	19	14	24	14	10
ENSMUSG00000022052	PPP2R2A	6	6	5	10	10	6	51	52	50	63	38	33
ENSMUSG00000022064	PIBF1	1	5	1	2	3	1	14	15	6	17	9	8
ENSMUSG00000022070	BORA	8	12	11	11	16	16	70	79	62	79	74	66
ENSMUSG00000022074	TNFRSF10B	3	3	3	4	2	1	16	14	13	19	10	10
ENSMUSG00000022075	RHOBTB2	2	3	6	5	5	7	10	13	10	13	7	12
ENSMUSG00000022089	BIN3	2	6	1	1	3	3	8	6	9	11	8	5
ENSMUSG00000022090	PDLIM2	0	0	1	0	0	0	0	0	2	1	0	1
ENSMUSG00000022092	PPP3CC	0	1	0	0	0	0	0	1	2	0	0	1
ENSMUSG00000022094	SLC39A14	1	1	2	2	0	1	8	4	4	9	4	7
ENSMUSG00000022095	FAM160B2	1	0	1	0	0	2	6	6	4	4	3	4
ENSMUSG00000022099	DMTN	0	0	0	0	0	0	1	0	0	0	0	0
ENSMUSG00000022100	XPO7	2	1	4	6	13	9	39	40	26	38	9	33
ENSMUSG00000022102	DOK2	2	1	5	5	1	1	10	10	11	10	11	11
ENSMUSG00000022105	RB1	0	4	0	7	2	1	12	10	7	11	11	12
ENSMUSG00000022106	RCBTB2	3	1	0	3	1	2	15	10	15	17	6	11
ENSMUSG00000022108	ITM2B	12	16	14	24	13	14	143	134	114	131	105	98
ENSMUSG00000022109	MED4	3	2	1	8	7	5	31	25	21	32	16	21
ENSMUSG00000022110	SUCLA2	3	3	7	7	1	6	38	18	16	45	17	20
ENSMUSG00000022111	UCHL3	6	11	13	6	15	5	97	89	57	99	76	64
ENSMUSG00000022113	TRIM52	0	0	0	0	0	0	0	1	0	0	2	2
ENSMUSG00000022114	SPRY2	1	0	0	1	0	0	0	0	0	2	0	0
ENSMUSG00000022119	RBM26	39	53	49	62	54	42	244	240	214	296	178	199
ENSMUSG00000022120	RNF219	2	3	2	2	7	6	29	32	29	41	30	9

ENSMUSG00000022123	SCEL	0	0	0	0	0	0	0	1	0	0	0	0
ENSMUSG00000022124	FBXL3	19	23	22	23	22	23	99	91	86	87	83	75
ENSMUSG00000022125	CLN5	4	6	6	4	3	5	24	17	15	23	21	7
ENSMUSG00000022126	ACOD1	0	0	0	0	0	0	0	1	1	1	0	1
ENSMUSG00000022130	TGDS	0	2	0	1	1	1	7	6	2	5	5	1
ENSMUSG00000022131	GPR180	6	5	11	8	10	7	52	47	52	48	40	53
ENSMUSG00000022136	DNAJC3	40	46	60	63	72	60	190	208	141	223	152	155
ENSMUSG00000022139	MBNL2	4	7	3	6	2	8	23	24	21	31	17	24
ENSMUSG00000022141	NIPBL	26	30	20	26	40	28	148	140	91	150	93	114
ENSMUSG00000022142	NUP155	9	11	12	8	9	9	79	53	59	66	50	54
ENSMUSG00000022148	FYB	38	61	51	59	63	56	165	207	143	205	173	154
ENSMUSG00000022151	TTC33	3	0	1	3	0	5	7	9	7	8	7	7
ENSMUSG00000022157	MCPT8	0	0	0	0	0	1	1	0	0	0	0	1
ENSMUSG00000022159	RAB2B	1	0	2	2	1	0	6	8	5	4	4	5
ENSMUSG00000022160	METTL3	4	2	7	5	5	2	27	28	9	22	25	26
ENSMUSG00000022174	DAD1	10	11	10	15	14	10	91	73	54	83	73	66
ENSMUSG00000022175	LRP10	10	5	10	9	7	14	55	44	42	36	35	50
ENSMUSG00000022176	REM2	0	0	0	0	0	0	3	0	0	0	0	0
ENSMUSG00000022177	HAUS4	8	3	3	3	5	7	24	32	34	34	38	34
ENSMUSG00000022178	AJUBA	0	0	0	0	0	0	1	0	0	0	0	0
ENSMUSG00000022179	4931414P19F	0	0	0	1	2	1	5	2	3	4	4	5
ENSMUSG00000022181	C6	0	0	0	1	0	0	0	0	0	0	0	0
ENSMUSG00000022184	FBXO4	1	3	3	1	2	2	3	5	9	9	5	5
ENSMUSG00000022185	ACIN1	88	87	107	114	119	85	475	498	347	483	431	414
ENSMUSG00000022186	OXCT1	41	35	50	36	38	39	205	224	145	230	136	180
ENSMUSG00000022191	DROSHA	5	13	14	13	6	9	40	51	30	35	41	32
ENSMUSG00000022193	PSMB5	13	9	13	15	9	10	84	86	70	109	86	75
ENSMUSG00000022194	PABPN1	4	14	7	10	7	6	40	26	24	32	19	24
ENSMUSG00000022195	6030458C11F	3	4	10	8	4	4	20	36	23	33	15	21
ENSMUSG00000022197	PDZD2	0	2	0	1	1	1	0	2	0	1	1	0
ENSMUSG00000022199	SLC22A17	0	0	0	0	1	0	0	0	0	0	0	0
ENSMUSG00000022200	GOLPH3	12	22	8	19	12	8	82	87	64	88	65	77
ENSMUSG00000022201	ZFR	18	26	38	31	31	35	131	135	94	173	101	138
ENSMUSG00000022204	NGDN	8	4	13	9	17	6	58	59	46	55	45	61
ENSMUSG00000022205	SUB1	39	48	34	45	44	49	397	345	238	352	279	291
ENSMUSG00000022206	NPR3	0	1	0	0	0	1	0	0	0	0	0	0
ENSMUSG00000022210	DHRS4	5	2	5	8	5	2	15	29	30	21	13	20
ENSMUSG00000022211	CARMIL3	7	5	3	5	9	3	28	27	34	38	16	23
ENSMUSG00000022214	DCAF11	1	3	4	2	3	0	18	12	9	19	8	13
ENSMUSG00000022216	PSME1	10	18	16	10	10	16	83	101	71	94	75	102
ENSMUSG00000022217	EMC9	1	2	1	2	1	1	5	6	2	8	2	4
ENSMUSG00000022219	CIDEB	0	0	0	0	0	1	0	0	0	0	1	0
ENSMUSG00000022221	RIPK3	0	0	1	0	0	0	0	0	0	0	0	0
ENSMUSG00000022223	SDR39U1	0	2	1	0	0	0	8	8	6	10	3	7
ENSMUSG00000022231	SEMA5A	0	0	0	0	0	0	0	1	0	0	0	0
ENSMUSG00000022234	CCT5	42	61	59	55	58	59	322	378	275	334	259	336
ENSMUSG00000022236	ROPN1L	1	0	0	1	0	0	2	2	1	3	2	4
ENSMUSG00000022241	TARS	17	23	26	28	30	27	131	122	94	129	83	106
ENSMUSG00000022245	SKOR1	0	0	0	0	0	0	0	0	1	0	0	0
ENSMUSG00000022247	BRIX1	12	6	9	14	14	13	91	74	66	94	57	72
ENSMUSG00000022248	RAD1	4	6	12	11	3	4	25	28	21	25	16	21
ENSMUSG00000022253	NADK2	4	5	5	1	6	2	31	35	31	33	17	27
ENSMUSG00000022255	MTDH	179	273	215	299	262	254	753	763	585	786	585	700
ENSMUSG00000022261	SDC2	0	0	0	0	0	0	0	0	0	0	1	0
ENSMUSG00000022263	TRIO	8	14	8	12	14	13	59	50	35	72	33	41
ENSMUSG00000022265	ANK	4	3	7	8	5	8	38	44	36	34	31	40
ENSMUSG00000022270	FAM134B	5	8	5	11	12	5	36	31	25	30	27	26
ENSMUSG00000022272	MYO10	0	4	5	9	12	3	46	38	33	44	32	24

ENSMUSG00000022280	RNF19A	3	2	0	1	0	0	9	7	7	7	3	0
ENSMUSG00000022283	PABPC1	133	219	183	223	190	183	775	746	550	749	572	666
ENSMUSG00000022285	YWHAZ	63	84	105	106	99	103	383	424	354	434	296	402
ENSMUSG00000022286	GRHL2	0	0	0	0	0	0	0	0	0	1	0	0
ENSMUSG00000022292	RRM2B	0	0	2	0	3	3	0	2	1	5	4	5
ENSMUSG00000022295	ATP6V1C1	5	4	9	6	11	7	40	42	31	58	35	42
ENSMUSG00000022297	FZD6	3	4	4	5	5	5	28	28	21	29	23	29
ENSMUSG00000022299	SLC25A32	8	7	7	9	7	9	49	40	32	45	30	36
ENSMUSG00000022300	DCAF13	6	11	9	11	10	16	58	45	39	49	48	38
ENSMUSG00000022307	OXR1	2	6	14	13	10	5	37	46	39	74	38	43
ENSMUSG00000022309	ANGPT1	1	1	2	1	1	2	9	12	9	12	7	4
ENSMUSG00000022312	EIF3H	24	31	36	34	44	32	221	221	172	237	176	194
ENSMUSG00000022313	UTP23	2	5	4	2	6	2	19	28	21	27	18	19
ENSMUSG00000022314	RAD21	8	17	14	13	16	8	105	117	62	111	80	89
ENSMUSG00000022322	SHCBP1	13	14	14	11	14	10	51	74	49	64	52	45
ENSMUSG00000022323	RIDA	5	3	4	6	6	3	34	25	26	36	24	29
ENSMUSG00000022324	MATN2	0	0	0	0	0	0	0	1	0	0	0	0
ENSMUSG00000022325	POP1	12	15	11	14	22	14	88	77	68	93	60	80
ENSMUSG00000022329	STK3	2	8	6	11	5	4	46	46	26	43	32	27
ENSMUSG00000022335	ZFAT	1	0	0	0	0	0	4	4	2	1	2	1
ENSMUSG00000022336	EIF3E	59	76	69	66	86	70	499	508	381	448	363	363
ENSMUSG00000022337	EMC2	7	5	6	9	5	6	65	65	47	73	65	59
ENSMUSG00000022338	ENY2	14	18	18	22	22	17	137	123	94	137	98	116
ENSMUSG00000022339	EBAG9	8	4	4	5	5	6	27	34	26	45	22	20
ENSMUSG00000022340	SYBU	0	0	0	0	0	0	0	0	1	1	1	0
ENSMUSG00000022346	MYC	14	25	16	20	20	7	125	157	141	120	146	115
ENSMUSG00000022350	WASHC5	2	4	0	4	3	0	15	24	14	19	13	9
ENSMUSG00000022351	SQLE	10	11	11	10	13	8	71	83	47	87	66	70
ENSMUSG00000022353	MTSS1	8	19	4	8	6	17	39	41	32	63	39	33
ENSMUSG00000022354	NDUFB9	12	10	16	19	11	19	102	93	70	79	54	87
ENSMUSG00000022358	FBXO32	0	1	0	0	0	2	6	2	8	6	6	7
ENSMUSG00000022359	WDYHV1	2	1	3	0	1	1	8	14	5	14	14	10
ENSMUSG00000022360	ATAD2	17	13	24	28	27	28	148	138	128	161	92	91
ENSMUSG00000022361	ZHX1	7	13	5	10	8	4	56	37	30	48	31	47
ENSMUSG00000022362	GM29394	0	0	0	0	0	0	0	0	0	0	0	1
ENSMUSG00000022364	TBC1D31	4	8	8	8	11	4	23	29	18	38	16	27
ENSMUSG00000022365	DERL1	10	10	8	11	15	12	71	68	62	76	47	40
ENSMUSG00000022369	MTBP	6	3	2	4	6	5	32	35	28	44	21	25
ENSMUSG00000022370	MRPL13	14	21	15	14	22	8	102	81	79	99	66	69
ENSMUSG00000022372	SLA	11	8	11	7	15	13	28	42	25	42	41	31
ENSMUSG00000022375	LRRC6	0	0	0	0	0	0	1	0	0	0	1	0
ENSMUSG00000022377	ASAP1	4	5	3	3	3	5	9	16	11	10	5	11
ENSMUSG00000022378	FAM49B	11	13	17	9	13	14	107	101	61	88	65	72
ENSMUSG00000022385	GTSE1	4	0	2	4	4	3	13	17	11	10	14	9
ENSMUSG00000022386	TRMU	2	0	2	1	3	1	10	7	3	9	11	11
ENSMUSG00000022387	BRD1	19	16	32	28	20	19	98	112	96	125	77	98
ENSMUSG00000022389	TEF	2	2	0	0	3	0	4	9	7	9	7	6
ENSMUSG00000022390	ZC3H7B	3	2	3	6	5	4	36	43	19	38	18	29
ENSMUSG00000022391	RANGAP1	28	27	28	34	30	32	202	211	159	214	158	192
ENSMUSG00000022394	L3MBTL2	1	3	4	5	7	5	18	20	11	15	20	22
ENSMUSG00000022400	RBX1	16	15	12	14	16	19	122	106	84	133	94	109
ENSMUSG00000022401	XPNPEP3	4	4	3	3	0	3	16	10	12	12	9	12
ENSMUSG00000022403	ST13	22	45	23	45	31	31	148	136	107	163	107	124
ENSMUSG00000022404	SLC25A17	2	4	7	5	6	8	49	53	58	53	35	46
ENSMUSG00000022407	ADSL	9	13	10	18	13	11	56	69	54	46	42	61
ENSMUSG00000022412	MIEF1	2	6	6	4	6	3	12	20	19	21	20	25
ENSMUSG00000022414	TAB1	6	1	3	2	4	2	18	15	13	9	15	13
ENSMUSG00000022415	SYNGR1	0	0	1	0	0	0	4	0	0	0	2	0

ENSMUSG00000022419	DEPTOR	0	1	0	1	2	3	2	2	4	0	1	2
ENSMUSG00000022420	DNAL4	1	0	1	3	1	2	6	2	7	5	2	4
ENSMUSG00000022421	NPTXR	2	1	1	0	1	0	3	4	0	4	3	1
ENSMUSG00000022422	DSCC1	2	0	1	4	1	2	5	14	9	17	6	6
ENSMUSG00000022425	ENPP2	0	1	1	1	1	0	1	6	4	4	2	3
ENSMUSG00000022426	JOSD1	6	10	4	5	14	5	29	41	33	26	32	35
ENSMUSG00000022427	TOMM22	16	29	17	23	16	17	114	135	93	130	121	120
ENSMUSG00000022428	CBY1	0	2	0	1	1	3	10	11	7	14	6	9
ENSMUSG00000022433	CSNK1E	4	2	3	4	4	1	11	6	6	18	12	15
ENSMUSG00000022434	FAM118A	1	4	5	3	2	2	18	18	9	21	19	22
ENSMUSG00000022436	SH3BP1	16	20	15	25	20	16	74	79	67	71	80	73
ENSMUSG00000022437	SAMM50	9	8	11	8	7	14	37	43	38	51	35	45
ENSMUSG00000022439	PARVG	3	3	4	9	1	8	14	24	15	20	7	12
ENSMUSG00000022442	TTLL1	1	1	1	0	0	0	5	3	4	5	6	5
ENSMUSG00000022443	MYH9	156	219	190	228	184	202	844	933	687	949	629	765
ENSMUSG00000022445	CYP2D26	0	0	0	0	0	0	0	0	0	0	0	1
ENSMUSG00000022450	NDUFA6	29	32	35	49	36	33	226	194	138	187	168	175
ENSMUSG00000022451	TWF1	9	6	9	4	6	6	29	27	14	25	25	22
ENSMUSG00000022452	SMDT1	14	23	23	19	21	32	120	112	91	116	103	97
ENSMUSG00000022453	NAGA	2	3	2	3	5	4	9	7	6	21	10	4
ENSMUSG00000022454	NELL2	0	0	0	0	0	0	0	0	0	0	1	0
ENSMUSG00000022456	37865	0	0	0	0	0	0	0	0	0	0	0	1
ENSMUSG00000022462	SLC38A2	50	51	44	40	52	43	318	272	208	316	204	248
ENSMUSG00000022463	SREBF2	12	11	17	21	16	11	92	106	62	97	48	80
ENSMUSG00000022466	RPAP3	3	9	5	6	6	6	34	33	22	50	34	21
ENSMUSG00000022469	RAPGEF3	1	0	1	1	3	1	5	4	1	4	6	8
ENSMUSG00000022471	XRCC6	7	11	5	7	9	4	53	58	42	42	31	54
ENSMUSG00000022472	DESI1	5	4	2	8	6	9	25	39	21	26	21	18
ENSMUSG00000022474	PMM1	8	6	2	5	5	8	26	25	20	21	19	20
ENSMUSG00000022475	HDAC7	9	1	3	4	7	8	29	26	26	32	16	22
ENSMUSG00000022476	POLR3H	1	5	6	7	8	7	44	28	25	45	20	32
ENSMUSG00000022477	ACO2	11	10	8	14	10	9	67	68	49	86	50	51
ENSMUSG00000022479	VDR	0	1	0	1	0	0	2	2	3	1	0	1
ENSMUSG00000022483	COL2A1	0	0	0	0	0	0	1	0	1	2	0	0
ENSMUSG00000022484	HOXC10	0	3	2	2	5	1	16	14	8	17	7	17
ENSMUSG00000022485	HOXC5	0	0	1	1	0	0	0	1	0	0	2	0
ENSMUSG00000022488	NCKAP1L	5	5	1	4	5	4	19	32	15	15	18	15
ENSMUSG00000022489	PDE1B	4	3	3	4	9	4	8	8	8	9	11	7
ENSMUSG00000022490	PPP1R1A	0	0	0	0	0	0	1	0	0	0	0	0
ENSMUSG00000022498	TXNDC11	10	7	13	13	6	11	56	75	54	56	41	41
ENSMUSG00000022500	LITAF	4	14	6	4	8	3	45	29	22	43	19	33
ENSMUSG00000022501	PRM1	0	0	0	0	0	0	0	0	2	1	0	0
ENSMUSG00000022503	NUBP1	2	6	4	6	4	8	35	37	19	40	12	17
ENSMUSG00000022507	1810013L24R	6	12	4	6	5	12	31	44	35	32	38	27
ENSMUSG00000022508	BCL6	2	2	6	2	6	3	17	25	14	13	17	17
ENSMUSG00000022514	IL1RAP	1	1	3	1	1	1	9	12	9	7	4	5
ENSMUSG00000022515	ANKS3	2	5	6	5	6	7	18	15	21	24	17	17
ENSMUSG00000022516	NUDT16L1	4	7	7	8	5	11	28	26	29	44	23	19
ENSMUSG00000022517	MGRN1	0	2	3	0	3	1	9	10	11	11	8	15
ENSMUSG00000022519	SRL	0	1	0	1	0	0	2	5	2	1	3	3
ENSMUSG00000022521	CREBBP	30	38	32	34	36	40	123	156	95	153	94	89
ENSMUSG00000022526	ZFP251	5	9	13	14	6	10	44	39	18	46	32	32
ENSMUSG00000022529	ZFP263	7	9	6	9	11	12	58	48	30	40	41	32
ENSMUSG00000022533	ATP13A3	29	41	32	54	34	40	179	148	114	182	115	135
ENSMUSG00000022536	GLYR1	9	17	18	21	16	11	89	93	58	99	61	77
ENSMUSG00000022537	TMEM44	0	0	0	1	1	0	2	1	1	1	2	0
ENSMUSG00000022538	LSG1	8	11	12	11	7	5	57	64	39	59	38	54
ENSMUSG00000022540	ROGDI	1	1	0	4	0	3	3	7	2	5	6	8

ENSMUSG00000022543	4930451G09I	0	0	0	0	1	0	0	2	0	1	1	0
ENSMUSG00000022544	EEF2KMT	0	2	2	6	4	2	14	18	12	15	4	15
ENSMUSG00000022545	ERCC4	1	2	3	3	3	2	9	11	4	9	5	4
ENSMUSG00000022546	GPT	0	0	1	2	2	0	2	2	1	2	4	0
ENSMUSG00000022550	ADCK5	2	6	0	1	2	2	5	11	10	7	5	8
ENSMUSG00000022551	CYC1	20	20	19	23	35	20	175	183	137	175	170	155
ENSMUSG00000022552	SHARPIN	3	4	2	4	5	4	11	15	14	17	9	12
ENSMUSG00000022553	MAF1	7	7	4	7	4	2	47	44	37	42	25	27
ENSMUSG00000022554	HGH1	2	2	2	9	1	6	17	29	17	21	18	22
ENSMUSG00000022555	DGAT1	1	4	1	3	4	1	2	8	5	11	8	10
ENSMUSG00000022556	HSF1	7	2	4	7	5	3	35	20	19	33	24	24
ENSMUSG00000022557	BOP1	3	3	4	6	8	7	31	34	29	34	21	30
ENSMUSG00000022558	MROH1	4	4	2	0	7	6	16	22	8	14	18	12
ENSMUSG00000022559	FBXL6	2	5	6	7	7	10	39	45	39	45	31	48
ENSMUSG00000022560	SLC52A2	4	2	3	5	2	5	16	21	18	11	15	24
ENSMUSG00000022561	GPAA1	6	1	6	4	3	3	27	30	11	27	14	23
ENSMUSG00000022562	OPLAH	0	0	0	0	1	0	2	1	1	0	0	2
ENSMUSG00000022564	GRINA	13	6	3	5	9	7	36	28	32	37	25	31
ENSMUSG00000022565	PLEC	21	30	30	29	23	36	109	114	101	100	90	97
ENSMUSG00000022568	SCRIB	10	7	5	11	10	14	52	56	43	56	40	51
ENSMUSG00000022570	TSTA3	2	3	3	3	6	4	17	19	9	19	15	14
ENSMUSG00000022571	PYCRL	4	11	5	4	7	11	29	32	25	29	23	30
ENSMUSG00000022574	NAPRT	3	0	2	0	0	0	0	2	1	1	2	2
ENSMUSG00000022575	GSDMD	0	0	1	0	0	0	0	0	0	0	0	0
ENSMUSG00000022586	LY6I	0	0	0	0	0	0	0	0	0	1	0	0
ENSMUSG00000022587	LY6E	16	8	16	18	14	9	82	76	74	79	62	85
ENSMUSG00000022596	SLURP1	0	0	0	0	0	0	2	1	3	3	0	3
ENSMUSG00000022601	ZBTB11	164	225	236	267	242	261	1588	1495	1071	1613	1012	1164
ENSMUSG00000022604	CEP97	2	2	1	1	4	3	23	24	10	18	7	17
ENSMUSG00000022607	PTK2	0	0	0	0	0	0	1	0	0	1	0	0
ENSMUSG00000022610	MAPK12	0	0	1	1	0	0	1	0	0	0	0	1
ENSMUSG00000022614	LMF2	6	5	6	3	4	5	23	21	23	14	24	21
ENSMUSG00000022617	CHKB	3	4	4	4	3	3	9	13	8	13	13	13
ENSMUSG00000022620	ARSA	0	1	1	0	0	0	5	3	0	0	0	2
ENSMUSG00000022621	RABL2	0	2	1	1	2	1	3	8	6	12	6	7
ENSMUSG00000022634	YAF2	6	8	8	7	6	3	50	49	33	54	37	35
ENSMUSG00000022635	ZCRB1	35	30	30	42	35	30	201	219	165	204	202	179
ENSMUSG00000022637	CBLB	5	5	9	3	10	4	32	33	22	33	28	17
ENSMUSG00000022639	DUBR	2	1	1	0	0	0	3	6	1	5	4	3
ENSMUSG00000022641	BBX	31	38	29	32	27	43	223	208	150	224	180	161
ENSMUSG00000022652	MORC1	2	2	3	4	3	4	35	24	21	36	26	27
ENSMUSG00000022656	NECTIN3	0	0	0	0	0	0	0	1	0	0	0	0
ENSMUSG00000022657	CD96	3	12	8	8	11	4	70	57	37	81	46	53
ENSMUSG00000022658	TAGLN3	0	0	0	0	0	0	1	2	0	1	0	0
ENSMUSG00000022659	GCSAM	0	0	0	0	0	0	0	0	0	0	1	0
ENSMUSG00000022663	ATG3	3	9	7	16	10	11	33	39	30	40	34	29
ENSMUSG00000022664	SLC35A5	1	1	2	3	5	4	17	15	11	19	13	15
ENSMUSG00000022668	GTPBP8	0	3	3	2	1	0	9	6	6	7	4	8
ENSMUSG00000022671	MZT2	3	7	2	1	6	7	18	31	17	7	17	15
ENSMUSG00000022672	PRKDC	2	6	6	7	12	7	22	30	22	45	17	22
ENSMUSG00000022673	MCM4	14	30	27	38	24	27	178	205	130	293	137	186
ENSMUSG00000022674	UBE2V2	1	5	7	7	1	1	31	35	23	23	19	23
ENSMUSG00000022677	FOPNL	6	3	5	7	4	6	26	38	26	29	21	20
ENSMUSG00000022678	NDE1	1	5	7	6	4	6	19	20	15	21	18	15
ENSMUSG00000022679	MPV17L	0	3	1	1	1	1	0	0	2	4	2	2
ENSMUSG00000022680	PDXDC1	10	7	6	14	7	8	34	37	41	38	43	32
ENSMUSG00000022681	NTAN1	1	1	4	3	3	3	21	24	6	16	8	23
ENSMUSG00000022682	RRN3	10	10	5	16	7	7	58	55	37	50	43	43

ENSMUSG00000022684	BFAR	15	16	16	26	24	11	63	66	52	78	55	66
ENSMUSG00000022685	PARN	1	3	4	5	7	2	18	15	12	17	11	19
ENSMUSG00000022698	NAA50	26	24	25	32	24	33	160	133	107	148	85	94
ENSMUSG00000022701	CCDC191	6	6	20	12	13	7	46	49	39	59	32	34
ENSMUSG00000022702	HIRA	10	12	9	14	15	11	61	69	43	60	65	57
ENSMUSG00000022704	QTRT2	0	3	2	2	5	4	17	24	15	27	9	14
ENSMUSG00000022706	MRPL40	3	9	5	3	5	4	50	35	37	50	37	32
ENSMUSG00000022707	GBE1	3	3	1	0	2	1	18	11	9	11	4	10
ENSMUSG00000022708	ZBTB20	1	0	0	0	0	0	0	0	0	0	0	0
ENSMUSG00000022710	USP7	9	26	27	32	29	25	125	102	84	105	85	102
ENSMUSG00000022711	PMM2	9	6	4	9	5	11	42	37	24	30	34	33
ENSMUSG00000022718	DGCR8	5	4	8	8	6	3	19	26	10	25	15	27
ENSMUSG00000022721	TRMT2A	10	19	15	10	14	13	62	45	39	57	40	39
ENSMUSG00000022723	CRYBG3	2	0	2	2	5	3	23	17	14	10	7	18
ENSMUSG00000022724	RIOX2	11	10	11	5	8	6	46	73	40	45	39	43
ENSMUSG00000022742	CPOX	3	9	2	11	4	12	36	42	26	44	23	29
ENSMUSG00000022744	CLDND1	3	9	11	4	11	5	31	16	12	26	24	27
ENSMUSG00000022747	ST3GAL6	0	0	0	0	0	0	0	0	1	0	0	0
ENSMUSG00000022748	CMSS1	1	3	1	4	10	0	12	11	11	18	11	11
ENSMUSG00000022749	TBC1D23	1	4	5	6	4	4	14	16	11	15	16	7
ENSMUSG00000022750	KLHL22	6	7	7	5	11	4	21	28	20	26	16	22
ENSMUSG00000022751	NIT2	0	0	6	3	2	2	15	11	16	12	8	20
ENSMUSG00000022752	TOMM70A	10	19	8	15	14	13	87	100	68	95	70	71
ENSMUSG00000022753	TMEM30C	0	0	1	1	0	0	1	2	1	2	1	0
ENSMUSG00000022757	TFG	5	11	18	12	18	17	76	68	50	88	52	62
ENSMUSG00000022760	THAP7	3	3	1	1	3	3	19	14	4	10	7	6
ENSMUSG00000022761	LZTR1	3	2	5	7	7	6	21	19	23	29	13	15
ENSMUSG00000022763	AIFM3	0	0	0	0	0	0	0	1	0	0	0	0
ENSMUSG00000022765	SNAP29	3	2	3	4	3	5	13	15	13	17	12	19
ENSMUSG00000022768	CCDC116	0	2	0	2	0	0	3	3	2	4	2	3
ENSMUSG00000022769	SDF2L1	12	12	19	22	12	8	77	83	53	80	49	63
ENSMUSG00000022770	DLG1	8	5	10	9	11	7	42	42	24	47	31	30
ENSMUSG00000022771	PPIL2	31	43	36	42	53	39	278	265	201	236	216	216
ENSMUSG00000022772	SENP5	7	11	10	17	9	14	55	49	34	53	44	37
ENSMUSG00000022773	YPEL1	5	7	5	6	6	6	27	32	23	25	34	25
ENSMUSG00000022774	NCBP2	6	8	5	7	15	9	60	82	51	63	43	53
ENSMUSG00000022779	TOP3B	4	4	2	6	5	4	15	16	10	18	17	12
ENSMUSG00000022780	MELTF	0	1	0	0	0	0	0	0	0	0	0	0
ENSMUSG00000022781	PAK2	24	35	16	20	27	23	119	124	100	133	76	98
ENSMUSG00000022787	WDR53	3	3	5	1	5	2	12	19	14	19	7	19
ENSMUSG00000022789	DNM1L	18	25	17	21	27	18	122	113	92	130	84	87
ENSMUSG00000022791	TNK2	23	20	22	32	28	18	81	73	69	88	56	78
ENSMUSG00000022792	YARS2	5	7	8	15	6	2	45	51	43	41	42	37
ENSMUSG00000022797	TFRC	31	43	33	28	47	31	235	282	203	290	188	192
ENSMUSG00000022799	ARHGAP31	3	1	4	7	5	1	8	10	4	9	12	5
ENSMUSG00000022800	FYTTD1	8	13	9	19	13	10	99	98	60	89	41	66
ENSMUSG00000022801	LRCH3	4	4	9	7	7	6	33	29	36	46	18	26
ENSMUSG00000022802	LMLN	0	0	1	1	1	1	2	1	3	4	1	1
ENSMUSG00000022803	POPDC2	0	0	0	1	0	0	0	0	0	0	1	1
ENSMUSG00000022807	OSBPL11	3	1	2	3	4	3	11	21	6	16	9	16
ENSMUSG00000022808	SNX4	5	2	9	1	7	5	15	36	24	32	14	22
ENSMUSG00000022811	ZFP148	14	22	24	30	23	23	98	107	87	117	91	71
ENSMUSG00000022812	GSK3B	20	29	22	32	34	23	107	130	95	114	62	82
ENSMUSG00000022814	UMPS	6	9	16	13	9	12	82	83	48	97	75	76
ENSMUSG00000022817	ITGB5	2	1	2	2	0	2	13	6	7	7	7	5
ENSMUSG00000022820	NDUFB4	12	13	11	15	16	10	62	50	47	64	52	64
ENSMUSG00000022822	ABCC5	10	9	12	9	10	18	44	46	30	62	52	50
ENSMUSG00000022827	RABL3	0	2	3	1	1	1	8	8	9	5	8	9

ENSMUSG00000022828	GTF2E1	0	3	4	6	5	3	32	34	28	28	24	20
ENSMUSG00000022831	HCLS1	9	10	6	17	14	18	63	80	65	78	49	62
ENSMUSG00000022833	CCDC14	2	5	2	0	3	2	9	13	8	19	12	6
ENSMUSG00000022837	IQCB1	2	1	5	2	6	2	2	7	3	8	3	10
ENSMUSG00000022838	EAF2	0	0	0	0	0	0	2	1	1	0	0	0
ENSMUSG00000022841	AP2M1	2	4	3	4	5	5	13	16	11	21	9	17
ENSMUSG00000022843	CLCN2	0	1	0	1	1	1	2	2	0	3	2	3
ENSMUSG00000022847	THPO	0	0	0	0	0	0	0	0	0	1	0	0
ENSMUSG00000022848	DIRC2	5	2	6	8	2	5	38	35	26	46	25	38
ENSMUSG00000022849	HSPBAP1	6	8	5	2	7	6	25	34	25	28	25	28
ENSMUSG00000022853	EHHADH	0	0	0	0	0	0	0	0	1	0	1	0
ENSMUSG00000022855	SENP2	43	45	43	48	49	42	189	165	146	157	129	149
ENSMUSG00000022856	TMEM41A	5	3	2	2	3	4	20	25	14	23	13	19
ENSMUSG00000022858	TRA2B	35	38	34	46	38	43	192	179	132	177	153	169
ENSMUSG00000022861	DGKG	0	0	0	0	0	0	1	0	0	1	2	0
ENSMUSG00000022863	BTG3	0	0	0	1	0	1	8	11	5	9	11	7
ENSMUSG00000022864	D16ERTD472	3	7	2	6	7	9	28	30	21	21	21	20
ENSMUSG00000022865	CXADR	0	0	0	0	0	1	0	0	1	1	2	0
ENSMUSG00000022867	USP25	9	15	14	14	8	19	75	80	43	60	30	52
ENSMUSG00000022875	KNG1	0	0	0	0	0	0	0	2	3	2	3	2	1
ENSMUSG00000022876	SAMSN1	3	8	7	2	9	3	40	36	24	36	25	25
ENSMUSG00000022878	ADIPOQ	0	0	0	0	0	0	1	0	0	0	0	0
ENSMUSG00000022881	RFC4	9	15	13	17	18	14	81	75	66	88	57	59
ENSMUSG00000022884	EIF4A2	22	23	19	17	35	25	146	155	113	154	107	109
ENSMUSG00000022885	ST6GAL1	1	3	1	1	5	4	7	10	9	17	2	6
ENSMUSG00000022887	MASP1	0	0	0	0	0	0	0	0	1	0	0	0
ENSMUSG00000022889	MRPL39	6	9	9	12	10	11	32	47	36	63	32	47
ENSMUSG00000022890	ATP5J	38	68	56	50	84	56	549	541	390	513	418	462
ENSMUSG00000022895	ETS2	0	1	2	2	2	0	10	8	11	13	3	8
ENSMUSG00000022897	DYRK1A	22	25	20	34	18	27	99	90	78	126	74	94
ENSMUSG00000022898	DSCR3	1	5	3	5	4	2	18	25	20	13	9	17
ENSMUSG00000022899	SLC15A2	0	2	2	2	1	0	1	6	4	4	4	2
ENSMUSG00000022900	ILDR1	0	0	0	0	0	0	0	0	2	1	0	0
ENSMUSG00000022901	CD86	0	1	0	0	0	0	1	1	0	0	0	0
ENSMUSG00000022905	KPNA1	1	11	12	10	8	7	55	50	33	52	38	30
ENSMUSG00000022906	PARP9	1	3	2	1	3	0	20	19	9	19	8	4
ENSMUSG00000022911	ARL13B	0	2	2	0	1	0	1	0	5	4	3	1
ENSMUSG00000022912	PROS1	0	0	0	0	0	0	0	0	0	1	1	0
ENSMUSG00000022913	PSMG1	3	16	11	18	14	12	65	67	52	57	47	35
ENSMUSG00000022914	BRWD1	9	15	8	19	18	16	63	66	40	57	46	39
ENSMUSG00000022935	GRIK1	0	0	0	0	0	0	1	0	0	0	0	0
ENSMUSG00000022940	PIGP	27	27	29	37	32	43	185	190	134	225	131	139
ENSMUSG00000022941	RIPPLY3	0	0	0	0	0	0	0	0	1	0	0	0
ENSMUSG00000022945	CHAF1B	11	8	8	13	13	14	57	68	54	58	46	56
ENSMUSG00000022946	DOPEY2	1	0	0	1	0	0	1	2	1	3	2	1
ENSMUSG00000022948	SETD4	0	1	1	2	1	0	15	12	8	13	8	11
ENSMUSG00000022949	CLIC6	1	1	5	6	2	2	13	13	5	15	8	12
ENSMUSG00000022951	RCAN1	1	1	1	1	1	0	2	5	3	1	1	1
ENSMUSG00000022952	RUNX1	10	7	7	11	7	8	42	50	41	59	28	37
ENSMUSG00000022956	ATP5O	32	52	30	34	35	28	204	228	179	222	180	165
ENSMUSG00000022957	ITSN1	0	0	0	1	1	0	0	0	0	0	3	0
ENSMUSG00000022960	DONSON	4	3	7	5	1	2	11	22	7	18	7	5
ENSMUSG00000022961	SON	150	157	148	195	177	203	546	550	395	624	386	470
ENSMUSG00000022962	GART	24	32	35	32	30	26	135	172	113	172	101	136
ENSMUSG00000022964	TMEM50B	1	1	2	3	2	1	7	18	6	10	6	6
ENSMUSG00000022965	IFNGR2	8	13	22	11	18	14	47	50	46	66	50	41
ENSMUSG00000022967	IFNAR1	4	4	5	9	4	10	18	36	31	28	22	28
ENSMUSG00000022969	IL10RB	3	2	4	0	4	4	7	7	12	7	11	20

ENSMUSG00000022971	IFNAR2	3	1	0	4	1	1	9	7	3	4	11	6
ENSMUSG00000022972	1110004E09F	1	3	2	2	0	5	14	20	9	22	16	17
ENSMUSG00000022973	SYNJ1	5	1	3	7	7	2	18	20	21	22	18	15
ENSMUSG00000022974	PAXBP1	17	28	20	31	34	35	129	143	101	154	117	124
ENSMUSG00000022978	MIS18A	13	10	5	12	14	9	179	151	85	228	55	87
ENSMUSG00000022982	SOD1	105	136	104	146	132	137	787	808	601	784	558	778
ENSMUSG00000022983	SCAF4	14	12	21	21	18	18	84	84	55	71	69	73
ENSMUSG00000022992	KANSL2	1	1	2	2	1	1	8	4	7	6	6	6
ENSMUSG00000022995	ENAH	0	1	0	0	0	0	0	0	1	2	1	0
ENSMUSG00000022996	WNT10B	3	5	3	2	6	5	23	18	20	25	20	13
ENSMUSG00000022999	LMBR1L	6	4	3	4	3	2	19	21	13	13	13	22
ENSMUSG00000023004	TUBA1B	38	51	27	55	55	39	276	347	241	303	273	295
ENSMUSG00000023007	PRPF40B	1	0	0	0	0	0	1	0	0	0	0	1
ENSMUSG00000023008	FMNL3	4	4	3	6	3	3	17	14	11	12	13	10
ENSMUSG00000023009	NCKAP5L	4	0	1	1	3	0	12	4	2	4	3	2
ENSMUSG00000023010	TMBIM6	3	9	16	13	20	23	80	66	60	73	60	64
ENSMUSG00000023015	RACGAP1	17	8	17	12	6	13	64	82	45	58	58	43
ENSMUSG00000023018	SMARCD1	4	4	8	6	2	7	21	29	22	30	22	24
ENSMUSG00000023019	GPD1	0	0	0	0	0	0	0	2	0	0	0	1
ENSMUSG00000023020	COX14	13	8	9	17	7	12	56	77	48	44	40	45
ENSMUSG00000023021	CERS5	7	9	5	13	9	5	43	47	37	49	48	44
ENSMUSG00000023022	LIMA1	6	5	4	7	7	4	31	17	20	39	10	24
ENSMUSG00000023025	LARP4	20	29	19	29	19	16	145	137	106	170	112	103
ENSMUSG00000023026	DIP2B	9	9	12	14	12	14	44	47	45	48	37	37
ENSMUSG00000023027	ATF1	13	4	5	10	12	9	91	80	54	112	54	52
ENSMUSG00000023030	SLC11A2	2	6	7	7	2	2	18	15	16	20	11	20
ENSMUSG00000023031	CELA1	0	0	0	1	0	1	0	0	0	0	0	0
ENSMUSG00000023032	SLC4A8	1	0	0	1	2	1	2	1	3	5	2	3
ENSMUSG00000023033	SCN8A	4	2	1	1	2	2	0	1	1	4	2	3
ENSMUSG00000023034	NR4A1	9	8	7	14	7	10	42	33	37	44	38	46
ENSMUSG00000023036	PCDHGC4	1	0	0	0	0	0	0	0	1	0	0	0
ENSMUSG00000023044	CSAD	3	2	3	1	0	1	7	9	11	13	9	8
ENSMUSG00000023045	SOAT2	2	3	2	4	5	2	6	3	9	6	6	6
ENSMUSG00000023046	IGFBP6	0	0	1	1	2	1	15	19	15	16	14	14
ENSMUSG00000023047	AMHR2	0	0	0	1	0	0	1	0	0	0	0	0
ENSMUSG00000023048	PRR13	10	9	7	11	5	7	50	46	49	48	40	35
ENSMUSG00000023050	MAP3K12	56	69	63	70	70	62	191	225	217	232	184	215
ENSMUSG00000023051	TARBP2	0	0	0	0	0	1	0	1	1	0	0	1
ENSMUSG00000023055	CALCOCO1	2	0	1	2	1	1	5	6	4	5	1	7
ENSMUSG00000023066	RTTN	1	3	5	5	4	0	6	7	5	16	7	5
ENSMUSG00000023068	NUS1	17	18	19	18	22	21	84	89	61	83	73	66
ENSMUSG00000023072	CEP89	1	4	6	6	8	5	30	24	18	47	15	22
ENSMUSG00000023074	MOSPD1	0	1	2	1	1	1	10	14	7	12	5	9
ENSMUSG00000023075	AKIRIN1	4	3	3	2	8	8	59	70	33	72	35	44
ENSMUSG00000023079	GTF2IRD1	0	0	0	0	0	0	0	0	0	0	1	0
ENSMUSG00000023084	LRRC71	1	0	0	0	0	0	0	1	2	2	1	2
ENSMUSG00000023087	NOCT	6	9	3	9	3	7	34	26	20	47	22	34
ENSMUSG00000023088	ABCC1	8	8	7	5	7	6	28	39	20	31	13	34
ENSMUSG00000023089	NDUFA5	36	38	46	74	43	43	325	394	257	386	265	316
ENSMUSG00000023094	MSRB2	1	1	0	0	1	0	5	3	9	5	3	10
ENSMUSG00000023104	RFC2	12	15	16	18	17	18	98	96	64	100	71	70
ENSMUSG00000023106	DENR	19	32	16	90	21	25	136	128	104	160	91	113
ENSMUSG00000023110	PRMT5	20	22	19	29	23	18	143	147	124	132	104	115
ENSMUSG00000023118	SYMPK	14	8	16	10	10	10	54	61	51	56	53	71
ENSMUSG00000023122	SULT1C2	0	0	0	0	0	0	1	0	0	0	0	0
ENSMUSG00000023132	GZMA	0	0	0	0	0	0	0	1	1	0	0	1
ENSMUSG00000023143	NAGPA	4	4	0	0	1	1	11	17	14	11	12	15
ENSMUSG00000023147	WRB	9	18	11	11	26	11	53	58	31	57	46	49

ENSMUSG00000023150	IVNS1ABP	31	57	44	61	52	51	309	306	269	322	239	288
ENSMUSG00000023156	RPP14	5	2	5	7	5	6	35	31	27	24	20	21
ENSMUSG00000023159	PSG29	0	0	0	0	0	0	0	0	0	0	1	0
ENSMUSG00000023169	SLC38A1	54	64	79	80	74	66	285	282	226	325	195	261
ENSMUSG00000023170	GPS2	3	11	6	25	12	10	53	56	36	54	42	47
ENSMUSG00000023175	BSG	14	21	24	22	25	30	161	139	102	144	115	130
ENSMUSG00000023186	VWA5A	0	5	2	3	0	1	9	18	10	18	11	13
ENSMUSG00000023191	P3H3	0	0	0	0	0	0	0	0	0	0	0	1
ENSMUSG00000023206	IL15RA	2	2	3	2	3	4	16	21	16	20	16	18
ENSMUSG00000023235	CCL25	2	3	3	7	4	2	21	18	22	20	17	20
ENSMUSG00000023249	PARP3	1	4	9	1	3	7	18	18	14	18	11	24
ENSMUSG00000023259	SLC26A6	0	0	0	0	1	0	3	0	1	1	1	1
ENSMUSG00000023262	ACY1	0	1	0	2	3	2	5	4	8	10	4	6
ENSMUSG00000023266	FRS3	1	3	0	2	0	1	1	1	0	2	0	2
ENSMUSG00000023272	CRELD2	5	6	6	12	12	11	41	25	23	40	28	21
ENSMUSG00000023277	TWF2	15	16	16	15	24	22	88	96	101	107	69	74
ENSMUSG00000023284	ZFP605	0	0	2	2	2	0	2	5	6	3	7	3
ENSMUSG00000023286	UBE2J2	5	10	9	12	10	7	41	54	41	43	37	44
ENSMUSG00000023307	MARCH5	9	6	3	5	6	6	56	56	35	59	26	41
ENSMUSG00000023328	ACHE	0	0	0	0	0	0	1	0	0	1	0	0
ENSMUSG00000023330	DTWD1	5	3	6	2	5	4	16	28	26	16	18	15
ENSMUSG00000023345	POC1A	3	2	2	2	5	5	20	20	11	15	27	16
ENSMUSG00000023348	TRIP6	1	1	3	2	1	2	2	6	0	2	2	1
ENSMUSG00000023353	AGAP3	1	9	9	14	9	9	57	39	36	31	37	38
ENSMUSG00000023367	TMEM176A	1	0	0	0	0	1	2	2	1	1	0	1
ENSMUSG00000023393	SLC17A9	8	4	7	8	10	10	34	32	24	34	31	25
ENSMUSG00000023403	STK31	1	0	0	0	0	0	0	1	0	2	0	0
ENSMUSG00000023411	NFATC4	0	0	0	0	0	0	0	1	0	0	1	0
ENSMUSG00000023443	ESX1	0	0	1	1	0	0	4	3	0	6	1	3
ENSMUSG00000023452	PISD	2	2	2	2	2	5	20	16	15	13	12	16
ENSMUSG00000023456	TPI1	7	21	16	13	28	15	148	154	111	125	87	128
ENSMUSG00000023460	RAB12	12	17	24	21	20	19	56	77	46	74	58	60
ENSMUSG00000023467	TULP2	0	0	0	0	0	0	1	1	0	0	0	0
ENSMUSG00000023473	CELSR3	0	0	0	0	0	0	1	0	0	0	0	0
ENSMUSG00000023484	PRPH	0	0	0	0	0	0	0	0	0	0	0	1
ENSMUSG00000023495	PCBP4	1	2	3	4	5	0	5	4	9	10	6	5
ENSMUSG00000023505	CDCA3	9	9	12	15	14	15	53	64	60	79	54	58
ENSMUSG00000023571	C1QTNF12	0	1	1	0	2	1	1	1	3	4	2	2
ENSMUSG00000023572	CCNDBP1	3	4	5	5	1	6	31	37	19	30	33	26
ENSMUSG00000023707	OGFOD2	3	7	5	15	9	9	40	57	49	52	45	46
ENSMUSG00000023723	MRPS23	6	7	4	5	7	11	37	39	23	32	27	28
ENSMUSG00000023755	RHEBL1	3	4	1	1	2	2	9	14	4	11	11	15
ENSMUSG00000023764	SFI1	5	4	1	2	8	3	13	13	10	17	10	8
ENSMUSG00000023781	HES7	0	0	0	0	0	0	0	0	0	1	0	0
ENSMUSG00000023791	PIGX	2	4	4	0	4	1	13	11	18	13	6	13
ENSMUSG00000023800	TIAM2	0	1	0	0	0	0	4	1	2	2	3	0
ENSMUSG00000023805	SYNJ2	0	0	1	1	2	0	8	8	5	3	5	4
ENSMUSG00000023809	RPS6KA2	0	0	0	0	0	0	2	1	0	1	0	0
ENSMUSG00000023826	PARK2	0	0	0	0	0	0	0	1	0	0	0	0
ENSMUSG00000023827	AGPAT4	1	0	1	3	2	2	6	4	0	3	2	6
ENSMUSG00000023828	SLC22A3	0	0	0	0	0	0	1	1	0	0	1	0
ENSMUSG00000023830	IGF2R	0	1	1	0	1	3	1	3	6	7	6	2
ENSMUSG00000023832	ACAT2	7	10	13	10	6	13	75	71	41	84	41	82
ENSMUSG00000023845	LNPEP	44	56	55	65	59	56	201	212	177	246	164	195
ENSMUSG00000023852	CHD1	55	59	72	87	82	77	405	394	298	477	283	353
ENSMUSG00000023861	MPC1	0	0	0	0	0	0	1	3	1	4	3	4
ENSMUSG00000023868	PDE10A	0	0	0	0	0	0	0	0	0	0	0	1
ENSMUSG00000023873	1700010I14R	2	0	2	1	3	0	6	5	3	8	3	6

ENSMUSG00000023882	ZFP54	1	2	2	0	1	1	8	3	6	2	2	6
ENSMUSG00000023883	PHF10	28	24	34	34	31	26	140	155	124	177	103	135
ENSMUSG00000023892	ZFP51	3	4	2	3	2	1	9	22	13	13	7	18
ENSMUSG00000023904	HCFC1R1	0	1	3	1	2	3	12	14	9	13	8	12
ENSMUSG00000023905	TNFRSF12A	3	4	0	1	2	2	12	7	8	4	6	10
ENSMUSG00000023908	PKMYT1	5	11	16	12	8	13	45	38	40	45	28	51
ENSMUSG00000023909	PAQR4	2	6	4	2	6	10	16	17	15	17	16	18
ENSMUSG00000023919	CENPQ	2	8	7	5	13	7	75	76	43	75	35	57
ENSMUSG00000023921	MUT	4	9	4	7	6	6	50	35	30	62	27	28
ENSMUSG00000023923	TBC1D5	0	2	1	2	4	3	6	10	6	17	7	6
ENSMUSG00000023927	SATB1	43	41	65	65	60	62	225	233	150	251	179	165
ENSMUSG00000023932	CDC5L	16	23	12	18	15	12	107	97	75	118	64	77
ENSMUSG00000023935	SPATS1	1	1	1	1	0	0	2	3	0	1	3	2
ENSMUSG00000023938	AARS2	12	4	5	4	10	5	25	18	14	23	21	22
ENSMUSG00000023939	MRPL14	14	14	12	19	30	19	114	121	97	111	93	88
ENSMUSG00000023940	SGO1	7	10	9	7	9	9	63	65	44	74	51	50
ENSMUSG00000023942	SLC29A1	0	0	1	1	6	2	0	0	1	1	0	0
ENSMUSG00000023944	HSP90AB1	303	446	360	469	437	460	2463	2406	1781	2762	1835	2109
ENSMUSG00000023947	NFKBIE	0	0	0	0	0	1	0	2	2	1	0	1
ENSMUSG00000023951	VEGFA	13	7	11	12	9	15	66	59	41	76	44	54
ENSMUSG00000023952	GTPBP2	11	12	7	11	12	13	36	27	30	33	22	34
ENSMUSG00000023953	POLH	26	44	38	51	56	34	256	276	234	264	218	255
ENSMUSG00000023963	CYP39A1	0	0	0	0	0	0	0	1	0	0	0	0
ENSMUSG00000023965	FBXL17	3	3	3	2	3	1	13	12	8	10	11	11
ENSMUSG00000023966	RSPH9	0	0	0	0	0	0	0	0	0	1	0	0
ENSMUSG00000023967	MRPS18A	3	3	6	3	5	6	17	26	15	29	33	35
ENSMUSG00000023968	CRIP3	0	0	0	0	0	0	0	0	0	1	1	0
ENSMUSG00000023971	RRP36	2	5	4	1	3	1	32	11	14	24	16	10
ENSMUSG00000023972	PTK7	0	0	0	0	0	0	1	0	0	0	0	0
ENSMUSG00000023973	CNPY3	0	4	4	8	7	4	15	28	19	22	18	18
ENSMUSG00000023977	UBR2	20	23	27	31	31	36	130	148	103	137	111	112
ENSMUSG00000023979	GUCA1B	0	0	0	2	2	0	2	1	1	1	1	1
ENSMUSG00000023980	TAF8	8	6	4	10	9	8	20	31	38	31	40	28
ENSMUSG00000023984	GM20517	0	0	0	0	0	0	0	0	0	1	0	0
ENSMUSG00000023988	BYSL	1	7	7	10	2	8	53	49	35	55	55	61
ENSMUSG00000023990	TFEB	0	6	2	4	4	0	13	10	12	11	11	13
ENSMUSG00000023991	FOXP4	3	4	4	2	5	3	14	15	14	16	10	8
ENSMUSG00000023994	NFYA	6	8	11	9	5	10	51	47	41	63	29	48
ENSMUSG00000024002	BRD4	23	19	32	42	30	27	125	166	122	179	120	118
ENSMUSG00000024006	STK38	14	20	15	13	13	12	108	100	67	126	93	80
ENSMUSG00000024007	PPIL1	6	9	11	15	17	13	61	94	41	66	44	66
ENSMUSG00000024011	PI16	0	1	0	0	0	0	0	0	0	0	0	0
ENSMUSG00000024012	MTCH1	7	10	8	14	12	10	59	59	43	45	49	45
ENSMUSG00000024013	FGD2	0	0	0	0	0	0	0	0	1	0	0	0
ENSMUSG00000024014	PIM1	15	22	9	23	21	12	74	68	54	67	58	74
ENSMUSG00000024018	CCDC167	8	3	6	4	5	6	29	49	26	37	27	36
ENSMUSG00000024019	CMTR1	2	2	1	2	2	0	10	16	7	15	5	13
ENSMUSG00000024026	GLO1	11	13	7	10	10	10	61	56	46	47	53	58
ENSMUSG00000024033	RSPH1	0	0	0	1	0	0	0	0	0	0	1	0
ENSMUSG00000024034	TMPRSS3	0	1	0	0	1	1	0	1	0	1	0	0
ENSMUSG00000024037	WDR4	7	5	6	2	2	4	27	24	13	18	22	22
ENSMUSG00000024038	NDUFV3	34	39	36	47	46	39	289	300	211	290	226	270
ENSMUSG00000024039	CBS	0	0	0	0	0	0	0	0	0	1	0	0
ENSMUSG00000024042	SIK1	6	7	4	5	14	6	19	14	17	25	23	16
ENSMUSG00000024045	AKAP8	0	0	0	0	0	0	0	1	0	0	0	0
ENSMUSG00000024048	MYL12A	19	37	22	36	46	37	316	274	211	292	198	244
ENSMUSG00000024049	MYOM1	1	3	1	3	1	1	10	11	8	7	7	5
ENSMUSG00000024050	WIZ	4	6	10	7	9	4	25	29	23	35	22	31

ENSMUSG00000024052	LPIN2	4	5	6	6	8	7	35	35	23	27	28	24
ENSMUSG00000024054	SMCHD1	8	17	15	22	11	14	75	78	57	107	66	66
ENSMUSG00000024055	CYP4F13	2	0	1	4	1	1	10	12	6	6	7	7
ENSMUSG00000024056	NDC80	17	20	28	21	31	27	109	144	82	112	109	106
ENSMUSG00000024063	LBH	11	21	17	25	19	14	72	94	43	77	49	69
ENSMUSG00000024065	EHD3	0	0	0	0	0	1	0	0	0	0	0	0
ENSMUSG00000024066	XDH	8	8	17	9	12	10	39	57	37	47	29	41
ENSMUSG00000024067	DPY30	5	17	8	7	12	13	69	66	51	66	60	67
ENSMUSG00000024068	SPAST	4	5	4	7	6	6	30	29	27	34	19	23
ENSMUSG00000024069	SLC30A6	2	2	3	1	4	0	22	20	11	11	9	15
ENSMUSG00000024070	PRKD3	4	6	1	2	4	7	26	20	19	23	21	18
ENSMUSG00000024072	YIPF4	12	12	10	14	10	8	53	77	55	65	34	44
ENSMUSG00000024073	BIRC6	78	92	83	81	94	58	340	384	261	373	296	301
ENSMUSG00000024074	CRIM1	8	17	7	10	9	13	45	44	36	40	32	44
ENSMUSG00000024077	STRN	1	2	1	1	2	1	5	10	7	8	7	6
ENSMUSG00000024078	TTC27	3	1	1	1	2	2	18	14	11	15	12	11
ENSMUSG00000024079	EIF2AK2	6	5	4	9	4	7	14	23	13	14	12	30
ENSMUSG00000024081	CEBPZ	18	28	20	30	27	18	132	156	116	174	138	104
ENSMUSG00000024082	NDUFAF7	1	5	0	6	4	2	11	12	12	8	6	12
ENSMUSG00000024083	PJA2	5	17	9	13	18	9	77	88	53	77	61	52
ENSMUSG00000024085	MAN2A1	5	6	8	11	1	6	25	16	21	34	19	16
ENSMUSG00000024091	VAPA	37	48	63	48	40	44	448	380	269	349	244	242
ENSMUSG00000024095	HNRNPLL	7	13	8	8	9	6	46	50	33	54	39	25
ENSMUSG00000024096	RALBP1	46	72	65	85	57	86	302	308	227	339	215	273
ENSMUSG00000024097	SRSF7	16	18	13	20	15	24	186	165	114	181	111	119
ENSMUSG00000024098	TWSG1	6	4	7	8	12	6	31	28	20	42	24	27
ENSMUSG00000024099	NDUFV2	17	21	29	25	29	26	134	137	110	143	100	140
ENSMUSG00000024101	WASHC1	2	7	12	9	4	5	41	44	34	54	43	36
ENSMUSG00000024104	WASHC2	13	17	16	15	16	7	64	60	43	95	56	57
ENSMUSG00000024118	TEDC2	4	4	1	6	4	4	17	20	15	20	11	29
ENSMUSG00000024120	LRPPRC	5	8	4	3	5	4	34	34	14	39	26	34
ENSMUSG00000024121	ATP6V0C	1	0	0	0	0	0	1	0	1	2	0	0
ENSMUSG00000024122	PDPK1	62	54	52	84	100	60	300	288	229	380	221	269
ENSMUSG00000024124	PRSS30	0	0	1	0	0	0	0	0	0	0	0	0
ENSMUSG00000024127	PREPL	1	1	0	1	0	0	4	7	1	2	3	3
ENSMUSG00000024130	ABCA3	0	0	0	0	0	0	0	1	0	1	1	1
ENSMUSG00000024131	SLC3A1	0	0	0	1	0	0	2	2	1	2	1	1
ENSMUSG00000024132	ECI1	6	1	8	6	10	5	37	41	46	39	38	38
ENSMUSG00000024135	SRBD1	0	2	1	2	2	1	4	7	2	5	5	5
ENSMUSG00000024136	DNASE1L2	0	0	1	0	2	0	0	1	0	0	0	3
ENSMUSG00000024137	E4F1	6	6	0	6	13	6	33	35	35	26	31	20
ENSMUSG00000024142	MLST8	1	1	0	0	2	4	13	8	15	7	4	5
ENSMUSG00000024143	RHOQ	1	2	2	2	2	0	8	7	6	9	7	10
ENSMUSG00000024145	PIGF	3	4	1	2	2	3	8	12	5	12	5	11
ENSMUSG00000024146	CRIPT	0	0	2	2	1	2	8	10	9	7	11	7
ENSMUSG00000024150	MCFD2	5	2	2	4	4	5	19	22	19	19	23	11
ENSMUSG00000024151	MSH2	4	5	4	4	3	5	33	37	25	34	23	30
ENSMUSG00000024160	SPSB3	1	0	2	1	1	1	6	10	5	7	9	8
ENSMUSG00000024163	MAPK8IP3	26	30	29	30	40	42	149	158	127	122	96	102
ENSMUSG00000024165	JPT2	6	2	11	9	9	11	36	43	36	44	26	35
ENSMUSG00000024169	IFT140	1	2	0	0	0	2	7	11	4	4	4	9
ENSMUSG00000024170	TELO2	5	13	7	10	8	7	31	53	33	43	30	45
ENSMUSG00000024174	POT1B	3	6	2	2	8	5	17	25	16	23	12	22
ENSMUSG00000024176	SOX8	0	2	0	1	0	0	0	0	0	0	0	0
ENSMUSG00000024177	NME4	0	0	0	1	2	2	3	6	5	4	4	12
ENSMUSG00000024180	TMEM8	1	1	0	1	1	1	3	11	3	1	3	8
ENSMUSG00000024181	MRPL28	13	20	18	11	24	20	108	118	93	101	93	93
ENSMUSG00000024182	AXIN1	8	5	6	6	14	8	38	29	32	34	38	39

ENSMUSG00000024184	PDIA2	0	0	0	0	1	0	0	0	0	0	0	0
ENSMUSG00000024186	RGS11	0	0	0	1	0	1	1	0	3	1	0	1
ENSMUSG00000024187	FAM234A	2	2	0	2	1	1	8	11	8	8	2	9
ENSMUSG00000024188	LUC7L	46	38	47	62	62	45	265	284	231	321	245	264
ENSMUSG00000024190	DUSP1	2	5	1	1	3	7	12	15	9	19	9	9
ENSMUSG00000024191	BNIP1	7	4	5	4	8	5	24	21	16	18	28	17
ENSMUSG00000024193	PHF1	3	4	2	2	2	3	12	12	10	12	6	13
ENSMUSG00000024194	CUTA	12	11	13	15	13	12	79	75	56	88	55	68
ENSMUSG00000024197	PLIN3	19	30	37	35	29	34	125	109	127	119	98	99
ENSMUSG00000024201	KDM4B	3	1	1	3	5	2	19	18	14	16	13	15
ENSMUSG00000024205	RPL36-PS2	12	7	11	3	10	10	42	44	35	46	35	49
ENSMUSG00000024206	RFX2	0	0	0	0	0	0	0	3	0	2	2	3
ENSMUSG00000024208	UQCC2	23	14	21	13	12	22	131	116	85	122	89	101
ENSMUSG00000024212	MLLT1	4	3	5	4	4	8	31	24	25	27	15	24
ENSMUSG00000024213	NUDT3	21	17	14	24	19	19	100	99	65	101	65	65
ENSMUSG00000024215	SPDEF	0	0	0	0	0	0	0	0	0	0	1	0
ENSMUSG00000024217	SNRPC	1	7	3	5	3	2	16	13	13	9	12	21
ENSMUSG00000024218	TAF11	0	4	1	5	2	1	13	14	3	15	3	9
ENSMUSG00000024219	ANKS1	0	2	0	1	5	3	9	5	3	7	9	4
ENSMUSG00000024220	ZFP523	1	1	1	2	3	1	3	5	5	6	3	8
ENSMUSG00000024222	FKBP5	7	11	8	5	11	10	60	63	45	54	46	51
ENSMUSG00000024231	CUL2	8	12	7	11	6	11	53	44	41	53	50	46
ENSMUSG00000024234	MTPAP	3	5	4	2	3	3	22	17	16	12	17	17
ENSMUSG00000024235	MAP3K8	0	1	3	3	2	1	15	14	6	8	5	10
ENSMUSG00000024236	SVIL	2	4	2	3	5	3	19	21	5	13	11	16
ENSMUSG00000024238	ZEB1	8	10	12	17	16	5	47	33	33	43	49	40
ENSMUSG00000024240	EPC1	4	5	2	2	5	5	22	24	20	40	31	35
ENSMUSG00000024241	SOS1	5	7	4	3	4	3	14	16	13	19	10	15
ENSMUSG00000024242	MAP4K3	0	0	0	0	0	0	1	4	2	2	1	1
ENSMUSG00000024246	THUMPD2	1	1	2	1	0	0	2	3	2	3	2	2
ENSMUSG00000024248	COX7A2L	4	8	7	14	8	12	40	35	40	50	30	43
ENSMUSG00000024251	THADA	0	2	0	1	1	3	5	10	4	5	6	7
ENSMUSG00000024253	DYNC2LI1	0	1	0	1	0	1	0	4	0	3	0	2
ENSMUSG00000024258	POLR2D	3	8	5	7	5	8	50	60	38	44	36	33
ENSMUSG00000024259	SLC25A46	3	4	9	3	11	4	37	42	23	47	27	33
ENSMUSG00000024260	SAP130	0	5	1	7	2	1	17	15	14	12	11	13
ENSMUSG00000024261	SYT4	0	0	0	0	0	0	1	0	0	0	0	0
ENSMUSG00000024268	CELF4	0	0	0	1	0	0	2	0	0	0	1	0
ENSMUSG00000024269	TPGS2	2	1	2	5	3	3	27	29	5	20	16	26
ENSMUSG00000024270	SLC39A6	28	48	24	29	25	29	180	181	106	174	130	140
ENSMUSG00000024271	ELP2	16	19	14	24	14	9	89	95	80	111	61	86
ENSMUSG00000024273	2700062C07F	8	5	4	11	7	4	20	24	19	23	24	36
ENSMUSG00000024274	ZSCAN30	0	0	1	0	1	0	0	0	1	2	1	2
ENSMUSG00000024276	ZFP397	6	10	12	11	2	10	42	41	30	51	26	31
ENSMUSG00000024277	MAPRE2	10	9	18	17	26	10	88	95	103	106	66	85
ENSMUSG00000024283	WAC	8	6	5	11	8	10	54	52	43	65	39	41
ENSMUSG00000024286	CCNY	10	7	11	7	9	7	49	54	37	53	38	47
ENSMUSG00000024287	THOC1	14	24	18	32	27	18	85	110	73	103	83	80
ENSMUSG00000024290	ROCK1	63	87	60	118	78	85	350	375	268	415	257	304
ENSMUSG00000024293	ESCO1	7	7	3	15	11	6	49	34	22	37	15	21
ENSMUSG00000024294	MIB1	3	10	19	16	18	10	70	69	65	99	49	53
ENSMUSG00000024295	CYP4F41-PS	0	0	0	0	0	1	4	5	5	1	2	2
ENSMUSG00000024298	ZFP871	6	17	10	18	17	19	64	53	41	69	35	41
ENSMUSG00000024299	ADAMTS10	17	16	29	28	23	16	77	103	75	89	63	71
ENSMUSG00000024300	MYO1F	7	5	3	5	5	4	27	33	25	35	20	21
ENSMUSG00000024301	KIFC5B	1	9	5	5	6	4	24	24	19	21	30	21
ENSMUSG00000024302	DTNA	0	0	0	1	0	1	1	1	4	2	4	2
ENSMUSG00000024304	CDH2	0	1	0	1	0	0	0	0	0	0	0	0

ENSMUSG00000024308	TAPBP	6	7	9	10	12	10	40	46	36	52	45	48
ENSMUSG00000024309	PFDN6	32	19	29	36	27	30	178	178	169	156	157	152
ENSMUSG00000024312	WDR46	9	8	13	6	10	11	82	95	57	74	81	75
ENSMUSG00000024313	MEP1B	0	0	0	0	0	0	0	0	1	0	0	0
ENSMUSG00000024317	RNF138	18	18	27	23	20	20	119	135	77	148	77	99
ENSMUSG00000024319	VPS52	2	4	2	7	1	6	19	15	26	21	20	13
ENSMUSG00000024325	RING1	0	0	1	0	0	1	8	3	7	2	7	8
ENSMUSG00000024327	SLC39A7	0	1	0	0	0	0	0	0	1	0	2	0
ENSMUSG00000024330	COL11A2	2	0	1	0	0	0	1	0	0	1	0	0
ENSMUSG00000024335	BRD2	64	85	70	106	62	51	286	350	252	344	243	285
ENSMUSG00000024338	PSMB8	2	15	16	19	13	5	71	76	57	58	48	52
ENSMUSG00000024339	TAP2	5	1	2	10	3	10	33	30	15	20	16	23
ENSMUSG00000024346	PFDN1	13	14	10	17	13	13	84	99	83	99	51	79
ENSMUSG00000024347	PSD2	0	0	0	0	0	0	0	0	0	0	0	1
ENSMUSG00000024349	TMEM173	5	7	13	10	10	11	40	55	30	54	37	54
ENSMUSG00000024350	DNAJC18	3	2	1	4	1	2	5	11	4	3	4	8
ENSMUSG00000024352	SPATA24	1	0	1	2	0	1	4	3	2	3	1	2
ENSMUSG00000024354	SLC23A1	0	0	0	0	0	0	0	1	0	0	0	0
ENSMUSG00000024357	SIL1	4	3	8	6	2	4	21	14	8	19	13	15
ENSMUSG00000024359	HSPA9	21	30	18	32	34	31	209	189	135	203	157	169
ENSMUSG00000024360	ETF1	32	31	41	43	33	41	234	246	172	222	186	195
ENSMUSG00000024369	NELFE	2	2	7	6	4	6	42	39	23	34	21	35
ENSMUSG00000024370	CDC23	4	4	4	6	4	8	31	44	33	44	31	28
ENSMUSG00000024371	C2	1	0	0	0	0	0	0	0	1	0	0	0
ENSMUSG00000024376	EPB41L4A	0	0	2	0	0	0	2	3	0	2	2	1
ENSMUSG00000024378	STARD4	11	17	15	12	16	17	110	139	81	111	75	66
ENSMUSG00000024381	BIN1	0	0	0	0	0	0	1	1	1	0	0	0
ENSMUSG00000024382	ERCC3	4	2	1	4	3	1	16	15	11	16	9	23
ENSMUSG00000024383	MAP3K2	1	5	4	5	12	6	20	20	17	35	22	27
ENSMUSG00000024384	IWS1	14	20	10	16	14	18	63	67	43	92	44	60
ENSMUSG00000024387	CSNK2B	14	27	28	31	29	35	157	169	141	157	137	169
ENSMUSG00000024391	APOM	0	0	0	0	0	0	0	1	0	0	0	0
ENSMUSG00000024392	BAG6	29	31	38	58	32	40	152	183	139	181	109	149
ENSMUSG00000024393	PRRC2A	30	34	41	52	37	41	199	226	173	189	163	180
ENSMUSG00000024399	LTB	11	19	22	16	19	15	19	22	8	10	10	18
ENSMUSG00000024400	WDR33	10	7	8	11	4	7	27	30	30	38	26	27
ENSMUSG00000024401	TNF	2	2	3	4	3	1	3	0	4	2	2	4
ENSMUSG00000024402	LTA	2	2	1	1	1	4	7	2	3	4	1	6
ENSMUSG00000024403	ATP6V1G2	2	0	0	0	0	1	1	3	2	3	2	4
ENSMUSG00000024404	RIOK3	7	7	12	11	11	12	72	55	46	80	39	55
ENSMUSG00000024409	PSORS1C2	0	0	0	0	0	0	0	0	0	1	0	1
ENSMUSG00000024410	3110002H16I	5	6	9	5	7	6	41	31	24	33	17	26
ENSMUSG00000024413	NPC1	4	3	4	3	5	1	15	12	9	13	10	11
ENSMUSG00000024414	MRPL27	0	4	5	2	6	1	23	31	22	25	16	15
ENSMUSG00000024422	DHX16	7	7	6	9	5	7	44	34	35	49	27	39
ENSMUSG00000024423	IMPACT	1	3	3	3	0	4	19	19	24	29	14	24
ENSMUSG00000024425	NDFIP1	9	8	11	6	14	7	47	55	36	87	34	48
ENSMUSG00000024426	ATAT1	0	1	1	1	0	0	4	7	3	1	7	2
ENSMUSG00000024427	SPRY4	22	25	29	33	26	16	108	108	89	128	81	123
ENSMUSG00000024429	GNL1	12	6	11	19	10	5	51	54	48	46	43	49
ENSMUSG00000024431	NR3C1	8	16	5	16	8	17	59	54	47	51	37	39
ENSMUSG00000024436	MRPS18B	0	0	0	0	1	0	1	1	0	0	2	3
ENSMUSG00000024442	0610009O20I	3	6	10	4	6	5	31	29	32	26	21	21
ENSMUSG00000024446	RPP21	10	9	11	18	22	19	82	80	60	79	58	84
ENSMUSG00000024454	HDAC3	13	21	15	22	13	22	57	81	66	79	55	77
ENSMUSG00000024456	DIAPH1	15	24	8	13	17	18	71	80	67	63	69	63
ENSMUSG00000024457	TRIM26	4	9	4	9	9	5	33	27	25	31	24	34
ENSMUSG00000024459	H2-M5	0	0	0	2	0	1	2	2	0	2	0	2

ENSMUSG00000024462	GABBR1	7	12	7	8	15	11	28	39	27	38	18	16
ENSMUSG00000024471	MYOT	0	0	0	0	0	0	1	0	1	0	0	0
ENSMUSG00000024472	DCP2	4	7	11	14	11	3	20	32	24	40	23	30
ENSMUSG00000024474	IK	18	16	16	22	23	22	130	126	90	135	99	102
ENSMUSG00000024477	PGGT1B	7	7	3	8	12	9	27	22	15	26	11	26
ENSMUSG00000024480	AP3S1	0	0	2	2	2	1	16	6	7	19	7	8
ENSMUSG00000024483	ANKHD1	21	32	20	29	24	29	110	121	71	125	68	91
ENSMUSG00000024486	HBEGF	0	1	0	0	0	0	0	0	0	0	0	1
ENSMUSG00000024487	YIPF5	2	0	3	5	3	3	24	35	21	37	13	29
ENSMUSG00000024491	RBM27	5	16	10	11	10	15	60	53	36	47	47	50
ENSMUSG00000024493	LARS	9	12	12	6	7	6	39	45	35	42	38	38
ENSMUSG00000024498	TCERG1	29	28	25	49	35	25	172	162	108	171	149	136
ENSMUSG00000024501	DPYSL3	0	0	0	0	0	0	0	0	0	0	0	1
ENSMUSG00000024502	JAKMIP2	1	1	3	3	2	1	7	6	8	13	7	6
ENSMUSG00000024505	DTWD2	2	2	1	0	3	0	8	11	8	5	5	3
ENSMUSG00000024507	HSD17B4	3	3	4	7	5	3	34	47	37	35	21	34
ENSMUSG00000024511	RAB27B	0	0	0	0	1	0	0	0	0	1	0	0
ENSMUSG00000024513	MBD2	10	12	23	20	24	12	100	116	91	128	89	86
ENSMUSG00000024515	SMAD4	10	8	4	8	11	13	30	46	27	42	25	26
ENSMUSG00000024516	SEC11C	33	54	54	53	64	64	251	313	226	298	260	219
ENSMUSG00000024524	GNAL	0	2	1	1	2	1	4	3	3	4	5	1
ENSMUSG00000024525	IMPA2	1	0	6	3	6	3	27	23	24	20	27	20
ENSMUSG00000024527	AFG3L2	5	5	4	8	7	9	49	42	28	36	27	25
ENSMUSG00000024528	SRFBP1	5	4	12	11	8	6	37	33	23	41	36	30
ENSMUSG00000024529	LOX	0	0	0	0	0	0	0	0	0	0	0	1
ENSMUSG00000024530	PRELID3A	1	1	1	1	1	2	6	11	3	9	6	7
ENSMUSG00000024533	SPIRE1	3	1	5	7	15	4	40	33	29	36	30	26
ENSMUSG00000024534	SNCAIP	0	0	2	0	0	0	5	4	2	4	3	5
ENSMUSG00000024535	SNX24	0	0	0	1	3	0	11	4	0	8	6	8
ENSMUSG00000024537	PSMG2	8	11	6	2	8	9	40	47	24	49	22	42
ENSMUSG00000024538	PPIC	14	14	19	28	19	15	111	121	97	123	94	92
ENSMUSG00000024539	PTPN2	5	12	6	17	13	12	89	81	70	96	58	67
ENSMUSG00000024542	CEP192	11	18	13	11	19	8	72	73	55	72	66	67
ENSMUSG00000024544	LDLRAD4	2	9	1	2	3	2	15	9	7	15	11	9
ENSMUSG00000024548	SETBP1	0	0	0	0	0	0	0	0	0	0	0	1
ENSMUSG00000024553	GALR1	0	0	0	0	0	0	0	0	0	0	1	0
ENSMUSG00000024556	ME2	15	15	9	6	9	12	62	64	43	47	40	55
ENSMUSG00000024560	CXXC1	2	9	8	3	13	5	31	30	24	35	18	31
ENSMUSG00000024561	MBD1	3	5	9	9	9	6	25	49	29	42	22	21
ENSMUSG00000024563	SMAD2	2	7	2	1	1	3	25	22	6	14	18	14
ENSMUSG00000024565	SALL3	0	1	3	0	0	1	1	3	0	1	0	2
ENSMUSG00000024566	ATP9B	8	2	8	5	6	8	31	29	13	25	19	21
ENSMUSG00000024570	RBFA	5	4	6	11	10	9	53	48	33	43	41	25
ENSMUSG00000024571	GM16286	17	12	16	20	14	15	86	86	88	112	84	79
ENSMUSG00000024575	PDE6A	1	0	1	1	0	1	1	2	0	2	2	0
ENSMUSG00000024576	CSNK1A1	33	38	31	54	31	31	214	200	166	278	159	155
ENSMUSG00000024579	PCYOX1L	35	36	32	42	40	39	122	147	99	156	116	152
ENSMUSG00000024580	GRPEL2	4	2	2	3	3	6	10	16	20	12	19	18
ENSMUSG00000024581	NAPG	3	9	5	3	10	10	48	42	34	52	28	34
ENSMUSG00000024583	TXNL1	19	18	15	28	19	24	135	157	111	135	114	113
ENSMUSG00000024587	NARS	31	48	37	47	49	45	220	228	169	261	158	182
ENSMUSG00000024588	FECH	2	0	0	1	0	0	4	4	1	2	3	3
ENSMUSG00000024589	NEDD4L	0	0	0	0	0	0	0	1	0	0	0	0
ENSMUSG00000024590	LMNB1	23	22	33	46	28	25	176	179	135	206	164	166
ENSMUSG00000024593	MEGF10	3	4	1	4	1	1	14	8	14	11	14	8
ENSMUSG00000024594	PRRC1	8	6	10	5	11	9	47	54	38	37	26	48
ENSMUSG00000024597	SLC12A2	2	5	4	3	4	5	32	14	20	24	23	29
ENSMUSG00000024601	ISOC1	2	3	3	7	2	6	37	25	17	36	14	23

ENSMUSG00000024603	DCTN4	15	15	18	17	20	18	70	80	56	87	67	76
ENSMUSG00000024604	RBM22	6	5	11	9	6	12	68	53	55	69	41	44
ENSMUSG00000024608	RPS14	208	257	248	305	261	272	1686	1710	1273	1544	1276	1559
ENSMUSG00000024610	CD74	0	1	0	0	0	0	0	0	0	0	0	0
ENSMUSG00000024613	TCOF1	20	23	21	27	29	26	134	112	104	134	91	114
ENSMUSG00000024614	TMX3	0	0	0	0	0	0	0	0	0	0	1	0
ENSMUSG00000024621	CSF1R	0	0	0	0	0	1	1	6	3	4	1	2
ENSMUSG00000024622	HMGXB3	5	4	5	9	6	0	21	33	15	26	20	20
ENSMUSG00000024639	GNAQ	0	0	0	1	0	0	1	1	1	0	0	1
ENSMUSG00000024640	PSAT1	18	15	12	15	22	18	107	93	71	102	60	74
ENSMUSG00000024642	TLE4	7	13	8	9	6	10	64	55	38	57	36	42
ENSMUSG00000024644	CNDP2	4	3	0	7	3	2	17	17	18	14	9	18
ENSMUSG00000024645	TIMM21	7	3	2	1	3	4	21	24	21	26	15	20
ENSMUSG00000024646	CYB5A	9	7	5	4	4	4	25	28	17	33	28	29
ENSMUSG00000024654	ASRGL1	5	4	4	5	8	5	25	27	28	33	28	22
ENSMUSG00000024659	ANXA1	5	5	5	1	6	5	30	26	18	29	20	21
ENSMUSG00000024660	INCENP	27	35	14	49	24	20	113	115	116	149	118	142
ENSMUSG00000024661	FTH1	16	22	16	29	23	26	152	135	125	127	125	112
ENSMUSG00000024665	FADS2	2	3	4	6	6	4	32	31	13	32	20	22
ENSMUSG00000024666	TMEM138	2	1	0	3	2	3	11	15	13	9	8	10
ENSMUSG00000024667	TMEM216	1	1	3	2	1	0	2	8	3	5	1	6
ENSMUSG00000024668	SDHAF2	6	6	4	6	2	6	35	30	32	40	18	36
ENSMUSG00000024669	CD5	0	0	0	0	0	0	0	0	0	1	0	0
ENSMUSG00000024675	MS4A4C	0	0	0	0	0	0	0	1	0	0	0	0
ENSMUSG00000024677	MS4A6B	1	0	0	0	0	0	2	3	1	0	0	1
ENSMUSG00000024678	MS4A4D	0	0	0	0	0	0	0	0	0	1	0	0
ENSMUSG00000024683	MRPL16	4	3	2	2	3	4	18	16	8	23	23	21
ENSMUSG00000024687	OSBP	2	10	4	8	5	10	45	48	39	41	34	23
ENSMUSG00000024691	FAM111A	24	31	38	40	37	30	229	223	159	232	148	166
ENSMUSG00000024695	ZFP91	41	37	37	63	40	49	181	166	123	199	133	166
ENSMUSG00000024696	LPXN	0	0	0	0	0	0	3	1	1	1	2	1
ENSMUSG00000024697	GNA14	0	0	0	1	0	0	0	0	0	0	0	0
ENSMUSG00000024712	RFK	22	30	25	43	45	28	107	132	106	115	90	112
ENSMUSG00000024725	OSTF1	8	9	5	3	9	6	55	32	36	42	34	40
ENSMUSG00000024726	CARNMT1	2	3	1	3	3	5	8	13	15	12	11	15
ENSMUSG00000024727	TRPM6	0	0	0	0	0	0	0	0	1	0	0	1
ENSMUSG00000024732	CCDC86	0	0	3	5	3	0	6	8	4	11	5	10
ENSMUSG00000024735	PRPF19	14	21	17	21	22	21	114	117	85	89	88	90
ENSMUSG00000024736	TMEM132A	0	0	0	0	0	0	0	1	0	0	0	0
ENSMUSG00000024740	DDB1	15	30	34	28	31	26	118	128	119	143	97	126
ENSMUSG00000024742	FEN1	12	15	8	15	16	14	87	92	63	108	62	68
ENSMUSG00000024747	ALDH1A7	0	0	0	0	0	0	1	0	0	0	0	0
ENSMUSG00000024750	ZFAND5	14	13	14	15	16	15	87	91	61	94	74	65
ENSMUSG00000024754	TMEM2	10	8	8	6	5	11	47	32	20	32	29	37
ENSMUSG00000024758	RTN3	24	35	37	24	33	25	130	156	113	196	113	141
ENSMUSG00000024759	ATL3	8	8	5	14	5	12	38	43	31	38	43	51
ENSMUSG00000024764	NAA40	5	8	11	10	9	9	53	52	44	49	36	45
ENSMUSG00000024767	OTUB1	21	21	20	17	23	22	87	86	58	76	73	81
ENSMUSG00000024769	CDC42BPG	0	0	0	0	0	0	1	0	0	0	1	0
ENSMUSG00000024772	EHD1	1	4	3	3	5	3	17	13	13	17	9	19
ENSMUSG00000024773	ATG2A	4	4	1	3	4	3	28	23	26	30	18	16
ENSMUSG00000024774	ANKRD22	0	0	0	1	0	0	0	0	0	0	0	0
ENSMUSG00000024776	STAMBPL1	17	11	14	18	13	16	63	61	54	71	56	54
ENSMUSG00000024777	PPP2R5B	1	1	0	2	1	2	4	5	2	0	1	5
ENSMUSG00000024778	FAS	0	0	0	0	0	0	1	2	1	1	0	0
ENSMUSG00000024780	CDC37L1	7	4	6	12	5	8	45	44	24	57	35	39
ENSMUSG00000024781	LIPA	4	1	3	2	2	3	12	14	10	18	13	4
ENSMUSG00000024782	AK3	0	4	4	2	4	1	21	27	18	16	21	18

ENSMUSG00000024784	GPHA2	0	0	0	0	0	0	1	0	0	0	0	0
ENSMUSG00000024785	RCL1	4	10	6	7	9	7	40	34	35	40	26	24
ENSMUSG00000024787	SNX15	4	3	3	0	1	1	6	2	5	6	5	7
ENSMUSG00000024789	JAK2	4	11	10	5	2	3	38	35	29	29	29	36
ENSMUSG00000024790	SAC3D1	2	3	2	3	5	2	13	16	12	17	17	17
ENSMUSG00000024791	CDCA5	2	3	2	8	4	2	20	20	23	23	19	21
ENSMUSG00000024792	ZFPL1	0	4	1	2	0	0	11	10	7	6	5	10
ENSMUSG00000024793	TNFRSF25	0	0	0	0	0	0	1	0	1	0	0	2
ENSMUSG00000024795	KIF20B	22	38	29	41	41	45	190	179	106	217	140	125
ENSMUSG00000024797	VPS51	1	2	0	1	0	0	9	4	3	5	5	6
ENSMUSG00000024799	TM7SF2	1	0	0	0	0	1	4	3	6	3	4	4
ENSMUSG00000024800	RPP30	8	8	9	6	2	9	44	49	38	42	38	48
ENSMUSG00000024805	PCGF5	4	4	5	5	4	8	14	13	13	21	11	15
ENSMUSG00000024807	SYVN1	7	13	5	6	12	7	66	42	44	42	32	53
ENSMUSG00000024811	TNKS2	9	9	15	16	14	17	87	95	78	94	79	65
ENSMUSG00000024812	TJP2	0	0	1	1	0	0	0	0	0	0	1	0
ENSMUSG00000024816	FRMD8	4	2	4	0	0	4	4	8	13	3	9	7
ENSMUSG00000024817	UHRF2	6	15	11	19	11	16	80	69	38	79	52	39
ENSMUSG00000024824	RAD9A	10	18	17	10	16	10	73	59	66	68	61	76
ENSMUSG00000024826	DPF2	6	12	6	10	6	6	40	45	33	36	31	40
ENSMUSG00000024829	MRPL21	6	7	12	8	12	11	69	64	38	92	51	56
ENSMUSG00000024830	RPS6KB2	17	16	22	23	32	28	137	168	116	145	115	135
ENSMUSG00000024831	IGHMBP2	7	7	8	13	13	13	54	49	50	63	37	36
ENSMUSG00000024833	POLA2	13	15	11	14	15	20	67	78	44	49	50	64
ENSMUSG00000024835	CORO1B	0	0	0	1	0	0	2	0	2	0	2	4
ENSMUSG00000024837	DMRT1	0	0	0	1	0	0	0	0	0	0	0	0
ENSMUSG00000024841	EIF1AD	6	11	4	6	9	11	41	39	34	50	26	35
ENSMUSG00000024842	CABP4	0	0	0	0	0	0	0	0	1	0	0	0
ENSMUSG00000024843	CHKA	15	27	21	34	15	11	63	64	45	81	50	58
ENSMUSG00000024844	BANF1	8	9	7	13	10	16	60	72	60	69	54	61
ENSMUSG00000024845	TMEM134	5	5	4	6	5	3	22	27	20	21	16	27
ENSMUSG00000024846	CST6	0	0	0	0	0	0	0	0	0	0	1	0
ENSMUSG00000024847	AIP	9	4	4	7	6	2	34	31	22	37	29	28
ENSMUSG00000024851	PITPNM1	20	8	30	17	29	22	61	66	56	80	38	53
ENSMUSG00000024853	SF3B2	28	39	41	46	45	27	186	181	144	218	149	162
ENSMUSG00000024854	POLD4	2	3	5	4	1	1	11	11	12	15	8	8
ENSMUSG00000024855	PACS1	4	7	5	2	3	4	17	20	19	14	17	9
ENSMUSG00000024856	CDK2AP2	1	1	7	7	8	5	24	28	20	25	25	23
ENSMUSG00000024858	GRK2	22	13	21	27	24	17	97	128	93	114	74	91
ENSMUSG00000024862	KLC2	2	1	0	1	3	0	6	9	4	3	5	8
ENSMUSG00000024867	PIP5K1B	2	1	0	2	2	3	5	11	6	6	9	9
ENSMUSG00000024869	NUDT8	0	0	0	0	0	0	0	1	0	0	0	0
ENSMUSG00000024870	RAB1B	4	5	1	8	4	5	19	18	23	22	16	24
ENSMUSG00000024871	DOC2G	0	0	0	0	0	0	0	1	1	0	0	1
ENSMUSG00000024873	CNIH2	0	0	0	0	0	0	1	1	0	2	2	0
ENSMUSG00000024875	YIF1A	2	2	2	1	3	2	15	20	14	14	7	12
ENSMUSG00000024878	CBWD1	6	4	3	2	2	1	29	18	9	26	10	21
ENSMUSG00000024883	RIN1	0	0	0	0	0	0	0	0	0	2	0	0
ENSMUSG00000024887	ASAH2	0	0	0	1	0	0	3	2	3	6	0	2
ENSMUSG00000024889	RCE1	1	1	3	0	1	1	11	11	15	11	6	5
ENSMUSG00000024891	SLC29A2	0	0	0	0	0	0	1	2	0	0	0	3
ENSMUSG00000024892	PCX	0	4	1	0	2	1	7	6	8	4	1	8
ENSMUSG00000024896	MINPP1	8	8	12	21	17	15	57	61	45	61	42	62
ENSMUSG00000024897	APBA1	0	0	1	0	0	0	0	0	0	0	0	0
ENSMUSG00000024900	CPT1A	3	0	1	3	1	1	13	4	9	9	6	6
ENSMUSG00000024902	MRPL11	3	9	3	7	4	5	40	29	26	40	21	31
ENSMUSG00000024906	MUS81	2	1	7	2	1	1	9	6	5	7	6	7
ENSMUSG00000024908	PPP6R3	6	10	6	11	8	10	30	37	20	46	28	36

ENSMUSG00000024909	EFEMP2	0	0	0	0	0	2	2	0	0	0	1	0
ENSMUSG00000024910	CTSW	0	0	0	0	1	1	1	5	2	1	2	0
ENSMUSG00000024911	FIBP	1	4	1	3	1	0	6	14	7	9	7	8
ENSMUSG00000024912	FOSL1	0	0	0	0	0	0	0	0	0	1	0	0
ENSMUSG00000024913	LRP5	0	0	0	0	0	0	1	0	0	1	0	1
ENSMUSG00000024914	DRAP1	10	17	15	13	7	20	74	63	47	58	58	63
ENSMUSG00000024921	SMARCA2	11	10	10	12	21	8	51	63	46	82	60	53
ENSMUSG00000024922	OVOL1	0	0	0	0	0	0	0	0	0	0	1	0
ENSMUSG00000024925	RNASEH2C	7	20	10	27	14	18	107	122	80	109	84	122
ENSMUSG00000024926	KAT5	4	1	3	2	5	1	12	19	17	13	15	17
ENSMUSG00000024927	RELA	3	4	12	10	3	1	19	27	9	20	26	21
ENSMUSG00000024935	SLC1A1	0	0	0	0	0	0	0	0	0	0	0	1
ENSMUSG00000024937	EHBP1L1	2	2	3	3	2	3	10	13	9	15	11	6
ENSMUSG00000024940	LTBP3	0	0	0	2	1	0	5	5	5	4	6	6
ENSMUSG00000024941	SCYL1	8	12	3	13	7	13	45	60	39	49	27	50
ENSMUSG00000024942	CAPN1	4	5	0	5	6	7	5	12	5	8	7	9
ENSMUSG00000024943	SMC5	32	62	34	84	56	40	232	225	192	267	161	191
ENSMUSG00000024944	ARL2	3	2	1	2	1	2	17	22	12	11	13	8
ENSMUSG00000024947	MEN1	5	9	5	11	6	7	21	30	27	16	14	30
ENSMUSG00000024948	MAP4K2	2	2	3	1	3	1	4	4	8	6	7	4
ENSMUSG00000024949	SF1	7	12	19	24	19	15	73	70	53	69	46	70
ENSMUSG00000024952	RPS6KA4	0	0	1	0	0	0	0	0	1	0	0	0
ENSMUSG00000024953	PRDX5	16	7	17	15	18	12	78	94	61	103	75	90
ENSMUSG00000024955	ESRRA	3	3	4	6	5	8	22	33	18	16	18	18
ENSMUSG00000024957	KCNK4	0	0	0	0	0	0	0	0	0	0	0	1
ENSMUSG00000024958	GPR137	2	1	0	0	1	0	2	0	1	0	3	2
ENSMUSG00000024959	BAD	2	3	1	3	3	1	10	11	9	7	13	5
ENSMUSG00000024960	PLCB3	5	3	4	1	5	6	12	12	17	16	12	7
ENSMUSG00000024962	VEGFB	7	8	6	9	9	6	33	41	31	38	26	34
ENSMUSG00000024963	DNAJC4	1	0	0	1	1	2	9	7	7	4	9	6
ENSMUSG00000024965	FERMT3	15	19	14	24	21	12	76	91	66	95	65	97
ENSMUSG00000024966	STIP1	12	11	21	21	18	15	118	130	105	127	86	101
ENSMUSG00000024968	RCOR2	0	0	0	1	0	1	2	8	1	6	2	3
ENSMUSG00000024969	MARK2	20	22	16	26	29	23	93	110	75	109	87	94
ENSMUSG00000024970	AI846148	1	2	2	3	1	1	10	9	9	6	3	12
ENSMUSG00000024974	SMC3	34	44	37	43	49	25	198	165	132	237	135	141
ENSMUSG00000024975	PDCD4	14	15	13	16	10	15	74	86	69	98	63	55
ENSMUSG00000024976	SHOC2	2	1	3	0	2	5	20	13	9	14	2	17
ENSMUSG00000024978	GPAM	3	1	2	3	3	1	6	8	6	9	10	6
ENSMUSG00000024981	ACSL5	9	14	20	12	19	12	71	64	44	77	58	47
ENSMUSG00000024982	ZDHHC6	4	2	5	5	1	2	18	17	10	13	11	11
ENSMUSG00000024983	VTI1A	5	1	2	2	3	1	10	9	7	9	10	9
ENSMUSG00000024985	TCF7L2	0	0	0	0	1	0	0	0	0	1	0	1
ENSMUSG00000024986	HHEX	0	0	0	0	0	0	1	0	0	0	0	0
ENSMUSG00000024989	CEP55	10	10	20	16	13	13	71	69	54	83	56	58
ENSMUSG00000024991	EIF3A	93	131	127	164	143	132	733	721	581	929	594	528
ENSMUSG00000024993	FAM45A	1	1	1	1	1	2	9	10	7	13	3	9
ENSMUSG00000024997	PRDX3	47	80	82	99	79	74	335	360	274	374	236	329
ENSMUSG00000024999	NOC3L	5	4	5	3	3	5	28	28	26	34	21	26
ENSMUSG00000025001	HELLS	15	33	24	46	31	41	184	146	114	186	106	107
ENSMUSG00000025002	CYP2C55	0	1	1	0	0	0	0	1	2	0	0	0
ENSMUSG00000025006	SORBS1	1	3	2	1	1	1	2	9	0	8	3	6
ENSMUSG00000025007	ALDH18A1	10	9	12	13	8	12	65	84	65	75	58	51
ENSMUSG00000025008	TCTN3	1	0	0	0	0	1	5	0	3	5	4	4
ENSMUSG00000025010	CCNJ	7	10	5	4	5	8	19	24	11	25	12	16
ENSMUSG00000025014	DNTT	0	0	0	1	0	0	1	1	2	0	0	0
ENSMUSG00000025016	TM9SF3	13	20	23	26	18	28	95	90	59	102	75	73
ENSMUSG00000025019	LCOR	1	1	4	10	1	4	15	7	11	19	12	12

ENSMUSG00000025020	SLIT1	0	0	0	0	0	0	1	0	1	2	0	0
ENSMUSG00000025024	SMNDC1	8	9	9	12	13	13	71	72	50	79	46	50
ENSMUSG00000025025	MXI1	0	0	0	0	2	1	2	2	1	5	2	2
ENSMUSG00000025026	ADD3	0	1	0	0	0	0	1	0	0	0	0	0
ENSMUSG00000025027	XPNPEP1	0	4	2	4	1	2	4	2	7	4	7	5
ENSMUSG00000025034	TRIM8	15	23	21	12	7	21	51	38	31	64	37	43
ENSMUSG00000025035	ARL3	0	3	0	4	4	3	26	16	15	14	22	13
ENSMUSG00000025036	SFXN2	4	3	4	6	3	2	22	21	8	25	13	15
ENSMUSG00000025040	FUNDC1	3	1	0	2	7	2	22	15	17	22	20	9
ENSMUSG00000025041	NT5C2	3	4	3	4	5	2	16	24	21	16	23	22
ENSMUSG00000025047	PDCD11	10	15	8	20	21	19	77	64	58	88	64	60
ENSMUSG00000025049	TAF5	1	7	11	7	4	8	36	32	26	46	16	28
ENSMUSG00000025050	PCGF6	3	8	9	7	3	2	47	52	38	41	34	41
ENSMUSG00000025059	GK	1	5	0	3	7	4	26	18	20	22	15	8
ENSMUSG00000025060	SLK	16	38	25	47	40	44	175	174	106	252	133	136
ENSMUSG00000025064	COL17A1	0	0	0	0	0	0	1	1	1	0	0	0
ENSMUSG00000025066	SFR1	33	40	47	57	57	37	328	508	273	364	232	258
ENSMUSG00000025076	CASP7	0	0	0	0	0	0	1	0	0	1	0	1
ENSMUSG00000025077	DCLRE1A	1	1	1	3	2	2	5	9	5	7	2	6
ENSMUSG00000025078	NHLRC2	5	1	6	7	0	4	21	21	12	19	12	14
ENSMUSG00000025085	ABLIM1	26	31	24	37	31	28	126	113	85	127	79	104
ENSMUSG00000025086	TRUB1	2	4	6	5	6	4	19	19	10	18	17	16
ENSMUSG00000025094	SLC18A2	1	1	0	0	1	1	6	0	2	6	3	2
ENSMUSG00000025102	3110040N11I	0	1	1	1	1	0	7	3	4	12	3	5
ENSMUSG00000025103	BTBD1	1	7	4	2	4	6	28	27	21	23	27	26
ENSMUSG00000025104	HDGFL3	0	0	1	0	0	0	0	0	0	0	0	0
ENSMUSG00000025130	P4HB	39	44	41	42	25	30	235	260	179	224	171	180
ENSMUSG00000025132	ARHGDIA	29	24	25	28	34	26	166	157	112	157	80	153
ENSMUSG00000025133	INTS4	5	4	6	2	7	8	31	35	17	24	16	22
ENSMUSG00000025134	ALYREF	80	98	97	133	111	123	635	552	429	655	410	484
ENSMUSG00000025135	ANAPC11	1	5	2	4	5	7	23	38	36	51	26	41
ENSMUSG00000025137	PCYT2	6	8	6	9	12	6	46	45	32	47	37	24
ENSMUSG00000025138	SIRT7	3	5	6	8	4	4	38	32	24	29	21	30
ENSMUSG00000025139	TOLLIP	6	6	4	8	6	7	18	20	22	24	25	24
ENSMUSG00000025140	PYCR1	0	0	0	0	0	0	3	2	1	4	1	3
ENSMUSG00000025142	ASPSCR1	6	3	2	6	2	8	12	5	11	12	7	12
ENSMUSG00000025144	CENPX	1	7	3	9	11	3	26	37	19	37	33	34
ENSMUSG00000025145	LRRC45	10	5	3	4	0	6	14	17	8	14	11	13
ENSMUSG00000025147	MOB2	3	4	6	3	7	2	18	22	28	19	20	25
ENSMUSG00000025153	FASN	11	12	20	18	22	15	102	128	117	120	87	124
ENSMUSG00000025154	ARHGAP19	2	4	5	3	5	6	18	24	16	19	15	19
ENSMUSG00000025155	DUS1L	7	13	4	6	7	5	33	25	16	18	24	34
ENSMUSG00000025156	GPS1	0	0	0	0	1	0	2	1	2	1	2	0
ENSMUSG00000025157	ZDHHC16	5	9	3	7	5	7	35	34	23	19	21	28
ENSMUSG00000025158	RFNG	2	5	3	1	4	2	13	14	5	9	7	6
ENSMUSG00000025159	MMS19	10	11	10	11	14	7	62	48	42	57	36	45
ENSMUSG00000025161	SLC16A3	0	0	0	0	0	0	0	0	0	0	0	1
ENSMUSG00000025162	CSNK1D	7	7	7	14	15	12	50	38	37	58	43	50
ENSMUSG00000025163	CD7	8	18	16	21	15	12	37	49	23	39	35	28
ENSMUSG00000025165	SECTM1A	11	1	5	6	6	2	24	21	27	15	23	30
ENSMUSG00000025169	OGFOD3	1	2	1	0	0	1	6	1	3	4	5	1
ENSMUSG00000025171	UBTD1	1	1	0	1	2	2	4	1	2	3	4	3
ENSMUSG00000025173	WDR45B	3	3	2	9	1	5	40	30	18	39	27	27
ENSMUSG00000025175	FN3K	1	0	0	0	0	0	0	0	0	0	0	0
ENSMUSG00000025176	HOGA1	1	0	0	0	1	0	2	0	0	0	0	0
ENSMUSG00000025178	PI4K2A	4	8	7	7	7	7	44	35	28	25	23	30
ENSMUSG00000025184	R3HCC1L	1	3	2	5	3	4	22	19	13	15	15	13
ENSMUSG00000025188	HPS1	2	1	0	2	4	2	8	7	2	4	4	7

ENSMUSG00000025190	GOT1	10	13	6	20	15	9	94	95	62	87	59	71
ENSMUSG00000025192	ENTPD7	8	10	8	14	7	3	26	33	11	25	12	17
ENSMUSG00000025193	CUTC	0	0	0	0	1	0	1	1	2	0	1	0
ENSMUSG00000025195	DNMBP	2	1	1	0	4	1	7	9	5	11	5	9
ENSMUSG00000025197	CYP2C23	0	0	0	0	0	0	0	0	0	1	0	0
ENSMUSG00000025198	ERLIN1	1	1	5	3	5	3	21	20	20	32	15	16
ENSMUSG00000025199	CHUK	10	5	8	13	10	5	54	60	42	77	33	35
ENSMUSG00000025200	CWF19L1	3	2	2	4	4	6	11	5	7	7	8	9
ENSMUSG00000025201	BLOC1S2-PS	0	0	0	0	0	0	0	0	0	0	1	0
ENSMUSG00000025203	SCD2	43	62	54	69	75	64	405	427	303	440	281	321
ENSMUSG00000025204	NDUFB8	10	12	14	8	14	6	69	63	45	45	44	42
ENSMUSG00000025207	SEMA4G	1	1	0	0	0	0	0	0	0	0	1	0
ENSMUSG00000025208	MRPL43	8	10	9	13	10	4	54	60	43	61	45	45
ENSMUSG00000025209	TWNK	1	2	6	3	1	1	11	13	12	12	19	12
ENSMUSG00000025212	SFXN3	0	1	2	1	1	2	4	5	3	3	7	8
ENSMUSG00000025213	KAZALD1	0	0	0	1	0	0	0	3	0	0	3	2
ENSMUSG00000025216	LBX1	2	5	3	0	3	3	6	5	2	5	3	7
ENSMUSG00000025217	BTRC	4	3	1	2	2	3	8	10	9	11	8	12
ENSMUSG00000025218	POLL	2	2	0	2	1	5	6	4	6	8	5	4
ENSMUSG00000025220	MGEA5	9	17	13	20	11	18	73	63	45	70	68	48
ENSMUSG00000025223	LDB1	9	20	13	12	19	15	75	99	70	119	62	72
ENSMUSG00000025224	GBF1	9	6	18	12	9	13	29	54	47	39	37	23
ENSMUSG00000025225	NFKB2	4	5	8	8	6	9	21	31	23	30	23	28
ENSMUSG00000025226	FBXL15	1	3	0	0	0	0	6	3	4	4	4	3
ENSMUSG00000025227	MFSD13A	0	0	0	3	0	0	2	0	0	0	0	0
ENSMUSG00000025228	ACTR1A	4	2	7	2	3	4	27	36	15	20	28	27
ENSMUSG00000025231	SUFU	4	4	0	0	2	4	16	25	12	21	11	18
ENSMUSG00000025234	ARIH1	4	20	14	14	20	11	66	60	48	64	56	80
ENSMUSG00000025235	BBS4	0	1	0	0	0	0	8	3	7	3	7	2
ENSMUSG00000025236	ADPGK	5	2	2	3	0	3	17	17	13	11	17	16
ENSMUSG00000025237	PARP6	2	1	3	2	3	3	7	12	6	8	1	9
ENSMUSG00000025239	LIMD1	22	22	16	23	28	29	102	118	90	111	104	76
ENSMUSG00000025240	SACM1L	6	8	11	10	8	7	37	39	35	51	37	33
ENSMUSG00000025241	FYCO1	3	2	2	3	0	2	2	9	5	2	5	4
ENSMUSG00000025243	SLC6A20B	6	7	4	5	3	4	8	8	7	13	4	7
ENSMUSG00000025245	LZTFL1	1	2	1	3	2	1	8	14	17	18	10	10
ENSMUSG00000025246	TBL1X	10	14	11	17	13	17	66	76	53	85	47	72
ENSMUSG00000025255	ZFHX4	0	1	0	0	0	0	0	2	0	1	0	0
ENSMUSG00000025257	RIBC1	0	1	0	1	0	0	0	1	0	2	0	2
ENSMUSG00000025260	HSD17B10	7	6	7	10	4	5	38	46	20	38	24	37
ENSMUSG00000025261	HUWE1	10	18	29	30	19	20	94	100	65	117	77	83
ENSMUSG00000025262	FAM120C	1	0	0	0	0	0	5	2	2	3	2	2
ENSMUSG00000025264	TSR2	4	1	4	6	4	3	21	20	14	21	16	15
ENSMUSG00000025265	FGD1	0	0	0	0	0	0	1	0	0	2	1	2
ENSMUSG00000025266	GNL3L	3	7	6	7	10	4	45	30	28	37	29	34
ENSMUSG00000025268	MAGED2	4	6	2	5	1	2	19	22	10	16	16	18
ENSMUSG00000025269	APEX2	2	0	0	2	0	1	6	3	4	5	6	6
ENSMUSG00000025271	PFKFB1	0	0	1	1	0	1	1	1	1	3	2	2
ENSMUSG00000025278	FLNB	1	3	4	2	1	1	13	12	8	13	12	16
ENSMUSG00000025279	DNASE1L3	0	0	0	0	0	0	0	0	0	1	0	0
ENSMUSG00000025280	POLR3A	0	3	2	2	3	1	9	10	5	17	12	7
ENSMUSG00000025283	SAT1	1	1	2	6	4	2	18	17	11	12	10	11
ENSMUSG00000025287	ACOT9	1	1	2	2	3	2	12	15	9	11	9	7
ENSMUSG00000025289	PRDX4	17	21	18	26	27	22	165	208	121	127	139	115
ENSMUSG00000025290	RPS24	173	255	181	278	267	238	1615	1679	1241	1805	1184	1328
ENSMUSG00000025314	PTPRJ	1	1	0	2	2	2	2	3	6	6	5	2
ENSMUSG00000025316	BANP	11	13	6	10	8	15	47	43	25	55	36	37
ENSMUSG00000025317	CAR5A	2	1	0	2	1	1	0	0	0	0	1	0

ENSMUSG00000025323	SP4	8	2	7	5	7	6	20	37	17	35	15	13
ENSMUSG00000025324	ATP10A	1	4	3	11	8	4	20	26	19	19	14	3
ENSMUSG00000025326	UBE3A	45	48	54	79	71	65	197	223	153	259	140	172
ENSMUSG00000025332	KDM5C	11	9	7	10	10	7	23	39	26	36	34	38
ENSMUSG00000025337	SBDS	11	11	21	22	13	25	104	131	84	125	73	88
ENSMUSG00000025340	RABGEF1	7	6	5	7	5	7	29	20	24	32	28	27
ENSMUSG00000025350	RDH5	0	1	0	0	0	0	0	0	0	0	0	0
ENSMUSG00000025351	CD63	0	0	0	0	0	0	0	1	1	0	0	0
ENSMUSG00000025352	GDF11	2	2	7	5	4	4	18	13	13	17	8	7
ENSMUSG00000025353	ORMDL2	1	4	3	0	5	5	24	23	11	19	13	23
ENSMUSG00000025354	DNAJC14	2	5	3	4	1	3	26	30	14	21	17	19
ENSMUSG00000025355	MMP19	1	0	0	1	0	0	2	2	0	1	0	1
ENSMUSG00000025357	DGKA	42	60	66	75	63	57	204	247	172	247	146	200
ENSMUSG00000025358	CDK2	17	32	32	35	34	20	138	138	104	130	106	141
ENSMUSG00000025359	PMEL	9	5	16	9	14	6	28	31	24	29	16	26
ENSMUSG00000025362	RPS26	228	249	282	316	284	244	1167	1311	1010	1314	992	1178
ENSMUSG00000025364	PA2G4	23	42	26	40	52	41	216	278	185	291	199	243
ENSMUSG00000025366	ESYT1	18	20	29	33	21	24	79	57	48	60	49	75
ENSMUSG00000025369	SMARCC2	8	12	11	20	13	11	56	67	43	66	53	48
ENSMUSG00000025371	CHMP6	0	2	2	1	2	3	14	11	6	8	11	6
ENSMUSG00000025372	BAIAP2	1	3	2	4	3	3	3	8	2	4	2	7
ENSMUSG00000025373	RNF41	1	4	3	3	5	0	15	10	12	10	16	4
ENSMUSG00000025374	NABP2	5	7	4	11	3	4	29	35	35	44	27	38
ENSMUSG00000025377	TEPSIN	3	2	1	0	2	2	5	4	7	7	1	8
ENSMUSG00000025381	CNPY2	6	6	7	5	7	7	42	49	43	32	34	36
ENSMUSG00000025383	IL23A	0	0	1	0	0	0	0	0	0	1	1	0
ENSMUSG00000025384	FAAP100	0	0	3	0	0	2	5	5	2	6	1	5
ENSMUSG00000025386	PDE6G	0	0	0	0	0	0	0	0	0	1	0	0
ENSMUSG00000025393	ATP5B	133	130	134	180	169	167	993	1120	783	1057	801	919
ENSMUSG00000025395	PRIM1	37	41	30	30	36	28	177	185	128	219	142	143
ENSMUSG00000025401	MYO1A	0	0	0	1	0	1	1	2	1	0	1	1
ENSMUSG00000025402	NAB2	1	1	4	2	1	1	5	4	2	5	4	4
ENSMUSG00000025403	SHMT2	13	26	28	31	27	26	116	152	86	122	87	120
ENSMUSG00000025404	R3HDM2	4	5	2	5	8	1	18	28	18	28	17	15
ENSMUSG00000025407	GLI1	0	1	0	0	2	2	6	14	3	5	10	5
ENSMUSG00000025408	DDIT3	9	7	6	8	5	8	28	30	33	27	28	39
ENSMUSG00000025409	MBD6	15	13	7	15	14	8	42	35	36	45	21	33
ENSMUSG00000025410	DCTN2	10	9	9	13	11	6	47	70	43	58	30	55
ENSMUSG00000025413	TTC4	3	6	3	4	8	4	19	20	20	24	17	36
ENSMUSG00000025417	PIP4K2C	4	0	5	3	3	6	19	10	8	14	16	18
ENSMUSG00000025420	KATNAL2	0	0	0	0	0	1	2	0	1	2	1	0
ENSMUSG00000025421	HDHD2	0	1	0	1	1	2	4	2	0	2	4	3
ENSMUSG00000025422	AGAP2	22	29	36	46	39	29	102	122	86	95	77	107
ENSMUSG00000025423	PIAS2	6	6	11	4	7	6	33	41	23	38	22	35
ENSMUSG00000025428	ATP5A1	38	52	47	63	52	58	399	370	260	324	275	301
ENSMUSG00000025429	PSTPIP2	0	0	0	1	0	1	3	1	1	6	1	1
ENSMUSG00000025432	AVIL	1	0	0	0	1	0	1	1	2	2	4	1
ENSMUSG00000025436	ATP23	1	0	1	3	3	2	10	10	4	10	9	12
ENSMUSG00000025437	USP33	13	9	21	14	16	11	71	105	58	101	42	73
ENSMUSG00000025439	CLNS1A	4	10	10	8	9	5	54	49	31	54	38	46
ENSMUSG00000025450	GM9752	0	0	0	0	0	0	2	4	4	0	2	2
ENSMUSG00000025451	PAIP1	80	113	113	107	101	107	467	472	387	469	392	436
ENSMUSG00000025453	NNT	1	2	4	2	0	1	4	5	1	6	8	4
ENSMUSG00000025461	CD163L1	0	0	0	1	1	1	0	3	2	2	0	1
ENSMUSG00000025464	PAOX	0	0	0	1	2	1	1	4	4	2	5	2
ENSMUSG00000025465	ECHS1	3	6	10	5	3	6	29	44	25	42	28	34
ENSMUSG00000025466	FUOM	1	0	0	0	0	0	2	0	1	0	1	0
ENSMUSG00000025468	CALY	0	0	0	0	0	0	0	0	0	0	1	0

ENSMUSG00000025470	ZFP511	2	0	0	6	2	2	12	9	6	6	9	12
ENSMUSG00000025473	ADAM8	2	1	2	1	1	0	5	4	3	3	3	5
ENSMUSG00000025474	TUBGCP2	1	1	3	3	6	3	17	18	18	22	11	13
ENSMUSG00000025484	BET1L	0	1	3	3	4	4	13	21	7	11	10	10
ENSMUSG00000025485	RIC8A	5	4	10	7	6	4	27	39	27	32	19	40
ENSMUSG00000025486	SIRT3	7	8	6	10	11	9	39	29	19	30	27	27
ENSMUSG00000025487	PSMD13	10	9	16	13	11	12	67	83	51	59	48	50
ENSMUSG00000025491	IFITM1	0	2	1	0	0	1	3	1	3	3	3	0
ENSMUSG00000025494	SIGIRR	1	3	2	8	6	7	27	14	18	19	20	27
ENSMUSG00000025495	PTDSS2	1	1	1	2	2	1	13	18	12	11	9	9
ENSMUSG00000025497	CDHR5	0	0	0	0	0	0	0	0	0	1	0	0
ENSMUSG00000025498	IRF7	1	0	0	2	0	1	2	5	2	1	1	1
ENSMUSG00000025499	HRAS	7	5	14	5	7	8	50	42	34	45	25	37
ENSMUSG00000025500	LMNTD2	0	0	0	0	0	0	0	0	1	0	0	0
ENSMUSG00000025503	TALDO1	6	6	6	14	9	12	65	56	49	60	38	58
ENSMUSG00000025505	TMEM80	1	2	0	3	3	4	5	9	3	11	2	7
ENSMUSG00000025507	PIDD1	1	1	4	3	6	9	19	18	17	13	17	14
ENSMUSG00000025508	RPLP2	156	197	174	236	207	184	1398	1417	1032	1311	1203	1257
ENSMUSG00000025509	PNPLA2	6	5	5	2	4	0	20	29	16	19	23	23
ENSMUSG00000025510	CD151	0	1	1	3	0	0	5	8	1	7	5	10
ENSMUSG00000025511	TSPAN4	13	20	13	21	18	15	94	89	51	110	72	76
ENSMUSG00000025512	CHID1	2	5	9	5	4	2	28	26	24	28	28	30
ENSMUSG00000025515	MUC2	0	0	0	0	0	0	1	0	0	0	0	0
ENSMUSG00000025521	TMEM192	3	5	4	3	0	2	12	13	6	17	7	11
ENSMUSG00000025525	APOOL	1	1	4	2	7	3	8	13	13	15	21	10
ENSMUSG00000025531	CHM	4	5	1	7	1	3	26	18	16	28	13	11
ENSMUSG00000025532	CRCP	2	2	4	2	5	5	17	14	13	11	11	12
ENSMUSG00000025533	ASL	8	16	13	15	11	13	69	83	60	84	46	63
ENSMUSG00000025534	GUSB	5	11	12	20	18	23	78	87	61	79	67	53
ENSMUSG00000025537	PHKG1	1	0	0	0	0	0	0	2	2	0	0	0
ENSMUSG00000025538	SUMF2	1	2	4	1	2	3	11	15	15	18	12	16
ENSMUSG00000025544	TM9SF2	5	9	6	15	9	8	51	56	45	61	44	36
ENSMUSG00000025545	CLYBL	1	2	2	3	3	3	10	6	5	9	11	13
ENSMUSG00000025555	FARP1	1	2	3	7	5	3	12	24	7	19	13	16
ENSMUSG00000025557	SLC15A1	0	0	0	0	0	1	0	2	2	1	2	4
ENSMUSG00000025558	DOCK9	0	2	1	4	0	0	10	6	3	6	3	2
ENSMUSG00000025571	TNRC6C	70	93	68	85	87	121	189	170	129	304	138	184
ENSMUSG00000025572	TMC6	6	8	8	6	6	8	14	28	16	13	15	15
ENSMUSG00000025574	TK1	3	6	10	6	8	11	39	41	36	28	34	45
ENSMUSG00000025575	CANT1	2	2	1	4	3	1	12	13	9	11	7	2
ENSMUSG00000025577	CBX2	3	5	1	1	1	2	18	13	8	14	14	12
ENSMUSG00000025578	CBX8	2	6	4	5	6	5	6	11	9	15	10	17
ENSMUSG00000025579	GAA	3	3	4	1	2	0	7	7	7	8	10	6
ENSMUSG00000025580	EIF4A3	21	13	24	31	22	20	118	157	102	122	110	108
ENSMUSG00000025583	RPTOR	1	5	7	6	5	6	25	25	30	22	25	30
ENSMUSG00000025584	PDE8A	0	0	0	0	0	0	1	0	0	0	0	0
ENSMUSG00000025586	CPEB1	0	0	0	2	0	0	2	0	3	3	3	3
ENSMUSG00000025591	TMA16	5	6	9	7	10	6	37	31	21	27	20	33
ENSMUSG00000025592	DACH2	0	0	0	0	0	0	1	0	0	0	0	0
ENSMUSG00000025597	KLHL4	0	0	0	0	0	0	1	0	0	0	0	0
ENSMUSG00000025602	ZFP202	0	0	0	0	0	0	0	0	0	0	1	0
ENSMUSG00000025607	COPG2	1	2	1	2	2	5	11	13	4	8	13	9
ENSMUSG00000025609	MKLN1	13	14	13	24	17	15	62	62	53	75	48	55
ENSMUSG00000025612	BACH1	2	1	2	2	0	2	17	12	13	14	4	8
ENSMUSG00000025613	CCT8	79	70	72	92	80	74	401	435	350	385	277	350
ENSMUSG00000025616	USP16	10	18	8	18	15	20	65	78	68	89	55	82
ENSMUSG00000025626	PHF6	3	7	12	14	7	9	50	33	32	69	31	40
ENSMUSG00000025630	HPRT	24	23	30	34	24	23	222	250	183	302	171	177

ENSMUSG00000025645	CCDC51	0	1	1	1	2	0	6	8	8	8	5	9
ENSMUSG00000025646	ATRIP	1	4	10	7	4	1	29	26	15	25	17	24
ENSMUSG00000025647	SHISA5	11	25	21	10	18	18	96	77	55	81	55	81
ENSMUSG00000025648	PFKFB4	1	0	0	0	0	0	0	1	1	0	0	0
ENSMUSG00000025650	COL7A1	0	0	1	1	0	0	3	0	1	1	7	2
ENSMUSG00000025651	UQCRC1	28	18	21	26	35	17	156	152	95	167	128	134
ENSMUSG00000025702	MARCH8	0	2	7	5	2	2	12	10	12	20	8	14
ENSMUSG00000025716	MYO3A	0	0	0	0	0	0	1	0	0	0	0	0
ENSMUSG00000025722	WDR73	5	2	3	4	4	4	18	18	7	19	6	21
ENSMUSG00000025723	NMB	0	0	0	1	0	0	1	1	0	5	1	3
ENSMUSG00000025724	SEC11A	8	15	5	16	15	12	80	71	55	100	57	58
ENSMUSG00000025728	PIGQ	4	4	4	6	3	1	25	24	17	21	15	19
ENSMUSG00000025730	RAB40C	4	2	3	2	7	3	13	9	12	16	14	17
ENSMUSG00000025731	METTL26	6	7	10	10	9	8	50	66	42	53	47	46
ENSMUSG00000025732	MCRIP2	4	5	8	5	4	5	39	29	33	38	32	36
ENSMUSG00000025733	RHOT2	7	12	10	9	10	13	48	45	32	52	36	41
ENSMUSG00000025735	RHBDL1	0	0	0	1	0	0	0	0	0	0	0	0
ENSMUSG00000025736	JMJD8	3	3	4	10	4	8	29	21	24	21	20	21
ENSMUSG00000025737	WDR24	3	1	3	3	2	1	25	24	17	15	17	12
ENSMUSG00000025738	FBXL16	1	4	2	3	3	4	20	19	16	29	12	22
ENSMUSG00000025739	GNG13	0	5	3	6	0	2	12	15	8	8	6	7
ENSMUSG00000025742	PRPS2	1	0	0	0	0	1	7	6	5	9	5	2
ENSMUSG00000025745	HADHA	3	3	2	7	8	10	38	29	20	33	28	26
ENSMUSG00000025746	IL6	0	0	0	0	0	0	0	3	0	2	0	0
ENSMUSG00000025747	TYMS	7	22	10	12	17	11	77	47	49	75	38	57
ENSMUSG00000025754	AGBL1	0	0	0	0	0	0	0	1	0	0	0	0
ENSMUSG00000025757	HSPA4L	4	2	5	6	2	9	14	20	17	19	17	25
ENSMUSG00000025758	PLK4	11	22	17	21	18	18	96	98	102	124	84	103
ENSMUSG00000025759	MFSD8	3	3	2	3	3	3	24	22	17	20	7	14
ENSMUSG00000025762	LARP1B	16	17	12	29	16	22	97	98	61	110	72	88
ENSMUSG00000025764	JADE1	23	28	22	38	49	51	244	209	142	201	140	164
ENSMUSG00000025766	D3ERTD751E	2	3	2	0	0	2	13	13	8	15	3	6
ENSMUSG00000025779	LY96	1	1	0	1	0	0	3	4	4	4	3	2
ENSMUSG00000025780	ITIH5	20	28	21	21	15	19	104	90	62	111	79	78
ENSMUSG00000025781	ATP5C1	36	34	31	39	43	41	302	316	240	300	244	224
ENSMUSG00000025782	TAF3	23	17	21	28	27	27	92	109	67	130	84	76
ENSMUSG00000025783	4930412O13I	0	0	0	0	0	0	0	0	0	0	0	1
ENSMUSG00000025784	CLEC3B	1	1	0	0	0	0	0	1	0	3	1	0
ENSMUSG00000025785	EXOSC7	11	14	14	16	14	11	64	69	64	67	58	63
ENSMUSG00000025786	ZDHHC3	9	4	10	6	9	9	33	40	30	40	25	25
ENSMUSG00000025787	TGM4	0	0	0	0	1	0	1	0	0	1	2	2
ENSMUSG00000025789	ST8SIA2	0	0	0	0	0	0	0	0	0	1	0	0
ENSMUSG00000025791	PGM2	1	3	4	5	2	4	21	31	13	24	17	21
ENSMUSG00000025792	SLC25A10	0	5	4	1	2	4	15	8	6	12	6	10
ENSMUSG00000025793	HGS	0	7	2	2	2	3	24	23	15	8	9	13
ENSMUSG00000025794	RPL14	159	198	169	210	228	186	907	959	658	951	767	817
ENSMUSG00000025795	RASSF3	4	1	6	2	2	3	10	5	3	7	5	4
ENSMUSG00000025804	CCR1	0	0	0	0	0	1	1	1	0	0	1	0
ENSMUSG00000025809	ITGB1	7	15	14	11	13	15	90	90	49	93	76	82
ENSMUSG00000025810	NRP1	0	1	1	0	0	0	1	1	1	0	0	0
ENSMUSG00000025812	PARD3	0	1	0	1	0	0	1	1	1	1	0	0
ENSMUSG00000025815	DHTKD1	0	0	0	0	0	0	1	0	0	0	0	1
ENSMUSG00000025816	SEC61A2	1	3	2	12	13	9	30	38	25	37	29	32
ENSMUSG00000025817	NUDT5	14	6	14	25	12	16	75	70	40	77	43	69
ENSMUSG00000025821	ZFP282	2	2	2	5	4	1	13	14	11	17	14	17
ENSMUSG00000025823	PDIA4	81	123	106	121	123	113	343	423	320	406	270	360
ENSMUSG00000025825	ISCU	0	0	0	0	0	0	3	5	6	9	6	12
ENSMUSG00000025857	DNAAF5	2	8	6	13	6	6	47	48	36	39	35	45

ENSMUSG00000025858	GET4	3	1	3	3	2	4	20	20	19	17	18	17
ENSMUSG00000025860	XIAP	6	7	9	12	8	8	63	48	34	48	35	25
ENSMUSG00000025862	STAG2	12	23	5	17	16	11	75	76	50	89	45	59
ENSMUSG00000025868	HIGD2A	2	8	7	8	11	5	47	50	36	45	29	36
ENSMUSG00000025869	NOP16	8	8	11	9	8	3	58	62	33	56	40	52
ENSMUSG00000025870	ARL10	0	2	1	0	0	1	1	1	0	1	0	1
ENSMUSG00000025871	4833439L19R	6	1	3	1	4	3	17	19	9	9	7	13
ENSMUSG00000025872	THOC3	6	22	17	10	13	9	61	63	40	70	57	41
ENSMUSG00000025873	FAF2	10	14	6	19	14	14	54	51	37	47	34	43
ENSMUSG00000025875	TSPAN17	0	0	1	0	0	0	0	0	0	0	0	0
ENSMUSG00000025876	UNC5A	7	8	7	6	4	7	23	30	15	46	17	29
ENSMUSG00000025877	HK3	0	0	0	0	1	1	1	0	1	4	1	0
ENSMUSG00000025878	UIMC1	12	16	9	15	18	19	69	64	32	80	42	51
ENSMUSG00000025880	SMAD7	7	13	5	14	12	6	29	45	28	57	39	28
ENSMUSG00000025888	CASP1	0	0	1	0	0	0	0	0	0	0	0	0
ENSMUSG00000025893	KBTBD3	1	1	0	4	3	1	7	9	4	9	3	8
ENSMUSG00000025894	AASDHPPT	4	10	11	12	7	9	45	49	48	42	30	46
ENSMUSG00000025898	CWF19L2	15	35	8	24	14	17	75	69	43	72	59	72
ENSMUSG00000025899	ALKBH8	5	8	3	3	6	4	26	33	26	46	20	33
ENSMUSG00000025900	RP1	0	0	0	0	0	0	0	0	1	0	0	0
ENSMUSG00000025902	SOX17	0	0	0	0	0	0	0	1	2	1	0	1
ENSMUSG00000025903	LYPLA1	5	9	3	6	6	1	26	28	23	28	24	27
ENSMUSG00000025907	RB1CC1	15	23	15	27	22	29	95	102	66	92	72	82
ENSMUSG00000025909	SNTG1	0	0	0	0	0	0	0	0	1	0	0	0
ENSMUSG00000025911	ADHFE1	0	1	0	0	0	0	9	2	7	4	5	5
ENSMUSG00000025912	MYBL1	5	12	9	5	10	9	48	42	39	45	32	31
ENSMUSG00000025915	SGK3	0	0	0	0	0	0	2	1	0	1	1	2
ENSMUSG00000025916	PPP1R42	0	1	0	0	0	0	1	1	5	1	0	0
ENSMUSG00000025917	COPS5	7	9	14	16	16	9	99	94	67	108	88	69
ENSMUSG00000025921	RDH10	1	1	1	4	0	2	6	17	12	15	3	8
ENSMUSG00000025922	GM5045	0	0	0	0	0	0	1	0	0	0	0	1
ENSMUSG00000025925	TERF1	5	13	7	18	15	11	61	60	60	81	51	44
ENSMUSG00000025927	TFAP2B	0	0	0	0	0	0	0	0	1	0	0	0
ENSMUSG00000025933	TMEM14A	0	0	0	0	0	0	2	0	0	3	1	4
ENSMUSG00000025934	GSTA3	0	0	0	0	0	0	0	1	1	0	0	0
ENSMUSG00000025935	TRAM1	64	56	47	61	44	51	173	211	133	171	133	166
ENSMUSG00000025937	LACTB2	3	2	4	3	4	3	13	13	11	24	13	15
ENSMUSG00000025938	SLCO5A1	0	0	0	0	0	0	1	1	0	0	0	0
ENSMUSG00000025939	UBE2W	5	4	2	11	7	4	41	24	20	42	18	22
ENSMUSG00000025940	TMEM70	5	5	5	9	14	4	63	37	52	57	38	38
ENSMUSG00000025949	PIKFYVE	8	8	12	19	19	13	73	80	45	73	44	58
ENSMUSG00000025950	IDH1	1	1	0	5	1	0	24	13	18	17	15	14
ENSMUSG00000025956	METTL21A	0	4	2	5	5	5	17	23	7	15	16	10
ENSMUSG00000025958	CREB1	12	20	18	9	13	16	53	59	31	48	50	48
ENSMUSG00000025959	KLF7	6	16	8	8	8	8	18	25	15	49	32	17
ENSMUSG00000025962	FASTKD2	6	4	8	9	7	8	56	71	41	53	43	37
ENSMUSG00000025963	MDH1B	0	0	0	1	0	0	0	0	0	0	0	0
ENSMUSG00000025967	EEF1B2	180	205	235	259	255	267	1133	1114	812	1165	818	921
ENSMUSG00000025968	NDUFS1	24	29	29	42	40	22	155	169	106	157	121	130
ENSMUSG00000025969	NRP2	3	5	9	13	13	16	40	65	35	51	33	38
ENSMUSG00000025971	MAIP1	1	4	4	4	2	10	23	18	21	35	16	19
ENSMUSG00000025977	BOLL	0	0	0	0	0	1	0	0	0	0	0	0
ENSMUSG00000025979	MOB4	14	25	13	24	21	23	104	126	88	145	86	97
ENSMUSG00000025980	HSPD1	50	84	84	83	88	82	784	777	560	920	539	612
ENSMUSG00000025981	COQ10B	2	4	3	3	0	3	10	13	18	12	16	18
ENSMUSG00000025982	SF3B1	114	161	139	157	144	146	718	785	568	848	548	598
ENSMUSG00000025983	CCDC150	0	0	0	0	0	0	0	0	0	1	0	0
ENSMUSG00000025986	SLC39A10	14	28	26	46	22	24	128	143	98	187	105	147

ENSMUSG00000025995	WDR75	18	20	37	26	34	22	140	183	113	158	97	117
ENSMUSG00000025997	IKZF2	78	87	60	85	68	86	304	296	210	339	219	242
ENSMUSG00000026000	LANCL1	4	6	4	6	18	12	45	68	51	43	42	52
ENSMUSG00000026003	ACADL	20	31	33	33	47	39	200	195	135	214	129	155
ENSMUSG00000026004	KANSL1L	5	9	6	6	7	3	37	33	27	32	28	36
ENSMUSG00000026005	RPE	11	30	13	18	16	17	110	117	79	120	68	82
ENSMUSG00000026009	ICOS	0	1	1	1	1	2	1	0	0	2	0	1
ENSMUSG00000026011	CTLA4	3	2	1	1	1	0	3	4	2	3	3	0
ENSMUSG00000026012	CD28	6	10	5	6	6	11	18	19	13	23	20	20
ENSMUSG00000026014	RAPH1	9	17	12	12	12	13	48	68	45	55	30	43
ENSMUSG00000026017	CARF	1	0	0	1	3	1	6	6	4	6	7	4
ENSMUSG00000026018	ICA1L	0	0	0	0	1	0	1	1	0	1	1	0
ENSMUSG00000026019	WDR12	24	22	19	37	30	38	209	196	136	196	131	138
ENSMUSG00000026020	NOP58	134	214	209	290	220	212	978	1116	761	1308	792	897
ENSMUSG00000026021	SUMO1	3	8	9	12	7	8	63	64	42	72	40	40
ENSMUSG00000026023	CDK15	1	2	1	2	1	3	5	1	5	6	1	0
ENSMUSG00000026024	ALS2	1	0	0	0	0	0	0	0	0	0	2	1
ENSMUSG00000026027	STRADB	0	1	1	0	3	0	5	6	3	5	1	11
ENSMUSG00000026028	TRAK2	5	6	3	4	7	4	31	32	12	27	24	33
ENSMUSG00000026029	CASP8	7	19	18	16	18	17	56	49	46	71	56	55
ENSMUSG00000026031	CFLAR	13	14	14	14	13	12	61	54	46	72	49	55
ENSMUSG00000026032	NDUFB3	22	17	19	20	19	22	145	131	96	121	107	123
ENSMUSG00000026034	CLK1	36	37	36	26	37	46	193	210	155	257	162	163
ENSMUSG00000026035	PPIL3	2	3	4	4	6	3	14	15	13	21	8	17
ENSMUSG00000026036	NIF3L1	4	8	0	4	4	8	26	26	21	29	20	34
ENSMUSG00000026037	ORC2	14	12	17	20	12	17	97	108	97	110	76	104
ENSMUSG00000026039	SGOL2A	12	13	17	15	16	20	73	80	45	90	49	54
ENSMUSG00000026043	COL3A1	0	0	0	0	0	0	0	0	0	0	1	0
ENSMUSG00000026049	TEX30	15	21	24	20	9	14	153	141	90	172	111	92
ENSMUSG00000026058	KHDRBS2	0	0	0	0	0	0	1	0	0	0	0	0
ENSMUSG00000026068	IL18RAP	7	13	6	10	17	16	53	67	46	60	44	46
ENSMUSG00000026069	IL1RL1	0	0	0	0	0	0	0	1	0	0	0	0
ENSMUSG00000026070	IL18R1	6	2	5	6	1	1	10	16	15	23	6	12
ENSMUSG00000026073	IL1R2	0	0	0	0	0	0	0	0	0	1	0	0
ENSMUSG00000026074	MAP4K4	41	56	37	60	50	54	247	251	149	268	154	199
ENSMUSG00000026078	PDCL3	24	36	26	31	26	21	258	203	190	479	147	164
ENSMUSG00000026080	CHST10	1	0	0	0	0	0	0	0	0	0	0	0
ENSMUSG00000026082	REV1	9	15	18	26	23	23	97	95	60	115	80	76
ENSMUSG00000026083	EIF5B	170	276	190	248	200	219	762	760	519	921	647	612
ENSMUSG00000026087	MRPL30	10	8	15	12	13	8	90	83	70	83	75	79
ENSMUSG00000026088	MITD1	8	5	10	15	10	8	49	42	33	66	34	41
ENSMUSG00000026090	2010300C02F	0	0	1	0	0	0	1	0	0	0	0	1
ENSMUSG00000026094	STK17B	42	33	40	38	46	46	173	179	177	226	132	138
ENSMUSG00000026095	ASNSD1	4	5	6	6	6	5	29	30	20	24	21	18
ENSMUSG00000026096	OSGEPL1	1	1	5	3	3	0	25	23	14	16	12	20
ENSMUSG00000026097	ORMDL1	9	9	5	15	7	6	28	28	23	33	24	31
ENSMUSG00000026098	PMS1	7	6	7	3	2	7	24	31	16	21	13	14
ENSMUSG00000026102	INPP1	9	6	10	11	13	12	47	48	45	55	43	36
ENSMUSG00000026103	GLS	77	108	103	113	99	84	500	473	337	632	346	401
ENSMUSG00000026104	STAT1	4	4	6	4	7	3	21	15	14	14	11	16
ENSMUSG00000026107	NABP1	12	12	14	19	23	17	94	104	81	119	82	86
ENSMUSG00000026109	TMEFF2	0	0	0	0	0	0	4	8	4	10	2	6
ENSMUSG00000026110	MGAT4A	6	7	2	10	5	3	9	8	9	15	12	8
ENSMUSG00000026111	UNC50	1	12	4	7	7	6	49	43	27	42	43	46
ENSMUSG00000026112	COA5	10	12	9	14	8	8	58	72	39	44	50	42
ENSMUSG00000026113	INPP4A	72	99	84	111	98	105	400	368	295	418	254	313
ENSMUSG00000026116	TMEM131	5	1	6	9	4	5	43	40	20	28	34	40
ENSMUSG00000026117	ZAP70	3	3	9	11	3	9	33	39	38	24	29	22

ENSMUSG00000026121	SEMA4C	2	4	2	8	4	4	43	36	25	33	22	28
ENSMUSG00000026123	PLEKHB2	6	5	4	3	4	5	19	18	19	30	22	23
ENSMUSG00000026125	PRSS39	0	0	0	0	0	0	0	0	0	0	1	0
ENSMUSG00000026126	PTPN18	8	25	16	16	13	11	63	75	49	59	48	47
ENSMUSG00000026127	IMP4	11	16	11	14	9	11	58	51	39	76	45	41
ENSMUSG00000026131	DST	3	8	3	7	7	12	1	6	2	9	3	2
ENSMUSG00000026134	PRIM2	13	16	16	21	17	10	84	103	65	95	60	68
ENSMUSG00000026135	ZFP142	5	11	3	8	8	11	19	28	21	30	16	20
ENSMUSG00000026142	RHBDD1	2	7	1	2	5	4	12	16	12	15	6	18
ENSMUSG00000026150	MFF	7	11	7	10	10	9	75	76	62	85	50	58
ENSMUSG00000026153	FAM135A	0	0	0	0	0	0	0	0	0	0	0	1
ENSMUSG00000026154	SDHAF4	2	4	3	2	4	2	24	23	19	25	14	16
ENSMUSG00000026155	SMAP1	12	28	24	23	21	27	69	70	52	98	60	76
ENSMUSG00000026156	B3GAT2	1	1	1	1	0	6	8	11	10	9	7	8
ENSMUSG00000026158	OGFRL1	0	0	0	0	0	0	0	0	0	1	0	0
ENSMUSG00000026159	AGFG1	7	5	6	7	19	11	60	85	36	73	39	44
ENSMUSG00000026162	NHEJ1	0	1	0	0	1	1	1	4	1	3	2	4
ENSMUSG00000026163	SPHKAP	0	0	0	0	0	0	0	0	1	0	0	0
ENSMUSG00000026167	WNT10A	2	0	0	3	2	2	5	10	9	6	10	9
ENSMUSG00000026171	RNF25	3	2	3	2	4	1	14	25	7	18	7	11
ENSMUSG00000026172	BCS1L	2	2	6	4	2	0	17	24	14	16	13	9
ENSMUSG00000026173	PLCD4	0	0	1	2	1	0	3	2	1	4	4	1
ENSMUSG00000026174	CNOT9	14	17	21	23	10	16	56	63	39	55	55	56
ENSMUSG00000026175	VIL1	1	4	1	2	2	2	3	10	4	5	3	1
ENSMUSG00000026176	CTDSP1	7	8	3	8	4	5	34	34	18	39	26	27
ENSMUSG00000026177	SLC11A1	0	0	0	0	0	0	0	0	0	0	1	0
ENSMUSG00000026179	PNKD	2	3	6	7	3	3	19	28	10	23	14	27
ENSMUSG00000026181	PPM1F	4	3	3	2	6	3	14	17	15	20	18	18
ENSMUSG00000026187	XRCC5	9	16	18	20	16	23	108	79	69	89	50	67
ENSMUSG00000026188	TMEM169	1	0	0	0	0	0	2	2	0	1	2	1
ENSMUSG00000026189	PECR	3	1	3	2	2	2	14	23	11	16	17	11
ENSMUSG00000026192	ATIC	13	26	17	24	31	23	140	139	136	144	106	153
ENSMUSG00000026193	FN1	0	0	0	0	0	0	0	1	0	0	0	0
ENSMUSG00000026196	BARD1	8	10	3	12	9	9	45	58	25	49	24	43
ENSMUSG00000026197	ZFAND2B	2	2	4	2	5	1	21	12	20	23	11	21
ENSMUSG00000026198	ABCB6	0	1	0	0	0	0	0	0	0	1	0	0
ENSMUSG00000026199	ANKZF1	4	7	2	14	10	6	42	48	37	40	18	30
ENSMUSG00000026200	GLB1L	2	2	5	1	1	1	9	7	8	3	8	9
ENSMUSG00000026201	STK16	9	24	11	14	26	13	73	64	56	66	65	74
ENSMUSG00000026202	TUBA4A	14	14	12	12	19	12	58	70	52	59	35	71
ENSMUSG00000026203	DNAJB2	0	0	2	0	0	0	3	5	1	2	1	3
ENSMUSG00000026205	SLC23A3	0	0	0	0	0	0	0	0	2	1	0	0
ENSMUSG00000026207	SPEG	0	1	0	0	0	0	0	0	0	0	0	0
ENSMUSG00000026209	DNPEP	5	2	4	9	5	3	27	29	24	20	15	24
ENSMUSG00000026213	STK11IP	1	6	2	7	2	2	10	14	11	10	7	19
ENSMUSG00000026219	TRIP12	6	12	12	27	24	23	113	105	77	107	81	93
ENSMUSG00000026222	SP100	7	4	13	6	5	5	34	37	25	33	28	30
ENSMUSG00000026223	ITM2C	9	15	14	8	9	8	88	81	57	77	61	68
ENSMUSG00000026224	4933407L21R	0	0	0	0	0	0	0	0	0	0	1	0
ENSMUSG00000026227	2810459M11	0	0	0	1	1	0	2	2	1	1	0	1
ENSMUSG00000026229	PSMD1	20	41	41	55	37	36	151	134	104	128	134	117
ENSMUSG00000026234	NCL	175	232	203	332	246	217	1265	1434	1039	1358	981	1143
ENSMUSG00000026237	NMUR1	0	0	0	0	0	0	1	3	1	2	0	0
ENSMUSG00000026238	PTMA	76	130	95	136	114	120	791	708	512	833	515	636
ENSMUSG00000026239	PDE6D	6	2	0	2	1	1	9	7	11	9	12	3
ENSMUSG00000026240	COPS7B	3	7	1	0	5	6	20	17	10	19	13	22
ENSMUSG00000026241	NPPC	0	0	0	0	0	0	1	0	0	0	0	0
ENSMUSG00000026245	FARSB	4	7	10	12	9	8	54	43	31	54	47	47

ENSMUSG00000026248	MRPL44	13	1	7	18	9	11	61	62	47	68	62	55
ENSMUSG00000026254	EIF4E2	8	13	14	10	16	14	65	76	52	65	47	54
ENSMUSG00000026255	EFHD1	0	0	1	0	0	0	0	0	0	0	0	0
ENSMUSG00000026258	3110079O15I	0	0	0	0	0	0	0	0	0	1	0	0
ENSMUSG00000026260	NDUFA10	9	13	22	15	10	14	76	80	62	105	57	59
ENSMUSG00000026269	RNPEPL1	9	3	1	1	5	11	16	23	12	27	17	23
ENSMUSG00000026270	CAPN10	1	1	8	5	1	0	10	7	6	14	13	9
ENSMUSG00000026273	MTERF4	0	3	2	1	0	1	8	11	5	6	4	11
ENSMUSG00000026274	PASK	3	3	3	0	1	0	13	20	13	14	12	8
ENSMUSG00000026275	PPP1R7	4	6	4	5	6	4	47	42	29	42	35	39
ENSMUSG00000026276	37500	0	1	0	1	1	0	4	1	8	5	2	3
ENSMUSG00000026277	STK25	23	40	36	41	39	30	114	114	104	114	104	105
ENSMUSG00000026279	THAP4	3	5	6	1	5	4	29	17	18	14	19	16
ENSMUSG00000026280	ATG4B	4	6	3	4	7	1	15	21	16	18	20	19
ENSMUSG00000026281	DTYMK	16	22	20	34	27	34	130	155	102	162	135	117
ENSMUSG00000026283	ING5	5	7	1	4	9	7	26	41	21	18	25	16
ENSMUSG00000026285	PDCD1	0	0	0	0	0	0	0	0	1	0	0	0
ENSMUSG00000026288	INPP5D	2	3	3	6	4	3	17	12	13	21	13	10
ENSMUSG00000026289	ATG16L1	5	6	5	4	10	4	28	25	11	20	24	14
ENSMUSG00000026305	LRRFIP1	9	20	13	10	14	11	66	93	49	85	58	64
ENSMUSG00000026307	SCLY	0	3	4	4	0	5	8	9	9	5	7	16
ENSMUSG00000026308	KLHL30	0	0	0	0	0	0	0	0	0	0	0	1
ENSMUSG00000026309	ILKAP	4	6	11	14	10	12	44	49	44	59	46	45
ENSMUSG00000026311	ASB1	1	3	3	1	1	1	12	9	7	11	12	13
ENSMUSG00000026313	HDAC4	20	32	23	20	14	16	30	39	26	56	33	36
ENSMUSG00000026317	CLN8	0	0	0	1	0	0	0	0	0	0	0	0
ENSMUSG00000026319	2310035C23F	8	30	8	17	22	14	47	52	33	61	38	40
ENSMUSG00000026321	TNFRSF11A	0	0	1	0	0	0	0	0	0	0	0	1
ENSMUSG00000026322	HTR4	0	0	0	0	0	0	0	1	0	0	0	0
ENSMUSG00000026333	GIN1	2	0	0	5	3	1	12	18	12	14	9	15
ENSMUSG00000026339	CCDC93	0	5	2	1	1	2	16	8	6	15	10	5
ENSMUSG00000026341	ACTR3	26	39	31	35	43	29	226	217	175	213	176	181
ENSMUSG00000026347	TMEM163	0	1	0	1	1	0	3	6	4	2	4	5
ENSMUSG00000026348	ACMSD	0	0	0	0	0	0	0	0	0	0	1	0
ENSMUSG00000026349	CCNT2	11	20	15	17	14	12	69	56	43	70	47	60
ENSMUSG00000026353	UBXN4	36	58	45	50	38	42	201	199	153	222	138	179
ENSMUSG00000026354	LCT	0	0	1	0	0	0	3	0	0	2	2	1
ENSMUSG00000026355	MCM6	16	24	30	28	33	22	204	177	147	181	154	162
ENSMUSG00000026356	DARS	23	40	30	41	35	33	223	202	148	262	153	217
ENSMUSG00000026357	RGS18	1	1	0	1	0	0	2	0	1	1	0	3
ENSMUSG00000026358	RGS1	0	0	0	0	1	0	0	0	0	0	0	0
ENSMUSG00000026360	RGS2	6	8	1	2	2	1	24	24	10	22	13	8
ENSMUSG00000026361	CDC73	5	10	12	10	11	12	52	75	44	70	39	28
ENSMUSG00000026365	CFH	0	0	0	0	0	0	0	0	1	1	0	0
ENSMUSG00000026368	F13B	0	0	0	0	0	0	0	1	0	1	0	0
ENSMUSG00000026374	TSN	59	47	40	46	55	45	145	180	112	183	136	149
ENSMUSG00000026377	NIFK	3	7	9	7	11	8	31	41	28	32	32	30
ENSMUSG00000026383	EPB41L5	3	0	5	1	2	6	17	26	16	26	17	22
ENSMUSG00000026384	PTPN4	7	4	6	6	5	5	25	21	19	22	20	13
ENSMUSG00000026385	DBI	14	34	22	42	27	31	267	282	204	236	181	205
ENSMUSG00000026388	3110009E18F	0	2	3	4	0	3	9	8	5	11	8	8
ENSMUSG00000026389	STEAP3	4	0	2	5	2	0	15	20	16	20	20	15
ENSMUSG00000026393	NEK7	16	18	11	11	15	22	55	54	40	74	49	62
ENSMUSG00000026394	ATP6V1G3	3	1	3	0	2	0	3	3	1	8	2	5
ENSMUSG00000026395	PTPRC	144	205	159	227	178	189	971	970	661	1239	696	709
ENSMUSG00000026404	DDX59	0	1	0	1	1	1	2	5	5	7	3	2
ENSMUSG00000026409	PFKFB2	0	0	1	1	0	1	3	2	3	2	1	2
ENSMUSG00000026411	TMEM9	0	2	1	6	2	2	17	12	11	7	18	12

ENSMUSG00000026413	PKP1	0	1	0	0	0	0	1	0	0	0	0	2
ENSMUSG00000026414	TNNT2	0	0	0	0	0	0	0	0	1	0	0	0
ENSMUSG00000026418	TNNI1	2	3	5	7	3	2	14	17	17	15	14	13
ENSMUSG00000026421	CSRP1	5	3	1	8	2	2	25	19	17	37	12	15
ENSMUSG00000026425	SRGAP2	1	3	8	7	8	8	27	19	18	35	17	19
ENSMUSG00000026426	ARL8A	7	5	2	4	2	6	29	51	31	32	25	25
ENSMUSG00000026427	EIF2D	2	1	2	4	3	3	9	12	5	15	6	10
ENSMUSG00000026429	UBE2T	6	4	3	3	1	6	45	34	31	31	40	30
ENSMUSG00000026430	RASSF5	4	5	3	12	9	9	48	41	33	43	44	41
ENSMUSG00000026432	AVPR1B	0	0	0	0	0	0	1	0	1	0	0	0
ENSMUSG00000026433	RAB29	1	2	2	3	6	3	7	6	11	8	12	9
ENSMUSG00000026434	NUCKS1	70	103	72	125	110	85	439	437	353	462	349	385
ENSMUSG00000026436	ELK4	13	22	14	16	25	20	44	50	45	66	41	53
ENSMUSG00000026439	RBBP5	5	4	7	6	4	4	14	19	13	15	10	18
ENSMUSG00000026447	PIK3C2B	2	6	5	3	6	1	11	23	13	20	15	12
ENSMUSG00000026452	SYT2	1	0	0	0	1	0	0	0	0	0	0	0
ENSMUSG00000026455	KLHL12	2	0	3	7	1	2	9	7	11	10	9	13
ENSMUSG00000026456	CYB5R1	10	8	5	4	6	4	26	33	17	20	17	20
ENSMUSG00000026457	ADIPOR1	31	40	42	38	30	45	148	139	116	176	102	136
ENSMUSG00000026458	PPFIA4	0	0	1	0	1	1	0	0	0	0	0	0
ENSMUSG00000026463	ATP2B4	0	0	0	1	0	0	0	0	0	1	0	1
ENSMUSG00000026466	TOR1AIP1	7	9	7	14	8	5	42	54	40	64	46	46
ENSMUSG00000026468	LHX4	1	0	0	1	0	2	2	0	2	0	0	1
ENSMUSG00000026469	XPR1	2	9	10	4	9	5	44	29	25	38	19	33
ENSMUSG00000026470	STX6	5	9	4	6	6	5	15	18	12	17	17	11
ENSMUSG00000026471	MR1	0	0	0	2	4	0	5	3	3	5	2	6
ENSMUSG00000026473	GLUL	2	5	8	13	6	5	61	61	35	49	28	47
ENSMUSG00000026480	NCF2	0	0	0	0	0	0	0	1	0	0	0	0
ENSMUSG00000026482	RGL1	0	0	0	0	0	0	1	0	4	0	0	1
ENSMUSG00000026483	FAM129A	8	11	12	17	11	13	46	28	35	45	41	30
ENSMUSG00000026484	RNF2	15	9	9	17	10	7	54	63	57	73	53	48
ENSMUSG00000026489	COQ8A	0	1	0	0	1	0	1	2	0	1	1	3
ENSMUSG00000026491	AHCTF1	11	13	12	21	16	12	86	82	68	83	62	74
ENSMUSG00000026492	TFB2M	0	0	1	1	0	4	9	8	13	10	5	6
ENSMUSG00000026495	EFCAB2	0	0	2	0	0	0	4	0	1	1	4	2
ENSMUSG00000026496	PARP1	12	23	26	25	26	22	159	152	135	152	113	139
ENSMUSG00000026499	ACBD3	8	9	6	6	5	6	26	29	26	58	31	27
ENSMUSG00000026500	COX20	9	5	4	7	15	6	47	30	30	41	28	36
ENSMUSG00000026502	DESI2	7	4	3	4	4	5	40	27	15	31	26	26
ENSMUSG00000026504	SDCCAG8	0	2	0	3	1	4	13	14	7	7	6	7
ENSMUSG00000026509	CAPN2	5	7	10	12	4	6	40	46	27	47	31	42
ENSMUSG00000026510	TRP53BP2	2	4	3	5	5	2	22	34	24	37	32	15
ENSMUSG00000026511	SRP9	10	10	11	12	10	10	43	53	41	50	40	49
ENSMUSG00000026516	NVL	27	27	27	27	35	14	158	161	134	182	87	147
ENSMUSG00000026519	TMEM63A	3	4	2	1	2	7	18	10	4	17	5	12
ENSMUSG00000026520	PYCR2	22	33	29	42	33	37	201	186	146	186	148	158
ENSMUSG00000026525	OPN3	0	0	0	0	0	0	2	1	1	1	2	0
ENSMUSG00000026526	FH1	12	20	31	22	27	33	120	128	86	134	95	108
ENSMUSG00000026544	DUSP23	0	0	0	0	0	0	0	0	1	0	0	0
ENSMUSG00000026547	TAGLN2	22	39	37	26	42	40	107	149	92	122	97	101
ENSMUSG00000026553	COPA	14	16	12	16	14	10	89	91	65	87	55	79
ENSMUSG00000026554	DCAF8	7	11	7	14	7	13	44	76	42	62	48	64
ENSMUSG00000026558	UCK2	14	14	18	9	15	20	76	74	55	83	49	77
ENSMUSG00000026563	TADA1	1	1	0	1	5	5	15	9	11	17	12	10
ENSMUSG00000026565	POU2F1	10	9	3	14	8	10	32	30	21	27	15	27
ENSMUSG00000026566	MPZL1	2	1	3	3	2	2	12	12	15	11	9	8
ENSMUSG00000026567	ADCY10	0	0	0	0	0	0	0	3	0	0	0	0
ENSMUSG00000026568	MPC2	12	10	15	13	9	8	72	79	69	92	65	67

ENSMUSG00000026571	DCAF6	23	25	25	22	30	25	115	135	82	105	98	106
ENSMUSG00000026575	NME7	2	2	2	3	3	1	12	12	5	9	4	17
ENSMUSG00000026576	ATP1B1	1	1	1	2	3	1	0	2	1	0	2	3
ENSMUSG00000026577	BLZF1	3	3	1	2	0	6	20	20	18	24	16	17
ENSMUSG00000026578	CCDC181	2	1	3	2	4	1	14	11	9	10	11	15
ENSMUSG00000026580	SELP	0	0	0	0	0	0	0	0	0	0	0	1
ENSMUSG00000026584	SCYL3	2	5	3	8	2	6	17	15	27	24	18	22
ENSMUSG00000026585	KIFAP3	2	0	2	5	5	0	10	11	7	22	11	20
ENSMUSG00000026586	PRRX1	0	0	0	0	0	0	0	0	0	0	0	1
ENSMUSG00000026594	RALGPS2	5	11	9	6	11	5	36	48	24	39	41	21
ENSMUSG00000026596	ABL2	1	13	7	12	8	4	23	35	20	38	20	33
ENSMUSG00000026600	SOAT1	30	35	34	36	33	35	206	214	147	189	145	164
ENSMUSG00000026601	AXDND1	3	3	2	2	2	2	9	8	10	16	8	11
ENSMUSG00000026603	SMYD2	4	8	5	5	10	3	29	37	20	43	27	38
ENSMUSG00000026604	PTPN14	0	0	0	0	0	0	0	0	0	1	0	0
ENSMUSG00000026605	CENPF	19	38	30	58	33	21	138	114	82	146	116	89
ENSMUSG00000026608	KCTD3	3	0	2	6	0	3	16	8	8	11	7	8
ENSMUSG00000026615	EPRS	58	119	75	81	89	74	308	312	234	320	243	252
ENSMUSG00000026616	CR2	0	1	0	0	0	0	0	1	0	0	1	0
ENSMUSG00000026617	BPNT1	1	2	3	2	4	1	28	29	27	23	20	23
ENSMUSG00000026618	IARS2	4	3	2	7	4	6	30	30	21	35	14	25
ENSMUSG00000026621	MARC1	0	0	0	0	0	0	0	0	0	0	0	1
ENSMUSG00000026622	NEK2	0	2	5	0	0	0	13	16	8	19	10	12
ENSMUSG00000026623	LPGAT1	14	29	11	19	22	26	46	56	37	45	43	43
ENSMUSG00000026626	PPP2R5A	14	23	20	15	22	25	88	96	54	108	69	77
ENSMUSG00000026627	TMEM206	0	0	0	0	5	2	11	2	6	5	7	4
ENSMUSG00000026630	BATF3	1	3	4	1	2	2	13	17	12	13	10	10
ENSMUSG00000026632	TATDN3	0	0	1	1	1	1	1	5	3	6	4	2
ENSMUSG00000026634	ANGEL2	6	5	5	7	9	4	32	38	25	19	27	16
ENSMUSG00000026637	TRAF5	4	3	3	2	1	3	9	4	10	13	4	11
ENSMUSG00000026639	LAMB3	0	0	0	0	0	0	2	2	0	0	1	1
ENSMUSG00000026641	USF1	6	7	7	12	9	10	20	30	22	22	25	32
ENSMUSG00000026643	NMT2	16	23	11	31	20	21	106	70	67	97	84	76
ENSMUSG00000026646	SUV39H2	8	3	6	7	7	6	61	39	32	50	35	37
ENSMUSG00000026648	DCLRE1C	4	5	6	9	8	9	49	37	19	39	28	29
ENSMUSG00000026649	CFAP126	0	0	0	0	1	0	1	3	2	2	5	1
ENSMUSG00000026655	FAM107B	9	12	12	5	10	8	31	28	26	26	21	26
ENSMUSG00000026657	FRMD4A	2	1	1	2	2	2	9	10	8	8	3	3
ENSMUSG00000026659	DUSP12	4	3	1	3	2	2	8	15	9	12	13	12
ENSMUSG00000026662	SEPHS1	12	17	13	25	19	10	67	82	50	90	50	64
ENSMUSG00000026663	ATF6	6	5	2	7	5	7	28	13	15	31	20	26
ENSMUSG00000026664	PHYH	5	7	10	13	12	12	91	91	67	85	67	87
ENSMUSG00000026667	UHMK1	11	10	7	7	8	9	38	45	31	53	29	27
ENSMUSG00000026669	MCM10	19	25	22	31	28	13	125	113	113	123	83	102
ENSMUSG00000026670	UAP1	6	13	10	18	9	5	55	62	51	65	41	54
ENSMUSG00000026672	OPTN	0	0	0	0	0	0	0	1	0	0	0	0
ENSMUSG00000026674	DDR2	0	0	0	0	0	0	0	1	0	0	0	0
ENSMUSG00000026675	HSD17B7	1	1	2	2	3	1	10	10	8	12	10	14
ENSMUSG00000026679	ENKUR	0	1	0	0	0	0	1	0	0	0	0	1
ENSMUSG00000026683	NUF2	1	4	4	5	5	4	11	28	13	26	13	12
ENSMUSG00000026687	ALDH9A1	9	16	13	18	16	11	92	95	70	104	49	76
ENSMUSG00000026688	MGST3	0	0	0	0	0	0	1	1	0	0	1	0
ENSMUSG00000026694	METTL13	1	7	0	7	5	3	16	16	9	20	11	12
ENSMUSG00000026696	VAMP4	3	4	3	4	4	1	20	18	24	29	15	20
ENSMUSG00000026698	PIGC	3	2	0	1	2	3	8	4	4	5	7	1
ENSMUSG00000026701	PRDX6	12	13	16	15	20	14	93	120	72	128	83	98
ENSMUSG00000026705	KLHL20	6	2	3	1	1	4	18	23	9	19	9	13
ENSMUSG00000026707	NSUN6	1	4	2	3	1	1	7	12	6	12	5	11

ENSMUSG00000026708	CENPL	4	4	0	3	3	0	13	24	15	20	9	10
ENSMUSG00000026709	DARS2	3	2	3	5	4	3	19	18	14	25	15	10
ENSMUSG00000026712	MRC1	0	0	0	0	0	0	1	4	2	0	1	0
ENSMUSG00000026715	SERPINC1	2	3	4	5	2	6	11	13	5	8	15	5
ENSMUSG00000026718	STAM	5	7	4	10	7	4	42	46	40	58	28	29
ENSMUSG00000026721	RABGAP1L	7	5	0	5	4	2	22	15	13	25	9	17
ENSMUSG00000026723	TRDMT1	1	1	2	0	2	1	1	5	1	1	2	3
ENSMUSG00000026727	RSU1	17	15	23	17	9	10	80	64	66	85	68	70
ENSMUSG00000026728	VIM	41	76	78	77	78	98	398	463	346	462	348	403
ENSMUSG00000026729	4930562F07F	0	1	0	1	0	1	4	4	3	5	4	2
ENSMUSG00000026730	PTER	2	1	2	3	4	3	34	22	27	31	21	19
ENSMUSG00000026736	4930426L09F	0	0	0	0	0	0	2	0	1	0	0	0
ENSMUSG00000026737	PIP4K2A	17	21	18	13	18	20	84	97	57	113	52	71
ENSMUSG00000026739	BMI1	6	1	0	7	5	8	31	31	19	41	20	26
ENSMUSG00000026740	DNAJC1	26	46	45	41	49	40	181	161	139	203	146	160
ENSMUSG00000026743	MLLT10	17	23	26	25	24	23	108	116	80	155	93	102
ENSMUSG00000026749	NEK6	2	4	5	4	5	5	34	37	27	38	26	35
ENSMUSG00000026750	PSMB7	22	23	27	34	21	30	125	167	128	169	123	143
ENSMUSG00000026753	PPP6C	6	5	9	5	6	5	40	44	43	45	37	30
ENSMUSG00000026754	GOLGA1	7	7	5	16	6	9	37	33	29	39	24	24
ENSMUSG00000026755	ARPC5L	19	21	21	34	21	23	158	163	120	162	89	116
ENSMUSG00000026761	ORC4	13	13	13	13	7	8	57	66	43	66	42	49
ENSMUSG00000026764	KIF5C	0	3	0	1	1	0	1	1	1	1	0	3
ENSMUSG00000026765	LYPD6B	0	0	0	1	0	1	2	0	1	2	0	0
ENSMUSG00000026766	MMADHC	4	6	5	7	8	4	51	49	23	42	28	43
ENSMUSG00000026767	MINDY3	9	11	12	14	15	4	65	76	59	68	51	45
ENSMUSG00000026770	IL2RA	0	1	0	0	0	0	2	1	3	0	1	2
ENSMUSG00000026771	SPOPL	3	6	6	10	3	5	30	36	23	42	15	31
ENSMUSG00000026773	PFKFB3	4	7	5	4	8	7	61	71	59	61	64	51
ENSMUSG00000026774	4931423N10I	0	0	0	1	0	0	1	5	0	1	1	0
ENSMUSG00000026775	YME1L1	105	125	142	144	157	145	875	975	700	942	660	814
ENSMUSG00000026778	PRKCQ	8	3	10	5	7	9	17	21	21	16	16	17
ENSMUSG00000026779	MASTL	8	5	7	9	8	6	27	27	17	28	20	19
ENSMUSG00000026781	ACBD5	15	9	15	16	8	10	51	64	39	51	38	47
ENSMUSG00000026782	ABI2	17	21	15	22	22	14	100	77	48	101	44	62
ENSMUSG00000026784	PDSS1	4	5	7	9	12	9	39	58	50	39	34	41
ENSMUSG00000026785	PKN3	2	2	1	2	1	3	7	4	7	9	4	5
ENSMUSG00000026786	APBB1IP	13	21	21	20	25	34	173	174	107	181	116	140
ENSMUSG00000026788	ZBTB43	2	8	5	8	5	5	16	19	20	27	26	18
ENSMUSG00000026790	ODF2	2	5	5	3	9	5	7	10	6	11	6	12
ENSMUSG00000026791	SLC2A8	0	2	0	1	1	1	5	2	2	3	2	5
ENSMUSG00000026792	LRSAM1	0	1	0	0	0	0	6	3	3	1	3	5
ENSMUSG00000026796	FAM129B	0	0	1	0	0	0	1	0	1	0	0	0
ENSMUSG00000026797	STXBP1	2	2	2	1	0	2	10	6	9	10	4	10
ENSMUSG00000026798	COQ4	0	1	1	1	2	1	10	11	6	11	8	9
ENSMUSG00000026799	MED27	1	0	5	6	5	2	15	14	15	26	10	17
ENSMUSG00000026803	TTF1	8	8	12	11	10	9	33	24	23	40	22	27
ENSMUSG00000026805	BARHL1	0	0	0	0	0	0	1	0	0	0	0	0
ENSMUSG00000026806	DDX31	2	4	4	2	3	4	15	13	13	21	14	22
ENSMUSG00000026807	AK8	2	5	1	1	1	2	5	8	2	6	4	5
ENSMUSG00000026809	SPACA9	0	4	1	3	2	2	22	22	12	19	19	17
ENSMUSG00000026810	DPM2	7	9	6	7	8	8	34	41	45	41	25	35
ENSMUSG00000026811	ST6GALNAC6	7	10	11	11	8	5	37	43	31	42	31	32
ENSMUSG00000026812	TSC1	9	8	14	10	9	12	44	50	36	61	37	45
ENSMUSG00000026814	ENG	3	1	3	1	2	4	9	4	2	6	8	4
ENSMUSG00000026816	GTF3C5	0	2	2	1	3	0	6	17	17	14	10	8
ENSMUSG00000026817	AK1	0	0	0	0	0	2	2	0	0	0	1	1
ENSMUSG00000026818	CEL	85	86	107	106	108	97	400	451	301	416	310	348

ENSMUSG00000026819	SLC25A25	1	2	3	0	0	1	23	10	9	15	13	12
ENSMUSG00000026820	PTGES2	3	1	0	3	2	0	9	8	8	5	5	6
ENSMUSG00000026821	RALGDS	1	1	2	3	3	3	8	3	4	5	5	4
ENSMUSG00000026822	LCN2	1	0	0	0	0	0	0	0	1	0	0	0
ENSMUSG00000026826	NR4A2	6	6	5	7	7	6	22	28	22	37	27	18
ENSMUSG00000026827	GPD2	30	23	25	32	22	23	145	122	83	149	118	114
ENSMUSG00000026830	ERMN	0	0	0	0	0	0	0	0	0	0	0	1
ENSMUSG00000026831	1700007K13F	0	0	0	1	0	0	0	1	0	0	0	0
ENSMUSG00000026832	CYTIP	21	15	22	22	26	16	105	87	72	100	68	86
ENSMUSG00000026834	ACVR1C	1	0	0	0	1	0	1	1	0	0	1	2
ENSMUSG00000026836	ACVR1	1	5	5	7	5	1	20	22	16	31	14	27
ENSMUSG00000026839	UPP2	1	0	0	1	0	0	0	0	0	0	2	0
ENSMUSG00000026840	LAMC3	0	1	0	0	0	0	0	0	0	0	0	0
ENSMUSG00000026842	ABL1	4	8	9	9	6	8	40	26	24	34	32	32
ENSMUSG00000026843	FUBP3	13	15	20	19	19	19	128	115	86	130	89	97
ENSMUSG00000026848	TOR1B	29	35	34	43	43	35	154	116	101	144	104	112
ENSMUSG00000026849	TOR1A	4	9	3	7	11	6	46	36	31	44	30	30
ENSMUSG00000026851	BC005624	10	11	9	11	13	7	61	51	27	57	46	51
ENSMUSG00000026853	CRAT	3	5	2	6	1	6	7	7	5	13	4	8
ENSMUSG00000026854	USP20	10	4	3	5	6	2	38	36	21	22	26	19
ENSMUSG00000026856	DOLPP1	6	9	8	14	10	11	34	27	25	25	37	34
ENSMUSG00000026857	NTMT1	4	4	5	9	2	9	41	47	43	43	37	28
ENSMUSG00000026858	MIGA2	1	2	1	1	1	1	8	6	6	9	4	7
ENSMUSG00000026860	SH3GLB2	3	4	6	3	8	5	28	38	23	19	14	28
ENSMUSG00000026864	HSPA5	97	106	114	155	130	113	930	943	664	865	582	759
ENSMUSG00000026867	GAPVD1	18	24	16	15	22	13	85	130	78	117	82	91
ENSMUSG00000026869	PSMD5	4	7	5	3	4	9	29	28	18	29	24	28
ENSMUSG00000026872	ZEB2	0	0	0	1	0	0	0	0	0	0	1	0
ENSMUSG00000026873	PHF19	11	6	11	4	7	3	45	65	31	40	34	37
ENSMUSG00000026874	HC	0	1	0	0	0	0	0	0	0	0	0	1
ENSMUSG00000026875	TRAF1	0	0	0	0	0	0	0	1	0	0	0	0
ENSMUSG00000026878	RAB14	15	21	29	39	30	37	154	145	122	166	111	108
ENSMUSG00000026879	GSN	1	2	0	0	2	3	15	10	11	12	8	11
ENSMUSG00000026883	DAB2IP	1	2	4	3	7	4	7	12	12	9	12	16
ENSMUSG00000026885	TTLL11	0	0	0	0	0	0	0	0	0	1	0	0
ENSMUSG00000026887	MRRF	1	1	0	0	2	1	4	12	5	5	5	5
ENSMUSG00000026889	RBM18	5	6	5	9	6	7	51	54	35	43	33	40
ENSMUSG00000026893	GCA	4	1	2	2	0	3	13	12	11	19	3	9
ENSMUSG00000026894	MORN5	0	0	0	0	0	0	0	0	0	1	0	0
ENSMUSG00000026895	NDUFA8	11	15	15	7	20	14	97	115	72	114	90	96
ENSMUSG00000026896	IFIH1	0	1	1	0	1	1	1	6	4	3	3	1
ENSMUSG00000026914	PSMD14	21	29	24	33	33	21	215	170	122	225	154	130
ENSMUSG00000026915	STRBP	22	35	32	28	23	27	144	157	104	152	113	124
ENSMUSG00000026917	WDR5	23	19	19	18	18	20	106	105	77	114	87	85
ENSMUSG00000026918	BRD3	57	68	63	91	79	75	297	261	212	299	227	247
ENSMUSG00000026919	LCN4	1	1	2	3	3	6	6	9	5	7	8	8
ENSMUSG00000026921	EGFL7	0	0	0	0	0	0	1	0	1	0	1	0
ENSMUSG00000026922	AGPAT2	0	0	0	1	1	0	0	0	3	1	0	1
ENSMUSG00000026923	NOTCH1	0	0	0	0	0	0	0	0	1	0	2	0
ENSMUSG00000026924	SEC16A	3	10	7	8	14	11	43	53	36	49	40	35
ENSMUSG00000026925	INPP5E	2	3	4	8	2	1	24	18	8	17	19	19
ENSMUSG00000026926	PMPCA	24	22	18	10	15	19	119	124	85	93	81	105
ENSMUSG00000026927	SDCCAG3	19	33	34	34	33	40	127	104	77	131	72	117
ENSMUSG00000026928	CARD9	0	0	0	0	0	0	0	0	2	0	0	0
ENSMUSG00000026930	GPSM1	3	1	2	8	2	2	9	13	7	9	13	12
ENSMUSG00000026932	NACC2	0	1	0	1	0	0	0	1	0	1	0	0
ENSMUSG00000026933	CAMSAP1	8	6	2	6	8	9	27	31	14	31	26	28
ENSMUSG00000026941	MAMDC4	0	0	1	1	3	0	1	3	1	9	2	2

ENSMUSG00000026942	TRAF2	3	7	2	8	5	9	23	25	26	25	24	24
ENSMUSG00000026944	ABCA2	10	9	10	10	9	9	39	47	33	41	24	39
ENSMUSG00000026946	NMI	2	2	2	5	4	3	24	36	31	32	25	21
ENSMUSG00000026950	NEB	1	2	0	3	0	2	5	5	2	5	3	4
ENSMUSG00000026955	SAPCD2	4	10	9	6	10	4	38	32	32	40	20	35
ENSMUSG00000026956	UAP1L1	3	2	1	5	4	2	9	22	9	4	14	12
ENSMUSG00000026958	DPP7	0	0	0	1	0	0	1	1	0	1	0	1
ENSMUSG00000026959	GRIN1	0	0	0	0	0	0	0	1	0	0	0	0
ENSMUSG00000026960	ARL6IP6	15	15	4	10	16	11	154	166	126	215	88	114
ENSMUSG00000026963	TMEM210	0	0	0	0	0	0	0	0	0	0	1	0
ENSMUSG00000026965	ANAPC2	6	8	11	9	13	3	51	56	35	55	45	58
ENSMUSG00000026966	SSNA1	7	13	8	20	15	8	74	65	49	59	47	79
ENSMUSG00000026969	FAM166A	1	7	2	5	3	6	21	15	11	13	17	27
ENSMUSG00000026970	RBMS1	21	44	23	34	31	39	150	123	83	159	90	91
ENSMUSG00000026972	ARRDC1	3	8	4	8	8	9	28	29	23	35	17	21
ENSMUSG00000026974	ZMYND19	8	10	11	11	8	11	61	69	56	63	48	51
ENSMUSG00000026975	DPH7	2	0	5	3	2	2	14	11	7	20	6	14
ENSMUSG00000026977	MARCH7	8	22	13	13	19	22	112	132	102	162	106	90
ENSMUSG00000026979	PSD4	22	23	27	18	33	15	127	108	70	132	101	104
ENSMUSG00000026980	LY75	3	4	6	4	2	1	22	23	18	36	21	24
ENSMUSG00000026987	BAZ2B	7	17	9	20	11	12	64	78	59	93	59	71
ENSMUSG00000026988	WDSUB1	0	1	0	2	0	0	6	3	2	3	2	5
ENSMUSG00000026989	DAPL1	0	0	0	0	0	0	0	0	0	2	0	0
ENSMUSG00000026991	PKP4	6	12	10	11	14	9	57	57	39	68	51	43
ENSMUSG00000026994	GALNT3	0	1	1	5	2	4	3	4	3	5	4	1
ENSMUSG00000026999	NUP35	1	5	3	4	5	1	30	28	21	38	14	26
ENSMUSG00000027001	DUSP19	3	4	4	6	1	3	27	33	16	20	19	14
ENSMUSG00000027006	DNAJC10	13	22	14	21	17	13	44	56	36	60	47	44
ENSMUSG00000027007	SSFA2	20	28	27	22	23	25	157	160	112	171	94	97
ENSMUSG00000027009	ITGA4	2	6	4	2	3	4	14	15	9	15	11	9
ENSMUSG00000027010	SLC25A12	7	5	3	9	3	8	49	33	31	34	28	35
ENSMUSG00000027011	UBE2E3	8	13	12	18	23	14	115	91	81	141	75	87
ENSMUSG00000027012	DYNC1I2	19	34	24	32	29	35	113	119	87	152	90	103
ENSMUSG00000027014	CWC22	4	4	4	4	3	4	31	55	45	53	41	34
ENSMUSG00000027015	CYBRD1	0	0	0	0	0	0	0	0	1	0	0	0
ENSMUSG00000027018	HAT1	15	23	17	23	23	20	138	148	89	140	120	98
ENSMUSG00000027030	STK39	24	15	16	20	22	19	63	87	58	94	67	63
ENSMUSG00000027067	SSRP1	41	43	50	42	50	32	278	285	204	277	211	228
ENSMUSG00000027068	DHRS9	0	0	0	0	0	0	0	2	0	1	0	0
ENSMUSG00000027071	P2RX3	0	0	1	0	0	0	1	1	0	0	0	0
ENSMUSG00000027075	SLC43A1	0	0	0	0	0	0	0	0	0	0	1	0
ENSMUSG00000027076	TIMM10	1	6	12	9	7	7	40	48	29	29	24	36
ENSMUSG00000027079	CLP1	6	6	5	8	4	5	30	18	28	37	18	23
ENSMUSG00000027080	MED19	10	8	20	13	7	6	63	55	44	78	46	50
ENSMUSG00000027086	FASTKD1	0	7	7	7	5	6	27	27	19	25	21	24
ENSMUSG00000027087	ITGAV	5	7	9	6	9	6	42	37	29	29	20	30
ENSMUSG00000027088	PHOSPHO2	1	6	1	6	2	4	19	28	14	23	17	16
ENSMUSG00000027091	ZC3H15	53	81	54	97	66	61	360	385	275	382	303	338
ENSMUSG00000027099	MTX2	4	14	18	25	19	19	112	98	94	128	81	103
ENSMUSG00000027102	HOXD8	5	4	5	5	5	4	19	20	11	28	20	19
ENSMUSG00000027104	ATF2	10	9	14	16	9	13	81	65	61	65	57	61
ENSMUSG00000027108	OLA1	18	24	27	29	26	32	232	275	173	224	149	187
ENSMUSG00000027109	SP3	27	29	31	24	30	36	182	217	159	229	133	165
ENSMUSG00000027111	ITGA6	0	3	4	0	3	2	3	10	6	14	5	4
ENSMUSG00000027115	KIF18A	1	7	3	5	6	8	33	32	25	46	32	30
ENSMUSG00000027122	ARL14EP	14	18	19	12	23	13	60	67	52	75	53	62
ENSMUSG00000027130	SLC12A6	7	8	2	3	2	4	39	32	20	41	30	23
ENSMUSG00000027131	EMC4	0	2	0	4	1	0	15	17	9	19	12	10

ENSMUSG00000027132	KATNBL1	6	0	3	1	12	4	27	20	19	21	17	20
ENSMUSG00000027133	NOP10	21	21	26	27	37	31	182	219	164	181	142	165
ENSMUSG00000027134	LPCAT4	6	20	11	12	9	15	56	53	50	44	30	44
ENSMUSG00000027160	CCDC34	16	34	20	48	30	34	114	109	66	147	90	83
ENSMUSG00000027162	LIN7C	6	17	16	19	11	12	59	47	35	62	42	42
ENSMUSG00000027163	COMMD9	0	2	1	0	0	2	5	17	3	7	5	10
ENSMUSG00000027164	TRAF6	2	0	2	4	2	0	13	8	15	14	15	12
ENSMUSG00000027166	DNAJC24	2	2	1	2	3	0	5	11	9	5	6	6
ENSMUSG00000027167	ELP4	3	1	2	2	2	3	9	15	13	8	14	10
ENSMUSG00000027168	PAX6	0	0	0	0	1	0	1	0	0	0	0	1
ENSMUSG00000027170	EIF3M	26	31	23	22	29	23	179	209	122	173	157	133
ENSMUSG00000027171	PRRG4	3	2	1	0	0	5	8	11	6	8	6	5
ENSMUSG00000027173	DEPDC7	0	0	0	0	0	0	1	1	1	0	1	0
ENSMUSG00000027175	TCP11L1	0	0	0	0	1	0	0	0	0	1	0	0
ENSMUSG00000027176	CSTF3	5	12	12	14	15	9	46	67	40	71	46	46
ENSMUSG00000027177	HIPK3	1	6	4	3	3	3	26	21	16	18	9	15
ENSMUSG00000027180	FBXO3	8	10	7	16	14	11	86	62	50	76	53	49
ENSMUSG00000027184	CAPRIN1	16	32	27	39	23	28	154	192	119	174	106	151
ENSMUSG00000027185	NAT10	8	7	14	15	12	7	75	62	41	68	51	51
ENSMUSG00000027187	CAT	7	16	21	13	16	13	95	102	69	108	67	88
ENSMUSG00000027189	TRIM44	6	7	11	8	8	5	40	35	34	34	24	42
ENSMUSG00000027193	API5	6	6	7	9	13	10	51	53	47	52	48	37
ENSMUSG00000027194	TTC17	4	1	1	0	0	2	7	10	6	12	9	6
ENSMUSG00000027195	HSD17B12	15	25	22	20	24	15	110	114	87	134	87	95
ENSMUSG00000027196	ALKBH3OS1	0	0	0	0	0	0	0	0	0	0	1	0
ENSMUSG00000027198	EXT2	10	15	15	15	19	21	66	69	65	82	61	60
ENSMUSG00000027199	GATM	0	0	0	0	0	0	0	1	0	1	0	0
ENSMUSG00000027201	MYEF2	15	22	23	23	27	25	175	128	116	128	82	133
ENSMUSG00000027203	DUT	68	121	98	109	93	127	399	423	377	552	329	364
ENSMUSG00000027206	COPS2	9	15	15	14	26	11	145	137	89	158	91	85
ENSMUSG00000027207	GALK2	2	4	2	7	4	8	30	30	17	29	24	31
ENSMUSG00000027208	FGF7	0	0	0	1	1	0	2	2	0	1	0	1
ENSMUSG00000027209	FAM227B	1	2	1	0	1	0	7	2	4	2	7	6
ENSMUSG00000027210	MEIS2	0	0	1	0	0	0	0	1	0	0	0	0
ENSMUSG00000027215	CD82	5	15	13	10	11	11	45	56	33	54	42	49
ENSMUSG00000027219	SLC28A2	5	6	14	10	15	8	36	50	33	50	45	32
ENSMUSG00000027222	PEX16	3	1	0	0	1	1	4	5	5	5	5	5
ENSMUSG00000027223	MAPK8IP1	0	0	1	0	0	0	0	0	0	1	0	0
ENSMUSG00000027227	SORD	10	23	23	21	26	17	82	104	92	74	69	103
ENSMUSG00000027230	CREB3L1	2	0	0	0	0	1	2	3	3	7	4	5
ENSMUSG00000027236	EIF3J1	7	15	8	21	16	15	94	85	67	86	47	65
ENSMUSG00000027238	FRMD5	0	0	0	0	0	0	2	0	1	0	1	0
ENSMUSG00000027239	MDK	5	6	6	1	4	7	14	20	10	15	8	10
ENSMUSG00000027242	WDR76	23	33	26	37	30	25	147	175	107	160	108	127
ENSMUSG00000027243	HARBI1	1	0	1	4	4	0	10	12	5	12	4	6
ENSMUSG00000027244	ATG13	1	3	5	3	3	5	19	18	17	18	7	13
ENSMUSG00000027245	HYPK	8	12	12	11	6	11	55	76	32	74	44	64
ENSMUSG00000027247	ARHGAP1	1	6	5	3	4	3	33	26	13	26	23	34
ENSMUSG00000027248	PDIA3	44	70	55	74	71	61	315	364	269	346	237	278
ENSMUSG00000027253	LRP4	0	0	0	0	0	0	0	1	0	0	0	0
ENSMUSG00000027254	MAP1A	1	0	1	1	2	2	2	3	1	0	4	2
ENSMUSG00000027255	ARFGAP2	1	0	1	1	0	1	6	6	6	11	10	9
ENSMUSG00000027257	PACSIN3	0	0	0	0	0	0	1	0	0	1	0	2
ENSMUSG00000027259	ADAL	0	0	1	1	1	1	3	3	4	4	4	3
ENSMUSG00000027263	TUBGCP4	12	13	15	18	10	18	46	45	39	59	39	39
ENSMUSG00000027272	UBR1	9	18	15	15	11	19	87	70	58	68	51	64
ENSMUSG00000027273	SNAP25	1	0	0	0	0	0	0	0	0	0	0	0
ENSMUSG00000027274	MKKS	0	1	1	0	1	0	0	1	1	4	0	3

ENSMUSG00000027276	JAG1	6	14	6	15	11	6	31	31	29	31	27	31
ENSMUSG00000027281	SLX4IP	0	2	2	1	0	2	5	4	5	5	3	9
ENSMUSG00000027282	MTCH2	7	10	10	9	11	14	58	75	62	94	64	76
ENSMUSG00000027284	CDAN1	9	11	8	7	3	9	36	61	38	50	45	31
ENSMUSG00000027285	HAUS2	10	5	12	24	15	10	71	70	46	73	54	69
ENSMUSG00000027286	LRRC57	3	2	5	4	3	4	36	27	14	25	18	23
ENSMUSG00000027287	SNAP23	2	6	8	4	7	7	40	46	33	43	41	32
ENSMUSG00000027288	ZFP106	58	66	67	70	74	60	308	301	214	299	220	212
ENSMUSG00000027291	VPS39	2	7	6	9	4	9	38	31	27	36	23	23
ENSMUSG00000027293	EHD4	5	10	10	7	7	10	44	46	41	40	39	29
ENSMUSG00000027296	ITPKA	0	0	0	0	0	0	1	0	1	0	1	2
ENSMUSG00000027298	TYRO3	6	3	1	7	5	1	20	17	8	16	9	18
ENSMUSG00000027300	UBOX5	1	1	1	1	2	0	9	10	7	8	7	7
ENSMUSG00000027301	OXT	0	0	0	0	0	1	0	0	1	1	0	0
ENSMUSG00000027303	PTPRA	7	12	13	14	19	15	84	87	64	78	46	66
ENSMUSG00000027304	RTF1	47	162	55	126	68	96	305	292	230	308	249	246
ENSMUSG00000027305	NDUFAF1	1	2	6	0	1	2	14	10	7	16	19	11
ENSMUSG00000027306	NUSAP1	19	29	14	28	16	19	98	100	74	139	78	85
ENSMUSG00000027309	4930402H24[illegible]	0	4	1	1	1	1	7	3	3	4	3	8
ENSMUSG00000027312	ATRN	2	3	2	4	1	4	16	20	14	18	19	16
ENSMUSG00000027313	CHAC1	3	2	2	6	7	1	11	10	9	13	5	16
ENSMUSG00000027314	DLL4	0	0	0	0	0	0	1	0	0	0	0	1
ENSMUSG00000027318	ADAM33	0	0	0	0	0	0	0	0	0	1	0	0
ENSMUSG00000027322	SIGLEC1	1	1	0	0	0	0	0	0	0	0	0	0
ENSMUSG00000027323	RAD51	8	19	10	16	15	17	89	115	80	116	87	71
ENSMUSG00000027324	RPUSD2	1	5	1	5	2	8	12	5	10	15	6	12
ENSMUSG00000027326	KNL1	33	29	34	49	34	37	183	164	119	239	133	130
ENSMUSG00000027327	1700037H04[illegible]	1	3	3	4	7	3	22	19	13	24	15	11
ENSMUSG00000027329	SPEF1	0	1	1	0	0	0	1	3	0	0	1	1
ENSMUSG00000027330	CDC25B	2	6	2	8	4	2	25	15	16	31	20	18
ENSMUSG00000027331	KNSTRN	5	13	6	8	6	7	34	42	35	38	30	36
ENSMUSG00000027332	IVD	10	12	8	5	6	11	34	49	38	63	35	34
ENSMUSG00000027333	SMOX	0	3	1	0	0	0	5	2	0	0	3	2
ENSMUSG00000027339	RASSF2	24	30	24	29	36	31	150	138	115	124	111	136
ENSMUSG00000027340	SLC23A2	7	8	3	3	3	6	23	34	13	34	23	33
ENSMUSG00000027341	TMEM230	0	0	1	3	0	1	7	5	5	4	3	7
ENSMUSG00000027342	PCNA	10	13	14	16	20	14	119	155	103	115	106	111
ENSMUSG00000027346	GPCPD1	3	6	6	6	4	11	31	18	23	23	10	18
ENSMUSG00000027347	RASGRP1	41	42	51	56	68	42	208	224	168	218	189	182
ENSMUSG00000027349	FAM98B	16	16	14	16	13	22	93	100	72	92	69	70
ENSMUSG00000027351	SPRED1	24	16	16	20	16	23	58	54	49	60	39	40
ENSMUSG00000027353	MCM8	3	3	5	4	3	5	21	29	16	29	16	17
ENSMUSG00000027357	CRLS1	14	16	13	20	22	22	95	127	88	98	78	96
ENSMUSG00000027360	HDC	0	0	0	0	0	0	0	2	1	1	1	1
ENSMUSG00000027361	GABPB1	5	4	2	8	7	4	11	13	14	28	11	18
ENSMUSG00000027363	USP8	8	12	10	8	11	10	55	51	38	63	42	43
ENSMUSG00000027364	USP50	0	1	2	10	2	5	30	24	17	22	10	15
ENSMUSG00000027365	TRPM7	41	46	38	56	47	44	250	276	194	292	198	189
ENSMUSG00000027366	SPPL2A	11	16	12	8	6	13	83	78	31	91	42	47
ENSMUSG00000027367	STARD7	11	17	10	16	11	19	98	93	63	86	78	56
ENSMUSG00000027368	DUSP2	4	5	5	8	7	3	34	38	21	23	15	25
ENSMUSG00000027374	MRPS5	9	16	20	13	13	6	75	57	70	74	46	56
ENSMUSG00000027378	NPHP1	2	1	1	0	1	1	5	2	2	4	1	2
ENSMUSG00000027379	BUB1	6	9	5	10	13	9	91	58	52	68	53	43
ENSMUSG00000027380	ACOXL	1	1	1	0	2	2	4	11	3	7	5	5
ENSMUSG00000027381	BCL2L11	2	4	4	2	1	3	13	12	12	15	7	9
ENSMUSG00000027384	NDUFAF5	2	1	3	1	2	3	7	14	5	11	8	12
ENSMUSG00000027387	ZC3H8	5	6	7	7	8	5	36	36	29	34	38	22

ENSMUSG00000027394	TTL	1	1	1	1	1	1	6	10	5	7	8	6
ENSMUSG00000027395	POLR1B	5	12	1	8	5	4	33	34	29	33	26	29
ENSMUSG00000027397	SLC20A1	13	9	12	19	7	11	45	47	37	55	31	39
ENSMUSG00000027404	SNRPB	12	19	18	10	19	17	115	125	72	119	89	104
ENSMUSG00000027405	NOP56	91	131	127	155	140	137	716	743	592	814	671	593
ENSMUSG00000027406	IDH3B	12	24	13	18	16	13	99	107	69	97	84	93
ENSMUSG00000027408	CPXM1	0	0	0	1	0	0	0	2	2	0	0	2
ENSMUSG00000027411	VPS16	13	15	16	18	18	17	73	78	58	70	73	71
ENSMUSG00000027422	RRBP1	57	77	82	97	64	79	279	314	215	360	219	293
ENSMUSG00000027423	SNX5	30	41	33	45	46	33	239	265	184	226	174	182
ENSMUSG00000027424	MGME1	10	11	9	11	4	8	58	59	37	48	44	51
ENSMUSG00000027425	KAT14	5	5	5	1	4	6	18	21	11	20	14	13
ENSMUSG00000027427	POLR3F	3	6	3	4	2	2	22	24	15	26	16	18
ENSMUSG00000027428	RBBP9	0	0	0	0	0	0	0	1	0	0	0	0
ENSMUSG00000027429	SEC23B	7	18	10	10	17	11	68	83	79	103	76	77
ENSMUSG00000027430	DTD1	7	4	7	12	7	5	42	54	43	47	34	40
ENSMUSG00000027433	XRN2	26	23	30	20	28	20	167	179	128	215	127	137
ENSMUSG00000027439	GZF1	3	4	7	9	8	4	25	28	17	23	21	20
ENSMUSG00000027447	CST3	10	9	13	10	14	11	60	77	45	64	49	51
ENSMUSG00000027452	ACSS1	0	0	0	1	0	0	0	0	0	0	0	0
ENSMUSG00000027454	GINS1	10	1	4	5	8	4	36	52	25	37	24	35
ENSMUSG00000027455	NSFL1C	5	3	6	6	12	3	22	37	30	31	20	32
ENSMUSG00000027456	SDCBP2	0	1	1	0	0	0	0	0	0	1	1	0
ENSMUSG00000027459	FAM110A	3	0	0	1	1	1	7	10	10	7	13	7
ENSMUSG00000027460	ANGPT4	0	0	0	0	0	0	0	0	1	0	0	0
ENSMUSG00000027465	TBC1D20	7	4	5	6	6	13	49	46	41	55	40	44
ENSMUSG00000027466	RBCK1	9	9	5	7	13	16	48	53	45	70	40	60
ENSMUSG00000027469	TPX2	19	24	22	32	30	24	142	135	82	137	101	116
ENSMUSG00000027472	PDRG1	6	5	1	5	6	3	34	41	22	27	35	23
ENSMUSG00000027475	KIF3B	0	4	3	4	2	5	11	17	7	20	4	18
ENSMUSG00000027478	DNMT3B	4	7	3	4	3	6	34	39	29	33	29	33
ENSMUSG00000027479	MAPRE1	18	33	37	32	33	25	153	150	102	146	92	104
ENSMUSG00000027487	CDK5RAP1	0	1	0	1	0	0	6	9	4	7	8	6
ENSMUSG00000027489	NECAB3	3	1	0	0	0	2	10	19	9	11	8	9
ENSMUSG00000027490	E2F1	19	8	12	9	20	10	101	91	84	88	78	75
ENSMUSG00000027496	AURKA	2	2	4	3	3	3	22	16	13	11	9	8
ENSMUSG00000027498	CSTF1	1	4	2	0	2	0	6	6	6	16	11	10
ENSMUSG00000027502	RTF2	1	3	1	2	2	1	23	23	9	15	15	18
ENSMUSG00000027505	FAM209	0	0	0	0	0	0	0	0	0	0	1	0
ENSMUSG00000027506	TPD52	4	4	3	4	9	6	42	33	22	56	41	21
ENSMUSG00000027508	PAG1	4	5	16	5	8	11	37	31	28	46	21	26
ENSMUSG00000027509	RAE1	2	4	0	1	1	5	26	21	22	29	12	15
ENSMUSG00000027510	RBM38	8	9	7	12	11	10	64	72	57	66	51	61
ENSMUSG00000027519	RAB22A	1	6	4	2	6	2	9	15	10	22	12	11
ENSMUSG00000027520	ZDBF2	0	0	0	0	0	0	0	0	0	1	0	0
ENSMUSG00000027522	STX16	1	4	6	10	8	3	25	21	15	17	18	16
ENSMUSG00000027523	GNAS	69	95	86	167	119	108	617	702	526	593	524	610
ENSMUSG00000027531	IMPA1	4	5	6	7	9	5	55	56	37	59	30	37
ENSMUSG00000027534	SNX16	1	4	2	7	2	2	22	16	20	22	16	18
ENSMUSG00000027536	CHMP4C	0	0	0	0	0	1	1	1	1	3	3	1
ENSMUSG00000027540	PTPN1	6	12	9	10	17	9	51	55	30	56	33	53
ENSMUSG00000027544	NFATC2	1	2	4	3	3	0	13	11	6	10	5	15
ENSMUSG00000027546	ATP9A	1	0	0	2	0	0	5	1	3	2	3	3
ENSMUSG00000027550	LRRCC1	8	16	22	12	22	15	120	103	75	112	70	58
ENSMUSG00000027551	ZFP64	1	1	1	2	1	2	2	6	8	10	4	11
ENSMUSG00000027552	E2F5	7	3	10	8	7	5	49	56	31	46	40	37
ENSMUSG00000027555	CAR13	0	0	0	0	0	0	1	0	0	0	0	0
ENSMUSG00000027559	CAR3	0	0	1	0	0	0	4	3	1	2	1	1

ENSMUSG00000027562	CAR2	0	0	0	0	0	1	1	4	3	0	2	1
ENSMUSG00000027566	PSMA7	73	80	88	109	124	94	734	682	534	706	553	578
ENSMUSG00000027569	MRGBP	9	12	8	6	9	7	48	49	37	53	42	30
ENSMUSG00000027570	COL9A3	0	0	0	0	0	0	0	3	0	0	1	0
ENSMUSG00000027573	GID8	1	2	1	3	0	1	11	7	3	5	8	6
ENSMUSG00000027575	ARFGAP1	1	5	3	5	13	7	27	21	23	32	26	24
ENSMUSG00000027580	HELZ2	1	2	0	3	3	3	13	15	12	9	7	16
ENSMUSG00000027582	ZGPAT	0	0	0	2	1	0	4	3	0	1	4	4
ENSMUSG00000027583	ZBTB46	0	0	0	0	0	0	1	0	0	0	0	0
ENSMUSG00000027589	PCMTD2	2	3	1	4	3	3	15	24	10	24	13	12
ENSMUSG00000027593	RALY	27	27	22	39	28	41	166	179	136	161	136	165
ENSMUSG00000027597	AHCY	4	5	4	5	5	4	24	22	21	30	17	19
ENSMUSG00000027598	ITCH	13	15	13	10	10	13	68	85	56	90	59	71
ENSMUSG00000027599	ARMC1	5	20	9	13	14	9	65	53	41	62	45	53
ENSMUSG00000027601	MTFR1	10	15	13	17	9	18	81	91	73	81	63	56
ENSMUSG00000027602	MAP1LC3A	0	0	0	2	1	0	2	1	0	1	2	5
ENSMUSG00000027603	GGT7	0	0	0	0	0	0	0	0	1	0	1	0
ENSMUSG00000027605	ACSS2	2	4	5	10	4	4	25	24	16	28	24	37
ENSMUSG00000027606	DNAJC5B	0	1	0	0	0	0	0	0	0	0	0	0
ENSMUSG00000027610	GSS	2	4	4	0	4	3	31	17	19	25	13	16
ENSMUSG00000027611	PROCR	0	0	0	0	0	0	0	0	1	0	0	0
ENSMUSG00000027612	MMP24	0	1	1	0	2	1	4	8	3	7	5	8
ENSMUSG00000027613	EIF6	4	10	8	13	11	9	57	68	47	69	48	58
ENSMUSG00000027615	HPS3	8	6	3	8	9	7	26	19	28	38	22	23
ENSMUSG00000027618	NFS1	5	8	9	7	9	9	46	46	23	49	27	37
ENSMUSG00000027620	RBM39	110	228	161	197	180	129	736	888	553	859	614	564
ENSMUSG00000027624	EPB41L1	0	1	0	0	0	0	0	0	0	0	0	0
ENSMUSG00000027628	AAR2	0	3	0	1	2	0	13	13	8	13	5	10
ENSMUSG00000027630	TBL1XR1	16	11	11	13	12	9	39	41	33	45	21	52
ENSMUSG00000027634	NDRG3	7	10	10	6	7	11	35	46	30	38	28	49
ENSMUSG00000027635	DSN1	8	7	12	6	11	11	48	40	37	59	38	42
ENSMUSG00000027636	SLA2	4	10	7	9	6	5	44	48	38	36	39	38
ENSMUSG00000027637	1110008F13F	6	9	10	10	17	10	55	74	48	68	52	56
ENSMUSG00000027639	SAMHD1	24	21	18	18	21	20	115	120	87	125	69	99
ENSMUSG00000027641	RBL1	6	4	7	8	10	7	26	42	28	46	31	17
ENSMUSG00000027642	RPN2	18	21	16	14	17	18	117	110	86	154	81	81
ENSMUSG00000027646	SRC	0	0	0	0	0	0	0	0	1	0	0	0
ENSMUSG00000027649	CTNNBL1	18	19	20	26	25	18	113	115	120	141	93	119
ENSMUSG00000027650	TTI1	6	4	11	7	7	6	36	29	36	38	30	28
ENSMUSG00000027651	RPRD1B	5	6	12	14	7	7	58	60	45	57	43	39
ENSMUSG00000027652	RALGAPB	2	8	8	7	6	1	25	19	16	33	22	16
ENSMUSG00000027654	FAM83D	4	3	4	0	7	3	16	20	7	30	10	10
ENSMUSG00000027655	DHX35	3	5	3	1	1	1	11	12	10	13	11	18
ENSMUSG00000027660	SKIL	2	1	3	3	5	2	23	30	18	29	10	22
ENSMUSG00000027663	ZMAT3	5	5	8	7	4	3	28	23	13	33	16	15
ENSMUSG00000027665	PIK3CA	42	53	33	55	33	37	155	177	131	169	115	153
ENSMUSG00000027667	ZFP639	4	15	6	9	8	7	45	49	29	47	37	46
ENSMUSG00000027668	MFN1	7	7	7	5	5	2	27	44	21	27	25	21
ENSMUSG00000027669	GNB4	1	10	4	7	6	3	15	22	16	19	15	20
ENSMUSG00000027671	ACTL6A	18	12	13	5	13	12	108	124	86	104	76	80
ENSMUSG00000027673	NDUFB5	19	25	18	32	24	27	110	119	77	109	76	105
ENSMUSG00000027674	PEX5L	0	0	0	0	0	0	1	0	0	0	0	0
ENSMUSG00000027676	CCDC39	3	4	0	0	0	0	5	3	6	10	5	4
ENSMUSG00000027677	TTC14	15	30	13	35	11	16	132	128	96	128	82	85
ENSMUSG00000027678	NCOA3	2	4	4	6	3	5	23	23	13	22	13	25
ENSMUSG00000027679	DNAJC19	5	7	4	7	4	5	50	56	31	67	36	37
ENSMUSG00000027680	FXR1	19	29	28	42	31	18	117	124	87	138	80	101
ENSMUSG00000027684	MECOM	0	0	0	0	0	0	1	0	0	0	2	0

ENSMUSG00000027690	SLC2A2	0	0	0	0	1	0	1	0	1	1	0	0
ENSMUSG00000027692	TNIK	6	8	7	12	7	3	33	33	23	43	26	29
ENSMUSG00000027695	PLD1	1	3	0	0	0	1	14	7	7	6	5	0
ENSMUSG00000027698	NCEH1	5	1	4	4	1	3	18	25	14	25	17	17
ENSMUSG00000027699	ECT2	21	18	20	33	28	21	181	206	160	242	126	140
ENSMUSG00000027702	LRRC34	0	0	0	1	0	0	0	1	0	0	0	1
ENSMUSG00000027706	SEC62	126	153	110	250	151	134	403	367	281	422	345	371
ENSMUSG00000027708	DCUN1D1	5	10	7	8	10	3	43	41	46	45	19	31
ENSMUSG00000027709	MCCC1	1	2	6	3	2	6	16	24	13	24	5	24
ENSMUSG00000027710	ACAD9	0	1	2	1	4	1	10	9	2	12	9	10
ENSMUSG00000027712	ANXA5	2	1	1	0	0	1	13	5	9	8	9	6
ENSMUSG00000027713	1810062G17I	1	0	1	1	0	1	4	1	3	2	4	0
ENSMUSG00000027714	EXOSC9	10	9	5	21	6	8	77	71	38	63	46	58
ENSMUSG00000027715	CCNA2	14	22	24	21	18	17	88	131	87	126	78	109
ENSMUSG00000027716	TRPC3	0	3	0	0	0	0	0	0	0	0	1	0
ENSMUSG00000027720	IL2	5	6	4	13	19	8	56	49	29	47	31	55
ENSMUSG00000027722	SPATA5	4	2	5	2	4	3	14	19	15	11	12	12
ENSMUSG00000027737	SLC7A11	29	47	42	48	37	48	205	200	151	244	154	155
ENSMUSG00000027739	RAB33B	2	4	2	2	3	4	18	19	20	15	15	16
ENSMUSG00000027742	COG6	1	7	5	5	8	7	16	20	19	26	13	15
ENSMUSG00000027744	STOML3	0	0	0	0	0	0	0	0	0	1	0	0
ENSMUSG00000027746	UFM1	19	17	24	19	21	19	118	117	98	128	82	116
ENSMUSG00000027751	SUPT20	25	33	41	36	30	41	184	191	148	199	146	171
ENSMUSG00000027752	EXOSC8	45	42	54	61	98	44	294	298	253	313	266	257
ENSMUSG00000027763	MBNL1	74	88	83	104	91	80	365	338	245	384	207	252
ENSMUSG00000027770	DHX36	37	47	53	40	42	48	331	320	229	337	246	241
ENSMUSG00000027774	GFM1	10	14	15	14	21	14	98	106	97	115	71	94
ENSMUSG00000027775	MFSD1	2	4	4	4	4	3	13	18	10	24	10	11
ENSMUSG00000027778	IFT80	1	2	1	2	2	3	4	7	7	13	10	8
ENSMUSG00000027782	KPNA4	7	4	3	9	16	6	75	66	43	63	41	41
ENSMUSG00000027784	PPM1L	4	1	1	1	1	3	9	10	4	14	8	13
ENSMUSG00000027787	NMD3	15	18	24	19	20	29	80	105	63	101	84	79
ENSMUSG00000027800	TM4SF1	0	0	0	0	0	0	1	0	0	0	0	0
ENSMUSG00000027803	WWTR1	1	0	0	1	0	0	1	0	1	0	0	0
ENSMUSG00000027804	PPID	27	36	32	39	36	36	202	198	165	233	150	187
ENSMUSG00000027805	PFN2	1	2	2	2	1	3	16	16	8	7	6	13
ENSMUSG00000027806	TSC22D2	15	13	16	25	19	22	93	107	73	105	66	80
ENSMUSG00000027808	SERP1	24	28	31	46	41	32	206	191	165	248	121	179
ENSMUSG00000027809	ETFDH	1	6	3	5	5	0	26	26	14	26	20	32
ENSMUSG00000027810	EIF2A	15	24	28	24	29	37	152	149	116	140	118	114
ENSMUSG00000027811	4930579G24I	7	3	6	15	6	5	51	44	38	52	21	33
ENSMUSG00000027822	SLC33A1	5	7	9	3	7	6	68	47	36	82	50	63
ENSMUSG00000027823	GMPS	13	23	25	25	23	26	81	91	76	91	58	82
ENSMUSG00000027824	VMN2R1	0	0	0	0	0	0	0	0	0	1	1	0
ENSMUSG00000027827	KCNAB1	0	3	0	2	0	1	1	0	1	3	1	2
ENSMUSG00000027828	SSR3	139	149	140	179	177	154	441	544	356	480	368	404
ENSMUSG00000027829	CCNL1	46	80	88	79	84	84	556	612	420	604	450	425
ENSMUSG00000027834	SERPINI1	2	2	1	3	2	2	12	9	11	15	10	8
ENSMUSG00000027835	PDCD10	12	7	5	11	10	9	40	56	37	61	32	38
ENSMUSG00000027843	PTPN22	21	17	13	26	15	15	61	60	48	56	53	42
ENSMUSG00000027845	DCLRE1B	3	3	3	4	1	6	18	15	18	20	5	11
ENSMUSG00000027848	OLFML3	0	0	0	1	0	0	2	5	0	4	1	2
ENSMUSG00000027852	NRAS	20	20	18	31	17	21	108	104	50	90	70	86
ENSMUSG00000027854	SIKE1	7	7	4	4	8	10	35	29	24	30	23	21
ENSMUSG00000027855	SYCP1	0	0	0	0	0	0	0	0	0	2	0	0
ENSMUSG00000027860	VANGL1	0	0	0	0	1	0	2	1	0	0	0	0
ENSMUSG00000027861	CASQ2	0	0	0	1	2	1	0	0	0	0	0	0
ENSMUSG00000027863	CD2	1	0	1	0	0	0	1	1	0	0	0	1

ENSMUSG00000027864	PTGFRN	0	0	0	0	0	1	0	0	0	0	1	0
ENSMUSG00000027865	GDAP2	0	1	4	0	1	1	14	8	6	10	7	8
ENSMUSG00000027867	SPAG17	0	0	0	0	0	0	2	0	0	0	0	0
ENSMUSG00000027868	TBX15	0	0	0	0	0	0	0	0	1	0	0	0
ENSMUSG00000027878	NOTCH2	48	81	75	98	92	82	344	331	249	353	223	302
ENSMUSG00000027879	SEC22B	7	8	13	9	11	13	66	72	59	88	43	58
ENSMUSG00000027881	PRPF38B	61	78	52	94	83	74	505	459	329	596	365	375
ENSMUSG00000027882	STXBP3	2	1	5	1	3	2	26	20	13	22	18	16
ENSMUSG00000027883	GPSM2	6	6	2	4	2	2	37	32	31	44	27	21
ENSMUSG00000027884	CLCC1	9	12	14	10	14	7	57	51	40	63	35	51
ENSMUSG00000027889	AMPD2	18	36	21	29	35	21	98	118	91	120	94	110
ENSMUSG00000027890	GSTM4	0	0	2	1	0	0	1	2	0	2	2	3
ENSMUSG00000027893	AHCYL1	5	8	7	9	7	12	34	50	37	47	34	45
ENSMUSG00000027894	SLC6A17	0	0	0	1	0	0	0	0	0	0	0	0
ENSMUSG00000027896	SLC16A4	0	3	0	0	0	0	1	1	2	1	1	2
ENSMUSG00000027900	DRAM2	5	3	8	8	8	5	44	31	37	44	25	35
ENSMUSG00000027901	DENND2D	14	41	20	27	26	22	109	105	76	107	86	75
ENSMUSG00000027902	CHIL6	0	0	0	0	0	1	6	1	1	3	0	0
ENSMUSG00000027905	DDX20	4	13	14	10	12	11	51	50	56	55	46	53
ENSMUSG00000027907	S100A11	4	5	2	6	6	3	41	30	32	33	18	46
ENSMUSG00000027931	NPR1	0	0	0	0	0	0	0	0	0	1	0	0
ENSMUSG00000027932	SLC27A3	0	0	0	1	1	0	1	3	4	2	2	3
ENSMUSG00000027933	INTS3	8	8	9	7	8	6	38	36	40	44	24	31
ENSMUSG00000027935	RAB13	0	2	1	0	0	2	5	4	2	10	5	8
ENSMUSG00000027936	CRTC2	5	8	11	6	12	5	44	52	36	38	23	30
ENSMUSG00000027937	JTB	16	14	14	13	13	9	79	91	63	91	66	74
ENSMUSG00000027939	NUP210L	1	1	0	0	1	0	1	1	0	1	0	0
ENSMUSG00000027940	TPM3	53	57	60	86	70	63	400	456	311	439	373	385
ENSMUSG00000027942	4933434E20F	15	14	13	7	17	20	76	71	63	80	53	58
ENSMUSG00000027944	HAX1	2	1	3	1	6	1	16	15	10	11	3	11
ENSMUSG00000027947	IL6RA	6	6	5	7	7	7	20	24	19	21	13	6
ENSMUSG00000027950	CHRNB2	1	2	1	4	5	2	9	16	9	13	7	9
ENSMUSG00000027951	ADAR	8	7	11	12	8	6	51	48	37	46	24	34
ENSMUSG00000027952	PMVK	3	7	4	4	5	6	32	49	48	41	34	30
ENSMUSG00000027953	SLC50A1	3	3	4	4	3	13	40	48	28	38	30	33
ENSMUSG00000027955	FAM198B	0	1	1	1	1	0	3	4	1	4	4	0
ENSMUSG00000027957	SLC35A3	13	18	20	33	26	14	65	86	56	72	45	73
ENSMUSG00000027959	SASS6	16	18	19	27	15	23	98	95	68	111	86	78
ENSMUSG00000027961	LRRC39	3	2	4	3	2	2	18	14	17	20	15	16
ENSMUSG00000027963	EXTL2	3	2	3	4	3	4	16	26	10	21	9	16
ENSMUSG00000027965	OLFM3	13	15	18	16	18	22	55	61	43	53	43	48
ENSMUSG00000027967	NEUROG2	0	0	0	1	0	0	1	2	1	2	1	3
ENSMUSG00000027968	LARP7	24	34	27	35	43	30	165	177	109	168	112	124
ENSMUSG00000027977	NDST3	0	0	0	0	0	0	1	0	0	0	0	0
ENSMUSG00000027978	PRSS12	1	10	8	16	8	12	47	42	44	37	35	42
ENSMUSG00000027981	RNPC3	1	4	4	7	6	5	31	35	30	49	30	25
ENSMUSG00000027983	CYP2U1	3	0	3	1	7	0	5	6	5	7	1	6
ENSMUSG00000027984	HADH	26	35	22	28	22	29	176	180	121	149	119	152
ENSMUSG00000027985	LEF1	33	32	26	20	32	27	136	138	109	168	93	131
ENSMUSG00000027993	TRIM2	5	5	7	7	3	4	24	20	18	26	17	12
ENSMUSG00000027997	CASP6	0	0	3	3	4	0	11	9	5	7	5	5
ENSMUSG00000027998	PLRG1	8	7	3	6	6	7	44	52	34	45	27	40
ENSMUSG00000027999	PLA2G12A	4	5	9	7	7	8	37	38	28	37	24	37
ENSMUSG00000028004	NPY2R	0	0	0	1	0	0	0	0	0	0	0	0
ENSMUSG00000028005	GUCY1B1	0	0	0	0	0	0	0	0	0	0	1	0
ENSMUSG00000028008	ASIC5	0	0	0	0	0	0	1	0	0	0	0	0
ENSMUSG00000028010	GAR1	27	27	29	33	33	28	165	193	125	184	131	141
ENSMUSG00000028011	TDO2	0	0	0	0	0	0	0	0	0	1	0	0

ENSMUSG00000028012	RRH	0	0	1	0	0	2	0	2	1	0	0	0
ENSMUSG00000028013	PPA2	2	2	4	1	1	1	13	19	10	15	10	17
ENSMUSG00000028015	CTSO	0	2	1	0	2	1	11	12	8	8	7	8
ENSMUSG00000028016	INTS12	0	0	0	0	1	0	12	6	5	3	2	6
ENSMUSG00000028017	EGF	0	0	0	1	3	1	11	4	4	7	7	2
ENSMUSG00000028018	GSTCD	0	0	1	0	3	1	4	4	4	11	4	5
ENSMUSG00000028019	PDGFC	0	0	0	0	0	0	0	0	0	0	0	1
ENSMUSG00000028024	ENPEP	0	0	1	1	0	0	2	2	1	2	2	0
ENSMUSG00000028029	AIMP1	11	16	8	22	19	19	112	103	70	99	72	91
ENSMUSG00000028030	TBCK	0	3	0	4	0	1	10	8	5	5	9	6
ENSMUSG00000028031	DKK2	0	0	1	0	0	0	0	0	0	1	0	0
ENSMUSG00000028032	PAPSS1	6	1	4	5	2	6	35	34	31	28	14	26
ENSMUSG00000028033	KCNQ5	2	7	3	4	6	4	16	12	21	25	14	12
ENSMUSG00000028034	FUBP1	32	32	30	43	30	30	182	213	118	209	115	135
ENSMUSG00000028035	DNAJB4	2	2	5	1	3	4	20	11	10	26	12	17
ENSMUSG00000028042	ZBTB7B	3	1	2	2	4	0	6	13	6	4	4	10
ENSMUSG00000028044	CKS1B	14	27	24	24	26	22	203	143	108	154	125	128
ENSMUSG00000028047	THBS3	2	2	2	1	1	1	8	9	1	3	4	5
ENSMUSG00000028048	GBA	2	6	1	4	3	4	21	24	13	15	12	18
ENSMUSG00000028049	SCAMP3	6	8	6	6	3	9	53	35	29	37	23	27
ENSMUSG00000028051	HCN3	2	2	2	3	3	2	9	12	14	5	10	8
ENSMUSG00000028053	ASH1L	25	37	28	46	32	42	139	116	108	185	107	128
ENSMUSG00000028057	RIT1	2	5	3	3	1	0	18	21	15	19	10	16
ENSMUSG00000028059	ARHGEF2	21	28	42	35	33	21	131	160	92	158	94	117
ENSMUSG00000028060	2810403A07F	23	25	21	17	24	19	107	107	73	119	76	90
ENSMUSG00000028062	LAMTOR2	10	18	12	17	10	8	88	107	82	119	79	86
ENSMUSG00000028063	LMNA	1	0	3	5	0	3	2	5	4	6	3	3
ENSMUSG00000028064	SEMA4A	0	0	0	0	0	1	0	1	0	1	1	3
ENSMUSG00000028066	PMF1	5	13	13	13	16	12	71	79	53	68	36	59
ENSMUSG00000028068	IQGAP3	4	3	1	3	1	1	4	9	8	9	4	3
ENSMUSG00000028069	GPATCH4	32	30	32	41	37	36	202	215	208	224	199	205
ENSMUSG00000028070	NAXE	2	9	6	1	4	5	29	28	24	30	22	31
ENSMUSG00000028071	SH2D2A	0	0	0	0	0	0	0	1	1	0	0	0
ENSMUSG00000028072	NTRK1	0	1	0	0	0	0	0	0	0	0	0	0
ENSMUSG00000028073	PEAR1	9	18	10	18	14	16	70	68	56	62	47	58
ENSMUSG00000028076	CD1D1	0	1	2	0	0	1	3	3	0	1	6	3
ENSMUSG00000028078	DCLK2	1	0	1	2	0	1	4	7	3	4	4	1
ENSMUSG00000028080	LRBA	2	1	2	1	3	3	8	3	7	16	3	9
ENSMUSG00000028081	RPS3A1	224	230	244	291	284	269	1382	1448	1156	1304	1030	1176
ENSMUSG00000028085	GATB	3	2	2	0	0	2	7	6	11	12	2	8
ENSMUSG00000028086	FBXW7	22	36	25	38	33	40	84	126	73	136	92	76
ENSMUSG00000028088	FMO5	0	1	0	0	0	0	2	0	0	0	2	0
ENSMUSG00000028089	CHD1L	4	6	8	4	7	8	39	42	40	40	33	22
ENSMUSG00000028093	ACP6	5	7	5	14	6	6	21	25	22	23	23	21
ENSMUSG00000028096	GPR89	9	17	14	14	14	10	68	61	45	53	42	57
ENSMUSG00000028098	RNF115	5	6	6	12	8	5	46	50	31	44	40	33
ENSMUSG00000028099	POLR3C	2	7	6	15	8	7	36	43	35	47	30	36
ENSMUSG00000028100	NUDT17	0	0	0	0	0	0	0	0	0	0	1	1
ENSMUSG00000028101	PIAS3	2	4	3	3	2	5	19	20	15	18	15	15
ENSMUSG00000028102	PEX11B	1	3	0	0	5	1	9	11	15	8	8	6
ENSMUSG00000028104	POLR3GL	9	9	9	4	7	8	40	38	34	45	44	24
ENSMUSG00000028106	RPRD2	8	24	17	21	23	14	92	87	78	94	83	105
ENSMUSG00000028107	TARS2	5	9	10	16	16	16	40	65	28	41	25	34
ENSMUSG00000028108	ECM1	0	0	0	1	0	0	1	3	5	6	4	3
ENSMUSG00000028111	CTSK	0	0	0	0	0	1	1	0	0	1	2	0
ENSMUSG00000028114	METTL14	4	5	6	1	4	2	39	27	26	29	23	19
ENSMUSG00000028124	GCLM	4	4	5	12	11	6	49	65	22	48	39	44
ENSMUSG00000028126	PIP5K1A	12	14	12	23	22	28	103	115	95	136	75	96

ENSMUSG00000028127	ABCD3	1	7	4	5	12	7	18	37	21	29	18	25
ENSMUSG00000028133	RWDD3	0	1	0	2	1	1	5	0	2	3	1	1
ENSMUSG00000028134	PTBP2	10	6	5	12	13	12	59	55	46	61	52	52
ENSMUSG00000028136	SNX27	15	27	20	33	29	19	85	69	52	83	50	54
ENSMUSG00000028137	CELF3	0	0	0	0	0	0	1	1	0	0	0	1
ENSMUSG00000028138	ADH5	19	27	26	27	27	19	142	155	106	172	125	130
ENSMUSG00000028139	RIIAD1	2	0	0	2	1	0	3	1	2	5	7	2
ENSMUSG00000028140	MRPL9	8	4	13	17	9	9	51	65	40	70	57	45
ENSMUSG00000028141	OAZ3	0	0	0	0	0	1	1	1	1	3	1	2
ENSMUSG00000028145	THEM4	1	0	1	1	0	0	3	7	7	4	5	8
ENSMUSG00000028149	RAP1GDS1	10	11	5	16	15	9	41	40	42	43	31	48
ENSMUSG00000028150	RORC	0	1	0	1	0	1	0	0	0	0	0	0
ENSMUSG00000028152	TSPAN5	2	6	1	5	2	4	12	13	14	13	12	13
ENSMUSG00000028156	EIF4E	4	13	15	15	22	12	96	69	56	73	62	66
ENSMUSG00000028158	MTTP	3	1	5	8	5	2	12	11	9	17	15	9
ENSMUSG00000028159	DAPP1	4	4	0	4	1	3	21	20	10	14	15	9
ENSMUSG00000028161	PPP3CA	137	201	180	210	182	216	775	804	547	776	569	568
ENSMUSG00000028163	NFKB1	11	22	19	35	31	27	133	137	101	139	67	106
ENSMUSG00000028164	MANBA	5	6	4	5	8	2	33	19	19	22	29	22
ENSMUSG00000028165	CISD2	14	20	17	20	21	17	87	75	51	101	58	70
ENSMUSG00000028167	BDH2	1	0	0	0	0	0	0	0	0	0	0	0
ENSMUSG00000028174	RPE65	0	0	0	0	0	0	1	0	0	0	0	0
ENSMUSG00000028175	DEPDC1A	0	0	0	4	1	3	11	8	8	7	3	6
ENSMUSG00000028180	ZRANB2	54	79	57	92	70	65	388	359	268	427	324	351
ENSMUSG00000028182	LRRIQ3	0	0	0	0	0	1	0	0	1	0	0	1	0
ENSMUSG00000028184	ADGRL2	1	0	0	1	2	3	6	12	9	12	3	6
ENSMUSG00000028185	DNASE2B	0	0	0	0	0	0	1	0	0	1	0	1
ENSMUSG00000028187	RPF1	7	13	7	15	13	8	54	59	50	71	57	51
ENSMUSG00000028188	SPATA1	0	2	2	2	1	3	8	8	5	6	7	4
ENSMUSG00000028189	CTBS	0	1	0	0	1	0	6	3	3	4	0	3
ENSMUSG00000028191	BCL10	6	13	10	8	12	7	54	56	39	60	44	57
ENSMUSG00000028199	CRYZ	1	0	0	0	0	0	1	5	2	2	0	2
ENSMUSG00000028207	ASPH	0	0	0	0	0	0	2	1	0	1	0	0
ENSMUSG00000028211	TRP53INP1	3	2	1	4	1	0	8	6	12	15	6	4
ENSMUSG00000028212	CCNE2	11	17	18	16	11	14	50	56	47	60	48	57
ENSMUSG00000028214	GEM	11	9	5	10	4	8	46	49	27	51	29	31
ENSMUSG00000028217	CDH17	1	1	0	0	1	0	0	0	0	0	1	1
ENSMUSG00000028218	FAM92A	2	3	10	8	6	6	50	40	40	49	28	40
ENSMUSG00000028221	TMEM55A	7	3	4	6	7	9	31	32	27	40	23	43
ENSMUSG00000028223	DECR1	2	5	2	5	4	3	16	19	23	16	11	5
ENSMUSG00000028224	NBN	6	7	7	6	10	6	42	52	39	52	47	25
ENSMUSG00000028228	CPNE3	7	12	10	10	9	8	44	60	41	52	30	40
ENSMUSG00000028229	RMDN1	2	2	4	5	0	0	6	12	9	7	6	10
ENSMUSG00000028232	TMEM68	3	1	0	3	1	3	8	9	13	11	8	5
ENSMUSG00000028233	TGS1	16	16	16	25	26	11	99	87	63	101	62	79
ENSMUSG00000028234	RPS20	177	185	210	207	223	200	853	857	680	925	688	724
ENSMUSG00000028243	UBXN2B	1	0	2	2	1	0	6	6	2	16	5	5
ENSMUSG00000028245	NSMAF	5	4	6	5	7	5	29	26	26	30	16	17
ENSMUSG00000028247	COQ3	1	1	0	2	1	1	8	7	3	4	5	6
ENSMUSG00000028248	PNISR	57	72	51	60	64	65	363	338	257	375	273	293
ENSMUSG00000028249	SDCBP	2	8	16	9	10	12	46	49	38	58	32	35
ENSMUSG00000028251	TSTD3	5	1	3	2	3	2	20	18	9	22	11	14
ENSMUSG00000028252	CCNC	13	17	12	17	13	15	105	95	65	93	65	88
ENSMUSG00000028256	ODF2L	5	10	8	12	15	7	35	39	32	73	30	42
ENSMUSG00000028261	NDUFAF4	18	23	18	26	23	23	159	136	107	141	120	118
ENSMUSG00000028266	LMO4	16	28	17	24	30	21	78	93	55	92	65	75
ENSMUSG00000028268	GBP3	0	1	1	3	2	0	2	6	7	9	4	3
ENSMUSG00000028270	GBP2	0	0	1	0	2	0	0	0	0	0	0	3

ENSMUSG00000028271	GTF2B	11	9	6	11	13	8	67	66	50	81	51	62
ENSMUSG00000028273	PDLIM5	14	14	22	8	15	6	90	83	74	101	66	71
ENSMUSG00000028274	RNGTT	6	5	7	10	5	5	58	54	31	61	38	31
ENSMUSG00000028277	UBE2J1	3	4	5	10	1	5	23	27	21	33	22	26
ENSMUSG00000028278	RRAGD	0	0	0	1	1	0	0	0	0	1	0	2
ENSMUSG00000028282	CASP8AP2	43	41	46	54	41	49	192	185	133	216	135	165
ENSMUSG00000028284	MAP3K7	9	4	3	11	6	10	38	35	36	42	30	28
ENSMUSG00000028291	AKIRIN2	18	17	19	34	30	17	112	117	78	138	85	100
ENSMUSG00000028292	RARS2	2	2	1	1	1	4	21	14	7	16	15	14
ENSMUSG00000028293	SLC35A1	5	5	3	7	4	4	24	17	14	27	16	15
ENSMUSG00000028294	CFAP206	0	0	0	0	0	0	0	0	0	0	0	1
ENSMUSG00000028295	SMIM8	2	1	1	2	1	3	21	18	11	19	19	18
ENSMUSG00000028300	3110043O21I	0	1	2	0	1	0	3	7	3	7	6	2
ENSMUSG00000028309	RNF20	21	22	24	25	29	24	119	119	89	124	100	108
ENSMUSG00000028312	SMC2	18	28	32	42	39	38	187	161	97	199	118	114
ENSMUSG00000028318	POLR1E	2	2	0	5	2	4	17	26	17	19	17	16
ENSMUSG00000028322	EXOSC3	10	9	10	5	14	8	51	67	59	45	39	53
ENSMUSG00000028327	STRA6L	0	0	0	0	0	0	0	0	0	0	1	0
ENSMUSG00000028328	TMOD1	2	4	9	0	6	16	27	22	18	31	6	21
ENSMUSG00000028329	XPA	1	4	4	6	3	9	37	44	27	38	39	33
ENSMUSG00000028330	NCBP1	10	8	10	5	12	13	54	67	36	62	56	59
ENSMUSG00000028331	TRMO	0	1	0	0	0	0	3	1	2	7	3	3
ENSMUSG00000028332	HEMGN	0	0	0	0	0	0	0	0	0	0	1	0
ENSMUSG00000028333	ANP32B	69	89	101	143	122	96	565	546	385	472	430	461
ENSMUSG00000028334	NANS	10	10	15	21	17	7	67	86	51	64	53	55
ENSMUSG00000028337	CORO2A	6	10	11	9	14	9	67	79	50	69	53	65
ENSMUSG00000028339	COL15A1	0	0	0	0	0	0	0	1	1	0	0	0
ENSMUSG00000028341	NR4A3	0	0	0	0	0	0	0	0	0	0	1	0
ENSMUSG00000028343	ERP44	7	7	10	7	11	6	41	53	37	64	43	36
ENSMUSG00000028344	INVS	3	5	2	0	2	4	16	18	11	9	9	13
ENSMUSG00000028345	TEX10	6	18	11	14	11	16	57	54	57	72	43	57
ENSMUSG00000028348	CAVIN4	0	0	0	0	0	0	0	0	0	0	1	0
ENSMUSG00000028356	AMBP	0	0	0	0	0	0	2	2	2	4	0	0
ENSMUSG00000028357	KIF12	0	0	0	0	0	0	0	1	0	0	0	0
ENSMUSG00000028358	ZFP618	0	0	0	0	0	0	0	1	0	0	0	0
ENSMUSG00000028367	TXN1	25	41	31	59	43	44	292	288	249	277	204	243
ENSMUSG00000028381	UGCG	45	66	68	77	70	95	325	309	227	345	232	250
ENSMUSG00000028382	PTBP3	22	31	23	22	28	19	137	121	98	156	90	95
ENSMUSG00000028383	HSDL2	5	4	6	8	5	8	35	32	33	47	29	48
ENSMUSG00000028385	SNX30	2	6	3	2	7	3	12	10	14	8	6	17
ENSMUSG00000028386	SLC46A2	0	0	0	0	0	1	1	0	0	0	0	0
ENSMUSG00000028391	WDR31	0	0	0	1	0	1	1	0	0	2	0	0
ENSMUSG00000028393	ALAD	1	2	1	4	2	1	11	14	8	12	15	16
ENSMUSG00000028394	POLE3	6	5	14	12	5	7	58	58	49	69	43	54
ENSMUSG00000028397	KDM4C	0	7	8	9	2	1	23	32	17	25	21	14
ENSMUSG00000028398	DMAC1	9	5	10	9	17	16	63	60	60	63	41	56
ENSMUSG00000028399	PTPRD	0	0	0	0	0	0	0	0	1	0	0	0
ENSMUSG00000028402	MPDZ	0	1	0	0	0	0	1	0	0	0	0	0
ENSMUSG00000028403	ZDHHC21	8	5	7	8	6	11	25	21	18	33	25	17
ENSMUSG00000028405	ACO1	3	9	4	6	5	1	23	22	13	18	11	13
ENSMUSG00000028407	SMIM27	4	2	0	6	7	3	23	25	22	23	30	20
ENSMUSG00000028409	SMU1	15	13	9	18	9	15	74	100	60	77	60	75
ENSMUSG00000028410	DNAJA1	17	18	26	28	17	22	128	130	104	153	87	112
ENSMUSG00000028411	APTX	0	1	2	3	0	0	7	6	1	7	7	6
ENSMUSG00000028413	B4GALT1	7	6	8	12	5	4	37	31	27	40	29	26
ENSMUSG00000028414	FKTN	3	3	3	3	4	2	10	5	9	12	14	9
ENSMUSG00000028416	BAG1	9	20	6	13	10	14	108	89	67	105	62	89
ENSMUSG00000028417	TAL2	0	0	0	0	0	0	0	0	0	1	0	0

ENSMUSG00000028419	CHMP5	10	8	6	10	6	5	67	60	48	73	40	42
ENSMUSG00000028423	NFX1	3	6	14	15	10	15	70	71	43	93	46	48
ENSMUSG00000028426	RAD23B	18	30	17	31	21	25	122	137	62	159	93	91
ENSMUSG00000028430	NOL6	5	2	2	7	3	4	26	32	28	23	20	33
ENSMUSG00000028431	IKBKAP	6	7	10	5	8	5	27	34	25	26	24	25
ENSMUSG00000028433	UBAP2	5	18	10	16	16	11	71	86	46	75	48	70
ENSMUSG00000028436	DCAF12	5	5	11	4	4	4	60	48	28	36	43	31
ENSMUSG00000028437	UBAP1	3	2	9	2	6	2	17	20	16	23	13	16
ENSMUSG00000028438	KIF24	0	1	1	2	1	3	5	3	2	10	1	5
ENSMUSG00000028439	FAM219A	0	0	1	1	1	1	10	5	8	8	1	9
ENSMUSG00000028441	1110017D15R	0	0	0	0	0	0	0	0	0	0	1	0
ENSMUSG00000028443	NUDT2	0	1	2	0	1	2	13	13	6	19	10	6
ENSMUSG00000028445	ENHO	0	0	0	0	0	0	0	0	0	1	0	0
ENSMUSG00000028447	DCTN3	9	1	7	6	5	7	43	43	29	47	26	35
ENSMUSG00000028451	1700022I11R	0	0	0	1	0	0	2	1	2	0	2	3
ENSMUSG00000028452	VCP	28	36	33	32	28	55	188	184	156	204	149	159
ENSMUSG00000028453	FANCG	0	2	2	1	1	2	13	8	6	11	5	10
ENSMUSG00000028454	PIGO	2	2	2	2	0	2	10	9	6	10	8	8
ENSMUSG00000028455	STOML2	11	9	18	20	20	6	66	69	62	90	72	65
ENSMUSG00000028456	UNC13B	0	0	0	0	0	0	0	0	0	0	0	1
ENSMUSG00000028458	TESK1	1	0	0	3	1	1	12	11	8	13	9	5
ENSMUSG00000028459	CD72	0	0	0	0	0	0	1	0	0	2	1	0
ENSMUSG00000028461	CCDC107	8	9	6	10	9	5	51	57	39	40	38	56
ENSMUSG00000028465	TLN1	18	22	15	26	29	32	95	116	121	114	92	117
ENSMUSG00000028466	CREB3	2	4	3	5	4	6	17	9	12	9	7	13
ENSMUSG00000028467	GBA2	2	2	4	1	3	5	7	14	4	6	6	9
ENSMUSG00000028468	RGP1	3	0	4	1	0	3	10	12	7	11	6	11
ENSMUSG00000028470	HINT2	1	1	0	2	1	1	5	9	11	11	7	10
ENSMUSG00000028476	RECK	1	0	0	1	0	1	2	1	1	2	0	0
ENSMUSG00000028478	CLTA	4	18	24	15	17	20	104	106	79	143	75	99
ENSMUSG00000028479	GNE	4	8	6	5	1	4	15	21	19	18	16	19
ENSMUSG00000028480	GLIPR2	0	0	1	1	0	2	5	4	2	3	3	4
ENSMUSG00000028483	SNAPC3	4	2	1	11	8	8	33	28	23	42	18	21
ENSMUSG00000028484	PSIP1	26	43	39	50	41	40	246	209	164	275	161	207
ENSMUSG00000028495	RPS6	24	37	28	42	34	43	145	161	109	150	111	106
ENSMUSG00000028496	MLLT3	3	4	4	2	3	4	7	7	8	15	8	9
ENSMUSG00000028497	HACD4	0	0	0	0	0	0	0	0	0	0	1	0
ENSMUSG00000028514	USP24	10	7	5	15	9	10	38	38	29	38	20	32
ENSMUSG00000028521	SLC35D1	4	1	3	4	7	9	19	18	10	14	18	18
ENSMUSG00000028522	MIER1	28	19	34	36	37	18	103	120	81	107	81	100
ENSMUSG00000028524	SGIP1	0	0	0	0	0	0	0	1	0	0	0	0
ENSMUSG00000028525	PDE4B	3	3	4	2	2	1	23	24	10	32	20	11
ENSMUSG00000028527	AK4	2	3	6	1	3	1	12	11	15	16	8	11
ENSMUSG00000028530	JAK1	13	24	16	16	28	23	107	119	88	123	73	96
ENSMUSG00000028538	ST3GAL3	1	3	0	1	2	2	6	7	4	5	7	8
ENSMUSG00000028540	DPH2	5	5	3	2	4	4	28	18	10	23	20	21
ENSMUSG00000028541	B4GALT2	0	1	0	0	2	0	1	0	0	0	0	0
ENSMUSG00000028542	SLC6A9	4	6	8	7	7	8	27	22	17	35	16	21
ENSMUSG00000028549	ITGB3BP	3	3	5	4	4	2	23	22	26	24	13	27
ENSMUSG00000028551	CDKN2C	2	2	2	2	1	5	18	9	7	17	9	7
ENSMUSG00000028552	EPS15	2	13	8	8	5	5	62	51	34	63	48	33
ENSMUSG00000028553	ANGPTL3	0	0	0	0	0	0	0	0	0	0	0	1
ENSMUSG00000028555	TTC39A	1	0	0	0	1	0	4	2	1	7	2	2
ENSMUSG00000028557	RNF11	8	2	4	7	12	10	44	55	27	52	31	39
ENSMUSG00000028559	OSBPL9	5	6	6	9	6	5	18	27	19	23	28	24
ENSMUSG00000028560	USP1	17	20	14	24	20	13	108	87	70	114	67	83
ENSMUSG00000028563	TM2D1	1	1	3	2	0	3	30	13	16	15	19	16
ENSMUSG00000028565	NFIA	13	18	7	17	17	15	37	23	24	51	22	25

ENSMUSG00000028567	TXNDC12	3	5	6	2	4	3	17	13	15	17	11	15
ENSMUSG00000028568	BTF3L4	1	2	0	1	0	4	6	7	5	14	6	7
ENSMUSG00000028572	HOOK1	0	0	0	0	0	0	1	0	0	1	0	0
ENSMUSG00000028573	FGGY	0	3	0	1	2	1	16	12	9	10	12	7
ENSMUSG00000028575	EQTN	1	2	0	0	0	0	3	0	1	1	1	0
ENSMUSG00000028576	IFT74	3	4	5	8	3	5	43	33	23	33	20	24
ENSMUSG00000028577	PLAA	24	26	19	23	26	27	143	136	82	145	92	112
ENSMUSG00000028578	CAAP1	3	6	5	5	4	2	35	40	21	44	20	23
ENSMUSG00000028580	PUM1	23	32	29	28	24	21	141	126	98	163	84	129
ENSMUSG00000028581	LAPTM5	31	35	28	38	33	25	189	182	123	193	138	170
ENSMUSG00000028582	CC2D1B	6	5	2	12	7	7	38	42	19	29	16	28
ENSMUSG00000028587	ORC1	7	5	2	7	9	5	39	31	24	53	24	26
ENSMUSG00000028599	TNFRSF1B	0	0	0	0	1	2	1	3	1	2	0	2
ENSMUSG00000028603	SCP2	2	3	2	4	4	4	31	50	28	35	24	28
ENSMUSG00000028607	CPT2	2	0	2	2	2	0	20	24	18	17	14	12
ENSMUSG00000028608	0610037L13R	4	3	0	2	1	2	15	11	8	12	12	14
ENSMUSG00000028609	MAGOH	23	24	37	35	29	26	176	201	126	169	133	130
ENSMUSG00000028613	LRP8	3	2	4	4	5	6	29	11	9	20	13	16
ENSMUSG00000028614	NDC1	3	8	13	12	8	13	39	50	29	50	24	35
ENSMUSG00000028617	LRRC42	5	12	6	6	7	10	46	43	24	51	32	34
ENSMUSG00000028618	TMEM59	17	18	14	16	21	9	105	96	89	128	79	83
ENSMUSG00000028619	TCEANC2	2	1	2	3	4	2	13	16	11	18	7	11
ENSMUSG00000028621	CYB5RL	0	0	1	0	0	0	4	3	3	4	3	6
ENSMUSG00000028622	MRPL37	4	6	3	8	6	3	27	24	12	34	21	21
ENSMUSG00000028629	EXO5	1	1	2	6	4	0	14	14	7	11	12	15
ENSMUSG00000028630	DYRK2	45	38	36	24	35	39	107	129	103	142	88	102
ENSMUSG00000028633	CTPS	10	15	13	8	12	12	62	88	61	75	57	71
ENSMUSG00000028634	HIVEP3	0	2	1	0	2	1	2	6	4	9	2	4
ENSMUSG00000028636	PPCS	1	3	0	1	1	0	9	6	3	2	5	6
ENSMUSG00000028637	CCDC30	0	0	0	0	0	0	0	0	1	0	0	0
ENSMUSG00000028639	YBX1	192	270	197	246	246	202	2153	1849	1382	2131	1490	1559
ENSMUSG00000028641	P3H1	0	2	2	2	0	1	8	5	3	2	9	7
ENSMUSG00000028643	SVBP	1	4	4	9	6	9	28	33	25	25	25	30
ENSMUSG00000028645	SLC2A1	10	12	15	16	17	11	87	118	68	91	53	59
ENSMUSG00000028646	RRAGC	6	13	10	12	6	5	48	52	42	45	34	41
ENSMUSG00000028647	MYCBP	4	12	7	14	11	15	84	85	65	65	52	62
ENSMUSG00000028648	NDUFS5	2	1	1	1	3	0	12	11	14	21	14	14
ENSMUSG00000028649	MACF1	93	90	82	141	126	106	438	453	342	468	373	398
ENSMUSG00000028651	PPIE	8	12	6	11	16	7	51	47	37	59	46	39
ENSMUSG00000028653	TRIT1	10	4	7	4	4	9	19	32	17	35	20	22
ENSMUSG00000028655	MFSD2A	1	0	0	1	0	0	0	0	0	0	0	0
ENSMUSG00000028656	CAP1	60	79	83	83	92	70	291	319	261	302	248	285
ENSMUSG00000028657	PPT1	63	68	80	112	76	70	175	203	158	199	136	175
ENSMUSG00000028668	ELOA	12	10	20	21	19	18	100	86	62	104	68	80
ENSMUSG00000028669	PITHD1	1	4	5	0	1	1	8	7	10	13	6	8
ENSMUSG00000028670	LYPLA2	13	6	8	6	17	7	45	50	33	45	28	55
ENSMUSG00000028671	GALE	3	2	7	3	4	2	19	15	8	13	9	12
ENSMUSG00000028672	HMGCL	4	0	2	1	0	0	13	18	13	14	12	15
ENSMUSG00000028673	FUCA1	2	1	0	2	1	0	8	8	7	13	11	7
ENSMUSG00000028675	PNRC2	10	15	8	11	8	7	81	72	50	88	62	44
ENSMUSG00000028676	SRSF10	28	45	32	60	49	47	201	193	166	222	163	169
ENSMUSG00000028677	RNF220	9	13	10	20	16	15	58	74	40	84	57	57
ENSMUSG00000028678	KIF2C	15	12	14	9	13	12	73	59	39	89	46	64
ENSMUSG00000028680	PLK3	0	0	0	0	0	0	0	5	1	2	0	1
ENSMUSG00000028681	PTCH2	0	0	0	0	0	0	0	1	0	0	0	2
ENSMUSG00000028683	EIF2B3	2	3	3	5	6	3	23	31	22	23	20	15
ENSMUSG00000028684	UROD	2	5	4	6	4	4	33	28	20	22	24	28
ENSMUSG00000028687	MUTYH	3	3	1	0	1	2	6	13	4	15	6	8

ENSMUSG00000028688	TOE1	3	7	4	1	7	4	39	38	18	37	39	24
ENSMUSG00000028689	CCDC163	5	4	8	5	15	3	29	28	30	22	16	31
ENSMUSG00000028690	MMACHC	2	2	4	1	3	1	10	7	13	8	10	11
ENSMUSG00000028691	PRDX1	68	70	91	86	74	79	505	548	365	491	374	433
ENSMUSG00000028692	AKR1A1	24	40	22	30	44	31	185	192	140	152	114	167
ENSMUSG00000028693	NASP	35	25	43	55	44	37	272	260	203	268	210	189
ENSMUSG00000028696	IPP	3	3	2	3	2	2	12	11	12	6	10	9
ENSMUSG00000028698	PIK3R3	1	1	0	1	0	0	1	0	1	5	4	3
ENSMUSG00000028700	POMGNT1	3	16	6	6	5	4	26	25	26	25	31	22
ENSMUSG00000028701	LURAP1	0	1	1	0	1	1	0	0	0	1	1	1
ENSMUSG00000028702	RAD54L	13	15	18	16	18	16	81	69	43	67	51	65
ENSMUSG00000028703	LRRC41	9	6	4	17	6	8	28	24	34	37	28	32
ENSMUSG00000028706	NSUN4	0	1	4	1	4	7	25	16	19	22	18	16
ENSMUSG00000028707	DMBX1	0	0	0	0	0	0	0	0	1	0	0	0
ENSMUSG00000028708	MKNK1	2	4	0	5	5	5	17	27	18	26	11	15
ENSMUSG00000028709	MOB3C	3	7	0	4	0	0	13	11	6	10	6	6
ENSMUSG00000028710	ATPAF1	1	3	1	4	0	1	10	10	10	19	9	8
ENSMUSG00000028717	TAL1	0	0	0	0	0	0	0	1	0	0	0	0
ENSMUSG00000028718	STIL	5	9	12	10	11	5	50	42	25	41	34	16
ENSMUSG00000028719	CMPK1	15	27	30	24	28	31	147	129	112	152	90	132
ENSMUSG00000028729	EBNA1BP2	11	30	18	26	22	16	139	185	118	153	117	119
ENSMUSG00000028737	ALDH4A1	0	1	1	0	0	1	2	0	1	5	1	1
ENSMUSG00000028741	MRTO4	12	4	5	4	17	8	69	42	54	62	41	47
ENSMUSG00000028743	AKR7A5	2	0	0	0	1	4	6	3	6	8	6	9
ENSMUSG00000028744	PQLC2	0	0	0	0	1	0	1	3	1	1	2	1
ENSMUSG00000028745	CAPZB	45	58	52	60	51	46	244	260	200	247	176	203
ENSMUSG00000028756	PINK1	0	0	1	1	0	2	6	12	4	1	8	5
ENSMUSG00000028757	DDOST	43	44	43	44	43	47	238	262	211	246	195	197
ENSMUSG00000028759	HP1BP3	17	27	16	22	25	20	128	97	89	122	87	103
ENSMUSG00000028760	EIF4G3	26	39	38	48	51	44	122	144	109	145	81	117
ENSMUSG00000028766	ALPL	0	0	0	0	0	0	0	0	0	1	0	0
ENSMUSG00000028771	PTPN12	5	3	2	1	1	3	13	8	12	14	20	11
ENSMUSG00000028772	ZCCHC17	14	27	12	20	23	13	95	94	83	105	81	102
ENSMUSG00000028773	FABP3	0	0	0	0	1	0	0	1	0	0	1	0
ENSMUSG00000028779	PEF1	5	2	5	4	4	1	25	38	15	20	15	20
ENSMUSG00000028785	HPCA	0	0	0	0	0	1	0	0	0	0	0	0
ENSMUSG00000028786	TMEM54	0	0	0	0	0	0	0	0	0	0	1	0
ENSMUSG00000028788	PTP4A2	112	121	108	147	111	129	448	445	310	549	329	365
ENSMUSG00000028790	KHDRBS1	10	21	22	20	19	23	115	107	87	149	78	72
ENSMUSG00000028792	AK2	36	23	40	46	44	47	227	220	177	241	171	212
ENSMUSG00000028793	RNF19B	6	2	2	5	1	2	24	16	21	23	11	16
ENSMUSG00000028795	CCDC28B	4	6	3	4	2	0	21	23	17	23	25	21
ENSMUSG00000028796	PHC2	8	13	13	4	10	13	34	38	30	31	24	42
ENSMUSG00000028797	TMEM234	13	23	36	29	24	29	142	154	95	150	106	128
ENSMUSG00000028798	EIF3I	75	94	101	121	114	101	495	547	409	547	489	476
ENSMUSG00000028799	ZFP362	1	0	2	2	1	0	7	13	7	7	6	5
ENSMUSG00000028800	HDAC1	18	30	27	28	23	20	202	177	140	222	171	165
ENSMUSG00000028803	NIPAL3	0	0	0	2	1	3	8	5	2	10	4	12
ENSMUSG00000028804	CSMD2	0	0	0	0	0	0	2	0	0	1	2	2
ENSMUSG00000028809	SRRM1	49	87	75	97	75	82	427	423	290	526	342	351
ENSMUSG00000028811	YARS	12	20	24	15	24	13	120	139	111	124	97	105
ENSMUSG00000028820	SFPQ	40	71	57	102	83	71	379	441	293	446	307	324
ENSMUSG00000028821	SYF2	9	5	8	9	11	6	58	46	35	55	34	37
ENSMUSG00000028822	TMEM50A	6	5	9	13	12	8	45	51	26	66	38	38
ENSMUSG00000028826	TMEM57	5	11	7	10	12	7	35	39	41	64	32	39
ENSMUSG00000028830	AU040320	4	3	5	2	2	3	17	14	9	16	16	14
ENSMUSG00000028832	STMN1	3	1	4	3	1	0	20	12	12	15	9	25
ENSMUSG00000028833	NCDN	1	3	6	4	1	4	25	15	15	21	16	19

ENSMUSG00000028837	PSMB2	48	44	51	70	57	51	332	329	272	312	248	296
ENSMUSG00000028838	EXTL1	0	0	0	0	0	0	1	0	0	0	0	0
ENSMUSG00000028840	ZFP593	4	6	7	8	4	5	42	48	30	41	35	41
ENSMUSG00000028841	CNKSR1	1	0	0	1	0	0	1	0	0	0	0	0
ENSMUSG00000028842	AGO3	4	9	8	9	14	4	36	28	34	41	13	27
ENSMUSG00000028843	SH3BGRL3	0	2	3	3	2	1	12	9	10	12	6	6
ENSMUSG00000028845	TEKT2	0	0	1	1	2	1	2	3	4	5	0	1
ENSMUSG00000028847	TRAPPC3	10	6	5	6	3	2	24	40	21	48	27	33
ENSMUSG00000028848	GPN2	0	2	3	1	3	1	6	13	8	2	4	4
ENSMUSG00000028849	MAP7D1	2	11	12	5	15	12	38	39	51	53	33	39
ENSMUSG00000028850	GPATCH3	1	0	0	1	1	1	4	5	6	7	4	5
ENSMUSG00000028851	NUDC	11	13	14	12	16	12	70	102	42	70	61	53
ENSMUSG00000028854	SLC9A1	2	3	4	1	4	1	9	13	14	17	12	12
ENSMUSG00000028857	TMEM222	4	4	4	4	6	2	29	19	17	23	13	16
ENSMUSG00000028860	SYTL1	1	1	0	3	1	2	10	8	7	11	5	8
ENSMUSG00000028861	MRPS15	12	22	13	33	19	18	117	119	76	110	87	99
ENSMUSG00000028863	MEAF6	3	4	1	1	2	3	12	8	17	21	8	27
ENSMUSG00000028868	WASF2	12	9	7	12	9	10	46	51	38	41	36	37
ENSMUSG00000028869	GNL2	32	61	40	69	60	50	264	267	175	274	225	240
ENSMUSG00000028873	CDCA8	6	12	15	13	17	12	86	78	71	108	69	80
ENSMUSG00000028876	EPHA10	2	0	1	0	0	0	0	1	2	0	1	2
ENSMUSG00000028878	FAM76A	4	1	1	2	3	2	16	8	4	9	9	14
ENSMUSG00000028879	STX12	3	3	1	4	5	1	11	24	11	28	23	12
ENSMUSG00000028882	PPP1R8	4	15	7	7	5	9	55	48	35	65	38	38
ENSMUSG00000028884	RPA2	7	9	7	9	13	11	60	59	47	55	56	59
ENSMUSG00000028886	EYA3	3	6	5	5	3	2	23	25	8	25	16	11
ENSMUSG00000028889	YRDC	2	19	9	8	10	6	72	84	54	69	68	55
ENSMUSG00000028890	MTF1	5	4	5	5	13	6	32	40	17	41	28	28
ENSMUSG00000028893	SESN2	2	3	5	3	11	6	28	29	15	22	20	26
ENSMUSG00000028894	INPP5B	14	17	20	17	12	13	77	86	63	70	46	75
ENSMUSG00000028896	RCC1	13	23	17	20	22	31	80	89	76	76	56	82
ENSMUSG00000028898	TRNAU1AP	2	6	5	6	5	9	28	26	16	15	8	14
ENSMUSG00000028899	TAF12	2	3	3	4	3	0	18	27	19	17	15	19
ENSMUSG00000028901	GMEB1	4	2	1	5	2	3	21	14	9	13	8	9
ENSMUSG00000028902	SF3A3	19	26	18	28	31	24	163	158	146	173	130	133
ENSMUSG00000028906	EPB41	6	4	11	2	8	9	49	63	42	43	34	50
ENSMUSG00000028907	UTP11	18	32	18	37	42	33	199	202	147	237	165	172
ENSMUSG00000028909	PTPRU	0	0	1	0	0	0	0	0	0	0	0	0
ENSMUSG00000028910	MECR	2	3	4	3	1	4	21	23	11	20	22	18
ENSMUSG00000028911	SRSF4	7	14	9	12	18	18	73	71	54	95	65	84
ENSMUSG00000028914	CASP9	2	1	2	1	1	1	9	14	6	7	6	3
ENSMUSG00000028917	PLEKHM2	4	1	2	4	2	5	16	17	9	12	10	5
ENSMUSG00000028919	ARHGEF19	29	25	30	38	35	36	99	158	94	128	74	122
ENSMUSG00000028920	FBXO42	4	3	3	3	4	1	15	14	13	15	9	16
ENSMUSG00000028923	NECAP2	2	2	4	5	1	5	21	23	14	17	14	17
ENSMUSG00000028926	CDK14	0	0	0	0	1	0	0	0	0	0	0	0
ENSMUSG00000028927	PADI2	0	0	0	0	0	0	0	0	0	0	1	0
ENSMUSG00000028931	KCNAB2	4	7	7	5	13	12	28	45	31	40	28	31
ENSMUSG00000028932	PSMC2	15	12	12	21	14	26	94	113	84	99	107	103
ENSMUSG00000028933	XRCC2	0	2	2	4	3	6	4	5	3	5	5	5
ENSMUSG00000028936	RPL22	150	163	163	184	201	161	1164	1201	903	998	972	980
ENSMUSG00000028937	ACOT7	4	2	5	4	4	5	35	42	34	40	22	35
ENSMUSG00000028943	ESPN	0	0	0	0	0	1	0	1	0	0	0	0
ENSMUSG00000028944	PRKAG2	1	3	4	5	8	7	30	32	23	34	32	19
ENSMUSG00000028945	RHEB	13	17	14	19	25	14	92	96	71	112	68	97
ENSMUSG00000028948	NOL9	7	5	7	16	9	3	43	41	33	44	32	50
ENSMUSG00000028949	SMARCD3	0	0	0	0	1	0	1	4	2	2	5	8
ENSMUSG00000028952	ZBTB48	1	1	4	4	1	1	13	13	15	7	11	11

ENSMUSG00000028953	ABCF2	1	3	1	2	1	3	13	13	14	9	11	13
ENSMUSG00000028954	NUB1	9	9	7	7	10	10	46	39	31	43	35	32
ENSMUSG00000028955	VAMP3	4	1	1	8	1	5	22	21	32	22	20	17
ENSMUSG00000028957	PER3	0	2	3	2	0	3	4	10	3	7	8	3
ENSMUSG00000028958	TMUB1	1	1	2	2	2	2	4	5	3	6	4	5
ENSMUSG00000028959	FASTK	3	3	7	13	10	5	30	31	27	21	22	19
ENSMUSG00000028960	UBE4B	5	1	6	10	3	4	35	17	19	39	19	31
ENSMUSG00000028961	PGD	11	21	15	19	21	19	103	107	82	94	62	80
ENSMUSG00000028962	SLC4A2	2	3	2	8	9	4	22	20	17	21	22	28
ENSMUSG00000028964	PARK7	25	19	21	13	25	15	130	128	106	152	118	128
ENSMUSG00000028965	TNFRSF9	1	0	1	0	1	0	6	8	4	3	4	1
ENSMUSG00000028969	CDK5	11	7	9	4	5	11	26	35	29	20	25	24
ENSMUSG00000028970	ABCB1B	12	23	16	22	27	20	110	104	66	147	104	93
ENSMUSG00000028971	CORT	0	0	0	0	0	0	0	0	1	0	2	0
ENSMUSG00000028973	ABCB8	3	1	3	3	4	7	15	26	12	23	16	13
ENSMUSG00000028974	DFFA	1	2	3	4	3	0	8	11	8	25	10	13
ENSMUSG00000028975	PEX14	5	3	2	6	4	0	12	13	11	15	12	12
ENSMUSG00000028977	CASZ1	2	2	3	2	0	4	13	14	12	13	23	11
ENSMUSG00000028979	MASP2	0	0	0	0	0	0	1	1	0	1	1	0
ENSMUSG00000028980	H6PD	0	0	0	0	1	0	0	0	0	0	0	0
ENSMUSG00000028986	KLHL7	2	7	3	4	3	2	27	21	25	23	18	19
ENSMUSG00000028990	LZIC	1	3	1	0	0	0	2	1	2	5	3	3
ENSMUSG00000028991	MTOR	4	4	7	3	6	4	26	21	21	23	27	24
ENSMUSG00000028992	NMNAT1	0	0	0	1	0	0	2	0	2	1	2	2
ENSMUSG00000028995	FAM126A	18	22	9	17	18	16	44	39	29	52	38	38
ENSMUSG00000028996	RBP7	0	0	0	0	0	0	0	0	0	0	0	1
ENSMUSG00000028998	TOMM7	25	37	33	46	23	27	171	181	163	176	134	169
ENSMUSG00000028999	RINT1	4	7	8	9	7	5	26	27	22	30	23	31
ENSMUSG00000029001	FBXO44	0	0	1	0	0	0	0	0	0	0	0	0
ENSMUSG00000029003	MAD2L2	2	1	3	2	3	2	15	23	12	19	15	12
ENSMUSG00000029004	KMT2E	46	57	49	67	56	65	233	255	174	284	196	210
ENSMUSG00000029007	AGTRAP	4	12	5	9	12	12	52	49	36	62	41	48
ENSMUSG00000029009	MTHFR	1	1	1	1	0	2	3	5	4	10	3	3
ENSMUSG00000029012	ORC5	2	2	2	0	1	3	7	10	3	10	3	3
ENSMUSG00000029014	DNAJC2	16	28	18	35	26	18	146	119	108	128	81	102
ENSMUSG00000029016	CLCN6	2	1	4	4	3	1	3	12	4	11	5	10
ENSMUSG00000029017	PMPCB	10	12	11	9	24	9	71	80	54	54	62	55
ENSMUSG00000029020	MFN2	2	4	2	6	1	4	14	12	10	14	13	18
ENSMUSG00000029022	MIIP	1	3	1	4	5	1	15	20	9	14	10	14
ENSMUSG00000029027	DFFB	1	5	3	8	5	4	17	22	14	20	13	11
ENSMUSG00000029028	LRRC47	2	2	4	5	5	5	21	16	20	24	19	28
ENSMUSG00000029029	WRAP73	1	0	1	2	0	2	9	17	7	18	10	15
ENSMUSG00000029030	TPRGL	2	5	7	8	3	3	31	46	23	39	21	22
ENSMUSG00000029033	ACAP3	2	1	3	1	6	2	9	11	2	10	11	8
ENSMUSG00000029034	INTS11	2	6	2	3	3	3	27	30	31	29	14	25
ENSMUSG00000029036	ATAD3A	9	13	11	14	13	9	59	55	40	52	35	41
ENSMUSG00000029038	SSU72	9	10	16	19	7	17	88	76	65	78	47	78
ENSMUSG00000029047	PEX10	0	3	1	4	2	1	8	14	4	9	7	9
ENSMUSG00000029048	RER1	4	8	7	8	11	9	37	36	31	23	39	43
ENSMUSG00000029049	MORN1	0	0	0	0	0	0	0	0	2	0	0	0
ENSMUSG00000029050	SKI	6	4	5	8	7	4	29	40	20	37	28	27
ENSMUSG00000029054	GABRD	0	0	0	2	3	0	0	2	1	3	1	2
ENSMUSG00000029055	PLCH2	0	0	0	0	0	0	0	0	0	0	1	0
ENSMUSG00000029056	PANK4	9	8	12	11	11	14	37	45	42	40	37	29
ENSMUSG00000029060	MIB2	0	0	0	5	1	1	9	12	7	11	10	10
ENSMUSG00000029061	MMP23	0	0	0	0	0	0	0	0	0	2	0	0
ENSMUSG00000029062	CDK11B	81	92	86	102	97	93	434	537	351	505	440	451
ENSMUSG00000029063	NADK	6	9	11	14	4	10	57	48	38	39	41	33

ENSMUSG00000029064	GNB1	67	86	93	101	90	73	207	206	197	253	160	219
ENSMUSG00000029066	MRPL20	17	19	17	21	16	18	81	92	71	70	76	95
ENSMUSG00000029068	CCNL2	16	19	26	19	10	19	106	100	82	113	79	92
ENSMUSG00000029071	DVL1	5	14	5	14	10	3	37	25	29	27	25	38
ENSMUSG00000029072	TAS1R3	0	9	1	0	0	1	1	3	2	0	0	2
ENSMUSG00000029073	CPTP	0	0	2	0	0	3	9	8	4	7	10	6
ENSMUSG00000029075	TNFRSF4	0	0	0	1	0	0	0	1	1	1	2	1
ENSMUSG00000029076	SDF4	13	6	4	13	14	13	52	48	51	60	36	49
ENSMUSG00000029088	KCNIP4	0	0	0	0	0	0	0	0	0	1	0	1
ENSMUSG00000029089	5730480H06I	0	3	1	2	0	2	11	10	11	16	7	9
ENSMUSG00000029090	ADGRA3	0	0	1	1	0	0	1	3	1	0	0	0
ENSMUSG00000029093	SORCS2	0	0	0	0	0	0	0	0	0	0	0	1
ENSMUSG00000029096	HTRA3	0	0	0	0	0	0	0	2	2	0	0	0
ENSMUSG00000029097	TRMT44	1	0	1	0	3	2	1	0	4	5	2	5
ENSMUSG00000029098	ACOX3	4	3	1	4	0	2	3	15	6	9	7	13
ENSMUSG00000029101	RGS12	0	1	0	2	1	3	1	2	3	1	7	2
ENSMUSG00000029104	HTT	8	5	6	17	12	11	18	62	28	43	21	44
ENSMUSG00000029106	ADD1	9	9	6	15	14	9	53	47	40	56	43	54
ENSMUSG00000029110	RNF4	10	24	21	27	15	18	117	119	98	153	102	158
ENSMUSG00000029111	NELFA	5	8	11	18	12	14	75	87	66	91	52	97
ENSMUSG00000029119	MAN2B2	1	0	3	2	2	3	11	9	5	10	10	12
ENSMUSG00000029121	CRMP1	5	11	8	7	6	10	46	66	42	42	38	33
ENSMUSG00000029122	EVC	0	0	0	0	0	0	0	0	1	0	0	0
ENSMUSG00000029125	STX18	1	1	0	3	1	1	12	9	8	7	7	6
ENSMUSG00000029126	NSG1	0	0	1	0	1	0	0	0	1	0	1	1
ENSMUSG00000029127	ZBTB49	2	0	3	0	0	1	3	2	4	2	4	4
ENSMUSG00000029128	RAB28	6	7	6	8	9	7	21	20	17	24	9	15
ENSMUSG00000029131	DNAJB6	1	8	4	4	1	5	17	13	16	30	14	15
ENSMUSG00000029134	PLB1	0	0	0	0	0	0	0	0	1	0	0	0
ENSMUSG00000029135	FOSL2	2	2	1	0	2	1	2	5	8	7	5	4
ENSMUSG00000029136	RBKS	0	0	0	1	0	0	4	8	3	7	2	3
ENSMUSG00000029138	4930548H24I	0	0	0	0	0	0	0	0	0	1	0	0
ENSMUSG00000029141	SLC4A1AP	1	7	8	6	5	10	34	35	23	32	23	32
ENSMUSG00000029145	EIF2B4	6	6	2	7	5	4	29	28	27	34	32	25
ENSMUSG00000029146	SNX17	5	8	9	14	9	6	38	44	35	37	39	47
ENSMUSG00000029147	PPM1G	34	54	45	42	47	32	188	217	146	198	135	153
ENSMUSG00000029148	NRBP1	10	3	10	9	5	5	27	25	18	31	26	27
ENSMUSG00000029149	KRTCAP3	1	1	0	1	0	0	1	1	1	1	2	2
ENSMUSG00000029152	OCIAD1	12	8	16	11	9	15	76	84	59	72	68	79
ENSMUSG00000029154	CWH43	0	0	0	0	0	0	1	0	0	0	0	0
ENSMUSG00000029156	SGCB	2	2	3	5	2	1	15	19	8	9	8	10
ENSMUSG00000029162	KHK	0	0	0	0	0	0	0	0	2	3	0	0
ENSMUSG00000029163	EMILIN1	0	0	1	0	0	1	0	0	0	0	0	0
ENSMUSG00000029165	AGBL5	1	1	1	1	1	0	12	12	7	19	6	10
ENSMUSG00000029166	MAPRE3	2	1	1	0	3	0	13	7	2	7	3	5
ENSMUSG00000029167	PPARGC1A	3	1	4	1	4	1	17	17	12	27	14	11
ENSMUSG00000029169	DHX15	22	24	29	35	31	26	244	275	229	293	167	209
ENSMUSG00000029171	PGM1	8	8	5	12	9	12	57	80	49	62	47	57
ENSMUSG00000029173	SEPSECS	2	3	1	3	4	7	14	19	18	17	15	19
ENSMUSG00000029174	TBC1D1	4	5	6	14	7	16	49	45	32	36	31	25
ENSMUSG00000029175	SLC35F6	0	1	1	1	0	0	6	5	5	2	4	3
ENSMUSG00000029176	ANAPC4	7	7	14	11	9	19	50	48	43	55	46	50
ENSMUSG00000029177	CENPA	14	18	21	17	19	17	107	143	83	119	89	97
ENSMUSG00000029178	KLF3	1	0	0	0	0	0	0	0	0	1	0	0
ENSMUSG00000029179	ZCCHC4	1	1	5	0	3	3	11	7	5	10	13	5
ENSMUSG00000029185	FAM114A1	0	1	0	0	0	0	3	5	2	2	3	4
ENSMUSG00000029186	PI4K2B	13	7	3	6	6	7	52	42	28	33	21	25
ENSMUSG00000029188	SLC34A2	0	0	0	0	1	0	0	0	0	0	0	1

ENSMUSG00000029190	D5ERTD579E	3	8	1	3	5	3	11	12	7	14	11	8
ENSMUSG00000029191	RFC1	51	58	31	33	60	45	259	230	139	265	177	215
ENSMUSG00000029192	TBC1D14	1	0	0	1	0	0	5	2	0	0	3	2
ENSMUSG00000029195	KLB	0	0	0	0	0	0	0	0	0	0	1	0
ENSMUSG00000029196	TADA2B	5	2	3	5	3	4	18	17	7	13	6	17
ENSMUSG00000029198	GRPEL1	7	10	5	2	5	7	34	44	40	57	39	45
ENSMUSG00000029199	LIAS	9	14	10	12	13	12	45	53	52	61	46	42
ENSMUSG00000029201	UGDH	3	8	3	6	8	7	61	46	39	61	43	39
ENSMUSG00000029202	PDS5A	11	39	21	28	31	29	132	131	94	153	90	108
ENSMUSG00000029203	UBE2K	13	24	23	29	42	23	138	143	92	133	95	105
ENSMUSG00000029204	RHOH	3	3	4	8	6	4	26	22	16	13	20	23
ENSMUSG00000029205	CHRNA9	0	0	0	0	1	0	0	0	0	0	0	0
ENSMUSG00000029207	APBB2	4	6	8	7	9	8	20	34	9	14	14	17
ENSMUSG00000029208	GUF1	4	8	11	7	11	7	60	60	45	73	51	46
ENSMUSG00000029209	GNPDA2	4	4	5	5	2	3	35	42	16	30	17	27
ENSMUSG00000029212	GABRB1	0	1	0	0	1	0	0	2	1	2	0	1
ENSMUSG00000029213	COMMD8	14	12	11	19	9	8	91	89	47	97	56	81
ENSMUSG00000029217	TEC	0	1	3	1	1	3	4	6	1	3	4	7
ENSMUSG00000029221	SLC30A9	4	10	5	9	4	7	36	31	21	40	28	25
ENSMUSG00000029227	FIP1L1	21	33	22	33	26	36	158	145	108	187	123	133
ENSMUSG00000029228	LNX1	0	0	0	0	0	0	1	1	1	2	0	2
ENSMUSG00000029229	CHIC2	6	5	4	7	11	9	43	41	31	53	40	31
ENSMUSG00000029233	SRD5A3	2	1	1	0	1	1	2	4	2	2	3	2
ENSMUSG00000029234	TMEM165	8	7	12	12	11	8	70	71	54	71	57	47
ENSMUSG00000029236	NMU	0	0	0	0	0	0	0	0	0	0	0	1
ENSMUSG00000029238	CLOCK	5	6	10	4	7	7	23	17	19	29	17	19
ENSMUSG00000029246	PPAT	12	25	20	26	19	20	145	132	101	147	78	92
ENSMUSG00000029247	PAICS	31	32	22	38	30	27	249	262	229	283	194	237
ENSMUSG00000029249	REST	19	15	17	22	22	9	59	61	54	80	43	53
ENSMUSG00000029250	POLR2B	14	15	10	21	19	15	86	82	62	92	58	67
ENSMUSG00000029253	CENPC1	9	17	14	15	12	18	80	70	62	112	75	63
ENSMUSG00000029254	STAP1	5	8	9	11	12	7	63	54	41	62	33	45
ENSMUSG00000029255	GNRHR	0	0	0	0	0	0	1	0	0	0	0	0
ENSMUSG00000029263	PIGG	0	0	1	2	2	2	5	5	7	6	1	2
ENSMUSG00000029265	DR1	16	17	30	27	17	20	117	126	70	108	87	96
ENSMUSG00000029267	MTF2	41	43	60	65	43	66	254	273	166	300	160	184
ENSMUSG00000029270	FAM69A	3	6	5	5	3	4	21	28	19	19	20	33
ENSMUSG00000029275	GFI1	1	4	2	4	3	4	12	18	13	10	15	13
ENSMUSG00000029276	GLMN	1	5	4	10	6	6	43	32	23	44	23	26
ENSMUSG00000029279	BRDT	0	1	0	4	1	1	14	8	7	6	4	8
ENSMUSG00000029283	CDC7	4	7	10	6	8	9	44	50	37	50	41	30
ENSMUSG00000029287	TGFBR3	0	0	0	1	4	0	3	14	4	4	7	4
ENSMUSG00000029290	ZFP326	17	23	28	30	30	31	104	119	88	117	125	104
ENSMUSG00000029291	RUFY3	2	1	2	5	3	6	17	10	9	16	15	11
ENSMUSG00000029298	GBP9	3	1	2	1	3	4	6	8	14	16	6	6
ENSMUSG00000029299	ABCG3	6	7	7	2	6	8	39	35	25	53	26	27
ENSMUSG00000029304	SPP1	17	13	11	11	16	12	95	101	68	113	59	75
ENSMUSG00000029310	NUDT9	3	4	1	3	1	3	12	24	10	21	18	20
ENSMUSG00000029311	HSD17B11	0	0	0	0	0	0	0	1	0	0	0	1
ENSMUSG00000029312	KLHL8	0	0	0	2	0	1	1	5	1	1	1	0
ENSMUSG00000029313	AFF1	12	9	10	12	19	13	63	57	35	67	44	48
ENSMUSG00000029319	COQ2	3	6	7	4	1	2	20	22	11	29	23	25
ENSMUSG00000029322	PLAC8	0	0	1	0	0	0	2	0	1	0	1	0
ENSMUSG00000029326	ENOPH1	0	3	3	3	5	3	15	23	16	15	10	21
ENSMUSG00000029328	HNRNPDL	30	46	38	52	54	42	292	256	203	336	187	222
ENSMUSG00000029338	ANTXR2	2	4	3	4	4	2	24	22	12	19	16	18
ENSMUSG00000029344	TPST2	6	2	3	7	6	4	22	24	13	26	13	23
ENSMUSG00000029345	TFIP11	8	3	3	8	5	0	24	36	23	26	38	28

ENSMUSG00000029346	SRRD	4	3	2	7	4	6	17	17	12	9	22	15
ENSMUSG00000029359	TESC	0	0	0	0	1	1	0	1	2	1	1	2
ENSMUSG00000029360	GM9754	0	1	0	1	2	2	2	8	5	7	7	4
ENSMUSG00000029361	NOS1	0	0	0	0	0	0	1	0	0	0	0	0
ENSMUSG00000029363	RFC5	4	9	7	3	6	7	40	42	27	39	33	32
ENSMUSG00000029364	WSB2	6	2	1	1	2	2	13	6	6	11	1	9
ENSMUSG00000029366	DCK	10	19	15	24	6	10	99	95	82	117	86	88
ENSMUSG00000029370	RASSF6	0	0	0	2	0	0	0	1	1	0	0	1
ENSMUSG00000029376	MTHFD2L	3	4	5	7	4	3	20	16	19	24	16	13
ENSMUSG00000029379	CXCL3	0	0	0	0	0	0	0	0	0	1	0	0
ENSMUSG00000029384	2010109A12F	1	0	0	0	0	0	0	0	0	1	0	0
ENSMUSG00000029385	CCNG2	0	5	1	2	3	2	29	20	18	22	14	9
ENSMUSG00000029386	TCTN2	0	0	0	0	0	0	0	0	0	1	0	0
ENSMUSG00000029387	GTF2H3	32	36	39	48	40	35	278	260	246	262	199	251
ENSMUSG00000029388	EIF2B1	11	17	11	17	18	11	114	100	70	91	86	99
ENSMUSG00000029389	DDX55	28	31	22	49	47	30	141	163	146	169	119	156
ENSMUSG00000029390	TMED2	86	113	107	118	159	102	411	472	417	512	426	410
ENSMUSG00000029392	RILPL1	2	1	0	1	0	0	4	2	2	4	4	2
ENSMUSG00000029394	CDK2AP1	4	4	3	3	9	4	24	25	16	27	24	19
ENSMUSG00000029397	RCHY1	5	6	5	2	2	2	19	17	13	26	13	13
ENSMUSG00000029401	RILPL2	4	13	8	9	10	12	64	62	43	55	47	48
ENSMUSG00000029402	SNRNP35	5	3	1	3	4	5	20	29	13	18	18	21
ENSMUSG00000029403	CDKL2	1	0	0	0	0	1	0	0	0	0	0	0
ENSMUSG00000029404	ARL6IP4	20	25	29	25	16	13	127	113	102	107	81	91
ENSMUSG00000029405	G3BP2	29	32	25	37	33	40	175	178	145	193	131	127
ENSMUSG00000029406	PITPNM2	16	14	12	16	21	11	67	78	53	58	43	62
ENSMUSG00000029407	USO1	19	31	27	27	30	32	123	145	94	150	111	133
ENSMUSG00000029408	ABCB9	68	81	96	83	96	76	346	398	297	361	262	353
ENSMUSG00000029410	PPEF2	1	0	0	0	0	0	0	0	0	0	2	0
ENSMUSG00000029413	NAAA	5	3	1	5	4	5	19	24	17	25	19	21
ENSMUSG00000029414	KNTC1	4	7	6	2	9	7	49	42	39	45	32	31
ENSMUSG00000029415	SDAD1	6	9	9	17	19	16	64	67	62	97	66	64
ENSMUSG00000029416	SLC15A4	2	1	0	4	2	0	6	7	8	2	9	10
ENSMUSG00000029419	GM996	2	0	1	0	1	3	2	3	4	5	4	4
ENSMUSG00000029422	RSRC2	69	99	73	93	103	76	456	443	284	514	384	375
ENSMUSG00000029426	SCARB2	3	6	4	4	7	5	29	26	19	39	26	27
ENSMUSG00000029427	ZCCHC8	27	29	27	37	39	36	117	151	123	135	118	129
ENSMUSG00000029428	STX2	1	1	0	0	0	1	3	0	1	0	1	0
ENSMUSG00000029430	RAN	32	35	43	42	45	26	364	313	304	360	266	310
ENSMUSG00000029431	B3GNT4	2	0	1	0	0	0	5	4	2	5	3	3
ENSMUSG00000029432	NIPSNAP2	6	5	6	3	4	4	46	44	23	31	31	26
ENSMUSG00000029433	DIABLO	5	9	10	7	7	2	21	34	25	29	23	21
ENSMUSG00000029434	VPS33A	2	3	9	4	6	6	17	19	22	19	23	15
ENSMUSG00000029437	IL31	0	0	0	0	0	0	0	1	0	0	0	0
ENSMUSG00000029438	BCL7A	7	12	5	9	24	18	60	67	37	58	34	49
ENSMUSG00000029439	SFSWAP	30	18	20	14	29	28	142	136	97	130	95	103
ENSMUSG00000029440	PSMD9	5	2	10	9	4	1	30	27	24	36	17	24
ENSMUSG00000029442	WDR66	1	0	1	0	0	0	2	4	2	2	3	0
ENSMUSG00000029446	PSPH	14	16	12	18	22	23	147	150	109	137	113	112
ENSMUSG00000029447	CCT6A	55	86	87	75	79	71	552	634	472	605	447	460
ENSMUSG00000029449	RHOF	1	0	4	1	2	3	11	13	6	11	6	14
ENSMUSG00000029452	TMEM116	1	0	0	1	1	0	2	2	0	2	1	0
ENSMUSG00000029454	MAPKAPK5	0	0	0	0	0	0	0	0	0	0	1	0
ENSMUSG00000029455	ALDH2	5	5	6	8	8	9	44	40	31	27	29	32
ENSMUSG00000029456	ACAD10	0	0	2	1	1	0	2	2	4	3	2	2
ENSMUSG00000029458	BRAP	9	9	12	15	20	13	69	68	40	78	71	68
ENSMUSG00000029461	FAM168A	4	10	5	7	8	6	26	31	25	46	39	34
ENSMUSG00000029462	VPS29	11	15	5	9	9	14	88	71	44	86	49	75

ENSMUSG00000029463	FAM216A	1	1	2	0	3	3	19	19	15	17	8	17
ENSMUSG00000029464	GPN3	10	6	12	8	8	12	74	51	42	76	45	39
ENSMUSG00000029465	ARPC3	15	16	24	26	22	27	150	181	141	150	111	116
ENSMUSG00000029466	ANAPC7	4	8	5	5	8	4	36	56	19	25	24	23
ENSMUSG00000029467	ATP2A2	28	33	29	39	46	32	194	220	146	217	178	193
ENSMUSG00000029468	P2RX7	4	1	1	6	1	2	13	9	11	11	6	9
ENSMUSG00000029469	IFT81	0	1	1	1	1	1	3	3	4	7	2	0
ENSMUSG00000029470	P2RX4	5	2	3	4	5	3	22	7	9	11	11	15
ENSMUSG00000029471	CAMKK2	5	7	7	1	5	10	25	21	10	17	13	18
ENSMUSG00000029472	ANAPC5	11	15	18	17	19	15	83	113	94	83	79	81
ENSMUSG00000029474	RNF34	4	2	0	2	7	4	24	31	19	40	16	18
ENSMUSG00000029475	KDM2B	4	4	0	4	1	0	4	10	9	10	6	9
ENSMUSG00000029478	NCOR2	10	15	14	19	11	12	55	46	27	66	35	59
ENSMUSG00000029480	DHX37	1	1	4	4	4	5	12	18	14	12	13	11
ENSMUSG00000029482	AACS	12	12	10	15	11	9	81	106	71	103	67	72
ENSMUSG00000029486	MRPL1	6	6	4	11	6	9	56	52	31	60	37	58
ENSMUSG00000029499	PXMP2	0	0	1	0	0	1	1	0	1	1	0	3
ENSMUSG00000029500	PGAM5	1	8	1	6	8	5	20	23	21	17	18	19
ENSMUSG00000029501	ANKLE2	6	7	4	14	7	6	44	39	29	48	19	29
ENSMUSG00000029502	GOLGA3	4	6	3	5	6	6	26	27	20	32	20	30
ENSMUSG00000029504	DDX51	2	3	4	3	8	2	25	32	26	27	18	21
ENSMUSG00000029505	EP400	13	30	19	22	19	22	81	95	61	117	74	78
ENSMUSG00000029507	PUS1	6	7	4	8	4	7	45	38	25	36	31	39
ENSMUSG00000029512	ULK1	4	1	1	5	4	0	13	11	8	12	4	10
ENSMUSG00000029513	PRKAB1	3	2	3	8	3	4	17	11	13	17	12	17
ENSMUSG00000029516	CIT	24	39	32	33	32	41	125	112	100	179	96	109
ENSMUSG00000029517	ANKRD7	0	0	0	0	0	0	1	1	1	1	1	0
ENSMUSG00000029518	RAB35	2	6	5	3	3	4	30	26	22	34	13	19
ENSMUSG00000029521	CHEK2	13	9	11	13	14	7	58	56	46	73	47	56
ENSMUSG00000029524	SIRT4	0	1	1	2	0	0	5	4	1	3	2	3
ENSMUSG00000029528	PXN	0	2	3	1	1	2	9	7	7	8	6	5
ENSMUSG00000029530	CCR9	0	0	1	0	0	0	1	1	0	4	1	1
ENSMUSG00000029534	ST7	1	1	0	1	2	0	6	8	6	8	4	5
ENSMUSG00000029535	TRIAP1	2	2	4	6	1	4	17	22	15	14	15	10
ENSMUSG00000029536	GATC	1	2	0	3	6	2	20	10	11	14	12	14
ENSMUSG00000029538	SRSF9	30	44	51	64	50	33	398	455	377	411	370	378
ENSMUSG00000029544	CABP1	0	0	1	0	0	0	0	1	0	2	1	0
ENSMUSG00000029545	ACADS	6	17	8	14	8	8	70	81	48	68	47	68
ENSMUSG00000029547	INTS1	7	17	14	17	19	15	59	69	57	69	55	67
ENSMUSG00000029550	SPPL3	7	12	12	7	8	11	42	47	38	54	60	39
ENSMUSG00000029551	PSMG3	2	5	3	4	5	5	31	32	27	24	19	21
ENSMUSG00000029552	TES	4	5	2	5	1	6	18	20	15	20	7	17
ENSMUSG00000029554	MAD1L1	4	3	1	4	3	1	6	13	7	9	7	8
ENSMUSG00000029557	MRM2	0	1	0	0	0	2	9	6	4	8	5	7
ENSMUSG00000029559	2210016L21R	12	8	13	12	13	15	106	91	65	80	63	89
ENSMUSG00000029560	SNX8	2	1	1	2	2	1	8	7	7	7	7	9
ENSMUSG00000029569	TMEM168	1	2	0	2	2	0	7	4	5	6	6	7
ENSMUSG00000029570	LFNG	2	4	5	5	6	3	13	16	14	13	13	8
ENSMUSG00000029571	TMEM106B	2	5	4	5	4	5	23	24	11	27	5	15
ENSMUSG00000029575	MMAB	1	0	0	1	0	0	2	7	4	5	1	4
ENSMUSG00000029577	UBE3B	0	4	3	4	2	7	11	18	15	9	18	9
ENSMUSG00000029578	WIPI2	9	8	11	16	18	9	43	48	45	51	40	48
ENSMUSG00000029580	ACTB	5047	6325	5552	6563	6002	6187	23890	23603	17739	26221	16169	20889
ENSMUSG00000029587	ZFP12	4	0	6	4	2	1	9	10	3	8	9	11
ENSMUSG00000029591	UNG	6	12	15	12	11	9	77	76	71	78	55	73
ENSMUSG00000029592	USP30	2	1	3	4	1	1	13	16	14	13	14	10
ENSMUSG00000029594	RBM19	3	5	4	2	4	4	20	18	22	22	22	20
ENSMUSG00000029597	SDS	0	0	0	0	0	0	0	0	0	1	1	0

ENSMUSG00000029598	PLBD2	3	3	0	3	2	7	12	23	13	20	9	13
ENSMUSG00000029599	DDX54	14	19	16	9	19	17	106	88	66	85	63	80
ENSMUSG00000029600	RITA1	0	0	0	0	0	2	13	11	5	10	1	4
ENSMUSG00000029601	IQCD	1	0	0	0	0	0	1	1	2	0	0	5
ENSMUSG00000029605	OAS1B	0	1	1	0	2	0	2	4	2	3	0	5
ENSMUSG00000029607	ANKRD61	0	1	0	0	0	0	0	0	1	1	0	0
ENSMUSG00000029608	RPH3A	0	0	0	0	0	0	0	0	0	0	0	1
ENSMUSG00000029610	AIMP2	1	9	6	6	6	2	29	31	25	27	29	32
ENSMUSG00000029613	EIF2AK1	1	4	3	4	3	4	18	21	13	20	18	19
ENSMUSG00000029614	RPL6	147	175	155	189	203	184	1176	1272	935	1209	955	1120
ENSMUSG00000029616	ERP29	32	36	24	34	41	30	182	202	154	176	147	137
ENSMUSG00000029617	CCZ1	6	7	5	8	12	7	39	38	37	34	24	40
ENSMUSG00000029621	ARPC1A	30	15	27	38	41	21	138	154	106	162	131	111
ENSMUSG00000029622	ARPC1B	66	74	72	91	76	83	422	431	274	458	267	332
ENSMUSG00000029623	PDAP1	39	52	46	50	56	46	286	316	241	338	256	286
ENSMUSG00000029624	PTCD1	4	0	3	3	3	2	9	13	8	20	8	13
ENSMUSG00000029625	CPSF4	8	5	3	14	10	9	53	59	36	58	40	55
ENSMUSG00000029627	ZKSCAN14	0	3	0	1	2	1	4	5	6	2	6	2
ENSMUSG00000029629	PHF14	11	36	25	28	15	17	74	79	53	81	54	54
ENSMUSG00000029632	NDUFA4	61	58	63	76	44	64	580	584	418	554	463	550
ENSMUSG00000029634	RNF6	9	12	9	7	8	7	40	64	47	65	46	41
ENSMUSG00000029635	CDK8	1	7	5	6	4	8	27	31	16	30	31	25
ENSMUSG00000029638	GLCCI1	0	0	0	1	0	0	1	2	0	3	1	0
ENSMUSG00000029640	USP12	1	2	1	6	2	2	9	6	4	7	2	3
ENSMUSG00000029641	RASL11A	0	0	0	0	0	2	0	0	2	3	2	1
ENSMUSG00000029642	POLR1D	16	16	20	12	20	23	92	96	57	96	84	85
ENSMUSG00000029647	PAN3	70	70	70	80	79	78	294	350	224	288	211	294
ENSMUSG00000029649	POMP	11	11	16	11	7	13	85	84	64	91	71	79
ENSMUSG00000029650	SLC46A3	0	1	0	1	0	0	0	1	0	0	1	1
ENSMUSG00000029655	N4BP2L2	31	53	36	57	51	50	228	234	167	303	160	206
ENSMUSG00000029657	HSPH1	16	19	22	40	26	26	148	147	117	173	101	145
ENSMUSG00000029659	MEDAG	0	0	0	0	0	0	0	0	0	1	0	0
ENSMUSG00000029663	GNGT1	0	0	0	0	0	0	1	0	0	0	0	0
ENSMUSG00000029664	TFPI2	0	0	0	0	0	1	0	0	0	0	0	0
ENSMUSG00000029669	TSPAN12	0	0	0	0	0	0	0	0	0	2	0	0
ENSMUSG00000029670	ING3	10	7	8	9	12	18	50	72	49	70	43	49
ENSMUSG00000029671	WNT16	0	0	0	0	0	0	1	0	0	0	0	0
ENSMUSG00000029672	FAM3C	24	32	26	27	30	25	97	110	69	102	72	92
ENSMUSG00000029673	AUTS2	1	0	0	0	0	0	0	0	0	0	0	0
ENSMUSG00000029674	LIMK1	6	2	6	2	2	4	22	19	14	21	16	17
ENSMUSG00000029676	POT1A	0	2	0	0	2	1	10	12	9	10	9	4
ENSMUSG00000029681	BCL7B	0	6	5	2	7	7	23	30	19	33	18	21
ENSMUSG00000029684	WASL	4	1	0	0	2	3	11	16	6	17	11	8
ENSMUSG00000029686	CUL1	25	34	30	30	42	27	158	167	142	225	136	123
ENSMUSG00000029687	EZH2	51	46	44	40	65	42	272	298	225	279	229	230
ENSMUSG00000029695	AASS	33	58	45	58	62	52	333	402	301	425	235	304
ENSMUSG00000029699	SSC4D	0	0	0	0	0	0	0	0	1	1	0	0
ENSMUSG00000029701	RBM28	30	37	31	35	38	30	171	172	133	211	132	152
ENSMUSG00000029703	LRWD1	5	16	12	13	9	12	61	65	49	65	56	70
ENSMUSG00000029705	CUX1	33	39	47	39	43	44	157	149	120	161	121	145
ENSMUSG00000029708	GCC1	0	0	0	0	0	1	1	1	2	2	0	0
ENSMUSG00000029710	EPHB4	0	0	0	1	0	0	0	0	0	0	0	0
ENSMUSG00000029713	GNB2	35	42	39	42	53	34	237	220	166	215	153	198
ENSMUSG00000029714	GIGYF1	40	43	55	52	59	61	205	222	159	209	129	205
ENSMUSG00000029715	POP7	3	3	4	7	1	3	26	23	19	22	21	19
ENSMUSG00000029716	TFR2	0	0	0	0	0	0	1	0	0	0	0	0
ENSMUSG00000029718	PCOLCE	0	0	0	0	0	0	1	1	0	0	1	0
ENSMUSG00000029722	AGFG2	13	17	10	17	16	21	67	62	45	75	62	56

ENSMUSG00000029723	TSC22D4	14	24	22	25	40	17	118	105	89	115	97	106
ENSMUSG00000029725	PPP1R35	8	10	8	15	15	16	71	60	50	50	67	52
ENSMUSG00000029726	MEPCE	6	6	9	3	2	3	22	25	8	19	25	25
ENSMUSG00000029729	ZKSCAN1	4	6	3	8	4	5	26	33	21	22	18	17
ENSMUSG00000029730	MCM7	29	37	33	43	40	22	228	241	213	271	173	239
ENSMUSG00000029735	TPK1	0	1	1	1	0	0	6	6	6	7	3	8
ENSMUSG00000029752	ASNS	16	20	22	25	18	22	101	120	78	124	64	103
ENSMUSG00000029754	DLX6	7	3	7	11	10	1	17	16	8	22	15	15
ENSMUSG00000029759	PON3	0	0	0	0	0	0	0	1	0	0	0	0
ENSMUSG00000029761	CALD1	0	0	0	0	0	0	0	0	0	0	1	0
ENSMUSG00000029763	EXOC4	12	6	11	6	14	13	64	63	45	67	42	57
ENSMUSG00000029771	IRF5	6	7	3	7	6	3	43	47	31	38	46	38
ENSMUSG00000029772	AHCYL2	8	12	8	9	8	10	29	25	17	47	26	28
ENSMUSG00000029775	KLHDC10	6	2	6	12	7	6	39	37	28	33	26	27
ENSMUSG00000029776	HIBADH	12	17	11	12	10	9	64	52	41	64	45	72
ENSMUSG00000029777	GARS	40	47	38	43	42	30	209	257	190	248	191	223
ENSMUSG00000029778	ADCYAP1R1	0	0	1	0	0	0	0	0	0	0	0	0
ENSMUSG00000029780	NT5C3	17	19	23	24	23	14	105	89	67	97	74	103
ENSMUSG00000029782	TMEM209	8	7	5	18	11	6	33	39	28	37	23	26
ENSMUSG00000029787	AVL9	3	3	7	5	2	4	21	19	10	22	9	14
ENSMUSG00000029790	CEP41	0	1	2	1	2	3	3	2	4	8	5	1
ENSMUSG00000029798	HERC6	8	5	7	7	5	4	28	38	30	38	36	26
ENSMUSG00000029802	ABCG2	5	5	5	6	4	4	26	26	19	29	20	29
ENSMUSG00000029804	HERC3	2	2	2	2	0	1	8	17	10	12	9	5
ENSMUSG00000029810	TMEM176B	0	2	1	0	0	2	4	4	2	1	8	3
ENSMUSG00000029814	IGF2BP3	11	16	19	21	18	20	59	49	45	64	26	37
ENSMUSG00000029815	MALSU1	7	9	7	15	7	7	42	55	41	37	39	47
ENSMUSG00000029817	TRA2A	53	62	60	72	68	69	301	313	215	337	210	236
ENSMUSG00000029821	GSDME	12	18	18	24	25	13	71	84	69	97	45	69
ENSMUSG00000029822	OSBPL3	12	8	8	11	13	9	45	38	33	37	33	31
ENSMUSG00000029823	LUC7L2	116	190	145	163	189	155	830	837	653	955	586	706
ENSMUSG00000029826	ZC3HAV1	19	33	19	29	18	30	101	101	96	129	82	97
ENSMUSG00000029828	4921507P07F	0	0	0	0	1	0	0	0	0	0	0	0
ENSMUSG00000029832	NFE2L3	0	0	0	0	0	0	0	1	0	0	0	0
ENSMUSG00000029833	TRIM24	8	10	9	9	10	4	40	48	36	53	36	36
ENSMUSG00000029836	CBX3	14	11	19	27	24	15	57	58	35	59	42	51
ENSMUSG00000029840	MTPN	11	21	20	28	23	28	152	161	102	171	110	114
ENSMUSG00000029847	SLC23A4	0	0	0	0	0	1	1	2	1	2	1	0
ENSMUSG00000029848	STRA8	0	0	0	0	0	0	0	1	0	0	0	0
ENSMUSG00000029859	EPHA1	0	0	0	1	0	0	0	2	2	1	0	0
ENSMUSG00000029860	ZYX	11	20	17	20	21	17	85	110	75	95	91	87
ENSMUSG00000029861	FAM131B	0	0	0	0	0	0	0	0	0	1	1	0
ENSMUSG00000029862	CLCN1	0	0	0	0	0	0	3	1	1	1	0	2
ENSMUSG00000029863	CASP2	7	9	10	15	12	12	85	65	62	88	51	53
ENSMUSG00000029869	EPHB6	0	0	0	0	0	0	1	5	2	5	2	1
ENSMUSG00000029875	CCDC184	0	0	0	0	0	0	1	1	0	0	0	0
ENSMUSG00000029882	2210010C04F	0	0	0	0	0	0	1	0	0	0	0	1
ENSMUSG00000029910	MAD2L1	19	20	27	37	32	32	129	160	125	148	111	144
ENSMUSG00000029911	SSBP1	20	27	19	35	23	29	188	165	133	201	118	132
ENSMUSG00000029913	PRDM5	10	9	9	11	17	9	54	52	33	60	38	45
ENSMUSG00000029916	AGK	1	2	5	1	2	1	19	14	18	17	9	13
ENSMUSG00000029918	MRPS33	5	2	4	2	4	1	14	19	18	14	14	13
ENSMUSG00000029919	HPGDS	17	21	19	30	23	18	90	85	88	110	66	97
ENSMUSG00000029920	SMARCAD1	7	14	9	10	8	7	52	56	40	82	45	29
ENSMUSG00000029922	MKRN1	8	11	8	10	14	8	54	48	38	55	44	38
ENSMUSG00000029923	RAB19	1	3	3	3	4	4	3	12	7	14	4	5
ENSMUSG00000029924	SLC37A3	4	9	5	10	8	7	24	38	21	21	27	25
ENSMUSG00000029925	TBXAS1	1	0	0	0	0	0	0	0	0	0	2	0

ENSMUSG00000029992	GFPT1	5	11	15	10	13	13	90	78	45	106	49	61
ENSMUSG00000029993	NFU1	7	6	5	6	10	2	42	51	36	40	39	37
ENSMUSG00000029998	PCYOX1	2	5	1	4	2	1	13	13	16	19	19	13
ENSMUSG00000029999	TGFA	0	0	0	0	0	0	1	0	0	0	0	0
ENSMUSG00000030000	ADD2	0	0	0	0	0	0	0	0	1	0	0	0
ENSMUSG00000030002	DUSP11	14	14	31	24	32	22	100	114	70	127	67	87
ENSMUSG00000030007	CCT7	40	49	62	67	80	67	466	492	368	531	378	435
ENSMUSG00000030008	PRADC1	1	1	1	1	3	1	12	16	12	9	12	11
ENSMUSG00000030016	ZFP638	36	52	52	64	41	38	254	304	192	298	197	211
ENSMUSG00000030019	FBXL14	20	28	34	30	36	32	136	157	108	128	113	117
ENSMUSG00000030029	LRIG1	1	0	0	0	1	0	2	2	2	4	1	3
ENSMUSG00000030031	KBTBD8	2	3	2	0	1	1	5	5	11	4	3	1
ENSMUSG00000030032	WDR54	0	1	0	0	0	0	1	0	1	1	0	1
ENSMUSG00000030034	INO80B	1	3	1	6	6	4	24	23	18	16	18	23
ENSMUSG00000030035	WBP1	1	1	1	2	0	5	17	11	11	17	9	13
ENSMUSG00000030036	MOGS	8	11	16	23	10	13	98	78	58	84	61	74
ENSMUSG00000030042	POLE4	7	22	10	18	15	18	102	113	83	107	66	54
ENSMUSG00000030045	MRPL19	12	20	17	11	14	17	108	103	100	101	75	86
ENSMUSG00000030047	ARHGAP25	0	0	0	1	0	0	0	0	0	0	0	1
ENSMUSG00000030051	APLF	3	3	2	4	2	2	14	20	14	15	12	12
ENSMUSG00000030055	RAB43	0	0	0	0	1	1	0	1	2	4	1	0
ENSMUSG00000030056	ISY1	6	16	7	11	11	13	60	66	46	53	45	52
ENSMUSG00000030057	CNBP	67	78	55	88	75	93	548	566	400	493	381	409
ENSMUSG00000030058	COPG1	11	21	15	20	23	24	104	112	93	115	81	83
ENSMUSG00000030059	TMF1	80	98	78	114	89	116	461	505	338	518	335	375
ENSMUSG00000030060	HMCES	1	2	1	0	2	1	11	16	13	11	8	12
ENSMUSG00000030061	UBA3	17	15	19	23	17	15	86	98	65	131	77	72
ENSMUSG00000030062	RPN1	24	44	40	41	43	34	256	247	152	287	210	197
ENSMUSG00000030064	FRMD4B	2	1	1	1	3	1	2	2	2	7	1	1
ENSMUSG00000030067	FOXP1	147	206	161	169	179	178	523	452	387	595	367	397
ENSMUSG00000030068	GM20696	1	0	1	0	1	0	1	2	1	1	0	3
ENSMUSG00000030069	PROK2	0	0	0	0	0	0	0	1	0	0	0	0
ENSMUSG00000030074	GXYLT2	0	0	0	0	0	0	0	0	0	0	0	1
ENSMUSG00000030077	CHL1	2	1	1	3	1	1	13	21	13	13	3	21
ENSMUSG00000030079	RUVBL1	1	5	12	8	8	3	47	42	30	34	25	47
ENSMUSG00000030082	SEC61A1	32	37	45	57	37	36	201	230	199	209	138	189
ENSMUSG00000030083	ABTB1	1	1	2	2	4	0	11	9	9	7	6	13
ENSMUSG00000030084	PLXNA1	0	0	0	0	1	0	0	0	0	0	0	1
ENSMUSG00000030088	ALDH1L1	0	0	0	0	0	0	1	0	0	0	0	0
ENSMUSG00000030091	NUP210	17	11	12	15	20	20	67	64	60	68	48	66
ENSMUSG00000030092	CNTN6	1	1	0	0	0	1	1	3	4	6	0	0
ENSMUSG00000030094	XPC	4	3	4	5	8	3	20	32	12	29	19	17
ENSMUSG00000030095	TMEM43	2	5	2	5	3	6	17	30	22	31	15	27
ENSMUSG00000030096	SLC6A6	41	47	44	40	33	55	219	210	136	295	157	164
ENSMUSG00000030098	GRIP2	0	0	0	0	0	0	0	0	0	1	0	0
ENSMUSG00000030101	SUMF1	1	1	3	3	3	2	13	13	10	7	12	9
ENSMUSG00000030102	ITPR1	11	15	12	18	17	15	54	84	49	73	58	57
ENSMUSG00000030103	BHLHE40	1	0	2	1	1	0	12	4	5	13	9	8
ENSMUSG00000030104	EDEM1	13	20	15	21	23	25	65	86	48	69	56	61
ENSMUSG00000030105	ARL8B	8	7	11	8	9	7	50	48	37	50	43	44
ENSMUSG00000030107	USP18	0	0	0	0	0	1	1	2	1	3	1	1
ENSMUSG00000030110	RET	1	2	0	2	0	0	1	2	0	2	0	0
ENSMUSG00000030116	MFAP5	0	0	0	0	0	0	0	0	0	0	0	1
ENSMUSG00000030120	MLF2	9	14	10	10	17	5	59	58	44	57	52	62
ENSMUSG00000030122	PTMS	5	7	9	5	7	3	44	20	32	40	27	25
ENSMUSG00000030123	PLXND1	0	0	0	0	0	0	0	1	0	0	0	0
ENSMUSG00000030124	LAG3	1	0	0	0	1	1	1	0	0	0	0	0
ENSMUSG00000030125	LRRC23	0	0	0	0	0	0	0	0	2	0	0	0

ENSMUSG00000030126	TMCC1	6	12	6	7	10	7	38	31	19	31	18	22
ENSMUSG00000030127	COPS7A	6	2	2	7	6	4	19	20	27	32	17	26
ENSMUSG00000030134	RASGEF1A	0	0	0	0	0	0	0	2	1	2	1	1
ENSMUSG00000030137	TUBA8	1	2	1	1	0	1	5	4	6	4	4	6
ENSMUSG00000030138	BMS1	21	18	10	38	26	22	112	109	81	146	91	81
ENSMUSG00000030145	ZFP248	3	2	2	0	6	1	7	16	4	14	18	11
ENSMUSG00000030149	KLRK1	0	1	1	1	1	0	6	7	1	6	4	5
ENSMUSG00000030154	KLRB1F	1	0	1	1	0	1	2	6	3	4	3	1
ENSMUSG00000030155	CLEC2E	0	0	0	0	0	0	0	1	0	0	0	0
ENSMUSG00000030156	CD69	6	14	17	17	9	16	50	48	39	69	35	31
ENSMUSG00000030157	CLEC2D	6	7	5	4	5	3	20	20	8	19	19	22
ENSMUSG00000030159	CLEC1B	0	0	0	0	0	0	0	1	0	0	0	0
ENSMUSG00000030161	GABARAPL1	6	9	12	3	7	12	40	39	38	46	36	35
ENSMUSG00000030165	KLRD1	3	7	1	10	11	6	38	37	25	47	27	31
ENSMUSG00000030166	RAD52	2	5	3	2	2	2	10	8	12	8	7	13
ENSMUSG00000030168	ADIPOR2	6	3	12	9	8	11	32	44	26	45	24	36
ENSMUSG00000030173	KLRA5	0	0	0	0	0	0	1	0	0	0	1	1
ENSMUSG00000030177	CCDC77	5	8	18	23	15	13	59	71	67	97	44	62
ENSMUSG00000030178	KLRA13-PS	1	3	3	2	4	1	8	8	5	8	8	7
ENSMUSG00000030180	KDM5A	29	31	31	30	46	34	142	146	131	169	126	169
ENSMUSG00000030188	MAGOHB	0	0	0	0	0	0	1	0	0	0	0	0
ENSMUSG00000030189	YBX3	0	0	1	0	0	0	0	0	1	0	0	1
ENSMUSG00000030199	ETV6	1	1	4	6	4	3	10	15	10	10	12	7
ENSMUSG00000030200	BCL2L14	0	0	0	0	1	0	1	0	1	1	0	1
ENSMUSG00000030201	LRP6	10	13	10	5	13	17	45	44	19	39	24	46
ENSMUSG00000030203	DUSP16	4	3	4	8	7	1	30	21	19	26	15	22
ENSMUSG00000030204	DDX47	4	4	14	4	12	10	47	43	48	51	31	49
ENSMUSG00000030213	ATF7IP	7	9	11	22	12	10	61	58	42	69	37	55
ENSMUSG00000030214	PLBD1	0	0	0	0	0	0	1	0	0	0	0	0
ENSMUSG00000030216	WBP11	36	55	33	59	41	42	281	300	218	350	218	220
ENSMUSG00000030217	ART4	0	0	0	1	0	0	1	0	0	4	1	1
ENSMUSG00000030219	ERP27	0	0	0	1	0	0	1	1	0	3	1	0
ENSMUSG00000030220	ARHGDIB	25	26	16	43	23	22	132	144	106	151	89	137
ENSMUSG00000030224	STRAP	9	17	13	18	19	15	124	134	85	111	82	92
ENSMUSG00000030225	DERA	3	7	4	4	3	2	28	11	12	19	18	24
ENSMUSG00000030231	PLEKHA5	4	13	10	9	14	12	36	39	23	35	38	42
ENSMUSG00000030232	AEBP2	6	5	11	13	16	7	62	52	50	46	47	40
ENSMUSG00000030243	RECQL	3	2	4	2	5	4	21	20	15	13	15	20
ENSMUSG00000030244	GYS2	0	0	1	0	0	0	0	0	0	0	0	0
ENSMUSG00000030245	GOLT1B	15	16	10	21	15	14	46	53	47	50	39	44
ENSMUSG00000030247	KCNJ8	0	0	0	0	0	0	0	0	0	1	0	0
ENSMUSG00000030254	RAD18	5	9	6	6	8	10	37	33	32	38	22	35
ENSMUSG00000030255	SSPN	0	2	1	5	3	4	11	17	6	12	12	14
ENSMUSG00000030256	BHLHE41	1	1	0	1	2	0	5	3	6	4	4	4
ENSMUSG00000030257	SRGAP3	0	0	0	0	0	0	3	1	0	1	2	1
ENSMUSG00000030263	LRMP	13	21	22	23	19	23	119	114	98	143	111	121
ENSMUSG00000030264	THUMPD3	1	4	7	10	8	6	34	31	25	33	25	24
ENSMUSG00000030265	KRAS	7	13	7	12	9	7	48	52	35	61	49	39
ENSMUSG00000030268	BCAT1	8	11	10	9	11	6	67	65	55	67	37	60
ENSMUSG00000030269	MTMR14	5	6	9	4	9	4	36	24	24	27	28	21
ENSMUSG00000030270	CPNE9	0	1	0	0	1	0	4	2	0	0	1	2
ENSMUSG00000030271	OGG1	0	2	0	1	2	0	13	6	6	10	8	7
ENSMUSG00000030272	CAMK1	0	1	1	2	1	1	1	4	2	2	0	1
ENSMUSG00000030275	ETNK1	16	21	22	16	19	26	109	100	73	103	58	69
ENSMUSG00000030276	TTLL3	2	1	3	4	2	6	7	10	6	13	8	7
ENSMUSG00000030279	C2CD5	6	4	7	10	7	6	38	51	39	41	46	39
ENSMUSG00000030282	CMAS	4	4	4	2	11	5	35	33	15	23	20	22
ENSMUSG00000030283	ST8SIA1	16	16	15	17	8	8	74	86	29	67	53	58

ENSMUSG00000030284	CRELD1	0	0	0	1	0	0	0	0	0	2	0	0
ENSMUSG00000030286	EMC3	3	2	5	8	10	7	33	37	26	24	25	21
ENSMUSG00000030287	ITPR2	17	21	14	10	23	17	73	60	48	81	49	42
ENSMUSG00000030291	MED21	7	8	5	4	13	12	76	81	43	103	53	69
ENSMUSG00000030292	SMCO2	0	0	0	0	0	0	0	0	1	0	0	0
ENSMUSG00000030298	SEC13	11	10	15	14	23	19	108	129	96	98	81	106
ENSMUSG00000030301	CCDC91	0	0	0	0	0	0	0	0	0	1	0	0
ENSMUSG00000030303	FAR2	9	8	7	5	5	10	21	30	18	29	16	15
ENSMUSG00000030304	ERGIC2	30	38	38	49	40	57	116	100	93	161	96	107
ENSMUSG00000030309	CAPRIN2	2	4	5	12	4	3	20	31	22	34	22	23
ENSMUSG00000030313	DENND5B	0	0	0	1	0	0	1	0	1	0	1	0
ENSMUSG00000030314	ATG7	22	13	17	9	16	15	23	38	31	38	25	31
ENSMUSG00000030315	VGLL4	5	3	5	6	0	6	19	17	11	20	10	17
ENSMUSG00000030316	TAMM41	5	2	1	4	2	3	26	22	26	16	12	21
ENSMUSG00000030319	CAND2	29	39	44	44	52	41	235	207	188	253	229	240
ENSMUSG00000030321	EFCAB12	0	0	0	0	0	0	0	1	0	0	0	0
ENSMUSG00000030322	MBD4	2	4	2	5	4	2	13	24	14	9	16	20
ENSMUSG00000030323	IFT122	1	0	0	1	0	1	2	6	5	3	1	8
ENSMUSG00000030327	NECAP1	1	3	1	3	3	3	14	21	11	20	7	10
ENSMUSG00000030330	ING4	1	0	1	0	1	0	3	4	3	3	2	0
ENSMUSG00000030335	MRPL51	6	8	9	13	7	6	58	55	42	52	39	43
ENSMUSG00000030336	CD27	18	19	20	25	18	32	151	150	120	134	135	135
ENSMUSG00000030337	VAMP1	1	0	0	4	0	1	9	8	8	8	5	4
ENSMUSG00000030341	TNFRSF1A	0	2	0	0	0	0	0	0	1	0	0	1
ENSMUSG00000030345	DYRK4	0	0	0	0	0	0	1	0	2	0	1	0
ENSMUSG00000030346	RAD51AP1	13	9	15	13	13	6	71	60	45	75	51	48
ENSMUSG00000030347	D6WSU163E	1	1	0	2	3	2	8	16	17	10	6	5
ENSMUSG00000030357	FKBP4	18	24	22	32	25	25	152	169	133	163	141	165
ENSMUSG00000030359	PZP	0	0	0	0	0	1	0	0	0	0	0	0
ENSMUSG00000030364	CLEC2H	0	0	0	0	0	1	0	0	0	0	0	0
ENSMUSG00000030374	STRN4	7	10	4	6	5	5	36	33	34	36	24	30
ENSMUSG00000030382	SLC27A5	0	0	0	0	0	0	0	0	0	1	1	0
ENSMUSG00000030386	ZFP606	3	3	3	4	3	5	22	19	21	19	12	17
ENSMUSG00000030393	ZIK1	0	2	3	2	1	4	5	8	9	8	4	5
ENSMUSG00000030397	MARK4	3	3	4	1	3	1	16	7	7	13	10	17
ENSMUSG00000030400	ERCC2	6	13	7	15	11	11	51	38	55	61	44	48
ENSMUSG00000030401	RTN2	0	0	1	0	0	0	0	0	0	0	1	0
ENSMUSG00000030403	VASP	12	16	19	22	32	18	85	95	93	95	73	86
ENSMUSG00000030407	QPCTL	3	5	4	4	5	3	9	11	14	20	12	11
ENSMUSG00000030417	PDCD5	22	24	32	31	29	29	243	233	184	230	159	172
ENSMUSG00000030421	URI1	7	12	8	9	19	13	56	47	45	64	45	38
ENSMUSG00000030423	POP4	4	1	5	3	5	1	27	28	31	36	29	23
ENSMUSG00000030424	ZFP939	0	0	0	2	0	1	0	1	0	0	3	1
ENSMUSG00000030428	TTYH1	0	1	0	0	0	0	1	1	0	0	0	1
ENSMUSG00000030431	TMEM238	0	1	1	2	1	3	9	3	0	2	4	3
ENSMUSG00000030432	RPL28	59	62	55	73	78	80	408	417	293	385	314	394
ENSMUSG00000030435	U2AF2	15	18	6	17	13	15	68	58	41	53	47	60
ENSMUSG00000030446	ZFP273	0	0	2	1	0	0	4	2	0	2	4	2
ENSMUSG00000030447	CYFIP1	9	11	16	14	11	16	64	55	39	78	52	52
ENSMUSG00000030451	HERC2	1	5	7	5	8	8	34	35	27	26	18	25
ENSMUSG00000030452	NIPA2	10	16	8	19	19	8	62	61	47	68	39	50
ENSMUSG00000030465	PSD3	0	0	0	0	0	0	0	0	2	1	0	0
ENSMUSG00000030469	ZFP719	1	2	0	3	0	0	6	10	4	9	3	9
ENSMUSG00000030471	ZDHHC13	2	0	0	3	1	3	20	12	11	18	15	9
ENSMUSG00000030493	FAAP24	1	5	5	3	3	2	19	28	9	22	15	23
ENSMUSG00000030494	RHPN2	1	1	0	0	0	0	1	0	0	2	0	1
ENSMUSG00000030498	GAS2	0	1	2	1	2	5	22	18	22	25	14	14
ENSMUSG00000030500	SLC17A6	0	0	0	0	0	0	0	0	0	0	1	0

ENSMUSG00000030505	PRMT3	5	7	4	4	6	3	36	28	35	39	24	25
ENSMUSG00000030509	ASB7	4	5	6	5	5	1	21	32	19	27	17	15
ENSMUSG00000030510	CERS3	0	1	0	0	0	0	0	1	0	0	0	0
ENSMUSG00000030512	SNRPA1	10	10	12	9	9	12	82	82	46	66	47	62
ENSMUSG00000030519	APBA2	2	2	5	5	3	3	14	13	17	11	6	22
ENSMUSG00000030521	MPHOSPH10	19	32	30	28	30	24	154	161	105	155	135	109
ENSMUSG00000030522	MTMR10	0	2	0	1	1	0	1	0	3	2	3	2
ENSMUSG00000030523	TRPM1	3	6	5	3	2	1	12	17	8	12	11	12
ENSMUSG00000030527	CRTC3	18	16	9	13	7	9	65	58	28	65	36	51
ENSMUSG00000030528	BLM	3	2	7	9	9	13	32	30	35	33	24	32
ENSMUSG00000030530	FURIN	1	6	6	3	3	4	20	23	13	19	15	18
ENSMUSG00000030532	HDDC3	0	0	0	1	0	0	1	0	0	2	0	1
ENSMUSG00000030533	UNC45A	2	3	7	1	4	4	13	18	8	17	11	15
ENSMUSG00000030534	VPS33B	1	0	5	1	4	3	15	6	11	18	6	11
ENSMUSG00000030536	IQGAP1	19	36	21	48	29	26	148	128	82	135	97	111
ENSMUSG00000030538	CIB1	5	6	7	4	2	4	32	19	11	30	27	24
ENSMUSG00000030539	SEMA4B	11	12	9	16	10	7	40	48	32	36	30	48
ENSMUSG00000030541	IDH2	0	0	0	0	0	1	0	0	0	0	0	1
ENSMUSG00000030543	MESP2	0	0	0	0	0	0	0	0	0	0	0	1
ENSMUSG00000030545	PEX11A	0	0	2	0	1	2	6	2	5	6	5	6
ENSMUSG00000030551	NR2F2	0	0	0	0	0	0	0	0	0	1	0	0
ENSMUSG00000030555	TTC23	0	0	0	0	0	0	0	0	1	0	0	1
ENSMUSG00000030556	LRRC28	0	1	0	0	0	0	6	2	0	1	5	7
ENSMUSG00000030557	MEF2A	2	6	7	11	4	8	21	36	19	39	27	17
ENSMUSG00000030560	CTSC	13	18	16	17	17	13	135	153	102	142	116	95
ENSMUSG00000030583	SIPA1L3	3	6	2	6	3	6	11	19	9	9	7	12
ENSMUSG00000030584	DPF1	0	2	0	0	2	3	9	12	6	8	7	4
ENSMUSG00000030587	2200002D01I	2	0	0	0	0	1	3	2	0	1	0	4
ENSMUSG00000030588	YIF1B	4	5	4	7	7	2	22	32	10	17	19	22
ENSMUSG00000030589	RASGRP4	0	1	1	1	1	1	8	7	5	4	1	9
ENSMUSG00000030590	FAM98C	2	2	3	2	2	4	8	17	20	11	9	13
ENSMUSG00000030591	PSMD8	8	18	14	18	12	15	135	123	84	120	93	100
ENSMUSG00000030592	RYR1	0	0	0	0	0	1	0	1	0	0	0	0
ENSMUSG00000030595	NFKBIB	4	4	3	3	2	3	23	26	21	16	14	23
ENSMUSG00000030598	FBXO17	2	4	1	1	4	1	17	18	14	9	11	11
ENSMUSG00000030600	LRFN1	1	1	2	0	0	1	12	4	3	9	2	6
ENSMUSG00000030602	PAK4	1	2	3	2	1	2	4	20	11	12	4	9
ENSMUSG00000030603	PSMC4	17	21	20	23	23	18	111	131	82	102	92	114
ENSMUSG00000030604	ZFP626	2	1	3	4	4	3	10	18	11	13	9	4
ENSMUSG00000030605	MFGE8	1	3	0	3	2	3	15	22	7	18	22	6
ENSMUSG00000030609	AEN	0	2	0	2	1	1	4	5	7	10	6	4
ENSMUSG00000030610	DET1	2	0	0	0	1	0	2	2	7	2	5	2
ENSMUSG00000030611	MRPS11	3	3	3	7	5	2	24	27	19	30	14	26
ENSMUSG00000030612	MRPL46	8	6	5	12	15	5	54	55	31	45	47	41
ENSMUSG00000030614	TMEM126B	4	5	0	8	1	2	17	23	16	14	18	15
ENSMUSG00000030615	TMEM126A	6	11	11	12	6	18	69	70	54	73	35	60
ENSMUSG00000030616	SYTL2	39	80	51	62	59	61	308	332	198	379	230	237
ENSMUSG00000030619	EED	3	7	5	4	2	6	40	42	17	44	30	32
ENSMUSG00000030621	ME3	0	0	0	0	0	0	0	1	0	0	0	0
ENSMUSG00000030629	ZFAND6	40	54	49	55	53	56	229	249	174	263	169	178
ENSMUSG00000030630	FAH	1	6	4	5	3	2	11	12	8	16	14	17
ENSMUSG00000030641	DDIAS	1	2	3	4	6	2	19	20	11	15	7	8
ENSMUSG00000030643	RAB30	0	0	0	0	0	0	0	1	0	0	1	0
ENSMUSG00000030647	NDUFC2	29	49	32	41	32	34	247	253	199	247	173	211
ENSMUSG00000030649	ANAPC15	1	4	2	4	1	2	12	20	9	11	13	10
ENSMUSG00000030652	COQ7	1	0	1	2	1	3	7	12	5	12	6	7
ENSMUSG00000030654	ARL6IP1	15	30	27	20	36	28	114	109	82	115	84	98
ENSMUSG00000030655	SMG1	38	48	58	42	42	40	240	227	201	223	180	191

ENSMUSG00000030659	NUCB2	0	0	0	0	0	0	1	0	0	0	0	0
ENSMUSG00000030660	PIK3C2A	1	0	1	0	1	0	4	5	4	1	4	3
ENSMUSG00000030662	IPO5	21	39	29	31	31	36	189	165	140	220	135	143
ENSMUSG00000030663	1110004F10R	11	10	14	12	16	11	80	68	51	68	53	54
ENSMUSG00000030666	CALCB	0	0	0	0	0	0	0	0	1	0	0	0
ENSMUSG00000030671	PDE3B	7	7	17	15	16	15	71	75	47	66	49	55
ENSMUSG00000030672	MYLPF	1	1	1	1	0	0	6	9	4	3	3	11
ENSMUSG00000030674	QPRT	0	0	0	0	1	0	0	0	1	0	0	0
ENSMUSG00000030677	KIF22	14	17	12	11	16	18	54	75	54	52	53	64
ENSMUSG00000030678	MAZ	18	16	15	16	22	12	88	75	60	108	71	91
ENSMUSG00000030681	MVP	1	7	4	7	2	5	16	22	21	17	20	15
ENSMUSG00000030682	CDIPT	2	3	1	2	4	6	30	29	23	26	21	24
ENSMUSG00000030683	SEZ6L2	1	0	1	1	1	0	2	2	2	2	2	3
ENSMUSG00000030685	KCTD13	0	1	1	2	1	2	11	6	6	5	5	6
ENSMUSG00000030689	INO80E	9	10	4	13	7	6	51	61	51	66	49	61
ENSMUSG00000030691	FCHSD2	3	4	3	12	6	5	22	18	21	32	18	18
ENSMUSG00000030695	ALDOA	41	39	44	39	28	37	301	323	229	237	166	221
ENSMUSG00000030697	PPP4C	34	41	62	50	53	32	141	138	119	147	124	116
ENSMUSG00000030699	TBX6	0	0	1	0	0	1	0	0	0	0	0	0
ENSMUSG00000030703	GDPD3	0	0	0	0	0	0	1	1	1	3	2	2
ENSMUSG00000030704	RAB6A	12	13	9	15	6	9	65	70	51	63	37	52
ENSMUSG00000030706	MRPL48	1	2	1	6	3	3	11	20	13	21	14	15
ENSMUSG00000030707	CORO1A	85	100	89	109	130	92	595	632	463	626	525	521
ENSMUSG00000030708	DNAJB13	1	1	0	2	1	0	1	4	7	1	3	6
ENSMUSG00000030711	SULT1A1	0	0	0	0	0	0	0	1	1	1	0	0
ENSMUSG00000030714	SGF29	2	4	2	5	1	7	18	16	19	34	15	12
ENSMUSG00000030717	NUPR1	0	1	0	1	0	1	1	2	0	0	1	3
ENSMUSG00000030718	PPME1	2	2	4	3	7	4	25	23	18	21	23	22
ENSMUSG00000030720	CLN3	0	1	1	0	1	0	1	2	0	1	2	2
ENSMUSG00000030722	NFATC2IP	2	2	0	12	7	6	13	24	18	26	17	20
ENSMUSG00000030724	CD19	0	0	0	0	0	0	0	0	0	0	0	1
ENSMUSG00000030725	LIPT2	1	0	1	0	1	0	1	1	1	2	1	0
ENSMUSG00000030726	POLD3	12	15	13	22	15	18	58	66	48	76	53	57
ENSMUSG00000030727	RABEP2	1	2	1	1	1	3	6	5	7	5	9	5
ENSMUSG00000030729	PGM2L1	1	2	1	0	2	1	8	6	6	7	2	1
ENSMUSG00000030730	ATP2A1	0	0	0	0	0	0	0	0	0	1	0	0
ENSMUSG00000030733	SH2B1	2	6	2	3	1	8	17	18	18	16	17	19
ENSMUSG00000030738	EIF3C	37	38	34	54	49	58	247	223	207	260	191	222
ENSMUSG00000030741	SPNS1	2	2	7	6	6	6	18	17	13	25	18	18
ENSMUSG00000030742	LAT	17	16	14	23	15	24	86	87	73	78	61	81
ENSMUSG00000030744	RPS3	257	303	277	316	364	305	1501	1674	1135	1520	1231	1363
ENSMUSG00000030745	IL21R	1	0	1	2	4	2	1	4	4	13	4	2
ENSMUSG00000030748	IL4RA	3	5	3	7	4	4	15	16	14	13	6	13
ENSMUSG00000030750	NSMCE1	8	13	5	12	11	17	71	76	59	71	46	63
ENSMUSG00000030751	PSMA1	23	30	29	34	35	33	207	236	165	244	152	159
ENSMUSG00000030752	KDM8	1	1	0	2	0	0	2	3	3	2	2	1
ENSMUSG00000030753	THAP12	13	25	23	28	22	24	76	67	73	93	42	74
ENSMUSG00000030754	COPB1	15	18	12	19	15	14	76	103	56	112	77	85
ENSMUSG00000030759	FAR1	9	7	10	18	9	8	65	64	80	59	48	65
ENSMUSG00000030760	ACER3	1	7	7	5	6	5	49	39	26	36	24	32
ENSMUSG00000030761	MYO7A	1	0	0	0	0	0	0	0	0	0	1	0
ENSMUSG00000030763	LCMT1	1	0	3	4	4	3	18	21	14	18	13	13
ENSMUSG00000030766	ARHGAP17	3	9	3	3	4	0	28	19	8	28	22	22
ENSMUSG00000030768	DISP1	2	0	1	2	1	1	5	3	6	5	3	5
ENSMUSG00000030774	PAK1	0	0	0	0	0	0	0	0	0	1	0	0
ENSMUSG00000030775	TRAT1	0	1	0	1	0	1	1	3	5	3	2	4
ENSMUSG00000030779	RBBP6	55	86	65	85	97	88	381	346	257	454	314	305
ENSMUSG00000030780	BC017158	0	0	0	0	1	0	1	0	2	0	0	0

ENSMUSG00000030782	TGFB1I1	0	0	0	0	0	1	0	0	0	0	2	0
ENSMUSG00000030785	COX6A2	3	3	4	5	6	3	6	6	9	9	8	11
ENSMUSG00000030786	ITGAM	0	0	0	0	0	0	0	0	0	0	1	0
ENSMUSG00000030788	RNF141	0	4	1	4	1	3	9	6	8	9	3	8
ENSMUSG00000030790	ADM	0	1	0	1	0	0	1	5	4	2	3	3
ENSMUSG00000030793	PYCARD	14	13	18	12	8	17	68	65	52	88	52	58
ENSMUSG00000030795	FUS	51	66	84	94	70	83	389	441	352	444	338	343
ENSMUSG00000030796	TEAD2	0	1	0	0	1	1	5	3	1	1	4	4
ENSMUSG00000030798	CD37	2	2	5	7	10	9	19	31	15	26	16	21
ENSMUSG00000030800	PRSS8	0	0	0	0	0	0	0	2	1	1	1	0
ENSMUSG00000030801	KAT8	1	3	2	1	4	2	9	16	6	10	16	15
ENSMUSG00000030802	BCKDK	5	2	2	3	1	4	17	14	12	16	15	19
ENSMUSG00000030805	STX4A	10	7	4	7	5	6	53	46	34	42	21	39
ENSMUSG00000030806	STX1B	0	0	1	0	0	0	0	1	0	0	0	0
ENSMUSG00000030811	FBXL19	3	1	4	2	3	0	10	7	9	14	8	10
ENSMUSG00000030814	BCL7C	4	2	8	3	1	7	19	21	23	17	26	15
ENSMUSG00000030815	PHKG2	7	7	5	6	11	3	20	34	23	20	14	23
ENSMUSG00000030816	RNF40	4	6	5	6	10	4	26	33	34	24	14	31
ENSMUSG00000030822	PRR14	1	2	3	2	2	3	19	18	14	18	5	15
ENSMUSG00000030823	9130019O22I	0	0	1	1	4	2	5	3	2	3	3	3
ENSMUSG00000030824	NUCB1	1	7	10	6	6	6	29	24	24	36	23	36
ENSMUSG00000030826	BCAT2	1	2	1	2	1	2	13	15	13	9	6	8
ENSMUSG00000030830	ITGAL	1	0	1	1	0	0	0	0	0	0	0	1
ENSMUSG00000030835	NOMO1	3	3	4	5	6	5	18	29	17	25	24	30
ENSMUSG00000030839	SERGEF	0	2	1	4	3	1	7	11	5	5	3	3
ENSMUSG00000030842	LAMTOR1	5	3	7	7	6	6	32	28	23	36	26	30
ENSMUSG00000030844	RGS10	0	0	0	0	0	0	0	0	1	0	0	0
ENSMUSG00000030846	TIAL1	14	30	27	23	20	18	149	136	95	141	86	107
ENSMUSG00000030847	BAG3	1	4	8	3	4	6	13	9	12	16	10	10
ENSMUSG00000030850	ATE1	8	5	12	11	7	12	44	44	39	46	34	37
ENSMUSG00000030858	1700007K09F	0	0	0	0	0	0	1	0	0	0	0	0
ENSMUSG00000030861	ACADSB	1	3	0	2	2	3	16	9	5	8	3	10
ENSMUSG00000030867	PLK1	11	11	8	9	11	9	51	62	41	58	47	62
ENSMUSG00000030868	DCTN5	16	12	12	11	13	11	66	101	67	73	71	73
ENSMUSG00000030869	NDUFAB1	17	11	13	18	17	16	113	91	74	94	76	75
ENSMUSG00000030870	UBFD1	11	15	13	14	10	14	61	56	41	61	41	50
ENSMUSG00000030871	EARS2	0	0	1	1	2	1	2	4	1	8	4	5
ENSMUSG00000030872	GGA2	4	8	5	6	10	4	34	26	24	29	22	35
ENSMUSG00000030876	METTL9	15	13	7	19	13	9	134	101	77	126	79	101
ENSMUSG00000030877	MFSD13B	0	0	0	0	0	0	0	1	0	0	0	0
ENSMUSG00000030878	CDR2	4	2	3	3	0	2	14	13	14	16	8	12
ENSMUSG00000030879	MRPL17	14	13	16	19	20	14	96	99	69	115	86	95
ENSMUSG00000030880	POLR3E	5	1	7	6	4	4	40	33	22	30	32	30
ENSMUSG00000030881	ARFIP2	2	9	8	10	8	7	25	44	44	35	24	36
ENSMUSG00000030882	DNHD1	0	5	1	2	4	1	7	12	5	11	8	10
ENSMUSG00000030884	UQCRC2	27	29	25	32	31	25	196	218	151	206	147	177
ENSMUSG00000030887	PDZD9	4	4	3	10	8	10	57	58	46	53	53	49
ENSMUSG00000030888	RRP8	5	10	3	4	10	4	30	33	40	51	35	31
ENSMUSG00000030890	ILK	2	7	1	5	6	3	17	22	17	26	32	25
ENSMUSG00000030894	TPP1	3	7	8	6	8	6	33	29	21	37	26	27
ENSMUSG00000030895	HPX	0	0	0	0	0	0	2	0	0	1	0	0
ENSMUSG00000030921	TRIM30A	4	1	4	3	2	6	22	15	17	25	20	21
ENSMUSG00000030922	LYRM1	1	0	0	1	0	1	3	0	1	3	1	2
ENSMUSG00000030924	REXO5	1	9	2	3	3	5	24	24	11	18	21	19
ENSMUSG00000030929	ERI2	0	1	0	3	1	2	6	10	5	4	7	7
ENSMUSG00000030934	OAT	2	9	8	12	18	10	112	123	59	102	76	71
ENSMUSG00000030935	ACSM3	0	0	0	0	0	0	1	0	1	4	3	1
ENSMUSG00000030942	THUMPD1	6	8	7	3	9	7	49	66	43	65	40	40

ENSMUSG00000030946	LHPP	0	2	1	0	1	1	3	2	1	2	1	3
ENSMUSG00000030956	FAM53B	5	5	4	3	4	4	13	18	16	20	19	17
ENSMUSG00000030960	EEF1AKMT2	1	3	5	4	3	1	14	21	15	21	15	16
ENSMUSG00000030965	ABRAXAS2	5	1	0	4	4	3	19	20	17	22	14	17
ENSMUSG00000030966	TRIM21	1	0	1	5	2	0	9	7	8	6	6	9
ENSMUSG00000030967	ZRANB1	24	29	27	28	33	19	107	143	58	117	83	95
ENSMUSG00000030970	CTBP2	0	0	0	1	1	0	0	0	1	1	0	0
ENSMUSG00000030978	RRM1	3	8	19	12	13	15	85	104	62	83	67	79
ENSMUSG00000030979	UROS	2	1	3	0	0	1	3	7	5	7	2	4
ENSMUSG00000030980	KNOP1	31	59	43	64	39	52	225	281	163	262	205	204
ENSMUSG00000030982	9030624J02R	5	3	5	9	4	5	21	12	13	28	14	19
ENSMUSG00000030983	BCCIP	24	29	20	23	20	20	131	122	121	149	134	92
ENSMUSG00000030986	DHX32	13	22	19	23	19	20	84	82	50	69	56	73
ENSMUSG00000030987	STIM1	21	19	20	27	14	12	98	107	65	81	67	109
ENSMUSG00000030990	PGAP2	6	9	5	9	8	3	46	55	37	39	32	36
ENSMUSG00000031004	MKI67	57	86	58	105	75	79	360	309	252	457	240	249
ENSMUSG00000031007	ATP6AP2	9	11	3	8	5	12	49	53	28	46	34	42
ENSMUSG00000031010	USP9X	3	3	3	4	3	5	15	7	9	17	8	3
ENSMUSG00000031012	CASK	0	0	0	0	0	0	0	2	0	2	1	1
ENSMUSG00000031015	SWAP70	6	8	8	11	9	7	40	50	30	37	27	29
ENSMUSG00000031016	WEE1	2	3	7	5	1	3	11	17	13	27	12	13
ENSMUSG00000031021	TMEM9B	4	4	3	3	4	8	14	18	6	20	15	14
ENSMUSG00000031022	BC051019	0	1	0	0	0	0	1	1	1	0	0	0
ENSMUSG00000031024	ST5	0	0	0	0	0	0	0	1	0	0	0	0
ENSMUSG00000031028	TUB	0	0	0	0	0	0	0	1	1	0	0	0
ENSMUSG00000031029	EIF3F	32	24	27	37	39	38	196	199	153	198	163	168
ENSMUSG00000031059	NDUFB11	12	29	26	27	25	22	187	195	127	158	136	145
ENSMUSG00000031060	RBM10	6	14	15	12	10	14	76	78	61	64	70	54
ENSMUSG00000031065	CDK16	7	9	12	8	8	11	44	52	41	39	32	38
ENSMUSG00000031066	USP11	3	2	1	5	3	1	5	9	14	7	14	8
ENSMUSG00000031068	GLRX3	12	14	18	17	20	14	139	170	113	137	97	102
ENSMUSG00000031072	ORAOV1	0	1	3	2	1	1	7	5	4	12	2	6
ENSMUSG00000031077	FADD	1	1	1	1	1	0	9	6	3	6	3	5
ENSMUSG00000031079	ZFP300	0	0	2	1	1	1	1	3	2	4	3	2
ENSMUSG00000031090	NADSYN1	0	1	0	0	0	0	4	2	1	4	1	7
ENSMUSG00000031093	DOCK11	2	2	3	5	5	5	19	25	13	21	17	4
ENSMUSG00000031095	CUL4B	6	10	8	3	6	7	41	53	25	47	37	34
ENSMUSG00000031101	SASH3	7	15	15	17	18	14	86	91	70	101	72	72
ENSMUSG00000031103	ELF4	4	3	5	6	8	0	26	33	26	37	26	30
ENSMUSG00000031104	RAB33A	0	0	0	0	0	0	0	0	0	1	0	1
ENSMUSG00000031105	SLC25A14	1	0	0	1	1	0	2	3	3	2	3	5
ENSMUSG00000031107	RBMX2	4	0	4	14	6	5	22	19	19	30	17	10
ENSMUSG00000031109	ENOX2	2	1	2	0	1	1	5	3	9	6	0	5
ENSMUSG00000031112	STK26	8	7	9	15	15	8	76	80	50	91	68	56
ENSMUSG00000031129	SLC9A9	2	1	1	5	2	5	10	14	12	7	5	10
ENSMUSG00000031131	VGLL1	3	4	4	5	4	3	18	20	18	36	20	7
ENSMUSG00000031132	CD40LG	3	4	3	2	2	5	18	7	10	14	13	15
ENSMUSG00000031133	ARHGEF6	9	12	8	18	14	9	39	49	37	46	30	32
ENSMUSG00000031134	RBMX	4	3	5	2	2	4	19	19	16	22	12	19
ENSMUSG00000031143	CCDC22	37	70	70	79	73	67	203	271	189	222	184	212
ENSMUSG00000031144	SYP	0	0	0	1	0	0	0	0	0	0	0	0
ENSMUSG00000031145	PRICKLE3	0	0	0	0	0	0	2	2	0	0	1	4
ENSMUSG00000031146	PLP2	0	0	4	0	0	2	5	4	2	8	3	4
ENSMUSG00000031148	GPKOW	2	1	2	3	1	1	10	16	7	15	8	5
ENSMUSG00000031149	PRAF2	2	2	0	0	0	0	6	3	3	9	10	9
ENSMUSG00000031150	CCDC120	0	0	1	0	0	0	0	0	0	0	0	0
ENSMUSG00000031153	GRIPAP1	10	11	8	17	14	20	59	56	61	76	46	59
ENSMUSG00000031154	OTUD5	8	12	9	13	17	11	52	79	53	64	49	63

ENSMUSG00000031155	PIM2	10	13	12	10	5	11	66	65	37	62	50	42
ENSMUSG00000031156	SLC35A2	4	4	3	4	4	4	20	20	17	18	17	15
ENSMUSG00000031157	PQBP1	2	1	6	4	3	4	27	25	19	32	23	18
ENSMUSG00000031158	TIMM17B	4	1	2	3	4	4	15	12	15	23	20	14
ENSMUSG00000031161	HDAC6	0	3	2	3	5	4	10	13	12	13	11	7
ENSMUSG00000031163	GLOD5	0	0	0	0	0	0	0	1	0	0	0	0
ENSMUSG00000031165	WAS	4	9	6	13	17	5	46	45	41	38	30	39
ENSMUSG00000031166	WDR13	4	3	0	3	3	3	11	5	7	11	7	9
ENSMUSG00000031167	RBM3	3	4	3	5	5	4	27	36	14	34	27	29
ENSMUSG00000031168	EBP	5	3	4	5	4	4	32	25	31	26	21	23
ENSMUSG00000031171	FTSJ1	3	0	2	7	2	2	11	18	7	23	16	10
ENSMUSG00000031196	F8	0	0	1	0	0	0	3	2	2	7	2	0
ENSMUSG00000031197	VBP1	7	20	11	13	9	9	83	92	82	113	76	80
ENSMUSG00000031198	FUNDC2	8	7	4	5	7	5	55	66	40	49	37	33
ENSMUSG00000031200	MTCP1	0	0	1	0	1	0	1	2	1	0	0	2
ENSMUSG00000031201	BRCC3	21	17	18	21	15	10	89	72	76	98	54	71
ENSMUSG00000031207	MSN	27	46	29	46	45	40	143	181	128	192	111	138
ENSMUSG00000031220	AWAT2	1	0	0	0	0	0	1	3	3	0	1	1
ENSMUSG00000031221	IGBP1	9	9	4	2	5	4	36	41	29	45	29	34
ENSMUSG00000031226	PBDC1	3	3	8	11	5	3	30	45	32	39	33	28
ENSMUSG00000031227	MAGEE1	1	0	0	0	3	0	2	0	0	0	0	0
ENSMUSG00000031229	ATRX	91	84	93	128	133	116	483	520	366	612	355	370
ENSMUSG00000031231	COX7B	83	95	99	123	130	108	801	907	610	964	596	695
ENSMUSG00000031232	MAGT1	49	50	45	56	52	60	197	196	144	244	155	168
ENSMUSG00000031239	ITM2A	0	3	1	3	1	0	11	11	5	8	7	9
ENSMUSG00000031242	2610002M06	2	1	1	6	0	3	13	12	12	18	8	9
ENSMUSG00000031245	HMGN5	27	36	38	58	31	41	185	225	160	222	143	157
ENSMUSG00000031246	SH3BGRL	14	8	18	15	22	13	80	119	62	132	57	62
ENSMUSG00000031255	SYTL4	1	0	0	0	0	0	0	0	0	0	0	0
ENSMUSG00000031256	CSTF2	6	9	7	4	8	4	62	68	42	66	40	44
ENSMUSG00000031257	NOX1	0	2	2	2	4	1	8	8	5	10	6	11
ENSMUSG00000031258	XKRX	0	0	0	0	0	0	1	0	0	0	0	1
ENSMUSG00000031262	CENPI	4	2	2	2	3	1	20	20	17	16	12	18
ENSMUSG00000031264	BTK	0	0	0	0	0	0	0	1	0	0	0	0
ENSMUSG00000031266	GLA	0	3	3	3	1	2	11	15	9	5	12	9
ENSMUSG00000031274	COL4A5	0	0	0	0	0	0	0	0	0	0	0	1
ENSMUSG00000031278	ACSL4	11	14	10	9	11	10	75	73	45	89	38	49
ENSMUSG00000031290	LRCH2	0	0	0	0	1	1	1	0	0	0	0	0
ENSMUSG00000031292	CDKL5	0	0	0	0	0	0	2	5	2	0	1	1
ENSMUSG00000031295	PHKA2	1	0	3	2	2	4	13	11	6	7	10	13
ENSMUSG00000031297	SLC7A3	0	0	0	0	3	1	1	2	6	3	3	5
ENSMUSG00000031299	PDHA1	14	19	19	17	23	17	76	97	81	84	66	90
ENSMUSG00000031302	NLGN3	0	0	0	1	0	0	0	0	0	0	0	1
ENSMUSG00000031303	MAP3K15	2	1	0	4	1	2	9	8	6	7	4	7
ENSMUSG00000031304	IL2RG	7	9	10	14	12	9	82	60	51	53	36	67
ENSMUSG00000031309	RPS6KA3	16	11	25	33	35	36	151	127	105	175	117	116
ENSMUSG00000031310	ZMYM3	4	2	4	5	1	0	10	19	12	22	19	20
ENSMUSG00000031311	NONO	10	6	7	8	10	9	53	67	36	53	39	27
ENSMUSG00000031312	ITGB1BP2	0	0	0	0	0	0	0	2	1	0	1	1
ENSMUSG00000031314	TAF1	20	30	17	29	26	17	138	145	75	147	101	125
ENSMUSG00000031320	RPS4X	50	67	68	84	63	69	585	699	455	604	437	531
ENSMUSG00000031328	FLNA	8	18	15	16	8	6	46	55	42	40	41	52
ENSMUSG00000031333	ABCB7	14	13	5	9	6	11	41	36	23	36	26	26
ENSMUSG00000031342	GPM6B	0	0	0	0	0	0	1	2	0	0	0	1
ENSMUSG00000031347	CETN2	6	7	7	11	9	7	63	80	61	50	52	53
ENSMUSG00000031349	NSDHL	5	1	3	0	2	3	7	18	13	21	13	19
ENSMUSG00000031352	HCCS	98	119	123	141	120	155	687	701	509	771	443	468
ENSMUSG00000031353	RBBP7	13	14	10	13	14	17	129	119	98	165	99	82

ENSMUSG00000031354	AMELX	0	1	0	1	0	0	0	0	0	0	2	0
ENSMUSG00000031355	ARHGAP6	0	0	0	0	0	1	2	0	0	0	2	0
ENSMUSG00000031357	SYAP1	0	1	0	2	1	1	4	7	6	10	6	4
ENSMUSG00000031358	MSL3	4	5	3	14	6	8	24	34	27	51	20	25
ENSMUSG00000031360	CTPS2	4	4	7	3	5	6	16	17	13	25	11	14
ENSMUSG00000031362	XLR4C	1	6	7	7	4	6	18	33	16	35	14	16
ENSMUSG00000031365	ZFP275	6	11	6	6	12	13	43	35	31	46	38	44
ENSMUSG00000031367	AP1S2	0	1	1	0	1	1	2	4	1	4	2	3
ENSMUSG00000031370	ZRSR2	6	9	6	9	10	7	54	58	37	41	33	40
ENSMUSG00000031371	HAUS7	6	1	7	5	3	1	27	26	15	18	17	17
ENSMUSG00000031378	ABCD1	2	2	0	2	0	1	5	6	3	7	2	4
ENSMUSG00000031380	VEGFD	0	0	0	0	0	0	0	0	1	0	0	0
ENSMUSG00000031381	PIGA	5	3	6	9	5	3	19	29	13	17	19	19
ENSMUSG00000031382	ASB11	0	0	0	0	0	0	0	0	0	1	0	0
ENSMUSG00000031385	PLXNB3	1	0	0	0	0	0	0	1	0	0	0	1
ENSMUSG00000031386	HCFC1	10	15	15	13	13	12	87	68	53	89	57	64
ENSMUSG00000031387	RENBP	0	0	0	0	2	0	0	0	0	1	1	0
ENSMUSG00000031388	NAA10	10	9	11	9	10	12	51	44	45	61	33	50
ENSMUSG00000031389	ARHGAP4	9	11	8	5	6	9	46	52	42	32	47	45
ENSMUSG00000031390	AVPR2	1	2	3	1	2	1	5	9	5	7	9	8
ENSMUSG00000031391	L1CAM	4	3	3	3	3	0	14	8	7	5	10	10
ENSMUSG00000031392	IRAK1	20	16	18	22	26	16	90	83	78	109	75	88
ENSMUSG00000031393	MECP2	6	9	5	6	12	10	23	19	26	26	14	23
ENSMUSG00000031397	TKTL1	1	1	0	0	0	1	2	4	5	2	4	4
ENSMUSG00000031398	PLXNA3	2	3	1	1	2	2	14	7	3	10	7	7
ENSMUSG00000031399	FAM3A	1	0	2	0	2	3	10	4	9	5	10	5
ENSMUSG00000031400	G6PDX	3	1	2	5	6	3	21	18	10	12	19	5
ENSMUSG00000031402	MPP1	1	5	11	5	9	13	31	42	34	32	32	29
ENSMUSG00000031403	DKC1	50	114	71	141	88	97	595	449	346	664	326	411
ENSMUSG00000031410	NXF7	0	0	0	0	0	0	0	1	0	0	0	0
ENSMUSG00000031422	MORF4L2	27	45	38	34	26	34	309	339	229	307	221	291
ENSMUSG00000031424	KIR3DL1	1	0	1	0	0	0	0	0	0	0	0	0
ENSMUSG00000031425	PLP1	0	3	0	2	1	2	6	6	6	5	3	1
ENSMUSG00000031428	ZCCHC18	4	2	3	3	1	2	11	10	3	7	6	5
ENSMUSG00000031429	PSMD10	2	1	0	3	3	1	29	22	24	18	11	16
ENSMUSG00000031431	TSC22D3	2	4	4	4	7	9	51	43	30	54	23	34
ENSMUSG00000031432	PRPS1	6	5	3	7	3	5	26	32	18	42	17	15
ENSMUSG00000031433	RBM41	0	4	3	0	0	0	4	3	7	11	4	7
ENSMUSG00000031438	RNF128	5	2	7	1	2	3	23	24	12	25	11	13
ENSMUSG00000031441	ATP11A	5	2	4	6	6	2	19	18	15	18	10	23
ENSMUSG00000031445	PROZ	0	0	0	0	0	0	0	0	1	1	3	0
ENSMUSG00000031446	CUL4A	5	6	7	6	10	9	42	56	33	45	31	29
ENSMUSG00000031447	LAMP1	13	9	9	16	16	17	64	62	57	70	41	70
ENSMUSG00000031453	RASA3	3	4	4	11	3	5	31	29	23	25	17	15
ENSMUSG00000031458	COPRS	1	0	2	5	2	1	5	8	4	9	7	4
ENSMUSG00000031461	MYOM2	0	0	0	0	0	0	1	0	0	0	0	0
ENSMUSG00000031465	ANGPT2	0	3	2	0	0	4	2	1	0	5	1	2
ENSMUSG00000031467	AGPAT5	15	24	21	21	25	20	88	106	66	109	74	61
ENSMUSG00000031478	NEK3	1	0	2	1	3	0	1	7	5	6	6	3
ENSMUSG00000031479	VPS36	19	20	19	25	20	17	112	125	71	115	106	101
ENSMUSG00000031480	THSD1	0	0	0	0	0	0	0	2	3	0	0	4
ENSMUSG00000031482	SLC25A15	1	3	2	0	1	4	14	17	9	5	9	12
ENSMUSG00000031483	ERLIN2	0	4	1	0	1	2	14	16	17	11	8	7
ENSMUSG00000031485	PLPBP	3	4	4	1	2	1	17	11	14	18	8	12
ENSMUSG00000031487	BRF2	2	1	1	3	1	5	4	3	4	9	7	6
ENSMUSG00000031488	RAB11FIP1	7	6	2	2	4	2	21	22	14	27	15	19
ENSMUSG00000031490	EIF4EBP1	7	7	7	10	15	7	56	55	53	66	49	32
ENSMUSG00000031505	NAXD	7	5	2	2	2	3	18	14	13	18	15	17

ENSMUSG00000031506	PTPN7	38	58	51	55	56	49	201	170	146	200	144	182
ENSMUSG00000031508	ANKRD10	13	16	14	21	22	22	108	122	80	159	87	86
ENSMUSG00000031511	ARHGEF7	1	3	3	7	5	7	26	18	15	12	17	14
ENSMUSG00000031513	LEPROTL1	7	5	8	12	5	7	33	21	25	34	22	24
ENSMUSG00000031516	DCTN6	5	3	1	3	4	3	19	23	29	35	23	18
ENSMUSG00000031519	ASB5	0	0	0	0	0	0	3	0	0	0	0	1
ENSMUSG00000031521	AGA	2	1	0	1	2	3	17	18	18	16	10	7
ENSMUSG00000031527	ERI1	5	3	3	8	10	4	28	28	16	26	28	33
ENSMUSG00000031529	TNKS	7	3	5	3	2	5	26	20	26	23	19	24
ENSMUSG00000031530	DUSP4	1	1	0	1	0	3	8	9	7	9	8	4
ENSMUSG00000031532	SARAF	9	7	4	15	12	7	51	36	32	36	26	40
ENSMUSG00000031533	MRPS31	4	10	6	10	9	5	35	44	18	35	26	30
ENSMUSG00000031534	SMIM19	0	2	0	0	1	1	2	2	1	3	2	1
ENSMUSG00000031536	POLB	2	1	6	4	3	5	29	20	16	22	23	26
ENSMUSG00000031537	IKBKB	12	11	11	8	9	11	42	37	24	55	36	32
ENSMUSG00000031539	AP3M2	0	0	1	1	1	0	5	4	2	3	2	6
ENSMUSG00000031540	KAT6A	6	11	14	14	23	14	47	48	41	60	39	44
ENSMUSG00000031543	ANK1	0	0	0	0	0	0	0	0	0	0	1	0
ENSMUSG00000031545	GPAT4	4	5	13	11	10	17	61	43	34	50	29	31
ENSMUSG00000031546	GINS4	0	4	5	8	3	2	18	20	23	21	19	20
ENSMUSG00000031555	ADAM9	0	3	2	3	10	3	20	33	20	16	24	16
ENSMUSG00000031556	TM2D2	5	2	6	5	3	4	19	17	17	18	19	17
ENSMUSG00000031557	PLEKHA2	6	6	9	10	4	3	28	26	25	24	17	24
ENSMUSG00000031562	DCTD	1	3	4	3	3	6	12	15	16	16	12	17
ENSMUSG00000031563	WWC2	0	1	0	1	3	0	3	5	2	5	1	6
ENSMUSG00000031565	FGFR1	0	1	0	0	0	0	2	0	0	0	1	0
ENSMUSG00000031568	RWDD4A	3	7	7	3	2	7	53	45	26	42	28	40
ENSMUSG00000031570	PLPP5	9	7	2	8	5	7	28	31	31	34	37	31
ENSMUSG00000031574	STAR	2	0	0	1	0	1	5	3	1	4	1	2
ENSMUSG00000031575	ASH2L	15	20	21	16	19	12	86	106	81	120	75	77
ENSMUSG00000031576	KCNU1	0	0	0	0	0	0	0	1	1	0	0	0
ENSMUSG00000031577	TTI2	3	6	1	3	2	2	19	18	10	17	13	10
ENSMUSG00000031578	MAK16	10	9	13	17	12	14	93	76	57	85	76	71
ENSMUSG00000031583	WRN	3	3	4	5	8	12	23	26	23	25	22	19
ENSMUSG00000031584	GSR	22	21	20	25	32	26	105	79	75	117	63	83
ENSMUSG00000031585	GTF2E2	5	9	2	5	14	5	51	41	35	60	45	30
ENSMUSG00000031590	FRG1	8	11	13	11	6	10	56	59	53	57	42	56
ENSMUSG00000031591	ASAH1	5	10	8	12	10	14	90	78	61	90	46	70
ENSMUSG00000031592	PCM1	10	27	22	33	26	28	109	132	82	137	83	90
ENSMUSG00000031594	FGL1	0	0	0	0	0	0	0	1	0	0	0	0
ENSMUSG00000031600	VPS37A	4	5	5	8	9	5	40	34	25	33	25	19
ENSMUSG00000031601	CNOT7	9	9	8	7	12	6	62	71	42	87	62	42
ENSMUSG00000031604	MSMO1	7	10	12	22	18	9	132	122	107	117	85	91
ENSMUSG00000031605	KLHL2	2	8	2	6	8	13	33	34	24	44	21	35
ENSMUSG00000031608	GALNT7	5	8	11	11	11	10	47	63	46	66	49	47
ENSMUSG00000031609	SAP30	5	16	8	10	12	11	68	74	51	67	59	61
ENSMUSG00000031617	TMEM184C	5	7	9	5	12	8	36	36	35	51	30	33
ENSMUSG00000031618	NR3C2	0	0	0	0	0	0	0	0	1	0	0	0
ENSMUSG00000031622	SIN3B	28	44	28	30	25	36	140	135	93	145	102	144
ENSMUSG00000031626	SORBS2	1	0	0	0	0	0	0	0	0	0	1	0
ENSMUSG00000031627	IRF2	10	12	16	18	15	11	81	85	70	98	65	64
ENSMUSG00000031628	CASP3	11	12	21	26	22	22	103	90	75	104	75	91
ENSMUSG00000031629	CENPU	1	2	2	1	4	3	11	11	12	13	9	11
ENSMUSG00000031631	CFAP97	1	2	7	4	9	4	13	12	16	13	12	17
ENSMUSG00000031633	SLC25A4	52	65	59	53	65	57	431	429	323	452	337	356
ENSMUSG00000031634	UFSP2	8	12	10	8	10	10	52	72	37	63	39	46
ENSMUSG00000031637	LRP2BP	0	0	1	0	0	0	0	0	2	1	0	0
ENSMUSG00000031639	TLR3	0	0	0	0	0	0	1	1	0	0	0	1

ENSMUSG00000031641	CBR4	0	1	0	1	3	1	7	12	6	5	2	6
ENSMUSG00000031642	SH3RF1	1	2	2	1	2	6	12	19	11	13	11	13
ENSMUSG00000031644	NEK1	6	5	4	3	7	6	18	20	13	19	13	14
ENSMUSG00000031652	N4BP1	5	4	7	8	9	9	41	29	25	29	26	37
ENSMUSG00000031657	HEATR3	7	2	2	3	2	2	19	26	12	22	21	18
ENSMUSG00000031659	ADCY7	1	7	6	5	2	7	29	28	23	32	16	19
ENSMUSG00000031660	BRD7	23	24	12	27	31	15	111	105	79	116	82	94
ENSMUSG00000031661	NKD1	2	5	1	4	4	3	16	17	11	25	7	9
ENSMUSG00000031662	SNX20	1	1	3	2	2	1	12	13	3	9	7	13
ENSMUSG00000031666	RBL2	8	18	12	11	12	13	61	62	43	54	36	49
ENSMUSG00000031667	AKTIP	16	9	17	19	29	13	55	52	34	57	36	36
ENSMUSG00000031668	EIF2AK3	6	6	5	6	8	9	44	43	30	42	21	29
ENSMUSG00000031669	GINS3	4	4	3	4	1	2	25	23	21	22	12	17
ENSMUSG00000031671	SETD6	4	6	9	5	8	5	27	27	18	29	22	21
ENSMUSG00000031672	GOT2	3	7	2	6	5	1	23	43	17	23	23	20
ENSMUSG00000031673	CDH11	0	0	0	0	0	0	1	1	0	0	0	0
ENSMUSG00000031681	SMAD1	3	1	1	3	4	3	14	6	9	18	6	8
ENSMUSG00000031683	LSM6	10	20	19	26	23	19	87	94	60	77	71	77
ENSMUSG00000031684	SLC10A7	0	2	3	3	1	2	14	19	9	17	9	12
ENSMUSG00000031691	TNPO2	6	20	8	19	13	13	71	78	62	86	44	67
ENSMUSG00000031696	VPS35	9	15	12	18	17	16	86	131	89	92	90	83
ENSMUSG00000031697	ORC6	25	22	31	29	27	19	161	151	145	210	151	151
ENSMUSG00000031698	MYLK3	0	0	0	0	1	0	1	0	0	0	0	0
ENSMUSG00000031700	GPT2	0	2	2	4	2	0	10	9	6	11	5	8
ENSMUSG00000031701	DNAJA2	12	11	10	14	14	12	95	115	65	96	73	74
ENSMUSG00000031703	ITFG1	2	2	3	1	6	4	15	18	3	17	17	11
ENSMUSG00000031706	RFX1	0	1	2	1	2	5	10	6	8	8	12	11
ENSMUSG00000031708	TECR	14	21	20	33	24	23	144	148	103	123	96	107
ENSMUSG00000031711	ZFP330	5	14	13	12	11	9	57	43	44	55	44	48
ENSMUSG00000031712	IL15	0	2	2	1	2	1	9	11	4	10	4	3
ENSMUSG00000031715	SMARCA5	30	46	48	66	49	53	270	298	222	316	207	214
ENSMUSG00000031722	HP	0	0	0	0	0	0	0	0	0	1	0	0
ENSMUSG00000031723	TXNL4B	3	4	2	3	2	1	3	8	5	4	7	4
ENSMUSG00000031728	ZFP821	5	1	2	7	2	6	14	10	13	18	8	10
ENSMUSG00000031729	IST1	8	6	6	7	7	3	44	55	45	44	34	41
ENSMUSG00000031730	DHODH	1	3	3	2	4	0	18	14	13	18	14	16
ENSMUSG00000031731	AP1G1	6	15	11	7	11	9	58	46	40	62	41	44
ENSMUSG00000031732	PHLPP2	7	4	5	5	7	3	22	20	14	19	13	14
ENSMUSG00000031736	CRNDE	0	0	0	1	1	0	6	2	5	2	5	0
ENSMUSG00000031740	MMP2	1	0	0	0	0	1	1	0	1	0	0	2
ENSMUSG00000031748	GNAO1	1	1	0	0	0	0	1	0	0	1	1	0
ENSMUSG00000031749	ST3GAL2	1	7	0	1	1	1	5	10	6	3	7	7
ENSMUSG00000031750	IL34	0	0	0	0	0	0	1	0	0	1	0	0
ENSMUSG00000031751	AMFR	22	49	36	38	35	35	153	172	131	192	116	159
ENSMUSG00000031753	COG4	3	3	1	2	6	7	17	7	8	7	10	9
ENSMUSG00000031754	NUDT21	5	4	6	10	8	6	40	45	36	32	28	23
ENSMUSG00000031755	BBS2	0	1	2	0	0	1	1	6	3	2	2	2
ENSMUSG00000031756	CENPN	2	3	4	2	3	4	19	16	17	17	19	14
ENSMUSG00000031758	CDYL2	11	11	10	12	13	6	31	35	26	38	20	36
ENSMUSG00000031762	MT2	0	0	0	0	0	0	1	4	0	0	0	1
ENSMUSG00000031765	MT1	1	0	1	0	0	0	0	0	0	0	0	0
ENSMUSG00000031770	HERPUD1	4	4	5	4	10	4	49	56	48	51	41	35
ENSMUSG00000031772	CNTNAP4	0	0	0	0	0	0	2	0	0	0	0	0
ENSMUSG00000031774	FAM192A	9	11	4	8	7	10	51	73	31	53	35	45
ENSMUSG00000031776	ARL2BP	14	26	17	17	25	12	114	123	93	114	78	100
ENSMUSG00000031781	CIAPIN1	4	6	4	6	2	1	32	27	18	19	17	28
ENSMUSG00000031782	COQ9	6	6	4	4	6	8	26	19	16	18	9	20
ENSMUSG00000031783	POLR2C	9	16	15	14	14	17	72	78	58	71	60	82

ENSMUSG00000031787	KATNB1	3	4	5	2	3	4	15	8	14	17	9	15
ENSMUSG00000031791	TMEM38A	1	0	0	0	1	0	3	3	2	2	6	1
ENSMUSG00000031792	USB1	3	1	1	3	2	2	12	19	10	12	7	16
ENSMUSG00000031796	CFAP20	5	10	2	5	1	9	37	28	18	32	18	32
ENSMUSG00000031799	TPM4	14	19	14	23	17	16	94	107	63	112	80	85
ENSMUSG00000031802	PHXR4	0	1	1	2	2	1	1	1	4	4	2	3
ENSMUSG00000031803	B3GNT3	0	2	0	0	0	0	2	3	4	4	1	0
ENSMUSG00000031805	JAK3	3	2	1	0	1	0	5	8	4	5	6	8
ENSMUSG00000031807	PGLS	7	10	6	3	5	9	49	62	38	53	34	46
ENSMUSG00000031808	SLC27A1	1	0	0	0	0	0	0	1	0	0	0	4
ENSMUSG00000031809	1700018B08F	0	0	0	0	0	0	0	0	0	0	0	1
ENSMUSG00000031812	MAP1LC3B	6	11	7	6	7	3	53	53	36	42	28	46
ENSMUSG00000031813	MVB12A	3	6	5	4	8	5	37	27	23	16	21	23
ENSMUSG00000031816	MTHFSD	2	3	5	5	1	2	11	18	13	22	11	11
ENSMUSG00000031818	COX4I1	86	87	93	111	87	100	664	686	477	647	513	598
ENSMUSG00000031819	EMC8	5	5	7	9	5	7	48	38	26	49	35	41
ENSMUSG00000031820	BABAM1	9	12	9	10	4	1	41	39	34	40	37	48
ENSMUSG00000031821	GINS2	13	20	14	30	20	14	92	95	72	80	66	73
ENSMUSG00000031822	GSE1	6	17	16	18	18	13	65	59	45	67	65	60
ENSMUSG00000031823	ZDHHC7	3	2	2	4	5	3	15	16	13	9	9	14
ENSMUSG00000031826	USP10	12	16	16	14	25	24	118	128	78	104	114	93
ENSMUSG00000031827	COTL1	34	47	47	48	36	53	252	257	180	256	174	207
ENSMUSG00000031828	KLHL36	0	1	0	1	2	0	10	7	1	7	3	8
ENSMUSG00000031831	DNAAF1	2	3	0	1	0	1	5	2	1	3	2	1
ENSMUSG00000031832	TAF1C	0	2	3	1	7	1	9	11	13	9	8	10
ENSMUSG00000031833	MAST3	5	9	2	9	7	7	21	27	32	32	22	28
ENSMUSG00000031834	PIK3R2	4	4	6	2	5	4	19	23	12	21	22	21
ENSMUSG00000031835	MBTPS1	9	7	9	11	11	11	61	47	47	69	53	59
ENSMUSG00000031838	IFI30	7	7	8	7	1	10	45	62	26	58	37	53
ENSMUSG00000031839	HSBP1	11	14	10	11	27	12	90	97	89	85	54	66
ENSMUSG00000031840	RAB3A	1	2	0	0	0	2	6	6	1	4	3	9
ENSMUSG00000031842	PDE4C	4	3	4	5	7	8	11	21	19	16	15	23
ENSMUSG00000031843	MPHOSPH6	5	7	6	11	13	5	45	53	43	58	36	43
ENSMUSG00000031844	HSD17B2	1	0	0	0	0	0	1	2	3	1	0	1
ENSMUSG00000031848	LSM4	7	9	12	18	7	16	71	65	39	68	64	69
ENSMUSG00000031858	MAU2	6	6	10	12	12	19	50	49	40	57	35	58
ENSMUSG00000031860	PBX4	0	1	0	3	0	2	8	7	3	6	0	7
ENSMUSG00000031861	LPAR2	0	1	0	0	1	0	1	0	2	1	1	1
ENSMUSG00000031862	ATP13A1	10	11	13	16	14	13	57	51	45	61	36	50
ENSMUSG00000031864	INTS10	1	2	1	3	2	0	13	15	5	10	6	11
ENSMUSG00000031865	DCTN1	1	0	1	3	3	2	13	10	13	15	4	12
ENSMUSG00000031872	BEAN1	1	0	0	1	0	0	0	0	0	0	0	0
ENSMUSG00000031875	CMTM3	0	0	0	1	0	0	2	0	1	0	1	0
ENSMUSG00000031878	NAE1	4	12	9	10	3	9	39	39	28	55	27	40
ENSMUSG00000031879	FAM96B	5	5	3	1	2	3	14	15	19	14	11	9
ENSMUSG00000031880	RRAD	1	1	1	1	1	2	7	13	4	5	6	2
ENSMUSG00000031883	CAR7	0	0	0	1	0	0	1	1	0	1	2	3
ENSMUSG00000031885	CBFB	5	13	12	15	12	6	56	48	36	85	36	58
ENSMUSG00000031887	TRADD	2	3	1	6	3	3	14	15	13	8	8	10
ENSMUSG00000031889	D230025D16I	1	3	3	3	1	1	11	13	10	12	11	13
ENSMUSG00000031893	TSNAXIP1	0	0	1	0	0	0	3	0	1	0	0	1
ENSMUSG00000031896	CTRL	0	0	0	0	0	1	0	4	1	1	0	2
ENSMUSG00000031897	PSMB10	18	13	14	15	16	10	85	88	66	80	72	78
ENSMUSG00000031901	DUS2	2	0	1	1	3	1	12	10	4	5	7	3
ENSMUSG00000031902	NFATC3	21	18	13	17	13	12	71	90	57	90	68	56
ENSMUSG00000031903	PLA2G15	1	6	4	4	3	2	14	13	10	13	9	8
ENSMUSG00000031904	SLC7A6	3	6	8	8	8	5	41	54	31	47	38	33
ENSMUSG00000031906	SMPD3	0	1	2	0	0	2	2	5	3	6	5	5

ENSMUSG00000031907	ZFP90	0	0	0	0	1	2	1	0	0	1	3	2
ENSMUSG00000031910	HAS3	0	0	0	0	0	0	3	1	0	2	0	0
ENSMUSG00000031913	VPS4A	2	1	4	4	3	2	12	23	10	20	14	11
ENSMUSG00000031916	COG8	1	9	10	5	0	3	25	27	25	22	19	26
ENSMUSG00000031917	NIP7	14	10	19	14	24	17	133	131	84	129	109	100
ENSMUSG00000031918	MTMR2	16	6	13	16	14	17	71	82	65	70	64	64
ENSMUSG00000031921	TERF2	6	9	4	7	6	5	36	36	32	47	32	29
ENSMUSG00000031922	CEP57	10	8	16	18	15	14	81	74	59	87	54	63
ENSMUSG00000031924	CYB5B	16	21	15	25	28	21	85	106	79	106	78	92
ENSMUSG00000031925	MAML2	0	1	0	0	0	0	0	0	0	0	1	2	0
ENSMUSG00000031928	MRE11A	1	5	3	3	1	2	35	37	22	26	24	29
ENSMUSG00000031930	WWP2	1	2	1	4	1	0	12	7	15	12	18	17
ENSMUSG00000031931	ANKRD49	4	9	5	8	9	11	53	54	59	47	48	33
ENSMUSG00000031932	GPR83	0	0	0	0	0	0	1	0	0	0	0	0
ENSMUSG00000031933	IZUMO1R	0	0	1	0	0	0	0	0	0	0	0	1
ENSMUSG00000031934	PANX1	4	1	1	6	3	6	24	21	13	24	15	17
ENSMUSG00000031935	MED17	4	5	7	5	2	4	26	32	29	39	14	25
ENSMUSG00000031938	4931406C07F	1	3	0	2	0	1	10	8	10	13	14	2
ENSMUSG00000031939	TAF1D	68	114	90	127	112	88	466	526	314	549	371	365
ENSMUSG00000031948	KARS	12	34	22	22	26	24	120	133	89	105	90	118
ENSMUSG00000031949	ADAT1	1	0	0	1	1	2	8	7	4	6	5	6
ENSMUSG00000031950	GABARAPL2	4	2	8	4	6	4	15	18	11	14	24	15
ENSMUSG00000031951	TMEM231	1	4	3	2	1	0	12	13	3	12	4	12
ENSMUSG00000031952	CHST5	0	0	0	0	0	0	0	0	0	1	0	0
ENSMUSG00000031953	TMEM170	1	2	0	0	0	1	0	3	2	3	0	3
ENSMUSG00000031954	CFDP1	8	10	10	12	6	9	72	74	52	82	63	49
ENSMUSG00000031955	BCAR1	0	1	2	2	1	2	4	5	7	4	6	7
ENSMUSG00000031958	LDHD	0	0	0	5	0	1	4	10	5	8	5	5
ENSMUSG00000031959	WDR59	2	2	4	0	2	6	16	16	6	17	7	15
ENSMUSG00000031960	AARS	12	11	15	23	18	16	92	75	60	67	58	79
ENSMUSG00000031963	BMPER	0	0	0	0	0	0	2	0	2	1	0	0
ENSMUSG00000031967	AFG3L1	11	10	8	15	8	7	42	66	26	62	43	45
ENSMUSG00000031969	ACAD8	0	2	3	5	2	1	8	19	11	20	13	6
ENSMUSG00000031971	CCSAP	5	4	7	3	2	5	21	30	14	24	17	20
ENSMUSG00000031972	ACTA1	0	0	0	0	0	0	3	1	0	0	0	0
ENSMUSG00000031974	ABCB10	11	11	11	19	12	17	113	84	72	99	77	102
ENSMUSG00000031976	URB2	2	5	8	1	3	4	26	17	18	27	25	14
ENSMUSG00000031979	COG2	2	5	8	3	8	6	22	29	16	26	16	25
ENSMUSG00000031982	ARV1	0	0	1	0	1	0	5	5	5	1	2	3
ENSMUSG00000031983	2310022B05F	2	1	0	1	0	0	4	4	3	2	0	2
ENSMUSG00000031984	2810004N23I	15	17	23	28	29	20	102	101	92	112	101	85
ENSMUSG00000031985	GNPAT	3	4	2	6	6	11	32	30	25	45	29	31
ENSMUSG00000031986	SPRTN	1	4	4	2	4	1	15	17	9	14	14	11
ENSMUSG00000031987	EGLN1	13	15	7	12	17	14	67	68	56	66	49	43
ENSMUSG00000031988	VPS26B	1	3	4	2	5	0	13	12	14	31	7	19
ENSMUSG00000031990	JAM3	0	0	1	1	0	0	0	0	0	0	1	2
ENSMUSG00000031993	SNX19	4	2	1	1	2	2	9	8	4	6	9	5
ENSMUSG00000031995	ST14	2	1	3	0	3	2	12	8	12	11	6	8
ENSMUSG00000031996	APLP2	1	5	5	5	6	4	33	25	27	33	26	42
ENSMUSG00000032000	BIRC3	3	2	2	2	3	0	14	13	8	17	11	14
ENSMUSG00000032002	DCUN1D5	5	3	6	6	9	3	59	64	56	48	41	39
ENSMUSG00000032009	SESN3	2	7	7	6	1	7	26	19	17	33	14	15
ENSMUSG00000032010	USP2	0	1	0	0	3	0	3	6	2	1	1	5
ENSMUSG00000032011	THY1	0	1	0	0	0	0	0	0	0	0	1	0
ENSMUSG00000032018	SC5D	11	10	9	23	13	10	84	99	74	82	60	70
ENSMUSG00000032020	UBASH3B	14	15	21	20	19	16	70	74	55	68	58	63
ENSMUSG00000032021	CRTAM	0	1	0	1	0	0	1	1	0	0	0	0
ENSMUSG00000032023	JHY	0	0	0	0	0	0	0	1	0	0	0	0

ENSMUSG00000032026	REXO2	15	10	19	10	13	12	71	71	59	71	55	67
ENSMUSG00000032030	CUL5	19	22	18	31	22	18	104	115	83	140	85	88
ENSMUSG00000032035	ETS1	2	1	6	2	2	1	11	9	10	14	5	9
ENSMUSG00000032038	ST3GAL4	2	1	1	1	4	1	13	7	10	9	7	6
ENSMUSG00000032040	DCPS	2	1	3	4	1	3	22	23	19	15	13	19
ENSMUSG00000032041	TIRAP	1	2	0	0	0	0	2	3	3	16	6	9
ENSMUSG00000032042	SRPR	5	12	4	12	7	7	38	43	32	45	22	32
ENSMUSG00000032044	RPUSD4	4	3	4	4	3	3	27	17	15	16	15	20
ENSMUSG00000032046	ABHD12	3	2	1	1	5	1	15	22	5	17	9	6
ENSMUSG00000032047	ACAT1	30	34	28	39	39	39	239	252	156	267	205	198
ENSMUSG00000032050	RDX	32	38	28	58	26	33	198	175	121	258	161	145
ENSMUSG00000032051	FDX1	8	6	10	17	5	6	49	44	25	45	31	43
ENSMUSG00000032053	POU2AF1	0	0	1	2	1	0	5	1	4	3	2	3
ENSMUSG00000032058	PPP2R1B	3	5	6	7	4	3	36	22	24	35	18	27
ENSMUSG00000032059	ALG9	0	0	1	0	0	0	4	5	5	5	2	5
ENSMUSG00000032064	DIXDC1	1	0	0	0	0	0	0	0	0	0	0	0
ENSMUSG00000032067	PTS	2	1	2	3	0	0	2	17	7	9	4	8
ENSMUSG00000032077	BUD13	2	2	3	2	1	1	9	15	12	13	10	5
ENSMUSG00000032078	ZPR1	3	8	5	7	10	8	35	39	33	37	30	29
ENSMUSG00000032085	TAGLN	0	1	1	0	0	1	0	1	0	1	1	3
ENSMUSG00000032086	BACE1	0	3	3	0	2	1	10	6	9	8	9	6
ENSMUSG00000032087	DSCAML1	0	0	0	0	1	0	0	0	0	0	0	0
ENSMUSG00000032093	CD3E	27	55	34	40	37	38	174	187	138	206	133	185
ENSMUSG00000032094	CD3D	1	1	1	1	1	2	10	7	8	10	3	4
ENSMUSG00000032096	ARCN1	16	15	16	9	19	14	86	121	90	108	75	79
ENSMUSG00000032097	DDX6	32	47	28	45	48	52	237	233	170	240	177	184
ENSMUSG00000032101	DDX25	0	0	1	0	0	0	2	2	1	2	2	2
ENSMUSG00000032103	PUS3	9	9	4	11	13	5	45	53	45	58	36	43
ENSMUSG00000032109	NLRX1	0	0	0	0	0	0	0	0	0	0	1	0
ENSMUSG00000032112	TRAPPC4	3	7	3	4	4	6	27	36	15	33	18	22
ENSMUSG00000032113	CHEK1	8	15	13	3	10	7	66	63	45	61	41	50
ENSMUSG00000032114	SLC37A4	3	2	2	1	4	3	10	10	14	7	4	10
ENSMUSG00000032115	HYOU1	18	33	26	30	22	17	101	112	77	121	90	119
ENSMUSG00000032116	STT3A	14	23	22	33	17	15	156	145	99	147	117	119
ENSMUSG00000032118	FEZ1	0	0	0	0	0	0	0	0	0	0	1	0
ENSMUSG00000032119	HINFP	3	1	4	2	2	1	8	11	9	16	9	13
ENSMUSG00000032120	C2CD2L	3	2	0	2	2	1	5	6	8	10	7	7
ENSMUSG00000032121	TMEM218	0	0	0	0	0	0	1	1	2	1	2	3
ENSMUSG00000032122	SLC37A2	0	0	0	0	0	0	0	1	0	1	2	0
ENSMUSG00000032123	DPAGT1	3	4	4	2	2	3	17	16	10	14	12	7
ENSMUSG00000032126	HMBS	29	25	36	34	28	29	64	66	42	55	37	56
ENSMUSG00000032127	VPS11	6	5	1	10	3	3	11	17	11	20	14	20
ENSMUSG00000032131	ABCG4	0	2	0	0	0	0	3	0	0	2	0	2
ENSMUSG00000032135	MCAM	0	0	0	0	0	0	0	1	0	0	0	0
ENSMUSG00000032171	PIN1	6	7	10	9	7	13	55	70	43	62	50	66
ENSMUSG00000032175	TYK2	2	6	5	3	4	2	16	14	15	18	7	15
ENSMUSG00000032178	ILF3	25	35	22	33	29	29	129	145	107	167	94	130
ENSMUSG00000032180	TMED1	1	6	4	3	3	2	17	24	12	11	14	19
ENSMUSG00000032181	SCG3	0	0	0	0	0	0	1	0	0	0	0	0
ENSMUSG00000032182	YIPF2	1	2	4	4	4	4	16	15	12	18	11	10
ENSMUSG00000032184	LYSMD2	3	6	2	8	6	6	43	37	20	55	37	26
ENSMUSG00000032185	CARM1	6	8	10	11	6	6	46	35	47	35	36	45
ENSMUSG00000032186	TMOD2	0	0	0	0	0	0	0	0	2	0	0	0
ENSMUSG00000032187	SMARCA4	33	51	33	44	28	27	184	188	160	179	132	183
ENSMUSG00000032192	GNB5	0	1	1	0	0	0	0	2	1	1	0	1
ENSMUSG00000032193	LDLR	7	15	9	9	7	5	52	54	31	67	31	53
ENSMUSG00000032194	KANK2	0	0	1	0	0	0	0	0	0	0	0	0
ENSMUSG00000032199	POLR2M	22	21	18	25	21	23	116	115	71	103	80	99

ENSMUSG00000032202	RAB27A	7	8	3	10	5	5	47	40	33	45	30	33
ENSMUSG00000032204	AQP9	2	1	0	0	4	0	1	2	1	1	1	2
ENSMUSG00000032212	SLTM	74	73	72	88	74	98	364	394	259	383	293	290
ENSMUSG00000032215	RSL24D1	21	17	21	36	23	15	116	98	80	123	93	95
ENSMUSG00000032216	NEDD4	42	44	53	64	61	62	346	347	275	350	242	289
ENSMUSG00000032217	RNF111	4	7	8	6	6	13	44	53	37	60	46	36
ENSMUSG00000032218	CCNB2	7	17	12	10	12	11	51	48	45	63	55	58
ENSMUSG00000032220	MYO1E	7	7	4	4	6	5	36	46	26	34	26	24
ENSMUSG00000032221	MNS1	33	43	34	45	44	38	146	170	147	236	146	135
ENSMUSG00000032224	FAM81A	1	2	2	1	2	1	0	0	3	2	1	0
ENSMUSG00000032226	GCNT3	2	4	11	3	7	4	12	16	6	16	10	12
ENSMUSG00000032228	TCF12	6	7	7	8	9	9	37	47	34	52	38	31
ENSMUSG00000032231	ANXA2	8	14	19	18	27	28	92	93	82	103	85	89
ENSMUSG00000032235	ICE2	1	7	8	6	7	8	26	39	17	41	14	32
ENSMUSG00000032238	RORA	12	7	5	6	6	15	20	26	21	34	14	18
ENSMUSG00000032239	RP9	52	47	49	53	44	64	252	268	192	269	186	207
ENSMUSG00000032243	ITGA11	0	0	0	1	0	0	1	0	0	0	0	0
ENSMUSG00000032244	FEM1B	3	5	5	3	5	8	26	12	11	34	19	14
ENSMUSG00000032245	CLN6	3	0	5	2	3	0	11	10	6	5	5	8
ENSMUSG00000032249	ANP32A	15	32	26	37	25	22	177	209	160	166	137	139
ENSMUSG00000032251	IRAK1BP1	2	2	0	2	3	1	12	15	15	25	8	15
ENSMUSG00000032252	GLCE	1	2	3	5	3	3	12	15	9	18	3	7
ENSMUSG00000032253	PHIP	33	34	22	52	44	43	139	154	106	195	122	116
ENSMUSG00000032254	KIF23	14	28	28	21	30	27	106	141	90	143	86	108
ENSMUSG00000032258	LCA5	2	5	6	3	2	4	24	30	22	40	15	16
ENSMUSG00000032261	SH3BGRL2	1	0	2	1	0	0	2	1	2	2	3	4
ENSMUSG00000032263	BCKDHB	1	2	3	3	1	1	12	19	8	12	14	11
ENSMUSG00000032264	ZW10	5	1	5	6	6	7	26	22	14	35	18	24
ENSMUSG00000032265	FAM46A	16	2	8	18	18	12	42	45	22	37	34	30
ENSMUSG00000032267	USP28	3	4	1	8	11	6	25	29	26	24	21	25
ENSMUSG00000032278	PAQR5	0	0	0	0	0	0	0	3	0	0	2	0
ENSMUSG00000032279	IDH3A	12	12	10	18	14	8	101	118	82	128	62	104
ENSMUSG00000032280	TLE3	8	11	16	12	14	17	56	54	46	57	50	48
ENSMUSG00000032281	ACSBG1	0	0	0	0	2	1	5	4	1	1	0	1
ENSMUSG00000032288	IMP3	14	13	23	29	21	22	98	93	96	81	83	75
ENSMUSG00000032290	PTPN9	1	2	4	0	0	0	6	8	4	13	7	11
ENSMUSG00000032293	IREB2	9	11	13	15	16	15	60	52	53	60	45	51
ENSMUSG00000032294	PKM	37	34	30	30	46	37	373	382	245	307	234	259
ENSMUSG00000032295	MAN2C1	3	2	3	5	7	5	16	14	17	14	12	8
ENSMUSG00000032297	CELF6	0	1	0	0	0	0	0	0	0	0	0	0
ENSMUSG00000032298	NEIL1	0	0	0	0	0	0	3	3	1	4	2	5
ENSMUSG00000032299	COMMD4	5	6	4	6	6	3	12	22	8	11	10	16
ENSMUSG00000032300	1700017B05F	2	3	1	6	2	1	21	16	13	13	13	18
ENSMUSG00000032301	PSMA4	16	26	26	21	25	23	203	157	137	182	140	177
ENSMUSG00000032303	CHRNA3	0	0	0	0	0	0	0	0	0	1	0	0
ENSMUSG00000032305	FAM219B	7	6	11	9	9	7	52	32	24	31	19	34
ENSMUSG00000032306	MPI	0	0	0	0	0	0	2	1	1	4	1	2
ENSMUSG00000032307	UBE2Q2	8	9	7	8	5	6	32	31	25	38	26	29
ENSMUSG00000032308	ULK3	1	2	1	4	2	3	3	6	4	5	5	8
ENSMUSG00000032309	FBXO22	3	9	4	9	7	5	29	29	21	36	30	25
ENSMUSG00000032311	NRG4	0	2	2	0	0	2	4	8	8	6	7	7
ENSMUSG00000032312	CSK	31	34	40	38	43	38	228	232	182	224	185	213
ENSMUSG00000032314	ETFA	17	14	14	15	13	20	135	159	114	150	116	84
ENSMUSG00000032316	CLK3	11	6	19	10	11	7	46	61	52	56	55	53
ENSMUSG00000032318	ISL2	0	0	0	0	2	0	0	1	0	2	1	0
ENSMUSG00000032320	RCN2	17	13	14	11	12	19	75	94	55	97	86	64
ENSMUSG00000032322	PSTPIP1	6	11	5	4	9	9	26	21	16	32	26	21
ENSMUSG00000032324	TSPAN3	4	8	22	9	13	9	89	88	57	93	54	61

ENSMUSG00000032328	TMEM30A	29	40	44	39	41	39	121	145	121	131	122	92
ENSMUSG00000032329	HMG20A	3	2	5	3	8	4	13	9	10	13	5	10
ENSMUSG00000032330	COX7A2	35	45	50	65	64	60	480	468	319	489	365	380
ENSMUSG00000032332	COL12A1	0	0	0	1	0	0	0	2	1	0	0	1
ENSMUSG00000032333	STOML1	1	1	1	2	1	2	3	5	4	5	4	7
ENSMUSG00000032336	NPTN	7	7	3	6	9	3	53	39	20	58	25	32
ENSMUSG00000032342	MTO1	2	6	7	3	4	8	28	21	16	13	17	24
ENSMUSG00000032343	IMPG1	0	0	1	0	0	0	0	0	1	0	0	0
ENSMUSG00000032344	MB21D1	0	0	0	0	0	0	0	0	1	0	1	1
ENSMUSG00000032349	ELOVL5	8	5	5	5	13	9	45	46	43	79	49	36
ENSMUSG00000032350	GCLC	5	9	9	11	6	11	42	48	26	39	26	30
ENSMUSG00000032352	LRRC1	1	2	1	3	3	5	12	15	14	22	4	11
ENSMUSG00000032353	TMED3	0	1	0	1	2	1	7	5	7	6	3	7
ENSMUSG00000032356	RASGRF1	0	0	0	0	1	0	0	0	0	0	0	0
ENSMUSG00000032357	TINAG	0	0	0	0	0	0	0	0	0	0	0	1
ENSMUSG00000032366	TPM1	18	17	20	32	26	23	110	138	100	137	92	103
ENSMUSG00000032368	ZIC1	1	0	0	0	0	0	0	0	0	0	0	0
ENSMUSG00000032370	LACTB	7	4	2	6	9	2	30	23	23	30	19	28
ENSMUSG00000032372	PLSCR2	1	0	0	0	0	0	0	0	0	0	0	0
ENSMUSG00000032373	CAR12	8	11	13	21	7	6	36	50	38	47	35	44
ENSMUSG00000032375	APH1B	1	1	0	1	1	2	2	2	1	3	3	0
ENSMUSG00000032376	USP3	7	7	12	5	8	9	33	39	38	48	38	36
ENSMUSG00000032380	DAPK2	0	0	0	0	0	0	0	0	0	1	0	0
ENSMUSG00000032381	FAM96A	11	21	18	19	20	20	147	138	84	113	119	118
ENSMUSG00000032382	SNX1	6	6	4	1	0	6	29	26	12	23	15	21
ENSMUSG00000032383	PPIB	22	20	18	26	19	22	133	157	116	150	107	136
ENSMUSG00000032384	CSNK1G1	5	5	5	5	2	4	19	34	29	46	31	27
ENSMUSG00000032386	TRIP4	2	5	2	3	4	3	18	18	16	13	15	11
ENSMUSG00000032387	RBPMS2	0	0	0	0	0	0	0	0	0	0	1	0
ENSMUSG00000032388	SPG21	4	3	9	4	8	4	31	37	27	39	31	28
ENSMUSG00000032392	PARP16	0	0	1	0	1	0	6	3	2	2	3	3
ENSMUSG00000032393	DPP8	4	9	10	7	7	9	40	41	32	31	24	34
ENSMUSG00000032396	DIS3L	2	3	4	2	2	0	8	13	8	7	16	9
ENSMUSG00000032397	TIPIN	12	14	8	22	24	15	145	132	84	152	91	111
ENSMUSG00000032398	SNAPC5	2	3	0	2	2	4	7	5	4	8	11	5
ENSMUSG00000032399	RPL4	314	341	385	397	414	344	2241	2438	1925	2376	1746	2089
ENSMUSG00000032400	ZWILCH	13	6	12	14	12	11	65	51	52	65	39	43
ENSMUSG00000032401	LCTL	1	0	0	0	0	0	3	0	4	2	2	1
ENSMUSG00000032402	SMAD3	7	8	4	13	5	9	57	56	46	57	45	42
ENSMUSG00000032403	2300009A05R	5	8	4	9	11	8	42	29	29	37	36	33
ENSMUSG00000032405	PIAS1	6	10	6	8	8	9	40	41	39	54	34	41
ENSMUSG00000032407	U2SURP	41	56	63	72	68	70	315	286	247	381	253	257
ENSMUSG00000032409	ATR	4	5	2	9	7	12	17	29	17	19	18	15
ENSMUSG00000032410	XRN1	10	11	10	11	18	6	63	39	29	50	26	33
ENSMUSG00000032411	TFDP2	5	7	12	8	7	10	39	35	26	62	32	35
ENSMUSG00000032412	ATP1B3	36	48	45	48	49	53	212	234	187	212	166	172
ENSMUSG00000032413	RASA2	2	5	9	6	2	9	13	7	13	17	11	8
ENSMUSG00000032415	UBE2CBP	1	1	1	0	0	1	3	4	5	6	5	5
ENSMUSG00000032417	RWDD2A	0	1	0	0	0	0	0	4	0	1	4	0
ENSMUSG00000032418	ME1	0	0	0	0	0	2	5	5	4	5	4	1
ENSMUSG00000032420	NT5E	0	0	0	0	0	0	0	0	1	1	0	0
ENSMUSG00000032422	SNX14	7	11	7	6	8	7	37	41	30	43	41	38
ENSMUSG00000032423	SYNCRIP	19	41	34	58	42	36	221	240	153	269	151	178
ENSMUSG00000032425	ZFP949	0	0	1	3	0	1	7	5	4	5	6	10
ENSMUSG00000032431	CRTAP	0	1	1	1	2	1	4	3	3	3	3	3
ENSMUSG00000032434	CMTM6	7	7	4	9	12	8	37	24	17	26	10	24
ENSMUSG00000032435	DYNC1LI1	8	11	11	17	8	14	63	76	53	61	49	70
ENSMUSG00000032436	CMTM7	13	12	17	13	18	20	98	88	61	79	79	77

ENSMUSG00000032437	STT3B	11	10	6	11	15	10	55	59	39	76	39	41
ENSMUSG00000032440	TGFBR2	1	3	1	0	0	0	11	13	6	6	6	5
ENSMUSG00000032443	ZCWPW2	0	0	0	0	0	0	0	0	0	0	0	2
ENSMUSG00000032446	EOMES	1	0	0	0	1	0	2	1	0	0	0	1
ENSMUSG00000032449	SLC25A36	12	13	8	16	12	9	64	62	44	66	56	49
ENSMUSG00000032456	NMNAT3	1	0	1	3	0	0	10	4	3	7	5	10
ENSMUSG00000032458	COPB2	12	14	20	14	15	22	89	103	82	93	80	85
ENSMUSG00000032459	MRPS22	4	4	5	8	11	4	54	49	39	45	31	43
ENSMUSG00000032462	PIK3CB	2	0	2	5	2	0	7	8	7	11	8	7
ENSMUSG00000032463	FAIM	6	4	5	11	6	11	40	44	31	41	25	30
ENSMUSG00000032468	ARMC8	10	5	10	5	7	7	39	31	25	55	30	33
ENSMUSG00000032469	DBR1	2	3	6	3	3	6	44	55	34	27	33	15
ENSMUSG00000032470	MRAS	0	0	0	0	0	0	0	0	0	1	0	1
ENSMUSG00000032475	NCK1	2	5	5	3	5	0	23	22	20	24	16	13
ENSMUSG00000032477	CDC25A	11	11	9	14	22	12	92	99	57	87	65	74
ENSMUSG00000032478	NME6	1	1	1	4	4	3	12	10	7	14	8	11
ENSMUSG00000032479	MAP4	11	4	6	6	9	9	45	40	33	37	32	30
ENSMUSG00000032480	DHX30	10	4	6	10	9	7	38	54	28	38	34	35
ENSMUSG00000032481	SMARCC1	42	49	50	71	53	45	157	149	142	230	126	150
ENSMUSG00000032482	CSPG5	0	0	0	0	0	0	0	0	1	3	1	1	2
ENSMUSG00000032485	SCAP	17	22	17	17	16	21	96	91	67	102	86	94
ENSMUSG00000032489	KIF9	0	0	0	1	0	0	1	1	0	0	0	0
ENSMUSG00000032491	NRADD	0	0	0	0	0	0	1	1	0	1	1	1
ENSMUSG00000032492	PTH1R	0	0	0	0	0	1	0	0	0	0	0	0
ENSMUSG00000032495	LRRC2	0	0	4	1	1	4	3	2	2	4	3	3
ENSMUSG00000032497	LRRFIP2	5	6	5	3	1	1	23	17	14	15	11	10
ENSMUSG00000032498	MLH1	3	4	5	4	6	7	29	36	24	24	20	22
ENSMUSG00000032501	TRIB1	0	3	3	2	2	3	20	21	13	21	7	18
ENSMUSG00000032503	ARPP21	3	9	9	6	8	8	30	38	23	47	27	29
ENSMUSG00000032504	PDCD6IP	17	16	17	25	12	21	143	122	105	160	91	121
ENSMUSG00000032507	FBXL2	0	2	2	3	6	0	7	7	8	19	7	7
ENSMUSG00000032508	MYD88	1	1	1	1	4	2	19	24	16	16	17	16
ENSMUSG00000032512	WDR48	10	8	16	9	13	8	41	53	41	44	30	46
ENSMUSG00000032513	GORASP1	0	3	0	3	0	0	2	2	2	3	0	3
ENSMUSG00000032514	TTC21A	1	0	0	0	0	0	0	0	0	0	0	0
ENSMUSG00000032515	CSRNP1	3	4	1	1	3	2	3	6	10	7	6	9
ENSMUSG00000032518	RPSA	176	199	224	242	237	222	1216	1312	948	1145	962	1112
ENSMUSG00000032519	SLC25A38	2	1	0	3	3	4	8	10	9	5	8	5
ENSMUSG00000032523	HHATL	0	0	0	0	0	0	1	0	0	0	0	0
ENSMUSG00000032525	NKTR	27	43	40	35	43	46	183	184	116	256	108	160
ENSMUSG00000032526	SS18L2	25	28	29	34	29	27	152	176	106	151	108	153
ENSMUSG00000032527	PCCB	0	0	2	3	2	2	12	13	7	13	11	5
ENSMUSG00000032534	CEP63	1	1	1	0	0	1	5	7	5	5	2	5
ENSMUSG00000032536	TRAK1	2	0	2	0	2	2	6	12	2	15	6	3
ENSMUSG00000032537	EPHB1	0	0	0	0	1	0	2	0	0	3	0	2
ENSMUSG00000032540	ABHD5	3	1	0	2	0	1	4	14	8	17	9	10
ENSMUSG00000032547	RYK	27	22	39	30	39	27	95	95	71	98	68	79
ENSMUSG00000032549	RAB6B	1	0	0	0	0	1	6	11	5	6	2	7
ENSMUSG00000032551	1110059G10I	3	4	3	4	5	3	26	18	15	31	20	18
ENSMUSG00000032553	SRPRB	4	2	2	5	1	3	24	22	21	24	18	24
ENSMUSG00000032555	TOPBP1	16	30	33	34	30	32	156	170	160	184	128	136
ENSMUSG00000032556	BFSP2	0	3	0	1	0	1	3	5	2	4	4	3
ENSMUSG00000032557	UBA5	5	9	12	7	12	10	45	44	23	38	29	30
ENSMUSG00000032558	NPHP3	0	2	0	2	0	1	2	3	4	3	1	3
ENSMUSG00000032560	DNAJC13	4	10	9	2	9	5	32	39	22	32	25	39
ENSMUSG00000032562	GNAI2	32	35	23	36	30	30	156	196	131	182	96	163
ENSMUSG00000032563	MRPL3	1	7	3	3	4	5	27	28	27	33	20	28
ENSMUSG00000032565	NUDT16	2	0	1	0	1	1	1	5	0	4	5	2

ENSMUSG00000032567	ASTE1	1	4	3	4	4	3	16	10	17	14	6	12
ENSMUSG00000032570	ATP2C1	8	14	8	8	3	14	39	51	33	50	28	39
ENSMUSG00000032571	PIK3R4	3	4	5	3	8	5	18	21	12	31	18	18
ENSMUSG00000032575	MANF	10	13	9	15	6	12	58	84	45	57	43	51
ENSMUSG00000032577	MAPKAPK3	4	3	3	0	4	2	23	27	11	19	21	17
ENSMUSG00000032579	HEMK1	1	0	1	0	1	1	3	2	2	1	0	0
ENSMUSG00000032580	RBM5	16	30	20	26	34	28	122	111	83	155	77	96
ENSMUSG00000032582	RBM6	20	20	18	37	18	20	100	91	64	132	83	96
ENSMUSG00000032583	MON1A	0	0	1	4	0	0	17	6	7	4	11	7
ENSMUSG00000032586	TRAIP	4	4	1	8	5	6	20	33	21	29	30	22
ENSMUSG00000032589	BSN	1	4	7	5	5	6	35	28	18	22	27	31
ENSMUSG00000032590	APEH	5	4	6	2	11	3	43	35	22	31	25	28
ENSMUSG00000032593	AMIGO3	0	0	0	1	0	1	1	2	0	0	0	2
ENSMUSG00000032594	IP6K1	6	10	2	4	5	6	26	32	27	34	37	30
ENSMUSG00000032595	CDHR4	0	1	0	0	0	0	0	0	0	0	2	0
ENSMUSG00000032596	UBA7	2	2	0	2	0	0	4	2	3	2	0	3
ENSMUSG00000032598	NCKIPSD	3	4	4	6	6	5	24	26	23	14	18	14
ENSMUSG00000032599	IP6K2	4	5	9	8	10	7	31	28	19	47	29	21
ENSMUSG00000032601	PRKAR2A	37	43	49	59	57	56	249	232	182	231	189	197
ENSMUSG00000032602	SLC25A20	5	0	3	3	2	0	16	10	11	19	10	16
ENSMUSG00000032604	QARS	13	12	17	14	23	11	86	91	55	85	67	57
ENSMUSG00000032606	NICN1	0	0	0	0	0	2	3	3	4	2	3	4
ENSMUSG00000032607	AMT	1	0	2	0	2	1	6	6	3	12	6	9
ENSMUSG00000032609	KLHDC8B	0	0	0	1	0	0	2	2	0	0	1	0
ENSMUSG00000032611	1700102P08F	0	0	1	1	0	0	2	2	0	0	1	0
ENSMUSG00000032612	USP4	4	6	1	5	12	6	28	41	28	35	25	25
ENSMUSG00000032615	NT5M	1	0	0	1	1	2	10	8	5	8	9	8
ENSMUSG00000032621	SREK1	47	47	48	54	47	49	248	243	180	264	189	202
ENSMUSG00000032624	EML4	11	23	14	20	28	13	81	58	52	76	65	69
ENSMUSG00000032633	FLCN	8	10	11	11	9	10	52	57	53	52	42	53
ENSMUSG00000032637	ATXN2L	26	32	31	36	28	17	104	109	70	120	90	105
ENSMUSG00000032641	GPR19	2	0	0	2	0	0	5	8	7	10	11	5
ENSMUSG00000032643	FHL3	26	19	39	41	40	31	82	80	37	82	52	71
ENSMUSG00000032648	PYGM	0	0	0	0	0	0	1	0	0	1	4	2
ENSMUSG00000032652	CREBL2	3	6	2	8	7	3	16	11	11	18	16	10
ENSMUSG00000032657	FAM189B	5	13	5	6	3	4	30	28	15	21	18	25
ENSMUSG00000032666	1700025G04I	5	5	12	7	2	4	16	23	15	13	10	18
ENSMUSG00000032667	PON2	4	8	8	12	8	10	38	33	26	41	26	30
ENSMUSG00000032673	PRORSD1	1	7	2	4	2	0	22	12	12	31	11	13
ENSMUSG00000032688	MALT1	4	6	4	6	3	3	19	19	26	23	14	11
ENSMUSG00000032691	NLRP3	0	0	0	0	0	0	0	0	0	1	0	0
ENSMUSG00000032698	LMO2	0	0	0	1	0	2	4	2	5	4	3	2
ENSMUSG00000032705	EXD2	4	1	5	2	2	4	11	10	9	7	9	12
ENSMUSG00000032712	2810474O19I	45	97	62	76	81	48	295	309	234	335	259	260
ENSMUSG00000032714	SYDE1	0	0	0	0	2	0	1	0	1	1	6	0
ENSMUSG00000032715	TRIB3	1	1	3	3	0	3	15	8	6	12	12	9
ENSMUSG00000032717	MDFI	0	0	0	0	0	0	0	1	0	0	0	0
ENSMUSG00000032719	SBSPON	0	0	0	0	0	0	0	0	0	1	0	0
ENSMUSG00000032724	ABTB2	2	3	2	3	10	0	11	13	4	6	9	14
ENSMUSG00000032727	MIER3	4	3	6	10	3	4	21	31	19	32	17	22
ENSMUSG00000032733	SNX33	0	0	1	0	0	1	1	3	2	0	2	1
ENSMUSG00000032737	INPPL1	10	2	5	8	5	5	21	38	23	33	21	27
ENSMUSG00000032740	CCDC88A	1	1	0	0	0	0	1	1	1	1	0	0
ENSMUSG00000032741	TPCN1	2	4	2	6	2	7	18	22	13	18	14	21
ENSMUSG00000032743	D430042O09	0	2	1	1	1	0	4	7	2	3	1	4
ENSMUSG00000032745	GPBP1	7	19	22	14	14	26	85	106	82	122	69	64
ENSMUSG00000032750	GAB3	0	2	0	0	0	1	5	4	3	4	1	0
ENSMUSG00000032754	SLC8B1	1	2	0	2	2	3	8	6	6	4	0	2

ENSMUSG00000032757	BET1	3	3	5	5	6	9	52	41	39	52	31	34
ENSMUSG00000032763	ILVBL	1	1	0	7	2	3	15	13	15	14	8	15
ENSMUSG00000032769	TRPA1	1	1	1	1	0	1	5	4	1	4	6	4
ENSMUSG00000032777	GTF3C1	3	4	7	5	3	2	23	23	15	29	18	23
ENSMUSG00000032782	CNTROB	4	2	3	3	6	6	16	28	16	28	17	13
ENSMUSG00000032783	TROAP	6	2	1	5	4	4	25	33	23	23	21	18
ENSMUSG00000032786	ALAS1	0	2	1	1	6	3	14	16	13	18	15	14
ENSMUSG00000032788	PDXK	1	6	4	4	5	3	18	23	11	19	16	19
ENSMUSG00000032803	CDV3	24	28	20	25	31	21	122	115	84	126	108	104
ENSMUSG00000032806	SLC10A3	0	0	0	0	0	0	4	2	2	3	5	2
ENSMUSG00000032812	ARAP1	1	1	3	0	0	0	7	10	4	8	4	9
ENSMUSG00000032815	FANCA	8	10	7	7	15	6	40	33	17	29	18	21
ENSMUSG00000032816	IGDCC4	0	0	0	0	0	1	0	1	1	1	0	1
ENSMUSG00000032834	PWP2	7	4	3	5	5	4	17	18	16	22	17	12
ENSMUSG00000032840	2410131K14F	2	1	0	1	2	1	5	7	5	6	9	10
ENSMUSG00000032842	ABCC10	1	3	1	3	1	1	5	6	7	9	8	10
ENSMUSG00000032849	ABCC4	1	3	4	1	3	3	14	11	7	16	16	7
ENSMUSG00000032850	RNFT2	0	0	0	1	0	0	1	2	1	2	1	0
ENSMUSG00000032855	PKD1	6	4	2	1	7	1	14	10	7	14	12	14
ENSMUSG00000032867	FBXW8	1	0	7	6	8	3	24	35	23	32	26	29
ENSMUSG00000032869	PSMF1	2	2	4	2	5	1	16	14	11	12	7	10
ENSMUSG00000032870	SMAP2	5	5	3	7	4	5	15	20	16	33	20	21
ENSMUSG00000032872	CYB5R4	5	3	4	6	9	7	33	30	23	31	15	29
ENSMUSG00000032875	ARHGEF17	51	46	62	60	52	69	267	306	226	295	193	268
ENSMUSG00000032883	ACSL3	9	16	12	13	18	14	94	91	87	106	79	88
ENSMUSG00000032892	RANGRF	0	0	1	0	0	1	3	3	1	1	2	3
ENSMUSG00000032897	NFYC	2	6	3	4	4	4	18	21	15	20	13	19
ENSMUSG00000032898	FBXO21	6	4	2	8	2	4	9	20	15	23	18	15
ENSMUSG00000032899	STYK1	0	0	1	0	1	1	2	4	1	1	2	4
ENSMUSG00000032902	SLC16A1	154	223	191	248	223	188	695	788	644	793	541	716
ENSMUSG00000032905	ATG12	4	9	5	6	6	9	32	35	26	43	31	23
ENSMUSG00000032908	SGPP2	0	0	0	0	0	0	0	0	0	4	1	2
ENSMUSG00000032911	CSPG4	0	0	0	0	0	1	0	0	0	0	0	0
ENSMUSG00000032913	LRIG2	5	1	5	5	5	2	18	14	13	28	14	21
ENSMUSG00000032932	HSPA13	4	9	8	10	8	7	35	28	24	40	26	20
ENSMUSG00000032939	NUP93	13	15	12	11	14	11	64	64	54	90	48	62
ENSMUSG00000032946	RASGRP2	0	1	0	2	3	0	3	0	1	0	1	0
ENSMUSG00000032952	AP4B1	0	2	3	2	3	1	4	13	3	11	9	9
ENSMUSG00000032959	PEBP1	9	5	5	4	8	11	40	39	34	30	45	44
ENSMUSG00000032965	IFT57	2	2	3	6	4	1	11	14	7	10	17	14
ENSMUSG00000032966	FKBP1A	12	11	16	15	12	11	117	112	88	104	84	109
ENSMUSG00000032977	FAM207A	4	4	8	4	2	2	24	26	21	27	14	30
ENSMUSG00000032997	CHPF	8	14	10	14	20	14	59	73	63	77	64	60
ENSMUSG00000032998	FOXJ3	8	3	6	8	8	10	20	22	17	30	9	16
ENSMUSG00000033004	MYCBP2	37	32	27	44	48	41	146	152	114	175	121	129
ENSMUSG00000033009	OGFOD1	4	2	3	5	2	4	10	22	6	21	10	21
ENSMUSG00000033014	TRIM33	9	16	16	27	12	20	80	76	57	95	68	78
ENSMUSG00000033016	NFATC1	2	3	4	3	1	4	15	27	15	23	14	23
ENSMUSG00000033020	POLR2F	14	21	21	30	26	25	120	142	93	144	103	132
ENSMUSG00000033021	GMPPA	1	3	2	3	6	5	11	14	11	17	11	14
ENSMUSG00000033022	CDO1	0	2	0	1	1	1	6	2	1	2	1	0
ENSMUSG00000033024	KLRA9	0	0	0	0	0	0	0	1	0	0	0	1
ENSMUSG00000033029	1700088E04F	0	0	0	0	0	0	1	0	0	1	0	1
ENSMUSG00000033031	C330027C09F	34	66	39	48	65	46	227	235	154	259	198	183
ENSMUSG00000033033	CALHM2	0	0	0	0	0	0	1	0	2	2	0	0
ENSMUSG00000033036	GM7879	0	0	0	0	0	0	1	0	0	0	0	0
ENSMUSG00000033039	MICALL1	0	1	3	1	2	2	6	8	9	7	10	6
ENSMUSG00000033047	EIF3L	14	7	9	17	12	12	91	112	74	70	60	85

ENSMUSG00000033054	NPAT	9	12	7	11	13	6	54	50	40	62	34	43
ENSMUSG00000033055	ANKRD54	1	0	5	6	5	2	19	24	10	13	22	24
ENSMUSG00000033059	PYGB	5	5	8	5	5	7	47	54	42	47	54	61
ENSMUSG00000033060	LMO7	0	1	1	0	2	1	0	2	1	3	2	3
ENSMUSG00000033065	PFKM	5	1	3	7	3	0	13	12	10	17	10	16
ENSMUSG00000033066	GAS7	0	0	0	0	0	0	0	0	0	0	1	0
ENSMUSG00000033068	ENTPD6	7	6	5	5	3	5	26	41	22	36	30	36
ENSMUSG00000033075	SENP1	5	7	11	12	8	8	35	36	25	34	32	31
ENSMUSG00000033083	TBC1D4	3	3	2	3	3	3	19	8	8	18	20	14
ENSMUSG00000033088	TRIOBP	1	1	4	3	0	6	13	12	10	9	10	12
ENSMUSG00000033096	APMAP	2	4	3	1	4	2	27	35	24	29	13	17
ENSMUSG00000033099	NOL12	5	1	3	7	3	3	22	17	21	24	17	12
ENSMUSG00000033102	CDC14B	3	3	0	3	4	4	13	14	13	24	9	8
ENSMUSG00000033105	LSS	4	5	7	12	3	8	46	48	34	53	40	34
ENSMUSG00000033106	SLC7A6OS	2	6	2	4	10	4	21	15	14	25	23	18
ENSMUSG00000033107	RNF125	5	13	7	12	5	8	27	38	22	37	19	31
ENSMUSG00000033114	SLC35D2	0	1	0	0	0	0	2	1	1	3	4	5
ENSMUSG00000033124	ATG9A	1	3	2	0	1	0	2	2	0	6	2	3
ENSMUSG00000033126	YBEY	2	2	0	0	0	0	7	6	4	4	6	12
ENSMUSG00000033128	GGA1	4	5	4	1	0	3	19	15	18	13	6	24
ENSMUSG00000033147	SLC22A15	1	2	2	3	3	0	9	4	5	13	6	16
ENSMUSG00000033157	ABHD10	4	1	4	1	2	1	20	25	13	16	10	19
ENSMUSG00000033159	CNPPD1	8	13	5	9	9	6	52	55	46	48	38	43
ENSMUSG00000033161	ATP1A1	23	39	32	22	30	36	133	170	136	152	105	145
ENSMUSG00000033166	DIS3	8	17	17	18	10	13	74	82	56	114	58	41
ENSMUSG00000033177	TMPRSS7	0	0	1	0	0	0	3	1	1	1	2	2
ENSMUSG00000033184	TMED7	24	26	30	40	33	32	146	137	137	184	93	136
ENSMUSG00000033186	MZT1	11	15	11	17	12	10	202	162	99	239	122	120
ENSMUSG00000033187	BC016579	0	1	0	0	1	0	0	0	1	0	1	0
ENSMUSG00000033192	LPCAT2	1	0	0	1	0	0	3	2	2	2	1	1
ENSMUSG00000033207	MAMDC2	0	0	0	0	0	0	1	0	0	0	1	0
ENSMUSG00000033208	S100B	0	0	0	1	1	0	0	0	1	0	1	0
ENSMUSG00000033209	TTC28	1	1	6	5	6	3	17	15	14	28	15	14
ENSMUSG00000033210	SLC9C1	0	0	0	0	0	0	0	0	1	0	0	0
ENSMUSG00000033213	AA467197	1	0	0	1	0	0	3	2	3	6	2	2
ENSMUSG00000033216	EEFSEC	7	5	2	6	4	4	24	29	18	15	23	16
ENSMUSG00000033219	GM9758	0	0	0	0	0	0	1	0	0	0	0	0
ENSMUSG00000033220	RAC2	25	22	16	25	35	29	154	179	139	141	116	131
ENSMUSG00000033222	TTF2	5	8	9	10	10	8	48	47	20	53	31	38
ENSMUSG00000033227	WNT6	39	24	30	36	33	21	178	176	146	179	138	202
ENSMUSG00000033228	SCAF11	18	42	28	44	42	49	173	214	142	228	159	174
ENSMUSG00000033233	TRIM45	12	22	18	19	24	15	78	93	63	75	61	71
ENSMUSG00000033237	ARID2	21	25	25	20	24	19	68	70	58	100	56	56
ENSMUSG00000033249	HSF4	0	0	0	1	1	0	1	1	1	1	1	1
ENSMUSG00000033253	SZT2	8	9	9	8	9	4	12	20	14	21	15	25
ENSMUSG00000033255	GM5134	0	0	0	0	0	0	1	0	0	0	1	0
ENSMUSG00000033256	SHF	0	0	0	0	0	0	0	2	1	1	0	1
ENSMUSG00000033257	TTLL4	14	18	15	19	27	22	96	105	75	108	72	83
ENSMUSG00000033272	SLC35A4	5	3	11	3	5	3	35	34	20	30	23	30
ENSMUSG00000033276	STK36	1	1	1	0	0	1	3	3	2	3	1	3
ENSMUSG00000033282	RPGRIP1L	1	0	1	3	2	1	8	4	6	7	4	5
ENSMUSG00000033285	WDR3	19	23	22	17	22	22	167	174	127	166	130	121
ENSMUSG00000033294	NOC4L	4	6	7	9	7	6	51	56	34	48	44	34
ENSMUSG00000033306	LPP	2	3	2	4	4	3	9	20	8	14	10	9
ENSMUSG00000033307	MIF	37	56	54	62	49	47	414	426	282	420	279	355
ENSMUSG00000033308	DPYD	0	0	0	0	0	0	1	0	0	0	0	0
ENSMUSG00000033313	FBXL8	0	2	1	1	1	0	3	3	7	5	5	2
ENSMUSG00000033318	GSTT2	16	35	28	30	38	25	151	129	116	167	100	150

ENSMUSG00000033319	FEM1C	3	3	4	3	4	1	19	22	15	15	20	11
ENSMUSG00000033323	CTDP1	2	4	3	2	2	6	14	11	12	13	9	9
ENSMUSG00000033326	KDM4A	6	7	5	8	5	6	38	55	35	40	21	33
ENSMUSG00000033335	DNM2	3	14	12	19	22	8	55	58	38	62	60	63
ENSMUSG00000033350	CHST2	0	0	0	0	0	1	1	0	0	1	0	0
ENSMUSG00000033352	MAP2K4	4	6	5	6	5	5	32	39	27	39	19	32
ENSMUSG00000033356	PUS7L	0	1	2	5	2	1	10	8	12	11	4	12
ENSMUSG00000033364	USP37	11	19	18	25	16	21	70	94	44	88	57	54
ENSMUSG00000033365	IPO13	7	4	5	5	6	6	18	27	14	31	23	32
ENSMUSG00000033368	TRIM69	0	0	0	0	0	1	0	0	0	0	1	0
ENSMUSG00000033373	FNTB	1	1	2	1	1	2	6	13	8	13	6	5
ENSMUSG00000033379	ATP6V0B	12	21	19	17	22	18	79	90	79	123	74	92
ENSMUSG00000033382	TRAPPC8	17	6	17	17	11	9	58	62	30	68	42	52
ENSMUSG00000033386	FRRS1	10	13	7	30	26	15	61	86	55	85	54	60
ENSMUSG00000033389	ARHGAP44	0	0	0	1	0	0	0	0	0	0	0	1
ENSMUSG00000033392	CLASP2	32	51	49	64	40	37	259	262	195	289	175	223
ENSMUSG00000033396	SPG11	0	2	2	5	2	1	9	9	11	6	6	8
ENSMUSG00000033400	AGL	0	1	2	3	2	2	14	12	7	15	4	8
ENSMUSG00000033405	NUDT15	0	1	0	0	0	0	1	0	0	0	0	0
ENSMUSG00000033409	SYCE1L	0	0	0	0	0	0	1	0	0	1	0	0
ENSMUSG00000033411	CTDSPL2	9	20	12	21	15	12	61	58	50	77	37	44
ENSMUSG00000033416	GUCD1	2	2	0	0	1	2	7	3	1	7	4	5
ENSMUSG00000033417	CACUL1	4	11	2	7	5	2	37	38	19	40	26	23
ENSMUSG00000033419	SNAP91	0	0	0	1	0	0	0	0	0	0	0	0
ENSMUSG00000033420	ANTXR1	1	1	0	0	0	1	0	0	0	1	0	0
ENSMUSG00000033423	ERI3	6	6	3	4	6	2	25	40	19	29	26	32
ENSMUSG00000033429	MCEE	3	6	5	0	7	6	30	46	25	30	28	31
ENSMUSG00000033430	TERF2IP	6	4	1	5	3	4	28	20	15	15	15	22
ENSMUSG00000033434	GTPBP6	2	5	1	4	2	6	11	9	10	11	14	6
ENSMUSG00000033439	TRMT13	6	7	6	8	9	9	61	52	39	70	43	35
ENSMUSG00000033444	SPECC1L	2	3	5	3	6	2	21	25	17	18	12	11
ENSMUSG00000033446	LPAR6	7	9	3	7	5	7	16	19	16	27	13	18
ENSMUSG00000033450	TAGAP	0	1	0	0	0	0	1	2	1	1	0	1
ENSMUSG00000033454	ZBTB1	7	8	11	15	13	15	50	53	35	65	38	43
ENSMUSG00000033458	FAN1	0	1	1	0	0	0	1	1	1	0	0	0
ENSMUSG00000033475	TOMM6	1	0	1	0	0	1	1	3	2	4	4	0
ENSMUSG00000033478	FAM160B1	9	2	1	10	4	2	21	26	22	24	15	20
ENSMUSG00000033486	CATSPER2	0	0	1	1	3	1	5	3	0	4	0	3
ENSMUSG00000033487	FNDC3A	5	2	6	5	9	6	29	21	19	26	29	15
ENSMUSG00000033498	STRC	4	3	5	6	8	6	36	52	31	29	28	32
ENSMUSG00000033499	LARP4B	43	61	63	64	51	59	153	182	104	172	121	129
ENSMUSG00000033502	CDC14A	8	11	6	12	11	9	70	69	50	67	40	37
ENSMUSG00000033526	PPIP5K1	6	6	8	11	6	6	25	28	22	27	23	20
ENSMUSG00000033530	TTC7B	5	7	10	8	8	9	37	39	22	41	26	32
ENSMUSG00000033538	CASP4	1	0	0	0	0	0	0	2	1	2	0	0
ENSMUSG00000033540	IDUA	0	1	0	0	0	1	4	6	5	3	2	3
ENSMUSG00000033543	GTF2A2	12	14	17	22	18	21	112	124	71	107	73	84
ENSMUSG00000033544	ANGPTL1	1	1	0	0	0	0	0	0	0	0	0	0
ENSMUSG00000033545	ZNRF1	24	24	34	44	24	34	136	142	140	161	126	151
ENSMUSG00000033554	DPH5	5	9	8	6	1	5	48	39	24	44	30	30
ENSMUSG00000033557	FAM20B	2	2	1	2	5	1	16	17	12	17	17	22
ENSMUSG00000033565	RBFOX2	0	0	0	0	0	0	1	0	0	0	0	0
ENSMUSG00000033569	ADGRB3	0	0	0	1	0	0	0	0	0	0	0	0
ENSMUSG00000033577	MYO6	1	2	2	5	4	2	5	8	8	19	5	11
ENSMUSG00000033581	IGF2BP2	0	1	1	0	1	0	7	6	2	9	6	2
ENSMUSG00000033589	REEP4	5	4	2	3	0	0	3	9	5	11	6	8
ENSMUSG00000033594	SPATA2L	1	0	0	0	1	0	3	3	1	2	0	3
ENSMUSG00000033596	RFWD3	5	1	2	5	5	3	24	27	28	23	11	18

ENSMUSG00000033597	CASKIN1	0	0	0	0	0	0	1	0	3	0	0	0
ENSMUSG00000033610	PANK1	2	3	2	1	0	0	15	12	8	10	5	6
ENSMUSG00000033615	CPLX1	0	1	0	0	0	0	0	0	0	0	0	1
ENSMUSG00000033618	MAP3K13	1	3	0	0	1	2	3	2	2	3	5	1
ENSMUSG00000033623	PCGF3	6	5	2	2	7	5	22	20	26	33	21	25
ENSMUSG00000033624	PDPR	2	1	1	1	0	0	2	2	2	4	7	2
ENSMUSG00000033628	PIK3C3	1	3	8	3	3	2	29	27	23	34	22	32
ENSMUSG00000033629	HACD3	6	9	10	6	5	5	31	36	22	27	34	43
ENSMUSG00000033632	AW554918	1	2	1	3	3	5	14	8	10	12	7	6
ENSMUSG00000033634	NAT8F2	0	0	0	0	0	0	0	1	1	0	0	0
ENSMUSG00000033644	PIWIL2	1	0	0	0	0	0	1	0	0	2	2	1
ENSMUSG00000033653	VPS8	0	1	0	0	0	0	3	11	4	4	3	5
ENSMUSG00000033658	DDX19B	1	6	1	7	1	3	23	23	19	27	13	23
ENSMUSG00000033669	ZFP7	1	1	1	3	2	1	5	9	9	3	5	9
ENSMUSG00000033671	CEP350	1	1	4	3	2	5	27	27	17	23	12	10
ENSMUSG00000033684	QSOX1	0	1	0	0	1	0	0	1	1	0	0	0
ENSMUSG00000033685	UCP2	16	9	17	18	18	16	95	82	71	101	73	82
ENSMUSG00000033688	1300017J02R	0	0	0	0	0	0	0	1	0	0	0	0
ENSMUSG00000033697	ARHGAP39	0	2	1	2	0	0	2	10	3	15	11	4
ENSMUSG00000033701	ACBD6	5	8	8	11	8	10	45	53	30	54	28	43
ENSMUSG00000033703	FUK	1	2	1	0	3	1	2	5	3	3	4	6
ENSMUSG00000033705	STARD9	2	4	1	3	1	5	16	13	19	15	9	12
ENSMUSG00000033706	SMYD5	5	2	3	7	3	1	20	26	20	14	16	13
ENSMUSG00000033712	CCAR2	1	3	1	4	1	2	14	18	14	10	11	11
ENSMUSG00000033713	FOXN3	36	46	40	51	44	53	136	127	90	113	101	137
ENSMUSG00000033720	SFXN5	0	2	1	3	1	3	25	25	8	18	8	21
ENSMUSG00000033721	VAV3	0	0	0	0	0	0	0	0	0	2	0	0
ENSMUSG00000033728	LRRC14	2	3	2	2	0	1	12	6	11	8	4	10
ENSMUSG00000033730	EGR3	2	0	0	0	1	0	1	4	2	2	3	3
ENSMUSG00000033732	SF3B3	18	23	20	22	23	14	84	84	79	81	69	67
ENSMUSG00000033735	SPR	5	3	3	5	3	6	24	26	18	24	17	22
ENSMUSG00000033737	FNDC3C1	0	0	0	0	0	0	0	0	1	0	0	0
ENSMUSG00000033739	FKBPL	1	1	0	0	0	1	5	1	0	3	4	1
ENSMUSG00000033740	ST18	0	0	0	0	1	0	0	0	0	0	0	0
ENSMUSG00000033751	GADD45GIP1	19	20	20	17	18	31	143	124	95	138	121	127
ENSMUSG00000033752	MND1	5	4	2	10	5	5	22	22	14	14	19	14
ENSMUSG00000033760	RBM4B	11	12	13	9	9	5	38	24	18	20	23	20
ENSMUSG00000033762	RECQL4	4	7	1	6	12	11	34	41	25	48	34	28
ENSMUSG00000033763	MTSS1L	0	0	0	1	0	0	2	0	0	1	0	0
ENSMUSG00000033767	TMEM131L	3	5	6	6	8	9	19	24	11	30	11	18
ENSMUSG00000033768	NRXN2	0	0	0	0	0	0	0	1	0	0	0	1
ENSMUSG00000033769	EXOC6B	0	2	0	2	0	2	7	13	7	6	1	4
ENSMUSG00000033773	RPAP2	1	4	2	4	5	1	7	18	12	13	12	13
ENSMUSG00000033781	ASB13	0	0	3	1	1	1	13	5	2	4	10	6
ENSMUSG00000033790	TUBGCP5	5	2	3	5	3	6	33	39	36	38	28	25
ENSMUSG00000033792	ATP7A	0	1	1	2	1	1	0	3	1	4	2	3
ENSMUSG00000033793	ATP6V1H	8	6	10	11	10	11	45	58	53	46	47	47
ENSMUSG00000033799	FAM208B	8	8	15	12	9	10	65	70	46	60	43	49
ENSMUSG00000033805	EPHX4	0	0	0	0	0	0	2	0	0	1	0	3
ENSMUSG00000033808	TMEM87A	41	66	55	65	73	61	222	249	185	218	181	197
ENSMUSG00000033809	ALG3	2	5	0	2	0	2	8	9	11	7	3	8
ENSMUSG00000033813	TCEA1	6	7	3	0	7	6	30	31	19	30	31	24
ENSMUSG00000033819	PPP1R16A	2	3	4	2	1	1	9	11	10	14	5	8
ENSMUSG00000033826	DNAH8	0	1	1	1	2	0	1	0	0	1	0	3
ENSMUSG00000033845	MRPL15	8	15	7	12	7	6	66	78	53	70	49	59
ENSMUSG00000033847	PLA2G4C	1	4	3	2	0	0	6	10	3	12	8	6
ENSMUSG00000033849	B3GALT2	0	0	0	0	0	0	1	0	1	1	0	0
ENSMUSG00000033862	CDK10	3	8	2	7	4	3	11	22	11	21	16	20

ENSMUSG00000033863	KLF9	0	0	0	0	0	0	0	0	0	0	2	0
ENSMUSG00000033871	PPARGC1B	3	6	6	7	6	7	19	19	11	13	15	17
ENSMUSG00000033872	BEST4-PS	0	0	0	0	0	0	0	1	0	0	0	0
ENSMUSG00000033880	LGALS3BP	0	0	0	0	0	0	1	1	0	0	0	1
ENSMUSG00000033883	D3ERTD254E	6	5	5	7	9	4	11	19	22	30	15	24
ENSMUSG00000033885	PXK	1	2	1	0	3	2	10	11	9	11	6	11
ENSMUSG00000033902	MAPKBP1	0	1	3	0	1	0	1	1	1	3	1	0
ENSMUSG00000033904	CCP110	8	7	3	5	4	10	33	30	24	50	21	23
ENSMUSG00000033906	ZDHHC15	1	1	2	2	2	1	2	9	2	9	1	5
ENSMUSG00000033909	USP36	18	15	23	22	29	21	79	111	73	120	66	86
ENSMUSG00000033910	GUCY1A1	0	3	2	0	1	1	2	1	0	2	1	1
ENSMUSG00000033916	CHMP2A	10	20	14	20	12	12	84	81	51	79	48	68
ENSMUSG00000033917	GDE1	3	6	5	4	7	2	36	38	26	35	31	32
ENSMUSG00000033918	PARL	3	2	3	6	5	1	29	23	19	26	24	18
ENSMUSG00000033931	RBM34	4	15	11	13	5	15	59	45	32	61	31	44
ENSMUSG00000033933	VHL	0	1	3	0	0	2	15	16	10	5	8	8
ENSMUSG00000033938	NDUFB7	21	46	22	31	49	34	231	231	144	202	157	186
ENSMUSG00000033940	BRK1	3	9	13	23	10	6	84	64	64	77	54	71
ENSMUSG00000033943	MGA	19	12	15	19	24	14	77	81	53	97	53	80
ENSMUSG00000033948	ZSWIM5	0	0	0	0	0	0	0	0	0	0	1	0	0
ENSMUSG00000033949	TRIM36	3	1	1	0	0	2	2	4	3	2	3	3
ENSMUSG00000033952	ASPM	6	10	11	14	9	15	46	53	36	69	30	35
ENSMUSG00000033953	PPP3R1	5	3	3	2	10	5	14	16	15	18	4	14
ENSMUSG00000033955	TNKS1BP1	0	3	0	0	1	1	6	5	5	5	6	1
ENSMUSG00000033961	ZFP446	0	0	1	1	0	1	6	5	3	4	6	4
ENSMUSG00000033963	FANCD2OS	1	0	1	0	0	0	3	8	4	4	2	0
ENSMUSG00000033964	ZBTB41	2	1	1	1	3	1	10	6	4	14	9	8
ENSMUSG00000033965	SLC16A2	0	1	0	0	0	0	0	0	0	0	0	0
ENSMUSG00000033966	CDKL4	0	0	0	0	0	0	1	1	0	1	0	1
ENSMUSG00000033967	RNF225	0	2	0	1	0	1	4	2	5	3	4	5
ENSMUSG00000033970	RFC3	14	14	13	18	12	13	92	90	69	90	82	81
ENSMUSG00000033972	ZFP944	7	2	6	3	0	8	29	22	24	32	26	21
ENSMUSG00000033983	COIL	1	2	1	3	3	6	17	28	11	17	19	11
ENSMUSG00000033985	TESK2	3	1	5	4	5	5	23	18	21	23	11	29
ENSMUSG00000033987	DNAH17	0	1	0	0	0	0	1	0	1	0	0	0
ENSMUSG00000033991	TTC37	0	10	6	17	10	8	28	30	31	43	18	25
ENSMUSG00000034006	PQLC1	0	0	0	2	2	0	3	5	1	1	3	3
ENSMUSG00000034007	SCAPER	1	6	2	8	6	4	14	17	19	26	14	11
ENSMUSG00000034021	PDS5B	8	5	4	7	9	12	51	50	36	47	32	37
ENSMUSG00000034022	CPSF1	4	6	14	15	15	10	57	59	40	46	45	50
ENSMUSG00000034023	FANCD2	7	5	9	3	8	8	33	28	25	35	24	32
ENSMUSG00000034024	CCT2	55	59	50	81	72	58	359	406	295	363	316	328
ENSMUSG00000034028	CD226	7	6	8	9	7	5	36	24	21	28	26	19
ENSMUSG00000034032	RPAP1	0	7	5	9	3	10	32	38	18	27	29	25
ENSMUSG00000034035	CCDC17	0	0	0	0	0	0	0	0	0	0	1	0
ENSMUSG00000034037	FGD5	2	1	2	3	2	2	3	1	1	2	0	0
ENSMUSG00000034041	LYL1	0	1	3	0	4	0	7	6	6	5	2	9
ENSMUSG00000034042	GPBP1L1	36	53	71	60	74	56	245	232	181	272	211	209
ENSMUSG00000034057	MYRFL	0	0	0	0	0	0	0	0	0	1	0	0
ENSMUSG00000034059	YPEL4	1	0	0	0	0	0	0	1	0	0	0	0
ENSMUSG00000034063	4930590J08R	0	1	1	0	0	0	2	1	2	2	0	0
ENSMUSG00000034064	POGLUT1	2	2	5	7	7	10	31	42	26	37	20	28
ENSMUSG00000034066	FARP2	36	33	27	23	43	28	61	76	62	78	60	65
ENSMUSG00000034075	ZDHHC5	7	10	9	8	6	5	50	40	49	53	34	46
ENSMUSG00000034083	CCDC174	7	13	19	21	15	14	69	77	68	87	63	63
ENSMUSG00000034088	HDLBP	14	28	24	30	22	20	122	165	98	149	123	133
ENSMUSG00000034101	CTNND1	0	0	0	0	0	0	0	0	0	0	1	0
ENSMUSG00000034105	TLDC1	0	0	0	0	0	1	2	2	2	4	3	4

ENSMUSG00000034108	CCS	5	5	13	4	15	5	36	42	34	39	38	31
ENSMUSG00000034109	GOLIM4	12	14	12	25	16	22	54	54	50	77	37	59
ENSMUSG00000034110	KCTD7	2	1	1	1	3	2	7	9	4	9	4	12
ENSMUSG00000034111	TMED8	0	1	1	3	5	5	12	9	8	10	7	5
ENSMUSG00000034115	SCN11A	0	0	0	0	0	0	3	1	0	0	0	0
ENSMUSG00000034116	VAV1	13	7	9	18	8	6	45	51	41	55	26	37
ENSMUSG00000034117	PTGDR2	0	0	1	0	0	0	1	0	1	1	0	1
ENSMUSG00000034118	TPST1	1	2	1	5	5	0	14	15	17	13	16	12
ENSMUSG00000034120	SRSF2	31	53	39	39	56	42	355	374	304	395	282	332
ENSMUSG00000034121	MKS1	0	0	0	0	0	0	2	2	2	5	4	4	2
ENSMUSG00000034126	POMT2	0	0	2	3	0	1	3	9	7	8	5	6
ENSMUSG00000034135	SIK3	4	1	1	2	6	1	18	22	16	19	17	8
ENSMUSG00000034139	SERPINI2	0	0	0	0	0	0	1	0	1	0	0	0
ENSMUSG00000034152	EXOC3	0	4	6	7	3	4	16	26	13	16	15	17
ENSMUSG00000034154	INO80	10	9	8	20	13	17	52	44	37	55	44	38
ENSMUSG00000034156	TSPOAP1	1	7	2	4	5	3	8	8	6	7	7	10
ENSMUSG00000034157	CIPC	5	4	6	3	6	5	26	26	18	26	18	20
ENSMUSG00000034158	LRRC58	11	15	11	17	10	14	60	45	45	91	34	52
ENSMUSG00000034160	OGT	10	23	22	20	22	12	94	109	66	83	80	80
ENSMUSG00000034163	ZFC3H1	10	10	10	11	16	16	65	61	34	92	33	40
ENSMUSG00000034164	EMID1	10	5	7	11	5	10	26	35	31	35	33	24
ENSMUSG00000034165	CCND3	13	16	12	26	31	21	135	125	124	139	92	127
ENSMUSG00000034168	IRF2BPL	23	31	28	38	28	45	132	127	92	124	74	102
ENSMUSG00000034171	FAAH	17	27	14	24	21	21	82	95	69	100	78	100
ENSMUSG00000034173	ZBED5	1	1	1	0	1	0	3	2	3	0	3	3
ENSMUSG00000034175	RHBDD3	1	0	3	0	1	1	6	3	8	6	3	7
ENSMUSG00000034177	RNF43	5	3	7	6	3	6	29	37	30	30	23	29
ENSMUSG00000034187	NSF	4	2	4	3	7	9	21	22	17	29	21	20
ENSMUSG00000034189	HSDL1	4	6	9	6	11	2	35	22	30	35	30	26
ENSMUSG00000034190	CHMP7	2	2	3	4	3	0	7	8	4	13	14	13
ENSMUSG00000034192	LSM3	0	6	2	4	6	5	29	35	14	28	27	23
ENSMUSG00000034194	R3HCC1	2	1	3	4	3	0	13	17	6	12	8	11
ENSMUSG00000034201	GAS2L1	3	1	1	1	3	2	6	2	7	7	5	9
ENSMUSG00000034203	CHCHD4	7	11	11	19	12	9	81	95	45	80	70	77
ENSMUSG00000034205	LOXL2	0	0	0	0	0	0	1	0	0	1	0	0
ENSMUSG00000034206	POLQ	4	5	5	11	5	10	40	36	31	52	28	29
ENSMUSG00000034209	RASL10A	0	0	0	0	0	0	0	0	1	0	0	0
ENSMUSG00000034210	EFCAB14	18	17	15	17	19	7	43	50	44	59	38	42
ENSMUSG00000034211	MRPS17	11	16	9	15	7	12	68	60	35	74	49	51
ENSMUSG00000034216	VPS18	3	4	3	3	3	6	26	23	15	20	19	27
ENSMUSG00000034218	ATM	6	6	6	7	3	7	26	25	21	18	17	13
ENSMUSG00000034219	41883	1	0	0	0	0	0	0	0	0	0	0	0
ENSMUSG00000034226	RHOV	0	0	0	0	0	0	0	0	0	0	1	0
ENSMUSG00000034227	FOXJ1	0	0	1	0	0	0	0	0	0	1	0	0
ENSMUSG00000034235	USP54	2	0	3	3	3	1	10	8	6	9	7	10
ENSMUSG00000034243	GOLGB1	17	29	24	43	25	41	103	104	70	144	92	85
ENSMUSG00000034247	PLEKHM1	3	1	1	2	1	0	7	12	7	8	7	5
ENSMUSG00000034248	SLC25A37	2	10	12	14	9	11	41	35	23	60	44	32
ENSMUSG00000034252	SENP6	20	33	29	39	30	37	189	185	138	217	156	126
ENSMUSG00000034254	AGPAT1	29	29	23	35	33	28	149	151	113	151	103	108
ENSMUSG00000034255	ARHGAP27	1	1	0	0	0	0	1	2	0	1	1	1
ENSMUSG00000034259	EXOSC4	3	2	4	4	4	4	42	48	38	40	25	35
ENSMUSG00000034263	INTS14	4	3	6	1	3	2	18	22	21	20	12	19
ENSMUSG00000034266	BATF	8	5	3	5	14	10	20	29	29	33	22	18
ENSMUSG00000034269	SETD5	43	39	55	61	42	39	223	274	198	222	160	192
ENSMUSG00000034274	THOC5	1	2	6	3	3	4	12	21	11	18	14	16
ENSMUSG00000034278	DNAJC17	8	8	7	8	6	12	34	21	24	34	28	35
ENSMUSG00000034282	EVPL	0	0	0	0	0	0	0	0	0	2	0	0

ENSMUSG00000034285	NIPSNAP1	3	5	4	4	3	3	24	26	18	23	15	20
ENSMUSG00000034290	NEK9	3	7	2	6	5	8	29	24	22	22	33	30
ENSMUSG00000034292	TRAF3IP1	2	2	3	1	0	0	4	8	5	5	6	6
ENSMUSG00000034297	MED13	7	8	12	9	7	6	43	47	44	32	45	37
ENSMUSG00000034300	FAM53C	2	2	3	1	2	3	17	11	16	14	17	10
ENSMUSG00000034303	CCDC15	0	1	0	1	0	3	7	8	9	9	3	5
ENSMUSG00000034311	KIF4	7	9	6	6	8	7	49	35	19	43	39	16
ENSMUSG00000034312	IQSEC1	27	36	30	38	35	39	163	192	151	185	111	154
ENSMUSG00000034317	TRIM59	11	6	7	9	8	13	27	38	34	39	29	20
ENSMUSG00000034320	SLC26A2	2	5	3	5	1	0	10	16	14	12	11	12
ENSMUSG00000034321	EXOSC1	15	6	10	12	10	18	64	61	44	99	52	61
ENSMUSG00000034324	TMEM132C	0	0	0	0	1	0	0	0	0	0	0	0
ENSMUSG00000034327	KCTD9	1	0	1	0	1	0	6	11	6	5	3	1
ENSMUSG00000034329	BRIP1	3	1	6	5	3	7	27	24	23	38	19	15
ENSMUSG00000034333	ZBED4	2	1	2	5	5	2	5	5	7	5	2	6
ENSMUSG00000034334	FAM151B	0	0	0	0	0	0	0	1	0	0	1	2
ENSMUSG00000034341	WBP2	1	4	6	3	2	1	25	43	20	28	19	23
ENSMUSG00000034342	CBL	7	16	12	15	6	19	52	72	42	54	70	58
ENSMUSG00000034343	UBE2F	3	5	4	3	5	4	18	11	18	21	20	16
ENSMUSG00000034345	GTF2H5	17	17	30	30	24	34	176	170	151	234	140	198
ENSMUSG00000034349	SMC4	87	141	106	176	138	127	608	571	429	655	519	450
ENSMUSG00000034353	RAMP1	0	0	0	1	0	0	0	0	0	0	0	0
ENSMUSG00000034354	MTMR3	6	5	8	10	5	14	34	47	34	39	32	39
ENSMUSG00000034361	CPNE2	0	1	0	0	1	1	0	2	0	0	0	0
ENSMUSG00000034371	TKFC	1	4	3	2	1	1	8	7	4	6	4	3
ENSMUSG00000034377	TULP4	8	3	6	7	6	2	13	21	19	20	18	19
ENSMUSG00000034379	WDR5B	5	5	6	7	4	4	39	38	32	36	21	25
ENSMUSG00000034384	BARHL2	0	0	1	0	0	0	0	0	0	0	0	0
ENSMUSG00000034390	CMIP	1	6	2	0	4	4	12	17	12	22	10	14
ENSMUSG00000034401	SPATA6	1	0	0	0	0	0	0	0	0	0	0	0
ENSMUSG00000034403	PJA1	0	11	3	6	4	6	43	29	18	47	23	37
ENSMUSG00000034412	TBC1D10A	4	4	2	2	2	2	7	9	6	16	11	10
ENSMUSG00000034416	PKD1L2	0	0	0	0	0	0	0	1	0	0	0	0
ENSMUSG00000034422	PARP14	0	1	0	1	0	0	0	1	0	0	0	0
ENSMUSG00000034424	GCSH	10	10	4	13	11	11	67	47	48	66	41	57
ENSMUSG00000034427	MYO15B	0	0	0	0	0	0	0	1	0	0	0	0
ENSMUSG00000034429	ZFP707	2	2	1	0	3	3	6	7	7	3	6	9
ENSMUSG00000034430	ZXDC	2	3	3	2	4	0	15	11	14	21	8	15
ENSMUSG00000034432	COPS8	3	6	2	9	2	4	33	40	23	28	39	30
ENSMUSG00000034437	GM9761	0	0	0	0	0	0	0	0	0	1	0	1
ENSMUSG00000034438	GBP8	10	14	12	13	19	16	44	61	39	63	44	43
ENSMUSG00000034442	TRMT5	2	1	0	6	1	9	14	12	8	11	6	13
ENSMUSG00000034445	CYB561A3	1	0	0	0	0	0	1	2	1	2	1	0
ENSMUSG00000034449	DHRS11	1	1	2	2	2	0	10	9	5	12	9	8
ENSMUSG00000034453	POLR3B	0	1	3	1	3	3	15	9	13	17	6	9
ENSMUSG00000034456	UROC1	0	0	0	0	0	0	1	0	0	0	0	0
ENSMUSG00000034459	IFIT1	0	0	0	1	0	0	0	0	0	0	0	1
ENSMUSG00000034460	SIX4	0	1	0	0	0	0	0	0	0	1	0	0
ENSMUSG00000034467	DYNLRB2	0	0	0	0	0	0	0	0	0	0	0	1
ENSMUSG00000034471	CASKIN2	2	3	2	1	3	1	1	3	5	2	4	1
ENSMUSG00000034473	SEC22A	1	4	0	2	4	2	25	16	9	13	9	10
ENSMUSG00000034480	DIAPH2	5	4	4	8	2	5	19	21	16	22	14	18
ENSMUSG00000034484	SNX2	10	17	3	15	13	12	46	64	60	77	46	59
ENSMUSG00000034485	UACA	0	0	0	0	0	0	0	1	0	0	0	0
ENSMUSG00000034487	KDELC2	3	5	4	5	9	6	23	19	23	20	20	14
ENSMUSG00000034501	PCNX4	1	2	1	4	2	2	10	15	6	13	6	12
ENSMUSG00000034509	MAD2L1BP	3	4	3	1	2	3	16	18	15	9	11	11
ENSMUSG00000034518	HMGXB4	6	14	10	11	6	7	35	35	38	43	36	34

ENSMUSG00000034522	ZFP395	0	6	3	4	0	5	21	14	12	17	8	14
ENSMUSG00000034525	ICE1	12	8	7	5	7	5	32	33	34	48	40	31
ENSMUSG00000034528	HSD17B13	0	0	0	2	0	0	12	5	3	3	3	4
ENSMUSG00000034538	ZFP418	0	0	0	0	0	0	0	5	2	3	0	0
ENSMUSG00000034543	MORC2A	7	4	6	3	3	4	29	17	18	25	17	25
ENSMUSG00000034544	RSRC1	13	18	12	12	12	14	65	53	38	62	37	41
ENSMUSG00000034560	WASHC4	7	9	7	9	4	14	63	46	41	62	23	39
ENSMUSG00000034563	CCPG1	0	2	1	1	0	2	8	7	10	6	6	3
ENSMUSG00000034566	ATP5H	48	33	40	46	47	37	320	356	264	319	247	273
ENSMUSG00000034570	INPP5J	0	0	0	1	0	0	0	0	0	0	0	0
ENSMUSG00000034573	PTPN13	0	1	0	1	1	1	2	2	1	2	0	1
ENSMUSG00000034574	DAAM1	3	0	2	2	2	2	15	13	7	10	13	5
ENSMUSG00000034575	PAPD7	9	11	9	11	27	20	72	82	50	71	50	50
ENSMUSG00000034579	PLA2G3	0	0	0	0	0	0	0	0	0	0	1	0
ENSMUSG00000034584	EXPH5	0	0	0	1	0	0	0	0	1	2	1	0
ENSMUSG00000034586	HID1	1	0	0	0	0	0	0	1	0	1	0	0
ENSMUSG00000034587	8430429K09F	1	0	2	1	0	0	0	1	2	1	0	1
ENSMUSG00000034591	SLC41A2	0	0	0	1	0	0	0	0	0	0	1	0
ENSMUSG00000034593	MYO5A	8	12	12	11	4	7	55	42	35	71	43	41
ENSMUSG00000034595	PPP1R18	14	9	12	19	11	7	65	55	55	61	42	53
ENSMUSG00000034601	2700049A03F	3	3	3	5	9	3	20	31	15	27	13	13
ENSMUSG00000034602	MON2	4	9	4	8	11	9	39	32	31	50	28	28
ENSMUSG00000034610	ZCCHC11	31	62	43	63	50	65	230	247	169	259	195	184
ENSMUSG00000034612	CHST11	1	1	0	1	2	1	0	2	2	0	3	0
ENSMUSG00000034613	PPM1H	15	10	10	13	18	17	50	63	36	61	46	54
ENSMUSG00000034614	PIK3IP1	1	2	5	5	4	6	21	22	15	27	25	26
ENSMUSG00000034616	SSH3	0	0	0	2	0	0	1	1	0	1	1	0
ENSMUSG00000034617	MTRR	3	3	4	5	8	5	16	23	11	29	24	17
ENSMUSG00000034620	TMEM5	4	11	3	10	13	10	43	59	39	51	31	49
ENSMUSG00000034621	GPATCH8	15	20	15	17	15	18	81	79	45	67	67	56
ENSMUSG00000034636	ZYG11B	1	3	3	4	2	6	21	20	8	22	18	16
ENSMUSG00000034640	TIPARP	8	13	10	13	14	11	58	71	38	67	47	48
ENSMUSG00000034647	ANKRD12	64	72	62	92	59	68	270	244	173	269	230	198
ENSMUSG00000034653	YTHDC2	2	3	3	6	5	3	16	15	14	17	7	9
ENSMUSG00000034656	CACNA1A	4	3	4	3	5	0	19	15	10	9	18	10
ENSMUSG00000034659	TMEM109	4	1	4	5	2	1	23	11	22	21	10	17
ENSMUSG00000034663	BMP2K	6	7	5	5	4	5	33	21	19	33	18	26
ENSMUSG00000034664	ITGA2B	0	0	0	1	1	0	1	1	2	0	1	1
ENSMUSG00000034667	XPOT	9	7	4	7	11	11	63	54	49	62	57	47
ENSMUSG00000034673	PBX2	9	19	5	9	12	13	48	40	40	46	32	55
ENSMUSG00000034674	TDG	2	4	4	4	4	4	0	4	6	7	10	5	11
ENSMUSG00000034681	RNPS1	4	1	1	5	1	5	25	22	16	25	20	13
ENSMUSG00000034686	PRR7	1	3	4	5	0	0	7	10	10	3	8	9
ENSMUSG00000034706	DNAIC2	0	0	0	1	0	0	0	1	0	3	2	0
ENSMUSG00000034707	GNS	1	3	4	7	1	1	14	10	6	10	13	11
ENSMUSG00000034708	GRN	4	4	3	4	7	3	18	19	11	19	13	18
ENSMUSG00000034709	PPP1R21	2	1	2	0	1	1	6	3	5	2	4	2
ENSMUSG00000034714	TTYH2	0	0	1	0	1	0	0	0	1	1	0	0
ENSMUSG00000034723	TMX4	1	1	2	2	2	5	20	17	10	16	10	8
ENSMUSG00000034724	CNOT6L	16	20	18	15	22	15	90	68	52	74	57	52
ENSMUSG00000034729	MRPS10	3	3	0	4	2	3	24	18	17	16	19	20
ENSMUSG00000034731	DGKH	3	4	1	5	2	7	12	11	5	11	13	13
ENSMUSG00000034738	NOSTRIN	0	1	1	1	0	0	0	0	0	1	3	0
ENSMUSG00000034744	NAGK	3	1	0	0	5	1	6	6	5	7	6	8
ENSMUSG00000034748	SIRT6	3	7	9	3	2	3	21	19	10	15	12	20
ENSMUSG00000034757	TMUB2	0	1	1	2	4	1	15	15	11	15	9	11
ENSMUSG00000034758	TLE6	0	0	0	0	2	1	1	1	0	0	0	0
ENSMUSG00000034762	GLIS1	0	0	0	0	1	0	0	0	0	2	0	2

ENSMUSG00000034765	DUSP5	0	0	0	0	1	0	0	0	0	0	0	1
ENSMUSG00000034771	TLE2	0	0	0	0	0	0	1	0	1	0	1	0
ENSMUSG00000034773	BC030867	1	2	0	1	2	3	6	14	8	15	9	9
ENSMUSG00000034781	GNA11	13	18	9	11	13	9	27	31	23	50	28	36
ENSMUSG00000034785	DIO1	0	0	0	0	0	0	0	0	0	1	0	0
ENSMUSG00000034786	GPSM3	7	8	8	8	2	11	40	40	21	46	34	45
ENSMUSG00000034789	RAB24	7	16	21	25	16	21	86	114	92	102	91	77
ENSMUSG00000034792	GNA15	0	0	0	0	0	0	0	0	0	2	0	0
ENSMUSG00000034793	G6PC3	0	2	1	2	1	0	7	9	10	3	8	7
ENSMUSG00000034795	CCDC122	1	0	1	1	1	2	13	6	4	5	3	3
ENSMUSG00000034796	CPNE7	0	1	0	0	0	0	0	1	0	0	0	0
ENSMUSG00000034799	UNC13A	6	6	7	7	6	5	27	29	24	35	24	33
ENSMUSG00000034800	ZFP661	1	2	0	1	0	3	3	2	2	2	0	1
ENSMUSG00000034801	SOS2	4	2	7	11	4	5	32	24	21	36	24	25
ENSMUSG00000034807	COLGALT1	13	9	8	9	15	13	68	73	57	53	44	68
ENSMUSG00000034813	GRIP1	0	0	1	0	0	0	0	0	0	0	1	0
ENSMUSG00000034818	CELF5	0	1	0	0	0	0	1	0	0	0	0	0
ENSMUSG00000034820	CPSF7	7	9	8	11	19	12	58	64	43	86	47	51
ENSMUSG00000034826	NUP54	0	4	5	3	1	8	6	14	10	16	17	13
ENSMUSG00000034829	NXNL1	0	0	0	0	0	0	0	0	0	1	0	0
ENSMUSG00000034832	TET3	11	15	12	17	10	12	53	74	36	67	38	54
ENSMUSG00000034833	TESPA1	8	3	3	8	3	8	23	28	18	25	15	27
ENSMUSG00000034848	TTC21B	6	10	7	11	7	6	39	35	29	39	27	26
ENSMUSG00000034850	TMEM127	5	8	1	7	5	6	25	35	22	32	24	31
ENSMUSG00000034854	MFSD12	1	0	0	1	0	1	3	3	2	5	7	2
ENSMUSG00000034858	FAM214A	2	5	5	2	2	3	24	29	19	24	16	17
ENSMUSG00000034863	ANO8	2	3	5	5	1	5	12	12	10	23	10	17
ENSMUSG00000034867	ANKRD27	7	8	11	9	8	5	25	28	27	34	30	30
ENSMUSG00000034868	MYL12B	5	12	11	11	3	11	54	63	31	60	37	44
ENSMUSG00000034875	NUDT19	4	7	9	7	8	3	38	67	36	44	34	36
ENSMUSG00000034880	MRPL34	2	13	7	8	17	6	77	57	46	66	41	59
ENSMUSG00000034883	LRR1	3	9	4	2	5	5	22	23	22	37	18	27
ENSMUSG00000034889	CACTIN	2	10	13	3	6	3	30	34	23	33	29	32
ENSMUSG00000034892	RPS29	151	173	184	237	237	209	793	796	628	759	619	705
ENSMUSG00000034893	COG3	3	2	1	2	3	3	15	23	7	14	7	7
ENSMUSG00000034898	FILIP1	0	1	3	1	0	2	5	2	1	0	1	5
ENSMUSG00000034902	PIP5K1C	1	3	0	1	4	4	6	10	4	7	4	8
ENSMUSG00000034903	COBLL1	11	21	13	19	19	18	99	93	83	100	71	79
ENSMUSG00000034906	NCAPH	7	3	4	14	4	3	34	34	27	46	32	38
ENSMUSG00000034908	SIDT2	1	4	5	5	6	4	18	18	21	19	13	15
ENSMUSG00000034911	USHBP1	0	0	0	1	0	0	0	1	0	0	0	0
ENSMUSG00000034917	TJP3	0	0	0	0	0	0	0	2	0	0	0	0
ENSMUSG00000034926	DHCR24	28	29	32	17	27	24	108	98	71	119	88	109
ENSMUSG00000034928	RNF44	19	30	26	31	36	25	157	169	134	218	125	137
ENSMUSG00000034930	RTKN	0	0	1	1	0	0	0	0	0	0	0	0
ENSMUSG00000034931	DHX8	0	3	3	1	3	3	20	22	8	21	10	18
ENSMUSG00000034932	MRPL54	3	3	2	4	4	2	22	26	16	19	21	20
ENSMUSG00000034940	SYNRG	6	5	5	4	5	8	23	26	13	20	16	28
ENSMUSG00000034947	TMEM106A	0	1	1	0	2	0	2	2	0	3	2	1
ENSMUSG00000034951	COG7	2	3	4	1	3	2	5	8	10	19	10	15
ENSMUSG00000034958	ATCAY	0	0	0	0	0	0	1	1	0	0	0	0
ENSMUSG00000034959	RUBCNL	0	0	0	0	0	0	0	0	0	1	0	0
ENSMUSG00000034968	LBX2	0	0	0	0	0	0	1	0	0	3	1	1
ENSMUSG00000034973	DOPEY1	6	4	2	4	3	4	16	17	10	28	17	19
ENSMUSG00000034974	DAPK3	2	1	2	4	2	4	21	21	18	14	8	22
ENSMUSG00000034990	OTOA	0	0	1	0	0	0	0	0	0	1	0	1
ENSMUSG00000034993	VAT1	1	1	0	2	4	0	8	10	7	14	4	6
ENSMUSG00000034994	EEF2	177	206	198	224	244	193	1107	1227	897	1082	833	1028

ENSMUSG00000034998	FOXN2	4	6	6	6	15	6	40	38	22	65	32	40
ENSMUSG00000035000	DPP4	0	0	0	0	0	0	1	1	0	1	1	0
ENSMUSG00000035007	RUNDC1	2	1	0	1	1	1	6	3	7	12	7	3
ENSMUSG00000035011	ZBTB7A	41	64	62	64	44	67	187	165	128	223	129	144
ENSMUSG00000035020	EPGN	0	0	0	0	0	0	1	0	0	0	0	0
ENSMUSG00000035021	BAZ1A	13	26	27	35	23	14	72	95	66	97	54	75
ENSMUSG00000035024	NCAPD3	9	17	14	27	13	5	54	76	54	87	42	47
ENSMUSG00000035027	MAP2K2	4	11	8	9	12	13	66	62	50	44	52	66
ENSMUSG00000035033	TBR1	0	0	0	0	1	0	0	1	1	1	0	0
ENSMUSG00000035045	ZC3H12B	1	0	2	1	2	2	5	5	5	5	3	5
ENSMUSG00000035047	KRI1	10	16	14	14	13	23	71	72	66	76	60	70
ENSMUSG00000035048	ANAPC13	5	3	6	8	7	6	46	42	47	38	40	49
ENSMUSG00000035049	RRP12	1	3	1	10	4	6	11	20	8	14	17	13
ENSMUSG00000035051	DHX57	0	3	0	2	0	0	11	9	5	7	10	6
ENSMUSG00000035062	ZC4H2	0	1	1	2	0	3	9	7	6	6	9	5
ENSMUSG00000035064	EEF2K	0	0	1	2	0	0	1	3	0	3	0	1
ENSMUSG00000035067	XKR6	0	1	0	0	1	0	2	0	0	1	0	0
ENSMUSG00000035069	OMA1	0	1	0	1	2	1	3	5	2	4	3	5
ENSMUSG00000035078	MTMR9	6	6	4	9	4	10	22	40	23	36	26	30
ENSMUSG00000035086	BECN1	14	8	14	14	22	17	78	97	55	101	74	93
ENSMUSG00000035093	SECISBP2L	31	27	25	35	31	40	139	108	121	148	93	100
ENSMUSG00000035109	SHC4	1	1	2	1	0	2	4	2	2	4	2	5
ENSMUSG00000035112	WNK4	0	0	1	1	0	0	5	5	3	2	1	1
ENSMUSG00000035125	GCFC2	16	9	8	11	5	14	41	39	27	42	27	25
ENSMUSG00000035126	WDR78	0	4	2	0	2	0	0	5	1	8	0	3
ENSMUSG00000035139	SECISBP2	13	7	8	12	11	10	35	34	35	32	34	42
ENSMUSG00000035142	NUBPL	1	0	2	1	2	1	9	6	5	3	8	8
ENSMUSG00000035150	EIF2S3X	10	15	17	19	19	11	53	73	44	76	55	56
ENSMUSG00000035151	ELMOD2	0	3	0	2	1	1	4	5	3	7	6	5
ENSMUSG00000035152	AP2B1	7	4	4	7	6	6	40	22	14	20	24	24
ENSMUSG00000035158	MITF	0	0	0	0	0	0	0	0	0	0	1	0
ENSMUSG00000035161	INTS6	26	52	52	43	32	37	121	102	85	149	87	71
ENSMUSG00000035168	TANC1	2	3	5	6	6	6	18	25	13	19	12	16
ENSMUSG00000035171	1110059E24F	2	0	4	2	3	2	13	10	5	24	4	6
ENSMUSG00000035172	PLEKHH3	0	2	1	0	3	1	4	3	4	2	4	3
ENSMUSG00000035173	CCDC186	0	0	0	0	0	0	0	0	0	0	0	1
ENSMUSG00000035177	NLRP2	0	0	0	0	0	0	0	1	0	0	1	0
ENSMUSG00000035179	PPP1R32	0	0	1	3	0	1	2	1	1	1	1	0
ENSMUSG00000035181	HEATR5A	4	3	2	4	3	2	24	20	11	19	16	13
ENSMUSG00000035183	SLC24A5	32	37	20	38	33	27	116	108	76	127	86	82
ENSMUSG00000035189	ANO4	0	1	0	1	0	1	5	2	5	3	3	3
ENSMUSG00000035198	TUBG1	4	4	3	5	6	6	16	22	23	21	24	25
ENSMUSG00000035199	ARL6IP5	15	24	18	27	24	20	189	178	119	198	119	179
ENSMUSG00000035200	CHRNB4	0	0	1	0	0	0	1	0	0	1	0	0
ENSMUSG00000035202	LARS2	45	55	40	61	54	59	520	549	418	522	357	527
ENSMUSG00000035203	EPN1	9	10	15	16	19	5	68	78	56	71	43	70
ENSMUSG00000035206	SPPL2B	1	1	0	1	0	0	7	6	2	10	4	6
ENSMUSG00000035208	SLFN8	21	25	19	22	15	27	61	51	38	70	45	45
ENSMUSG00000035212	LEPROT	2	3	4	3	2	4	5	12	4	6	5	3
ENSMUSG00000035215	LSM7	9	8	8	10	8	10	129	139	94	112	114	115
ENSMUSG00000035227	SPCS2	20	34	24	39	45	38	199	215	137	215	166	182
ENSMUSG00000035228	CCDC106	0	1	0	0	0	0	3	1	0	2	2	3
ENSMUSG00000035232	PDK3	0	7	6	2	4	2	19	22	10	12	16	22
ENSMUSG00000035234	ABRAXAS1	3	3	3	7	3	0	14	12	9	22	10	16
ENSMUSG00000035235	TRIM13	0	0	1	0	1	0	1	2	3	3	2	5
ENSMUSG00000035236	SCAI	13	26	16	21	22	26	75	62	63	80	45	54
ENSMUSG00000035237	LCAT	0	0	0	0	1	0	1	0	0	1	0	2
ENSMUSG00000035239	NEU3	1	0	0	0	0	0	3	0	0	1	2	1

ENSMUSG00000035242	OAZ1	4	6	9	4	8	6	43	36	26	40	32	51
ENSMUSG00000035245	EOGT	0	0	3	4	3	4	14	28	17	23	15	18
ENSMUSG00000035246	PCYT1B	1	1	0	0	0	0	1	0	0	2	1	1
ENSMUSG00000035247	HECTD1	17	28	36	41	46	28	143	174	126	169	137	144
ENSMUSG00000035248	ZCCHC6	22	20	28	26	39	35	142	134	97	149	125	113
ENSMUSG00000035262	AMH	0	0	0	0	0	0	0	2	0	1	0	0
ENSMUSG00000035266	HELQ	9	4	10	7	8	10	27	40	24	32	21	23
ENSMUSG00000035268	PKIG	3	2	2	2	2	3	15	12	12	21	16	11
ENSMUSG00000035270	IMPG2	0	0	1	0	0	1	2	2	3	6	4	2
ENSMUSG00000035273	HPSE	0	0	0	0	1	0	0	2	2	5	2	1
ENSMUSG00000035274	TPBG	1	0	0	0	0	0	0	0	0	0	0	0
ENSMUSG00000035275	RAVER2	0	0	0	0	2	0	2	2	1	2	2	0
ENSMUSG00000035278	PLEKHJ1	21	12	22	20	23	25	123	124	112	99	104	106
ENSMUSG00000035279	SSC5D	0	0	0	0	0	0	0	0	0	0	1	0
ENSMUSG00000035284	VPS13C	2	1	1	1	0	3	13	7	5	10	6	6
ENSMUSG00000035285	NAT14	0	0	1	0	0	1	0	0	0	0	0	0
ENSMUSG00000035293	G2E3	6	6	9	12	14	11	55	60	31	52	37	32
ENSMUSG00000035295	WDR38	4	4	5	7	2	0	24	23	18	20	14	22
ENSMUSG00000035297	COPS4	8	5	2	6	10	6	53	64	50	47	29	33
ENSMUSG00000035298	KLHL35	0	0	0	0	1	0	0	0	0	0	0	0
ENSMUSG00000035299	MID1	0	0	0	0	0	0	0	0	0	1	0	0
ENSMUSG00000035310	LIN54	5	1	1	5	6	4	15	20	11	29	18	18
ENSMUSG00000035311	GNPTAB	3	8	7	7	6	13	47	38	39	64	39	28
ENSMUSG00000035325	SEC31A	7	8	5	7	9	12	41	52	36	46	41	44
ENSMUSG00000035329	FBXO33	24	31	22	24	28	25	102	117	78	113	96	80
ENSMUSG00000035337	UCHL4	0	0	2	0	0	1	1	1	3	2	0	0
ENSMUSG00000035342	LZTS2	0	1	0	1	2	0	2	3	0	6	0	5
ENSMUSG00000035351	NUP37	1	3	10	4	4	4	29	15	21	22	16	17
ENSMUSG00000035354	UVRAG	3	4	4	7	3	3	21	19	16	19	16	18
ENSMUSG00000035355	KCNH4	0	0	0	0	0	0	0	0	0	0	0	1
ENSMUSG00000035356	NFKBIZ	1	2	2	1	3	1	15	13	5	18	7	7
ENSMUSG00000035365	PARPBP	1	1	2	5	3	1	15	11	9	7	3	12
ENSMUSG00000035367	RMI1	3	8	11	9	5	0	18	31	19	28	19	25
ENSMUSG00000035372	1810055G02I	0	3	3	3	1	1	5	5	10	4	5	6
ENSMUSG00000035376	HACD2	5	4	2	6	4	5	23	18	16	20	22	24
ENSMUSG00000035378	SHQ1	3	2	3	3	2	3	20	16	14	23	19	16
ENSMUSG00000035382	PCSK7	1	2	4	4	3	1	26	17	13	21	14	22
ENSMUSG00000035383	PMCH	0	0	1	0	0	0	3	0	1	2	0	0
ENSMUSG00000035392	DENND1A	0	3	6	3	4	5	18	24	19	21	19	23
ENSMUSG00000035394	CFAP53	0	0	2	0	0	0	4	1	0	3	2	1
ENSMUSG00000035397	KLF16	4	1	7	1	9	6	27	20	14	27	20	8
ENSMUSG00000035399	OSER1	0	0	0	0	0	0	1	0	1	1	1	2
ENSMUSG00000035401	EMSY	19	39	25	40	29	26	100	89	70	101	67	87
ENSMUSG00000035403	CRB2	0	0	0	0	1	1	0	1	1	1	3	1
ENSMUSG00000035435	ABCA17	0	1	0	0	0	0	0	0	0	0	0	0
ENSMUSG00000035437	RABGAP1	6	11	18	16	11	17	69	69	61	86	57	68
ENSMUSG00000035439	HAUS8	0	1	2	4	2	2	17	15	10	20	13	10
ENSMUSG00000035443	THYN1	13	17	14	12	12	9	79	86	57	84	62	68
ENSMUSG00000035448	CCR3	0	0	0	0	0	0	0	0	0	1	0	0
ENSMUSG00000035455	FIGNL1	12	14	4	15	10	8	67	76	52	56	46	59
ENSMUSG00000035456	PRDM8	0	0	1	0	0	0	0	1	0	0	1	0
ENSMUSG00000035459	STAB2	0	0	0	0	0	0	0	1	0	0	0	0
ENSMUSG00000035469	RCBTB1	5	4	2	3	5	3	22	20	12	39	22	20
ENSMUSG00000035476	TAB3	3	5	3	2	3	4	18	11	5	12	11	7
ENSMUSG00000035478	MBD3	7	9	9	15	12	2	40	40	43	40	31	54
ENSMUSG00000035491	FTHL17A	0	0	0	0	0	0	0	1	0	0	0	0
ENSMUSG00000035495	TSTD2	2	3	6	6	6	3	28	26	14	27	14	23
ENSMUSG00000035505	COX18	0	0	2	2	1	1	10	9	8	9	8	16

ENSMUSG00000035506	SLC12A8	0	0	0	0	0	0	0	0	0	0	0	1
ENSMUSG00000035513	NTNG2	0	0	0	0	0	0	1	1	0	0	0	0
ENSMUSG00000035521	GNPTG	17	18	24	19	26	14	82	126	86	109	82	90
ENSMUSG00000035529	PRDM4	0	3	1	3	3	0	14	14	10	9	6	8
ENSMUSG00000035530	EIF1	28	40	23	56	43	34	229	217	164	200	157	179
ENSMUSG00000035539	CCDC180	0	0	0	0	0	0	0	0	1	0	0	0
ENSMUSG00000035545	LENG8	3	10	10	10	9	7	29	32	23	42	22	33
ENSMUSG00000035547	CAPN5	0	1	0	0	0	1	1	6	1	1	1	2
ENSMUSG00000035559	MPV17L2	2	3	5	1	3	0	12	21	11	18	13	13
ENSMUSG00000035569	ANKRD11	80	100	94	132	118	137	406	406	255	502	323	327
ENSMUSG00000035572	DCAF10	1	4	6	4	4	7	12	11	9	18	6	14
ENSMUSG00000035575	UTP6	9	10	6	12	12	12	61	65	46	45	52	51
ENSMUSG00000035576	L3MBTL1	0	0	0	0	0	0	0	1	0	0	0	0
ENSMUSG00000035578	IQCG	1	0	3	4	0	2	8	11	8	13	12	6
ENSMUSG00000035582	GDPD4	0	0	0	0	0	0	0	0	0	0	0	1
ENSMUSG00000035585	TSEN34	0	3	2	2	3	0	11	18	16	15	7	10
ENSMUSG00000035595	1600002K03F	0	1	0	2	2	0	5	5	12	3	4	9
ENSMUSG00000035596	MBOAT7	1	3	1	5	0	1	14	15	9	6	5	17
ENSMUSG00000035597	PRPF39	39	50	58	78	61	53	324	321	256	326	197	270
ENSMUSG00000035601	TRMT10B	0	1	1	2	0	0	5	5	4	4	8	2
ENSMUSG00000035614	TOGARAM1	2	2	9	6	6	4	27	35	17	38	20	19
ENSMUSG00000035615	FRMPD1	0	0	0	0	0	0	1	0	0	0	0	0
ENSMUSG00000035620	RIC8B	2	1	0	3	0	1	11	10	5	18	9	8
ENSMUSG00000035621	MIDN	15	17	26	24	18	31	85	100	91	101	62	82
ENSMUSG00000035623	RSF1	20	30	19	29	21	17	116	129	73	113	81	87
ENSMUSG00000035629	RUBCN	2	1	3	0	0	0	3	4	4	3	2	2
ENSMUSG00000035632	CNOT3	5	6	11	9	11	6	57	42	42	59	26	43
ENSMUSG00000035637	GRHPR	2	0	3	2	3	2	15	12	8	12	6	9
ENSMUSG00000035640	CBARP	2	5	4	4	4	2	13	17	15	16	4	14
ENSMUSG00000035642	AAMDC	7	1	5	4	4	6	26	17	17	21	19	24
ENSMUSG00000035649	ZCCHC7	16	20	14	24	29	19	117	107	87	121	64	72
ENSMUSG00000035666	GTF3C4	2	7	4	8	5	2	34	31	16	29	20	23
ENSMUSG00000035671	ZSWIM4	2	2	1	1	1	1	9	7	11	5	6	10
ENSMUSG00000035673	SBNO2	5	9	6	8	1	0	15	25	18	20	8	19
ENSMUSG00000035674	NDUFA3	34	39	43	29	53	30	224	223	175	221	163	220
ENSMUSG00000035678	TNFSF9	1	3	2	5	2	2	6	7	9	11	7	5
ENSMUSG00000035681	KCNC2	0	1	1	0	2	1	0	0	0	1	1	0
ENSMUSG00000035683	MELK	1	0	3	1	3	1	11	13	5	12	6	7
ENSMUSG00000035686	THRSP	0	0	0	0	0	0	1	0	0	0	0	0
ENSMUSG00000035692	ISG15	1	0	0	0	0	0	0	0	2	0	3	1
ENSMUSG00000035694	CAPS2	0	0	0	0	0	0	0	1	0	0	0	0
ENSMUSG00000035696	RNF38	4	4	5	7	1	5	25	24	12	19	18	20
ENSMUSG00000035697	ARHGAP45	44	40	37	52	57	38	135	209	156	242	151	171
ENSMUSG00000035699	SLC51A	0	0	0	0	0	0	6	1	2	5	2	2
ENSMUSG00000035704	ALG8	4	3	5	6	9	1	29	31	26	34	23	25
ENSMUSG00000035711	DOK3	0	1	0	0	1	1	1	0	0	0	1	2
ENSMUSG00000035713	USP35	1	1	0	0	0	1	6	3	2	2	5	2
ENSMUSG00000035722	ABCA7	1	1	2	4	5	1	7	9	6	9	10	12
ENSMUSG00000035725	PRKX	6	7	6	3	4	5	11	13	6	25	7	14
ENSMUSG00000035726	SUPT16	80	117	119	108	113	103	518	534	375	620	428	439
ENSMUSG00000035745	GRIN3B	1	1	0	1	1	1	3	9	5	4	1	7
ENSMUSG00000035754	WDR18	17	12	8	14	10	8	56	60	50	72	54	39
ENSMUSG00000035757	SELENOO	2	2	3	3	1	4	10	14	5	14	10	6
ENSMUSG00000035759	BBS10	0	1	1	0	0	0	1	2	0	0	0	1
ENSMUSG00000035762	TMEM161B	4	7	6	7	4	5	27	37	27	28	18	34
ENSMUSG00000035764	FBXO45	6	12	7	13	6	11	33	33	23	32	24	30
ENSMUSG00000035765	DYM	3	0	2	2	1	2	7	7	10	8	4	11
ENSMUSG00000035769	XYLB	0	1	0	1	0	0	1	4	2	0	4	2

ENSMUSG00000035770	DYNC1LI2	4	2	2	4	10	3	27	27	28	25	24	27
ENSMUSG00000035772	MRPS2	2	6	8	13	4	2	27	32	22	36	24	23
ENSMUSG00000035776	CD99L2	0	2	1	1	1	1	9	6	3	3	8	11
ENSMUSG00000035778	GGTA1	1	7	2	4	2	5	6	8	8	19	4	10
ENSMUSG00000035781	R3HDM4	12	11	12	19	13	12	63	86	58	71	72	69
ENSMUSG00000035783	ACTA2	0	0	1	0	0	0	1	0	0	0	0	1
ENSMUSG00000035790	CEP19	1	5	2	3	4	2	10	8	7	13	10	12
ENSMUSG00000035798	ZDHHC17	3	2	1	6	6	5	33	29	29	30	26	19
ENSMUSG00000035824	TK2	0	0	2	0	1	0	1	4	1	3	3	2
ENSMUSG00000035828	PIM3	5	9	4	14	8	4	37	35	48	45	39	45
ENSMUSG00000035834	POLR3G	5	5	5	3	5	4	20	17	18	28	15	20
ENSMUSG00000035835	PLPPR3	0	0	0	0	0	0	0	0	0	0	1	1
ENSMUSG00000035840	LYSMD3	3	4	6	7	4	4	34	33	20	33	18	17
ENSMUSG00000035842	DDX11	5	12	4	7	11	0	28	37	22	31	30	35
ENSMUSG00000035845	ALG12	1	1	1	2	2	2	3	3	2	4	1	5
ENSMUSG00000035847	IDS	1	2	1	0	2	0	7	4	3	9	3	8
ENSMUSG00000035851	YTHDC1	26	31	37	42	25	27	121	130	82	182	115	119
ENSMUSG00000035852	MISP	0	0	1	0	0	0	0	0	0	0	0	0
ENSMUSG00000035863	PALM	6	8	10	11	8	6	37	38	36	33	25	38
ENSMUSG00000035868	ZFP983	0	1	0	1	0	2	2	3	5	2	2	2
ENSMUSG00000035873	PAWR	0	0	0	0	0	0	1	0	0	1	0	2
ENSMUSG00000035875	AI182371	0	0	0	0	0	0	1	0	2	0	2	0
ENSMUSG00000035877	ZHX3	0	1	1	7	2	2	4	7	3	7	3	4
ENSMUSG00000035885	COX8A	31	35	40	41	36	48	372	356	293	335	285	344
ENSMUSG00000035890	RNF126	14	17	20	14	19	20	85	83	66	79	64	79
ENSMUSG00000035891	CERK	4	10	12	4	3	8	41	36	34	47	36	25
ENSMUSG00000035898	UBA6	18	7	16	10	12	12	43	47	32	53	28	35
ENSMUSG00000035900	GRAMD4	1	4	2	3	0	0	2	5	2	2	5	3
ENSMUSG00000035901	DENND5A	0	0	1	0	0	0	0	0	0	0	0	0
ENSMUSG00000035919	BBS9	6	6	11	1	2	3	27	26	15	22	18	24
ENSMUSG00000035929	H2-Q4	8	6	3	9	7	7	32	27	21	28	22	27
ENSMUSG00000035933	COG5	4	4	5	7	6	4	31	28	16	33	16	30
ENSMUSG00000035936	ALDH5A1	0	3	2	2	1	2	8	9	8	13	7	5
ENSMUSG00000035941	IBTK	5	6	4	5	8	12	52	39	20	50	24	37
ENSMUSG00000035944	TTC38	0	0	0	0	0	0	1	2	1	0	3	1
ENSMUSG00000035948	ACSS3	0	0	0	0	0	0	0	0	0	0	0	1
ENSMUSG00000035949	FBXW2	10	12	17	11	11	10	60	57	38	67	47	40
ENSMUSG00000035953	TMEM55B	3	5	5	11	9	7	28	36	26	37	25	34
ENSMUSG00000035954	DOCK4	0	0	0	0	0	0	1	0	0	1	0	0
ENSMUSG00000035958	TDP2	12	10	5	17	4	12	65	60	49	71	50	93
ENSMUSG00000035960	APEX1	4	8	6	6	13	11	66	59	46	60	38	49
ENSMUSG00000035967	INTS6L	9	4	6	9	6	8	32	29	25	21	30	21
ENSMUSG00000035983	GM7008	0	0	0	0	0	0	0	0	0	1	0	0
ENSMUSG00000035984	NME5	0	0	0	0	0	0	1	1	1	1	0	0
ENSMUSG00000035992	FNIP1	3	8	9	10	10	11	50	55	40	57	43	38
ENSMUSG00000036002	FAM214B	0	0	0	0	0	0	1	2	3	1	1	4
ENSMUSG00000036006	RIPOR2	0	0	0	2	3	0	6	7	2	7	5	8
ENSMUSG00000036009	METTL25	0	1	0	0	0	1	3	7	5	2	3	4
ENSMUSG00000036019	TMTC2	0	0	0	0	0	1	0	0	0	0	0	0
ENSMUSG00000036022	FAM122B	1	2	3	2	3	4	12	6	5	10	4	11
ENSMUSG00000036023	PARP2	6	7	6	8	11	10	41	42	26	34	29	43
ENSMUSG00000036026	TMEM63B	2	4	5	5	5	10	23	28	20	17	18	23
ENSMUSG00000036030	PRTG	0	0	0	1	0	0	0	0	0	0	0	0
ENSMUSG00000036036	ZFP57	0	0	1	1	2	2	2	1	1	2	1	3
ENSMUSG00000036040	ADAMTSL2	2	0	1	3	4	1	5	6	4	8	9	5
ENSMUSG00000036046	5031439G07I	3	2	0	0	3	2	7	7	6	12	6	7
ENSMUSG00000036052	DNAJB5	0	2	0	1	0	0	0	1	0	1	0	1
ENSMUSG00000036053	FMNL2	0	0	0	0	0	0	0	0	0	0	1	0

ENSMUSG00000036054	SUGP2	2	3	0	1	4	2	9	16	12	19	11	17
ENSMUSG00000036057	PTPN23	6	3	2	5	2	1	11	11	8	8	12	9
ENSMUSG00000036061	SMUG1	1	0	0	1	1	0	5	7	3	8	2	2
ENSMUSG00000036067	SLC2A6	0	1	2	1	2	4	12	9	6	15	10	8
ENSMUSG00000036073	GALT	3	3	1	2	4	1	16	9	15	14	8	11
ENSMUSG00000036078	SIGMAR1	3	3	7	6	3	7	34	35	17	37	30	31
ENSMUSG00000036086	ZRANB3	7	4	2	3	3	4	15	6	11	16	9	14
ENSMUSG00000036087	SLAIN2	6	11	10	19	9	8	66	47	34	62	35	41
ENSMUSG00000036093	ARL5A	3	3	8	6	9	7	24	14	23	23	10	22
ENSMUSG00000036097	SLF2	5	5	6	9	6	6	25	31	15	25	6	13
ENSMUSG00000036098	MYRF	0	0	0	0	0	0	0	0	0	0	1	0
ENSMUSG00000036099	VEZT	5	11	5	6	7	4	27	19	9	22	11	21
ENSMUSG00000036104	RAB3GAP1	2	6	4	12	9	9	33	40	31	28	28	42
ENSMUSG00000036109	MBNL3	1	1	2	2	3	3	6	9	1	10	11	9
ENSMUSG00000036112	METAP2	21	41	32	38	29	30	175	174	141	208	147	174
ENSMUSG00000036114	RPP25L	2	4	8	5	8	3	22	26	22	34	16	35
ENSMUSG00000036117	IL5	0	0	0	0	0	0	0	1	0	0	0	0
ENSMUSG00000036120	RFXANK	2	1	4	2	4	1	6	11	7	10	4	13
ENSMUSG00000036138	ACAA1A	5	5	10	9	7	4	27	24	28	29	23	25
ENSMUSG00000036139	HOXC9	1	4	1	7	6	5	6	10	5	15	7	5
ENSMUSG00000036151	TM6SF2	0	1	0	0	0	0	0	3	0	1	0	2
ENSMUSG00000036155	MGAT5	4	5	5	8	5	8	21	28	25	24	22	29
ENSMUSG00000036158	PRICKLE1	0	4	1	1	0	1	5	7	4	5	4	7
ENSMUSG00000036160	SURF6	6	9	9	7	7	8	59	47	39	62	38	38
ENSMUSG00000036167	PPHLN1	6	3	6	7	11	5	32	34	23	26	28	29
ENSMUSG00000036168	CCDC38	9	7	13	8	7	7	31	28	18	26	22	18
ENSMUSG00000036180	GATAD2A	11	27	14	14	14	21	93	82	79	100	83	69
ENSMUSG00000036181	HIST1H1C	10	8	14	14	12	8	104	95	65	93	83	82
ENSMUSG00000036185	SAPCD1	0	0	0	0	1	1	2	0	1	2	1	0
ENSMUSG00000036186	FAM69B	0	0	0	0	1	0	1	1	0	0	0	1
ENSMUSG00000036188	ANKMY2	0	5	4	2	4	6	15	24	16	33	14	14
ENSMUSG00000036192	RORB	0	0	0	0	0	0	2	0	0	0	1	1
ENSMUSG00000036196	SLC26A8	0	0	1	0	0	0	1	0	1	0	0	0
ENSMUSG00000036197	GXYLT1	7	8	9	14	11	7	38	43	26	34	30	36
ENSMUSG00000036199	NDUFA13	58	84	70	90	74	77	505	571	380	518	410	498
ENSMUSG00000036202	RIF1	41	82	58	86	77	70	398	343	256	415	247	300
ENSMUSG00000036206	SH3BP4	0	0	0	0	0	0	0	1	0	0	0	0
ENSMUSG00000036208	NEPRO	3	4	1	4	2	1	11	12	8	10	7	13
ENSMUSG00000036214	ZNRD1AS	0	1	0	0	0	1	10	6	4	4	2	5
ENSMUSG00000036223	SKA1	7	8	5	4	12	4	27	41	18	25	35	18
ENSMUSG00000036231	AGR3	0	0	0	1	0	0	0	0	0	0	0	0
ENSMUSG00000036241	UBE2R2	35	38	31	29	22	48	135	128	76	192	115	110
ENSMUSG00000036246	GMIP	3	4	6	12	6	7	33	34	40	44	32	45
ENSMUSG00000036249	RBM43	1	1	0	4	2	2	14	20	13	19	15	16
ENSMUSG00000036256	IGFBP7	0	1	1	1	3	3	8	10	12	5	6	6
ENSMUSG00000036257	PNPLA8	3	3	2	2	6	3	16	11	7	15	8	6
ENSMUSG00000036270	EDC4	4	6	10	6	8	6	40	36	26	53	23	41
ENSMUSG00000036275	9530068E07F	13	15	6	16	10	13	65	54	39	46	30	29
ENSMUSG00000036278	MACROD1	0	0	0	0	0	0	1	2	1	1	2	1
ENSMUSG00000036281	SNAPC4	6	8	12	19	12	9	66	60	39	51	49	60
ENSMUSG00000036282	NAA30	5	3	11	7	7	6	39	28	35	52	33	19
ENSMUSG00000036285	NOA1	9	7	10	8	5	2	49	49	28	44	32	36
ENSMUSG00000036291	AP5M1	1	2	8	8	8	4	45	36	30	38	39	23
ENSMUSG00000036292	GRAMD1C	1	3	1	1	0	1	2	4	2	3	3	5
ENSMUSG00000036299	BC031181	6	7	3	17	4	4	19	37	26	42	35	31
ENSMUSG00000036304	ZDHHC23	1	1	0	1	0	0	1	2	0	0	2	3
ENSMUSG00000036305	RPL39-PS	0	0	0	1	0	0	3	1	1	0	2	3
ENSMUSG00000036306	LZTS1	1	0	1	0	0	2	0	2	0	2	0	4

ENSMUSG00000036309	SKP1A	150	198	208	238	221	192	897	1052	784	1021	786	947
ENSMUSG00000036315	ZNRD1	3	7	3	6	7	5	19	22	12	22	12	14
ENSMUSG00000036323	SRP72	23	38	32	28	25	32	191	167	104	207	130	153
ENSMUSG00000036327	QSOX2	5	3	1	3	4	6	11	21	22	25	11	15
ENSMUSG00000036330	SLC18A1	0	0	0	0	0	0	1	1	1	0	0	1
ENSMUSG00000036333	KIDINS220	4	10	10	13	11	4	21	32	14	33	12	23
ENSMUSG00000036334	IGSF10	0	0	0	0	0	0	0	1	0	1	0	1
ENSMUSG00000036339	TMEM260	0	2	0	1	0	1	3	6	6	4	5	3
ENSMUSG00000036352	UBAC1	3	5	10	4	5	2	31	29	31	20	13	27
ENSMUSG00000036368	RMDN2	0	1	0	0	0	1	1	0	0	0	1	0
ENSMUSG00000036371	SERBP1	248	280	255	361	346	300	1478	1428	1104	1428	977	1248
ENSMUSG00000036372	TMEM258	23	17	18	36	27	18	166	191	141	177	130	162
ENSMUSG00000036376	ABT1	0	1	1	3	3	5	8	12	12	15	12	19
ENSMUSG00000036377	C530008M17	0	0	0	0	0	0	1	0	0	0	0	0
ENSMUSG00000036381	P2RY14	2	0	0	2	0	0	1	0	0	3	1	1
ENSMUSG00000036390	GADD45A	17	18	23	27	21	17	74	78	79	64	50	73
ENSMUSG00000036391	SEC24A	5	5	8	10	6	3	45	47	32	39	30	30
ENSMUSG00000036398	PPP1R11	26	27	18	28	26	20	129	123	95	125	76	91
ENSMUSG00000036402	GNG12	5	3	0	1	2	3	9	14	8	10	11	14
ENSMUSG00000036403	CEP135	2	7	0	8	4	7	27	26	9	28	23	14
ENSMUSG00000036411	9530077C05F	0	2	2	0	1	2	3	6	2	3	3	0
ENSMUSG00000036427	GPI1	4	11	10	12	11	13	114	108	76	116	73	67
ENSMUSG00000036430	TBCC	1	6	4	5	8	9	35	26	21	30	22	36
ENSMUSG00000036432	SIAH2	4	10	6	7	6	5	29	30	15	29	19	14
ENSMUSG00000036435	EXOC1	2	4	3	2	5	8	15	23	10	16	19	13
ENSMUSG00000036438	CALM2	14	19	14	16	17	18	101	104	79	142	74	82
ENSMUSG00000036442	THAP11	9	9	5	7	6	10	42	41	30	34	23	33
ENSMUSG00000036450	HIF1AN	2	2	1	4	4	1	11	14	12	19	2	18
ENSMUSG00000036452	ARHGAP26	1	2	4	3	3	2	13	9	15	13	15	13
ENSMUSG00000036461	ELF1	19	23	16	20	17	22	116	122	77	145	84	107
ENSMUSG00000036466	MEGF11	0	0	1	0	0	0	0	0	0	0	1	1
ENSMUSG00000036469	MARCH1	0	0	0	0	0	0	1	0	0	1	0	0
ENSMUSG00000036473	TBC1D24	0	1	3	1	1	2	7	8	8	12	8	6
ENSMUSG00000036478	BTG1	0	0	1	0	1	1	3	2	2	0	3	1
ENSMUSG00000036499	EEA1	20	22	20	28	27	32	106	117	73	144	85	91
ENSMUSG00000036501	FAM13B	4	10	9	7	4	6	40	20	21	43	32	30
ENSMUSG00000036502	TMEM255A	1	5	7	6	8	4	13	17	15	13	9	20
ENSMUSG00000036503	RNF13	2	5	0	5	0	5	26	31	26	30	14	22
ENSMUSG00000036504	PHPT1	8	8	8	6	7	8	30	32	25	34	23	25
ENSMUSG00000036513	COMMD2	2	7	10	2	3	5	27	29	18	28	21	25
ENSMUSG00000036526	CARD11	0	0	1	0	1	0	0	0	0	1	0	0
ENSMUSG00000036529	SBF1	8	7	13	17	15	14	47	56	37	46	40	59
ENSMUSG00000036533	CDC42EP3	0	0	1	2	1	3	9	6	9	8	12	9
ENSMUSG00000036534	SLC38A7	0	3	1	0	1	0	7	3	4	2	2	2
ENSMUSG00000036537	RNF113A1	2	5	2	2	2	4	15	10	12	11	7	9
ENSMUSG00000036550	CNOT1	13	16	9	15	15	21	67	66	59	81	55	50
ENSMUSG00000036551	AKAP14	0	0	0	0	0	0	0	2	0	0	1	1
ENSMUSG00000036552	ERMARD	1	3	3	2	1	2	7	1	11	11	4	10
ENSMUSG00000036553	SH3TC1	0	1	0	0	1	0	0	3	0	1	3	1
ENSMUSG00000036555	IQCE	5	5	6	7	4	7	30	18	19	29	24	25
ENSMUSG00000036561	PPP6R2	9	14	18	12	12	13	33	51	43	36	31	30
ENSMUSG00000036565	TTYH3	2	0	2	1	0	1	6	6	4	7	4	4
ENSMUSG00000036568	BICRAL	6	6	1	2	6	3	25	23	18	38	15	14
ENSMUSG00000036572	UPF3B	22	21	12	21	16	20	77	88	69	91	78	85
ENSMUSG00000036574	TEX44	0	0	0	0	0	0	2	0	1	0	3	2
ENSMUSG00000036580	SPG20	2	6	2	2	6	6	37	25	30	29	19	33
ENSMUSG00000036585	FGF1	0	0	0	0	0	0	1	1	0	0	0	0
ENSMUSG00000036587	FUT7	0	0	0	0	0	0	0	0	0	0	0	1

ENSMUSG00000036591	ARHGAP21	12	20	25	23	22	15	94	105	80	99	69	64
ENSMUSG00000036599	CHST12	4	3	2	1	1	5	12	15	8	13	7	10
ENSMUSG00000036602	ALX1	0	0	0	0	0	0	0	0	0	0	0	1
ENSMUSG00000036606	PLXNB2	0	1	0	1	1	1	0	0	1	1	0	0
ENSMUSG00000036611	EEPD1	0	0	0	0	0	0	1	0	0	0	0	0
ENSMUSG00000036613	EIPR1	2	2	2	2	2	2	14	14	7	15	5	9
ENSMUSG00000036615	RFXAP	10	1	8	6	6	9	36	40	28	45	31	33
ENSMUSG00000036617	ETL4	0	1	0	0	0	0	0	1	0	1	1	1
ENSMUSG00000036620	MGAT4B	2	4	2	3	1	3	20	12	10	17	14	14
ENSMUSG00000036622	ATP13A2	10	13	16	20	12	8	65	67	54	67	54	56
ENSMUSG00000036632	ALG5	5	13	1	7	5	7	55	50	43	62	29	43
ENSMUSG00000036636	CLCN7	7	6	6	21	6	8	23	25	20	35	22	19
ENSMUSG00000036639	NUDT1	3	2	2	0	0	0	13	9	16	14	6	12
ENSMUSG00000036641	CCDC148	0	1	0	2	1	1	6	5	1	3	4	0
ENSMUSG00000036644	TBC1D9B	1	2	4	2	0	3	14	19	14	12	5	16
ENSMUSG00000036646	MAN1B1	6	14	10	6	4	8	33	48	27	44	30	31
ENSMUSG00000036655	COLEC11	0	0	0	1	0	0	1	0	0	1	0	0
ENSMUSG00000036672	CENPT	3	2	2	2	3	2	13	17	15	13	12	20
ENSMUSG00000036678	AAAS	3	14	5	9	9	6	29	31	29	21	19	23
ENSMUSG00000036686	CC2D1A	2	3	4	5	3	3	17	32	22	21	15	14
ENSMUSG00000036687	TMEM184A	1	0	2	0	1	0	1	3	2	3	1	1
ENSMUSG00000036693	NOP14	55	46	45	50	50	51	225	266	189	269	201	231
ENSMUSG00000036698	AGO2	8	12	5	7	9	10	39	50	38	45	34	31
ENSMUSG00000036707	CAB39	24	25	20	34	23	29	135	142	90	128	83	109
ENSMUSG00000036712	CYLD	6	9	1	7	8	4	23	27	28	28	28	25
ENSMUSG00000036721	ZSCAN12	1	0	0	0	0	1	0	3	0	3	1	4
ENSMUSG00000036733	RBM42	0	0	0	0	0	1	1	0	0	0	0	0
ENSMUSG00000036737	OXSR1	6	14	6	9	14	8	41	52	33	39	34	38
ENSMUSG00000036748	CUEDC2	1	3	5	4	7	10	16	25	20	21	23	29
ENSMUSG00000036751	COX6B1	47	42	38	45	68	46	372	416	271	444	306	361
ENSMUSG00000036752	TUBB4B	15	21	27	26	19	21	141	161	111	120	121	117
ENSMUSG00000036760	KCNK9	0	0	0	0	0	0	0	1	0	0	0	0
ENSMUSG00000036764	DNAJC12	0	1	0	0	0	2	6	4	5	7	7	1
ENSMUSG00000036766	DNER	0	0	0	1	0	0	0	0	0	0	0	0
ENSMUSG00000036768	KIF15	34	38	41	46	31	47	224	243	188	270	177	161
ENSMUSG00000036769	WDR44	2	3	1	3	4	7	23	16	18	38	17	8
ENSMUSG00000036775	DECR2	4	2	0	2	3	0	7	18	6	11	7	11
ENSMUSG00000036777	ANLN	3	4	9	10	9	6	24	40	18	38	24	26
ENSMUSG00000036779	PAPD5	3	6	6	13	11	9	47	47	23	41	23	29
ENSMUSG00000036781	RPS27L	16	29	14	38	28	28	229	239	155	218	161	192
ENSMUSG00000036792	MBD5	7	3	4	4	2	2	21	10	8	24	17	14
ENSMUSG00000036800	FAM135B	0	0	0	0	0	0	1	0	0	0	0	0
ENSMUSG00000036810	CNEP1R1	3	3	9	7	4	6	43	51	26	50	28	32
ENSMUSG00000036814	SLC6A20A	0	0	0	0	0	0	0	0	1	0	1	1
ENSMUSG00000036815	DPP10	0	1	0	1	0	0	0	0	2	0	0	0
ENSMUSG00000036817	SUN1	2	4	8	9	8	7	38	53	37	33	36	42
ENSMUSG00000036819	JMJD4	1	4	5	3	4	3	19	20	11	25	7	14
ENSMUSG00000036820	AMDHD2	0	0	0	1	0	1	1	2	4	7	7	2
ENSMUSG00000036822	TOPORS	19	19	14	23	22	21	113	104	82	125	73	79
ENSMUSG00000036825	SSX2IP	3	4	2	0	7	8	12	9	9	9	7	14
ENSMUSG00000036833	PNPLA7	2	4	7	6	2	4	21	38	15	14	22	17
ENSMUSG00000036834	PLCH1	2	0	2	2	1	1	11	7	3	9	12	11
ENSMUSG00000036835	PSENEN	1	2	1	3	2	0	24	13	9	16	11	18
ENSMUSG00000036840	SIAH1A	5	2	4	5	4	2	22	11	13	28	16	16
ENSMUSG00000036845	LIN37	0	2	0	3	3	3	14	24	10	17	9	16
ENSMUSG00000036850	MRPL41	8	9	7	7	7	5	44	69	42	49	47	50
ENSMUSG00000036854	HSPB6	0	0	0	1	0	0	0	0	1	0	0	1
ENSMUSG00000036856	WNT4	0	1	0	0	0	1	0	0	0	1	0	0

ENSMUSG00000036858	PTCRA	0	0	0	0	0	0	0	0	0	0	0	1
ENSMUSG00000036860	MRPL55	4	5	4	9	4	3	35	24	19	28	27	30
ENSMUSG00000036864	PROSER3	1	1	2	3	4	1	7	6	8	5	2	5
ENSMUSG00000036867	SMAD6	0	0	0	0	0	1	1	0	3	0	1	0
ENSMUSG00000036873	2410004B18F	9	8	10	8	11	9	39	45	40	62	37	37
ENSMUSG00000036875	DNA2	4	5	8	4	8	5	29	31	15	21	13	20
ENSMUSG00000036879	PHKB	0	6	7	5	3	3	17	22	12	24	17	10
ENSMUSG00000036880	ACAA2	4	0	0	2	3	6	15	21	11	18	12	14
ENSMUSG00000036882	ARHGAP33	1	4	2	0	4	1	8	4	5	9	4	8
ENSMUSG00000036885	ARHGEF26	0	0	0	0	1	0	1	0	1	1	1	2
ENSMUSG00000036890	GTDC1	0	0	0	0	0	0	4	3	0	5	2	5
ENSMUSG00000036893	EHMT1	22	29	34	39	45	30	155	150	101	175	120	137
ENSMUSG00000036898	ZFP157	2	4	7	3	6	4	24	28	10	29	23	23
ENSMUSG00000036908	UNC93B1	1	0	0	2	1	0	0	0	0	1	0	0
ENSMUSG00000036912	PIWIL4	0	0	0	0	0	0	0	0	1	1	0	0
ENSMUSG00000036915	KIRREL2	0	0	0	1	0	0	0	0	0	0	0	0
ENSMUSG00000036916	ZFP280C	1	8	6	7	8	5	31	32	16	56	28	20
ENSMUSG00000036918	TTC7	0	0	2	6	3	1	11	10	5	7	7	5
ENSMUSG00000036928	STAG3	0	0	0	0	0	0	1	1	1	0	0	0
ENSMUSG00000036931	NFKBID	4	2	1	5	4	0	7	12	6	10	4	4
ENSMUSG00000036932	AIFM1	4	15	4	13	7	9	54	61	57	64	59	51
ENSMUSG00000036934	4921524J17R	8	11	3	6	10	7	60	47	31	38	30	38
ENSMUSG00000036940	KDM1A	2	8	8	8	5	7	47	32	26	47	35	18
ENSMUSG00000036941	ELAC1	1	1	4	1	4	1	5	12	5	7	5	6
ENSMUSG00000036943	RAB8B	2	6	6	1	4	10	14	26	15	29	17	14
ENSMUSG00000036944	TMEM71	2	0	2	2	2	4	13	5	4	16	8	6
ENSMUSG00000036948	BC037034	6	3	8	6	6	6	18	24	12	30	17	20
ENSMUSG00000036955	KIF1BP	2	2	2	4	3	4	19	16	14	15	12	16
ENSMUSG00000036959	BCORL1	6	3	4	6	4	1	17	21	5	18	11	9
ENSMUSG00000036961	WNT8B	0	0	0	0	0	0	0	0	0	0	0	1
ENSMUSG00000036964	TRIM17	0	0	0	0	0	0	0	0	0	0	2	0
ENSMUSG00000036966	SPRYD3	3	3	1	3	2	2	2	2	5	12	15	8
ENSMUSG00000036968	CNPY4	4	7	9	14	9	9	34	29	19	22	20	25
ENSMUSG00000036975	TMEM177	0	1	3	1	0	0	6	5	1	4	6	2
ENSMUSG00000036977	ANAPC10	3	1	0	2	4	5	11	16	19	21	12	13
ENSMUSG00000036980	TAF6	45	57	43	68	38	40	137	126	128	145	102	164
ENSMUSG00000036983	TFB1M	4	3	2	1	1	4	16	11	12	16	7	10
ENSMUSG00000036985	ZDHHC9	3	1	3	2	3	1	11	9	8	17	9	8
ENSMUSG00000036986	PML	6	3	1	2	5	4	10	11	10	15	7	14
ENSMUSG00000036989	TRIM3	4	1	0	0	1	1	13	10	6	13	8	4
ENSMUSG00000036990	OTUD4	22	16	10	18	9	14	97	70	43	100	57	50
ENSMUSG00000036992	NXT1	8	2	2	1	6	6	33	48	34	38	24	26
ENSMUSG00000037001	ZFP39	1	3	3	2	4	1	24	18	16	20	21	18
ENSMUSG00000037003	TNS2	0	0	0	0	0	0	0	0	0	1	0	0
ENSMUSG00000037005	XPNPEP2	0	0	0	0	0	0	0	0	0	1	0	0
ENSMUSG00000037007	ZFP113	3	5	6	6	3	2	14	16	2	12	6	15
ENSMUSG00000037010	APLN	0	0	0	0	0	0	1	0	1	0	0	0
ENSMUSG00000037012	HK1	0	1	4	1	4	2	21	23	16	19	21	18
ENSMUSG00000037013	SS18	10	10	4	9	3	5	27	51	42	52	45	42
ENSMUSG00000037017	ZSCAN21	0	3	1	1	7	1	14	15	11	12	10	17
ENSMUSG00000037020	WDR62	2	1	2	2	1	1	5	8	4	2	1	2
ENSMUSG00000037022	MMAA	1	0	2	1	1	5	3	5	3	3	1	8
ENSMUSG00000037029	ZFP146	2	3	2	6	6	3	12	10	7	7	5	9
ENSMUSG00000037032	APBB1	3	3	0	1	5	2	16	14	10	13	9	10
ENSMUSG00000037049	SMPD1	1	8	6	4	5	5	44	39	29	39	34	28
ENSMUSG00000037058	PAIP2	3	20	9	13	19	13	122	109	101	118	74	112
ENSMUSG00000037062	SH3GLB1	8	17	15	21	15	8	70	74	70	89	68	50
ENSMUSG00000037070	RBMXL1	13	13	10	13	25	10	117	130	98	138	120	87

ENSMUSG00000037071	SCD1	0	0	0	0	0	0	0	0	0	0	0	1
ENSMUSG00000037072	SELENOF	17	22	23	40	33	31	152	215	139	180	141	171
ENSMUSG00000037075	RNF139	2	8	7	3	5	4	21	27	19	24	27	23
ENSMUSG00000037085	TRMT12	2	0	2	0	5	2	4	5	1	8	3	4
ENSMUSG00000037089	SLC35B2	5	8	5	6	5	9	38	44	26	60	42	38
ENSMUSG00000037096	GM9762	0	0	0	0	0	0	1	0	0	0	0	0
ENSMUSG00000037098	RAB11FIP3	1	0	1	2	4	2	5	4	3	6	4	3
ENSMUSG00000037103	DCAF15	5	9	15	14	18	14	55	84	40	59	35	36
ENSMUSG00000037104	SOCS5	0	0	2	2	1	0	8	5	3	3	6	1
ENSMUSG00000037106	FER1L6	1	0	1	1	1	1	3	7	8	12	7	2
ENSMUSG00000037108	ZCWPW1	8	13	6	15	16	8	65	82	48	77	61	64
ENSMUSG00000037110	RALGAPA2	3	8	9	8	9	7	36	35	25	37	32	30
ENSMUSG00000037111	SETD7	8	11	12	16	23	18	70	88	67	79	64	69
ENSMUSG00000037112	SIK2	0	3	2	2	4	2	8	6	9	19	9	11
ENSMUSG00000037119	FAM91A1	5	5	5	3	5	4	36	26	25	37	17	27
ENSMUSG00000037126	PSD	1	0	2	1	0	0	2	9	4	5	5	3
ENSMUSG00000037134	PRMT9	6	0	2	1	0	2	12	24	16	20	11	13
ENSMUSG00000037140	TAS2R108	0	0	0	0	0	0	1	0	0	0	0	0
ENSMUSG00000037143	CFAP61	0	2	1	1	2	0	11	2	5	9	8	8
ENSMUSG00000037148	ARHGAP10	5	4	2	4	1	1	12	21	11	21	12	15
ENSMUSG00000037149	DDX1	10	17	7	20	12	14	74	73	62	74	53	61
ENSMUSG00000037151	LRRC20	1	0	0	0	2	0	5	2	0	7	6	2
ENSMUSG00000037152	NDUFC1	57	35	41	46	52	41	329	282	227	320	228	268
ENSMUSG00000037161	MGARP	1	0	0	0	0	0	0	0	0	0	0	0
ENSMUSG00000037167	SPACA5	0	0	0	0	0	0	0	0	0	0	0	1
ENSMUSG00000037172	E330009J07R	9	9	11	13	13	11	55	67	45	68	43	48
ENSMUSG00000037174	ELF2	8	10	11	13	10	7	71	48	39	72	38	57
ENSMUSG00000037185	KRT80	0	0	0	0	1	0	0	0	1	2	2	1
ENSMUSG00000037188	GRHL3	0	0	0	0	0	0	0	0	0	1	0	0
ENSMUSG00000037190	CYB561D2	1	0	0	1	1	0	7	7	3	2	6	6
ENSMUSG00000037197	RBM17	16	32	29	23	35	23	150	156	113	168	153	140
ENSMUSG00000037204	ATG101	1	1	3	2	2	2	11	13	9	9	11	11
ENSMUSG00000037206	ISLR	0	0	0	0	0	1	0	2	0	0	0	0
ENSMUSG00000037210	FAM193A	10	7	13	11	15	13	54	61	47	65	39	52
ENSMUSG00000037211	SPRY1	1	2	2	2	0	1	3	7	11	8	7	7
ENSMUSG00000037214	THAP1	4	6	3	3	1	3	12	21	8	14	14	14
ENSMUSG00000037216	LIPT1	0	0	1	0	1	0	5	5	1	9	1	4
ENSMUSG00000037217	SYN1	0	0	0	1	0	0	1	0	0	1	0	0
ENSMUSG00000037221	MOSPD3	0	0	1	0	2	0	7	4	0	2	5	5
ENSMUSG00000037224	ZFYVE28	2	1	0	0	0	2	2	5	3	2	0	4
ENSMUSG00000037225	FGF2	0	0	0	0	0	0	7	1	0	1	1	1
ENSMUSG00000037234	HOOK3	12	12	11	10	15	12	43	52	37	55	32	31
ENSMUSG00000037235	MXD4	11	16	28	20	18	15	64	87	58	99	61	66
ENSMUSG00000037236	MATR3	22	28	27	40	38	41	242	235	197	285	187	184
ENSMUSG00000037239	SPRED3	0	1	0	0	0	1	0	1	1	0	0	0
ENSMUSG00000037242	CLIC4	16	13	11	22	17	13	77	86	56	92	57	62
ENSMUSG00000037243	ZFP692	2	7	2	5	6	6	15	18	15	16	10	25
ENSMUSG00000037251	POMK	2	1	3	1	1	2	3	6	3	8	5	2
ENSMUSG00000037253	MEX3C	11	13	9	9	9	10	61	59	42	69	45	61
ENSMUSG00000037257	AAGAB	7	8	8	8	9	6	39	52	32	32	40	49
ENSMUSG00000037259	DZANK1	0	1	0	0	0	0	2	4	2	2	1	1
ENSMUSG00000037260	HGSNAT	4	1	2	0	1	1	6	5	4	6	3	3
ENSMUSG00000037262	KIN	13	26	12	14	20	18	97	88	66	95	77	81
ENSMUSG00000037266	RSRP1	81	101	112	100	108	124	864	888	709	950	658	704
ENSMUSG00000037270	4932438A13F	22	51	33	43	50	49	151	144	96	174	100	110
ENSMUSG00000037275	GEMIN5	5	4	8	8	8	2	30	35	23	33	28	30
ENSMUSG00000037278	TMEM97	4	2	3	4	1	4	33	33	26	28	17	23
ENSMUSG00000037286	STAG1	13	27	12	25	16	14	75	83	54	86	57	58

ENSMUSG00000037287	TBCEL	8	17	13	12	9	9	41	31	16	46	33	41
ENSMUSG00000037295	LDLRAP1	3	2	2	0	7	2	9	10	8	8	7	11
ENSMUSG00000037296	LSM1	7	5	8	11	3	4	45	49	46	56	38	41
ENSMUSG00000037300	TTC13	0	2	3	4	3	3	14	24	18	25	12	14
ENSMUSG00000037306	MAN1C1	0	0	0	1	3	2	1	1	2	3	1	0
ENSMUSG00000037307	BANF2	0	0	0	0	0	0	0	1	0	0	0	0
ENSMUSG00000037313	TACC3	6	12	7	12	17	13	52	78	44	98	61	59
ENSMUSG00000037315	JADE3	1	2	0	0	2	0	10	10	5	13	7	3
ENSMUSG00000037316	BAG4	10	10	7	11	12	9	74	72	38	66	35	48
ENSMUSG00000037318	TRAF3IP3	5	10	8	12	7	9	58	46	29	62	39	33
ENSMUSG00000037321	TAP1	2	2	4	4	4	1	11	15	11	19	15	9
ENSMUSG00000037325	BBS7	0	0	1	1	0	2	8	6	3	11	3	5
ENSMUSG00000037326	CAPN15	4	11	6	12	6	2	25	29	30	33	23	36
ENSMUSG00000037331	LARP1	32	57	45	41	56	52	196	151	142	184	142	165
ENSMUSG00000037336	MFSD2B	0	0	0	0	0	0	0	1	0	1	1	0
ENSMUSG00000037337	MAP4K1	6	7	5	8	11	7	42	43	37	33	50	44
ENSMUSG00000037339	FAM53A	6	5	5	9	5	3	36	45	34	41	30	48
ENSMUSG00000037343	TAF2	6	9	12	8	7	13	41	43	28	48	28	37
ENSMUSG00000037344	SLC12A9	1	2	3	4	3	3	15	8	12	11	6	17
ENSMUSG00000037348	PAQR7	2	2	0	4	0	0	7	8	11	4	6	10
ENSMUSG00000037349	NUDT22	0	0	0	0	1	0	0	0	0	1	0	0
ENSMUSG00000037351	ACTR1B	17	13	9	12	18	11	58	80	48	59	44	54
ENSMUSG00000037353	LETMD1	6	4	7	10	8	4	29	31	29	45	24	29
ENSMUSG00000037355	UVSSA	12	9	8	11	4	8	42	36	29	57	36	37
ENSMUSG00000037361	SF3B6	11	21	13	14	14	16	116	110	93	115	81	82
ENSMUSG00000037362	NOV	0	0	1	0	0	0	0	0	1	0	0	0
ENSMUSG00000037363	LETM2	3	2	0	0	1	2	10	8	3	14	7	9
ENSMUSG00000037364	SRRT	33	49	46	43	48	40	204	252	191	256	187	195
ENSMUSG00000037366	PAFAH2	3	3	2	1	2	1	15	10	6	12	7	10
ENSMUSG00000037369	KDM6A	8	12	5	8	13	8	54	70	37	53	32	33
ENSMUSG00000037373	CTBP1	13	12	13	13	24	28	123	127	91	127	78	76
ENSMUSG00000037376	TRMT6	8	12	11	18	19	13	75	98	59	70	75	75
ENSMUSG00000037395	RCOR3	5	10	4	5	6	6	28	28	14	38	14	23
ENSMUSG00000037400	ATP11B	26	39	40	31	39	31	120	154	93	181	141	116
ENSMUSG00000037405	ICAM1	3	4	6	4	5	2	25	23	24	28	21	27
ENSMUSG00000037406	HTRA4	0	0	0	0	0	0	0	0	0	0	0	1
ENSMUSG00000037408	CNNM4	0	1	3	2	3	4	5	3	4	7	5	13
ENSMUSG00000037410	TBC1D2B	2	0	1	3	1	1	6	11	12	6	4	7
ENSMUSG00000037411	SERPINE1	0	0	0	0	0	0	0	0	0	0	0	1
ENSMUSG00000037415	RANBP10	3	3	3	3	8	4	21	28	18	27	10	21
ENSMUSG00000037416	DMXL1	6	9	5	7	10	7	29	42	24	35	35	32
ENSMUSG00000037419	ENDOD1	0	1	2	2	2	0	4	6	4	8	6	6
ENSMUSG00000037426	DEPDC5	2	1	1	0	5	2	16	10	12	12	10	14
ENSMUSG00000037428	VGF	0	0	0	0	0	0	0	0	0	0	1	0
ENSMUSG00000037432	FER1L5	0	0	0	0	1	0	1	0	0	0	1	0
ENSMUSG00000037434	SLC30A1	6	10	12	9	7	22	54	53	29	47	27	37
ENSMUSG00000037437	ADAM32	0	0	0	0	0	0	0	2	0	0	0	0
ENSMUSG00000037438	UQCRH-PS1	0	0	0	0	0	0	1	1	0	0	0	0
ENSMUSG00000037443	CEP85	0	3	1	2	2	4	17	14	10	11	12	10
ENSMUSG00000037446	TULP1	0	0	0	0	0	0	0	0	1	0	0	0
ENSMUSG00000037447	ARID5A	4	4	4	6	11	7	24	34	24	23	17	23
ENSMUSG00000037458	AZIN1	15	9	11	19	11	10	95	78	74	83	57	64
ENSMUSG00000037461	INTS7	9	8	7	11	9	9	45	46	43	52	39	36
ENSMUSG00000037465	KLF10	5	5	6	5	8	9	39	34	19	27	23	17
ENSMUSG00000037466	TEDC1	5	4	7	4	7	9	24	30	22	19	18	26
ENSMUSG00000037470	UGGT1	14	18	14	12	18	10	73	73	57	75	54	67
ENSMUSG00000037474	DTL	12	21	17	13	21	22	112	97	88	132	81	93
ENSMUSG00000037475	THOC2	30	73	48	67	47	58	205	231	180	263	165	166

ENSMUSG00000037486	ASXL2	15	22	20	35	35	28	163	160	100	174	106	120
ENSMUSG00000037487	UBR5	16	17	16	22	15	19	81	81	59	109	72	74
ENSMUSG00000037490	SLC2A12	8	26	17	22	17	21	50	51	47	69	37	45
ENSMUSG00000037492	ZMAT4	0	0	1	0	0	0	0	0	0	0	0	0
ENSMUSG00000037493	CIB2	0	0	0	0	0	0	1	0	0	0	0	0
ENSMUSG00000037499	NENF	1	3	6	7	2	4	15	14	11	13	9	14
ENSMUSG00000037503	FAM168B	13	14	8	12	18	19	69	94	69	93	66	58
ENSMUSG00000037509	ARHGEF4	4	6	8	5	5	5	25	33	23	21	25	23
ENSMUSG00000037514	PANK2	0	1	3	3	8	0	19	18	10	17	19	21
ENSMUSG00000037519	PPFIA1	8	7	2	6	8	9	27	37	37	38	54	21	14
ENSMUSG00000037523	MAVS	2	6	0	1	2	2	16	26	21	16	8	15
ENSMUSG00000037525	BCDIN3D	2	4	0	2	3	1	7	8	5	10	6	14
ENSMUSG00000037526	ATG14	2	4	1	3	5	4	14	12	5	23	8	17
ENSMUSG00000037531	MRPL47	5	16	11	32	18	20	105	121	78	110	103	102
ENSMUSG00000037533	RAPGEF6	23	16	16	14	15	13	82	104	65	104	63	77
ENSMUSG00000037535	1700021A07F	0	0	1	0	0	0	2	3	0	1	0	1
ENSMUSG00000037536	FBXO34	3	5	2	7	9	3	12	15	13	21	18	12
ENSMUSG00000037541	SHANK2	0	0	0	0	0	0	0	0	0	1	0	0
ENSMUSG00000037542	ALDH8A1	1	0	0	0	0	0	1	0	0	0	0	0
ENSMUSG00000037544	DLGAP5	1	6	6	6	7	6	25	30	25	32	15	28
ENSMUSG00000037552	PLEKHG2	75	90	83	105	95	80	358	404	337	388	268	376
ENSMUSG00000037553	ZDHHC18	0	0	3	4	7	2	17	13	12	10	8	14
ENSMUSG00000037563	RPS16	94	117	104	136	113	116	755	856	596	824	672	697
ENSMUSG00000037568	VASH2	0	0	0	0	0	0	0	0	0	0	1	0
ENSMUSG00000037570	MCRS1	5	3	2	4	2	2	6	8	5	8	5	20
ENSMUSG00000037572	WDHD1	16	24	22	15	20	21	128	129	91	158	95	112
ENSMUSG00000037573	TOB1	1	9	5	7	10	8	50	51	43	66	37	41
ENSMUSG00000037577	EPHX3	0	0	0	0	0	0	0	1	1	0	0	0
ENSMUSG00000037578	PKD2L1	0	0	0	0	0	0	1	0	0	0	0	0
ENSMUSG00000037580	GCH1	3	3	2	4	3	1	8	5	4	15	7	9
ENSMUSG00000037593	BC030499	0	0	0	0	0	0	0	0	0	0	0	1
ENSMUSG00000037594	BC022687	1	0	1	0	0	0	1	1	1	0	3	4
ENSMUSG00000037600	KDF1	0	1	0	0	0	0	0	0	0	0	0	0
ENSMUSG00000037601	NME1	45	45	51	55	50	35	332	352	260	313	256	280
ENSMUSG00000037606	OSBPL5	1	0	2	1	0	1	1	6	5	3	5	4
ENSMUSG00000037608	BCLAF1	67	108	111	123	94	87	471	540	321	481	385	460
ENSMUSG00000037613	TNFRSF23	1	0	0	0	0	1	0	2	0	0	0	0
ENSMUSG00000037617	SPAG1	3	3	0	1	1	1	8	8	10	14	8	6
ENSMUSG00000037621	ATOH8	0	1	0	0	0	0	0	0	0	0	0	0
ENSMUSG00000037622	WDTC1	0	0	1	1	0	1	2	5	8	5	7	5
ENSMUSG00000037628	CDKN3	0	1	0	1	0	1	5	1	5	4	4	4
ENSMUSG00000037640	ZFP60	0	5	1	1	1	6	12	10	15	10	10	10
ENSMUSG00000037643	PRKCI	2	10	5	10	4	3	37	44	26	37	21	25
ENSMUSG00000037646	VPS13B	3	6	5	6	7	5	23	26	19	32	27	23
ENSMUSG00000037649	H2-DMA	0	0	0	0	0	0	0	1	0	2	0	1
ENSMUSG00000037652	PHC3	6	7	2	6	4	5	34	36	17	29	26	30
ENSMUSG00000037656	SLC20A2	1	1	4	1	2	5	11	16	8	21	8	6
ENSMUSG00000037661	GPR160	0	0	0	0	0	0	1	1	1	2	3	1
ENSMUSG00000037664	CDKN1C	5	13	18	10	13	11	56	53	48	53	49	43
ENSMUSG00000037669	LDAH	5	2	7	1	4	1	15	19	9	16	13	14
ENSMUSG00000037674	RFX7	12	17	11	15	17	19	96	106	63	131	79	87
ENSMUSG00000037679	INF2	1	1	1	3	3	0	3	4	1	8	2	2
ENSMUSG00000037681	ESYT3	0	0	0	0	0	0	0	0	1	0	2	1
ENSMUSG00000037683	ARMC3	2	0	1	1	0	1	4	7	4	4	5	3
ENSMUSG00000037685	ATP8A1	3	5	2	2	2	1	6	11	5	8	6	10
ENSMUSG00000037692	AHDC1	7	12	11	12	9	11	31	31	31	26	24	21
ENSMUSG00000037697	DDHD1	7	13	11	14	10	9	37	33	26	46	23	36
ENSMUSG00000037703	LZTS3	1	0	0	0	0	0	0	2	1	0	1	0

ENSMUSG00000037706	CD81	0	2	0	0	0	0	1	0	0	1	3	0
ENSMUSG00000037709	FAM13A	0	0	0	0	0	0	1	0	1	0	0	0
ENSMUSG00000037710	CISD1	8	4	3	6	5	7	40	46	34	45	25	30
ENSMUSG00000037712	FERMT2	0	0	0	0	0	0	1	0	0	0	0	0
ENSMUSG00000037716	CCDC33	0	0	0	0	0	0	0	1	0	0	0	0
ENSMUSG00000037720	TMEM33	10	5	6	12	12	14	83	45	48	62	53	57
ENSMUSG00000037722	GNPNAT1	8	4	6	2	4	3	20	22	23	18	13	13
ENSMUSG00000037725	CKAP2	9	5	2	11	7	8	48	35	28	51	31	45
ENSMUSG00000037730	MYNN	7	5	3	5	3	7	23	32	35	40	24	24
ENSMUSG00000037736	LIMCH1	0	0	0	0	0	0	4	0	0	0	0	0
ENSMUSG00000037737	ACTRT3	0	0	0	0	0	1	0	0	1	0	0	0
ENSMUSG00000037738	NEK5	0	0	0	0	0	0	0	0	0	0	0	1
ENSMUSG00000037740	MRPS26	3	6	14	12	10	8	53	45	27	43	35	39
ENSMUSG00000037742	EEF1A1	11	10	21	20	12	11	99	88	69	139	80	78
ENSMUSG00000037750	FAM222B	0	3	1	3	2	1	13	14	8	6	4	2
ENSMUSG00000037752	XKR8	0	1	0	2	0	0	2	3	1	5	4	2
ENSMUSG00000037754	PPP1R16B	5	10	16	15	8	12	43	56	32	40	37	31
ENSMUSG00000037759	PTGER2	1	0	0	0	0	0	0	0	0	0	0	0
ENSMUSG00000037761	ACTR5	5	8	4	5	7	6	39	31	26	17	19	23
ENSMUSG00000037772	MRPL23	1	0	0	0	1	0	1	2	1	4	2	2
ENSMUSG00000037773	PCED1A	1	3	5	1	3	8	15	18	9	13	5	14
ENSMUSG00000037787	APOPT1	0	4	2	4	1	6	30	15	12	17	17	11
ENSMUSG00000037788	VOPP1	3	0	4	2	1	2	9	8	5	7	8	9
ENSMUSG00000037791	PHF12	12	15	14	11	17	13	65	59	43	76	48	59
ENSMUSG00000037795	N4BP2	11	9	10	13	9	9	50	45	32	53	39	38
ENSMUSG00000037801	IQCH	0	0	0	1	0	0	2	1	0	0	0	3
ENSMUSG00000037805	RPL10A	31	51	47	54	41	35	289	257	178	298	199	223
ENSMUSG00000037808	FAM76B	1	12	8	7	7	10	58	71	42	69	43	48
ENSMUSG00000037813	D630003M21	0	0	0	0	0	0	1	0	0	1	0	0
ENSMUSG00000037815	CTNNA1	5	8	5	12	10	6	38	25	25	43	25	33
ENSMUSG00000037816	FBXW17	0	7	2	1	1	2	14	10	8	7	11	12
ENSMUSG00000037818	ABHD18	7	5	3	3	4	3	24	25	17	39	19	24
ENSMUSG00000037822	SMIM14	3	4	3	5	5	4	39	28	24	31	17	16
ENSMUSG00000037824	TSPAN14	0	1	1	0	3	2	6	4	6	9	1	4
ENSMUSG00000037826	PPM1K	7	10	11	8	4	9	32	48	27	50	34	43
ENSMUSG00000037833	SH2D4B	0	0	0	0	0	1	0	0	0	0	0	0
ENSMUSG00000037845	FDXACB1	0	1	0	1	0	1	3	3	1	5	3	1
ENSMUSG00000037846	RTKN2	1	1	1	1	3	4	5	11	9	14	5	10
ENSMUSG00000037847	NMRK1	0	0	1	0	0	1	3	1	0	2	1	1
ENSMUSG00000037851	IARS	7	17	19	16	24	13	83	95	38	72	47	61
ENSMUSG00000037852	CPE	0	0	0	0	0	0	0	0	0	0	1	0
ENSMUSG00000037855	ZFP365	0	1	1	0	0	0	0	0	0	0	0	0
ENSMUSG00000037857	NUFIP2	41	44	29	62	42	33	208	228	171	244	142	196
ENSMUSG00000037860	AIM2	10	8	8	6	4	7	2	3	3	7	1	2
ENSMUSG00000037868	EGR2	0	0	0	0	0	0	0	2	0	0	0	1
ENSMUSG00000037872	ACKR1	1	1	0	0	1	2	10	7	6	6	8	11
ENSMUSG00000037876	JMJD1C	6	7	15	12	17	7	86	58	43	83	57	44
ENSMUSG00000037885	STK35	59	59	69	80	65	62	229	285	215	280	196	255
ENSMUSG00000037890	WDR19	1	1	0	1	0	2	2	1	1	0	4	2
ENSMUSG00000037894	H2AFZ	4	10	12	11	15	6	73	80	60	75	55	56
ENSMUSG00000037896	RCOR1	7	15	6	16	15	14	86	70	66	105	55	42
ENSMUSG00000037904	ANKRD9	1	0	0	2	0	0	4	4	2	3	3	3
ENSMUSG00000037905	BRI3BP	20	35	32	41	34	36	147	143	138	163	119	133
ENSMUSG00000037907	ANKRD13B	7	7	8	7	12	7	31	40	37	46	28	31
ENSMUSG00000037913	TMEM156	1	2	0	4	2	2	5	12	3	5	8	6
ENSMUSG00000037916	NDUFV1	9	7	17	13	15	13	78	94	60	88	63	74
ENSMUSG00000037921	DDX60	0	0	0	0	0	1	1	1	2	1	0	1
ENSMUSG00000037922	BANK1	0	0	0	0	1	0	0	0	0	0	0	0

ENSMUSG00000037926	SSH2	13	13	15	17	19	17	54	69	42	64	46	36
ENSMUSG00000037933	BICD2	7	8	5	3	10	5	34	46	36	48	25	39
ENSMUSG00000037935	SMARCE1	11	14	9	15	16	12	87	87	70	85	80	60
ENSMUSG00000037936	SCARB1	1	0	0	1	0	0	4	4	1	7	1	1
ENSMUSG00000037938	CHCHD5	2	0	4	0	3	1	10	5	4	8	8	4
ENSMUSG00000037940	INPP4B	2	0	0	0	1	0	1	1	1	2	0	0
ENSMUSG00000037944	CCR7	1	0	0	0	1	0	2	3	1	1	0	0
ENSMUSG00000037946	FGD3	3	0	0	3	4	3	8	13	6	20	8	7
ENSMUSG00000037949	ANO10	0	1	2	1	1	1	13	8	14	15	5	5
ENSMUSG00000037957	WDR20	2	4	2	5	2	1	25	22	25	26	13	14
ENSMUSG00000037958	NSRP1	10	19	9	13	18	30	72	54	43	82	52	56
ENSMUSG00000037960	CARD19	7	7	5	9	5	5	28	25	22	22	20	30
ENSMUSG00000037962	RFLNA	0	0	0	0	0	0	0	0	0	0	1	1
ENSMUSG00000037965	ZC3H7A	47	69	52	85	92	75	385	325	209	392	228	255
ENSMUSG00000037966	NINJ1	1	2	2	0	2	0	16	5	10	15	10	18
ENSMUSG00000037971	1110032A03I	1	1	1	3	4	0	6	14	5	8	9	7
ENSMUSG00000037972	SNN	1	0	1	1	2	1	4	11	2	7	4	6
ENSMUSG00000037979	CCDC92	0	0	1	0	1	0	4	5	2	6	6	5
ENSMUSG00000037991	RMI2	10	9	7	7	3	5	39	35	23	22	20	29
ENSMUSG00000037992	RARA	3	14	10	8	6	7	32	33	26	35	17	25
ENSMUSG00000037993	DHX38	4	5	20	8	15	14	46	39	32	40	44	41
ENSMUSG00000037994	SLC9B2	0	1	1	0	2	0	8	10	3	12	2	11
ENSMUSG00000037997	PARP11	1	1	4	2	1	4	9	14	11	11	12	10
ENSMUSG00000037999	ARAP2	4	9	2	7	7	9	19	19	14	28	19	17
ENSMUSG00000038000	ACD	8	6	6	3	11	4	25	22	24	27	18	29
ENSMUSG00000038002	CRAMP1L	8	17	16	17	18	18	77	52	49	69	69	49
ENSMUSG00000038005	HPF1	12	11	14	12	15	17	55	73	50	53	52	55
ENSMUSG00000038007	ACER2	19	29	25	30	33	26	111	143	90	117	80	88
ENSMUSG00000038010	CCDC138	1	0	0	2	3	0	6	6	8	5	1	3
ENSMUSG00000038011	DNAH10	0	0	0	0	0	1	0	0	0	0	0	0
ENSMUSG00000038013	WIPF2	6	8	8	12	10	5	26	29	23	32	23	20
ENSMUSG00000038014	FAM120A	41	33	42	51	31	55	249	234	162	236	168	204
ENSMUSG00000038020	RAPGEFL1	0	0	0	0	0	0	1	0	1	1	1	0
ENSMUSG00000038023	ATP6V0A2	2	4	5	8	3	4	21	21	28	22	13	14
ENSMUSG00000038024	DENND4C	1	0	1	2	1	3	4	8	6	5	3	1
ENSMUSG00000038025	PHF2	4	5	9	6	6	6	33	35	25	30	23	33
ENSMUSG00000038028	TIGAR	0	2	6	2	0	3	10	13	5	8	5	13
ENSMUSG00000038034	IGSF8	4	8	6	6	3	4	20	22	15	32	27	24
ENSMUSG00000038037	SOCS1	3	1	0	4	3	7	18	21	22	15	11	18
ENSMUSG00000038039	GCC2	5	20	18	30	17	18	89	75	72	88	70	63
ENSMUSG00000038042	PTPDC1	0	0	0	0	0	0	2	2	1	2	2	2
ENSMUSG00000038045	SULT6B1	0	0	0	0	0	0	0	0	0	0	1	0
ENSMUSG00000038046	MRM3	2	6	5	6	6	4	19	20	12	26	14	31
ENSMUSG00000038047	HAUS6	31	21	24	37	27	27	154	167	131	181	136	122
ENSMUSG00000038055	DEXI	0	4	1	0	2	0	6	9	6	5	6	9
ENSMUSG00000038056	KMT2C	10	16	9	18	10	12	41	56	44	69	38	42
ENSMUSG00000038057	DBIL5	0	0	0	0	0	0	0	0	0	0	1	0
ENSMUSG00000038058	NOD1	0	0	1	1	1	1	1	0	2	3	0	3
ENSMUSG00000038065	MTURN	0	0	0	0	1	0	1	2	0	1	1	1
ENSMUSG00000038069	CDKN2AIP	7	10	11	5	11	6	55	47	49	34	32	48
ENSMUSG00000038072	GALNT11	0	1	4	4	2	1	18	10	14	20	14	17
ENSMUSG00000038074	FKBP14	0	2	1	4	2	0	6	9	3	4	4	6
ENSMUSG00000038079	TMEM237	4	10	4	5	6	4	23	27	24	30	23	17
ENSMUSG00000038080	KDM1B	4	17	11	12	10	3	41	38	37	60	32	34
ENSMUSG00000038084	OPA1	11	6	4	11	5	8	33	44	41	52	40	41
ENSMUSG00000038085	CNBD2	2	1	3	0	1	0	3	4	3	3	1	1
ENSMUSG00000038095	SBNO1	81	118	95	125	101	115	401	416	314	452	296	355
ENSMUSG00000038102	TRAPPC11	1	9	6	3	3	4	35	47	18	36	21	24

ENSMUSG00000038116	PHF20	9	11	4	11	7	18	36	38	24	43	31	33
ENSMUSG00000038119	CDON	1	0	0	0	1	0	3	3	2	4	3	2
ENSMUSG00000038121	FAM210A	5	7	11	6	10	7	30	49	20	29	20	19
ENSMUSG00000038122	TBC1D32	0	1	4	2	0	0	12	9	4	16	6	3
ENSMUSG00000038126	MPHOSPH9	12	11	10	12	14	15	56	54	29	70	43	40
ENSMUSG00000038127	CCDC50	23	23	29	25	28	32	105	110	78	165	87	78
ENSMUSG00000038128	CAMK4	1	4	4	8	3	6	16	12	10	11	15	13
ENSMUSG00000038132	RBM24	0	0	0	0	0	0	0	0	0	0	1	0
ENSMUSG00000038135	CRYGN	0	0	0	0	0	0	0	0	0	1	0	0
ENSMUSG00000038141	TMEM181A	7	3	1	17	5	10	16	23	10	11	13	25
ENSMUSG00000038145	SNRK	2	4	0	5	4	5	7	12	7	13	8	7
ENSMUSG00000038150	ORMDL3	2	5	6	8	6	6	27	16	13	20	19	14
ENSMUSG00000038151	PRDM1	0	0	0	1	0	1	0	1	0	0	0	3
ENSMUSG00000038152	5033430I15R	2	0	0	0	1	0	2	1	2	2	1	4
ENSMUSG00000038156	SPON1	0	1	0	0	0	0	0	0	0	1	1	0
ENSMUSG00000038160	ATG5	10	3	4	5	8	2	28	34	25	19	9	16
ENSMUSG00000038168	P3H2	0	0	0	0	0	0	0	0	1	0	0	0
ENSMUSG00000038170	PDE4DIP	6	2	7	11	7	11	20	33	19	28	31	21
ENSMUSG00000038172	TTC39B	1	6	3	2	3	2	16	19	13	25	13	13
ENSMUSG00000038173	ENPP6	0	0	0	1	0	0	0	1	1	0	0	0
ENSMUSG00000038174	FAM126B	6	11	10	16	10	3	39	45	21	32	16	33
ENSMUSG00000038175	MYLIP	0	1	0	0	1	0	0	0	1	3	1	0
ENSMUSG00000038178	SLC43A2	2	0	1	2	0	2	7	4	2	5	7	10
ENSMUSG00000038179	SLAMF7	1	0	0	0	0	0	0	0	0	0	0	0
ENSMUSG00000038180	SPAG4	0	1	0	0	0	0	4	3	2	1	3	2
ENSMUSG00000038181	CHPF2	3	5	4	2	7	4	25	20	20	21	20	23
ENSMUSG00000038187	BTBD10	1	2	0	1	2	1	8	13	8	5	10	9
ENSMUSG00000038188	SCARF1	0	0	0	0	0	0	0	1	0	0	1	0
ENSMUSG00000038195	RILP	0	0	0	0	0	0	0	0	0	3	0	0
ENSMUSG00000038205	PRKAB2	3	3	4	3	2	1	15	5	11	8	13	8
ENSMUSG00000038206	FBXO8	2	5	4	2	5	4	30	37	17	29	18	29
ENSMUSG00000038208	PGAP3	0	3	4	4	4	1	4	9	7	8	1	7
ENSMUSG00000038210	HOXA11	0	0	0	0	0	0	0	0	0	1	0	0
ENSMUSG00000038212	MFSD14B	5	4	5	5	5	3	19	30	21	34	25	20
ENSMUSG00000038213	TAPBPL	4	3	3	5	6	3	18	15	19	15	8	17
ENSMUSG00000038214	BEND3	1	1	3	4	3	3	13	13	11	14	4	14
ENSMUSG00000038215	CEP44	5	2	2	7	13	10	35	39	35	58	45	40
ENSMUSG00000038217	TLCD2	1	0	0	0	0	0	0	0	0	0	0	0
ENSMUSG00000038224	SERPINF2	0	0	0	0	0	0	0	0	1	0	0	0
ENSMUSG00000038225	PRIMPOL	2	2	0	2	0	3	9	14	7	20	9	15
ENSMUSG00000038236	HOXA7	0	0	0	0	0	0	0	0	1	0	0	0
ENSMUSG00000038239	HRC	0	0	0	0	0	0	0	0	0	0	1	0
ENSMUSG00000038240	PDSS2	3	3	0	1	1	0	12	15	5	11	11	11
ENSMUSG00000038241	CEP250	9	14	6	18	10	15	43	56	44	68	37	58
ENSMUSG00000038248	SOBP	0	0	0	0	0	0	0	1	1	0	0	0
ENSMUSG00000038250	USP38	9	5	2	10	5	10	56	62	35	55	53	37
ENSMUSG00000038252	NCAPD2	10	12	23	19	15	20	82	74	70	75	79	77
ENSMUSG00000038256	BCL9	5	2	1	5	1	3	11	18	9	14	5	9
ENSMUSG00000038260	TRPM4	0	0	0	0	1	0	0	0	0	0	1	1
ENSMUSG00000038268	OVCA2	1	0	0	1	0	0	6	4	2	5	4	2
ENSMUSG00000038271	IFFO1	1	2	2	5	6	2	9	8	8	13	5	11
ENSMUSG00000038274	FAU	160	171	183	223	221	205	703	727	567	692	600	643
ENSMUSG00000038279	NOP2	11	9	16	14	22	11	79	83	47	82	53	67
ENSMUSG00000038280	OSTM1	2	8	6	7	3	9	20	28	17	25	16	19
ENSMUSG00000038286	BPHL	1	1	2	0	2	0	7	15	8	7	7	10
ENSMUSG00000038290	SMG6	3	7	4	4	2	6	16	19	5	18	10	13
ENSMUSG00000038291	SNX25	16	11	18	16	13	21	87	70	67	88	60	60
ENSMUSG00000038292	CCDC155	0	0	0	0	0	0	1	0	1	0	1	0

ENSMUSG00000038295	ATG9B	0	0	0	0	0	0	0	0	0	1	0	1
ENSMUSG00000038296	GALNT18	0	0	0	0	0	0	2	1	1	2	1	0
ENSMUSG00000038298	PDZK1	0	1	1	0	1	4	4	3	1	5	3	3
ENSMUSG00000038299	WDR36	32	26	36	29	21	27	187	195	147	181	144	147
ENSMUSG00000038300	PTH2	0	0	0	0	0	0	0	1	0	0	0	0
ENSMUSG00000038301	SNX10	9	7	6	8	3	6	41	35	27	35	30	31
ENSMUSG00000038302	AFG1L	0	0	0	0	1	0	1	6	3	3	4	4
ENSMUSG00000038304	CD160	24	41	33	42	43	28	201	215	141	215	147	177
ENSMUSG00000038305	SPATS2L	0	0	0	0	0	0	0	0	0	0	1	0
ENSMUSG00000038312	EDEM2	5	7	4	9	6	6	32	35	25	20	19	22
ENSMUSG00000038319	KCNH2	0	0	0	0	0	0	1	0	0	0	0	0
ENSMUSG00000038323	1700066M21	1	3	2	3	2	0	9	8	9	12	7	3
ENSMUSG00000038324	TRPC4AP	17	20	19	21	23	25	101	116	95	106	92	97
ENSMUSG00000038332	SESN1	10	5	6	3	8	4	53	49	33	41	48	41
ENSMUSG00000038335	TSR1	12	16	9	18	14	12	88	71	51	91	73	70
ENSMUSG00000038342	MLXIP	10	8	13	4	4	4	34	44	36	44	36	41
ENSMUSG00000038344	TXLNG	5	2	5	4	1	10	20	15	15	34	12	17
ENSMUSG00000038346	ZFP384	2	8	8	6	8	5	12	24	22	21	6	26
ENSMUSG00000038349	PLCL1	0	0	0	0	0	0	0	0	1	0	0	1
ENSMUSG00000038351	SGSM2	1	5	6	2	3	6	13	10	12	11	10	10
ENSMUSG00000038352	ARL5C	2	2	2	1	2	1	12	4	8	4	2	4
ENSMUSG00000038354	ANKRD35	0	0	0	0	0	0	0	1	0	0	0	0
ENSMUSG00000038365	FBXO25	1	0	0	0	1	0	5	7	4	7	7	5
ENSMUSG00000038366	LASP1	1	1	1	1	2	0	6	11	6	9	5	7
ENSMUSG00000038368	FOCAD	0	0	1	2	0	0	1	4	0	1	2	0
ENSMUSG00000038369	NCOA6	6	8	12	11	16	12	51	39	33	50	32	38
ENSMUSG00000038371	SBF2	8	7	6	4	5	12	39	34	34	58	25	40
ENSMUSG00000038372	GMDS	4	4	2	2	3	1	8	15	11	5	21	13
ENSMUSG00000038374	RBM8A	25	41	36	32	33	26	221	222	157	215	171	210
ENSMUSG00000038375	TRP53INP2	0	0	0	1	1	0	2	3	0	1	0	2
ENSMUSG00000038379	TTK	12	12	6	17	8	15	75	54	66	44	44	49
ENSMUSG00000038383	PIGU	4	15	4	7	6	9	29	31	28	29	21	29
ENSMUSG00000038384	SETD1B	8	10	18	21	14	14	62	64	33	64	42	49
ENSMUSG00000038387	RRAS	0	0	1	0	0	0	0	2	1	3	2	4
ENSMUSG00000038388	MPP6	19	23	20	35	21	21	133	117	93	113	115	115
ENSMUSG00000038390	GPR162	0	0	1	0	0	1	0	0	0	2	0	0
ENSMUSG00000038393	TXNIP	20	32	26	24	28	21	109	146	93	134	105	121
ENSMUSG00000038398	UPF3A	4	2	3	5	3	6	14	23	23	31	18	13
ENSMUSG00000038406	SCAF1	2	8	4	10	9	9	32	36	25	32	27	36
ENSMUSG00000038408	1700018A04F	0	0	0	0	0	0	0	0	0	0	0	1
ENSMUSG00000038412	HIGD1A	7	13	8	7	11	7	119	103	74	79	76	82
ENSMUSG00000038415	FOXQ1	0	2	5	0	3	1	8	3	9	10	9	5
ENSMUSG00000038416	CDC16	8	12	8	13	18	16	56	73	44	69	39	60
ENSMUSG00000038417	FIG4	2	6	2	5	2	3	30	22	17	21	22	24
ENSMUSG00000038418	EGR1	1	1	2	2	1	2	10	11	11	12	7	10
ENSMUSG00000038422	HDHD3	0	0	0	0	0	1	0	0	1	0	0	0
ENSMUSG00000038425	POLI	0	4	9	2	5	2	26	16	14	15	18	18
ENSMUSG00000038429	USP5	4	6	6	5	8	9	38	40	32	39	35	36
ENSMUSG00000038437	MLLT6	12	15	13	16	19	13	69	77	52	87	50	81
ENSMUSG00000038446	CDC40	7	11	2	12	12	11	34	49	39	54	27	37
ENSMUSG00000038451	SPSB2	0	2	1	0	1	0	5	4	2	2	5	7
ENSMUSG00000038453	SRCIN1	0	0	0	0	0	0	0	0	0	0	0	1
ENSMUSG00000038456	DENND2A	0	1	0	1	0	0	0	1	2	1	0	2
ENSMUSG00000038459	ABHD17C	21	23	38	30	31	30	119	111	83	133	71	104
ENSMUSG00000038462	UQCRFS1	31	43	38	37	39	42	255	236	203	282	219	210
ENSMUSG00000038467	CHMP4B	16	28	16	33	30	25	139	133	107	145	117	86
ENSMUSG00000038473	NOS1AP	0	1	0	0	0	0	1	4	3	1	1	1
ENSMUSG00000038481	CDK19	8	7	6	6	14	6	22	29	30	21	21	19

ENSMUSG00000038482	TFDP1	17	23	24	25	34	23	155	139	88	201	115	103
ENSMUSG00000038485	SOCS7	5	5	3	3	3	2	16	19	13	15	19	13
ENSMUSG00000038486	SV2A	0	1	1	0	2	1	6	5	0	3	5	1
ENSMUSG00000038489	POLR2L	1	2	0	3	4	1	6	7	4	7	9	8
ENSMUSG00000038491	CDK19OS	0	0	0	0	0	0	0	1	0	1	2	0
ENSMUSG00000038495	OTUD7B	4	5	9	12	6	9	30	53	41	35	38	33
ENSMUSG00000038497	TMCO3	2	2	1	1	4	2	9	8	6	10	12	12
ENSMUSG00000038500	PRR3	9	5	9	5	8	11	33	47	24	30	29	24
ENSMUSG00000038502	PTOV1	5	8	7	9	6	3	40	32	21	22	13	29
ENSMUSG00000038503	MESD	3	1	5	5	3	2	20	21	18	34	27	21
ENSMUSG00000038506	DCUN1D2	0	1	2	2	3	1	8	11	8	6	7	13
ENSMUSG00000038507	PARP12	1	1	0	0	0	1	6	2	4	5	6	3
ENSMUSG00000038510	RPF2	4	11	12	16	10	12	56	84	79	123	67	80
ENSMUSG00000038515	GRTP1	0	11	1	10	4	6	16	19	9	11	19	16
ENSMUSG00000038517	TBKBP1	0	0	0	1	1	0	0	1	1	0	1	0
ENSMUSG00000038518	JARID2	20	35	32	43	42	20	170	178	116	151	123	145
ENSMUSG00000038520	TBC1D17	1	7	2	3	2	0	4	10	12	2	4	8
ENSMUSG00000038522	MFSD4B1	0	0	0	0	0	0	0	0	0	1	0	0
ENSMUSG00000038523	1700003F12F	0	0	0	0	0	0	0	2	1	0	1	0
ENSMUSG00000038524	FCHSD1	1	0	1	0	1	0	6	6	3	2	9	6
ENSMUSG00000038525	ARMC10	6	9	10	10	14	9	40	31	38	48	27	28
ENSMUSG00000038526	CAR14	0	0	2	1	0	0	1	3	1	4	0	0
ENSMUSG00000038528	MFSD4B5	0	0	0	0	0	0	0	0	0	0	0	1
ENSMUSG00000038533	CBFA2T2	1	0	4	4	4	2	14	22	11	24	10	19
ENSMUSG00000038534	OSBPL7	3	2	5	2	1	2	12	9	10	12	2	8
ENSMUSG00000038535	ZFP280D	2	9	3	7	10	7	32	33	21	44	22	23
ENSMUSG00000038538	UBN2	25	37	33	32	33	40	117	119	91	130	94	97
ENSMUSG00000038539	ATF5	1	2	4	0	3	2	6	14	11	10	5	6
ENSMUSG00000038540	TMC3	0	0	0	0	0	0	0	2	0	0	0	0
ENSMUSG00000038541	SRD5A2	0	0	0	0	0	0	0	0	0	1	0	0
ENSMUSG00000038542	PCID2	2	0	1	1	2	2	3	12	2	7	3	7
ENSMUSG00000038543	BC028528	0	1	0	0	0	0	0	0	1	0	0	1
ENSMUSG00000038544	INIP	16	12	10	14	17	17	58	55	45	55	38	51
ENSMUSG00000038545	CUL7	13	13	11	16	7	4	55	61	37	64	45	73
ENSMUSG00000038546	RANBP9	17	13	13	18	8	3	54	71	42	67	49	60
ENSMUSG00000038550	CIART	0	1	0	0	0	0	0	0	0	0	1	2
ENSMUSG00000038560	SP6	1	5	6	6	4	8	29	13	19	20	11	22
ENSMUSG00000038563	EFL1	3	6	5	3	6	6	23	18	20	21	17	14
ENSMUSG00000038564	IFT172	0	1	0	0	0	0	1	0	0	4	0	1
ENSMUSG00000038569	RAD9B	4	1	4	5	4	4	19	27	25	25	21	22
ENSMUSG00000038570	SAXO2	0	0	0	1	0	1	4	4	2	4	2	2
ENSMUSG00000038578	SUSD1	0	3	1	0	1	1	2	3	2	2	0	5
ENSMUSG00000038582	PPTC7	15	11	11	11	12	16	53	47	39	48	48	45
ENSMUSG00000038583	PLN	0	0	0	0	1	0	0	0	0	0	0	0
ENSMUSG00000038587	AKAP12	1	1	1	4	1	0	6	4	2	3	5	3
ENSMUSG00000038593	TCTN1	1	0	2	1	2	1	3	9	3	5	3	2
ENSMUSG00000038598	AI481877	0	0	0	0	0	0	1	0	0	1	0	0
ENSMUSG00000038604	RIPOR1	1	1	1	1	4	2	15	10	11	16	11	14
ENSMUSG00000038605	SAMD10	0	0	2	0	1	1	5	4	1	4	0	3
ENSMUSG00000038608	DOCK10	12	19	14	20	16	12	79	84	65	82	57	59
ENSMUSG00000038611	PHRF1	14	12	15	15	15	11	66	95	57	76	52	78
ENSMUSG00000038612	MCL1	17	18	16	25	26	21	83	74	82	129	91	83
ENSMUSG00000038615	NFE2L1	12	6	6	15	11	18	75	71	54	73	49	70
ENSMUSG00000038618	RASSF7	0	1	0	0	0	0	1	3	4	5	2	4
ENSMUSG00000038619	ENSA	12	9	6	8	11	4	54	38	26	31	36	25
ENSMUSG00000038622	MED30	11	16	10	10	11	10	108	118	84	115	75	94
ENSMUSG00000038623	TM6SF1	0	2	2	1	0	1	0	1	0	0	0	0
ENSMUSG00000038628	POLR3K	4	7	7	4	7	6	39	41	22	30	30	25

ENSMUSG00000038633	DEGS1	2	4	7	7	6	6	35	45	37	41	30	41
ENSMUSG00000038637	LRRC56	0	0	0	0	0	0	1	1	0	1	0	1
ENSMUSG00000038644	POLD1	20	17	25	32	29	24	88	124	98	119	94	111
ENSMUSG00000038646	FAM103A1	2	1	1	2	1	3	23	21	15	24	12	20
ENSMUSG00000038648	CREB3L2	7	9	12	12	11	7	29	30	34	35	27	30
ENSMUSG00000038650	RNH1	13	16	11	12	13	11	75	99	60	73	81	86
ENSMUSG00000038658	RIC1	20	9	20	14	15	18	77	72	58	72	50	54
ENSMUSG00000038664	HERC1	27	30	22	23	19	25	115	120	88	154	90	106
ENSMUSG00000038665	DGKI	0	0	0	0	0	0	1	0	0	0	0	0
ENSMUSG00000038671	ARFRP1	5	5	4	8	11	8	43	39	36	43	51	34
ENSMUSG00000038679	TRPS1	4	9	7	5	3	6	14	13	6	8	6	6
ENSMUSG00000038683	PAK1IP1	18	22	18	25	24	20	138	167	100	160	135	146
ENSMUSG00000038685	RTEL1	1	1	3	3	2	9	19	9	12	21	14	17
ENSMUSG00000038690	ATP5J2	47	50	39	65	50	44	322	411	284	330	271	298
ENSMUSG00000038692	HOXB4	0	1	0	0	0	0	0	0	0	0	0	0
ENSMUSG00000038695	JOSD2	0	1	0	0	0	0	0	0	0	0	0	0
ENSMUSG00000038696	MAPKAP1	6	6	9	6	7	3	34	32	23	35	33	31
ENSMUSG00000038697	TAF5L	14	15	14	23	24	18	82	101	55	84	58	58
ENSMUSG00000038705	GMEB2	2	3	1	5	3	2	17	15	11	22	12	13
ENSMUSG00000038708	GOLGA4	18	35	34	26	42	31	136	119	85	123	104	118
ENSMUSG00000038712	MINDY1	2	2	2	0	0	2	7	2	6	6	5	8
ENSMUSG00000038717	ATP5L	1	5	3	9	6	8	17	11	11	18	9	26
ENSMUSG00000038718	PBX3	4	21	8	14	14	15	57	67	38	53	50	52
ENSMUSG00000038722	BUD31	9	9	9	14	13	10	58	62	48	69	55	52
ENSMUSG00000038732	MBOAT1	3	4	2	2	2	6	11	11	10	19	20	13
ENSMUSG00000038733	WDR26	39	38	38	50	33	43	227	189	142	238	169	175
ENSMUSG00000038736	NUDCD1	7	4	8	12	7	6	46	40	32	37	42	37
ENSMUSG00000038740	MVB12B	8	8	5	6	8	2	30	33	25	29	23	30
ENSMUSG00000038742	ANGPTL6	0	2	1	2	0	1	3	0	2	5	3	3
ENSMUSG00000038759	NUP205	10	13	12	23	10	11	75	78	46	69	51	56
ENSMUSG00000038762	ABCF1	36	82	39	59	46	46	274	299	226	328	193	279
ENSMUSG00000038766	GABPB2	8	9	12	16	12	17	48	51	29	64	25	27
ENSMUSG00000038773	KDM3B	4	3	3	0	1	4	12	14	7	12	4	2
ENSMUSG00000038774	ASCC3	8	2	9	7	8	5	29	37	18	52	19	24
ENSMUSG00000038777	SEMA6C	0	1	0	0	0	1	1	3	3	1	3	0
ENSMUSG00000038780	SMURF1	1	2	0	2	0	1	4	4	4	6	4	2
ENSMUSG00000038781	STAP2	0	0	0	0	0	0	0	1	0	1	0	0
ENSMUSG00000038782	1700028J19R	0	0	1	1	0	0	0	1	0	1	0	0
ENSMUSG00000038784	CNOT4	16	26	25	19	15	21	100	92	48	103	66	74
ENSMUSG00000038797	ZSCAN2	0	0	0	0	0	0	1	1	1	2	1	1
ENSMUSG00000038803	OST4	8	12	6	10	8	5	63	45	30	39	35	34
ENSMUSG00000038806	SDE2	2	6	9	5	4	5	24	34	29	38	15	40
ENSMUSG00000038807	RAP1GAP2	0	1	1	0	1	0	1	1	0	1	3	2
ENSMUSG00000038812	TRMT112	1	5	2	9	6	5	14	33	19	22	27	31
ENSMUSG00000038816	CTNNAL1	4	9	7	4	7	7	24	19	17	28	24	18
ENSMUSG00000038822	HACE1	2	3	2	2	1	2	17	31	24	22	24	18
ENSMUSG00000038827	FAM206A	1	1	1	0	1	0	9	6	6	5	2	8
ENSMUSG00000038828	TMEM214	2	4	1	5	7	2	14	17	22	21	16	17
ENSMUSG00000038831	RALGPS1	0	4	0	4	1	1	10	7	6	9	7	9
ENSMUSG00000038836	AGBL3	1	1	2	2	1	1	12	8	9	8	6	3
ENSMUSG00000038838	VARS2	2	2	5	1	3	2	11	16	9	18	9	18
ENSMUSG00000038843	GCNT1	0	0	0	0	0	0	0	0	0	2	0	0
ENSMUSG00000038844	KIF16B	7	6	2	9	9	6	24	23	31	37	19	23
ENSMUSG00000038845	PHB	2	1	1	4	4	3	26	33	28	28	17	21
ENSMUSG00000038848	YTHDF1	11	8	10	13	8	7	66	55	40	66	43	35
ENSMUSG00000038855	ITPKB	2	10	10	10	11	5	37	38	25	34	24	36
ENSMUSG00000038859	BAIAP2L1	1	0	0	0	0	0	0	0	0	0	0	0
ENSMUSG00000038860	GARNL3	1	1	1	0	3	0	6	15	3	12	5	14

ENSMUSG00000038861	PI4KB	17	9	17	24	14	12	55	73	48	68	44	67
ENSMUSG00000038866	ZCCHC2	1	5	6	6	2	3	20	20	18	15	17	18
ENSMUSG00000038871	BPGM	5	4	1	3	6	6	15	23	14	25	19	18
ENSMUSG00000038876	RNF146	5	4	2	6	6	3	23	30	21	27	18	27
ENSMUSG00000038880	MRPS34	11	7	18	12	19	23	71	89	71	62	59	81
ENSMUSG00000038884	A230050P20I	1	1	6	5	2	4	3	1	1	3	2	2
ENSMUSG00000038886	MAN2A2	5	6	9	8	10	4	26	26	18	30	19	31
ENSMUSG00000038888	CTU1	1	2	0	0	1	0	2	12	4	6	4	7
ENSMUSG00000038893	FAM117A	1	6	3	2	2	0	19	10	4	13	8	13
ENSMUSG00000038895	ZFP653	0	3	2	1	1	3	4	6	6	6	3	6
ENSMUSG00000038900	RPL12	168	207	229	222	246	219	1327	1360	935	1306	992	1128
ENSMUSG00000038902	POGZ	17	18	12	16	18	18	80	74	67	75	44	81
ENSMUSG00000038903	CCDC68	0	0	0	0	0	0	0	0	0	0	0	1
ENSMUSG00000038909	KAT7	3	7	6	3	3	6	22	39	23	30	22	30
ENSMUSG00000038910	PLCL2	8	14	14	22	13	20	49	52	40	52	40	52
ENSMUSG00000038914	DIDO1	13	16	22	18	25	20	97	105	94	89	58	92
ENSMUSG00000038925	E330034G19I	1	0	0	0	0	0	0	0	0	0	0	0
ENSMUSG00000038930	RCCD1	2	2	6	4	6	6	19	21	10	23	17	13
ENSMUSG00000038936	SCCPDH	2	2	4	1	3	2	12	11	5	6	6	7
ENSMUSG00000038943	PRC1	9	15	10	19	16	17	73	76	61	91	52	58
ENSMUSG00000038949	CNST	3	3	1	3	1	4	10	11	11	14	15	10
ENSMUSG00000038954	SUPT3	0	4	4	4	3	2	19	22	13	18	11	17
ENSMUSG00000038957	EDC3	3	5	4	3	3	1	38	24	17	27	16	15
ENSMUSG00000038963	SLCO4A1	0	0	0	0	0	2	0	1	0	0	0	0
ENSMUSG00000038965	UBE2L3	15	15	11	22	23	24	94	86	73	85	61	89
ENSMUSG00000038970	LMTK2	2	5	6	3	5	6	12	19	6	7	11	8
ENSMUSG00000038975	RABGGTB	26	27	26	27	39	17	190	196	111	172	158	103
ENSMUSG00000038982	BLOC1S5	0	0	0	0	0	0	4	0	0	5	1	0
ENSMUSG00000038987	CFAP157	0	2	0	0	0	6	6	10	5	10	5	10
ENSMUSG00000038990	CABLES2	2	6	4	5	2	1	10	19	9	16	9	12
ENSMUSG00000038991	TXNDC5	10	13	10	10	5	12	51	55	42	43	33	61
ENSMUSG00000039000	UBE3C	6	12	5	6	6	2	39	47	23	38	26	28
ENSMUSG00000039001	RPS21	192	213	272	308	275	233	1564	1621	1268	1569	1256	1338
ENSMUSG00000039004	BMP6	0	0	0	0	0	0	0	1	2	1	1	1
ENSMUSG00000039007	CPQ	1	2	1	5	3	1	16	17	5	9	9	9
ENSMUSG00000039016	TIMM8B	25	17	13	24	27	16	124	127	86	126	101	119
ENSMUSG00000039018	MTG1	1	0	3	0	1	2	5	5	7	6	5	6
ENSMUSG00000039021	TTC16	0	1	1	1	0	0	3	6	2	4	6	8
ENSMUSG00000039031	ARHGAP18	4	2	8	12	11	2	19	25	27	38	32	21
ENSMUSG00000039033	TASP1	0	2	0	0	1	2	1	2	1	3	5	3
ENSMUSG00000039041	ADRM1	4	2	5	3	3	3	20	22	16	29	25	31
ENSMUSG00000039043	ARPIN	0	0	0	0	0	0	0	0	0	0	0	1
ENSMUSG00000039046	USP6NL	7	16	20	10	21	21	67	52	44	62	37	58
ENSMUSG00000039047	PIGK	4	12	12	9	6	10	44	53	39	43	36	24
ENSMUSG00000039048	FOXRED1	1	6	2	5	3	2	13	16	10	12	14	17
ENSMUSG00000039050	OSBPL2	3	6	6	5	3	2	33	23	17	33	21	16
ENSMUSG00000039055	EME1	4	3	5	13	5	4	21	26	14	30	29	22
ENSMUSG00000039058	AK5	0	0	0	0	0	0	2	0	0	1	0	0
ENSMUSG00000039063	ECHDC3	0	0	0	0	0	0	0	0	0	1	0	0
ENSMUSG00000039065	FAM173B	1	0	0	0	2	0	3	10	3	6	4	4
ENSMUSG00000039067	PSMD7	14	20	20	18	19	13	93	105	80	103	66	88
ENSMUSG00000039068	ZZZ3	9	9	8	15	11	15	49	59	29	63	39	42
ENSMUSG00000039069	MTG2	1	1	1	4	3	2	7	12	11	15	7	15
ENSMUSG00000039086	SS18L1	0	3	0	0	2	1	7	6	8	6	7	6
ENSMUSG00000039087	RREB1	2	2	3	4	1	4	11	12	12	12	19	18
ENSMUSG00000039089	L3MBTL3	7	2	3	10	9	4	21	24	25	24	26	18
ENSMUSG00000039092	SPTLC3	0	1	0	0	0	0	0	0	0	0	0	0
ENSMUSG00000039096	RSAD1	0	0	2	0	2	0	3	6	7	3	2	4

ENSMUSG00000039100	MARCH6	3	5	6	4	9	4	25	37	23	30	14	17
ENSMUSG00000039103	NEXN	1	1	0	0	0	1	1	3	3	2	1	1
ENSMUSG00000039105	ATP6V1G1	7	13	7	10	12	7	71	60	62	71	47	57
ENSMUSG00000039108	LSM14B	17	13	21	15	17	29	131	113	98	120	82	93
ENSMUSG00000039110	MYCBPAP	0	0	1	0	1	0	0	1	2	1	1	0
ENSMUSG00000039117	TAF4	12	8	9	14	10	8	73	77	60	82	60	37
ENSMUSG00000039126	PRUNE2	4	7	4	6	12	6	25	14	9	21	11	11
ENSMUSG00000039128	CDC123	23	28	25	28	28	27	148	160	116	149	123	125
ENSMUSG00000039130	ZC3HC1	14	11	7	5	10	14	49	68	49	64	49	45
ENSMUSG00000039131	GIPC2	0	0	0	1	0	0	0	0	0	0	0	0
ENSMUSG00000039133	9330171B17F	1	0	0	0	0	0	0	0	0	1	0	0
ENSMUSG00000039145	CAMK1D	4	7	3	3	9	3	37	27	20	30	21	32
ENSMUSG00000039148	SART1	22	23	23	26	18	15	96	96	69	96	77	82
ENSMUSG00000039153	RUNX2	0	0	0	1	3	1	3	2	1	4	1	1
ENSMUSG00000039154	SHD	0	1	1	0	0	0	1	1	1	2	0	2
ENSMUSG00000039156	STIM2	10	14	13	18	13	12	65	42	37	58	27	35
ENSMUSG00000039157	FAM102A	3	7	3	7	2	3	28	33	11	33	23	12
ENSMUSG00000039158	AKNA	2	4	7	6	8	2	24	19	26	22	21	26
ENSMUSG00000039159	UBE2H	9	15	16	16	12	14	88	59	55	74	56	74
ENSMUSG00000039163	CMC1	4	2	5	4	5	3	19	19	11	20	10	15
ENSMUSG00000039164	NAIF1	0	0	2	0	0	0	1	3	3	5	2	5
ENSMUSG00000039166	AKAP7	3	0	0	2	4	1	4	6	2	7	4	3
ENSMUSG00000039176	POLG	8	3	8	8	10	3	28	35	30	30	31	32
ENSMUSG00000039178	TBC1D19	1	3	1	5	2	1	7	12	12	9	7	8
ENSMUSG00000039182	AW209491	0	2	0	2	3	1	14	5	5	9	3	13
ENSMUSG00000039183	NUBP2	2	4	5	6	7	6	21	27	16	36	24	23
ENSMUSG00000039187	FANCI	2	5	1	5	2	4	18	15	17	17	12	13
ENSMUSG00000039191	RBPJ	4	9	11	2	2	4	24	22	17	30	17	25
ENSMUSG00000039193	NLRC4	1	0	0	0	0	0	1	1	0	0	1	1
ENSMUSG00000039195	1110008P14F	6	1	2	3	2	4	21	11	15	16	14	9
ENSMUSG00000039197	ADK	28	41	43	48	50	48	260	289	223	323	224	209
ENSMUSG00000039199	ZDHHC1	0	0	0	1	0	0	0	2	0	1	0	2
ENSMUSG00000039201	TBC1D25	0	2	1	0	1	0	8	2	2	12	4	6
ENSMUSG00000039202	ABHD2	21	20	27	33	22	13	81	88	71	91	76	68
ENSMUSG00000039205	CIZ1	7	10	10	6	12	3	49	53	43	54	48	38
ENSMUSG00000039206	DAGLB	6	21	5	9	8	4	36	37	23	22	23	26
ENSMUSG00000039208	METRNL	0	1	0	1	0	1	4	4	2	5	3	1
ENSMUSG00000039210	GPATCH2	1	3	1	1	1	4	0	1	1	0	0	2
ENSMUSG00000039217	IL18	0	0	0	0	0	0	1	1	1	1	0	0
ENSMUSG00000039218	SRRM2	348	493	455	494	491	417	2182	2376	1714	2260	1728	2113
ENSMUSG00000039219	ARID4B	30	35	31	38	26	40	113	163	99	157	111	127
ENSMUSG00000039220	PPP1R10	13	5	5	11	5	9	33	47	33	40	32	32
ENSMUSG00000039221	RPL22L1	19	27	11	16	24	21	121	127	85	129	100	82
ENSMUSG00000039230	TBCD	3	5	7	2	5	5	15	22	14	20	17	16
ENSMUSG00000039231	SUV39H1	1	6	9	6	4	11	33	39	34	34	20	32
ENSMUSG00000039232	STX11	0	0	0	0	0	0	2	1	2	1	0	2
ENSMUSG00000039233	TBCE	6	4	0	3	4	2	18	19	14	8	4	11
ENSMUSG00000039234	SEC24D	9	19	14	24	22	13	101	129	108	90	79	80
ENSMUSG00000039236	ISG20	0	0	0	0	0	0	0	2	1	2	0	4
ENSMUSG00000039239	TGFB2	0	0	0	0	0	0	0	1	1	1	0	1
ENSMUSG00000039242	B3GALNT2	5	1	2	1	3	2	21	15	19	10	14	23
ENSMUSG00000039244	E130309D02F	4	3	2	6	3	3	20	14	17	24	17	15
ENSMUSG00000039246	LYPLAL1	0	0	0	1	0	0	0	0	0	0	0	0
ENSMUSG00000039253	FN3KRP	3	3	3	5	5	2	33	18	20	20	8	17
ENSMUSG00000039254	POMT1	0	5	2	6	5	1	14	15	13	28	17	21
ENSMUSG00000039262	PRRC2B	13	15	13	20	22	15	84	89	60	66	57	67
ENSMUSG00000039263	NPEPL1	5	5	5	3	3	3	17	23	17	24	19	28
ENSMUSG00000039264	GIMAP3	0	0	1	0	0	0	0	0	0	0	0	0

ENSMUSG00000039275	FOXK2	5	4	3	13	5	7	31	30	24	28	17	30
ENSMUSG00000039278	PCSK1N	0	0	0	0	0	0	0	0	0	0	1	0
ENSMUSG00000039285	AZI2	5	7	6	5	5	2	33	32	21	42	20	24
ENSMUSG00000039286	FNDC3B	0	1	1	4	1	2	12	9	11	8	6	8
ENSMUSG00000039294	BC017643	3	3	4	6	2	5	22	24	18	22	23	13
ENSMUSG00000039298	CDK5RAP2	4	6	8	10	8	11	35	34	22	48	27	32
ENSMUSG00000039304	TNFSF10	0	0	3	0	1	2	5	7	7	9	3	5
ENSMUSG00000039307	HEXDC	1	3	2	3	4	1	5	10	5	10	10	13
ENSMUSG00000039308	NDST2	1	1	0	0	0	0	5	10	3	6	3	13
ENSMUSG00000039316	RFTN1	6	3	4	2	3	5	16	22	19	24	12	13
ENSMUSG00000039318	RAB3GAP2	3	7	2	10	6	10	15	19	19	23	21	23
ENSMUSG00000039328	RNF122	0	1	1	3	0	0	1	1	1	0	1	2
ENSMUSG00000039335	SPATA16	0	0	0	0	0	0	0	0	0	0	1	0
ENSMUSG00000039342	ANKAR	0	0	1	0	0	0	0	0	0	0	0	0
ENSMUSG00000039345	METTL22	0	2	2	3	1	0	6	9	1	5	5	7
ENSMUSG00000039354	SMARCAL1	10	6	10	11	7	8	38	40	32	31	19	34
ENSMUSG00000039356	EXOSC2	14	28	18	33	22	19	150	137	124	141	116	109
ENSMUSG00000039357	FUT11	4	6	7	5	7	7	22	18	13	24	18	26
ENSMUSG00000039361	PICALM	32	38	40	40	38	37	164	165	101	171	100	135
ENSMUSG00000039367	SEC24C	11	10	6	14	14	10	49	56	38	59	30	39
ENSMUSG00000039382	WDR45	4	3	3	2	3	2	11	12	13	14	6	16
ENSMUSG00000039384	DUSP10	14	9	7	9	8	15	55	67	39	64	48	53
ENSMUSG00000039385	CDH6	0	0	0	0	0	0	0	0	0	1	0	0
ENSMUSG00000039396	NEIL3	12	11	6	15	17	16	42	63	47	50	33	45
ENSMUSG00000039405	PRSS23	0	0	0	0	0	0	0	0	0	1	0	0
ENSMUSG00000039414	HEATR5B	3	1	1	1	0	1	7	4	1	11	4	7
ENSMUSG00000039419	CNTNAP2	1	0	0	3	1	0	9	6	4	12	3	7
ENSMUSG00000039427	ALG1	3	3	2	1	3	2	11	7	2	9	9	12
ENSMUSG00000039428	TMEM135	2	3	6	4	3	6	29	30	23	36	20	27
ENSMUSG00000039431	MTMR7	0	0	0	0	1	0	0	1	0	0	0	1
ENSMUSG00000039438	TTC36	0	1	1	0	0	0	0	1	1	0	0	0
ENSMUSG00000039449	PRPF18	2	11	8	9	7	4	38	57	29	52	28	34
ENSMUSG00000039452	SNX22	1	0	2	2	0	1	7	15	12	10	6	4
ENSMUSG00000039456	MORC3	9	18	10	12	9	11	101	93	54	87	48	59
ENSMUSG00000039458	MTMR12	2	0	5	6	2	3	14	27	17	23	8	11
ENSMUSG00000039461	TCTA	0	0	0	0	0	1	3	1	0	1	4	2
ENSMUSG00000039463	SLC9A8	2	5	5	8	5	6	12	17	15	11	11	9
ENSMUSG00000039470	ZDHHC2	11	10	14	9	13	6	25	35	28	41	22	38
ENSMUSG00000039473	UBN1	11	6	10	17	13	13	70	78	57	71	52	62
ENSMUSG00000039474	WFS1	0	1	0	1	0	1	1	4	1	4	0	4
ENSMUSG00000039477	TNRC18	2	5	4	4	3	2	33	19	20	29	12	20
ENSMUSG00000039478	MICU3	4	5	2	4	8	5	27	47	35	44	26	27
ENSMUSG00000039480	NT5DC1	1	2	2	5	3	2	11	21	12	24	13	13
ENSMUSG00000039483	ASB6	2	7	9	10	6	10	39	38	31	49	36	40
ENSMUSG00000039485	TSPYL4	1	3	2	2	2	1	10	16	10	13	5	11
ENSMUSG00000039492	CCDC27	0	0	0	0	0	0	0	0	0	0	1	0
ENSMUSG00000039496	CDNF	0	0	1	0	0	0	0	0	0	0	0	1
ENSMUSG00000039497	DSE	19	29	19	34	28	31	66	105	60	78	65	63
ENSMUSG00000039501	ZNFX1	3	0	0	0	2	1	3	1	4	6	1	3
ENSMUSG00000039509	NUP133	18	18	22	29	21	26	122	127	82	133	97	103
ENSMUSG00000039512	UHRF1BP1	5	1	3	1	2	2	10	11	5	6	5	10
ENSMUSG00000039515	PTPA	6	12	11	17	17	8	88	72	58	74	57	73
ENSMUSG00000039521	FOXP3	12	79	16	10	14	23	56	73	52	71	47	64
ENSMUSG00000039523	CEP104	2	2	2	2	1	4	12	10	8	8	10	8
ENSMUSG00000039530	TUSC3	2	1	5	3	3	5	27	13	19	33	11	20
ENSMUSG00000039531	ZUFSP	9	8	5	7	6	10	27	42	32	50	34	24
ENSMUSG00000039536	STAU1	5	3	5	8	5	3	26	24	30	31	18	33
ENSMUSG00000039545	FLICR	2	0	0	0	2	4	0	0	1	5	0	3

ENSMUSG00000039552	RSPH4A	0	0	0	0	0	0	1	0	0	0	0	0
ENSMUSG00000039556	PPP1R3F	1	0	1	0	0	0	4	4	2	2	0	5
ENSMUSG00000039563	2210406O10I	0	0	0	0	1	0	1	0	1	1	0	0
ENSMUSG00000039568	UBALD1	8	11	8	12	3	11	33	46	27	32	29	32
ENSMUSG00000039577	NPHP4	0	0	0	0	0	1	0	0	0	0	1	1
ENSMUSG00000039579	GRIN3A	0	1	0	0	0	0	1	0	0	0	0	0
ENSMUSG00000039585	MYO9A	0	4	4	5	6	2	21	25	16	25	13	15
ENSMUSG00000039599	FAM149B	1	2	2	0	1	2	7	8	5	13	4	5
ENSMUSG00000039611	TMEM246	0	0	0	0	0	0	0	1	0	0	0	0
ENSMUSG00000039615	STUB1	17	14	14	15	17	18	101	103	86	90	85	85
ENSMUSG00000039616	MOCOS	0	0	0	0	0	0	0	0	1	0	1	0
ENSMUSG00000039621	PREX1	10	6	10	10	11	11	57	42	28	48	29	43
ENSMUSG00000039623	AP5Z1	2	4	2	0	2	1	6	4	5	4	6	6
ENSMUSG00000039630	HNRNPU	158	228	200	242	228	276	1077	1031	831	1218	774	910
ENSMUSG00000039632	CCDC151	0	0	0	0	0	0	0	1	0	0	0	1
ENSMUSG00000039633	LONRF1	2	0	2	0	1	2	3	4	3	5	3	4
ENSMUSG00000039634	ZFP189	0	0	0	0	0	1	4	4	3	7	2	3
ENSMUSG00000039637	CORO7	14	22	20	22	30	21	95	108	64	99	83	87
ENSMUSG00000039639	KCNE1	0	0	0	0	0	0	0	0	0	1	0	0
ENSMUSG00000039640	MRPL12	8	14	19	18	20	18	109	109	84	92	92	101
ENSMUSG00000039646	VASN	0	0	0	0	0	2	3	1	0	0	0	1
ENSMUSG00000039648	KYAT1	3	2	0	1	1	1	8	10	8	14	5	6
ENSMUSG00000039652	CPEB3	3	3	6	4	3	1	22	20	13	19	12	10
ENSMUSG00000039653	BAAT	0	0	0	0	0	0	0	0	0	0	0	1
ENSMUSG00000039656	RXRB	8	7	7	17	10	6	37	43	32	43	26	41
ENSMUSG00000039660	SPOUT1	8	1	7	8	5	1	37	39	29	39	23	31
ENSMUSG00000039662	ICMT	3	4	0	5	3	2	14	18	13	13	8	9
ENSMUSG00000039670	OXLD1	0	1	2	0	2	0	2	3	2	1	2	3
ENSMUSG00000039671	ZMYND8	16	12	19	16	19	13	86	56	52	78	54	68
ENSMUSG00000039678	TBC1D13	5	3	4	7	3	4	17	18	19	23	5	18
ENSMUSG00000039680	MRPS6	6	6	7	5	3	8	26	24	21	29	22	24
ENSMUSG00000039682	LAP3	8	8	12	15	13	16	80	100	71	87	66	83
ENSMUSG00000039686	ZER1	2	2	1	1	2	2	13	23	10	10	8	12
ENSMUSG00000039697	NCOA7	34	29	16	39	28	28	190	226	124	247	175	166
ENSMUSG00000039699	BATF2	0	0	0	0	1	0	0	0	0	0	0	0
ENSMUSG00000039701	USP53	5	6	5	8	6	6	26	28	14	26	12	19
ENSMUSG00000039703	NPLOC4	2	8	3	6	4	4	38	27	25	34	28	24
ENSMUSG00000039704	LMBRD2	4	2	3	4	2	2	11	12	10	23	10	9
ENSMUSG00000039713	PLEKHG5	0	0	0	0	0	1	0	0	0	0	1	0
ENSMUSG00000039714	CPLX3	0	0	0	0	0	0	1	0	0	0	0	0
ENSMUSG00000039715	WDR34	1	1	2	4	3	1	14	14	14	17	13	15
ENSMUSG00000039716	DOCK3	0	0	1	0	0	0	0	1	0	1	1	1
ENSMUSG00000039720	GOT1L1	0	0	0	0	0	1	0	0	0	0	0	0
ENSMUSG00000039725	TRP53RKA	1	1	0	1	4	1	5	7	6	6	4	5
ENSMUSG00000039737	PRKRIP1	7	17	14	12	13	16	63	74	56	70	56	63
ENSMUSG00000039738	SLX4	3	1	4	2	1	2	17	14	11	12	14	12
ENSMUSG00000039740	ALG2	0	1	2	3	3	5	23	27	16	33	18	18
ENSMUSG00000039745	HTATIP2	1	0	3	1	2	2	5	12	12	17	12	15
ENSMUSG00000039747	ORAI2	7	7	10	12	18	14	44	41	44	52	44	54
ENSMUSG00000039748	EXO1	0	0	6	7	7	4	48	44	21	42	23	24
ENSMUSG00000039753	FBXL5	6	4	7	9	7	3	46	39	20	42	29	30
ENSMUSG00000039754	ALKBH4	1	3	5	2	7	4	33	26	22	28	28	34
ENSMUSG00000039756	DNTTIP2	43	39	33	41	42	25	210	207	140	249	162	154
ENSMUSG00000039759	THAP3	2	3	2	3	2	1	15	14	15	15	14	9
ENSMUSG00000039763	DNAJC28	0	1	1	1	0	1	2	1	1	4	1	5
ENSMUSG00000039768	DNAJC11	6	4	7	5	9	10	39	30	29	33	20	33
ENSMUSG00000039770	YPEL5	6	3	3	3	2	1	16	19	18	22	16	18
ENSMUSG00000039771	POLR2J	12	12	12	13	8	18	80	87	79	85	65	78

ENSMUSG00000039774	GALNT12	4	7	9	2	8	4	28	46	35	51	22	28
ENSMUSG00000039781	CEP131	0	0	1	0	2	1	5	4	2	3	3	3
ENSMUSG00000039782	CPEB2	1	0	1	2	0	1	2	0	1	2	2	1
ENSMUSG00000039787	CERCAM	14	12	9	12	11	5	53	60	50	63	36	55
ENSMUSG00000039789	ZFP597	3	3	5	3	4	5	13	9	10	20	16	11
ENSMUSG00000039795	ZFAND1	0	1	2	2	1	1	1	5	6	4	5	6
ENSMUSG00000039798	2600006K01F	0	0	0	0	0	0	0	0	2	0	1	2
ENSMUSG00000039801	2410089E03F	8	6	8	9	10	5	21	29	14	36	25	20
ENSMUSG00000039804	NCOA5	0	2	1	5	4	1	14	12	4	12	13	15
ENSMUSG00000039810	ZC3H10	0	3	3	2	8	6	13	20	15	18	10	12
ENSMUSG00000039814	XKR5	1	0	0	0	0	0	1	2	2	0	2	1
ENSMUSG00000039824	MYL6B	1	3	0	1	0	0	2	0	4	3	0	1
ENSMUSG00000039826	TRUB2	3	2	9	2	8	3	13	17	10	15	13	19
ENSMUSG00000039828	WDR70	8	4	8	12	13	8	39	38	23	35	22	28
ENSMUSG00000039834	ZFP335	2	2	7	1	6	3	9	11	4	13	4	8
ENSMUSG00000039835	NHSL1	3	7	5	2	7	2	14	8	12	12	10	8
ENSMUSG00000039840	EPG5	2	2	1	3	2	2	9	5	9	9	4	10
ENSMUSG00000039841	ZFP800	12	19	21	25	19	22	110	87	70	136	68	82
ENSMUSG00000039842	MCPH1	3	6	2	8	5	4	21	12	20	27	19	17
ENSMUSG00000039844	RAPGEF1	17	29	28	34	20	19	145	164	108	131	122	140
ENSMUSG00000039849	PCIF1	4	5	3	2	2	6	22	21	16	14	14	32
ENSMUSG00000039850	ENDOV	3	1	0	1	2	3	10	11	3	11	6	6
ENSMUSG00000039852	RERE	15	20	26	34	19	19	90	80	82	121	92	73
ENSMUSG00000039853	TRIM14	0	0	0	0	1	1	0	3	2	2	1	2
ENSMUSG00000039860	SRRM3	0	0	0	1	0	0	0	0	0	0	0	0
ENSMUSG00000039873	NEURL2	0	0	0	0	0	0	1	1	1	1	3	1
ENSMUSG00000039878	SLC39A5	0	0	0	0	0	0	0	3	0	1	2	1
ENSMUSG00000039879	HECA	6	8	10	5	5	13	41	41	33	35	24	29
ENSMUSG00000039883	LRRC17	0	0	0	1	0	0	0	0	0	0	0	0
ENSMUSG00000039886	TMEM120A	1	2	2	2	1	0	6	10	11	4	8	6
ENSMUSG00000039887	ALG14	2	0	0	2	3	2	20	15	6	15	5	13
ENSMUSG00000039891	TXLNB	0	0	1	0	0	0	2	1	1	2	1	0
ENSMUSG00000039901	9130011E15F	0	0	1	0	0	0	4	2	6	4	2	3
ENSMUSG00000039908	SLC26A11	10	11	11	14	14	10	60	60	46	65	48	51
ENSMUSG00000039910	CITED2	16	26	24	24	14	16	145	140	116	161	106	119
ENSMUSG00000039914	COQ10A	0	0	0	0	1	0	0	1	1	1	1	1
ENSMUSG00000039917	RHBDD2	1	2	3	2	3	1	8	7	10	6	6	9
ENSMUSG00000039929	URB1	7	6	7	5	6	8	26	37	22	32	30	28
ENSMUSG00000039934	GSAP	0	0	0	0	0	0	0	0	0	0	1	0
ENSMUSG00000039936	PIK3CD	13	24	28	27	19	22	99	117	80	111	63	97
ENSMUSG00000039942	PTGER4	6	4	6	4	4	5	22	21	16	37	15	10
ENSMUSG00000039943	PLCB4	0	0	0	0	0	0	0	0	1	0	0	0
ENSMUSG00000039952	DAG1	11	1	3	4	4	2	26	22	15	28	25	22
ENSMUSG00000039953	CLSTN1	0	0	1	0	0	0	0	1	0	0	0	0
ENSMUSG00000039958	ETFBKMT	2	2	2	5	1	2	16	21	20	18	17	11
ENSMUSG00000039959	HIP1	4	2	8	4	5	3	22	23	11	16	19	9
ENSMUSG00000039963	CCDC40	0	0	0	0	0	1	1	0	0	0	0	0
ENSMUSG00000039967	ZFP292	35	31	27	47	39	42	174	170	136	182	142	142
ENSMUSG00000039968	RSBN1L	21	32	30	35	31	92	100	110	86	200	81	86
ENSMUSG00000039976	TBC1D16	1	0	4	2	2	5	17	8	5	10	6	6
ENSMUSG00000039977	DEUP1	0	0	0	0	0	0	0	0	1	0	0	0
ENSMUSG00000039981	ZC3H12D	0	0	0	0	0	1	0	1	0	0	0	1
ENSMUSG00000039982	DTX4	0	0	0	0	0	0	0	1	0	0	0	0
ENSMUSG00000039983	CCDC32	5	6	4	2	3	1	21	38	20	26	11	23
ENSMUSG00000039985	FAM60A	1	6	0	5	3	3	12	20	7	16	8	3
ENSMUSG00000039987	PHTF2	4	5	10	10	4	11	22	32	22	27	19	19
ENSMUSG00000039988	ANKRD13C	13	30	21	27	27	26	84	118	84	145	94	74
ENSMUSG00000039989	CBX4	5	8	10	15	15	11	47	29	39	53	27	35

ENSMUSG00000039990	EDRF1	2	4	2	7	6	0	21	18	12	25	16	8
ENSMUSG00000039994	TIMELESS	9	8	19	21	15	11	56	95	72	78	63	62
ENSMUSG00000039997	IFI203	3	6	3	3	6	7	25	21	16	34	14	10
ENSMUSG00000040003	MAGI2	0	0	0	0	0	0	2	0	0	0	1	3
ENSMUSG00000040006	GINM1	2	5	6	10	4	5	18	58	22	29	19	26
ENSMUSG00000040007	BAHD1	10	6	6	6	9	6	25	42	32	38	24	43
ENSMUSG00000040009	GNAZ	0	0	3	0	0	1	0	1	0	0	0	0
ENSMUSG00000040010	SLC7A5	16	30	25	36	20	34	110	132	87	122	81	98
ENSMUSG00000040016	PTGER3	1	0	0	0	0	0	0	0	0	0	0	0
ENSMUSG00000040018	COX15	6	4	5	3	6	3	25	25	21	23	20	15
ENSMUSG00000040021	LATS1	7	8	9	3	7	4	28	30	18	25	26	24
ENSMUSG00000040022	RAB11FIP2	0	1	1	3	0	3	11	11	12	11	4	8
ENSMUSG00000040025	YTHDF2	45	36	45	52	39	65	205	183	135	212	119	146
ENSMUSG00000040026	SAA3	0	0	0	0	0	0	0	0	1	0	0	0
ENSMUSG00000040028	ELAVL1	10	31	26	18	20	25	84	70	74	120	63	66
ENSMUSG00000040029	IPO8	8	15	10	11	12	10	59	61	64	69	48	49
ENSMUSG00000040033	STAT2	3	3	1	4	0	9	19	30	13	16	16	18
ENSMUSG00000040034	NUP43	4	6	8	6	5	3	25	21	27	30	24	18
ENSMUSG00000040037	NEGR1	0	0	0	0	0	0	0	0	0	0	2	0
ENSMUSG00000040040	IFT88	1	3	2	3	1	1	11	8	17	10	8	3
ENSMUSG00000040043	RBMS2	0	0	2	2	1	3	9	3	4	6	3	4
ENSMUSG00000040044	ORC3	3	6	5	9	6	10	33	34	34	42	21	32
ENSMUSG00000040048	NDUFB10	35	48	34	52	45	32	203	216	150	192	193	207
ENSMUSG00000040054	BAZ2A	21	16	18	22	26	26	105	117	82	100	100	85
ENSMUSG00000040061	PLCB2	0	0	0	1	0	0	0	0	0	0	0	0
ENSMUSG00000040078	PTGES3-PS	0	0	0	0	0	0	1	0	0	1	2	0
ENSMUSG00000040084	BUB1B	18	19	9	31	19	15	110	125	86	109	104	101
ENSMUSG00000040093	BMF	4	5	1	3	6	2	30	25	24	33	19	27
ENSMUSG00000040097	FLYWCH1	5	5	8	5	11	5	27	24	19	28	23	32
ENSMUSG00000040102	KLHL42	3	4	2	4	3	3	14	25	14	23	17	23
ENSMUSG00000040105	PLPP6	2	1	4	5	2	1	15	18	15	21	9	9
ENSMUSG00000040111	GRAMD1B	3	5	6	1	4	5	34	40	28	35	20	35
ENSMUSG00000040112	MRPS35	6	8	8	11	8	2	35	34	24	34	30	38
ENSMUSG00000040121	REP15	0	0	0	0	0	0	0	1	0	0	0	0
ENSMUSG00000040123	ZMYM5	13	20	23	21	21	23	100	128	82	135	73	72
ENSMUSG00000040124	GORAB	0	1	0	2	1	2	15	4	7	9	8	5
ENSMUSG00000040127	SDR9C7	0	0	0	0	0	0	0	0	0	0	1	0
ENSMUSG00000040128	PNRC1	7	7	9	8	10	8	29	35	22	33	33	36
ENSMUSG00000040139	9430038I01R	5	7	0	4	3	6	21	23	18	22	20	24
ENSMUSG00000040151	HS2ST1	8	8	13	10	11	1	35	38	38	45	28	47
ENSMUSG00000040158	TAX1BP3	0	0	1	0	0	0	0	0	0	0	0	0
ENSMUSG00000040167	IKZF5	6	11	4	13	8	11	57	63	62	80	49	53
ENSMUSG00000040174	ALKBH3	1	1	1	2	3	1	9	8	7	7	6	7
ENSMUSG00000040177	2310057M21	5	3	3	2	5	4	10	28	24	25	14	16
ENSMUSG00000040187	ARNTL2	0	2	1	2	2	1	9	8	7	8	8	10
ENSMUSG00000040188	SCAMP2	2	3	1	1	3	1	16	7	10	10	13	12
ENSMUSG00000040195	NEMP1	6	5	4	2	7	9	27	24	18	26	19	18
ENSMUSG00000040204	PCLAF	56	67	77	76	72	70	399	376	284	391	249	318
ENSMUSG00000040205	CUZD1	0	0	0	0	0	0	0	0	0	0	2	0
ENSMUSG00000040209	ZFP704	15	24	21	13	22	15	54	58	40	72	47	51
ENSMUSG00000040212	EMP3	33	37	29	38	34	30	209	230	192	214	147	177
ENSMUSG00000040213	KYAT3	2	2	6	4	3	5	21	21	14	26	12	15
ENSMUSG00000040220	GAS8	1	3	1	2	3	1	17	16	14	12	10	23
ENSMUSG00000040225	PRRC2C	63	95	83	115	109	108	464	468	346	514	316	375
ENSMUSG00000040229	GPR34	9	9	12	9	11	7	41	45	32	59	35	35
ENSMUSG00000040231	SYNGR4	0	0	0	0	0	0	1	1	0	0	0	1
ENSMUSG00000040234	TM7SF3	6	2	7	3	2	3	15	11	9	21	8	14
ENSMUSG00000040236	TRAPPC5	2	4	4	6	2	6	31	40	13	28	12	29

ENSMUSG00000040242	FGFR1OP2	27	44	31	34	30	36	210	256	210	263	175	161
ENSMUSG00000040247	TBC1D10C	6	13	10	10	6	10	51	45	45	36	29	39
ENSMUSG00000040249	LRP1	0	0	0	0	1	0	0	0	0	0	0	0
ENSMUSG00000040250	INTS13	6	11	8	8	7	6	54	53	29	40	31	35
ENSMUSG00000040253	GBP7	5	3	8	6	6	6	24	25	19	29	27	27
ENSMUSG00000040254	SEMA3D	0	0	0	0	0	0	0	0	0	0	1	0
ENSMUSG00000040263	KLHDC4	2	4	3	5	4	9	43	47	34	31	40	27
ENSMUSG00000040264	GBP2B	0	0	0	0	0	0	1	1	0	0	0	0
ENSMUSG00000040265	DNM3	0	0	1	0	1	0	2	0	1	0	0	2
ENSMUSG00000040268	PLEKHA1	5	0	4	3	2	2	22	21	13	14	10	11
ENSMUSG00000040269	MRPS28	19	20	18	17	14	16	113	103	63	96	72	81
ENSMUSG00000040270	BACH2	39	43	45	55	48	56	207	160	130	216	119	145
ENSMUSG00000040272	ACCS	3	0	2	0	1	2	3	4	3	5	3	1
ENSMUSG00000040274	CDK6	0	0	0	0	2	0	0	0	0	0	0	0
ENSMUSG00000040280	NDUFA4L2	0	0	0	0	0	2	1	0	3	1	1	2
ENSMUSG00000040282	BC052040	2	2	4	1	5	2	21	15	8	11	5	8
ENSMUSG00000040283	BTNL9	0	0	0	0	0	0	0	0	0	1	0	0
ENSMUSG00000040287	STAC3	1	0	1	0	1	0	0	1	1	1	1	0
ENSMUSG00000040289	HEY1	0	0	0	0	0	0	1	0	0	0	0	1
ENSMUSG00000040296	DDX58	0	1	1	1	1	4	3	8	5	7	11	8
ENSMUSG00000040297	SUCO	26	27	26	30	26	18	153	160	121	179	122	117
ENSMUSG00000040302	RBM48	0	1	3	4	7	5	15	30	18	20	28	23
ENSMUSG00000040312	CCHCR1	7	10	4	11	6	4	25	21	24	16	22	20
ENSMUSG00000040321	ZFP770	8	8	6	6	11	10	39	29	21	40	20	21
ENSMUSG00000040322	SLC25A24	4	13	2	9	10	15	52	44	37	57	32	34
ENSMUSG00000040325	DCAF1	5	9	5	6	7	8	27	42	37	46	27	37
ENSMUSG00000040327	CUL9	0	0	0	0	0	0	0	0	0	2	1	1
ENSMUSG00000040329	IL7	0	0	1	0	0	1	0	0	1	0	1	1
ENSMUSG00000040331	NSMCE4A	12	13	15	16	25	19	117	138	101	112	77	114
ENSMUSG00000040339	FAM102B	11	14	8	8	6	8	60	51	63	69	37	47
ENSMUSG00000040345	ARHGAP9	12	15	16	17	18	16	78	79	71	75	64	76
ENSMUSG00000040351	ANKIB1	3	6	7	7	9	7	32	22	30	31	25	26
ENSMUSG00000040354	MARS	20	17	13	15	15	11	77	80	63	93	63	74
ENSMUSG00000040356	SKIV2L	11	15	20	20	19	17	78	84	60	61	69	72
ENSMUSG00000040359	UFL1	6	7	8	6	10	13	42	37	33	62	38	32
ENSMUSG00000040363	BCOR	4	2	5	4	8	4	30	30	26	25	28	17
ENSMUSG00000040365	TRIM41	1	4	3	2	2	0	12	9	7	14	7	9
ENSMUSG00000040367	LRRD1	0	0	0	0	0	1	1	1	0	0	0	1
ENSMUSG00000040370	ETFRF1	1	6	5	1	3	2	18	19	12	13	13	10
ENSMUSG00000040372	GPR63	0	0	0	0	0	1	0	0	0	0	1	0
ENSMUSG00000040374	PEX2	2	3	5	6	4	5	25	31	26	25	20	12
ENSMUSG00000040380	CBLN3	1	0	0	0	0	0	0	0	0	0	0	0
ENSMUSG00000040383	AQR	14	18	12	10	11	9	60	68	46	71	55	49
ENSMUSG00000040385	PPP1CA	23	62	42	38	59	53	239	289	218	248	212	264
ENSMUSG00000040387	KLHL32	0	0	2	2	1	1	3	3	1	6	3	0
ENSMUSG00000040389	WDR47	1	1	0	2	0	2	6	8	5	4	5	7
ENSMUSG00000040390	MAP3K10	3	3	4	2	1	2	20	21	11	16	18	10
ENSMUSG00000040396	ABHD13	2	5	5	3	8	3	14	21	15	19	15	11
ENSMUSG00000040407	AKAP9	21	26	34	34	33	23	121	117	83	133	103	97
ENSMUSG00000040410	FBXL4	0	1	0	1	0	0	1	2	0	1	1	1
ENSMUSG00000040412	5330417C22F	0	0	0	1	0	0	0	0	0	2	0	0
ENSMUSG00000040413	TIMD2	0	0	0	0	0	0	0	0	0	1	0	0
ENSMUSG00000040414	SLC25A28	4	1	4	3	10	4	26	30	18	25	25	24
ENSMUSG00000040415	DTX3	7	4	10	8	8	6	33	24	26	16	21	20
ENSMUSG00000040420	CDH18	0	0	0	0	0	0	1	0	0	0	0	0
ENSMUSG00000040423	RC3H1	6	8	6	13	11	9	59	67	41	64	34	42
ENSMUSG00000040424	HIPK4	0	0	0	0	0	0	0	1	0	0	1	0
ENSMUSG00000040428	PLEKHA4	0	0	0	0	0	0	1	0	0	0	0	0

ENSMUSG00000040429	MTERF1A	2	0	0	3	1	0	4	4	3	4	4	3
ENSMUSG00000040430	PITPNC1	34	56	32	30	41	41	86	85	77	95	66	79
ENSMUSG00000040433	ZBTB38	14	20	12	19	17	18	61	53	53	60	34	47
ENSMUSG00000040434	LARGE2	0	0	1	0	0	0	0	0	0	0	0	0
ENSMUSG00000040435	PPP1R15A	3	4	6	2	3	3	19	22	19	22	22	19
ENSMUSG00000040441	SLC26A10	0	0	0	0	0	0	3	4	2	3	4	1
ENSMUSG00000040446	RPRD1A	5	0	2	3	7	3	18	17	15	29	10	11
ENSMUSG00000040447	SPNS2	0	0	1	0	1	0	0	1	0	2	1	0
ENSMUSG00000040451	SGMS1	2	4	1	7	3	3	29	23	25	25	16	20
ENSMUSG00000040452	CDH12	0	1	1	1	2	0	3	7	2	4	5	5
ENSMUSG00000040455	USP45	6	9	6	8	6	9	26	43	17	52	33	31
ENSMUSG00000040459	ARGLU1	44	96	70	99	81	85	578	601	422	542	381	465
ENSMUSG00000040462	OS9	3	6	4	8	4	5	24	29	19	25	24	33
ENSMUSG00000040463	MYBBP1A	59	78	61	91	60	59	338	369	281	344	270	328
ENSMUSG00000040466	BLVRB	1	0	3	3	1	1	3	9	3	7	5	4
ENSMUSG00000040472	RABGGTA	5	2	0	1	7	0	9	10	4	7	11	10
ENSMUSG00000040479	DGKZ	17	19	26	22	20	14	82	108	65	91	72	104
ENSMUSG00000040481	BPTF	49	66	58	74	55	63	217	195	145	246	138	178
ENSMUSG00000040482	DXO	4	1	1	3	2	0	10	17	9	21	14	17
ENSMUSG00000040483	XAF1	0	0	0	0	1	0	0	2	1	0	0	1
ENSMUSG00000040488	LTBP4	0	0	1	0	0	0	0	0	0	0	0	0
ENSMUSG00000040495	CHRM4	0	0	0	0	0	0	0	0	1	0	0	0
ENSMUSG00000040502	MARCH9	2	2	2	0	3	2	6	11	10	5	6	2
ENSMUSG00000040506	AMBRA1	2	1	5	3	1	8	17	12	15	18	17	15
ENSMUSG00000040511	PVR	0	2	0	2	1	0	7	3	5	5	1	9
ENSMUSG00000040520	MANEA	6	4	5	3	1	3	15	22	13	21	19	10
ENSMUSG00000040521	TSFM	17	11	17	18	17	15	71	91	79	78	80	73
ENSMUSG00000040524	ZFP609	0	2	0	1	0	0	0	0	0	1	0	0
ENSMUSG00000040528	MILR1	3	2	0	1	2	2	8	23	6	12	7	18
ENSMUSG00000040532	ABHD11	3	4	11	9	16	1	34	35	28	30	27	25
ENSMUSG00000040537	ADAM22	0	0	0	1	0	0	0	1	0	3	0	0
ENSMUSG00000040548	TEX2	2	2	0	5	2	1	6	3	8	7	2	10
ENSMUSG00000040549	CKAP5	17	18	12	28	21	23	84	77	71	112	58	86
ENSMUSG00000040550	OTUD6B	5	7	10	8	9	12	67	49	38	74	40	45
ENSMUSG00000040552	C3AR1	1	0	0	0	0	0	0	0	0	0	0	1
ENSMUSG00000040560	WDR7	1	1	1	0	2	0	11	2	7	8	5	5
ENSMUSG00000040564	APOC1	0	0	0	0	0	0	0	0	0	0	0	1
ENSMUSG00000040565	BTAF1	4	9	8	6	5	9	25	36	33	47	28	28
ENSMUSG00000040570	RUNDC3B	2	3	2	0	2	3	10	6	4	5	4	4
ENSMUSG00000040583	CYP2B13	0	0	0	0	2	0	0	1	0	0	0	2
ENSMUSG00000040584	ABCB1A	0	4	0	0	4	2	11	6	1	8	8	6
ENSMUSG00000040586	OFD1	4	4	3	4	7	3	18	35	18	28	19	13
ENSMUSG00000040591	1110051M20	1	4	3	0	0	1	11	17	11	15	11	12
ENSMUSG00000040592	CD79B	0	0	0	0	0	0	0	0	0	0	1	0
ENSMUSG00000040594	RANBP17	0	3	1	3	2	1	7	8	3	6	4	7
ENSMUSG00000040596	POGK	0	1	0	0	2	1	3	2	3	4	3	2
ENSMUSG00000040599	MIS12	4	12	9	6	4	9	35	20	15	30	12	24
ENSMUSG00000040610	TLX3	0	0	0	0	0	1	0	0	0	0	0	0
ENSMUSG00000040613	APOBEC1	1	0	2	2	0	1	5	6	6	12	2	9
ENSMUSG00000040618	PCK2	11	21	30	22	18	18	86	97	89	87	63	77
ENSMUSG00000040620	DHX33	2	1	4	2	6	4	15	21	5	12	18	18
ENSMUSG00000040621	GEMIN8	2	0	1	1	0	0	5	15	4	5	8	6
ENSMUSG00000040624	PLEKHG1	0	0	4	1	1	0	4	5	3	4	3	5
ENSMUSG00000040631	DOK4	2	3	2	1	3	2	8	8	8	5	5	4
ENSMUSG00000040640	ERC2	0	0	0	0	0	0	1	0	0	0	0	0
ENSMUSG00000040648	PPIP5K2	6	11	8	9	7	12	42	61	36	65	43	62
ENSMUSG00000040651	FAM208A	17	14	21	23	23	23	98	98	60	103	59	67
ENSMUSG00000040652	OAZ2	2	0	2	1	2	1	10	8	6	10	10	12

ENSMUSG00000040659	EFHD2	5	11	10	9	12	15	88	84	50	75	76	84
ENSMUSG00000040661	RAD54L2	10	11	8	6	3	9	36	36	30	28	22	31
ENSMUSG00000040666	SH3BGR	3	3	5	3	6	3	22	17	14	19	13	14
ENSMUSG00000040667	NUP88	10	29	23	26	28	27	136	158	109	168	121	129
ENSMUSG00000040669	PHC1	0	1	0	2	1	0	8	8	5	7	9	4
ENSMUSG00000040675	MTHFD1L	4	6	7	8	4	6	37	26	29	37	29	28
ENSMUSG00000040681	HMGN1	34	45	48	65	68	54	303	327	231	335	214	261
ENSMUSG00000040687	MADD	7	5	14	8	13	8	34	47	26	31	20	37
ENSMUSG00000040688	TBL3	9	13	5	18	17	18	39	50	46	50	45	65
ENSMUSG00000040697	DNAJC16	3	2	2	1	4	2	5	15	8	10	5	4
ENSMUSG00000040699	LIMD2	26	42	34	39	49	30	188	221	132	190	142	152
ENSMUSG00000040701	AP1G2	1	5	4	5	1	3	14	17	17	20	15	12
ENSMUSG00000040710	ST8SIA4	4	11	9	10	8	8	25	25	24	30	12	15
ENSMUSG00000040711	SH3PXD2B	0	0	0	0	0	0	0	1	0	0	0	0
ENSMUSG00000040712	CAMTA2	3	1	3	3	1	2	18	13	13	16	10	10
ENSMUSG00000040713	CREG1	3	4	6	4	5	4	26	39	28	41	22	29
ENSMUSG00000040714	KLC3	1	0	1	2	1	0	13	15	5	15	9	11
ENSMUSG00000040717	IL17RD	1	1	3	2	0	1	12	13	11	10	5	9
ENSMUSG00000040720	VIRMA	5	10	11	3	10	9	38	48	39	45	40	47
ENSMUSG00000040721	ZFHX2	3	1	2	3	6	4	11	14	8	10	7	11
ENSMUSG00000040723	RCSD1	16	20	20	11	18	12	78	74	55	106	61	70
ENSMUSG00000040724	KCNA2	0	0	0	0	0	0	0	1	0	0	0	0
ENSMUSG00000040725	HNRNPUL1	68	89	78	63	94	72	497	489	354	454	303	468
ENSMUSG00000040726	HESX1	0	1	0	0	2	1	1	3	0	4	2	2
ENSMUSG00000040728	ESRP1	0	0	0	0	0	0	0	1	0	0	0	0
ENSMUSG00000040731	EIF4H	21	19	32	25	32	32	210	245	148	202	137	186
ENSMUSG00000040734	PPP1R13L	0	1	1	0	1	1	0	0	2	2	1	0
ENSMUSG00000040738	INTS8	8	7	16	18	9	19	75	69	64	73	56	56
ENSMUSG00000040746	RNF167	10	11	12	5	12	5	38	55	19	60	35	38
ENSMUSG00000040747	CD53	82	87	84	82	89	92	503	555	361	507	337	451
ENSMUSG00000040749	SIAH1B	1	0	1	0	1	0	7	10	5	10	5	9
ENSMUSG00000040751	LAT2	1	1	0	1	3	2	2	12	10	9	5	7
ENSMUSG00000040760	APPL1	12	14	8	16	18	17	56	60	36	75	48	63
ENSMUSG00000040761	SPEN	22	16	15	14	17	23	66	62	45	80	47	74
ENSMUSG00000040767	SNRNP25	7	8	5	5	10	3	21	33	26	41	34	26
ENSMUSG00000040771	OARD1	3	1	4	6	5	4	34	25	23	34	18	21
ENSMUSG00000040774	CEPT1	11	10	6	7	10	11	66	58	62	64	45	44
ENSMUSG00000040782	COP1	3	10	9	10	4	8	59	47	42	45	31	38
ENSMUSG00000040785	TTC3	56	69	72	109	94	76	303	282	173	344	222	241
ENSMUSG00000040795	IQCC	2	3	3	5	6	3	10	13	10	15	14	14
ENSMUSG00000040811	EML2	3	4	5	10	9	3	19	40	19	33	16	27
ENSMUSG00000040813	TEX264	3	5	3	4	2	1	25	25	11	17	16	15
ENSMUSG00000040818	DENND6A	2	2	3	3	1	5	17	12	14	11	11	13
ENSMUSG00000040820	HLCS	1	0	2	2	2	0	6	1	1	8	7	4
ENSMUSG00000040822	1700123O20I	5	8	6	3	3	5	38	38	30	46	36	41
ENSMUSG00000040824	SNRPD2	21	28	15	20	23	27	163	162	129	180	145	158
ENSMUSG00000040828	CATSPERD	0	0	0	0	0	0	2	0	2	1	0	0
ENSMUSG00000040838	GM11639	0	0	0	1	1	0	1	0	0	0	0	0
ENSMUSG00000040840	4930579G18I	0	1	2	2	1	1	6	8	5	8	2	5
ENSMUSG00000040842	SZRD1	16	14	21	28	22	7	68	69	51	75	63	82
ENSMUSG00000040843	TIPRL	3	13	11	10	15	9	56	61	58	85	43	41
ENSMUSG00000040848	SFT2D2	2	7	5	7	3	4	17	22	8	23	15	10
ENSMUSG00000040850	PSME4	10	12	7	6	10	12	48	64	38	50	34	48
ENSMUSG00000040852	PLEKHH2	0	0	0	0	0	0	0	0	0	1	0	0
ENSMUSG00000040857	ERF	1	1	0	3	1	2	2	14	11	7	5	7
ENSMUSG00000040859	BSDC1	10	5	5	4	11	4	42	35	31	45	29	33
ENSMUSG00000040860	CROCC	0	0	0	0	1	2	2	3	3	3	2	2
ENSMUSG00000040865	INO80D	5	15	14	19	22	16	78	77	45	75	33	42

ENSMUSG00000040866	RSPH6A	0	0	0	0	0	1	0	0	0	0	0	0
ENSMUSG00000040877	WDR25	0	0	0	0	0	0	0	0	1	2	2	1
ENSMUSG00000040883	TMEM205	1	0	0	0	0	0	0	0	0	0	0	0
ENSMUSG00000040888	GFER	12	10	14	8	8	10	71	60	33	41	35	35
ENSMUSG00000040899	CCR6	0	0	0	0	0	0	0	0	0	1	0	0
ENSMUSG00000040913	FBXW4	3	1	1	0	6	4	9	7	8	11	4	13
ENSMUSG00000040918	SLC19A2	5	1	1	2	0	2	15	13	5	12	5	8
ENSMUSG00000040928	S100PBP	3	7	5	4	9	5	34	27	29	34	23	19
ENSMUSG00000040929	RFX3	9	7	6	9	2	5	31	24	19	26	19	26
ENSMUSG00000040936	ULK4	4	17	0	9	7	5	51	28	24	35	26	28
ENSMUSG00000040940	ARHGEF1	17	27	19	26	18	21	55	72	54	71	58	93
ENSMUSG00000040943	TET2	4	9	4	9	10	8	30	46	18	34	28	41
ENSMUSG00000040945	RCC2	28	28	29	32	31	25	162	153	109	147	102	132
ENSMUSG00000040952	RPS19	590	708	721	869	808	770	2050	2099	1631	2030	1596	1989
ENSMUSG00000040957	CABLES1	0	0	0	0	0	0	1	4	2	0	2	2	0
ENSMUSG00000040964	ARHGEF10L	21	19	20	18	23	17	50	85	46	81	57	67
ENSMUSG00000040966	SLC22A2	0	0	0	0	0	0	0	0	1	0	0	0
ENSMUSG00000040987	MILL2	0	0	0	0	0	0	0	0	0	1	0	0
ENSMUSG00000040990	SH3KBP1	119	123	134	170	157	133	599	731	526	720	535	586
ENSMUSG00000040997	ABHD4	0	0	0	0	1	0	1	3	5	1	3	2
ENSMUSG00000040998	NPNT	0	0	1	1	0	0	0	0	1	0	0	0
ENSMUSG00000041000	TRIM62	0	0	1	0	0	0	0	0	0	1	0	0
ENSMUSG00000041009	IQCF4	0	0	0	0	1	0	0	0	0	0	0	0
ENSMUSG00000041012	CMTM8	0	0	0	0	0	0	0	1	0	0	0	0
ENSMUSG00000041020	MAP7D2	0	0	0	0	0	0	0	2	0	0	0	0
ENSMUSG00000041025	IFFO2	0	0	0	2	0	0	4	2	2	1	2	1
ENSMUSG00000041028	GHITM	15	25	16	14	29	20	140	147	72	141	101	121
ENSMUSG00000041035	GM17018	0	1	2	3	0	2	9	19	13	19	12	10
ENSMUSG00000041037	IRGQ	1	0	2	0	1	2	7	5	5	3	5	2
ENSMUSG00000041040	FAM117B	6	11	11	5	5	7	37	37	26	52	28	29
ENSMUSG00000041057	WDR43	39	55	60	53	54	46	247	280	191	309	194	215
ENSMUSG00000041058	WWP1	4	7	11	13	3	12	28	26	21	29	17	18
ENSMUSG00000041064	PIF1	0	4	2	0	2	4	5	9	12	10	10	15
ENSMUSG00000041075	FZD7	2	3	1	2	3	2	7	4	7	3	4	2
ENSMUSG00000041079	RWDD2B	2	0	1	1	1	2	6	7	2	4	4	5
ENSMUSG00000041084	OSTC	21	32	18	38	28	23	171	152	127	188	136	150
ENSMUSG00000041096	TSPYL2	3	8	2	9	3	4	32	28	20	39	17	20
ENSMUSG00000041112	ELMO1	9	23	14	17	22	23	76	80	50	101	59	66
ENSMUSG00000041115	IQSEC2	0	1	0	0	0	0	0	2	4	3	0	1
ENSMUSG00000041124	MSANTD4	0	4	3	3	5	4	11	20	18	23	13	9
ENSMUSG00000041126	H2AFV	14	22	18	23	21	27	129	138	88	122	118	95
ENSMUSG00000041130	ZFP598	13	16	12	27	19	18	90	101	80	95	67	78
ENSMUSG00000041132	N4BP2L1	3	5	8	12	13	10	40	56	23	38	26	33
ENSMUSG00000041133	SMC1A	26	35	34	67	53	39	158	177	150	210	168	158
ENSMUSG00000041134	CYYR1	0	0	0	0	0	0	1	0	0	0	0	0
ENSMUSG00000041135	RIPK2	0	1	0	1	7	3	10	9	11	23	10	9
ENSMUSG00000041138	NME8	0	0	0	0	0	0	0	0	0	1	0	0
ENSMUSG00000041143	TMCO4	0	2	0	3	0	2	8	7	4	6	3	2
ENSMUSG00000041147	BRCA2	9	13	20	11	18	22	67	78	42	97	52	55
ENSMUSG00000041153	OSGIN2	2	3	1	7	6	2	24	15	19	20	15	12
ENSMUSG00000041161	OTUD3	1	0	0	2	3	0	2	4	1	9	2	3
ENSMUSG00000041164	ZMIZ2	5	4	6	1	13	8	30	25	18	38	30	26
ENSMUSG00000041168	LONP1	31	15	32	27	33	29	158	154	110	147	146	148
ENSMUSG00000041180	HECTD2	1	3	3	1	1	0	0	2	3	6	1	1
ENSMUSG00000041187	PRKD2	6	10	9	12	7	11	46	57	37	43	45	40
ENSMUSG00000041189	CHRNB1	0	0	2	2	0	0	1	0	0	0	0	0
ENSMUSG00000041199	RPUSD1	1	2	3	2	0	1	8	6	11	8	8	9
ENSMUSG00000041203	TRIR	6	9	11	7	7	6	43	61	46	56	35	45

ENSMUSG00000041215	YEATS2	14	8	15	21	22	19	80	83	66	92	60	63
ENSMUSG00000041216	CLVS1	0	0	0	0	0	0	0	0	1	0	0	0
ENSMUSG00000041219	ARHGAP11A	14	11	9	13	11	9	62	67	60	69	40	56
ENSMUSG00000041220	ELOVL6	34	42	34	47	50	47	172	153	131	202	139	124
ENSMUSG00000041225	ARHGAP12	6	9	2	7	11	2	36	36	28	42	34	34
ENSMUSG00000041229	PHF8	1	4	1	7	1	2	20	19	12	26	17	15
ENSMUSG00000041231	UBLCP1	1	2	0	1	2	1	2	4	3	3	5	3
ENSMUSG00000041235	CHD7	122	190	201	220	189	178	661	691	526	798	509	593
ENSMUSG00000041236	VPS41	4	2	4	5	3	4	24	19	14	22	17	16
ENSMUSG00000041237	PKLR	0	0	0	0	0	0	0	0	0	0	2	0	1
ENSMUSG00000041238	RBBP8	23	28	26	42	31	25	164	163	107	161	124	115
ENSMUSG00000041241	MUL1	1	4	2	1	2	1	3	6	12	6	8	9
ENSMUSG00000041245	WNK3	0	0	0	0	0	1	0	0	0	0	0	0
ENSMUSG00000041258	ZFP236	2	7	2	7	2	3	12	22	18	25	18	16
ENSMUSG00000041263	RUSC1	5	7	6	6	9	0	25	22	16	24	22	20
ENSMUSG00000041264	USPL1	12	14	13	11	7	13	52	59	66	61	52	53
ENSMUSG00000041268	DMXL2	1	2	2	0	3	5	8	4	3	11	6	7
ENSMUSG00000041272	TOX	5	5	4	10	3	6	34	27	23	32	26	26
ENSMUSG00000041278	TTC1	1	3	4	6	10	6	26	24	10	27	17	22
ENSMUSG00000041287	SOX15	0	1	1	0	1	0	2	2	0	1	0	2
ENSMUSG00000041297	CDK13	12	9	14	19	6	21	65	68	58	79	47	64
ENSMUSG00000041298	KATNAL1	2	4	2	2	2	0	7	8	7	12	6	6
ENSMUSG00000041303	GTF3C3	6	10	5	9	13	8	63	58	37	63	49	44
ENSMUSG00000041308	SNTB2	10	13	7	14	4	8	65	57	51	54	37	59
ENSMUSG00000041313	SLC7A1	23	26	34	29	24	25	141	123	99	146	104	120
ENSMUSG00000041319	THOC6	2	2	3	3	1	3	27	19	17	22	12	17
ENSMUSG00000041323	AK7	0	0	0	0	0	0	0	0	1	0	0	0
ENSMUSG00000041324	INHBA	0	0	0	0	1	0	0	1	0	0	0	0
ENSMUSG00000041328	PCF11	13	23	21	21	20	23	88	96	66	102	63	88
ENSMUSG00000041341	ATG2B	5	3	5	4	4	0	9	22	12	21	11	17
ENSMUSG00000041343	ANKRD42	0	0	0	1	0	0	1	0	0	0	0	0
ENSMUSG00000041346	WRAP53	1	0	5	1	1	0	13	9	8	8	9	13
ENSMUSG00000041347	BDKRB1	0	0	0	1	0	0	0	0	0	0	0	0
ENSMUSG00000041353	TMEM29	2	3	5	2	5	4	28	32	19	35	19	27
ENSMUSG00000041354	RGL2	6	6	4	14	15	9	33	53	27	45	30	36
ENSMUSG00000041355	SSR2	41	56	55	61	61	53	315	286	222	293	248	251
ENSMUSG00000041360	PUM3	10	10	15	11	10	16	86	96	73	123	76	88
ENSMUSG00000041361	MYZAP	0	0	0	1	0	0	0	0	0	0	0	0
ENSMUSG00000041362	SHTN1	1	1	5	3	4	5	22	10	12	17	11	11
ENSMUSG00000041375	CCDC9	3	9	3	5	2	6	17	22	14	18	20	27
ENSMUSG00000041396	METTL18	2	2	0	2	2	4	9	9	7	5	5	5
ENSMUSG00000041406	BC055324	1	7	1	2	6	6	16	21	11	23	18	25
ENSMUSG00000041408	WAPL	20	26	28	24	39	39	133	118	106	154	87	109
ENSMUSG00000041415	DICER1	12	15	14	13	16	12	57	58	40	59	45	37
ENSMUSG00000041417	PIK3R1	17	22	23	31	26	29	126	117	76	150	86	98
ENSMUSG00000041420	MEIS3	0	2	0	2	3	1	0	8	5	4	1	0
ENSMUSG00000041426	HIBCH	4	1	1	1	3	2	17	12	14	16	6	19
ENSMUSG00000041429	NTHL1	3	0	1	2	1	0	7	4	2	7	3	5
ENSMUSG00000041431	CCNB1	5	15	2	11	11	3	65	87	72	85	58	56
ENSMUSG00000041438	UTP4	7	15	8	9	12	13	50	71	41	67	52	47
ENSMUSG00000041439	MFSD6	20	27	15	22	25	21	47	51	35	60	33	44
ENSMUSG00000041440	GK5	0	0	0	1	0	0	0	0	0	0	0	0
ENSMUSG00000041453	RPL21	132	188	179	221	204	198	861	888	680	929	642	822
ENSMUSG00000041459	TARDBP	13	23	20	20	21	16	165	205	128	180	156	143
ENSMUSG00000041471	FAM35A	0	1	3	2	1	2	4	9	2	7	3	4
ENSMUSG00000041477	DCP1B	1	0	0	3	1	2	2	5	2	2	1	3
ENSMUSG00000041483	ZFP281	6	15	14	15	13	7	47	39	28	44	38	26
ENSMUSG00000041488	STX3	1	0	0	0	1	0	1	0	0	0	1	1

ENSMUSG00000041491	CEP78	0	0	0	5	6	3	15	8	14	26	4	16
ENSMUSG00000041498	KIF14	3	3	2	5	4	3	15	28	14	29	16	15
ENSMUSG00000041506	RRP9	14	5	4	8	5	5	30	39	27	33	28	30
ENSMUSG00000041528	RNF123	3	5	5	6	1	3	18	17	12	16	15	14
ENSMUSG00000041530	AGO1	5	4	1	7	2	3	36	34	27	43	31	30
ENSMUSG00000041540	SOX5	0	0	0	0	0	0	0	0	0	0	0	1
ENSMUSG00000041556	FBXO2	0	0	0	0	0	0	0	0	0	0	1	0
ENSMUSG00000041560	NOP53	25	18	25	27	26	25	96	100	114	125	114	106
ENSMUSG00000041570	CAMSAP2	3	3	0	0	3	2	6	4	7	7	9	8
ENSMUSG00000041571	SELENOW	3	3	3	8	4	4	26	25	15	21	15	23
ENSMUSG00000041578	CRX	0	0	0	0	0	0	1	0	0	0	0	0
ENSMUSG00000041594	TMTC4	2	2	0	3	0	1	9	8	6	8	6	7
ENSMUSG00000041607	MBP	3	7	4	2	1	4	19	26	10	27	21	20
ENSMUSG00000041609	BICDL1	1	0	1	0	0	1	1	1	1	1	2	4
ENSMUSG00000041617	CCDC74A	0	1	0	0	0	1	0	3	0	1	1	2
ENSMUSG00000041623	D11WSU47E	3	1	0	2	5	2	6	7	3	2	8	7
ENSMUSG00000041625	GGACT	20	21	30	27	31	25	187	178	146	185	126	151
ENSMUSG00000041629	FAM104A	6	7	11	7	11	5	32	40	23	38	22	23
ENSMUSG00000041632	MRPS27	2	2	3	3	3	1	13	14	15	17	16	11
ENSMUSG00000041633	KCTD12B	0	0	0	0	0	0	0	0	0	1	0	0
ENSMUSG00000041638	GCN1L1	1	0	3	1	1	1	16	17	11	10	11	8
ENSMUSG00000041642	KIF21B	6	6	15	14	9	5	54	51	35	62	46	35
ENSMUSG00000041645	DDX24	10	18	12	25	25	19	80	85	89	106	74	81
ENSMUSG00000041649	KLF8	1	1	0	0	0	1	4	3	5	7	3	2
ENSMUSG00000041650	PCCA	66	50	56	55	45	64	199	229	190	214	157	190
ENSMUSG00000041658	RRAGB	0	0	0	0	1	0	1	4	1	2	2	0
ENSMUSG00000041670	RIMS1	0	0	1	0	0	0	0	0	0	0	0	0
ENSMUSG00000041671	PYROXD1	0	3	4	3	1	1	11	14	6	11	4	7
ENSMUSG00000041673	LRRC18	0	0	0	0	0	0	1	0	0	0	0	0
ENSMUSG00000041679	LRRC29	0	0	0	0	1	1	2	0	2	0	0	1
ENSMUSG00000041684	BIVM	0	0	0	0	1	0	0	0	0	0	0	0
ENSMUSG00000041685	FCHO2	5	7	5	11	11	13	44	41	39	67	53	38
ENSMUSG00000041697	COX6A1	47	39	48	81	59	45	365	380	311	408	280	332
ENSMUSG00000041700	LHFPL1	2	2	1	0	1	0	3	7	4	5	5	7
ENSMUSG00000041702	BTBD7	5	12	8	9	6	11	38	51	38	47	41	36
ENSMUSG00000041703	ZIC5	7	16	10	21	22	12	64	81	50	84	41	56
ENSMUSG00000041712	UBR7	6	9	7	5	9	9	45	33	30	39	25	37
ENSMUSG00000041716	GM20604	1	2	1	2	0	4	14	5	9	14	5	13
ENSMUSG00000041718	ALG13	7	7	4	5	7	5	21	29	27	27	30	27
ENSMUSG00000041720	PI4KA	6	11	12	5	12	7	58	52	32	40	52	53
ENSMUSG00000041733	COQ5	1	5	4	2	1	2	26	36	17	29	15	28
ENSMUSG00000041734	KIRREL	0	0	0	0	0	0	0	0	0	0	0	1
ENSMUSG00000041736	TSPO	4	7	8	9	6	13	63	70	49	44	29	54
ENSMUSG00000041740	RNF10	25	30	24	21	21	27	111	132	96	145	82	119
ENSMUSG00000041747	UTP15	6	4	10	8	6	11	39	26	26	35	18	26
ENSMUSG00000041757	PLEKHA6	0	0	0	0	0	0	0	1	0	0	1	0
ENSMUSG00000041762	GPR155	2	3	1	1	2	3	14	13	12	15	12	8
ENSMUSG00000041763	TPP2	36	51	53	41	56	44	261	317	222	298	208	225
ENSMUSG00000041765	UBAC2	6	8	4	10	7	7	39	58	44	46	42	52
ENSMUSG00000041769	PPP2R2D	4	2	6	5	11	8	28	35	26	52	41	27
ENSMUSG00000041773	ENC1	7	16	15	14	10	7	59	104	54	82	64	61
ENSMUSG00000041774	YDJC	5	2	0	5	3	4	26	23	21	22	17	22
ENSMUSG00000041775	MAPK1IP1	1	2	1	2	3	1	14	8	5	16	10	6
ENSMUSG00000041777	CIR1	6	4	9	6	2	8	18	17	19	24	18	21
ENSMUSG00000041779	TRAM2	2	5	1	2	1	4	9	6	5	11	8	7
ENSMUSG00000041781	CPSF2	11	11	13	13	15	15	84	80	58	122	72	79
ENSMUSG00000041782	LAD1	0	0	0	0	0	0	0	2	0	0	0	1
ENSMUSG00000041801	PHLDA3	0	0	1	0	0	0	0	0	0	0	0	0

ENSMUSG00000041815	POLDIP3	3	8	5	5	5	4	27	32	19	28	19	23
ENSMUSG00000041817	FAM169A	1	0	0	0	0	0	1	1	3	2	0	1
ENSMUSG00000041831	SYTL3	1	2	2	0	1	1	22	24	11	13	16	20
ENSMUSG00000041836	PTPRE	1	1	1	3	3	1	4	5	2	1	10	3
ENSMUSG00000041837	PDCD7	3	5	3	3	5	7	20	18	27	34	16	16
ENSMUSG00000041840	HAUS1	1	2	5	4	5	1	12	19	12	15	16	12
ENSMUSG00000041841	RPL37	167	211	182	222	205	220	900	887	717	875	780	913
ENSMUSG00000041842	FHDC1	0	1	1	1	0	1	2	4	2	5	2	0
ENSMUSG00000041846	PPP4R3A	28	27	30	41	36	24	189	161	142	244	125	156
ENSMUSG00000041849	CARD6	1	0	3	4	6	3	20	15	14	20	13	17
ENSMUSG00000041852	TCF20	40	32	31	42	35	32	186	179	121	176	121	142
ENSMUSG00000041859	MCM3	23	37	38	34	37	31	255	278	201	256	182	203
ENSMUSG00000041870	ANKRD13A	7	3	9	7	6	6	39	51	37	41	37	50
ENSMUSG00000041872	IL17F	1	1	0	0	0	1	0	1	0	2	2	1
ENSMUSG00000041879	IPO9	10	28	18	22	21	16	95	94	90	86	68	87
ENSMUSG00000041881	NDUFA7	33	38	33	39	35	29	239	197	175	201	186	217
ENSMUSG00000041886	MACC1	0	1	0	0	0	0	0	0	0	0	1	0
ENSMUSG00000041890	GIT2	7	10	8	16	10	9	55	50	38	64	31	46
ENSMUSG00000041891	LMAN1	10	16	14	21	21	21	68	82	57	93	70	63
ENSMUSG00000041895	WIPI1	0	0	0	0	0	0	0	0	1	0	0	0
ENSMUSG00000041907	GPR45	0	0	1	1	0	0	1	0	0	0	0	0
ENSMUSG00000041911	DLX1	2	5	4	3	10	6	23	22	14	34	25	20
ENSMUSG00000041912	TDRKH	0	0	0	0	0	0	0	0	1	0	0	0
ENSMUSG00000041915	AMMECR1L	5	5	5	4	7	4	14	20	16	20	12	18
ENSMUSG00000041920	SLC16A6	11	6	7	8	16	4	25	26	9	20	17	25
ENSMUSG00000041921	METAP1D	13	10	14	11	11	11	54	60	42	71	49	47
ENSMUSG00000041926	RNPEP	8	14	10	11	6	14	53	63	40	52	44	52
ENSMUSG00000041930	FAM222A	0	0	0	0	0	0	2	1	0	5	1	1
ENSMUSG00000041935	AW549877	3	5	0	5	4	5	26	20	13	22	12	13
ENSMUSG00000041936	AGRN	0	0	0	0	0	1	0	0	0	0	0	0
ENSMUSG00000041939	MVK	1	0	1	2	2	3	13	6	12	14	10	8
ENSMUSG00000041949	TANGO6	1	4	0	0	0	0	7	7	2	7	3	5
ENSMUSG00000041954	TNFRSF18	0	1	0	0	0	0	0	0	0	0	0	1
ENSMUSG00000041958	PIGS	1	3	2	2	5	0	25	22	19	12	16	17
ENSMUSG00000041959	S100A10	26	52	47	52	45	50	329	282	223	279	247	282
ENSMUSG00000041961	ZNRF3	14	23	20	28	16	27	82	88	71	91	54	76
ENSMUSG00000041966	DCAF17	5	4	7	4	3	11	23	23	23	28	24	16
ENSMUSG00000041974	SPIDR	1	2	2	3	2	3	18	19	2	18	14	9
ENSMUSG00000041975	METTL8	1	5	5	2	5	2	11	11	10	22	10	18
ENSMUSG00000041977	ARHGEF11	2	2	1	1	0	2	8	10	9	11	10	5
ENSMUSG00000041992	RAPGEF5	0	0	0	0	0	0	0	0	0	0	1	0
ENSMUSG00000041995	ZBED3	6	2	5	2	2	1	18	17	12	17	13	12
ENSMUSG00000041997	TLK1	40	33	36	43	44	34	220	182	155	227	139	179
ENSMUSG00000042010	ACACB	0	0	0	0	0	0	0	1	0	1	1	1
ENSMUSG00000042015	WDR41	2	2	0	3	3	3	11	14	9	16	16	11
ENSMUSG00000042029	NCAPG2	13	19	20	19	23	17	69	73	69	99	56	73
ENSMUSG00000042032	MAT2B	3	9	7	4	6	7	64	65	29	69	33	56
ENSMUSG00000042035	IGSF3	0	1	0	0	1	0	0	0	0	0	1	0
ENSMUSG00000042042	CSGALNACT2	7	4	10	10	12	7	14	17	13	16	10	13
ENSMUSG00000042043	TBCA	39	56	38	42	50	41	352	307	210	292	211	245
ENSMUSG00000042046	DSTYK	1	1	0	1	1	1	0	1	1	0	1	1
ENSMUSG00000042050	WDR60	23	19	29	33	39	33	102	113	75	125	100	105
ENSMUSG00000042055	WDR11	0	1	2	3	4	1	11	6	9	6	4	4
ENSMUSG00000042063	ZFP386	9	11	6	12	5	6	38	41	41	43	30	37
ENSMUSG00000042064	MYO3B	0	0	0	1	0	1	0	2	0	0	0	1
ENSMUSG00000042073	ABHD14B	0	1	0	1	1	1	12	8	8	5	6	2
ENSMUSG00000042078	SVOP	0	0	0	0	0	1	0	0	0	0	0	0
ENSMUSG00000042079	HNRNPF	11	15	18	19	15	15	145	119	101	143	101	123

ENSMUSG00000042082	ARSB	3	1	1	1	1	3	9	7	13	5	9	5
ENSMUSG00000042087	4933440N22I	1	0	0	0	1	2	6	4	2	2	3	4
ENSMUSG00000042097	ZFP239	0	1	0	5	5	5	10	11	7	16	5	11
ENSMUSG00000042104	UGGT2	0	0	1	0	1	1	1	2	0	4	2	0
ENSMUSG00000042105	INPP5F	4	6	2	11	7	6	21	20	12	33	15	18
ENSMUSG00000042106	FAM212A	1	0	0	0	0	0	1	0	0	0	0	0
ENSMUSG00000042109	CSDC2	0	0	0	0	0	0	0	0	0	1	0	0
ENSMUSG00000042111	CCDC115	2	4	6	9	6	6	41	43	29	45	29	22
ENSMUSG00000042116	VWA1	0	0	0	1	0	0	0	0	0	0	0	0
ENSMUSG00000042121	SSH1	5	2	4	5	4	4	8	18	11	14	12	14
ENSMUSG00000042133	PPIG	64	78	100	113	103	89	493	516	338	467	361	380
ENSMUSG00000042138	MSANTD2	5	7	9	4	7	11	38	38	23	46	28	32
ENSMUSG00000042148	COX10	5	4	6	4	9	8	23	22	12	27	17	23
ENSMUSG00000042155	KLHL23	1	0	2	2	1	2	1	4	1	1	3	2
ENSMUSG00000042156	DZIP1	5	11	12	14	14	14	65	56	37	61	33	49
ENSMUSG00000042165	GM9774	5	6	7	4	7	6	25	15	15	17	15	17
ENSMUSG00000042167	PAPD4	5	7	8	7	10	9	75	57	37	51	32	38
ENSMUSG00000042178	ARMC5	1	0	3	1	1	2	5	5	8	9	5	12
ENSMUSG00000042182	BEND6	0	1	0	1	0	0	3	2	2	2	2	1
ENSMUSG00000042185	NFRKB	4	2	3	5	7	3	34	39	23	23	28	31
ENSMUSG00000042195	SLC35F2	3	5	2	1	7	3	16	19	21	17	19	15
ENSMUSG00000042197	ZFP451	19	21	20	29	20	25	95	116	83	139	82	95
ENSMUSG00000042198	CHCHD7	5	2	3	4	3	1	16	23	20	25	20	21
ENSMUSG00000042200	CDRT4	0	0	0	0	0	0	0	0	0	1	0	0
ENSMUSG00000042202	SLC35E2	4	8	10	6	6	7	35	28	30	30	18	24
ENSMUSG00000042203	TBC1D22B	1	2	5	2	4	4	22	17	16	20	16	17
ENSMUSG00000042207	KDM5B	18	16	17	14	14	8	44	69	43	52	44	43
ENSMUSG00000042208	0610010F05F	5	8	6	6	8	9	32	33	19	35	27	16
ENSMUSG00000042210	ABHD14A	2	4	0	8	4	4	16	16	19	18	13	17
ENSMUSG00000042211	FBXO38	5	6	2	4	7	5	24	20	12	29	18	21
ENSMUSG00000042213	ZFAND4	1	2	3	1	0	1	10	6	5	13	7	7
ENSMUSG00000042215	BAG2	7	3	7	7	8	4	54	41	23	40	19	27
ENSMUSG00000042225	AMMECR1	8	4	5	9	5	11	17	22	17	27	13	22
ENSMUSG00000042229	RABIF	3	0	1	2	3	2	19	13	12	8	12	18
ENSMUSG00000042254	CILP	0	0	1	0	0	0	0	0	0	0	0	0
ENSMUSG00000042258	ISL1	0	0	1	1	0	0	0	0	0	0	0	0
ENSMUSG00000042262	CCR8	0	2	1	2	1	3	8	11	9	7	6	10
ENSMUSG00000042271	NXT2	1	1	0	0	1	2	4	5	3	2	4	5
ENSMUSG00000042275	PELO	0	0	1	0	0	0	0	0	0	0	0	0
ENSMUSG00000042284	ITGA1	5	10	2	9	6	6	16	22	20	25	10	21
ENSMUSG00000042286	STAB1	0	2	1	0	1	2	5	5	2	2	2	4
ENSMUSG00000042292	MKL1	4	6	5	6	4	5	14	26	21	23	27	25
ENSMUSG00000042293	GM5617	1	0	1	1	2	0	7	2	5	7	4	4
ENSMUSG00000042298	TTC19	1	2	1	6	3	3	14	13	11	14	5	9
ENSMUSG00000042302	EHBP1	2	1	0	2	3	3	7	7	2	4	2	6
ENSMUSG00000042303	SGSM3	0	2	1	1	3	3	10	10	7	11	10	10
ENSMUSG00000042305	TMEM183A	10	7	16	13	18	6	62	76	46	63	44	43
ENSMUSG00000042308	SETD1A	4	6	5	12	7	9	50	44	33	47	26	40
ENSMUSG00000042312	S100A13	7	9	14	10	11	8	61	75	45	64	55	52
ENSMUSG00000042320	PROX2	0	1	0	2	3	0	2	7	2	8	6	3
ENSMUSG00000042323	PBRM1	9	16	15	38	17	16	110	81	76	109	78	78
ENSMUSG00000042328	HPS4	1	0	1	0	0	2	4	4	1	10	12	8
ENSMUSG00000042331	SPECC1	0	0	0	0	1	0	0	1	2	1	0	0
ENSMUSG00000042333	TNFRSF14	0	2	0	0	0	0	0	1	0	0	0	0
ENSMUSG00000042340	CTF1	0	0	0	1	0	0	0	0	0	0	0	0
ENSMUSG00000042345	UBASH3A	0	2	0	1	1	0	3	5	2	5	5	3
ENSMUSG00000042348	ARL15	0	2	0	2	1	1	4	7	4	5	4	2
ENSMUSG00000042349	IKBKE	0	1	2	3	1	1	5	11	8	4	6	5

ENSMUSG00000042350	AREL1	4	1	0	3	2	2	14	10	6	15	8	18
ENSMUSG00000042351	GRAP2	6	11	11	31	9	14	70	73	54	98	54	60
ENSMUSG00000042354	GNL3	63	89	66	85	117	93	416	502	336	507	391	374
ENSMUSG00000042359	OSBPL6	0	2	0	0	0	0	1	2	2	1	0	1
ENSMUSG00000042363	LGALSL	1	3	0	1	1	4	6	12	4	14	4	4
ENSMUSG00000042364	SNX18	12	18	8	20	6	8	38	16	27	32	28	37
ENSMUSG00000042369	RBM45	37	65	48	71	50	50	287	262	227	299	218	254
ENSMUSG00000042380	SMIM12	2	7	10	6	7	9	35	35	26	35	29	27
ENSMUSG00000042389	TSEN2	0	1	0	2	3	0	11	12	5	5	7	8
ENSMUSG00000042390	GATAD2B	35	47	51	43	36	39	176	195	159	195	110	159
ENSMUSG00000042396	RBM7	9	8	10	9	12	9	58	71	44	61	33	49
ENSMUSG00000042401	CRTAC1	0	0	0	0	0	0	0	1	0	0	0	0
ENSMUSG00000042404	DENND4B	2	0	5	3	2	2	17	19	3	27	13	12
ENSMUSG00000042406	ATF4	88	116	110	105	130	111	603	580	485	576	404	492
ENSMUSG00000042408	ZMYM6	0	1	2	2	3	2	13	8	7	12	7	6
ENSMUSG00000042410	AGPS	15	18	22	15	22	15	81	112	82	107	86	82
ENSMUSG00000042414	PRDM14	0	0	0	0	0	1	0	0	0	0	0	0
ENSMUSG00000042417	CCNO	0	0	0	0	0	0	2	2	0	0	0	0
ENSMUSG00000042419	NFKBIL1	3	6	6	7	7	10	36	28	19	28	24	25
ENSMUSG00000042423	FBRS	4	10	8	10	12	6	45	54	43	43	30	36
ENSMUSG00000042426	DHX29	12	11	8	12	9	9	58	58	35	64	39	39
ENSMUSG00000042428	MGAT3	0	0	0	0	0	0	3	0	0	0	0	1
ENSMUSG00000042429	ADORA1	0	0	0	0	0	1	0	0	0	0	0	0
ENSMUSG00000042444	MINDY2	6	9	3	8	7	3	35	40	32	38	26	23
ENSMUSG00000042446	ZMYM4	5	10	7	8	10	7	44	34	34	62	28	43
ENSMUSG00000042447	MIOS	2	5	6	3	5	3	17	19	16	19	16	10
ENSMUSG00000042460	C1GALT1	20	26	18	25	20	24	64	66	43	61	37	52
ENSMUSG00000042462	DCTPP1	15	21	23	22	24	21	145	125	111	164	134	142
ENSMUSG00000042472	ZFP410	2	3	3	4	6	7	37	26	23	35	17	27
ENSMUSG00000042473	TBC1D8B	1	2	2	4	1	4	6	11	6	8	8	3
ENSMUSG00000042476	ABCB4	0	1	0	0	0	0	0	2	2	1	1	0
ENSMUSG00000042477	TFAP2E	0	0	0	0	0	0	0	0	0	0	1	0
ENSMUSG00000042485	MUSTN1	0	0	0	0	0	0	0	1	1	1	0	3
ENSMUSG00000042487	LEO1	5	10	5	12	16	12	59	53	29	48	45	52
ENSMUSG00000042489	CLSPN	17	21	18	33	24	19	113	99	64	119	93	80
ENSMUSG00000042492	TBC1D10B	2	2	2	2	5	2	17	17	17	12	11	17
ENSMUSG00000042496	PRDM10	3	5	2	6	9	2	25	20	13	14	21	19
ENSMUSG00000042500	AGO4	0	1	0	0	0	1	0	0	0	0	0	0
ENSMUSG00000042502	CD2BP2	4	7	9	7	8	8	40	37	28	56	32	36
ENSMUSG00000042505	SDHAF3	0	1	1	3	2	0	5	6	6	11	12	5
ENSMUSG00000042506	USP22	3	3	6	6	7	0	19	20	19	26	11	21
ENSMUSG00000042507	ELMSAN1	1	2	5	6	4	3	23	24	23	31	16	29
ENSMUSG00000042508	DMTF1	65	52	60	66	68	61	261	315	227	268	212	207
ENSMUSG00000042510	AA986860	0	0	0	0	0	0	0	1	0	2	0	1
ENSMUSG00000042520	UBAP2L	44	44	51	48	56	41	204	228	172	217	161	190
ENSMUSG00000042523	DNAL1	0	1	1	1	3	2	5	7	2	8	8	5
ENSMUSG00000042524	SUN2	10	13	14	14	15	12	53	52	33	60	30	50
ENSMUSG00000042535	GTPBP1	1	4	3	1	2	5	18	16	12	22	23	20
ENSMUSG00000042541	SEM1	55	50	46	66	59	55	474	532	325	424	385	412
ENSMUSG00000042548	ASXL1	5	6	8	7	14	7	33	35	28	48	28	33
ENSMUSG00000042549	MAP2K3OS	1	1	2	0	1	2	3	6	4	5	5	1
ENSMUSG00000042557	SIN3A	6	8	12	15	15	8	43	56	31	48	38	47
ENSMUSG00000042558	ADPRHL2	3	3	2	5	5	2	28	21	22	26	24	13
ENSMUSG00000042564	FAM227A	0	0	0	1	0	0	2	1	0	0	0	0
ENSMUSG00000042567	NEK10	1	0	0	0	0	0	0	1	0	0	0	0
ENSMUSG00000042569	DHRS7B	1	2	0	3	1	4	20	17	14	15	15	17
ENSMUSG00000042570	MIER2	4	7	4	7	7	8	21	17	18	17	14	16
ENSMUSG00000042572	UBE2Q1	23	26	35	26	31	28	132	146	110	159	101	115

ENSMUSG00000042579	4632404H12F	2	4	1	4	5	4	16	16	7	21	12	9
ENSMUSG00000042581	THSD7B	0	0	0	0	0	0	1	1	0	2	0	0
ENSMUSG00000042589	CUX2	0	0	0	1	0	0	0	0	0	0	1	0
ENSMUSG00000042590	IPO11	8	5	6	8	10	4	44	46	45	45	26	38
ENSMUSG00000042594	SH2B3	6	5	3	6	4	4	22	27	21	19	16	20
ENSMUSG00000042595	FAM199X	8	13	9	14	12	12	40	52	21	40	30	25
ENSMUSG00000042599	KDM7A	9	12	12	18	13	17	60	63	44	57	33	54
ENSMUSG00000042605	ATXN2	12	13	7	17	15	17	80	75	38	66	55	51
ENSMUSG00000042606	HIRIP3	8	21	12	14	11	16	63	71	55	62	47	61
ENSMUSG00000042608	STK40	5	20	7	10	14	11	54	49	35	49	33	37
ENSMUSG00000042613	PBXIP1	13	11	15	7	12	17	46	62	44	56	41	69
ENSMUSG00000042616	OSCP1	1	1	3	3	0	1	3	5	6	7	4	5
ENSMUSG00000042622	MAFF	0	0	2	0	0	0	0	2	0	2	2	1
ENSMUSG00000042625	SAFB2	43	43	33	41	33	45	166	151	103	156	155	141
ENSMUSG00000042626	SHC1	0	2	1	1	0	0	17	17	10	17	11	16
ENSMUSG00000042628	ZFYVE1	1	4	5	5	3	1	24	15	6	7	7	7
ENSMUSG00000042632	PLA2G6	1	1	0	2	2	0	2	4	7	6	4	8
ENSMUSG00000042641	RGSL1	0	0	0	0	0	0	0	0	0	0	1	0
ENSMUSG00000042642	FLAD1	2	0	2	2	0	2	9	4	8	9	2	8
ENSMUSG00000042644	ITPR3	4	3	3	4	2	7	23	20	15	20	17	20
ENSMUSG00000042647	ACAD12	1	1	1	0	3	2	3	3	2	7	4	6
ENSMUSG00000042650	ALKBH5	16	23	23	26	37	23	156	131	123	145	89	103
ENSMUSG00000042660	WDR55	2	6	4	10	4	7	39	32	32	29	20	21
ENSMUSG00000042670	IMMP1L	5	3	4	7	5	8	27	39	22	37	17	20
ENSMUSG00000042671	RGS8	0	0	0	1	0	0	0	0	0	0	0	0
ENSMUSG00000042675	YPEL3	3	1	5	2	3	1	10	7	2	11	6	10
ENSMUSG00000042677	ZC3H12A	3	1	1	0	0	1	6	3	2	4	0	9
ENSMUSG00000042678	MYO15	0	0	0	0	0	0	0	1	0	0	0	0
ENSMUSG00000042680	GAREM1	0	0	0	0	0	1	0	0	1	1	1	0
ENSMUSG00000042682	SELENOK	13	10	12	7	7	6	81	86	51	106	72	62
ENSMUSG00000042688	MAPK6	5	11	11	9	7	9	50	51	26	34	26	24
ENSMUSG00000042694	STN1	0	0	0	2	2	0	10	8	4	8	1	7
ENSMUSG00000042699	DHX9	32	53	36	59	37	42	237	275	179	264	173	205
ENSMUSG00000042700	SIPA1L1	15	30	22	27	28	16	99	107	72	96	79	113
ENSMUSG00000042705	COMMD10	2	2	1	1	2	1	6	14	5	12	6	14
ENSMUSG00000042707	DNALI1	3	1	2	4	2	1	10	4	7	7	6	6
ENSMUSG00000042709	ATPAF2	4	3	4	5	9	10	23	38	19	25	23	22
ENSMUSG00000042712	TCEAL9	0	0	0	0	0	0	0	0	0	1	1	0
ENSMUSG00000042719	NAA25	27	45	29	46	36	33	152	168	140	164	130	114
ENSMUSG00000042726	TRAFD1	3	8	5	6	5	5	27	32	17	31	35	33
ENSMUSG00000042729	WDR74	17	10	15	21	18	19	69	70	73	87	65	85
ENSMUSG00000042737	DPM3	14	18	18	26	20	24	126	136	107	131	107	146
ENSMUSG00000042742	BMT2	2	10	6	9	3	4	43	46	36	48	37	32
ENSMUSG00000042743	SGTB	0	0	0	0	0	0	1	1	0	1	0	0
ENSMUSG00000042744	HECTD4	6	15	18	16	17	11	58	54	33	51	46	58
ENSMUSG00000042745	ID1	0	0	0	0	0	0	0	0	1	0	0	0
ENSMUSG00000042747	KRTCAP2	37	44	38	42	43	31	168	231	173	176	152	197
ENSMUSG00000042750	BEX2	0	0	0	0	0	0	0	0	0	0	0	1
ENSMUSG00000042757	TMEM108	0	0	0	0	0	2	0	0	0	0	0	0
ENSMUSG00000042763	MANEAL	0	0	1	1	0	0	1	2	0	0	2	1
ENSMUSG00000042766	TRIM46	0	1	1	0	1	0	2	1	0	2	0	1
ENSMUSG00000042772	SMG7	19	31	38	43	35	29	118	126	94	149	92	144
ENSMUSG00000042784	MUC1	0	0	0	0	2	0	0	0	0	2	0	0
ENSMUSG00000042787	EXOG	1	2	3	5	2	2	12	6	7	9	5	5
ENSMUSG00000042790	RNF214	2	4	4	6	11	7	19	23	13	26	7	19
ENSMUSG00000042797	AQP11	1	0	0	0	0	0	2	2	0	4	0	2
ENSMUSG00000042807	HECW2	0	0	0	0	0	1	1	1	0	2	1	1
ENSMUSG00000042810	KRBA1	0	1	1	2	3	1	0	0	0	1	0	0

ENSMUSG00000042814	MCTS2	0	1	0	0	0	1	0	0	0	0	1	2
ENSMUSG00000042816	GPR151	0	0	0	0	0	0	1	0	0	0	0	0
ENSMUSG00000042817	FLT3	0	1	0	0	0	0	1	0	0	0	0	0
ENSMUSG00000042831	ALKBH6	3	2	2	3	6	0	10	8	5	10	8	7
ENSMUSG00000042834	NREP	0	0	0	0	0	0	1	0	0	0	0	0
ENSMUSG00000042842	SERPINB6B	2	0	2	2	1	1	8	14	11	12	12	11
ENSMUSG00000042851	ZC3H6	1	5	0	0	2	0	6	5	3	5	1	3
ENSMUSG00000042854	TRP53RKB	1	1	1	0	1	0	12	6	7	6	7	6
ENSMUSG00000042857	GM9776	0	0	0	0	0	0	2	3	1	4	0	1
ENSMUSG00000042870	TOM1	1	0	0	0	0	0	0	0	0	0	0	0
ENSMUSG00000042873	LHFPL4	3	2	4	3	5	4	16	20	13	20	19	27
ENSMUSG00000042901	AIDA	0	4	2	2	3	2	20	26	18	17	10	15
ENSMUSG00000042903	FOXO4	6	4	10	8	4	3	27	27	17	29	24	16
ENSMUSG00000042909	OLFR648	0	0	0	0	0	0	0	0	2	0	0	1
ENSMUSG00000042940	9230106D20[illegible]	0	1	0	0	0	0	0	0	0	0	0	0
ENSMUSG00000042942	GREB1L	0	0	0	0	0	0	0	0	0	0	2	0
ENSMUSG00000042962	GM5436	2	0	2	3	2	1	14	3	3	5	4	7
ENSMUSG00000042978	SBK1	1	0	1	1	0	0	2	1	1	1	1	2
ENSMUSG00000042992	BORCS5	2	2	2	1	1	3	5	6	8	1	3	4
ENSMUSG00000042997	NHLRC3	0	0	1	0	0	2	9	4	3	3	2	3
ENSMUSG00000043004	GNG2	24	16	19	20	23	18	125	119	116	116	101	110
ENSMUSG00000043008	KLHL6	2	4	6	4	3	5	12	8	18	13	14	18
ENSMUSG00000043015	NEMP2	2	6	6	5	3	5	23	16	17	21	16	21
ENSMUSG00000043017	PTGIR	0	0	2	0	0	0	0	0	0	0	0	0
ENSMUSG00000043019	EDEM3	10	16	19	15	16	19	46	51	26	76	43	34
ENSMUSG00000043024	GM16433	0	0	0	0	0	0	0	0	0	1	0	2
ENSMUSG00000043036	CCDC63	0	0	0	0	0	1	3	1	1	1	0	2
ENSMUSG00000043050	TNP2	3	3	0	4	2	1	20	28	13	19	11	15
ENSMUSG00000043051	DISC1	0	0	0	2	0	0	0	0	1	2	0	0
ENSMUSG00000043059	ZFP513	1	0	3	2	1	0	11	4	6	9	5	7
ENSMUSG00000043061	TMEM18	2	3	4	1	2	3	10	8	3	10	4	7
ENSMUSG00000043065	SPICE1	1	3	2	2	2	1	11	10	9	12	4	6
ENSMUSG00000043066	VMN1R66	0	0	0	0	0	0	0	0	0	1	0	0
ENSMUSG00000043067	DPY19L1	9	21	22	22	25	23	103	102	61	98	53	67
ENSMUSG00000043079	SYNPO	0	0	0	1	0	0	0	0	0	0	0	0
ENSMUSG00000043090	ZFP866	0	2	2	2	1	1	3	6	4	2	5	6
ENSMUSG00000043091	TUBA1C	12	10	16	9	12	8	69	93	67	82	53	59
ENSMUSG00000043102	QRFP	1	1	0	0	0	0	1	1	1	1	0	2
ENSMUSG00000043110	LRRN4	1	0	0	0	0	1	1	2	1	2	1	1
ENSMUSG00000043131	MOB1A	17	11	17	17	18	17	108	119	88	144	95	105
ENSMUSG00000043140	TMEM186	4	8	7	6	6	3	13	13	11	16	9	21
ENSMUSG00000043154	PPP2R3A	1	4	2	2	1	3	4	5	5	7	3	5
ENSMUSG00000043157	ARL11	0	0	1	0	0	0	0	1	0	0	0	0
ENSMUSG00000043162	PYURF	1	2	0	0	2	2	7	4	7	3	7	3
ENSMUSG00000043168	4930426D05[illegible]	0	0	0	0	0	0	1	0	0	0	0	0
ENSMUSG00000043183	SIMC1	1	5	3	3	8	4	25	29	17	16	21	26
ENSMUSG00000043190	RFESD	2	3	3	7	2	3	17	13	8	20	8	12
ENSMUSG00000043207	ZMPSTE24	8	18	7	13	14	6	63	48	41	59	31	34
ENSMUSG00000043241	UPF2	28	56	44	79	36	42	158	143	109	218	132	128
ENSMUSG00000043243	FAM129C	0	0	1	0	1	4	3	1	0	2	1	0
ENSMUSG00000043251	EXOC3L	0	0	0	0	0	0	0	1	0	0	0	0
ENSMUSG00000043252	TMEM64	9	7	3	6	5	5	31	24	26	25	21	21
ENSMUSG00000043257	PIGV	0	0	0	1	0	0	3	1	1	4	3	0
ENSMUSG00000043262	UEVLD	0	1	1	1	0	0	5	5	5	0	0	4
ENSMUSG00000043279	TRIM56	7	10	8	11	8	11	31	42	20	45	29	29
ENSMUSG00000043282	TEDDM1B	0	0	0	0	0	0	0	0	0	0	1	0
ENSMUSG00000043284	TMEM11	5	2	4	10	7	6	38	33	21	28	28	30
ENSMUSG00000043286	PNPLA1	0	0	0	1	0	0	4	3	3	3	3	1

ENSMUSG00000043289	MEI4	0	2	1	0	1	2	5	1	1	1	1	1
ENSMUSG00000043290	ZFP784	0	0	0	0	0	0	1	1	1	1	0	0
ENSMUSG00000043298	SMCO3	0	0	0	0	0	0	1	0	0	0	0	0
ENSMUSG00000043311	D17H6S53E	4	7	13	4	5	8	33	41	27	27	26	35
ENSMUSG00000043323	FBRSL1	2	2	4	3	8	3	27	27	18	26	20	16
ENSMUSG00000043336	FILIP1L	31	55	43	43	54	45	222	191	170	298	188	150
ENSMUSG00000043340	6530409C15F	0	0	0	0	0	0	0	0	1	1	0	1
ENSMUSG00000043372	HEXIM2	0	0	0	0	1	0	1	4	1	2	1	3
ENSMUSG00000043384	GPRASP1	2	3	1	1	3	4	17	12	9	15	9	20
ENSMUSG00000043385	OLFR267	0	0	0	0	0	0	0	1	0	1	0	0
ENSMUSG00000043391	2510009E07F	1	0	0	2	0	0	1	5	6	8	1	3
ENSMUSG00000043410	HFM1	0	0	0	0	0	0	0	0	0	2	0	0
ENSMUSG00000043411	USP48	11	17	18	31	10	11	78	74	49	69	70	56
ENSMUSG00000043415	OTUD1	0	0	0	0	0	0	0	1	0	0	0	0
ENSMUSG00000043419	CHD3OS	0	0	0	0	0	1	0	0	0	0	0	0
ENSMUSG00000043421	HILPDA	0	3	1	5	1	3	9	7	14	18	8	16
ENSMUSG00000043424	EIF3J2	0	0	0	2	1	0	2	4	2	5	0	3
ENSMUSG00000043432	LENG9	1	6	5	14	3	2	26	15	17	21	15	16
ENSMUSG00000043445	PGP	17	10	20	15	14	20	111	125	91	131	90	101
ENSMUSG00000043456	ZFP536	0	0	0	0	0	1	0	0	0	0	0	0
ENSMUSG00000043460	ELFN2	1	0	0	2	1	0	2	1	0	2	0	0
ENSMUSG00000043467	ZBTB37	3	3	4	8	6	3	20	16	11	11	10	11
ENSMUSG00000043483	GM6863	0	0	0	1	1	0	1	3	3	1	3	2
ENSMUSG00000043487	ACOT6	0	0	0	0	0	2	0	2	0	0	0	1
ENSMUSG00000043488	FRMD8OS	0	1	0	0	0	0	2	2	3	1	1	1
ENSMUSG00000043505	GIMAP5	31	28	25	33	34	21	119	143	94	124	108	104
ENSMUSG00000043510	HSCB	4	2	1	4	6	2	14	5	13	12	9	13
ENSMUSG00000043531	SORCS1	0	0	0	0	0	0	0	1	0	0	0	0
ENSMUSG00000043535	SETX	11	21	19	20	16	16	96	81	68	87	60	61
ENSMUSG00000043541	CASC1	0	4	1	7	0	2	16	16	12	17	10	14
ENSMUSG00000043542	ZC2HC1A	0	5	4	4	2	1	15	25	9	20	12	13
ENSMUSG00000043557	MDGA1	0	0	0	0	0	0	0	0	0	1	0	0
ENSMUSG00000043572	PARS2	0	0	0	1	2	1	0	1	2	3	2	2
ENSMUSG00000043583	4930470P17F	0	0	1	0	0	0	0	0	0	0	0	0
ENSMUSG00000043602	ZFP3	0	0	0	0	0	0	2	2	1	1	2	1
ENSMUSG00000043618	EIF5AL3-PS	0	0	0	0	0	0	0	0	1	1	0	0
ENSMUSG00000043623	4933412A08F	1	1	0	0	0	0	0	2	0	0	0	0
ENSMUSG00000043629	1700019D03F	0	0	0	0	0	0	0	0	0	0	0	1
ENSMUSG00000043631	ECM2	1	0	0	0	0	0	1	1	1	1	0	1
ENSMUSG00000043633	FAM221B	0	0	2	0	1	0	3	4	2	7	1	5
ENSMUSG00000043644	0610009L18R	0	0	0	0	1	0	0	0	1	1	0	0
ENSMUSG00000043659	NPSR1	0	0	0	0	0	0	0	0	0	0	1	0
ENSMUSG00000043661	GM9785	0	0	0	0	0	0	0	0	1	0	0	1
ENSMUSG00000043671	DPY19L3	4	6	1	3	4	3	5	12	7	10	7	5
ENSMUSG00000043683	FEM1A	5	9	10	12	12	10	61	47	36	64	40	45
ENSMUSG00000043687	1190005I06R	0	0	0	0	0	0	1	0	1	1	1	0
ENSMUSG00000043702	PDE12	3	2	7	4	6	10	30	33	31	34	16	30
ENSMUSG00000043716	RPL7	31	52	58	68	64	55	310	301	269	281	227	282
ENSMUSG00000043733	PTPN11	7	10	14	9	11	7	58	47	33	48	40	30
ENSMUSG00000043747	1520401A03F	0	0	0	0	0	0	0	0	0	0	1	1
ENSMUSG00000043753	DMRTA1	1	0	1	0	0	0	0	1	0	0	1	0
ENSMUSG00000043770	GM12481	0	0	0	0	0	0	0	0	2	0	0	0
ENSMUSG00000043782	BICDL2	0	0	0	0	0	0	0	0	1	2	0	0
ENSMUSG00000043789	VWCE	0	0	0	0	0	0	1	0	0	0	0	0
ENSMUSG00000043794	D830025C05I	0	0	1	0	2	3	0	1	1	0	1	0
ENSMUSG00000043807	LY6G5B	1	0	0	0	0	0	3	4	1	1	3	3
ENSMUSG00000043831	LYSMD4	0	0	0	0	0	0	0	0	0	0	0	1
ENSMUSG00000043832	CLEC4A3	0	0	0	0	0	0	0	0	1	0	0	0

ENSMUSG00000043843	TMEM145	0	0	0	0	0	0	0	0	0	0	0	1
ENSMUSG00000043866	TAF10	5	9	14	9	10	10	35	36	38	50	32	35
ENSMUSG00000043872	ZMYM1	12	16	9	16	12	12	56	63	34	52	42	50
ENSMUSG00000043873	CHIL5	0	0	0	0	0	0	0	0	1	1	1	1
ENSMUSG00000043881	KBTBD7	3	0	0	0	1	0	3	1	3	3	1	2
ENSMUSG00000043885	SLC36A4	0	0	0	0	0	0	1	0	0	0	0	0
ENSMUSG00000043895	S1PR2	0	0	0	0	0	0	0	0	0	0	1	1
ENSMUSG00000043903	ZFP469	0	0	0	0	0	0	0	1	0	0	0	0
ENSMUSG00000043909	TRP53BP1	14	10	10	8	21	19	43	45	48	56	44	46
ENSMUSG00000043923	CCDC84	22	36	27	45	30	30	175	144	113	148	151	129
ENSMUSG00000043925	OLFR544	0	1	0	2	0	1	2	0	0	4	0	1
ENSMUSG00000043929	KLHL15	0	7	0	4	6	3	26	11	13	22	18	12
ENSMUSG00000043931	GIMAP7	0	2	1	4	1	2	14	14	9	15	9	12
ENSMUSG00000043932	KLRI2	0	0	0	0	0	0	1	0	0	0	0	1
ENSMUSG00000043953	CCRL2	0	0	0	0	0	0	1	0	1	1	0	0
ENSMUSG00000043962	THRAP3	32	34	36	48	50	24	173	187	172	239	165	171
ENSMUSG00000043964	ORAI3	1	2	3	0	3	3	7	13	13	12	9	14
ENSMUSG00000043987	CEP164	11	12	10	12	8	6	33	36	29	34	34	32
ENSMUSG00000043991	PURA	13	20	22	21	22	20	83	81	66	95	67	75
ENSMUSG00000043998	MGAT2	18	20	16	21	12	22	96	122	80	112	99	102
ENSMUSG00000043999	GPR75	0	0	0	0	0	0	1	0	0	0	0	0
ENSMUSG00000044005	GLS2	1	1	0	0	0	0	0	4	3	1	0	3
ENSMUSG00000044006	CILP2	1	0	0	0	0	0	0	1	0	0	0	0
ENSMUSG00000044018	MRPL50	7	4	10	6	9	16	57	69	40	58	53	47
ENSMUSG00000044024	RELL2	1	1	3	2	3	3	8	10	10	9	5	7
ENSMUSG00000044026	SLC35G1	5	4	6	5	6	8	38	29	25	39	22	30
ENSMUSG00000044030	IRF2BP1	2	3	2	1	0	1	11	7	10	8	4	11
ENSMUSG00000044033	CCDC141	0	0	0	0	0	0	0	0	0	0	0	2
ENSMUSG00000044034	NPB	0	0	0	0	0	0	1	0	1	1	0	1
ENSMUSG00000044037	ALS2CL	0	0	0	0	0	0	0	1	0	0	0	1
ENSMUSG00000044052	CCR10	0	0	0	0	0	0	2	0	0	0	0	0
ENSMUSG00000044066	CEP68	4	2	2	4	6	2	14	20	11	23	17	20
ENSMUSG00000044067	GPR22	0	0	0	0	1	0	0	0	0	0	0	0
ENSMUSG00000044068	ZRSR1	6	9	3	11	8	10	33	32	33	40	31	33
ENSMUSG00000044072	EML6	0	0	0	0	0	0	1	0	0	0	0	0
ENSMUSG00000044080	S100A1	0	1	2	5	2	3	19	16	15	16	14	17
ENSMUSG00000044081	ZFP85OS	0	0	0	0	0	0	0	1	0	0	1	1
ENSMUSG00000044083	EFCAB8	0	0	0	0	2	0	0	0	1	2	0	0
ENSMUSG00000044086	LMOD3	4	5	4	4	10	8	25	41	23	33	30	32
ENSMUSG00000044092	C130050O18I	0	0	0	0	0	0	1	0	0	2	1	0
ENSMUSG00000044098	RSBN1	13	16	22	24	19	27	103	96	55	128	53	84
ENSMUSG00000044134	FAM109A	1	0	3	3	0	0	6	4	3	2	4	4
ENSMUSG00000044139	PRSS53	0	0	0	0	0	0	0	0	0	2	0	1
ENSMUSG00000044145	1810024B03F	4	2	1	2	1	0	17	13	10	10	6	13
ENSMUSG00000044147	ARF6	43	49	53	60	52	45	291	258	207	240	189	230
ENSMUSG00000044148	1810030O07I	2	5	2	3	8	3	14	20	20	32	12	19
ENSMUSG00000044149	NKRF	0	4	3	5	2	6	17	30	8	23	14	16
ENSMUSG00000044150	BCLAF3	3	7	4	3	7	3	25	24	15	30	15	14
ENSMUSG00000044155	LSM8	6	8	11	7	13	9	106	140	74	117	69	78
ENSMUSG00000044162	TNIP3	0	0	0	0	0	0	0	2	2	1	0	2
ENSMUSG00000044165	BCL2L15	0	0	0	0	0	0	2	0	0	1	0	0
ENSMUSG00000044167	FOXO1	18	16	12	18	16	23	58	53	43	56	30	53
ENSMUSG00000044176	SPINK10	1	0	0	2	0	1	3	2	4	2	4	3
ENSMUSG00000044197	GPR146	1	3	2	3	4	3	15	10	7	16	4	3
ENSMUSG00000044199	S1PR4	3	11	8	9	8	6	27	31	29	36	21	32
ENSMUSG00000044201	CDC25C	3	9	4	6	6	10	38	31	29	39	26	28
ENSMUSG00000044221	GRSF1	4	4	10	11	10	11	63	67	56	57	37	48
ENSMUSG00000044224	DNAJC21	10	19	17	23	19	24	128	110	103	108	88	95

ENSMUSG00000044229	NXPE4	0	0	0	0	0	0	0	0	1	0	0	0
ENSMUSG00000044244	IL20RB	0	0	2	1	0	0	0	0	0	1	3	0
ENSMUSG00000044250	PCED1B	17	13	19	21	15	16	70	87	58	67	59	67
ENSMUSG00000044258	CTLA2A	0	0	0	0	0	0	0	0	1	0	0	0
ENSMUSG00000044279	CRB3	1	0	0	0	0	0	0	0	0	0	0	0
ENSMUSG00000044285	GM1821	9	8	12	4	14	3	13	25	17	24	19	21
ENSMUSG00000044287	NRN1L	0	0	0	0	0	0	0	1	0	0	0	0
ENSMUSG00000044303	CDKN2A	36	39	22	24	41	33	213	248	150	213	187	232
ENSMUSG00000044308	UBR3	15	18	11	21	20	14	80	111	63	102	88	74
ENSMUSG00000044328	TRP53I13	1	3	1	3	4	2	3	6	4	11	5	11
ENSMUSG00000044330	GM9790	0	0	0	0	0	0	0	0	1	0	0	0
ENSMUSG00000044339	ALKBH2	0	5	2	2	0	1	7	15	5	10	4	2
ENSMUSG00000044340	PHLPP1	42	38	36	55	44	42	340	391	253	391	258	281
ENSMUSG00000044348	SLC25A53	1	4	2	3	2	3	9	17	12	7	8	13
ENSMUSG00000044349	SNHG11	0	0	0	1	0	0	0	0	0	1	0	0
ENSMUSG00000044350	LACC1	3	1	5	4	0	3	25	9	8	16	17	13
ENSMUSG00000044352	SOWAHA	0	0	0	0	0	0	1	0	0	0	0	0
ENSMUSG00000044365	CXXC4	0	2	2	2	0	0	3	1	0	5	0	1
ENSMUSG00000044367	SLC16A13	0	1	0	0	0	0	0	0	0	0	0	0
ENSMUSG00000044390	TIGD3	0	0	0	0	0	0	0	0	0	2	0	0
ENSMUSG00000044408	SPTSSA	10	11	8	25	12	28	99	108	62	121	83	68
ENSMUSG00000044424	GM9493	0	0	0	0	0	0	0	1	1	0	0	0
ENSMUSG00000044433	CAMSAP3	0	0	0	0	0	0	0	0	0	1	0	0
ENSMUSG00000044441	OLFR1442	0	1	0	0	0	0	0	0	0	0	0	0
ENSMUSG00000044442	N6AMT1	4	4	3	4	2	4	19	16	13	15	21	15
ENSMUSG00000044452	ZFP507	3	3	0	1	1	6	21	24	15	21	9	18
ENSMUSG00000044453	FFAR1	0	0	1	0	0	0	0	0	0	0	0	0
ENSMUSG00000044456	RIN3	5	5	5	1	6	7	44	30	30	29	19	33
ENSMUSG00000044465	FAM160A2	7	9	11	9	12	6	36	35	20	36	31	23
ENSMUSG00000044468	FAM46C	0	0	1	1	1	0	0	0	0	0	1	0
ENSMUSG00000044469	TNFAIP8L1	1	0	0	0	0	1	0	0	0	1	0	0
ENSMUSG00000044471	LNCPINT	0	0	1	0	1	1	1	1	2	1	1	1
ENSMUSG00000044475	ASCC1	2	2	0	1	1	1	9	9	6	11	9	8
ENSMUSG00000044477	ZFAND3	0	4	3	5	4	9	36	43	29	35	30	37
ENSMUSG00000044496	2510039O18I	4	9	2	7	9	6	45	53	37	49	37	32
ENSMUSG00000044501	ZFP758	1	2	6	1	2	1	4	7	12	11	3	3
ENSMUSG00000044502	BOD1	3	5	6	4	5	4	18	12	10	14	12	14
ENSMUSG00000044505	LINGO4	0	0	0	0	0	0	0	0	0	0	1	1
ENSMUSG00000044533	RPS2	204	242	239	269	287	262	1044	1047	748	1096	809	984
ENSMUSG00000044548	DACT1	0	0	0	0	2	0	0	0	1	0	0	1
ENSMUSG00000044550	TCEAL3	0	0	0	0	0	0	0	0	0	1	0	0
ENSMUSG00000044551	9930012K11F	1	1	1	1	0	3	5	1	1	3	1	1
ENSMUSG00000044566	CAGE1	0	0	0	0	0	0	1	0	0	0	1	1
ENSMUSG00000044573	ACP1	1	5	5	5	2	10	37	37	27	46	18	20
ENSMUSG00000044581	4932415D10F	0	0	0	0	0	0	0	0	0	1	0	0
ENSMUSG00000044595	DND1	2	1	0	0	0	0	5	5	2	3	2	5
ENSMUSG00000044600	SMIM7	1	5	4	8	7	4	31	23	21	23	23	13
ENSMUSG00000044609	GM9294	0	0	0	0	0	0	1	0	0	0	0	0
ENSMUSG00000044617	ZBTB39	1	0	1	2	2	2	6	6	6	15	5	8
ENSMUSG00000044626	LIPH	0	0	0	0	0	0	0	0	0	0	0	1
ENSMUSG00000044627	SWI5	34	35	31	47	43	44	282	286	234	300	201	256
ENSMUSG00000044629	CNRIP1	2	4	0	3	5	2	17	18	12	35	9	14
ENSMUSG00000044633	B530045E10F	13	23	19	14	16	11	57	68	40	77	49	52
ENSMUSG00000044636	CSRNP2	1	1	0	0	1	1	1	1	2	1	1	0
ENSMUSG00000044641	PARD6B	0	2	0	0	1	0	1	2	5	0	3	1
ENSMUSG00000044667	PLPPR4	0	0	0	0	0	0	1	0	0	0	0	0
ENSMUSG00000044694	2010007H06F	1	0	0	0	0	1	2	3	2	5	2	0
ENSMUSG00000044700	TMEM201	8	1	3	4	9	6	30	27	16	29	29	21

ENSMUSG00000044701	IL27	0	0	0	0	0	0	0	0	0	1	0	0
ENSMUSG00000044702	PALB2	2	7	7	0	4	7	25	20	13	39	15	22
ENSMUSG00000044709	GEMIN7	4	2	3	1	3	4	6	15	11	14	11	8
ENSMUSG00000044712	SLC38A6	0	2	1	3	2	1	7	9	10	7	6	13
ENSMUSG00000044715	GSKIP	3	1	4	0	1	5	9	15	6	15	12	8
ENSMUSG00000044719	E230025N22I	0	1	0	0	0	0	0	0	0	0	0	0
ENSMUSG00000044726	ERICH5	0	0	0	0	0	0	1	0	0	0	0	0
ENSMUSG00000044730	9930104L06R	1	3	4	3	4	2	19	17	15	12	13	15
ENSMUSG00000044734	SERPINB1A	1	2	1	0	0	2	1	5	3	3	4	3
ENSMUSG00000044751	GM12231	0	0	0	0	0	0	0	0	0	1	0	0
ENSMUSG00000044757	GM6430	0	0	0	0	0	0	0	1	0	0	0	0
ENSMUSG00000044763	TRMT10C	12	15	10	15	23	16	68	59	45	64	56	43
ENSMUSG00000044768	D1ERTD622E	5	6	6	3	5	0	21	21	16	20	14	13
ENSMUSG00000044770	SCML4	1	0	1	1	0	1	3	4	1	1	6	4
ENSMUSG00000044783	HJURP	28	40	41	33	52	36	219	251	196	267	195	194
ENSMUSG00000044786	ZFP36	1	1	1	6	4	4	15	12	14	16	9	12
ENSMUSG00000044788	FADS6	2	0	1	0	3	1	5	4	2	2	2	2
ENSMUSG00000044791	SETD2	17	28	29	35	18	28	100	108	86	128	74	83
ENSMUSG00000044792	ISCA1	0	0	2	1	1	0	9	5	7	7	5	8
ENSMUSG00000044795	CYB5D1	0	0	1	0	0	0	1	0	0	0	0	1
ENSMUSG00000044807	ZFP354C	0	0	0	0	0	0	0	0	0	1	0	1
ENSMUSG00000044814	OLFR543	1	1	2	3	0	0	0	1	1	1	2	1
ENSMUSG00000044820	AY074887	0	0	0	2	0	0	0	1	1	0	1	2
ENSMUSG00000044824	OLFR545	0	0	0	1	0	1	0	0	0	0	0	0
ENSMUSG00000044847	LSM11	2	2	1	2	2	6	20	22	11	21	8	10
ENSMUSG00000044854	1700056E22R	1	1	0	1	1	0	0	0	1	0	1	0
ENSMUSG00000044857	LEMD2	2	5	3	5	3	2	18	17	14	12	17	14
ENSMUSG00000044860	GM1123	0	0	0	0	0	0	0	0	1	1	0	1
ENSMUSG00000044867	GIMAP1OS	4	4	3	2	3	3	16	10	5	11	2	5
ENSMUSG00000044876	ZFP444	8	6	3	4	6	3	25	35	20	15	19	25
ENSMUSG00000044881	COA4	0	3	1	0	0	1	6	6	3	7	1	1
ENSMUSG00000044894	UQCRQ	33	33	41	43	43	43	302	290	223	287	231	249
ENSMUSG00000044906	4930503L19R	7	13	14	7	9	9	78	66	37	96	35	41
ENSMUSG00000044916	1700029I15R	0	0	0	0	0	0	0	0	0	0	1	0
ENSMUSG00000044921	RASSF9	0	1	0	0	0	0	0	0	0	0	0	0
ENSMUSG00000044927	H1FX	0	0	0	0	0	0	0	0	0	0	1	0
ENSMUSG00000044934	ZFP367	11	14	9	8	10	11	55	44	57	69	39	42
ENSMUSG00000044937	TTC41	1	0	2	0	0	0	1	1	1	3	0	3
ENSMUSG00000044938	KLHL31	0	0	0	0	0	0	0	0	1	0	0	0
ENSMUSG00000044948	CFAP43	0	0	0	1	0	2	1	2	0	1	2	1
ENSMUSG00000044949	UBTD2	0	2	0	0	2	0	5	3	2	1	1	3
ENSMUSG00000044950	PWWP2A	12	15	15	19	21	7	63	60	32	75	43	53
ENSMUSG00000044952	KCTD21	0	1	0	0	0	1	0	0	1	0	0	1
ENSMUSG00000044957	PP2D1	0	0	0	0	0	0	0	1	0	0	0	0
ENSMUSG00000044966	FBXO48	2	0	0	0	1	0	2	0	1	0	0	1
ENSMUSG00000044968	NAPEPLD	3	1	2	2	0	4	5	6	6	9	3	4
ENSMUSG00000044976	WDR72	0	1	0	0	0	0	0	0	0	0	0	0
ENSMUSG00000044982	SFT2D3	4	2	3	3	1	0	5	10	6	6	8	6
ENSMUSG00000044986	TST	0	0	1	0	0	0	0	2	0	1	0	0
ENSMUSG00000044991	1110034G24I	0	0	0	0	0	0	3	2	0	1	1	0
ENSMUSG00000045005	FZD5	3	1	1	3	0	3	0	1	1	7	2	5
ENSMUSG00000045019	ACER1	0	0	0	0	0	0	0	0	0	1	0	0
ENSMUSG00000045027	PRSS22	0	0	0	0	0	0	0	1	0	0	0	0
ENSMUSG00000045031	CETN4	0	0	1	0	0	2	3	5	3	3	1	4
ENSMUSG00000045045	LRFN4	0	0	2	1	0	0	2	4	2	1	1	4
ENSMUSG00000045055	RPSA-PS2	0	0	0	0	1	1	2	1	0	0	0	3
ENSMUSG00000045071	E130308A19R	5	3	2	6	5	6	9	16	21	23	9	11
ENSMUSG00000045078	RNF216	0	0	0	0	0	0	0	1	0	0	0	1

ENSMUSG00000045092	S1PR1	4	0	3	3	1	5	15	15	5	18	7	16
ENSMUSG00000045098	KMT5B	6	7	6	4	7	6	26	31	23	25	27	26
ENSMUSG00000045100	SLC25A26	2	2	2	3	1	2	8	11	10	6	7	10
ENSMUSG00000045102	POLN	1	3	2	2	1	1	7	10	8	9	2	6
ENSMUSG00000045106	CCDC73	2	6	3	3	3	4	8	14	12	14	10	14
ENSMUSG00000045107	SAYSD1	1	1	1	2	1	1	7	5	3	9	1	6
ENSMUSG00000045128	RPL18A	29	33	40	40	30	27	223	217	174	232	140	224
ENSMUSG00000045132	OLFR620	0	0	0	0	0	0	0	0	0	0	0	1
ENSMUSG00000045136	TUBB2B	1	1	0	1	0	1	3	4	5	4	2	4
ENSMUSG00000045140	PIGW	2	0	1	0	2	0	4	1	1	1	1	3
ENSMUSG00000045160	BOLA3	6	6	12	19	12	10	64	77	55	48	50	72
ENSMUSG00000045165	AI467606	13	20	17	10	13	13	91	108	75	90	51	57
ENSMUSG00000045176	BORCS6	0	0	0	0	1	0	10	8	4	8	5	1
ENSMUSG00000045193	CIRBP	4	1	0	2	5	3	23	14	6	14	15	18
ENSMUSG00000045205	DPY19L4	3	3	0	2	5	5	16	15	11	15	8	10
ENSMUSG00000045210	VCPIP1	21	19	15	29	23	15	72	79	54	85	64	61
ENSMUSG00000045211	NUDT18	0	0	0	0	2	0	1	4	0	1	1	1
ENSMUSG00000045215	ASXL3	0	1	0	0	0	0	0	0	0	0	0	0
ENSMUSG00000045216	HS6ST1	4	8	2	4	5	13	28	21	30	20	15	32
ENSMUSG00000045225	OLFR1152	1	0	0	1	0	0	0	2	0	0	1	0
ENSMUSG00000045231	BC106179	1	0	1	0	0	0	2	1	0	1	0	1
ENSMUSG00000045237	1110012L19R	2	0	3	1	0	2	9	8	9	5	3	13
ENSMUSG00000045238	A730035I17R	0	0	0	0	0	0	0	1	0	1	1	0
ENSMUSG00000045248	MED26	1	3	0	2	4	4	11	13	11	21	6	13
ENSMUSG00000045251	ZFP688	0	1	1	0	1	0	1	4	4	3	9	7
ENSMUSG00000045252	ZFP574	3	1	2	2	5	4	8	14	10	12	15	6
ENSMUSG00000045257	MORN2	0	0	1	0	0	0	2	0	0	1	2	1
ENSMUSG00000045259	KLHDC9	0	1	0	0	0	0	1	0	1	0	0	0
ENSMUSG00000045268	ZFP691	5	3	5	4	5	1	24	32	22	27	18	16
ENSMUSG00000045273	CENPH	11	10	16	15	15	9	57	52	47	93	69	73
ENSMUSG00000045275	LCA5L	0	1	0	0	0	1	1	1	7	4	0	2
ENSMUSG00000045282	TMEM86B	1	1	3	1	1	0	5	3	5	3	2	5
ENSMUSG00000045284	DCAF12L1	0	0	0	1	0	0	0	0	0	0	0	0
ENSMUSG00000045287	RTN4RL1	3	1	4	9	11	8	30	34	29	22	20	23
ENSMUSG00000045291	E330021D16F	1	3	0	2	1	0	9	9	4	8	6	9
ENSMUSG00000045294	INSIG1	69	86	85	113	84	106	485	450	384	449	304	394
ENSMUSG00000045302	PREB	15	17	21	24	17	6	60	87	51	72	63	59
ENSMUSG00000045312	LHFPL2	0	0	0	1	0	0	0	0	0	0	0	1
ENSMUSG00000045319	PROSER2	0	0	0	0	0	1	0	0	0	0	0	0
ENSMUSG00000045326	FNDC7	0	0	0	0	0	0	0	0	0	2	0	0
ENSMUSG00000045327	6330549D23F	2	1	1	0	0	1	5	5	1	5	3	2
ENSMUSG00000045328	CENPE	48	66	70	120	85	76	354	345	281	365	287	243
ENSMUSG00000045345	9530056K15F	0	0	1	0	0	2	1	1	0	3	1	2
ENSMUSG00000045348	NYAP1	1	1	1	0	0	0	1	6	2	6	7	1
ENSMUSG00000045349	SH2D5	0	0	0	0	1	0	1	3	2	1	2	3
ENSMUSG00000045362	TNFRSF26	2	10	7	9	11	14	26	31	23	24	30	26
ENSMUSG00000045374	WDR81	0	0	0	3	0	0	4	4	5	0	4	5
ENSMUSG00000045377	TMEM88	0	1	1	1	0	0	5	5	2	3	5	4
ENSMUSG00000045378	PXT1	0	1	0	0	0	0	0	0	0	0	0	0
ENSMUSG00000045381	OLFR433	0	1	0	0	0	0	0	0	0	0	0	0
ENSMUSG00000045382	CXCR4	4	11	12	7	6	5	18	33	21	33	24	26
ENSMUSG00000045404	KCNK13	21	38	28	40	32	36	165	181	146	191	137	164
ENSMUSG00000045409	TRIM39	11	6	8	12	11	6	27	32	33	38	30	33
ENSMUSG00000045410	AKR1E1	3	8	4	9	1	3	28	27	31	32	21	25
ENSMUSG00000045411	2410002F23F	5	4	11	5	6	9	70	44	55	57	38	52
ENSMUSG00000045414	1190002N15I	2	3	5	4	9	7	23	36	18	30	26	29
ENSMUSG00000045427	HNRNPH2	4	0	2	3	1	2	30	34	16	35	25	16
ENSMUSG00000045435	TMEM60	4	9	6	6	3	3	38	33	24	34	32	27

ENSMUSG00000045438	COX19	1	1	4	1	1	1	15	22	16	16	12	20
ENSMUSG00000045440	INSM2	0	0	0	0	0	0	0	0	1	1	0	0
ENSMUSG00000045441	GPRIN3	14	13	21	7	20	22	54	46	44	61	45	45
ENSMUSG00000045464	2810002D19I	0	5	3	1	0	2	16	15	12	7	8	12
ENSMUSG00000045466	ZFP956	3	4	4	0	2	1	11	16	11	15	10	8
ENSMUSG00000045467	TTLL13	0	0	0	0	0	0	0	0	1	0	0	0
ENSMUSG00000045482	TRRAP	8	10	6	18	13	10	45	53	36	46	36	50
ENSMUSG00000045503	SYS1	6	6	1	6	6	6	17	16	20	20	4	15
ENSMUSG00000045509	GPR150	0	0	0	0	0	0	0	0	0	0	0	1
ENSMUSG00000045519	ZFP560	2	2	3	4	5	4	14	9	10	16	8	13
ENSMUSG00000045521	TSSK2	0	1	0	0	0	0	0	1	0	0	0	0
ENSMUSG00000045538	DDX28	2	2	0	2	4	1	6	12	11	6	8	13
ENSMUSG00000045576	ST7L	2	1	2	2	3	1	10	5	5	7	5	7
ENSMUSG00000045594	GLB1	8	4	3	5	8	3	25	31	24	35	17	13
ENSMUSG00000045598	ZFP553	1	5	3	2	2	3	22	17	18	28	14	26
ENSMUSG00000045608	DBX2	0	0	0	0	0	2	1	0	0	1	3	0
ENSMUSG00000045624	ESF1	17	26	13	34	24	25	110	109	68	91	86	92
ENSMUSG00000045625	PIGZ	0	0	0	0	1	0	1	0	1	0	0	0
ENSMUSG00000045631	TMPRSS12	1	0	0	0	0	0	0	1	0	1	0	0
ENSMUSG00000045639	ZFP629	2	3	0	1	4	3	8	4	9	4	7	3
ENSMUSG00000045648	VWC2L	0	0	0	0	0	0	0	1	0	0	0	0
ENSMUSG00000045655	FAM216B	0	1	0	0	0	0	0	0	0	0	0	0
ENSMUSG00000045659	PLEKHA7	0	0	0	0	1	1	5	15	2	5	4	3
ENSMUSG00000045664	CDC42EP2	0	0	0	1	0	0	0	0	0	0	0	1
ENSMUSG00000045665	MFSD5	4	2	6	5	3	5	14	22	22	20	18	15
ENSMUSG00000045671	SPRED2	3	4	7	5	3	1	28	40	26	26	18	19
ENSMUSG00000045672	COL27A1	0	0	0	0	0	0	0	1	0	0	0	0
ENSMUSG00000045679	PQLC3	0	0	0	0	0	0	2	4	3	5	3	4
ENSMUSG00000045690	WDR89	35	53	31	56	48	39	168	165	145	201	146	141
ENSMUSG00000045694	GM4997	27	26	27	45	27	30	98	110	75	131	66	104
ENSMUSG00000045725	PRR15	5	3	6	3	4	3	33	30	26	35	23	33
ENSMUSG00000045744	BRICD5	0	0	0	0	0	0	0	2	0	1	0	0
ENSMUSG00000045746	B230317F23I	0	0	0	1	1	1	0	0	1	1	1	2
ENSMUSG00000045751	MMS22L	4	7	6	8	2	6	27	40	30	33	22	22
ENSMUSG00000045752	TSSC4	3	4	3	3	12	8	44	36	32	26	25	26
ENSMUSG00000045757	ZFP764	3	0	4	1	0	2	7	8	7	3	9	7
ENSMUSG00000045761	TOGARAM2	0	0	0	0	0	0	0	1	0	0	1	0
ENSMUSG00000045767	B230219D22I	17	21	26	20	17	23	72	82	47	87	60	76
ENSMUSG00000045775	SLC16A5	0	0	1	0	0	0	0	0	0	0	0	0
ENSMUSG00000045795	WHAMM	2	7	3	6	7	4	23	24	25	21	11	14
ENSMUSG00000045799	GM9800	0	0	0	0	0	0	1	1	1	0	0	1
ENSMUSG00000045813	GM9801	0	0	0	0	0	0	0	1	0	0	0	0
ENSMUSG00000045817	ZFP36L2	4	6	1	2	4	5	10	10	3	8	10	13
ENSMUSG00000045822	ZSWIM3	0	0	1	1	0	1	5	4	2	2	1	2
ENSMUSG00000045826	PTPRCAP	69	50	67	98	77	62	535	581	401	529	386	506
ENSMUSG00000045827	SERPINB9	0	0	0	0	0	1	2	2	0	3	1	1
ENSMUSG00000045854	LYRM2	2	0	5	2	0	1	12	10	11	8	7	8
ENSMUSG00000045867	CRADD	0	1	1	1	0	0	3	3	3	2	6	4
ENSMUSG00000045868	GVIN1	0	1	1	0	0	0	0	3	0	1	2	1
ENSMUSG00000045886	GM9803	2	2	3	1	2	2	4	7	5	7	5	8
ENSMUSG00000045896	PAIP2B	1	2	1	2	2	3	11	7	14	15	9	14
ENSMUSG00000045903	NPAS4	1	0	1	0	1	0	1	4	3	0	4	5
ENSMUSG00000045912	C2CD4C	0	0	0	0	0	0	0	1	0	0	0	0
ENSMUSG00000045917	TMEM268	0	1	5	1	0	0	3	4	4	5	3	3
ENSMUSG00000045932	IFIT2	0	0	2	1	0	2	8	8	6	5	3	4
ENSMUSG00000045934	MTMR11	1	0	1	0	0	0	0	1	0	0	2	0
ENSMUSG00000045942	BC049762	0	0	0	0	0	0	2	0	0	0	0	0
ENSMUSG00000045948	MRPS12	5	1	6	5	7	3	21	24	26	23	24	31

ENSMUSG00000045954	CAVIN2	0	0	0	0	0	0	0	0	0	0	0	1
ENSMUSG00000045962	WNK1	21	23	23	23	27	17	126	118	79	122	82	90
ENSMUSG00000045969	ING1	8	10	5	16	12	9	60	58	38	50	37	45
ENSMUSG00000045973	SLC25A51	4	6	7	4	6	6	25	38	22	21	26	38
ENSMUSG00000045975	C2CD2	0	2	2	1	2	0	4	3	1	8	4	4
ENSMUSG00000045980	TMEM104	0	1	2	1	2	0	3	8	4	2	6	7
ENSMUSG00000045983	EIF4G1	52	70	57	77	62	63	314	322	259	314	250	274
ENSMUSG00000045996	POLR2K	0	0	1	1	0	0	3	5	8	7	6	4
ENSMUSG00000046010	ZFP830	7	3	6	10	9	8	36	35	31	42	36	25
ENSMUSG00000046020	POFUT1	2	2	3	3	5	5	32	22	10	21	19	21
ENSMUSG00000046027	STARD5	1	1	1	3	2	2	8	10	4	5	9	6
ENSMUSG00000046031	FAM26F	0	0	1	1	1	1	7	5	5	7	8	3
ENSMUSG00000046032	SNX12	0	1	3	3	5	2	12	21	14	25	17	17
ENSMUSG00000046034	OTULIN	4	0	3	7	7	2	16	19	19	24	14	24
ENSMUSG00000046057	GM15428	0	0	1	0	0	0	0	0	0	0	1	0
ENSMUSG00000046062	PPP1R15B	19	18	18	29	17	23	67	90	60	91	67	72
ENSMUSG00000046079	LRRC8D	1	0	2	1	2	3	10	11	4	8	11	4
ENSMUSG00000046096	BC030336	5	5	7	10	6	6	33	46	31	48	22	31
ENSMUSG00000046101	MCMDC2	13	15	16	20	17	14	58	52	52	66	54	48
ENSMUSG00000046110	SERINC4	1	0	2	1	0	0	0	2	1	2	2	1
ENSMUSG00000046111	CEP295	19	17	17	33	19	19	94	104	58	125	78	86
ENSMUSG00000046138	9930021J03R	11	17	24	18	9	20	44	51	41	79	34	40
ENSMUSG00000046139	PATL1	5	10	4	16	2	7	39	28	21	18	18	26
ENSMUSG00000046157	TMEM229B	2	5	2	6	1	5	19	23	15	22	10	16
ENSMUSG00000046168	KCNRG	1	0	0	0	0	0	0	0	0	0	0	1
ENSMUSG00000046169	ADAMTS6	2	4	3	1	1	2	14	7	6	5	6	6
ENSMUSG00000046179	E2F8	7	8	12	9	10	8	49	49	35	47	18	43
ENSMUSG00000046185	ZFP84	1	2	2	3	4	3	10	9	3	16	9	8
ENSMUSG00000046201	SCAF8	5	8	8	13	11	13	38	42	36	50	38	38
ENSMUSG00000046207	PIK3R6	0	0	0	1	0	0	0	1	0	0	0	0
ENSMUSG00000046213	CYM	0	0	0	0	0	0	1	0	0	1	2	0
ENSMUSG00000046219	C230012O17I	0	0	0	0	0	0	0	2	0	0	0	0
ENSMUSG00000046223	PLAUR	0	0	0	0	0	0	1	0	0	0	0	0
ENSMUSG00000046229	SCAND1	12	18	23	14	18	15	110	113	85	90	71	112
ENSMUSG00000046230	VPS13A	13	17	12	25	14	18	68	63	42	72	67	53
ENSMUSG00000046240	HEPACAM	0	0	0	0	0	0	0	1	0	0	0	0
ENSMUSG00000046245	PILRA	1	2	2	1	0	1	6	11	5	16	7	8
ENSMUSG00000046269	USP27X	0	0	0	0	0	0	1	0	2	0	0	0
ENSMUSG00000046280	SHE	0	0	1	0	0	0	0	0	0	1	0	0
ENSMUSG00000046295	ANKLE1	1	3	5	10	5	4	30	29	27	31	25	21
ENSMUSG00000046311	ZFP62	8	8	5	11	7	6	61	48	43	42	28	27
ENSMUSG00000046312	AI464131	0	0	0	0	0	0	1	0	0	0	0	0
ENSMUSG00000046324	ERMP1	2	1	2	4	1	5	13	14	12	11	8	15
ENSMUSG00000046329	SLC25A23	20	24	29	41	44	31	128	170	131	150	104	149
ENSMUSG00000046330	RPL37A	252	313	308	378	390	311	1372	1459	1071	1287	1038	1305
ENSMUSG00000046337	FAM178B	0	0	0	0	1	0	1	0	0	3	0	0
ENSMUSG00000046345	SMCO1	0	0	0	0	0	0	0	0	0	1	1	0
ENSMUSG00000046351	ZFP322A	5	9	11	4	9	15	38	49	34	52	31	34
ENSMUSG00000046364	RPL27A	25	31	30	25	39	44	146	170	112	150	131	143
ENSMUSG00000046378	ASPHD1	1	1	0	0	0	0	2	1	0	4	0	0
ENSMUSG00000046380	JRK	0	0	0	0	1	0	0	2	0	1	0	0
ENSMUSG00000046388	GM9806	0	2	0	0	0	1	1	1	2	3	2	1
ENSMUSG00000046402	RBP1	1	0	0	0	0	0	1	0	0	0	1	0
ENSMUSG00000046404	YOD1	8	4	4	7	8	4	26	25	16	26	11	22
ENSMUSG00000046415	B430212C06F	0	0	0	1	1	0	0	0	1	3	0	0
ENSMUSG00000046417	LRRC75A	0	0	0	0	1	0	0	0	0	0	0	0
ENSMUSG00000046431	OLFR518	0	0	0	1	0	0	0	0	0	0	0	0
ENSMUSG00000046432	BEX3	19	13	26	26	25	17	167	171	110	168	112	123

ENSMUSG00000046434	HNRNPA1	13	17	17	23	11	14	74	95	68	77	71	80
ENSMUSG00000046441	CMTR2	1	1	1	2	0	0	4	7	4	10	4	4
ENSMUSG00000046442	PPM1E	0	0	0	0	0	0	0	0	0	1	0	0
ENSMUSG00000046447	CAMK2N1	21	28	17	30	31	18	99	87	65	93	68	66
ENSMUSG00000046456	TMEM150B	0	0	0	0	0	0	0	0	0	0	0	1
ENSMUSG00000046487	MOSPD4	0	0	0	0	0	0	1	0	0	0	0	0
ENSMUSG00000046516	COX17	25	18	27	10	24	23	101	126	105	93	56	62
ENSMUSG00000046519	GOLPH3L	1	6	3	2	8	4	21	26	16	30	31	20
ENSMUSG00000046523	KCTD4	0	0	0	0	0	0	0	0	2	0	0	0
ENSMUSG00000046541	ZFP526	0	0	1	0	0	0	5	4	3	1	1	2
ENSMUSG00000046546	FAM43A	0	1	0	0	0	1	0	1	0	0	0	0
ENSMUSG00000046550	SPIN2C	2	3	3	1	2	4	14	13	8	9	6	10
ENSMUSG00000046556	ZFP319	0	0	1	0	4	3	10	10	5	15	6	4
ENSMUSG00000046562	UNC119B	6	9	6	6	9	5	41	57	32	50	29	41
ENSMUSG00000046567	4930430F08R	1	0	1	2	3	1	13	14	9	9	9	12
ENSMUSG00000046573	LYRM4	15	17	10	19	11	13	39	33	28	33	28	30
ENSMUSG00000046574	PRR12	3	4	5	7	6	6	17	20	16	18	15	20
ENSMUSG00000046580	GM7862	0	0	0	0	0	0	1	0	0	0	0	0
ENSMUSG00000046591	TICRR	12	17	20	17	18	11	50	52	32	50	31	41
ENSMUSG00000046598	BDH1	2	1	8	3	3	4	15	15	13	24	21	17
ENSMUSG00000046603	TCAIM	0	1	1	1	1	0	3	8	0	4	1	4
ENSMUSG00000046605	B3GNTL1	2	2	3	1	1	4	14	10	11	22	12	9
ENSMUSG00000046620	RPS11-PS4	0	0	0	0	0	0	1	0	0	1	2	1
ENSMUSG00000046658	ZFP316	0	0	0	1	0	0	0	0	0	0	0	0
ENSMUSG00000046667	RBM12B1	1	2	3	0	0	1	9	12	3	8	8	9
ENSMUSG00000046668	CXXC5	12	9	19	23	12	19	38	67	51	59	39	49
ENSMUSG00000046671	MTFR1L	4	3	0	2	1	0	13	10	14	8	13	12
ENSMUSG00000046675	TMEM251	4	6	3	3	3	6	17	22	23	26	12	29
ENSMUSG00000046678	OLFR981	0	0	0	0	0	0	1	0	0	0	0	0
ENSMUSG00000046679	C87436	0	3	3	5	0	2	13	9	11	11	10	17
ENSMUSG00000046694	FAM46B	0	0	0	0	0	0	0	0	0	0	0	1
ENSMUSG00000046707	CSNK2A2	20	14	25	23	18	11	89	112	87	97	92	81
ENSMUSG00000046711	HMGA1	1	0	2	0	1	1	1	5	1	4	4	2
ENSMUSG00000046718	BST2	2	2	2	3	5	3	18	28	18	25	14	17
ENSMUSG00000046721	RPL14-PS1	26	28	25	31	27	34	92	103	91	114	96	83
ENSMUSG00000046722	CDC42SE1	8	7	8	13	3	13	55	61	31	61	48	64
ENSMUSG00000046727	CYSTM1	0	0	0	0	0	0	1	0	1	0	0	0
ENSMUSG00000046731	KCTD11	1	1	0	1	1	2	5	5	4	4	1	3
ENSMUSG00000046747	GM9812	0	0	0	1	0	0	1	0	0	0	0	0
ENSMUSG00000046753	CCDC66	8	14	11	10	12	10	68	56	44	64	37	34
ENSMUSG00000046756	MRPS7	6	7	10	6	7	9	37	56	39	41	45	48
ENSMUSG00000046782	TTC6	0	0	0	1	0	0	0	0	0	0	0	0
ENSMUSG00000046785	EPM2AIP1	2	8	3	3	6	2	29	21	18	25	13	21
ENSMUSG00000046791	RIOX1	7	4	8	4	13	0	38	48	25	30	19	35
ENSMUSG00000046792	ZFP787	4	2	0	2	4	3	7	13	16	10	19	8
ENSMUSG00000046806	3110062M04	0	1	1	0	1	0	9	10	11	9	4	5
ENSMUSG00000046807	LRRC75B	0	0	0	0	0	1	0	0	0	0	0	0
ENSMUSG00000046808	ATP10D	0	1	0	2	2	0	1	4	3	3	4	5
ENSMUSG00000046814	GCHFR	0	0	0	0	0	0	1	0	0	0	0	0
ENSMUSG00000046818	DDIT4L	0	0	0	0	0	0	0	0	0	1	0	0
ENSMUSG00000046822	SLC39A3	1	0	1	1	1	0	2	3	3	5	3	2
ENSMUSG00000046836	BROX	0	0	2	4	4	3	14	18	4	16	17	13
ENSMUSG00000046854	PIP5KL1	0	0	0	0	0	0	0	2	0	0	0	0
ENSMUSG00000046861	HECTD3	0	1	1	2	1	2	12	9	6	9	5	7
ENSMUSG00000046862	PRAMEF8	0	2	1	0	1	1	5	6	5	6	5	6
ENSMUSG00000046865	FBL	11	8	13	13	12	9	86	71	72	79	61	77
ENSMUSG00000046873	MBTPS2	4	6	3	2	5	3	22	26	14	19	13	19
ENSMUSG00000046876	ATXN1	6	9	6	11	2	13	23	39	34	37	23	28

ENSMUSG00000046879	IRGM1	0	0	2	1	2	0	2	2	3	3	0	1
ENSMUSG00000046897	ZFP740	28	28	28	18	23	27	81	96	76	98	64	91
ENSMUSG00000046908	LTB4R1	0	0	0	0	0	0	1	0	0	0	0	0
ENSMUSG00000046909	TEFM	1	0	1	1	0	1	14	8	6	8	8	8
ENSMUSG00000046934	CSL	0	0	0	0	0	0	0	0	0	0	1	0
ENSMUSG00000046947	ADCK2	1	0	1	1	0	0	3	3	2	1	1	2
ENSMUSG00000046949	NQO2	1	3	1	3	2	2	4	14	8	7	4	16
ENSMUSG00000046958	4930432E11F	0	0	0	0	0	0	1	0	0	0	0	0
ENSMUSG00000046962	ZBTB21	5	3	6	3	4	6	19	29	25	32	18	23
ENSMUSG00000046982	TSHZ1	22	38	17	33	23	31	100	93	74	135	71	86
ENSMUSG00000046985	TAPT1	4	4	5	5	4	6	26	23	17	23	20	21
ENSMUSG00000046991	WDR27	0	1	0	0	0	0	0	2	2	4	1	0
ENSMUSG00000046993	GM5637	0	0	0	0	0	0	0	1	0	0	0	0
ENSMUSG00000046994	37316	2	9	8	3	5	5	56	47	41	53	34	37
ENSMUSG00000046999	1110032F04F	0	0	0	0	1	0	0	0	0	0	0	1
ENSMUSG00000047003	ZFP41	0	0	0	0	0	1	0	0	0	0	0	0
ENSMUSG00000047022	MIPOL1	1	1	0	2	2	0	6	3	6	8	3	1
ENSMUSG00000047030	SPATA2	3	1	1	0	3	0	3	7	4	4	2	6
ENSMUSG00000047036	ZFP445	13	20	16	13	17	13	71	98	58	92	56	57
ENSMUSG00000047040	PRR15L	0	0	0	0	0	0	0	0	1	0	0	0
ENSMUSG00000047044	D030056L22F	4	12	14	17	17	10	76	61	61	70	52	77
ENSMUSG00000047045	TMEM164	0	2	8	11	2	2	16	25	24	26	19	11
ENSMUSG00000047067	DUSP28	2	2	1	1	1	1	7	5	2	4	5	6
ENSMUSG00000047084	NGRN	5	2	0	0	4	2	12	13	11	15	13	9
ENSMUSG00000047085	LRRC4B	0	0	0	0	0	0	0	0	1	1	0	1
ENSMUSG00000047090	TMEM198B	0	3	1	1	1	0	2	4	5	2	4	3
ENSMUSG00000047094	OFCC1	0	0	0	0	0	0	0	0	0	0	0	1
ENSMUSG00000047098	RNF31	3	1	1	2	3	1	10	13	6	11	4	4
ENSMUSG00000047115	FAM221A	1	1	2	5	3	1	21	7	12	23	16	12
ENSMUSG00000047117	ANKDD1B	0	0	0	0	0	0	0	1	1	2	0	1
ENSMUSG00000047123	TICAM1	3	0	0	1	3	2	11	9	5	10	7	13
ENSMUSG00000047126	CLTC	37	45	38	35	29	32	181	218	162	203	159	143
ENSMUSG00000047139	CD24A	3	0	2	0	2	1	0	0	0	0	0	0
ENSMUSG00000047141	ZFP654	4	3	7	3	4	2	21	20	17	19	27	14
ENSMUSG00000047143	DMRTA2	3	1	2	7	6	4	17	19	14	18	11	10
ENSMUSG00000047146	TET1	7	8	7	7	12	6	33	42	30	48	26	41
ENSMUSG00000047153	KHNYN	6	9	9	5	8	14	22	34	32	28	15	20
ENSMUSG00000047155	CYP4X1	0	0	0	0	0	0	0	1	0	0	0	0
ENSMUSG00000047161	CHST9	0	0	0	0	0	0	0	1	0	0	0	0
ENSMUSG00000047180	NEURL3	1	5	4	10	4	5	16	28	17	21	10	18
ENSMUSG00000047187	RAB2A	2	7	9	12	5	3	21	39	33	40	19	30
ENSMUSG00000047193	DYNC2H1	3	4	0	5	9	5	12	17	10	11	10	14
ENSMUSG00000047205	DUSP18	1	1	2	2	0	0	0	1	1	2	1	1
ENSMUSG00000047213	YTHDF3	8	8	14	16	15	9	78	71	65	94	58	52
ENSMUSG00000047215	RPL9	148	176	150	205	174	191	718	754	620	744	582	661
ENSMUSG00000047221	FAM185A	1	2	1	0	1	0	8	7	8	11	6	6
ENSMUSG00000047227	GM527	0	0	1	0	1	0	2	1	1	2	5	2
ENSMUSG00000047238	MAGEH1	1	8	5	3	7	5	39	36	28	41	18	20
ENSMUSG00000047242	TAF9B	1	0	0	1	2	1	6	6	5	5	5	5
ENSMUSG00000047246	HIST1H2BE	2	1	2	2	1	2	15	10	4	12	11	13
ENSMUSG00000047248	C2CD3	4	1	0	3	2	6	11	13	18	22	18	13
ENSMUSG00000047260	EMC6	9	16	17	18	16	15	68	96	46	72	69	80
ENSMUSG00000047264	ZFP358	1	0	1	0	1	0	4	2	1	0	1	0
ENSMUSG00000047281	SFN	1	0	1	0	1	2	4	5	6	4	4	5
ENSMUSG00000047284	NEURL4	6	7	5	2	10	8	19	19	17	21	19	20
ENSMUSG00000047343	METTL21C	2	1	1	2	2	1	4	9	3	6	4	4
ENSMUSG00000047344	LANCL3	0	0	0	1	0	0	1	0	0	0	0	2
ENSMUSG00000047347	TDG-PS	0	0	0	0	0	0	0	0	0	3	1	0

ENSMUSG00000047361	GM973	2	1	2	3	3	3	3	2	4	5	3	5
ENSMUSG00000047363	CSTAD	0	0	0	0	1	0	1	0	0	1	0	0
ENSMUSG00000047368	ABHD17B	18	19	18	16	13	8	117	121	76	129	67	87
ENSMUSG00000047369	DNAH14	3	3	1	9	5	8	28	37	22	25	27	27
ENSMUSG00000047371	ZFP768	0	0	0	0	0	1	2	1	0	0	1	0
ENSMUSG00000047379	B4GAT1	0	0	0	0	0	0	0	1	0	0	0	0
ENSMUSG00000047388	ATMIN	1	3	4	2	3	5	13	13	12	10	13	13
ENSMUSG00000047407	TGIF1	2	2	4	4	8	5	23	19	17	22	25	28
ENSMUSG00000047412	ZBTB44	16	12	19	12	14	21	81	82	58	70	54	47
ENSMUSG00000047415	GPR68	4	3	4	5	1	2	15	13	8	15	10	21
ENSMUSG00000047417	REXO1	6	15	5	14	19	13	50	63	49	59	47	62
ENSMUSG00000047419	CMYA5	0	1	0	0	0	0	0	0	0	1	0	0
ENSMUSG00000047423	AI837181	0	5	2	2	4	4	14	10	14	8	11	15
ENSMUSG00000047428	DLK2	0	0	0	0	0	0	0	0	0	0	0	2
ENSMUSG00000047434	XXYLT1	7	7	5	6	4	7	17	28	18	18	14	14
ENSMUSG00000047443	ERFE	0	0	0	0	0	0	0	0	0	1	0	0
ENSMUSG00000047446	ARL4A	4	8	5	7	5	8	39	35	23	37	29	20
ENSMUSG00000047454	GPHN	3	3	0	3	3	4	14	11	5	13	6	10
ENSMUSG00000047459	DYNLRB1	17	17	16	17	16	8	76	73	72	99	57	83
ENSMUSG00000047466	8030462N17I	23	30	26	25	29	24	75	100	63	85	79	63
ENSMUSG00000047473	ZFP30	2	0	1	0	0	0	8	5	2	8	4	0
ENSMUSG00000047492	INHBE	0	0	0	0	0	0	1	0	0	0	0	0
ENSMUSG00000047507	BAIAP3	1	2	0	4	1	0	3	7	6	3	7	4
ENSMUSG00000047514	TSPYL1	4	8	8	22	9	9	56	44	35	53	47	43
ENSMUSG00000047515	BC049715	0	4	1	1	0	3	7	4	5	4	2	3
ENSMUSG00000047534	MIS18BP1	14	28	22	37	28	26	133	157	115	182	123	113
ENSMUSG00000047539	FBXO28	2	2	6	6	6	4	18	21	22	25	14	24
ENSMUSG00000047547	CLTB	5	8	3	8	12	6	32	35	25	27	41	35
ENSMUSG00000047554	TMEM41B	4	2	0	0	6	4	15	22	8	26	14	14
ENSMUSG00000047557	LXN	0	0	0	0	0	0	0	1	0	0	0	0
ENSMUSG00000047565	ACOT10	0	0	0	0	0	0	0	0	0	0	1	0
ENSMUSG00000047583	TYW3	0	0	0	1	0	0	3	2	1	1	1	0
ENSMUSG00000047603	ZFP235	2	0	1	0	0	2	0	1	4	0	4	1
ENSMUSG00000047604	FRAT2	2	2	0	3	0	4	11	19	11	14	8	15
ENSMUSG00000047613	A430005L14F	4	1	1	3	6	2	19	12	13	12	15	17
ENSMUSG00000047617	PAXX	0	2	2	2	3	0	20	21	12	23	17	19
ENSMUSG00000047632	FGFBP3	0	1	1	1	1	0	2	13	4	10	8	2
ENSMUSG00000047635	2810006K23F	0	2	0	1	1	3	9	10	8	18	6	11
ENSMUSG00000047648	FBXO30	3	9	4	3	1	5	24	17	11	24	17	23
ENSMUSG00000047649	CD3EAP	17	25	17	20	15	15	103	93	87	94	100	108
ENSMUSG00000047654	TSSK6	0	0	0	0	0	0	1	1	0	0	0	0
ENSMUSG00000047656	TRPT1	3	1	0	0	0	0	1	4	3	5	8	6
ENSMUSG00000047669	MSL3L2	0	1	1	1	0	0	3	6	7	2	1	4
ENSMUSG00000047675	RPS8	888	982	953	1193	1153	1144	3718	3973	2903	3595	3024	3411
ENSMUSG00000047676	RPSA-PS10	0	2	4	7	4	2	30	20	10	22	14	25
ENSMUSG00000047678	GPR82	0	1	1	0	0	0	0	0	0	0	1	0
ENSMUSG00000047694	YIPF6	5	5	4	4	8	1	14	29	20	23	11	16
ENSMUSG00000047710	CHAMP1	5	5	7	6	4	6	29	24	27	22	26	25
ENSMUSG00000047712	UST	5	12	6	6	10	6	39	33	26	44	27	20
ENSMUSG00000047714	PPP1R2	5	2	5	2	3	7	31	23	22	34	19	24
ENSMUSG00000047719	UBIAD1	0	1	1	1	4	2	7	9	5	6	5	3
ENSMUSG00000047720	4922502D21I	0	0	0	0	0	0	0	1	0	0	0	0
ENSMUSG00000047721	BOLA2	11	15	20	13	22	25	125	136	90	120	99	110
ENSMUSG00000047731	WBP1L	7	3	6	10	6	13	29	41	20	41	16	19
ENSMUSG00000047733	TMEM262	0	0	1	0	0	0	0	1	0	0	0	0
ENSMUSG00000047735	SAMD9L	9	10	17	30	20	23	101	89	46	113	61	57
ENSMUSG00000047749	ZC3HAV1L	1	1	3	4	9	2	18	16	9	23	14	11
ENSMUSG00000047757	FANCB	2	5	3	7	6	5	23	25	13	18	16	12

ENSMUSG00000047767	ATG16L2	6	9	9	6	7	6	25	24	17	22	22	26
ENSMUSG00000047777	PHF13	3	4	5	2	5	1	6	14	8	18	8	7
ENSMUSG00000047786	LIX1	0	0	0	1	1	0	0	0	1	0	0	1
ENSMUSG00000047789	SLC38A9	4	7	4	6	6	6	21	21	17	12	18	15
ENSMUSG00000047793	SNED1	0	0	0	0	0	0	0	0	0	0	1	1
ENSMUSG00000047804	AKAP10	2	1	2	3	3	2	14	14	9	6	3	7
ENSMUSG00000047810	CCDC88B	10	16	17	18	21	11	66	65	56	75	71	64
ENSMUSG00000047819	TIGD4	0	0	0	0	0	0	0	1	0	0	0	0
ENSMUSG00000047824	PYGO2	5	9	4	12	7	5	44	43	37	38	38	53
ENSMUSG00000047832	CDCA4	6	13	10	13	7	10	42	41	47	63	28	41
ENSMUSG00000047843	BRI3	0	0	2	0	1	0	1	1	1	4	0	2
ENSMUSG00000047854	STX19	0	0	0	0	0	0	0	0	0	0	1	0
ENSMUSG00000047866	LONP2	4	9	8	10	6	4	42	41	29	34	33	42
ENSMUSG00000047867	GIMAP6	27	45	36	41	41	36	225	179	150	220	158	161
ENSMUSG00000047875	GPR157	0	0	0	0	0	0	0	1	0	0	0	0
ENSMUSG00000047878	A4GALT	0	0	0	0	0	1	0	0	0	0	0	0
ENSMUSG00000047879	USP14	12	14	11	26	18	12	90	87	55	99	53	73
ENSMUSG00000047880	CXCR5	1	1	0	0	0	0	2	3	0	1	2	0
ENSMUSG00000047881	RELL1	31	24	29	24	28	26	59	77	49	83	65	67
ENSMUSG00000047888	TNRC6B	19	29	31	43	35	34	105	122	85	152	85	111
ENSMUSG00000047898	CCR4	0	0	0	0	0	0	1	1	0	0	0	0
ENSMUSG00000047905	GM8566	0	1	0	1	0	0	1	5	0	4	4	1
ENSMUSG00000047909	ANKRD16	2	9	17	6	11	12	46	52	29	57	43	49
ENSMUSG00000047911	NPM2	1	0	0	0	0	0	3	0	0	2	2	0
ENSMUSG00000047921	TRAPPC9	2	3	1	2	1	4	15	30	10	13	14	19
ENSMUSG00000047935	SOX1OT	0	0	0	0	0	0	0	0	0	1	0	0
ENSMUSG00000047940	STPG2	0	0	0	0	0	0	0	0	1	0	0	0
ENSMUSG00000047945	MARCKSL1	4	1	2	3	1	5	10	16	9	22	4	12
ENSMUSG00000047953	GP5	0	0	0	0	0	0	0	1	1	2	1	1
ENSMUSG00000047959	KCNA3	1	1	0	1	0	0	0	0	0	0	2	0
ENSMUSG00000047963	STBD1	0	1	3	1	2	1	8	3	7	4	2	4
ENSMUSG00000047965	RPL9-PS7	1	0	1	0	1	1	2	0	0	0	0	1
ENSMUSG00000047986	PALM3	0	1	0	3	1	0	5	4	3	5	6	10
ENSMUSG00000047989	INO80C	2	3	4	4	5	3	22	38	21	20	15	25
ENSMUSG00000047996	PRRG1	3	8	3	6	3	2	28	24	15	20	18	14
ENSMUSG00000048000	GIGYF2	9	6	11	22	14	22	67	77	46	80	42	63
ENSMUSG00000048003	CATSPER4	0	0	0	0	0	0	0	0	0	1	0	0
ENSMUSG00000048007	TIMM8A1	0	0	0	2	0	0	2	5	1	0	0	1
ENSMUSG00000048012	ZFP473	0	0	0	0	0	0	0	0	0	1	1	1
ENSMUSG00000048029	ENO4	0	0	0	0	0	0	1	0	0	0	0	0
ENSMUSG00000048039	ISG20L2	10	12	14	18	11	22	79	78	69	102	57	46
ENSMUSG00000048040	ARXES2	2	1	2	5	2	3	12	16	19	29	9	15
ENSMUSG00000048047	ZBTB33	3	2	5	3	2	5	21	26	9	22	11	14
ENSMUSG00000048058	LDLRAD3	0	0	0	1	0	0	0	1	1	0	2	0
ENSMUSG00000048076	ARF1	11	21	13	26	17	27	128	132	105	113	105	113
ENSMUSG00000048087	GM4737	2	3	3	5	4	6	22	11	10	7	10	16
ENSMUSG00000048100	TAF13	1	0	2	0	3	2	6	10	5	7	4	7
ENSMUSG00000048106	4632415L05R	11	11	11	15	9	14	56	64	39	40	48	44
ENSMUSG00000048109	RBM15	46	40	37	60	39	55	177	197	153	202	150	157
ENSMUSG00000048118	ARID4A	13	26	21	26	29	16	103	93	82	104	87	97
ENSMUSG00000048142	NAT8L	0	0	0	0	0	0	0	0	0	1	0	0
ENSMUSG00000048148	NWD1	0	0	0	1	0	0	4	1	2	5	2	3
ENSMUSG00000048154	KMT2D	40	52	47	56	48	57	181	221	141	242	154	188
ENSMUSG00000048163	SELPLG	4	3	3	2	3	2	15	16	15	15	6	12
ENSMUSG00000048164	GM7204	0	0	0	0	0	0	0	1	0	0	0	0
ENSMUSG00000048170	MCMBP	36	43	45	57	38	43	189	170	126	205	120	152
ENSMUSG00000048174	TMEM81	1	2	1	4	1	3	9	4	4	6	1	7
ENSMUSG00000048175	ASB8	2	5	3	1	2	2	14	5	8	14	10	12

ENSMUSG00000048185	GM7075	0	0	0	0	0	0	0	0	0	1	0	0
ENSMUSG00000048191	MUC6	0	0	0	0	0	0	0	1	0	0	0	0
ENSMUSG00000048218	AMIGO2	0	0	0	0	1	0	2	1	0	0	1	1
ENSMUSG00000048222	MFAP1B	12	18	15	16	11	11	88	100	74	90	56	79
ENSMUSG00000048226	OLFR1212	0	0	0	0	0	0	0	0	1	0	0	0
ENSMUSG00000048232	FBXO10	0	1	0	0	1	1	2	5	2	7	4	2
ENSMUSG00000048234	RNF149	0	0	2	2	2	0	6	7	5	9	6	5
ENSMUSG00000048240	GNG7	0	0	0	0	0	0	0	0	0	0	0	1
ENSMUSG00000048249	CREBRF	173	183	202	293	215	235	844	882	615	996	600	709
ENSMUSG00000048251	RIT1	7	23	14	19	14	21	49	43	42	75	37	33
ENSMUSG00000048264	DIP2C	3	3	3	8	1	4	24	37	19	22	26	16
ENSMUSG00000048271	RBM33	9	9	9	11	9	9	41	48	35	61	45	37
ENSMUSG00000048277	SYNGR2	8	12	7	14	22	10	72	83	50	71	46	54
ENSMUSG00000048279	SACS	8	11	22	17	19	8	59	64	30	64	39	47
ENSMUSG00000048280	ZFP738	3	0	1	4	2	4	15	11	7	16	7	11
ENSMUSG00000048281	DLEU7	1	0	0	0	0	0	0	0	0	0	0	0
ENSMUSG00000048285	FRMD6	4	7	2	3	4	1	7	17	14	17	16	10
ENSMUSG00000048307	ANKRD46	1	2	2	2	3	2	13	14	5	4	8	7
ENSMUSG00000048310	PSKH1	0	1	2	1	1	1	9	8	6	9	8	6
ENSMUSG00000048327	CKAP2L	6	11	10	11	9	10	46	54	42	62	54	46
ENSMUSG00000048330	RIC3	0	0	0	0	0	0	0	0	1	0	0	0
ENSMUSG00000048334	GM8258	1	1	0	0	1	0	0	1	0	0	1	1
ENSMUSG00000048349	POU4F1	3	4	1	3	12	9	30	34	25	34	23	27
ENSMUSG00000048351	COA7	3	1	5	4	5	5	15	18	12	12	18	21
ENSMUSG00000048368	OMD	0	0	1	1	0	0	1	3	0	1	0	0
ENSMUSG00000048371	PDP2	14	9	5	12	7	9	45	46	43	51	31	42
ENSMUSG00000048376	F2R	0	0	0	2	1	1	1	1	3	3	0	1
ENSMUSG00000048379	SOCS4	5	13	3	9	5	8	35	30	28	45	19	24
ENSMUSG00000048388	FAM171B	6	20	12	16	11	21	38	39	22	37	33	37
ENSMUSG00000048398	GM5065	1	0	1	0	0	0	0	0	0	1	0	0
ENSMUSG00000048406	B330016D10I	0	0	0	0	1	1	0	1	1	0	1	0
ENSMUSG00000048410	ZFP407	3	1	1	2	1	1	8	6	1	13	5	4
ENSMUSG00000048429	TIMM29	3	2	3	5	3	3	30	23	21	31	29	20
ENSMUSG00000048439	NUPL2	1	1	0	1	0	0	7	5	2	9	3	7
ENSMUSG00000048440	CYP4F16	0	0	0	0	1	0	1	3	0	0	1	0
ENSMUSG00000048445	CCDC57	0	1	0	0	0	0	0	1	0	1	2	1
ENSMUSG00000048458	FAM212B	0	1	0	0	0	1	0	0	0	0	1	0
ENSMUSG00000048473	SULT6B2	0	0	0	0	0	1	0	1	0	0	1	0
ENSMUSG00000048481	MYPOP	1	2	2	1	2	3	1	7	9	1	3	6
ENSMUSG00000048483	ZDHHC22	0	0	0	0	0	1	0	0	0	0	0	0
ENSMUSG00000048486	FITM2	0	1	0	0	1	0	4	4	3	2	2	3
ENSMUSG00000048490	NRIP1	15	17	15	21	21	11	58	69	36	48	53	53
ENSMUSG00000048495	TYW5	10	14	13	15	14	16	84	76	48	90	62	55
ENSMUSG00000048497	MMGT2	7	4	7	7	3	2	21	39	30	28	18	21
ENSMUSG00000048520	FBXL13	0	0	0	0	0	0	0	0	0	1	0	0
ENSMUSG00000048521	CXCR6	8	18	12	15	16	15	39	52	34	56	34	43
ENSMUSG00000048534	JAML	3	3	9	6	8	3	29	23	18	23	14	18
ENSMUSG00000048537	PHLDB1	0	0	0	0	0	0	0	0	0	1	0	0
ENSMUSG00000048540	NHLH2	0	0	0	1	0	0	0	0	0	0	0	1
ENSMUSG00000048546	TOB2	10	8	5	4	10	7	38	25	15	42	13	32
ENSMUSG00000048550	THNSL1	2	0	1	1	1	0	15	12	7	16	9	10
ENSMUSG00000048562	SP8	1	6	3	7	5	1	16	11	11	17	10	16
ENSMUSG00000048574	CCNB1-PS	0	0	0	1	0	0	0	0	0	0	0	0
ENSMUSG00000048578	MLEC	19	21	22	29	16	29	95	108	80	107	68	89
ENSMUSG00000048581	E130311K13F	2	3	2	3	2	2	23	13	11	16	15	7
ENSMUSG00000048602	MORC2B	0	0	0	0	1	0	0	0	0	0	0	0
ENSMUSG00000048603	GM9828	0	0	0	0	0	0	2	1	2	0	2	3
ENSMUSG00000048620	OLFR1336	0	0	0	0	0	0	1	0	0	0	0	0

ENSMUSG00000048644	CTXN1	0	0	1	1	0	1	1	2	0	1	2	0
ENSMUSG00000048647	EXD1	0	0	0	0	1	0	0	0	0	0	0	1
ENSMUSG00000048652	SAMD13	2	1	5	2	3	3	11	13	7	23	14	7
ENSMUSG00000048655	CCDC169	0	0	0	0	0	0	0	1	0	1	2	1
ENSMUSG00000048661	LEMD3	4	1	4	3	11	8	26	26	22	43	22	21
ENSMUSG00000048668	RHNO1	8	13	17	12	12	13	51	65	39	59	32	55
ENSMUSG00000048677	TPCN2	2	1	3	0	2	0	2	5	3	2	3	2
ENSMUSG00000048696	MEX3D	0	4	0	3	2	3	20	30	23	29	20	24
ENSMUSG00000048701	CCDC6	5	9	6	8	4	8	39	44	37	58	35	33
ENSMUSG00000048707	TPRN	4	5	6	2	1	5	19	22	12	15	18	21
ENSMUSG00000048731	GGNBP1	0	0	1	0	1	0	0	0	2	1	0	0
ENSMUSG00000048732	KLHL11	0	1	0	1	0	1	1	3	1	3	1	1
ENSMUSG00000048755	MCAT	1	0	0	3	0	0	8	3	6	6	2	5
ENSMUSG00000048756	FOXO3	24	35	35	42	39	38	97	77	83	87	57	80
ENSMUSG00000048758	RPL29	22	44	53	47	32	50	265	308	212	251	198	231
ENSMUSG00000048764	TMPRSS11F	0	0	0	0	0	0	0	0	0	1	0	0
ENSMUSG00000048772	TMEM53	0	0	0	1	0	0	0	0	1	0	0	0
ENSMUSG00000048787	DCUN1D3	1	0	0	3	0	0	8	5	4	8	4	6
ENSMUSG00000048796	CYB561D1	1	0	1	1	2	1	5	7	1	7	4	3
ENSMUSG00000048799	CEP120	5	5	4	2	3	3	22	30	21	20	12	22
ENSMUSG00000048807	SLC35E4	0	0	1	1	2	0	0	0	0	2	2	2
ENSMUSG00000048827	PKD1L3	0	0	0	1	1	0	3	2	1	3	2	2
ENSMUSG00000048832	VPS37C	2	2	4	1	1	2	5	12	6	5	5	8
ENSMUSG00000048833	SLC39A9	1	7	5	4	6	7	24	26	14	24	15	19
ENSMUSG00000048856	SLC25A47	0	0	0	0	0	0	2	0	0	0	0	2
ENSMUSG00000048865	ARHGAP30	25	29	24	53	33	26	175	162	140	156	111	166
ENSMUSG00000048874	PHF3	30	28	31	33	31	34	147	126	105	172	92	130
ENSMUSG00000048878	HEXIM1	9	6	5	7	9	9	52	41	35	68	53	45
ENSMUSG00000048895	CDK5R1	1	3	6	1	5	0	15	7	10	24	11	7
ENSMUSG00000048897	ZFP710	3	2	0	0	2	2	8	9	5	11	4	11
ENSMUSG00000048905	4930539E08F	1	0	0	1	0	0	1	0	1	4	0	0
ENSMUSG00000048911	RNF24	3	0	0	0	2	1	6	7	5	9	2	4
ENSMUSG00000048915	EFNA5	0	0	0	0	0	0	1	0	0	0	1	0
ENSMUSG00000048916	GM5540	0	0	0	0	0	0	1	0	0	0	0	0
ENSMUSG00000048920	FKRP	1	1	4	1	3	4	15	19	11	22	16	20
ENSMUSG00000048921	ZFP689	0	0	0	0	1	0	2	4	3	1	1	4
ENSMUSG00000048922	CDCA2	3	1	2	2	2	2	15	17	13	23	12	19
ENSMUSG00000048924	CCDC125	4	4	3	6	5	7	27	27	23	32	17	23
ENSMUSG00000048930	TADA3	2	4	1	1	1	1	11	7	10	13	7	19
ENSMUSG00000048938	NR1H5	0	0	0	0	0	1	0	0	0	0	0	0
ENSMUSG00000048960	PREX2	0	1	0	0	1	0	1	1	0	1	0	0
ENSMUSG00000048967	YJEFN3	1	0	1	0	0	0	2	0	3	2	1	1
ENSMUSG00000048970	C1GALT1C1	1	2	2	3	1	1	24	16	9	9	15	21
ENSMUSG00000048992	PRSS32	0	3	2	2	1	0	4	4	1	5	3	4
ENSMUSG00000048997	ATXN7L2	0	1	2	1	3	2	4	7	7	9	11	11
ENSMUSG00000049001	NDNF	0	1	0	0	0	2	1	3	2	0	1	3
ENSMUSG00000049011	OLFR729	0	0	0	0	0	0	0	0	1	0	0	0
ENSMUSG00000049036	TMEM121	1	2	2	3	0	0	11	14	9	12	10	11
ENSMUSG00000049037	CLEC4A1	0	0	1	0	0	0	0	1	0	1	1	0
ENSMUSG00000049038	MTERF2	2	1	2	3	2	2	13	14	5	11	8	12
ENSMUSG00000049076	ACAP2	8	11	10	13	7	10	56	46	47	64	35	47
ENSMUSG00000049090	ZADH2	7	8	7	7	9	4	31	30	24	26	15	21
ENSMUSG00000049091	SEPHS2	47	62	67	81	83	56	429	441	297	428	336	417
ENSMUSG00000049092	GPR137C	0	0	0	0	1	0	1	2	2	2	0	1
ENSMUSG00000049103	CCR2	1	1	4	1	1	2	3	6	1	2	2	3
ENSMUSG00000049106	DCAF5	5	14	10	9	6	9	53	47	27	51	31	49
ENSMUSG00000049109	THEMIS	1	1	0	2	0	1	15	14	16	15	12	11
ENSMUSG00000049115	AGTR1A	1	0	0	0	0	0	4	3	5	5	3	1

ENSMUSG00000049122	FRMD3	0	0	0	0	0	0	0	0	1	0	0	0
ENSMUSG00000049124	GM8186	0	0	0	0	1	1	11	6	7	11	11	11
ENSMUSG00000049149	OLFR1258	0	0	0	0	0	0	0	0	0	0	1	0
ENSMUSG00000049154	FAM183B	0	0	0	0	0	0	1	1	0	1	0	0
ENSMUSG00000049160	TEX50	1	0	0	0	0	0	0	0	0	0	1	0
ENSMUSG00000049164	ZFP518A	4	3	4	4	5	8	19	32	24	34	20	25
ENSMUSG00000049176	FRMPD4	0	0	0	0	0	0	0	0	0	1	0	1
ENSMUSG00000049184	PURG	0	1	1	3	1	1	4	6	2	4	3	0
ENSMUSG00000049225	PDP1	3	2	1	1	4	4	10	6	8	3	5	7
ENSMUSG00000049230	GM9833	0	0	0	0	0	2	0	0	1	1	0	2
ENSMUSG00000049231	GM12497	0	0	0	0	0	0	0	1	0	0	0	1
ENSMUSG00000049232	TIGD2	7	13	4	13	12	6	37	28	26	35	28	25
ENSMUSG00000049235	GM7324	5	9	8	3	5	7	11	15	10	20	6	7
ENSMUSG00000049246	HSPE1-PS2	0	0	0	1	0	0	0	0	0	0	0	0
ENSMUSG00000049285	MBLAC1	0	0	2	1	0	2	3	4	3	5	3	4
ENSMUSG00000049287	IBA57	1	2	0	0	1	2	6	4	3	2	7	2
ENSMUSG00000049288	LIX1L	0	2	0	2	1	0	3	7	5	7	3	4
ENSMUSG00000049295	ZFP219	4	3	9	6	7	5	14	20	20	23	18	24
ENSMUSG00000049299	TRAPPC1	3	3	3	6	1	3	18	13	13	14	16	16
ENSMUSG00000049300	PRMT6	1	2	1	1	1	2	11	14	10	21	6	9
ENSMUSG00000049305	CCDC71	2	1	1	1	2	1	4	16	11	14	11	8
ENSMUSG00000049313	SORL1	1	2	1	0	0	1	2	2	1	3	2	2
ENSMUSG00000049323	SMCR8	5	5	6	5	5	9	28	28	19	33	26	29
ENSMUSG00000049327	KMT5A	77	106	86	101	134	104	511	545	389	561	404	453
ENSMUSG00000049336	TENM2	0	0	0	0	0	0	0	0	1	0	0	0
ENSMUSG00000049339	RETREG2	6	8	9	5	6	3	39	39	29	48	18	29
ENSMUSG00000049350	ZG16	0	0	0	1	0	0	0	0	0	0	0	0
ENSMUSG00000049353	RD3	0	0	0	0	0	0	0	0	1	0	0	1
ENSMUSG00000049354	DCAF7	4	7	6	5	12	7	35	29	28	29	24	33
ENSMUSG00000049357	4933408B17F	3	2	2	1	2	1	5	3	3	3	2	5
ENSMUSG00000049387	COX7B2	0	0	0	0	0	0	1	0	0	0	0	0
ENSMUSG00000049396	GEMIN4	0	0	0	0	0	0	1	0	1	4	2	0
ENSMUSG00000049401	OGFR	5	8	9	6	8	7	28	44	28	34	32	36
ENSMUSG00000049404	RARRES1	0	0	0	0	0	1	0	0	0	0	0	0
ENSMUSG00000049409	PROKR1	0	0	0	0	0	0	0	0	1	0	0	0
ENSMUSG00000049411	TMEM241	1	0	0	0	1	1	6	4	4	3	4	6
ENSMUSG00000049421	ZFP260	5	8	10	6	9	5	39	46	21	33	29	35
ENSMUSG00000049422	CHCHD10	0	0	0	1	0	0	1	0	1	0	0	0
ENSMUSG00000049439	CYP20A1	1	3	3	3	5	2	28	30	20	23	22	23
ENSMUSG00000049470	AFF4	8	9	8	7	8	8	49	52	25	63	33	34
ENSMUSG00000049482	CTU2	10	10	17	11	18	16	56	61	49	62	54	52
ENSMUSG00000049488	TMEM67	0	1	0	2	1	1	3	6	2	2	3	3
ENSMUSG00000049489	FAM58B	0	3	3	4	2	1	12	15	6	23	6	10
ENSMUSG00000049493	PLS1	1	0	0	1	0	0	0	1	0	2	3	3
ENSMUSG00000049502	DTX3L	5	2	4	2	3	2	11	17	14	16	8	19
ENSMUSG00000049504	PROSER1	2	6	3	9	9	8	22	35	21	24	19	23
ENSMUSG00000049516	SPTY2D1	7	9	6	12	7	8	31	40	20	39	24	25
ENSMUSG00000049517	RPS23	78	110	93	122	140	98	695	770	569	690	618	617
ENSMUSG00000049532	SALL2	0	1	0	0	0	1	0	0	0	0	0	0
ENSMUSG00000049550	CLIP1	20	32	30	36	26	30	135	134	103	183	134	123
ENSMUSG00000049553	POLR1A	14	37	25	22	15	24	72	75	66	67	56	63
ENSMUSG00000049556	LINGO1	0	0	0	0	0	0	1	0	0	0	0	0
ENSMUSG00000049562	AP5B1	0	0	0	1	1	0	3	1	2	2	0	4
ENSMUSG00000049571	CFAP46	3	0	0	2	1	0	1	2	1	2	0	2
ENSMUSG00000049577	ZFPM1	10	9	4	17	10	11	39	37	41	39	33	43
ENSMUSG00000049580	TSKU	0	0	0	0	1	0	0	0	0	0	0	0
ENSMUSG00000049588	CCDC69	0	0	0	1	0	0	2	2	4	0	0	1
ENSMUSG00000049600	ZBTB45	0	3	2	3	2	1	8	11	3	8	7	7

ENSMUSG00000049606	ZFP644	11	15	8	26	15	17	78	65	51	75	40	60
ENSMUSG00000049608	GPR55	0	0	0	0	0	0	0	0	0	1	0	2
ENSMUSG00000049612	OMG	0	0	1	0	0	1	0	0	0	1	0	2
ENSMUSG00000049624	SLC17A5	2	1	2	5	5	1	8	7	10	14	11	7
ENSMUSG00000049625	TIFAB	0	0	1	0	0	0	0	0	0	0	0	0
ENSMUSG00000049630	C1QL3	0	0	0	0	0	1	0	0	0	0	0	0
ENSMUSG00000049643	2310022A10I	1	6	6	2	3	1	20	17	11	22	14	18
ENSMUSG00000049657	ZBTB5	1	1	2	5	2	1	7	8	6	11	8	11
ENSMUSG00000049658	BDP1	23	34	12	27	30	26	110	110	83	140	60	79
ENSMUSG00000049659	AFTPH	8	21	13	17	13	16	65	81	57	58	44	51
ENSMUSG00000049670	MORN4	0	0	0	0	0	0	0	0	0	0	0	1
ENSMUSG00000049672	ZBTB14	3	3	0	0	4	0	1	5	5	4	3	2
ENSMUSG00000049680	URGCP	0	2	3	0	3	5	15	17	11	12	16	13
ENSMUSG00000049686	ORAI1	1	1	0	1	2	0	1	6	2	4	0	4
ENSMUSG00000049687	FAM109B	0	1	0	0	0	0	0	0	0	0	0	0
ENSMUSG00000049699	UCN2	0	0	0	0	0	0	0	0	1	0	0	0
ENSMUSG00000049717	LIG4	1	2	0	3	1	1	12	10	5	7	3	6
ENSMUSG00000049728	ZFP668	1	2	2	3	2	1	6	4	4	6	4	7
ENSMUSG00000049739	ZFP646	2	2	3	4	4	1	4	14	7	9	9	8
ENSMUSG00000049744	ARHGAP15	11	12	12	23	17	11	73	73	57	86	41	57
ENSMUSG00000049751	RPL36AL	8	16	13	11	15	20	47	50	45	57	37	38
ENSMUSG00000049755	ZFP672	1	4	4	4	4	2	9	6	4	17	8	8
ENSMUSG00000049760	2410015M20	18	27	27	29	29	24	186	205	152	197	122	166
ENSMUSG00000049764	ZFP280B	5	7	2	5	6	4	15	23	19	17	11	16
ENSMUSG00000049775	TMSB4X	337	429	403	564	502	475	2773	2960	1945	2691	2280	2190
ENSMUSG00000049792	BAG5	1	5	2	9	6	5	15	17	19	26	16	25
ENSMUSG00000049799	LRRC19	0	1	0	0	0	0	0	0	0	1	0	0
ENSMUSG00000049800	SERTAD2	8	21	14	11	16	16	69	73	53	62	53	54
ENSMUSG00000049804	ARMCX4	3	8	4	5	8	4	21	35	22	36	24	29
ENSMUSG00000049811	FAM161A	0	0	0	0	0	0	2	4	1	5	1	0
ENSMUSG00000049823	ZBTB12	2	0	0	1	0	0	0	2	1	0	1	0
ENSMUSG00000049832	RBX1-PS	0	0	0	1	0	0	1	0	0	0	1	1
ENSMUSG00000049858	SUOX	0	0	0	1	0	0	1	6	2	2	3	3
ENSMUSG00000049866	ARL4C	4	11	5	11	9	5	44	43	33	55	21	31
ENSMUSG00000049871	NLRC3	5	11	15	21	17	13	52	50	45	59	44	41
ENSMUSG00000049878	RLF	7	5	6	13	8	14	29	49	37	64	32	47
ENSMUSG00000049882	VCPKMT	4	2	2	2	3	1	14	16	9	5	9	14
ENSMUSG00000049892	RASD1	1	0	0	0	1	0	1	0	1	1	0	0
ENSMUSG00000049904	TMEM17	0	0	1	0	0	0	3	3	1	4	2	4
ENSMUSG00000049907	RASL11B	0	0	0	0	1	1	8	4	3	3	6	6
ENSMUSG00000049916	2610318N02I	4	2	6	1	3	5	11	20	12	12	17	17
ENSMUSG00000049922	SLC35C1	2	3	3	2	5	5	15	24	17	22	18	21
ENSMUSG00000049926	OLFR921	0	0	0	0	0	0	0	0	0	0	0	1
ENSMUSG00000049932	H2AFX	17	22	39	35	39	25	149	187	132	157	119	141
ENSMUSG00000049939	LRRC4	0	0	0	0	0	0	0	2	0	1	0	0
ENSMUSG00000049940	PGRMC2	7	19	20	15	20	16	56	62	59	57	53	53
ENSMUSG00000049950	RPP38	4	3	5	7	3	4	19	40	12	18	16	21
ENSMUSG00000049957	CCDC137	3	1	1	3	6	2	20	15	9	22	26	13
ENSMUSG00000049960	MRPS16	2	8	6	9	6	8	40	38	30	43	18	33
ENSMUSG00000049969	PLEKHF2	4	6	8	6	2	8	32	23	12	26	20	14
ENSMUSG00000050002	IDNK	4	0	1	4	5	2	26	21	10	18	19	16
ENSMUSG00000050017	PITPNB	6	10	4	5	8	8	58	51	55	61	37	39
ENSMUSG00000050022	AMZ1	0	0	0	0	0	0	0	1	1	1	1	0
ENSMUSG00000050029	RAP2C	7	8	7	7	16	11	48	64	29	65	32	35
ENSMUSG00000050035	FHL4	1	0	0	0	0	0	1	0	0	0	1	0
ENSMUSG00000050043	TMX2	7	5	6	7	3	6	21	34	29	26	25	20
ENSMUSG00000050050	CCDC158	0	1	0	0	0	0	1	0	0	0	0	2
ENSMUSG00000050052	TDRP	5	7	6	4	6	3	44	50	38	47	54	40

ENSMUSG00000050064	ZFP697	0	0	0	0	0	0	0	0	0	1	0	0
ENSMUSG00000050071	BEX1	0	0	0	0	0	0	0	0	0	0	0	1
ENSMUSG00000050074	SPINK8	0	0	0	0	0	0	2	1	2	3	0	1
ENSMUSG00000050075	GPR171	9	18	11	20	10	10	80	64	48	80	47	56
ENSMUSG00000050079	RSPRY1	2	4	9	6	7	6	23	28	18	29	19	27
ENSMUSG00000050088	1600012H06I	4	7	4	6	3	1	27	34	14	39	19	31
ENSMUSG00000050089	AKAP4	0	0	0	0	0	0	0	0	1	0	0	0
ENSMUSG00000050100	HMX2	1	0	1	1	1	1	8	5	4	5	5	4
ENSMUSG00000050106	TMC8	0	0	0	0	0	0	2	4	4	5	1	2
ENSMUSG00000050107	HASPIN	10	13	13	8	16	19	34	64	33	48	44	41
ENSMUSG00000050121	OPALIN	0	0	0	0	0	0	1	2	0	2	2	0
ENSMUSG00000050132	SARM1	0	2	0	0	0	0	1	2	0	1	2	0
ENSMUSG00000050144	SLC25A44	2	4	3	4	5	5	11	10	16	10	9	15
ENSMUSG00000050147	F2RL3	0	0	0	0	0	0	0	1	0	2	0	0
ENSMUSG00000050148	UBQLN2	2	9	4	10	8	6	50	35	37	51	23	29
ENSMUSG00000050150	SLC9B1	0	0	0	0	0	0	1	1	1	1	0	0
ENSMUSG00000050174	NUDT6	0	1	2	0	3	3	4	9	5	10	4	3
ENSMUSG00000050188	LSM10	1	3	3	0	2	4	10	12	5	9	9	14
ENSMUSG00000050192	EIF5A2	2	1	0	0	2	2	7	3	1	5	5	2
ENSMUSG00000050195	SCD4	0	0	0	1	0	0	0	1	0	2	1	0
ENSMUSG00000050199	LGR4	13	15	15	16	14	6	53	73	58	65	52	50
ENSMUSG00000050212	EVA1B	1	0	0	0	2	0	0	0	1	0	0	0
ENSMUSG00000050213	SNIP1	6	8	6	3	11	7	38	37	24	42	40	26
ENSMUSG00000050229	PIGM	11	7	12	8	7	7	38	59	32	46	36	44
ENSMUSG00000050240	HIC2	5	1	6	4	5	4	12	12	7	17	5	11
ENSMUSG00000050241	KLRE1	0	1	1	2	1	1	1	6	5	5	3	6
ENSMUSG00000050244	HEATR1	10	8	12	9	8	11	28	42	35	45	36	60
ENSMUSG00000050271	PRAG1	0	0	1	1	3	0	6	10	5	8	3	6
ENSMUSG00000050295	FOXC1	0	0	0	0	0	0	1	0	0	0	0	1
ENSMUSG00000050299	GM9843	21	30	34	35	31	16	105	108	60	96	76	85
ENSMUSG00000050304	SLC25A2	0	0	0	0	0	0	1	3	0	1	0	0
ENSMUSG00000050310	RICTOR	31	31	36	34	32	37	163	151	126	143	98	145
ENSMUSG00000050312	NSUN3	0	2	1	3	2	1	4	8	14	14	4	3
ENSMUSG00000050315	SYNPO2	0	2	0	0	0	0	4	10	5	12	7	4
ENSMUSG00000050323	NDUFAF6	5	5	6	8	6	2	29	23	20	18	23	18
ENSMUSG00000050332	AMER1	5	5	8	6	13	6	38	40	24	37	24	25
ENSMUSG00000050335	LGALS3	0	0	0	0	0	0	0	0	0	0	0	2
ENSMUSG00000050350	GPR18	0	0	0	0	1	0	5	4	1	3	0	3
ENSMUSG00000050357	CARMIL2	5	4	5	10	9	9	26	49	32	39	37	22
ENSMUSG00000050359	SPRR1A	0	0	0	0	0	0	0	1	0	0	0	0
ENSMUSG00000050366	OLFR524	0	0	0	0	0	0	2	0	0	0	0	0
ENSMUSG00000050373	SNX21	0	1	0	1	0	0	1	1	4	4	2	1
ENSMUSG00000050379	38961	6	5	7	8	7	4	35	32	31	40	39	36
ENSMUSG00000050382	KIF7	5	10	6	10	12	9	36	40	25	28	34	25
ENSMUSG00000050390	C77080	1	0	1	1	2	2	3	6	3	4	6	3
ENSMUSG00000050410	TCF19	4	6	5	9	10	9	27	31	28	27	15	32
ENSMUSG00000050428	FBXO46	9	11	15	7	9	7	31	34	37	27	23	27
ENSMUSG00000050439	ENTHD1	0	0	0	1	0	0	0	2	0	2	0	0
ENSMUSG00000050440	HAMP	0	0	0	0	0	0	1	0	0	0	0	0
ENSMUSG00000050468	ASTL	0	0	0	0	0	0	0	0	0	0	1	0
ENSMUSG00000050471	FAM118B	0	1	0	0	0	0	4	5	3	6	4	5
ENSMUSG00000050473	SLC35D3	1	0	1	1	0	2	2	2	0	0	0	0
ENSMUSG00000050490	GM8394	2	0	0	0	0	0	1	0	0	3	0	0
ENSMUSG00000050503	FBXL22	5	8	3	5	6	1	24	27	27	26	20	16
ENSMUSG00000050511	OPRD1	0	0	0	0	0	0	1	0	0	0	0	1
ENSMUSG00000050538	B230217C12I	0	0	0	0	0	0	0	1	0	0	0	0
ENSMUSG00000050545	FAM228B	0	0	2	0	0	1	2	0	3	2	1	0
ENSMUSG00000050549	FAM241A	1	5	6	3	3	5	22	28	22	44	23	20

ENSMUSG00000050550	GM11868	0	0	0	0	0	0	0	0	1	0	0	0
ENSMUSG00000050552	LAMTOR4	7	8	14	19	9	10	47	62	44	55	48	48
ENSMUSG00000050555	HYLS1	3	6	6	5	6	7	34	42	30	34	20	31
ENSMUSG00000050556	KCNB1	0	0	0	0	0	0	1	1	0	0	4	1
ENSMUSG00000050565	TOR1AIP2	8	7	9	8	10	5	64	51	37	55	44	36
ENSMUSG00000050567	MAML1	0	3	1	2	4	2	22	26	12	21	21	23
ENSMUSG00000050592	FAM78A	20	21	16	14	22	13	113	97	86	110	78	76
ENSMUSG00000050600	ZFP831	1	2	0	0	2	0	5	2	1	2	3	1
ENSMUSG00000050605	ZFP61	0	1	0	0	0	0	0	0	0	0	0	0
ENSMUSG00000050608	MINOS1	22	20	26	32	32	35	230	245	169	232	205	216
ENSMUSG00000050619	ZSCAN29	1	2	3	8	2	1	14	16	7	16	10	16
ENSMUSG00000050621	RPS27RT	45	50	70	97	61	57	233	191	162	180	132	158
ENSMUSG00000050627	GPD1L	2	2	4	6	4	1	15	16	8	15	12	12
ENSMUSG00000050628	UBALD2	5	4	4	1	4	5	10	20	15	13	11	6
ENSMUSG00000050666	VSTM4	1	0	0	2	0	4	5	9	6	8	5	6
ENSMUSG00000050668	GPATCH11	1	3	0	1	1	1	18	15	6	6	6	4
ENSMUSG00000050675	GP1BA	0	0	0	0	0	0	0	1	0	0	0	0
ENSMUSG00000050697	PRKAA1	3	2	5	8	12	3	43	37	24	43	28	28
ENSMUSG00000050702	4930563M21	0	0	0	0	0	0	0	1	0	0	0	0
ENSMUSG00000050705	2310061I04R	6	10	15	10	8	7	50	60	48	64	30	42
ENSMUSG00000050708	FTL1	2	4	5	2	2	4	13	6	9	8	11	14
ENSMUSG00000050711	SCG2	0	0	0	0	0	0	0	0	0	0	0	1
ENSMUSG00000050714	ZBTB26	4	3	3	2	5	5	18	18	11	26	9	18
ENSMUSG00000050721	PLEKHO2	1	2	0	2	2	2	7	4	5	10	2	13
ENSMUSG00000050732	VAMP8	5	1	1	4	2	2	18	21	14	13	14	16
ENSMUSG00000050737	PTGES	0	0	0	0	0	0	0	0	1	0	0	0
ENSMUSG00000050762	PRSS27	0	0	0	1	1	0	0	1	1	1	0	1
ENSMUSG00000050786	CCDC126	4	12	7	12	6	13	31	30	23	25	34	22
ENSMUSG00000050796	B3GALT6	1	1	1	1	0	1	4	6	7	9	7	5
ENSMUSG00000050812	AI314180	26	30	38	42	37	23	222	175	139	192	131	164
ENSMUSG00000050821	FAM131A	0	0	0	0	0	0	0	0	0	0	0	1
ENSMUSG00000050824	SSTR5	0	0	0	0	0	0	1	0	0	0	0	0
ENSMUSG00000050846	ZFP623	0	0	0	1	0	1	1	3	3	4	2	2
ENSMUSG00000050855	ZFP940	4	4	0	3	4	3	10	7	9	10	5	12
ENSMUSG00000050856	ATP5K	46	50	41	54	58	53	267	298	228	285	243	236
ENSMUSG00000050860	PHOSPHO1	0	0	0	0	0	1	0	0	0	1	0	0
ENSMUSG00000050876	SPATA31D1A	0	0	0	0	0	0	0	0	0	1	0	1
ENSMUSG00000050890	PDIK1L	4	10	7	5	6	4	19	18	13	22	12	18
ENSMUSG00000050891	TATDN1	4	9	2	2	6	6	24	32	18	23	19	29
ENSMUSG00000050900	GM7327	0	0	0	0	0	0	0	0	0	0	0	1
ENSMUSG00000050908	TVP23A	0	0	0	2	0	0	4	3	1	2	3	4
ENSMUSG00000050912	TMEM123	15	21	13	21	25	19	93	127	66	128	73	80
ENSMUSG00000050914	ANKRD37	0	2	3	1	1	1	25	28	18	23	10	13
ENSMUSG00000050919	ZFP366	0	1	0	0	0	0	1	0	0	0	0	0
ENSMUSG00000050921	P2RY10	4	11	7	10	10	7	30	29	23	42	9	26
ENSMUSG00000050926	DCAF12L2	0	0	0	0	0	0	0	1	0	0	0	0
ENSMUSG00000050931	SGMS2	0	0	0	0	0	1	0	2	0	0	2	1
ENSMUSG00000050936	GM42743	1	0	1	1	0	0	0	0	0	1	0	0
ENSMUSG00000050944	EFCAB5	0	0	0	0	0	0	3	1	1	2	1	0
ENSMUSG00000050947	AMIGO1	0	0	0	1	0	0	4	1	0	0	3	0
ENSMUSG00000050953	GJA1	1	5	1	3	1	3	17	20	16	21	10	8
ENSMUSG00000050954	ZFP169	4	1	0	0	1	3	8	2	0	2	2	2
ENSMUSG00000050957	INSL6	0	0	1	0	0	1	5	0	2	1	4	1
ENSMUSG00000050965	PRKCA	4	4	6	7	7	6	26	25	16	22	18	19
ENSMUSG00000050973	GDPGP1	0	1	1	1	0	4	4	3	2	3	5	9
ENSMUSG00000050989	SELENON	0	1	2	1	1	1	2	1	3	3	1	0
ENSMUSG00000051007	GATD1	2	5	9	13	5	10	46	49	28	45	28	38
ENSMUSG00000051008	4930412M03	0	0	0	0	0	0	0	0	0	0	1	0

ENSMUSG00000051034	ZFP11	0	0	0	0	0	0	0	0	0	0	1	0
ENSMUSG00000051074	4930579K19F	0	1	0	0	0	1	3	1	0	1	0	1
ENSMUSG00000051098	MBLAC2	0	1	1	3	1	2	7	8	6	10	8	8
ENSMUSG00000051111	SV2C	0	0	0	0	0	0	0	0	0	1	0	0
ENSMUSG00000051113	FAM71E1	0	1	0	0	0	0	0	0	1	1	0	1
ENSMUSG00000051118	OLFR77	0	0	0	0	0	1	0	0	0	0	0	0
ENSMUSG00000051124	GIMAP9	43	46	55	43	41	45	246	282	171	286	219	212
ENSMUSG00000051133	GM6293	0	0	0	0	0	0	0	0	1	0	0	1
ENSMUSG00000051146	CAMK2N2	0	1	1	0	3	2	13	6	3	7	2	5
ENSMUSG00000051147	NAT2	4	4	0	0	4	3	18	15	13	15	15	18
ENSMUSG00000051149	ADNP	0	0	0	0	0	0	1	0	0	1	0	0
ENSMUSG00000051154	COMMD3	2	1	1	2	2	1	8	6	7	6	1	5
ENSMUSG00000051166	EML5	2	6	5	0	5	4	9	10	16	13	4	11
ENSMUSG00000051169	RPUSD3	0	1	1	1	0	1	2	2	1	5	7	3
ENSMUSG00000051184	ZFP524	0	1	0	1	0	0	6	8	6	8	2	5
ENSMUSG00000051185	FAM174A	1	0	0	3	4	2	15	16	14	24	7	8
ENSMUSG00000051212	GPR183	0	0	0	0	0	0	0	0	0	1	2	0
ENSMUSG00000051220	ERCC6L	3	4	3	8	3	1	11	19	10	16	14	15
ENSMUSG00000051223	BZW1	36	36	36	48	51	31	231	240	169	244	168	184
ENSMUSG00000051224	TCEANC	1	2	0	1	0	3	5	6	3	9	1	6
ENSMUSG00000051225	FAM83A	0	0	0	0	0	1	0	0	0	0	0	0
ENSMUSG00000051228	NYX	0	0	0	1	0	0	0	0	0	0	0	0
ENSMUSG00000051232	TMEM199	3	0	1	3	4	3	28	21	16	28	18	16
ENSMUSG00000051234	RNF7	7	8	8	12	11	13	61	61	49	77	45	50
ENSMUSG00000051235	GEN1	1	8	6	7	9	7	32	43	28	41	28	29
ENSMUSG00000051238	SWSAP1	0	0	0	2	0	0	3	0	3	4	1	5
ENSMUSG00000051246	MSANTD1	0	0	0	1	1	0	1	2	0	1	3	3
ENSMUSG00000051255	GM6563	0	0	1	2	0	0	3	5	2	3	2	4
ENSMUSG00000051256	JAGN1	2	7	1	8	3	4	21	19	13	21	15	14
ENSMUSG00000051257	TRAP1A	0	0	0	0	0	0	0	0	0	0	0	1
ENSMUSG00000051278	ZGRF1	7	9	15	7	10	11	46	51	51	57	41	34
ENSMUSG00000051285	PCMTD1	11	7	4	11	8	6	36	42	26	45	29	28
ENSMUSG00000051297	2410124H12F	0	9	3	3	6	1	16	15	19	17	19	25
ENSMUSG00000051306	USP42	6	5	6	4	9	6	18	29	16	28	15	19
ENSMUSG00000051316	TAF7	13	25	28	28	41	28	132	164	114	162	107	140
ENSMUSG00000051319	1500011K16F	0	0	0	0	0	0	0	1	0	1	0	0
ENSMUSG00000051323	PCDH19	0	0	0	0	0	0	0	0	0	0	0	1
ENSMUSG00000051329	NUP160	15	9	9	8	5	13	78	49	45	66	43	49
ENSMUSG00000051335	GFOD1	0	4	3	2	1	0	7	14	7	9	5	8
ENSMUSG00000051341	ZFP52	6	6	5	1	4	7	31	32	17	30	24	23
ENSMUSG00000051344	PLEKHM3	0	0	1	1	0	1	2	3	0	2	1	2
ENSMUSG00000051346	SPRYD4	0	1	2	5	2	0	11	10	12	10	6	5
ENSMUSG00000051351	ZFP46	0	0	1	2	2	2	6	6	4	6	4	4
ENSMUSG00000051355	COMMD1	3	3	6	3	2	6	31	41	15	29	29	23
ENSMUSG00000051378	KIF18B	2	1	2	0	4	0	9	15	6	5	12	5
ENSMUSG00000051390	ZBTB22	1	1	0	2	1	4	12	19	8	11	10	12
ENSMUSG00000051391	YWHAG	13	5	16	19	16	19	117	132	82	131	84	89
ENSMUSG00000051396	GM45902	1	3	2	0	0	2	4	5	4	15	5	6
ENSMUSG00000051401	KCTD16	1	0	0	0	0	0	0	0	0	0	0	0
ENSMUSG00000051403	PPP1R37	5	1	5	4	3	2	13	18	17	13	11	16
ENSMUSG00000051412	VAMP7	3	7	6	2	3	3	31	37	29	30	26	25
ENSMUSG00000051413	PLAGL2	2	7	9	10	8	10	63	49	31	44	42	42
ENSMUSG00000051427	CCDC157	0	1	0	1	1	0	4	3	2	3	5	5
ENSMUSG00000051444	BBS12	2	1	3	3	5	3	14	10	12	18	12	13
ENSMUSG00000051451	CREBZF	4	7	7	3	6	11	46	43	33	31	31	36
ENSMUSG00000051455	MEIOC	0	0	0	0	0	0	0	0	0	0	1	0
ENSMUSG00000051457	SPN	0	0	0	0	0	0	0	0	0	2	0	1
ENSMUSG00000051469	ZFP24	19	20	33	27	21	37	129	135	83	152	79	85

ENSMUSG00000051483	CBR1	8	10	12	9	8	8	37	46	26	34	24	39
ENSMUSG00000051493	OLFR974	0	0	0	0	0	1	0	0	0	0	0	0
ENSMUSG00000051495	IRF2BP2	18	34	17	39	24	17	141	138	109	159	123	132
ENSMUSG00000051498	TLR6	0	0	0	0	0	1	1	1	1	1	0	3
ENSMUSG00000051499	ZFP786	1	1	0	1	0	0	2	1	0	1	0	2
ENSMUSG00000051502	UFSP1	0	0	0	0	0	0	1	1	2	0	0	0
ENSMUSG00000051506	WDFY4	0	0	1	1	0	0	0	0	0	0	0	0
ENSMUSG00000051510	MAFG	13	18	15	16	14	17	49	51	36	51	21	49
ENSMUSG00000051517	ARHGEF39	1	3	0	3	3	5	6	8	6	7	8	5
ENSMUSG00000051518	RPS19BP1	4	11	5	9	7	2	55	40	35	49	30	49
ENSMUSG00000051537	GM5124	0	1	1	0	0	0	2	1	0	3	1	2
ENSMUSG00000051550	ZFP579	3	3	4	1	0	3	6	11	9	12	11	8
ENSMUSG00000051557	PUSL1	2	4	2	6	4	3	17	16	10	14	15	14
ENSMUSG00000051590	MAP3K19	0	1	0	0	0	3	7	4	9	7	5	4
ENSMUSG00000051606	2010001K21F	0	0	0	0	0	0	0	1	0	0	0	0
ENSMUSG00000051615	RAP2A	2	6	5	2	3	6	11	14	10	17	9	15
ENSMUSG00000051627	HIST1H1E	9	13	13	17	12	12	37	23	21	37	29	20
ENSMUSG00000051638	GM9857	0	0	0	0	0	0	0	1	0	0	0	0
ENSMUSG00000051639	GM5812	1	0	0	0	1	0	1	1	3	2	4	0
ENSMUSG00000051650	B3GNT2	0	1	0	2	1	2	6	5	1	1	3	4
ENSMUSG00000051652	LRRC3	0	0	0	0	0	0	1	0	0	0	0	0
ENSMUSG00000051671	COA6	5	3	4	5	5	8	42	46	23	46	22	40
ENSMUSG00000051674	DCUN1D4	2	0	1	3	3	2	11	8	6	9	14	12
ENSMUSG00000051675	TRIM32	3	8	3	3	2	4	22	22	11	21	21	20
ENSMUSG00000051695	PCBP1	59	91	78	107	84	71	433	502	387	475	360	403
ENSMUSG00000051703	TMEM198	0	0	2	3	3	1	2	3	4	2	0	5
ENSMUSG00000051705	SENP8	4	3	3	3	1	0	7	8	5	13	5	3
ENSMUSG00000051716	APON	0	0	1	0	0	0	2	0	0	1	0	2
ENSMUSG00000051721	WDCP	4	3	3	9	9	6	35	29	26	26	22	25
ENSMUSG00000051723	RPL31-PS13	6	4	7	1	2	1	3	8	6	6	10	7
ENSMUSG00000051727	KCTD14	0	0	0	1	1	0	4	1	3	2	3	3
ENSMUSG00000051730	METTL5	15	21	23	40	19	16	99	108	99	122	102	109
ENSMUSG00000051735	RINL	9	6	13	8	13	11	31	45	32	48	32	48
ENSMUSG00000051736	FAM229B	0	0	0	0	0	0	0	0	1	4	2	2
ENSMUSG00000051747	TTN	2	1	3	0	3	3	2	0	0	4	1	2
ENSMUSG00000051768	XRCC1	2	4	2	4	12	9	32	36	29	30	14	37
ENSMUSG00000051786	TUBGCP6	5	6	6	6	2	7	10	18	20	18	6	10
ENSMUSG00000051804	ADAM6B	1	0	0	1	0	0	2	1	0	1	0	0
ENSMUSG00000051811	COX6B2	0	0	0	0	0	0	1	0	0	0	0	0
ENSMUSG00000051832	E230016K23F	0	0	0	0	0	0	1	0	0	0	0	0
ENSMUSG00000051839	GYPA	0	0	0	0	0	0	0	0	0	1	0	0
ENSMUSG00000051851	RTL8C	0	0	0	0	0	0	0	0	0	0	1	0
ENSMUSG00000051853	ARF3	4	3	6	5	2	6	27	14	22	23	15	16
ENSMUSG00000051855	MEST	0	0	0	1	1	1	0	0	1	0	1	0
ENSMUSG00000051864	TBC1D22A	1	1	0	2	1	0	0	8	5	8	7	9
ENSMUSG00000051890	KLHDC1	0	0	2	2	0	0	8	5	4	5	7	4
ENSMUSG00000051934	SPATS2	0	1	0	0	0	0	0	0	0	0	0	0
ENSMUSG00000051977	PRDM9	4	4	3	7	9	6	26	20	19	16	27	24
ENSMUSG00000051978	ERICH1	3	5	4	7	5	9	39	32	16	47	24	34
ENSMUSG00000051984	SEC31B	0	2	0	0	0	0	0	0	0	1	0	1
ENSMUSG00000051989	SMIM11	8	8	12	16	11	14	92	92	70	85	78	60
ENSMUSG00000051998	LAX1	1	0	1	5	2	0	11	5	8	6	4	7
ENSMUSG00000052005	GM9864	0	1	0	0	0	0	0	0	1	0	0	0
ENSMUSG00000052013	BTLA	7	6	5	6	3	10	50	41	31	34	25	20
ENSMUSG00000052031	TAGAP1	9	13	11	14	19	13	107	108	74	128	68	75
ENSMUSG00000052033	PFDN4	16	17	16	17	19	19	171	155	143	137	120	139
ENSMUSG00000052040	KLF13	17	25	33	26	23	22	71	84	58	97	61	64
ENSMUSG00000052056	ZFP217	16	7	12	14	10	12	30	41	39	54	24	34

ENSMUSG00000052062	PARD3B	0	0	0	0	0	0	0	1	0	0	0	0
ENSMUSG00000052085	DOCK8	3	0	4	2	2	2	1	0	0	0	0	1
ENSMUSG00000052087	RGS14	3	10	5	10	5	5	34	41	30	30	25	25
ENSMUSG00000052102	GNPDA1	0	2	2	0	0	0	5	4	3	2	2	2
ENSMUSG00000052105	MTCL1	0	2	0	0	2	0	2	2	1	6	0	0
ENSMUSG00000052131	AKR1B7	0	0	0	0	0	0	0	0	0	0	2	0
ENSMUSG00000052137	RBM12B2	13	18	18	18	14	15	97	84	65	100	64	78
ENSMUSG00000052139	BABAM2	1	2	1	4	2	2	10	10	6	10	2	5
ENSMUSG00000052142	RASAL3	14	15	18	20	13	12	78	63	62	60	54	71
ENSMUSG00000052144	PPP4R2	12	10	7	16	16	13	50	61	50	62	44	56
ENSMUSG00000052146	RPS10	0	5	2	6	6	2	10	8	6	6	7	9
ENSMUSG00000052151	PLPP2	0	2	0	0	1	0	1	4	1	1	0	2
ENSMUSG00000052154	GM9870	0	0	0	0	0	0	1	0	0	0	0	0
ENSMUSG00000052155	ACVR2A	4	5	5	10	10	11	29	33	26	61	30	23
ENSMUSG00000052160	PLD4	0	0	0	0	0	0	0	1	0	0	0	0
ENSMUSG00000052192	GM5963	14	11	18	8	16	20	53	58	56	53	45	52
ENSMUSG00000052214	OPA3	7	11	6	8	4	8	28	24	18	21	21	24
ENSMUSG00000052221	PPP1R36	0	0	0	0	0	0	0	0	0	0	1	0
ENSMUSG00000052241	A930035D04I	0	0	0	0	0	0	0	1	0	0	0	0
ENSMUSG00000052253	ZFP622	10	9	7	17	10	10	55	64	52	73	55	61
ENSMUSG00000052270	FPR2	0	0	0	0	0	0	0	1	0	0	0	0
ENSMUSG00000052271	BHLHA15	1	0	1	0	0	2	3	0	1	2	1	1
ENSMUSG00000052281	TSPAN32OS	0	0	0	0	1	0	1	0	0	1	0	0
ENSMUSG00000052291	5330438D12I	1	2	2	2	1	2	7	4	2	5	2	2
ENSMUSG00000052293	TAF9	0	0	0	1	0	0	0	0	0	0	0	0
ENSMUSG00000052296	PPP6R1	6	3	10	12	13	6	38	47	29	46	27	50
ENSMUSG00000052298	CDC42SE2	2	8	6	8	6	4	37	35	22	35	18	35
ENSMUSG00000052299	LTN1	9	13	16	10	13	12	59	72	53	58	52	48
ENSMUSG00000052301	DOC2A	0	0	0	0	1	2	11	11	12	12	13	10
ENSMUSG00000052310	SLC39A1	50	83	44	68	69	46	323	337	208	328	232	292
ENSMUSG00000052316	LRRC15	0	0	0	0	0	0	0	0	0	0	1	0
ENSMUSG00000052331	ANKRD44	23	21	26	26	24	19	120	132	67	123	81	89
ENSMUSG00000052336	CX3CR1	13	9	17	11	14	12	68	64	72	61	44	71
ENSMUSG00000052337	IMMT	17	28	24	29	32	30	169	169	124	197	121	136
ENSMUSG00000052369	TMEM106C	2	1	1	0	2	3	8	12	7	15	8	8
ENSMUSG00000052371	HOXD3OS1	0	0	1	0	2	1	4	7	2	5	8	2
ENSMUSG00000052372	IL1RAPL1	0	0	0	0	0	0	0	0	0	2	0	0
ENSMUSG00000052374	ACTN2	0	0	0	1	0	0	1	1	0	2	2	4
ENSMUSG00000052384	NRROS	5	10	13	18	7	14	38	49	36	60	29	47
ENSMUSG00000052395	RFT1	2	1	1	3	2	1	12	13	8	6	8	7
ENSMUSG00000052397	EZR	21	31	34	27	29	32	231	217	149	228	147	162
ENSMUSG00000052406	REXO4	11	4	8	9	9	16	50	61	44	41	46	59
ENSMUSG00000052407	CCDC171	1	0	0	1	2	2	6	8	3	9	5	5
ENSMUSG00000052419	2610001J05R	10	10	16	10	12	8	49	46	42	53	41	44
ENSMUSG00000052423	B4GALT3	3	0	1	2	1	0	22	10	5	8	12	9
ENSMUSG00000052428	TMCO1	9	14	16	18	21	10	89	95	62	105	64	71
ENSMUSG00000052435	CEBPE	1	0	0	0	0	0	0	0	0	0	0	0
ENSMUSG00000052446	ZFP961	6	9	11	4	6	5	46	27	22	39	20	19
ENSMUSG00000052456	ASNA1	6	5	7	7	6	10	41	37	34	39	33	41
ENSMUSG00000052459	ATP6V1A	5	12	4	9	7	8	57	52	39	52	36	41
ENSMUSG00000052477	C130026I21R	0	0	0	0	0	0	0	0	0	2	1	0
ENSMUSG00000052482	GM8174	0	0	0	0	0	0	0	1	0	0	0	0
ENSMUSG00000052485	TMEM171	2	1	0	2	0	1	1	4	2	5	0	2
ENSMUSG00000052488	CHERP	21	28	22	34	22	25	95	115	79	129	88	88
ENSMUSG00000052512	NAV2	7	15	19	25	23	19	70	56	55	81	39	67
ENSMUSG00000052516	ROBO2	0	0	0	0	0	0	1	0	0	0	0	0
ENSMUSG00000052525	SPDYA	0	1	0	1	0	0	2	1	1	2	1	1
ENSMUSG00000052533	NUP188	17	12	8	18	21	12	61	62	33	70	47	67

ENSMUSG00000052534	PBX1	0	0	0	0	0	0	0	0	0	1	0	0
ENSMUSG00000052539	MAGI3	0	0	0	0	0	0	0	0	0	1	0	0
ENSMUSG00000052544	ST6GALNAC3	2	1	0	1	0	2	2	5	5	9	0	6
ENSMUSG00000052557	GAN	3	0	0	2	5	10	18	14	15	19	8	15
ENSMUSG00000052563	D930048N14	0	0	0	0	0	0	1	2	1	0	2	0
ENSMUSG00000052565	HIST1H1D	0	2	4	1	0	2	3	3	6	5	4	1
ENSMUSG00000052566	HOOK2	0	3	0	4	1	3	7	4	7	5	4	10
ENSMUSG00000052572	DLG2	0	0	0	1	0	1	0	0	0	0	0	0
ENSMUSG00000052593	ADAM17	5	9	11	11	17	12	55	68	42	51	39	35
ENSMUSG00000052605	ISOC2B	0	0	1	0	0	1	5	5	0	1	9	4
ENSMUSG00000052609	PLEKHG3	3	5	4	7	6	5	20	20	13	19	12	15
ENSMUSG00000052632	ASAP2	0	0	1	0	0	0	0	1	1	0	1	0
ENSMUSG00000052656	RNF103	4	7	3	6	4	4	29	37	20	31	22	17
ENSMUSG00000052658	5830454E08F	0	0	0	0	0	0	0	0	0	1	0	0
ENSMUSG00000052673	GM9887	0	0	0	0	0	0	0	1	0	0	1	0
ENSMUSG00000052675	ZFP112	0	0	0	1	0	0	1	1	1	1	0	0
ENSMUSG00000052676	ZMAT1	0	0	0	2	1	0	6	6	4	5	3	3
ENSMUSG00000052681	RAP1B	11	18	21	21	19	24	154	130	85	156	87	106
ENSMUSG00000052698	TLN2	3	4	3	3	2	2	16	9	14	19	9	12
ENSMUSG00000052707	TNRC6A	31	32	32	59	48	39	132	178	92	177	99	161
ENSMUSG00000052712	BC004004	2	1	1	1	4	1	8	10	10	2	8	7
ENSMUSG00000052713	ZFP608	0	0	0	0	1	0	3	1	0	3	1	2
ENSMUSG00000052724	GM9888	0	0	0	0	0	0	0	1	1	1	0	0
ENSMUSG00000052727	MAP1B	0	0	0	0	0	0	0	0	1	0	0	1
ENSMUSG00000052736	KLRC2	0	0	0	0	0	0	0	1	0	0	0	0
ENSMUSG00000052738	SUCLG1	18	22	23	24	14	23	169	203	126	174	127	135
ENSMUSG00000052748	SWT1	19	20	12	14	12	20	55	59	40	65	44	36
ENSMUSG00000052751	REPIN1	3	3	1	2	6	2	11	15	14	14	14	8
ENSMUSG00000052752	TRAF7	14	19	9	16	17	15	58	83	42	62	48	52
ENSMUSG00000052760	A630001G21I	4	9	5	6	8	2	25	24	26	28	19	23
ENSMUSG00000052763	ZFP212	0	0	2	1	0	2	5	20	8	6	5	5
ENSMUSG00000052783	GRK4	2	1	0	3	2	0	8	11	11	10	12	6
ENSMUSG00000052794	1700030K09F	4	5	5	3	11	5	33	24	32	27	32	26
ENSMUSG00000052798	NUP107	5	10	16	6	9	13	44	59	35	50	50	41
ENSMUSG00000052812	ATAD2B	3	9	7	8	7	8	56	51	30	56	33	44
ENSMUSG00000052825	GM9892	2	2	1	3	0	5	6	10	9	7	7	6
ENSMUSG00000052833	SAE1	16	24	18	25	26	15	131	126	94	120	103	130
ENSMUSG00000052837	JUNB	11	16	17	14	16	16	62	55	51	62	39	65
ENSMUSG00000052850	TAS2R137	0	0	0	0	0	0	0	0	0	0	0	1
ENSMUSG00000052852	REEP1	0	0	0	0	0	0	0	0	0	1	0	0
ENSMUSG00000052906	UBXN8	3	9	7	12	8	8	43	34	35	34	36	30
ENSMUSG00000052911	LAMB2	0	0	0	0	0	1	0	0	0	0	0	0
ENSMUSG00000052914	CYP2J6	0	0	0	0	0	0	0	0	0	0	0	1
ENSMUSG00000052915	MSL1	9	20	17	22	23	20	82	87	70	121	54	78
ENSMUSG00000052917	SENP7	4	6	7	7	6	4	43	47	40	30	40	35
ENSMUSG00000052920	PRKG1	0	1	0	0	1	0	1	0	0	2	0	0
ENSMUSG00000052921	ARHGEF15	0	0	0	0	0	0	0	3	0	0	1	0
ENSMUSG00000052926	RNASEH2A	4	10	10	9	13	4	44	53	32	59	40	40
ENSMUSG00000052928	CTIF	0	1	0	0	0	0	0	0	0	0	0	0
ENSMUSG00000052934	FBXO31	1	3	4	1	2	5	11	12	18	9	18	9
ENSMUSG00000052949	RNF157	1	3	1	0	2	2	3	6	4	3	4	8
ENSMUSG00000052962	MRPL35	9	6	9	11	5	8	51	45	40	53	49	63
ENSMUSG00000052997	UBA2	14	7	18	18	25	16	173	165	140	191	103	126
ENSMUSG00000053007	CREB5	0	0	0	0	0	0	0	1	0	0	0	0
ENSMUSG00000053012	KRCC1	4	0	2	4	4	4	21	14	11	18	4	10
ENSMUSG00000053030	SPINK2	3	4	0	5	4	0	5	7	5	9	5	5
ENSMUSG00000053040	APH1C	0	0	0	0	0	0	0	0	1	0	0	0
ENSMUSG00000053046	BRSK2	0	0	0	0	0	0	0	0	0	1	0	0

ENSMUSG00000053062	JAM2	0	0	0	0	0	0	1	0	0	0	0	0
ENSMUSG00000053063	CLEC12A	0	1	0	0	0	0	0	1	1	1	0	1
ENSMUSG00000053080	2700081O15I	1	8	2	10	2	3	24	27	15	30	17	18
ENSMUSG00000053081	1700069B07F	0	0	1	0	0	0	0	0	0	0	0	0
ENSMUSG00000053091	LINS1	2	1	1	0	0	3	18	5	8	4	5	6
ENSMUSG00000053094	TMEM248	17	16	15	20	22	8	59	91	42	75	61	58
ENSMUSG00000053101	GPR141	6	7	9	11	6	9	37	34	28	52	19	35
ENSMUSG00000053110	YAP1	0	1	0	0	0	0	0	0	0	0	0	0
ENSMUSG00000053113	SOCS3	0	0	0	0	0	0	1	0	1	0	0	0
ENSMUSG00000053119	CHMP3	5	4	4	8	4	9	32	32	24	36	25	22
ENSMUSG00000053121	GM5129	0	0	0	0	0	0	0	1	0	0	0	0
ENSMUSG00000053128	RNF26	2	1	2	0	2	4	13	6	8	11	5	8
ENSMUSG00000053134	SUPT7L	5	4	4	9	5	6	20	22	11	32	24	17
ENSMUSG00000053137	MAPK11	1	0	2	1	1	1	10	5	6	6	3	3
ENSMUSG00000053153	SPAG16	0	0	0	0	1	0	1	0	0	1	0	0
ENSMUSG00000053158	FES	1	0	0	0	0	0	1	0	0	0	0	1
ENSMUSG00000053173	RPL18-PS2	2	1	0	1	2	0	6	5	5	4	5	4
ENSMUSG00000053175	BCL3	1	0	0	1	2	1	2	2	1	2	2	1
ENSMUSG00000053178	MTERF1B	0	0	0	0	0	0	4	3	3	2	1	3
ENSMUSG00000053181	A830005F24F	0	0	0	0	0	0	0	0	1	1	0	1
ENSMUSG00000053182	GM609	0	0	0	0	0	0	1	0	0	0	0	1
ENSMUSG00000053192	MLLT11	1	1	0	0	1	2	5	6	7	4	4	6
ENSMUSG00000053198	PRX	0	1	0	0	0	0	0	2	0	0	1	1
ENSMUSG00000053199	ARHGAP20	1	0	0	0	0	0	0	0	0	0	0	0
ENSMUSG00000053205	STYX	0	1	1	5	1	2	12	14	9	11	9	7
ENSMUSG00000053208	KAT2B-PS	0	0	0	0	0	0	0	0	0	0	1	0
ENSMUSG00000053226	DAND5	1	1	0	1	0	0	2	3	1	2	0	2
ENSMUSG00000053253	NDFIP2	6	11	8	14	6	7	59	61	49	101	57	28
ENSMUSG00000053286	TRMT1L	33	20	20	18	22	14	122	123	73	106	99	107
ENSMUSG00000053289	DDX10	7	18	14	23	18	14	73	67	44	87	53	49
ENSMUSG00000053291	RAB4B	0	0	0	0	0	0	0	0	0	0	1	0
ENSMUSG00000053293	POM121	18	21	33	29	22	30	122	104	80	103	85	86
ENSMUSG00000053297	AI854703	0	0	0	0	1	2	0	0	0	1	2	0
ENSMUSG00000053310	NRGN	0	0	0	1	0	1	2	1	0	0	2	1
ENSMUSG00000053317	SEC61B	38	48	39	51	53	33	335	392	311	396	263	325
ENSMUSG00000053329	D10JHU81E	1	4	4	4	2	1	18	15	22	19	15	18
ENSMUSG00000053332	GAS5	111	132	129	151	160	149	752	822	560	772	774	660
ENSMUSG00000053333	DIS3L2	0	2	1	0	1	0	7	14	10	7	2	6
ENSMUSG00000053334	FICD	0	2	0	0	0	0	1	1	3	4	2	4
ENSMUSG00000053347	ZFP943	1	2	1	1	0	0	6	14	5	11	10	4
ENSMUSG00000053358	GM9905	1	0	0	0	1	0	1	0	0	0	2	0
ENSMUSG00000053390	ZFP952	4	3	6	10	7	3	22	29	15	22	17	20
ENSMUSG00000053398	PHGDH	14	19	18	24	18	32	169	202	154	177	129	159
ENSMUSG00000053399	ADAMTS18	3	3	1	0	4	3	1	1	0	0	0	0
ENSMUSG00000053411	CBX7	3	4	6	3	5	5	13	18	18	18	9	12
ENSMUSG00000053436	MAPK14	53	53	41	67	62	50	225	206	170	229	175	224
ENSMUSG00000053442	4930597O21I	0	0	0	0	0	0	0	0	1	0	0	0
ENSMUSG00000053453	THOC7	13	26	23	27	20	34	152	177	124	161	155	114
ENSMUSG00000053460	GGCX	1	3	6	12	11	7	32	36	25	35	29	27
ENSMUSG00000053461	HHIPL2	0	0	0	0	0	0	0	0	0	0	1	0
ENSMUSG00000053469	TG	0	0	0	0	1	1	4	1	3	3	0	2
ENSMUSG00000053470	KDM3A	5	14	9	12	15	8	78	62	52	58	31	41
ENSMUSG00000053477	TCF4	0	0	0	1	0	0	0	0	0	0	0	0
ENSMUSG00000053483	USP21	1	7	4	4	7	2	15	13	12	19	12	6
ENSMUSG00000053510	NRD1	15	19	16	26	29	14	90	75	60	107	66	76
ENSMUSG00000053519	KCNIP1	0	0	0	0	0	0	0	0	1	0	0	0
ENSMUSG00000053522	LGALS7	0	0	0	0	0	0	0	0	1	0	0	2
ENSMUSG00000053536	CSTF2T	3	2	2	2	3	3	11	18	8	11	15	6

ENSMUSG00000053541	GM4759	34	39	44	53	41	38	178	199	124	249	123	132
ENSMUSG00000053552	EBF4	0	0	0	1	0	0	0	0	0	0	0	0
ENSMUSG00000053553	3110082I17R	4	9	4	6	6	0	15	29	17	29	22	20
ENSMUSG00000053559	SMAGP	0	0	0	0	1	0	1	0	0	2	0	0
ENSMUSG00000053560	IER2	14	30	29	20	15	23	79	104	83	99	79	73
ENSMUSG00000053565	EIF3K	19	37	31	28	34	31	120	150	111	150	116	139
ENSMUSG00000053574	4930563E22F	0	0	0	0	0	0	1	1	0	0	0	0
ENSMUSG00000053580	TANC2	0	0	1	0	0	0	0	0	0	0	0	0
ENSMUSG00000053581	ZFAND2A	2	5	6	4	0	2	15	20	17	19	16	13
ENSMUSG00000053588	A730061H03I	0	0	0	0	0	0	0	1	0	0	0	0
ENSMUSG00000053600	ZFP472	2	8	3	7	7	5	42	43	27	49	46	25
ENSMUSG00000053604	RPIA	3	3	7	0	6	1	25	40	17	16	20	27
ENSMUSG00000053617	SH3PXD2A	0	1	0	1	1	0	0	0	1	0	0	0
ENSMUSG00000053624	GYKL1	1	0	0	2	1	1	8	6	4	3	3	8
ENSMUSG00000053641	DENND4A	2	3	0	5	3	2	14	19	12	16	24	17
ENSMUSG00000053644	ALDH7A1	1	1	1	0	0	1	1	5	0	5	2	2
ENSMUSG00000053647	GPER1	0	0	0	0	0	0	1	0	0	0	1	0
ENSMUSG00000053684	BC048403	0	1	0	1	2	1	5	7	1	2	4	0
ENSMUSG00000053687	DPEP2	0	0	0	0	0	0	0	0	0	0	0	1
ENSMUSG00000053702	NEBL	11	7	7	8	10	7	52	50	41	50	30	45
ENSMUSG00000053714	4732471J01R	1	1	0	1	0	0	0	1	0	1	4	2
ENSMUSG00000053716	DUSP7	2	11	6	7	10	2	28	27	21	25	19	31
ENSMUSG00000053730	TMEM39B	2	8	3	4	7	8	17	21	30	34	24	33
ENSMUSG00000053746	PTRH1	5	11	7	9	7	12	46	52	31	24	29	34
ENSMUSG00000053754	CHD8	44	61	45	73	57	61	235	263	173	259	182	218
ENSMUSG00000053768	CHCHD3	11	15	12	10	20	15	56	60	48	55	52	55
ENSMUSG00000053769	LYSMD1	1	1	0	2	0	1	3	5	4	4	1	3
ENSMUSG00000053774	UBXN7	8	6	9	7	6	10	32	46	36	34	25	33
ENSMUSG00000053797	KRT16	0	0	0	0	0	0	0	0	0	1	0	0
ENSMUSG00000053799	EXOC6	0	0	0	0	1	1	9	7	4	6	4	5
ENSMUSG00000053801	GRWD1	4	3	5	8	6	8	37	30	26	43	28	29
ENSMUSG00000053819	CAMK2D	39	58	45	67	59	50	238	208	172	234	166	216
ENSMUSG00000053835	H2-T24	0	0	0	0	0	1	2	1	1	0	0	0
ENSMUSG00000053838	NUDCD3	12	8	9	19	5	12	37	44	28	45	24	45
ENSMUSG00000053841	TXLNA	8	12	14	20	25	18	75	73	50	95	73	79
ENSMUSG00000053856	DNAJC5G	0	0	0	0	0	0	0	1	0	0	0	0
ENSMUSG00000053870	FPGT	1	1	1	1	1	2	5	5	2	4	3	10
ENSMUSG00000053877	SRCAP	2	2	0	1	1	4	7	5	4	6	5	3
ENSMUSG00000053897	SLC39A8	0	2	2	0	3	2	12	5	10	13	6	7
ENSMUSG00000053898	ECH1	3	7	8	8	6	9	25	36	36	45	33	42
ENSMUSG00000053907	MAT2A	19	22	24	36	22	30	200	207	148	224	155	177
ENSMUSG00000053914	KDM4D	0	0	0	0	0	0	0	1	1	0	0	0
ENSMUSG00000053916	NANP	0	0	0	0	0	0	0	0	1	0	0	0
ENSMUSG00000053925	GM9929	1	0	0	1	0	0	0	1	0	0	1	0
ENSMUSG00000053929	CYHR1	2	14	8	6	6	11	24	37	21	20	24	25
ENSMUSG00000053950	ADNP2	0	5	4	11	3	5	19	18	11	16	12	10
ENSMUSG00000053964	LGALS4	0	1	0	0	0	0	2	1	0	1	1	1
ENSMUSG00000053965	PDE5A	0	0	0	1	0	1	0	0	0	0	1	0
ENSMUSG00000053985	ZFP14	0	1	0	0	0	0	0	0	1	0	1	1
ENSMUSG00000054006	D630008O14	0	0	1	0	0	1	0	0	0	0	0	0
ENSMUSG00000054008	NDST1	2	2	0	4	6	1	12	12	11	18	10	17
ENSMUSG00000054021	SIRT5	0	3	3	1	4	1	5	6	4	5	6	4
ENSMUSG00000054027	NT5DC3	0	3	3	1	2	3	13	11	7	11	5	9
ENSMUSG00000054036	OLFR279	0	0	0	0	0	1	0	0	0	0	0	0
ENSMUSG00000054051	ERCC6	2	2	2	6	1	4	24	20	13	11	11	12
ENSMUSG00000054057	A930004D18I	1	0	3	3	1	1	7	4	6	6	3	2
ENSMUSG00000054065	PKP3	0	0	0	3	0	0	1	0	0	0	1	1
ENSMUSG00000054072	IIGP1	0	0	0	0	1	0	0	0	0	0	0	0

ENSMUSG00000054074	SKIDA1	9	8	7	6	4	11	31	29	15	28	16	21
ENSMUSG00000054079	UTP18	16	32	34	43	34	32	151	154	135	143	96	119
ENSMUSG00000054083	CAPN12	3	4	4	5	3	1	20	15	10	22	25	22
ENSMUSG00000054091	1810037I17R	17	27	31	35	29	15	160	185	121	168	119	139
ENSMUSG00000054099	SLC25A40	0	2	2	2	0	4	14	13	12	23	5	16
ENSMUSG00000054115	SKP2	6	6	3	3	4	2	28	13	20	40	14	16
ENSMUSG00000054116	E130116L18R	0	0	1	0	0	0	0	0	0	0	0	0
ENSMUSG00000054135	A430110L20F	0	2	0	0	0	0	0	1	0	0	2	0
ENSMUSG00000054150	SYNE3	1	1	1	1	1	2	0	0	0	0	0	0
ENSMUSG00000054161	FAM83E	1	0	0	0	0	0	0	3	2	1	1	1
ENSMUSG00000054181	A930012O16I	0	0	0	0	0	0	1	1	1	1	0	0
ENSMUSG00000054191	KLF1	0	1	0	0	0	0	0	0	0	0	0	0
ENSMUSG00000054199	GON4L	3	11	9	10	7	10	44	55	42	48	46	34
ENSMUSG00000054200	FFAR4	0	0	0	0	0	0	0	0	0	0	2	0
ENSMUSG00000054204	ALKAL2	0	0	0	1	0	1	3	1	2	0	2	0
ENSMUSG00000054206	GZMM	0	0	0	0	0	0	0	1	1	0	0	0
ENSMUSG00000054226	TPRKB	2	3	6	4	0	4	25	18	25	25	13	15
ENSMUSG00000054237	FRA10AC1	7	3	5	6	5	2	31	23	21	25	22	23
ENSMUSG00000054256	MSI1	0	2	2	1	2	0	4	7	8	8	4	2
ENSMUSG00000054258	GM5082	0	1	0	0	0	0	0	0	0	0	0	0
ENSMUSG00000054277	ARFGAP3	6	1	5	4	3	5	22	14	19	33	20	19
ENSMUSG00000054280	PRR14L	6	5	5	6	6	3	19	24	16	31	29	23
ENSMUSG00000054293	P2RY10B	0	0	0	0	1	0	0	0	0	0	0	0
ENSMUSG00000054302	EAPP	3	7	4	5	2	2	14	18	12	18	14	19
ENSMUSG00000054304	D130007C19I	0	2	2	2	1	0	3	2	6	3	5	2
ENSMUSG00000054309	CPSF3	16	14	9	12	12	11	72	87	62	73	57	54
ENSMUSG00000054312	MRPS21	11	9	3	16	9	12	96	125	92	100	76	85
ENSMUSG00000054321	TAF4B	3	4	1	4	2	3	18	20	9	23	14	17
ENSMUSG00000054364	RHOB	4	4	4	3	4	3	10	19	17	16	13	7
ENSMUSG00000054381	ZFP747	0	1	3	1	0	2	8	8	11	5	11	8
ENSMUSG00000054383	PNMA1	0	0	0	0	0	0	0	1	2	0	0	0
ENSMUSG00000054387	MDM4	4	10	3	6	12	10	30	41	25	60	38	39
ENSMUSG00000054400	CKLF	2	2	3	3	2	3	8	7	8	18	10	7
ENSMUSG00000054404	SLFN5	2	2	3	4	2	3	14	12	11	13	7	5
ENSMUSG00000054405	DNAJC8	12	25	26	26	25	23	137	140	91	129	98	126
ENSMUSG00000054408	SPCS3	30	52	28	31	40	42	103	113	85	99	78	87
ENSMUSG00000054414	SLC30A7	2	6	6	3	3	4	34	33	26	46	22	31
ENSMUSG00000054426	A930005H10I	1	1	1	3	5	3	2	7	6	5	3	8
ENSMUSG00000054428	ATPIF1	30	28	46	45	36	46	318	292	202	273	217	246
ENSMUSG00000054434	TMEM120B	0	0	0	3	1	0	4	5	2	6	5	3
ENSMUSG00000054435	GIMAP4	2	3	7	5	3	2	14	11	13	17	9	7
ENSMUSG00000054452	AES	26	21	22	32	35	26	177	155	139	177	131	182
ENSMUSG00000054455	VAPB	4	4	2	2	9	1	19	13	16	33	16	19
ENSMUSG00000054469	LCLAT1	2	0	3	0	1	4	8	12	4	11	7	11
ENSMUSG00000054474	THNSL2	0	0	0	0	1	0	1	0	0	0	0	0
ENSMUSG00000054484	TMEM62	4	7	1	4	2	2	11	18	11	22	5	9
ENSMUSG00000054499	DEDD2	3	0	1	1	5	1	11	14	8	9	6	8
ENSMUSG00000054509	PARP4	12	11	13	15	15	11	58	77	65	57	43	70
ENSMUSG00000054514	ATAD3AOS	0	0	1	0	0	0	6	1	2	2	0	0
ENSMUSG00000054517	TRIM65	3	3	3	4	2	3	22	15	9	14	13	16
ENSMUSG00000054519	ZFP867	1	0	0	0	0	0	0	1	1	0	3	1
ENSMUSG00000054520	SH3BP2	0	0	0	1	1	0	10	12	6	5	7	4
ENSMUSG00000054580	PLA2R1	0	0	0	0	0	0	0	0	0	0	0	1
ENSMUSG00000054582	PABPC1L	0	0	0	2	0	0	0	0	1	2	0	0
ENSMUSG00000054598	9130230L23R	0	0	0	0	0	1	0	0	0	0	1	0
ENSMUSG00000054604	CGGBP1	5	8	7	5	6	8	40	38	32	53	23	29
ENSMUSG00000054611	KDM2A	8	12	19	22	16	12	78	79	58	103	43	49
ENSMUSG00000054619	METTL7A1	3	1	2	2	3	2	6	14	8	12	2	8

ENSMUSG00000054648	ZFP869	3	3	8	2	4	5	19	24	16	32	14	23
ENSMUSG00000054672	5830411N06I	0	0	0	0	0	0	1	1	1	1	0	1
ENSMUSG00000054676	1600014C10F	0	2	3	1	2	4	4	13	3	2	4	5
ENSMUSG00000054693	ADAM10	33	37	44	45	36	50	174	132	122	178	111	128
ENSMUSG00000054702	AP1S3	3	12	2	5	1	8	38	18	31	45	16	30
ENSMUSG00000054708	ANKRD24	0	0	0	0	1	0	0	0	0	1	0	1
ENSMUSG00000054715	ZSCAN22	0	1	0	0	0	0	1	2	0	1	2	1
ENSMUSG00000054716	ZFP771	2	6	4	2	3	2	28	20	15	13	12	20
ENSMUSG00000054717	HMGB2	82	112	142	118	110	127	486	571	489	520	396	496
ENSMUSG00000054720	LRRC8C	10	4	10	9	13	12	47	34	43	46	45	40
ENSMUSG00000054723	VMAC	2	1	1	3	1	0	9	8	3	5	2	6
ENSMUSG00000054733	MSRA	1	0	0	0	0	0	2	1	0	0	0	0
ENSMUSG00000054737	ZFP182	3	3	6	2	3	2	22	17	6	21	13	5
ENSMUSG00000054766	SET	114	158	140	179	161	122	890	892	690	916	615	728
ENSMUSG00000054770	KCTD18	4	5	4	7	3	4	31	14	8	21	20	13
ENSMUSG00000054792	KLHL18	2	0	3	3	3	0	3	3	3	8	4	5
ENSMUSG00000054808	ACTN4	22	40	33	29	48	34	115	153	110	148	100	121
ENSMUSG00000054814	USP46	5	0	2	3	4	3	19	10	12	23	16	10
ENSMUSG00000054822	1700041G16I	0	0	0	1	0	0	0	0	0	0	0	0
ENSMUSG00000054823	NSD3	10	20	21	21	23	14	80	108	62	98	77	90
ENSMUSG00000054836	ELP6	3	3	2	3	3	2	13	21	11	12	17	11
ENSMUSG00000054850	SMIM10L2A	0	0	0	0	1	0	3	3	3	4	0	5
ENSMUSG00000054855	RND1	0	0	0	0	0	0	0	1	0	0	4	0
ENSMUSG00000054871	TMEM158	0	0	1	0	0	0	0	0	0	0	0	0
ENSMUSG00000054874	PCNX3	1	5	5	3	2	1	15	16	20	17	13	14
ENSMUSG00000054892	TXK	2	0	0	0	0	0	0	2	3	0	2	1
ENSMUSG00000054894	ATP5S	1	2	0	1	4	0	7	3	7	9	4	7
ENSMUSG00000054920	KLHL5	2	4	2	1	3	1	26	19	13	23	7	16
ENSMUSG00000054932	AFP	0	0	0	0	0	0	0	0	2	0	0	0
ENSMUSG00000054934	KCNMB4	0	0	0	0	0	0	0	0	1	0	0	0
ENSMUSG00000054939	ZFP174	0	0	0	0	0	0	0	0	0	0	1	1
ENSMUSG00000054942	MIGA1	16	25	20	24	26	22	71	79	47	68	58	60
ENSMUSG00000054944	5330416C01F	1	0	0	2	2	0	2	4	0	4	1	3
ENSMUSG00000054945	GM9958	0	0	0	0	0	0	0	2	0	2	0	0
ENSMUSG00000054951	9130008F23F	0	2	1	2	3	1	7	7	4	7	3	8
ENSMUSG00000054966	LMNTD1	0	0	0	0	0	0	1	0	0	2	1	1
ENSMUSG00000054967	ZFP647	0	0	0	0	0	0	0	0	1	1	1	1
ENSMUSG00000054999	NAALADL1	0	0	0	0	2	0	0	1	1	0	1	0
ENSMUSG00000055024	EP300	14	16	23	18	20	12	83	114	87	112	69	77
ENSMUSG00000055027	SMYD1	0	0	0	0	0	0	0	1	0	0	0	0
ENSMUSG00000055044	PDLIM1	2	9	6	4	10	5	41	48	19	33	33	37
ENSMUSG00000055053	NFIC	1	0	3	6	8	3	10	19	20	28	12	28
ENSMUSG00000055065	DDX17	12	14	18	23	25	23	100	101	73	123	88	86
ENSMUSG00000055067	SMYD3	0	3	1	1	1	2	11	7	4	11	12	8
ENSMUSG00000055072	GM9964	0	0	0	0	0	0	0	1	0	0	0	0
ENSMUSG00000055093	GM8430	51	40	49	51	48	61	147	191	112	178	172	120
ENSMUSG00000055116	ARNTL	0	0	0	0	1	1	6	5	5	6	1	2
ENSMUSG00000055128	CGRRF1	4	4	0	2	3	2	14	21	15	16	13	13
ENSMUSG00000055137	SUGCT	0	0	1	0	1	3	0	1	4	5	2	1
ENSMUSG00000055148	KLF2	5	10	8	3	9	6	47	41	30	46	27	36
ENSMUSG00000055172	C1RA	0	1	0	0	0	1	0	0	0	0	0	0
ENSMUSG00000055184	FAM72A	1	2	3	1	0	2	8	10	10	10	6	13
ENSMUSG00000055188	RBM3OS	0	0	1	0	0	0	0	0	0	2	0	0
ENSMUSG00000055200	SERTAD3	2	1	3	2	0	1	5	4	4	7	5	5
ENSMUSG00000055202	ZFP811	2	4	2	4	2	1	9	13	5	13	6	7
ENSMUSG00000055204	ANKRD17	26	44	26	74	41	29	138	152	106	155	102	123
ENSMUSG00000055210	FOXD2	0	0	0	0	0	0	0	1	0	0	1	0
ENSMUSG00000055239	KCMF1	21	18	10	19	16	13	105	116	86	94	62	100

ENSMUSG00000055240	ZFP101	10	12	11	18	14	6	62	60	40	75	41	49
ENSMUSG00000055271	9330161L09R	0	0	0	0	1	0	0	1	0	0	0	1
ENSMUSG00000055296	TMEM245	1	9	4	6	1	4	32	22	11	33	16	15
ENSMUSG00000055302	MRFAP1	18	19	15	26	16	15	87	84	56	83	49	75
ENSMUSG00000055319	SEC23IP	9	13	3	7	12	4	55	67	42	65	50	58
ENSMUSG00000055320	TEAD1	0	0	0	0	0	0	0	0	0	1	0	0
ENSMUSG00000055323	GM9967	0	0	0	0	0	0	0	0	1	1	0	2
ENSMUSG00000055334	SNUPN	1	2	1	0	2	2	5	10	4	16	5	5
ENSMUSG00000055341	ZFP457	0	0	0	0	1	1	1	2	1	2	0	0
ENSMUSG00000055371	STAM2	0	1	3	3	2	1	21	13	8	18	13	8
ENSMUSG00000055401	FBXO6	1	1	0	3	1	0	2	2	3	4	4	1
ENSMUSG00000055413	H2-Q5	0	0	0	0	0	0	0	0	0	0	1	0
ENSMUSG00000055424	GM9970	0	0	0	0	0	0	1	0	0	0	0	0
ENSMUSG00000055435	MAF	57	63	72	89	67	77	384	339	260	393	248	287
ENSMUSG00000055436	SRSF11	216	264	280	290	275	245	1281	1277	1003	1543	1112	1100
ENSMUSG00000055447	CD47	34	36	35	33	39	33	77	81	67	73	64	54
ENSMUSG00000055471	ALK	0	0	0	1	0	0	0	0	0	0	0	0
ENSMUSG00000055480	ZFP458	0	2	1	1	0	1	5	4	5	2	9	3
ENSMUSG00000055491	PPRC1	9	10	13	7	11	14	67	60	47	68	40	56
ENSMUSG00000055493	EPM2A	0	0	0	0	0	0	0	1	1	1	0	0
ENSMUSG00000055494	GM14168	0	0	0	0	1	0	0	1	2	0	0	0
ENSMUSG00000055497	GM9974	1	0	1	0	0	1	1	7	3	5	2	3
ENSMUSG00000055523	GUCY2G	0	0	0	0	0	0	0	0	0	0	1	0
ENSMUSG00000055531	CPSF6	8	11	15	15	17	16	142	119	93	126	76	92
ENSMUSG00000055553	KXD1	2	4	4	6	6	5	29	18	21	16	11	16
ENSMUSG00000055560	ZFP459	0	0	1	1	0	0	5	7	3	2	2	1
ENSMUSG00000055561	SPINK5	0	0	0	2	1	0	2	0	0	2	0	1
ENSMUSG00000055567	UNC80	0	0	0	0	0	0	0	1	0	0	1	0
ENSMUSG00000055612	CDCA7	9	18	7	17	17	13	122	126	79	127	98	119
ENSMUSG00000055629	B4GALNT4	0	0	0	0	2	0	2	1	2	1	1	0
ENSMUSG00000055633	ZFP580	0	0	0	0	0	0	0	1	1	0	0	0
ENSMUSG00000055639	DACH1	0	0	0	0	0	0	0	0	0	1	0	0
ENSMUSG00000055652	KLHL25	1	0	1	1	0	3	11	7	4	8	4	9
ENSMUSG00000055660	METTL4	0	0	3	2	1	0	9	3	8	4	5	5
ENSMUSG00000055670	ZZEF1	7	10	15	8	16	16	56	56	34	58	44	46
ENSMUSG00000055676	GM5069	0	1	0	0	2	0	0	0	0	0	1	0
ENSMUSG00000055681	COPE	12	24	16	28	17	18	138	128	98	139	94	112
ENSMUSG00000055692	TMEM191C	2	1	1	3	1	2	8	9	4	4	8	5
ENSMUSG00000055707	KLHL26	1	1	0	1	1	5	10	8	6	8	11	8
ENSMUSG00000055717	SLAIN1	1	4	5	7	1	2	40	35	19	30	16	20
ENSMUSG00000055720	UBL7	2	1	0	1	0	0	10	11	2	10	6	7
ENSMUSG00000055723	RRAS2	0	0	0	2	1	3	11	13	5	6	7	6
ENSMUSG00000055725	PAQR3	0	1	0	1	0	0	0	1	1	0	0	2
ENSMUSG00000055748	GSDMC4	0	0	0	0	0	0	0	0	0	0	1	0
ENSMUSG00000055760	GEMIN6	3	1	2	3	1	5	15	12	9	12	6	13
ENSMUSG00000055761	NKAIN3	0	0	0	0	0	0	1	2	0	0	0	1
ENSMUSG00000055762	EEF1D	35	42	36	49	42	36	248	272	245	282	205	221
ENSMUSG00000055780	USP26	0	1	0	0	0	0	0	0	0	0	0	0
ENSMUSG00000055805	FMNL1	12	16	7	19	23	18	65	66	34	66	63	55
ENSMUSG00000055817	MTA3	5	2	4	6	9	7	13	7	10	7	19	13
ENSMUSG00000055818	A230083G16	0	0	0	0	0	0	0	0	0	2	0	0
ENSMUSG00000055833	1700034H15	0	0	0	0	1	0	0	0	0	0	0	1
ENSMUSG00000055835	ZFP1	6	6	6	3	6	5	25	48	25	38	32	25
ENSMUSG00000055839	ELOB	53	65	52	72	51	55	301	324	239	322	277	353
ENSMUSG00000055850	RNF181	13	9	15	10	12	8	65	53	47	61	40	52
ENSMUSG00000055862	IZUMO4	23	29	13	35	23	26	106	125	78	108	78	100
ENSMUSG00000055865	FAM19A3	41	51	41	44	52	47	154	215	131	170	143	155
ENSMUSG00000055866	PER2	0	1	0	1	2	0	3	7	5	3	4	5

ENSMUSG00000055884	FANCM	5	6	10	11	5	6	33	40	23	33	25	26
ENSMUSG00000055897	PPP4R1L-PS	4	2	1	3	3	4	12	14	13	12	7	5
ENSMUSG00000055900	TMEM69	0	2	0	1	2	1	14	12	8	11	6	8
ENSMUSG00000055912	TMEM150A	1	4	4	2	2	1	11	8	7	10	6	6
ENSMUSG00000055917	ZFP277	6	0	7	3	6	4	29	23	16	27	17	17
ENSMUSG00000055923	AASDH	1	4	2	1	1	0	9	10	3	7	8	10
ENSMUSG00000055932	FTO	1	5	3	7	11	4	18	18	7	15	13	13
ENSMUSG00000055943	EMC7	9	8	7	19	12	17	54	76	40	77	46	46
ENSMUSG00000055945	PRR18	0	0	0	0	0	0	0	0	1	0	0	0
ENSMUSG00000055960	SKINT4	0	0	0	0	0	0	0	0	0	1	0	0
ENSMUSG00000055991	ZKSCAN5	1	0	1	0	1	0	5	3	0	4	3	4
ENSMUSG00000056004	9330182L06R	0	3	0	1	0	1	2	6	4	6	5	6
ENSMUSG00000056008	GM11541	0	0	0	0	0	0	0	1	0	0	1	0
ENSMUSG00000056014	A430033K04F	4	1	2	0	2	2	7	6	5	10	5	7
ENSMUSG00000056018	CCDC7B	0	0	2	0	1	0	4	0	1	1	0	0
ENSMUSG00000056019	ZFP709	2	0	1	3	1	0	8	7	4	11	4	7
ENSMUSG00000056032	BC018473	0	0	0	0	0	0	0	0	0	1	0	0
ENSMUSG00000056043	RGS9BP	0	0	0	0	0	0	0	0	0	0	0	1
ENSMUSG00000056050	MIA3	20	30	18	34	29	39	120	113	91	125	67	100
ENSMUSG00000056055	SAG	0	2	1	0	2	1	2	2	3	6	2	3
ENSMUSG00000056069	FAM105A	2	3	2	4	2	1	11	11	14	18	9	9
ENSMUSG00000056073	GRIK2	0	0	0	0	0	0	0	0	0	0	0	1
ENSMUSG00000056076	EIF3B	20	31	29	31	32	20	150	150	134	179	127	132
ENSMUSG00000056091	ST3GAL5	10	9	8	10	8	11	39	33	26	50	26	24
ENSMUSG00000056116	H2-T22	2	2	1	5	2	0	22	17	14	23	21	20
ENSMUSG00000056121	FEZ2	0	1	3	0	0	1	3	3	1	1	3	5
ENSMUSG00000056124	B4GALT6	0	0	0	0	0	0	2	1	0	2	1	3
ENSMUSG00000056130	TICAM2	0	0	0	0	0	0	1	2	0	0	0	1
ENSMUSG00000056131	PGM3	7	6	3	4	7	1	12	12	13	23	11	24
ENSMUSG00000056144	TRIM34A	0	1	1	0	0	0	1	3	1	1	2	2
ENSMUSG00000056145	AI504432	15	13	8	14	14	21	33	41	25	35	30	29
ENSMUSG00000056153	SOCS6	0	3	2	3	5	1	10	12	10	9	14	9
ENSMUSG00000056155	NANOS3	0	0	0	0	0	0	0	0	0	1	0	0
ENSMUSG00000056167	CNOT10	7	6	5	10	9	9	49	53	38	73	43	41
ENSMUSG00000056185	SNX32	15	13	13	11	10	6	48	32	30	37	38	21
ENSMUSG00000056201	CFL1	235	270	239	299	283	275	1388	1710	1153	1544	1160	1303
ENSMUSG00000056204	PGPEP1	0	0	1	0	0	0	0	1	1	0	0	0
ENSMUSG00000056209	NPM3	9	18	13	12	12	11	80	75	78	74	52	82
ENSMUSG00000056211	R3HDM1	20	20	18	28	20	19	111	93	82	106	49	99
ENSMUSG00000056215	LRGUK	1	1	0	0	0	1	1	2	1	5	4	2
ENSMUSG00000056216	CEBPG	5	8	2	13	7	12	40	37	28	42	36	28
ENSMUSG00000056228	CARS2	3	10	8	10	4	4	26	29	26	34	28	24
ENSMUSG00000056234	NCOA4	1	0	0	0	2	0	3	2	4	6	2	4
ENSMUSG00000056257	GM5447	0	0	0	0	0	0	0	0	0	0	0	1
ENSMUSG00000056260	LRIF1	34	36	38	56	43	56	269	293	210	343	204	219
ENSMUSG00000056267	CEP70	2	2	1	9	6	1	19	20	13	26	11	8
ENSMUSG00000056268	DENND1B	7	20	12	22	16	17	40	46	45	48	29	41
ENSMUSG00000056271	LMAN1L	0	0	0	0	0	0	0	0	0	0	1	0
ENSMUSG00000056290	MS4A4B	8	10	8	19	5	10	45	31	31	29	25	42
ENSMUSG00000056305	USP39	4	4	3	10	6	3	41	37	23	44	28	32
ENSMUSG00000056310	TYW1	8	3	5	4	7	3	19	22	17	31	30	28
ENSMUSG00000056342	USP34	40	47	40	54	52	44	274	237	173	285	194	238
ENSMUSG00000056366	FABP3-PS1	0	0	0	0	0	0	0	0	0	0	0	1
ENSMUSG00000056367	ACTR3B	0	1	0	1	0	1	3	2	0	4	2	2
ENSMUSG00000056383	AI987944	4	6	3	3	2	1	15	11	8	17	6	9
ENSMUSG00000056394	LIG1	20	27	17	27	23	21	120	110	104	122	115	93
ENSMUSG00000056412	GM12396	0	0	0	0	0	0	2	0	1	1	1	1
ENSMUSG00000056413	ADAP1	0	0	0	0	0	0	0	0	2	0	1	0

ENSMUSG00000056418	BC043934	2	0	0	0	0	0	0	3	0	0	0	1
ENSMUSG00000056423	UTS2B	1	0	0	0	0	0	1	0	0	0	0	0
ENSMUSG00000056429	TGOLN1	20	27	26	20	17	31	88	80	63	80	70	81
ENSMUSG00000056458	MOK	0	1	0	0	0	1	0	0	1	0	0	0
ENSMUSG00000056459	ZBTB25	0	2	2	1	4	0	7	4	6	4	5	4
ENSMUSG00000056476	MED12L	0	0	2	2	2	2	6	9	4	2	7	7
ENSMUSG00000056486	CHN1	3	1	1	3	4	2	29	17	14	18	12	28
ENSMUSG00000056493	FOXK1	1	1	5	6	4	0	24	23	19	15	27	17
ENSMUSG00000056498	TMEM154	2	0	0	1	2	0	3	3	0	2	3	1
ENSMUSG00000056501	CEBPB	9	9	16	10	4	14	41	53	30	61	36	42
ENSMUSG00000056508	1700001K19F	0	0	0	0	0	0	1	0	0	0	0	0
ENSMUSG00000056515	RAB31	0	0	0	0	0	0	0	1	0	0	0	0
ENSMUSG00000056531	CCDC18	22	34	23	36	32	29	108	127	80	135	89	89
ENSMUSG00000056536	PIGN	3	4	5	6	2	6	14	24	13	22	18	15
ENSMUSG00000056537	RLIM	11	15	10	23	15	21	75	98	63	73	44	78
ENSMUSG00000056579	TUG1	5	4	0	5	8	4	31	22	19	28	17	26
ENSMUSG00000056592	ZFP658	0	1	0	0	1	0	0	2	2	0	3	0
ENSMUSG00000056598	DRC3	0	0	0	0	0	0	0	0	1	0	0	1
ENSMUSG00000056602	FRY	0	0	0	0	0	0	2	1	0	0	0	1
ENSMUSG00000056608	CHD9	4	7	4	5	3	4	16	20	12	24	11	12
ENSMUSG00000056612	PPP1R14B	20	16	13	28	28	21	117	119	98	117	93	103
ENSMUSG00000056617	4931429L15R	0	0	0	0	0	0	0	0	0	0	0	1
ENSMUSG00000056629	FKBP2	10	13	9	15	13	20	74	87	72	71	57	62
ENSMUSG00000056632	DSG3	0	0	0	0	0	0	0	0	2	0	0	0
ENSMUSG00000056665	THEM6	0	1	2	2	1	2	10	10	5	7	8	11
ENSMUSG00000056666	RETSAT	2	2	1	2	2	1	11	12	11	17	9	9
ENSMUSG00000056682	4930465K10F	0	0	0	0	0	0	1	0	0	0	0	0
ENSMUSG00000056687	GM11696	0	0	0	0	0	1	1	0	0	1	2	1
ENSMUSG00000056692	D17WSU92E	6	13	5	9	8	8	43	49	36	41	28	42
ENSMUSG00000056698	ELMOD3	2	1	6	2	2	3	7	9	6	15	7	9
ENSMUSG00000056706	KRTAP7-1	0	0	0	0	1	0	0	0	0	0	0	0
ENSMUSG00000056708	IER5	5	11	7	5	6	6	45	49	27	44	40	19
ENSMUSG00000056718	GM13199	0	1	0	1	0	0	4	1	1	4	1	2
ENSMUSG00000056724	NBEAL2	2	4	2	5	8	0	8	8	11	11	10	6
ENSMUSG00000056735	A930024E05F	2	1	0	0	1	1	2	3	1	2	0	2
ENSMUSG00000056749	NFIL3	2	0	1	2	3	0	12	11	13	14	9	15
ENSMUSG00000056753	C330011M18	0	0	0	0	0	0	1	1	1	0	0	1
ENSMUSG00000056763	CSPP1	10	28	13	24	16	26	87	104	96	114	81	72
ENSMUSG00000056770	SETD3	3	3	4	10	7	6	23	35	31	32	26	34
ENSMUSG00000056772	RPS6-PS2	0	0	0	0	0	0	0	1	0	0	2	0
ENSMUSG00000056777	RPL21-PS13	0	0	0	0	0	0	0	0	1	0	0	0
ENSMUSG00000056820	TSNAX	4	6	5	10	7	1	25	21	23	21	19	15
ENSMUSG00000056832	TTC26	0	0	1	0	1	1	2	3	0	2	4	3
ENSMUSG00000056836	GM6851	0	0	0	0	1	0	0	0	0	1	1	0
ENSMUSG00000056849	GM16415	0	0	0	0	0	0	0	1	1	0	0	0
ENSMUSG00000056851	PCBP2	102	125	129	136	153	127	702	699	540	731	531	682
ENSMUSG00000056856	JAKMIP3	0	0	0	0	0	0	0	0	0	1	0	0
ENSMUSG00000056877	RPS15A-PS1	0	0	0	0	0	0	0	0	0	1	0	0
ENSMUSG00000056880	GADL1	1	1	0	1	0	0	1	0	0	0	1	0
ENSMUSG00000056888	GLIPR1	9	16	10	9	6	9	38	43	31	37	22	21
ENSMUSG00000056895	HIST3H2BA	7	6	12	8	13	9	56	65	48	55	49	67
ENSMUSG00000056899	IMMP2L	0	0	0	0	0	0	0	0	0	0	1	0
ENSMUSG00000056904	GM5620	0	0	0	0	0	0	0	1	0	0	0	0
ENSMUSG00000056917	SIPA1	17	21	14	21	27	19	89	74	73	76	58	84
ENSMUSG00000056919	CEP162	10	16	23	13	17	20	49	63	37	72	47	43
ENSMUSG00000056938	ACBD4	0	0	2	0	2	1	6	4	3	6	2	4
ENSMUSG00000056941	COMMD7	1	11	5	9	6	9	37	49	29	64	31	39
ENSMUSG00000056952	TATDN2	3	8	2	4	4	3	24	30	27	25	26	26

ENSMUSG00000056962	JMJD6	15	12	9	18	16	15	76	83	54	71	65	71
ENSMUSG00000056966	GJC3	0	0	0	0	0	0	1	0	0	0	0	0
ENSMUSG00000056987	FAM71D	0	0	0	0	0	0	0	0	1	1	0	0
ENSMUSG00000056999	IDE	10	11	13	15	10	15	42	69	46	57	46	68
ENSMUSG00000057036	GM7536	16	14	24	20	12	19	62	67	54	63	46	64
ENSMUSG00000057054	INCA1	0	0	0	0	0	0	0	0	1	0	0	0
ENSMUSG00000057058	SKAP1	0	0	0	0	0	0	0	0	2	4	1	0
ENSMUSG00000057069	ERO1LB	3	3	2	1	2	0	11	12	8	8	5	6
ENSMUSG00000057093	ZFP607B	0	1	0	0	1	1	5	4	2	2	3	2
ENSMUSG00000057098	EBF1	0	0	0	0	1	0	1	2	0	1	0	1
ENSMUSG00000057101	ZFP180	2	4	3	5	5	3	15	13	6	19	13	14
ENSMUSG00000057103	NAT8F1	1	0	0	1	0	0	9	8	5	4	2	6
ENSMUSG00000057110	CNTRL	38	51	41	53	41	51	222	205	163	252	164	162
ENSMUSG00000057113	NPM1	136	192	159	199	182	165	1328	1339	939	1563	901	1044
ENSMUSG00000057123	GJA5	0	0	0	0	0	0	0	0	2	0	0	1
ENSMUSG00000057130	TXNL4A	1	1	3	7	7	3	22	21	16	34	24	16
ENSMUSG00000057132	RPGRIP1	226	293	226	259	235	279	736	693	479	722	491	590
ENSMUSG00000057133	CHD6	72	131	109	126	120	146	539	494	350	621	346	430
ENSMUSG00000057134	ADO	5	9	4	8	10	12	26	34	22	41	20	21
ENSMUSG00000057137	TMEM140	1	0	0	0	1	0	0	2	1	0	1	1
ENSMUSG00000057143	TRIM12C	4	3	6	4	4	6	17	20	7	21	15	9
ENSMUSG00000057147	DPH6	1	2	0	2	2	5	10	5	5	11	4	7
ENSMUSG00000057156	HOMEZ	2	3	1	1	3	1	14	21	18	18	14	18
ENSMUSG00000057176	CCDC189	0	0	0	1	3	0	1	3	2	4	4	3
ENSMUSG00000057177	GSK3A	3	1	3	6	12	4	41	36	27	34	19	31
ENSMUSG00000057181	5730455P16F	0	2	0	3	3	6	9	13	12	19	5	15
ENSMUSG00000057182	SCN3A	8	6	6	9	9	9	46	35	24	50	23	34
ENSMUSG00000057191	AB124611	0	2	1	1	1	1	11	13	6	12	4	9
ENSMUSG00000057193	SLC44A2	11	8	4	9	6	9	29	45	32	31	24	36
ENSMUSG00000057219	ARMC7	1	1	0	1	1	4	10	11	1	7	2	7
ENSMUSG00000057223	GM6578	0	0	0	0	0	0	0	0	0	2	1	1
ENSMUSG00000057229	DMAC2	2	1	2	3	1	2	9	2	10	12	10	9
ENSMUSG00000057230	AAK1	16	29	22	35	24	34	133	130	105	127	103	107
ENSMUSG00000057234	METTL15	0	1	0	0	1	0	2	2	1	6	3	3
ENSMUSG00000057236	RBBP4	67	84	75	108	98	78	677	667	475	766	505	589
ENSMUSG00000057244	GM6139	0	0	0	0	1	0	0	0	0	0	0	0
ENSMUSG00000057262	GM10020	0	0	0	0	0	1	0	1	0	0	0	0
ENSMUSG00000057265	BBOF1	0	2	1	0	0	3	6	2	4	5	5	3
ENSMUSG00000057270	OLFR1501	0	0	0	1	0	0	0	0	0	0	0	0
ENSMUSG00000057278	SNRPG	20	31	17	30	21	22	177	181	156	191	148	170
ENSMUSG00000057286	ST6GALNAC2	1	1	0	1	0	1	3	1	5	4	1	2
ENSMUSG00000057322	RPL38	72	101	97	102	92	89	531	594	421	572	408	494
ENSMUSG00000057329	BCL2	2	3	10	16	9	8	23	12	14	17	8	15
ENSMUSG00000057335	CEP170	13	14	7	12	13	12	62	72	45	55	35	39
ENSMUSG00000057337	CHST3	0	0	0	0	0	0	1	0	0	0	0	0
ENSMUSG00000057342	SPHK2	0	6	0	4	4	3	12	12	9	9	7	16
ENSMUSG00000057359	GM17494	0	0	0	2	2	0	1	6	3	0	0	2
ENSMUSG00000057363	UXS1	6	11	6	12	15	15	40	51	26	42	29	44
ENSMUSG00000057367	BIRC2	2	8	8	15	5	7	28	39	24	30	20	32
ENSMUSG00000057370	GM8724	0	0	0	0	0	0	0	0	0	0	0	1
ENSMUSG00000057375	YIPF1	3	2	0	5	3	0	11	21	15	13	15	13
ENSMUSG00000057378	RYR3	2	3	0	2	3	1	11	15	4	5	10	6
ENSMUSG00000057388	MRPL18	38	58	42	102	67	54	283	302	215	326	226	257
ENSMUSG00000057406	NSD2	11	17	18	22	25	18	102	103	81	103	63	73
ENSMUSG00000057409	ZFP53	8	10	8	13	6	6	36	39	41	36	37	31
ENSMUSG00000057411	FAM173A	0	2	2	3	1	1	8	5	5	8	7	10
ENSMUSG00000057421	LAS1L	4	13	2	14	3	9	44	47	27	50	28	43
ENSMUSG00000057439	KIR3DL2	0	0	0	0	0	0	1	0	0	1	0	0

ENSMUSG00000057440	MPP7	3	2	1	9	2	3	10	11	15	12	11	16
ENSMUSG00000057447	OLFR1205	0	0	0	0	0	0	0	0	1	0	0	0
ENSMUSG00000057457	PHEX	0	0	1	0	0	0	2	1	0	1	0	1
ENSMUSG00000057497	FAM136A	3	2	4	10	3	7	20	26	19	39	31	26
ENSMUSG00000057499	GM6177	0	0	0	0	0	0	0	1	0	0	0	0
ENSMUSG00000057506	BLOC1S2	2	5	2	4	2	2	22	20	13	29	19	12
ENSMUSG00000057522	SPOP	22	25	36	30	37	35	78	118	71	108	56	81
ENSMUSG00000057530	ECE1	0	1	2	3	0	1	0	0	0	3	1	0
ENSMUSG00000057531	DTNBP1	1	9	9	7	7	6	37	28	18	28	21	22
ENSMUSG00000057541	PUS7	12	14	12	22	19	18	119	101	84	99	83	74
ENSMUSG00000057551	ZFP317	5	0	9	3	4	7	15	11	8	17	9	11
ENSMUSG00000057554	LGALS8	1	5	3	3	7	3	23	20	9	18	13	15
ENSMUSG00000057561	EIF1A	23	33	23	33	44	41	157	187	132	207	115	156
ENSMUSG00000057572	ZBTB8OS	2	3	6	5	7	3	24	24	16	13	22	21
ENSMUSG00000057580	GM10012	1	0	0	0	0	0	0	0	0	1	0	1
ENSMUSG00000057594	ARL16	3	2	4	1	7	0	19	19	20	15	21	17
ENSMUSG00000057596	TRIM30D	1	3	2	0	0	0	8	12	9	16	2	9
ENSMUSG00000057605	GM6807	14	23	19	19	21	18	61	60	52	51	55	46
ENSMUSG00000057606	COLQ	0	0	0	0	0	0	0	0	0	0	0	1
ENSMUSG00000057637	PRDM2	5	5	1	4	7	6	20	28	12	24	22	23
ENSMUSG00000057649	BRD9	17	12	19	10	15	14	64	89	59	74	44	76
ENSMUSG00000057657	RPS18-PS3	38	36	42	48	47	45	122	190	123	160	112	119
ENSMUSG00000057666	GAPDH	13	25	18	18	13	19	217	240	170	234	156	216
ENSMUSG00000057667	BLOC1S3	0	0	1	0	0	0	3	2	2	3	2	6
ENSMUSG00000057672	PKN1	4	3	9	4	7	2	48	36	18	41	27	25
ENSMUSG00000057691	ZFP746	2	3	2	2	3	4	13	8	14	15	11	7
ENSMUSG00000057696	RPL30-PS1	0	2	0	1	0	0	1	1	1	0	5	1
ENSMUSG00000057706	MEX3B	3	4	2	3	0	5	6	7	6	11	7	12
ENSMUSG00000057738	SPTAN1	12	6	14	18	11	10	66	61	44	54	36	43
ENSMUSG00000057751	MEGF6	1	0	0	0	0	0	0	0	0	0	0	0
ENSMUSG00000057762	GM6169	4	2	3	1	3	7	7	4	4	8	7	7
ENSMUSG00000057766	ANKRD29	0	0	0	1	0	2	5	9	11	8	5	2
ENSMUSG00000057778	CYB5D2	0	0	0	2	3	0	5	7	3	7	2	4
ENSMUSG00000057788	DDX49	7	4	4	12	6	11	40	37	26	28	29	36
ENSMUSG00000057789	BAK1	10	11	9	13	9	14	50	49	36	52	48	39
ENSMUSG00000057816	1700007G11I	0	0	0	0	0	0	0	1	0	1	0	0
ENSMUSG00000057817	OLFR1446	0	0	0	0	0	0	0	0	0	0	1	0
ENSMUSG00000057835	ZFP119A	0	1	0	2	2	2	12	5	5	9	2	1
ENSMUSG00000057836	XLR3A	1	1	2	0	1	0	1	12	4	9	6	4
ENSMUSG00000057841	RPL32	281	342	359	418	380	353	1948	1871	1464	1903	1487	1684
ENSMUSG00000057842	ZFP595	2	1	0	4	1	1	13	12	6	13	10	4
ENSMUSG00000057858	FAM204A	10	11	7	14	13	10	80	65	30	67	47	48
ENSMUSG00000057863	RPL36	95	117	117	145	164	112	699	752	539	750	550	670
ENSMUSG00000057880	ABAT	0	0	0	0	0	0	0	1	1	1	0	0
ENSMUSG00000057894	ZFP329	1	6	3	5	5	4	23	20	16	10	11	11
ENSMUSG00000057897	CAMK2B	0	0	0	3	1	1	4	7	7	5	2	4
ENSMUSG00000057914	CACNB2	1	0	0	0	1	0	0	0	0	1	0	0
ENSMUSG00000057948	UNC13D	6	5	5	3	1	4	10	19	8	11	5	13
ENSMUSG00000057963	ITPK1	0	0	2	4	6	4	10	18	17	22	17	15
ENSMUSG00000057967	FGF18	0	0	0	0	0	1	0	0	0	0	0	0
ENSMUSG00000057982	ZFP809	2	1	2	1	1	2	5	11	4	8	5	1
ENSMUSG00000058006	MDN1	10	13	16	16	15	17	84	76	57	80	60	53
ENSMUSG00000058013	40787	10	27	13	16	15	14	104	101	76	107	64	80
ENSMUSG00000058046	4933430I17R	0	0	0	0	0	0	0	0	0	1	0	1
ENSMUSG00000058050	GM9234	0	0	0	0	0	0	1	0	0	0	0	0
ENSMUSG00000058056	PALLD	16	15	25	23	18	19	66	69	47	68	43	60
ENSMUSG00000058064	GM10036	9	8	8	13	10	7	25	30	27	30	15	26
ENSMUSG00000058076	SDHC	11	6	12	10	13	4	63	69	59	59	58	62

ENSMUSG00000058093	ZFP729B	2	1	3	0	5	1	8	12	10	10	5	10
ENSMUSG00000058099	NFAM1	0	0	0	0	0	0	0	0	0	1	0	0
ENSMUSG00000058101	1700084J12R	1	1	1	0	0	0	4	0	0	0	0	0
ENSMUSG00000058126	TPM3-RS7	4	9	5	8	13	10	18	31	20	31	17	24
ENSMUSG00000058135	GSTM1	1	1	0	0	0	0	4	4	1	1	2	3
ENSMUSG00000058159	T2	0	0	0	0	0	0	0	0	0	0	1	0
ENSMUSG00000058173	SMCO4	3	2	3	4	5	6	21	29	22	19	12	19
ENSMUSG00000058174	GM5148	0	0	0	0	1	0	1	1	0	0	0	1
ENSMUSG00000058192	ZFP846	0	6	4	2	0	3	9	7	11	14	3	6
ENSMUSG00000058230	ARHGAP35	8	10	4	9	10	9	28	25	22	28	24	26
ENSMUSG00000058239	USF2	7	8	6	11	15	10	37	36	36	61	33	44
ENSMUSG00000058240	CRYZL1	2	2	1	1	1	1	11	11	5	7	11	9
ENSMUSG00000058246	GM10037	0	0	0	0	0	0	1	0	0	0	0	0
ENSMUSG00000058252	TCP11X2	1	1	0	1	1	1	4	6	6	1	7	6
ENSMUSG00000058254	TSPAN7	0	0	0	0	0	0	0	1	0	0	0	0
ENSMUSG00000058258	IDI1	1	1	1	1	1	0	4	2	2	4	3	2
ENSMUSG00000058267	MRPS14	6	10	12	9	14	5	124	87	81	99	65	80
ENSMUSG00000058290	ESPL1	6	7	9	13	9	7	54	48	38	46	37	45
ENSMUSG00000058291	ZFP68	4	5	4	4	6	8	30	31	27	27	20	23
ENSMUSG00000058297	SPOCK2	7	10	11	14	10	5	38	68	42	74	48	49
ENSMUSG00000058298	MCM9	0	4	1	7	4	4	9	14	11	12	8	9
ENSMUSG00000058301	UPF1	36	45	54	44	48	49	182	169	137	198	136	216
ENSMUSG00000058317	UBE2E2	2	3	3	2	3	4	20	9	6	20	11	9
ENSMUSG00000058318	PHF21A	20	26	20	30	19	16	89	76	77	109	69	86
ENSMUSG00000058331	ZFP85	1	2	0	1	0	1	6	4	1	6	4	2
ENSMUSG00000058351	SMIM4	11	12	24	24	15	19	100	102	83	102	81	98
ENSMUSG00000058355	ABCE1	21	21	18	18	26	22	112	125	101	117	85	92
ENSMUSG00000058357	GM6612	0	0	0	0	0	0	3	0	0	0	0	1
ENSMUSG00000058360	GM10040	0	0	0	0	0	2	1	1	0	0	0	1
ENSMUSG00000058385	HIST1H2BG	1	0	0	0	0	0	1	0	0	1	0	0
ENSMUSG00000058388	PHTF1	10	15	7	8	21	8	26	30	23	41	21	21
ENSMUSG00000058392	RRP1B	46	45	56	49	56	36	241	239	188	268	197	208
ENSMUSG00000058402	ZFP420	1	2	0	1	2	2	6	7	3	3	4	4
ENSMUSG00000058407	TXNDC9	9	7	7	15	6	6	38	42	27	43	28	42
ENSMUSG00000058430	RPL27A-PS2	0	2	3	6	1	3	9	2	8	6	4	6
ENSMUSG00000058440	NRF1	2	5	7	8	14	6	30	29	17	31	18	36
ENSMUSG00000058443	RPL10-PS3	23	20	22	27	32	30	108	119	82	95	72	106
ENSMUSG00000058444	MAP2K5	4	1	7	3	3	2	25	28	19	29	10	13
ENSMUSG00000058446	ZNRF2	15	14	16	28	19	28	83	125	68	113	82	80
ENSMUSG00000058454	DHCR7	6	10	11	11	16	7	93	77	70	85	58	55
ENSMUSG00000058486	WDR91	5	3	2	3	3	0	11	16	14	10	7	10
ENSMUSG00000058492	SCP2-PS2	2	2	0	2	5	1	10	4	6	3	2	1
ENSMUSG00000058498	RNF207	1	0	1	0	1	0	3	1	1	3	1	2
ENSMUSG00000058503	FAM133B	34	45	38	47	34	38	153	172	92	172	109	149
ENSMUSG00000058546	RPL23A	98	151	137	154	140	121	761	788	568	845	670	691
ENSMUSG00000058550	DPPA4	0	0	0	0	0	0	0	0	0	0	1	0
ENSMUSG00000058558	RPL5	85	113	83	151	121	92	830	930	628	786	634	814
ENSMUSG00000058567	GM2531	1	1	1	2	0	3	4	3	3	5	1	2
ENSMUSG00000058569	TMED9	28	30	34	32	40	38	208	218	166	227	135	174
ENSMUSG00000058571	GPC6	0	0	0	0	0	0	0	1	0	0	0	0
ENSMUSG00000058586	SERHL	1	1	2	0	1	3	10	10	9	10	6	6
ENSMUSG00000058587	TMOD3	8	14	17	16	11	10	64	67	66	89	60	61
ENSMUSG00000058589	ANKS1B	0	0	0	0	0	0	0	0	2	0	0	0
ENSMUSG00000058594	FBXO18	2	2	6	4	5	6	23	25	25	19	10	13
ENSMUSG00000058600	RPL30	52	69	66	59	71	69	318	335	240	299	254	276
ENSMUSG00000058603	RPL28-PS1	19	15	31	20	26	23	49	66	57	70	46	55
ENSMUSG00000058638	ZFP110	3	1	3	6	1	9	16	16	14	24	13	22
ENSMUSG00000058655	EIF4B	14	15	19	20	18	23	94	111	75	105	74	85

ENSMUSG00000058672	TUBB2A	3	1	4	3	3	8	15	17	16	25	21	17
ENSMUSG00000058690	CCSER2	2	3	3	5	4	3	15	8	3	18	12	12
ENSMUSG00000058700	RPL21-PS12	0	0	0	0	0	0	0	1	0	0	0	0
ENSMUSG00000058704	MEMO1	13	11	8	17	11	5	132	131	77	134	86	95
ENSMUSG00000058706	0610030E20F	2	6	8	10	4	1	18	20	21	27	32	23
ENSMUSG00000058709	EGLN2	4	5	3	5	1	6	20	30	12	14	15	32
ENSMUSG00000058715	FCER1G	1	0	0	0	2	0	0	3	0	1	1	0
ENSMUSG00000058729	LIN9	5	4	2	6	12	7	38	24	37	59	24	20
ENSMUSG00000058748	ZFP958	1	6	5	3	4	11	21	29	18	24	18	22
ENSMUSG00000058755	OSM	0	0	0	0	1	0	0	0	2	0	0	0
ENSMUSG00000058756	THRA	3	5	3	3	2	5	29	31	24	19	18	32
ENSMUSG00000058761	RNF169	6	5	2	7	4	1	14	18	14	27	9	12
ENSMUSG00000058773	HIST1H1B	1	3	4	1	4	2	11	10	2	6	11	10
ENSMUSG00000058793	CDS2	2	7	17	4	13	8	52	44	33	47	37	32
ENSMUSG00000058799	NAP1L1	56	80	72	101	73	67	553	554	371	602	403	439
ENSMUSG00000058806	COL13A1	2	6	8	2	2	3	21	21	14	19	14	13
ENSMUSG00000058809	HSPD1-PS3	0	0	1	0	0	0	0	2	0	1	0	0
ENSMUSG00000058818	PIRB	0	0	0	1	0	0	1	1	0	1	0	0
ENSMUSG00000058833	REX1BD	1	5	3	0	4	3	23	23	19	20	19	25
ENSMUSG00000058835	ABI1	19	10	15	26	20	16	99	116	72	105	91	73
ENSMUSG00000058838	RPS27A-PS2	0	0	0	0	1	0	0	1	0	1	2	1
ENSMUSG00000058869	GM4832	0	0	0	0	0	0	0	1	0	0	0	0
ENSMUSG00000058881	ZFP516	1	2	0	1	3	1	6	11	9	6	6	8
ENSMUSG00000058886	DEAF1	1	3	5	2	2	2	14	11	16	24	22	17
ENSMUSG00000058897	COL25A1	0	0	0	1	0	0	0	0	0	0	0	0
ENSMUSG00000058900	RSL1	0	1	0	0	0	0	1	3	0	3	0	1
ENSMUSG00000058905	GM10051	2	4	4	3	4	6	18	11	11	21	9	9
ENSMUSG00000058925	1700011I03R	0	0	0	0	0	0	0	0	0	0	0	1
ENSMUSG00000058932	GM2174	0	0	0	0	0	1	1	0	0	0	1	0
ENSMUSG00000058979	HDHD5	1	0	0	1	2	1	7	9	3	2	5	3
ENSMUSG00000058997	VWA8	1	1	2	2	1	1	12	10	4	10	5	7
ENSMUSG00000059005	HNRNPA3	8	9	14	23	17	16	96	117	80	107	65	81
ENSMUSG00000059013	SH2D3C	3	6	6	6	6	6	25	20	15	22	15	16
ENSMUSG00000059027	9630013D21I	6	0	3	1	3	5	16	7	7	9	12	9
ENSMUSG00000059031	OLFR482	0	0	0	0	0	0	0	0	0	2	0	0
ENSMUSG00000059033	RPL18A-PS1	0	0	0	0	0	1	1	0	1	1	1	0
ENSMUSG00000059040	ENO1B	10	7	11	4	7	9	34	26	33	40	29	30
ENSMUSG00000059058	TMA7-PS	0	0	0	0	0	0	2	0	0	0	0	0
ENSMUSG00000059060	RAD51B	2	2	2	1	2	1	9	8	4	5	6	3
ENSMUSG00000059064	GM10059	0	0	0	0	0	0	0	1	4	0	1	0
ENSMUSG00000059070	RPL18	63	85	74	90	98	74	480	482	378	478	339	436
ENSMUSG00000059108	IFITM6	0	0	0	0	0	0	0	0	0	2	0	0
ENSMUSG00000059112	OLFR1263	0	0	0	0	1	0	0	0	0	0	0	0
ENSMUSG00000059119	NAP1L4	13	22	17	19	19	14	111	118	90	109	98	75
ENSMUSG00000059142	ZFP945	9	12	11	9	8	6	56	55	35	56	53	62
ENSMUSG00000059146	NTRK3	0	0	0	1	0	1	0	0	0	0	0	1
ENSMUSG00000059149	MFSD4A	1	0	0	3	1	1	5	2	1	3	2	3
ENSMUSG00000059150	RPL26-PS4	1	1	2	2	0	3	10	8	6	2	3	2
ENSMUSG00000059159	GM8129	0	1	9	4	3	3	10	13	10	9	16	13
ENSMUSG00000059183	MTFMT	3	3	4	2	2	5	17	15	20	27	20	19
ENSMUSG00000059208	HNRNPM	47	58	57	98	81	53	366	393	293	363	313	262
ENSMUSG00000059244	GM10062	0	0	0	0	0	0	0	1	0	0	0	1
ENSMUSG00000059246	FOXB1	1	4	1	4	2	0	0	2	0	0	1	0
ENSMUSG00000059248	40057	8	13	7	10	15	2	72	62	41	53	46	40
ENSMUSG00000059252	TPT1-PS5	0	0	0	0	0	0	0	0	0	0	1	0
ENSMUSG00000059263	USP47	10	7	12	12	12	12	57	68	55	71	44	68
ENSMUSG00000059273	ZC3H4	12	7	7	4	15	16	46	57	42	54	24	36
ENSMUSG00000059277	R74862	0	0	0	0	0	0	1	0	1	0	1	0

ENSMUSG00000059278	NAA38	4	5	7	8	8	6	42	40	32	51	33	46
ENSMUSG00000059288	CDYL	3	9	5	14	4	5	18	11	9	12	16	13
ENSMUSG00000059291	RPL11	168	206	174	206	204	197	717	924	633	745	592	696
ENSMUSG00000059305	VPREB1	0	0	0	0	0	0	0	0	1	0	2	0
ENSMUSG00000059316	SLC27A4	0	0	1	1	0	2	6	5	7	12	7	7
ENSMUSG00000059319	OLFR295	0	0	0	0	0	0	1	0	0	0	0	0
ENSMUSG00000059323	TONSL	6	3	5	4	5	11	18	31	21	30	22	23
ENSMUSG00000059336	SLC14A1	1	0	0	1	2	1	0	0	1	0	1	0
ENSMUSG00000059343	ALDOART1	0	0	1	0	0	0	1	2	1	0	0	1
ENSMUSG00000059352	GM10064	0	0	0	0	0	0	0	0	0	0	0	1
ENSMUSG00000059355	WDR83OS	2	0	1	3	1	2	9	9	6	10	5	4
ENSMUSG00000059363	FXN	2	2	6	2	4	4	14	11	9	18	13	14
ENSMUSG00000059409	PPP2R5D	4	3	3	6	3	6	39	26	25	14	24	38
ENSMUSG00000059423	ZFP933	1	1	0	0	2	1	3	5	3	7	4	5
ENSMUSG00000059430	ACTG2	0	1	0	0	0	0	0	0	0	1	0	0
ENSMUSG00000059436	MAX	6	6	7	11	4	5	45	42	36	39	27	28
ENSMUSG00000059439	BCAS3	1	2	1	0	2	2	4	4	2	8	4	1
ENSMUSG00000059447	HADHB	3	1	1	5	1	1	23	18	19	12	18	21
ENSMUSG00000059456	PTK2B	3	5	2	4	4	4	22	16	10	24	15	14
ENSMUSG00000059461	GM7331	0	0	0	0	0	0	0	1	1	0	0	0
ENSMUSG00000059474	MBTD1	11	8	12	12	13	21	59	67	46	91	52	62
ENSMUSG00000059475	ZFP426	5	5	2	2	4	3	19	30	17	23	10	17
ENSMUSG00000059479	B3GNT8	9	14	8	12	5	6	32	40	35	34	26	28
ENSMUSG00000059482	2610301B20F	0	2	1	2	0	1	9	18	10	19	5	13
ENSMUSG00000059483	GM5528	1	0	0	0	0	0	0	1	3	1	0	0
ENSMUSG00000059486	KBTBD2	8	11	2	9	7	7	49	45	40	53	26	47
ENSMUSG00000059488	OLFR727	0	0	0	0	0	0	0	0	0	0	1	0
ENSMUSG00000059495	ARHGEF12	0	0	1	0	0	0	0	0	0	0	0	0
ENSMUSG00000059498	FCGR3	4	2	1	4	3	1	29	23	7	19	18	12
ENSMUSG00000059518	ZNHIT1	6	5	1	0	6	5	20	23	24	33	22	26
ENSMUSG00000059534	UQCR10	30	27	34	30	25	28	234	199	170	223	179	207
ENSMUSG00000059540	TCEA2	1	0	1	0	1	0	4	4	1	4	3	4
ENSMUSG00000059552	TRP53	21	20	27	24	25	21	103	94	72	90	69	82
ENSMUSG00000059554	CCDC28A	1	1	1	3	3	1	13	11	4	9	13	7
ENSMUSG00000059555	TOR4A	0	2	2	2	0	3	5	9	9	10	4	4
ENSMUSG00000059565	GM5292	0	0	1	1	0	2	1	2	2	1	1	0
ENSMUSG00000059586	NSMCE2	3	6	2	9	2	7	34	47	40	39	37	35
ENSMUSG00000059588	CALCRL	12	21	11	11	15	12	60	52	41	57	41	46
ENSMUSG00000059625	SOHLH1	0	0	0	0	0	0	0	0	1	0	0	0
ENSMUSG00000059631	1500035N22I	0	0	0	0	0	0	1	2	1	1	1	1
ENSMUSG00000059659	GM10069	0	0	0	0	1	0	2	0	2	3	1	0
ENSMUSG00000059669	TAF1B	1	2	3	2	1	2	11	5	11	9	12	11
ENSMUSG00000059674	CDH24	1	5	1	0	3	3	7	6	5	8	4	8
ENSMUSG00000059689	ZFP637	3	2	3	0	2	3	9	8	4	7	4	6
ENSMUSG00000059706	A830035A12I	0	0	0	0	0	0	0	0	0	0	1	0
ENSMUSG00000059708	AKAP17B	3	2	3	6	2	2	8	9	13	17	12	7
ENSMUSG00000059713	RCAN3	0	1	2	1	1	1	2	6	2	2	3	2
ENSMUSG00000059714	FLOT1	1	1	1	1	4	2	5	12	9	15	10	18
ENSMUSG00000059734	NDUFS8	8	6	7	6	5	6	46	52	36	39	34	44
ENSMUSG00000059742	KCNH7	0	0	1	0	0	0	7	2	3	7	1	1
ENSMUSG00000059743	FDPS	3	2	7	2	2	2	16	25	5	11	9	17
ENSMUSG00000059751	RPS3A3	0	1	1	1	0	0	3	2	3	0	2	2
ENSMUSG00000059772	SLX1B	0	5	3	6	6	14	18	17	13	17	12	13
ENSMUSG00000059775	RPS26-PS1	1	0	0	2	3	2	2	2	0	4	3	5
ENSMUSG00000059776	RPL13-PS6	1	0	0	0	1	1	1	2	1	1	1	0
ENSMUSG00000059791	NRM	5	2	5	6	5	3	21	16	19	14	13	11
ENSMUSG00000059796	EIF4A1	120	122	128	157	139	115	1037	1039	734	995	766	870
ENSMUSG00000059810	RGS3	3	2	1	3	1	4	8	12	6	8	5	9

ENSMUSG00000059811	ATL2	0	3	4	0	1	4	16	9	5	14	20	11
ENSMUSG00000059816	FAM159A	1	0	0	0	0	1	0	4	0	0	2	1
ENSMUSG00000059820	AU019823	10	5	12	7	3	8	41	50	37	53	36	40
ENSMUSG00000059824	DBP	1	0	3	0	1	2	10	8	4	6	3	5
ENSMUSG00000059834	SCLT1	0	7	4	16	3	8	33	40	18	43	20	15
ENSMUSG00000059835	RPL13-PS3	0	0	1	0	0	2	1	3	0	1	1	1
ENSMUSG00000059839	ZFP874B	1	1	2	4	3	4	9	6	8	11	6	9
ENSMUSG00000059842	ZFP341	0	0	0	0	0	1	1	2	1	3	0	4
ENSMUSG00000059851	KMT5C	5	9	12	5	11	6	38	36	32	43	29	35
ENSMUSG00000059857	NTNG1	0	0	0	1	0	0	0	0	0	0	0	1
ENSMUSG00000059866	TNIP2	2	0	1	2	1	0	9	16	11	8	5	5
ENSMUSG00000059883	IRAK4	2	1	0	1	4	2	4	4	7	4	4	6
ENSMUSG00000059890	UBE4A	5	6	18	16	7	11	42	42	29	46	30	30
ENSMUSG00000059895	PTP4A3	7	2	3	7	5	3	28	20	20	20	15	23
ENSMUSG00000059897	ZFP930	2	1	1	7	3	3	15	12	5	25	9	8
ENSMUSG00000059912	RPL21-PS6	2	1	2	3	0	3	3	3	1	3	1	2
ENSMUSG00000059920	4930453N24I	4	4	2	3	9	3	31	19	16	13	12	18
ENSMUSG00000059923	GRB2	12	12	16	21	17	11	72	70	67	92	74	46
ENSMUSG00000059939	9430015G10I	1	3	3	1	4	2	17	8	9	13	15	12
ENSMUSG00000059970	HSPA2	3	0	0	1	0	1	2	8	5	5	4	3
ENSMUSG00000059975	ZFP74	3	3	2	1	4	3	14	18	7	19	7	11
ENSMUSG00000059981	TAOK2	14	20	9	19	20	15	63	74	50	75	48	83
ENSMUSG00000059994	FCRL1	0	0	0	0	0	0	0	0	0	0	1	1
ENSMUSG00000059995	ATXN7L3	1	3	4	5	1	3	30	18	17	26	15	20
ENSMUSG00000060002	CHPT1	3	3	2	3	7	1	18	12	8	11	10	8
ENSMUSG00000060012	KIF13B	2	2	6	2	8	8	18	27	17	23	18	15
ENSMUSG00000060019	GM10073	3	8	7	10	7	5	29	27	18	19	29	28
ENSMUSG00000060032	H2AFJ	22	31	37	39	50	23	265	230	165	238	190	205
ENSMUSG00000060036	RPL3	77	106	98	130	91	95	668	638	454	665	487	529
ENSMUSG00000060038	DHPS	3	3	2	7	8	2	28	38	18	26	18	27
ENSMUSG00000060068	GM12222	0	0	0	0	0	0	1	1	0	0	0	0
ENSMUSG00000060073	PSMA3	18	25	17	28	35	13	203	197	141	190	153	138
ENSMUSG00000060090	RP2	2	6	1	5	1	4	22	24	14	20	16	17
ENSMUSG00000060093	HIST1H4A	0	0	2	0	1	0	1	0	1	0	0	1
ENSMUSG00000060098	PRMT7	2	6	5	7	5	5	29	29	24	29	28	26
ENSMUSG00000060121	GEMIN2	2	0	2	4	1	1	17	12	17	16	9	13
ENSMUSG00000060126	TPT1	15	23	11	22	19	22	190	196	149	175	127	172
ENSMUSG00000060128	GM2710	0	0	1	0	0	0	0	0	1	1	1	0
ENSMUSG00000060131	ATP8B4	0	2	5	3	2	1	1	2	1	3	0	1
ENSMUSG00000060143	GM10076	6	17	18	17	18	9	49	51	44	51	35	46
ENSMUSG00000060147	SERPINB6A	3	8	14	15	1	3	51	35	28	58	27	41
ENSMUSG00000060149	BC002059	4	1	4	5	4	6	40	24	22	53	15	13
ENSMUSG00000060152	POP5	8	10	11	24	13	18	99	130	85	91	95	86
ENSMUSG00000060166	ZDHHC8	6	4	3	11	7	6	29	42	23	32	23	23
ENSMUSG00000060176	KIF27	0	0	0	0	0	0	1	0	0	0	0	0
ENSMUSG00000060181	SLC35E3	4	7	4	3	5	4	8	18	12	10	5	10
ENSMUSG00000060183	CXCL11	1	0	0	0	0	0	0	0	0	0	0	0
ENSMUSG00000060198	GM11353	0	0	1	1	2	1	4	0	5	2	2	2
ENSMUSG00000060201	SPINK7	0	0	0	0	0	0	0	1	0	0	0	0
ENSMUSG00000060216	ARRB2	6	6	6	7	8	4	40	43	48	28	28	35
ENSMUSG00000060224	PYROXD2	0	0	1	0	0	0	0	0	0	0	0	0
ENSMUSG00000060227	CASC4	21	29	17	19	23	31	61	64	45	67	63	64
ENSMUSG00000060244	ALYREF2	13	20	23	13	29	8	81	83	57	70	52	77
ENSMUSG00000060261	GTF2I	12	18	7	12	17	16	87	82	52	79	73	85
ENSMUSG00000060268	GM1661	0	0	0	0	0	0	1	0	0	0	0	0
ENSMUSG00000060275	NRG2	0	0	0	0	0	0	0	1	0	0	0	0
ENSMUSG00000060279	AP2A1	10	10	17	5	11	3	32	50	27	35	28	43
ENSMUSG00000060288	PPIH	8	7	4	8	14	8	40	46	34	46	21	44

ENSMUSG00000060301	2610008E11F	1	0	0	4	0	4	4	5	2	7	2	7
ENSMUSG00000060314	ZFP941	0	0	0	0	0	0	0	1	0	0	0	0
ENSMUSG00000060371	CALN1	0	1	0	0	0	0	0	0	0	1	0	0
ENSMUSG00000060373	HNRNPC	19	25	30	43	33	27	206	193	145	235	161	196
ENSMUSG00000060376	BCKDHA	45	60	60	69	54	58	185	216	169	187	160	188
ENSMUSG00000060377	RPL36A-PS1	1	1	3	4	1	2	3	13	5	10	7	12
ENSMUSG00000060380	C030014I23R	0	0	1	0	0	0	0	0	0	0	0	0
ENSMUSG00000060397	ZFP128	0	0	0	0	0	1	1	0	0	1	4	0
ENSMUSG00000060402	CHST8	0	0	0	0	0	0	0	1	0	0	0	0
ENSMUSG00000060419	RPS16-PS2	5	7	10	17	11	12	41	59	41	45	38	43
ENSMUSG00000060427	ZFP868	0	1	0	1	0	0	5	5	1	5	4	5
ENSMUSG00000060438	RPS10-PS1	7	10	4	8	6	5	23	28	23	21	24	15
ENSMUSG00000060441	TRIM5	6	1	2	4	1	1	11	13	8	5	14	13
ENSMUSG00000060450	RNF14	4	2	4	9	4	2	28	22	10	33	14	11
ENSMUSG00000060470	ADGRG3	1	0	1	0	0	1	0	0	0	0	0	0
ENSMUSG00000060475	WTAP	81	89	103	110	102	98	512	566	431	605	383	444
ENSMUSG00000060477	IRAK2	0	1	1	1	2	2	6	10	7	2	4	4
ENSMUSG00000060487	SAMD5	0	0	0	0	0	0	0	0	0	0	0	1
ENSMUSG00000060509	XCR1	1	0	0	0	0	0	0	1	0	1	0	0
ENSMUSG00000060510	ZFP266	5	10	11	7	11	8	47	55	32	51	31	49
ENSMUSG00000060519	TOR3A	0	1	3	1	0	1	0	4	2	2	1	2
ENSMUSG00000060534	DCC	0	0	0	0	0	0	0	0	0	1	0	0
ENSMUSG00000060538	TMEM219	1	2	2	6	1	3	14	9	8	10	11	17
ENSMUSG00000060548	TNFRSF19	15	12	11	22	19	13	86	99	56	75	60	55
ENSMUSG00000060566	RPL21-PS3	0	2	1	1	0	1	3	2	1	0	3	4
ENSMUSG00000060579	FHIT	0	0	1	0	0	0	0	0	1	0	0	2
ENSMUSG00000060591	IFITM2	0	1	2	1	3	1	6	3	3	5	1	5
ENSMUSG00000060600	ENO3	1	0	3	0	1	0	17	8	3	3	6	2
ENSMUSG00000060601	NR1H2	4	2	5	6	4	4	15	13	10	13	15	20
ENSMUSG00000060636	RPL35A	78	76	85	106	102	86	355	354	282	327	278	347
ENSMUSG00000060639	HIST1H4I	0	0	0	0	0	0	1	0	1	0	0	0
ENSMUSG00000060657	MARF1	10	19	10	19	13	17	65	46	48	71	52	56
ENSMUSG00000060671	ATP8B2	13	11	15	24	20	21	60	69	39	72	44	54
ENSMUSG00000060675	PLA2G16	0	0	0	0	0	1	0	0	0	0	0	0
ENSMUSG00000060678	HIST1H4C	0	0	0	1	1	0	0	2	1	0	1	1
ENSMUSG00000060679	MRPS9	10	15	18	18	14	16	101	87	64	109	79	64
ENSMUSG00000060681	SLC9A6	1	0	1	1	0	2	9	7	6	7	4	5
ENSMUSG00000060703	CD302	0	1	0	0	0	0	2	0	4	0	0	0
ENSMUSG00000060708	BLOC1S4	4	4	3	4	4	2	14	19	15	22	9	11
ENSMUSG00000060715	1700019A02F	1	0	0	0	0	1	0	3	0	4	1	2
ENSMUSG00000060716	PLEKHH1	0	1	0	0	0	0	4	3	0	3	2	0
ENSMUSG00000060727	GM12571	0	1	0	0	0	0	0	0	0	0	0	0
ENSMUSG00000060730	GM10086	30	33	29	29	31	40	106	111	99	127	99	114
ENSMUSG00000060733	IPMK	1	2	2	3	5	4	14	16	9	13	7	8
ENSMUSG00000060739	NSA2	6	9	6	15	16	5	47	51	42	41	33	40
ENSMUSG00000060743	H3F3A	9	6	10	16	11	11	110	116	91	177	81	73
ENSMUSG00000060771	TSGA10	2	2	1	1	1	1	4	3	2	0	2	2
ENSMUSG00000060791	GMFG	6	1	6	6	6	6	24	36	29	39	22	26
ENSMUSG00000060794	TSSK5	0	0	0	0	0	0	1	0	0	0	0	0
ENSMUSG00000060798	INTU	4	4	5	6	6	8	41	41	17	42	23	33
ENSMUSG00000060802	B2M	41	60	63	67	56	47	380	349	274	364	262	348
ENSMUSG00000060803	GSTP1	0	0	0	1	0	0	0	1	2	3	2	5
ENSMUSG00000060843	CTNNA3	0	0	0	0	0	0	1	0	0	0	0	0
ENSMUSG00000060860	UBE2S	1	3	2	2	1	1	4	3	4	3	2	4
ENSMUSG00000060862	ZBTB40	1	2	1	1	1	4	5	6	3	7	4	7
ENSMUSG00000060904	ARL1	6	7	14	12	12	12	62	54	41	59	48	50
ENSMUSG00000060923	ACYP2	0	2	1	1	1	0	8	8	4	7	5	8
ENSMUSG00000060935	TMEM263	11	11	9	14	13	16	61	51	37	49	27	47

ENSMUSG00000060938	RPL26	147	199	159	219	200	218	874	962	666	878	700	774
ENSMUSG00000060950	TRMT61A	1	0	2	3	0	4	12	6	10	7	14	14
ENSMUSG00000060961	SLC4A4	1	0	0	0	0	0	4	1	4	4	3	2
ENSMUSG00000060962	DMKN	0	0	0	0	0	1	0	0	0	0	0	0
ENSMUSG00000060981	HIST1H4H	1	0	0	0	0	2	1	1	2	0	0	2
ENSMUSG00000060985	TDRD5	1	0	0	0	0	0	1	0	2	3	1	1
ENSMUSG00000060992	COPZ1	5	8	5	5	5	4	36	38	28	40	34	32
ENSMUSG00000061024	RRS1	9	15	12	17	13	18	107	100	82	96	77	79
ENSMUSG00000061028	CLASRP	9	6	13	11	11	8	61	82	66	65	51	51
ENSMUSG00000061032	RRP1	17	21	20	16	17	24	87	114	74	112	83	109
ENSMUSG00000061039	OLFR920	0	1	0	1	1	0	0	1	0	0	0	0
ENSMUSG00000061046	HAGHL	3	2	4	3	4	2	30	23	15	27	14	23
ENSMUSG00000061062	GM10093	0	0	0	0	0	1	0	1	0	1	0	1
ENSMUSG00000061079	ZFP143	0	0	2	2	2	0	7	8	6	12	4	5
ENSMUSG00000061086	MYL4	0	1	0	1	0	0	9	8	5	9	3	9
ENSMUSG00000061104	SAP18B	0	2	1	5	1	3	13	7	6	4	8	6
ENSMUSG00000061111	MCRIP1	3	0	1	6	2	2	14	16	13	16	10	22
ENSMUSG00000061118	DNAJC30	0	2	1	3	1	1	6	6	7	3	8	3
ENSMUSG00000061119	PRCP	1	1	0	1	0	0	10	13	6	13	7	13
ENSMUSG00000061130	PPM1B	3	4	4	4	3	2	35	28	17	32	27	25
ENSMUSG00000061132	BLNK	0	0	0	0	0	0	0	1	1	0	0	0
ENSMUSG00000061136	PRPF40A	72	97	88	95	100	76	391	446	311	444	339	330
ENSMUSG00000061167	RPL15-PS3	0	0	1	0	0	0	0	1	0	0	1	0
ENSMUSG00000061175	FNIP2	9	10	7	8	9	5	32	39	30	43	30	43
ENSMUSG00000061186	SFMBT2	15	14	14	25	9	17	92	90	51	74	54	54
ENSMUSG00000061207	STK19	2	5	3	1	10	2	16	16	11	23	16	16
ENSMUSG00000061232	H2-K1	81	104	84	121	107	111	610	575	424	545	424	520
ENSMUSG00000061242	GM9104	0	0	0	0	0	0	1	1	1	2	0	0
ENSMUSG00000061244	EXOC5	20	12	21	22	19	20	105	106	66	105	95	87
ENSMUSG00000061272	GM14173	1	0	0	0	0	0	0	0	0	0	0	0
ENSMUSG00000061273	MMGT1	0	4	5	1	7	1	9	16	11	11	6	12
ENSMUSG00000061286	EXOSC5	5	7	6	2	8	6	46	31	28	31	21	24
ENSMUSG00000061288	TAOK3	10	16	11	28	25	26	69	84	65	83	63	62
ENSMUSG00000061306	SLC38A10	273	321	296	336	408	318	1409	1509	1091	1447	1098	1265
ENSMUSG00000061313	DDHD2	6	5	4	3	0	9	15	20	20	22	20	29
ENSMUSG00000061315	NACA	28	30	28	45	48	38	300	346	263	284	263	299
ENSMUSG00000061322	DNAIC1	0	0	0	0	0	0	0	0	0	1	0	0
ENSMUSG00000061330	GM6436	0	0	0	0	0	0	0	1	0	0	0	0
ENSMUSG00000061356	NUGGC	2	0	0	1	1	1	11	11	4	10	9	2
ENSMUSG00000061360	PHF5A	8	12	14	8	12	6	34	60	31	42	31	36
ENSMUSG00000061371	ZFP873	2	1	1	0	1	4	6	2	2	5	0	4
ENSMUSG00000061374	FIZ1	4	2	0	3	4	2	19	19	5	7	16	14
ENSMUSG00000061390	UBA52-PS	0	0	0	0	0	0	0	2	1	0	0	0
ENSMUSG00000061393	ACVR2B	0	0	0	0	0	0	0	1	1	1	0	0
ENSMUSG00000061410	ZCCHC14	0	0	0	0	0	0	1	0	0	0	0	0
ENSMUSG00000061411	NOL4L	2	0	1	3	0	3	6	11	5	5	3	9
ENSMUSG00000061414	CRACR2A	2	3	1	4	3	2	8	17	7	14	9	17
ENSMUSG00000061436	HIPK2	2	1	5	7	5	5	11	11	7	13	11	14
ENSMUSG00000061455	STX17	2	4	2	2	1	2	9	13	8	16	9	10
ENSMUSG00000061458	NOL10	3	4	7	3	6	5	41	36	29	43	37	26
ENSMUSG00000061461	SMIM20	0	4	3	4	1	0	4	4	1	7	3	3
ENSMUSG00000061474	MRPS36	1	2	0	0	2	0	4	5	10	4	6	2
ENSMUSG00000061477	RPS7	55	60	57	75	79	80	344	450	295	326	293	346
ENSMUSG00000061479	SNRPA	12	4	10	6	4	4	45	50	47	43	44	46
ENSMUSG00000061482	HIST1H4D	3	0	1	2	1	0	3	2	0	1	2	6
ENSMUSG00000061488	RPL27A-PS1	0	0	0	0	0	0	1	0	0	0	0	0
ENSMUSG00000061510	GM10101	0	0	1	1	4	1	7	8	4	3	3	5
ENSMUSG00000061518	COX5B	27	27	30	32	23	38	249	310	198	247	195	258

ENSMUSG00000061524	ZIC2	0	1	0	1	0	1	11	2	3	5	3	6
ENSMUSG00000061531	TMEM236	0	0	0	0	0	0	1	0	0	0	0	0
ENSMUSG00000061533	CEP128	3	2	3	6	6	2	17	23	7	26	17	14
ENSMUSG00000061536	SEC22C	0	1	5	2	3	1	4	7	5	3	4	5
ENSMUSG00000061544	ZFP229	1	0	0	0	0	0	0	0	1	0	1	0
ENSMUSG00000061559	WDR61	8	8	9	8	8	9	57	45	44	70	35	44
ENSMUSG00000061561	OLFR373	0	0	0	0	0	0	0	1	1	0	0	0
ENSMUSG00000061589	DOT1L	23	19	29	35	21	30	117	124	84	125	94	94
ENSMUSG00000061603	AKAP6	0	0	0	0	0	0	0	0	0	0	1	0
ENSMUSG00000061607	MDC1	6	10	4	0	10	5	24	36	15	33	24	34
ENSMUSG00000061613	U2AF1	26	23	22	27	17	19	108	129	90	129	81	135
ENSMUSG00000061615	HIST1H2AB	0	0	0	0	1	0	1	2	0	2	0	1
ENSMUSG00000061650	MED9	1	2	4	1	1	4	10	9	4	13	13	22
ENSMUSG00000061665	CD2AP	7	21	15	25	19	13	103	109	53	114	78	74
ENSMUSG00000061666	GDPD1	0	1	1	2	0	1	3	2	2	4	2	3
ENSMUSG00000061669	RPL34-PS2	0	0	1	0	1	0	1	1	0	1	0	0
ENSMUSG00000061684	RPL21-PS8	1	0	0	1	0	1	4	4	3	12	3	4
ENSMUSG00000061689	DLGAP4	6	11	4	8	13	6	50	41	33	38	36	47
ENSMUSG00000061702	TMEM91	5	2	2	3	2	2	7	8	2	7	6	9
ENSMUSG00000061723	TNNT3	1	0	0	0	0	0	1	0	0	1	0	0
ENSMUSG00000061728	BTNL7-PS	0	0	0	2	0	1	1	2	1	0	0	0
ENSMUSG00000061731	EXT1	16	16	22	12	17	10	79	79	44	88	60	63
ENSMUSG00000061751	KALRN	9	8	6	11	11	8	23	15	16	29	14	21
ENSMUSG00000061755	BOD1L	30	30	25	38	35	25	91	77	56	106	62	74
ENSMUSG00000061758	AKR1B10	3	6	4	1	7	2	26	14	17	22	23	16
ENSMUSG00000061759	ARMT1	3	6	3	1	4	1	25	24	23	22	16	22
ENSMUSG00000061778	MOSPD2	2	4	2	2	2	1	20	8	14	19	10	25
ENSMUSG00000061780	CFD	0	0	0	0	0	0	0	0	0	1	0	0
ENSMUSG00000061787	RPS17	134	137	180	172	165	165	974	1058	755	1051	903	959
ENSMUSG00000061815	RUFY4	0	0	0	0	0	0	0	1	0	0	0	0
ENSMUSG00000061816	MYL1	0	0	0	0	0	0	6	4	5	5	3	3
ENSMUSG00000061833	GM6311	0	1	0	1	0	0	0	1	1	2	0	0
ENSMUSG00000061838	SUCLG2	0	0	2	0	0	1	1	2	1	0	0	2
ENSMUSG00000061848	GM5805	0	2	5	3	5	8	26	14	18	15	20	30
ENSMUSG00000061859	PATJ	2	0	1	1	1	1	5	5	3	4	3	5
ENSMUSG00000061878	SPHK1	0	0	0	1	0	0	0	0	0	0	0	0
ENSMUSG00000061882	CCDC62	0	0	1	1	0	0	1	0	3	0	0	2
ENSMUSG00000061887	SSBP3	30	30	30	38	38	44	119	95	83	133	88	92
ENSMUSG00000061894	ZSCAN20	1	1	3	0	4	1	2	4	5	6	7	8
ENSMUSG00000061904	SLC25A3	54	55	48	70	75	66	412	481	312	468	347	366
ENSMUSG00000061911	MYT1L	0	0	0	0	0	0	0	0	1	0	0	0
ENSMUSG00000061950	PPP4R1	3	5	1	3	3	4	20	18	10	17	13	16
ENSMUSG00000061972	OLFR99	0	0	0	0	0	0	0	0	0	0	1	0
ENSMUSG00000061979	RCC1L	3	2	3	3	3	4	23	22	16	30	22	19
ENSMUSG00000061981	FLOT2	1	1	3	1	3	1	10	17	11	9	6	9
ENSMUSG00000061983	RPS12	314	320	351	390	397	357	2107	2076	1553	2129	1646	1958
ENSMUSG00000061984	OLFR392	0	0	1	0	0	0	0	0	0	0	0	0
ENSMUSG00000061988	RPL10A-PS2	0	3	2	3	3	2	3	5	2	3	3	4
ENSMUSG00000061991	HIST1H2AF	0	0	0	1	0	1	0	1	1	2	1	0
ENSMUSG00000061992	GM4953	0	1	0	0	0	0	0	3	0	0	0	2
ENSMUSG00000062006	RPL34	282	349	342	442	378	357	1914	1887	1448	1926	1491	1714
ENSMUSG00000062007	HSH2D	2	1	1	2	0	1	9	5	1	1	2	8
ENSMUSG00000062014	GMFB	5	8	5	9	6	6	54	51	44	61	38	44
ENSMUSG00000062031	PGGHG	3	1	0	4	1	0	4	6	6	8	3	3
ENSMUSG00000062040	ZFP27	2	1	4	2	0	5	7	8	10	5	9	5
ENSMUSG00000062044	LMTK3	0	0	0	0	1	0	1	0	1	1	0	0
ENSMUSG00000062054	IAH1	0	3	4	5	2	1	14	13	9	16	10	13
ENSMUSG00000062070	PGK1	0	1	0	1	1	0	13	12	4	6	8	6

ENSMUSG00000062074	WISP3	0	0	0	1	0	0	0	0	0	0	0	0
ENSMUSG00000062075	LMNB2	5	5	5	2	3	3	29	17	19	25	18	20
ENSMUSG00000062078	QK	14	10	14	15	16	14	73	82	55	86	54	52
ENSMUSG00000062081	GM6055	0	0	0	0	0	0	0	0	0	1	0	0
ENSMUSG00000062082	CD200R4	0	1	0	3	5	0	8	4	3	9	3	6
ENSMUSG00000062083	RPL13A-PS1	59	59	42	51	45	68	188	173	137	146	103	118
ENSMUSG00000062093	GM10110	4	5	3	2	0	5	8	9	11	10	8	12
ENSMUSG00000062101	ZFP119B	1	0	0	0	0	1	2	0	1	0	2	0
ENSMUSG00000062110	SCFD2	1	0	3	3	4	2	10	21	16	13	14	16
ENSMUSG00000062115	RAI1	6	15	11	18	17	11	37	47	31	49	35	48
ENSMUSG00000062116	ZFP954	2	0	2	1	2	1	14	7	15	9	12	8
ENSMUSG00000062127	CTTNBP2NL	0	0	0	0	0	0	0	0	1	0	0	0
ENSMUSG00000062152	RPL21-PS14	0	0	0	0	0	0	2	2	1	2	2	2
ENSMUSG00000062168	PPEF1	0	0	0	0	0	1	0	0	0	0	0	0
ENSMUSG00000062169	CNIH4	6	9	13	8	13	9	41	43	35	53	32	45
ENSMUSG00000062170	FMR1NB	0	0	0	0	0	0	0	1	0	1	0	1
ENSMUSG00000062175	TGIF2	1	1	0	0	0	0	4	4	2	3	3	6
ENSMUSG00000062190	LANCL2	0	1	2	0	3	1	3	7	1	5	6	2
ENSMUSG00000062198	2700097O09I	5	9	4	2	5	5	24	21	18	41	26	17
ENSMUSG00000062202	BTBD9	19	21	20	32	17	19	77	81	69	75	67	74
ENSMUSG00000062203	GSPT1	15	16	28	24	19	17	95	115	80	115	103	103
ENSMUSG00000062209	ERBB4	0	0	0	0	0	0	1	2	0	2	1	0
ENSMUSG00000062210	TNFAIP8	3	11	8	10	5	5	27	26	28	40	23	23
ENSMUSG00000062232	RAPGEF2	0	7	4	6	13	9	30	25	18	31	25	21
ENSMUSG00000062234	GAK	8	10	5	9	7	7	43	46	35	29	20	32
ENSMUSG00000062248	CKS2	2	3	2	1	2	2	20	18	15	24	27	15
ENSMUSG00000062270	MORF4L1	6	9	9	10	10	4	37	39	43	73	37	39
ENSMUSG00000062278	GM11562	0	0	0	0	0	1	0	0	0	0	0	1
ENSMUSG00000062279	GM10237	0	0	0	0	0	0	0	0	1	0	0	0
ENSMUSG00000062284	GM6030	2	2	2	1	1	1	1	3	3	8	3	4
ENSMUSG00000062327		0	0	0	0	0	0	1	0	0	1	0	0
ENSMUSG00000062328	RPL17	131	163	163	164	163	198	847	837	639	880	618	730
ENSMUSG00000062352	ITGB1BP1	2	4	2	3	4	3	19	12	20	17	17	23
ENSMUSG00000062353	GM15772	2	4	10	4	4	3	18	28	20	27	16	12
ENSMUSG00000062365	GM4968	0	0	0	0	0	0	0	1	0	0	1	0
ENSMUSG00000062373	TMEM65	6	9	9	5	9	10	32	44	35	58	26	40
ENSMUSG00000062376	BORCS7	3	2	5	5	2	5	16	13	11	9	18	15
ENSMUSG00000062382	FTL1-PS1	8	9	13	11	16	15	37	38	19	30	20	25
ENSMUSG00000062397	ZFP706	13	28	9	27	27	9	126	100	105	128	85	98
ENSMUSG00000062421	ARF2	1	3	1	0	1	2	5	5	3	7	3	2
ENSMUSG00000062456	RPL9-PS6	23	36	45	35	48	34	123	170	126	142	105	106
ENSMUSG00000062458	GM8623	0	1	0	0	0	0	0	1	0	1	2	3
ENSMUSG00000062461	GM5453	0	0	0	0	0	0	0	1	1	0	0	1
ENSMUSG00000062470	FBXL12OS	0	0	1	1	0	1	4	2	5	2	2	6
ENSMUSG00000062472	GM4945	0	0	0	0	0	0	1	0	0	0	0	0
ENSMUSG00000062480	ACAT3	3	4	3	4	2	5	17	18	17	27	12	21
ENSMUSG00000062510	NSL1	5	8	8	5	8	3	30	38	23	31	15	25
ENSMUSG00000062515	FABP4	0	0	0	0	0	0	0	0	0	0	1	0
ENSMUSG00000062519	ZFP398	3	2	4	7	10	8	18	24	16	23	18	22
ENSMUSG00000062526	MPPE1	2	2	5	6	3	2	18	12	7	18	10	14
ENSMUSG00000062545	TLR12	7	7	5	14	6	5	20	22	18	25	12	24
ENSMUSG00000062554	GM12751	1	0	0	0	0	1	1	0	0	0	0	0
ENSMUSG00000062580	TIMM17A	6	5	3	8	7	11	57	58	41	55	38	42
ENSMUSG00000062582	RPL30-PS8	0	0	0	0	0	0	0	0	0	1	0	1
ENSMUSG00000062585	CNR2	0	0	0	0	0	0	1	0	0	0	0	0
ENSMUSG00000062590	ARMC9	1	3	2	2	0	1	7	14	4	4	6	7
ENSMUSG00000062591	TUBB4A	0	0	0	0	1	1	2	2	0	2	0	1
ENSMUSG00000062604	SRPK2	31	49	38	61	52	40	202	191	151	210	166	165

ENSMUSG00000062611	RPS3A2	2	5	1	2	4	1	13	11	13	15	7	7
ENSMUSG00000062619	2310039H08I	5	5	6	6	4	1	22	25	22	25	16	14
ENSMUSG00000062627	MYSM1	9	17	16	20	17	14	75	70	44	83	39	59
ENSMUSG00000062646	GANC	2	2	1	1	3	3	10	16	12	14	13	17
ENSMUSG00000062647	RPL7A	141	200	131	164	176	157	1104	1219	847	1096	851	1039
ENSMUSG00000062661	NCS1	0	0	0	0	0	0	0	0	1	0	0	0
ENSMUSG00000062683	ATP5G2	0	0	0	1	1	0	1	3	1	1	2	0
ENSMUSG00000062687	CKS1BRT	1	7	6	7	6	5	21	14	20	19	12	12
ENSMUSG00000062691	CEBPZOS	2	3	7	1	8	3	22	29	22	14	13	21
ENSMUSG00000062704	9430002A10I	0	0	0	0	0	0	1	1	2	0	1	0
ENSMUSG00000062729	PPOX	5	5	9	2	1	7	25	22	21	19	20	18
ENSMUSG00000062742	GM5239	0	0	0	0	0	1	0	2	2	1	1	3
ENSMUSG00000062743	ZFP677	0	0	0	0	2	0	5	6	0	4	2	6
ENSMUSG00000062753	AI413582	5	3	7	4	2	5	31	35	22	29	26	24
ENSMUSG00000062758	GM16477	0	0	0	0	0	0	1	1	1	0	0	0
ENSMUSG00000062761	ZFP512	5	3	3	7	6	5	17	33	15	24	18	17
ENSMUSG00000062762	EI24	3	2	5	12	6	4	20	22	11	20	8	12
ENSMUSG00000062778	CHIA1	0	0	0	0	0	0	2	1	0	1	0	1
ENSMUSG00000062782	OLFR527	0	0	1	0	0	0	0	0	0	0	0	0
ENSMUSG00000062794	ZFP599	2	1	0	3	0	0	5	8	11	6	6	7
ENSMUSG00000062797	HIKESHI	5	11	5	9	14	6	59	62	43	58	33	49
ENSMUSG00000062822	4833420G17I	7	16	12	26	12	20	88	81	76	101	70	71
ENSMUSG00000062825	ACTG1	0	0	0	2	0	4	9	0	0	3	2	2
ENSMUSG00000062846	GM14176	0	0	0	0	0	0	0	0	0	0	0	1
ENSMUSG00000062866	PHACTR2	1	3	5	3	2	5	18	5	10	16	10	16
ENSMUSG00000062867	IMPDH2	17	9	8	30	10	14	86	97	78	80	65	73
ENSMUSG00000062874	GM4978	1	0	0	0	0	0	1	0	1	0	1	0
ENSMUSG00000062896	RPL31-PS11	0	0	0	0	0	0	1	0	0	0	0	0
ENSMUSG00000062901	KLHL24	14	8	10	11	12	10	38	31	41	32	21	27
ENSMUSG00000062905	VMN1R32	0	0	0	0	0	1	2	2	0	1	2	2
ENSMUSG00000062906	HDAC10	1	9	6	8	6	5	30	23	21	27	27	17
ENSMUSG00000062908	ACADM	10	10	6	11	6	10	72	72	50	73	64	54
ENSMUSG00000062929	CFL2	1	2	6	5	3	2	27	19	14	24	10	17
ENSMUSG00000062931	ZFP938	4	3	2	2	6	6	23	36	22	24	19	26
ENSMUSG00000062933	GM10123	1	0	0	0	0	0	0	1	0	0	0	1
ENSMUSG00000062937	MTAP	4	2	1	4	9	6	26	44	17	51	17	45
ENSMUSG00000062939	STAT4	2	3	2	2	3	2	8	8	6	15	9	16
ENSMUSG00000062944	9130023H24I	1	2	0	1	2	1	6	5	1	1	2	1
ENSMUSG00000062949	ATP11C	4	7	3	8	12	5	14	19	14	24	19	19
ENSMUSG00000062962	GM6378	0	0	0	0	0	0	0	0	0	0	1	0
ENSMUSG00000062963	UFC1	11	10	10	10	15	11	77	59	61	71	49	55
ENSMUSG00000062980	CPED1	0	0	0	0	0	0	0	1	3	1	0	0
ENSMUSG00000062981	MRPL42	19	18	13	20	23	13	112	124	113	126	100	103
ENSMUSG00000062995	ICA1	1	3	3	1	2	3	4	8	8	7	8	5
ENSMUSG00000062997	RPL35	81	78	78	70	87	69	598	566	447	573	437	492
ENSMUSG00000063001	GM9701	0	0	2	0	1	1	1	1	1	0	1	3
ENSMUSG00000063015	CCNI	9	5	11	8	13	9	50	38	44	51	42	38
ENSMUSG00000063018	2010204K13I	5	4	4	4	5	3	24	44	23	25	29	31
ENSMUSG00000063019	MANBAL	0	4	5	2	5	5	30	24	23	25	18	15
ENSMUSG00000063021	HIST1H2AK	0	0	1	1	0	1	1	0	1	1	2	0
ENSMUSG00000063047	ZFP780B	4	6	2	1	2	1	12	21	13	15	13	11
ENSMUSG00000063049	ING2	4	3	5	0	6	7	31	36	28	19	31	24
ENSMUSG00000063052	LRRC40	7	11	13	8	10	9	89	90	70	85	48	52
ENSMUSG00000063063	CTNNA2	0	0	0	0	0	0	0	0	1	0	0	0
ENSMUSG00000063065	MAPK3	1	5	1	4	2	3	12	22	10	12	18	15
ENSMUSG00000063077	KIF1B	193	292	219	326	282	231	1212	1416	1037	1585	976	1105
ENSMUSG00000063087	GM10125	0	1	0	0	0	0	0	0	0	0	0	0
ENSMUSG00000063108	ZFP26	9	9	11	8	4	10	33	41	23	33	32	35

ENSMUSG00000063129	ALDOART2	0	0	0	0	0	0	1	0	0	0	0	0
ENSMUSG00000063130	CALML3	0	0	0	0	0	0	1	0	0	0	0	0
ENSMUSG00000063142	KCNMA1	1	0	0	0	0	0	0	0	0	0	0	0
ENSMUSG00000063146	CLIP2	0	0	0	0	1	0	1	0	0	0	0	0
ENSMUSG00000063160	NUMBL	0	0	1	0	1	1	11	8	3	4	5	2
ENSMUSG00000063163	SPEER2	0	0	1	0	1	0	2	5	5	10	5	3
ENSMUSG00000063172	HSPB11	6	5	3	2	4	5	12	15	15	27	15	12
ENSMUSG00000063179	PSTK	4	2	6	8	11	9	43	36	29	44	35	33
ENSMUSG00000063200	NOL7	36	41	31	58	44	43	212	225	166	223	149	179
ENSMUSG00000063229	LDHA	37	47	36	46	34	34	333	376	245	297	192	215
ENSMUSG00000063235	PTPMT1	5	5	7	4	11	6	45	53	33	45	25	33
ENSMUSG00000063236	1110038F14R	6	4	5	3	4	3	24	21	19	22	18	21
ENSMUSG00000063253	SCOC	3	1	2	6	3	4	18	16	11	19	9	12
ENSMUSG00000063268	PARP10	0	2	2	5	0	5	20	18	22	15	12	14
ENSMUSG00000063273	NAA15	40	91	64	75	71	63	261	327	232	322	217	224
ENSMUSG00000063275	HACD1	14	15	11	12	11	8	44	47	33	45	40	43
ENSMUSG00000063281	ZFP35	3	3	6	4	7	2	15	20	14	23	16	19
ENSMUSG00000063286	GM8995	43	48	64	65	54	56	276	271	174	283	207	204
ENSMUSG00000063316	RPL27	57	77	66	74	69	72	365	419	315	393	301	327
ENSMUSG00000063317	USP31	7	6	11	10	9	9	44	40	25	65	26	35
ENSMUSG00000063320	1190007I07R	2	2	4	5	1	2	16	17	11	19	7	11
ENSMUSG00000063328	GM9396	0	0	0	1	1	0	7	7	1	5	2	2
ENSMUSG00000063334	KRR1	7	16	6	20	15	12	60	69	58	77	53	67
ENSMUSG00000063354	SLC39A4	0	0	1	0	1	0	0	1	0	0	0	1
ENSMUSG00000063358	MAPK1	52	59	63	62	59	63	485	486	328	546	375	365
ENSMUSG00000063362	ALG11	2	6	8	3	8	6	23	14	15	17	17	19
ENSMUSG00000063364	3300002I08R	0	1	1	1	1	1	1	3	2	3	4	2
ENSMUSG00000063382	BCL9L	1	5	4	4	6	6	18	21	11	22	10	21
ENSMUSG00000063406	TMED5	46	50	46	56	47	52	234	284	192	312	179	193
ENSMUSG00000063410	STK24	5	6	4	8	15	9	55	54	29	44	41	32
ENSMUSG00000063412	GM10131	7	7	11	6	9	7	33	24	24	44	23	27
ENSMUSG00000063439	B9D2	2	0	3	1	2	1	5	10	7	3	5	15
ENSMUSG00000063442	GM7117	0	2	1	0	3	0	5	9	3	4	6	6
ENSMUSG00000063445	NMRAL1	4	9	2	13	9	8	46	48	50	50	39	41
ENSMUSG00000063450	SYNE2	1	1	2	1	1	1	0	0	0	1	0	0
ENSMUSG00000063455	D630045J12R	1	1	1	0	0	1	4	5	6	7	2	3
ENSMUSG00000063457	RPS15	104	125	135	138	152	125	716	758	575	711	564	686
ENSMUSG00000063480	SNU13	3	4	3	2	4	3	23	25	15	13	12	16
ENSMUSG00000063488	ZKSCAN7	0	0	0	0	1	0	1	4	1	1	0	2
ENSMUSG00000063511	SNRNP70	31	29	27	47	40	48	237	283	192	236	211	215
ENSMUSG00000063524	ENO1	5	5	4	11	7	5	73	89	59	75	29	61
ENSMUSG00000063529	STMND1	0	0	0	0	0	0	0	0	0	0	0	1
ENSMUSG00000063531	SEMA3E	0	0	0	0	0	0	0	0	1	0	0	0
ENSMUSG00000063535	ZFP773	3	2	1	4	1	6	13	21	16	15	14	11
ENSMUSG00000063548	GM4825	0	0	0	0	0	0	0	0	1	0	0	0
ENSMUSG00000063550	NUP98	19	26	29	21	29	25	134	126	116	116	108	113
ENSMUSG00000063556	GM10132	0	0	1	0	2	1	5	8	2	5	5	3
ENSMUSG00000063558	AOX1	0	0	0	0	0	0	0	1	0	0	0	0
ENSMUSG00000063568	JAZF1	0	0	0	0	0	0	0	4	3	2	1	1
ENSMUSG00000063576	KLHDC3	10	17	11	19	18	24	85	78	65	67	79	83
ENSMUSG00000063594	GNG8	0	0	0	0	0	0	0	0	1	0	0	0
ENSMUSG00000063611	GM10134	0	0	0	0	0	1	0	0	0	0	0	1
ENSMUSG00000063623	C230062I16R	0	1	0	1	0	0	3	0	0	1	1	0
ENSMUSG00000063646	JAKMIP1	3	4	4	9	9	6	20	21	19	20	15	25
ENSMUSG00000063656	GM10135	0	0	0	0	0	0	1	0	0	0	0	0
ENSMUSG00000063659	ZBTB18	8	8	6	6	8	9	28	33	17	29	9	27
ENSMUSG00000063689	HIST2H2AB	1	0	0	2	0	1	0	0	2	1	0	0
ENSMUSG00000063694	CYCS	8	11	5	5	8	9	40	55	33	45	41	42

ENSMUSG00000063696	GM8730	0	0	0	0	0	0	1	1	0	1	0	0
ENSMUSG00000063698	SFXN4	1	0	4	0	3	0	8	4	3	4	8	9
ENSMUSG00000063704	MAPK15	0	0	0	0	0	0	1	1	0	1	0	0
ENSMUSG00000063714	SP3OS	0	2	2	2	2	1	3	6	2	5	4	2
ENSMUSG00000063754	GM10136	1	3	4	1	2	1	7	6	2	6	2	7
ENSMUSG00000063765	CHADL	0	0	0	0	0	1	2	0	1	1	0	0
ENSMUSG00000063785	UTP14A	11	16	16	17	18	18	70	80	67	104	70	70
ENSMUSG00000063787	CHCHD1	8	17	9	13	14	13	87	74	57	97	81	87
ENSMUSG00000063800	PRPF38A	2	2	7	6	7	4	27	34	17	24	21	20
ENSMUSG00000063801	AP3S2	1	3	5	3	4	6	14	14	17	17	18	24
ENSMUSG00000063802	HSPBP1	0	2	4	0	6	3	16	22	12	22	12	22
ENSMUSG00000063804	LIN28B	5	5	6	8	5	9	41	37	23	44	31	30
ENSMUSG00000063808	GPATCH1	8	6	1	4	6	5	22	22	16	32	12	21
ENSMUSG00000063810	ALMS1	2	3	3	3	5	1	8	10	13	15	9	12
ENSMUSG00000063816	GM4987	1	2	1	1	1	1	7	7	5	6	8	5
ENSMUSG00000063820	ARL9	1	1	0	1	0	1	3	1	0	2	2	1
ENSMUSG00000063849	PPCDC	1	2	3	2	3	0	6	7	8	10	9	8
ENSMUSG00000063856	GPX1	7	6	7	10	7	5	35	50	33	53	32	36
ENSMUSG00000063870	CHD4	506	623	650	695	746	673	2577	2838	2111	2869	2203	2370
ENSMUSG00000063875	RPS6-PS1	4	0	0	0	1	0	10	7	6	4	2	4
ENSMUSG00000063882	UQCRH	53	54	66	72	75	66	449	485	386	449	387	391
ENSMUSG00000063884	PTCD3	3	10	9	9	6	4	36	27	15	39	28	33
ENSMUSG00000063887	NLGN1	0	1	0	0	0	0	0	0	0	0	2	0
ENSMUSG00000063888	RPL7L1	9	8	9	8	10	8	87	80	72	89	64	59
ENSMUSG00000063889	CREM	1	1	2	1	4	2	9	8	8	11	11	5
ENSMUSG00000063894	ZKSCAN8	3	1	3	1	0	2	7	7	5	6	5	6
ENSMUSG00000063895	NUPL1	0	0	0	0	0	0	0	0	0	0	1	0
ENSMUSG00000063897	CAAA011183	0	0	1	3	4	0	7	9	10	11	9	13
ENSMUSG00000063902	GM7964	0	0	0	0	1	0	0	1	1	0	0	0
ENSMUSG00000063904	DPP3	5	9	8	11	8	7	52	55	38	50	41	47
ENSMUSG00000063931	PEPD	2	1	1	1	1	1	7	11	1	9	7	9
ENSMUSG00000063952	BRPF3	1	1	3	3	0	3	12	4	14	19	7	9
ENSMUSG00000063953	AMD2	3	3	4	3	1	0	9	13	9	12	4	8
ENSMUSG00000063972	NR6A1	3	1	2	0	8	3	17	20	10	12	8	6
ENSMUSG00000064006	OLFR190	0	0	0	0	0	0	0	0	1	0	0	0
ENSMUSG00000064030	PYM1	9	7	2	3	5	4	31	22	17	15	23	14
ENSMUSG00000064032	GM10143	0	2	2	3	3	5	13	19	15	18	8	19
ENSMUSG00000064036	MRO	0	0	0	0	0	0	0	0	1	1	0	0
ENSMUSG00000064037	GPN1	11	10	6	13	11	8	62	47	30	56	29	49
ENSMUSG00000064043	TRERF1	19	19	24	25	21	17	74	68	62	106	52	69
ENSMUSG00000064061	DZIP3	9	10	18	15	11	8	84	80	43	77	54	55
ENSMUSG00000064065	IPCEF1	12	15	4	11	15	15	43	47	28	40	30	27
ENSMUSG00000064068	MTX1	4	5	8	6	5	6	50	52	33	44	39	42
ENSMUSG00000064080	FBLN2	3	3	2	4	7	2	20	25	12	26	19	18
ENSMUSG00000064081	GM8973	0	0	0	0	1	0	0	1	0	0	0	0
ENSMUSG00000064090	VRK2	1	1	0	3	8	6	15	27	18	13	7	16
ENSMUSG00000064105	CNNM2	5	7	3	2	5	4	18	15	13	23	16	15
ENSMUSG00000064109	HCST	0	0	0	0	0	0	0	1	0	0	0	0
ENSMUSG00000064120	MOCS1	0	0	1	2	0	2	6	4	1	4	0	2
ENSMUSG00000064125	PRR36	0	0	2	0	1	0	3	2	4	3	2	3
ENSMUSG00000064127	MED14	4	9	8	12	5	1	54	43	37	44	46	25
ENSMUSG00000064128	CENPJ	9	11	11	18	12	12	51	53	31	59	40	41
ENSMUSG00000064138	FAM172A	3	2	4	5	8	2	33	26	17	26	26	26
ENSMUSG00000064141	ZFP69	0	2	0	0	1	1	0	2	2	2	0	0
ENSMUSG00000064145	ARIH2	2	4	3	8	7	5	26	9	16	25	27	16
ENSMUSG00000064177	GHRL	0	0	0	0	0	0	1	0	0	1	0	0
ENSMUSG00000064179	TNNT1	5	6	2	9	11	3	3	5	5	7	6	4
ENSMUSG00000064181	RAB3IP	1	0	1	1	3	3	6	12	12	5	8	6

ENSMUSG00000064193	GM4735	0	0	0	0	0	0	0	0	0	0	1	0
ENSMUSG00000064202	4430402I18R	0	0	1	0	0	1	2	2	4	4	3	1
ENSMUSG00000064210	ANO6	13	22	5	19	11	10	36	28	20	33	28	28
ENSMUSG00000064215	IFI27	1	3	1	0	1	0	9	10	5	10	4	7
ENSMUSG00000064231	GM5321	0	1	0	0	0	0	1	0	0	2	1	3
ENSMUSG00000064247	PLCXD1	0	0	0	0	1	1	5	4	4	3	2	3
ENSMUSG00000064254	ETHE1	0	4	2	2	1	0	9	5	3	16	6	5
ENSMUSG00000064259	VMN1R13	2	1	1	0	1	0	3	2	1	10	6	6
ENSMUSG00000064262	GIMAP8	15	15	11	16	20	17	89	82	65	104	60	61
ENSMUSG00000064264	ZFP428	1	2	1	3	2	2	11	10	7	11	7	8
ENSMUSG00000064267	HVCN1	6	6	4	7	6	4	35	37	31	29	34	39
ENSMUSG00000064272	GPBAR1	0	0	1	0	0	0	0	0	0	0	0	0
ENSMUSG00000064280	CCDC146	0	0	0	0	0	0	0	1	0	0	0	0
ENSMUSG00000064281	RPL19-PS1	0	1	2	0	0	0	2	2	2	1	1	1
ENSMUSG00000064284	CDPF1	0	1	0	2	0	1	3	2	6	2	1	4
ENSMUSG00000064288	HIST1H4K	0	0	0	0	0	0	0	0	0	1	0	0
ENSMUSG00000064289	TANK	22	16	17	18	17	17	120	135	81	148	97	84
ENSMUSG00000064299	4921528I07R	0	0	0	0	0	0	0	0	1	0	0	0
ENSMUSG00000064302	CLASP1	10	13	5	10	5	7	32	32	15	37	26	29
ENSMUSG00000064307	LRRC51	0	1	0	0	0	0	4	5	3	3	0	0
ENSMUSG00000064308	2410137M14	0	0	0	0	0	0	0	0	0	1	0	0
ENSMUSG00000064317	GM10146	0	0	0	0	0	0	0	0	0	0	0	2
ENSMUSG00000064326	SIVA1	7	6	13	12	9	14	52	80	64	62	60	58
ENSMUSG00000064329	SCN1A	0	2	3	5	2	1	5	11	4	7	0	3
ENSMUSG00000064330	PDE6H	0	2	1	0	3	0	13	14	12	12	6	7
ENSMUSG00000064336	MT-TF	0	0	0	0	0	0	1	0	0	1	0	0
ENSMUSG00000064337	MT-RNR1	336	876	483	698	632	758	4531	3686	2216	7667	2285	4194
ENSMUSG00000064338	MT-TV	0	0	0	0	0	0	0	0	2	0	0	0
ENSMUSG00000064339	MT-RNR2	1574	5444	2305	3375	2728	11292	26170	13480	6897	41780	10274	8307
ENSMUSG00000064340	MT-TL1	0	1	0	0	1	0	10	4	2	6	0	2
ENSMUSG00000064341	MT-ND1	625	749	715	856	903	675	4591	5631	3787	5404	4056	4488
ENSMUSG00000064342	MT-TI	1	0	0	1	0	0	0	1	1	4	1	1
ENSMUSG00000064343	MT-TQ	0	0	1	3	2	1	4	6	5	15	9	5
ENSMUSG00000064344	MT-TM	0	0	0	2	1	2	5	4	7	11	6	4
ENSMUSG00000064345	MT-ND2	128	156	144	208	135	166	930	934	693	1040	613	927
ENSMUSG00000064346	MT-TW	0	0	0	0	0	0	0	1	2	2	0	0	1
ENSMUSG00000064347	MT-TA	0	0	0	0	1	0	0	1	1	1	2	0
ENSMUSG00000064348	MT-TN	0	0	0	0	0	0	0	1	1	0	0	1
ENSMUSG00000064349	MT-TC	0	0	0	0	0	0	2	1	0	1	0	1
ENSMUSG00000064350	MT-TY	0	0	0	0	0	0	1	3	1	0	0	0
ENSMUSG00000064351	MT-CO1	29	39	39	48	36	35	271	297	208	441	206	241
ENSMUSG00000064352	MT-TS1	0	0	0	0	0	0	1	2	0	2	0	0
ENSMUSG00000064354	MT-CO2	0	0	0	0	0	0	0	1	0	2	1	0
ENSMUSG00000064357	MT-ATP6	0	0	0	0	0	0	3	0	1	2	2	1
ENSMUSG00000064358	MT-CO3	0	0	0	0	1	0	0	0	0	0	0	0
ENSMUSG00000064363	MT-ND4	249	335	298	367	310	283	1333	1721	1214	1556	1067	1408
ENSMUSG00000064367	MT-ND5	20	16	25	20	14	14	191	174	152	245	126	140
ENSMUSG00000064368	MT-ND6	11	13	20	16	9	18	157	160	90	186	108	81
ENSMUSG00000064369	MT-TE	0	0	1	1	2	0	8	10	7	6	7	4
ENSMUSG00000064370	MT-CYTB	492	637	655	787	742	624	3078	3451	2939	3373	2310	3230
ENSMUSG00000064371	MT-TT	0	2	3	1	0	1	6	2	6	9	3	6
ENSMUSG00000064372	MT-TP	2	1	1	0	0	3	15	8	15	7	12	9
ENSMUSG00000064373	SELENOP	3	2	3	4	4	4	31	25	25	32	28	27
ENSMUSG00000064380	GM26448	0	0	0	0	0	0	0	0	0	1	0	0
ENSMUSG00000064398	GM23628	0	0	1	0	0	0	1	0	0	0	1	2
ENSMUSG00000064408	GM23924	0	0	0	1	0	0	0	0	0	0	0	0
ENSMUSG00000064410	GM22247	0	0	0	0	0	0	0	0	0	0	0	1
ENSMUSG00000064422	GM22502	0	1	0	1	0	0	0	0	1	1	1	0

ENSMUSG00000064427	GM22748	0	0	0	0	0	0	0	0	0	0	0	1
ENSMUSG00000064435	GM25588	2	0	0	1	0	1	0	1	0	1	1	0
ENSMUSG00000064437	SNORD49B	0	0	0	0	0	0	1	0	0	1	0	0
ENSMUSG00000064442	GM26225	0	0	0	0	0	0	0	0	0	1	0	1
ENSMUSG00000064451	SNORA23	1	0	0	0	0	0	0	0	0	0	0	0
ENSMUSG00000064460	GM25080	0	1	0	0	0	0	0	0	0	0	0	0
ENSMUSG00000064492	GM22060	1	0	0	0	0	0	0	1	1	0	2	0
ENSMUSG00000064494	GM22063	2	10	2	0	0	4	10	5	2	8	2	5
ENSMUSG00000064515	N-R5S162	0	0	0	0	0	0	0	0	0	0	1	0
ENSMUSG00000064540	SNORD42A	0	0	0	0	0	0	2	0	0	0	0	0
ENSMUSG00000064542	GM22980	0	0	0	0	0	0	1	0	0	1	2	0
ENSMUSG00000064554	N-R5S173	0	0	0	0	0	0	0	0	0	0	1	0
ENSMUSG00000064565	GM24145	1	0	1	0	0	1	0	0	1	0	0	0
ENSMUSG00000064600	GM25636	1	2	1	0	2	1	1	2	0	1	1	2
ENSMUSG00000064602	SNORA41	0	0	0	0	0	0	1	0	0	0	0	1
ENSMUSG00000064612	GM26457	0	0	0	0	0	0	0	0	1	0	0	0
ENSMUSG00000064637	SNORA20	0	0	0	0	0	0	0	0	1	0	0	0
ENSMUSG00000064647	GM23301	0	0	0	0	0	0	0	1	0	0	0	0
ENSMUSG00000064673	GM22805	0	0	0	0	0	0	0	0	0	0	1	0
ENSMUSG00000064719	GM25930	1	0	1	0	1	0	0	2	4	3	1	6
ENSMUSG00000064738	GM23054	0	0	0	1	1	0	3	2	0	0	0	1
ENSMUSG00000064746	GM24707	3	4	3	9	3	5	8	8	7	8	10	8
ENSMUSG00000064765	GM26456	0	0	0	0	0	0	0	0	0	1	0	0
ENSMUSG00000064770	N-R5S29	0	0	0	0	0	0	0	0	1	0	0	0
ENSMUSG00000064811	GM22358	0	0	0	0	0	0	0	0	2	2	0	1
ENSMUSG00000064834	GM25696	0	0	0	0	0	0	1	0	0	0	0	0
ENSMUSG00000064844	GM26202	1	0	0	0	0	0	1	0	0	1	1	0
ENSMUSG00000064857	N-R5S25	0	0	0	2	0	0	1	1	1	4	0	0
ENSMUSG00000064867	GM25039	0	0	0	0	0	0	0	0	0	1	0	0
ENSMUSG00000064871	SNORD58B	0	0	0	0	0	0	1	0	2	0	0	0
ENSMUSG00000064884	GM24204	0	0	0	0	0	0	0	0	1	0	1	0
ENSMUSG00000064897	GM22504	0	0	0	0	1	0	2	0	0	1	0	1
ENSMUSG00000064901	SNORA21	0	0	0	0	0	0	0	0	1	2	0	0
ENSMUSG00000064907	GM25405	0	0	0	0	0	0	0	0	0	0	1	0
ENSMUSG00000064917	GM22574	1	1	0	1	1	0	6	9	2	7	2	5
ENSMUSG00000064925	SNORA62	0	0	0	0	0	0	0	0	0	0	0	1
ENSMUSG00000064927	GM22043	0	2	0	0	0	0	1	0	1	0	0	0
ENSMUSG00000064936	GM23722	0	1	0	0	0	0	2	1	2	1	0	0
ENSMUSG00000064941	GM23238	0	0	0	0	0	0	0	0	0	2	0	0
ENSMUSG00000064945	RNY3	2	2	1	0	2	2	3	6	2	4	5	3
ENSMUSG00000064964	GM25147	0	0	1	0	0	0	0	0	1	0	0	0
ENSMUSG00000064967	GM24405	0	0	0	0	0	0	0	0	0	0	0	1
ENSMUSG00000064994	GM22422	0	0	0	0	0	0	1	0	0	0	0	0
ENSMUSG00000065004	GM26326	0	0	0	0	0	0	0	0	0	0	0	1
ENSMUSG00000065034	GM22363	1	0	0	0	0	0	0	0	0	0	0	0
ENSMUSG00000065037	RN7SK	0	2	1	1	0	2	4	4	3	4	3	5
ENSMUSG00000065047	GM24045	0	0	0	0	0	0	0	1	0	0	1	0
ENSMUSG00000065055	N-R5S213	0	0	0	1	0	0	0	0	0	0	0	0
ENSMUSG00000065061	GM22884	0	0	0	0	0	0	0	0	1	0	0	0
ENSMUSG00000065074	GM24535	0	0	0	0	0	0	1	0	0	0	0	0
ENSMUSG00000065080	GM26497	1	0	0	0	0	0	0	1	0	1	0	0
ENSMUSG00000065100	GM26132	0	1	0	1	0	0	0	0	0	0	0	1
ENSMUSG00000065126	SNORD104	1	1	0	0	0	0	6	12	5	8	6	13
ENSMUSG00000065145	VAULTRC5	1	0	0	0	0	1	1	3	1	2	3	4
ENSMUSG00000065147	SNORA31	0	0	0	1	0	0	2	1	1	2	0	1
ENSMUSG00000065162	GM23645	0	0	0	0	0	0	0	0	2	0	3	3
ENSMUSG00000065191	GM23458	0	0	0	0	0	0	1	0	1	0	0	0
ENSMUSG00000065196	SNORD85	0	1	0	0	0	0	0	0	0	0	0	0

ENSMUSG00000065203	GM24614	0	1	0	0	0	0	0	0	0	0	1	0
ENSMUSG00000065208	GM24616	0	0	0	0	0	0	0	0	0	1	0	1
ENSMUSG00000065219	SNORD32A	0	0	0	2	0	0	0	1	1	0	0	1
ENSMUSG00000065227	GM25790	0	0	0	0	0	0	0	0	1	0	0	0
ENSMUSG00000065232	GM22973	0	0	0	0	0	1	0	0	0	0	0	1
ENSMUSG00000065236	N-R5S197	0	0	0	0	0	0	1	0	0	0	0	0
ENSMUSG00000065239	GM22971	0	0	0	0	0	0	0	0	0	0	1	0
ENSMUSG00000065298	GM26121	0	0	0	0	0	0	0	0	0	0	0	1
ENSMUSG00000065299	GM23138	0	0	0	0	0	1	0	0	0	0	1	0
ENSMUSG00000065301	GM23248	0	0	0	0	0	0	2	0	0	0	0	1
ENSMUSG00000065304	GM23245	0	0	0	0	0	0	0	1	0	0	0	0
ENSMUSG00000065320	GM22056	0	0	0	0	0	0	1	0	0	0	0	0
ENSMUSG00000065353	SNORA73B	2	2	1	1	2	1	4	4	8	8	4	6
ENSMUSG00000065360	GM24412	0	0	1	0	0	0	1	0	1	0	0	0
ENSMUSG00000065374	GM22738	0	0	0	0	0	0	0	1	0	0	0	0
ENSMUSG00000065380	GM23095	0	0	0	0	0	0	0	1	0	0	0	0
ENSMUSG00000065420	MIR142B	0	0	1	0	0	0	0	0	1	2	0	4
ENSMUSG00000065492	MIR34B	0	0	1	0	0	0	0	0	0	0	0	0
ENSMUSG00000065503	MIR351	0	0	0	0	0	0	0	2	0	0	0	1
ENSMUSG00000065578	MIR181B-2	0	0	0	0	0	0	1	0	0	0	0	0
ENSMUSG00000065591	GM22753	0	0	0	0	0	1	0	0	0	0	0	0
ENSMUSG00000065606	MIR16-2	0	0	0	0	0	0	1	0	0	0	0	0
ENSMUSG00000065623	GM24762	0	0	0	0	0	0	1	0	0	0	0	0
ENSMUSG00000065642	SNORA69	0	0	0	0	0	0	0	0	0	1	0	0
ENSMUSG00000065647	GM25896	0	0	0	0	0	0	0	0	1	0	0	0
ENSMUSG00000065657	GM25337	0	0	0	0	0	1	1	0	0	0	0	1
ENSMUSG00000065658	GM23102	0	0	0	0	0	0	1	0	0	0	0	0
ENSMUSG00000065669	GM22581	0	0	0	0	0	0	0	0	1	2	0	0
ENSMUSG00000065701	RNY1	0	0	1	0	0	1	3	0	1	1	0	0
ENSMUSG00000065715	SNORD7	0	0	0	0	0	2	0	0	0	3	0	0
ENSMUSG00000065725	GM26165	0	0	0	0	0	0	0	0	1	0	0	0
ENSMUSG00000065728	GM26175	0	1	0	0	0	0	1	1	0	1	0	0
ENSMUSG00000065729	GM26176	1	0	0	0	0	0	0	0	0	0	0	0
ENSMUSG00000065734	SNORD49A	0	0	0	0	0	0	1	0	0	0	0	0
ENSMUSG00000065750	GM23346	1	0	2	0	1	3	1	1	0	3	0	1
ENSMUSG00000065767	GM23849	0	1	1	0	1	0	1	1	1	3	2	0
ENSMUSG00000065778	GM22154	0	0	0	0	0	0	0	0	0	0	1	0
ENSMUSG00000065799	GM25517	0	0	0	0	0	0	0	0	1	1	0	0
ENSMUSG00000065808	GM23037	0	0	0	0	0	0	0	0	0	0	0	1
ENSMUSG00000065812	GM24696	0	0	0	0	1	0	0	0	0	1	0	0
ENSMUSG00000065818	SNORD35A	1	0	0	0	0	0	2	0	1	1	0	1
ENSMUSG00000065836	GM23502	0	0	0	0	1	0	0	1	1	1	1	0
ENSMUSG00000065874	GM25683	0	0	0	1	0	0	0	0	0	0	0	0
ENSMUSG00000065878	SNORD34	0	0	0	0	0	0	0	0	1	0	1	2
ENSMUSG00000065884	GM22864	0	0	0	1	0	0	0	0	0	0	0	0
ENSMUSG00000065915	GM24445	0	0	0	0	0	1	1	0	0	0	0	0
ENSMUSG00000065922	N-R5-8S1	0	0	0	0	1	0	0	0	3	0	2	2
ENSMUSG00000065934	GM25630	0	0	0	0	0	0	0	0	0	0	0	1
ENSMUSG00000065944	RNU2-10	0	0	0	0	0	1	0	0	0	0	0	0
ENSMUSG00000065952	C330021F23F	0	0	0	1	0	0	0	0	0	1	0	0
ENSMUSG00000065954	TACC1	121	166	113	178	158	167	316	318	248	332	221	322
ENSMUSG00000065979	CPPED1	1	1	0	2	3	2	16	18	16	16	11	16
ENSMUSG00000065990	AURKAIP1	8	11	7	18	16	7	75	102	68	85	65	79
ENSMUSG00000066026	DHRS3	0	1	0	0	0	0	1	1	1	3	0	1
ENSMUSG00000066036	UBR4	9	17	15	7	18	17	62	65	52	72	44	57
ENSMUSG00000066037	HNRNPR	18	19	26	25	18	17	84	97	76	102	72	82
ENSMUSG00000066042	MED18	12	18	12	17	15	21	74	69	65	61	45	65
ENSMUSG00000066043	PHACTR4	0	1	1	1	1	1	14	8	9	10	14	11

ENSMUSG00000066057	GM1976	0	1	0	1	0	1	2	2	2	5	2	2
ENSMUSG00000066068	GM13611	2	1	1	3	2	3	5	4	2	6	2	7
ENSMUSG00000066090	INSL5	0	0	0	0	0	0	1	0	0	1	0	0
ENSMUSG00000066116	GM10154	0	1	0	1	0	0	0	3	0	2	0	1
ENSMUSG00000066148	PRPF4	8	10	13	8	13	11	38	45	43	46	35	49
ENSMUSG00000066149	CDC26	1	2	2	4	3	7	22	16	19	23	28	24
ENSMUSG00000066150	SLC31A1	0	2	3	5	1	3	16	21	13	27	9	21
ENSMUSG00000066151	FKBP15	3	5	5	4	4	5	12	23	22	22	13	15
ENSMUSG00000066158	AY512931	0	0	0	0	0	0	0	0	0	1	0	0
ENSMUSG00000066170	E230001N04I	0	0	0	0	0	2	1	3	0	3	1	0
ENSMUSG00000066175	2510046G10I	0	0	0	0	0	0	1	1	1	2	2	3
ENSMUSG00000066180	GM10155	0	0	0	1	1	0	3	3	2	2	0	0
ENSMUSG00000066224	ARID3C	0	0	0	0	0	0	0	0	0	1	0	0
ENSMUSG00000066232	IPO7	25	33	38	42	35	31	202	186	151	213	142	189
ENSMUSG00000066233	TMEM42	1	2	0	0	2	2	6	3	3	12	6	6
ENSMUSG00000066258	TRIM12A	6	8	3	6	5	7	17	17	15	21	20	18
ENSMUSG00000066278	VPS37B	13	12	18	9	12	15	40	33	34	74	44	36
ENSMUSG00000066306	NUMA1	13	16	17	18	14	10	47	42	37	57	27	50
ENSMUSG00000066315	GM12918	1	0	0	0	0	1	3	0	0	1	0	0
ENSMUSG00000066324	IMPAD1	21	25	28	26	24	24	96	98	84	117	63	85
ENSMUSG00000066357	WDR6	5	4	3	2	6	3	31	36	21	44	26	32
ENSMUSG00000066362	RPS13-PS1	2	3	1	4	2	2	12	17	13	19	12	18
ENSMUSG00000066372	VMN2R65	0	0	0	0	0	0	0	1	0	0	0	0
ENSMUSG00000066378	GM10160	0	0	0	0	0	0	0	0	0	0	1	0
ENSMUSG00000066389	RPL31-PS1	0	0	1	1	0	2	3	1	1	1	3	2
ENSMUSG00000066392	NRXN3	0	1	0	2	1	1	2	2	3	8	2	2
ENSMUSG00000066406	AKAP13	20	27	35	28	27	32	81	95	60	97	70	101
ENSMUSG00000066407	GM10263	0	0	1	0	3	0	2	1	0	1	0	1
ENSMUSG00000066415	MSL2	8	15	12	9	17	14	63	56	41	69	46	46
ENSMUSG00000066440	ZFYVE26	5	1	1	3	4	4	11	22	8	12	5	14
ENSMUSG00000066441	RDH11	4	7	6	8	7	9	42	44	29	46	34	31
ENSMUSG00000066442	MTHFS	1	0	1	1	1	0	2	0	1	1	1	0
ENSMUSG00000066443	GM10163	1	1	4	1	2	2	1	5	4	2	2	4
ENSMUSG00000066475	GM10268	0	0	0	0	0	0	1	2	0	0	1	0
ENSMUSG00000066478	GM5745	2	1	1	5	1	0	2	1	2	3	5	7
ENSMUSG00000066487	GM5786	0	0	1	0	0	0	0	0	1	0	1	0
ENSMUSG00000066489	RPL26-PS2	0	0	0	0	0	1	0	0	0	0	0	0
ENSMUSG00000066491	GM6265	0	0	0	0	0	0	1	0	0	5	0	1
ENSMUSG00000066537	VMN2R57	0	0	0	0	0	0	0	1	0	0	0	0
ENSMUSG00000066538	GM6254	0	0	0	0	0	0	1	0	0	0	0	0
ENSMUSG00000066543	RPL17-PS9	0	0	1	0	0	0	1	0	1	3	1	1
ENSMUSG00000066551	HMGB1	30	35	26	51	45	21	223	210	152	234	167	193
ENSMUSG00000066554	GM10167	0	0	0	0	0	0	1	0	0	0	1	0
ENSMUSG00000066568	LSM14A	9	16	12	19	17	7	83	96	73	104	70	60
ENSMUSG00000066571	4931406P16F	4	3	2	3	5	1	18	24	15	18	8	18
ENSMUSG00000066592	GM7589	0	0	0	0	1	1	0	1	2	1	0	3
ENSMUSG00000066595	FLVCR1	4	9	5	10	5	6	30	19	19	38	24	24
ENSMUSG00000066613	ZFP932	3	0	2	3	4	7	28	29	16	46	27	19
ENSMUSG00000066620	GM4883	1	0	0	0	0	0	0	0	0	0	0	0
ENSMUSG00000066621	TECPR1	7	11	6	6	11	6	37	46	33	46	36	38
ENSMUSG00000066626	GM5121	2	0	1	1	0	5	12	8	4	4	6	2
ENSMUSG00000066629	RPL36-PS3	0	1	0	0	0	0	3	1	2	1	0	0
ENSMUSG00000066637	TTC32	2	5	4	2	6	5	24	27	18	26	19	12
ENSMUSG00000066640	FBXL18	2	0	1	3	3	0	6	11	4	6	5	9
ENSMUSG00000066643	WDR35	0	1	0	0	1	1	9	7	6	4	4	8
ENSMUSG00000066647	GM5113	0	1	0	1	1	0	1	0	0	2	0	2
ENSMUSG00000066652	LEFTY2	0	0	0	0	0	0	0	0	0	0	1	0
ENSMUSG00000066687	ZBTB16	0	1	0	0	0	0	0	1	0	1	0	0

ENSMUSG00000066714	GM8062	0	0	0	0	0	0	0	1	0	1	0	0
ENSMUSG00000066724	GM10175	5	5	4	8	9	7	17	23	13	7	9	16
ENSMUSG00000066735	VKORC1L1	5	17	19	11	13	15	53	49	46	48	37	42
ENSMUSG00000066771	GM5614	1	2	0	0	0	1	4	0	0	2	2	1
ENSMUSG00000066798	ZBTB6	6	10	11	12	9	12	51	46	25	65	37	41
ENSMUSG00000066800	RNASEL	4	3	1	3	4	5	14	9	12	17	12	16
ENSMUSG00000066809	GM10180	0	0	0	2	0	0	1	1	0	1	0	0
ENSMUSG00000066829	ZFP810	1	2	1	1	0	2	2	11	7	4	1	3
ENSMUSG00000066838	ZFP772	0	1	0	0	2	0	3	4	3	3	3	1
ENSMUSG00000066839	ECSIT	8	10	10	16	9	8	53	56	36	55	35	55
ENSMUSG00000066850	VMN1R65	0	0	0	0	0	0	0	0	0	0	1	0
ENSMUSG00000066877	NCK2	11	10	7	16	13	17	72	59	48	68	49	46
ENSMUSG00000066878	GM10184	1	3	0	3	0	0	3	1	4	3	1	3
ENSMUSG00000066880	ZFP617	2	2	0	1	2	3	14	10	14	8	6	4
ENSMUSG00000066892	FBXL12	8	4	3	6	3	3	25	30	25	26	20	15
ENSMUSG00000066900	SUDS3	6	4	7	10	11	5	32	56	28	50	44	31
ENSMUSG00000066902	RPS23-PS2	0	0	0	1	1	1	6	2	0	1	1	2
ENSMUSG00000066952	MYO1H	3	3	1	5	3	1	16	17	11	11	15	8
ENSMUSG00000066975	CRYBA4	0	0	0	0	0	0	0	1	0	0	0	0
ENSMUSG00000066979	BUB3	10	14	9	18	20	14	80	81	62	81	61	73
ENSMUSG00000067006	SERPINB5	0	0	0	0	0	0	0	1	1	0	1	0
ENSMUSG00000067017	GM3608	0	1	0	0	0	1	0	0	0	0	0	0
ENSMUSG00000067038	RPS12-PS3	0	0	0	0	0	0	0	1	0	0	2	1
ENSMUSG00000067058	RPS15A-PS5	0	0	1	1	0	0	1	2	3	1	1	2
ENSMUSG00000067071	HES6	1	1	1	3	2	0	10	16	5	16	9	11
ENSMUSG00000067121	GM7027	0	0	0	0	0	0	2	0	0	1	1	2
ENSMUSG00000067147	RPL7A-PS11	0	0	0	0	0	0	0	1	0	0	0	0
ENSMUSG00000067148	POLR1C	3	12	9	9	10	7	45	55	31	55	45	48
ENSMUSG00000067150	XPO5	6	5	10	13	15	10	55	59	45	58	48	50
ENSMUSG00000067158	COL4A4	0	0	0	0	0	0	1	1	0	2	0	0
ENSMUSG00000067194	EIF1AX	23	20	21	19	28	25	142	149	119	162	117	106
ENSMUSG00000067199	FRAT1	0	6	9	8	2	1	20	17	11	16	16	11
ENSMUSG00000067203	H2-K2	3	0	2	3	0	1	11	9	5	8	3	5
ENSMUSG00000067212	H2-T23	5	6	1	4	6	3	22	22	16	25	18	33
ENSMUSG00000067215	USP51	1	0	0	3	2	1	4	10	7	11	5	8
ENSMUSG00000067219	NIPAL1	1	1	2	1	0	0	0	1	1	1	0	0
ENSMUSG00000067220	CNGA1	4	8	3	3	9	4	10	3	7	10	8	10
ENSMUSG00000067224	GM3695	0	1	0	0	0	0	3	1	0	0	1	0
ENSMUSG00000067231	CYP2C65	0	0	0	0	0	0	0	0	0	1	0	0
ENSMUSG00000067242	LGI1	0	0	0	0	0	1	0	0	0	0	0	0
ENSMUSG00000067274	RPLP0	75	98	110	90	120	97	575	583	404	655	435	472
ENSMUSG00000067285	GM16223	0	1	0	0	1	0	2	1	1	0	0	0
ENSMUSG00000067288	RPS28	152	170	199	233	224	225	720	810	592	742	628	683
ENSMUSG00000067336	BMPR2	8	10	13	13	14	15	71	57	34	61	43	55
ENSMUSG00000067341	H2-EB2	0	0	0	0	0	0	1	1	0	0	0	0
ENSMUSG00000067344	RPS25-PS1	1	1	0	1	2	1	5	9	7	7	9	5
ENSMUSG00000067351	RPS15A-PS2	1	0	0	0	0	0	0	0	1	0	0	0
ENSMUSG00000067352	GM14149	1	1	2	1	1	2	1	1	0	1	3	3
ENSMUSG00000067365	TMEM128	7	6	8	5	7	4	25	17	15	22	11	16
ENSMUSG00000067367	LYAR	43	42	35	42	42	35	283	277	208	292	208	229
ENSMUSG00000067369	TRMT2B	0	7	3	5	4	2	14	12	10	19	11	17
ENSMUSG00000067370	B3GALT4	0	0	0	0	0	0	0	1	1	0	1	0
ENSMUSG00000067399	TRIM43C	0	0	0	0	0	0	0	0	0	1	0	0
ENSMUSG00000067424	ZFP563	1	3	5	1	2	1	3	8	1	11	4	2
ENSMUSG00000067430	ZFP763	1	3	4	1	0	2	9	9	9	11	11	12
ENSMUSG00000067455	HIST1H4J	0	0	0	0	0	0	0	0	0	0	1	1
ENSMUSG00000067529	OLFR1423	0	0	0	0	0	0	0	0	0	0	0	1
ENSMUSG00000067545	OLFR1419	0	0	0	0	0	0	0	2	0	0	0	0

ENSMUSG00000067567	HDAC8	0	1	2	1	1	0	10	10	7	5	5	3
ENSMUSG00000067591	KLRA3	0	0	0	0	0	0	2	0	0	0	0	1
ENSMUSG00000067597	DGAT2L6	0	0	0	0	0	0	1	0	1	0	0	1
ENSMUSG00000067599	KLRA7	0	0	0	0	0	1	4	1	2	0	1	5
ENSMUSG00000067608	PCNA-PS2	0	0	0	0	1	0	0	1	2	0	1	2
ENSMUSG00000067629	SYNGAP1	5	6	5	4	3	4	8	10	8	12	5	7
ENSMUSG00000067649	MAGEB18	0	0	0	0	0	0	0	0	0	1	0	0
ENSMUSG00000067653	ANKRD23	0	0	3	0	0	1	6	6	2	7	8	3
ENSMUSG00000067713	PRKAG1	4	3	8	2	5	1	18	41	12	17	19	14
ENSMUSG00000067714	LPAR5	0	0	0	0	0	0	0	0	0	1	0	0
ENSMUSG00000067722	BC003965	6	10	10	6	14	14	60	75	62	69	62	56
ENSMUSG00000067745	GM14243	0	0	0	0	0	1	0	0	0	0	0	0
ENSMUSG00000067768	XLR4B	0	7	4	5	6	6	38	34	24	35	19	24
ENSMUSG00000067786	NNAT	0	0	0	0	0	1	0	0	0	0	0	1
ENSMUSG00000067787	BLCAP	0	1	0	2	2	2	13	10	4	14	6	5
ENSMUSG00000067795	4930444P10F	0	0	0	0	0	0	1	0	0	0	0	0
ENSMUSG00000067825	PEX26	2	4	4	2	2	0	11	11	8	16	5	8
ENSMUSG00000067847	ROMO1	26	32	37	39	40	33	235	260	210	265	208	232
ENSMUSG00000067851	ARFGEF1	13	13	10	13	18	12	79	97	53	91	47	65
ENSMUSG00000067860	ZIC3	1	1	2	1	1	2	9	4	5	10	4	11
ENSMUSG00000067869	TCEA1-PS1	0	0	0	0	0	0	0	1	1	1	1	1
ENSMUSG00000067870	RPL31-PS8	41	63	36	42	44	59	116	161	181	164	114	125
ENSMUSG00000067873	HTATSF1	8	8	8	21	17	12	92	99	38	93	55	80
ENSMUSG00000067878	MAP7D3	1	4	1	4	3	2	11	11	6	10	9	9
ENSMUSG00000067924	RTL8B	0	0	2	0	0	0	5	6	3	5	2	2
ENSMUSG00000067925	RTL8A	0	0	0	0	0	0	0	0	0	1	0	0
ENSMUSG00000067929	GM10226	0	0	0	0	0	0	1	0	0	0	0	0
ENSMUSG00000067931	ZFP948	7	8	4	5	7	4	31	56	33	46	27	33
ENSMUSG00000067942	ZFP160	5	2	3	3	11	5	32	33	26	41	19	24
ENSMUSG00000067995	GTF2F2	6	7	3	2	4	2	30	24	14	37	22	27
ENSMUSG00000068009	BPIFB6	0	0	0	0	0	0	0	0	0	0	0	1
ENSMUSG00000068014	GM14648	0	0	0	0	0	0	0	0	0	0	1	0
ENSMUSG00000068015	LRCH1	0	1	0	0	3	0	5	5	4	6	3	12
ENSMUSG00000068036	AFDN	0	0	0	0	1	0	0	0	0	0	0	0
ENSMUSG00000068039	TCP1	43	42	38	51	46	55	300	316	240	322	241	283
ENSMUSG00000068040	TM9SF4	5	12	9	11	10	12	56	54	31	48	39	39
ENSMUSG00000068101	CENPM	3	2	2	0	5	4	9	13	4	18	18	14
ENSMUSG00000068105	TNFRSF13C	0	0	0	0	0	0	1	0	0	0	0	0
ENSMUSG00000068113	GM4907	5	2	3	9	5	2	25	23	18	33	16	23
ENSMUSG00000068114	CCDC134	1	1	5	2	2	5	10	11	5	13	13	5
ENSMUSG00000068115	NINL	1	0	0	0	0	0	0	0	0	0	0	0
ENSMUSG00000068117	MEI1	0	0	0	1	0	0	0	0	0	1	1	2
ENSMUSG00000068129	CST7	4	8	6	1	5	11	18	21	16	30	18	25
ENSMUSG00000068130	ZFP442	1	0	1	0	1	1	3	2	3	10	5	1
ENSMUSG00000068134	ZFP120	9	5	3	10	8	9	33	36	20	37	22	24
ENSMUSG00000068141	GM10232	0	0	0	0	0	0	2	1	0	1	0	1
ENSMUSG00000068154	INSM1	0	0	0	1	0	0	2	0	2	1	0	2
ENSMUSG00000068184	NDUFAF2	13	12	8	18	13	16	89	96	79	83	77	90
ENSMUSG00000068206	PICK1	2	8	4	4	1	2	9	17	10	11	8	7
ENSMUSG00000068220	LGALS1	182	212	235	255	236	222	1357	1444	1080	1429	1032	1254
ENSMUSG00000068227	IL2RB	0	0	0	0	0	0	0	1	0	0	0	0
ENSMUSG00000068240	GM11808	5	12	5	7	7	10	27	31	26	22	24	23
ENSMUSG00000068243	GM7079	0	0	0	0	0	0	0	0	0	0	0	1
ENSMUSG00000068245	PHF11D	0	0	0	0	0	0	0	0	0	1	0	0
ENSMUSG00000068250	AMN1	1	6	1	5	6	1	11	23	13	13	13	15
ENSMUSG00000068259	OLFR461	0	0	0	0	0	0	0	0	0	0	2	0
ENSMUSG00000068262	GM5879	1	5	1	1	2	4	7	8	5	4	7	9
ENSMUSG00000068263	EFCC1	0	0	1	0	0	0	0	0	0	0	0	0

ENSMUSG00000068264	AP5S1	1	1	1	0	1	1	5	2	3	2	3	2
ENSMUSG00000068267	CENPB	8	11	9	13	5	11	72	70	56	59	49	62
ENSMUSG00000068284	USF3	2	2	7	5	2	1	26	20	21	21	12	14
ENSMUSG00000068290	DDRGK1	6	3	3	6	3	5	29	35	17	28	20	20
ENSMUSG00000068299	NAT8F4	1	0	0	0	1	1	4	1	3	2	1	1
ENSMUSG00000068314	GM6899	0	0	0	0	0	0	1	0	0	0	0	0
ENSMUSG00000068323	SLC4A5	0	0	0	0	0	0	2	0	0	0	0	0
ENSMUSG00000068324	GM10029	0	0	0	0	0	0	0	0	0	1	1	1
ENSMUSG00000068328	AUP1	22	36	41	36	44	34	174	183	168	177	152	174
ENSMUSG00000068329	HTRA2	14	14	14	12	10	11	77	74	57	71	61	77
ENSMUSG00000068335	DOK1	3	4	4	5	3	8	17	26	19	24	22	38
ENSMUSG00000068391	CHRAC1	1	5	0	3	0	1	4	10	6	12	8	7
ENSMUSG00000068394	CEP152	3	8	6	2	10	9	20	23	18	21	12	16
ENSMUSG00000068396	RPL34-PS1	4	5	1	7	6	9	11	11	16	27	9	6
ENSMUSG00000068397	GM10240	5	2	11	7	7	6	19	22	21	11	8	10
ENSMUSG00000068417	PNP2	0	1	0	0	0	0	2	2	1	1	0	0
ENSMUSG00000068463	B630019A10I	2	2	5	4	2	1	13	9	7	9	8	7
ENSMUSG00000068479	MFAP1A	2	7	9	2	4	5	39	37	27	35	19	30
ENSMUSG00000068522	AARD	0	0	0	0	0	0	0	0	1	0	0	0
ENSMUSG00000068523	GNG5	4	7	9	6	4	4	46	53	41	40	28	48
ENSMUSG00000068551	ZFP467	0	0	0	0	1	0	0	0	0	0	0	0
ENSMUSG00000068579	RPL7A-PS3	0	0	0	0	0	0	0	0	1	0	2	0
ENSMUSG00000068580	ZFYVE19	4	1	3	5	6	6	24	28	20	26	17	18
ENSMUSG00000068587	MGAM	1	0	0	0	0	0	0	0	0	2	0	0
ENSMUSG00000068601	GM10244	0	0	0	0	0	0	1	2	0	2	1	0
ENSMUSG00000068663	CLEC16A	2	0	1	3	1	2	5	3	2	3	8	4
ENSMUSG00000068674	GM10247	1	3	4	3	1	2	11	10	11	8	2	8
ENSMUSG00000068686	CD59B	0	0	0	0	0	0	0	1	0	0	0	0
ENSMUSG00000068689	RPS19-PS9	0	0	0	0	0	0	0	0	0	0	1	0
ENSMUSG00000068706	GM10250	1	3	4	4	3	3	6	15	10	7	10	9
ENSMUSG00000068732	TMEM167B	10	12	10	20	13	9	50	77	51	57	55	72
ENSMUSG00000068739	SARS	14	33	18	27	22	33	142	165	137	172	118	130
ENSMUSG00000068740	CELSR2	0	1	0	0	0	0	3	0	0	0	0	0
ENSMUSG00000068742	CRY2	0	2	4	2	1	0	8	8	5	5	9	7
ENSMUSG00000068747	SORT1	0	0	0	0	0	0	2	0	0	0	0	0
ENSMUSG00000068748	PTPRZ1	0	0	0	1	0	1	1	2	0	0	1	0
ENSMUSG00000068749	PSMA5	18	19	21	20	16	23	133	158	106	168	103	128
ENSMUSG00000068794	COL28A1	0	0	0	0	0	0	1	1	0	0	0	1
ENSMUSG00000068798	RAP1A	20	15	14	19	21	19	73	108	81	122	67	84
ENSMUSG00000068808	OLFR1189	0	0	0	0	0	1	0	0	0	0	0	0
ENSMUSG00000068815	OLFR1143	0	0	0	0	1	0	0	0	0	0	1	0
ENSMUSG00000068816	OLFR152	0	0	0	0	0	0	1	0	0	0	0	0
ENSMUSG00000068823	CSDE1	72	104	76	90	85	82	523	488	337	540	324	441
ENSMUSG00000068854	HIST2H2BE	7	6	4	3	6	5	33	29	25	29	19	21
ENSMUSG00000068855	HIST2H2AC	2	3	3	0	2	1	1	3	2	1	1	2
ENSMUSG00000068856	SF3B4	5	5	11	6	4	10	40	40	33	36	23	51
ENSMUSG00000068882	SSB	150	222	188	292	197	223	1190	1236	888	1412	850	968
ENSMUSG00000068917	CLK2	6	17	11	22	14	28	54	79	58	89	58	54
ENSMUSG00000068921	DAP3	6	12	10	20	18	13	61	80	49	69	71	64
ENSMUSG00000068922	MSTO1	4	6	4	8	5	5	41	56	38	41	35	33
ENSMUSG00000068923	SYT11	6	1	2	7	1	3	11	11	11	13	6	11
ENSMUSG00000068959	ZFP619	0	0	0	0	0	0	0	0	1	1	0	1
ENSMUSG00000068966	ZBTB34	15	29	33	39	29	30	137	189	146	171	117	153
ENSMUSG00000068969	RPL32-PS	0	0	0	0	0	0	0	1	0	0	0	0
ENSMUSG00000068999	VMN2R5	0	0	0	0	0	0	1	1	0	1	1	0
ENSMUSG00000069020	URM1	7	8	8	11	7	7	46	45	24	37	39	44
ENSMUSG00000069041	SLC25A31	0	0	0	0	1	0	1	0	0	0	0	0
ENSMUSG00000069089	CDK7	8	8	5	8	4	2	42	30	23	30	32	27

ENSMUSG00000069094	PDE7A	37	33	26	32	34	31	165	145	104	125	110	128
ENSMUSG00000069114	ZBTB10	7	8	6	5	16	5	42	39	26	56	29	40
ENSMUSG00000069117	GM10260	1	0	0	1	2	1	1	1	0	0	0	2
ENSMUSG00000069125	RPS24-PS2	0	0	0	0	0	0	0	0	1	0	1	0
ENSMUSG00000069135	FGFR1OP	19	9	14	13	14	9	63	79	48	80	50	48
ENSMUSG00000069171	NR2F1	12	28	22	25	18	19	85	105	80	101	70	101
ENSMUSG00000069184	ZFP72	1	2	0	1	1	0	7	6	3	5	9	5
ENSMUSG00000069188	GM13192	0	0	0	0	0	0	3	0	1	0	0	2
ENSMUSG00000069189	ZDHHC11	0	0	0	0	0	0	0	0	0	0	0	1
ENSMUSG00000069196	GM10161	0	0	0	0	0	0	0	0	0	1	0	2
ENSMUSG00000069206	ZFP874A	0	2	0	2	1	0	3	3	1	2	2	6
ENSMUSG00000069208	ZFP825	1	1	3	1	2	2	7	3	5	19	4	2
ENSMUSG00000069223	4930451E10F	0	0	0	0	0	0	3	0	0	0	1	2
ENSMUSG00000069237	FAM8A1	5	4	5	3	2	2	15	31	20	24	26	24
ENSMUSG00000069265	HIST1H3A	0	0	0	0	0	0	0	1	0	0	1	0
ENSMUSG00000069266	HIST1H4B	0	0	0	0	0	1	0	0	0	0	0	0
ENSMUSG00000069267	HIST1H3B	0	0	0	0	1	0	0	1	0	0	0	0
ENSMUSG00000069268	HIST1H2BF	0	0	1	0	0	1	1	0	0	0	0	0
ENSMUSG00000069270	HIST1H2AC	1	0	1	2	0	0	4	9	3	6	10	6
ENSMUSG00000069272	HIST1H2AE	0	0	3	5	3	1	6	14	4	9	9	8
ENSMUSG00000069273	HIST1H3E	0	0	0	1	0	0	0	0	1	0	0	1
ENSMUSG00000069274	HIST1H4F	0	0	0	0	0	0	0	1	0	0	0	1
ENSMUSG00000069299	VMN1R188	0	1	0	0	0	0	0	0	0	0	0	1
ENSMUSG00000069308	HIST1H2BP	0	0	0	0	0	0	0	2	1	1	2	0
ENSMUSG00000069309	HIST1H2AN	1	0	0	0	2	0	0	2	0	0	0	0
ENSMUSG00000069310	HIST1H3C	0	2	2	0	1	0	1	1	0	1	1	1
ENSMUSG00000069324	GM5096	0	0	0	0	0	0	0	0	1	0	0	0
ENSMUSG00000069376	GM5507	0	0	0	0	0	0	0	0	0	1	0	0
ENSMUSG00000069379	GM4950	0	1	0	1	0	0	0	0	0	2	0	0
ENSMUSG00000069390	OLFR707	0	0	0	0	0	0	0	0	0	2	0	0
ENSMUSG00000069439	GM8444	0	0	0	0	1	0	1	0	0	0	1	0
ENSMUSG00000069475	GM6020	0	0	0	0	0	0	1	1	0	0	0	0
ENSMUSG00000069495	EPC2	5	11	5	11	11	12	60	55	47	54	38	55
ENSMUSG00000069520	TMEM19	1	2	1	3	3	2	9	5	5	7	2	9
ENSMUSG00000069539	SCYL2	5	3	3	4	6	3	18	39	20	21	17	24
ENSMUSG00000069540	GM4925	0	1	0	0	0	0	1	1	1	0	0	0
ENSMUSG00000069565	DAZAP1	11	11	17	27	17	16	100	93	62	77	53	85
ENSMUSG00000069601	ANK3	0	1	0	0	0	0	4	0	0	2	1	1
ENSMUSG00000069631	STRADA	1	2	2	4	6	4	5	9	11	19	6	4
ENSMUSG00000069633	PEX11G	0	2	1	2	0	1	9	8	4	1	2	9
ENSMUSG00000069662	MARCKS	3	4	2	1	4	2	29	16	14	30	19	19
ENSMUSG00000069678	PCGF1	3	3	0	4	5	1	11	15	11	6	13	17
ENSMUSG00000069682	GM10275	20	52	43	52	52	39	149	135	124	132	116	122
ENSMUSG00000069713	4933406P04F	0	0	0	0	0	0	0	1	0	0	0	0
ENSMUSG00000069729	ARID1B	40	88	53	173	57	74	188	159	115	238	130	143
ENSMUSG00000069743	ZFP820	0	1	5	5	2	5	22	19	19	17	10	15
ENSMUSG00000069744	PSMB3	24	38	40	42	41	34	204	223	163	226	159	172
ENSMUSG00000069755	ZFP125	1	2	0	0	2	1	3	0	3	3	2	1
ENSMUSG00000069769	MSI2	13	21	14	22	18	22	99	109	57	97	65	83
ENSMUSG00000069793	SLFN9	8	10	6	9	16	8	46	35	27	42	30	25
ENSMUSG00000069804	GM10277	0	1	0	0	0	0	0	2	0	2	0	0
ENSMUSG00000069808	FAM57A	1	3	0	1	1	1	7	6	6	8	4	5
ENSMUSG00000069833	AHNAK	16	21	17	24	20	16	67	61	44	66	42	56
ENSMUSG00000069844	SCO1	12	9	11	12	10	13	56	52	39	49	39	57
ENSMUSG00000069855	SLC47A2	0	1	0	0	0	0	1	1	0	1	2	0
ENSMUSG00000069862	RPS12-PS9	247	209	231	249	245	289	906	1396	696	825	640	677
ENSMUSG00000069867	PABPN1L	0	0	0	0	0	0	0	1	1	0	0	0
ENSMUSG00000069874	IRGM2	0	0	0	0	0	0	2	1	0	0	2	1

ENSMUSG00000069895	ATXN1L	5	3	5	5	4	5	22	11	12	26	15	11
ENSMUSG00000069899	GM12166	6	3	7	6	5	3	10	13	9	13	9	10
ENSMUSG00000069910	SPDL1	18	8	5	15	16	9	70	82	58	116	47	58
ENSMUSG00000069913	ANP32-PS	0	0	0	0	0	0	0	1	0	1	0	0
ENSMUSG00000069920	B3GNT9	1	1	0	3	0	0	5	1	1	7	5	13
ENSMUSG00000069925	4932416K20R	0	0	0	0	0	0	0	1	0	0	0	0
ENSMUSG00000069972	RPS13-PS2	15	9	12	12	8	14	44	53	40	51	33	51
ENSMUSG00000070000	FCHO1	4	7	6	4	7	5	42	47	38	27	38	37
ENSMUSG00000070002	ELL	4	8	5	9	7	3	21	20	24	36	20	23
ENSMUSG00000070003	SSBP4	1	0	1	1	2	2	4	1	2	4	4	5
ENSMUSG00000070031	SP140	1	3	5	3	3	1	12	8	14	10	16	4
ENSMUSG00000070034	SP110	8	8	8	8	1	6	40	32	28	49	28	35
ENSMUSG00000070047	FAT1	0	0	0	0	0	0	0	0	1	1	0	0
ENSMUSG00000070056	MFHAS1	29	35	32	21	29	10	96	82	59	85	67	90
ENSMUSG00000070074	MIR484	0	0	0	0	0	0	1	0	0	0	0	0
ENSMUSG00000070166	GM25821	0	0	0	0	0	0	0	0	0	0	0	1
ENSMUSG00000070167	SNORA57	1	0	1	2	2	4	18	19	10	7	5	12
ENSMUSG00000070214	GM23058	1	0	0	0	0	0	1	2	0	0	0	0
ENSMUSG00000070225	N-R5S33	0	0	0	0	0	1	0	0	0	0	0	0
ENSMUSG00000070271	GM13268	0	0	0	0	1	0	0	0	0	0	0	0
ENSMUSG00000070282	3000002C10R	2	0	0	0	0	0	2	2	3	4	5	3
ENSMUSG00000070283	NDUFAF3	3	0	4	2	3	2	11	18	10	21	19	16
ENSMUSG00000070284	GMPPB	0	0	2	1	2	2	9	14	6	5	6	7
ENSMUSG00000070287	SLC35G2	0	0	0	0	0	0	0	1	0	0	0	0
ENSMUSG00000070305	MPZL3	1	0	1	1	2	1	1	1	2	3	4	3
ENSMUSG00000070315	4930581F22R	0	0	1	1	1	0	1	6	1	3	1	2
ENSMUSG00000070319	EIF3G	18	22	29	22	32	29	144	155	119	158	115	156
ENSMUSG00000070327	RNF213	9	24	23	22	13	11	70	94	68	72	62	72
ENSMUSG00000070343	GM10288	28	22	31	32	29	33	105	77	101	82	104	89
ENSMUSG00000070345	HSF5	0	0	0	1	0	0	1	1	0	0	0	0
ENSMUSG00000070348	CCND1	0	2	1	0	1	2	2	2	1	8	1	2
ENSMUSG00000070354	EVI2	0	0	0	0	0	0	2	0	0	0	1	0
ENSMUSG00000070368	PROK1	0	0	1	0	0	0	1	0	0	0	1	0
ENSMUSG00000070369	ITGAD	0	0	0	0	0	0	0	0	0	1	0	0
ENSMUSG00000070371	PRSS36	0	1	1	1	0	0	2	0	2	4	0	0
ENSMUSG00000070372	CAPZA1	6	7	9	15	7	8	29	35	29	28	33	34
ENSMUSG00000070385	AMPD1	0	0	0	0	0	0	1	2	3	0	3	4
ENSMUSG00000070394	TMEM256	9	8	10	5	5	6	5	2	0	1	3	2
ENSMUSG00000070407	HS3ST3B1	12	14	14	8	23	17	65	62	36	47	33	32
ENSMUSG00000070420	ZSCAN25	0	0	0	0	0	0	1	0	1	0	0	1
ENSMUSG00000070421	OLFR658	0	0	0	0	0	0	0	0	0	0	0	1
ENSMUSG00000070425	XNTRPC	0	1	0	0	0	0	0	0	1	2	1	0
ENSMUSG00000070426	RNF121	0	2	2	3	3	2	9	8	13	10	3	6
ENSMUSG00000070427	IL18BP	1	0	0	1	2	0	2	4	4	3	4	9
ENSMUSG00000070461	9230112E08R	0	0	0	0	0	0	2	4	1	0	0	1
ENSMUSG00000070462	TLNRD1	2	2	1	1	2	1	18	19	6	4	9	7
ENSMUSG00000070471	ERICH6	0	0	0	0	0	0	1	1	0	0	0	0
ENSMUSG00000070490	GM10293	0	0	0	0	0	0	0	0	0	1	0	0
ENSMUSG00000070493	CHCHD2	36	67	70	82	80	61	487	537	367	463	377	437
ENSMUSG00000070498	TMEM132B	0	0	0	0	0	0	0	1	0	0	0	0
ENSMUSG00000070520	NSMCE3	1	4	3	13	9	7	46	30	18	35	31	26
ENSMUSG00000070544	TOP1	32	54	39	104	52	43	211	212	153	239	140	172
ENSMUSG00000070565	RASAL2	1	0	0	0	0	0	0	0	0	0	0	1
ENSMUSG00000070570	SLC17A7	1	1	0	0	0	0	1	0	0	0	0	0
ENSMUSG00000070572	TRMT112-PS2	0	0	0	0	0	0	0	1	0	0	0	1
ENSMUSG00000070583	FV1	0	0	0	1	0	0	0	0	2	0	0	0
ENSMUSG00000070601	VMN2R84	0	1	0	2	0	1	3	2	1	1	0	1
ENSMUSG00000070637	SRARP	0	0	0	0	0	0	0	0	0	0	1	0

ENSMUSG00000070639	LRRC8B	1	4	1	5	3	4	20	20	21	21	16	15
ENSMUSG00000070667	RPL31-PS10	0	0	1	1	1	0	2	0	0	0	0	2
ENSMUSG00000070687	HTR1D	0	0	0	0	0	0	0	0	1	0	0	0
ENSMUSG00000070691	RUNX3	23	33	19	69	31	44	148	128	93	136	80	114
ENSMUSG00000070692	RPS12-PS10	1	4	9	11	2	7	30	23	16	22	19	24
ENSMUSG00000070697	UTP3	30	50	38	42	34	44	193	245	193	252	224	197
ENSMUSG00000070699	SARS2	2	1	1	0	1	6	8	12	9	10	3	9
ENSMUSG00000070705	EID2B	2	0	1	2	2	1	6	9	8	5	3	6
ENSMUSG00000070709	ZFP974	0	1	0	0	0	0	1	0	3	0	0	0
ENSMUSG00000070713	GM10282	5	4	7	3	5	6	33	17	14	23	15	19
ENSMUSG00000070730	RMDN3	5	7	8	8	4	3	42	31	29	37	27	35
ENSMUSG00000070733	FRYL	5	6	14	8	7	11	47	49	34	39	18	35
ENSMUSG00000070737	TMEM35B	0	0	1	1	2	0	1	7	3	4	4	9
ENSMUSG00000070738	DGKD	20	22	13	13	19	16	75	125	82	92	72	85
ENSMUSG00000070780	RBM47	0	0	0	0	0	1	1	2	2	2	0	0
ENSMUSG00000070806	ZMYND12	0	0	0	0	0	0	1	0	1	1	0	0
ENSMUSG00000070808	BICRA	1	3	4	0	6	4	14	19	12	24	10	13
ENSMUSG00000070814	ZSWIM9	1	1	0	1	0	1	8	4	3	6	2	0
ENSMUSG00000070822	ZSCAN18	0	0	0	1	0	0	0	0	0	0	1	0
ENSMUSG00000070858	GM1673	1	1	2	4	1	1	8	6	3	9	7	8
ENSMUSG00000070866	ZFP804A	0	0	0	0	0	0	0	0	0	1	0	0
ENSMUSG00000070871	CCNYL1	2	0	1	0	1	0	5	0	1	7	1	1
ENSMUSG00000070877	LDLRAD1	0	0	0	0	0	0	0	0	1	0	0	0
ENSMUSG00000070880	GAD1	0	0	0	0	0	0	1	0	1	1	0	0
ENSMUSG00000070883	CCDC173	2	11	10	12	7	9	29	38	26	30	32	27
ENSMUSG00000070896	RPL23A-PS1	0	0	0	1	0	0	0	0	1	0	0	1
ENSMUSG00000070923	KLHL9	8	8	9	14	18	20	71	63	35	78	39	62
ENSMUSG00000070934	RRAGA	20	27	34	20	23	38	179	184	131	152	129	167
ENSMUSG00000070939	TGFBRAP1	1	3	3	5	2	1	9	17	9	15	6	12
ENSMUSG00000070953	RABEPK	8	8	2	11	8	10	56	65	48	60	36	49
ENSMUSG00000070972	DNAJC25	8	7	8	7	9	6	29	58	21	39	17	28
ENSMUSG00000070980	ACTL7B	0	0	0	0	0	0	0	0	0	1	0	0
ENSMUSG00000070997	1700055D18F	0	0	0	0	0	0	1	1	0	2	1	2
ENSMUSG00000071014	NDUFB6	17	22	18	22	18	24	165	183	123	172	139	132
ENSMUSG00000071037	CAMKMT	0	0	0	0	1	2	1	1	1	0	1	0
ENSMUSG00000071041	IMPDH2-PS	1	4	4	1	2	1	5	5	2	8	6	9
ENSMUSG00000071052	RPL7A-PS5	0	0	1	0	0	1	0	1	1	1	0	0
ENSMUSG00000071054	SAFB	22	19	32	24	27	23	122	118	83	128	104	108
ENSMUSG00000071068	TREML2	0	1	0	1	0	0	0	0	0	0	0	0
ENSMUSG00000071072	PTGES3	91	109	114	130	110	107	449	423	343	444	347	403
ENSMUSG00000071073	LRRC73	0	1	2	2	1	2	2	2	1	3	1	4
ENSMUSG00000071074	YIPF3	1	8	5	6	5	2	24	34	29	30	27	30
ENSMUSG00000071076	JUND	12	21	16	21	19	23	125	124	98	123	91	120
ENSMUSG00000071078	NR2C2AP	2	2	6	4	4	5	26	20	21	24	22	22
ENSMUSG00000071103	1700029J07R	0	0	0	0	0	0	3	5	2	0	0	2
ENSMUSG00000071104	CCDC110	1	0	0	0	0	0	0	0	0	0	1	0
ENSMUSG00000071112	SPX	0	0	1	0	1	0	1	2	3	3	0	0
ENSMUSG00000071113	MBOAT4	0	0	0	0	0	0	1	2	2	0	0	0
ENSMUSG00000071141	RPL36A-PS3	0	0	0	0	0	0	0	1	1	0	0	1
ENSMUSG00000071158	GM156	0	0	0	0	0	0	1	0	0	0	0	0
ENSMUSG00000071172	SRSF3	34	44	38	44	39	34	312	288	195	289	238	218
ENSMUSG00000071180	SMIM15	16	25	15	13	20	14	77	116	71	110	70	89
ENSMUSG00000071181	3830408C21F	0	0	0	0	0	0	0	2	2	0	0	1
ENSMUSG00000071192	WFIKKN1	0	0	0	1	1	0	2	2	0	2	3	3
ENSMUSG00000071202	CCDC78	7	7	3	4	7	3	22	11	16	11	13	23
ENSMUSG00000071203	NAIP5	0	1	1	0	2	1	1	1	2	0	0	1
ENSMUSG00000071226	CECR2	0	0	1	0	0	0	0	1	1	1	1	0
ENSMUSG00000071229	TIMM8A2	0	0	0	0	0	0	0	0	1	1	1	1

ENSMUSG00000071252	2210408I21R	0	0	0	2	0	1	5	2	2	1	2	3
ENSMUSG00000071253	SLC25A16	3	2	0	3	4	0	14	18	14	17	13	13
ENSMUSG00000071256	ZFP213	1	0	0	0	0	2	6	6	2	8	3	7
ENSMUSG00000071262	ZFP957	0	1	0	0	0	0	0	0	0	0	0	0
ENSMUSG00000071267	ZFP942	4	5	4	5	5	3	23	20	17	31	14	18
ENSMUSG00000071281	ZFP65	1	6	3	4	2	1	18	17	5	17	3	7
ENSMUSG00000071291	ZFP58	1	1	4	1	2	2	3	3	1	6	4	3
ENSMUSG00000071302	2610044O15I	1	1	8	2	1	4	14	13	11	16	12	9
ENSMUSG00000071303	RPS8-PS1	0	0	0	1	0	0	0	1	0	1	0	1
ENSMUSG00000071317	BVES	0	0	0	0	0	0	0	0	2	0	0	0
ENSMUSG00000071324	ARMC2	0	0	1	0	0	0	0	1	0	3	0	0
ENSMUSG00000071332	E230015B07F	0	0	0	0	0	1	0	0	0	0	1	0
ENSMUSG00000071337	TIA1	14	16	15	14	30	21	78	71	68	92	51	78
ENSMUSG00000071347	C1QTNF9	0	0	0	0	0	0	1	0	0	0	0	0
ENSMUSG00000071350	SETDB2	4	2	3	1	1	0	8	4	6	19	4	10
ENSMUSG00000071359	TBPL1	7	12	9	18	11	17	56	70	47	74	45	51
ENSMUSG00000071369	MAP3K5	1	0	1	2	4	2	1	1	4	6	3	6
ENSMUSG00000071379	HPCAL1	1	4	6	3	2	2	24	30	24	23	16	19
ENSMUSG00000071392	ECT2L	0	0	0	1	0	0	1	1	0	1	2	0
ENSMUSG00000071415	RPL23	116	141	121	161	159	131	752	860	643	786	566	708
ENSMUSG00000071419	RPS15-PS2	1	1	0	0	0	1	1	1	2	1	0	2
ENSMUSG00000071424	GRID2	0	0	0	0	0	0	0	1	0	1	0	0
ENSMUSG00000071451	PSMG4	5	5	4	3	3	5	29	39	18	30	22	19
ENSMUSG00000071454	DTNB	6	3	5	3	5	2	24	20	17	18	11	17
ENSMUSG00000071456	1110002L01R	0	4	3	2	4	3	20	16	12	24	12	26
ENSMUSG00000071475	RPL21-PS5	1	0	0	0	0	0	0	0	0	0	1	0
ENSMUSG00000071477	ZFP777	0	1	2	3	0	2	9	7	4	8	5	5
ENSMUSG00000071478	HIST1H2AD	0	0	0	0	0	1	0	0	1	0	1	0
ENSMUSG00000071497	NUTF2-PS1	8	18	23	13	17	14	39	37	45	49	37	38
ENSMUSG00000071516	HIST1H2AI	0	0	0	0	0	1	0	0	0	0	0	1
ENSMUSG00000071528	USMG5	42	34	53	45	60	50	500	492	348	470	446	394
ENSMUSG00000071531	GPRIN2	0	0	0	0	0	0	0	0	0	0	0	1
ENSMUSG00000071532	GM10335	0	0	0	0	0	0	0	1	0	0	0	0
ENSMUSG00000071533	PCNP	13	16	13	20	20	23	76	60	45	81	59	65
ENSMUSG00000071537	KLRG2	0	0	0	0	0	1	0	0	0	0	0	0
ENSMUSG00000071547	NT5DC2	4	4	0	5	3	3	17	23	12	21	14	12
ENSMUSG00000071562	STFA1	0	0	0	0	2	0	0	0	0	0	0	0
ENSMUSG00000071573	RNLS	0	0	0	0	0	0	3	0	1	0	2	0
ENSMUSG00000071632	2510002D24I	14	12	10	11	11	7	74	56	51	68	40	44
ENSMUSG00000071633	GM4952	0	0	0	0	0	0	1	0	1	0	1	1
ENSMUSG00000071636	RIMBP3	0	0	0	0	0	0	0	1	0	0	1	0
ENSMUSG00000071637	CEBPD	3	9	2	2	1	8	12	5	6	11	9	4
ENSMUSG00000071644	EEF1G	58	55	52	65	53	64	379	380	285	364	280	328
ENSMUSG00000071645	TUT1	2	7	4	3	5	7	32	31	25	23	16	15
ENSMUSG00000071646	MTA2	14	15	16	21	16	16	76	94	59	104	62	92
ENSMUSG00000071647	EML3	7	7	9	6	10	3	47	29	19	32	34	21
ENSMUSG00000071648	ROM1	1	1	0	0	0	0	0	4	1	1	2	4
ENSMUSG00000071649	B3GAT3	6	7	5	9	8	6	32	33	41	34	18	39
ENSMUSG00000071650	GANAB	18	19	20	21	21	20	89	116	90	111	69	86
ENSMUSG00000071652	INTS5	2	2	4	2	3	3	6	14	9	17	12	15
ENSMUSG00000071653	1810009A15I	0	0	0	0	0	0	1	0	1	0	2	0
ENSMUSG00000071654	UQCC3	2	2	9	5	3	8	24	33	15	17	23	13
ENSMUSG00000071655	UBXN1	15	18	17	15	19	16	110	109	69	105	83	90
ENSMUSG00000071656	LRRN4CL	0	0	0	0	0	0	1	0	0	2	1	2
ENSMUSG00000071657	BSCL2	5	2	0	3	2	6	23	18	17	29	14	13
ENSMUSG00000071658	GNG3	0	2	1	1	1	1	2	1	0	1	0	0
ENSMUSG00000071659	HNRNPUL2	20	27	19	30	27	24	96	155	94	113	108	107
ENSMUSG00000071660	TTC9C	2	7	6	10	9	11	51	52	24	44	40	38

ENSMUSG00000071661	ZBTB3	1	1	0	0	0	0	0	0	0	0	0	0
ENSMUSG00000071662	POLR2G	7	7	8	8	10	6	62	59	55	53	46	46
ENSMUSG00000071665	FOXR2	2	2	6	4	9	4	56	40	25	37	20	35
ENSMUSG00000071669	SNX29	0	1	0	0	0	1	0	1	0	1	0	0
ENSMUSG00000071671	GM10343	0	0	0	1	0	0	0	0	1	0	0	0
ENSMUSG00000071680	GM7123	0	0	0	0	0	0	0	0	1	0	0	0
ENSMUSG00000071691	GM960	1	0	0	0	0	0	0	1	0	2	0	0
ENSMUSG00000071708	SMS	8	17	13	13	7	12	113	70	60	78	71	73
ENSMUSG00000071711	MPST	0	3	3	4	1	4	24	19	13	21	6	12
ENSMUSG00000071713	CSF2RB	0	0	1	0	0	0	0	0	0	1	0	0
ENSMUSG00000071715	NCF4	0	0	0	0	0	0	0	1	0	0	0	1
ENSMUSG00000071724	SMPD5	0	0	0	0	0	0	0	0	0	1	0	0
ENSMUSG00000071748	GM14698	1	1	0	2	0	2	1	1	0	2	0	2
ENSMUSG00000071757	ZHX2	2	4	4	3	4	1	7	10	9	13	12	14
ENSMUSG00000071793	2610005L07R	0	0	0	0	0	1	1	0	0	0	0	0
ENSMUSG00000071796	6820431F20R	1	0	0	0	1	0	1	2	0	5	0	0
ENSMUSG00000071847	APCDD1	0	0	0	2	0	0	0	2	0	0	0	1
ENSMUSG00000071855	CCDC112	1	6	1	6	3	5	19	20	15	22	20	19
ENSMUSG00000071856	MCC	0	0	1	0	0	0	1	1	0	1	1	0
ENSMUSG00000071862	LRRTM2	0	0	1	0	0	0	0	1	0	0	1	0
ENSMUSG00000071866	PPIA	9	14	3	9	9	2	86	85	71	90	70	84
ENSMUSG00000071893	VMN1R4	0	0	0	0	0	0	1	1	0	0	0	0
ENSMUSG00000072066	6720489N17I	2	3	0	1	0	3	6	5	2	6	4	4
ENSMUSG00000072082	CCNF	3	9	6	2	8	2	14	30	22	27	20	32
ENSMUSG00000072109	A530040E14R	0	0	0	0	0	0	0	0	0	0	1	0
ENSMUSG00000072115	ANG	0	0	0	0	0	0	0	0	1	1	0	0
ENSMUSG00000072235	TUBA1A	3	7	9	4	6	2	40	51	31	47	35	40
ENSMUSG00000072244	TRIM6	0	0	0	0	0	0	0	0	2	0	0	0
ENSMUSG00000072255	RPL31-PS17	1	0	1	0	0	0	0	1	2	1	2	2
ENSMUSG00000072258	TAF1A	2	2	0	4	4	3	27	18	13	14	18	13
ENSMUSG00000072294	KLF12	0	1	0	0	1	1	2	2	2	2	6	2
ENSMUSG00000072324	GM8420	0	1	0	2	2	0	2	0	2	2	1	2
ENSMUSG00000072407	GM6419	0	0	0	0	0	0	0	0	0	1	0	0
ENSMUSG00000072419	DPPA2	0	1	0	0	0	0	0	0	0	0	0	0
ENSMUSG00000072437	NANOS1	2	2	2	2	1	0	7	5	4	10	1	4
ENSMUSG00000072460	ATP5L-PS1	1	0	0	0	0	0	1	0	0	0	0	0
ENSMUSG00000072476	GM9008	3	0	4	6	2	4	7	5	2	8	2	5
ENSMUSG00000072487	MROH5	0	1	0	0	0	0	0	0	0	0	1	1
ENSMUSG00000072492	GM10364	0	0	0	0	0	0	0	1	0	0	0	0
ENSMUSG00000072494	PPP1R3E	0	2	0	2	0	0	2	0	0	2	2	3
ENSMUSG00000072501	PHF20L1	18	29	22	48	28	37	125	144	104	140	122	94
ENSMUSG00000072568	FAM84B	9	13	9	12	8	17	95	78	59	86	59	73
ENSMUSG00000072571	TMEM253	0	0	0	0	0	0	0	0	0	1	1	0
ENSMUSG00000072572	SLC39A2	0	2	0	0	0	0	1	0	0	0	0	0
ENSMUSG00000072573	GM10369	0	0	0	0	0	0	1	1	1	2	0	0
ENSMUSG00000072576	GM16020	0	0	0	1	0	0	0	1	0	1	0	0
ENSMUSG00000072582	PTRH2	8	9	4	8	3	8	45	48	37	50	39	49
ENSMUSG00000072592	GM10373	0	0	0	0	0	0	0	0	1	0	0	1
ENSMUSG00000072612	GM10382	1	0	0	0	0	0	0	0	0	1	0	1
ENSMUSG00000072620	SLFN2	11	11	8	4	5	9	35	36	16	30	18	22
ENSMUSG00000072621	SLFN10-PS	29	47	37	45	53	50	183	165	119	206	171	158
ENSMUSG00000072623	ZFP9	4	10	8	11	4	6	22	35	11	34	21	20
ENSMUSG00000072640	LYRM9	2	0	1	1	0	2	4	10	4	4	3	4
ENSMUSG00000072647	ADAM1A	0	0	0	0	0	0	0	0	1	0	0	1
ENSMUSG00000072653	ZFP783	0	0	0	0	0	0	0	0	0	1	1	1
ENSMUSG00000072662	MANSC4	0	0	0	0	0	0	0	0	0	1	0	0
ENSMUSG00000072673	GM10392	1	0	0	0	0	0	0	0	1	2	1	0
ENSMUSG00000072683	GM7457	0	0	0	0	0	0	0	0	1	0	0	0

ENSMUSG00000072692	RPL37RT	36	33	34	48	28	36	153	107	99	135	106	128
ENSMUSG00000072693	GM10401	0	1	0	3	1	1	7	6	2	5	4	10
ENSMUSG00000072694	1500011B03F	2	1	2	1	3	2	9	12	1	6	5	9
ENSMUSG00000072704	SMIM10L1	37	33	36	51	37	29	216	205	139	247	130	195
ENSMUSG00000072714	RPL21-PS4	0	1	0	0	0	1	0	0	1	0	0	0
ENSMUSG00000072718	KLRA10	0	0	0	0	0	0	1	1	0	0	0	0
ENSMUSG00000072723	GM10044	0	0	0	0	0	1	0	0	0	0	0	0
ENSMUSG00000072761	GM6712	1	0	0	2	0	2	4	2	4	4	1	3
ENSMUSG00000072762	4930522L14R	1	1	0	5	0	1	20	17	18	34	16	18
ENSMUSG00000072763	5430403G16I	1	2	0	0	2	1	3	7	5	6	3	2
ENSMUSG00000072769	GM10419	0	0	0	1	0	0	0	1	0	0	0	0
ENSMUSG00000072770	ACRBP	0	1	1	0	2	0	7	7	3	0	3	3
ENSMUSG00000072772	GRCC10	1	0	0	0	0	0	0	0	1	0	0	0
ENSMUSG00000072774	ZFP951	0	0	0	0	0	0	0	1	0	0	0	0
ENSMUSG00000072789	GM10420	0	1	1	0	0	1	0	1	1	3	0	1
ENSMUSG00000072809	9330160F10R	0	1	1	2	2	0	6	5	3	7	0	5
ENSMUSG00000072812	AHNAK2	0	0	0	0	1	0	0	0	0	0	2	0
ENSMUSG00000072825	CEP170B	1	1	2	0	2	1	8	6	7	9	7	7
ENSMUSG00000072844	G530011O06	1	0	1	0	0	0	1	1	0	0	0	0
ENSMUSG00000072847	A530017D24I	0	1	0	1	1	1	3	5	6	6	5	1
ENSMUSG00000072872	RYBP	1	0	0	1	0	1	6	1	4	2	2	0
ENSMUSG00000072889	NFXL1	9	12	13	15	15	20	66	69	36	64	42	46
ENSMUSG00000072893	4933439C10F	0	0	1	1	2	2	3	8	6	4	5	2
ENSMUSG00000072898	GM10434	0	0	0	1	0	1	2	1	3	1	1	2
ENSMUSG00000072915	GM12258	1	0	0	0	1	0	0	2	0	2	1	1
ENSMUSG00000072919	NOXRED1	0	1	0	0	0	0	2	0	2	0	0	0
ENSMUSG00000072940	GM10443	1	0	0	0	0	1	0	0	0	0	0	1
ENSMUSG00000072946	PTGR2	7	3	5	7	3	1	29	20	16	17	15	32
ENSMUSG00000072949	ACOT1	0	0	0	0	0	0	0	0	1	0	0	0
ENSMUSG00000072955	TMSB15L	0	0	0	0	0	0	0	0	0	0	0	1
ENSMUSG00000072964	BHLHB9	4	1	1	5	1	2	12	10	12	24	10	11
ENSMUSG00000072969	ARMCX5	1	0	2	1	1	1	11	5	7	12	11	4
ENSMUSG00000072972	ADAM4	0	0	0	0	0	0	0	1	0	1	1	2
ENSMUSG00000072974	GM4787	0	0	0	0	0	0	1	2	1	0	0	0
ENSMUSG00000072980	OIP5	5	1	3	7	0	2	27	11	25	17	18	7
ENSMUSG00000072999	GM15401	0	0	0	0	0	0	0	0	0	1	0	0
ENSMUSG00000073000	GM10451	3	4	2	3	2	3	13	15	10	12	12	14
ENSMUSG00000073002	VAMP5	0	0	0	0	0	1	0	1	1	2	1	1
ENSMUSG00000073008	GPR174	0	1	0	0	1	0	0	5	2	2	4	4
ENSMUSG00000073010	GM5127	0	0	0	0	0	0	0	0	0	1	1	0
ENSMUSG00000073016	UPRT	0	0	0	1	0	0	0	2	2	1	0	1
ENSMUSG00000073062	ZXDB	4	6	4	6	4	9	26	27	22	19	20	26
ENSMUSG00000073067	9130019P16F	1	3	0	1	1	1	3	3	4	5	3	1
ENSMUSG00000073073	GM8098	0	0	0	0	0	0	0	0	0	0	0	1
ENSMUSG00000073079	SRP54	0	0	1	0	1	1	0	3	2	4	2	2
ENSMUSG00000073096	LRRC61	0	0	0	0	0	0	0	0	1	1	0	0
ENSMUSG00000073102	DRC1	1	0	0	0	0	0	0	0	0	0	0	1
ENSMUSG00000073125	XLR3B	0	0	0	0	1	0	2	3	0	2	5	8
ENSMUSG00000073130	GM1141	0	0	0	1	0	0	0	2	0	2	2	0
ENSMUSG00000073131	VMA21	7	4	5	10	7	12	48	47	29	40	28	37
ENSMUSG00000073139	TMEM185A	2	1	3	6	6	1	13	22	14	19	22	22
ENSMUSG00000073144	4930599N23I	0	1	0	0	0	0	1	1	0	0	0	0
ENSMUSG00000073145	GM12000	0	0	0	0	0	0	0	0	0	0	1	0
ENSMUSG00000073147	5031425E22F	3	1	7	5	2	4	18	19	12	19	7	17
ENSMUSG00000073155	1810058I24R	6	7	3	9	6	5	48	42	32	41	30	36
ENSMUSG00000073176	ZFP449	1	0	2	0	0	0	3	0	0	3	2	1
ENSMUSG00000073179	GM10478	0	0	0	1	1	0	0	1	1	2	1	0
ENSMUSG00000073233	GM9144	0	0	0	0	0	0	0	1	0	0	0	0

ENSMUSG00000073236	2500004C02F	1	1	0	4	4	2	12	10	6	8	9	2
ENSMUSG00000073242	DNMT3AOS	0	0	0	0	0	0	0	0	0	0	1	0
ENSMUSG00000073274	GM14636	0	0	1	1	1	0	1	0	1	0	1	1
ENSMUSG00000073290	GM10490	0	0	0	0	0	0	0	0	0	0	1	0
ENSMUSG00000073374	C030034I22R	0	1	2	1	0	1	6	7	1	3	2	5
ENSMUSG00000073394	RUNX2OS1	0	0	0	0	0	0	0	0	1	1	0	0
ENSMUSG00000073403	GM10499	2	0	0	0	0	0	0	0	0	0	0	0
ENSMUSG00000073405	H2-T-PS	1	0	2	1	0	2	9	13	6	9	4	8
ENSMUSG00000073406	H2-BL	0	0	0	0	0	0	1	0	0	1	0	0
ENSMUSG00000073411	H2-D1	57	68	64	95	66	94	427	454	356	460	324	416
ENSMUSG00000073412	LST1	0	0	0	0	0	0	0	0	0	2	0	0
ENSMUSG00000073415	GM10501	2	0	1	0	2	1	6	10	4	2	3	2
ENSMUSG00000073421	H2-AB1	0	0	0	0	0	0	1	0	0	0	0	0
ENSMUSG00000073422	H2-KE6	0	1	0	0	0	0	1	1	2	2	2	0
ENSMUSG00000073423	ZFP414	1	10	0	3	5	4	13	10	6	13	8	11
ENSMUSG00000073427	GM4924	2	2	0	4	0	1	5	8	5	5	6	2
ENSMUSG00000073430	GM10505	1	0	0	0	0	0	0	0	0	1	1	0
ENSMUSG00000073433	ARHGDIG	0	0	0	2	0	0	0	0	0	0	0	0
ENSMUSG00000073434	WDR90	4	2	6	4	7	5	17	18	12	18	14	18
ENSMUSG00000073435	NME3	0	0	0	0	0	0	0	1	0	0	0	0
ENSMUSG00000073436	EME2	1	1	0	1	2	1	6	2	2	5	4	6
ENSMUSG00000073437	D330041H03I	0	0	0	3	2	1	4	2	3	4	3	8
ENSMUSG00000073460	PNLDC1	0	0	0	0	1	2	2	1	4	1	3	3
ENSMUSG00000073468	SFT2D1	3	5	4	7	10	6	42	33	27	50	31	25
ENSMUSG00000073471	RSPH3A	0	1	0	0	0	0	0	1	1	1	0	0
ENSMUSG00000073478	D730003I15R	0	1	1	0	0	0	7	7	2	1	2	4
ENSMUSG00000073481	MARC2	4	4	11	12	9	6	58	53	51	59	54	43
ENSMUSG00000073489	IFI204	0	0	0	0	0	0	1	1	1	1	1	0
ENSMUSG00000073491	IFI213	0	1	1	0	1	2	1	0	0	1	0	0
ENSMUSG00000073529	F830208F22R	0	0	0	0	0	0	0	0	1	0	0	0
ENSMUSG00000073538	E330020D12F	3	2	2	2	3	2	10	15	10	19	8	15
ENSMUSG00000073542	CEP76	2	3	1	5	1	2	11	16	7	12	8	6
ENSMUSG00000073557	PPP1R12B	1	2	5	3	5	3	17	18	16	15	16	14
ENSMUSG00000073559	GM8618	0	0	0	0	0	0	0	2	1	0	0	0
ENSMUSG00000073563	CSNK1G3	2	10	13	3	2	5	46	31	19	26	20	22
ENSMUSG00000073569	GM10540	0	0	0	0	0	0	0	1	0	0	0	0
ENSMUSG00000073590	3222401L13R	0	0	1	0	0	0	1	0	0	0	0	0
ENSMUSG00000073598	1700066B19F	1	1	1	0	0	1	1	1	1	0	0	0
ENSMUSG00000073600	PROB1	0	0	0	0	0	1	1	1	0	0	1	3
ENSMUSG00000073607	GM10548	0	0	0	0	0	1	0	1	0	1	2	0
ENSMUSG00000073609	D2HGDH	0	0	0	3	0	2	6	3	3	2	0	2
ENSMUSG00000073616	COPS9	15	19	18	21	18	20	161	141	120	143	119	107
ENSMUSG00000073627	C130036L24F	2	2	0	0	0	0	6	5	3	9	3	4
ENSMUSG00000073633	FBXO36	0	0	1	1	0	0	4	2	2	1	3	4
ENSMUSG00000073639	RAB18	12	9	7	10	6	9	43	29	31	48	26	28
ENSMUSG00000073640	RPL27-PS3	10	10	17	15	13	13	36	45	35	50	31	34
ENSMUSG00000073643	WDFY1	5	10	6	13	8	8	26	25	25	41	29	31
ENSMUSG00000073647	GM10557	0	0	0	0	1	0	3	1	2	6	1	2
ENSMUSG00000073650	CATIP	0	0	0	0	0	0	0	0	0	2	0	1
ENSMUSG00000073656	GM10558	0	0	0	0	0	0	0	0	1	0	0	0
ENSMUSG00000073664	NBEAL1	7	15	6	12	9	6	30	36	24	41	30	37
ENSMUSG00000073676	HSPE1	47	60	68	74	63	83	513	591	389	516	395	447
ENSMUSG00000073678	PGAP1	0	1	0	5	0	1	9	10	4	4	6	5
ENSMUSG00000073682	GM10563	1	1	0	0	0	0	1	0	1	2	1	2
ENSMUSG00000073684	FAAP20	2	4	1	0	3	2	10	13	6	10	6	10
ENSMUSG00000073700	KLHL21	1	1	6	3	4	4	31	24	19	22	19	26
ENSMUSG00000073702	RPL31	100	123	133	152	145	143	739	803	579	771	511	708
ENSMUSG00000073705	CENPS	0	2	2	4	0	3	17	14	6	11	18	16

ENSMUSG00000073722	4931408C20F	0	1	1	0	1	1	5	4	1	4	3	1
ENSMUSG00000073725	LMBRD1	101	139	116	123	130	117	714	818	578	863	587	602
ENSMUSG00000073730	4933415F23F	0	0	2	2	0	0	0	0	1	1	1	2
ENSMUSG00000073733	RSG1	0	1	0	0	0	0	3	1	3	2	3	2
ENSMUSG00000073739	GM16287	1	0	0	0	0	0	0	1	2	0	1	0
ENSMUSG00000073753	GM10571	0	0	0	0	0	0	1	0	1	0	0	0
ENSMUSG00000073755	5730409E04F	3	3	2	5	7	7	12	16	9	18	15	18
ENSMUSG00000073758	SH3D21	1	1	0	0	0	0	2	0	1	3	1	1
ENSMUSG00000073761	4933427I04R	0	0	0	0	0	0	0	0	0	0	0	1
ENSMUSG00000073771	BTBD19	0	0	0	1	0	0	2	3	2	3	1	3
ENSMUSG00000073774	KNCN	0	0	0	0	0	0	0	0	0	0	0	1
ENSMUSG00000073775	KTI12	7	2	4	3	7	3	26	26	30	25	22	24
ENSMUSG00000073778	FADDOS	0	1	0	0	0	0	0	0	0	0	0	0
ENSMUSG00000073781	GM6471	0	0	0	0	0	1	0	0	0	0	0	0
ENSMUSG00000073787	GM10575	0	1	0	0	0	0	0	0	1	0	0	0
ENSMUSG00000073791	EFCAB7	2	0	0	2	0	1	14	14	8	5	4	6
ENSMUSG00000073792	ALG6	6	3	5	3	6	1	12	10	7	14	5	17
ENSMUSG00000073802	CDKN2B	0	0	0	0	1	0	0	2	0	1	2	1
ENSMUSG00000073838	TUFM	4	5	6	4	7	5	24	19	22	25	17	33
ENSMUSG00000073856	IQCK	0	0	0	1	0	0	1	0	0	0	1	0
ENSMUSG00000073867	AA474408	0	1	0	0	1	0	2	1	1	2	0	2
ENSMUSG00000073889	IL11RA1	1	2	0	0	2	0	1	0	2	3	1	0
ENSMUSG00000073898	OLFR713	0	0	0	0	0	0	0	1	3	2	1	0
ENSMUSG00000073899	OLFR1532-PS	0	0	0	0	0	0	2	0	2	1	0	0
ENSMUSG00000073902	GM1966	2	1	1	0	0	3	11	14	9	19	17	13
ENSMUSG00000073910	MOB3B	0	0	0	1	2	2	5	6	6	8	4	4
ENSMUSG00000073927	OLFR652	0	0	0	1	0	0	0	0	1	0	0	0
ENSMUSG00000073929	TRIM30E-PS1	0	0	0	0	0	0	0	0	0	1	0	0
ENSMUSG00000073968	TRIM68	0	0	0	0	0	1	1	2	3	1	0	1
ENSMUSG00000073972	OLFR553	0	0	0	0	0	1	0	0	0	0	0	0
ENSMUSG00000073973	OLFR552	1	0	0	0	0	0	0	0	0	0	0	0
ENSMUSG00000073974	OLFR551	0	0	0	0	0	0	0	0	0	0	0	1
ENSMUSG00000073975	OLFR550	0	0	0	0	0	0	0	1	0	0	0	0
ENSMUSG00000073978	OLFR548-PS1	0	0	0	0	0	0	0	0	1	0	0	0
ENSMUSG00000073980	GM9574	0	0	0	0	0	0	0	0	0	1	0	0
ENSMUSG00000073982	RHOG	1	5	11	1	2	3	21	17	9	25	12	18
ENSMUSG00000073985	GM10602	0	0	0	0	0	0	0	1	1	0	1	1
ENSMUSG00000073987	GGH	0	4	3	3	2	0	16	17	12	7	11	2
ENSMUSG00000074001	KLHL40	0	0	1	0	0	1	0	1	0	0	0	0
ENSMUSG00000074004	B3GNT6	0	0	0	0	0	0	1	0	0	0	0	0
ENSMUSG00000074024	4632427E13F	7	5	4	6	2	9	23	17	11	25	21	19
ENSMUSG00000074030	EXOC8	3	2	4	2	2	2	9	11	16	12	10	9
ENSMUSG00000074034	GM5921	1	0	0	1	1	1	1	4	5	6	6	7
ENSMUSG00000074037	MC1R	1	0	0	1	0	0	0	2	0	0	0	0
ENSMUSG00000074039	4930520O04I	1	1	0	1	0	1	5	2	1	3	1	3
ENSMUSG00000074052	BC048644	0	0	0	0	0	1	0	0	0	1	2	0
ENSMUSG00000074063	OSGIN1	0	0	1	1	1	1	1	2	0	0	0	1
ENSMUSG00000074064	MLYCD	1	1	2	1	3	0	4	5	0	6	4	9
ENSMUSG00000074088	SNRNP40	21	20	26	25	30	28	145	131	116	143	97	110
ENSMUSG00000074102	RBM15B	5	14	7	16	7	14	29	39	32	42	26	32
ENSMUSG00000074121	NTF5	0	0	0	0	0	0	0	0	0	0	0	1
ENSMUSG00000074129	RPL13A	65	72	68	70	59	71	328	347	260	345	269	355
ENSMUSG00000074136	4930513N10I	0	2	0	2	1	3	2	4	2	5	0	2
ENSMUSG00000074141	IL4I1	0	0	0	0	0	0	0	1	0	0	2	1
ENSMUSG00000074151	NLRC5	4	2	1	6	5	2	24	29	15	22	13	20
ENSMUSG00000074165	ZFP788	2	1	2	0	9	2	20	16	10	18	13	14
ENSMUSG00000074166	AW146154	2	3	4	3	3	2	10	8	6	11	9	5
ENSMUSG00000074170	PLEKHF1	0	1	0	0	0	0	0	0	1	0	0	0

ENSMUSG00000074178	GM10638	0	0	0	0	1	0	1	0	0	0	0	0
ENSMUSG00000074182	ZNHIT6	2	7	8	3	11	2	38	47	33	50	32	34
ENSMUSG00000074203	G430095P16I	0	0	0	0	0	0	3	1	2	1	1	0
ENSMUSG00000074210	E130208F15R	0	0	0	0	0	0	0	0	1	1	0	0
ENSMUSG00000074211	SDHAF1	1	6	5	2	3	3	19	10	10	18	9	14
ENSMUSG00000074212	DNAJB14	6	7	7	7	8	8	21	26	14	21	19	21
ENSMUSG00000074213	GM10642	0	0	0	0	0	0	0	0	5	1	1	1
ENSMUSG00000074215	GM10643	0	0	0	0	0	1	1	0	1	0	1	1
ENSMUSG00000074218	COX7A1	1	0	0	0	0	0	2	1	0	1	0	2
ENSMUSG00000074219	GM10644	2	0	0	0	0	0	0	2	0	0	1	1
ENSMUSG00000074220	ZFP382	0	1	0	0	1	1	6	3	1	1	0	0
ENSMUSG00000074221	ZFP568	3	7	2	4	6	7	25	27	19	30	12	24
ENSMUSG00000074224	4932431P20F	0	0	0	0	0	0	0	0	0	0	0	1
ENSMUSG00000074238	AP1AR	9	15	16	14	20	17	69	77	51	92	63	53
ENSMUSG00000074240	CIB3	0	0	0	0	0	0	0	0	0	1	1	0
ENSMUSG00000074247	DDA1	6	7	3	11	11	6	27	32	32	34	28	39
ENSMUSG00000074250	GM10653	0	0	0	0	0	0	0	0	1	0	0	0
ENSMUSG00000074252	GM10654	0	0	0	0	0	0	0	1	1	0	0	0
ENSMUSG00000074261	ERICH4	0	0	0	0	0	0	0	0	1	0	0	0
ENSMUSG00000074264	AMY1	6	4	6	8	7	7	18	28	7	20	10	14
ENSMUSG00000074269	REC114	0	0	0	1	0	0	1	3	4	3	2	2
ENSMUSG00000074277	PHLDB3	0	0	0	0	0	0	0	1	0	0	0	0
ENSMUSG00000074284	GM10658	1	0	0	0	0	1	2	2	2	2	2	2
ENSMUSG00000074300	BC030870	0	0	1	0	0	0	0	0	0	0	0	0
ENSMUSG00000074305	PEAK1	6	6	6	7	10	7	25	24	15	42	25	25
ENSMUSG00000074340	OVGP1	6	5	6	3	3	4	15	19	7	18	15	8
ENSMUSG00000074355	GM10676	0	1	0	0	0	1	0	0	2	0	0	0
ENSMUSG00000074357	AA386476	0	0	0	0	0	0	1	2	1	0	1	1
ENSMUSG00000074358	CCDC61	3	6	5	4	0	1	10	17	11	9	11	8
ENSMUSG00000074385	GM10684	0	1	2	0	0	1	3	2	0	3	3	0
ENSMUSG00000074397	FOXR1	0	0	0	0	0	0	0	0	0	0	0	1
ENSMUSG00000074398	GM15441	1	0	0	0	0	0	1	0	0	0	0	1
ENSMUSG00000074403	HIST2H3B	0	0	0	0	0	0	1	2	0	0	0	0
ENSMUSG00000074405	ZFP865	4	3	3	2	6	2	12	17	15	10	6	15
ENSMUSG00000074406	ZFP628	1	0	3	0	0	0	7	6	3	3	1	7
ENSMUSG00000074412	GM10689	0	0	1	3	1	1	1	5	4	5	5	1
ENSMUSG00000074415	2610203C20F	0	0	0	1	0	0	0	1	1	1	1	1
ENSMUSG00000074452	PATE2	0	2	0	3	0	0	6	4	1	1	1	4
ENSMUSG00000074466	GM15417	1	1	0	0	0	0	1	0	1	0	1	2
ENSMUSG00000074472	ZFP872	0	0	0	0	1	0	0	0	0	1	0	0
ENSMUSG00000074476	SPC24	15	19	13	19	9	17	104	99	79	117	83	83
ENSMUSG00000074479	GM10704	0	0	0	0	0	0	1	1	0	0	0	0
ENSMUSG00000074480	MEX3A	7	5	6	9	7	10	33	22	18	30	22	26
ENSMUSG00000074483	BGLAP	0	0	0	0	0	0	0	0	0	0	0	1
ENSMUSG00000074497	A430078G23I	0	0	0	0	0	1	1	1	1	0	1	3
ENSMUSG00000074513	ARFIP1	6	3	11	4	9	6	29	27	22	26	20	30
ENSMUSG00000074516	GM10709	0	0	0	0	0	0	0	0	1	0	0	0
ENSMUSG00000074519	ZFP971	0	1	0	0	0	0	1	0	0	4	0	1
ENSMUSG00000074547	RPS8-PS4	0	5	3	5	4	8	22	20	14	14	12	16
ENSMUSG00000074553	EIF1-PS1	0	0	0	0	0	0	0	0	0	1	0	0
ENSMUSG00000074564	GM10720	0	0	0	0	0	3	4	1	0	7	1	0
ENSMUSG00000074576	MOCS3	1	2	1	1	2	0	2	5	6	9	6	9
ENSMUSG00000074577	RIPOR3	0	0	0	0	0	0	0	0	1	0	0	0
ENSMUSG00000074578	ZFAS1	27	34	32	39	31	42	158	237	147	194	175	132
ENSMUSG00000074579	LEKR1	0	0	0	1	0	0	1	4	1	0	3	0
ENSMUSG00000074580	4931440P22F	1	2	1	1	1	0	3	3	2	3	2	2
ENSMUSG00000074582	ARFGEF2	2	10	2	5	2	7	27	22	12	46	20	18
ENSMUSG00000074603	GM10729	0	0	0	0	0	0	1	0	0	0	0	0

ENSMUSG00000074604	MGST2	1	1	0	0	0	0	1	0	2	0	0	1
ENSMUSG00000074607	TOX2	1	0	0	0	0	0	0	0	0	0	0	0
ENSMUSG00000074627	MROH8	0	0	0	0	0	0	1	0	0	0	0	0
ENSMUSG00000074629	4930518I15R	2	3	0	1	1	2	6	5	7	4	1	5
ENSMUSG00000074634	TMEM267	0	0	1	1	1	0	3	2	1	5	2	2
ENSMUSG00000074635	3110070M22	0	0	0	0	0	0	1	0	0	0	1	0
ENSMUSG00000074643	CPNE1	0	0	0	0	0	0	0	1	0	0	2	0
ENSMUSG00000074646	6430550D23F	2	0	0	1	2	0	1	1	1	1	1	2
ENSMUSG00000074649	BC029722	8	9	9	1	9	9	42	51	43	60	32	40
ENSMUSG00000074652	MYH7B	3	3	2	2	4	1	6	5	9	8	6	7
ENSMUSG00000074655	GM1527	11	11	11	11	7	11	19	21	21	29	18	19
ENSMUSG00000074656	EIF2S2	217	265	240	278	279	240	1042	1104	793	1085	844	878
ENSMUSG00000074657	KIF5A	0	0	0	0	0	1	1	1	0	1	0	0
ENSMUSG00000074678	DEFB25	0	0	0	0	0	0	1	0	0	0	0	0
ENSMUSG00000074695	IL22	0	0	0	0	0	0	0	0	0	0	0	1
ENSMUSG00000074698	CSNK2A1	14	25	27	25	22	22	111	125	107	140	85	111
ENSMUSG00000074715	CCL28	0	0	0	0	0	0	1	0	0	0	0	0
ENSMUSG00000074731	ZFP345	3	2	2	6	2	5	28	34	30	35	28	21
ENSMUSG00000074733	ZFP950	15	16	12	17	15	11	54	54	39	54	39	38
ENSMUSG00000074735	GM21994	0	0	0	0	0	0	1	1	0	0	1	0
ENSMUSG00000074738	FNDC10	1	1	0	2	1	0	4	1	5	2	3	8
ENSMUSG00000074746	PDZD8	14	11	13	10	10	14	42	42	42	56	40	39
ENSMUSG00000074748	ATXN7L3B	25	22	30	30	39	45	122	116	78	136	82	106
ENSMUSG00000074749	KIZ	2	2	4	2	1	3	19	14	7	19	13	10
ENSMUSG00000074754	SMIM26	7	13	9	12	6	9	44	49	37	37	46	50
ENSMUSG00000074771	ANKEF1	0	0	0	0	0	0	0	1	0	0	0	0
ENSMUSG00000074780	ANAPC15-PS	0	0	0	0	0	0	0	1	0	0	0	0
ENSMUSG00000074781	UBE2N	3	3	2	5	3	4	26	20	15	27	13	10
ENSMUSG00000074785	PLXNC1	0	7	2	2	6	7	19	14	16	14	16	11
ENSMUSG00000074793	HSPA12B	0	1	0	1	0	0	0	1	0	1	1	0
ENSMUSG00000074794	ARRDC3	0	3	1	3	1	5	7	18	9	16	9	15
ENSMUSG00000074797	ITPA	5	5	8	4	6	8	48	53	41	56	36	40
ENSMUSG00000074800	GM4149	24	43	27	43	25	27	76	98	95	92	71	101
ENSMUSG00000074802	GAS2L3	4	4	8	5	6	7	38	33	23	32	13	26
ENSMUSG00000074805	IL1BOS	0	0	0	0	0	0	0	0	0	1	0	0
ENSMUSG00000074807	GM10762	2	1	0	2	1	0	6	3	4	5	3	4
ENSMUSG00000074811	HPS6	2	1	4	1	4	4	10	15	13	11	13	15
ENSMUSG00000074817	PAPOLB	0	0	0	0	0	0	0	0	0	0	0	1
ENSMUSG00000074818	PDZD7	0	0	0	0	0	0	0	0	1	0	0	0
ENSMUSG00000074825	ITPRIPL1	14	11	8	19	14	6	21	31	22	36	19	26
ENSMUSG00000074826	GM10767	2	0	0	0	0	1	2	1	1	1	0	1
ENSMUSG00000074829	2010315B03F	1	0	1	0	3	4	6	3	1	10	5	5
ENSMUSG00000074862	BC025920	0	0	0	0	0	0	1	1	0	4	0	2
ENSMUSG00000074863	PLATR25	3	6	4	2	8	9	24	21	15	28	25	10
ENSMUSG00000074865	ZFP934	2	2	1	2	4	1	10	15	13	11	7	15
ENSMUSG00000074867	ZFP808	1	3	1	3	2	1	5	5	7	9	4	7
ENSMUSG00000074876	SPATA5L1	1	1	0	2	1	0	3	0	6	5	3	5
ENSMUSG00000074884	SERF2	5	13	5	9	7	13	63	69	33	75	57	47
ENSMUSG00000074886	GRK6	17	20	14	13	16	19	75	101	87	89	71	87
ENSMUSG00000074890	LCMT2	5	2	3	2	3	1	12	15	13	12	9	7
ENSMUSG00000074892	B3GALT5	6	2	6	1	3	0	9	17	16	7	8	9
ENSMUSG00000074899	SPTBN5	1	0	0	0	0	0	2	2	0	1	0	0
ENSMUSG00000074903	GM2058	0	0	0	0	0	0	1	0	0	0	0	0
ENSMUSG00000074909	RANBP6	7	8	12	8	12	13	27	38	33	52	40	30
ENSMUSG00000074912	GM14207	0	0	0	0	0	0	0	0	0	1	0	0
ENSMUSG00000074916	CHST14	0	0	1	1	1	0	2	0	0	0	0	4
ENSMUSG00000074918	INAFM2	7	14	13	17	10	15	51	69	42	53	29	49
ENSMUSG00000074922	FAM122A	5	4	3	3	6	3	13	12	5	2	8	7

ENSMUSG00000074923	PAK6	1	0	0	0	0	0	0	0	1	1	2	0
ENSMUSG00000074925	PTAR1	5	10	14	8	6	5	47	30	16	36	19	33
ENSMUSG00000074987	WT1OS	0	0	0	0	0	0	1	0	0	0	0	0
ENSMUSG00000074991	GABRR3	0	0	0	1	0	2	0	0	0	0	1	1
ENSMUSG00000074994	QSER1	7	7	11	12	11	11	47	50	38	61	49	41
ENSMUSG00000075000	NRBF2	0	0	1	0	1	0	1	3	2	4	0	1
ENSMUSG00000075014	GM10800	8	76	12	16	10	137	1772	280	25	1113	136	13
ENSMUSG00000075015	GM10801	2	8	2	4	1	10	156	27	7	116	12	2
ENSMUSG00000075023	ACCSL	0	0	0	0	0	0	0	0	1	0	0	0
ENSMUSG00000075027	4631405J19R	0	1	0	0	0	0	0	0	0	0	1	0
ENSMUSG00000075028	PRDM11	4	2	5	4	4	4	7	15	15	18	10	18
ENSMUSG00000075031	HIST1H2BB	1	0	0	0	0	0	1	0	1	2	1	0
ENSMUSG00000075033	NXPE3	9	2	2	6	3	7	10	14	12	28	12	16
ENSMUSG00000075040	ZFP408	3	3	5	3	0	3	18	16	14	11	11	9
ENSMUSG00000075042	4930431P03R	0	0	0	1	0	0	0	0	0	0	0	0
ENSMUSG00000075054	YAE1D1	1	0	1	1	1	1	6	4	4	9	2	8
ENSMUSG00000075093	OLFR1231	0	0	0	0	0	0	0	0	0	0	0	1
ENSMUSG00000075104	OLFR1219	0	0	0	0	0	1	0	0	0	0	0	0
ENSMUSG00000075121	OLFR1195	0	0	0	0	0	0	0	0	1	0	0	0
ENSMUSG00000075122	CD80	3	0	2	1	3	0	2	8	5	6	7	6
ENSMUSG00000075160	OLFR259	0	0	0	1	0	0	0	0	0	0	0	0
ENSMUSG00000075227	ZNHIT2	5	4	2	5	4	2	17	13	11	13	13	13
ENSMUSG00000075229	CCDC58	12	5	4	13	7	9	49	46	42	51	31	41
ENSMUSG00000075232	AMD1	3	4	3	2	1	2	20	18	10	15	17	12
ENSMUSG00000075254	HEG1	15	17	14	18	17	24	52	75	45	71	42	50
ENSMUSG00000075256	CERKL	1	3	3	6	6	3	11	14	17	16	17	15
ENSMUSG00000075266	CENPW	21	13	20	30	25	20	125	117	87	118	95	97
ENSMUSG00000075267	PJVK	0	1	0	0	0	0	0	0	0	0	0	0
ENSMUSG00000075269	BEX6	0	2	0	0	0	0	3	2	2	0	2	2
ENSMUSG00000075271	TTC30A1	3	2	1	0	0	1	9	5	3	12	10	4
ENSMUSG00000075273	TTC30B	0	3	0	2	0	0	6	6	2	3	2	2
ENSMUSG00000075276	GM13653	2	0	1	0	0	0	0	0	1	1	0	0
ENSMUSG00000075279	MRPL23-PS1	2	1	5	0	1	1	8	11	11	6	8	19
ENSMUSG00000075284	WIPF1	22	35	35	49	40	27	159	194	150	230	153	133
ENSMUSG00000075286	GM1968	0	0	0	0	0	0	0	1	0	0	0	0
ENSMUSG00000075288	GM6913	0	0	0	0	0	0	0	0	0	1	0	0
ENSMUSG00000075289	CARNS1	0	0	1	0	0	0	1	4	5	2	2	3
ENSMUSG00000075318	SCN2A	1	7	1	2	2	3	8	11	1	16	7	11
ENSMUSG00000075324	FIGN	0	0	0	0	1	1	0	0	1	0	0	0
ENSMUSG00000075327	ZBTB2	1	4	3	5	7	1	18	12	13	30	15	18
ENSMUSG00000075359	GM20775	0	0	0	0	1	0	0	0	0	0	0	0
ENSMUSG00000075370	IGLL1	0	0	0	1	0	1	0	0	0	1	1	0
ENSMUSG00000075376	RC3H2	10	21	19	24	17	17	84	99	58	77	53	58
ENSMUSG00000075377	OLFR362	1	0	0	0	0	0	0	0	0	0	0	0
ENSMUSG00000075389	2810410L24R	0	0	1	0	0	1	0	0	0	0	1	1
ENSMUSG00000075390	RPL36-PS8	0	0	0	0	0	0	0	1	0	0	0	1
ENSMUSG00000075394	HOXC4	1	0	3	2	0	0	5	7	4	5	3	4
ENSMUSG00000075408	6030408B16R	0	0	0	0	0	0	0	0	0	1	0	0
ENSMUSG00000075415	FNBP1	26	23	36	26	35	29	117	106	82	121	94	89
ENSMUSG00000075419	DOLK	1	2	1	4	3	4	11	12	6	9	10	13
ENSMUSG00000075425	GM13547	0	0	0	1	0	0	0	1	0	0	0	0
ENSMUSG00000075463	4930594M22	2	1	0	0	1	0	1	3	0	0	0	0
ENSMUSG00000075465	GM10837	16	21	25	24	28	26	51	48	61	66	57	64
ENSMUSG00000075467	DNLZ	2	4	9	8	6	10	40	55	34	39	36	40
ENSMUSG00000075470	DEAF1	6	6	6	11	4	13	50	28	27	21	20	18
ENSMUSG00000075480	GM10840	0	0	0	0	0	0	0	0	0	0	0	1
ENSMUSG00000075486	COMMD6	5	3	8	3	8	3	35	31	25	28	24	31
ENSMUSG00000075502	KBTBD6	2	0	1	0	3	0	8	5	4	5	4	1

ENSMUSG00000075511	1700001L05R	0	0	0	0	0	0	1	0	1	3	1	1
ENSMUSG00000075512	GM1587	0	0	1	0	0	0	0	0	0	0	1	0
ENSMUSG00000075514	GM13375	2	0	0	0	0	0	0	0	0	0	0	2
ENSMUSG00000075528	AARSD1	0	1	0	0	0	0	1	1	3	3	2	4
ENSMUSG00000075534	GM13262	0	0	0	1	1	0	1	3	1	1	1	1
ENSMUSG00000075553	GM5464	0	0	0	0	0	0	0	0	0	0	0	1
ENSMUSG00000075569	RSPH10B	0	0	1	1	1	0	1	4	1	5	1	4
ENSMUSG00000075573	DEFB47	0	0	0	0	0	0	0	1	0	0	0	0
ENSMUSG00000075576	GM12359	2	1	1	0	0	0	4	1	2	5	0	1
ENSMUSG00000075581	GM16409	1	0	0	0	0	0	0	0	0	1	1	0
ENSMUSG00000075587	GM11531	0	0	0	0	0	1	0	1	0	1	1	0
ENSMUSG00000075591	GM10874	1	0	0	1	0	0	2	2	3	1	1	0
ENSMUSG00000075595	ZFP652	4	9	3	9	9	12	44	30	34	55	26	33
ENSMUSG00000075600	ZC3H3	0	0	0	0	1	3	5	6	2	2	3	1
ENSMUSG00000075700	SELENOT	28	41	40	54	48	43	182	200	141	190	135	164
ENSMUSG00000075701	SELENOS	7	17	19	28	22	16	134	126	83	111	80	91
ENSMUSG00000075703	SELENOI	11	12	11	26	25	19	48	54	41	54	44	57
ENSMUSG00000075704	TXNRD2	3	6	3	3	3	2	10	14	13	9	11	11
ENSMUSG00000075705	MSRB1	2	3	3	4	0	1	14	7	5	10	5	10
ENSMUSG00000075706	GPX4	2	3	3	4	3	3	28	27	20	24	22	23
ENSMUSG00000076039	GM22137	0	0	0	0	0	1	0	0	0	0	0	0
ENSMUSG00000076060	MIR704	0	0	0	0	0	0	0	0	0	0	0	1
ENSMUSG00000076135	GM24276	0	0	0	0	0	0	2	0	2	1	2	0
ENSMUSG00000076136	GM24951	2	2	0	3	4	2	26	33	21	30	16	20
ENSMUSG00000076137	GM26384	1	3	1	1	0	0	3	4	4	2	6	5
ENSMUSG00000076258	GM23935	31	18	31	30	19	20	96	96	71	114	91	85
ENSMUSG00000076281	GM24270	0	0	0	0	0	0	0	0	0	1	0	0
ENSMUSG00000076294	GM26154	0	0	0	0	0	0	0	0	1	0	0	0
ENSMUSG00000076333	MIR599	0	0	0	0	0	0	0	0	1	0	0	0
ENSMUSG00000076364	GM24049	1	0	0	0	0	0	1	0	1	1	0	1
ENSMUSG00000076370	GM22369	0	0	0	0	0	0	0	0	0	1	0	0
ENSMUSG00000076376	MIR674	0	0	0	0	0	0	1	0	0	0	0	0
ENSMUSG00000076430	HUS1B	0	0	0	0	0	0	0	0	0	1	0	0
ENSMUSG00000076432	YWHAQ	9	6	6	9	11	14	79	66	40	121	42	58
ENSMUSG00000076435	ACSF2	0	3	1	1	1	1	9	7	7	9	12	7
ENSMUSG00000076437	SELENOH	14	21	14	26	37	24	128	133	106	140	102	108
ENSMUSG00000076444	MIR761	0	1	0	0	0	0	0	0	0	0	0	0
ENSMUSG00000076454	MIR762	0	0	0	0	0	0	1	0	0	0	0	0
ENSMUSG00000076469	TRBV13-2	0	1	0	2	0	0	4	3	5	4	6	2
ENSMUSG00000076497	TRBJ2-7	0	0	0	0	0	0	1	0	1	1	0	0
ENSMUSG00000076498	TRBC2	104	130	139	133	109	122	867	886	666	876	620	834
ENSMUSG00000076617	IGHM	37	63	71	64	76	74	344	400	318	401	293	330
ENSMUSG00000076709	IGHV1-47	0	0	0	0	0	0	2	0	0	0	0	0
ENSMUSG00000076731	IGHV8-12	0	0	0	0	0	0	0	0	0	0	1	0
ENSMUSG00000076749	TCRG-C1	0	1	0	0	1	0	0	0	0	0	0	0
ENSMUSG00000076911	TRAJ18	0	0	0	0	1	0	0	1	0	0	0	0
ENSMUSG00000076912	TRAJ17	0	0	0	0	0	0	0	1	0	0	0	0
ENSMUSG00000076913	TRAJ16	0	0	0	1	0	0	0	0	0	0	0	0
ENSMUSG00000076914	TRAJ15	0	0	0	0	0	0	1	0	0	0	0	0
ENSMUSG00000076915	TRAJ14	0	0	0	0	0	0	0	1	0	0	0	0
ENSMUSG00000076917	TRAJ12	0	0	0	0	0	0	0	0	0	1	0	0
ENSMUSG00000076919	TRAJ9	0	0	0	0	0	0	0	0	0	0	0	1
ENSMUSG00000076924	TRAJ4	0	0	0	0	0	0	0	0	0	1	0	0
ENSMUSG00000076928	TRAC	57	81	44	77	75	63	295	329	243	260	209	260
ENSMUSG00000077082	GM23766	0	0	2	0	0	0	0	0	0	0	0	0
ENSMUSG00000077148	GM22935	0	0	0	0	0	0	0	0	1	0	0	1
ENSMUSG00000077167	GM24119	0	0	0	0	0	0	0	0	0	1	0	0
ENSMUSG00000077192	SNORA17	0	0	0	0	0	0	2	0	1	0	0	0

ENSMUSG00000077222	GM22270	0	0	0	0	0	0	1	0	0	1	0	0
ENSMUSG00000077227	GM22272	0	0	1	0	0	0	0	0	0	0	0	0
ENSMUSG00000077274	GM22786	0	0	1	0	0	0	0	0	0	1	0	0
ENSMUSG00000077285	GM26394	0	0	0	0	0	0	1	0	0	0	1	0
ENSMUSG00000077323	RNU11	0	0	0	0	0	1	0	0	0	1	1	0
ENSMUSG00000077345	SNORD70	0	0	0	0	0	0	0	0	1	0	1	0
ENSMUSG00000077380	GM22661	0	0	0	0	0	0	1	0	0	0	0	0
ENSMUSG00000077385	GM22657	1	0	0	0	0	0	0	0	0	0	0	0
ENSMUSG00000077391	GM24336	0	0	0	0	0	0	0	2	0	0	1	0
ENSMUSG00000077393	GM24338	0	0	0	0	0	0	0	1	0	0	0	0
ENSMUSG00000077394	GM24339	0	0	1	0	0	0	0	0	0	0	0	0
ENSMUSG00000077403	GM25419	0	0	0	0	0	0	1	0	0	0	1	0
ENSMUSG00000077450	RAB11B	3	7	6	6	8	7	21	16	21	26	22	19
ENSMUSG00000077457	SNORD65	0	2	0	0	0	0	4	4	3	2	2	0
ENSMUSG00000077505	GM24233	0	0	0	1	0	0	1	2	1	2	0	1
ENSMUSG00000077506	SCARNA9	0	0	0	0	0	0	0	0	0	0	1	0
ENSMUSG00000077565	GM23201	0	0	0	0	0	0	1	0	1	0	0	0
ENSMUSG00000077575	GM24888	1	0	0	0	0	0	0	0	0	0	0	0
ENSMUSG00000077611	GM23946	0	0	0	0	0	0	0	0	0	1	0	0
ENSMUSG00000077625	SNORD4A	0	0	0	0	0	0	0	1	0	1	0	1
ENSMUSG00000077637	GM22771	0	0	0	0	0	0	0	0	0	1	0	0
ENSMUSG00000077649	GM24989	0	0	0	0	0	1	0	0	0	0	0	0
ENSMUSG00000077711	AF357399	0	1	0	1	0	0	0	0	0	0	0	0
ENSMUSG00000077734	SNORD83B	0	0	0	0	0	0	0	0	0	0	1	2
ENSMUSG00000077737	SNORD72	0	0	0	0	0	0	0	3	2	2	0	1
ENSMUSG00000077758	GM23527	0	0	0	0	0	1	0	0	0	0	0	0
ENSMUSG00000078087	RPS12L1	1	1	0	1	0	1	3	3	2	1	4	0
ENSMUSG00000078126	RPL23A-PS3	0	0	0	0	0	0	0	3	0	0	1	0
ENSMUSG00000078128	GM2178	0	0	0	0	0	0	0	0	0	1	0	0
ENSMUSG00000078134	GM12355	3	4	5	5	0	6	5	11	7	11	10	15
ENSMUSG00000078139	AK157302	0	0	0	0	0	0	1	0	0	0	0	1
ENSMUSG00000078143	GM17344	0	1	0	0	1	0	1	3	3	3	1	2
ENSMUSG00000078144	CAPNS2	0	0	1	0	0	0	0	0	0	0	0	0
ENSMUSG00000078153	PSME2B	2	2	2	2	1	5	9	19	4	12	8	5
ENSMUSG00000078154	GM12184	2	5	5	2	5	4	7	20	9	24	11	14
ENSMUSG00000078157	4931440F15F	0	0	0	0	1	0	0	0	0	0	1	0
ENSMUSG00000078161	ERICH3	0	1	0	0	0	0	0	0	0	0	0	1
ENSMUSG00000078183	GM15610	0	0	0	1	0	0	1	0	0	0	0	0
ENSMUSG00000078184	RBM8A2	0	4	0	1	1	0	4	5	1	5	3	5
ENSMUSG00000078185	CHML	1	2	2	0	4	0	7	8	14	10	6	5
ENSMUSG00000078192	GM7290	0	0	1	0	2	1	4	2	4	2	5	3
ENSMUSG00000078193	GM2000	126	165	168	156	139	167	458	519	507	453	418	441
ENSMUSG00000078201	TMEM203	0	2	2	5	2	2	5	12	9	8	9	9
ENSMUSG00000078238	GM12854	0	0	0	0	0	0	1	0	0	0	0	0
ENSMUSG00000078240	GM3550	9	13	19	19	17	20	57	43	37	53	37	38
ENSMUSG00000078247	AIRN	3	5	5	3	4	7	13	20	15	14	14	12
ENSMUSG00000078249	HMGA1B	1	2	4	4	1	6	5	15	7	13	7	10
ENSMUSG00000078289	GM10923	0	0	0	0	0	0	0	0	0	0	0	1
ENSMUSG00000078291	GM3940	0	0	0	0	0	0	0	3	2	1	0	1
ENSMUSG00000078317	F8A	1	0	0	1	0	4	7	4	5	7	2	13
ENSMUSG00000078348	SF3B5	7	8	8	16	11	6	45	61	52	69	46	48
ENSMUSG00000078350	SMIM1	0	1	0	0	2	0	1	2	3	4	2	1
ENSMUSG00000078370	GM6316	0	0	0	0	0	1	2	1	2	0	0	0
ENSMUSG00000078377	GM4294	0	0	0	0	0	0	0	0	1	0	2	0
ENSMUSG00000078427	SARNP	1	6	12	8	10	5	78	77	47	76	30	61
ENSMUSG00000078429	CTDSP2	2	7	5	6	2	3	17	27	11	28	14	21
ENSMUSG00000078435	AU041133	1	2	4	0	1	2	2	5	2	6	1	3
ENSMUSG00000078439	SMIM24	0	0	0	0	1	0	0	1	0	0	0	0

ENSMUSG00000078440	DOHH	11	11	13	14	11	13	55	43	53	59	41	59
ENSMUSG00000078451	PPIL6	0	0	0	0	0	0	2	1	0	0	1	0
ENSMUSG00000078453	ABRACL	7	13	8	17	13	12	104	97	52	80	63	76
ENSMUSG00000078485	PLEKHN1	0	1	3	3	2	1	4	6	1	10	2	8
ENSMUSG00000078490	CFAP74	1	1	0	0	1	4	5	7	9	10	5	7
ENSMUSG00000078495	ZFP984	2	0	2	3	0	0	15	11	2	16	8	11
ENSMUSG00000078515	DDI2	11	11	15	14	18	18	63	63	41	75	38	56
ENSMUSG00000078517	EMC1	6	6	6	6	12	6	39	32	23	38	14	36
ENSMUSG00000078521	AUNIP	2	2	2	3	4	5	18	11	7	12	14	15
ENSMUSG00000078537	B020031M17	0	0	0	0	0	1	0	0	0	2	0	1
ENSMUSG00000078546	ZFP995	2	2	3	6	4	4	13	17	12	11	14	10
ENSMUSG00000078552	DCDC2B	1	1	1	2	0	0	5	4	4	9	4	7
ENSMUSG00000078554	FAM229A	0	0	1	0	0	0	0	0	1	1	1	0
ENSMUSG00000078566	BNIP3	0	1	1	0	1	0	14	9	11	15	10	13
ENSMUSG00000078570	1110065P20R	1	6	4	2	11	2	25	24	17	17	25	30
ENSMUSG00000078572	NDUFAF8	11	6	6	9	14	6	39	37	24	37	33	29
ENSMUSG00000078578	UBE2D3	41	55	47	58	41	59	410	434	300	392	283	302
ENSMUSG00000078580	E430018J23R	0	0	0	0	1	2	2	3	2	2	3	3
ENSMUSG00000078584	AU022252	6	2	1	1	5	3	16	14	8	21	5	12
ENSMUSG00000078588	CCDC24	3	3	1	1	2	2	8	4	6	6	5	4
ENSMUSG00000078598	SKINT5	0	0	0	0	0	0	0	0	0	0	1	1
ENSMUSG00000078606	GM4070	0	0	0	0	0	0	0	0	1	0	0	1
ENSMUSG00000078607	1810010H24R	0	0	0	0	0	0	0	0	1	0	1	0
ENSMUSG00000078611	GM5901	0	0	0	1	0	0	0	0	0	0	0	0
ENSMUSG00000078616	TRIM30C	0	0	0	0	0	0	1	0	0	0	0	0
ENSMUSG00000078619	SMARCD2	0	4	3	4	2	6	24	25	10	20	12	20
ENSMUSG00000078622	CCDC47	8	11	18	18	10	18	76	102	65	101	55	60
ENSMUSG00000078630	TOMT	1	1	1	1	2	1	5	3	0	7	3	7
ENSMUSG00000078635	1700028N14R	0	0	0	0	0	0	0	0	1	0	0	0
ENSMUSG00000078636	GM7336	10	8	10	15	10	7	35	39	29	37	44	47
ENSMUSG00000078650	G6PC	0	0	0	0	0	0	0	1	0	0	0	0
ENSMUSG00000078651	AOC2	1	1	0	1	2	1	8	8	7	7	4	3
ENSMUSG00000078652	PSME3	8	18	12	17	16	19	56	81	68	83	54	79
ENSMUSG00000078653	CNTD1	7	12	11	13	9	9	45	40	43	39	32	58
ENSMUSG00000078656	VPS25	1	1	0	0	0	1	0	2	0	2	1	0
ENSMUSG00000078671	CHD2	12	27	25	30	27	27	128	105	68	109	70	77
ENSMUSG00000078676	CASC3	6	11	7	5	13	7	47	38	20	50	28	35
ENSMUSG00000078681	TM2D3	2	2	3	3	0	3	6	10	6	14	9	9
ENSMUSG00000078684	5830417I10R	0	0	0	0	0	0	1	1	0	2	5	3
ENSMUSG00000078695	CISD3	4	4	2	2	4	2	22	21	10	14	17	24
ENSMUSG00000078700	D030028A08R	2	7	2	8	2	4	26	19	13	31	13	19
ENSMUSG00000078713	TOMM5	3	9	4	9	10	12	65	68	44	67	54	58
ENSMUSG00000078716	TMEM8B	1	0	0	1	0	0	0	2	0	1	1	4
ENSMUSG00000078737	GM2128	0	0	0	0	0	0	0	1	0	0	0	0
ENSMUSG00000078762	HAUS5	0	7	3	5	6	5	23	32	19	29	17	21
ENSMUSG00000078763	SLFN1	0	0	0	0	0	0	0	0	0	0	1	0
ENSMUSG00000078768	ZFP566	1	0	1	2	4	2	12	12	14	11	9	6
ENSMUSG00000078771	EVI2A	1	0	2	0	0	1	3	1	0	2	0	3
ENSMUSG00000078773	RAD54B	3	2	4	10	4	2	17	21	16	27	17	21
ENSMUSG00000078779	ZFP59	0	1	0	0	0	0	1	1	0	0	0	1
ENSMUSG00000078784	1810022K09R	22	23	26	23	28	35	210	214	170	221	166	161
ENSMUSG00000078786	BC024978	3	0	2	1	0	0	3	2	3	5	2	1
ENSMUSG00000078787	CYP2T4	0	0	1	0	1	0	0	0	0	0	0	1
ENSMUSG00000078794	DACT3	3	2	2	3	1	6	11	4	8	9	5	5
ENSMUSG00000078812	EIF5A	73	73	66	100	76	65	477	525	375	485	370	444
ENSMUSG00000078813	LENG1	3	5	4	2	5	6	22	21	17	25	20	17
ENSMUSG00000078838	GM17382	0	0	0	0	0	1	0	0	0	0	0	0
ENSMUSG00000078851	HIST3H2A	16	14	11	9	11	18	66	57	47	71	50	45

ENSMUSG00000078853	IGTP	0	1	0	0	1	0	2	4	0	3	1	5
ENSMUSG00000078861	ZFP931	1	4	1	0	1	1	7	9	3	10	9	5
ENSMUSG00000078862	GM14326	1	0	0	0	0	0	2	1	1	3	2	2
ENSMUSG00000078864	GM14322	0	0	0	0	0	0	1	0	0	0	0	1
ENSMUSG00000078866	ZFP970	0	0	0	0	0	0	0	1	0	2	0	0
ENSMUSG00000078867	GM14418	0	0	0	0	0	0	0	0	0	1	1	1
ENSMUSG00000078868	GM14412	0	4	0	0	3	1	3	4	3	7	0	2
ENSMUSG00000078869	GM14409	1	0	0	0	1	1	4	3	1	0	2	1
ENSMUSG00000078870	GM14410	0	0	0	0	0	0	1	0	0	2	0	1
ENSMUSG00000078872	GM14401	1	0	0	0	0	0	1	2	1	2	0	0
ENSMUSG00000078876	GM14408	0	0	0	0	0	0	1	0	0	0	0	0
ENSMUSG00000078877	GM14295	1	1	1	1	1	1	2	5	3	6	2	3
ENSMUSG00000078878	GM14305	0	1	0	0	3	0	2	1	2	3	1	2
ENSMUSG00000078902	GM14443	3	1	0	0	1	0	2	3	0	2	0	1
ENSMUSG00000078906	GM14444	0	0	0	0	0	0	0	1	0	0	0	0
ENSMUSG00000078908	MON1B	1	1	0	2	1	0	9	11	4	3	2	3
ENSMUSG00000078915	HSP25-PS1	0	0	0	1	0	0	0	0	0	0	0	0
ENSMUSG00000078917	GM11281	0	0	0	0	1	1	0	0	0	0	0	0
ENSMUSG00000078919	DPM1	0	1	5	3	2	4	11	9	5	18	8	8
ENSMUSG00000078923	UBE2V1	0	0	0	0	0	0	0	1	0	0	0	1
ENSMUSG00000078926	CDC20B	0	0	0	0	0	0	0	0	0	0	0	1
ENSMUSG00000078934	9230113P08F	0	0	0	0	0	0	0	2	0	0	0	0
ENSMUSG00000078941	AK6	7	8	6	9	5	5	57	40	31	46	46	38
ENSMUSG00000078942	NAIP6	0	1	0	0	0	0	0	0	0	0	0	1
ENSMUSG00000078945	NAIP2	3	1	3	0	2	2	10	9	7	4	4	3
ENSMUSG00000078970	WDR92	1	1	1	4	5	3	14	28	15	19	20	14
ENSMUSG00000078974	SEC61G	0	4	0	2	1	1	13	10	5	7	5	10
ENSMUSG00000078994	ZFP429	1	0	0	0	1	1	6	2	3	3	6	4
ENSMUSG00000078995	ZFP456	2	0	1	2	0	1	2	3	0	3	0	4
ENSMUSG00000079003	SAMD1	5	7	10	8	7	3	34	34	24	38	25	37
ENSMUSG00000079008	GM14124	0	0	0	0	0	0	0	1	0	0	0	0
ENSMUSG00000079009	GM14139	1	1	2	2	2	2	6	9	6	12	4	5
ENSMUSG00000079016	GM11034	0	0	0	0	1	0	0	0	0	0	0	0
ENSMUSG00000079017	IFI27L2A	1	1	0	1	0	0	1	1	1	2	0	0
ENSMUSG00000079018	LY6C1	0	0	0	0	0	0	0	0	1	0	1	1
ENSMUSG00000079020	SLC45A4	2	2	0	1	3	1	7	6	3	8	5	3
ENSMUSG00000079033	MEF2B	0	0	0	0	0	0	0	1	0	0	0	0
ENSMUSG00000079036	ALKBH1	11	5	11	9	16	17	56	54	32	53	30	44
ENSMUSG00000079038	D130040H23I	1	3	2	8	6	10	21	14	9	31	15	24
ENSMUSG00000079043	FASTKD5	2	1	0	0	0	1	5	6	2	8	4	2
ENSMUSG00000079065	BC005561	25	40	28	29	39	34	139	137	104	154	107	100
ENSMUSG00000079069	8430423G03I	0	1	0	0	0	0	0	0	0	0	0	0
ENSMUSG00000079071	GM14085	0	0	0	1	1	0	2	1	0	1	1	3
ENSMUSG00000079083	JRKL	9	5	7	8	3	7	29	34	38	33	29	37
ENSMUSG00000079084	CCDC82	14	4	9	8	7	7	33	56	26	51	32	32
ENSMUSG00000079104	PRPS1L3	1	0	2	1	1	4	14	8	8	15	5	8
ENSMUSG00000079108	SRP54B	0	1	0	1	0	0	0	2	1	0	2	0
ENSMUSG00000079109	PMS2	1	5	8	6	8	2	27	25	19	23	13	29
ENSMUSG00000079110	CAPN3	11	13	12	10	18	15	112	117	84	104	81	99
ENSMUSG00000079111	KDELR2	39	49	42	49	63	47	264	294	212	266	204	226
ENSMUSG00000079137	RPL27-PS1	1	0	0	0	1	0	2	1	0	0	1	0
ENSMUSG00000079139	GM4204	1	2	3	0	2	0	7	7	3	9	4	9
ENSMUSG00000079144	A130010J15R	0	10	2	3	5	2	17	16	16	15	11	20
ENSMUSG00000079173	ZAN	0	0	1	0	1	0	1	0	0	0	1	0
ENSMUSG00000079177	FAM228A	0	4	1	0	2	3	2	2	6	10	4	6
ENSMUSG00000079179	RAB10OS	2	7	5	4	4	5	33	24	19	14	21	17
ENSMUSG00000079184	MPHOSPH8	51	58	59	62	64	55	187	214	161	254	174	168
ENSMUSG00000079197	PSME2	8	18	11	13	19	13	108	120	81	105	83	86

ENSMUSG00000079215	ZFP664	2	8	7	9	10	10	46	39	28	46	39	22
ENSMUSG00000079224	GM6565	0	0	0	0	0	0	0	2	0	1	0	0
ENSMUSG00000079225	GM9531	10	10	9	21	11	13	47	39	28	43	28	30
ENSMUSG00000079227	CCR5	0	0	0	0	0	0	0	0	0	0	1	0
ENSMUSG00000079235	CCDC13	0	0	1	0	0	0	0	1	0	0	0	0
ENSMUSG00000079242	C730034F03F	0	0	0	0	0	0	1	1	1	1	2	1
ENSMUSG00000079259	TRIM71	0	0	0	0	0	0	0	0	0	0	1	0
ENSMUSG00000079260	TMPPE	2	0	1	0	1	0	3	2	1	2	0	1
ENSMUSG00000079277	HOXD3	0	0	0	0	0	0	1	0	0	0	0	1
ENSMUSG00000079283	2310009B15F	0	2	3	4	3	1	9	11	8	19	10	14
ENSMUSG00000079297	GM2223	0	0	0	0	0	0	0	0	0	0	0	2
ENSMUSG00000079298	KLRB1B	0	0	0	0	0	0	2	1	0	1	0	1
ENSMUSG00000079299	KLRB1	0	0	0	0	0	0	1	0	0	0	0	0
ENSMUSG00000079304	4933413G19I	0	0	0	0	0	0	0	0	0	1	0	0
ENSMUSG00000079316	RAB9	4	8	11	8	18	12	58	79	53	61	51	51
ENSMUSG00000079317	TRAPPC2	0	1	0	0	1	0	1	3	1	2	1	2
ENSMUSG00000079334	NAT6	0	0	0	0	1	1	2	7	2	3	1	3
ENSMUSG00000079357	GM11100	0	4	1	5	12	2	35	31	23	40	30	20
ENSMUSG00000079363	GBP4	3	9	6	11	9	11	42	55	32	56	32	26
ENSMUSG00000079414	GM11110	0	2	0	0	0	1	2	2	3	11	5	5
ENSMUSG00000079415	CNTF	0	0	0	1	0	0	0	0	0	0	0	0
ENSMUSG00000079418	ATG4A	0	3	1	0	0	0	11	22	5	26	11	13
ENSMUSG00000079419	MS4A6C	0	0	0	0	0	0	3	0	0	0	0	0
ENSMUSG00000079426	ARPC4	8	17	10	20	13	16	58	50	45	72	50	71
ENSMUSG00000079427	MTHFSL	0	0	0	2	0	1	1	4	3	4	4	3
ENSMUSG00000079429	MROH2A	0	0	0	1	0	0	1	5	0	2	2	1
ENSMUSG00000079435	RPL36A	83	108	96	98	112	115	494	581	427	538	379	532
ENSMUSG00000079436	KCNJ13	0	0	0	0	0	0	1	1	0	0	0	0
ENSMUSG00000079442	ST6GALNAC4	4	7	6	4	6	2	24	29	21	28	16	29
ENSMUSG00000079467	GM14966	0	0	0	1	0	1	0	1	2	0	3	1
ENSMUSG00000079469	PIGB	1	3	1	0	3	2	5	5	4	12	5	11
ENSMUSG00000079470	UTP14B	2	1	3	4	6	9	34	31	18	48	20	22
ENSMUSG00000079477	RAB7	13	14	22	20	24	20	123	103	71	121	99	119
ENSMUSG00000079478	SSSCA1	0	0	0	0	0	0	1	0	0	0	0	0
ENSMUSG00000079480	PIN4	1	2	5	4	1	4	27	35	20	21	15	22
ENSMUSG00000079481	NHSL2	0	0	0	0	1	0	0	1	1	0	0	0
ENSMUSG00000079487	MED12	0	2	1	1	1	1	6	6	7	5	5	7
ENSMUSG00000079491	H2-T10	1	1	1	1	3	3	7	6	4	9	5	3
ENSMUSG00000079499	6530402F18F	2	5	3	5	0	2	6	6	7	11	6	6
ENSMUSG00000079502	CFAP77	1	1	0	0	0	0	1	1	0	1	1	0
ENSMUSG00000079505	GM11131	0	0	2	3	1	1	3	3	1	2	1	4
ENSMUSG00000079508	APOO	0	0	3	0	1	1	19	15	7	16	8	10
ENSMUSG00000079509	ZFX	9	5	5	11	12	13	32	51	31	78	34	34
ENSMUSG00000079511	GM42688	0	0	0	0	0	0	0	0	0	0	1	0
ENSMUSG00000079523	TMSB10	60	56	58	79	60	70	655	675	429	669	455	487
ENSMUSG00000079547	H2-DMB1	0	0	0	0	0	0	0	2	1	1	0	0
ENSMUSG00000079550	MPP4	0	0	1	0	0	0	2	0	0	0	0	1
ENSMUSG00000079553	KIFC1	9	8	8	16	14	16	57	67	50	64	51	50
ENSMUSG00000079555	HAUS3	26	23	18	45	29	28	133	176	133	156	115	124
ENSMUSG00000079557	MARCH2	3	3	5	3	1	1	16	18	12	7	13	12
ENSMUSG00000079559	COLCA2	0	1	1	1	0	0	0	0	0	2	0	0
ENSMUSG00000079562	MAEA	8	5	14	9	9	16	56	72	47	59	56	39
ENSMUSG00000079563	PGLYRP2	3	4	12	9	7	5	18	14	16	16	11	21
ENSMUSG00000079597	GM5483	0	0	0	0	0	1	0	0	0	0	0	0
ENSMUSG00000079598	CLEC2L	0	0	0	0	0	1	0	0	0	1	0	0
ENSMUSG00000079602	9230102O04I	1	0	0	0	0	0	0	1	1	1	2	2
ENSMUSG00000079603	GM13218	0	1	0	0	2	1	4	4	1	4	1	1
ENSMUSG00000079605	ZBTB9	1	0	0	2	0	0	1	5	3	0	4	2

ENSMUSG00000079608	STARD6	1	0	0	0	0	0	0	2	3	1	0	0
ENSMUSG00000079610	ANKRD39	6	3	2	3	5	5	15	16	11	17	11	4
ENSMUSG00000079614	SEH1L	4	5	9	8	6	5	30	36	25	38	26	28
ENSMUSG00000079626	RHOX7B	0	0	0	0	0	2	1	0	0	0	0	0
ENSMUSG00000079627	RHOX2H	0	0	0	0	0	0	0	1	2	0	0	0
ENSMUSG00000079635	RHOX4C	0	0	0	0	1	0	0	0	0	0	1	0
ENSMUSG00000079641	RPL39	79	87	79	109	88	94	587	569	446	544	472	507
ENSMUSG00000079644	GM1110	0	0	0	0	0	0	0	0	0	3	1	1
ENSMUSG00000079652	FAM71F2	0	0	0	0	0	0	0	0	1	0	1	0
ENSMUSG00000079657	RAB26	0	0	0	0	0	0	1	1	0	0	0	0
ENSMUSG00000079658	ELOC	17	25	38	38	41	30	222	282	166	221	175	216
ENSMUSG00000079659	TMEM243	41	45	37	42	43	51	241	219	199	216	162	199
ENSMUSG00000079671	2610203C22F	0	0	0	0	0	1	1	0	0	1	0	0
ENSMUSG00000079685	ULBP1	12	26	21	26	20	18	73	83	55	93	50	60
ENSMUSG00000079694	GM14862	0	0	0	0	0	0	0	1	0	0	0	0
ENSMUSG00000079698	VMN2R93	0	0	0	0	0	0	0	0	0	0	1	0
ENSMUSG00000079737	3110001I22R	0	2	1	1	0	2	3	4	5	3	2	2
ENSMUSG00000079808	AC168977.1	0	1	0	0	1	0	1	0	0	0	0	0
ENSMUSG00000079845	XLR4A	9	2	4	3	6	3	28	36	21	27	23	20
ENSMUSG00000079852	KLRA4	0	0	0	0	0	0	0	2	0	0	0	0
ENSMUSG00000079853	KLRA1	1	0	0	0	0	0	0	1	0	1	1	2
ENSMUSG00000079884	GM10698	0	0	1	0	0	0	0	0	0	0	0	2
ENSMUSG00000079915	GM16073	0	0	0	0	0	1	0	0	0	0	0	0
ENSMUSG00000079941	GM11273	0	0	0	0	1	1	1	1	1	0	1	0
ENSMUSG00000079942	RPL28-PS3	0	1	3	0	2	3	6	4	7	3	3	6
ENSMUSG00000079962	GM12643	0	0	0	0	0	0	0	0	1	0	0	0
ENSMUSG00000079965	GM14853	0	0	1	0	0	0	0	1	0	0	2	1
ENSMUSG00000079988	GM11970	0	0	0	0	0	0	1	0	0	1	0	1
ENSMUSG00000080006	RPS19-PS7	0	0	0	0	0	1	0	2	0	2	1	0
ENSMUSG00000080021	GM5915	0	2	1	1	2	0	1	2	2	2	2	2
ENSMUSG00000080054	RPL12-PS1	2	4	5	3	3	1	10	7	8	6	5	13
ENSMUSG00000080058	GM11175	0	0	0	0	0	0	2	0	0	1	1	0
ENSMUSG00000080059	RPS19-PS3	6	4	7	3	7	5	28	17	25	16	16	16
ENSMUSG00000080115	METTL21B	0	0	0	0	0	0	1	2	0	1	2	0
ENSMUSG00000080180	GM17235	0	0	0	0	0	0	0	0	0	0	0	1
ENSMUSG00000080198	GM13573	0	0	0	0	0	0	0	0	1	0	2	0
ENSMUSG00000080237	GM14239	0	1	0	3	0	0	0	3	3	4	1	2
ENSMUSG00000080268	BRMS1	0	0	0	0	0	0	0	0	0	0	1	0
ENSMUSG00000080316	SPACA6	0	1	0	0	1	0	2	4	3	5	2	1
ENSMUSG00000080374	GM22953	0	0	2	1	1	0	1	1	4	3	1	0
ENSMUSG00000080440	GM25848	0	0	0	1	0	0	0	0	0	0	0	0
ENSMUSG00000080455	GM24194	0	0	0	1	0	0	0	0	0	1	0	0
ENSMUSG00000080468	MIR1195	0	0	0	0	2	0	0	0	0	1	0	2
ENSMUSG00000080469	SNORD98	0	0	0	0	0	0	0	0	0	0	0	1
ENSMUSG00000080478	SNORD23	0	0	0	0	0	0	0	0	0	0	1	0
ENSMUSG00000080485	GM23493	1	0	0	0	0	0	1	0	0	2	0	0
ENSMUSG00000080538	GM25541	0	0	0	0	1	0	0	0	0	2	1	0
ENSMUSG00000080544	GM22714	1	0	0	0	0	0	0	1	0	2	1	1
ENSMUSG00000080610	SNORD110	0	0	0	0	0	0	0	1	0	0	1	0
ENSMUSG00000080704	GM6031	0	0	0	0	0	0	0	0	0	1	0	0
ENSMUSG00000080710	GM8107	0	0	0	0	1	0	1	0	0	1	0	0
ENSMUSG00000080712	HIST3H2BB-P	0	1	1	0	0	1	0	0	0	0	0	0
ENSMUSG00000080717	B230307C23F	0	10	7	10	11	6	51	59	36	45	34	37
ENSMUSG00000080718	GM8019	0	0	0	0	0	0	0	1	0	0	0	0
ENSMUSG00000080719	GM15532	0	0	0	0	1	0	3	3	1	2	4	2
ENSMUSG00000080727	C920021L13F	1	1	1	1	2	0	2	3	2	3	6	3
ENSMUSG00000080731	RPS2-PS9	0	0	0	0	0	0	0	0	1	0	1	0
ENSMUSG00000080746	RPSA-PS12	0	0	1	0	1	1	2	1	2	2	1	1

ENSMUSG00000080747	GM14016	0	0	0	0	0	0	0	0	1	0	0	0
ENSMUSG00000080748	GM15661	0	0	0	0	1	0	1	0	0	0	0	0
ENSMUSG00000080763	GM12341	0	1	0	0	0	0	0	1	0	0	0	0
ENSMUSG00000080772	GM12543	0	0	1	0	0	0	0	0	0	0	0	0
ENSMUSG00000080775	GM6368	0	0	1	1	0	0	1	1	0	1	4	2
ENSMUSG00000080776	GM12174	0	1	0	0	0	0	0	0	0	0	0	0
ENSMUSG00000080778	GM7199	0	0	0	0	0	0	0	1	0	0	0	0
ENSMUSG00000080779	GM8731	0	0	1	1	0	0	0	1	0	1	0	0
ENSMUSG00000080782	GM13495	0	0	0	0	0	0	0	0	0	0	0	1
ENSMUSG00000080783	GM8250	0	0	0	0	0	0	1	0	0	0	0	0
ENSMUSG00000080797	GM15760	0	0	0	1	0	0	0	1	0	3	1	1
ENSMUSG00000080810	GM11737	0	0	0	0	0	0	1	2	1	1	0	1
ENSMUSG00000080811	GM14513	0	0	0	1	1	0	0	0	0	1	1	0
ENSMUSG00000080816	GM12447	0	0	0	0	0	0	0	1	0	0	0	1
ENSMUSG00000080823	ATP5L-PS2	0	0	0	0	1	0	0	0	0	3	0	1
ENSMUSG00000080824	GM9001	0	0	0	0	0	0	0	2	2	2	1	2
ENSMUSG00000080831	GM12529	0	0	0	0	0	0	0	0	1	0	0	1
ENSMUSG00000080836	GM6444	0	0	0	0	1	0	1	0	1	0	0	0
ENSMUSG00000080839	GM11625	0	0	0	0	0	0	0	0	0	1	1	0
ENSMUSG00000080840	GM11349	0	0	0	0	0	0	0	0	0	0	0	1
ENSMUSG00000080845	GM9115	0	0	0	0	0	1	0	0	0	0	0	0
ENSMUSG00000080848	GM9385	4	14	12	14	14	18	32	39	32	35	37	51
ENSMUSG00000080851	GM12094	0	0	0	0	0	0	0	1	0	0	0	0
ENSMUSG00000080855	RPL35-PS1	0	0	0	0	0	0	0	1	0	0	0	0
ENSMUSG00000080859	RPL10-PS1	0	0	0	0	1	0	0	0	1	0	0	0
ENSMUSG00000080860	GM13815	0	1	1	0	1	0	0	0	0	0	0	0
ENSMUSG00000080866	GM5687	2	2	2	2	1	0	1	4	3	2	0	4
ENSMUSG00000080868	GM15745	0	0	0	0	0	0	1	0	0	0	0	0
ENSMUSG00000080873	RPL30-PS3	0	0	0	0	0	0	1	1	1	1	1	1
ENSMUSG00000080875	GM7332	0	0	0	0	0	0	0	0	1	0	0	0
ENSMUSG00000080877	RPL22-PS1	0	0	0	0	0	0	2	0	0	1	1	0
ENSMUSG00000080885	RPL10-PS6	0	0	0	0	0	0	1	1	0	1	0	0
ENSMUSG00000080893	GM15920	0	0	1	0	0	0	0	1	1	0	0	1
ENSMUSG00000080896	GM14567	0	0	0	0	0	0	0	1	0	0	0	0
ENSMUSG00000080904	GM11966	2	1	4	2	6	3	17	18	12	14	13	11
ENSMUSG00000080921	RPL38-PS2	2	2	4	3	2	1	10	15	10	5	11	12
ENSMUSG00000080922	GM2214	0	0	1	0	0	0	0	1	0	0	0	0
ENSMUSG00000080928	HMGB1-PS6	1	0	0	0	0	0	0	0	1	0	0	1
ENSMUSG00000080932	GM10224	0	0	0	1	0	0	1	0	1	1	0	0
ENSMUSG00000080944	GM12967	2	1	2	2	0	1	1	2	2	2	2	3
ENSMUSG00000080949	GM13683	0	0	0	0	0	0	0	1	0	0	0	0
ENSMUSG00000080950	GM7278	0	1	0	0	0	0	0	0	0	0	0	0
ENSMUSG00000080957	GM15739	0	0	0	0	0	0	0	0	0	0	0	1
ENSMUSG00000080972	GM16061	0	0	0	0	0	0	1	0	0	2	0	0
ENSMUSG00000080979	GM13675	0	0	0	0	0	0	0	0	0	1	0	0
ENSMUSG00000080989	GM14048	0	1	0	0	0	0	0	0	0	0	0	0
ENSMUSG00000080993	GM12903	0	0	1	0	0	0	0	0	0	0	0	0
ENSMUSG00000080996	GM4986	0	0	0	0	0	0	2	3	0	0	1	1
ENSMUSG00000080998	GM5384	0	0	0	0	0	0	1	0	1	0	0	1
ENSMUSG00000081004	GM9157	0	0	0	0	0	0	0	0	0	1	0	0
ENSMUSG00000081011	GM11913	0	0	0	0	1	0	0	0	0	0	0	0
ENSMUSG00000081021	GM11964	0	0	0	0	0	0	0	1	0	0	0	0
ENSMUSG00000081023	GM15766	0	0	0	0	0	0	0	0	0	0	0	1
ENSMUSG00000081046	GM12846	0	0	0	0	0	0	1	0	0	1	0	0
ENSMUSG00000081049	RPS24-PS3	4	3	0	4	1	1	21	19	10	15	10	16
ENSMUSG00000081051	GM15427	2	6	3	7	4	4	19	15	15	15	7	16
ENSMUSG00000081055	GM6987	0	0	0	0	0	0	0	1	0	0	0	0
ENSMUSG00000081058	HIST2H3C2	0	1	0	1	1	1	7	7	3	7	6	7

ENSMUSG00000081076	RPSA-PS4	0	0	0	0	0	0	1	1	3	0	1	0
ENSMUSG00000081077	GM13899	0	0	0	1	1	0	0	0	0	0	0	0
ENSMUSG00000081078	GM12568	0	1	0	0	1	0	1	0	0	1	0	1
ENSMUSG00000081087	RPS15A-PS7	0	0	0	0	0	0	1	0	2	1	0	0
ENSMUSG00000081094	RPL19-PS11	0	0	1	0	0	2	1	0	0	3	2	4
ENSMUSG00000081100	GM14630	0	1	0	0	0	0	0	0	0	0	0	0
ENSMUSG00000081103	RPS12-PS26	0	0	0	0	0	0	0	0	0	1	0	2
ENSMUSG00000081113	GM7308	0	0	0	0	0	0	0	1	0	0	0	0
ENSMUSG00000081114	GM15390	0	0	0	0	0	0	0	0	0	0	0	1
ENSMUSG00000081118	GM6758	0	0	0	0	0	0	0	0	0	1	0	0
ENSMUSG00000081121	GM12791	0	0	0	0	0	0	0	0	1	0	0	0
ENSMUSG00000081125	RPL26-PS5	0	0	0	0	0	0	0	0	0	0	1	0
ENSMUSG00000081128	GM13328	0	0	0	0	0	0	0	0	1	0	0	0
ENSMUSG00000081136	GM12990	0	0	0	0	0	1	0	0	0	0	0	0
ENSMUSG00000081137	BC022960	1	0	0	0	0	0	1	0	1	1	0	2
ENSMUSG00000081143	GM15823	0	0	0	0	0	1	1	1	1	1	0	3
ENSMUSG00000081153	GM11401	1	0	1	1	0	0	0	2	0	0	0	0
ENSMUSG00000081159	GM6023	0	0	0	0	0	1	0	1	0	1	2	2
ENSMUSG00000081170	GM12922	0	0	0	1	0	0	1	1	0	0	0	0
ENSMUSG00000081179	GM13136	0	0	0	0	0	1	0	0	0	0	0	0
ENSMUSG00000081187	3110067C02F	0	0	0	0	0	0	0	0	0	0	1	1
ENSMUSG00000081191	GM11271	0	0	0	0	0	0	0	0	0	0	0	1
ENSMUSG00000081194	GM8424	0	0	0	0	0	0	0	0	1	0	0	0
ENSMUSG00000081195	RHOX2-PS	0	0	0	0	0	0	1	0	0	1	2	0
ENSMUSG00000081208	GM11400	0	0	0	0	0	0	0	0	0	1	0	0
ENSMUSG00000081210	GM12933	0	0	0	0	0	0	0	1	1	0	0	0
ENSMUSG00000081214	RPL35A-PS2	10	9	6	9	13	13	37	24	35	48	27	48
ENSMUSG00000081228	GM16089	0	0	0	0	0	0	1	0	0	1	0	0
ENSMUSG00000081233	GM12857	0	1	0	0	0	0	0	0	0	0	0	0
ENSMUSG00000081248	GM13787	0	0	0	0	1	0	0	0	0	0	0	0
ENSMUSG00000081249	GM11517	0	0	0	0	0	0	0	1	3	0	1	0
ENSMUSG00000081255	GM5380	1	0	0	0	0	0	0	0	0	0	0	0
ENSMUSG00000081256	GM15791	0	0	0	0	0	0	1	0	0	0	0	0
ENSMUSG00000081263	GM14859	0	0	0	0	0	0	0	0	0	0	2	0
ENSMUSG00000081275	GM13875	0	0	0	0	0	0	0	1	0	0	0	0
ENSMUSG00000081276	GM13132	0	0	0	0	0	0	0	0	0	1	0	0
ENSMUSG00000081285	GM18859	0	0	0	0	0	0	0	0	0	0	1	0
ENSMUSG00000081302	GM12020	0	0	1	0	0	0	0	1	0	1	0	0
ENSMUSG00000081306	GM15266	0	1	0	0	0	0	0	0	0	0	0	0
ENSMUSG00000081339	GM16044	0	0	0	0	0	0	0	1	1	0	0	0
ENSMUSG00000081344	GM14303	3	2	6	3	3	3	14	13	7	10	9	11
ENSMUSG00000081359	COX20-PS	0	2	0	0	0	0	0	0	0	0	1	1
ENSMUSG00000081378	RPS13-PS4	0	1	1	0	0	1	0	0	1	1	0	1
ENSMUSG00000081382	RPL18-PS1	0	0	0	1	0	0	4	2	2	4	4	4
ENSMUSG00000081391	GM11765	1	0	0	1	2	0	0	1	0	0	2	0
ENSMUSG00000081394	GM13215	0	0	0	0	0	1	0	0	1	1	0	0
ENSMUSG00000081400	GM13680	0	0	0	0	1	0	0	1	0	0	1	2
ENSMUSG00000081402	GM15455	0	0	0	0	1	0	0	0	0	0	0	1
ENSMUSG00000081406	RPS6-PS4	7	13	7	16	14	15	29	30	22	32	25	26
ENSMUSG00000081413	UQCRH-PS2	0	0	1	0	0	0	2	0	0	0	0	2
ENSMUSG00000081416	GM14383	0	0	0	0	0	0	0	0	0	1	0	0
ENSMUSG00000081421	GM13445	0	0	0	0	1	0	0	0	0	0	0	0
ENSMUSG00000081423	GM12882	0	0	1	0	0	0	0	1	0	0	1	0
ENSMUSG00000081429	RPS27-PS1	0	0	0	0	1	0	0	1	1	0	0	0
ENSMUSG00000081431	GM15483	1	0	0	0	0	0	0	0	0	0	0	0
ENSMUSG00000081434	GM14165	0	2	2	1	1	0	3	4	1	3	2	2
ENSMUSG00000081436	GM5912	0	0	0	0	0	0	0	0	0	1	0	0
ENSMUSG00000081453	GM6767	0	0	0	0	0	0	0	1	1	0	0	0

ENSMUSG00000081455	HMGB1-PS3	0	0	0	0	0	0	0	0	0	1	0	1
ENSMUSG00000081461	RPL30-PS11	0	0	0	0	0	0	0	0	0	0	0	1
ENSMUSG00000081467	GM13337	0	1	0	0	0	0	1	0	0	0	0	0
ENSMUSG00000081473	GM4853	0	2	0	1	0	0	0	0	0	1	0	0
ENSMUSG00000081480	GM13608	0	0	0	0	0	0	0	0	1	1	0	0
ENSMUSG00000081483	GM12445	0	0	0	1	0	0	0	0	0	0	0	0
ENSMUSG00000081485	GM12338	0	0	1	0	1	0	2	3	3	5	3	1
ENSMUSG00000081491	GM12778	0	0	1	3	0	1	0	3	0	1	1	0
ENSMUSG00000081494	GM14130	0	0	0	0	0	0	0	0	1	0	0	1
ENSMUSG00000081496	GM13428	0	0	0	1	0	0	0	0	0	0	0	0
ENSMUSG00000081499	GM12517	0	0	0	0	0	0	0	0	0	0	0	1
ENSMUSG00000081504	GM12508	0	0	0	0	0	0	0	2	0	2	0	1
ENSMUSG00000081512	GM15821	0	0	0	0	0	1	0	0	0	0	0	0
ENSMUSG00000081519	GM13331	0	0	0	0	0	1	0	0	0	0	0	0
ENSMUSG00000081520	GM16200	0	0	0	1	0	0	0	0	1	1	1	1
ENSMUSG00000081528	GM12924	0	0	0	1	0	0	0	0	0	1	0	0
ENSMUSG00000081534	SLC48A1	1	0	0	2	2	3	9	8	6	12	7	9
ENSMUSG00000081537	GM37832	0	0	0	0	0	0	0	0	0	1	0	0
ENSMUSG00000081552	GM12328	0	0	0	0	0	0	1	2	0	0	0	0
ENSMUSG00000081557	GM5697	0	0	0	0	0	0	1	0	0	0	0	0
ENSMUSG00000081559	GM12411	0	0	0	0	0	0	0	0	0	0	0	1
ENSMUSG00000081565	RPS12-PS5	0	1	0	0	0	0	6	3	0	0	1	2
ENSMUSG00000081568	GM15032	0	0	0	0	0	0	1	2	1	2	0	3
ENSMUSG00000081572	GM14138	0	0	0	0	0	0	2	2	2	2	0	0
ENSMUSG00000081574	GM14429	0	0	0	1	0	0	0	0	0	0	0	0
ENSMUSG00000081575	GM12090	0	0	0	1	0	1	0	1	0	1	2	0
ENSMUSG00000081594	GM15467	0	0	0	0	0	0	1	0	0	1	2	1
ENSMUSG00000081595	GM13036	0	0	0	0	0	0	0	0	0	0	0	1
ENSMUSG00000081600	GM12286	0	0	0	0	0	0	0	0	0	1	1	0
ENSMUSG00000081603	GM14681	1	1	2	0	1	0	2	2	2	2	3	1
ENSMUSG00000081604	GM11518	2	5	3	6	7	5	12	13	3	18	6	6
ENSMUSG00000081613	GM12109	0	0	0	0	0	0	0	0	0	0	1	0
ENSMUSG00000081616	GM14247	0	0	0	0	0	0	0	0	0	0	1	0
ENSMUSG00000081620	GM14633	0	0	0	0	0	1	1	0	0	0	0	0
ENSMUSG00000081629	EIF3S6-PS1	0	2	0	1	0	0	0	0	1	0	1	0
ENSMUSG00000081631	GM12005	0	1	1	1	1	1	3	2	1	6	2	0
ENSMUSG00000081633	GM8522	0	0	0	0	0	0	0	0	1	0	0	0
ENSMUSG00000081641	GM9439	0	0	1	0	0	0	0	0	0	0	0	0
ENSMUSG00000081642	GM13532	0	0	0	0	0	0	0	2	1	0	0	0
ENSMUSG00000081648	GM13423	3	3	2	3	2	0	5	7	2	7	2	5
ENSMUSG00000081650	GM16181	0	1	0	0	0	0	0	1	0	1	1	2
ENSMUSG00000081651	GM15530	0	0	0	0	0	0	0	0	0	0	0	1
ENSMUSG00000081657	GM15466	0	0	0	0	0	0	0	1	0	0	0	0
ENSMUSG00000081661	GM14450	0	0	0	0	0	0	0	0	0	1	0	0
ENSMUSG00000081669	NPM3-PS1	0	0	0	0	0	0	0	0	0	1	0	0
ENSMUSG00000081670	GM15697	0	0	0	2	2	1	2	0	6	1	3	2
ENSMUSG00000081673	GM14794	0	0	1	0	0	0	0	0	0	0	0	1
ENSMUSG00000081675	GM12712	0	0	0	0	0	0	1	0	0	0	1	0
ENSMUSG00000081678	GM12959	0	0	0	0	0	0	2	0	1	1	0	0
ENSMUSG00000081684	RPS2-PS13	1	3	7	2	1	1	7	7	8	10	6	7
ENSMUSG00000081692	GM15971	0	0	0	0	0	0	1	0	0	0	0	0
ENSMUSG00000081695	RPSA-PS5	0	0	0	0	0	0	0	1	0	1	0	0
ENSMUSG00000081703	GM6285	0	0	0	1	0	0	1	0	0	0	2	1
ENSMUSG00000081705	GM12582	0	0	0	0	0	0	0	0	0	1	0	0
ENSMUSG00000081715	RPS11-PS3	0	0	0	0	0	0	0	0	1	0	0	0
ENSMUSG00000081724	OLFR129	0	0	0	0	0	1	0	0	1	5	0	3
ENSMUSG00000081729	HSPA9-PS1	0	0	0	0	0	0	2	0	0	0	0	0
ENSMUSG00000081730	GM13785	0	0	0	0	1	0	1	2	2	0	0	0

ENSMUSG00000081731	CALR-PS	0	0	0	1	0	0	0	0	0	0	0	0
ENSMUSG00000081735	GM14530	0	0	0	0	0	0	0	1	0	0	0	0
ENSMUSG00000081739	MDM4-PS	0	1	0	0	0	1	2	3	0	0	1	1
ENSMUSG00000081740	GM14279	1	2	0	0	0	0	0	2	0	1	0	0
ENSMUSG00000081752	SMS-PS	0	0	0	0	0	0	0	0	0	0	0	2
ENSMUSG00000081769	GM12216	1	1	0	0	3	1	5	2	16	0	1	1
ENSMUSG00000081773	GM8302	0	0	0	0	0	0	0	1	0	0	0	0
ENSMUSG00000081778	GM6325	0	0	0	0	0	0	0	0	0	0	0	1
ENSMUSG00000081783	GM15599	0	1	0	0	0	0	1	0	0	0	0	0
ENSMUSG00000081788	GM5898	0	0	0	0	0	0	0	1	1	1	2	1
ENSMUSG00000081792	ANP32B-PS1	0	0	0	0	0	0	0	1	0	0	1	0
ENSMUSG00000081797	GM8662	0	0	0	0	0	0	0	1	0	0	0	0
ENSMUSG00000081810	GM11878	0	0	0	0	0	0	0	1	1	0	0	0
ENSMUSG00000081811	GM12713	0	0	0	0	0	0	0	0	1	0	0	0
ENSMUSG00000081812	GM4789	0	0	0	0	0	0	1	0	0	0	0	0
ENSMUSG00000081824	BC002163	0	0	0	0	0	0	1	0	0	1	0	1
ENSMUSG00000081855	RPL17-PS5	1	3	5	9	6	5	13	11	13	11	13	12
ENSMUSG00000081859	OLFR650-PS1	0	1	0	0	0	1	0	0	0	2	0	0
ENSMUSG00000081861	GM14441	0	0	1	0	0	0	0	0	0	0	0	0
ENSMUSG00000081866	GM12401	0	0	0	0	0	0	0	0	0	1	0	0
ENSMUSG00000081871	GM11488	0	0	0	0	0	0	0	1	2	0	0	0
ENSMUSG00000081873	GM15197	0	0	1	1	0	0	0	0	0	0	0	0
ENSMUSG00000081887	GM12097	0	0	0	0	0	0	0	1	0	0	0	0
ENSMUSG00000081892	GM8864	0	0	0	0	0	0	0	0	1	0	0	0
ENSMUSG00000081895	RPL17-PS10	1	2	2	0	0	2	5	3	11	4	6	6
ENSMUSG00000081897	GM15067	0	0	0	0	0	0	0	0	0	0	1	0
ENSMUSG00000081924	GM11191	0	0	0	0	0	0	0	1	0	0	0	0
ENSMUSG00000081926	GM15536	11	20	24	33	21	16	53	62	59	53	37	66
ENSMUSG00000081929	RPS11-PS2	0	1	2	0	1	1	2	2	1	2	2	3
ENSMUSG00000081931	GM15211	0	0	0	0	0	0	0	0	0	0	0	1
ENSMUSG00000081934	GM12193	0	0	0	0	0	0	0	1	0	0	0	0
ENSMUSG00000081943	GM9078	0	0	0	0	0	0	0	0	1	0	0	0
ENSMUSG00000081944	GM12276	0	0	0	0	0	0	1	1	0	0	2	0
ENSMUSG00000081946	GM11472	0	0	0	0	0	0	3	0	0	0	1	0
ENSMUSG00000081953	GM12435	0	0	0	0	0	0	1	1	2	0	1	0
ENSMUSG00000081965	GM11620	0	0	1	0	0	0	0	0	0	0	0	0
ENSMUSG00000081968	RPL23A-PS2	1	1	0	0	0	0	1	0	1	0	0	2
ENSMUSG00000081974	GM11960	1	0	0	0	0	1	0	0	0	0	0	0
ENSMUSG00000081979	RPS8-PS3	0	1	0	0	0	0	0	0	1	1	1	0
ENSMUSG00000081988	GM15746	0	1	0	0	0	0	0	0	0	1	0	0
ENSMUSG00000081992	GM13408	0	0	0	2	2	0	4	3	7	4	3	3
ENSMUSG00000081999	GM13461	0	0	0	0	0	0	0	0	0	0	0	1
ENSMUSG00000082000	GM12488	0	0	1	1	0	1	3	1	1	3	0	0
ENSMUSG00000082001	GM15607	0	0	0	0	0	0	1	0	0	2	0	1
ENSMUSG00000082011	GM15043	0	0	0	0	0	0	0	1	0	0	0	0
ENSMUSG00000082012	GM5257	0	0	0	0	0	0	0	0	0	1	0	0
ENSMUSG00000082013	GM13216	2	1	1	0	1	1	0	3	1	4	0	0
ENSMUSG00000082016	PGAM1-PS2	0	0	0	0	0	1	0	0	0	0	0	0
ENSMUSG00000082023	GM14407	0	0	0	0	0	0	0	1	0	0	0	0
ENSMUSG00000082029	H3F3C	0	0	0	0	0	0	1	0	0	0	0	0
ENSMUSG00000082035	RPL17-PS8	4	2	5	7	9	4	12	9	13	12	7	17
ENSMUSG00000082043	GM12848	0	0	0	0	0	0	2	0	0	0	0	0
ENSMUSG00000082044	SNRPERT	0	0	0	1	0	0	3	1	2	5	1	0
ENSMUSG00000082045	GM11585	1	0	0	0	0	0	0	0	0	0	0	0
ENSMUSG00000082051	GM16072	0	0	0	0	0	0	1	0	0	0	0	0
ENSMUSG00000082057	GM15789	1	2	1	0	0	0	0	1	0	1	2	3
ENSMUSG00000082061	GM12726	0	0	0	0	0	0	0	0	0	4	3	0
ENSMUSG00000082064	RPL5-PS2	0	1	0	0	0	0	0	0	1	0	0	1

ENSMUSG00000082072	GM15785	0	0	0	0	0	0	0	0	1	0	0	0
ENSMUSG00000082078	GM12098	0	0	0	0	1	0	1	2	0	1	0	3
ENSMUSG00000082088	GM15753	0	0	0	0	0	0	1	1	2	3	0	1
ENSMUSG00000082097	GM14039	0	0	0	0	0	0	0	0	0	2	1	0
ENSMUSG00000082099	GM12013	0	0	0	0	1	0	0	0	0	0	0	0
ENSMUSG00000082109	RPL19-PS12	0	0	0	0	0	0	0	2	0	1	0	0
ENSMUSG00000082110	GM11196	1	0	0	0	0	0	0	0	0	0	0	0
ENSMUSG00000082120	GM15720	1	0	0	0	0	1	1	4	0	0	0	0
ENSMUSG00000082127	GM13577	0	0	0	0	1	0	2	3	3	2	1	3
ENSMUSG00000082128	GM11824	0	0	0	0	0	0	0	0	1	0	0	0
ENSMUSG00000082130	GM15799	0	0	1	0	0	0	0	2	0	1	0	1
ENSMUSG00000082140	GM13094	0	0	0	0	0	0	1	0	0	0	0	0
ENSMUSG00000082145	GM12312	0	0	0	0	0	0	0	0	0	0	1	0
ENSMUSG00000082151	GM11810	0	0	0	0	0	0	1	0	0	0	1	0
ENSMUSG00000082157	GM12038	0	0	0	0	0	1	1	0	2	0	0	0
ENSMUSG00000082163	GM14276	1	2	2	0	0	1	2	2	1	4	1	1
ENSMUSG00000082165	GM7091	0	0	0	0	0	0	0	0	0	1	0	0
ENSMUSG00000082171	RPL27-PS2	0	0	0	0	0	0	0	1	0	0	0	0
ENSMUSG00000082180	GM12230	0	0	0	0	0	0	0	0	1	0	0	0
ENSMUSG00000082189	GM4784	0	0	1	0	0	0	0	0	0	0	0	0
ENSMUSG00000082190	GM13641	0	0	0	0	0	0	1	0	0	0	0	1
ENSMUSG00000082193	RPL5-PS1	2	3	2	5	3	5	12	9	14	9	7	10
ENSMUSG00000082208	GM15379	0	0	0	1	0	0	1	0	1	0	1	0
ENSMUSG00000082245	GM6292	0	0	0	0	0	0	0	0	0	0	0	1
ENSMUSG00000082246	GM13047	0	0	0	0	0	0	1	0	0	0	0	0
ENSMUSG00000082247	GM16074	0	0	0	0	0	0	0	0	0	0	1	0
ENSMUSG00000082258	GM15289	0	0	0	0	0	0	1	0	0	0	0	0
ENSMUSG00000082260	GM7429	0	0	0	0	0	0	0	2	0	0	0	1
ENSMUSG00000082263	GM11188	0	0	0	0	0	0	0	0	1	0	0	0
ENSMUSG00000082264	GM12799	0	0	0	0	0	1	1	1	0	0	0	0
ENSMUSG00000082272	GM11675	1	0	0	0	0	0	2	2	1	0	0	0
ENSMUSG00000082276	GM13885	0	0	0	0	0	0	1	0	0	0	0	0
ENSMUSG00000082280	GM5847	0	0	0	0	0	0	0	0	0	0	1	0
ENSMUSG00000082284	H3F3A-PS1	0	0	0	0	0	0	1	2	1	0	2	0
ENSMUSG00000082286	PISD-PS1	1	1	2	0	0	0	1	2	3	2	2	5
ENSMUSG00000082292	GM12250	1	0	0	0	0	0	0	0	0	1	0	0
ENSMUSG00000082296	GM11450	0	0	0	0	0	0	0	0	1	0	1	0
ENSMUSG00000082314	GM13296	0	0	0	0	0	0	0	0	0	0	0	1
ENSMUSG00000082315	GM16523	1	0	1	1	1	0	0	1	3	4	2	2
ENSMUSG00000082319	GM8822	2	2	1	1	4	2	6	3	2	6	4	5
ENSMUSG00000082320	GM11877	0	0	0	0	0	0	0	0	0	1	0	0
ENSMUSG00000082328	GM13921	0	0	0	0	0	0	0	0	1	0	0	0
ENSMUSG00000082329	GM14287	0	0	0	0	0	0	0	0	0	0	1	0
ENSMUSG00000082341	GM14143	0	0	0	0	0	0	0	0	0	1	0	0
ENSMUSG00000082345	GM13622	2	1	5	3	1	4	8	7	4	8	3	5
ENSMUSG00000082348	GM12807	0	0	0	1	0	0	0	0	0	0	0	0
ENSMUSG00000082353	VMN1R-PS1	0	0	0	0	0	0	0	0	0	0	1	0
ENSMUSG00000082361	BTC	0	0	0	0	0	0	0	1	0	1	0	1
ENSMUSG00000082383	GM9670	0	3	1	0	0	1	4	3	0	0	2	1
ENSMUSG00000082396	ZFP389	0	0	0	0	0	0	2	0	0	0	1	1
ENSMUSG00000082400	GM16017	0	0	0	1	0	0	0	0	0	0	0	0
ENSMUSG00000082418	GM13126	0	0	0	0	0	0	0	0	0	0	0	1
ENSMUSG00000082419	GM11425	13	9	8	10	12	12	24	47	29	34	30	38
ENSMUSG00000082423	GM15148	0	0	0	0	0	0	0	1	0	0	0	0
ENSMUSG00000082424	GM13292	0	2	3	1	1	0	4	4	4	2	3	1
ENSMUSG00000082436	GM11688	0	1	0	0	1	0	5	3	1	3	2	1
ENSMUSG00000082448	GM11676	0	0	0	0	0	0	0	1	0	0	0	0
ENSMUSG00000082455	GM13665	0	1	1	2	2	1	2	1	2	2	1	2

ENSMUSG00000082456	GM11598	0	0	0	0	0	0	1	1	0	0	0	0
ENSMUSG00000082465	RPS6-PS3	2	2	4	0	2	2	8	7	9	10	7	8
ENSMUSG00000082467	GM6743	0	0	0	0	0	0	1	0	0	0	0	0
ENSMUSG00000082474	GM8213	2	0	1	0	0	0	2	1	3	1	1	1
ENSMUSG00000082475	GM7206	0	1	0	0	0	0	2	0	0	0	2	1
ENSMUSG00000082476	GM12242	0	0	0	0	0	0	1	0	0	0	0	0
ENSMUSG00000082478	GM11619	0	0	0	0	0	0	0	0	0	0	1	0
ENSMUSG00000082480	GM11687	0	0	0	0	0	0	0	0	0	0	1	0
ENSMUSG00000082481	RPS12-PS19	0	0	0	0	0	0	0	1	0	0	0	0
ENSMUSG00000082487	GM11628	0	0	0	0	0	0	0	0	0	0	1	2
ENSMUSG00000082488	1700119I11R	0	0	0	0	0	0	0	1	0	0	0	0
ENSMUSG00000082491	GM5909	0	0	0	0	1	1	2	2	0	1	2	1
ENSMUSG00000082498	GM12937	0	0	0	1	0	0	0	0	0	0	0	0
ENSMUSG00000082503	GM12229	0	0	0	0	0	0	0	0	0	0	0	1
ENSMUSG00000082507	GM9378	0	0	0	0	0	0	0	0	0	1	0	0
ENSMUSG00000082515	GM12902	0	0	0	0	0	0	0	0	0	1	0	2
ENSMUSG00000082520	GM13349	1	0	1	0	1	0	3	6	1	3	5	4
ENSMUSG00000082529	GM11199	0	0	0	0	0	0	1	0	0	0	0	0
ENSMUSG00000082530	GM12168	0	0	0	0	0	0	1	0	0	0	0	0
ENSMUSG00000082531	GM11556	0	0	0	0	0	0	0	0	0	0	1	0
ENSMUSG00000082532	RPL31-PS6	0	0	1	0	0	0	0	0	1	2	1	0
ENSMUSG00000082535	GM7860	0	0	0	0	0	0	0	1	1	0	0	0
ENSMUSG00000082536	GM13456	0	2	0	0	0	0	0	0	1	1	0	1
ENSMUSG00000082549	GM14813	1	0	2	2	3	2	1	2	2	4	1	1
ENSMUSG00000082561	GM15580	1	0	0	1	0	1	0	2	1	0	3	2
ENSMUSG00000082575	GM13050	0	0	0	0	0	0	0	1	0	0	0	0
ENSMUSG00000082576	GM12058	0	0	0	0	0	0	0	0	0	2	1	1
ENSMUSG00000082579	GM11979	0	0	0	0	0	0	0	1	0	0	1	0
ENSMUSG00000082580	GM13182	0	0	0	0	0	1	0	1	0	0	0	0
ENSMUSG00000082596	GM14227	1	0	0	0	0	1	0	2	0	3	0	2
ENSMUSG00000082599	GM13141	0	0	0	0	0	0	0	0	0	0	1	0
ENSMUSG00000082605	GM16148	0	0	0	0	0	0	1	0	0	0	0	0
ENSMUSG00000082609	GM15464	0	0	0	0	0	0	0	0	1	2	0	1
ENSMUSG00000082616	GM6025	0	0	0	0	0	0	0	1	0	0	0	0
ENSMUSG00000082617	SMT3H2-PS	0	0	0	0	0	0	0	0	0	1	0	0
ENSMUSG00000082626	RPS13-PS5	0	0	1	1	0	0	0	1	1	0	0	1
ENSMUSG00000082633	GM15779	1	0	0	0	0	0	0	0	1	0	0	1
ENSMUSG00000082641	RPL30-PS5	0	0	0	0	1	1	0	0	0	0	0	0
ENSMUSG00000082645	GM15481	0	0	0	0	0	1	0	1	0	0	0	0
ENSMUSG00000082647	GM14056	0	0	0	0	0	0	0	1	0	0	1	1
ENSMUSG00000082649	GM12754	0	0	1	0	1	0	0	0	0	0	0	0
ENSMUSG00000082651	GM14001	0	0	0	0	0	0	0	0	0	1	0	0
ENSMUSG00000082655	GM7857	0	1	0	0	0	0	0	1	0	0	0	0
ENSMUSG00000082658	FAU-PS2	0	0	0	0	0	0	1	0	0	0	0	1
ENSMUSG00000082665	GM11470	0	0	1	0	0	0	0	0	0	0	1	0
ENSMUSG00000082674	GM11914	1	0	0	0	0	0	0	1	1	2	0	1
ENSMUSG00000082676	GM11843	0	0	0	0	0	0	1	0	0	0	0	0
ENSMUSG00000082678	GM12818	0	0	0	0	0	1	0	0	0	0	0	0
ENSMUSG00000082691	DYNLT1-PS1	0	0	0	0	0	1	0	0	0	0	0	0
ENSMUSG00000082711	GM12987	0	0	0	0	0	0	0	0	0	0	0	1
ENSMUSG00000082729	GM14845	0	0	0	0	0	0	0	0	0	0	0	1
ENSMUSG00000082733	GM15153	0	0	0	0	0	0	1	0	0	0	0	0
ENSMUSG00000082740	GM14127	0	0	0	1	0	0	1	0	0	0	0	0
ENSMUSG00000082741	GM9703	12	7	9	19	19	10	36	28	29	36	32	30
ENSMUSG00000082743	GM12988	0	0	0	0	0	0	1	0	0	0	0	0
ENSMUSG00000082746	RPS12-PS1	0	0	0	0	0	0	0	0	1	0	0	0
ENSMUSG00000082755	GM8692	1	0	1	2	1	0	3	4	4	7	3	4
ENSMUSG00000082762	GM12366	0	0	0	0	0	0	0	0	0	0	1	1

ENSMUSG00000082765	GM14411	0	0	0	0	0	0	1	1	1	1	0	0
ENSMUSG00000082769	GM15793	0	0	0	0	0	0	1	0	0	0	0	0
ENSMUSG00000082791	GM4875	0	0	0	0	0	0	1	0	0	0	0	0
ENSMUSG00000082792	GM12176	0	0	0	0	0	0	0	1	2	1	1	0
ENSMUSG00000082804	RPL17-PS1	0	0	0	0	0	0	0	1	0	2	1	0
ENSMUSG00000082806	RPL13-PS1	0	0	0	0	0	0	1	0	0	1	3	1
ENSMUSG00000082809	GM14150	0	0	0	0	0	0	0	0	2	0	0	0
ENSMUSG00000082812	GM14860	0	0	0	0	0	0	1	0	0	0	0	0
ENSMUSG00000082814	GM12956	0	0	0	0	0	0	0	0	0	0	1	0
ENSMUSG00000082820	GM13803	0	0	1	0	0	0	0	1	1	0	0	0
ENSMUSG00000082826	MIF-PS3	0	0	0	0	0	0	0	0	0	1	0	0
ENSMUSG00000082828	GM16106	0	0	0	0	0	0	0	0	0	0	0	2
ENSMUSG00000082848	GM16066	0	0	0	0	0	0	1	1	0	1	0	1
ENSMUSG00000082855	GM14537	0	1	0	1	0	1	1	0	0	1	1	0
ENSMUSG00000082867	GM15730	1	1	0	1	2	3	5	6	4	6	4	6
ENSMUSG00000082874	RPL15-PS1	5	13	6	4	5	5	17	15	14	31	12	30
ENSMUSG00000082876	GM11889	0	0	0	0	0	0	0	1	0	0	0	0
ENSMUSG00000082878	GM12583	0	0	0	0	0	0	0	0	0	0	0	1
ENSMUSG00000082895	RPSA-PS9	2	2	1	0	2	6	7	8	3	1	3	2
ENSMUSG00000082896	GM5844	0	0	0	2	0	0	1	0	2	1	2	3
ENSMUSG00000082901	GM14323	1	1	3	3	0	1	2	2	3	2	1	2
ENSMUSG00000082920	GM13864	1	0	0	0	0	0	0	0	1	0	0	0
ENSMUSG00000082922	GM6939	0	0	0	0	0	0	0	1	0	0	0	0
ENSMUSG00000082925	GM13135	0	0	0	0	0	0	0	0	0	0	1	0
ENSMUSG00000082926	GM8172	0	1	0	0	0	0	1	1	2	2	1	2
ENSMUSG00000082927	GM5863	0	0	0	0	0	0	0	1	0	0	0	1
ENSMUSG00000082931	GM13586	1	1	3	2	1	1	0	0	0	1	0	1
ENSMUSG00000082935	GM7658	0	0	1	0	1	0	0	3	0	1	0	0
ENSMUSG00000082936	GM16166	0	0	0	0	0	0	0	0	0	0	1	0
ENSMUSG00000082938	GM2810	0	1	0	0	0	0	0	0	0	0	0	0
ENSMUSG00000082947	GM12587	0	0	0	0	0	0	1	0	1	0	0	0
ENSMUSG00000082967	GM16082	0	0	0	0	0	0	0	1	1	0	0	0
ENSMUSG00000082969	GM12630	0	0	0	1	0	0	1	1	1	1	0	0
ENSMUSG00000082978	RPSA-PS11	3	1	6	6	6	1	14	15	18	16	8	20
ENSMUSG00000082980	OLFR1274-PS	0	0	0	0	0	0	0	1	0	0	0	0
ENSMUSG00000082987	RPS13-PS6	0	0	0	0	1	0	0	0	0	0	0	0
ENSMUSG00000082991	RPS10-PS3	0	0	0	0	1	0	0	0	0	0	0	0
ENSMUSG00000082996	GM6238	1	0	0	0	1	0	1	0	0	2	0	1
ENSMUSG00000082998	GM11221	0	0	0	0	0	0	0	0	2	1	0	1
ENSMUSG00000083001	GM13250	0	0	0	0	0	1	0	0	0	0	0	0
ENSMUSG00000083004	GM13413	0	0	0	0	0	1	2	0	0	0	0	0
ENSMUSG00000083007	GM13623	0	0	1	0	1	0	4	4	0	2	1	1
ENSMUSG00000083011	GM12816	0	0	0	2	1	0	0	2	2	1	0	2
ENSMUSG00000083012	FAM220A	0	0	0	0	0	0	1	2	0	1	0	0
ENSMUSG00000083016	GM12618	0	0	0	0	0	0	0	1	0	1	0	2
ENSMUSG00000083017	GM12163	0	0	0	0	0	0	1	0	0	0	0	0
ENSMUSG00000083019	GM6222	0	0	0	0	0	0	0	0	0	0	0	1
ENSMUSG00000083022	RPS15A-PS6	14	22	18	31	20	15	109	77	65	72	54	69
ENSMUSG00000083027	GM13140	0	0	0	0	0	0	0	0	1	1	0	0
ENSMUSG00000083033	GM9083	0	0	0	0	0	0	0	0	1	1	0	0
ENSMUSG00000083041	GMFG-PS	0	0	0	0	1	0	0	0	0	0	0	0
ENSMUSG00000083055	GM14140	0	0	0	0	0	0	0	0	1	0	0	0
ENSMUSG00000083061	GM12191	0	0	0	0	0	0	0	0	0	0	1	1
ENSMUSG00000083064	GM7785	0	0	0	1	0	0	1	1	0	1	0	0
ENSMUSG00000083072	GM13668	0	0	0	0	0	0	0	1	0	0	0	0
ENSMUSG00000083077	GM14126	0	0	0	0	0	0	0	0	0	1	0	0
ENSMUSG00000083084	GM14667	0	0	0	0	0	0	1	0	0	0	0	2
ENSMUSG00000083086	GM15531	0	0	0	0	0	0	0	0	0	0	1	0

ENSMUSG00000083087	GM11249	6	9	14	9	6	10	33	25	20	19	19	28
ENSMUSG00000083093	GM15385	0	0	0	0	0	0	1	2	0	0	0	0
ENSMUSG00000083097	GM14494	0	0	0	0	0	1	0	0	1	0	0	0
ENSMUSG00000083100	GM6044	0	0	0	0	0	0	0	0	0	1	0	0
ENSMUSG00000083107	GM5303	0	0	0	0	0	0	1	0	0	0	0	0
ENSMUSG00000083113	RPL10-PS4	0	0	0	0	0	0	0	0	0	0	0	1
ENSMUSG00000083118	GM11609	0	0	0	0	0	0	0	0	0	1	0	0
ENSMUSG00000083121	GM6341	0	0	0	0	0	1	0	1	1	1	1	0
ENSMUSG00000083152	APC-PS1	0	0	0	0	0	0	1	0	0	0	0	0
ENSMUSG00000083153	GM12459	0	0	0	0	0	0	0	0	0	0	1	0
ENSMUSG00000083161	GM11427	15	16	23	20	21	18	119	97	83	127	73	94
ENSMUSG00000083179	GM12693	0	0	0	0	0	0	0	1	0	1	1	0
ENSMUSG00000083182	GM13828	0	0	1	0	0	0	0	0	0	0	0	0
ENSMUSG00000083191	GM15571	0	0	0	0	0	0	0	1	0	0	0	0
ENSMUSG00000083199	GM9432	1	0	1	0	1	0	2	0	1	1	1	2
ENSMUSG00000083206	GM11338	0	0	0	0	0	0	0	1	0	0	0	0
ENSMUSG00000083209	GM11969	0	0	1	0	0	0	3	0	0	1	1	0
ENSMUSG00000083215	RBMX2-PS	0	0	0	0	0	0	0	0	0	0	0	1
ENSMUSG00000083219	GM11410	0	0	0	0	0	1	0	0	1	0	0	0
ENSMUSG00000083232	GM11796	0	0	0	0	0	0	0	1	0	0	0	1
ENSMUSG00000083245	GM11185	0	0	0	0	0	0	1	0	0	0	1	0
ENSMUSG00000083246	GM11839	0	0	0	0	0	0	0	0	0	0	1	0
ENSMUSG00000083253	GM7108	0	0	1	0	0	0	0	0	0	0	0	0
ENSMUSG00000083257	GM15568	0	0	0	0	0	0	0	0	0	1	0	0
ENSMUSG00000083260	GM13460	0	0	0	0	0	0	0	0	0	0	1	0
ENSMUSG00000083306	GM13868	2	4	0	2	2	1	3	0	2	6	2	4
ENSMUSG00000083307	AA414768	0	0	0	0	0	0	0	0	1	0	2	0
ENSMUSG00000083316	GM15185	0	0	0	0	0	1	0	0	0	0	0	0
ENSMUSG00000083326	RPL38-PS1	1	0	0	0	1	0	3	1	0	1	1	0
ENSMUSG00000083327	VCP-RS	0	0	0	0	0	0	0	0	0	0	0	1
ENSMUSG00000083328	GM11826	0	0	0	0	1	0	3	0	2	2	0	1
ENSMUSG00000083337	GM11539	28	24	38	43	36	29	128	110	92	110	80	94
ENSMUSG00000083340	GM13156	0	0	0	0	0	0	0	1	1	1	0	0
ENSMUSG00000083344	GM7363	0	0	0	0	0	0	2	0	0	1	0	1
ENSMUSG00000083353	GM12540	0	0	0	0	0	0	0	0	1	1	0	0
ENSMUSG00000083362	GM8606	1	1	0	2	0	0	3	3	2	4	1	2
ENSMUSG00000083364	LLPH-PS2	0	0	0	0	0	0	0	1	0	1	0	1
ENSMUSG00000083378	GM13196	0	0	1	0	0	0	1	0	0	4	2	0
ENSMUSG00000083379	GM4919	0	0	0	0	0	0	1	0	0	0	0	0
ENSMUSG00000083380	GM3244	1	1	0	0	0	0	3	3	1	3	1	3
ENSMUSG00000083382	GM6433	0	0	0	0	1	0	0	2	0	0	0	0
ENSMUSG00000083394	GM11703	0	1	0	0	0	1	0	0	0	1	1	0
ENSMUSG00000083396	GM15542	0	0	0	0	1	0	2	0	2	1	1	0
ENSMUSG00000083397	GM5466	0	0	0	0	0	2	1	0	2	1	0	0
ENSMUSG00000083411	RPL30-PS10	0	1	1	0	0	0	0	2	1	0	1	1
ENSMUSG00000083412	GM12268	0	0	0	0	0	0	0	0	1	0	0	0
ENSMUSG00000083414	GM14840	0	0	0	0	0	0	0	1	0	0	0	0
ENSMUSG00000083424	RPL35A-PS4	4	2	2	2	1	1	10	15	6	7	11	6
ENSMUSG00000083425	GM13992	0	0	0	0	0	0	0	0	0	0	0	1
ENSMUSG00000083429	GM15198	0	1	0	0	0	0	4	0	0	1	2	1
ENSMUSG00000083440	RPL7A-PS12	0	0	0	0	0	0	1	1	0	0	0	0
ENSMUSG00000083446	GM12826	0	0	0	0	0	0	1	0	0	0	0	0
ENSMUSG00000083449	GM13487	0	0	0	0	0	0	0	0	1	0	1	1
ENSMUSG00000083454	GM13226	0	0	0	0	0	0	0	0	0	1	1	0
ENSMUSG00000083460	GM12182	0	0	0	0	0	0	0	1	0	0	0	0
ENSMUSG00000083481	RPS8-PS2	0	1	0	0	0	0	0	0	0	0	0	0
ENSMUSG00000083483	GM14044	1	0	0	1	2	0	1	1	1	0	1	1
ENSMUSG00000083486	GM14521	0	0	0	0	0	0	0	0	0	0	1	0

ENSMUSG00000083496	GM11263	0	1	0	0	0	0	0	0	0	0	1	0
ENSMUSG00000083498	GM14172	0	0	0	0	0	0	1	0	0	0	0	0
ENSMUSG00000083527	GM12001	1	0	0	0	0	0	0	0	0	0	0	0
ENSMUSG00000083534	H2-M6-PS	0	0	0	0	0	1	0	0	1	1	0	0
ENSMUSG00000083539	GM12466	0	0	0	1	0	0	1	0	1	1	1	0
ENSMUSG00000083553	GM11989	0	0	0	0	0	0	1	2	1	1	0	2
ENSMUSG00000083561	GM5942	0	0	0	0	0	0	0	0	1	0	0	0
ENSMUSG00000083563	GM13340	1	2	0	2	0	0	0	2	3	0	2	1
ENSMUSG00000083567	GM11451	0	0	0	2	1	0	1	0	0	2	1	0
ENSMUSG00000083569	GM13666	1	0	0	0	0	0	0	0	0	0	0	1
ENSMUSG00000083572	GM11849	0	0	0	0	0	0	0	0	0	0	0	1
ENSMUSG00000083573	GM7312	0	0	0	0	0	0	0	0	0	0	1	0
ENSMUSG00000083579	GM15538	0	0	0	1	1	0	0	0	2	0	1	1
ENSMUSG00000083596	RPL21-PS15	0	0	0	0	0	0	0	1	0	0	0	0
ENSMUSG00000083619	GM14414	0	1	1	0	0	0	0	0	1	0	0	0
ENSMUSG00000083621	GM14586	23	26	20	35	24	29	87	83	56	78	72	62
ENSMUSG00000083624	GM12469	0	0	0	0	0	0	1	0	1	1	0	0
ENSMUSG00000083626	GM4459	0	0	0	0	0	0	0	0	0	0	1	0
ENSMUSG00000083627	GM12502	0	0	0	0	0	0	0	0	0	1	0	0
ENSMUSG00000083642	GM13148	0	0	0	0	0	1	0	0	0	0	0	0
ENSMUSG00000083645	GM14086	0	0	0	0	0	0	0	1	1	0	0	0
ENSMUSG00000083649	RASL2-9	0	0	0	0	0	0	0	0	0	0	1	0
ENSMUSG00000083651	GM11524	0	0	0	0	1	0	0	0	0	1	0	0
ENSMUSG00000083669	GM10169	0	0	0	0	0	0	0	1	0	1	0	0
ENSMUSG00000083672	KPNA2-PS	0	0	0	0	0	0	1	0	1	1	0	1
ENSMUSG00000083674	ZFP133-PS	0	0	0	0	0	0	0	2	0	2	0	0
ENSMUSG00000083678	GM12989	0	0	0	0	0	0	0	0	1	2	1	2
ENSMUSG00000083679	GM12892	0	1	0	0	0	0	0	0	0	0	0	0
ENSMUSG00000083687	GM15154	0	0	0	0	0	0	0	0	0	0	0	1
ENSMUSG00000083688	GM13743	0	0	0	0	0	0	0	0	0	0	1	0
ENSMUSG00000083692	GM9575	0	0	1	0	1	0	2	1	2	1	1	0
ENSMUSG00000083700	GM2986	0	0	0	0	0	0	0	0	0	0	0	1
ENSMUSG00000083705	GM8624	0	0	0	1	2	0	0	0	1	2	4	2
ENSMUSG00000083713	GM5883	0	0	0	0	0	0	0	0	1	0	0	0
ENSMUSG00000083716	GM13436	1	1	0	4	3	2	7	7	6	7	9	16
ENSMUSG00000083718	CCNB2-PS	0	0	0	0	1	1	2	2	2	5	4	1
ENSMUSG00000083725	GM11865	0	0	0	0	0	0	1	0	0	0	0	0
ENSMUSG00000083728	GM13854	0	0	0	0	0	0	1	0	0	0	0	0
ENSMUSG00000083757	RPS15A-PS4	1	3	3	7	4	5	23	12	10	15	12	13
ENSMUSG00000083772	GM15566	0	0	0	0	0	0	0	0	1	0	0	0
ENSMUSG00000083776	GM13473	0	0	0	1	0	0	0	0	0	0	0	0
ENSMUSG00000083780	GM12728	0	0	0	0	0	0	1	0	0	0	0	0
ENSMUSG00000083795	GM15670	0	0	0	0	0	0	0	0	0	0	0	1
ENSMUSG00000083808	GM3828	0	0	0	0	0	0	0	0	0	0	1	0
ENSMUSG00000083812	GM5054	0	0	1	0	0	0	1	0	0	0	0	0
ENSMUSG00000083816	GM13033	0	0	0	0	0	0	0	0	0	0	0	1
ENSMUSG00000083820	GM6415	0	0	0	0	0	0	0	0	0	1	1	0
ENSMUSG00000083822	HMGB1-PS5	0	2	0	0	0	0	0	0	0	0	1	0
ENSMUSG00000083826	GM13039	0	0	0	0	0	0	0	0	1	0	0	0
ENSMUSG00000083832	GM13771	0	0	0	0	0	1	0	0	0	0	0	1
ENSMUSG00000083833	GM13841	0	0	0	0	0	1	0	0	0	0	0	0
ENSMUSG00000083837	GM14427	2	1	2	4	1	1	1	3	1	3	2	0
ENSMUSG00000083841	GM1947	0	0	0	0	0	0	2	4	0	1	1	1
ENSMUSG00000083844	UBE2D-PS	1	0	3	2	1	2	13	7	10	10	11	10
ENSMUSG00000083849	GM13477	0	0	0	0	0	0	1	0	0	0	0	0
ENSMUSG00000083853	GM15696	0	0	0	0	1	0	3	4	2	3	1	1
ENSMUSG00000083854	DNAJC19-PS	0	0	0	0	0	0	1	0	0	1	1	0
ENSMUSG00000083857	GM12584	0	0	0	0	0	0	0	1	0	0	0	0

ENSMUSG00000083859	GM12003	0	0	0	0	0	0	0	0	0	1	0	0
ENSMUSG00000083863	GM13341	2	2	3	0	2	2	6	14	3	6	1	5
ENSMUSG00000083864	GM13719	0	1	0	0	0	0	0	0	0	0	0	0
ENSMUSG00000083866	GM13472	0	0	0	0	0	1	0	1	0	0	0	0
ENSMUSG00000083878	GM12034	0	0	0	2	0	0	2	2	1	1	2	1
ENSMUSG00000083879	GM14038	0	0	0	0	0	0	1	0	0	0	0	0
ENSMUSG00000083884	GM15388	0	0	0	0	0	0	1	0	0	0	0	0
ENSMUSG00000083885	GM13213	0	0	0	0	0	0	0	0	1	1	0	0
ENSMUSG00000083887	GM11930	0	0	0	0	1	0	0	0	0	0	0	1
ENSMUSG00000083892	GM1848	0	0	0	0	0	0	0	1	0	0	0	0
ENSMUSG00000083894	GM11489	0	0	1	0	0	0	0	0	0	0	0	0
ENSMUSG00000083897	GM14843	1	0	0	0	1	1	3	1	1	1	2	0
ENSMUSG00000083899	GM12346	3	2	1	3	3	2	4	10	4	4	2	5
ENSMUSG00000083906	GM15186	0	0	1	0	0	0	0	1	2	0	0	2
ENSMUSG00000083910	GM13604	0	0	0	0	0	0	0	1	0	0	0	0
ENSMUSG00000083911	GM4342	1	1	0	0	1	0	2	1	1	2	3	2
ENSMUSG00000083912	GM5391	0	0	0	0	0	0	0	0	1	0	0	1
ENSMUSG00000083914	RPS18-PS1	0	1	0	0	0	2	2	3	2	2	0	2
ENSMUSG00000083916	GM15476	0	0	0	0	0	0	0	0	0	1	0	0
ENSMUSG00000083921	GM15750	0	0	0	1	0	0	0	0	0	0	0	0
ENSMUSG00000083932	GM11438	0	0	0	0	0	0	0	0	1	0	0	0
ENSMUSG00000083943	GM11483	0	0	0	0	0	0	0	2	0	0	2	0
ENSMUSG00000083953	GM14049	1	0	0	0	0	0	0	0	0	1	0	1
ENSMUSG00000083974	GM16053	0	0	0	1	0	0	0	0	0	0	0	1
ENSMUSG00000083982	GM14650	0	0	0	0	0	0	0	0	0	1	0	0
ENSMUSG00000083985	GM12468	0	0	0	0	0	0	0	1	1	1	0	1
ENSMUSG00000083988	RPS10-PS4	0	0	0	0	0	0	0	0	0	1	1	0
ENSMUSG00000083989	GM12607	1	1	3	1	1	1	1	2	0	0	2	0
ENSMUSG00000083992	GM11478	51	65	76	66	62	59	199	194	146	178	151	193
ENSMUSG00000083993	GM14398	0	0	0	0	0	0	0	0	0	0	0	1
ENSMUSG00000083998	PRDX2-RS3	0	0	0	0	0	0	0	1	0	0	0	0
ENSMUSG00000084012	GM11956	0	1	0	2	0	0	0	1	0	0	1	2
ENSMUSG00000084017	RPL21-PS9	0	0	0	0	0	0	1	1	0	0	0	0
ENSMUSG00000084024	GM15937	0	0	0	0	0	0	0	1	1	0	1	0
ENSMUSG00000084038	GM11295	0	0	0	0	0	0	1	0	0	0	0	0
ENSMUSG00000084048	GM12042	0	0	0	1	0	0	0	0	0	1	1	1
ENSMUSG00000084050	GM14439	0	1	1	1	1	2	3	5	1	6	2	4
ENSMUSG00000084067	GM14269	0	0	0	0	0	0	0	2	0	0	0	0
ENSMUSG00000084072	GM11285	0	0	0	0	0	0	0	0	1	0	0	0
ENSMUSG00000084079	GM11631	0	0	0	0	0	0	0	0	1	0	0	1
ENSMUSG00000084081	GM12057	0	0	0	1	0	0	0	0	0	1	0	0
ENSMUSG00000084088	GM12941	0	0	0	0	0	0	0	0	1	0	0	0
ENSMUSG00000084093	GM16418	0	0	0	0	0	0	0	0	1	0	0	2
ENSMUSG00000084094	GM13961	1	0	0	0	0	0	0	0	0	0	1	0
ENSMUSG00000084098	GM13422	1	0	0	1	0	0	1	1	0	2	0	0
ENSMUSG00000084105	GM13252	0	0	0	0	0	0	0	0	0	0	0	1
ENSMUSG00000084106	GM6136	8	7	6	8	8	5	24	26	18	26	19	22
ENSMUSG00000084111	GM15710	18	28	28	41	31	29	76	87	75	101	81	79
ENSMUSG00000084113	GM14277	0	0	0	0	1	0	0	0	0	0	0	1
ENSMUSG00000084116	GM12460	0	0	0	0	0	0	0	0	1	0	0	0
ENSMUSG00000084124	GM13588	5	6	5	5	5	4	44	51	39	54	40	38
ENSMUSG00000084128	ESRP2	0	3	1	1	1	3	12	7	7	10	7	12
ENSMUSG00000084129	HMGB1-PS1	0	0	0	0	0	1	0	0	0	0	0	0
ENSMUSG00000084131	RPL3-PS2	0	0	0	0	1	1	0	2	0	1	0	3
ENSMUSG00000084132	GM15982	0	0	0	0	0	1	0	0	0	0	1	1
ENSMUSG00000084137	GM9085	0	0	0	0	0	0	0	1	0	0	0	0
ENSMUSG00000084144	GM12516	0	0	0	0	0	0	1	0	0	0	0	0
ENSMUSG00000084149	GM12944	0	0	0	0	1	0	1	0	0	0	0	0

ENSMUSG00000084154	GM15644	0	0	0	1	0	0	0	0	0	0	0	0
ENSMUSG00000084159	GM12696	0	0	0	1	0	0	0	0	0	2	2	0
ENSMUSG00000084166	GM6451	0	1	0	1	0	0	1	1	2	3	4	2
ENSMUSG00000084168	ATP5L2-PS	0	0	1	0	0	0	1	3	1	0	0	0
ENSMUSG00000084178	GM11759	0	1	1	0	2	2	4	5	4	4	2	6
ENSMUSG00000084184	GM13474	0	1	0	0	0	0	0	0	0	0	0	0
ENSMUSG00000084185	RPLP1-PS1	0	0	0	0	0	0	0	0	0	0	0	1
ENSMUSG00000084186	RPL36-PS10	2	2	2	4	3	2	7	8	8	11	10	5
ENSMUSG00000084190	GM11869	0	0	0	0	0	0	0	0	0	0	1	0
ENSMUSG00000084208	GM12792	0	0	0	1	0	0	0	0	0	0	0	0
ENSMUSG00000084221	HMGB1-PS4	0	0	0	0	0	0	1	2	0	0	1	0
ENSMUSG00000084224	GM12337	0	0	0	0	0	0	1	0	0	0	0	0
ENSMUSG00000084235	GM15421	0	0	0	0	0	0	0	1	0	0	0	0
ENSMUSG00000084247	GM14566	0	0	0	0	0	1	0	0	0	0	0	0
ENSMUSG00000084249	GM12267	0	0	0	0	0	0	0	0	0	1	0	0
ENSMUSG00000084252	GM12036	0	0	0	0	0	0	0	0	0	0	1	0
ENSMUSG00000084265	GM14541	0	0	0	0	0	0	1	0	0	1	0	0
ENSMUSG00000084273	GM12628	0	0	0	0	0	0	0	1	0	0	0	0
ENSMUSG00000084274	GM12504	21	27	23	21	23	29	89	76	46	82	55	70
ENSMUSG00000084284	TPT1-PS6	0	0	0	0	0	0	0	0	0	0	1	0
ENSMUSG00000084291	GM6654	0	1	1	0	0	0	1	1	1	0	0	0
ENSMUSG00000084292	GM13535	0	0	0	2	0	2	1	0	1	1	0	1
ENSMUSG00000084299	GM6630	0	0	0	0	0	0	0	0	0	0	2	0
ENSMUSG00000084304	GM6142	0	0	0	0	0	0	0	0	0	0	0	2
ENSMUSG00000084306	GM11743	0	0	0	0	0	0	0	2	0	0	0	0
ENSMUSG00000084307	GM12151	0	0	0	0	1	0	0	0	0	0	0	0
ENSMUSG00000084314	RPS15A-PS3	0	0	0	0	0	0	0	0	0	1	0	0
ENSMUSG00000084319	TPT1-PS3	0	1	0	0	0	0	0	2	1	2	0	1
ENSMUSG00000084320	GM14125	0	0	0	0	0	0	0	0	0	0	1	0
ENSMUSG00000084323	GM14438	1	1	0	5	1	3	1	3	1	1	4	5
ENSMUSG00000084325	GM15550	0	0	1	0	0	0	0	0	0	0	0	0
ENSMUSG00000084329	GM6733	0	0	0	1	0	0	0	0	0	0	0	0
ENSMUSG00000084331	GM8163	0	0	0	0	0	0	0	1	1	0	0	0
ENSMUSG00000084333	GM11599	0	0	0	0	0	0	1	1	0	0	0	0
ENSMUSG00000084340	GM14766	0	0	0	0	0	0	1	0	0	0	0	0
ENSMUSG00000084342	GM11224	0	0	0	0	1	0	0	2	0	0	0	1
ENSMUSG00000084345	RPL17-PS4	1	0	1	0	0	0	1	0	1	0	0	0
ENSMUSG00000084348	GM14608	0	1	1	0	0	0	2	0	1	2	3	2
ENSMUSG00000084349	RPL3-PS1	4	7	4	7	6	9	34	31	21	27	24	26
ENSMUSG00000084368	GM11572	0	0	0	0	0	1	0	0	0	0	0	0
ENSMUSG00000084373	RPS2-PS4	0	0	0	0	0	0	0	0	0	0	1	0
ENSMUSG00000084381	AA413626	0	0	0	0	0	0	1	1	1	1	0	0
ENSMUSG00000084383	GM13370	0	0	0	0	0	0	1	0	0	0	0	0
ENSMUSG00000084384	GM12251	0	0	0	0	0	0	0	0	0	1	0	0
ENSMUSG00000084403	RPS15A-PS8	0	2	1	0	0	0	0	1	1	0	1	0
ENSMUSG00000084411	GM11510	1	0	0	3	0	1	0	1	0	0	0	0
ENSMUSG00000084413	GM12017	0	0	0	0	0	0	0	1	1	0	0	1
ENSMUSG00000084416	RPL10A-PS1	21	39	31	42	31	30	100	117	73	103	102	109
ENSMUSG00000084417	GM23463	0	0	0	0	0	1	0	0	1	0	0	0
ENSMUSG00000084440	GM26254	0	0	0	0	0	0	0	0	0	1	0	0
ENSMUSG00000084453	GM24596	3	0	3	1	0	0	2	3	1	1	1	4
ENSMUSG00000084497	GM22107	0	0	0	0	0	1	2	1	1	1	0	1
ENSMUSG00000084593	GM22345	0	0	0	0	0	0	0	0	0	0	1	0
ENSMUSG00000084607	GM22208	0	0	0	0	1	0	1	3	0	1	3	1
ENSMUSG00000084625	GM25544	0	0	0	0	0	1	0	1	2	0	1	0
ENSMUSG00000084641	N-R5S21	0	0	0	0	0	0	0	0	0	0	1	0
ENSMUSG00000084648	GM24378	0	0	0	0	0	0	1	1	1	0	0	1
ENSMUSG00000084653	GM26091	0	0	0	0	0	0	0	0	1	0	0	0

ENSMUSG00000084686	GM22027	0	0	0	0	0	0	1	0	0	0	0	0
ENSMUSG00000084693	GM24894	0	0	0	0	0	0	0	0	0	1	0	0
ENSMUSG00000084739	GM22067	0	0	0	0	0	0	1	2	1	0	2	0
ENSMUSG00000084744	GM25291	0	0	0	0	0	1	0	1	1	1	0	1
ENSMUSG00000084755	GM16276	0	0	0	0	1	0	0	0	1	0	0	0
ENSMUSG00000084756	GM13881	0	0	0	0	0	0	1	0	0	0	0	0
ENSMUSG00000084760	GM15472	0	0	0	0	2	1	2	2	2	1	1	1
ENSMUSG00000084766	GM12714	0	0	0	0	0	1	0	0	0	0	0	0
ENSMUSG00000084771	A230072E10F	0	0	0	0	1	1	0	3	0	0	1	2
ENSMUSG00000084772	GM15473	0	0	0	1	0	0	0	0	0	0	0	0
ENSMUSG00000084775	GM16741	1	0	0	0	0	0	2	0	0	0	0	0
ENSMUSG00000084786	UBL5	11	13	16	14	15	21	132	130	108	122	109	114
ENSMUSG00000084790	GM15879	2	2	2	1	0	1	3	5	1	1	1	6
ENSMUSG00000084792	1700056N10I	1	2	3	1	0	0	3	7	4	5	10	6
ENSMUSG00000084796	MIR142HG	5	11	12	7	12	8	34	40	44	37	29	37
ENSMUSG00000084799	INO80DOS	5	7	1	4	2	2	18	13	8	17	10	12
ENSMUSG00000084803	5830444B04F	1	1	5	2	4	2	14	10	7	11	5	6
ENSMUSG00000084806	GM15232	0	0	0	0	0	0	1	2	0	3	1	0
ENSMUSG00000084808	9430091E24F	1	0	0	0	0	0	3	2	0	2	0	1
ENSMUSG00000084817	GM5526	0	1	1	0	0	0	1	0	1	0	0	0
ENSMUSG00000084821	GM15880	0	0	0	0	0	0	0	0	2	1	0	0
ENSMUSG00000084830	GM14539	1	0	4	2	3	2	11	11	8	4	7	16
ENSMUSG00000084843	B230312C02F	2	0	0	1	0	1	1	1	0	0	2	1
ENSMUSG00000084845	TMEM240	0	0	0	1	0	0	0	2	3	3	0	0
ENSMUSG00000084846	A730011C13F	1	1	1	0	1	1	2	3	6	3	1	4
ENSMUSG00000084849	GM16105	0	0	0	0	0	0	0	0	0	0	0	1
ENSMUSG00000084862	GM16278	1	0	0	0	0	0	0	0	0	0	0	0
ENSMUSG00000084864	1700027A07F	2	5	4	4	5	3	2	13	5	7	9	4
ENSMUSG00000084866	A930006K02F	2	0	1	0	2	1	9	4	5	10	2	7
ENSMUSG00000084878	LRRC8DOS	0	2	0	0	0	0	1	0	1	0	0	0
ENSMUSG00000084880	TOMM6OS	1	0	0	0	2	1	0	0	0	1	0	0
ENSMUSG00000084884	GM12289	0	0	1	0	0	0	0	0	0	0	0	0
ENSMUSG00000084904	GM14827	0	0	1	0	0	0	0	2	0	1	2	2
ENSMUSG00000084906	GM13794	0	0	0	0	0	0	0	0	0	0	1	0
ENSMUSG00000084908	C79798	0	0	0	0	0	0	0	0	0	1	0	0
ENSMUSG00000084911	GM16185	0	0	0	0	0	1	2	0	1	4	1	2
ENSMUSG00000084915	C230037L18F	0	0	0	0	0	0	0	2	1	4	0	1
ENSMUSG00000084917	GM17477	6	4	0	1	1	2	4	2	4	6	2	7
ENSMUSG00000084923	GM15611	0	0	0	0	0	0	0	1	0	0	0	0
ENSMUSG00000084925	1810062O18I	0	0	0	0	0	0	0	0	2	0	0	0
ENSMUSG00000084930	GM11706	1	1	0	0	0	0	0	0	2	2	1	0
ENSMUSG00000084936	GM11583	3	8	7	2	7	4	9	266	8	29	6	15
ENSMUSG00000084938	BB557941	0	0	0	1	0	1	0	1	0	2	0	2
ENSMUSG00000084941	GM11944	0	0	0	0	1	0	1	3	1	2	0	1
ENSMUSG00000084946	DLX1AS	1	2	0	1	2	0	1	6	1	5	3	1
ENSMUSG00000084947	GM15594	0	0	0	0	0	0	0	1	0	0	0	0
ENSMUSG00000084950	GM5577	0	0	0	0	0	0	0	0	0	1	0	0
ENSMUSG00000084957	BBIP1	3	2	3	5	1	1	12	13	15	15	15	13
ENSMUSG00000084968	GM12743	0	0	1	1	0	0	1	3	0	0	1	0
ENSMUSG00000084974	GM15567	0	0	0	0	0	0	0	1	1	0	0	0
ENSMUSG00000084978	GM11655	0	0	0	0	0	0	0	0	0	3	0	0
ENSMUSG00000084983	GM11789	0	0	0	0	0	0	0	0	1	0	0	0
ENSMUSG00000084984	FAR1OS	1	0	0	0	0	0	0	0	0	0	0	0
ENSMUSG00000084985	GM16135	0	1	0	0	0	0	0	0	0	1	1	0
ENSMUSG00000084986	GM16224	0	0	0	0	0	0	0	0	1	0	0	0
ENSMUSG00000084990	GM14549	0	0	0	0	0	0	1	2	1	1	0	0
ENSMUSG00000084992	GM13842	0	0	0	0	0	0	0	0	0	0	1	0
ENSMUSG00000084998	GM16279	0	0	0	0	0	1	1	0	0	1	1	0

ENSMUSG00000085023	GM12744	10	16	11	12	8	8	23	28	26	44	34	36
ENSMUSG00000085024	C230035I16R	0	0	0	0	0	0	3	0	2	3	2	3
ENSMUSG00000085028	SLC2A4RG-PS	1	3	2	1	2	3	9	17	15	11	11	14
ENSMUSG00000085030	2810455O05I	1	3	2	0	3	3	7	13	16	16	10	15
ENSMUSG00000085035	GM12031	0	0	0	0	0	0	0	0	1	1	0	0
ENSMUSG00000085037	4933421O10I	0	1	0	0	0	0	4	5	0	9	6	3
ENSMUSG00000085039	GM15927	0	0	0	1	0	0	2	0	1	0	1	1
ENSMUSG00000085042	ABHD11OS	0	0	0	0	0	0	1	1	0	1	0	0
ENSMUSG00000085048	GM15537	0	0	0	0	0	0	0	0	2	1	2	1
ENSMUSG00000085054	GM15834	2	2	0	3	1	4	6	3	1	4	2	6
ENSMUSG00000085063	GM16024	1	2	0	0	0	1	8	1	1	6	0	4
ENSMUSG00000085072	ICT1OS	0	0	0	0	0	0	1	0	0	0	0	0
ENSMUSG00000085078	C030013C21F	0	1	0	1	0	0	0	0	0	0	0	1
ENSMUSG00000085087	GM13528	0	0	0	0	0	0	0	0	0	0	0	1
ENSMUSG00000085094	GM15692	0	0	0	0	0	0	0	0	0	0	0	1
ENSMUSG00000085099	GM16288	1	0	0	0	0	0	0	0	0	0	1	1
ENSMUSG00000085102	1700010K24F	0	0	0	0	0	0	0	1	0	0	0	0
ENSMUSG00000085105	GM12758	1	1	0	1	1	1	2	1	1	0	1	2
ENSMUSG00000085112	A530072M11	0	0	1	1	0	0	0	0	0	2	0	0
ENSMUSG00000085116	GM2735	0	0	0	0	0	0	1	0	0	0	0	0
ENSMUSG00000085133	B930095G15I	1	1	3	2	2	0	4	7	5	8	4	5
ENSMUSG00000085134	GM11659	2	1	2	4	4	2	1	4	3	3	5	6
ENSMUSG00000085143	GM11520	0	0	0	0	0	0	0	0	0	1	1	1
ENSMUSG00000085147	GM12609	0	0	0	0	0	0	0	0	0	0	1	0
ENSMUSG00000085148	MIR22HG	0	0	0	0	1	0	1	1	1	2	6	0
ENSMUSG00000085152	GM11496	0	0	0	0	0	0	1	0	1	0	0	0
ENSMUSG00000085154	C130046K22F	0	0	0	0	0	0	0	0	0	1	0	0
ENSMUSG00000085156	SNHG15	19	20	20	29	28	21	134	148	101	128	108	97
ENSMUSG00000085166	GM15522	1	0	1	4	1	3	10	10	5	12	10	4
ENSMUSG00000085169	GM10785	0	1	0	0	0	0	0	1	0	1	0	2
ENSMUSG00000085172	GM6542	0	1	0	0	2	0	1	2	0	0	2	2
ENSMUSG00000085174	GM16206	0	0	0	0	0	0	1	0	0	0	0	0
ENSMUSG00000085175	GM11423	0	1	0	1	0	1	3	1	0	2	3	6
ENSMUSG00000085177	GM11209	0	0	0	0	0	0	1	0	1	0	0	0
ENSMUSG00000085180	AI838599	0	0	0	0	0	0	0	0	0	0	1	0
ENSMUSG00000085182	GM13596	1	0	0	0	0	0	0	1	0	0	0	0
ENSMUSG00000085185	BC028777	0	0	0	0	0	0	0	0	1	0	0	0
ENSMUSG00000085189	GM11963	0	0	0	0	0	0	0	1	0	0	0	1
ENSMUSG00000085201	NR6A1OS	1	0	0	0	0	0	1	0	0	0	0	0
ENSMUSG00000085204	GM15327	0	0	0	0	0	0	0	0	0	1	1	3
ENSMUSG00000085206	GM13380	0	0	0	0	0	1	0	2	1	0	0	1
ENSMUSG00000085207	GM11767	0	0	0	0	0	0	2	0	0	0	0	0
ENSMUSG00000085208	BRIP1OS	5	6	4	4	5	8	29	29	32	38	27	31
ENSMUSG00000085211	B430219N15I	0	0	0	0	0	0	0	0	0	1	0	0
ENSMUSG00000085213	GM13091	0	0	0	0	0	0	2	0	0	0	0	0
ENSMUSG00000085227	6330418K02F	1	1	1	1	1	0	4	10	2	9	6	1
ENSMUSG00000085229	GM11672	2	2	2	2	1	3	3	2	1	1	3	4
ENSMUSG00000085234	GM15614	0	0	0	0	0	0	1	0	2	1	0	1
ENSMUSG00000085236	2610206C17F	0	0	0	0	0	0	0	1	0	0	0	0
ENSMUSG00000085238	4930479D17I	0	0	1	0	0	0	0	0	0	0	0	0
ENSMUSG00000085241	SNHG3	29	40	37	34	48	36	240	241	198	282	287	244
ENSMUSG00000085242	GM11537	0	0	1	1	0	0	0	0	0	0	1	0
ENSMUSG00000085245	GM11713	2	1	0	0	0	0	3	4	0	2	2	2
ENSMUSG00000085248	GM11587	0	0	0	0	0	0	0	0	0	1	0	0
ENSMUSG00000085255	TACO1OS	0	0	0	0	0	0	1	2	0	3	1	0
ENSMUSG00000085256	DMRTA2OS	0	0	0	0	0	0	1	0	0	0	2	0
ENSMUSG00000085260	MED9OS	0	0	0	0	0	1	0	0	0	2	0	0
ENSMUSG00000085262	GM11574	0	0	1	0	3	0	4	0	1	0	1	0

ENSMUSG00000085278	GM12841	0	1	1	0	0	1	2	3	1	3	2	1
ENSMUSG00000085279	GM15965	0	0	0	0	0	0	0	0	0	1	0	0
ENSMUSG00000085286	UBE4BOS3	0	0	0	0	0	0	0	0	0	0	0	1
ENSMUSG00000085303	GM16751	0	0	0	0	0	0	1	0	0	0	0	0
ENSMUSG00000085304	GM12802	1	1	1	0	2	1	1	0	1	1	1	1
ENSMUSG00000085310	GM11491	1	0	0	0	0	1	5	3	4	0	1	1
ENSMUSG00000085311	GM15445	1	0	0	0	1	0	0	2	0	1	0	2
ENSMUSG00000085314	GM14866	0	0	0	0	0	0	0	1	0	0	0	1
ENSMUSG00000085315	A430018G15I	0	0	0	0	0	0	2	2	0	0	2	2
ENSMUSG00000085321	4930412F09R	0	0	0	0	0	0	0	0	1	0	0	0
ENSMUSG00000085326	GM15642	0	1	0	2	0	1	5	1	2	2	2	2
ENSMUSG00000085327	GM16104	0	0	0	0	0	0	0	0	0	0	0	1
ENSMUSG00000085334	GM12940	27	18	27	26	25	20	167	179	126	222	112	145
ENSMUSG00000085337	GM15964	1	1	0	6	4	4	14	7	10	16	14	14
ENSMUSG00000085340	GM16335	0	0	1	0	1	0	0	0	0	0	0	0
ENSMUSG00000085342	GM12254	0	0	0	1	0	1	1	1	0	4	5	0
ENSMUSG00000085347	HK1OS	0	0	0	0	0	0	0	0	0	0	1	2
ENSMUSG00000085352	GM4128	0	0	0	0	0	0	0	0	0	0	0	1
ENSMUSG00000085355	3010003L21R	2	1	1	2	0	1	2	2	2	3	2	4
ENSMUSG00000085361	GM16305	0	0	0	0	0	0	0	0	1	0	0	0
ENSMUSG00000085371	GM3160	0	0	0	0	0	0	1	0	0	0	0	0
ENSMUSG00000085376	GM14508	0	0	0	0	0	0	0	2	0	0	0	0
ENSMUSG00000085379	2310058D17I	0	0	2	0	1	0	6	3	4	1	2	7
ENSMUSG00000085385	SNHG17	6	9	5	4	9	5	32	32	28	34	32	26
ENSMUSG00000085386	GM13630	0	0	0	0	1	0	0	0	0	0	0	0
ENSMUSG00000085396	FIRRE	6	6	7	8	18	12	48	64	41	55	45	41
ENSMUSG00000085399	FOXD2OS	5	10	18	23	18	9	43	52	41	65	54	54
ENSMUSG00000085408	C530005A16I	0	0	0	0	0	0	0	0	0	2	1	0
ENSMUSG00000085412	HALR1	0	1	0	0	0	0	0	0	0	0	0	0
ENSMUSG00000085414	SSPNOS	0	0	1	1	0	1	1	2	0	3	0	1
ENSMUSG00000085415	SELENOK-PS1	0	0	0	0	1	0	2	2	2	4	1	1
ENSMUSG00000085423	GM15735	0	0	1	0	0	0	0	1	0	0	0	1
ENSMUSG00000085431	4930440I19R	0	0	0	0	1	0	2	0	2	1	4	3
ENSMUSG00000085433	GM16001	0	0	0	0	0	1	1	2	2	7	4	5
ENSMUSG00000085434	GM11725	0	0	0	0	0	0	0	0	0	0	1	0
ENSMUSG00000085436	ZFP335OS	0	0	0	2	0	0	1	0	1	2	2	3
ENSMUSG00000085437	1700047A11I	0	0	0	0	0	0	0	0	0	1	0	0
ENSMUSG00000085438	1700020I14R	53	44	40	59	40	29	158	176	120	179	119	110
ENSMUSG00000085442	GM3362	11	7	13	15	19	10	37	21	30	39	25	33
ENSMUSG00000085445	GM16348	0	0	0	0	0	0	0	0	0	0	1	0
ENSMUSG00000085457	1110046J04R	0	0	0	1	0	0	0	0	1	1	3	0
ENSMUSG00000085471	4933423P22R	0	0	0	0	0	0	0	0	1	0	0	0
ENSMUSG00000085482	A230083N12I	0	0	0	0	0	1	0	1	1	0	0	0
ENSMUSG00000085485	GM14024	0	0	0	0	0	1	3	5	2	3	1	2
ENSMUSG00000085492	TRMT61B	1	1	2	1	1	0	8	7	5	14	8	5
ENSMUSG00000085495	GM16796	0	0	0	0	0	0	1	0	0	0	0	0
ENSMUSG00000085504	GM14055	0	1	0	0	0	0	0	0	0	0	0	0
ENSMUSG00000085513	GM16363	0	0	0	0	0	0	0	1	0	0	0	0
ENSMUSG00000085527	GM15535	0	0	2	0	0	0	0	2	0	1	1	1
ENSMUSG00000085538	GM11455	0	0	1	0	0	0	0	0	0	0	0	0
ENSMUSG00000085546	GM14252	0	0	1	0	0	0	0	1	0	0	0	0
ENSMUSG00000085555	2610035F20R	0	0	0	0	0	1	0	0	0	1	0	1
ENSMUSG00000085558	4930412C18R	0	0	0	0	0	0	0	5	7	4	3	1
ENSMUSG00000085566	A730017L22R	0	0	0	0	1	0	0	1	3	2	4	0
ENSMUSG00000085568	GM14858	0	0	0	0	0	0	0	1	0	0	0	0
ENSMUSG00000085572	GM16234	0	0	0	0	0	0	0	0	0	1	0	2
ENSMUSG00000085578	GM11527	0	0	0	0	1	0	0	0	0	0	0	0
ENSMUSG00000085582	3110099E03R	0	0	0	0	1	0	0	0	0	0	0	0

ENSMUSG00000085585	GM12223	0	0	0	0	0	0	0	0	0	1	0	0
ENSMUSG00000085586	GM11613	0	0	0	0	0	0	1	0	0	2	0	1
ENSMUSG00000085589	A430078I02R	0	0	1	0	0	0	0	0	0	0	0	0
ENSMUSG00000085596	GM11476	0	0	0	0	0	0	0	1	0	0	0	0
ENSMUSG00000085603	GM11346	0	0	0	2	1	0	1	2	3	3	1	0
ENSMUSG00000085606	GM15792	0	0	0	0	0	0	1	0	0	1	0	0
ENSMUSG00000085612	GM15868	0	0	0	0	0	0	0	0	0	1	0	0
ENSMUSG00000085614	1700123M08	0	0	0	1	0	0	0	0	0	1	0	3
ENSMUSG00000085615	A330035P11F	0	0	0	1	0	0	0	0	0	1	0	1
ENSMUSG00000085620	G630018N14	0	0	0	0	0	0	1	0	0	0	0	0
ENSMUSG00000085622	3110056K07F	3	1	0	4	0	1	6	13	6	13	8	12
ENSMUSG00000085626	GM12898	1	0	0	0	0	0	1	3	1	2	0	1
ENSMUSG00000085628	APPBP2OS	0	0	0	0	0	0	0	1	0	1	0	1
ENSMUSG00000085633	BLOC1S6OS	1	0	0	0	0	0	1	0	1	0	1	0
ENSMUSG00000085634	GM15290	0	0	0	0	0	0	0	0	1	0	0	0
ENSMUSG00000085652	GM11999	0	1	0	0	0	0	0	0	0	0	0	0
ENSMUSG00000085655	GM15952	0	1	4	1	1	0	5	7	2	7	2	4
ENSMUSG00000085665	GM12059	1	0	0	0	0	0	0	0	0	0	0	0
ENSMUSG00000085667	GM12992	3	1	3	2	3	1	6	10	13	13	13	10
ENSMUSG00000085684	4930469K13F	0	0	0	0	0	0	0	1	0	0	0	0
ENSMUSG00000085687	GM16153	0	0	0	0	1	1	4	4	5	0	4	2
ENSMUSG00000085689	GM16838	0	0	0	0	0	0	1	0	0	0	0	0
ENSMUSG00000085704	4921531C22F	0	0	0	0	0	0	0	0	1	3	0	0
ENSMUSG00000085714	GM13008	0	0	0	0	0	0	3	1	0	3	2	0
ENSMUSG00000085720	GM7854	0	0	0	1	0	0	0	0	0	0	0	0
ENSMUSG00000085733	ARHGAP27OS	0	0	0	0	0	0	1	1	1	1	1	2
ENSMUSG00000085735	METTL5OS	2	6	7	7	6	5	28	30	19	29	13	16
ENSMUSG00000085741	5430405H02F	0	3	1	8	4	4	10	3	9	10	3	11
ENSMUSG00000085745	GM14372	0	1	0	0	0	0	0	0	0	0	0	0
ENSMUSG00000085749	GM12843	0	0	0	0	0	0	0	1	0	0	0	0
ENSMUSG00000085754	GM15886	0	0	0	0	0	0	0	1	0	0	0	0
ENSMUSG00000085760	GM14302	0	0	0	0	0	0	1	0	1	0	0	0
ENSMUSG00000085762	LRRC75AOS2	0	0	0	0	0	0	1	0	0	0	1	0
ENSMUSG00000085763	SMC2OS	0	0	0	1	0	0	0	0	0	0	0	0
ENSMUSG00000085765	GM12333	0	0	0	0	0	0	0	0	1	0	0	0
ENSMUSG00000085767	GM13563	0	0	0	0	0	1	0	1	1	0	1	2
ENSMUSG00000085771	C230066G23I	0	0	0	0	0	0	1	0	1	2	1	1
ENSMUSG00000085774	GM13055	0	0	2	1	0	0	0	0	0	2	1	2
ENSMUSG00000085776	5430402O13I	0	0	0	0	0	1	0	0	0	0	0	0
ENSMUSG00000085779	ATCAYOS	0	0	0	0	0	1	0	0	0	1	0	0
ENSMUSG00000085787	GM13092	2	1	1	3	0	0	1	2	9	3	2	6
ENSMUSG00000085791	RPL30-PS9	0	0	0	0	0	0	0	0	0	1	1	0
ENSMUSG00000085793	LIN52	4	3	1	3	3	2	17	18	14	16	6	13
ENSMUSG00000085794	VAX2OS	0	0	0	0	0	0	0	1	0	0	0	0
ENSMUSG00000085795	ZFP703	2	0	0	0	0	2	4	2	3	3	1	0
ENSMUSG00000085808	GM14718	0	1	1	1	1	0	5	6	4	2	2	7
ENSMUSG00000085810	GM16325	0	0	0	0	0	0	0	0	0	1	0	0
ENSMUSG00000085811	CEP112IT	0	0	0	0	1	0	0	0	0	0	0	0
ENSMUSG00000085812	GM15843	0	0	0	0	0	0	0	1	1	0	0	3
ENSMUSG00000085818	GM13267	0	0	0	0	0	1	0	0	0	0	0	0
ENSMUSG00000085822	GM15584	0	0	0	1	0	0	0	0	0	0	0	0
ENSMUSG00000085829	GM4285	0	1	0	0	0	0	2	0	0	0	1	1
ENSMUSG00000085847	GM14540	0	0	0	0	0	1	0	0	1	0	1	0
ENSMUSG00000085853	GM12167	0	1	0	0	0	0	0	2	1	1	0	0
ENSMUSG00000085857	GM15392	0	0	0	0	0	0	0	0	0	0	0	1
ENSMUSG00000085861	1700016G14I	0	0	0	0	0	0	0	1	0	0	0	0
ENSMUSG00000085862	GM13483	0	0	0	0	0	0	1	0	0	0	0	0
ENSMUSG00000085865	GM15966	0	0	1	0	0	0	0	1	0	0	0	0

ENSMUSG00000085873	TTC39AOS1	0	0	0	0	0	0	0	0	0	3	0	0
ENSMUSG00000085875	GM12905	0	1	0	0	0	0	3	4	1	3	0	0
ENSMUSG00000085881	GM15912	0	0	0	0	1	0	0	0	0	0	0	0
ENSMUSG00000085882	2610507I01R	4	1	1	0	0	0	5	5	3	6	4	5
ENSMUSG00000085884	GM15342	0	0	0	0	0	1	0	0	0	0	0	0
ENSMUSG00000085885	GM11906	0	0	0	0	0	0	0	1	1	1	0	0
ENSMUSG00000085887	ARHGAP27OS	0	0	0	0	0	1	0	0	0	1	0	0
ENSMUSG00000085893	GM12091	0	0	0	0	0	0	1	0	0	1	2	0
ENSMUSG00000085894	GM15832	3	4	1	2	2	4	13	10	8	11	5	15
ENSMUSG00000085897	GM11604	0	0	0	0	0	0	1	0	0	1	0	1
ENSMUSG00000085913	GM15601	6	0	0	3	0	1	4	5	4	3	2	4
ENSMUSG00000085929	GM13421	0	0	0	1	0	1	0	2	4	2	1	1
ENSMUSG00000085936	2610307P16R	0	2	0	0	0	0	2	2	3	4	4	4
ENSMUSG00000085952	GM12315	0	0	0	0	0	0	0	0	0	1	0	0
ENSMUSG00000085955	GM12356	0	0	0	0	0	0	0	0	0	0	1	0
ENSMUSG00000085956	4930481B07R	0	0	0	0	0	0	1	1	1	0	2	1
ENSMUSG00000085962	GM16984	0	0	0	1	0	0	0	0	0	0	1	1
ENSMUSG00000085965	2310016D23R	0	1	0	0	0	0	1	1	2	1	1	0
ENSMUSG00000085972	1110028F11R	0	0	0	0	0	0	0	1	0	0	0	0
ENSMUSG00000085975	GM13572	0	0	0	0	0	0	0	1	0	1	1	1
ENSMUSG00000085982	9530051G07R	0	0	0	0	0	0	0	0	0	1	0	0
ENSMUSG00000085984	1700001G11R	0	0	0	0	0	0	0	0	1	0	0	0
ENSMUSG00000085990	GM16731	2	2	0	3	1	8	12	13	15	22	5	15
ENSMUSG00000085995	GM2788	0	0	0	0	0	0	0	0	0	1	0	0
ENSMUSG00000086001	GM13448	0	0	0	1	0	0	0	0	0	0	0	0
ENSMUSG00000086003	B230206L02R	0	0	0	0	0	0	0	0	0	1	0	0
ENSMUSG00000086006	GM13293	0	0	0	0	0	0	0	1	0	0	0	0
ENSMUSG00000086008	GM8817	0	0	0	0	0	0	1	1	1	0	0	0
ENSMUSG00000086013	GM15706	0	0	0	0	0	0	1	0	0	0	0	0
ENSMUSG00000086015	4833417C18R	0	1	0	0	1	0	5	1	0	1	1	4
ENSMUSG00000086016	1700084C06R	0	0	0	0	0	1	0	0	0	0	0	1
ENSMUSG00000086018	GM13179	0	0	0	0	0	0	0	0	0	0	1	1
ENSMUSG00000086035	GM12610	0	0	0	0	0	0	0	0	0	2	1	1
ENSMUSG00000086039	GM12227	0	0	0	0	0	0	1	0	0	0	0	0
ENSMUSG00000086044	A330043C09R	0	0	1	0	0	0	0	0	0	0	0	0
ENSMUSG00000086047	9530046B11R	0	0	0	0	0	0	0	0	0	1	0	0
ENSMUSG00000086052	GM11802	0	0	0	0	0	0	0	0	0	1	0	0
ENSMUSG00000086054	HNF1AOS1	0	0	0	0	0	0	1	0	0	0	0	1
ENSMUSG00000086068	1700120G11R	2	2	2	1	3	1	4	6	10	6	12	10
ENSMUSG00000086074	GM13274	0	1	0	0	0	0	0	0	1	0	1	0
ENSMUSG00000086075	GM15728	0	0	0	0	0	0	0	0	1	1	0	0
ENSMUSG00000086080	1700123O12R	0	0	0	0	1	0	0	0	0	0	0	0
ENSMUSG00000086083	GM15829	0	0	0	0	0	0	1	0	0	0	0	0
ENSMUSG00000086090	GM12531	3	0	0	3	1	1	7	1	4	5	5	3
ENSMUSG00000086105	GM11636	0	0	0	0	0	0	0	0	1	0	1	1
ENSMUSG00000086109	GM13391	0	0	0	0	0	0	1	0	0	0	0	0
ENSMUSG00000086111	GM15326	0	0	0	0	0	0	2	0	2	0	1	1
ENSMUSG00000086112	4930563I02R	0	0	0	0	0	0	0	0	0	1	0	0
ENSMUSG00000086118	GM14169	0	0	0	0	0	0	0	0	0	0	0	1
ENSMUSG00000086123	GM16060	0	0	1	0	0	0	0	1	0	0	0	0
ENSMUSG00000086150	BACH2OS	0	0	0	0	1	0	1	0	2	3	0	0
ENSMUSG00000086154	GM16196	0	0	0	0	1	0	0	1	0	1	0	2
ENSMUSG00000086158	CCPG1OS	0	0	0	0	0	0	4	2	0	5	0	2
ENSMUSG00000086161	GM15389	0	0	1	0	0	0	0	1	0	0	0	2
ENSMUSG00000086165	GM15690	0	1	0	0	0	0	0	0	0	0	0	0
ENSMUSG00000086167	GM13827	0	0	0	0	0	1	1	0	1	1	0	1
ENSMUSG00000086173	KIS2	0	0	0	0	1	0	0	1	0	0	0	0
ENSMUSG00000086189	GM15462	1	0	0	0	0	0	0	0	0	0	0	0

ENSMUSG00000086192	GM13609	0	0	0	0	0	0	1	0	0	0	0	0
ENSMUSG00000086193	GM11508	4	1	3	0	1	2	0	5	3	5	0	1
ENSMUSG00000086199	BCAS3OS1	0	0	0	0	0	0	1	1	1	0	0	0
ENSMUSG00000086206	GM13783	0	0	0	0	0	0	0	0	0	0	0	1
ENSMUSG00000086212	MKLN1OS	0	0	0	1	0	0	4	0	2	2	0	0
ENSMUSG00000086213	A330040F15R	3	2	2	9	2	5	16	15	11	11	10	14
ENSMUSG00000086216	GM13610	0	0	0	0	1	0	0	0	1	2	0	1
ENSMUSG00000086234	USP46OS2	0	0	0	0	0	0	0	1	0	0	0	0
ENSMUSG00000086236	5830418P13R	0	0	0	0	0	0	2	1	1	0	0	3
ENSMUSG00000086237	GM15591	0	0	1	0	0	0	1	2	0	0	0	0
ENSMUSG00000086240	GM7846	0	0	0	0	0	1	2	3	4	3	1	1
ENSMUSG00000086241	4930483K19R	0	1	0	0	0	0	0	0	0	1	0	0
ENSMUSG00000086247	GM15787	0	0	0	0	0	0	2	2	0	0	0	0
ENSMUSG00000086255	GM11534	0	0	0	1	0	0	0	0	0	0	0	0
ENSMUSG00000086264	GM15850	0	0	0	0	0	0	0	0	0	0	0	2
ENSMUSG00000086275	1700121C08R	0	0	0	0	0	0	0	0	1	0	0	0
ENSMUSG00000086277	4930558K02R	4	6	4	5	8	8	37	37	35	39	22	26
ENSMUSG00000086279	GM15634	0	0	0	0	0	0	0	1	0	0	0	1
ENSMUSG00000086287	GM15972	0	0	0	0	0	0	0	0	2	1	0	1
ENSMUSG00000086288	GM15265	1	0	0	0	0	0	3	3	1	4	1	3
ENSMUSG00000086289	GM16933	0	0	1	2	0	0	0	1	2	6	1	0
ENSMUSG00000086290	SNHG12	11	17	11	11	21	13	98	93	78	87	88	69
ENSMUSG00000086291	GM15513	0	0	0	0	0	0	0	0	1	0	0	1
ENSMUSG00000086310	GM12278	0	0	0	0	0	0	0	1	0	0	0	1
ENSMUSG00000086316	NBDY	1	2	7	2	5	4	23	22	18	21	23	23
ENSMUSG00000086322	E130218I03R	0	1	2	0	0	1	5	3	4	2	3	2
ENSMUSG00000086324	GM15564	13	12	10	9	14	13	133	117	101	114	89	129
ENSMUSG00000086329	GM14233	0	0	0	0	0	0	0	0	1	0	0	0
ENSMUSG00000086330	1700007J10R	0	1	0	0	0	0	1	0	1	0	0	0
ENSMUSG00000086331	GM16310	0	0	1	0	0	0	0	2	0	0	0	0
ENSMUSG00000086340	1810059C17R	0	0	0	0	0	0	2	0	0	1	0	0
ENSMUSG00000086342	GM12932	0	0	0	0	0	0	2	1	0	2	1	2
ENSMUSG00000086350	B230369F24R	1	2	0	1	0	0	2	2	0	1	1	3
ENSMUSG00000086355	4933428C19R	0	0	0	0	0	0	0	0	0	1	0	0
ENSMUSG00000086358	GM13270	0	0	0	0	0	0	2	0	0	0	0	0
ENSMUSG00000086359	9630013K17R	0	0	0	0	0	0	0	1	0	0	0	0
ENSMUSG00000086368	GM13830	0	1	0	0	0	0	0	0	1	0	1	2
ENSMUSG00000086369	E330017L17R	1	0	0	0	0	0	0	0	0	0	0	0
ENSMUSG00000086370	FTX	1	2	8	6	1	2	15	18	11	14	10	10
ENSMUSG00000086379	1700026D11R	0	2	0	0	0	0	0	0	0	0	1	1
ENSMUSG00000086382	CHRNA1OS	0	1	0	0	1	1	3	4	1	4	1	2
ENSMUSG00000086390	1810019D21R	0	1	0	0	0	0	0	0	0	0	0	0
ENSMUSG00000086391	1700042O10R	0	0	0	0	0	0	0	1	0	0	1	0
ENSMUSG00000086392	MCCC1OS	0	0	0	0	0	0	0	0	0	1	0	0
ENSMUSG00000086395	A630014C17R	1	0	0	0	0	1	1	0	0	1	1	1
ENSMUSG00000086401	GM15559	1	12	1	0	2	4	2	1	0	5	2	1
ENSMUSG00000086416	GM14002	0	0	0	0	0	0	1	0	1	2	0	1
ENSMUSG00000086421	GM14091	0	0	1	0	0	0	1	1	0	1	0	0
ENSMUSG00000086429	GT(ROSA)26S	1	4	3	1	4	4	22	21	22	29	19	21
ENSMUSG00000086433	RSF1OS1	0	0	0	1	0	0	1	0	0	0	0	0
ENSMUSG00000086434	GM15200	0	0	0	0	0	0	0	0	0	1	0	0
ENSMUSG00000086438	ASB17OS	0	0	1	0	0	0	0	0	0	0	0	0
ENSMUSG00000086442	GM11722	0	0	0	1	1	0	0	0	0	0	0	0
ENSMUSG00000086447	GM13522	0	2	1	1	0	0	1	0	0	1	0	1
ENSMUSG00000086448	9330162012R	0	0	0	1	0	0	2	0	1	2	1	0
ENSMUSG00000086453	GM11457	0	0	0	0	0	0	1	2	0	0	1	0
ENSMUSG00000086454	PLATR14	0	0	0	0	0	1	0	0	0	0	0	0
ENSMUSG00000086459	1700030C12R	0	0	0	0	0	0	1	0	0	0	1	0

ENSMUSG00000086468	ETAA1OS	0	1	0	0	0	1	2	3	2	0	0	1
ENSMUSG00000086472	GM16172	0	0	0	0	0	0	0	0	1	0	0	0
ENSMUSG00000086473	GM16265	0	0	0	0	0	0	1	0	0	1	0	0
ENSMUSG00000086477	GM15506	0	0	0	1	0	1	1	1	1	1	0	0
ENSMUSG00000086486	IFT88OS	0	0	0	0	0	0	0	0	0	1	0	0
ENSMUSG00000086493	GM12204	0	0	0	0	0	0	0	0	0	0	0	1
ENSMUSG00000086495	GM13778	0	0	0	0	0	0	1	1	0	6	0	0
ENSMUSG00000086496	GM14204	0	0	0	0	0	0	0	0	0	0	1	0
ENSMUSG00000086502	B130055M24	0	0	0	0	0	0	0	0	0	1	0	0
ENSMUSG00000086504	KIF16BOS	0	0	0	0	0	0	0	0	0	0	1	0
ENSMUSG00000086513	9130208D14	0	0	0	0	0	0	0	1	1	0	0	0
ENSMUSG00000086527	GM15856	1	0	0	0	0	0	0	2	0	2	1	1
ENSMUSG00000086528	GM15731	0	0	0	0	0	0	0	0	1	0	0	0
ENSMUSG00000086533	MYPOPOS	0	0	1	0	0	0	0	0	0	1	0	0
ENSMUSG00000086536	GM12264	0	0	1	0	0	0	0	1	0	0	0	1
ENSMUSG00000086539	GM16759	0	0	0	0	0	0	0	0	1	1	1	0
ENSMUSG00000086544	CHN1OS3	0	3	0	1	0	0	1	5	3	2	1	4
ENSMUSG00000086554	9530034E10	3	0	0	2	0	2	3	2	2	0	2	0
ENSMUSG00000086557	GM15999	0	0	0	0	0	0	0	1	0	1	0	0
ENSMUSG00000086561	GM15540	0	0	0	0	0	0	0	1	0	1	0	0
ENSMUSG00000086567	GM2830	2	0	1	2	0	1	3	1	3	2	2	2
ENSMUSG00000086573	GM15384	0	0	0	1	0	0	0	0	0	0	0	0
ENSMUSG00000086582	GM16272	0	0	0	0	0	0	0	1	0	1	1	1
ENSMUSG00000086583	GM15500	8	11	14	12	9	11	44	41	27	42	38	26
ENSMUSG00000086585	GM16126	0	0	0	0	0	0	0	0	0	1	0	0
ENSMUSG00000086586	GM11192	0	0	0	0	0	0	1	0	0	0	0	0
ENSMUSG00000086593	GM16548	0	0	0	0	0	0	1	0	0	0	0	0
ENSMUSG00000086598	BTBD18	0	0	0	0	0	0	0	0	1	0	0	0
ENSMUSG00000086600	C030005K06	0	0	0	0	1	0	0	0	0	0	1	0
ENSMUSG00000086606	GM13205	0	0	0	0	0	0	2	0	0	1	1	0
ENSMUSG00000086618	GM13256	0	1	0	0	0	0	0	1	0	0	0	1
ENSMUSG00000086632	1700112J05R	2	2	1	4	3	2	7	9	1	5	2	5
ENSMUSG00000086638	4930405A21	0	1	0	0	0	0	0	2	0	1	0	1
ENSMUSG00000086645	GM15743	0	0	0	0	0	0	0	1	0	0	0	1
ENSMUSG00000086657	STAMOS	0	0	0	0	0	0	0	3	2	0	0	1
ENSMUSG00000086670	GM13194	0	0	0	0	0	0	1	0	0	0	0	0
ENSMUSG00000086671	GM13618	0	0	0	0	0	1	0	0	0	0	0	0
ENSMUSG00000086680	GM11592	0	0	0	0	0	1	0	0	0	0	0	0
ENSMUSG00000086682	GM16023	0	0	1	1	1	0	1	0	2	2	0	1
ENSMUSG00000086688	GM11560	0	0	0	1	0	0	1	1	0	0	0	1
ENSMUSG00000086693	A730081D07	0	0	0	0	0	0	0	0	0	1	0	0
ENSMUSG00000086695	GM15247	0	0	0	0	0	0	0	1	0	0	0	0
ENSMUSG00000086697	4933427G23	0	0	0	0	0	0	1	1	0	0	0	0
ENSMUSG00000086700	GM15747	0	0	0	0	0	3	0	0	0	0	1	0
ENSMUSG00000086707	GM12088	0	0	0	0	0	0	0	0	1	0	0	0
ENSMUSG00000086708	GM15577	1	0	0	0	0	0	0	0	0	0	1	0
ENSMUSG00000086714	0610009E02	1	0	0	0	0	0	2	5	1	2	1	1
ENSMUSG00000086717	GM15655	1	4	1	1	2	2	6	1	31	12	5	4
ENSMUSG00000086718	GM16069	0	0	0	0	0	0	1	2	1	1	0	2
ENSMUSG00000086721	GM13750	0	0	0	0	0	0	0	1	0	0	0	0
ENSMUSG00000086725	A630052C17	0	0	0	1	0	1	3	2	1	6	2	3
ENSMUSG00000086728	MAN2C1OS	2	3	2	1	17	1	4	3	5	4	4	6
ENSMUSG00000086732	GM6117	0	0	0	0	0	0	0	0	0	1	0	0
ENSMUSG00000086735	GM13977	0	0	1	0	0	0	2	2	0	2	1	0
ENSMUSG00000086740	GM17029	0	0	0	1	0	1	0	1	0	0	0	0
ENSMUSG00000086742	GM16201	0	0	0	1	0	0	0	0	1	0	0	0
ENSMUSG00000086743	HNRNPA1L2-	0	0	0	0	0	0	0	0	1	0	0	0
ENSMUSG00000086748	GM13261	2	4	1	3	5	7	22	28	32	12	20	26

ENSMUSG00000086753	GM15751	0	0	0	0	0	0	0	0	0	0	0	1
ENSMUSG00000086754	GM16098	0	0	0	0	1	0	0	0	0	0	0	0
ENSMUSG00000086773	GM16192	0	0	0	0	0	0	0	0	1	0	0	0
ENSMUSG00000086779	GM13562	0	0	0	0	0	0	0	0	1	1	0	0
ENSMUSG00000086782	E130102H24I	0	0	0	0	0	0	1	0	0	0	0	0
ENSMUSG00000086784	ISOC2A	0	1	3	4	1	1	9	14	4	10	6	3
ENSMUSG00000086785	GM6081	0	0	0	0	0	0	0	0	0	0	0	1
ENSMUSG00000086794	GM11642	0	0	0	0	0	0	0	1	0	0	0	0
ENSMUSG00000086796	4932702P03I	0	0	0	0	0	0	1	0	0	0	1	0
ENSMUSG00000086799	4930502E09I	0	0	0	0	0	0	0	0	0	1	0	0
ENSMUSG00000086805	4932443L11I	0	0	0	0	0	0	0	0	1	0	0	0
ENSMUSG00000086806	GM13054	0	0	0	0	0	0	0	1	0	0	0	0
ENSMUSG00000086816	GM16726	0	0	1	0	0	0	0	2	1	3	1	0
ENSMUSG00000086835	GM11775	0	0	0	0	0	0	0	0	0	1	0	0
ENSMUSG00000086836	GM13748	0	0	0	0	0	0	2	0	0	2	0	0
ENSMUSG00000086837	GM16618	0	0	0	0	0	0	0	0	1	0	0	1
ENSMUSG00000086839	GM11973	0	0	1	0	0	0	0	0	0	0	0	0
ENSMUSG00000086841	2410006H16I	16	27	21	19	25	16	102	103	99	91	82	94
ENSMUSG00000086846	GM13642	0	0	0	0	0	0	1	0	0	0	0	0
ENSMUSG00000086849	GM13840	0	1	0	0	0	0	0	1	0	0	0	0
ENSMUSG00000086851	A430108G06I	0	1	0	0	0	0	0	0	0	0	0	0
ENSMUSG00000086859	SNHG20	12	8	11	13	12	10	96	72	76	79	74	70
ENSMUSG00000086860	GM1720	0	0	0	0	0	1	0	0	1	0	0	1
ENSMUSG00000086862	GM13546	0	0	0	0	0	0	2	4	1	2	3	2
ENSMUSG00000086863	GM15543	1	0	0	1	0	0	0	0	1	1	0	0
ENSMUSG00000086873	GM15672	0	1	0	0	2	0	3	5	2	6	2	3
ENSMUSG00000086877	A230072C01I	0	0	0	1	1	0	3	3	0	3	4	3
ENSMUSG00000086881	GM13594	0	0	0	0	0	0	0	1	2	1	2	0
ENSMUSG00000086882	GM13352	0	0	0	0	0	0	0	0	0	1	0	0
ENSMUSG00000086884	GM16225	0	0	0	0	0	0	1	0	0	1	0	0
ENSMUSG00000086889	PHF2OS1	0	0	0	0	0	0	0	0	0	0	1	0
ENSMUSG00000086893	GM11365	1	0	0	0	0	0	0	0	1	0	0	0
ENSMUSG00000086894	GM15708	1	0	0	0	0	1	1	0	0	1	0	0
ENSMUSG00000086909	GM4926	0	0	0	0	0	0	0	0	1	0	0	0
ENSMUSG00000086914	GM16124	0	0	0	0	0	0	1	0	3	1	0	0
ENSMUSG00000086915	GM16365	1	0	0	0	0	0	1	0	0	0	0	0
ENSMUSG00000086916	GM15903	0	0	0	0	1	2	0	1	0	1	1	0
ENSMUSG00000086919	GM11497	0	0	0	0	0	1	0	0	0	0	0	0
ENSMUSG00000086920	GM12207	0	0	1	0	0	0	0	0	0	0	0	1
ENSMUSG00000086922	GM13835	0	0	0	0	0	0	2	1	2	0	2	2
ENSMUSG00000086925	GM6286	0	1	0	0	0	0	0	0	0	0	0	0
ENSMUSG00000086935	GM15956	0	0	0	0	1	0	0	2	1	1	0	0
ENSMUSG00000086937	GM15063	0	0	0	0	0	0	2	1	1	0	1	1
ENSMUSG00000086938	4930481A15I	0	1	1	1	0	1	7	4	4	9	2	6
ENSMUSG00000086942	GM15489	0	0	0	0	0	1	0	1	1	0	0	1
ENSMUSG00000086944	GM15859	0	2	0	1	0	0	1	1	1	0	1	1
ENSMUSG00000086946	GM15527	0	1	0	0	0	0	0	0	1	1	1	1
ENSMUSG00000086953	AKNAOS	0	0	0	0	0	0	0	0	0	2	0	0
ENSMUSG00000086962	GM12248	0	0	0	0	0	0	1	0	0	0	0	0
ENSMUSG00000086965	RTL10	0	0	0	0	0	1	0	0	1	0	1	0
ENSMUSG00000086968	4933431E20I	0	0	0	0	0	0	0	0	1	0	0	0
ENSMUSG00000086993	RSF1OS2	0	0	2	0	0	0	2	2	1	0	3	4
ENSMUSG00000086995	GM13544	1	1	0	2	1	2	0	1	0	1	0	0
ENSMUSG00000086997	GM12981	0	0	0	0	0	0	2	1	1	0	1	1
ENSMUSG00000087002	GM16277	0	0	0	1	0	0	0	0	0	0	0	0
ENSMUSG00000087014	GM16364	0	0	0	0	1	0	0	0	0	1	0	0
ENSMUSG00000087017	4930417H01I	0	0	0	0	0	0	1	1	0	1	2	0
ENSMUSG00000087020	GM16283	0	0	0	1	0	0	0	0	0	0	1	0

ENSMUSG00000087024	B230119M05	0	0	0	0	0	0	1	0	0	0	0	0
ENSMUSG00000087037	4930432L08R	0	0	0	0	0	0	0	0	0	0	0	1
ENSMUSG00000087040	GM14033	0	0	0	0	0	0	0	1	0	0	0	0
ENSMUSG00000087047	1700110K17R	0	0	0	0	0	0	0	0	0	1	0	0
ENSMUSG00000087050	DHRS13OS	1	9	5	7	3	2	14	19	12	16	13	9
ENSMUSG00000087055	GM11948	0	0	0	0	0	0	0	1	0	0	0	0
ENSMUSG00000087060	ELDR	2	3	3	5	4	3	15	17	12	12	13	4
ENSMUSG00000087066	GM15518	0	0	0	0	0	0	1	0	0	0	2	0
ENSMUSG00000087067	GM11532	0	0	2	0	0	1	2	1	1	3	3	3
ENSMUSG00000087088	GM16638	0	1	1	1	0	0	1	1	1	2	3	0
ENSMUSG00000087097	4930586N03I	0	0	0	0	0	0	1	1	0	0	0	0
ENSMUSG00000087107	AI662270	12	25	28	30	27	31	176	174	111	152	112	156
ENSMUSG00000087108	GM16093	0	0	0	0	0	0	0	0	0	0	1	0
ENSMUSG00000087119	ATG4A-PS	0	0	0	0	0	0	0	1	4	2	0	0
ENSMUSG00000087120	GM12279	0	0	1	0	0	0	0	0	0	1	0	0
ENSMUSG00000087122	4930403D09R	1	0	0	0	0	0	0	1	0	0	0	1
ENSMUSG00000087128	GM12655	0	0	0	1	0	0	2	0	0	5	0	0
ENSMUSG00000087129	GM16316	0	0	0	0	0	0	0	1	1	0	0	0
ENSMUSG00000087138	GM15545	0	0	1	0	0	1	1	3	2	4	1	2
ENSMUSG00000087141	PLCXD2	3	2	3	2	4	6	20	17	10	13	6	10
ENSMUSG00000087143	A830082K12R	3	4	1	0	1	2	7	12	5	11	14	8
ENSMUSG00000087153	GM6483	0	0	0	0	0	0	0	1	0	0	0	0
ENSMUSG00000087155	GM15008	0	0	0	0	0	0	0	0	0	0	1	0
ENSMUSG00000087165	2010001A14R	2	0	0	0	1	0	2	1	0	3	2	1
ENSMUSG00000087174	5530601H04R	1	3	1	3	5	2	26	13	13	28	14	23
ENSMUSG00000087175	GM15133	0	0	1	0	0	1	0	1	0	0	0	0
ENSMUSG00000087176	D230022J07R	0	0	0	0	0	0	1	0	0	1	0	1
ENSMUSG00000087177	E130307A14R	0	0	1	1	0	1	8	4	2	7	6	4
ENSMUSG00000087178	A230056P14R	0	0	0	0	0	0	0	1	0	3	1	1
ENSMUSG00000087190	D430001F17R	0	0	1	0	0	0	0	0	0	0	0	0
ENSMUSG00000087193	GM14820	1	0	0	0	0	0	0	0	0	0	0	0
ENSMUSG00000087213	2810408I11R	2	6	5	2	7	6	26	27	22	30	17	12
ENSMUSG00000087217	GM13209	0	1	0	0	0	0	0	0	0	0	0	0
ENSMUSG00000087221	BC037032	0	0	0	0	0	0	0	0	0	0	0	1
ENSMUSG00000087222	E030042O20R	0	0	0	0	0	0	0	0	0	0	0	1
ENSMUSG00000087231	E230016M11	1	3	0	2	0	0	3	0	3	4	2	4
ENSMUSG00000087233	GM43213	0	1	0	1	0	0	0	0	0	0	0	0
ENSMUSG00000087236	KIF28	0	0	0	0	0	0	0	0	0	2	0	0
ENSMUSG00000087249	GM16062	1	2	2	1	1	1	20	32	21	20	14	19
ENSMUSG00000087253	GM12043	0	0	0	0	0	0	0	0	1	0	0	1
ENSMUSG00000087260	LAMTOR5	4	9	6	2	4	6	56	58	36	44	36	44
ENSMUSG00000087266	GM15991	0	0	0	0	0	0	0	1	0	0	0	0
ENSMUSG00000087269	D330023K18R	0	1	0	1	0	1	1	1	1	3	3	1
ENSMUSG00000087286	GM9013	0	0	0	1	0	0	3	0	2	4	0	3
ENSMUSG00000087293	GM14341	0	0	0	0	0	0	0	0	1	0	0	0
ENSMUSG00000087294	GM13556	0	0	0	0	0	0	1	0	1	0	0	0
ENSMUSG00000087298	GM9392	0	0	0	0	0	0	1	0	0	0	0	0
ENSMUSG00000087299	GM12953	0	0	1	0	0	3	0	0	1	1	1	1
ENSMUSG00000087305	A430035B10R	0	2	0	0	1	0	1	4	0	0	0	0
ENSMUSG00000087307	GM12925	0	0	0	0	0	0	0	0	0	1	0	0
ENSMUSG00000087314	PRKAG2OS2	0	0	0	0	0	0	0	0	0	0	1	0
ENSMUSG00000087327	GM15884	0	0	0	0	1	0	1	0	2	0	2	0
ENSMUSG00000087336	GM15860	0	0	0	0	0	0	0	0	0	1	1	1
ENSMUSG00000087341	0610040F04R	0	0	0	0	0	0	0	0	0	0	1	0
ENSMUSG00000087347	1700047K16R	0	0	0	0	1	0	0	0	0	1	0	0
ENSMUSG00000087348	GM15582	1	0	0	0	0	0	0	0	0	0	0	0
ENSMUSG00000087352	GM12999	1	2	1	2	0	1	18	6	7	20	5	9
ENSMUSG00000087354	4930404I05R	1	6	2	9	5	7	23	34	28	46	25	43

ENSMUSG00000087365	C430049B03F	1	0	0	0	1	0	3	2	0	0	1	1
ENSMUSG00000087367	GM15491	0	0	0	1	1	1	0	1	2	3	3	2
ENSMUSG00000087368	BC065397	0	2	1	2	0	3	6	5	4	6	5	0
ENSMUSG00000087370	TMEM170B	3	4	3	5	1	1	12	10	12	20	7	15
ENSMUSG00000087371	GM15541	0	0	0	0	0	0	0	1	1	0	1	0
ENSMUSG00000087373	GM15892	4	2	2	1	3	1	4	10	6	6	6	16
ENSMUSG00000087377	AV099323	1	0	1	1	1	1	2	15	7	6	4	9
ENSMUSG00000087380	2210408F21F	1	0	0	0	0	0	0	0	0	0	0	0
ENSMUSG00000087385	FRG2F1	0	0	1	0	0	2	1	2	2	2	2	2
ENSMUSG00000087387	GM15420	1	0	0	0	0	0	0	0	0	0	0	0
ENSMUSG00000087389	GM15592	0	0	0	0	0	0	1	1	0	0	1	0
ENSMUSG00000087390	GM7598	0	0	0	0	0	0	1	1	0	0	1	2
ENSMUSG00000087393	1700023C21F	0	0	0	0	0	0	0	1	0	0	0	0
ENSMUSG00000087394	GM12945	0	0	0	0	0	0	0	0	1	0	0	0
ENSMUSG00000087396	4933407K13F	1	0	0	0	0	0	1	0	0	0	0	0
ENSMUSG00000087403	KANTR	2	8	3	5	1	7	14	10	7	13	5	4
ENSMUSG00000087408	CERS1	0	0	0	1	0	0	1	0	0	2	0	1
ENSMUSG00000087412	GM15501	103	116	133	170	154	166	393	440	355	451	328	361
ENSMUSG00000087424	5730405O15I	0	0	0	1	0	0	0	0	0	2	1	1
ENSMUSG00000087429	GM16235	0	0	0	1	0	0	0	0	2	0	0	0
ENSMUSG00000087430	GM13405	0	0	0	0	0	0	2	0	1	1	1	0
ENSMUSG00000087433	GM14167	0	0	0	0	0	0	1	1	0	0	1	1
ENSMUSG00000087440	4930577N17I	1	0	0	0	0	0	4	6	1	0	2	0
ENSMUSG00000087442	GM14453	1	2	2	2	1	4	19	10	3	7	14	14
ENSMUSG00000087444	GM5475	0	0	0	0	0	0	0	1	0	1	0	0
ENSMUSG00000087445	GM14286	1	0	0	0	0	0	1	0	1	2	1	1
ENSMUSG00000087449	GM12971	0	1	0	0	0	0	0	2	4	1	2	0
ENSMUSG00000087452	GM11998	0	1	1	1	1	0	14	20	6	9	12	8
ENSMUSG00000087454	GM13174	1	1	0	0	0	0	0	1	0	0	1	0
ENSMUSG00000087458	GM13999	0	1	0	0	0	0	0	0	0	0	0	0
ENSMUSG00000087475	4933406I18R	0	1	0	0	0	0	0	1	3	2	0	1
ENSMUSG00000087478	4930506C21F	1	0	0	0	0	0	1	2	3	2	0	2
ENSMUSG00000087484	2900089D17F	0	0	0	0	0	0	1	0	0	0	0	1
ENSMUSG00000087492	GM15700	0	0	0	0	1	0	0	0	0	0	0	0
ENSMUSG00000087497	2810001G20I	3	2	2	3	3	6	18	16	18	19	16	12
ENSMUSG00000087501	GM12116	0	0	0	0	0	0	0	0	0	1	0	0
ENSMUSG00000087502	GM16091	0	0	0	0	0	0	1	0	0	1	0	0
ENSMUSG00000087505	GM15241	0	0	0	0	0	0	0	1	0	0	0	0
ENSMUSG00000087519	AV039307	0	0	0	0	0	0	0	1	2	1	0	1
ENSMUSG00000087523	GM12319	0	0	0	0	0	0	0	2	0	0	0	1
ENSMUSG00000087528	9830144P21F	1	0	0	1	0	1	0	0	0	2	0	0
ENSMUSG00000087535	ZMIZ1OS1	0	0	0	0	0	0	0	1	0	0	0	0
ENSMUSG00000087543	GM16576	1	0	0	0	0	0	0	0	0	1	0	0
ENSMUSG00000087549	GM13427	0	0	0	1	0	0	2	1	0	0	1	1
ENSMUSG00000087552	GM15819	0	1	0	0	0	0	0	0	1	0	0	0
ENSMUSG00000087556	GM15764	1	0	0	0	0	0	3	0	0	1	0	0
ENSMUSG00000087568	MAATS1OS	0	0	0	1	0	0	2	2	0	2	1	2
ENSMUSG00000087574	C030037D09I	0	0	0	0	0	1	1	0	0	1	0	1
ENSMUSG00000087578	CCDC142OS	0	2	0	0	0	0	2	3	1	2	2	0
ENSMUSG00000087579	HECTD2OS	0	1	1	0	0	1	0	2	1	0	1	1
ENSMUSG00000087589	D430040D24I	0	0	0	0	0	0	1	0	1	0	0	1
ENSMUSG00000087590	EPB41L4AOS	11	15	11	18	24	11	87	81	67	83	79	67
ENSMUSG00000087593	GM16174	0	0	0	1	0	0	1	0	0	0	0	0
ENSMUSG00000087596	GM11715	1	3	2	1	1	3	12	10	7	17	8	28
ENSMUSG00000087610	GM16253	0	0	0	1	1	0	1	2	0	0	0	0
ENSMUSG00000087616	GM14257	0	0	0	0	0	0	0	0	0	0	1	0
ENSMUSG00000087623	GM12404	0	0	0	0	0	0	0	0	1	0	0	0
ENSMUSG00000087629	GM12246	0	0	0	0	0	0	0	0	0	1	1	0

ENSMUSG00000087633	GM14455	0	0	0	0	0	0	0	0	0	0	1	0
ENSMUSG00000087640	GM14798	0	0	0	0	0	0	1	0	0	1	0	0
ENSMUSG00000087641	GM12811	0	0	0	0	0	0	1	0	1	0	1	2
ENSMUSG00000087644	GM14703	0	0	1	0	0	0	0	1	0	0	0	0
ENSMUSG00000087651	1500009L16R	1	2	0	1	0	1	0	0	0	0	0	0
ENSMUSG00000087652	GM15918	0	0	0	0	0	0	0	0	0	0	0	1
ENSMUSG00000087654	GM12256	0	0	0	0	0	0	1	0	0	0	0	0
ENSMUSG00000087659	GM12606	0	0	0	0	0	0	1	0	0	0	0	0
ENSMUSG00000087660	B230398E01F	0	0	1	0	0	0	2	1	0	5	3	1
ENSMUSG00000087667	GM13381	0	0	0	0	1	2	0	0	0	1	0	0
ENSMUSG00000087670	9530036M11	2	3	4	1	3	2	9	9	11	12	4	4
ENSMUSG00000087679	TMEM250-PS	3	3	2	2	5	2	17	13	12	21	15	20
ENSMUSG00000087687	PET100	8	5	5	9	7	4	39	55	38	50	32	39
ENSMUSG00000087698	GM13031	0	0	0	0	0	0	0	0	0	1	0	0
ENSMUSG00000087700	GM15283	0	1	1	0	1	4	14	20	6	5	5	12
ENSMUSG00000087701	GM13493	0	0	0	0	0	0	0	0	1	1	2	1
ENSMUSG00000087731	GM24649	0	0	0	0	0	0	1	0	0	0	0	0
ENSMUSG00000087754	GM23470	0	0	0	0	0	0	0	0	0	0	0	1
ENSMUSG00000087800	GM25569	0	0	0	0	0	0	0	0	0	1	0	0
ENSMUSG00000087804	GM23434	0	0	0	0	0	0	0	0	0	0	1	0
ENSMUSG00000087819	GM25117	0	1	1	0	0	0	0	0	1	1	1	0
ENSMUSG00000087925	GM23232	0	0	0	0	0	0	0	0	0	1	1	0
ENSMUSG00000087938	GM26049	1	0	0	0	0	0	0	0	0	0	0	0
ENSMUSG00000088028	GM22141	0	0	0	0	0	0	0	0	0	0	0	1
ENSMUSG00000088066	GM23253	0	0	0	0	0	0	0	0	1	0	0	0
ENSMUSG00000088119	GM24060	0	0	0	0	0	0	0	0	0	0	0	1
ENSMUSG00000088145	GM24719	0	0	0	0	0	0	0	0	0	0	0	1
ENSMUSG00000088171	GM25220	1	0	0	2	1	1	4	1	0	4	0	2
ENSMUSG00000088177	GM23098	1	0	0	0	0	0	0	0	0	0	0	0
ENSMUSG00000088185	SCARNA2	0	0	0	0	0	0	0	0	1	0	1	2
ENSMUSG00000088200	GM23753	0	0	0	0	0	0	0	1	0	0	0	0
ENSMUSG00000088208	GM23751	0	0	0	2	0	0	1	1	0	1	0	2
ENSMUSG00000088244	GM25909	0	0	0	0	0	0	0	1	0	0	0	0
ENSMUSG00000088252	SNORD13	0	0	0	0	0	0	0	0	0	2	0	0
ENSMUSG00000088254	GM24289	0	0	0	0	0	0	0	0	0	0	1	1
ENSMUSG00000088308	GM24507	0	0	0	0	0	1	0	0	1	0	0	1
ENSMUSG00000088338	GM22849	0	0	0	0	0	0	0	0	0	1	0	0
ENSMUSG00000088348	GM23865	0	1	1	0	0	0	0	0	0	2	0	0
ENSMUSG00000088359	GM24009	0	1	2	0	0	0	2	0	4	1	2	0
ENSMUSG00000088420	GM22551	0	0	0	0	0	0	0	0	0	1	0	0
ENSMUSG00000088422	GM22553	0	0	0	0	0	0	0	0	1	0	0	0
ENSMUSG00000088464	MIR1970	0	0	0	0	0	0	0	0	0	1	0	0
ENSMUSG00000088478	MIR1946B	1	0	0	1	0	0	1	0	0	0	0	1
ENSMUSG00000088494	GM24399	1	0	0	0	0	0	0	0	0	0	0	0
ENSMUSG00000088514	GM24436	0	0	0	1	0	0	0	0	0	0	0	0
ENSMUSG00000088549	GM24959	0	0	0	1	0	0	5	1	3	6	3	0
ENSMUSG00000088555	GM22098	0	0	0	0	0	0	0	1	0	0	0	0
ENSMUSG00000088566	GM23783	0	0	0	0	0	0	1	0	0	0	0	0
ENSMUSG00000088591	GM23964	0	0	0	0	0	0	0	1	0	0	0	0
ENSMUSG00000088609	GM24187	0	0	0	0	0	0	0	0	0	0	0	1
ENSMUSG00000088625	GM23318	0	0	1	0	0	0	0	0	0	2	0	1
ENSMUSG00000088650	GM24854	1	0	0	2	0	0	4	0	0	1	0	2
ENSMUSG00000088651	GM24853	2	0	2	1	0	0	2	0	3	4	2	2
ENSMUSG00000088688	GM24000	1	0	0	0	0	0	1	0	0	0	0	0
ENSMUSG00000088689	SCARNA17	0	0	0	0	0	0	0	0	0	1	0	1
ENSMUSG00000088790	GM22541	0	0	0	0	0	0	1	0	0	0	0	0
ENSMUSG00000088793	GM22540	1	0	0	0	0	0	0	0	0	0	0	0
ENSMUSG00000088819	GM22388	0	0	1	0	0	0	0	0	0	0	0	0

ENSMUSG00000088822	GM25225	0	0	0	0	0	0	1	0	0	0	0	0
ENSMUSG00000088832	GM23543	0	0	0	0	0	0	0	0	0	0	1	0
ENSMUSG00000088835	GM23547	0	1	0	0	0	0	0	0	0	0	0	1
ENSMUSG00000088856	GM24727	0	0	0	0	0	0	0	0	1	0	0	0
ENSMUSG00000088958	SCARNA8	0	1	0	1	0	0	0	0	0	0	1	1
ENSMUSG00000088982	GM25600	0	0	0	1	0	0	0	0	0	1	0	0
ENSMUSG00000088990	GM22767	0	0	0	0	0	0	0	0	1	0	0	0
ENSMUSG00000089014	GM25520	0	1	0	0	0	0	0	1	0	0	0	0
ENSMUSG00000089029	GM25009	0	0	0	0	0	0	0	0	0	0	0	1
ENSMUSG00000089074	GM23817	0	0	0	0	0	0	1	0	0	0	1	0
ENSMUSG00000089080	GM24003	0	0	1	0	0	0	0	0	0	0	0	0
ENSMUSG00000089081	GM24002	0	0	0	0	0	0	0	0	0	1	0	0
ENSMUSG00000089103	GM23114	0	0	0	0	0	0	0	1	2	1	1	0
ENSMUSG00000089129	GM26407	0	0	1	0	1	0	0	0	0	0	0	0
ENSMUSG00000089155	GM23582	0	0	0	0	0	0	0	0	0	0	1	0
ENSMUSG00000089161	GM24130	0	0	0	1	1	0	0	0	0	2	0	0
ENSMUSG00000089177	GM22435	0	0	0	0	0	0	0	0	0	1	0	0
ENSMUSG00000089209	GM26215	0	0	0	0	0	0	0	1	0	0	0	0
ENSMUSG00000089214	MIR1931	1	0	0	0	0	0	4	0	2	2	3	0
ENSMUSG00000089235	GM23119	2	0	1	0	0	0	1	0	3	2	0	1
ENSMUSG00000089255	SNORA78	0	0	0	0	0	1	3	1	3	2	0	0
ENSMUSG00000089292	GM23209	0	0	0	0	1	0	1	1	0	0	0	0
ENSMUSG00000089308	GM24837	0	0	0	0	0	0	0	0	0	0	1	0
ENSMUSG00000089313	GM23182	1	0	0	0	0	0	0	0	0	0	0	0
ENSMUSG00000089350	GM25487	0	0	0	0	0	1	1	0	0	0	1	0
ENSMUSG00000089353	GM25488	0	0	0	0	0	0	0	0	0	1	0	0
ENSMUSG00000089417	GM22009	0	0	0	0	0	0	1	1	0	0	0	0
ENSMUSG00000089475	GM25363	0	0	0	0	0	0	0	0	0	0	1	2
ENSMUSG00000089493	GM26361	0	0	0	0	0	0	0	0	1	0	0	1
ENSMUSG00000089525	GM23833	0	1	0	0	0	1	0	0	0	0	0	0
ENSMUSG00000089536	SCARNA3A	0	0	0	0	0	0	0	0	0	1	0	0
ENSMUSG00000089586	GM25348	0	0	0	0	0	0	1	1	0	0	0	0
ENSMUSG00000089598	GM26158	0	0	0	0	0	0	0	0	0	0	1	0
ENSMUSG00000089636	1700058P15F	0	0	0	0	0	0	0	0	1	0	0	0
ENSMUSG00000089639	GM2862	0	0	0	0	0	0	1	0	0	0	0	0
ENSMUSG00000089640	GM6397	0	0	0	0	0	0	0	0	0	0	1	0
ENSMUSG00000089643	GM16301	0	0	0	0	0	0	0	0	0	1	0	0
ENSMUSG00000089646	GM15796	0	0	0	0	0	0	0	1	0	0	1	0
ENSMUSG00000089647	GM2245	5	3	4	6	4	2	10	12	8	17	13	16
ENSMUSG00000089657	GM16585	1	1	0	0	0	0	0	3	0	3	1	2
ENSMUSG00000089661	MIA	0	0	0	0	0	0	0	0	0	1	1	0
ENSMUSG00000089662	GM14057	0	0	0	2	0	0	0	0	3	0	2	1
ENSMUSG00000089663	GM15651	0	0	0	1	0	0	2	2	1	3	1	3
ENSMUSG00000089673	GM16546	0	0	1	0	0	0	0	0	0	0	0	0
ENSMUSG00000089682	BCL2L2	0	0	0	0	0	0	1	0	1	0	0	0
ENSMUSG00000089683	4930570N18I	0	0	0	0	0	0	0	0	1	0	0	0
ENSMUSG00000089686	MND1-PS	0	0	0	0	2	0	1	1	0	1	0	1
ENSMUSG00000089695	GM15946	0	0	0	0	0	0	0	1	0	1	0	0
ENSMUSG00000089697	GM15947	0	1	1	0	0	0	3	1	3	0	1	2
ENSMUSG00000089702	GM16568	0	0	0	0	0	0	1	2	2	2	0	1
ENSMUSG00000089706	B230216N24I	0	0	0	0	0	0	0	0	0	0	0	1
ENSMUSG00000089715	CBX6	46	44	44	52	55	42	144	142	86	167	127	142
ENSMUSG00000089716	GM6264	0	0	0	0	0	0	0	0	1	0	0	0
ENSMUSG00000089726	MIR17HG	3	5	3	3	6	5	23	29	24	33	27	38
ENSMUSG00000089729	GM16552	0	0	0	0	0	0	0	0	0	1	0	0
ENSMUSG00000089737	GM15688	0	0	0	0	0	0	1	0	0	0	0	0
ENSMUSG00000089745	1810008B01F	0	0	0	1	0	0	0	1	0	0	0	0
ENSMUSG00000089746	GM3513	0	0	0	0	0	0	0	0	0	0	0	1

ENSMUSG00000089762	IER5L	0	0	0	1	0	0	1	0	0	0	0	3
ENSMUSG00000089764	GM16580	2	1	1	2	0	1	7	8	2	6	1	7
ENSMUSG00000089766	GM16538	1	0	0	0	1	0	1	1	1	0	0	0
ENSMUSG00000089768	TMSB15B1	0	0	0	0	0	0	3	2	0	1	2	3
ENSMUSG00000089769	GM16574	0	0	0	0	1	0	1	0	0	1	0	0
ENSMUSG00000089774	SLC5A3	1	2	1	5	1	3	10	5	5	9	1	4
ENSMUSG00000089782	GM3531	0	0	0	0	0	0	0	2	0	0	0	1
ENSMUSG00000089785	SLC25A5-PS	0	0	0	0	0	0	0	1	1	1	0	0
ENSMUSG00000089792	GM16571	0	1	0	0	0	0	0	1	0	0	0	0
ENSMUSG00000089796	GM16575	0	0	0	0	0	0	0	0	0	1	0	0
ENSMUSG00000089798	1700028K03F	0	0	0	1	0	0	2	1	1	0	2	1
ENSMUSG00000089806	GM16570	0	0	0	0	0	1	0	0	0	0	0	0
ENSMUSG00000089810	GM16536	0	2	1	2	0	0	6	3	8	3	2	4
ENSMUSG00000089817	GM7162	0	0	0	1	0	1	0	0	2	0	0	0
ENSMUSG00000089824	RBM12	3	7	4	5	4	7	39	28	16	46	31	29
ENSMUSG00000089827	1700023H06F	0	1	0	0	0	0	1	0	0	0	0	0
ENSMUSG00000089829	GM16565	0	1	0	0	0	1	1	1	3	1	0	3
ENSMUSG00000089832	SHKBP1	3	3	2	4	4	4	6	15	10	10	6	7
ENSMUSG00000089838	GM2962	0	0	1	0	0	0	0	0	0	1	0	1
ENSMUSG00000089839	RPS19-PS11	0	0	3	0	1	1	0	0	1	3	1	2
ENSMUSG00000089847	TIMM10B	6	4	3	4	10	9	34	24	37	38	29	27
ENSMUSG00000089857	ZFP882	1	3	3	2	1	1	7	3	5	10	5	4
ENSMUSG00000089862	UMAD1	2	1	1	1	1	1	12	11	9	10	7	9
ENSMUSG00000089874	9230117E06F	0	0	0	0	0	0	0	0	2	0	0	0
ENSMUSG00000089875	ETOHD2	2	5	7	7	6	2	25	26	21	29	22	24
ENSMUSG00000089883	GM3355	0	0	0	0	0	0	3	1	1	0	0	2
ENSMUSG00000089889	0610040B10F	0	0	0	0	0	1	3	3	2	4	1	3
ENSMUSG00000089902	GM13625	27	43	33	55	36	54	145	156	145	205	120	151
ENSMUSG00000089905	GM16563	0	0	0	0	0	0	0	0	0	1	0	0
ENSMUSG00000089911	MFSD14A	8	13	11	9	9	14	85	68	43	78	54	47
ENSMUSG00000089917	UCKL1	2	2	2	2	3	2	13	12	12	21	9	7
ENSMUSG00000089940	GM4117	0	0	0	2	1	0	1	1	2	5	5	1
ENSMUSG00000089949	GM16137	0	0	0	0	0	1	0	0	0	0	0	0
ENSMUSG00000089965	GM16236	0	0	0	0	1	0	0	0	0	0	0	0
ENSMUSG00000089968	NCKAP5LOS	0	0	0	0	0	0	0	1	0	0	0	0
ENSMUSG00000089983	2010320O07I	0	0	0	1	0	0	0	0	0	0	0	0
ENSMUSG00000089984	FBXO24	0	0	0	0	0	0	0	0	0	1	0	0
ENSMUSG00000089988	GM16238	0	1	0	0	0	0	2	1	2	2	1	2
ENSMUSG00000089992	G6PD2	0	1	0	0	0	0	1	1	1	0	1	0
ENSMUSG00000089993	GM5822	0	0	0	0	0	0	0	0	0	0	0	1
ENSMUSG00000089994	GM16239	0	0	0	0	0	0	0	1	0	0	0	0
ENSMUSG00000089997	1810020O05I	0	0	1	0	0	0	0	0	0	0	0	0
ENSMUSG00000089998	PHTF1OS	1	3	3	4	3	4	19	8	7	14	11	16
ENSMUSG00000090000	IER3IP1	14	12	24	33	21	16	50	51	42	43	42	39
ENSMUSG00000090002	GM16006	0	0	0	0	0	0	0	0	1	0	0	0
ENSMUSG00000090005	GM16540	0	0	0	0	0	2	0	0	1	1	0	1
ENSMUSG00000090007	RPL30-PS2	0	0	0	0	0	0	0	2	0	0	0	0
ENSMUSG00000090015	GM15446	6	2	1	4	7	3	13	19	7	27	16	11
ENSMUSG00000090019	GIMAP1	14	6	12	11	14	5	74	71	52	67	48	56
ENSMUSG00000090023	GM16302	0	0	0	1	0	0	0	2	2	3	2	0
ENSMUSG00000090027	GM15740	0	0	0	0	0	0	1	1	0	1	0	0
ENSMUSG00000090030	A430072P03F	0	0	0	1	1	0	1	1	1	2	0	2
ENSMUSG00000090031	4732440D04F	0	0	0	0	0	0	1	2	0	1	4	1
ENSMUSG00000090035	GALNT4	14	14	13	16	10	18	71	72	51	84	57	57
ENSMUSG00000090054	GM15629	0	0	0	0	0	0	0	0	0	1	0	0
ENSMUSG00000090061	NWD2	0	0	0	0	0	3	0	0	0	0	0	0
ENSMUSG00000090063	DLX6OS1	0	0	0	0	0	0	1	1	0	2	0	0
ENSMUSG00000090066	1110002E22F	0	0	0	0	0	0	0	0	1	0	0	0

ENSMUSG00000090069	E430024P14F	0	0	0	0	0	0	0	0	0	2	1	0
ENSMUSG00000090070	GM16577	0	0	0	0	0	0	0	0	0	0	0	1
ENSMUSG00000090075	GM16251	1	0	0	0	0	0	0	0	1	0	0	0
ENSMUSG00000090083	RNF8	4	3	7	8	6	5	29	22	20	36	29	27
ENSMUSG00000090086	AI480526	1	1	4	5	3	2	4	8	6	11	6	10
ENSMUSG00000090098	ALMS1-PS2	0	0	0	0	0	0	1	2	2	2	1	0
ENSMUSG00000090100	TTBK2	3	3	0	4	1	0	7	5	10	9	4	6
ENSMUSG00000090101	SNHG9	7	8	10	9	16	11	28	35	28	37	31	36
ENSMUSG00000090107	GM11492	0	0	0	1	0	0	0	0	0	0	0	0
ENSMUSG00000090110	CMC4	2	3	2	0	3	5	19	23	19	21	13	16
ENSMUSG00000090112	SHPRH	6	12	6	6	9	5	39	50	23	49	31	30
ENSMUSG00000090114	GM3076	0	0	0	0	0	0	1	1	0	1	0	0
ENSMUSG00000090115	USP49	2	3	2	5	2	1	13	9	6	17	7	14
ENSMUSG00000090117	GM16541	1	0	0	0	0	0	1	1	0	1	0	0
ENSMUSG00000090118	GM16163	0	2	0	0	0	0	1	1	4	2	0	0
ENSMUSG00000090119	GM8185	1	1	0	0	0	0	0	0	0	0	0	0
ENSMUSG00000090125	POU3F1	0	0	0	0	0	0	0	0	1	0	0	0
ENSMUSG00000090126	4930519F09F	0	0	0	0	1	0	0	2	0	0	1	0
ENSMUSG00000090136	GM10177	8	12	6	7	8	6	29	26	17	40	25	22
ENSMUSG00000090137	UBA52	0	3	0	2	0	0	9	6	4	3	8	6
ENSMUSG00000090141	GM614	0	0	0	0	0	0	0	1	0	0	1	0
ENSMUSG00000090142	GM15795	0	0	0	0	0	0	0	0	0	1	0	0
ENSMUSG00000090150	ACAD11	2	1	0	3	0	2	7	10	6	11	10	9
ENSMUSG00000090157	GM16534	0	0	0	0	0	0	0	1	0	2	0	0
ENSMUSG00000090164	BC035044	0	0	0	0	0	0	0	0	1	0	0	0
ENSMUSG00000090188	GM7418	0	1	1	0	0	0	3	4	0	0	2	1
ENSMUSG00000090191	9230105E05F	0	0	0	0	0	0	0	0	1	0	0	0
ENSMUSG00000090202	4930503B20F	0	0	0	0	0	0	0	2	1	1	1	0
ENSMUSG00000090206	TEPP	0	1	0	0	3	1	3	5	3	5	5	1
ENSMUSG00000090207	4930524O07I	0	0	0	0	0	0	3	1	0	2	1	0
ENSMUSG00000090210	ITGA10	0	2	0	0	2	0	3	2	0	6	2	6
ENSMUSG00000090213	TMEM189	7	11	9	13	12	14	47	38	36	36	27	37
ENSMUSG00000090214	GM15657	0	0	0	0	0	0	0	1	0	3	0	1
ENSMUSG00000090215	TRIM34B	0	0	0	0	0	0	0	1	0	1	1	0
ENSMUSG00000090216	GM16549	0	1	0	0	0	0	0	1	0	0	0	0
ENSMUSG00000090220	PITPNM2OS1	0	0	0	1	0	0	0	0	0	0	1	0
ENSMUSG00000090223	PCP4	0	0	2	1	0	0	2	5	0	2	2	1
ENSMUSG00000090230	GM16315	1	3	3	3	1	4	12	10	13	14	13	11
ENSMUSG00000090244	GM16572	0	0	1	0	0	0	0	0	0	0	0	0
ENSMUSG00000090246	GM17017	0	0	0	0	1	0	0	1	0	1	0	2
ENSMUSG00000090248	GM14027	0	0	0	0	0	0	2	2	0	0	1	1
ENSMUSG00000090256	ALMS1-PS1	0	0	0	0	0	1	0	0	0	1	0	0
ENSMUSG00000090258	CHURC1	1	1	2	2	2	0	19	8	2	10	13	12
ENSMUSG00000090266	METTL23	22	17	34	18	20	17	106	125	121	100	92	125
ENSMUSG00000090272	MNDAL	13	19	13	8	20	10	106	111	63	103	82	87
ENSMUSG00000090273	PRR22	2	2	0	2	0	3	3	3	3	0	2	2
ENSMUSG00000090290	TARBP1	3	3	5	7	4	3	14	23	15	15	20	9
ENSMUSG00000090305	GM5459	0	0	0	0	1	0	1	0	0	0	0	1
ENSMUSG00000090319	GM4462	0	0	0	0	0	0	0	0	0	1	0	0
ENSMUSG00000090323	GM5263	0	0	0	0	0	0	0	0	1	0	0	0
ENSMUSG00000090327	GM17111	0	0	0	0	0	0	0	1	0	0	0	1
ENSMUSG00000090330	9130221H12F	3	2	0	3	2	1	18	23	11	9	12	11
ENSMUSG00000090334	GM17149	0	0	0	0	0	1	0	0	0	0	0	0
ENSMUSG00000090336	4932443I19R	0	0	0	0	1	1	1	1	3	3	1	0
ENSMUSG00000090357	GM17077	0	0	0	0	1	0	0	0	0	0	0	0
ENSMUSG00000090387	GM17056	1	1	2	2	0	0	1	1	3	2	3	3
ENSMUSG00000090389	CDV3-PS	0	1	0	0	0	0	0	1	1	1	0	0
ENSMUSG00000090394	4930523C07F	18	9	26	34	21	22	103	102	75	105	90	79

ENSMUSG00000090397	GM17096	0	0	0	0	0	0	0	0	1	0	1	0
ENSMUSG00000090406	GM17058	0	0	1	0	0	0	0	1	0	2	0	1
ENSMUSG00000090441	GM17651	0	0	0	0	0	0	0	1	0	0	0	0
ENSMUSG00000090458	GM17122	0	0	1	1	1	0	6	1	0	1	1	1
ENSMUSG00000090467	GM4613	0	0	2	0	0	0	2	2	0	3	0	1
ENSMUSG00000090474	GM9372	0	0	1	0	0	0	0	0	0	0	0	0
ENSMUSG00000090489	GM17415	0	2	0	0	0	0	0	0	0	0	0	0
ENSMUSG00000090516	RPS11-PS1	1	2	0	0	1	1	1	2	1	4	2	2
ENSMUSG00000090523	GYPC	3	6	1	8	7	4	21	34	23	31	25	23
ENSMUSG00000090527	GM5538	0	0	0	0	0	0	0	0	0	0	0	1
ENSMUSG00000090544	GM6576	0	0	1	1	0	0	0	0	1	0	1	1
ENSMUSG00000090553	SNRPE	20	21	29	37	37	25	179	197	160	163	190	152
ENSMUSG00000090559	GM17137	0	0	0	0	0	0	0	0	0	0	0	1
ENSMUSG00000090564	A430057M04	0	0	1	1	0	0	5	3	1	0	0	1
ENSMUSG00000090592	GM17571	0	0	0	0	0	0	0	1	0	1	0	2
ENSMUSG00000090602	GM5611	0	0	0	0	0	0	1	0	0	1	0	0
ENSMUSG00000090608	GM17200	0	1	1	1	0	2	4	4	3	2	3	2
ENSMUSG00000090610	GM3571	0	1	0	0	0	0	1	0	0	0	1	0
ENSMUSG00000090624	GM17060	0	0	0	0	0	0	1	0	0	1	0	0
ENSMUSG00000090625	GM20721	35	31	32	59	42	34	227	234	146	225	202	169
ENSMUSG00000090626	TEX9	8	13	20	19	25	17	76	73	62	105	62	68
ENSMUSG00000090634	GM8126	0	0	0	0	0	0	0	0	0	0	1	0
ENSMUSG00000090637	GM6189	0	0	0	1	0	0	1	0	0	0	0	0
ENSMUSG00000090641	ZFP712	1	0	1	1	0	1	0	2	2	2	3	2
ENSMUSG00000090665	GAD1-PS	0	0	0	0	0	0	0	0	1	0	0	0
ENSMUSG00000090671	GM5067	0	0	1	0	0	0	0	0	0	0	0	0
ENSMUSG00000090674	GM17082	0	0	1	0	0	1	0	0	0	0	0	0
ENSMUSG00000090693	TRIM43A	0	0	1	0	0	0	1	0	0	0	0	0
ENSMUSG00000090698	APOLD1	3	4	3	2	5	3	13	10	6	8	7	8
ENSMUSG00000090708	GM17196	0	0	0	0	0	0	0	0	0	0	0	1
ENSMUSG00000090733	RPS27	248	279	260	349	348	341	1196	1116	904	1094	828	1017
ENSMUSG00000090758	GM17178	2	2	0	1	0	0	0	0	3	3	1	6
ENSMUSG00000090761	GM17201	0	0	0	0	0	0	1	2	0	0	2	0
ENSMUSG00000090774	VMN2R74	0	0	0	0	0	0	0	0	0	1	0	0
ENSMUSG00000090799	KLHL33	0	0	0	0	0	0	1	0	0	0	0	0
ENSMUSG00000090805	CAAA011657	0	0	0	0	0	0	0	1	0	0	0	0
ENSMUSG00000090812	SAMD15	0	0	0	0	0	0	2	1	0	0	0	0
ENSMUSG00000090840	1700092M07	0	0	0	0	0	0	2	1	3	1	0	0
ENSMUSG00000090841	MYL6	8	10	8	4	2	10	52	55	52	59	45	49
ENSMUSG00000090843	HEATR4	1	0	0	0	0	0	0	0	0	0	0	0
ENSMUSG00000090853	GM17364	0	0	0	0	0	0	1	0	0	0	0	0
ENSMUSG00000090860	GM17134	1	6	3	6	1	2	13	21	13	12	12	8
ENSMUSG00000090861	ARF4OS	0	0	0	0	0	0	0	1	0	0	0	0
ENSMUSG00000090862	RPS13	111	132	139	176	157	136	634	699	604	715	545	730
ENSMUSG00000090873	GM17112	0	0	0	0	0	1	0	0	0	0	0	0
ENSMUSG00000090891	D6ERTD527E	0	1	1	0	0	1	0	3	1	2	0	2
ENSMUSG00000090935	SYNJ2BP	11	19	14	17	15	25	98	87	52	81	53	60
ENSMUSG00000090936	GM17705	0	0	0	0	0	0	0	0	0	1	0	0
ENSMUSG00000090941	GM17212	0	0	0	0	0	1	0	0	1	0	0	0
ENSMUSG00000090946	CCDC71L	4	13	6	4	8	5	49	38	17	47	47	33
ENSMUSG00000090952	GM17251	2	4	3	3	5	3	13	14	9	7	6	13
ENSMUSG00000090963	GM17655	0	1	0	2	1	0	2	4	1	4	2	4
ENSMUSG00000091019	GM7502	0	0	0	0	0	0	0	1	0	1	0	0
ENSMUSG00000091021	GM17300	7	26	6	11	11	16	88	60	60	115	60	70
ENSMUSG00000091028	GM10722	0	1	0	0	0	1	8	2	2	7	0	1
ENSMUSG00000091050	9330020H09F	0	0	0	0	1	0	2	4	2	4	4	4
ENSMUSG00000091060	7420461P10F	0	0	0	0	1	0	0	0	0	0	0	0
ENSMUSG00000091071	1700030C10F	0	0	0	1	0	0	0	0	0	0	0	1

ENSMUSG00000091086	RPL6L	1	1	0	1	0	0	1	1	2	0	0	1
ENSMUSG00000091091	KCNMB3	0	0	0	0	0	0	0	0	0	2	0	0
ENSMUSG00000091102	5830462I19R	1	0	0	1	0	0	0	1	0	1	0	0
ENSMUSG00000091119	CCDC152	3	5	4	8	3	8	30	9	14	28	11	9
ENSMUSG00000091133	GM17108	0	0	0	0	0	0	0	0	0	0	0	2
ENSMUSG00000091144	PHF11C	0	1	1	5	3	1	6	7	7	5	2	3
ENSMUSG00000091149	GM17020	0	0	0	0	0	0	0	0	0	1	0	0
ENSMUSG00000091154	PROSCOS	0	0	0	0	0	0	0	0	0	1	0	1
ENSMUSG00000091155	SERPINE3	0	0	0	0	0	1	0	0	1	1	0	0
ENSMUSG00000091165	GM17036	0	0	2	1	1	1	0	1	0	3	4	2
ENSMUSG00000091183	GM5141	1	1	1	0	1	0	5	6	8	6	1	6
ENSMUSG00000091191	GM17334	1	0	2	0	1	2	0	3	2	1	6	5
ENSMUSG00000091192	SARDHOS	0	0	0	0	0	0	0	0	0	0	1	0
ENSMUSG00000091198	GM5218	0	0	0	0	0	1	0	0	0	0	0	0
ENSMUSG00000091239	VMN2R76	0	0	0	0	0	0	0	0	1	0	0	0
ENSMUSG00000091259	VMN2R110	0	0	0	0	0	0	3	0	0	0	0	1
ENSMUSG00000091264	SMIM13	9	12	13	19	7	14	32	41	17	36	20	23
ENSMUSG00000091272	GM17641	0	0	0	0	0	0	1	0	0	0	0	0
ENSMUSG00000091285	GM17430	1	0	0	0	0	0	0	0	0	0	0	0
ENSMUSG00000091306	GM17606	2	4	7	10	6	7	23	19	18	27	22	14
ENSMUSG00000091313	FTH-PS2	0	0	0	0	0	0	0	1	1	0	0	0
ENSMUSG00000091315	GM17214	0	0	0	0	0	0	0	0	0	1	0	0
ENSMUSG00000091329	1700112D23I	0	0	1	0	0	0	0	1	0	0	0	0
ENSMUSG00000091337	EID1	9	7	10	8	9	18	51	36	24	46	34	34
ENSMUSG00000091361	GM5277	0	0	0	0	0	0	0	0	1	0	0	0
ENSMUSG00000091363	GM7701	0	0	1	0	0	0	1	0	0	1	0	0
ENSMUSG00000091367	GM17711	4	0	0	1	4	0	20	17	16	13	17	9
ENSMUSG00000091370	5730435O14I	0	0	0	0	0	0	0	1	0	0	0	0
ENSMUSG00000091378	GM4219	0	0	0	0	0	0	0	0	1	0	0	0
ENSMUSG00000091383	HIST1H2AL	0	0	0	0	0	1	0	1	0	0	0	0
ENSMUSG00000091390	GM17168	0	0	0	0	0	0	2	2	0	0	0	0
ENSMUSG00000091405	HIST2H4	1	0	0	0	0	1	0	4	0	1	1	1
ENSMUSG00000091419	GM17450	0	0	0	0	0	0	0	1	0	0	0	0
ENSMUSG00000091421	GM4202	0	0	0	0	0	0	1	0	0	0	1	0
ENSMUSG00000091423	GM17509	1	1	0	2	0	2	0	0	2	1	1	0
ENSMUSG00000091449	GM10269	1	5	4	5	1	2	19	8	14	16	9	13
ENSMUSG00000091460	GM7808	0	1	0	0	0	0	2	0	0	3	1	1
ENSMUSG00000091474	2610021A01I	0	1	2	4	2	0	15	14	12	8	9	8
ENSMUSG00000091478	GM10039	0	0	0	0	0	0	2	0	0	0	1	0
ENSMUSG00000091491	VMN2R97	0	0	0	0	0	1	3	1	0	0	1	0
ENSMUSG00000091498	MPC1-PS	0	0	0	0	0	0	0	0	0	0	1	1
ENSMUSG00000091509	GM17066	27	36	37	34	42	40	156	170	104	169	106	91
ENSMUSG00000091511	VMN2R87	0	0	0	0	0	0	1	0	0	0	0	0
ENSMUSG00000091512	LAMTOR3	1	4	8	11	8	5	53	64	37	57	56	32
ENSMUSG00000091514	GM17484	1	1	0	1	0	0	5	3	4	1	3	2
ENSMUSG00000091528	GM9268	0	0	0	0	0	0	1	0	0	1	0	0
ENSMUSG00000091531	OLFR102	0	0	0	0	0	0	0	0	0	0	0	1
ENSMUSG00000091534	GM17195	0	0	0	0	0	0	0	0	0	1	0	0
ENSMUSG00000091537	TMA7	10	13	12	21	14	17	101	89	66	105	89	76
ENSMUSG00000091549	GM6548	0	0	0	3	0	2	2	2	0	0	1	0
ENSMUSG00000091550	GM6811	0	0	0	0	0	0	0	1	0	0	0	0
ENSMUSG00000091572	VMN2R3	0	0	0	0	0	0	0	0	0	1	0	0
ENSMUSG00000091575	2010016I18R	13	20	20	33	9	16	62	62	52	50	50	59
ENSMUSG00000091577	GM6211	0	0	0	0	0	0	0	0	0	1	0	0
ENSMUSG00000091586	CYP4F17	0	1	4	1	3	2	5	3	1	8	6	6
ENSMUSG00000091601	OLFR93	0	0	0	0	0	0	0	0	0	1	0	0
ENSMUSG00000091604	GM17349	0	1	0	0	0	1	0	0	0	0	0	0
ENSMUSG00000091614	GM17657	0	0	0	0	0	0	0	1	0	0	0	0

ENSMUSG00000091623	GM17092	3	2	3	2	2	4	7	13	1	12	10	5
ENSMUSG00000091625	LSM5	0	6	4	8	9	8	61	56	52	57	40	30
ENSMUSG00000091631	VMN2R95	0	0	0	0	0	0	0	0	0	0	0	1
ENSMUSG00000091639	GM3756	0	0	0	0	0	0	0	1	0	0	0	1
ENSMUSG00000091649	PHF11B	0	0	0	0	0	0	0	1	0	0	1	1
ENSMUSG00000091655	GM8741	0	0	0	0	1	0	0	1	0	0	0	0
ENSMUSG00000091679	VMN2R96	7	5	9	5	4	5	23	37	12	42	8	22
ENSMUSG00000091695	GM17669	0	0	1	1	0	0	0	0	0	1	1	0
ENSMUSG00000091697	EIF3S6-PS2	0	0	1	0	0	0	2	1	1	3	0	2
ENSMUSG00000091698	GM6526	0	0	0	0	0	0	0	0	0	0	1	0
ENSMUSG00000091722	SIAH3	0	1	2	0	1	0	4	2	0	2	0	0
ENSMUSG00000091732	GM17541	1	0	0	0	0	0	2	2	1	1	1	0
ENSMUSG00000091735	GPR62	0	0	1	1	0	0	0	0	0	0	0	0
ENSMUSG00000091742	GM5093	0	0	0	0	0	0	0	1	0	1	0	1
ENSMUSG00000091764	ZFP964	0	0	0	0	1	0	0	0	0	0	0	0
ENSMUSG00000091780	SCO2	2	0	2	1	1	2	10	8	1	15	8	6
ENSMUSG00000091785	RPL29-PS5	0	0	0	0	0	0	2	4	2	3	0	1
ENSMUSG00000091786	GM8425	0	0	0	0	0	0	0	1	0	0	0	0
ENSMUSG00000091794	VMN2R18	0	0	0	0	0	0	1	1	0	2	0	1
ENSMUSG00000091803	COX16	0	0	1	1	4	2	2	7	2	5	7	2
ENSMUSG00000091806	GM5258	0	0	2	0	0	0	0	0	1	0	0	0
ENSMUSG00000091811	INAFM1	0	0	0	2	0	0	9	3	4	3	5	4
ENSMUSG00000091818	RPL19-PS4	0	0	0	0	0	0	0	0	0	1	0	0
ENSMUSG00000091831	GM4707	1	0	0	0	1	2	3	2	0	1	2	4
ENSMUSG00000091842	GM16490	3	7	4	6	7	6	19	17	14	18	14	20
ENSMUSG00000091844	GM8251	16	45	30	42	30	37	95	101	84	122	83	76
ENSMUSG00000091845	RPL36-PS12	3	2	5	1	4	0	10	9	7	12	4	8
ENSMUSG00000091859	VMN2R100	0	0	0	1	1	1	0	0	0	1	0	0
ENSMUSG00000091864	GM17102	0	0	0	0	0	0	0	0	0	1	0	0
ENSMUSG00000091876	GM3851	0	0	0	0	0	0	0	0	0	0	0	1
ENSMUSG00000091881	GM17146	0	0	1	0	0	0	0	0	0	0	0	0
ENSMUSG00000091896	UBE2D2A	8	4	3	10	9	12	56	59	38	64	58	49
ENSMUSG00000091897	GM17019	0	0	0	0	0	0	1	0	0	0	0	0
ENSMUSG00000091898	TNNC1	0	1	0	0	0	0	0	0	0	0	0	0
ENSMUSG00000091930	VMN2R52	0	0	0	0	0	0	0	0	0	1	0	0
ENSMUSG00000091931	GON7	2	1	3	6	1	5	22	20	22	33	21	18
ENSMUSG00000091947	6530401F13R	0	0	0	0	0	0	0	0	0	0	0	1
ENSMUSG00000091955	GM9844	0	1	1	0	1	0	1	2	5	2	2	2
ENSMUSG00000091957	RPS2-PS10	47	55	59	79	50	56	129	160	107	122	102	120
ENSMUSG00000091968	GM17115	0	1	0	0	0	0	0	0	1	0	0	1
ENSMUSG00000091970	GM4868	0	0	0	0	0	0	0	0	1	0	0	0
ENSMUSG00000091971	HSPA1A	0	0	0	0	0	0	2	0	0	0	0	0
ENSMUSG00000091985	GM17354	1	1	4	1	1	0	7	3	3	5	4	1
ENSMUSG00000091993	B930036N10	1	1	2	3	0	2	9	7	6	13	9	6
ENSMUSG00000091994	E130317F20R	1	0	0	0	0	1	3	1	1	3	2	3
ENSMUSG00000091996	BC049352	0	0	0	0	0	0	0	1	0	0	0	0
ENSMUSG00000091997	GM6611	0	0	0	0	1	0	0	0	0	1	0	1
ENSMUSG00000092006	GM17139	0	0	0	5	0	0	4	2	6	5	2	4
ENSMUSG00000092009	MYH15	0	0	0	1	0	0	1	0	0	0	0	1
ENSMUSG00000092014	GM4468	0	0	1	0	0	0	1	0	1	4	0	0
ENSMUSG00000092029	GM9027	0	0	0	0	0	1	0	2	0	0	1	0
ENSMUSG00000092072	GM4540	9	7	12	12	8	13	28	46	30	41	28	39
ENSMUSG00000092074	DYNLT1A	9	10	11	13	12	18	32	41	33	25	26	22
ENSMUSG00000092083	KCNB2	0	0	0	0	0	0	0	0	0	0	1	0
ENSMUSG00000092085	GM17224	0	1	0	0	0	0	0	1	3	0	1	1
ENSMUSG00000092103	GM17039	0	0	0	0	0	0	0	0	0	0	0	1
ENSMUSG00000092116	GM10320	0	0	0	0	0	0	2	4	3	1	5	2
ENSMUSG00000092118	FANCF	1	4	3	1	2	5	23	19	9	20	11	20

ENSMUSG00000092124	B930094E09F	0	0	0	0	0	0	0	0	0	1	0	0
ENSMUSG00000092182	GM20407	0	0	0	1	0	0	0	0	0	0	0	0
ENSMUSG00000092183	4930515G01I	0	0	0	0	0	0	0	1	0	0	0	0
ENSMUSG00000092187	GM20457	0	0	0	0	0	0	0	0	1	0	0	1
ENSMUSG00000092190	GM20470	0	0	0	0	0	0	1	0	0	0	0	1
ENSMUSG00000092192	DYX1C1	0	1	1	0	0	0	6	2	1	3	4	6
ENSMUSG00000092203	1110038B12F	43	53	77	48	49	49	305	331	254	344	241	260
ENSMUSG00000092212	SLC22A13B-P	1	0	0	0	0	0	0	1	1	1	2	0
ENSMUSG00000092216	GM19345	0	0	0	0	0	0	0	0	0	0	0	1
ENSMUSG00000092224	GM8815	0	1	0	0	0	0	0	1	0	0	0	0
ENSMUSG00000092230	GM18889	0	0	0	0	0	0	0	1	0	0	0	0
ENSMUSG00000092241	GM20522	0	0	0	1	0	0	0	0	0	2	0	2
ENSMUSG00000092260	ZFP963	1	0	0	0	2	2	1	2	1	1	1	4
ENSMUSG00000092261	GM20519	0	0	0	0	0	0	0	0	0	0	0	1
ENSMUSG00000092274	NEAT1	41	31	27	26	33	34	109	99	71	131	72	116
ENSMUSG00000092281	HMGB1-PS7	0	1	0	0	0	0	1	0	1	1	0	0
ENSMUSG00000092284	GM8801	0	0	0	0	0	0	0	0	0	1	0	0
ENSMUSG00000092286	DTNBOS	0	0	0	0	0	0	0	0	0	1	0	0
ENSMUSG00000092295	GM20543	0	0	0	0	0	0	0	0	0	1	0	0
ENSMUSG00000092300	CDK3-PS	0	1	0	0	0	2	1	1	1	2	0	2
ENSMUSG00000092341	MALAT1	139	249	218	461	289	253	1380	1271	998	1464	965	1216
ENSMUSG00000092344	HSPE1-PS3	0	0	0	0	0	0	0	0	0	1	1	0
ENSMUSG00000092349	PLATR17	0	0	0	0	0	0	0	0	0	0	1	0
ENSMUSG00000092358	GM20430	0	0	0	0	0	0	2	1	2	0	1	0
ENSMUSG00000092365	BC023719	0	0	0	0	0	0	1	0	1	1	0	2
ENSMUSG00000092368	A930015D03I	2	0	1	0	1	0	4	3	3	4	2	2
ENSMUSG00000092371	GM20511	0	0	0	0	0	0	0	0	1	0	0	0
ENSMUSG00000092373	GM20535	0	0	0	0	0	0	0	1	0	0	0	0
ENSMUSG00000092374	GM6623	0	0	0	0	0	0	0	0	0	0	1	0
ENSMUSG00000092381	GM20512	0	0	0	0	0	0	0	0	0	0	1	0
ENSMUSG00000092384	GM4189	0	0	0	0	1	0	0	0	1	4	0	1
ENSMUSG00000092386	GM20536	0	0	0	5	0	2	1	1	0	3	0	0
ENSMUSG00000092390	GM20541	0	2	4	0	2	4	7	12	3	11	11	7
ENSMUSG00000092394	GM20445	0	0	1	1	0	0	1	0	0	0	0	0
ENSMUSG00000092395	GM20463	2	2	2	4	2	2	18	12	11	18	7	12
ENSMUSG00000092416	ZFP141	2	3	2	1	1	5	13	9	11	18	6	4
ENSMUSG00000092417	GPANK1	5	5	1	7	3	4	29	22	17	18	12	13
ENSMUSG00000092418	GM20406	1	0	0	0	0	0	1	1	2	0	0	0
ENSMUSG00000092428	GM20545	0	0	0	0	0	0	1	0	0	0	0	0
ENSMUSG00000092457	GM8835	0	0	0	0	0	0	0	0	0	0	1	0
ENSMUSG00000092461	GM18833	0	0	0	0	0	0	0	0	1	0	0	0
ENSMUSG00000092495	GM20405	0	0	0	0	0	0	0	0	0	0	1	1
ENSMUSG00000092505	GM19246	0	0	0	0	0	0	0	0	0	0	0	1
ENSMUSG00000092536	GM20501	0	0	0	0	0	0	0	0	0	1	0	0
ENSMUSG00000092539	GM20468	0	0	0	0	0	0	0	0	1	0	0	1
ENSMUSG00000092540	GM8738	0	0	0	0	0	0	0	0	1	4	0	0
ENSMUSG00000092550	GM20496	0	0	0	0	0	0	0	0	0	2	0	0
ENSMUSG00000092556	OLFR755-PS1	0	0	0	0	0	0	0	1	0	1	0	0
ENSMUSG00000092557	GM8696	0	0	0	0	0	0	0	0	0	1	0	0
ENSMUSG00000092558	MED20	1	3	3	7	5	4	21	24	15	19	20	16
ENSMUSG00000092563	GM3617	0	0	0	0	0	0	0	1	0	0	0	0
ENSMUSG00000092564	BC051226	0	2	0	0	3	0	4	6	4	3	4	1
ENSMUSG00000092586	LY6G6C	0	0	0	0	0	0	0	0	1	0	0	0
ENSMUSG00000092591	GM20429	0	0	0	0	0	1	0	0	0	0	0	0
ENSMUSG00000092593	GM20492	0	1	0	0	0	0	0	0	0	0	0	0
ENSMUSG00000092607	SCNM1	4	1	2	2	6	7	12	17	10	28	20	21
ENSMUSG00000092622	KHDC3	0	0	0	0	0	1	2	1	3	1	0	0
ENSMUSG00000092625	GM20404	0	0	1	1	0	0	0	1	2	2	0	1

ENSMUSG00000092626	9130230N09I	1	0	1	0	2	4	6	4	8	4	8	2
ENSMUSG00000092674	GM24105	0	0	0	0	0	0	1	0	0	0	0	0
ENSMUSG00000092713	GM22858	0	0	0	0	0	0	1	0	0	1	0	0
ENSMUSG00000092796	GM22692	0	0	0	0	0	1	0	0	0	0	0	0
ENSMUSG00000092805	GM26461	0	0	1	1	1	0	1	0	0	0	0	1
ENSMUSG00000092814	GM23638	0	0	0	0	0	0	0	0	0	1	0	0
ENSMUSG00000092819	GM23639	0	0	0	0	1	0	1	1	2	1	2	3
ENSMUSG00000092827	MIR3091	0	0	0	0	0	0	2	0	0	1	0	0
ENSMUSG00000092837	RPPH1	0	0	0	0	0	0	0	0	0	0	1	0
ENSMUSG00000092851	GM25960	0	0	0	0	0	0	0	0	1	0	0	0
ENSMUSG00000092853	GM25959	0	0	0	0	1	1	0	2	2	1	0	1
ENSMUSG00000092958	MIR5128	0	2	1	1	0	2	0	2	0	1	1	3
ENSMUSG00000092981	MIR5125	0	0	0	0	0	0	1	1	1	1	0	1
ENSMUSG00000093006	GM24157	1	0	0	0	0	0	1	0	0	0	0	0
ENSMUSG00000093040	GM26141	0	0	0	0	0	0	0	1	1	0	0	0
ENSMUSG00000093044	SNORD8	0	0	0	0	1	0	0	1	1	1	0	0
ENSMUSG00000093105	GM25535	0	0	0	0	0	0	1	0	0	0	0	0
ENSMUSG00000093176	MIR5119	0	0	0	0	0	0	0	0	1	0	0	0
ENSMUSG00000093178	SNORD87	0	0	0	0	0	0	1	0	0	3	1	2
ENSMUSG00000093232	GM24111	0	0	0	0	0	0	0	0	0	0	0	1
ENSMUSG00000093239	GM24106	0	0	0	0	0	0	0	0	1	0	0	0
ENSMUSG00000093262	GM23413	0	1	0	0	0	0	3	0	0	2	0	0
ENSMUSG00000093289	SNORD92	0	0	0	0	0	0	1	0	0	0	1	2
ENSMUSG00000093314	MIR5136	5	7	9	5	6	3	24	16	17	21	16	16
ENSMUSG00000093322	GM24971	0	0	0	0	0	0	1	0	0	0	0	0
ENSMUSG00000093345	GM27956	0	0	0	0	0	0	0	0	0	1	0	0
ENSMUSG00000093352	GM23291	0	1	2	0	0	2	0	1	1	2	2	0
ENSMUSG00000093387	GM20732	0	0	0	0	0	0	1	0	0	0	0	0
ENSMUSG00000093392	GM6061	0	0	0	0	0	0	0	0	0	0	1	0
ENSMUSG00000093404	GM25190	1	0	0	0	0	0	0	1	0	0	0	0
ENSMUSG00000093408	MIR5617	0	0	0	0	0	0	0	0	0	0	1	0
ENSMUSG00000093424	6330562C20F	0	0	1	0	0	1	1	2	0	0	0	0
ENSMUSG00000093447	GM5139	0	0	0	0	0	0	1	1	1	0	0	0
ENSMUSG00000093465	GM20682	0	0	0	0	0	2	0	0	0	1	0	0
ENSMUSG00000093473	GM20620	1	0	0	0	0	0	0	0	0	0	0	0
ENSMUSG00000093483	AA465934	40	48	50	60	59	50	238	255	200	234	172	236
ENSMUSG00000093495	GM23368	1	0	0	0	0	0	2	1	2	0	0	1
ENSMUSG00000093507	GM20627	1	0	0	1	0	0	0	0	0	1	1	0
ENSMUSG00000093520	VMN1R-PS9	0	0	0	0	0	0	0	0	0	1	0	0
ENSMUSG00000093531	VMN2R-PS11	0	0	0	1	0	0	1	0	1	0	0	0
ENSMUSG00000093535	4930533I22R	0	0	0	0	0	0	0	0	1	0	0	0
ENSMUSG00000093559	GM20705	0	0	0	0	0	0	2	0	0	0	0	1
ENSMUSG00000093565	RAB26OS	3	3	9	11	10	9	38	30	28	46	33	27
ENSMUSG00000093577	GM20632	0	0	0	1	1	0	0	0	0	0	0	1
ENSMUSG00000093594	GM20707	0	0	1	1	0	0	2	1	2	1	0	0
ENSMUSG00000093598	A730085K08F	0	0	0	0	0	0	1	1	3	0	2	1
ENSMUSG00000093606	B130034C11F	0	0	1	0	0	0	0	3	2	5	0	1
ENSMUSG00000093627	VMN2R-PS12	0	0	0	0	0	0	0	1	0	0	0	0
ENSMUSG00000093629	PROX2OS	1	0	0	0	0	1	2	2	3	4	4	1
ENSMUSG00000093649	GM20614	0	0	0	0	0	0	0	0	1	0	0	0
ENSMUSG00000093651	GM5873	0	0	0	0	0	0	1	0	0	1	0	0
ENSMUSG00000093656	GM20628	0	0	1	0	0	0	0	0	0	0	0	1
ENSMUSG00000093661	EIF4E3	6	6	3	4	3	3	25	22	18	32	22	22
ENSMUSG00000093666	VMN1R-PS15	0	0	1	0	0	0	0	1	0	0	0	0
ENSMUSG00000093668	POU5F2	2	0	0	1	0	3	0	4	2	0	0	4
ENSMUSG00000093672	GM20655	0	0	0	0	0	0	0	1	0	0	0	0
ENSMUSG00000093673	GM20644	0	0	0	0	0	1	2	0	1	0	2	1
ENSMUSG00000093674	RPL41	116	159	120	189	156	167	905	950	671	813	708	763

ENSMUSG00000093677	GM20712	1	1	1	0	0	1	3	7	3	10	7	5
ENSMUSG00000093686	GM4705	2	2	4	3	5	0	10	6	3	6	5	9
ENSMUSG00000093695	GM20717	0	0	0	0	0	0	1	0	0	0	0	0
ENSMUSG00000093720	GM20635	0	0	0	0	0	0	0	0	1	0	0	0
ENSMUSG00000093726	GM20667	0	0	1	0	0	0	1	0	0	1	0	0
ENSMUSG00000093739	GM20692	0	0	0	0	0	0	0	0	0	1	0	0
ENSMUSG00000093751	GM3307	0	0	0	0	0	0	0	0	0	0	0	1
ENSMUSG00000093765	GM20658	0	0	0	1	0	0	0	0	0	0	0	0
ENSMUSG00000093768	GM20650	0	0	1	0	0	0	0	0	1	1	0	0
ENSMUSG00000093772	4931403E22F	0	0	0	1	1	0	1	0	0	0	2	0
ENSMUSG00000093798	GM8355	0	0	0	0	0	0	2	0	0	1	1	1
ENSMUSG00000093803	PPP2R3D	4	5	5	9	3	7	21	18	12	19	4	25
ENSMUSG00000093843	GM25939	0	1	0	0	0	0	0	0	0	0	0	1
ENSMUSG00000093858	GM19967	0	0	0	1	0	1	1	1	0	0	0	0
ENSMUSG00000093861	IGKV1-110	0	0	0	0	0	0	0	0	0	0	1	0
ENSMUSG00000093862	GM5117	0	0	0	0	0	0	1	0	0	0	0	0
ENSMUSG00000093874	GM24539	3	0	1	3	1	2	3	1	1	2	3	0
ENSMUSG00000093879	GM25457	0	0	0	0	0	0	0	0	0	1	0	0
ENSMUSG00000093885	RPL35A-PS5	3	0	0	0	2	1	1	2	0	3	0	2
ENSMUSG00000093896	IGHV1-76	0	0	0	1	0	0	4	2	0	1	0	0
ENSMUSG00000093904	TOMM20	3	6	2	12	8	5	27	47	18	47	29	22
ENSMUSG00000093930	HMGCS1	25	22	21	29	29	14	218	221	149	215	138	129
ENSMUSG00000094018	S100A2	0	0	0	0	0	0	0	0	0	0	1	0
ENSMUSG00000094024	ESP18	0	0	0	0	0	0	0	0	0	0	1	0
ENSMUSG00000094026	GM23002	0	0	0	0	0	0	0	0	0	1	0	0
ENSMUSG00000094075	IGHV1-80	0	0	0	0	0	0	0	0	1	0	0	0
ENSMUSG00000094092	GM15921	0	0	0	0	0	0	1	0	0	0	0	0
ENSMUSG00000094127	G530012D18	1	0	1	0	1	3	11	8	7	5	10	12
ENSMUSG00000094153	GM8807	0	0	0	0	0	0	0	1	1	0	0	0
ENSMUSG00000094185	GM5445	2	5	0	6	4	9	11	10	6	12	7	11
ENSMUSG00000094189	GM6134	1	0	0	0	0	0	0	1	0	0	1	0
ENSMUSG00000094191	RPL31-PS14	0	0	0	0	0	0	3	0	1	1	0	0
ENSMUSG00000094220	TRAV6-3	0	0	0	0	0	0	0	1	0	0	0	0
ENSMUSG00000094245	GM23966	0	0	0	0	0	0	2	2	0	0	1	0
ENSMUSG00000094248	HIST1H2AO	1	0	0	0	0	0	0	0	0	1	0	0
ENSMUSG00000094256	RPL21-PS7	0	0	0	0	0	0	0	0	1	0	1	0
ENSMUSG00000094281	GM8034	0	0	0	0	0	0	0	2	0	0	0	0
ENSMUSG00000094306	GM24924	0	1	0	0	1	1	0	0	1	1	1	2
ENSMUSG00000094318	GM13144	0	0	0	1	0	0	0	0	0	0	0	0
ENSMUSG00000094338	HIST1H2BL	0	0	0	0	0	0	0	0	0	0	1	1
ENSMUSG00000094344	GM11942	15	12	15	21	21	18	69	92	56	70	68	72
ENSMUSG00000094366	GM23730	0	0	0	0	0	0	0	0	0	1	0	0
ENSMUSG00000094372	GM21839	1	0	0	0	0	1	1	1	1	1	1	0
ENSMUSG00000094392	GM3788	1	0	0	0	1	0	0	3	2	5	2	3
ENSMUSG00000094400	GM23140	0	0	0	0	0	0	1	0	0	1	0	0
ENSMUSG00000094410	GM38394	0	0	0	0	0	0	0	1	0	0	0	0
ENSMUSG00000094437	GM9830	0	0	0	0	0	0	0	0	1	0	0	0
ENSMUSG00000094441	ZFP955A	6	4	5	2	6	1	25	28	14	32	16	19
ENSMUSG00000094446	GM6344	1	0	0	0	0	0	0	0	0	0	0	0
ENSMUSG00000094463	GM8546	0	0	0	0	0	0	0	0	0	0	0	1
ENSMUSG00000094483	PURB	33	49	29	47	58	39	177	172	128	190	134	191
ENSMUSG00000094498	GM10582	0	0	0	0	0	0	0	0	1	0	0	0
ENSMUSG00000094502	IGHV1-69	0	0	0	0	0	0	0	0	0	0	0	1
ENSMUSG00000094525	TRBV12-2	1	1	1	2	1	1	7	4	1	8	2	5
ENSMUSG00000094530	GM21399	0	0	0	0	0	0	0	0	0	0	1	0
ENSMUSG00000094557	RPL35A-PS7	0	0	0	0	0	0	0	1	0	1	0	1
ENSMUSG00000094594	TPD52-PS	0	0	0	0	0	0	0	0	0	0	1	0
ENSMUSG00000094595	FSBP	1	0	0	0	1	2	4	3	0	4	2	6

ENSMUSG00000094605	GM25873	0	0	0	0	0	0	0	0	0	0	0	1
ENSMUSG00000094622	GM3055	0	0	0	0	0	0	1	0	0	1	1	1
ENSMUSG00000094655	GM25360	0	1	0	0	1	0	0	0	0	0	0	1
ENSMUSG00000094664	RPL35A-PS6	0	0	0	0	0	0	0	1	1	1	1	0
ENSMUSG00000094689	IGHV1-81	0	0	0	0	0	0	0	0	0	0	0	1
ENSMUSG00000094696	GM6632	0	0	0	0	0	0	0	0	1	0	0	0
ENSMUSG00000094724	RNASET2B	0	0	0	0	0	0	0	2	0	1	2	1
ENSMUSG00000094772	RPL29-PS2	0	0	0	0	0	0	1	0	0	0	0	0
ENSMUSG00000094786	GM14403	0	0	0	0	0	0	4	7	2	3	4	4
ENSMUSG00000094787	IGHV1-54	0	0	0	1	0	0	0	0	1	0	1	0
ENSMUSG00000094796	BC147527	0	1	2	0	0	0	1	0	1	2	1	1
ENSMUSG00000094806	CYP2D10	0	0	0	0	0	0	1	0	0	0	0	0
ENSMUSG00000094815	GM22615	0	0	0	0	0	0	1	1	1	2	0	0
ENSMUSG00000094820	GM23805	0	0	0	0	0	0	1	0	0	1	0	0
ENSMUSG00000094832	GM21846	0	0	0	1	1	0	0	0	0	2	0	0
ENSMUSG00000094842	GM22639	0	0	0	0	0	0	0	1	0	0	0	0
ENSMUSG00000094843	PPP1R2-PS1	0	0	0	0	0	0	0	0	0	1	0	0
ENSMUSG00000094845	TMEM95	0	0	0	0	0	0	0	1	0	0	1	0
ENSMUSG00000094868	DLX6OS2	0	0	0	0	0	0	2	0	0	1	0	0
ENSMUSG00000094870	ZFP131	28	29	24	36	41	24	121	161	114	200	116	151
ENSMUSG00000094936	RBM4	0	0	2	1	0	2	5	5	3	10	6	5
ENSMUSG00000094940	IGHV1-84	0	0	0	0	0	0	0	0	0	0	0	1
ENSMUSG00000094942	GM3604	1	1	1	0	2	2	7	5	6	12	3	2
ENSMUSG00000094955	GM3699	0	0	4	1	0	0	1	2	2	3	1	4
ENSMUSG00000094974	RPS19-PS2	0	0	0	0	0	0	0	0	1	0	0	0
ENSMUSG00000094989	RPL9-PS4	0	1	0	3	0	0	1	1	0	0	0	1
ENSMUSG00000095026	GM3336	0	0	0	1	1	0	0	1	0	1	0	0
ENSMUSG00000095040	1700001J03R	0	0	1	0	0	0	2	2	3	2	1	1
ENSMUSG00000095041	AC149090.1	0	2	1	1	1	1	3	7	0	3	3	8
ENSMUSG00000095042	GM12537	0	0	0	0	0	1	0	0	0	0	0	0
ENSMUSG00000095068	GM26189	0	0	0	0	0	0	0	0	0	2	0	1
ENSMUSG00000095098	CCDC85B	5	3	8	9	5	10	25	30	23	32	20	24
ENSMUSG00000095105	EDARADD	2	2	3	5	4	3	3	5	4	6	4	3
ENSMUSG00000095111	GM23713	1	1	1	0	0	0	1	0	0	2	0	0
ENSMUSG00000095123	GM21781	2	0	0	1	2	3	5	2	0	2	3	7
ENSMUSG00000095127	IGHV1-82	2	0	0	1	1	0	4	2	2	6	4	5
ENSMUSG00000095150	RPS19-PS13	0	0	0	0	0	0	1	0	0	0	0	0
ENSMUSG00000095159	TUBB4B-PS1	0	0	0	1	0	0	0	1	0	0	1	1
ENSMUSG00000095168	GM4795	0	0	0	0	0	0	0	1	0	0	0	0
ENSMUSG00000095174	GM20069	0	0	0	0	1	0	0	1	0	0	0	0
ENSMUSG00000095178	GM22716	0	1	1	0	1	0	0	2	0	0	1	1
ENSMUSG00000095180	RHOX5	0	0	1	0	1	0	1	1	1	0	1	2
ENSMUSG00000095186	GM10718	0	0	0	0	0	1	7	0	0	5	0	0
ENSMUSG00000095193	GM20939	1	0	0	0	1	2	4	3	4	4	3	1
ENSMUSG00000095205	SNORD93	1	0	0	0	0	0	0	0	0	0	0	0
ENSMUSG00000095217	HIST1H2BN	0	0	0	0	0	0	1	0	0	0	0	0
ENSMUSG00000095253	ZFP799	0	1	0	3	2	1	5	8	5	8	6	10
ENSMUSG00000095280	GM21738	1	1	1	0	0	3	58	11	0	31	3	1
ENSMUSG00000095288	GM7618	0	0	0	1	0	0	0	0	0	3	0	2
ENSMUSG00000095315	GM10130	5	2	8	4	3	2	24	20	16	18	20	16
ENSMUSG00000095325	ZFP870	0	0	0	1	3	0	5	1	2	9	1	3
ENSMUSG00000095332	GM9821	0	0	0	0	0	0	1	0	0	0	0	0
ENSMUSG00000095334	GM21984	0	0	0	0	0	0	0	0	0	0	0	1
ENSMUSG00000095362	GM14325	0	0	0	0	0	0	0	2	0	2	0	1
ENSMUSG00000095427	RPS2-PS6	0	0	1	0	0	0	0	1	1	0	1	1
ENSMUSG00000095432	ZFP748	1	0	1	2	0	2	3	3	7	9	3	7
ENSMUSG00000095457	GM8989	0	0	0	0	0	0	1	1	0	2	0	0
ENSMUSG00000095465	GM6214	0	0	0	0	0	0	0	0	0	2	0	0

ENSMUSG00000095526	GM10243	4	7	14	8	14	8	30	28	30	19	17	20
ENSMUSG00000095530	SNORA19	0	0	0	0	0	0	0	0	0	0	0	1
ENSMUSG00000095531	GM25284	0	0	0	1	0	0	0	0	0	2	0	0
ENSMUSG00000095541	GM24617	0	0	0	0	0	0	0	0	0	0	1	0
ENSMUSG00000095547	GM10719	0	0	0	0	0	3	7	4	0	5	1	0
ENSMUSG00000095555	GM28010	0	1	0	0	1	1	1	0	1	0	0	1
ENSMUSG00000095562	GM21887	11	17	31	18	25	12	73	81	67	58	66	85
ENSMUSG00000095567	NOC2L	23	25	26	43	28	33	180	201	151	212	158	179
ENSMUSG00000095577	GM6619	0	1	0	0	0	0	0	0	0	0	0	0
ENSMUSG00000095588	GM12350	0	1	0	0	0	0	1	1	0	0	1	0
ENSMUSG00000095589	IGHV1-55	0	0	0	0	0	0	0	1	0	0	0	0
ENSMUSG00000095597	GM6472	2	9	7	6	9	4	18	24	17	22	11	16
ENSMUSG00000095600	GM9457	0	0	0	1	0	0	2	0	0	0	0	0
ENSMUSG00000095616	GM26244	0	0	0	0	0	0	0	2	0	1	1	1
ENSMUSG00000095620	2010005H15F	1	0	0	1	0	2	1	0	0	0	2	1
ENSMUSG00000095649	GM8979	0	0	0	0	0	0	1	0	0	1	0	0
ENSMUSG00000095677	DYNLT1F	1	2	1	1	2	0	8	4	2	5	4	1
ENSMUSG00000095687	RNASET2A	0	0	0	0	0	0	0	2	0	0	1	0
ENSMUSG00000095762	GM16378	0	0	0	0	0	0	0	1	0	0	0	0
ENSMUSG00000095771	IGKV14-111	0	0	0	0	0	0	0	1	0	1	0	1
ENSMUSG00000095780	GM24674	0	0	0	0	0	1	0	0	0	0	0	0
ENSMUSG00000095789	NUPR1L	0	0	0	0	0	0	0	0	2	0	1	0
ENSMUSG00000095836	GM21960	0	0	0	0	0	0	0	0	0	1	0	0
ENSMUSG00000095847	GM5451	0	1	0	0	0	0	0	0	0	1	0	1
ENSMUSG00000095858	OLFR104-PS	0	1	0	0	0	0	0	0	0	0	0	0
ENSMUSG00000095891	GM10717	0	0	0	0	0	5	43	9	0	43	6	1
ENSMUSG00000095930	NIM1K	0	0	0	0	1	0	0	2	3	1	1	2
ENSMUSG00000095947	GM25195	0	0	0	0	0	0	1	0	0	0	0	0
ENSMUSG00000095955	GM22360	0	0	0	0	0	0	0	0	0	1	0	0
ENSMUSG00000095959	GM10845	0	1	0	0	0	0	0	0	0	0	0	0
ENSMUSG00000095990	ZFP97	1	0	0	0	0	1	2	3	0	6	1	1
ENSMUSG00000096006	GM21596	0	5	7	1	4	2	24	29	12	15	4	11
ENSMUSG00000096010	HIST4H4	0	0	1	2	1	1	4	2	2	1	3	0
ENSMUSG00000096020	IGHV1-75	0	0	0	0	0	0	0	0	0	0	0	1
ENSMUSG00000096022	GM23421	0	0	0	0	0	0	0	0	0	0	0	1
ENSMUSG00000096030	GM24424	0	0	0	0	0	0	1	1	0	0	2	1
ENSMUSG00000096052	9930004E17F	0	0	0	0	0	0	1	0	1	0	0	0
ENSMUSG00000096054	SYNE1	1	8	4	5	9	1	19	21	22	18	17	19
ENSMUSG00000096056	GM21986	0	0	0	0	0	0	0	0	0	3	0	0
ENSMUSG00000096111	GM21917	0	0	0	0	0	0	1	0	0	0	0	0
ENSMUSG00000096112	GM26269	0	0	0	0	0	0	0	0	0	1	0	0
ENSMUSG00000096124	GM22308	0	0	0	0	0	0	1	0	0	0	0	0
ENSMUSG00000096141	DNAH7A	0	0	0	0	0	0	1	1	0	0	0	0
ENSMUSG00000096145	VKORC1	0	0	1	0	0	0	0	1	0	1	1	0
ENSMUSG00000096157	GM5472	0	0	0	0	0	0	0	0	0	1	0	1
ENSMUSG00000096171	GM8055	0	0	0	0	0	0	0	0	0	1	0	0
ENSMUSG00000096175	GM21761	0	0	0	1	0	0	2	0	0	0	0	0
ENSMUSG00000096177	GM5070	0	0	0	0	0	0	0	1	0	0	0	1
ENSMUSG00000096188	CMTM4	2	2	2	1	4	0	4	5	2	4	1	2
ENSMUSG00000096199	PTRHD1	5	2	5	3	1	2	16	17	12	13	6	7
ENSMUSG00000096201	GM10715	1	0	0	0	0	3	12	2	0	2	0	0
ENSMUSG00000096210	H1F0	5	6	5	7	13	11	44	52	38	52	44	39
ENSMUSG00000096220	OLFR775	0	0	0	0	0	0	1	0	0	0	0	0
ENSMUSG00000096221	1500002C15F	2	3	1	2	2	2	11	13	15	16	6	11
ENSMUSG00000096249	GM24263	0	0	0	0	0	0	0	1	0	0	0	0
ENSMUSG00000096255	DYNLT1	0	0	0	0	0	0	0	0	1	1	0	0
ENSMUSG00000096257	CCER2	0	0	0	0	0	0	0	0	0	1	0	0
ENSMUSG00000096280	GM23287	0	0	0	0	0	0	0	0	0	1	0	0

ENSMUSG00000096299	GM21814	0	4	3	1	1	0	6	0	6	6	3	6
ENSMUSG00000096310	GM23523	0	0	0	1	0	0	0	0	0	0	0	0
ENSMUSG00000096326	IGHV1-78	0	0	0	0	0	0	0	0	0	0	0	1
ENSMUSG00000096349	GM22513	0	0	0	0	0	0	0	0	0	2	1	0
ENSMUSG00000096351	SAMD11	4	3	3	2	4	8	28	27	22	21	21	26
ENSMUSG00000096354	GM8178	0	0	0	0	0	0	1	0	0	0	0	0
ENSMUSG00000096370	GM21992	0	0	0	0	1	0	0	0	3	1	4	1
ENSMUSG00000096385	GM11168	0	0	0	0	0	0	9	0	0	4	0	0
ENSMUSG00000096401	GM21811	0	0	1	0	0	0	0	0	0	1	0	0
ENSMUSG00000096433	ZFP994	5	3	3	5	3	4	26	15	13	15	18	12
ENSMUSG00000096434	GM22129	0	0	0	0	0	0	1	0	0	0	0	0
ENSMUSG00000096448	GM5808	0	0	0	0	0	0	0	0	0	0	0	1
ENSMUSG00000096449	GM4076	0	0	0	0	0	0	0	0	0	1	0	0
ENSMUSG00000096452	IGHV1-77	0	0	0	0	0	1	0	1	2	0	0	0
ENSMUSG00000096472	CDKN2D	6	2	10	6	8	8	40	43	29	47	33	38
ENSMUSG00000096486	GM5426	0	0	0	0	0	0	0	0	1	0	0	0
ENSMUSG00000096517	GM6919	0	0	0	0	0	0	0	1	1	0	0	0
ENSMUSG00000096519	GM10721	0	0	0	0	0	0	0	1	0	1	0	0
ENSMUSG00000096520	GM3376	0	1	0	0	0	0	0	0	0	0	0	0
ENSMUSG00000096544	GM4617	0	0	0	0	0	0	1	2	0	4	0	2
ENSMUSG00000096547	GM25235	0	1	0	0	0	0	0	0	0	0	0	0
ENSMUSG00000096551	TRAV10	0	0	0	0	0	0	2	0	0	0	0	0
ENSMUSG00000096563	GM24095	0	2	4	1	2	3	4	1	3	2	5	2
ENSMUSG00000096576	OOG1	0	0	0	0	0	0	0	1	0	0	0	0
ENSMUSG00000096600	TRAV6D-3	0	0	0	0	0	0	0	1	0	0	0	0
ENSMUSG00000096655	1700065D16R	0	1	0	0	0	0	1	1	0	0	0	0
ENSMUSG00000096681	RPS19-PS1	0	2	0	1	3	0	1	2	3	3	3	4
ENSMUSG00000096687	MFSD4B4	1	1	1	1	1	1	3	2	2	4	3	2
ENSMUSG00000096699	RPS19-PS4	1	0	0	2	0	1	0	5	2	0	2	2
ENSMUSG00000096722	HMGB1-PS9	0	0	0	0	0	0	0	0	0	1	0	0
ENSMUSG00000096727	PSMB9	3	4	2	4	5	7	13	21	19	21	14	19
ENSMUSG00000096736	GM17535	0	0	0	0	0	1	4	0	0	0	0	0
ENSMUSG00000096740	LBHD1	2	2	1	3	0	1	3	3	6	8	3	3
ENSMUSG00000096746	TRAV7D-3	0	0	0	0	0	0	1	0	0	0	0	0
ENSMUSG00000096768	GM47283	66	76	87	89	95	70	322	384	241	329	272	351
ENSMUSG00000096780	TMEM181B-P	39	44	38	56	48	41	172	186	162	182	135	195
ENSMUSG00000096794	OLFR905	0	0	0	1	0	0	0	0	0	0	0	0
ENSMUSG00000096801	GM23412	0	0	0	0	0	0	0	0	0	0	1	0
ENSMUSG00000096841	GM25596	0	1	1	0	0	0	1	0	1	0	1	0
ENSMUSG00000096842	GM10736	0	0	0	0	1	0	0	0	0	0	0	0
ENSMUSG00000096856	GM10778	0	0	0	0	0	0	0	1	0	1	0	0
ENSMUSG00000096883	SHISA8	0	0	0	0	0	0	1	0	0	0	0	0
ENSMUSG00000096910	ZFP955B	12	15	15	14	11	12	67	65	45	81	47	57
ENSMUSG00000096916	ZFP850	2	1	2	2	3	5	18	11	14	19	7	16
ENSMUSG00000096917	2500002B13R	2	0	0	0	1	0	4	2	2	2	1	1
ENSMUSG00000096920	RPS19-PS8	0	0	0	0	0	0	0	1	0	0	0	0
ENSMUSG00000096923	A730071L15R	0	0	0	0	1	1	1	1	4	2	1	2
ENSMUSG00000096926	GM26823	0	0	0	0	0	0	0	0	1	1	0	0
ENSMUSG00000096929	A330023F24R	0	0	0	0	0	0	0	0	1	0	2	3
ENSMUSG00000096931	GM26656	0	0	0	0	1	0	1	1	1	0	0	0
ENSMUSG00000096932	5033403F01R	0	0	0	1	1	1	1	1	0	2	0	1
ENSMUSG00000096935	1700113A16R	0	1	1	3	1	1	5	7	9	10	8	8
ENSMUSG00000096941	GM26720	0	0	0	0	0	0	0	0	0	0	0	1
ENSMUSG00000096942	RPS19-PS6	19	27	19	38	30	24	71	56	52	66	64	62
ENSMUSG00000096943	GM26721	0	0	0	0	0	0	0	0	0	2	0	0
ENSMUSG00000096944	GM26722	23	63	28	63	29	40	286	211	181	367	189	251
ENSMUSG00000096945	GM26546	0	0	0	0	0	0	0	0	0	0	0	1
ENSMUSG00000096946	FMR1OS	0	0	0	1	0	0	1	1	0	0	0	0

ENSMUSG00000096947	GM26723	0	0	1	0	0	0	2	2	1	1	0	0
ENSMUSG00000096950	GM9530	0	0	0	0	0	0	0	0	0	0	0	2
ENSMUSG00000096953	GM26571	0	0	0	0	0	1	3	1	1	0	1	2
ENSMUSG00000096954	GDAP10	1	1	1	1	0	1	2	2	2	6	0	0
ENSMUSG00000096959	4930509G22I	0	0	0	0	0	0	0	1	0	0	0	0
ENSMUSG00000096965	3300005D01F	0	0	0	0	0	0	0	0	0	0	1	0
ENSMUSG00000096967	GM26621	0	1	0	0	0	0	2	4	3	4	1	1
ENSMUSG00000096968	GM26620	0	0	2	0	0	0	1	0	0	0	0	2
ENSMUSG00000096975	GM16386	1	0	0	0	0	0	1	0	0	0	0	0
ENSMUSG00000096976	GM26882	0	0	0	2	0	2	1	4	1	2	2	2
ENSMUSG00000096977	GM10827	0	0	0	0	2	0	1	0	1	0	0	1
ENSMUSG00000096981	GM16845	0	0	0	0	0	0	1	0	0	0	0	0
ENSMUSG00000096983	2010015M23	1	1	1	2	0	3	3	12	12	9	5	7
ENSMUSG00000096986	4930509E16F	0	0	0	0	0	2	0	2	3	0	1	2
ENSMUSG00000096988	A930029G22I	0	1	0	2	0	0	1	1	0	0	0	2
ENSMUSG00000096990	GM26790	0	0	0	4	2	2	1	4	4	3	0	3
ENSMUSG00000096991	GM26789	0	0	0	0	0	0	0	0	0	0	1	0
ENSMUSG00000096992	GM26788	0	0	0	0	0	1	0	0	0	0	0	0
ENSMUSG00000096993	GM26787	0	0	0	0	0	0	0	1	0	0	1	1
ENSMUSG00000096995	2810029C07F	0	0	0	0	1	0	0	0	0	1	1	3
ENSMUSG00000097000	GM17435	0	1	0	4	1	1	2	5	4	7	3	4
ENSMUSG00000097006	9530082P21F	0	2	0	1	0	0	4	3	2	1	3	4
ENSMUSG00000097007	4930432B10F	0	0	0	0	0	0	0	1	0	0	0	0
ENSMUSG00000097008	GM26860	0	0	0	2	0	0	2	1	0	0	0	1
ENSMUSG00000097013	GM26710	0	2	1	1	0	0	3	5	1	4	0	1
ENSMUSG00000097014	GM26706	0	0	0	0	0	0	0	0	0	0	0	1
ENSMUSG00000097016	GM26708	0	0	0	0	0	0	0	0	0	0	0	1
ENSMUSG00000097019	GM26663	0	0	0	0	0	0	1	0	0	0	0	0
ENSMUSG00000097020	GM26613	1	0	0	0	0	0	0	0	0	1	1	0
ENSMUSG00000097021	4933433G15I	0	0	0	0	0	0	2	1	0	0	1	0
ENSMUSG00000097022	BC001981	0	1	0	0	0	0	4	0	1	3	1	2
ENSMUSG00000097026	GM26786	0	0	0	0	0	1	1	0	0	2	2	1
ENSMUSG00000097028	PTGS2OS	0	0	0	0	0	0	0	1	0	0	0	0
ENSMUSG00000097030	GM26693	0	0	0	0	0	0	1	0	0	0	0	0
ENSMUSG00000097032	4930539J05R	0	0	0	0	1	0	0	1	0	0	0	0
ENSMUSG00000097033	GM26819	1	0	0	0	0	0	5	7	6	3	4	3
ENSMUSG00000097035	GM26692	0	0	0	0	0	0	1	0	1	1	0	1
ENSMUSG00000097039	PVT1	0	5	2	5	6	4	22	23	10	14	14	9
ENSMUSG00000097042	GM17491	3	3	1	5	2	1	9	12	11	12	8	7
ENSMUSG00000097047	1110020A21F	0	0	0	0	1	0	1	0	1	0	0	0
ENSMUSG00000097048	1600020E01F	3	3	5	8	7	3	28	35	14	19	20	19
ENSMUSG00000097051	GM26836	0	0	0	0	0	1	0	1	0	1	0	0
ENSMUSG00000097055	GM4419	0	0	0	0	0	0	0	1	1	1	0	1
ENSMUSG00000097056	GM4262	0	0	0	0	0	0	0	0	1	1	1	0
ENSMUSG00000097057	GM17638	1	0	0	1	0	0	1	5	0	5	1	6
ENSMUSG00000097059	FAM120AOS	0	0	0	0	0	0	1	1	4	2	1	1
ENSMUSG00000097060	GM26759	9	7	6	23	14	14	34	36	24	53	27	28
ENSMUSG00000097061	9330151L19F	1	1	1	3	0	0	4	5	3	7	5	10
ENSMUSG00000097062	GM17586	1	1	0	0	0	0	4	4	0	0	1	1
ENSMUSG00000097073	9430037G07I	0	0	0	0	0	0	0	1	0	1	0	0
ENSMUSG00000097075	D830044I16R	0	0	0	0	1	0	0	0	0	0	0	0
ENSMUSG00000097077	GM16712	0	0	0	0	0	0	1	1	0	0	0	1
ENSMUSG00000097080	1700086O06I	0	2	0	1	0	0	5	4	3	3	2	3
ENSMUSG00000097081	GM10425	0	0	0	0	0	0	0	0	0	1	0	0
ENSMUSG00000097086	GM7672	0	0	0	0	0	0	0	0	0	0	1	0
ENSMUSG00000097088	GM26615	0	0	0	0	0	1	0	0	0	0	0	1
ENSMUSG00000097089	GM26879	4	6	6	12	7	5	12	18	13	30	8	22
ENSMUSG00000097090	GM26724	0	0	0	0	1	0	0	0	1	2	3	1

ENSMUSG00000097093	C330013E15F	1	0	0	1	0	0	2	0	1	1	1	0
ENSMUSG00000097099	GM9917	0	0	0	0	1	0	4	3	5	8	2	2
ENSMUSG00000097101	1810034E14F	0	1	1	1	0	1	3	1	2	4	0	2
ENSMUSG00000097103	GM2885	0	1	0	0	0	0	0	0	0	0	2	0
ENSMUSG00000097104	GM26579	0	0	0	0	0	0	0	0	1	0	0	0
ENSMUSG00000097107	PLATR6	0	0	0	0	0	0	0	1	0	0	0	0
ENSMUSG00000097109	GM26580	0	0	0	1	0	0	1	3	2	2	0	0
ENSMUSG00000097111	PEAK1OS	0	0	0	0	0	1	0	0	0	0	0	0
ENSMUSG00000097113	GM19705	1	1	1	4	2	2	8	5	7	6	9	9
ENSMUSG00000097117	GM26730	0	0	0	1	0	0	0	0	0	0	0	0
ENSMUSG00000097119	B230354K17F	14	20	13	18	15	10	52	65	43	66	47	44
ENSMUSG00000097121	D130020L05F	0	0	1	1	0	1	3	2	1	6	0	2
ENSMUSG00000097123	GM6297	0	0	0	0	1	0	2	1	0	1	1	0
ENSMUSG00000097125	GM26885	0	0	0	1	1	0	2	3	2	0	0	0
ENSMUSG00000097128	GM26884	0	0	0	0	0	0	0	1	0	0	0	0
ENSMUSG00000097131	D230017M19	0	0	0	0	0	0	1	0	0	2	0	0
ENSMUSG00000097133	GM26628	0	0	0	0	0	0	0	0	0	0	1	0
ENSMUSG00000097136	GM26772	1	0	1	1	0	0	1	1	1	2	2	1
ENSMUSG00000097138	GM26625	2	1	0	2	1	0	5	5	4	7	3	9
ENSMUSG00000097139	GM26626	0	0	0	1	0	0	0	0	0	0	0	0
ENSMUSG00000097140	GM26779	0	1	0	0	0	0	3	3	1	2	1	1
ENSMUSG00000097145	9230114K14F	3	6	0	4	3	4	9	8	9	12	7	14
ENSMUSG00000097146	GM4211	0	0	0	0	0	0	0	0	1	0	0	0
ENSMUSG00000097148	GM3839	1	1	0	1	2	0	0	2	0	0	1	1
ENSMUSG00000097149	G630030J09F	0	0	0	0	0	0	0	0	0	0	0	2
ENSMUSG00000097150	GM26513	0	0	0	0	0	0	1	0	0	0	0	0
ENSMUSG00000097151	GM26514	0	0	0	0	0	0	0	1	0	0	0	1
ENSMUSG00000097152	GM26515	0	2	1	0	0	1	0	0	0	1	0	1
ENSMUSG00000097154	GM26510	1	0	2	0	1	0	2	1	2	2	3	3
ENSMUSG00000097155	GM26511	0	1	1	1	0	1	2	1	3	0	1	1
ENSMUSG00000097158	GM26516	0	0	0	0	0	0	0	0	1	0	0	0
ENSMUSG00000097160	A630072L19F	0	0	0	1	0	0	0	0	0	2	1	1
ENSMUSG00000097162	2310010J17R	1	1	0	0	2	0	7	3	7	5	8	8
ENSMUSG00000097163	BC051077	0	0	0	0	1	0	0	0	0	0	0	0
ENSMUSG00000097164	CEP83OS	0	0	0	1	0	0	1	2	1	1	0	1
ENSMUSG00000097165	2210008F06F	0	0	0	0	0	0	0	0	0	2	0	0
ENSMUSG00000097166	9330179D12I	0	1	3	0	0	0	2	1	3	1	1	2
ENSMUSG00000097167	GM16740	0	0	0	1	1	1	3	5	2	0	5	1
ENSMUSG00000097168	C230088H06I	0	0	0	0	0	0	1	0	0	0	0	0
ENSMUSG00000097174	GM4890	1	0	1	0	1	1	3	4	5	5	2	6
ENSMUSG00000097176	GM26830	0	0	1	1	0	0	2	1	2	2	2	1
ENSMUSG00000097177	9330159M07	1	0	0	1	1	1	1	2	1	0	1	0
ENSMUSG00000097178	2310002F09F	0	0	0	0	0	0	0	0	1	0	0	0
ENSMUSG00000097180	2700038G22I	3	4	4	3	3	6	19	16	26	14	16	24
ENSMUSG00000097185	GM26596	1	0	0	1	0	0	5	5	2	2	1	1
ENSMUSG00000097186	GM26595	0	0	0	0	0	0	0	1	0	0	0	1
ENSMUSG00000097189	GM26594	0	0	0	0	0	0	0	1	0	1	0	0
ENSMUSG00000097191	5730422E09F	0	0	0	0	0	0	0	0	0	2	0	0
ENSMUSG00000097193	GM26664	0	0	2	0	0	0	2	0	2	3	2	2
ENSMUSG00000097194	9330175E14F	1	0	0	0	0	0	1	0	0	0	1	0
ENSMUSG00000097195	SNHG5	33	32	42	65	53	46	261	237	148	249	167	189
ENSMUSG00000097197	9530027J09R	0	0	1	2	1	0	3	1	3	3	2	2
ENSMUSG00000097203	4732419C18F	3	1	0	1	1	2	10	10	5	5	7	1
ENSMUSG00000097204	GM17690	1	0	0	0	0	0	1	0	1	2	1	1
ENSMUSG00000097206	GM26699	27	15	21	29	31	24	54	42	51	59	40	43
ENSMUSG00000097207	6030443J06R	0	0	0	0	0	0	1	0	0	1	0	1
ENSMUSG00000097208	GM26698	0	0	0	0	0	0	1	0	0	0	0	0
ENSMUSG00000097211	BC065403	0	1	1	0	0	0	1	0	0	0	0	0

ENSMUSG00000097213	GM26745	0	0	1	0	0	0	2	0	1	0	1	0
ENSMUSG00000097217	GM26549	0	0	0	0	0	0	4	3	0	3	1	0
ENSMUSG00000097219	GM26551	1	1	1	3	2	1	3	3	3	2	3	3
ENSMUSG00000097223	GM2449	0	0	0	0	0	0	0	0	0	0	1	0
ENSMUSG00000097230	GM26853	0	1	0	0	0	0	0	2	1	0	0	0
ENSMUSG00000097231	GM26852	0	0	1	1	0	1	1	0	2	1	1	1
ENSMUSG00000097233	GM17552	0	0	0	0	0	0	0	1	0	0	0	0
ENSMUSG00000097234	GM26518	2	0	1	0	1	0	1	0	2	0	2	2
ENSMUSG00000097236	4831440E17F	0	0	0	0	0	0	0	0	0	1	0	1
ENSMUSG00000097239	GM27029	0	0	0	0	1	1	1	2	3	3	0	4
ENSMUSG00000097246	GM26890	0	0	1	1	0	0	3	5	0	1	3	4
ENSMUSG00000097250	GM26771	0	0	0	0	0	0	0	0	0	0	0	2
ENSMUSG00000097251	5033417F24F	0	1	0	0	0	0	0	0	0	0	0	0
ENSMUSG00000097253	GM26770	1	16	2	1	3	1	10	7	4	3	5	4
ENSMUSG00000097255	RPL31-PS7	1	0	0	0	0	0	0	0	0	2	0	0
ENSMUSG00000097257	GM26768	0	0	0	0	0	0	0	1	0	2	1	0
ENSMUSG00000097258	GM26767	0	1	0	0	0	0	4	3	3	1	4	6
ENSMUSG00000097259	GM26766	0	0	0	0	0	0	1	0	0	0	0	0
ENSMUSG00000097261	4930532G15I	5	2	2	1	1	1	4	8	4	3	7	3
ENSMUSG00000097262	4933416M07	0	0	0	0	0	0	1	0	0	0	1	0
ENSMUSG00000097263	GM26804	0	0	0	0	0	0	0	1	0	0	0	0
ENSMUSG00000097271	GM9903	0	0	0	0	0	0	0	0	0	1	0	0
ENSMUSG00000097272	GM26649	1	0	4	5	0	4	6	4	4	12	7	9
ENSMUSG00000097274	GM47512	0	0	0	1	1	0	1	1	0	1	1	1
ENSMUSG00000097275	GM26648	0	0	1	0	2	0	2	1	1	2	0	0
ENSMUSG00000097277	2900076A07F	3	2	7	2	7	9	20	21	11	18	13	17
ENSMUSG00000097279	GM5106	0	0	0	0	0	0	4	0	1	4	0	6
ENSMUSG00000097280	AI849053	0	0	0	0	0	0	0	0	1	1	0	0
ENSMUSG00000097284	4930480K23F	0	0	0	0	0	1	0	0	0	0	0	0
ENSMUSG00000097287	D130017N08	0	0	0	0	0	0	0	1	0	0	0	0
ENSMUSG00000097290	1300002E11F	0	2	3	1	4	0	5	7	3	10	3	9
ENSMUSG00000097292	A230107N01I	1	0	0	0	0	0	0	0	0	0	0	3
ENSMUSG00000097295	HMGB1-PS8	0	0	0	1	0	0	0	1	0	0	1	0
ENSMUSG00000097296	GM26532	0	0	0	1	1	2	1	0	0	1	1	1
ENSMUSG00000097300	GM26835	0	0	0	0	0	0	1	0	0	0	0	0
ENSMUSG00000097301	AW121686	1	0	0	0	0	0	0	0	0	1	0	0
ENSMUSG00000097303	3110083C13F	1	0	0	0	1	0	1	0	1	2	0	2
ENSMUSG00000097304	RPL31-PS15	0	2	2	2	1	2	9	8	5	4	4	4
ENSMUSG00000097305	GM17276	0	0	0	0	0	0	0	1	1	0	0	0
ENSMUSG00000097307	GM26834	0	0	0	0	0	0	2	0	1	1	1	0
ENSMUSG00000097309	GM17249	0	0	0	0	0	0	1	1	0	0	0	2
ENSMUSG00000097312	GM26870	1	4	1	2	1	6	40	10	1	29	4	0
ENSMUSG00000097316	GM10516	0	0	1	0	0	0	1	0	0	0	0	0
ENSMUSG00000097317	GM17281	0	0	0	0	0	0	0	1	0	1	0	0
ENSMUSG00000097318	1700007L15R	0	0	0	0	0	0	1	0	0	0	0	1
ENSMUSG00000097323	4930426I24R	0	0	0	0	0	0	1	0	0	2	1	0
ENSMUSG00000097325	GM16897	0	0	0	0	0	0	0	0	1	0	1	0
ENSMUSG00000097327	E030030I06R	1	5	2	3	4	3	13	9	12	14	12	15
ENSMUSG00000097331	F420014N23I	1	0	0	0	0	0	2	5	1	5	1	1
ENSMUSG00000097333	ZFP87	2	0	0	1	1	2	9	7	4	8	2	5
ENSMUSG00000097334	2700012I20R	0	0	0	0	0	0	2	0	0	0	0	1
ENSMUSG00000097335	GM26563	0	0	0	2	3	0	3	9	4	7	4	4
ENSMUSG00000097337	GM26606	0	1	1	1	0	0	1	3	0	2	0	3
ENSMUSG00000097338	GM2464	0	0	0	0	0	0	0	0	0	1	0	0
ENSMUSG00000097339	GM26671	0	0	0	0	0	0	2	4	0	1	0	2
ENSMUSG00000097340	GM26617	0	2	0	3	0	1	1	4	1	4	0	1
ENSMUSG00000097343	9030407P20F	0	0	0	0	0	0	0	0	0	0	1	1
ENSMUSG00000097345	AA543186	0	0	0	1	0	0	0	0	0	0	1	1

ENSMUSG00000097347	GM17275	3	1	0	0	2	2	12	8	15	12	12	14
ENSMUSG00000097350	4732491K20R	0	1	0	0	0	1	2	0	0	1	0	2
ENSMUSG00000097353	A430046D13R	0	0	0	1	0	0	7	5	5	2	5	3
ENSMUSG00000097354	2310001H17R	4	2	8	4	11	6	25	20	17	32	22	32
ENSMUSG00000097357	GM16793	0	0	0	0	0	0	3	1	1	1	1	3
ENSMUSG00000097360	9430065F17R	1	0	0	0	3	1	2	2	2	3	2	3
ENSMUSG00000097364	GM26719	0	0	0	0	0	0	0	0	0	1	0	1
ENSMUSG00000097365	C030034L19R	1	3	2	2	1	0	5	2	1	4	1	3
ENSMUSG00000097367	GM26718	0	2	2	0	0	1	3	1	4	5	6	3
ENSMUSG00000097368	GM10390	0	0	0	0	0	0	0	0	0	1	0	0
ENSMUSG00000097369	GM26545	0	1	0	0	0	0	0	0	0	1	0	0
ENSMUSG00000097370	4930467K11R	0	0	0	0	0	0	1	0	0	0	0	0
ENSMUSG00000097375	6720427I07R	0	3	3	7	2	3	17	13	5	14	8	7
ENSMUSG00000097378	B230208H11R	4	1	0	1	1	1	9	4	5	15	3	5
ENSMUSG00000097379	GM26873	0	3	1	2	0	2	5	6	5	4	3	11
ENSMUSG00000097381	A230087F16R	0	0	0	0	0	0	1	0	0	0	0	0
ENSMUSG00000097383	1500026H17R	1	0	1	0	0	0	1	0	0	0	1	0
ENSMUSG00000097390	GM26785	0	0	0	0	0	0	1	0	1	2	0	1
ENSMUSG00000097392	D930016D06R	8	7	7	6	7	12	47	27	37	47	30	26
ENSMUSG00000097397	GM16861	0	0	0	0	0	0	0	0	0	0	1	1
ENSMUSG00000097399	GM26555	0	0	0	0	0	0	0	0	0	0	1	0
ENSMUSG00000097402	GM17399	0	1	0	0	0	0	0	0	0	0	0	0
ENSMUSG00000097403	9230116N13R	0	0	0	0	0	0	0	0	0	1	1	0
ENSMUSG00000097405	D630044L22R	0	0	0	0	0	0	0	0	0	0	1	0
ENSMUSG00000097407	4933408J17R	3	6	7	7	4	7	11	14	5	12	6	16
ENSMUSG00000097410	GM26668	0	1	1	1	1	0	1	1	0	2	1	2
ENSMUSG00000097411	B430218F22R	0	0	0	1	0	0	0	0	0	0	0	0
ENSMUSG00000097412	1810014B01R	1	4	4	3	4	3	38	31	36	41	28	28
ENSMUSG00000097414	B130046B21R	1	0	0	0	1	0	0	0	0	1	0	1
ENSMUSG00000097415	AU020206	22	16	25	26	24	20	82	114	86	112	96	72
ENSMUSG00000097416	GM26670	0	0	0	0	0	0	1	0	0	0	0	2
ENSMUSG00000097417	GM26669	0	4	6	15	5	9	38	38	19	41	23	34
ENSMUSG00000097418	MIR155HG	0	0	0	0	0	1	1	1	0	1	0	0
ENSMUSG00000097422	GM26521	1	2	0	2	0	0	1	1	1	2	2	0
ENSMUSG00000097423	GM26522	1	1	2	3	0	3	5	0	2	2	2	3
ENSMUSG00000097424	1700030M09	0	0	0	0	0	0	0	0	0	1	0	0
ENSMUSG00000097428	AW047730	0	1	0	0	0	0	1	0	3	2	0	0
ENSMUSG00000097429	GM26520	1	1	2	2	2	0	3	1	1	4	1	2
ENSMUSG00000097431	GM26782	1	0	0	1	1	0	2	2	2	3	3	4
ENSMUSG00000097439	GM16754	1	0	1	1	0	0	6	0	0	3	0	3
ENSMUSG00000097443	GM17529	0	0	0	0	0	0	1	2	0	0	0	0
ENSMUSG00000097445	GM26631	2	6	5	6	9	6	26	27	30	41	29	35
ENSMUSG00000097448	PLATR22	0	0	0	0	0	0	0	0	0	0	0	1
ENSMUSG00000097454	GM26892	0	0	0	0	0	0	0	0	1	0	0	0
ENSMUSG00000097457	2310031A07R	0	0	0	1	0	0	2	0	2	2	0	1
ENSMUSG00000097458	GM26697	0	0	0	0	0	0	0	0	0	1	0	0
ENSMUSG00000097460	GM26734	0	0	0	0	0	0	2	1	1	0	0	0
ENSMUSG00000097461	GM26735	0	0	0	0	0	0	0	0	0	1	0	0
ENSMUSG00000097467	GM26737	1	0	0	3	1	0	5	2	0	2	3	0
ENSMUSG00000097468	MDRL	0	1	0	2	0	0	2	2	1	2	1	3
ENSMUSG00000097469	GM26733	1	2	3	5	2	2	17	14	8	14	12	9
ENSMUSG00000097472	GM26586	0	0	0	0	0	0	2	1	0	1	1	3
ENSMUSG00000097476	GM26583	0	0	0	1	0	0	0	0	0	0	1	0
ENSMUSG00000097479	GM26582	5	5	2	5	3	5	21	26	19	22	14	21
ENSMUSG00000097487	PTGES3L	0	0	0	0	0	0	1	3	1	0	1	1
ENSMUSG00000097488	4732487G21R	0	0	0	0	0	1	2	3	4	0	2	0
ENSMUSG00000097493	9930014A18R	0	0	0	0	0	0	1	1	0	0	0	0
ENSMUSG00000097494	4933406C10R	0	0	0	0	0	0	1	2	2	3	1	2

ENSMUSG00000097503	3110045C21R	0	0	1	0	0	4	1	1	4	2	3	4
ENSMUSG00000097507	GM26538	1	1	0	0	0	0	1	2	0	1	0	0
ENSMUSG00000097509	B230322F03R	0	0	0	0	0	0	0	0	0	0	1	0
ENSMUSG00000097515	1700040D17R	1	0	0	0	0	0	0	0	0	0	0	0
ENSMUSG00000097517	GM26695	1	0	0	0	0	1	0	0	0	1	1	1
ENSMUSG00000097518	GM26694	2	1	1	0	0	3	10	12	7	11	10	1
ENSMUSG00000097519	4930558J18R	0	0	0	2	0	0	2	2	1	3	3	2
ENSMUSG00000097523	1700022N22R	0	0	0	0	0	0	0	1	1	0	0	1
ENSMUSG00000097525	PLATR31	0	0	0	0	0	0	0	0	1	1	0	0
ENSMUSG00000097526	GM26847	0	0	0	0	0	0	0	0	2	0	0	0
ENSMUSG00000097529	RP31-PS19	0	0	0	0	0	0	1	0	1	0	0	0
ENSMUSG00000097533	GM26590	0	0	0	1	0	1	6	2	5	2	0	3
ENSMUSG00000097534	GM16675	0	2	0	1	0	2	2	0	0	2	2	3
ENSMUSG00000097535	GM26592	0	0	0	0	0	0	1	0	0	0	0	0
ENSMUSG00000097536	2610037D02R	0	1	1	0	1	1	2	4	2	4	4	0
ENSMUSG00000097537	2610020C07R	2	3	1	4	2	1	5	7	6	17	5	10
ENSMUSG00000097546	GM26749	0	0	1	0	0	0	0	2	0	0	1	0
ENSMUSG00000097552	RPS19-PS12	1	0	0	0	0	0	0	0	0	0	1	0
ENSMUSG00000097554	GM26825	0	2	2	1	2	1	3	10	9	8	9	4
ENSMUSG00000097557	C130060C02R	0	0	0	0	0	0	1	0	1	1	1	0
ENSMUSG00000097560	GM26904	0	0	0	0	0	0	0	0	1	0	0	0
ENSMUSG00000097564	C430014B12R	0	0	0	0	1	0	0	0	0	0	0	0
ENSMUSG00000097567	GM26637	0	0	0	0	0	0	0	0	0	0	1	0
ENSMUSG00000097571	JPX	2	3	2	1	2	1	11	7	6	14	4	7
ENSMUSG00000097572	GM26797	0	0	1	0	0	0	1	0	0	0	0	0
ENSMUSG00000097573	G730003C15R	0	2	0	0	0	0	1	0	0	0	0	0
ENSMUSG00000097574	C920006O11R	0	0	3	0	0	2	2	1	2	3	2	0
ENSMUSG00000097576	D930030I03R	1	0	0	1	0	0	0	1	0	2	1	0
ENSMUSG00000097577	6230400D17R	0	1	0	0	0	0	2	2	0	2	1	2
ENSMUSG00000097578	GM26798	0	0	0	0	0	1	1	0	1	0	0	0
ENSMUSG00000097579	GM26799	1	1	0	5	2	1	6	1	6	3	2	2
ENSMUSG00000097580	GM5432	0	1	0	0	0	0	0	0	0	0	0	0
ENSMUSG00000097582	GM26527	1	2	1	0	0	1	0	3	2	2	1	1
ENSMUSG00000097583	6430590A07R	0	0	3	1	1	2	2	1	5	4	3	0
ENSMUSG00000097585	E230029C05R	0	0	0	1	1	1	0	2	0	1	0	0
ENSMUSG00000097587	4930578M01	0	0	0	1	0	0	0	1	1	1	1	0
ENSMUSG00000097589	DLEU2	9	18	8	24	13	10	53	63	54	59	45	41
ENSMUSG00000097590	GM26675	0	0	0	1	0	0	1	0	1	1	0	5
ENSMUSG00000097591	A330032B11R	0	0	0	0	0	0	1	0	0	0	1	0
ENSMUSG00000097595	1500002F19R	0	0	0	0	0	0	3	0	1	0	0	1
ENSMUSG00000097603	A430010J10R	0	1	0	0	0	0	0	0	0	0	1	2
ENSMUSG00000097605	9430098F02R	0	1	0	0	0	0	3	5	0	4	0	1
ENSMUSG00000097614	GM26508	0	0	1	2	0	0	1	1	0	0	0	0
ENSMUSG00000097616	1110019D14R	0	0	0	0	0	1	2	3	4	8	3	3
ENSMUSG00000097617	GM10687	0	0	0	0	0	0	1	0	0	0	0	1
ENSMUSG00000097621	GM26562	0	0	0	0	0	0	0	0	0	0	0	1
ENSMUSG00000097622	A330033J07R	0	0	0	0	0	0	0	0	0	0	0	1
ENSMUSG00000097625	GM26561	0	0	0	0	0	0	0	1	0	0	1	0
ENSMUSG00000097632	4930552P12R	0	0	0	0	0	0	0	1	0	0	0	0
ENSMUSG00000097635	GM26826	0	0	0	0	0	0	0	1	0	0	1	0
ENSMUSG00000097639	PLATR4	0	3	0	1	1	3	5	11	5	2	1	1
ENSMUSG00000097640	GM20033	0	1	0	0	0	0	0	0	0	0	0	0
ENSMUSG00000097643	A130051J06R	1	0	0	1	1	0	0	1	1	0	0	0
ENSMUSG00000097649	GM10561	0	0	1	0	0	1	0	1	1	0	0	0
ENSMUSG00000097657	GM7389	0	0	0	0	0	0	0	0	1	0	0	0
ENSMUSG00000097658	GM16755	0	0	1	9	0	0	2	2	2	3	2	4
ENSMUSG00000097660	GM26762	0	0	0	0	0	1	1	1	0	0	0	0
ENSMUSG00000097662	GM2093	0	1	0	0	0	0	0	0	0	0	0	0

ENSMUSG00000097664	GM26765	1	0	0	0	0	0	1	3	1	0	1	3
ENSMUSG00000097665	GM16853	3	1	4	2	0	1	9	11	5	10	7	3
ENSMUSG00000097667	GM26764	0	0	0	0	0	0	0	1	1	2	0	1
ENSMUSG00000097669	GM8649	1	2	0	2	2	0	4	6	1	2	2	6
ENSMUSG00000097672	GM26609	2	0	0	0	0	0	2	2	0	3	0	0
ENSMUSG00000097674	GM26610	0	0	0	1	0	0	0	0	2	3	0	0
ENSMUSG00000097675	1700101I11R	1	2	0	0	0	0	1	3	2	0	0	3
ENSMUSG00000097679	RPS19-PS5	0	0	0	0	0	0	0	0	0	1	0	0
ENSMUSG00000097680	GM26642	0	0	0	0	0	0	0	0	1	0	1	0
ENSMUSG00000097681	GM26643	0	0	0	0	0	0	1	0	0	1	1	0
ENSMUSG00000097685	GM26646	0	0	0	0	0	0	0	1	0	0	0	0
ENSMUSG00000097693	4930471M09	0	0	0	0	0	0	0	0	1	0	0	0
ENSMUSG00000097694	G730013B05I	1	3	0	0	2	0	3	4	7	0	1	3
ENSMUSG00000097699	5430400D12F	0	0	0	0	0	0	0	1	0	0	1	1
ENSMUSG00000097702	GM26739	0	0	0	1	0	0	1	1	0	0	0	2
ENSMUSG00000097703	GM26623	1	0	0	0	2	0	1	2	1	2	1	3
ENSMUSG00000097704	GM26741	0	0	0	0	0	0	0	0	1	0	0	0
ENSMUSG00000097706	E030037K01F	0	0	0	0	0	0	0	1	0	0	0	0
ENSMUSG00000097711	GM5523	0	0	0	0	0	0	0	0	0	1	0	0
ENSMUSG00000097715	GPR137B-PS	1	0	1	0	0	0	1	4	1	3	4	2
ENSMUSG00000097718	GM26896	0	0	1	0	0	0	1	0	1	1	0	0
ENSMUSG00000097722	GM26841	0	0	0	0	0	0	0	1	1	0	0	0
ENSMUSG00000097723	GM26848	0	0	0	0	1	0	0	0	0	0	1	1
ENSMUSG00000097729	2310015A10F	0	1	0	1	0	0	2	5	2	3	2	2
ENSMUSG00000097731	2700016F22R	6	7	6	1	8	4	5	8	5	13	9	16
ENSMUSG00000097737	GM26530	3	4	3	8	5	6	19	37	21	16	16	31
ENSMUSG00000097738	4930445N18I	0	0	0	0	0	0	0	1	0	0	0	0
ENSMUSG00000097740	E030044B06F	0	0	0	0	0	0	0	0	1	0	0	0
ENSMUSG00000097743	GM16973	5	3	5	3	10	4	28	32	16	23	28	19
ENSMUSG00000097746	GM6225	0	0	0	0	0	0	0	1	0	0	0	0
ENSMUSG00000097747	GM26534	0	0	0	0	0	1	1	0	3	4	1	1
ENSMUSG00000097748	GM26533	0	2	0	1	1	2	4	6	4	2	1	1
ENSMUSG00000097750	GM4673	1	2	0	0	2	1	5	6	4	4	2	5
ENSMUSG00000097751	GM26690	0	3	1	2	0	0	1	4	7	3	2	4
ENSMUSG00000097755	2010110K18F	1	0	2	2	1	0	7	1	2	3	2	2
ENSMUSG00000097757	GM17518	0	0	0	0	0	0	0	0	1	0	1	0
ENSMUSG00000097759	GM26753	2	0	1	0	0	2	6	5	0	6	3	3
ENSMUSG00000097760	6030442K20F	0	0	1	1	0	0	1	1	1	3	1	1
ENSMUSG00000097762	4732463B04F	0	0	0	0	0	0	0	1	0	0	0	0
ENSMUSG00000097763	GM26636	0	0	1	0	1	0	7	2	0	6	2	5
ENSMUSG00000097772	5430416N02I	11	3	8	13	9	9	34	57	33	63	46	31
ENSMUSG00000097775	4931440J10R	0	0	0	0	0	0	0	0	0	1	0	0
ENSMUSG00000097777	GM16794	2	1	0	0	1	0	4	7	2	4	4	2
ENSMUSG00000097781	9330136K24F	0	1	1	0	0	1	0	1	1	2	0	1
ENSMUSG00000097784	A430105J06R	0	0	0	0	0	0	2	1	1	1	0	0
ENSMUSG00000097785	B230217O12I	0	1	2	1	0	1	2	1	1	2	1	1
ENSMUSG00000097786	4933429H19I	1	0	0	1	0	0	2	0	0	1	1	0
ENSMUSG00000097787	2700046G09I	0	0	0	0	0	0	0	2	0	2	0	1
ENSMUSG00000097792	GM27042	0	0	0	0	0	0	2	2	0	2	0	2
ENSMUSG00000097793	GM17259	0	0	0	1	1	0	5	2	2	4	2	2
ENSMUSG00000097796	GM16702	2	4	6	3	5	2	14	22	15	18	19	19
ENSMUSG00000097797	GM26901	0	0	0	0	0	0	2	2	0	2	2	1
ENSMUSG00000097799	GM26899	4	3	5	5	7	3	8	13	8	20	10	11
ENSMUSG00000097800	B230344G16I	0	0	0	0	0	0	0	1	0	0	0	0
ENSMUSG00000097803	RPL31-PS16	0	0	0	0	0	0	1	1	0	2	0	1
ENSMUSG00000097811	2810425M01	0	0	0	0	0	1	3	1	3	1	3	3
ENSMUSG00000097812	GM26812	3	3	2	2	2	5	14	13	12	17	11	15
ENSMUSG00000097815	GM26809	0	1	1	0	0	0	0	0	3	0	1	1

ENSMUSG00000097817	GM26810	0	0	1	0	0	0	0	1	0	0	1	0
ENSMUSG00000097818	GM26681	1	0	0	0	0	0	0	0	1	0	0	0
ENSMUSG00000097820	E530011L22R	3	3	2	1	1	6	14	13	13	15	7	15
ENSMUSG00000097824	GM26593	0	2	0	0	0	0	0	0	0	0	0	1
ENSMUSG00000097828	6430562O15I	0	0	0	0	0	1	0	0	1	2	0	0
ENSMUSG00000097831	4930540M05	2	6	8	3	7	3	18	18	16	18	13	19
ENSMUSG00000097832	GM26912	0	0	0	0	0	1	0	0	0	0	0	0
ENSMUSG00000097835	GM26910	0	0	2	0	0	2	1	0	0	0	2	1
ENSMUSG00000097839	GM26909	1	0	0	0	0	0	3	2	2	2	0	0
ENSMUSG00000097842	9330104G04I	0	0	0	0	0	0	0	0	0	3	0	0
ENSMUSG00000097845	A230020J21R	0	0	0	0	0	0	0	0	1	0	0	0
ENSMUSG00000097855	A930007I19R	1	0	0	2	0	0	0	0	0	1	0	0
ENSMUSG00000097858	9530052C20F	0	0	0	0	0	0	0	0	1	0	0	0
ENSMUSG00000097859	GM26601	1	0	0	0	0	0	2	4	0	3	0	1
ENSMUSG00000097860	GM26857	0	0	0	0	0	0	0	0	0	1	0	0
ENSMUSG00000097861	GM26858	0	0	0	0	0	1	0	0	0	0	0	0
ENSMUSG00000097864	GM26856	0	1	0	0	0	1	0	3	0	2	0	0
ENSMUSG00000097867	LPPOS	1	2	2	0	0	1	4	7	5	4	2	3
ENSMUSG00000097868	A330069E16F	0	1	1	2	0	0	10	6	5	3	3	6
ENSMUSG00000097872	GM26702	0	0	0	0	0	0	1	0	0	1	0	0
ENSMUSG00000097874	C030010L15F	0	1	0	0	0	0	0	0	0	1	0	1
ENSMUSG00000097875	GM26704	0	1	0	0	0	0	1	1	0	0	1	0
ENSMUSG00000097876	GM16892	0	0	0	0	0	0	0	0	0	0	1	0
ENSMUSG00000097879	GM26869	0	0	0	0	0	0	0	0	0	1	0	0
ENSMUSG00000097882	0610038B21F	1	0	0	0	0	0	1	0	1	1	1	1
ENSMUSG00000097883	GM8357	2	0	1	0	1	0	1	4	2	1	1	2
ENSMUSG00000097885	5031434O11I	3	2	0	1	0	0	0	1	4	2	2	1
ENSMUSG00000097887	GM26542	0	0	1	1	0	0	0	0	0	0	0	1
ENSMUSG00000097889	GM26541	2	2	1	1	5	2	8	11	3	10	5	5
ENSMUSG00000097892	GM26801	0	0	0	0	0	0	0	0	0	0	1	0
ENSMUSG00000097893	1700034P13F	0	0	1	0	0	0	0	1	0	0	2	0
ENSMUSG00000097906	GM9625	0	0	0	1	0	0	1	2	0	1	1	0
ENSMUSG00000097908	4933404O12I	0	0	0	0	0	0	0	0	1	0	0	0
ENSMUSG00000097917	GM26839	0	0	0	1	0	1	3	1	1	0	0	2
ENSMUSG00000097926	GM26575	0	0	0	0	0	0	1	1	1	0	1	0
ENSMUSG00000097937	GM6967	0	0	0	1	0	0	0	0	0	0	0	0
ENSMUSG00000097943	GM7833	0	0	0	0	0	0	0	0	0	1	0	0
ENSMUSG00000097944	A130014A01I	0	3	1	1	3	2	5	4	2	3	5	3
ENSMUSG00000097971	GM26917	130	207	170	216	165	178	306	371	265	359	230	333
ENSMUSG00000097973	GM4929	0	0	0	0	0	0	0	0	1	0	0	0
ENSMUSG00000097977	GM5652	0	0	0	0	0	0	0	0	0	0	0	1
ENSMUSG00000097979	GM4691	0	0	0	0	0	0	0	0	0	0	0	1
ENSMUSG00000097987	GM17786	0	0	0	0	1	0	1	1	0	1	2	0
ENSMUSG00000097988	GM10535	0	0	0	0	0	0	0	0	1	1	0	0
ENSMUSG00000097989	GM4335	0	0	0	0	0	0	1	1	0	0	1	0
ENSMUSG00000097993	PTPRV	3	1	0	2	5	1	0	6	5	5	6	4
ENSMUSG00000097994	GM26982	0	0	0	0	0	0	0	4	1	5	0	2
ENSMUSG00000097995	GM24362	0	0	0	0	0	0	1	0	1	1	0	0
ENSMUSG00000097997	GM27017	1	1	0	0	0	0	0	0	1	0	0	1
ENSMUSG00000098001	GM27048	0	0	0	0	0	0	0	0	0	0	1	0
ENSMUSG00000098008	A930001A20I	0	1	0	1	0	1	5	3	1	6	3	3
ENSMUSG00000098012	GM8907	0	0	0	0	0	0	0	0	0	1	0	0
ENSMUSG00000098022	ZFP82	0	1	0	0	0	0	1	1	0	0	0	0
ENSMUSG00000098024	GM27003	0	0	0	1	2	0	0	0	0	2	0	0
ENSMUSG00000098033	GM9381	0	0	0	0	0	0	0	0	1	0	0	0
ENSMUSG00000098039	GM26925	0	0	0	0	0	0	0	0	0	0	0	1
ENSMUSG00000098041	GM26981	0	1	0	1	1	0	0	0	2	3	2	1
ENSMUSG00000098042	GM5710	0	0	0	0	0	0	0	0	0	1	0	0

ENSMUSG00000098044	GM9162	0	0	0	0	0	0	0	0	0	0	1	0
ENSMUSG00000098065	GM5177	0	0	0	0	0	0	0	0	0	0	1	2
ENSMUSG00000098066	GM26944	0	0	0	0	0	1	0	1	3	3	1	0
ENSMUSG00000098082	GM20574	0	0	0	0	0	0	1	0	1	0	0	0
ENSMUSG00000098083	GM8957	0	0	0	0	0	0	0	0	0	1	0	0
ENSMUSG00000098088	GM26916	2	0	2	2	0	0	0	2	6	4	0	2
ENSMUSG00000098090	2700099C18F	5	5	3	9	8	8	34	48	38	44	38	27
ENSMUSG00000098097	6530403H02F	0	1	0	0	0	0	0	0	0	0	0	0
ENSMUSG00000098104	GM6085	0	5	2	2	2	0	3	12	5	10	9	9
ENSMUSG00000098111	GM4654	0	0	0	0	0	0	0	0	0	2	0	0
ENSMUSG00000098112	BIN2	9	10	23	10	11	6	52	72	51	64	39	75
ENSMUSG00000098113	GM2445	0	0	0	0	0	0	0	0	1	0	0	0
ENSMUSG00000098118	GM26960	0	0	0	0	0	1	1	1	0	0	1	2
ENSMUSG00000098120	GM5914	1	0	2	0	1	3	4	6	5	10	6	4
ENSMUSG00000098121	GM27038	0	0	0	0	0	0	0	1	0	1	0	0
ENSMUSG00000098134	RNF113A2	5	3	2	7	5	7	17	21	16	17	18	16
ENSMUSG00000098135	GM6946	1	0	0	0	0	0	0	0	1	0	0	1
ENSMUSG00000098136	GM8513	0	0	0	0	0	0	0	0	0	1	0	0
ENSMUSG00000098143	GM26937	0	0	0	0	0	0	0	0	0	1	0	0
ENSMUSG00000098144	1700029N11I	0	0	0	0	0	0	0	0	0	0	1	0
ENSMUSG00000098145	GM26936	0	0	0	0	1	0	1	0	0	0	0	0
ENSMUSG00000098146	GM26935	0	0	0	0	0	0	0	1	3	1	2	1
ENSMUSG00000098149	GAPDH-PS14	0	1	0	0	0	0	4	1	3	2	1	0
ENSMUSG00000098155	GM27022	0	0	0	0	0	0	0	0	1	1	0	0
ENSMUSG00000098176	CCDC166	0	3	2	1	2	0	9	10	5	3	8	7
ENSMUSG00000098178	GM42418	857	1286	1077	1990	1326	1195	7096	7184	5069	7975	5484	7127
ENSMUSG00000098181	RPS12-PS24	0	0	0	0	0	0	1	1	0	1	2	0
ENSMUSG00000098183	GM27010	1	1	2	1	1	1	9	7	9	11	1	12
ENSMUSG00000098184	GM27011	0	0	0	0	0	0	0	0	0	1	0	0
ENSMUSG00000098186	GM7132	0	0	0	0	0	0	1	1	0	1	0	0
ENSMUSG00000098187	GM8927	0	0	0	0	0	0	0	0	2	0	0	0
ENSMUSG00000098188	SOWAHC	4	5	1	4	6	6	5	9	8	8	8	9
ENSMUSG00000098190	GM5210	0	0	0	0	0	0	1	0	0	0	0	0
ENSMUSG00000098194	GM7286	0	0	0	0	0	0	0	2	0	0	0	0
ENSMUSG00000098196	GM26964	0	0	0	0	0	1	2	0	0	6	0	0
ENSMUSG00000098198	GM9169	0	1	0	0	0	0	0	0	0	0	0	0
ENSMUSG00000098200	GM7763	0	0	0	0	0	0	0	0	0	0	1	0
ENSMUSG00000098201	GM26983	0	0	0	0	0	0	0	0	0	1	0	0
ENSMUSG00000098203	GM9568	0	0	0	0	0	0	0	0	0	1	0	0
ENSMUSG00000098206	A430106G13I	0	2	1	4	1	1	8	12	2	11	8	5
ENSMUSG00000098208	GM8349	0	0	0	0	0	0	0	0	0	1	0	0
ENSMUSG00000098222	GM8318	0	0	0	0	0	0	1	0	0	0	0	0
ENSMUSG00000098224	GM7985	0	0	0	0	0	0	1	0	0	0	0	0
ENSMUSG00000098232	GM3671	0	0	0	0	0	0	1	0	0	0	0	0
ENSMUSG00000098234	SNHG6	39	48	49	64	61	50	265	259	223	272	219	244
ENSMUSG00000098235	GM7497	0	0	0	0	0	0	0	1	0	0	0	0
ENSMUSG00000098243	GM4258	3	10	4	4	6	14	38	36	27	34	22	22
ENSMUSG00000098259	GM27616	0	0	0	0	0	0	0	0	1	0	0	0
ENSMUSG00000098274	RPL24	0	1	0	0	1	3	1	1	2	1	3	1
ENSMUSG00000098286	MIR8091	0	0	0	0	0	0	1	0	0	0	0	0
ENSMUSG00000098304	1700027I24R	0	0	0	0	0	0	0	0	1	0	0	0
ENSMUSG00000098308	GM27782	0	0	0	2	0	0	1	0	0	1	0	0
ENSMUSG00000098318	LOCKD	30	36	25	29	30	31	140	141	119	149	116	114
ENSMUSG00000098332	2310009A05F	1	2	1	2	4	3	23	10	7	13	12	18
ENSMUSG00000098336	MIR6388	0	0	0	0	0	0	1	0	0	0	0	0
ENSMUSG00000098340	GM27253	0	0	0	1	0	0	0	0	0	0	0	0
ENSMUSG00000098355	GM27553	0	0	1	5	1	0	2	9	3	3	2	3
ENSMUSG00000098362	MIR130C	0	0	0	0	0	0	0	0	0	1	0	0

ENSMUSG00000098374	GM28043	0	0	0	0	0	0	1	0	0	2	0	0
ENSMUSG00000098396	GM27997	0	0	0	0	0	0	0	1	0	0	0	0
ENSMUSG00000098401	MIR6377	0	0	0	0	0	0	0	1	0	0	0	0
ENSMUSG00000098410	MIR1957B	1	0	0	0	0	0	1	1	1	0	0	0
ENSMUSG00000098412	GM7870	0	0	0	1	0	0	0	0	0	0	0	0
ENSMUSG00000098434	2010110E17F	0	0	0	0	0	0	0	0	1	0	0	0
ENSMUSG00000098439	HM629797	0	0	0	0	0	0	0	0	0	1	0	0
ENSMUSG00000098447	GM27184	0	0	0	0	0	0	0	0	0	0	0	1
ENSMUSG00000098456	GM12117	0	0	0	0	0	0	1	0	0	0	0	0
ENSMUSG00000098483	MIR7078	0	0	0	0	0	0	0	1	1	0	0	0
ENSMUSG00000098492	GM19353	0	0	0	1	0	0	1	2	0	0	2	0
ENSMUSG00000098501	GM19272	1	0	1	0	0	0	0	1	0	1	1	0
ENSMUSG00000098525	GM27365	0	0	1	0	0	0	1	0	0	0	0	0
ENSMUSG00000098559	GM15013	0	0	0	0	0	0	1	1	0	0	0	0
ENSMUSG00000098573	GM27232	0	0	1	1	0	0	0	0	0	0	0	1
ENSMUSG00000098577	GM27445	0	0	1	0	0	0	0	0	0	1	0	0
ENSMUSG00000098602	MIR6946	0	0	0	0	0	0	0	0	0	0	0	1
ENSMUSG00000098626	GM27972	1	1	0	0	0	0	0	0	1	0	0	0
ENSMUSG00000098640	GM14214	0	0	0	0	0	0	0	0	0	0	1	0
ENSMUSG00000098645	GM27201	0	0	0	0	0	0	0	1	1	0	0	1
ENSMUSG00000098674	GM27813	0	0	0	0	0	0	0	0	1	0	0	0
ENSMUSG00000098678	MROH6	0	0	0	0	0	0	0	1	0	0	0	0
ENSMUSG00000098679	MIR7688	0	0	0	0	0	0	0	0	0	0	0	1
ENSMUSG00000098692	GM28041	0	0	0	0	0	0	1	0	2	0	0	0
ENSMUSG00000098702	1500015A07F	0	3	0	2	4	1	6	5	6	8	5	9
ENSMUSG00000098708	GM27252	0	0	0	0	0	0	1	2	0	0	1	0
ENSMUSG00000098711	GM28030	0	0	0	0	0	0	1	0	0	0	0	1
ENSMUSG00000098713	RPS2-PS5	0	0	1	0	0	0	0	0	0	0	0	0
ENSMUSG00000098715	GM28053	0	0	0	0	0	0	1	1	0	0	0	0
ENSMUSG00000098789	JMJD7	0	0	1	0	0	1	0	2	3	1	0	1
ENSMUSG00000098803	GM27468	0	0	0	0	0	0	0	0	0	1	0	0
ENSMUSG00000098837	GM20111	0	0	0	0	0	0	0	0	0	2	0	0
ENSMUSG00000098857	GM27395	0	0	0	1	0	0	0	0	0	0	0	1
ENSMUSG00000098859	GM27389	0	0	0	0	0	0	2	0	0	0	0	0
ENSMUSG00000098874	GM27960	0	0	0	0	1	0	0	0	0	0	0	0
ENSMUSG00000098886	MIR6385	0	0	0	0	0	0	0	1	1	0	0	1
ENSMUSG00000098889	GM27206	0	0	0	0	0	0	1	0	1	0	0	0
ENSMUSG00000098893	GM17828	0	0	0	0	0	0	0	0	0	1	0	1
ENSMUSG00000098905	ZFP953	1	3	1	2	1	0	7	5	3	7	3	3
ENSMUSG00000098912	1500004A13F	0	1	1	0	0	0	3	0	0	0	0	1
ENSMUSG00000098914	GM27747	0	0	0	0	1	1	1	1	1	0	0	1
ENSMUSG00000098915	RPL15-PS2	0	0	0	1	0	0	0	0	1	1	0	1
ENSMUSG00000098967	GM27845	1	0	0	0	0	0	3	3	2	3	0	1
ENSMUSG00000098973	MIR6236	9	7	7	10	14	10	32	35	26	36	22	38
ENSMUSG00000098977	GM27196	0	0	1	0	0	0	2	7	3	3	5	1
ENSMUSG00000098985	GM27219	0	0	0	0	0	0	4	2	1	4	3	3
ENSMUSG00000099021	RN7S1	0	0	0	0	0	0	0	0	0	0	1	0
ENSMUSG00000099024	GM27457	0	0	0	0	0	0	1	0	0	0	0	0
ENSMUSG00000099033	MIR7013	0	0	0	0	0	0	1	0	0	0	0	0
ENSMUSG00000099061	GM28050	0	0	0	0	0	0	1	0	0	0	0	0
ENSMUSG00000099083	ATF7	2	1	2	3	3	0	6	17	10	15	14	12
ENSMUSG00000099094	GM19569	1	0	0	0	0	1	0	1	1	5	0	1
ENSMUSG00000099098	1110035H17F	0	2	1	0	0	0	1	1	1	1	1	3
ENSMUSG00000099105	MIR7007	0	0	0	0	0	0	1	1	0	0	0	1
ENSMUSG00000099107	GM27898	0	0	0	0	0	0	1	1	0	0	0	0
ENSMUSG00000099133	GM27605	0	0	0	0	0	0	1	0	0	0	0	2
ENSMUSG00000099137	GM10603	0	1	0	0	0	0	2	1	0	2	1	0
ENSMUSG00000099139	1110028F18F	0	0	0	0	0	0	0	0	0	1	0	0

ENSMUSG00000099151	GM27803	5	4	3	7	4	4	7	5	7	15	9	6
ENSMUSG00000099310	GM19427	1	1	2	3	1	1	7	3	4	6	2	1
ENSMUSG00000099318	GM27735	0	0	0	0	0	0	0	0	0	1	0	0
ENSMUSG00000099330	MIR7053	1	0	0	0	0	0	0	0	0	0	0	0
ENSMUSG00000099337	GM29486	0	0	0	3	0	0	2	3	2	0	1	0
ENSMUSG00000099354	1700124L16R	0	0	0	0	0	0	1	2	1	1	0	3
ENSMUSG00000099373	GM19667	0	0	0	0	0	0	0	0	0	0	0	1
ENSMUSG00000099375	GM28187	8	11	8	15	8	9	64	42	39	92	36	31
ENSMUSG00000099391	1700003C15R	0	0	0	0	1	0	0	0	0	0	0	0
ENSMUSG00000099398	MS4A14	0	0	0	0	0	0	1	0	0	0	0	0
ENSMUSG00000099401	GM28375	0	0	1	0	0	0	0	0	0	0	0	0
ENSMUSG00000099419	1700001D01R	0	0	0	0	0	0	0	0	0	1	0	0
ENSMUSG00000099422	GM4275	0	1	0	0	0	0	0	1	0	0	0	0
ENSMUSG00000099423	GM17826	0	1	0	0	0	0	0	0	0	0	0	0
ENSMUSG00000099459	GM28556	0	0	0	0	0	0	0	2	0	0	0	0
ENSMUSG00000099465	GM3830	1	1	0	0	2	0	1	2	1	1	1	2
ENSMUSG00000099471	GM8451	0	1	0	3	3	1	1	3	3	4	3	0
ENSMUSG00000099472	GM29539	0	0	0	0	0	0	0	0	0	2	0	0
ENSMUSG00000099474	1700097N02R	0	0	0	0	0	0	1	2	0	0	0	0
ENSMUSG00000099478	GM28370	0	0	0	0	0	0	1	1	0	1	0	0
ENSMUSG00000099481	XNDC1	4	11	5	6	7	5	30	21	17	30	19	16
ENSMUSG00000099492	GM5525	0	0	0	0	0	0	0	0	1	0	0	0
ENSMUSG00000099517	HIST1H3G	0	2	0	0	0	0	3	5	1	1	2	1
ENSMUSG00000099543	GM29322	0	0	0	0	0	0	1	0	0	0	2	0
ENSMUSG00000099583	HIST1H3D	0	2	0	1	1	1	4	5	6	9	9	5
ENSMUSG00000099608	4933411E06R	0	0	0	1	0	0	0	0	0	0	0	0
ENSMUSG00000099625	GM29325	0	0	0	0	0	0	0	0	1	0	1	0
ENSMUSG00000099632	2900093K20R	1	2	0	0	0	0	2	3	1	1	2	2
ENSMUSG00000099647	GM5776	0	0	0	0	0	0	0	0	0	0	0	1
ENSMUSG00000099649	GM28406	0	0	0	0	0	0	0	0	0	1	0	0
ENSMUSG00000099655	2310034G01R	0	0	1	0	0	0	0	0	0	0	0	0
ENSMUSG00000099681	1700052K11R	0	0	0	2	2	1	6	1	3	7	1	7
ENSMUSG00000099686	GM29460	0	0	0	0	2	0	7	3	3	2	2	2
ENSMUSG00000099689	ZFP383	0	0	0	0	0	0	2	1	5	3	3	4
ENSMUSG00000099702	GM29013	0	0	0	0	0	0	0	0	0	0	1	0
ENSMUSG00000099706	GM29536	0	0	0	0	0	0	0	0	0	0	1	0
ENSMUSG00000099708	GM29361	0	0	1	2	1	0	3	4	0	1	3	3
ENSMUSG00000099752	GM29018	0	0	0	0	0	0	0	0	0	0	1	0
ENSMUSG00000099757	BE692007	0	1	0	0	0	0	1	0	0	2	0	0
ENSMUSG00000099759	1700030C10R	0	0	0	0	1	0	4	4	1	0	6	0
ENSMUSG00000099760	GM28800	0	0	0	0	0	0	1	0	1	0	0	0
ENSMUSG00000099764	RPS10-PS2	32	37	37	31	41	42	99	106	95	107	73	90
ENSMUSG00000099773	HMGB1-RS16	0	0	0	0	1	0	0	0	0	0	0	0
ENSMUSG00000099794	1700128A07R	1	0	0	0	0	0	0	0	0	0	0	0
ENSMUSG00000099801	GM29011	0	0	0	0	1	0	0	0	0	0	0	1
ENSMUSG00000099804	GM28497	0	0	0	0	0	1	0	0	0	0	0	0
ENSMUSG00000099825	1810012K16R	0	0	0	0	0	0	0	0	0	1	1	0
ENSMUSG00000099835	GM29668	0	0	0	0	0	0	0	0	0	1	0	0
ENSMUSG00000099842	GM4034	0	0	0	0	0	0	0	1	0	0	0	0
ENSMUSG00000099843	GM7160	0	0	0	0	0	2	2	0	3	4	3	3
ENSMUSG00000099851	GM29542	2	1	2	3	0	0	7	6	4	6	3	5
ENSMUSG00000099853	GM29328	1	0	0	0	0	0	1	1	0	0	0	0
ENSMUSG00000099858	GM6652	0	0	0	0	0	0	1	0	0	0	0	0
ENSMUSG00000099875	RBM3-PS	0	0	0	0	0	0	1	1	0	0	0	0
ENSMUSG00000099878	GM28178	0	0	0	0	0	0	1	1	0	0	0	0
ENSMUSG00000099881	2810013P06R	7	5	9	2	9	6	41	22	25	45	23	22
ENSMUSG00000099891	GM5575	0	0	0	0	0	0	1	0	2	1	0	1
ENSMUSG00000099902	GM12115	0	0	1	0	0	0	0	0	0	0	0	0

ENSMUSG00000099913	GM28551	1	3	1	0	1	0	1	2	1	1	3	3
ENSMUSG00000099923	1700105P06F	0	0	0	0	0	0	0	0	0	1	0	0
ENSMUSG00000099924	GM28320	1	2	3	2	2	2	6	2	4	2	4	2
ENSMUSG00000099925	GM28827	0	0	0	0	0	0	0	0	0	0	0	1
ENSMUSG00000099931	GM29358	1	0	0	0	1	0	1	1	0	2	0	1
ENSMUSG00000099949	GM29124	0	0	0	1	0	0	0	0	0	0	0	0
ENSMUSG00000099954	GM28112	2	2	2	7	3	2	11	9	6	11	3	5
ENSMUSG00000099959	GM29487	0	0	0	0	0	0	0	1	0	0	0	0
ENSMUSG00000099966	2810402E24F	4	4	2	6	3	5	11	15	7	10	6	8
ENSMUSG00000099997	TUBB4B-PS2	0	1	0	0	0	0	0	0	0	0	1	0
ENSMUSG00000100002	GM29642	0	0	0	0	0	0	0	0	0	1	0	0
ENSMUSG00000100005	B130024G19I	0	0	0	1	0	0	0	0	0	0	0	0
ENSMUSG00000100009	GM7967	7	9	8	14	4	11	49	58	41	42	49	43
ENSMUSG00000100017	2410022M11	0	0	0	0	0	0	2	4	0	5	2	0
ENSMUSG00000100018	GM7776	0	0	1	0	0	0	0	0	1	0	0	0
ENSMUSG00000100033	GM8337	0	0	0	0	0	0	0	1	0	0	0	1
ENSMUSG00000100039	GM28959	0	0	0	0	0	0	3	2	1	7	2	1
ENSMUSG00000100071	GM28707	0	0	1	1	0	0	2	0	0	1	3	0
ENSMUSG00000100075	1700018L02F	0	1	0	1	1	0	0	0	1	0	0	0
ENSMUSG00000100078	GM6170	1	0	0	0	0	0	0	0	1	1	1	0
ENSMUSG00000100079	IFNAB	0	0	0	0	0	0	0	0	1	0	0	0
ENSMUSG00000100084	2310068J16R	0	0	0	0	0	0	0	0	2	1	0	1
ENSMUSG00000100113	GM28592	0	1	0	0	1	0	0	0	0	1	1	2
ENSMUSG00000100130	GM28379	1	0	0	1	0	0	0	1	1	0	0	0
ENSMUSG00000100150	GM19585	5	1	0	1	4	7	10	11	11	12	3	11
ENSMUSG00000100153	GM5601	3	2	5	2	3	6	9	10	9	10	6	15
ENSMUSG00000100160	GM28527	0	0	0	0	0	0	0	0	0	2	0	0
ENSMUSG00000100164	2610306M01	0	1	0	0	1	0	6	4	5	3	3	3
ENSMUSG00000100182	1810006J02R	0	0	0	0	0	0	0	0	0	0	0	1
ENSMUSG00000100188	GM29561	0	0	0	0	0	0	0	0	0	1	0	0
ENSMUSG00000100199	GM20324	0	0	1	1	0	1	0	1	2	2	1	3
ENSMUSG00000100201	GM29093	0	0	0	0	0	0	1	2	0	1	0	0
ENSMUSG00000100204	GM4849	0	0	1	0	0	0	0	0	0	0	0	0
ENSMUSG00000100210	HIST1H3F	1	3	2	0	1	1	0	1	4	1	1	1
ENSMUSG00000100211	1700064M15	0	0	0	0	0	0	0	1	0	0	1	0
ENSMUSG00000100213	GM28151	1	0	0	0	0	0	1	1	0	0	0	0
ENSMUSG00000100215	GM8292	0	1	0	0	1	0	1	3	2	0	2	0
ENSMUSG00000100228	GM29257	1	0	0	1	1	1	7	3	4	6	0	1
ENSMUSG00000100235	GM28557	0	0	0	0	0	0	1	0	0	0	2	1
ENSMUSG00000100255	GM28196	0	1	0	0	0	0	0	1	1	0	0	1
ENSMUSG00000100280	GM28417	2	0	2	1	1	2	5	7	6	2	4	7
ENSMUSG00000100281	4930422M22	0	0	0	0	0	0	2	0	0	0	0	1
ENSMUSG00000100302	GM29670	0	0	0	0	0	0	0	1	0	1	0	0
ENSMUSG00000100313	GM28323	0	0	0	0	0	0	0	0	0	0	1	0
ENSMUSG00000100315	1700031P21F	0	0	0	0	0	0	0	1	0	0	0	0
ENSMUSG00000100320	GM29417	0	0	0	0	0	0	2	2	1	2	1	2
ENSMUSG00000100349	GM28188	0	0	0	0	0	2	4	2	5	3	4	5
ENSMUSG00000100351	GM7867	0	0	0	1	0	0	1	1	0	1	0	0
ENSMUSG00000100354	GM29113	0	0	0	1	0	0	0	0	0	1	0	0
ENSMUSG00000100392	GM28935	0	0	0	0	0	0	0	0	0	0	0	1
ENSMUSG00000100394	GM28791	3	6	3	6	1	0	4	5	10	6	8	6
ENSMUSG00000100396	GM29367	0	0	0	0	0	0	0	1	0	1	0	0
ENSMUSG00000100426	GM4208	3	3	5	6	1	3	26	23	22	24	17	17
ENSMUSG00000100450	GM9443	1	0	0	0	0	0	0	0	0	0	0	0
ENSMUSG00000100455	GM29170	1	0	3	2	0	2	6	11	5	9	6	2
ENSMUSG00000100480	GM29156	0	0	0	0	0	0	0	1	0	0	0	0
ENSMUSG00000100512	OVOL3	0	0	0	0	0	0	1	0	0	0	0	0
ENSMUSG00000100534	GM816	1	0	0	0	1	0	0	0	0	0	0	0

ENSMUSG00000100548	GM29585	0	0	0	0	1	0	0	0	1	0	0	0
ENSMUSG00000100551	GM19503	0	0	0	0	0	0	1	0	0	1	0	0
ENSMUSG00000100561	GM7516	0	0	0	0	0	0	0	1	0	0	0	0
ENSMUSG00000100590	GM4785	0	0	0	0	0	1	0	0	0	0	0	0
ENSMUSG00000100594	2810414N06I	0	0	0	0	0	0	2	1	1	0	2	0
ENSMUSG00000100595	GM19087	0	0	0	0	0	2	0	1	0	0	0	0
ENSMUSG00000100599	1700120C14F	0	0	1	5	1	1	2	2	1	4	3	2
ENSMUSG00000100605	GM29243	0	1	2	0	1	1	5	0	1	2	2	3
ENSMUSG00000100615	GM5511	0	0	0	0	0	0	0	0	0	0	1	0
ENSMUSG00000100622	GM20379	0	0	1	0	2	0	1	1	2	0	1	0
ENSMUSG00000100629	GM28192	0	0	0	0	0	0	0	1	0	0	0	0
ENSMUSG00000100632	9430060I03R	0	0	0	0	0	0	0	0	1	0	2	0
ENSMUSG00000100648	GM28659	0	0	0	0	0	0	0	0	0	0	0	1
ENSMUSG00000100652	GM7634	0	0	0	0	0	0	0	0	0	0	0	1
ENSMUSG00000100672	GM28404	0	0	0	0	0	0	0	0	0	1	0	0
ENSMUSG00000100680	1810044D09I	0	1	1	1	1	0	7	2	1	4	3	3
ENSMUSG00000100691	2010320M18	0	1	0	1	0	1	2	1	4	6	2	1
ENSMUSG00000100714	2010308F09F	0	0	0	0	0	0	2	0	0	0	1	0
ENSMUSG00000100720	GM10075	0	0	0	0	0	0	0	0	1	0	0	0
ENSMUSG00000100736	GM5147	0	0	0	0	0	0	0	1	0	1	0	0
ENSMUSG00000100747	1700084E18F	0	0	2	0	0	0	0	0	0	0	0	0
ENSMUSG00000100750	GM29084	1	0	0	0	0	0	0	0	0	0	0	0
ENSMUSG00000100755	RPS23-PS1	0	0	1	1	1	1	1	4	3	4	2	1
ENSMUSG00000100763	GM28777	0	0	0	0	0	0	1	0	1	0	0	2
ENSMUSG00000100764	GM29155	0	0	0	0	0	0	1	0	1	0	0	0
ENSMUSG00000100779	GM29292	0	0	0	0	0	0	0	0	0	0	0	2
ENSMUSG00000100801	GM15459	0	0	0	0	0	0	2	0	0	0	1	0
ENSMUSG00000100807	GM29521	2	2	2	6	4	1	6	8	6	7	8	4
ENSMUSG00000100813	GM28874	0	1	0	0	0	0	1	0	2	1	1	1
ENSMUSG00000100815	GM29112	0	0	0	0	0	0	0	0	1	0	0	0
ENSMUSG00000100826	SNHG14	3	9	4	7	5	8	31	22	32	30	24	9
ENSMUSG00000100830	GM28441	0	0	0	0	0	0	0	1	0	0	0	0
ENSMUSG00000100833	GM28988	0	0	0	0	0	0	0	0	0	1	1	0
ENSMUSG00000100862	GM10925	1	1	2	1	0	0	11	7	8	9	7	11
ENSMUSG00000100865	GM9320	34	51	39	45	43	51	161	170	125	171	104	114
ENSMUSG00000100876	2810454H06I	0	1	0	2	0	0	5	2	4	6	0	2
ENSMUSG00000100894	GM8304	0	0	0	0	0	0	0	0	0	0	1	0
ENSMUSG00000100901	GM18829	0	0	0	0	0	0	1	1	0	4	0	0
ENSMUSG00000100916	LHB	0	0	0	0	0	0	0	0	2	1	1	1
ENSMUSG00000100922	GM8520	0	0	0	0	0	0	0	1	0	0	0	1
ENSMUSG00000100927	GM28536	0	0	0	0	0	0	1	0	0	0	0	0
ENSMUSG00000100937	1700020D05I	0	1	0	0	0	0	0	1	0	1	0	0
ENSMUSG00000100954	GM10138	1	2	2	0	2	3	7	7	6	5	3	4
ENSMUSG00000100967	GM29666	1	0	0	2	1	1	1	1	1	1	0	1
ENSMUSG00000100975	GM28875	0	1	0	1	0	1	0	0	0	0	0	1
ENSMUSG00000100985	GM10640	0	0	0	0	0	0	0	0	0	1	0	0
ENSMUSG00000101013	A630072M18	1	3	0	13	8	3	11	13	6	14	4	10
ENSMUSG00000101037	GM28424	0	0	0	0	0	0	0	0	0	0	1	0
ENSMUSG00000101111	GM28437	0	0	0	0	0	2	6	0	6	4	3	2
ENSMUSG00000101122	GM17971	0	0	0	0	0	0	1	2	0	1	0	0
ENSMUSG00000101156	GM29114	0	0	0	0	0	0	0	0	0	1	0	0
ENSMUSG00000101172	GM28535	0	1	2	0	0	2	0	0	0	1	0	2
ENSMUSG00000101174	HOXD4	2	2	3	6	1	3	6	7	6	10	6	10
ENSMUSG00000101191	GM28809	0	0	0	0	0	0	0	0	0	0	1	0
ENSMUSG00000101223	4930568A12F	0	0	0	0	0	0	0	0	0	0	0	1
ENSMUSG00000101225	1700008J07R	1	0	0	0	0	0	1	3	3	1	3	1
ENSMUSG00000101229	GM29046	0	0	0	0	0	0	0	1	0	0	0	0
ENSMUSG00000101236	GM6750	0	0	0	0	0	0	0	0	1	0	0	0

ENSMUSG00000101242	GM28319	0	0	0	0	0	0	0	0	0	1	1	1
ENSMUSG00000101249	GM29216	0	0	0	0	3	0	4	3	7	5	3	2
ENSMUSG00000101262	GM8326	0	0	0	0	0	0	1	1	1	1	0	0
ENSMUSG00000101281	GM8449	0	1	0	0	0	0	0	0	0	0	0	0
ENSMUSG00000101308	GM28989	0	0	1	0	1	0	0	1	0	1	0	0
ENSMUSG00000101328	RPL30-PS6	0	0	0	0	0	0	3	1	4	1	1	0
ENSMUSG00000101330	GM10193	0	0	0	0	0	0	0	0	1	0	0	0
ENSMUSG00000101335	GM28229	0	2	0	1	0	3	0	4	2	9	3	5
ENSMUSG00000101355	HIST1H3H	0	0	0	0	0	0	0	0	1	0	0	0
ENSMUSG00000101360	4933417O13I	0	0	0	0	0	0	0	0	0	2	1	0
ENSMUSG00000101363	GM29490	1	0	0	0	0	0	0	0	0	0	0	0
ENSMUSG00000101365	GM19325	0	0	0	0	0	0	3	2	4	2	4	2
ENSMUSG00000101398	GM2617	0	0	0	0	0	1	0	0	0	0	0	0
ENSMUSG00000101431	GM7901	0	1	0	0	0	0	0	0	0	0	0	0
ENSMUSG00000101438	GM19412	1	1	0	0	2	0	1	2	2	5	2	4
ENSMUSG00000101450	GM28941	0	0	0	0	0	0	0	0	0	0	0	2
ENSMUSG00000101452	GM28530	0	0	0	0	0	0	2	2	1	0	0	0
ENSMUSG00000101456	GM29043	0	0	0	0	0	0	0	0	0	1	0	0
ENSMUSG00000101464	GM7436	0	0	0	0	0	0	0	0	1	0	0	0
ENSMUSG00000101483	1700016L21R	0	0	0	0	0	0	1	5	1	1	2	2
ENSMUSG00000101493	2810405F17R	0	0	0	0	0	0	0	0	0	2	0	1
ENSMUSG00000101496	GM8885	0	0	0	0	0	0	1	1	0	0	0	1
ENSMUSG00000101501	RPS27A-PS1	0	1	0	0	0	0	1	0	0	0	0	2
ENSMUSG00000101502	RPL7A-PS10	0	0	1	0	0	0	0	0	0	0	0	0
ENSMUSG00000101503	GM28364	0	0	0	0	0	0	1	0	1	0	0	1
ENSMUSG00000101517	4732465J04R	0	2	0	0	0	0	0	0	1	2	1	1
ENSMUSG00000101523	GM10031	2	1	3	0	1	2	2	2	2	7	1	3
ENSMUSG00000101583	GM20753	0	0	0	0	0	0	0	0	0	0	0	1
ENSMUSG00000101587	GM29036	0	0	0	0	0	0	0	1	0	0	1	0
ENSMUSG00000101588	GM28265	0	0	0	0	0	0	0	0	0	0	1	0
ENSMUSG00000101589	RBM6-PS1	1	0	0	0	0	0	0	2	0	0	1	1
ENSMUSG00000101595	GM29395	0	0	0	0	0	0	0	0	0	1	0	0
ENSMUSG00000101597	GM5621	0	1	1	0	0	0	0	0	0	0	1	0
ENSMUSG00000101599	GM20342	4	11	8	12	5	8	26	21	26	35	16	24
ENSMUSG00000101603	GM28730	0	0	0	0	0	0	0	1	0	0	0	0
ENSMUSG00000101609	KCNQ1OT1	82	123	74	152	103	139	345	367	239	455	224	246
ENSMUSG00000101612	GM7933	0	0	0	0	0	0	0	0	1	0	0	0
ENSMUSG00000101641	GM29560	0	1	1	1	0	0	0	2	1	2	0	1
ENSMUSG00000101652	GM6060	0	0	0	0	0	0	0	0	0	0	1	0
ENSMUSG00000101656	GM8004	0	0	0	0	0	0	0	1	0	0	0	0
ENSMUSG00000101674	4930444A19R	0	0	0	0	0	0	0	1	0	0	1	0
ENSMUSG00000101678	GM29609	0	0	0	0	1	0	0	1	0	0	0	0
ENSMUSG00000101682	GM29228	0	0	0	0	0	1	0	1	0	1	1	0
ENSMUSG00000101685	GM17837	0	0	0	0	0	0	0	1	0	0	0	0
ENSMUSG00000101695	1700094J05R	0	0	0	1	0	0	2	0	0	1	1	1
ENSMUSG00000101730	GM28580	0	0	0	0	0	0	0	0	0	0	0	1
ENSMUSG00000101731	3830432H09R	0	0	0	0	0	0	0	0	0	1	0	0
ENSMUSG00000101746	2310043L19R	0	1	0	0	0	0	1	0	0	0	0	0
ENSMUSG00000101778	GM29488	0	0	0	1	0	0	1	1	1	1	0	1
ENSMUSG00000101795	GM5835	0	0	1	2	0	0	2	1	0	1	1	1
ENSMUSG00000101800	RNF170-PS	0	0	0	0	0	0	0	1	0	0	0	0
ENSMUSG00000101823	GM29438	0	1	0	1	0	0	3	0	1	0	2	0
ENSMUSG00000101832	GM28982	0	0	0	0	0	0	0	1	1	0	1	0
ENSMUSG00000101834	GM5829	0	0	0	0	0	0	0	0	1	0	0	0
ENSMUSG00000101845	GM28198	0	0	0	0	0	0	0	0	0	1	0	0
ENSMUSG00000101856	1700096K18R	0	2	0	0	0	3	4	8	1	3	1	1
ENSMUSG00000101867	C78334	0	0	0	0	0	0	0	0	0	0	0	1
ENSMUSG00000101875	GM6028	0	0	0	0	0	1	1	0	0	0	1	2

ENSMUSG00000101892	9130401M01	2	2	0	3	0	1	6	15	6	16	5	8
ENSMUSG00000101932	GM10074	0	0	0	0	0	0	0	0	0	0	1	0
ENSMUSG00000101938	GM29605	1	0	0	0	0	0	0	0	0	0	0	0
ENSMUSG00000101939	GM28438	0	4	1	1	1	0	4	8	8	10	4	5
ENSMUSG00000101958	GM19085	0	0	0	1	0	0	0	0	0	0	0	0
ENSMUSG00000101959	LDHAL6B	0	0	0	0	0	0	0	0	0	0	0	1
ENSMUSG00000101964	GM28693	1	0	0	1	0	0	3	0	0	0	0	1
ENSMUSG00000101969	GM20125	0	0	0	0	0	0	0	1	1	0	0	1
ENSMUSG00000101970	1810026B05F	4	11	5	13	14	7	48	57	51	61	43	48
ENSMUSG00000101972	HIST1H3I	0	0	0	0	0	1	1	0	0	0	0	0
ENSMUSG00000102014	2900009J06R	0	0	0	0	0	0	0	1	0	0	0	1
ENSMUSG00000102037	BCL2A1A	1	0	0	0	0	0	0	0	0	0	0	0
ENSMUSG00000102048	GM6075	0	0	0	0	0	0	0	0	0	0	1	1
ENSMUSG00000102059	GM20257	0	0	0	0	0	0	2	1	0	2	0	3
ENSMUSG00000102064	GM28625	0	0	0	0	0	0	0	0	0	0	1	0
ENSMUSG00000102069	1700012I11R	0	0	0	0	0	0	2	1	1	0	0	0
ENSMUSG00000102070	GM28661	1	0	0	0	0	1	4	3	0	2	5	0
ENSMUSG00000102080	GM5092	0	0	0	0	0	0	0	0	0	0	2	0
ENSMUSG00000102101	ZBTB11OS1	0	0	0	0	1	1	2	1	2	2	3	4
ENSMUSG00000102112	1810041H14F	0	0	0	0	0	0	0	2	2	2	2	0
ENSMUSG00000102117	RPSA-PS1	0	0	1	2	1	2	1	1	9	7	4	4
ENSMUSG00000102135	GM37108	0	0	0	0	1	3	0	2	3	1	2	2
ENSMUSG00000102142	GM26930	0	0	1	1	1	0	2	1	2	4	0	4
ENSMUSG00000102143	GM38057	0	0	0	0	1	0	0	0	0	0	0	0
ENSMUSG00000102145	GM38056	0	1	0	0	0	0	0	0	1	0	0	1
ENSMUSG00000102151	GM37472	0	2	0	2	0	1	0	0	4	2	3	1
ENSMUSG00000102152	GM37475	0	0	0	0	0	0	1	0	0	0	0	0
ENSMUSG00000102153	GM37474	4	2	4	1	2	5	4	9	9	8	3	6
ENSMUSG00000102154	GM37469	0	0	0	0	0	0	0	1	0	0	0	1
ENSMUSG00000102157	GM37470	0	0	0	0	1	0	0	0	1	0	0	0
ENSMUSG00000102172	GM37873	0	0	0	0	1	0	0	0	0	0	0	0
ENSMUSG00000102175	GM6119	0	0	0	0	1	0	1	0	1	1	1	0
ENSMUSG00000102179	GM37868	0	0	0	0	0	2	0	0	0	0	0	1
ENSMUSG00000102189	GM37194	1	0	0	0	0	0	0	0	0	0	0	0
ENSMUSG00000102193	GM7299	0	0	0	1	1	0	1	0	3	2	1	1
ENSMUSG00000102201	GM36956	0	0	0	0	0	0	0	0	0	0	0	1
ENSMUSG00000102202	GM33280	0	0	0	1	0	1	0	2	0	0	0	0
ENSMUSG00000102207	GM10344	0	0	0	0	0	0	1	0	0	0	0	0
ENSMUSG00000102211	GM37490	1	0	0	0	0	0	1	1	4	1	1	2
ENSMUSG00000102212	C230085N15I	3	2	1	2	2	3	11	8	4	9	4	7
ENSMUSG00000102218	GM37493	0	0	0	0	0	0	0	1	0	0	0	0
ENSMUSG00000102219	GM6606	0	0	0	0	0	0	1	0	1	0	0	1
ENSMUSG00000102220	RPL23A-PS5	0	0	1	0	0	0	0	0	3	1	0	2
ENSMUSG00000102224	4930447F24F	0	0	0	0	0	0	0	0	0	0	1	2
ENSMUSG00000102225	GM37983	0	0	0	0	0	0	0	0	0	2	0	1
ENSMUSG00000102241	GM37716	0	2	0	0	0	1	4	2	5	5	1	1
ENSMUSG00000102243	GM37718	0	0	0	1	2	0	1	1	1	2	3	1
ENSMUSG00000102246	9430037O13I	3	1	1	0	2	8	8	8	9	8	6	4
ENSMUSG00000102252	SNRPN	0	1	1	0	1	0	4	1	1	1	0	2
ENSMUSG00000102258	GM38257	0	0	0	0	0	0	0	1	0	0	0	0
ENSMUSG00000102263	GM31415	0	0	0	1	0	0	0	0	0	0	0	0
ENSMUSG00000102275	GM37144	1	1	4	3	0	0	2	5	1	3	0	6
ENSMUSG00000102277	A130050O07I	2	9	1	2	2	2	3	2	1	6	6	2
ENSMUSG00000102278	GM37145	0	0	0	1	0	1	1	1	2	2	0	0
ENSMUSG00000102297	5730585A16F	0	0	0	0	0	0	0	0	0	1	0	0
ENSMUSG00000102302	GM38190	0	0	0	1	0	0	3	0	0	4	1	3
ENSMUSG00000102305	GM38192	0	0	0	0	0	0	2	1	0	0	1	0
ENSMUSG00000102307	GM38194	1	0	0	0	0	0	0	0	0	0	0	1

ENSMUSG00000102324	GM19721	0	0	0	0	0	0	0	0	0	1	0	0
ENSMUSG00000102332	GM19331	0	0	0	2	1	0	1	1	1	2	1	0
ENSMUSG00000102349	GM37376	10	8	10	24	14	15	66	53	40	82	46	50
ENSMUSG00000102352	GM38346	0	0	1	0	1	0	0	2	1	3	0	0
ENSMUSG00000102363	GM32999	0	0	0	0	1	0	0	1	0	0	0	1
ENSMUSG00000102365	GM37045	0	1	0	1	0	0	0	0	0	0	0	1
ENSMUSG00000102370	GM37973	0	0	0	0	0	1	0	1	0	0	0	0
ENSMUSG00000102374	GM38387	0	0	0	0	0	0	2	0	0	1	1	0
ENSMUSG00000102376	GM37975	0	0	0	0	0	0	0	1	1	1	1	0
ENSMUSG00000102383	GM38142	0	0	0	0	0	0	0	0	1	0	0	0
ENSMUSG00000102385	GM38144	0	1	0	0	0	0	0	0	0	0	0	0
ENSMUSG00000102389	D630023O14	0	0	1	0	0	0	0	0	0	1	0	0
ENSMUSG00000102401	GM37864	0	0	0	0	0	1	0	1	0	0	0	0
ENSMUSG00000102411	GM36936	0	0	0	1	1	1	0	1	1	2	2	3
ENSMUSG00000102415	A430110C17F	0	0	1	1	0	0	0	1	1	5	0	0
ENSMUSG00000102416	4933424G06I	0	0	0	0	0	0	1	0	0	0	0	0
ENSMUSG00000102418	SH2D1B1	0	0	0	0	0	1	0	0	2	0	0	0
ENSMUSG00000102419	GM36940	0	0	0	0	0	0	0	0	1	0	1	0
ENSMUSG00000102425	GM26616	0	0	0	0	0	0	0	0	0	2	2	0
ENSMUSG00000102428	PCDHGA12	1	1	0	0	0	2	1	0	0	1	0	0
ENSMUSG00000102436	GM38047	0	0	0	0	0	0	0	0	0	0	1	0
ENSMUSG00000102441	GM38032	0	0	0	0	0	0	0	1	0	0	0	0
ENSMUSG00000102448	GM37101	0	0	0	1	0	0	1	0	3	0	1	1
ENSMUSG00000102451	GM37655	0	0	0	0	0	0	0	1	0	0	0	0
ENSMUSG00000102459	GM38352	0	0	0	0	1	0	0	0	0	0	0	0
ENSMUSG00000102466	GM38200	0	1	0	0	0	0	1	1	2	3	0	2
ENSMUSG00000102467	GM37167	0	0	0	0	0	1	0	0	0	0	0	0
ENSMUSG00000102474	2610012C04F	0	1	0	0	0	0	1	0	0	0	0	0
ENSMUSG00000102478	BC085271	0	1	1	0	0	1	0	1	0	0	0	3
ENSMUSG00000102480	GM37926	0	0	0	1	0	0	0	0	0	0	0	1
ENSMUSG00000102482	GM37924	13	10	18	9	13	6	53	64	46	70	56	44
ENSMUSG00000102494	GM36988	0	0	0	0	0	0	1	0	0	0	0	0
ENSMUSG00000102496	GM36989	0	3	1	0	0	0	4	1	2	1	2	0
ENSMUSG00000102501	GM37372	0	0	0	1	0	0	0	0	1	0	0	0
ENSMUSG00000102503	GM37370	0	0	0	0	0	0	3	1	1	1	1	3
ENSMUSG00000102505	1600017P15F	0	0	0	0	0	0	0	1	0	0	0	0
ENSMUSG00000102521	GM37781	0	0	0	0	0	1	0	0	0	0	0	0
ENSMUSG00000102540	GM38134	0	0	0	0	0	0	0	0	1	0	0	0
ENSMUSG00000102543	PCDHGC5	0	0	0	0	0	0	0	0	1	0	0	0
ENSMUSG00000102544	GM5103	0	0	0	0	0	1	0	1	0	2	0	0
ENSMUSG00000102545	6430573P05F	1	0	1	0	0	0	0	0	0	0	0	0
ENSMUSG00000102550	GM8276	0	0	1	0	0	1	0	0	0	0	0	1
ENSMUSG00000102553	D930036K23I	1	0	1	2	0	0	1	1	3	3	0	1
ENSMUSG00000102555	6430511E19F	0	1	3	0	1	2	7	7	7	7	6	7
ENSMUSG00000102556	GM37569	0	0	1	0	1	0	2	3	2	3	1	1
ENSMUSG00000102559	GM37570	1	2	1	1	0	0	2	3	1	7	1	0
ENSMUSG00000102562	GM37694	0	0	0	0	0	0	0	0	0	0	0	1
ENSMUSG00000102570	GM37968	0	2	0	0	0	2	2	0	1	3	0	2
ENSMUSG00000102571	GM37967	0	1	0	0	1	0	1	1	1	2	1	1
ENSMUSG00000102573	GM7265	0	0	0	0	2	0	0	1	1	1	1	1
ENSMUSG00000102579	GM37965	0	1	0	0	0	0	0	0	0	1	0	0
ENSMUSG00000102593	GM38384	0	1	0	0	0	0	2	2	0	1	1	1
ENSMUSG00000102600	GM37266	0	1	0	0	0	1	0	3	0	2	2	2
ENSMUSG00000102602	A930004J17R	2	2	0	2	4	2	7	6	6	4	1	3
ENSMUSG00000102606	RPL21-PS1	0	0	1	0	0	0	0	0	0	0	0	0
ENSMUSG00000102619	GM37849	0	0	0	0	0	0	0	0	0	1	0	0
ENSMUSG00000102630	GM37289	0	2	0	2	0	1	2	4	1	2	1	3
ENSMUSG00000102632	GM37786	0	0	0	0	0	0	0	2	0	2	0	1

ENSMUSG00000102633	GM38224	0	0	0	0	0	0	1	0	0	1	0	0
ENSMUSG00000102642	A130048G24I	0	7	0	2	1	1	8	14	12	9	7	11
ENSMUSG00000102644	THAP6	1	2	0	1	1	0	4	4	3	3	4	1
ENSMUSG00000102649	GM38021	0	0	0	0	0	0	2	1	0	1	0	1
ENSMUSG00000102651	GM37080	0	1	1	0	3	0	0	0	1	1	1	1
ENSMUSG00000102652	GM37078	0	0	0	0	0	0	0	1	0	0	0	0
ENSMUSG00000102664	GM38380	1	0	1	1	0	1	1	1	1	2	3	1
ENSMUSG00000102666	GM8599	0	0	2	0	2	0	1	6	0	1	0	0
ENSMUSG00000102670	GM37436	0	0	2	2	0	0	5	5	1	4	2	4
ENSMUSG00000102672	GM37105	1	0	1	1	0	2	8	10	3	11	3	3
ENSMUSG00000102683	GM37375	0	0	0	0	0	0	0	0	0	1	0	0
ENSMUSG00000102687	GM37219	0	0	0	0	0	0	3	1	3	3	0	0
ENSMUSG00000102691	GM37780	4	1	4	1	2	2	11	16	9	6	7	10
ENSMUSG00000102704	GM38309	0	0	0	0	0	0	4	1	0	2	1	2
ENSMUSG00000102705	4632432E15F	0	0	0	0	0	0	0	1	0	0	0	0
ENSMUSG00000102709	GM38308	0	0	0	0	0	0	1	0	0	0	0	0
ENSMUSG00000102712	GM37758	1	0	0	0	0	0	1	0	0	0	0	0
ENSMUSG00000102713	GM29994	0	0	0	0	0	0	0	3	1	3	0	0
ENSMUSG00000102714	GM37618	0	0	1	1	1	0	0	1	1	2	0	2
ENSMUSG00000102719	GM37760	0	3	4	3	3	4	16	9	9	13	7	5
ENSMUSG00000102721	A630081D01I	0	0	1	2	2	3	4	0	2	1	1	4
ENSMUSG00000102725	GM37939	0	0	0	0	0	0	0	0	0	1	0	1
ENSMUSG00000102733	GM38262	0	0	0	0	0	0	0	0	0	1	0	1
ENSMUSG00000102737	1700016A09F	0	0	1	0	0	0	0	0	0	0	0	0
ENSMUSG00000102744	5830444F18F	1	2	2	2	2	2	6	3	6	4	4	3
ENSMUSG00000102745	GM37600	0	0	0	0	0	0	0	0	0	1	0	0
ENSMUSG00000102752	GM7694	1	0	0	0	0	0	0	0	0	0	1	1
ENSMUSG00000102762	4930403P22F	1	0	0	0	0	0	1	0	1	2	1	1
ENSMUSG00000102764	GM37183	0	0	0	0	0	0	0	1	0	1	0	0
ENSMUSG00000102770	GM38163	0	1	0	0	0	0	0	0	0	0	0	1
ENSMUSG00000102776	GM38162	0	4	1	4	4	5	8	8	6	12	6	13
ENSMUSG00000102779	GM38164	0	0	0	0	0	0	0	0	0	0	0	1
ENSMUSG00000102780	GM38253	0	0	0	0	0	0	0	0	0	1	0	0
ENSMUSG00000102797	GM37710	0	1	1	0	0	0	1	0	2	3	0	0
ENSMUSG00000102801	GM37478	0	0	0	0	1	0	0	0	1	0	0	1
ENSMUSG00000102803	GM37477	0	0	0	1	0	0	0	0	0	0	0	0
ENSMUSG00000102809	GM8643	0	0	0	0	1	1	0	0	0	0	0	0
ENSMUSG00000102822	1700113B19F	0	0	0	0	0	0	0	0	0	0	1	0
ENSMUSG00000102824	PDCD5-PS	0	1	0	0	0	1	3	3	1	2	3	3
ENSMUSG00000102825	GM36990	0	0	0	0	0	0	0	0	0	0	0	1
ENSMUSG00000102827	GM8242	0	1	0	1	0	0	1	0	1	1	2	0
ENSMUSG00000102830	GM37012	0	0	0	0	0	0	0	0	1	0	0	0
ENSMUSG00000102833	GM9694	0	0	0	0	0	0	1	0	0	0	0	0
ENSMUSG00000102840	GM38037	0	0	2	1	0	1	1	2	0	0	1	1
ENSMUSG00000102844	GM38034	0	0	0	0	1	1	6	1	1	0	1	2
ENSMUSG00000102845	GM38033	0	0	0	0	0	0	0	0	0	1	0	0
ENSMUSG00000102850	GM37082	0	2	0	1	0	1	1	1	2	2	1	3
ENSMUSG00000102856	GM37084	0	1	0	0	0	0	1	1	1	0	0	0
ENSMUSG00000102861	GM37637	0	1	0	0	0	0	0	0	0	0	0	0
ENSMUSG00000102863	GM37639	0	0	0	0	0	0	3	1	2	4	0	1
ENSMUSG00000102864	GM37634	0	0	1	0	0	0	1	0	1	0	0	1
ENSMUSG00000102869	2900097C17F	0	2	2	4	3	4	9	11	8	12	7	18
ENSMUSG00000102874	GM37137	0	0	0	0	0	0	1	0	1	0	1	0
ENSMUSG00000102875	GM37139	0	1	0	0	1	0	1	0	1	0	0	1
ENSMUSG00000102899	GM37850	0	1	0	0	0	1	0	0	1	1	0	0
ENSMUSG00000102905	GM38266	0	0	0	0	0	0	0	0	0	0	1	0
ENSMUSG00000102912	GM20731	0	0	0	0	1	0	0	0	0	0	0	0
ENSMUSG00000102919	GM37726	0	0	0	0	0	0	1	0	0	0	0	0

ENSMUSG00000102927	GM37152	0	0	0	0	0	0	0	0	0	0	0	1
ENSMUSG00000102930	GM38115	1	0	0	0	1	0	3	4	2	0	3	0
ENSMUSG00000102935	GM38355	0	0	0	0	1	0	0	0	0	2	0	1
ENSMUSG00000102939	GM38111	0	0	0	0	0	0	0	1	0	0	0	0
ENSMUSG00000102941	GM37552	0	0	0	0	0	0	0	0	0	1	0	0
ENSMUSG00000102942	IGHV1-33	1	0	0	0	0	0	0	0	0	0	0	0
ENSMUSG00000102950	GM37018	0	0	0	0	0	1	0	0	0	0	0	0
ENSMUSG00000102953	GM37019	1	0	0	1	0	0	0	0	0	2	0	3
ENSMUSG00000102956	GM37017	1	1	0	1	0	2	1	2	1	0	1	1
ENSMUSG00000102960	GM37943	0	0	0	0	0	0	1	0	1	1	0	1
ENSMUSG00000102961	GM19918	0	0	1	0	0	0	2	2	3	0	0	1
ENSMUSG00000102964	9430034N14I	0	0	0	0	0	0	0	1	0	3	0	1
ENSMUSG00000102970	GM37349	0	0	0	0	0	0	0	0	0	0	1	1
ENSMUSG00000102972	GM37348	0	0	0	0	0	0	0	0	0	1	0	0
ENSMUSG00000102973	E430014B02F	1	4	2	2	4	2	6	4	4	8	1	3
ENSMUSG00000102976	ZC3H11A	1	0	0	0	1	1	0	1	4	3	1	1
ENSMUSG00000102982	GM38319	0	0	0	0	0	0	0	0	0	0	1	0
ENSMUSG00000102990	GM37764	0	0	0	0	0	0	0	0	0	1	0	0
ENSMUSG00000102992	GM37766	0	0	0	0	0	0	0	1	0	0	0	0
ENSMUSG00000103000	GM37900	0	0	0	0	0	0	0	2	0	0	0	0
ENSMUSG00000103006	4933417C20F	0	0	0	0	0	0	0	0	0	0	0	1
ENSMUSG00000103009	GM4430	1	2	1	0	1	0	1	1	0	0	1	2
ENSMUSG00000103011	GM8860	0	0	1	0	0	0	0	0	0	0	0	0
ENSMUSG00000103013	GM37503	0	0	1	1	0	0	1	1	0	0	1	0
ENSMUSG00000103032	GM37121	0	0	0	0	0	0	0	0	1	0	0	0
ENSMUSG00000103034	GM8797	0	0	0	0	0	0	2	2	1	3	5	2
ENSMUSG00000103038	GM37124	5	5	4	2	1	2	3	3	4	9	4	5
ENSMUSG00000103039	GM37123	0	0	0	0	0	0	0	0	0	0	0	2
ENSMUSG00000103041	GM37305	0	0	0	0	1	0	2	2	1	3	2	0
ENSMUSG00000103046	GM37309	0	0	0	0	0	0	1	1	0	1	0	0
ENSMUSG00000103062	GM37200	0	0	0	0	0	0	0	1	1	3	2	1
ENSMUSG00000103076	GM37902	0	1	1	0	0	0	0	1	0	0	2	1
ENSMUSG00000103085	GM38120	0	1	0	2	1	0	2	2	0	0	0	0
ENSMUSG00000103094	GM37558	0	0	1	0	0	0	4	4	1	0	2	3
ENSMUSG00000103103	4833445I07R	0	0	0	0	1	0	0	2	2	2	0	0
ENSMUSG00000103114	GM32200	0	0	0	0	0	0	2	0	0	1	0	1
ENSMUSG00000103115	GM37584	1	0	0	0	0	1	0	1	4	5	0	2
ENSMUSG00000103123	GM37390	1	0	1	3	1	1	2	5	3	1	1	2
ENSMUSG00000103129	GM37393	2	0	0	2	2	0	9	11	7	6	8	7
ENSMUSG00000103131	GM37977	0	0	0	0	0	0	0	0	1	0	0	0
ENSMUSG00000103132	GM37978	0	2	0	0	0	0	1	1	1	0	0	0
ENSMUSG00000103133	GM37303	1	0	1	0	0	0	0	0	0	1	0	1
ENSMUSG00000103138	GM2238	0	0	0	0	0	0	0	1	0	0	0	0
ENSMUSG00000103139	GM37982	0	0	0	0	0	0	0	0	0	0	0	1
ENSMUSG00000103143	GM37742	0	0	0	0	0	0	0	1	0	1	0	0
ENSMUSG00000103146	GM37745	0	0	0	0	0	0	0	2	0	0	0	0
ENSMUSG00000103149	AA914427	0	0	0	0	0	0	2	0	0	0	0	1
ENSMUSG00000103151	GM38292	0	0	0	0	0	0	1	0	0	0	0	2
ENSMUSG00000103158	GM31925	0	0	0	1	0	0	0	0	0	1	0	0
ENSMUSG00000103160	C130012C08F	0	0	0	0	0	0	1	0	0	0	0	0
ENSMUSG00000103162	GM38147	0	0	0	0	0	0	0	0	0	0	0	1
ENSMUSG00000103164	GM38150	0	0	0	0	0	1	0	0	0	2	0	0
ENSMUSG00000103168	GM30948	1	1	2	3	2	2	3	8	6	8	5	4
ENSMUSG00000103178	FGFR3-PS	0	0	0	0	0	0	1	0	1	0	0	0
ENSMUSG00000103181	A330069K06F	0	0	1	0	1	0	0	0	0	0	0	0
ENSMUSG00000103192	GM37645	0	0	0	0	2	0	0	0	0	1	0	0
ENSMUSG00000103197	GM37642	0	0	0	0	0	0	1	0	2	2	0	2
ENSMUSG00000103199	GM37648	0	0	0	0	0	0	0	1	2	0	0	0

ENSMUSG00000103200	GM37328	1	0	0	0	0	0	1	0	0	0	0	0
ENSMUSG00000103216	GM37248	10	9	4	5	10	6	28	15	15	19	13	16
ENSMUSG00000103220	GM37728	0	0	0	1	0	0	2	1	1	0	0	0
ENSMUSG00000103222	GM37729	0	0	0	0	1	0	2	1	0	0	0	0
ENSMUSG00000103227	GM37733	0	2	0	2	1	0	5	0	0	2	0	2
ENSMUSG00000103238	GM36527	0	2	0	1	0	0	2	3	1	2	1	1
ENSMUSG00000103251	GM37519	1	0	0	0	0	1	0	1	1	0	1	0
ENSMUSG00000103253	GM37520	0	1	0	1	1	0	3	6	4	1	1	1
ENSMUSG00000103260	GM8146	0	0	0	0	0	0	0	0	0	1	0	0
ENSMUSG00000103272	GM37914	1	1	1	1	1	0	4	2	0	4	1	2
ENSMUSG00000103273	GM37913	0	0	0	0	0	0	0	0	1	0	0	0
ENSMUSG00000103280	GM37277	0	1	0	0	0	0	0	1	0	1	0	0
ENSMUSG00000103285	GM37274	0	2	2	0	0	2	4	6	3	4	0	6
ENSMUSG00000103291	GM38235	0	0	0	0	0	0	1	1	0	0	0	1
ENSMUSG00000103294	GM38233	0	0	1	0	0	0	0	0	1	0	0	0
ENSMUSG00000103303	GM37802	0	0	1	0	0	0	0	2	0	0	0	0
ENSMUSG00000103313	GM38357	0	0	1	0	0	0	1	0	2	4	2	2
ENSMUSG00000103314	GM38358	0	0	0	0	0	0	0	0	1	0	0	0
ENSMUSG00000103341	1700051O22I	0	0	0	2	0	0	0	0	0	0	0	0
ENSMUSG00000103343	GM37052	0	0	0	0	0	1	1	4	5	1	2	0
ENSMUSG00000103348	GM37053	0	0	0	0	0	1	0	0	0	0	0	0
ENSMUSG00000103351	GM6350	0	0	0	0	0	0	0	0	1	0	0	0
ENSMUSG00000103358	GM37593	1	1	0	0	1	1	0	5	0	3	3	0
ENSMUSG00000103360	GM38156	0	0	0	0	0	0	2	0	0	1	1	1
ENSMUSG00000103364	GM38157	0	1	0	0	0	0	0	1	1	2	0	0
ENSMUSG00000103366	C630004L07F	2	0	0	3	0	2	3	5	4	8	1	2
ENSMUSG00000103373	GM37238	2	1	0	1	1	0	2	3	3	2	1	3
ENSMUSG00000103374	GM37239	0	0	0	0	0	0	0	0	0	1	0	0
ENSMUSG00000103375	GM37181	0	0	0	0	0	0	0	0	1	0	0	1
ENSMUSG00000103378	GM37176	0	0	0	0	0	0	0	0	1	0	2	1
ENSMUSG00000103380	GM37756	0	0	0	0	0	0	0	2	0	0	0	0
ENSMUSG00000103382	GM37755	0	1	1	1	1	0	3	0	0	1	0	0
ENSMUSG00000103388	GM37581	0	1	0	0	0	0	0	0	0	0	1	0
ENSMUSG00000103391	GM38302	0	0	0	0	0	0	0	0	0	1	0	0
ENSMUSG00000103398	GM38307	0	0	0	0	0	0	0	0	1	0	0	0
ENSMUSG00000103400	GM15853	0	0	0	0	0	1	0	2	1	0	0	2
ENSMUSG00000103402	GM37930	0	0	0	0	0	0	0	0	0	1	0	0
ENSMUSG00000103403	GM37931	0	0	0	0	0	1	1	3	2	4	1	4
ENSMUSG00000103408	GM37933	0	0	0	0	0	0	0	1	1	0	0	0
ENSMUSG00000103409	LSMEM2	2	1	2	2	2	1	15	9	11	7	3	12
ENSMUSG00000103420	GM37537	0	0	0	0	0	0	0	1	0	0	0	0
ENSMUSG00000103423	GM5845	2	0	0	3	4	0	3	5	5	7	3	4
ENSMUSG00000103427	GM37534	0	0	0	0	1	0	2	2	6	3	2	5
ENSMUSG00000103432	6720464F23F	0	2	1	2	1	0	0	3	3	7	1	0
ENSMUSG00000103435	GM36992	0	0	0	0	0	0	0	0	0	0	1	0
ENSMUSG00000103436	GM36995	0	0	0	0	0	0	0	1	0	0	0	0
ENSMUSG00000103441	7530428D23I	0	0	0	0	0	0	0	0	0	0	1	0
ENSMUSG00000103443	GM37132	2	1	0	0	0	0	2	1	0	3	1	1
ENSMUSG00000103445	GM36948	0	1	4	1	3	1	3	4	0	2	2	4
ENSMUSG00000103454	GM38098	0	0	0	0	0	0	0	0	1	1	1	0
ENSMUSG00000103473	GM37696	0	0	0	0	0	0	0	0	1	0	0	0
ENSMUSG00000103495	GM37409	0	0	0	1	0	0	0	2	0	1	1	0
ENSMUSG00000103497	GM37407	0	0	0	0	2	2	1	5	0	2	2	1
ENSMUSG00000103505	GM38374	0	1	1	0	0	0	4	1	0	3	3	3
ENSMUSG00000103506	GM38376	0	0	1	0	0	0	1	2	2	3	1	0
ENSMUSG00000103507	GM38375	0	0	0	0	0	0	0	0	0	0	1	0
ENSMUSG00000103509	GM38372	0	0	0	0	0	0	1	0	0	1	0	0
ENSMUSG00000103520	1110025M09	0	0	0	0	0	0	1	1	0	1	1	1

ENSMUSG00000103522	9430087J23R	1	0	0	0	0	0	0	0	0	1	0	0
ENSMUSG00000103525	GM37262	0	0	0	0	0	0	0	1	0	1	1	1
ENSMUSG00000103532	GM4430	0	0	0	2	0	0	0	0	0	0	0	0
ENSMUSG00000103536	GM37837	0	0	0	0	0	1	0	0	0	0	0	0
ENSMUSG00000103543	GM37669	0	0	0	0	0	0	0	0	0	0	1	0
ENSMUSG00000103553	GM38218	0	0	0	0	0	0	1	2	0	0	0	1
ENSMUSG00000103556	GM38215	0	0	0	0	0	0	0	2	0	0	0	0
ENSMUSG00000103558	GM38220	0	0	0	2	0	0	1	2	1	2	3	0
ENSMUSG00000103564	GM35067	0	0	0	0	0	0	0	1	0	0	0	0
ENSMUSG00000103569	GM38067	0	0	1	2	0	1	3	2	1	1	0	0
ENSMUSG00000103570	C630004M23	0	0	0	0	0	1	1	1	0	2	0	3
ENSMUSG00000103574	GM37116	0	0	0	0	0	0	2	3	1	1	0	1
ENSMUSG00000103576	GM38188	0	0	0	0	0	0	0	1	0	0	0	0
ENSMUSG00000103583	GM38325	0	0	0	1	0	0	0	0	0	0	0	0
ENSMUSG00000103586	5830405F06F	0	0	0	0	0	0	0	0	0	1	1	1
ENSMUSG00000103590	GM37353	0	1	0	1	0	1	1	1	0	1	1	1
ENSMUSG00000103591	GM38365	35	50	31	38	47	67	87	67	44	124	38	49
ENSMUSG00000103593	GM37352	0	0	0	0	0	0	1	1	1	0	0	0
ENSMUSG00000103599	GM37356	0	0	0	0	0	0	0	0	0	1	0	0
ENSMUSG00000103611	GM37195	0	0	0	0	0	0	1	0	0	1	0	0
ENSMUSG00000103614	GM37949	0	0	0	0	0	0	1	0	1	0	0	0
ENSMUSG00000103618	GM37951	0	0	0	0	0	0	0	0	0	0	0	1
ENSMUSG00000103621	GM38366	1	1	1	1	0	0	5	2	1	5	1	2
ENSMUSG00000103624	GM3081	0	0	0	0	0	0	1	1	1	2	0	1
ENSMUSG00000103632	GM38329	0	1	1	1	0	0	3	3	3	4	1	0
ENSMUSG00000103651	GM37206	0	1	2	0	1	2	1	0	2	1	5	1
ENSMUSG00000103653	GSTP-PS	1	0	0	1	0	1	1	2	1	3	0	3
ENSMUSG00000103657	GM37204	0	2	0	0	2	1	1	1	2	4	0	1
ENSMUSG00000103672	GM37621	0	1	0	1	1	1	3	2	2	1	0	2
ENSMUSG00000103693	GM37529	0	1	1	1	0	0	1	1	0	0	0	2
ENSMUSG00000103696	GM37531	0	3	3	2	1	3	3	9	4	8	1	5
ENSMUSG00000103697	GM38020	0	1	1	0	4	1	1	0	2	2	2	3
ENSMUSG00000103703	GM42568	0	0	0	0	1	0	0	0	0	0	0	0
ENSMUSG00000103706	6820402A03F	0	0	0	0	1	0	1	0	1	1	1	0
ENSMUSG00000103711	TSTD1	0	1	1	1	0	0	1	1	0	1	0	0
ENSMUSG00000103715	4933431K14F	0	0	0	0	0	0	0	0	0	0	1	0
ENSMUSG00000103728	GM37099	1	2	1	2	2	6	1	2	0	1	2	1
ENSMUSG00000103730	GM38316	5	5	2	9	5	3	35	23	23	19	16	20
ENSMUSG00000103731	GM17530	0	0	0	0	0	0	1	1	3	1	0	1
ENSMUSG00000103737	GM7142	1	0	0	0	0	0	0	0	0	0	0	0
ENSMUSG00000103739	GM37653	0	0	0	0	1	2	0	0	3	0	0	1
ENSMUSG00000103741	GM38241	0	1	0	0	0	0	0	0	0	0	0	0
ENSMUSG00000103746	1700001G17I	0	0	0	0	1	0	0	0	0	0	0	0
ENSMUSG00000103748	GM38243	0	0	0	0	0	0	0	0	1	1	0	1
ENSMUSG00000103753	GM6934	0	0	0	0	0	0	0	0	0	1	0	0
ENSMUSG00000103756	GM37285	0	0	0	0	0	0	0	1	0	0	0	0
ENSMUSG00000103763	GM37860	0	0	0	0	0	0	1	0	0	1	0	0
ENSMUSG00000103772	GM36933	0	0	1	0	0	1	2	1	0	4	2	4
ENSMUSG00000103774	GM36932	1	0	1	3	2	1	3	1	1	1	4	2
ENSMUSG00000103778	GM36930	0	0	1	0	0	3	1	0	2	1	0	1
ENSMUSG00000103779	GM36931	0	0	0	0	0	0	1	0	0	3	0	0
ENSMUSG00000103783	GM37522	1	0	0	0	0	0	0	3	2	1	0	0
ENSMUSG00000103792	GM3745	0	0	0	0	0	0	0	0	1	0	0	0
ENSMUSG00000103803	GM37064	1	0	0	0	0	0	0	0	0	0	0	0
ENSMUSG00000103804	GM37062	0	0	0	0	0	0	0	0	0	1	0	0
ENSMUSG00000103817	GM6680	1	0	0	0	0	0	0	0	1	2	0	2
ENSMUSG00000103821	D430013B06I	0	1	1	3	0	2	3	2	4	7	6	6
ENSMUSG00000103822	6030460B20F	0	2	1	3	4	1	3	2	1	4	5	1

ENSMUSG00000103823	GM7899	0	1	0	0	0	0	0	0	0	0	0	1
ENSMUSG00000103827	GM32950	0	0	0	0	0	0	1	0	0	2	0	0
ENSMUSG00000103835	GM37612	1	4	1	1	1	2	2	5	3	2	1	3
ENSMUSG00000103839	GM37607	0	1	1	0	1	1	2	4	4	8	3	7
ENSMUSG00000103845	GM19026	0	0	0	0	0	0	1	0	0	0	1	0
ENSMUSG00000103846	GM37809	1	0	1	0	0	0	0	1	2	0	0	0
ENSMUSG00000103850	GM3933	0	0	0	0	0	0	0	0	1	0	0	0
ENSMUSG00000103857	GM37249	0	0	0	2	0	0	1	2	0	1	3	1
ENSMUSG00000103864	GM37415	0	0	1	0	0	0	0	0	0	1	0	1
ENSMUSG00000103867	9630010A21F	1	2	3	3	2	4	8	5	2	12	6	5
ENSMUSG00000103869	GM37420	0	1	0	2	2	1	1	5	2	2	6	2
ENSMUSG00000103880	GM37009	0	0	1	0	1	0	0	0	0	0	0	0
ENSMUSG00000103898	GM30238	0	0	0	0	1	0	0	1	0	0	0	0
ENSMUSG00000103899	GM37940	0	0	0	0	0	0	0	1	0	1	1	0
ENSMUSG00000103900	GM37500	0	0	0	0	0	0	0	1	0	0	0	1
ENSMUSG00000103901	GM37499	0	0	0	0	0	0	0	1	1	0	0	0
ENSMUSG00000103906	TIGD5	0	0	0	0	1	0	0	0	0	3	1	0
ENSMUSG00000103907	GM37498	0	1	0	0	0	0	3	4	3	3	2	4
ENSMUSG00000103920	GM37897	0	0	0	0	0	0	1	0	0	0	0	0
ENSMUSG00000103921	GM5841	0	1	0	0	0	0	0	0	0	0	0	0
ENSMUSG00000103925	B230112G18I	0	0	0	0	0	0	1	0	0	0	0	0
ENSMUSG00000103926	GM37894	1	0	0	0	0	0	1	1	0	1	1	2
ENSMUSG00000103928	GM37893	0	1	1	4	0	0	4	2	3	1	4	4
ENSMUSG00000103929	GM37892	0	0	0	0	0	0	0	0	0	0	0	1
ENSMUSG00000103933	GM36964	0	0	0	0	0	0	1	1	0	0	0	0
ENSMUSG00000103937	GM37186	0	0	0	0	0	0	2	0	0	1	0	0
ENSMUSG00000103940	GM38227	0	0	0	1	0	0	1	0	0	1	0	0
ENSMUSG00000103944	RPL26-PS3	0	0	0	0	0	0	0	0	0	1	0	0
ENSMUSG00000103945	GM38228	2	2	2	2	3	3	1	3	5	11	4	8
ENSMUSG00000103948	4930594C11F	0	0	0	0	0	0	0	0	0	1	0	1
ENSMUSG00000103951	GM37269	1	0	0	0	0	0	0	0	0	1	0	0
ENSMUSG00000103952	GM37268	0	0	0	0	0	0	0	0	0	1	2	0
ENSMUSG00000103957	GM10766	0	0	0	3	1	0	1	1	0	1	1	0
ENSMUSG00000103964	GM8407	0	0	0	0	0	0	0	0	0	1	0	0
ENSMUSG00000103966	GM37120	0	0	0	0	1	0	0	0	0	0	0	0
ENSMUSG00000103967	GM38214	0	0	0	0	0	0	1	0	0	0	0	0
ENSMUSG00000103973	BC055308	0	0	0	0	0	0	0	2	0	0	0	0
ENSMUSG00000103975	GM37676	1	1	4	2	3	4	12	10	10	18	7	11
ENSMUSG00000103976	GM37677	0	2	1	1	0	0	2	2	1	0	2	2
ENSMUSG00000103983	GM20045	1	4	1	0	2	0	6	6	5	8	7	7
ENSMUSG00000103984	GM37447	0	0	1	1	1	0	0	1	0	0	1	0
ENSMUSG00000103996	4833421G17I	3	1	1	3	1	4	8	6	2	11	4	7
ENSMUSG00000103999	GM38026	0	0	0	0	0	0	0	1	0	0	0	0
ENSMUSG00000104002	GM38336	0	0	1	0	0	0	1	0	0	1	0	0
ENSMUSG00000104010	GM37366	0	0	1	0	1	0	2	0	2	1	0	0
ENSMUSG00000104016	GM37362	0	0	0	1	0	1	2	0	0	0	0	0
ENSMUSG00000104018	4833412K13F	0	1	0	0	1	0	1	0	0	2	2	0
ENSMUSG00000104020	GM37215	0	0	0	1	0	0	0	0	0	0	1	2
ENSMUSG00000104026	GM37212	0	0	0	0	0	0	2	1	0	0	0	0
ENSMUSG00000104030	5330406M23	1	1	1	0	0	0	0	6	2	3	0	1
ENSMUSG00000104037	GM37776	0	1	0	1	0	0	0	2	0	0	0	0
ENSMUSG00000104040	GM37563	0	0	0	0	0	0	1	0	0	0	0	0
ENSMUSG00000104043	GM6525	0	1	0	0	0	0	3	1	4	2	0	2
ENSMUSG00000104044	GM37566	0	1	2	1	0	1	3	3	3	2	1	5
ENSMUSG00000104052	GM38125	0	0	0	0	0	0	1	0	0	0	3	0
ENSMUSG00000104053	GM38126	1	0	4	2	1	0	2	2	3	8	0	5
ENSMUSG00000104060	GM37954	0	0	0	1	1	1	2	1	0	0	2	0
ENSMUSG00000104061	GM37953	1	0	0	3	1	2	3	1	0	0	1	4

ENSMUSG00000104069	GM37198	0	1	0	1	1	0	0	2	0	1	2	1
ENSMUSG00000104074	GM37025	0	0	0	0	0	0	1	0	1	1	0	0
ENSMUSG00000104078	GM37023	0	0	1	0	0	0	0	0	0	0	1	1
ENSMUSG00000104094	GM37314	0	1	0	0	0	0	2	0	0	3	0	0
ENSMUSG00000104096	GM20356	0	0	0	0	0	0	0	0	0	0	0	1
ENSMUSG00000104103	GM9517	0	0	0	1	0	0	2	0	1	1	0	1
ENSMUSG00000104107	GM37879	0	0	1	0	0	0	1	1	0	1	0	1
ENSMUSG00000104113	GM37292	0	0	0	0	0	0	0	0	1	0	0	1
ENSMUSG00000104116	GM37296	0	0	0	0	0	0	1	0	0	0	1	0
ENSMUSG00000104118	GM37298	0	3	1	0	0	3	6	7	5	7	4	8
ENSMUSG00000104125	GM37488	0	0	0	0	0	0	0	0	0	1	0	1
ENSMUSG00000104135	GM36953	0	0	0	0	1	0	0	0	0	0	0	0
ENSMUSG00000104136	GM36955	0	0	0	0	0	0	0	0	0	1	1	0
ENSMUSG00000104138	GM36949	0	0	0	0	0	0	0	0	1	0	1	0
ENSMUSG00000104145	D130019J16R	0	3	0	1	2	1	12	7	2	3	3	9
ENSMUSG00000104154	GM38104	0	0	0	0	0	0	0	1	3	2	2	0
ENSMUSG00000104156	GM38102	0	0	0	4	0	0	2	1	0	1	1	1
ENSMUSG00000104158	GM38100	0	0	0	0	0	0	0	1	0	0	0	0
ENSMUSG00000104159	GM38099	0	0	0	0	0	0	0	0	0	1	0	0
ENSMUSG00000104164	GM38248	0	1	0	0	0	2	3	2	6	5	1	1
ENSMUSG00000104166	GM38247	0	1	0	0	0	0	1	0	0	0	1	1
ENSMUSG00000104168	GM38250	0	0	0	0	0	0	2	0	1	1	2	1
ENSMUSG00000104170	GM37702	0	0	1	0	0	0	0	0	0	0	0	0
ENSMUSG00000104173	GM37703	0	0	0	1	0	0	0	0	0	0	0	0
ENSMUSG00000104181	GM8850	0	0	0	0	1	0	1	0	0	0	0	0
ENSMUSG00000104195	B230377A18R	0	0	0	0	0	0	0	1	1	0	0	0
ENSMUSG00000104197	GM37632	0	0	0	3	0	2	2	4	3	4	0	0
ENSMUSG00000104200	GM37399	0	0	0	0	2	0	0	2	1	0	1	0
ENSMUSG00000104202	GM8101	0	0	0	0	0	1	4	1	1	1	0	1
ENSMUSG00000104204	GM37397	0	0	0	0	0	0	0	1	2	5	1	0
ENSMUSG00000104208	GM37401	0	0	3	3	1	0	2	2	5	6	0	1
ENSMUSG00000104211	GM37985	1	0	0	0	0	0	0	0	0	0	0	0
ENSMUSG00000104212	GM37986	0	0	0	0	0	1	0	1	1	2	2	2
ENSMUSG00000104213	IGHD	0	0	2	0	0	0	5	3	4	6	1	4
ENSMUSG00000104214	GM36638	0	0	0	0	0	0	1	0	0	0	1	0
ENSMUSG00000104218	GM9134	0	0	0	0	0	0	2	0	1	0	0	0
ENSMUSG00000104222	GM7292	0	0	0	0	0	0	0	0	0	1	0	0
ENSMUSG00000104239	GM37588	0	0	0	0	0	0	0	0	1	0	0	0
ENSMUSG00000104241	GM9442	1	0	0	0	0	0	1	4	2	5	3	0
ENSMUSG00000104251	GM18036	0	0	0	0	0	0	1	0	0	0	0	0
ENSMUSG00000104253	GM37174	0	0	0	0	1	2	0	0	0	0	0	0
ENSMUSG00000104268	GM37750	0	0	0	0	0	0	0	1	0	0	0	1
ENSMUSG00000104273	A530064N14R	0	0	1	0	2	1	2	1	3	0	3	2
ENSMUSG00000104275	GM37867	0	0	0	0	0	0	0	0	0	0	1	0
ENSMUSG00000104277	GM38299	2	0	0	0	0	0	1	0	1	2	0	0
ENSMUSG00000104279	GM38300	0	1	0	0	0	0	0	1	1	0	1	1
ENSMUSG00000104280	RPS2-PS11	0	0	0	0	0	0	0	1	0	1	1	0
ENSMUSG00000104291	A130071D04R	29	61	36	88	48	45	119	98	78	128	71	95
ENSMUSG00000104293	GM38043	0	3	0	0	0	0	2	2	1	3	2	0
ENSMUSG00000104301	WDR49	0	0	0	0	0	0	0	0	0	1	0	0
ENSMUSG00000104302	GM36975	0	0	0	1	0	0	0	0	0	0	0	0
ENSMUSG00000104315	GM37906	1	0	1	0	0	0	4	0	2	4	2	5
ENSMUSG00000104324	GM37320	0	0	1	0	0	0	1	1	0	0	1	0
ENSMUSG00000104325	GM37321	0	0	0	0	0	0	0	0	1	0	0	0
ENSMUSG00000104328	GM37323	0	0	0	0	0	0	1	0	0	0	0	0
ENSMUSG00000104329	GM37324	0	0	0	0	0	0	0	0	1	0	0	0
ENSMUSG00000104341	GM37688	0	0	0	0	0	0	0	0	1	0	0	0
ENSMUSG00000104342	GM36401	1	1	1	1	1	2	5	10	6	10	8	9

ENSMUSG00000104344	GM38077	0	0	0	0	0	0	0	0	0	0	1	1
ENSMUSG00000104346	PCDHGA3	0	0	0	1	0	0	0	0	0	0	0	0
ENSMUSG00000104348	GM37691	0	0	0	0	0	0	0	0	2	1	0	0
ENSMUSG00000104350	GM38244	1	1	0	3	0	0	6	3	0	7	1	6
ENSMUSG00000104351	GM37125	0	0	0	0	0	0	2	0	1	0	1	1
ENSMUSG00000104358	GM37127	0	2	0	0	0	1	0	0	0	1	0	0
ENSMUSG00000104369	GM38082	0	0	0	0	0	1	3	2	1	1	1	1
ENSMUSG00000104378	GM37510	0	2	0	0	1	1	1	6	4	1	1	1
ENSMUSG00000104379	GM37509	0	0	1	0	0	0	0	1	0	1	0	0
ENSMUSG00000104383	GM37553	0	0	0	0	1	0	1	2	1	1	0	1
ENSMUSG00000104388	GM37033	3	1	3	5	1	2	11	8	3	6	5	7
ENSMUSG00000104392	GM37962	1	0	0	0	2	1	1	2	0	0	0	2
ENSMUSG00000104394	GM37254	0	1	0	1	0	0	0	0	0	0	0	0
ENSMUSG00000104399	GM37963	0	0	0	0	0	0	0	1	1	0	0	0
ENSMUSG00000104406	GM38014	0	2	0	2	0	0	1	4	0	5	1	2
ENSMUSG00000104413	GM37065	0	0	0	0	0	1	0	1	0	0	0	0
ENSMUSG00000104415	GM37069	0	0	0	0	1	0	0	0	0	0	0	0
ENSMUSG00000104418	GM37070	0	0	0	0	2	0	1	0	0	0	0	1
ENSMUSG00000104432	A430027C01F	0	0	1	1	1	0	2	4	0	4	0	2
ENSMUSG00000104434	GM37421	0	0	0	0	0	0	0	0	0	0	0	1
ENSMUSG00000104435	GM37422	1	1	2	3	2	3	12	13	11	12	8	12
ENSMUSG00000104436	GM37423	2	3	1	1	2	3	3	3	6	5	4	7
ENSMUSG00000104444	GM33051	0	2	0	3	2	2	8	14	11	12	12	15
ENSMUSG00000104448	GM37256	1	0	0	0	0	0	1	1	0	0	0	0
ENSMUSG00000104449	GM37255	2	2	1	2	4	4	6	0	2	4	1	1
ENSMUSG00000104453	GM37829	0	1	0	3	1	2	9	7	1	13	6	3
ENSMUSG00000104459	GM37824	0	0	0	0	0	0	0	1	0	1	0	1
ENSMUSG00000104467	GM37660	1	1	0	2	0	0	2	5	4	1	1	3
ENSMUSG00000104469	GM37663	1	1	1	0	2	0	1	1	1	2	1	1
ENSMUSG00000104471	GM38208	0	0	0	0	0	0	0	0	0	2	1	0
ENSMUSG00000104485	GM38065	1	0	0	0	0	1	0	0	0	1	1	0
ENSMUSG00000104488	GM38062	0	0	1	0	0	2	0	1	0	2	3	2
ENSMUSG00000104493	GM19552	0	0	0	1	0	0	0	1	0	1	0	0
ENSMUSG00000104503	GM37738	1	0	0	0	0	0	0	0	0	1	0	0
ENSMUSG00000104508	GM37735	0	0	0	0	1	1	2	0	0	2	0	0
ENSMUSG00000104513	GM29813	0	0	0	0	0	0	0	0	0	0	1	0
ENSMUSG00000104515	GM37163	0	0	0	0	0	0	0	0	0	1	0	0
ENSMUSG00000104523	GM37335	0	0	0	2	0	1	5	5	6	4	2	6
ENSMUSG00000104524	GM37333	0	2	0	2	0	3	1	2	3	4	1	1
ENSMUSG00000104525	GM37334	0	0	1	0	0	0	0	0	0	0	0	0
ENSMUSG00000104545	E030032P16F	0	0	0	0	0	0	2	1	0	1	1	3
ENSMUSG00000104546	GM43858	0	0	0	0	0	0	1	0	2	1	0	2
ENSMUSG00000104548	GM43857	1	2	2	8	4	1	25	30	25	10	22	25
ENSMUSG00000104550	GM43589	0	0	0	0	0	0	0	0	0	0	0	1
ENSMUSG00000104559	GM43118	0	0	1	0	2	2	14	6	6	8	3	10
ENSMUSG00000104569	GM43054	0	0	0	0	0	0	1	0	0	2	1	1
ENSMUSG00000104571	GM43010	1	0	1	0	0	0	1	1	2	1	1	1
ENSMUSG00000104585	4921511C10F	0	0	0	0	0	0	0	0	0	0	1	1
ENSMUSG00000104592	GM42721	0	1	0	0	0	1	0	0	1	1	0	2
ENSMUSG00000104597	B230334C09F	1	0	0	1	0	0	3	1	1	1	1	0
ENSMUSG00000104606	GM43409	0	0	0	0	0	0	0	0	0	0	0	2
ENSMUSG00000104612	GM42449	2	1	0	1	0	0	0	1	3	2	2	2
ENSMUSG00000104621	GM43185	0	0	0	0	1	0	0	0	0	2	0	2
ENSMUSG00000104623	GM6394	9	16	16	15	10	14	30	42	32	40	27	35
ENSMUSG00000104631	GM43774	4	2	1	4	2	1	1	3	4	6	2	2
ENSMUSG00000104639	GM42939	1	0	0	1	0	0	0	0	0	0	0	0
ENSMUSG00000104641	GM43290	0	0	0	0	0	0	0	1	2	1	0	0
ENSMUSG00000104647	GM43485	0	0	0	0	0	0	0	0	0	1	0	0

ENSMUSG00000104648	GM42570	0	0	0	0	0	0	1	1	0	0	0	0
ENSMUSG00000104649	GM43712	34	56	43	52	49	48	159	144	92	138	96	119
ENSMUSG00000104662	GM43267	0	0	0	0	0	1	0	0	0	0	0	0
ENSMUSG00000104665	GM43366	0	0	1	0	0	0	0	0	0	0	0	0
ENSMUSG00000104671	GM43062	1	1	0	0	0	1	2	1	0	6	1	2
ENSMUSG00000104675	GM43689	0	0	0	0	0	2	3	4	2	4	2	2
ENSMUSG00000104682	GM42636	0	1	0	0	0	0	1	1	1	0	2	3
ENSMUSG00000104686	GM42815	0	0	0	0	0	0	0	0	0	1	0	0
ENSMUSG00000104693	GM42941	0	0	0	0	0	0	0	0	0	0	1	0
ENSMUSG00000104699	RPS4X-PS	0	0	0	0	1	0	0	0	1	0	0	0
ENSMUSG00000104701	GM42555	0	1	0	0	0	0	1	0	0	1	0	0
ENSMUSG00000104704	GM43133	0	0	0	0	1	0	0	2	0	1	0	1
ENSMUSG00000104706	GM43421	0	0	0	0	0	0	0	0	0	0	0	1
ENSMUSG00000104713	GBP6	0	1	0	0	1	0	0	0	0	1	0	0
ENSMUSG00000104719	4933437G19I	0	0	0	0	0	0	1	0	0	0	0	0
ENSMUSG00000104720	GM43138	0	0	2	0	0	0	0	0	0	0	0	0
ENSMUSG00000104721	GM42696	0	1	0	0	0	0	0	0	0	0	0	0
ENSMUSG00000104724	GM43162	0	0	0	0	3	0	1	2	0	2	0	3
ENSMUSG00000104728	GM42462	0	0	0	0	0	0	2	0	0	0	0	0
ENSMUSG00000104761	GM43511	0	0	1	0	1	0	1	0	1	1	0	2
ENSMUSG00000104771	GM44394	1	0	0	0	0	0	0	0	0	0	0	0
ENSMUSG00000104775	GM8182	0	0	0	0	0	0	0	0	1	0	0	0
ENSMUSG00000104777	GM4865	0	0	0	0	0	0	0	0	0	1	0	0
ENSMUSG00000104781	GM43303	0	0	0	0	0	0	0	0	0	1	0	0
ENSMUSG00000104793	GM43756	0	0	0	0	0	0	2	1	1	0	0	0
ENSMUSG00000104795	GM42783	1	1	1	1	1	4	3	3	6	10	4	4
ENSMUSG00000104803	GM43108	0	0	0	0	0	1	0	0	0	0	0	0
ENSMUSG00000104806	GM42566	1	0	1	0	0	0	2	0	0	1	0	1
ENSMUSG00000104814	GM42979	0	0	1	0	1	1	1	1	1	3	3	2
ENSMUSG00000104822	GM42967	0	0	0	1	1	0	0	3	4	2	2	0
ENSMUSG00000104830	5830487J09R	4	2	1	4	1	1	5	4	4	4	4	3
ENSMUSG00000104835	GM5547	1	2	1	1	0	0	6	4	1	3	3	5
ENSMUSG00000104846	GM43020	0	0	0	0	0	0	0	0	0	0	0	1
ENSMUSG00000104852	GM43201	5	4	3	3	4	14	8	7	1	15	2	6
ENSMUSG00000104854	MIR7116	0	0	0	0	0	0	1	0	0	0	0	0
ENSMUSG00000104862	GM6520	0	0	0	0	0	1	0	1	0	0	1	1
ENSMUSG00000104873	GM40332	0	1	0	0	0	0	1	0	0	1	0	0
ENSMUSG00000104874	GM43387	1	1	0	1	0	0	0	1	1	0	0	0
ENSMUSG00000104877	GM42651	0	1	0	0	0	0	1	0	1	0	1	0
ENSMUSG00000104882	GM43096	0	0	0	0	0	0	0	0	0	1	0	1
ENSMUSG00000104891	2510017J16R	0	0	0	0	3	0	2	2	0	1	0	1
ENSMUSG00000104897	GM43203	0	0	0	0	0	0	2	0	1	2	0	1
ENSMUSG00000104900	4930596I21R	0	0	0	0	0	0	1	1	2	0	0	0
ENSMUSG00000104903	GM43707	0	0	0	1	0	2	1	0	0	0	0	1
ENSMUSG00000104904	9330198I05R	0	0	0	0	0	0	0	0	0	3	0	0
ENSMUSG00000104906	GM43005	1	1	0	0	0	0	0	0	0	1	1	1
ENSMUSG00000104910	GM43331	0	0	0	0	1	0	1	0	2	1	0	1
ENSMUSG00000104916	GM42830	0	1	0	0	0	0	1	0	0	0	0	0
ENSMUSG00000104924	GM43002	0	1	0	0	0	0	1	0	0	0	1	0
ENSMUSG00000104925	GM43061	0	0	0	1	1	0	3	2	4	1	2	1
ENSMUSG00000104935	GM43414	5	8	8	9	8	7	28	31	31	36	23	35
ENSMUSG00000104940	GM42908	0	2	0	0	0	1	6	3	8	3	0	4
ENSMUSG00000104943	GM42868	0	0	0	0	0	0	1	1	0	0	0	0
ENSMUSG00000104947	GM43721	0	0	2	0	0	0	0	0	0	1	0	0
ENSMUSG00000104950	4833413G10I	0	0	0	1	0	0	2	0	3	1	1	0
ENSMUSG00000104951	GM43413	1	0	1	0	0	1	1	0	1	4	0	1
ENSMUSG00000104956	4930429D17I	0	0	0	1	0	0	0	0	0	0	0	0
ENSMUSG00000104960	SNHG8	46	38	43	43	61	42	136	190	123	160	190	125

ENSMUSG00000104965	GM43437	0	0	1	1	1	1	8	5	2	3	5	4
ENSMUSG00000104969	GM43445	1	1	1	1	0	0	3	1	1	2	3	3
ENSMUSG00000104970	GM43137	0	0	0	0	0	0	1	0	1	0	0	0
ENSMUSG00000104973	A530041M06	2	2	3	3	1	0	1	3	0	1	4	6
ENSMUSG00000104974	GM43686	0	0	0	0	0	0	0	0	0	0	0	1
ENSMUSG00000104983	GM42763	0	0	0	0	0	0	0	1	0	0	1	0
ENSMUSG00000104985	GM42917	0	0	0	0	0	0	1	1	0	0	0	0
ENSMUSG00000104986	1600023N17I	0	1	0	1	0	1	0	0	0	1	0	1
ENSMUSG00000105001	GM47275	0	0	0	0	0	0	1	0	0	0	0	0
ENSMUSG00000105003	GM40055	0	0	0	0	0	0	0	0	1	0	0	0
ENSMUSG00000105008	GM43652	2	0	1	5	0	2	2	2	1	3	3	0
ENSMUSG00000105019	GM43547	0	0	0	0	0	0	1	0	0	0	0	0
ENSMUSG00000105021	GM8234	0	0	0	0	0	0	0	0	1	0	2	0
ENSMUSG00000105029	GM44363	0	0	0	1	0	0	0	0	0	0	0	0
ENSMUSG00000105031	GM3511	30	45	37	35	40	44	97	113	82	82	88	102
ENSMUSG00000105033	GM43423	0	0	0	0	0	0	0	0	0	0	0	2
ENSMUSG00000105044	GM10416	0	0	0	0	0	0	0	0	1	0	1	0
ENSMUSG00000105058	GM43590	0	0	0	0	0	0	0	0	0	0	0	1
ENSMUSG00000105063	GM43488	1	0	0	0	0	0	1	1	0	0	0	0
ENSMUSG00000105065	GM42513	0	0	0	0	0	0	0	0	2	0	0	0
ENSMUSG00000105071	GM43336	1	0	0	0	1	0	1	3	2	0	1	1
ENSMUSG00000105072	GM44335	0	0	0	0	0	0	1	0	0	0	0	0
ENSMUSG00000105076	A930003O13I	0	0	0	0	0	0	0	0	0	1	0	0
ENSMUSG00000105077	GM4859	0	0	0	1	0	0	1	1	1	1	0	1
ENSMUSG00000105082	AQP10-PS	1	0	0	0	0	0	1	1	0	0	0	0
ENSMUSG00000105087	GM42552	0	0	0	0	0	0	0	0	0	0	0	1
ENSMUSG00000105095	8430422M14	0	0	1	1	1	0	0	0	2	1	3	4
ENSMUSG00000105096	GBP10	0	0	0	0	0	0	0	0	1	0	0	0
ENSMUSG00000105107	GM43412	0	0	0	0	0	0	0	0	0	2	0	0
ENSMUSG00000105108	GM42687	0	0	0	1	0	0	0	0	0	0	0	0
ENSMUSG00000105123	IGHV1-79	0	0	1	0	0	0	0	0	0	1	0	0
ENSMUSG00000105124	GM5297	0	1	0	0	0	0	1	2	0	0	1	0
ENSMUSG00000105130	GM43825	0	0	0	0	0	0	1	0	0	0	0	0
ENSMUSG00000105135	GM43667	0	0	0	0	1	1	0	2	2	2	0	3
ENSMUSG00000105137	GM42869	0	0	3	0	0	0	0	4	2	2	2	2
ENSMUSG00000105139	GM19391	0	1	0	0	1	1	1	4	1	1	0	6
ENSMUSG00000105144	RPL21-PS11	0	0	0	0	0	0	1	1	0	1	0	0
ENSMUSG00000105154	GM20128	0	0	0	0	0	0	0	0	0	1	0	0
ENSMUSG00000105155	GM42910	0	0	0	0	0	0	0	0	0	1	0	0
ENSMUSG00000105156	GM9057	0	0	0	0	0	1	0	1	0	0	1	0
ENSMUSG00000105161	GM42595	0	1	0	1	0	2	2	5	3	6	4	1
ENSMUSG00000105167	SNORD43	0	0	1	0	0	0	0	0	0	0	0	0
ENSMUSG00000105176	GM43668	3	1	0	1	0	2	11	15	4	7	7	6
ENSMUSG00000105179	GM42866	0	0	0	1	0	0	1	0	2	0	1	1
ENSMUSG00000105181	GM19620	0	0	0	0	1	1	2	4	0	3	2	3
ENSMUSG00000105196	MIR142	0	0	0	1	1	0	5	1	3	0	1	1
ENSMUSG00000105199	GM43581	9	9	5	9	6	19	17	19	14	36	12	19
ENSMUSG00000105201	GM43362	16	15	14	16	10	8	17	17	14	21	7	9
ENSMUSG00000105204	GM43738	0	1	0	0	0	0	0	0	0	0	0	0
ENSMUSG00000105228	GM43340	0	0	1	0	0	1	1	0	1	1	1	0
ENSMUSG00000105229	GM43149	0	0	0	0	0	0	0	0	0	1	0	0
ENSMUSG00000105232	GM47284	0	0	0	1	0	0	0	1	0	2	1	0
ENSMUSG00000105233	GM20568	0	0	0	0	1	2	0	0	0	0	0	0
ENSMUSG00000105245	GM31305	0	0	1	0	0	0	0	1	0	0	0	0
ENSMUSG00000105250	GM29736	0	0	0	0	0	0	0	1	0	0	0	1
ENSMUSG00000105255	GM42413	0	0	0	0	0	0	0	0	1	1	0	0
ENSMUSG00000105259	GM42874	1	0	0	0	0	0	0	0	1	0	1	0
ENSMUSG00000105260	GM40040	1	1	0	1	1	0	10	5	1	3	2	7

ENSMUSG00000105261	GM43333	0	0	0	4	0	2	4	14	4	3	2	7
ENSMUSG00000105263	GM42427	0	0	0	0	0	0	0	0	0	0	0	1
ENSMUSG00000105264	GM42716	0	0	0	0	1	0	0	0	0	0	0	0
ENSMUSG00000105265	SOX2OT	0	1	0	0	0	0	0	0	0	0	0	0
ENSMUSG00000105272	GM43071	0	1	0	0	0	0	0	0	1	0	0	0
ENSMUSG00000105283	GM33370	0	1	0	0	0	0	0	0	0	0	0	0
ENSMUSG00000105286	GM43140	0	0	0	0	0	0	1	0	0	0	0	0
ENSMUSG00000105294	GM43304	0	0	0	0	1	0	0	0	0	1	2	1
ENSMUSG00000105297	1700052H01F	0	1	0	0	0	0	0	0	0	0	0	0
ENSMUSG00000105304	GM43696	0	4	1	1	1	1	3	1	1	8	2	3
ENSMUSG00000105326	6030400A10F	0	0	0	1	0	0	0	0	0	0	0	0
ENSMUSG00000105327	GM9409	0	0	1	0	0	0	1	0	0	0	0	0
ENSMUSG00000105328	GM43180	0	0	0	0	0	0	1	0	0	0	0	0
ENSMUSG00000105331	GM29865	0	0	0	0	0	0	1	0	0	0	1	1
ENSMUSG00000105333	MIR684-2	0	0	1	0	0	0	0	0	0	0	0	0
ENSMUSG00000105339	GM42457	0	1	0	0	1	2	2	0	1	7	2	2
ENSMUSG00000105341	GM43817	0	0	0	0	0	0	1	0	1	0	0	0
ENSMUSG00000105349	GM43604	0	0	0	0	0	0	0	2	0	1	1	1
ENSMUSG00000105350	4930537H20F	0	0	0	1	1	0	0	1	0	0	0	0
ENSMUSG00000105358	GM43099	0	0	0	0	0	1	0	0	0	0	0	0
ENSMUSG00000105359	RPL21-PS10	0	1	0	0	0	1	0	0	0	0	0	0
ENSMUSG00000105377	GM43148	0	1	0	0	0	0	0	1	1	0	0	0
ENSMUSG00000105378	GM42515	0	5	1	1	1	2	3	3	3	5	4	7
ENSMUSG00000105382	GM43339	1	1	0	0	0	0	2	3	1	1	1	1
ENSMUSG00000105388	RPL36A-PS2	14	18	11	16	14	14	38	48	35	36	29	34
ENSMUSG00000105390	GM8458	0	0	0	0	0	0	0	0	0	1	1	0
ENSMUSG00000105392	GM42684	0	0	0	0	0	0	1	0	0	0	0	0
ENSMUSG00000105395	GM42672	0	0	0	0	0	0	0	0	0	0	1	0
ENSMUSG00000105406	GM43247	0	0	0	1	0	0	0	0	0	0	0	0
ENSMUSG00000105408	GM7285	2	0	1	2	3	0	11	6	6	4	4	3
ENSMUSG00000105412	GM43768	0	1	0	0	1	0	1	3	0	2	1	1
ENSMUSG00000105415	GM44364	1	1	2	2	1	1	8	10	9	13	6	12
ENSMUSG00000105423	GM36814	1	0	0	2	0	2	7	7	1	3	2	6
ENSMUSG00000105429	GM43692	0	1	0	0	0	0	1	3	0	1	0	2
ENSMUSG00000105434	GM43359	1	1	2	0	1	2	1	3	0	2	1	2
ENSMUSG00000105442	GM42614	0	0	0	0	0	0	2	0	1	4	0	1
ENSMUSG00000105447	GM43653	0	0	1	0	0	0	1	0	2	0	0	2
ENSMUSG00000105449	GM43379	0	0	0	3	1	0	0	4	1	4	0	0
ENSMUSG00000105457	GM43200	0	0	0	1	0	1	3	1	0	2	0	1
ENSMUSG00000105459	4930449I04R	1	0	0	0	0	0	0	0	0	0	0	0
ENSMUSG00000105460	GM42671	0	0	0	0	0	0	1	0	0	1	0	2
ENSMUSG00000105466	GM42998	0	0	1	0	0	0	0	0	1	2	0	2
ENSMUSG00000105471	A430073D23I	0	0	0	1	0	0	0	0	0	1	0	0
ENSMUSG00000105500	GM42591	0	0	0	0	0	0	0	0	0	0	1	0
ENSMUSG00000105501	5330426L24R	0	0	1	1	1	0	2	1	1	5	6	4
ENSMUSG00000105509	C130013H08I	0	0	0	0	0	0	1	0	0	1	0	1
ENSMUSG00000105510	GM43815	0	0	0	0	0	0	1	1	0	0	0	0
ENSMUSG00000105512	GM43714	0	0	0	0	0	0	0	0	0	1	0	0
ENSMUSG00000105514	GM42913	0	0	0	0	1	0	0	1	0	0	0	0
ENSMUSG00000105517	GM42436	0	0	0	2	1	2	2	2	2	1	2	1
ENSMUSG00000105519	E430021H15F	2	2	2	1	1	6	5	7	3	4	4	3
ENSMUSG00000105529	GM9480	0	1	0	0	1	0	1	0	0	5	2	2
ENSMUSG00000105541	GM43136	0	0	0	0	0	0	0	0	0	0	1	1
ENSMUSG00000105542	GM34248	0	0	0	1	0	0	0	1	0	0	0	0
ENSMUSG00000105543	GM43169	0	0	0	1	0	0	0	0	0	0	0	0
ENSMUSG00000105550	GM35585	0	1	1	0	0	0	0	0	0	0	0	0
ENSMUSG00000105552	GM19710	2	3	0	4	4	0	8	15	8	12	7	9
ENSMUSG00000105553	GM42514	0	0	0	1	0	0	0	1	0	2	0	0

ENSMUSG00000105554	GM7118	0	0	0	0	0	0	0	0	0	1	0	0
ENSMUSG00000105555	GM43856	0	0	0	0	0	0	0	0	0	1	0	0
ENSMUSG00000105561	GM43462	1	3	1	4	2	3	9	3	3	13	3	9
ENSMUSG00000105565	GM43566	0	3	2	0	2	1	3	5	2	6	7	4
ENSMUSG00000105572	GM43300	0	1	0	1	0	0	1	2	1	2	2	0
ENSMUSG00000105586	GM43009	2	0	3	0	3	1	4	6	9	5	4	7
ENSMUSG00000105597	4633401B06[illegible]	2	2	0	2	0	3	7	3	10	9	3	2
ENSMUSG00000105599	GM9238	1	0	0	1	1	0	0	1	0	1	1	0
ENSMUSG00000105601	GM42725	0	0	1	1	0	0	1	2	2	3	0	2
ENSMUSG00000105604	GM42838	0	0	0	0	0	0	0	1	0	0	0	0
ENSMUSG00000105607	GM43513	0	1	0	1	0	0	2	3	1	0	1	2
ENSMUSG00000105613	GM43684	0	2	0	0	0	1	1	1	1	0	1	1
ENSMUSG00000105616	GM3010	0	0	0	0	0	0	0	0	0	0	0	1
ENSMUSG00000105624	GM43187	0	0	0	0	0	0	0	0	0	1	0	0
ENSMUSG00000105636	GM43625	0	0	0	0	0	0	1	3	0	3	1	2
ENSMUSG00000105641	GM5853	0	0	0	0	0	0	0	1	0	0	0	1
ENSMUSG00000105643	GM42611	1	1	2	0	0	0	1	1	3	3	4	1
ENSMUSG00000105644	GM19739	0	0	0	0	0	0	2	1	0	0	1	1
ENSMUSG00000105645	GM6649	0	0	0	0	0	0	1	0	0	2	1	1
ENSMUSG00000105655	GM42659	4	7	5	12	6	5	14	13	12	32	9	14
ENSMUSG00000105659	4930509H03[illegible]	0	0	0	0	0	1	1	1	0	0	0	0
ENSMUSG00000105662	GM6639	0	0	0	0	0	0	0	0	0	0	1	0
ENSMUSG00000105669	GM18341	0	0	0	0	0	0	1	0	0	0	0	0
ENSMUSG00000105671	GM31306	1	0	0	0	0	0	1	2	1	1	1	0
ENSMUSG00000105674	GM42437	0	0	0	0	0	1	0	1	0	2	1	0
ENSMUSG00000105676	GM43403	0	0	0	0	0	0	0	0	0	0	0	1
ENSMUSG00000105677	GM43328	0	0	0	1	1	1	0	3	0	2	0	1
ENSMUSG00000105679	GM43338	0	0	0	0	0	0	2	0	0	0	0	0
ENSMUSG00000105681	GM43428	0	0	0	0	1	0	1	1	0	0	0	0
ENSMUSG00000105691	GM42876	0	1	0	3	0	0	1	1	1	0	2	0
ENSMUSG00000105692	GM43737	0	0	1	1	0	0	2	1	1	3	1	2
ENSMUSG00000105699	GM43703	1	0	0	0	0	0	4	2	0	0	0	1
ENSMUSG00000105700	GM42772	0	0	1	0	0	0	0	0	0	0	0	0
ENSMUSG00000105703	GM43305	0	1	0	0	0	0	0	0	0	1	0	0
ENSMUSG00000105707	GM43753	0	0	0	0	0	0	0	0	0	1	0	1
ENSMUSG00000105710	GM43859	1	0	0	1	0	1	1	2	0	0	0	0
ENSMUSG00000105716	GM43522	0	0	0	0	0	0	0	1	0	0	0	0
ENSMUSG00000105728	GM42819	1	0	1	1	0	0	1	0	2	2	2	0
ENSMUSG00000105731	GM43063	1	0	1	0	0	1	1	4	1	0	0	1
ENSMUSG00000105737	GM6105	0	0	0	0	0	0	0	1	1	0	0	0
ENSMUSG00000105740	GM42685	1	0	0	3	0	1	3	4	5	1	6	4
ENSMUSG00000105741	C79130	0	0	0	0	0	0	0	0	1	0	0	0
ENSMUSG00000105742	GM42748	0	0	0	0	0	0	2	0	1	1	0	0
ENSMUSG00000105745	GM42521	0	1	0	1	0	0	0	1	0	0	0	0
ENSMUSG00000105748	GM43088	0	1	0	1	0	0	2	3	0	0	1	0
ENSMUSG00000105755	GM43514	0	0	0	0	0	0	0	0	0	0	0	1
ENSMUSG00000105757	IGHV1-83	0	0	0	0	0	0	1	0	0	0	0	0
ENSMUSG00000105759	GM42425	0	1	0	0	0	0	0	0	0	0	0	0
ENSMUSG00000105762	GM43605	0	0	0	0	0	0	0	1	0	0	0	0
ENSMUSG00000105774	GM43006	0	1	0	0	0	0	0	0	0	0	1	0
ENSMUSG00000105784	GM35065	0	0	0	0	0	0	0	0	0	0	0	1
ENSMUSG00000105789	GM42997	0	0	0	0	0	0	0	0	0	3	0	1
ENSMUSG00000105796	GM42845	0	0	0	0	0	0	0	0	0	0	0	1
ENSMUSG00000105801	C430019N01[illegible]	0	0	1	1	0	0	2	1	1	1	0	0
ENSMUSG00000105803	GM43526	0	0	0	0	0	1	0	0	0	1	0	1
ENSMUSG00000105804	GM43654	0	0	0	0	0	0	1	0	0	0	0	0
ENSMUSG00000105810	GM43435	17	22	23	24	15	19	61	54	32	72	50	59
ENSMUSG00000105814	MIR703	13	11	9	24	11	5	64	69	49	81	58	60

ENSMUSG00000105815	GM43541	0	0	0	0	0	0	0	1	2	0	0	0
ENSMUSG00000105816	D030025E07F	0	0	0	0	0	0	1	1	1	1	1	0
ENSMUSG00000105824	GM43466	0	1	1	0	0	1	1	1	1	1	1	0
ENSMUSG00000105826	GM42141	0	0	0	0	0	0	0	0	0	3	0	1
ENSMUSG00000105827	HIST2H2BB	0	0	0	0	1	1	1	0	0	2	0	1
ENSMUSG00000105832	GM43841	0	1	0	0	0	0	0	1	1	1	0	0
ENSMUSG00000105834	4930592C13F	0	0	0	0	0	0	1	0	0	1	0	0
ENSMUSG00000105837	GM35986	0	0	0	0	0	0	0	0	0	1	0	0
ENSMUSG00000105842	GM43329	0	0	0	2	1	0	0	1	3	4	3	3
ENSMUSG00000105848	GM42683	0	0	0	0	0	0	0	0	0	1	0	0
ENSMUSG00000105851	9130604C24F	0	0	0	1	0	0	0	0	0	1	1	0
ENSMUSG00000105852	GM42890	0	0	0	0	0	1	1	1	0	8	2	2
ENSMUSG00000105854	GM8925	0	0	0	1	0	0	0	1	0	1	0	0
ENSMUSG00000105858	2310074N15I	0	0	0	0	0	0	0	0	0	1	0	1
ENSMUSG00000105866	GM6745	0	0	0	0	0	0	2	0	0	1	0	0
ENSMUSG00000105868	GM43766	1	1	1	0	2	1	2	5	5	2	5	2
ENSMUSG00000105870	7330423F06R	2	6	1	4	11	9	7	5	6	11	8	5
ENSMUSG00000105879	GM6204	4	5	4	4	11	6	30	26	19	16	33	29
ENSMUSG00000105881	4932422M17	1	2	0	1	0	0	4	2	0	2	3	6
ENSMUSG00000105893	WDR46-PS	1	0	0	2	0	0	1	1	0	0	0	0
ENSMUSG00000105895	GM42829	0	0	0	0	0	0	1	1	0	3	0	0
ENSMUSG00000105896	GM43311	0	0	0	0	0	0	0	1	0	0	1	0
ENSMUSG00000105914	GM19243	0	0	0	0	0	0	1	1	0	1	0	0
ENSMUSG00000105926	GM43386	0	0	0	0	0	0	0	0	0	0	0	1
ENSMUSG00000105928	GM9256	0	0	0	0	0	0	0	1	0	0	0	0
ENSMUSG00000105929	GM43693	0	0	1	0	0	1	0	1	0	0	0	0
ENSMUSG00000105935	GM43628	1	0	0	0	1	2	4	5	1	3	3	2
ENSMUSG00000105936	GM43544	0	1	4	0	0	0	2	1	3	2	1	1
ENSMUSG00000105942	GM43175	0	0	1	0	0	0	0	0	0	0	0	0
ENSMUSG00000105944	GM5075	1	0	0	1	0	0	0	1	0	1	0	0
ENSMUSG00000105950	GM43679	0	0	0	0	0	0	0	1	0	0	0	2
ENSMUSG00000105957	GM43254	0	1	1	0	0	0	0	0	0	0	0	2
ENSMUSG00000105962	GM42432	1	0	0	1	0	1	0	2	1	2	2	1
ENSMUSG00000105970	GM43360	0	0	0	0	0	0	0	0	1	3	1	1
ENSMUSG00000105973	GM42881	0	0	0	0	0	0	0	0	0	0	1	0
ENSMUSG00000105975	GM42609	0	0	0	0	0	0	0	1	0	2	1	2
ENSMUSG00000105976	GM42782	0	0	0	0	0	0	0	0	0	1	1	1
ENSMUSG00000105979	GM43411	0	0	0	0	0	0	0	0	1	0	0	0
ENSMUSG00000105981	2810428J06R	0	1	0	0	0	0	0	1	0	1	0	0
ENSMUSG00000105985	GM42993	0	0	1	0	0	0	0	0	0	0	0	0
ENSMUSG00000105986	GM43065	0	0	0	0	0	0	0	1	0	0	1	2
ENSMUSG00000105987	AI506816	3	2	5	2	4	3	26	24	23	27	17	28
ENSMUSG00000105992	GM43651	0	0	0	0	0	0	0	1	0	0	0	0
ENSMUSG00000106000	GM16508	0	0	0	0	0	0	0	1	1	0	0	0
ENSMUSG00000106001	GM42826	1	0	0	0	0	0	0	1	1	0	3	2
ENSMUSG00000106005	GM29151	0	0	0	0	0	1	0	0	0	1	2	0
ENSMUSG00000106009	GM9379	0	0	0	0	1	0	0	1	0	0	0	0
ENSMUSG00000106018	GM43527	0	0	0	0	0	0	0	1	0	0	0	0
ENSMUSG00000106019	GM43672	0	0	0	0	0	0	0	0	0	0	0	1
ENSMUSG00000106025	GM42940	2	1	2	3	0	1	9	12	7	7	5	7
ENSMUSG00000106032	GM42463	0	0	0	0	0	0	0	0	1	0	0	0
ENSMUSG00000106035	GM43545	1	0	0	0	0	0	0	0	0	0	1	0
ENSMUSG00000106037	GM4332	0	0	0	1	2	3	1	1	5	4	4	4
ENSMUSG00000106040	CYP3A63-PS	0	0	0	0	0	1	0	0	0	0	0	0
ENSMUSG00000106044	GM42860	0	0	0	0	0	1	0	1	0	0	0	0
ENSMUSG00000106057	GM43560	0	1	1	0	2	1	2	8	0	3	3	1
ENSMUSG00000106058	GM43013	0	0	0	0	0	0	0	1	0	0	0	0
ENSMUSG00000106061	GM43773	0	0	1	0	0	2	0	0	0	3	0	5

ENSMUSG00000106077	GM5660	0	0	0	1	0	0	0	1	1	0	1	0
ENSMUSG00000106082	4930404A12F	0	0	0	0	0	1	2	2	1	0	1	0
ENSMUSG00000106086	GM43352	1	0	0	0	0	1	1	2	0	3	1	1
ENSMUSG00000106087	GM43609	0	0	1	1	1	0	2	1	0	4	0	1
ENSMUSG00000106092	GM43072	0	0	0	0	0	0	0	0	1	1	0	0
ENSMUSG00000106093	GM42722	2	1	3	3	2	1	8	5	5	5	6	2
ENSMUSG00000106094	GM18916	1	0	0	0	0	0	0	0	0	0	1	0
ENSMUSG00000106099	GM42664	0	1	0	1	0	0	0	1	0	0	0	2
ENSMUSG00000106103	GM43081	0	1	1	0	0	0	0	0	3	1	0	1
ENSMUSG00000106105	GM4525	4	9	5	3	11	3	16	26	9	21	17	20
ENSMUSG00000106106	CT010467.1	248	1522	345	500	398	385	975	1159	857	1214	809	1172
ENSMUSG00000106108	GM43221	0	0	0	0	0	0	0	0	0	0	1	0
ENSMUSG00000106110	GM43571	0	1	1	3	0	1	1	5	1	5	1	5
ENSMUSG00000106115	GM43420	0	0	1	0	2	0	3	1	1	1	0	3
ENSMUSG00000106135	GM6543	0	0	0	0	0	0	0	0	0	0	0	2
ENSMUSG00000106144	GM43972	0	0	0	0	0	0	0	2	0	0	0	13
ENSMUSG00000106149	GM43430	0	0	0	0	0	0	0	4	3	2	2	1
ENSMUSG00000106153	GM43677	0	0	0	0	0	0	1	1	0	0	0	0
ENSMUSG00000106158	GM43216	0	0	0	0	0	0	1	0	0	0	0	0
ENSMUSG00000106166	GM43484	0	2	1	0	0	0	2	3	2	0	1	2
ENSMUSG00000106169	MIR6948	1	0	0	0	0	0	0	0	0	0	1	0
ENSMUSG00000106175	GM44313	0	0	0	1	0	1	0	0	0	0	0	0
ENSMUSG00000106184	GM43463	0	0	0	0	0	0	0	1	0	0	0	0
ENSMUSG00000106186	GM43627	0	0	0	0	0	1	2	0	0	0	1	0
ENSMUSG00000106189	GM42933	0	0	0	1	0	2	0	0	2	1	1	0
ENSMUSG00000106190	GM20768	0	0	0	0	0	0	0	0	0	0	1	1
ENSMUSG00000106197	GM42717	0	1	0	0	0	0	1	0	0	0	1	2
ENSMUSG00000106198	GM42504	0	0	0	0	0	0	1	0	0	0	0	0
ENSMUSG00000106208	GM4734	0	0	0	0	1	0	0	0	0	1	1	1
ENSMUSG00000106218	GM43713	1	0	0	0	0	1	0	0	0	0	0	0
ENSMUSG00000106219	5830416I19R	1	0	1	0	2	0	1	2	2	1	2	2
ENSMUSG00000106229	GM19409	0	0	1	1	0	0	0	1	0	0	0	0
ENSMUSG00000106230	GM29811	0	0	0	0	0	0	0	0	1	0	0	0
ENSMUSG00000106237	GM8066	0	0	0	0	0	0	0	0	0	0	0	2
ENSMUSG00000106241	GM43143	0	0	0	1	0	0	0	0	0	0	0	1
ENSMUSG00000106243	GM5050	0	0	0	0	0	0	1	1	0	0	0	0
ENSMUSG00000106251	GM42658	0	0	1	0	0	0	0	3	0	2	0	1
ENSMUSG00000106254	GM42907	0	0	0	0	0	0	0	0	0	2	1	0
ENSMUSG00000106258	RPL7A-PS7	16	19	24	25	23	24	56	67	58	68	56	55
ENSMUSG00000106261	GM34086	0	0	0	0	0	0	0	0	0	0	0	1
ENSMUSG00000106262	GM43375	0	0	0	0	0	0	0	1	0	0	0	1
ENSMUSG00000106266	GM43447	0	0	1	0	0	0	0	0	0	1	0	0
ENSMUSG00000106271	GM43752	0	0	0	0	0	0	0	0	0	0	1	0
ENSMUSG00000106281	GM44346	0	0	0	0	0	0	0	0	1	0	0	0
ENSMUSG00000106287	GM43585	0	0	0	0	0	0	1	2	1	1	0	0
ENSMUSG00000106288	GM43593	0	0	0	0	0	0	0	3	1	1	0	0
ENSMUSG00000106290	GM43429	0	0	0	0	0	1	0	2	0	1	2	1
ENSMUSG00000106291	1110003F10F	0	1	0	0	0	0	2	0	0	0	1	1
ENSMUSG00000106296	4632404M16	0	0	0	0	0	0	0	0	0	3	0	0
ENSMUSG00000106306	4933401H06F	0	0	1	0	0	0	0	0	0	0	0	0
ENSMUSG00000106313	GM9402	0	0	0	0	0	0	1	0	0	0	2	1
ENSMUSG00000106314	GM9632	0	0	0	0	0	0	1	0	0	0	0	0
ENSMUSG00000106317	GM43848	3	0	1	2	0	3	6	5	1	5	3	2
ENSMUSG00000106333	4930428O21F	0	1	0	0	0	0	0	1	0	1	1	1
ENSMUSG00000106334	GM43549	0	0	0	0	0	0	0	0	0	1	0	0
ENSMUSG00000106341	GM43330	0	0	0	0	2	0	3	0	1	0	0	0
ENSMUSG00000106357	GM8013	0	0	0	0	0	0	4	1	1	1	1	1
ENSMUSG00000106375	GM43361	1	1	2	5	1	1	3	4	5	2	7	5

ENSMUSG00000106379	LHFPL3	2	1	2	2	0	1	8	5	1	12	7	2
ENSMUSG00000106383	E330034L11R	3	2	2	3	5	6	12	5	5	11	5	6
ENSMUSG00000106388	RHOX7-PS2	7	5	12	12	8	6	26	42	36	36	24	27
ENSMUSG00000106391	GM42690	0	0	0	0	0	0	0	0	1	0	0	0
ENSMUSG00000106392	GM5870	0	0	1	0	0	0	0	1	0	1	0	0
ENSMUSG00000106406	MIR7682	0	0	0	0	0	0	0	0	0	1	0	0
ENSMUSG00000106415	GM42893	2	2	3	5	2	4	11	9	11	5	9	14
ENSMUSG00000106416	GM5857	0	0	0	0	0	1	1	0	2	4	0	1
ENSMUSG00000106420	GM9245	0	0	0	0	0	0	0	0	0	0	1	0
ENSMUSG00000106421	GM9645	0	0	0	0	0	0	0	1	0	1	0	0
ENSMUSG00000106427	GM42820	0	2	0	0	0	1	1	3	0	1	0	1
ENSMUSG00000106431	GM44492	0	0	0	0	0	0	0	1	0	0	0	0
ENSMUSG00000106434	GM42990	0	0	0	0	0	0	0	1	0	0	0	0
ENSMUSG00000106437	GM35667	0	0	0	0	0	0	0	0	1	0	0	0
ENSMUSG00000106441	GM42921	0	0	0	0	1	0	1	3	0	2	1	1
ENSMUSG00000106443	GM42702	0	0	1	0	0	0	0	0	0	0	0	0
ENSMUSG00000106447	GM42957	0	0	0	1	0	0	0	0	0	0	0	0
ENSMUSG00000106457	GM42585	1	0	0	1	0	0	0	0	2	1	2	0
ENSMUSG00000106458	GM42633	0	0	0	0	0	0	1	1	0	0	0	0
ENSMUSG00000106464	C130083M11	0	1	0	0	0	0	0	0	0	0	1	0
ENSMUSG00000106475	GM43011	0	3	2	1	2	0	4	1	5	4	2	5
ENSMUSG00000106476	RPL36-PS9	0	0	1	1	1	2	3	2	3	3	8	4
ENSMUSG00000106478	GM36551	0	0	0	0	1	0	0	0	0	1	0	0
ENSMUSG00000106496	GM7733	0	0	0	0	0	0	0	0	2	0	0	0
ENSMUSG00000106499	GM9403	0	0	0	0	0	0	1	0	0	1	0	0
ENSMUSG00000106501	GM42711	0	0	1	0	0	0	0	0	0	0	0	0
ENSMUSG00000106511	GM43521	0	1	0	0	0	0	0	0	0	0	0	0
ENSMUSG00000106518	GM42759	2	0	0	0	0	1	1	1	3	1	1	2
ENSMUSG00000106534	GM6366	0	0	0	1	0	0	0	0	0	0	0	0
ENSMUSG00000106535	GM43031	1	0	0	0	0	2	1	2	0	2	3	4
ENSMUSG00000106536	GM42508	0	0	0	0	0	1	0	0	1	0	1	0
ENSMUSG00000106539	GM43670	1	0	1	0	0	0	1	0	1	4	1	0
ENSMUSG00000106547	B230303O12I	1	0	0	0	2	0	4	8	0	6	6	2
ENSMUSG00000106549	GM42653	0	0	0	0	0	0	0	0	0	0	0	1
ENSMUSG00000106550	GM43646	0	0	1	0	1	1	2	2	1	0	0	1
ENSMUSG00000106562	GM5981	0	0	0	0	0	0	2	0	0	0	0	0
ENSMUSG00000106565	GM43582	0	0	0	0	1	0	1	4	0	1	0	1
ENSMUSG00000106566	GM43836	0	0	0	0	0	0	0	0	0	0	0	1
ENSMUSG00000106568	GM42814	2	2	4	5	3	6	13	15	5	8	8	9
ENSMUSG00000106572	4933417G07I	0	0	0	0	0	0	0	0	0	0	1	0
ENSMUSG00000106575	GM42613	0	0	0	0	1	0	0	1	0	0	0	0
ENSMUSG00000106576	GM4866	0	0	0	1	0	0	0	0	0	0	0	0
ENSMUSG00000106596	GM42822	0	0	0	0	0	0	1	1	1	0	0	0
ENSMUSG00000106599	IGHV1-73	0	0	0	0	0	0	1	1	0	1	0	1
ENSMUSG00000106604	GM42535	0	0	0	1	0	1	0	0	0	0	0	0
ENSMUSG00000106607	GM43178	1	0	0	0	1	0	0	0	1	0	3	1
ENSMUSG00000106609	GM43181	0	0	0	0	0	0	1	0	0	0	0	0
ENSMUSG00000106610	GM43103	0	0	0	0	0	0	0	0	0	1	0	0
ENSMUSG00000106617	GM36266	0	0	0	3	0	1	2	5	0	2	1	2
ENSMUSG00000106621	GM42752	1	0	1	1	1	0	2	4	1	3	2	5
ENSMUSG00000106623	GM43335	0	0	0	1	0	0	1	0	0	0	0	1
ENSMUSG00000106628	GM43558	0	0	1	0	0	0	2	1	0	1	0	0
ENSMUSG00000106631	GM42669	0	0	0	1	0	0	0	0	1	0	0	0
ENSMUSG00000106636	GM43813	0	3	4	2	5	1	9	11	5	14	6	4
ENSMUSG00000106637	GM42455	0	0	0	0	0	0	1	0	0	0	0	0
ENSMUSG00000106643	GM43422	0	0	0	0	0	0	1	0	0	0	0	0
ENSMUSG00000106646	GM42483	1	0	0	0	0	0	0	0	0	0	0	0
ENSMUSG00000106648	GM43176	0	0	0	2	0	0	1	1	1	1	0	1

ENSMUSG00000106662	GM43034	0	0	0	0	1	0	0	0	0	0	0	1
ENSMUSG00000106665	GM43389	0	0	0	0	0	0	0	0	0	1	0	0
ENSMUSG00000106674	GM20005	1	0	0	0	0	0	0	0	0	0	0	0
ENSMUSG00000106676	GM42895	2	5	2	1	0	1	4	1	1	5	3	2
ENSMUSG00000106678	GM43457	0	0	0	0	1	0	2	1	2	1	1	2
ENSMUSG00000106682	GM42648	0	1	0	1	0	1	5	1	2	3	2	2
ENSMUSG00000106696	GM42729	3	1	1	0	1	0	1	2	4	3	0	2
ENSMUSG00000106698	GM43599	1	0	0	0	0	0	2	1	1	4	0	0
ENSMUSG00000106702	GM42846	0	1	1	3	1	1	1	1	2	2	3	3
ENSMUSG00000106703	VMN2R-PS26	0	0	0	0	0	0	0	0	0	1	0	0
ENSMUSG00000106706	C530043K16F	4	5	0	3	8	4	6	8	7	9	5	10
ENSMUSG00000106707	GM43748	0	0	0	0	0	0	0	1	1	2	0	0
ENSMUSG00000106717	GM42798	5	1	4	7	3	5	12	10	7	9	9	7
ENSMUSG00000106720	GM43795	0	0	0	0	0	0	2	0	0	0	0	0
ENSMUSG00000106725	GM43363	0	0	0	0	0	0	0	1	0	0	0	0
ENSMUSG00000106732	GM43792	0	1	0	1	0	0	0	2	2	0	1	0
ENSMUSG00000106734	GM20559	8	6	7	8	11	8	31	36	20	26	21	12
ENSMUSG00000106736	GM42573	0	1	0	0	0	0	0	0	0	0	0	0
ENSMUSG00000106739	GM43499	0	0	0	0	0	0	0	0	0	0	0	1
ENSMUSG00000106740	GM21221	1	0	0	0	0	0	0	1	0	1	0	0
ENSMUSG00000106743	GM42847	0	0	0	0	0	0	0	0	1	1	0	0
ENSMUSG00000106751	GM42467	1	2	2	3	0	1	4	2	1	3	2	3
ENSMUSG00000106755	TPI-RS11	0	0	0	0	1	0	0	0	0	0	0	0
ENSMUSG00000106757	GM43482	0	1	0	0	1	0	2	1	0	3	1	2
ENSMUSG00000106758	GM42731	0	1	0	0	1	0	0	0	0	0	0	0
ENSMUSG00000106759	AC120011.1	0	0	0	0	0	0	0	1	0	1	0	0
ENSMUSG00000106764	GM42529	0	1	0	0	0	0	0	0	1	0	0	1
ENSMUSG00000106767	GM42727	6	13	8	9	13	11	37	31	24	39	20	19
ENSMUSG00000106772	GM43794	1	1	0	0	1	0	2	3	0	0	0	0
ENSMUSG00000106775	C130093G08I	0	0	0	0	0	0	0	0	0	0	1	0
ENSMUSG00000106777	GM43150	0	0	0	0	0	0	0	0	0	0	0	1
ENSMUSG00000106798	GM43275	0	0	0	0	0	0	0	2	1	0	1	1
ENSMUSG00000106801	GM42852	0	0	0	0	0	0	0	0	0	0	0	1
ENSMUSG00000106803	GM8930	0	0	0	0	0	0	0	1	0	0	0	0
ENSMUSG00000106807	GM10441	0	0	0	0	1	0	2	0	0	0	0	0
ENSMUSG00000106815	GM43658	0	0	0	0	0	0	0	1	0	0	0	1
ENSMUSG00000106820	D5ERTD605E	0	0	1	0	0	0	0	1	0	1	1	0
ENSMUSG00000106822	GM43533	0	0	0	0	0	0	0	0	0	1	0	1
ENSMUSG00000106825	2510016D11F	0	0	0	0	0	0	0	0	0	0	1	0
ENSMUSG00000106834	GM10459	0	0	0	1	0	0	0	3	0	0	2	2
ENSMUSG00000106840	GM42790	0	0	0	0	0	0	1	0	0	2	0	0
ENSMUSG00000106845	GM43156	0	0	0	0	0	0	0	0	0	0	1	0
ENSMUSG00000106847	PEG13	0	0	0	0	0	0	0	1	0	0	0	0
ENSMUSG00000106856	GM43761	0	0	0	0	0	0	2	1	1	2	3	0
ENSMUSG00000106858	GM43844	0	0	0	0	0	0	0	1	1	0	0	0
ENSMUSG00000106862	GM42811	0	0	0	0	0	0	0	1	0	0	0	0
ENSMUSG00000106864	GTF3C2	27	32	26	27	34	28	108	112	100	129	92	104
ENSMUSG00000106868	GM19798	0	0	0	0	0	0	0	1	0	0	0	0
ENSMUSG00000106869	GM42473	0	0	0	0	0	0	0	1	0	0	0	0
ENSMUSG00000106870	GM43681	0	1	0	0	0	0	2	2	0	2	1	0
ENSMUSG00000106874	GM20186	2	1	0	0	1	1	0	0	0	0	0	0
ENSMUSG00000106883	GM34091	0	0	0	0	0	0	0	0	0	0	1	1
ENSMUSG00000106891	GM43460	1	0	0	0	1	0	1	0	1	1	1	0
ENSMUSG00000106894	GM17130	0	0	0	0	0	0	0	0	0	0	1	0
ENSMUSG00000106895	GM4754	1	2	5	0	1	2	8	8	10	7	9	6
ENSMUSG00000106898	GM43125	1	0	0	0	1	0	0	0	0	0	0	0
ENSMUSG00000106902	4930557J02R	0	0	1	0	0	0	0	0	0	1	0	0
ENSMUSG00000106904	GM43029	0	0	0	0	0	0	0	0	1	1	0	2

ENSMUSG00000106915	GM42655	0	0	0	0	0	0	0	0	0	0	0	1
ENSMUSG00000106918	MRPL33	31	34	30	46	39	39	283	253	188	222	188	217
ENSMUSG00000106925	GM20072	1	0	0	0	0	0	0	0	0	0	0	0
ENSMUSG00000106926	RPL7-PS7	0	0	0	1	0	0	1	2	1	1	0	2
ENSMUSG00000106927	GM43598	0	0	0	0	0	0	2	1	2	0	0	1
ENSMUSG00000106928	GM43860	0	1	0	0	0	0	1	0	0	0	2	0
ENSMUSG00000106930	GM6450	0	0	0	0	0	0	1	0	0	0	0	0
ENSMUSG00000106940	GM42930	0	0	1	0	0	0	1	2	0	0	1	1
ENSMUSG00000106943	DANCR	4	7	7	3	4	1	31	41	22	39	38	26
ENSMUSG00000106944	GM43843	0	0	0	0	0	0	0	1	0	0	1	1
ENSMUSG00000106946	GM42856	0	2	1	0	0	1	0	1	0	1	0	1
ENSMUSG00000106959	GM42548	0	2	3	0	0	2	3	2	3	6	3	2
ENSMUSG00000106962	GM43633	0	2	0	0	0	0	1	2	0	2	2	0
ENSMUSG00000106967	GM42477	0	0	1	0	0	0	0	0	0	0	0	1
ENSMUSG00000106973	GM43415	0	0	0	0	0	0	0	1	0	0	0	0
ENSMUSG00000106978	N4BP2OS	1	0	3	0	2	0	0	2	2	1	0	1
ENSMUSG00000106981	GM45495	1	0	0	0	1	0	1	1	0	0	0	2
ENSMUSG00000106990	GM42547	1	2	1	1	2	6	15	13	10	18	6	13
ENSMUSG00000106992	GM43167	1	2	1	0	1	0	3	0	2	2	3	1
ENSMUSG00000106993	GM43417	0	0	0	0	0	0	1	0	0	0	1	0
ENSMUSG00000107000	GM43481	0	5	1	0	2	1	3	7	4	7	3	3
ENSMUSG00000107002	0610012G03I	3	5	2	7	5	6	26	32	25	21	19	26
ENSMUSG00000107009	6720475M21	0	0	0	0	0	0	1	1	0	4	0	2
ENSMUSG00000107012	4930528J11R	1	1	1	0	2	4	8	4	7	7	4	12
ENSMUSG00000107015	2900076G11I	1	1	0	1	0	0	1	0	1	0	0	0
ENSMUSG00000107017	GM43196	1	0	0	0	0	0	0	1	1	0	1	1
ENSMUSG00000107019	GM43682	0	0	2	0	0	0	0	0	3	1	0	0
ENSMUSG00000107023	GM42715	6	10	4	7	7	10	16	22	17	26	12	9
ENSMUSG00000107026	GM7504	0	0	0	0	0	0	1	0	0	0	0	0
ENSMUSG00000107029	GM43123	0	2	0	0	0	0	0	0	0	0	0	0
ENSMUSG00000107035	YBX1-PS2	0	0	0	0	0	0	1	1	0	1	0	2
ENSMUSG00000107041	GM42735	0	0	0	0	1	0	0	1	0	0	0	2
ENSMUSG00000107043	GM42849	0	0	0	0	0	0	0	0	0	0	0	1
ENSMUSG00000107048	GM43807	0	0	0	0	0	0	0	1	1	0	0	1
ENSMUSG00000107050	C030017G13I	1	0	2	1	0	3	3	1	4	1	1	0
ENSMUSG00000107054	C730045M19	1	0	3	0	0	1	2	4	2	5	7	3
ENSMUSG00000107061	GM19590	0	0	0	0	1	0	1	2	0	1	1	0
ENSMUSG00000107076	GM43480	1	0	0	0	0	0	0	0	0	0	0	2
ENSMUSG00000107083	GM43313	0	0	0	1	0	0	0	0	0	0	0	1
ENSMUSG00000107085	GM42883	0	0	0	0	0	0	0	0	0	0	0	2
ENSMUSG00000107091	GM43343	0	0	0	0	1	0	0	4	2	2	0	3
ENSMUSG00000107093	GM43452	0	0	0	0	0	0	0	0	1	0	0	0
ENSMUSG00000107094	GM43294	0	0	0	0	0	0	0	1	0	0	0	1
ENSMUSG00000107096	GM43597	3	0	2	0	2	3	7	5	7	6	6	10
ENSMUSG00000107102	GM42726	9	8	7	10	7	12	17	26	14	14	15	14
ENSMUSG00000107106	GM43760	0	1	0	0	0	0	0	0	0	0	0	0
ENSMUSG00000107109	GM42571	0	0	0	0	0	0	1	0	2	2	1	1
ENSMUSG00000107111	GM40304	0	0	0	0	0	0	0	1	0	1	0	0
ENSMUSG00000107118	GM42986	0	2	1	1	0	1	3	6	1	7	0	2
ENSMUSG00000107120	GM43059	0	0	0	0	1	0	0	0	2	2	1	1
ENSMUSG00000107121	1110006O24I	0	0	0	0	2	1	3	4	0	2	2	3
ENSMUSG00000107142	4930566F21F	0	0	0	0	0	0	1	0	0	0	0	0
ENSMUSG00000107143	GM6598	0	0	1	1	1	0	4	1	2	1	3	1
ENSMUSG00000107151	GM43810	0	0	0	0	0	0	2	0	0	0	1	0
ENSMUSG00000107152	GM43152	0	1	0	0	0	0	0	0	0	0	0	0
ENSMUSG00000107154	GM7041	1	0	0	0	0	0	0	0	0	0	0	0
ENSMUSG00000107155	GM43793	0	0	0	0	0	0	1	0	0	0	0	0
ENSMUSG00000107158	GM42728	2	0	0	2	0	0	2	2	0	0	0	3

ENSMUSG00000107160	GM42666	27	54	45	50	56	43	135	156	112	140	109	151
ENSMUSG00000107163	GM43496	0	0	0	0	0	0	0	1	0	0	0	0
ENSMUSG00000107165	GM43747	1	0	0	0	2	0	3	1	0	1	0	0
ENSMUSG00000107167	GM34653	0	0	0	0	0	0	0	0	0	0	0	1
ENSMUSG00000107168	GM42507	0	0	0	0	0	0	0	0	0	0	0	1
ENSMUSG00000107171	GM42572	0	0	0	0	0	0	0	1	0	0	2	0
ENSMUSG00000107173	GM43266	0	0	0	0	0	0	0	1	0	0	0	0
ENSMUSG00000107176	GM9794	23	32	33	29	34	36	113	119	96	139	69	89
ENSMUSG00000107178	GM42531	0	0	0	0	0	0	1	0	0	0	0	2
ENSMUSG00000107179	GM43069	0	0	0	0	0	0	0	0	0	1	1	1
ENSMUSG00000107182	GM43268	0	0	0	2	0	1	3	1	1	0	0	3
ENSMUSG00000107185	GM42642	0	0	0	0	0	0	0	0	0	0	1	0
ENSMUSG00000107191	GM43579	3	7	3	2	2	3	13	10	9	11	7	9
ENSMUSG00000107195	4930589O11I	0	0	1	0	0	1	0	1	0	2	0	0
ENSMUSG00000107197	GM43312	0	0	0	0	0	0	0	0	0	1	1	0
ENSMUSG00000107201	5930420M18	0	2	0	0	0	0	0	2	0	0	0	3
ENSMUSG00000107203	GM7542	0	0	0	0	0	0	0	0	1	0	0	0
ENSMUSG00000107205	GM42576	0	0	3	1	0	1	2	4	1	2	2	2
ENSMUSG00000107209	GM42599	0	0	0	1	0	0	0	1	1	0	0	1
ENSMUSG00000107212	GM5302	0	0	0	0	0	0	0	0	0	2	0	0
ENSMUSG00000107215	GM43197	0	1	0	0	0	0	1	0	0	2	0	1
ENSMUSG00000107216	2210412B16F	0	0	0	1	0	0	1	0	0	2	0	0
ENSMUSG00000107222	GM43198	0	0	0	0	0	0	0	1	0	0	0	0
ENSMUSG00000107225	GM43637	0	1	1	0	3	1	0	1	1	0	1	0
ENSMUSG00000107227	GM42559	0	1	0	1	0	1	1	1	1	2	0	2
ENSMUSG00000107230	GM19265	0	0	0	0	0	0	0	0	0	1	1	0
ENSMUSG00000107233	GM42959	0	0	0	0	0	0	1	0	1	0	0	2
ENSMUSG00000107238	GM42758	0	0	0	0	0	0	0	0	0	0	1	0
ENSMUSG00000107246	GM42560	0	0	0	0	0	0	0	1	0	0	0	0
ENSMUSG00000107254	GM43769	0	0	0	0	1	1	1	3	0	0	0	0
ENSMUSG00000107255	GM43416	0	0	0	0	0	0	0	0	1	0	0	0
ENSMUSG00000107257	GM43028	0	0	0	0	0	0	0	1	0	0	0	0
ENSMUSG00000107263	GM42471	0	0	0	0	0	0	1	0	0	0	0	0
ENSMUSG00000107278	GM42600	0	0	0	1	0	0	3	1	0	1	0	0
ENSMUSG00000107280	GM9495	0	0	0	0	0	1	0	0	0	0	0	0
ENSMUSG00000107283	MPV17	0	1	4	3	3	2	21	22	21	16	17	26
ENSMUSG00000107286	GM43788	1	0	1	1	2	1	4	1	0	2	2	0
ENSMUSG00000107288	GM42903	0	0	0	0	0	0	0	1	0	0	0	0
ENSMUSG00000107292	GM42733	0	0	0	0	0	0	0	0	0	0	0	1
ENSMUSG00000107304	GM43775	0	0	0	0	0	0	0	0	0	1	0	0
ENSMUSG00000107306	GM42577	0	0	0	0	0	0	0	0	0	1	1	1
ENSMUSG00000107313	GM43332	0	0	0	0	1	0	0	0	0	2	0	0
ENSMUSG00000107319	GM42670	0	1	0	0	0	0	1	1	0	0	0	0
ENSMUSG00000107320	GM42549	0	2	1	0	1	1	4	5	2	6	2	1
ENSMUSG00000107325	GM43425	0	0	0	0	1	0	0	0	0	0	0	0
ENSMUSG00000107331	GM42732	0	0	0	0	0	0	1	3	2	8	2	4
ENSMUSG00000107335	GM43372	0	0	0	0	0	0	0	0	1	0	0	0
ENSMUSG00000107336	GM43461	0	0	0	0	0	0	0	0	0	1	0	0
ENSMUSG00000107349	GM6655	0	0	0	0	0	0	0	0	0	1	0	0
ENSMUSG00000107352	GM43660	0	3	1	2	0	1	5	2	2	3	1	4
ENSMUSG00000107358	GM43168	0	0	0	0	0	1	0	0	0	1	0	0
ENSMUSG00000107362	GM40309	1	5	5	2	4	2	8	11	8	9	4	4
ENSMUSG00000107364	GM43051	0	1	0	0	0	0	0	0	0	1	0	0
ENSMUSG00000107368	1700018A23F	0	0	1	0	1	0	2	0	0	1	4	0
ENSMUSG00000107370	GM43588	0	0	1	0	0	0	1	1	0	0	0	0
ENSMUSG00000107374	GM43172	0	0	0	0	0	0	0	0	1	0	0	0
ENSMUSG00000107379	GM43126	1	1	2	3	2	2	7	7	2	10	5	9
ENSMUSG00000107383	GM4366	0	2	0	0	0	0	5	1	2	1	2	4

ENSMUSG00000107389	GM43124	0	0	0	0	0	0	0	0	0	0	1	0
ENSMUSG00000107402	4732416N19R	0	0	0	0	1	0	2	1	1	2	1	0
ENSMUSG00000107406	1700040L08R	0	0	0	0	1	1	0	0	0	0	0	1
ENSMUSG00000107411	GM19040	1	0	0	0	0	1	0	1	0	1	0	1
ENSMUSG00000107414	6330419E04R	0	0	0	0	0	0	3	1	1	2	1	2
ENSMUSG00000107429	GM44206	0	0	0	0	0	0	2	2	2	0	0	1
ENSMUSG00000107433	GM43882	0	1	1	0	1	0	2	5	2	4	0	1
ENSMUSG00000107443	GM7468	0	0	0	0	0	0	0	0	0	0	1	0
ENSMUSG00000107454	GM43875	0	0	0	0	0	1	2	1	1	0	1	1
ENSMUSG00000107455	GM43868	0	0	0	1	0	0	0	0	0	0	0	1
ENSMUSG00000107460	GM44186	0	0	0	0	0	0	0	2	0	1	0	1
ENSMUSG00000107461	GM44045	0	0	0	1	0	0	0	1	1	2	0	0
ENSMUSG00000107468	5730507A11R	0	0	0	0	0	0	1	1	0	2	0	1
ENSMUSG00000107470	GM3375	0	0	0	0	0	0	0	0	0	1	0	0
ENSMUSG00000107472	GM44265	0	0	0	0	0	1	0	1	0	0	0	0
ENSMUSG00000107476	ZFP862-PS	0	1	0	0	1	0	2	5	2	4	6	4
ENSMUSG00000107477	GM44366	0	0	0	0	0	0	0	0	0	0	0	1
ENSMUSG00000107479	2610300M13	0	0	0	0	0	0	0	0	0	1	0	0
ENSMUSG00000107481	4833403J16R	2	5	0	2	3	3	8	9	3	10	6	7
ENSMUSG00000107495	GM44215	0	0	0	1	0	0	0	0	0	0	0	0
ENSMUSG00000107500	GM2796	0	0	0	0	1	0	1	0	2	0	2	0
ENSMUSG00000107502	GM44065	1	0	0	0	0	0	1	0	0	0	0	0
ENSMUSG00000107509	GM44941	0	1	0	0	0	0	0	0	0	0	0	0
ENSMUSG00000107511	GM44440	0	0	0	0	0	0	1	0	0	0	0	0
ENSMUSG00000107515	GM43886	0	1	0	1	1	0	2	1	0	0	1	3
ENSMUSG00000107524	GM44136	1	0	0	0	0	0	3	0	2	0	0	0
ENSMUSG00000107529	GM44291	2	1	3	8	5	2	3	12	5	5	10	7
ENSMUSG00000107531	E330037G11R	0	1	2	6	1	0	2	1	1	4	3	2
ENSMUSG00000107534	GM43920	1	3	4	2	0	2	7	12	4	7	6	7
ENSMUSG00000107542	GM43969	0	0	0	0	0	1	0	0	0	0	0	0
ENSMUSG00000107549	GM43961	0	0	0	0	0	0	0	1	0	0	1	0
ENSMUSG00000107556	GM44237	0	0	0	0	0	0	1	0	0	0	0	0
ENSMUSG00000107560	GM44199	0	0	0	0	0	0	1	0	0	1	0	1
ENSMUSG00000107561	GM44264	0	0	0	0	0	0	0	0	0	1	0	1
ENSMUSG00000107574	GM17834	0	0	0	0	0	0	0	1	0	0	0	0
ENSMUSG00000107576	GM44093	2	1	0	2	0	1	7	3	0	0	1	1
ENSMUSG00000107577	GM44103	1	3	3	3	3	2	7	9	4	14	7	4
ENSMUSG00000107586	GM44283	0	0	0	2	0	0	0	1	2	4	1	1
ENSMUSG00000107591	GM6794	0	0	0	0	0	0	1	3	2	1	0	5
ENSMUSG00000107595	GM44441	0	0	0	0	0	0	0	0	0	1	0	0
ENSMUSG00000107596	GM18913	0	1	0	0	0	0	2	0	0	0	0	1
ENSMUSG00000107597	GM44764	0	0	0	0	1	0	1	1	1	0	0	0
ENSMUSG00000107604	GM44041	1	0	1	3	1	1	4	3	1	3	4	4
ENSMUSG00000107608	GM43863	0	0	0	0	0	1	0	1	0	0	1	1
ENSMUSG00000107610	D530018E20R	0	1	0	1	0	0	3	3	5	4	1	3
ENSMUSG00000107639	GM4374	0	0	0	0	0	0	0	0	1	0	0	0
ENSMUSG00000107659	GM44170	6	9	3	6	5	4	22	19	16	31	22	28
ENSMUSG00000107676	GM44178	0	0	0	0	0	0	1	0	0	2	0	0
ENSMUSG00000107681	4930528H21R	0	0	0	0	0	0	0	0	1	0	0	0
ENSMUSG00000107688	GM44091	0	0	1	0	0	0	0	2	0	5	1	0
ENSMUSG00000107689	GM44386	0	0	0	0	0	0	0	0	1	0	0	0
ENSMUSG00000107690	GM44044	0	0	2	2	2	1	3	3	1	6	3	3
ENSMUSG00000107698	GM44430	0	0	0	0	0	0	0	0	0	1	0	0
ENSMUSG00000107701	GM19287	0	0	0	0	0	0	1	0	0	2	0	0
ENSMUSG00000107713	GM6681	0	0	3	0	0	0	2	0	0	2	3	1
ENSMUSG00000107721	GM44261	3	2	2	1	2	3	14	13	4	6	10	8
ENSMUSG00000107722	2900060B14R	0	0	1	1	0	0	1	1	1	3	0	1
ENSMUSG00000107724	GM16042	0	0	0	0	0	0	0	1	0	0	0	0

ENSMUSG00000107726	GM44037	0	0	0	0	0	0	0	2	0	0	0	0
ENSMUSG00000107730	GM44238	1	1	0	2	0	1	4	4	2	3	2	2
ENSMUSG00000107734	GM30055	0	0	0	3	0	0	0	2	1	0	0	0
ENSMUSG00000107741	GM2011	2	0	0	0	1	0	7	2	0	0	2	6
ENSMUSG00000107742	9530085L11R	0	0	0	0	1	2	2	0	1	2	0	1
ENSMUSG00000107749	GM44321	1	0	0	0	0	0	1	0	1	1	0	1
ENSMUSG00000107750	GM44013	0	0	0	0	0	0	0	0	1	0	1	0
ENSMUSG00000107753	GM44371	0	1	0	0	0	0	1	0	0	0	0	0
ENSMUSG00000107756	GM44164	1	3	0	0	1	0	1	3	2	3	1	3
ENSMUSG00000107760	GM44401	0	0	0	0	0	0	1	0	0	0	0	0
ENSMUSG00000107761	2010008C14R	0	0	0	1	0	0	3	3	1	4	2	0
ENSMUSG00000107785	GM45083	0	1	3	1	1	4	10	4	4	3	5	2
ENSMUSG00000107788	GM6266	0	1	0	1	1	0	0	1	1	0	1	1
ENSMUSG00000107798	GM44064	0	0	0	0	0	0	0	0	1	0	0	0
ENSMUSG00000107799	GM38825	0	0	0	0	0	0	0	0	1	0	0	0
ENSMUSG00000107802	1700126G02R	0	0	0	0	0	0	0	0	1	0	0	0
ENSMUSG00000107804	GM44439	0	1	0	0	0	0	3	1	0	3	1	1
ENSMUSG00000107806	GM19726	0	0	0	0	0	0	0	0	0	0	0	1
ENSMUSG00000107813	GM44434	0	1	0	0	0	0	0	0	0	3	0	0
ENSMUSG00000107823	GM44436	0	0	0	0	0	0	0	0	1	0	0	0
ENSMUSG00000107838	GM45769	0	0	0	0	0	0	1	0	1	1	0	0
ENSMUSG00000107841	GM44185	0	0	0	0	0	0	0	1	0	1	0	0
ENSMUSG00000107865	GM44417	0	0	0	0	0	0	1	0	1	0	0	0
ENSMUSG00000107868	GM5112	0	0	0	0	0	0	0	1	0	0	1	0
ENSMUSG00000107869	GM29848	0	0	0	0	0	0	0	0	1	0	0	0
ENSMUSG00000107881	GM44250	0	0	0	1	0	1	1	0	0	0	1	0
ENSMUSG00000107884	GM44144	0	1	0	0	0	2	3	1	5	3	2	7
ENSMUSG00000107894	GM10388	0	1	0	0	0	0	0	1	0	2	1	0
ENSMUSG00000107896	GM8719	0	0	0	0	0	0	1	0	0	0	0	0
ENSMUSG00000107909	2610017A05R	0	1	1	1	0	0	0	0	1	3	0	2
ENSMUSG00000107911	GM7565	0	0	0	0	0	0	0	0	2	3	1	0
ENSMUSG00000107915	GM44014	0	0	0	0	0	0	0	0	1	0	0	0
ENSMUSG00000107920	GM7783	0	0	0	0	0	1	1	0	0	0	0	2
ENSMUSG00000107927	GM44090	0	8	4	5	6	6	4	10	5	10	6	6
ENSMUSG00000107932	GM44432	1	0	1	1	0	0	2	4	1	6	0	1
ENSMUSG00000107944	GM44280	0	0	0	0	0	1	0	0	2	1	0	0
ENSMUSG00000107951	GM6210	0	0	0	0	0	0	1	0	3	1	2	0
ENSMUSG00000107954	GM43942	0	0	0	1	0	0	0	0	1	1	1	0
ENSMUSG00000107955	GM44027	0	1	0	0	0	0	2	0	0	1	1	2
ENSMUSG00000107962	GM43980	0	0	0	4	0	1	2	0	0	3	1	1
ENSMUSG00000107968	GM44419	0	0	0	0	0	0	0	1	0	0	0	0
ENSMUSG00000107971	GM44260	0	0	0	0	1	0	0	0	0	0	0	0
ENSMUSG00000107976	GM44043	0	0	0	0	0	0	2	0	0	0	0	0
ENSMUSG00000107990	GM7890	0	1	0	0	1	0	3	3	1	4	1	1
ENSMUSG00000107991	GM43919	0	0	0	0	0	0	1	1	1	0	0	1
ENSMUSG00000107994	D830050J10R	0	0	0	0	0	0	1	0	0	1	0	0
ENSMUSG00000107996	GM44228	0	0	0	0	0	0	0	0	1	0	0	0
ENSMUSG00000108005	GM44435	0	1	0	0	0	0	2	0	1	0	0	0
ENSMUSG00000108008	GM43869	0	1	0	0	0	0	0	0	0	1	0	0
ENSMUSG00000108015	GM32591	0	0	0	0	0	0	0	0	1	0	0	0
ENSMUSG00000108030	9530062K07R	0	0	0	0	0	0	4	0	1	0	1	2
ENSMUSG00000108037	GM44597	0	0	0	0	0	0	0	0	0	2	0	0
ENSMUSG00000108042	B130021K23R	1	0	0	0	1	1	2	4	6	11	4	2
ENSMUSG00000108053	GM43890	1	0	0	0	0	0	0	0	0	0	0	3
ENSMUSG00000108059	GM44369	0	1	0	0	0	0	0	0	0	0	0	0
ENSMUSG00000108064	GM44423	0	0	0	0	2	0	0	0	0	0	0	0
ENSMUSG00000108084	GM7932	0	0	0	0	0	0	0	1	0	0	0	0
ENSMUSG00000108089	GM3148	0	0	0	0	0	0	0	0	0	0	0	1

ENSMUSG00000108094	GM31108	0	0	0	1	0	0	1	0	0	0	0	0
ENSMUSG00000108095	GM44067	1	1	2	5	1	0	3	8	4	6	7	9
ENSMUSG00000108101	GM45738	0	0	1	0	0	0	1	0	0	0	0	0
ENSMUSG00000108107	GM5312	0	0	0	0	1	0	1	1	1	1	2	3
ENSMUSG00000108108	GM44270	0	0	0	0	1	0	0	1	0	0	0	0
ENSMUSG00000108109	GM44287	0	0	0	0	0	0	0	0	0	1	0	0
ENSMUSG00000108120	9930120I10R	0	0	0	0	0	0	1	3	0	1	0	0
ENSMUSG00000108122	GM44116	0	0	1	1	0	0	3	0	0	1	0	0
ENSMUSG00000108123	GM43884	0	2	1	0	0	0	1	2	1	0	1	2
ENSMUSG00000108129	4930417O13I	0	0	0	0	0	0	1	0	0	0	0	0
ENSMUSG00000108132	GM44175	0	1	0	0	0	0	0	1	0	0	0	1
ENSMUSG00000108137	GM44053	0	0	0	0	0	0	0	0	0	1	0	1
ENSMUSG00000108148	GM44020	0	0	0	0	1	0	0	0	0	0	0	0
ENSMUSG00000108152	GM44152	1	4	0	0	1	1	3	8	9	7	0	5
ENSMUSG00000108155	GM44443	0	0	0	0	0	0	0	1	0	0	0	1
ENSMUSG00000108170	GM43966	0	0	1	0	0	1	0	0	1	0	1	1
ENSMUSG00000108173	GM44231	0	0	0	0	0	1	0	0	0	1	0	0
ENSMUSG00000108176	GM43965	0	0	0	1	0	0	0	0	0	0	3	1
ENSMUSG00000108177	GM7924	0	0	0	0	0	0	0	1	0	0	0	0
ENSMUSG00000108181	C030015A19I	0	0	0	0	0	0	0	3	4	3	2	1
ENSMUSG00000108195	GM44415	0	0	0	0	0	0	0	1	0	0	0	0
ENSMUSG00000108197	GM44214	0	0	0	0	0	0	0	1	0	0	0	0
ENSMUSG00000108199	GM44249	0	0	0	0	0	0	0	0	0	1	0	0
ENSMUSG00000108200	GM43931	0	0	0	0	0	0	0	0	0	0	2	0
ENSMUSG00000108207	1810059H22I	0	0	0	0	0	0	1	1	0	1	1	0
ENSMUSG00000108211	GM44130	0	0	0	0	0	0	0	0	0	2	0	1
ENSMUSG00000108213	GM44172	0	0	0	0	0	0	0	1	0	1	0	0
ENSMUSG00000108216	GM44153	0	1	0	0	1	0	3	6	3	1	1	3
ENSMUSG00000108217	GM43883	2	0	0	2	0	0	1	0	0	0	0	1
ENSMUSG00000108218	OLFR1372-PS	0	0	0	0	0	0	1	0	1	2	0	0
ENSMUSG00000108219	GM44101	1	2	0	0	0	1	1	0	4	2	1	1
ENSMUSG00000108227	4933412L11R	0	0	0	1	0	0	0	0	0	0	0	0
ENSMUSG00000108231	GM4045	1	0	0	0	0	1	3	1	2	6	2	5
ENSMUSG00000108236	0610033M10	0	0	0	0	0	0	0	0	0	0	1	0
ENSMUSG00000108237	GM19757	0	0	0	1	1	0	0	0	0	0	0	0
ENSMUSG00000108238	GM43984	1	1	0	0	0	0	1	3	0	2	1	1
ENSMUSG00000108249	GM43960	0	6	0	0	0	0	2	0	0	6	0	1
ENSMUSG00000108254	GM43908	0	2	1	0	0	0	0	0	1	0	0	0
ENSMUSG00000108258	GM43913	1	0	0	0	0	0	0	0	0	1	0	0
ENSMUSG00000108260	GM3793	0	0	1	0	0	1	0	0	1	0	0	0
ENSMUSG00000108268	GM44187	1	5	4	6	0	3	6	7	7	9	9	2
ENSMUSG00000108279	GM44024	0	0	0	0	1	0	2	3	0	0	1	1
ENSMUSG00000108291	GM44292	0	0	0	0	0	0	0	1	0	2	0	1
ENSMUSG00000108292	GM44050	0	0	0	0	0	0	0	0	0	0	1	0
ENSMUSG00000108293	PLA2G4C-PS	0	0	0	1	0	0	0	0	0	0	0	1
ENSMUSG00000108298	GM44226	0	0	0	4	4	0	0	2	0	2	0	4
ENSMUSG00000108306	GM44640	0	0	0	0	0	0	0	0	0	0	1	0
ENSMUSG00000108310	GM44712	0	0	0	0	0	0	0	1	0	0	0	0
ENSMUSG00000108312	GM44521	0	0	0	0	0	0	0	0	0	1	0	0
ENSMUSG00000108314	PRKCZ2	0	0	0	0	0	0	0	1	0	0	0	0
ENSMUSG00000108319	GM44552	0	0	0	0	0	0	0	3	1	4	0	0
ENSMUSG00000108322	5430431A17R	1	1	0	0	0	3	3	2	3	2	3	2
ENSMUSG00000108325	GM44898	0	0	0	0	0	0	1	0	0	0	0	0
ENSMUSG00000108332	D530033B14I	0	0	0	0	0	0	0	0	0	0	1	0
ENSMUSG00000108348	GM42372	0	0	0	1	0	0	0	0	0	0	1	0
ENSMUSG00000108353	GM45205	0	0	0	0	0	0	0	0	0	0	0	1
ENSMUSG00000108354	4931431B13R	0	0	0	0	0	0	0	1	1	0	0	0
ENSMUSG00000108357	SNRP1C-PS1	0	1	0	0	0	0	0	0	0	0	0	0

ENSMUSG00000108360	GM36159	0	0	0	0	0	0	0	0	0	0	0	1
ENSMUSG00000108365	GM44951	0	0	0	0	0	0	0	0	0	0	0	1
ENSMUSG00000108366	GM5586	20	18	17	19	15	12	56	64	51	70	46	53
ENSMUSG00000108368	GM45053	0	1	1	0	1	0	1	1	0	0	2	0
ENSMUSG00000108370	GM8209	1	0	0	0	1	1	1	1	1	0	0	0
ENSMUSG00000108388	GM44673	0	0	0	0	0	0	1	2	0	2	0	0
ENSMUSG00000108394	GM45477	0	1	0	1	0	0	0	0	1	1	1	3
ENSMUSG00000108396	GM6905	0	0	0	0	0	0	1	0	0	0	0	0
ENSMUSG00000108402	9430064I24R	1	0	1	0	1	1	1	1	2	1	1	0
ENSMUSG00000108413	BC026762	0	0	1	1	1	0	1	1	0	1	0	0
ENSMUSG00000108414	SNHG1	29	52	41	44	49	49	281	348	255	402	248	249
ENSMUSG00000108420	GM45141	0	0	0	0	0	0	0	0	0	0	0	2
ENSMUSG00000108435	GM45051	1	2	2	2	0	1	12	4	8	7	9	7
ENSMUSG00000108437	GM19366	0	0	0	0	0	0	0	2	0	0	1	1
ENSMUSG00000108441	GM32846	0	0	0	0	0	1	0	0	0	0	0	0
ENSMUSG00000108442	RPL15-PS5	0	0	0	0	0	1	1	0	0	1	0	0
ENSMUSG00000108443	GM44510	1	0	0	0	1	0	1	0	1	0	1	1
ENSMUSG00000108447	GM44567	0	0	0	1	1	0	2	3	3	5	2	1
ENSMUSG00000108448	GM21269	0	0	0	0	0	0	0	0	0	1	0	0
ENSMUSG00000108452	4930413G21I	0	0	0	0	1	0	1	0	0	1	0	1
ENSMUSG00000108460	GM39041	1	0	0	0	0	0	2	0	0	2	0	0
ENSMUSG00000108465	GM45110	1	2	0	0	1	1	4	2	3	3	1	3
ENSMUSG00000108466	GM44771	0	0	0	0	0	1	0	0	0	0	0	0
ENSMUSG00000108472	GM45212	0	0	0	0	0	0	1	0	0	0	0	0
ENSMUSG00000108477	GM44878	0	0	0	0	0	0	0	2	0	0	1	0
ENSMUSG00000108483	GM45184	0	1	0	0	0	0	0	0	0	0	0	0
ENSMUSG00000108484	GM18537	0	0	0	0	0	0	1	0	0	0	0	0
ENSMUSG00000108485	GM18805	0	0	0	0	0	0	0	1	0	0	0	0
ENSMUSG00000108486	GM44836	0	0	0	0	0	0	1	0	1	0	0	1
ENSMUSG00000108491	GM44523	0	0	1	0	0	0	0	0	0	0	0	0
ENSMUSG00000108511	GM44987	1	0	1	1	0	1	16	8	8	10	7	6
ENSMUSG00000108521	GM44639	1	0	2	0	0	0	3	3	1	3	2	0
ENSMUSG00000108522	GM44664	0	0	0	0	0	1	0	0	0	0	0	0
ENSMUSG00000108524	GM44876	0	0	0	0	0	0	0	1	0	0	0	0
ENSMUSG00000108526	GM45828	1	1	0	2	0	2	0	7	4	4	7	8
ENSMUSG00000108528	GM8319	0	0	0	0	0	0	1	0	0	0	0	0
ENSMUSG00000108542	GM44834	0	0	0	0	0	0	1	0	0	0	1	1
ENSMUSG00000108543	GM44735	1	0	0	0	0	0	0	2	0	1	0	0
ENSMUSG00000108546	GM44662	0	0	0	0	0	0	0	0	0	2	0	0
ENSMUSG00000108551	GM20274	0	1	0	1	0	0	1	4	1	2	1	1
ENSMUSG00000108554	GM44824	0	0	0	0	0	0	0	0	1	0	0	0
ENSMUSG00000108557	GM44835	1	0	0	2	1	0	0	1	3	1	0	0
ENSMUSG00000108560	GM44695	0	0	0	0	0	0	1	2	1	1	2	0
ENSMUSG00000108563	GM44686	0	0	0	0	0	0	0	1	2	0	0	0
ENSMUSG00000108571	GM6855	0	0	0	0	0	1	0	0	0	1	0	1
ENSMUSG00000108573	GM44986	0	0	1	2	3	0	2	4	5	2	0	1
ENSMUSG00000108574	GM44981	0	0	0	0	0	0	0	0	1	0	0	0
ENSMUSG00000108577	GM17811	0	0	0	0	0	0	0	0	0	1	0	0
ENSMUSG00000108583	GM44854	0	0	0	0	0	0	0	0	1	0	0	0
ENSMUSG00000108591	PARTICL	0	0	0	1	0	0	0	0	1	0	0	2
ENSMUSG00000108592	GM38973	0	0	0	1	0	0	0	0	1	0	0	0
ENSMUSG00000108599	GM10616	0	0	0	0	0	0	0	0	0	1	0	0
ENSMUSG00000108601	GM44645	0	0	0	0	0	1	0	1	1	0	1	0
ENSMUSG00000108606	CYP2B27-PS	0	0	0	0	1	0	2	0	0	1	0	0
ENSMUSG00000108607	GM44646	0	0	0	0	0	1	0	0	0	0	0	0
ENSMUSG00000108608	GM6916	0	0	0	0	0	0	0	1	0	0	0	0
ENSMUSG00000108609	GM7424	0	0	0	0	0	0	0	2	0	1	0	0
ENSMUSG00000108617	GM31749	0	0	0	0	0	0	0	1	0	0	0	0

ENSMUSG00000108621	GM37494	1	1	1	1	2	1	6	4	4	5	1	4
ENSMUSG00000108633	GM44694	0	0	1	2	0	2	2	0	3	4	1	8
ENSMUSG00000108648	GM44674	0	0	0	0	0	0	0	0	0	1	0	0
ENSMUSG00000108659	GM34121	0	0	0	0	0	0	3	1	2	2	0	1
ENSMUSG00000108677	GM44759	0	0	0	0	0	0	0	0	0	0	0	1
ENSMUSG00000108681	GM17825	0	1	1	0	0	0	0	0	0	0	0	0
ENSMUSG00000108689	GM45354	1	0	0	0	0	0	1	0	0	0	0	0
ENSMUSG00000108694	GM7287	0	0	0	0	0	0	1	0	1	1	0	0
ENSMUSG00000108696	GM5738	0	0	0	0	0	0	0	0	1	0	0	0
ENSMUSG00000108700	GM7482	0	1	0	0	0	1	1	1	0	1	1	0
ENSMUSG00000108701	GM44616	0	1	0	0	1	0	1	0	1	1	1	5
ENSMUSG00000108702	GM9333	0	0	0	0	0	0	0	1	0	1	0	1
ENSMUSG00000108704	GM44636	0	0	0	0	0	0	0	2	1	0	2	0
ENSMUSG00000108717	GM44940	0	0	0	0	0	0	0	0	0	0	0	1
ENSMUSG00000108732	2310043P16F	1	0	0	0	0	0	0	0	0	0	0	0
ENSMUSG00000108737	BC060293	0	0	0	1	2	0	7	5	3	3	5	3
ENSMUSG00000108738	GM44777	0	0	1	3	0	1	6	5	3	9	6	7
ENSMUSG00000108750	GM44750	3	0	1	3	0	2	1	1	1	2	2	0
ENSMUSG00000108760	GM31897	0	0	0	0	0	0	0	0	1	0	0	0
ENSMUSG00000108763	GM36028	1	0	1	0	0	0	3	2	1	3	2	2
ENSMUSG00000108766	GM44760	0	0	1	0	0	1	0	1	0	2	0	1
ENSMUSG00000108772	GM6063	0	0	0	0	0	0	0	0	0	0	1	0
ENSMUSG00000108780	5430434F05F	1	1	0	1	2	0	9	4	9	7	7	2
ENSMUSG00000108799	GLUD-PS	0	0	0	0	0	0	0	0	0	0	0	1
ENSMUSG00000108812	C130083A15I	0	0	2	1	0	0	0	1	1	0	2	1
ENSMUSG00000108813	RPL19-PS9	0	0	1	0	0	0	0	1	0	0	0	2
ENSMUSG00000108823	GM18959	0	0	0	0	0	0	0	2	0	0	0	0
ENSMUSG00000108825	GM45838	1	0	0	0	0	0	1	1	0	0	0	1
ENSMUSG00000108829	VMN2R-PS89	0	0	0	0	0	0	0	1	0	0	0	0
ENSMUSG00000108833	GM17909	0	0	0	0	0	0	0	1	0	0	1	0
ENSMUSG00000108842	GM45001	0	0	0	0	0	0	0	0	0	1	0	0
ENSMUSG00000108855	GM44917	0	0	0	1	0	0	1	0	0	0	0	0
ENSMUSG00000108878	AC154141.1	0	0	0	0	0	0	2	0	0	0	0	0
ENSMUSG00000108880	GM44560	0	1	0	1	0	1	2	1	0	1	4	1
ENSMUSG00000108886	GM44830	0	0	0	0	0	0	1	1	0	2	0	0
ENSMUSG00000108888	GM45231	0	0	0	0	0	0	0	1	0	0	0	0
ENSMUSG00000108896	GM20136	0	0	0	0	0	0	1	0	0	1	0	0
ENSMUSG00000108902	GM9377	0	0	0	0	0	0	1	0	0	0	0	0
ENSMUSG00000108911	GM45113	0	0	0	1	0	1	0	0	0	0	0	0
ENSMUSG00000108934	GM44732	0	0	0	1	0	0	1	1	1	2	2	4
ENSMUSG00000108940	GM45719	1	2	0	1	0	0	1	1	1	2	0	0
ENSMUSG00000108943	GM44559	0	0	0	0	0	0	0	1	0	1	1	0
ENSMUSG00000108946	GM18598	0	0	0	0	0	0	0	0	0	0	1	0
ENSMUSG00000108949	GM45069	0	0	0	1	0	0	0	0	0	0	0	0
ENSMUSG00000108950	9130015G15I	0	0	0	0	0	0	0	0	0	1	0	0
ENSMUSG00000108954	GM44901	0	1	1	0	0	0	0	0	0	1	1	1
ENSMUSG00000108955	GM44775	0	0	1	0	0	2	2	3	1	5	3	2
ENSMUSG00000108957	GM45235	0	3	0	1	1	1	1	0	2	6	2	2
ENSMUSG00000108959	GM44697	0	1	0	1	1	0	2	1	1	4	2	2
ENSMUSG00000108980	GM45155	0	1	0	0	0	0	0	0	0	0	0	0
ENSMUSG00000108984	GM44916	0	0	0	2	1	0	2	1	0	2	2	1
ENSMUSG00000109003	GM36011	0	0	0	0	0	0	0	1	0	0	0	0
ENSMUSG00000109005	GM45221	1	0	0	1	0	0	2	1	0	3	0	2
ENSMUSG00000109006	B230209E15F	0	0	0	0	0	0	0	0	0	0	1	0
ENSMUSG00000109010	GM45027	0	0	0	0	1	0	3	2	0	0	0	0
ENSMUSG00000109015	GM31024	0	1	0	0	0	0	0	1	0	0	0	1
ENSMUSG00000109016	GM44647	0	0	0	0	0	0	0	1	0	0	3	0
ENSMUSG00000109032	GM7972	1	0	0	0	1	0	0	3	1	0	1	1

ENSMUSG00000109038	GM45120	1	0	1	0	0	0	1	1	0	1	2	2
ENSMUSG00000109045	GM45084	0	0	0	1	0	0	0	0	1	0	0	0
ENSMUSG00000109046	GM44571	1	1	0	0	0	1	0	0	0	0	0	0
ENSMUSG00000109051	GM44913	1	3	0	0	0	1	2	3	3	1	3	1
ENSMUSG00000109059	GM9354	1	0	2	0	0	1	0	0	0	2	0	1
ENSMUSG00000109073	GM44975	0	0	0	1	0	0	1	1	0	0	0	0
ENSMUSG00000109086	GM5596	0	0	0	0	1	0	0	0	0	0	0	0
ENSMUSG00000109094	GM44587	0	0	0	0	0	0	0	0	0	1	0	0
ENSMUSG00000109095	GM44799	0	0	1	0	0	0	0	0	0	0	0	0
ENSMUSG00000109108	GM36371	1	0	1	0	1	1	0	2	0	0	1	0
ENSMUSG00000109111	6530437J22R	0	0	0	0	1	0	0	0	0	1	0	0
ENSMUSG00000109118	GM32031	1	1	1	2	1	1	16	6	10	11	4	9
ENSMUSG00000109122	A230103L15F	1	3	0	0	1	0	5	3	0	4	2	0
ENSMUSG00000109127	GM31135	0	0	0	0	0	0	0	1	0	0	0	0
ENSMUSG00000109128	GM45022	0	0	0	0	0	0	0	1	0	0	0	0
ENSMUSG00000109143	GM10046	0	0	0	0	0	0	1	0	0	0	0	0
ENSMUSG00000109147	4930431P19F	0	0	0	0	1	0	0	1	0	1	3	0
ENSMUSG00000109149	GM19656	0	0	0	0	0	0	0	0	0	1	0	0
ENSMUSG00000109154	GM44822	0	0	0	0	0	0	0	2	0	0	1	0
ENSMUSG00000109157	GM44829	2	8	7	8	8	12	25	18	11	33	16	19
ENSMUSG00000109161	GM39033	0	0	0	0	0	0	0	1	0	0	0	0
ENSMUSG00000109165	GM45148	0	1	0	0	0	0	0	0	0	0	2	0
ENSMUSG00000109168	GM44709	1	0	3	1	2	2	7	12	8	9	9	13
ENSMUSG00000109179	GM35339	1	0	0	0	0	0	0	1	0	0	1	0
ENSMUSG00000109181	GM5605	1	0	0	1	1	0	1	1	2	0	1	0
ENSMUSG00000109198	D7BWG0826I	0	0	0	0	0	0	1	1	0	0	1	0
ENSMUSG00000109206	GM45137	0	0	0	0	1	0	7	4	0	4	0	0
ENSMUSG00000109216	GM44907	0	0	0	0	0	0	0	1	0	0	0	0
ENSMUSG00000109231	GM45737	0	0	0	0	0	0	1	0	0	0	0	0
ENSMUSG00000109241	GM7434	0	0	0	0	0	1	1	0	1	2	1	1
ENSMUSG00000109244	GM44751	0	0	0	0	0	3	2	2	0	1	1	1
ENSMUSG00000109251	E230032D23F	0	2	0	2	0	2	4	7	2	6	2	3
ENSMUSG00000109268	9530078K11F	0	0	0	0	0	0	0	0	1	0	0	0
ENSMUSG00000109274	GM45133	0	0	0	0	0	0	0	1	0	3	1	1
ENSMUSG00000109279	GM45220	0	0	0	0	0	1	1	1	0	1	0	0
ENSMUSG00000109302	GM44967	1	0	0	3	1	1	5	2	1	1	0	2
ENSMUSG00000109305	1810010D01F	0	0	0	0	0	0	0	1	2	1	0	0
ENSMUSG00000109314	GM44699	0	0	0	0	0	0	1	0	0	1	0	0
ENSMUSG00000109317	GM45041	0	0	0	0	0	0	0	1	0	2	0	0
ENSMUSG00000109324	PRMT1	15	25	21	21	29	34	150	169	91	134	94	132
ENSMUSG00000109332	GM44742	0	0	0	0	0	0	0	0	0	0	0	1
ENSMUSG00000109336	SAMD4B	6	8	7	10	8	8	26	36	18	32	31	25
ENSMUSG00000109341	GM30873	0	0	0	0	0	0	0	1	0	0	0	0
ENSMUSG00000109351	GM44632	0	0	0	0	0	0	1	1	0	0	0	1
ENSMUSG00000109353	GM45183	0	0	0	0	0	0	0	0	0	0	1	0
ENSMUSG00000109357	GM45236	0	0	2	3	2	0	1	7	6	5	5	5
ENSMUSG00000109363	GM44668	0	0	0	1	2	0	1	1	1	1	0	1
ENSMUSG00000109378	U2AF1L4	0	0	0	0	1	0	0	0	0	1	0	0
ENSMUSG00000109379	GM44550	0	0	0	0	0	0	0	0	0	1	0	0
ENSMUSG00000109390	GM45718	1	0	0	0	0	0	1	0	0	3	0	2
ENSMUSG00000109399	GM44982	0	0	0	0	0	0	0	0	0	1	0	0
ENSMUSG00000109401	CPEB1OS1	0	0	0	0	0	0	0	0	0	0	0	1
ENSMUSG00000109408	A930037H05I	1	1	0	2	0	2	3	1	1	0	2	1
ENSMUSG00000109428	GM45698	0	0	0	0	0	0	0	1	0	1	1	0
ENSMUSG00000109429	GM45223	3	1	0	1	1	1	2	0	1	2	2	3
ENSMUSG00000109434	GM44937	0	0	0	0	0	2	1	0	1	2	0	0
ENSMUSG00000109448	GM45224	0	0	0	0	0	0	1	0	0	0	0	1
ENSMUSG00000109452	GM44530	0	0	0	0	1	0	0	0	0	0	0	0

ENSMUSG00000109455	GM44710	0	0	0	0	0	0	0	0	0	0	1	0
ENSMUSG00000109482	GM4756	0	0	0	0	0	0	0	3	0	0	0	0
ENSMUSG00000109488	GM6828	0	0	0	0	0	1	0	0	0	0	0	0
ENSMUSG00000109489	GM7451	0	0	0	2	0	0	0	0	2	1	1	0
ENSMUSG00000109495	GM47692	0	0	0	0	0	0	0	1	0	0	0	0
ENSMUSG00000109498	GM45222	1	0	0	0	0	1	1	2	0	4	0	1
ENSMUSG00000109503	GM44786	0	0	1	0	0	0	0	0	0	0	0	0
ENSMUSG00000109509	RPS12-PS4	54	51	56	54	58	61	235	346	214	218	136	184
ENSMUSG00000109511	NUP62	10	12	10	12	5	12	44	70	42	62	48	33
ENSMUSG00000109532	GM42397	0	0	1	0	0	0	0	0	0	0	0	0
ENSMUSG00000109536	9330162G02I	4	14	5	11	11	10	32	36	19	43	29	29
ENSMUSG00000109539	GM44667	0	0	0	0	0	0	1	0	0	1	0	0
ENSMUSG00000109548	GM45066	0	0	0	0	0	0	0	0	1	0	0	0
ENSMUSG00000109560	GM8463	0	0	0	1	0	0	0	0	0	0	0	2
ENSMUSG00000109564	MUC16	0	0	0	0	0	0	0	0	1	0	0	0
ENSMUSG00000109565	GM6579	0	0	0	0	0	0	0	0	0	0	0	1
ENSMUSG00000109570	GM44624	0	1	0	0	0	0	0	1	1	2	0	1
ENSMUSG00000109575	GM44831	0	0	0	1	0	0	1	0	0	3	0	0
ENSMUSG00000109576	GM44704	0	0	0	1	0	0	0	1	0	0	0	2
ENSMUSG00000109580	GM8268	0	1	0	1	0	1	0	2	1	1	1	1
ENSMUSG00000109585	GM45358	3	1	1	1	1	0	3	4	2	3	5	3
ENSMUSG00000109586	GM6853	0	0	0	1	0	0	0	0	0	0	0	0
ENSMUSG00000109599	GM31812	0	0	0	0	0	0	0	0	1	0	0	0
ENSMUSG00000109602	GM31597	0	1	1	0	0	0	3	2	2	4	2	2
ENSMUSG00000109606	GM45537	0	0	0	0	0	0	0	0	0	1	0	0
ENSMUSG00000109610	GM7432	0	1	1	1	3	1	4	5	2	4	0	3
ENSMUSG00000109613	GM45405	0	0	0	0	0	0	1	1	0	0	0	0
ENSMUSG00000109617	GM7669	0	0	0	1	0	0	0	0	0	0	0	0
ENSMUSG00000109638	GM45630	0	0	0	0	0	0	0	0	0	0	0	1
ENSMUSG00000109640	GM45413	0	0	0	0	1	0	1	1	2	0	0	0
ENSMUSG00000109642	GM45289	0	1	0	1	0	1	2	0	1	1	0	2
ENSMUSG00000109643	GM31545	0	0	0	1	0	0	0	0	0	0	0	0
ENSMUSG00000109644	0610005C13F	0	1	0	0	2	0	1	0	0	0	1	0
ENSMUSG00000109647	GM45510	0	0	0	0	0	0	0	0	0	0	1	0
ENSMUSG00000109652	GM45555	0	0	1	0	2	0	3	2	1	2	0	0
ENSMUSG00000109658	GM45660	1	1	0	0	0	0	0	1	1	1	1	2
ENSMUSG00000109673	GM9712	0	0	0	0	0	0	1	0	0	0	1	2
ENSMUSG00000109675	NXPE1-PS	0	0	1	0	0	0	1	0	0	0	0	0
ENSMUSG00000109679	GM45342	0	0	0	0	1	0	1	2	0	2	1	0
ENSMUSG00000109684	GM45745	0	0	0	0	0	0	0	1	0	1	0	0
ENSMUSG00000109693	GM45483	0	0	0	0	0	0	1	0	0	1	0	2
ENSMUSG00000109695	GM31166	1	0	0	0	0	0	1	0	0	1	1	0
ENSMUSG00000109704	GM38414	0	0	0	1	2	1	3	1	3	2	1	1
ENSMUSG00000109708	GM45809	0	0	0	1	1	1	1	1	1	0	1	1
ENSMUSG00000109712	GM8667	0	0	0	0	0	0	0	0	0	0	1	0
ENSMUSG00000109715	GM45606	1	0	0	0	0	0	0	0	1	2	0	0
ENSMUSG00000109717	GM34368	0	0	0	0	0	0	0	0	0	0	0	1
ENSMUSG00000109719	GM45266	0	2	0	0	0	0	0	1	1	1	2	0
ENSMUSG00000109724	GM18194	0	0	1	0	1	1	3	4	9	3	3	2
ENSMUSG00000109727	GM45464	0	1	0	1	3	3	0	3	1	4	0	4
ENSMUSG00000109728	GM45359	0	0	0	0	0	0	0	0	0	0	1	0
ENSMUSG00000109733	GM33940	0	0	0	0	0	0	1	0	0	0	0	0
ENSMUSG00000109739	GM17949	160	193	165	185	151	178	355	318	257	381	235	311
ENSMUSG00000109740	GM7561	0	0	0	0	0	0	0	0	0	2	0	0
ENSMUSG00000109754	GM39214	0	0	0	0	0	0	2	1	0	1	1	1
ENSMUSG00000109764	KLKB1	4	3	3	1	2	1	5	11	3	4	7	2
ENSMUSG00000109765	GM18227	0	1	0	0	0	0	0	0	0	0	0	0
ENSMUSG00000109771	GM35315	0	1	1	1	0	1	3	5	2	2	2	3

ENSMUSG00000109780	GM45447	0	0	0	0	0	0	0	0	0	1	0	0
ENSMUSG00000109781	GM45509	1	0	0	0	0	2	4	2	2	6	1	2
ENSMUSG00000109784	GM45493	0	0	0	0	0	0	0	1	0	0	0	0
ENSMUSG00000109787	GM45286	0	0	0	0	1	0	0	2	1	2	0	0
ENSMUSG00000109799	GM45515	0	0	0	0	0	0	0	0	0	1	1	0
ENSMUSG00000109807	GM45244	0	1	0	0	0	0	0	0	0	1	0	0
ENSMUSG00000109810	GM45592	0	0	0	0	0	0	0	2	0	1	1	0
ENSMUSG00000109812	GM45640	0	0	0	1	0	1	1	1	1	1	2	1
ENSMUSG00000109813	OLFR1279	0	0	0	0	0	0	0	1	0	0	0	0
ENSMUSG00000109820	GM45654	0	0	0	0	0	0	2	0	0	0	0	0
ENSMUSG00000109827	GM6213	0	0	0	0	0	0	0	0	1	0	0	0
ENSMUSG00000109841	E330011O21I	0	0	0	0	0	0	0	0	0	0	0	1
ENSMUSG00000109852	GM45360	1	1	0	0	2	0	1	1	1	1	2	2
ENSMUSG00000109854	GM45345	1	0	0	0	0	1	0	0	0	0	0	0
ENSMUSG00000109864	EID3	1	0	0	1	0	0	0	0	1	0	0	1
ENSMUSG00000109865	HSPA14	16	21	20	19	21	24	155	143	104	148	98	111
ENSMUSG00000109873	GM45407	0	1	1	1	1	0	1	1	0	0	0	1
ENSMUSG00000109875	GM45456	0	0	0	0	0	0	1	0	0	0	0	0
ENSMUSG00000109877	GM45609	0	1	0	0	0	0	0	0	0	0	0	1
ENSMUSG00000109879	GM10997	0	0	0	0	0	0	1	1	0	0	0	0
ENSMUSG00000109881	GM45507	0	0	0	0	0	1	6	2	3	4	0	1
ENSMUSG00000109893	GM9655	1	1	0	5	2	0	0	2	6	5	3	3
ENSMUSG00000109894	GM5904	0	0	0	0	0	0	1	2	0	0	0	0
ENSMUSG00000109899	GM6012	0	1	0	0	0	1	3	1	1	3	1	2
ENSMUSG00000109901	CHMP1B	5	4	2	2	4	3	26	27	18	33	14	22
ENSMUSG00000109904	GM45819	0	0	0	1	1	0	0	0	0	1	1	0
ENSMUSG00000109905	GM7380	1	0	0	0	0	0	0	0	1	0	1	1
ENSMUSG00000109925	GM45315	0	0	0	0	0	0	0	0	0	1	0	1
ENSMUSG00000109931	GM39929	0	0	0	0	0	0	0	1	1	0	0	0
ENSMUSG00000109933	GM7600	3	5	3	5	1	2	5	10	5	7	9	6
ENSMUSG00000109940	GM45399	0	0	0	0	0	0	4	2	3	4	2	3
ENSMUSG00000109956	GM45428	0	0	0	0	0	0	2	1	0	0	0	0
ENSMUSG00000109957	GM45353	1	0	0	0	0	0	0	2	1	0	0	1
ENSMUSG00000109966	GM34623	0	0	0	0	0	0	1	0	0	0	0	0
ENSMUSG00000109975	GM19196	0	0	0	1	0	0	3	2	1	1	0	1
ENSMUSG00000109984	GM38948	0	0	0	0	0	0	0	0	0	0	0	2
ENSMUSG00000110010	GM45629	1	0	0	0	1	0	4	2	1	4	1	3
ENSMUSG00000110020	GM45441	0	0	0	0	0	0	0	0	0	0	1	0
ENSMUSG00000110030	GM45546	0	0	0	0	0	0	1	0	0	0	0	1
ENSMUSG00000110038	GM45570	0	2	0	0	0	1	0	2	0	0	0	0
ENSMUSG00000110057	GM2225	0	1	1	0	0	0	5	1	1	2	3	2
ENSMUSG00000110058	GM5907	0	1	0	0	0	0	0	3	0	3	2	0
ENSMUSG00000110079	GM45292	0	0	1	1	1	0	2	0	3	1	1	1
ENSMUSG00000110087	GM45309	0	0	0	0	0	0	0	0	0	0	1	0
ENSMUSG00000110088	GM45343	5	5	6	3	6	4	8	10	9	16	5	16
ENSMUSG00000110089	GM36243	0	0	0	0	0	0	0	0	1	0	0	0
ENSMUSG00000110105	GM45844	0	0	0	0	0	0	0	1	0	0	0	0
ENSMUSG00000110109	GM5599	0	0	1	0	1	0	0	1	0	0	0	0
ENSMUSG00000110120	GM45251	0	1	0	1	1	0	1	0	0	1	0	1
ENSMUSG00000110126	GM9347	0	0	0	0	0	0	0	1	0	1	0	0
ENSMUSG00000110127	6430710M23	1	0	0	1	0	0	2	4	0	0	0	1
ENSMUSG00000110129	GM45453	0	0	0	0	0	0	0	0	0	1	1	1
ENSMUSG00000110138	4831440D22I	0	2	2	1	0	1	4	1	0	0	0	0
ENSMUSG00000110148	5830408C22I	2	0	0	0	0	0	2	0	1	1	1	1
ENSMUSG00000110156	GM42067	1	0	2	0	1	0	7	3	1	2	2	5
ENSMUSG00000110159	GM45501	1	2	0	3	1	1	5	7	4	8	4	7
ENSMUSG00000110164	GM7351	0	0	0	0	1	0	0	1	0	0	0	0
ENSMUSG00000110165	GM4032	0	0	0	0	0	0	0	1	0	0	0	0

ENSMUSG00000110176	GM35520	0	0	0	0	0	0	0	0	0	0	1	0
ENSMUSG00000110185	IGIP	0	0	0	0	0	1	1	2	4	6	3	7
ENSMUSG00000110187	GM45496	0	0	0	0	0	0	1	0	0	1	1	0
ENSMUSG00000110206	FLT3L	4	1	1	3	7	4	23	23	15	28	22	16
ENSMUSG00000110218	GM20219	0	0	1	1	0	0	2	1	1	2	6	4
ENSMUSG00000110225	GM45528	0	0	0	0	0	0	0	0	0	0	1	0
ENSMUSG00000110231	GM6329	2	0	2	3	3	1	10	6	9	9	7	9
ENSMUSG00000110234	GM45799	0	0	0	0	0	0	0	1	0	0	1	1
ENSMUSG00000110238	GM19269	0	0	1	0	1	1	5	1	5	2	2	5
ENSMUSG00000110239	GM45620	0	0	0	0	0	0	0	0	0	1	1	0
ENSMUSG00000110246	C130073E24F	0	0	0	0	0	0	0	0	0	0	0	1
ENSMUSG00000110247	GM45269	0	0	0	0	0	0	0	1	0	0	0	1
ENSMUSG00000110252	GM2961	0	0	0	0	0	0	1	0	0	0	0	0
ENSMUSG00000110256	GM45412	1	1	2	1	0	0	3	0	0	1	1	1
ENSMUSG00000110260	GM30504	0	0	0	0	0	0	0	1	0	0	0	0
ENSMUSG00000110266	GM32742	0	1	0	0	1	0	3	3	2	2	1	2
ENSMUSG00000110270	GM45607	0	0	0	0	0	0	1	0	1	0	0	0
ENSMUSG00000110275	GM5905	3	2	1	1	1	0	3	4	5	5	8	8
ENSMUSG00000110277	GM45871	0	0	0	0	0	0	2	1	1	3	0	0
ENSMUSG00000110279	GM45552	2	5	7	12	7	2	18	19	15	23	14	13
ENSMUSG00000110289	4930412F12F	0	1	1	0	0	0	2	0	3	0	1	1
ENSMUSG00000110295	GM45319	0	0	0	0	0	0	0	0	0	1	0	0
ENSMUSG00000110298	GM8189	67	54	66	69	55	48	334	400	289	383	245	284
ENSMUSG00000110313	GM45420	0	0	0	0	0	0	0	0	0	0	1	0
ENSMUSG00000110316	GM45311	1	0	0	0	1	0	0	0	1	0	1	0
ENSMUSG00000110317	GM45250	1	1	0	0	0	1	3	2	3	3	1	2
ENSMUSG00000110320	GM31152	0	0	0	0	0	0	0	0	0	2	0	0
ENSMUSG00000110325	GM45293	0	0	0	0	0	0	1	0	0	0	0	0
ENSMUSG00000110341	GM45424	0	0	0	0	0	0	0	0	0	0	0	1
ENSMUSG00000110344	GM45716	3	3	2	0	1	5	1	8	4	10	7	11
ENSMUSG00000110350	GM10252	0	0	0	0	0	0	1	1	0	0	0	0
ENSMUSG00000110360	GM45380	1	1	0	0	0	0	0	1	1	2	0	3
ENSMUSG00000110361	GM8291	0	0	0	0	0	0	0	0	0	0	0	1
ENSMUSG00000110368	GM45518	0	1	1	0	0	0	2	0	0	0	0	0
ENSMUSG00000110371	GM9619	0	0	0	0	0	0	0	0	1	0	0	1
ENSMUSG00000110378	GM45242	0	1	0	0	0	0	1	0	0	1	0	0
ENSMUSG00000110391	GM7269	0	0	0	0	0	0	0	0	1	1	0	0
ENSMUSG00000110393	GM36445	2	0	1	1	0	2	5	3	3	6	7	3
ENSMUSG00000110397	GM45540	0	0	0	0	0	0	0	0	1	0	0	0
ENSMUSG00000110404	RNF223	0	1	0	0	0	0	1	1	0	2	0	4
ENSMUSG00000110405	GM45534	2	0	0	2	2	0	0	0	0	2	2	2
ENSMUSG00000110407	GM45454	0	0	0	0	0	0	0	0	0	0	0	1
ENSMUSG00000110419	AW046200	1	0	1	0	0	0	0	1	0	0	1	1
ENSMUSG00000110424	1700012D14F	0	0	0	0	2	2	2	0	2	2	2	1
ENSMUSG00000110425	GM32390	0	0	0	0	0	0	1	0	0	0	0	0
ENSMUSG00000110434	RPS7-PS1	0	0	0	0	0	0	1	0	0	0	0	0
ENSMUSG00000110438	GM45756	0	0	0	0	0	0	0	0	0	0	1	0
ENSMUSG00000110440	GM45894	1	1	3	3	1	3	11	10	9	19	4	11
ENSMUSG00000110443	GM39197	0	0	0	0	0	0	0	0	0	0	0	1
ENSMUSG00000110444	GM10033	5	4	2	2	3	5	25	14	11	27	14	6
ENSMUSG00000110446	GM18646	0	0	0	0	0	0	1	0	0	0	0	0
ENSMUSG00000110456	GM45865	0	0	1	0	0	0	0	2	0	0	0	1
ENSMUSG00000110469	GM10358	0	0	0	0	0	0	1	0	1	0	1	1
ENSMUSG00000110499	GM45873	0	0	3	1	2	1	4	1	0	1	1	0
ENSMUSG00000110504	GM45797	0	0	0	0	1	0	1	1	0	0	1	3
ENSMUSG00000110509	GM45772	0	0	0	0	0	0	0	1	0	1	0	0
ENSMUSG00000110528	GM1943	0	0	0	0	0	0	0	0	1	0	0	0
ENSMUSG00000110529	GM45694	0	0	0	0	1	0	0	0	0	0	1	1

ENSMUSG00000110530	GM35934	0	0	0	0	0	0	0	0	0	1	3	1
ENSMUSG00000110532	GM35857	0	2	4	0	2	1	2	2	3	0	0	1
ENSMUSG00000110544	GM2607	0	0	0	0	0	0	0	0	0	0	1	0
ENSMUSG00000110545	GM7730	1	0	0	0	0	0	1	2	2	1	1	1
ENSMUSG00000110547	GM29773	4	3	5	6	2	10	19	24	14	17	14	21
ENSMUSG00000110550	GM45856	0	0	0	0	0	0	0	0	0	1	0	0
ENSMUSG00000110556	GM45762	0	0	0	0	0	0	1	1	1	3	0	3
ENSMUSG00000110558	GM45779	0	0	0	0	0	0	0	0	0	2	0	0
ENSMUSG00000110562	GM9204	0	0	0	0	0	0	1	0	0	0	0	0
ENSMUSG00000110575	6330537M06	1	0	1	0	1	0	0	2	2	0	1	1
ENSMUSG00000110580	D830024N08	0	0	0	1	0	0	0	0	2	0	2	0
ENSMUSG00000110586	4930578M07	1	1	0	0	0	0	0	1	2	3	0	3
ENSMUSG00000110588	GM45774	0	0	0	0	0	0	0	2	0	0	0	0
ENSMUSG00000110592	4930488N15I	0	0	0	1	0	1	0	0	0	0	0	0
ENSMUSG00000110594	GM33103	0	0	0	0	0	0	0	0	0	0	0	1
ENSMUSG00000110595	GM7990	1	0	0	0	0	1	1	2	1	3	1	3
ENSMUSG00000110597	GM8798	0	0	0	0	0	0	0	1	0	0	0	0
ENSMUSG00000110605	GM32856	4	6	8	3	1	10	29	19	17	29	18	19
ENSMUSG00000110607	GM45843	0	0	0	1	0	0	0	0	2	0	0	0
ENSMUSG00000110610	GM7769	0	0	0	0	0	0	1	0	0	0	0	0
ENSMUSG00000110611	GM20163	0	0	0	0	0	0	0	1	0	0	2	1
ENSMUSG00000110612	GM17827	0	0	0	0	0	0	0	0	0	1	0	0
ENSMUSG00000110615	GM45890	12	15	12	17	7	32	83	67	59	72	53	72
ENSMUSG00000110620	GM45733	7	6	7	2	1	8	8	22	11	11	7	8
ENSMUSG00000110622	GM16486	0	0	0	0	0	0	0	0	0	1	0	0
ENSMUSG00000110630	K230015D01I	0	0	0	0	0	0	0	0	1	1	0	0
ENSMUSG00000110631	GM42047	14	12	12	24	26	23	81	84	58	90	54	79
ENSMUSG00000110635	GM45853	0	1	0	0	0	0	0	0	0	0	0	0
ENSMUSG00000110647	GM17745	0	0	0	0	0	0	0	0	0	0	0	1
ENSMUSG00000110648	GM38574	0	0	0	0	0	0	0	0	0	1	1	1
ENSMUSG00000110654	GM45746	0	0	0	0	0	0	0	0	0	0	0	1
ENSMUSG00000110656	GM20735	0	0	0	1	0	0	1	1	0	0	0	2
ENSMUSG00000110660	GM5362	0	0	0	0	0	0	1	0	0	0	0	0
ENSMUSG00000110669	GM10060	1	0	1	1	0	0	1	1	1	1	1	0
ENSMUSG00000110679	RPL10-PS5	0	0	0	0	0	0	1	2	0	0	0	0
ENSMUSG00000110684	GM45866	0	0	0	0	0	0	1	2	0	0	0	0
ENSMUSG00000110685	GM5373	0	0	0	0	0	0	0	0	2	0	0	0
ENSMUSG00000110686	GM45855	0	1	2	0	2	1	2	0	1	1	0	1
ENSMUSG00000110689	GM45731	0	0	0	0	0	0	1	1	0	0	0	0
ENSMUSG00000110692	GM33460	0	1	1	0	0	0	3	1	1	2	6	0
ENSMUSG00000110700	GM5357	0	0	0	0	1	0	0	0	0	0	0	0
ENSMUSG00000110702	GM45767	0	0	0	0	1	0	0	0	1	0	0	0
ENSMUSG00000110704	GM45732	0	0	0	0	0	1	2	6	2	4	2	1
ENSMUSG00000110710	C78859	0	0	0	0	0	1	3	0	0	3	0	0
ENSMUSG00000110711	GM45760	0	0	1	0	0	0	0	0	0	0	0	0
ENSMUSG00000110738	GM47092	0	1	0	0	0	0	1	0	0	0	0	0
ENSMUSG00000110750	GM48702	0	0	0	0	0	0	0	0	0	0	0	1
ENSMUSG00000110751	C230053D17I	0	0	0	0	0	1	0	0	2	0	0	1
ENSMUSG00000110758	GM48092	0	0	0	0	0	0	0	1	0	0	0	0
ENSMUSG00000110759	GM6607	0	2	0	0	0	0	0	0	0	0	0	0
ENSMUSG00000110761	GM29761	0	0	0	0	0	0	0	0	0	0	2	2
ENSMUSG00000110767	GM31432	0	0	0	0	0	0	0	0	0	0	1	1
ENSMUSG00000110772	GM47856	0	0	0	0	0	0	0	0	0	1	0	0
ENSMUSG00000110773	AC061963.1	0	0	0	1	0	0	0	0	2	1	2	1
ENSMUSG00000110774	GM39318	0	0	0	0	0	0	0	0	0	2	0	0
ENSMUSG00000110790	GM47079	0	3	1	2	2	2	8	5	5	3	3	6
ENSMUSG00000110791	GM47508	0	0	0	0	0	0	1	0	0	0	0	1
ENSMUSG00000110795	GM47179	0	1	0	0	0	0	1	1	2	0	0	0

ENSMUSG00000110825	GM8959	0	0	0	0	0	0	0	1	0	0	0	1
ENSMUSG00000110829	GM47638	0	0	0	0	0	0	0	0	0	1	0	0
ENSMUSG00000110830	GM46218	0	0	0	0	0	0	0	0	1	1	1	0
ENSMUSG00000110831	GM48159	0	0	0	0	0	0	0	0	0	2	0	0
ENSMUSG00000110834	GM39469	0	1	0	0	2	3	5	4	3	4	6	5
ENSMUSG00000110840	GM47496	0	0	0	0	0	0	4	0	0	2	1	0
ENSMUSG00000110841	GPX4-PS2	15	21	14	25	17	9	51	28	47	48	42	40
ENSMUSG00000110845	GM47481	0	0	0	0	0	0	0	0	1	1	0	6
ENSMUSG00000110866	GM48362	1	0	0	0	0	0	0	0	0	0	0	0
ENSMUSG00000110869	AC122901.1	0	0	0	0	0	0	1	0	1	1	1	1
ENSMUSG00000110874	D030045P18I	1	0	0	0	0	0	0	0	0	1	0	0
ENSMUSG00000110887	CBX3-PS8	0	0	0	0	0	0	0	0	0	0	0	1
ENSMUSG00000110894	GM48335	0	0	0	0	0	0	0	1	0	0	0	0
ENSMUSG00000110898	GM40638	0	0	0	0	0	0	0	1	0	0	0	0
ENSMUSG00000110899	GM48840	0	0	0	0	1	0	1	0	0	0	0	0
ENSMUSG00000110903	GM47857	1	0	0	0	0	0	0	0	0	0	0	0
ENSMUSG00000110910	4921534A09I	0	0	0	0	0	0	1	0	0	0	0	0
ENSMUSG00000110914	GM48611	2	1	1	0	1	0	1	0	0	1	1	4
ENSMUSG00000110915	GM36198	0	0	0	0	0	0	0	1	0	0	0	0
ENSMUSG00000110916	2310003N18I	0	0	0	0	0	0	0	0	0	0	0	1
ENSMUSG00000110926	GM5917	0	0	0	1	0	0	0	0	0	0	0	0
ENSMUSG00000110928	GM48114	0	0	0	0	0	1	2	0	2	0	0	0
ENSMUSG00000110945	GM9856	0	2	1	0	0	1	3	2	7	4	9	4
ENSMUSG00000110957	GM7439	0	0	0	0	0	0	0	1	1	1	0	0
ENSMUSG00000110958	AC153891.1	0	0	0	0	1	0	5	4	3	4	5	2
ENSMUSG00000110972	5830462O15I	0	0	0	0	0	0	0	1	1	0	0	1
ENSMUSG00000110980	GM47204	1	0	0	0	0	0	1	2	0	1	0	0
ENSMUSG00000110982	GM48565	0	0	0	0	0	0	0	1	0	0	0	0
ENSMUSG00000110993	GM47963	0	0	0	1	0	0	0	0	0	0	0	1
ENSMUSG00000110997	2610028D06I	0	0	0	0	0	0	2	0	0	0	0	1
ENSMUSG00000111000	GM47108	0	0	0	0	0	0	1	0	0	0	0	0
ENSMUSG00000111004	GM48272	2	1	0	0	1	0	1	2	2	5	0	2
ENSMUSG00000111015	GM3126	0	0	0	0	0	0	0	0	1	0	0	0
ENSMUSG00000111017	GM48314	0	0	1	0	0	0	1	1	1	0	0	1
ENSMUSG00000111027	GM2981	0	0	0	0	0	0	0	1	0	0	0	0
ENSMUSG00000111030	GM47234	1	2	2	1	1	0	4	6	2	6	3	8
ENSMUSG00000111034	GM48066	0	0	0	0	0	0	0	0	1	1	0	0
ENSMUSG00000111039	GM47000	1	0	1	0	0	0	0	0	0	0	0	0
ENSMUSG00000111045	GM47598	0	0	0	0	0	0	1	0	0	0	0	0
ENSMUSG00000111055	D030034A15I	0	0	0	0	0	0	0	0	0	1	0	0
ENSMUSG00000111067	GM48714	0	0	0	0	0	0	0	0	0	0	1	0
ENSMUSG00000111069	GM48646	0	1	0	0	0	0	0	0	0	0	0	0
ENSMUSG00000111080	GM20300	0	0	0	0	2	1	5	0	0	4	0	1
ENSMUSG00000111082	GM30934	0	1	0	0	0	0	0	1	0	0	0	0
ENSMUSG00000111086	GM48671	0	0	0	0	0	1	0	1	0	0	0	3
ENSMUSG00000111088	9430081H08I	0	0	0	1	0	1	4	1	3	4	4	8
ENSMUSG00000111090	GM30698	0	0	0	0	0	0	0	0	0	2	0	0
ENSMUSG00000111118	GM6545	3	0	1	3	0	0	6	7	2	10	4	4
ENSMUSG00000111128	AC126804.1	2	1	0	1	0	1	3	4	2	3	4	3
ENSMUSG00000111131	9930024M15	1	0	1	1	2	2	1	1	1	1	0	1
ENSMUSG00000111132	GM48142	0	1	0	0	0	0	0	0	0	0	0	0
ENSMUSG00000111133	GM5831	0	0	0	0	0	0	0	1	0	0	0	0
ENSMUSG00000111151	GM5120	0	0	0	0	0	1	0	0	0	0	0	0
ENSMUSG00000111157	GM47887	0	0	0	0	0	0	0	1	0	0	0	0
ENSMUSG00000111160	GM48855	1	1	1	2	1	0	0	3	2	5	0	1
ENSMUSG00000111161	GM48849	0	0	0	0	1	1	0	0	0	0	0	0
ENSMUSG00000111162	GM7293	0	0	0	0	0	0	0	0	0	1	0	0
ENSMUSG00000111163	GM46136	0	0	0	0	1	0	1	2	1	1	0	0

ENSMUSG00000111172	GM39302	0	0	0	0	0	0	0	0	0	0	0	1
ENSMUSG00000111176	GM47233	0	0	0	0	0	0	0	1	0	0	0	0
ENSMUSG00000111181	GM48124	0	0	0	0	0	0	0	0	0	1	0	0
ENSMUSG00000111203	GM48719	0	0	0	0	0	0	1	0	1	0	0	0
ENSMUSG00000111212	GM47087	1	4	3	2	1	1	8	5	7	7	6	6
ENSMUSG00000111218	GM46999	0	1	0	0	0	0	0	0	0	0	0	0
ENSMUSG00000111228	GM48789	0	0	0	0	0	1	0	0	0	0	0	0
ENSMUSG00000111258	GM34397	0	0	0	1	0	0	0	0	0	0	0	0
ENSMUSG00000111264	E030022I16R	2	0	0	0	0	0	1	2	2	4	2	1
ENSMUSG00000111290	GM5122	0	0	0	1	0	0	0	0	0	0	0	0
ENSMUSG00000111291	GM48604	0	2	0	0	0	0	2	4	1	7	1	5
ENSMUSG00000111292	GM48627	0	0	0	0	0	0	0	0	0	1	1	0
ENSMUSG00000111293	GM34006	2	1	1	0	1	2	10	7	3	5	2	3
ENSMUSG00000111301	GM30671	0	0	0	0	0	0	0	1	0	0	0	0
ENSMUSG00000111312	GM47205	1	0	0	0	0	0	0	0	1	0	0	0
ENSMUSG00000111325	GM47140	0	0	0	0	0	0	0	1	0	1	0	0
ENSMUSG00000111329	A830035O19I	0	0	0	1	0	0	0	0	0	0	0	0
ENSMUSG00000111333	GM48670	0	0	0	0	0	0	0	1	0	3	1	1
ENSMUSG00000111335	GM39375	0	0	0	0	1	0	0	0	0	0	0	0
ENSMUSG00000111340	GM47171	0	0	0	0	0	0	0	0	0	0	0	1
ENSMUSG00000111341	GM47111	0	0	0	0	0	1	0	1	0	0	0	0
ENSMUSG00000111343	GM47175	0	0	0	0	0	1	0	1	1	0	0	0
ENSMUSG00000111345	GM48562	0	2	1	1	2	1	2	1	5	4	4	8
ENSMUSG00000111348	GM19531	0	0	0	0	0	0	0	0	1	0	0	0
ENSMUSG00000111356	RPL32L	0	0	0	0	0	0	3	0	0	2	0	3
ENSMUSG00000111361	GM47445	1	8	2	11	2	2	8	7	7	8	6	3
ENSMUSG00000111362	GM47389	0	0	0	0	0	0	0	0	0	0	0	1
ENSMUSG00000111365	GM47772	1	1	0	0	0	0	0	0	0	0	2	1
ENSMUSG00000111375	BTBD8	0	0	0	0	0	0	0	1	2	4	0	2
ENSMUSG00000111378	GM47230	0	0	0	0	1	0	0	0	0	0	1	0
ENSMUSG00000111380	D9WSU149	0	0	0	0	0	0	1	0	0	0	0	0
ENSMUSG00000111389	GM39465	0	0	0	0	0	0	0	0	1	0	0	0
ENSMUSG00000111390	GM48796	0	1	0	1	0	1	3	5	4	3	3	6
ENSMUSG00000111394	AC160637.1	0	0	0	0	0	0	2	0	0	4	2	1
ENSMUSG00000111397	GM48232	1	0	0	0	0	0	0	0	0	0	0	0
ENSMUSG00000111409	RNF26	0	0	0	0	0	0	1	1	1	0	2	0
ENSMUSG00000111414	GM18103	0	0	0	0	0	0	1	0	0	1	0	0
ENSMUSG00000111421	GM47643	0	0	0	0	0	0	0	2	1	0	0	0
ENSMUSG00000111424	GM48478	0	0	0	0	0	0	0	0	0	0	0	1
ENSMUSG00000111425	GM47324	0	0	0	0	0	0	1	1	1	0	0	0
ENSMUSG00000111434	GM47121	0	1	1	1	1	0	1	1	1	1	1	2
ENSMUSG00000111442	GM48334	1	0	1	1	0	0	2	1	1	3	2	0
ENSMUSG00000111446	GM6581	0	1	0	0	0	0	0	1	2	0	2	0
ENSMUSG00000111448	OLFR912	1	0	0	0	0	0	1	1	0	0	0	0
ENSMUSG00000111455	OLFR1142-PS	0	0	0	0	0	1	0	0	0	0	0	0
ENSMUSG00000111459	GM8031	0	0	0	0	0	0	0	0	0	0	0	1
ENSMUSG00000111471	GM47350	0	2	0	0	0	0	0	0	0	0	0	0
ENSMUSG00000111472	GM47103	0	0	0	0	0	0	0	0	0	0	1	0
ENSMUSG00000111481	GM47652	0	1	1	0	0	0	0	1	1	1	0	3
ENSMUSG00000111489	1700019J19R	0	0	0	0	0	0	1	1	1	2	0	1
ENSMUSG00000111491	GM47503	0	0	0	0	0	0	1	0	0	0	0	0
ENSMUSG00000111494	4930431F10R	0	0	0	0	0	0	0	0	1	0	0	0
ENSMUSG00000111496	GM47483	0	0	1	2	0	0	1	3	0	1	1	0
ENSMUSG00000111497	RAVER1	0	0	0	0	0	0	0	1	0	0	0	0
ENSMUSG00000111505	GM48673	0	0	0	0	0	0	0	1	0	2	0	0
ENSMUSG00000111515	GM5055	1	0	0	2	2	0	1	1	0	2	0	0
ENSMUSG00000111517	OLFR1238	0	1	0	0	0	0	0	0	0	0	0	0
ENSMUSG00000111521	GM48529	0	0	0	0	0	0	2	0	1	0	1	1

ENSMUSG00000111535	GM35154	0	0	0	0	0	0	0	0	0	0	1	0
ENSMUSG00000111537	GM7653	0	1	0	0	0	0	0	0	0	0	0	0
ENSMUSG00000111546	GM47050	25	35	33	36	42	37	130	145	128	164	105	135
ENSMUSG00000111547	GM48069	0	1	0	2	0	0	2	3	0	2	1	2
ENSMUSG00000111575	GM47926	0	0	0	0	0	0	0	0	0	1	0	0
ENSMUSG00000111590	OLFR1192-PS	0	0	0	0	0	0	0	0	0	1	0	0
ENSMUSG00000111593	GM48742	3	5	1	4	4	1	4	5	3	7	1	3
ENSMUSG00000111594	GM3365	0	0	0	1	0	0	0	0	0	1	0	0
ENSMUSG00000111605	4632418H02I	0	0	1	0	0	0	2	2	0	3	0	2
ENSMUSG00000111617	GM47148	0	0	0	0	0	1	0	0	0	0	0	0
ENSMUSG00000111629	4930442G15I	0	0	0	0	0	0	1	0	0	0	0	0
ENSMUSG00000111632	GM47327	0	0	1	0	0	0	0	0	2	0	0	4
ENSMUSG00000111633	GM39377	0	0	0	0	0	0	0	1	0	0	0	0
ENSMUSG00000111635	GM47240	0	0	0	0	0	0	2	0	0	0	0	1
ENSMUSG00000111649	AC126806.1	0	1	0	1	0	0	0	0	0	1	1	0
ENSMUSG00000111656	GM47232	1	0	0	0	0	0	2	1	0	0	0	1
ENSMUSG00000111661	GM46142	1	0	0	0	0	0	0	1	2	0	0	1
ENSMUSG00000111662	GM7435	0	1	0	0	0	0	0	2	0	1	0	4
ENSMUSG00000111665	A930033M14	0	0	0	0	0	0	1	0	0	0	0	0
ENSMUSG00000111671	GM6983	0	0	0	0	0	0	1	2	1	3	1	1
ENSMUSG00000111681	GM47640	0	0	0	0	0	0	0	0	0	2	1	1
ENSMUSG00000111684	AC073947.1	1	0	0	0	0	0	2	0	1	0	0	0
ENSMUSG00000111685	GM10686	0	0	0	0	0	0	0	0	0	0	0	1
ENSMUSG00000111690	GM48720	0	0	0	0	0	0	2	1	1	3	1	1
ENSMUSG00000111691	GM47002	0	0	0	0	0	0	0	0	0	1	0	0
ENSMUSG00000111713	GM20234	2	2	0	0	0	0	3	1	2	3	1	4
ENSMUSG00000111722	GM47270	0	0	0	0	0	0	0	0	0	1	0	0
ENSMUSG00000111733	GM47181	0	0	0	0	0	0	0	1	0	0	0	0
ENSMUSG00000111737	GM47248	0	0	0	0	0	0	1	0	0	1	1	0
ENSMUSG00000111738	GM18329	0	0	0	0	0	0	0	0	0	0	1	0
ENSMUSG00000111740	AC122335.1	0	0	1	0	0	0	3	0	2	0	0	1
ENSMUSG00000111743	GM48856	1	1	0	1	0	0	1	2	0	1	0	1
ENSMUSG00000111744	GM7780	1	1	0	1	0	2	3	0	1	2	0	2
ENSMUSG00000111748	GM34885	0	0	0	0	0	0	1	0	0	0	0	0
ENSMUSG00000111767	4930546K05I	0	0	0	0	0	0	0	0	0	0	1	0
ENSMUSG00000111792	GM33858	0	0	0	0	0	0	1	0	0	0	0	0
ENSMUSG00000111793	GM47950	0	0	0	0	0	0	1	0	0	0	0	0
ENSMUSG00000111808	GM18225	0	0	0	0	0	0	0	0	0	1	0	0
ENSMUSG00000111818	GM17749	0	0	0	0	0	0	0	0	0	1	0	0
ENSMUSG00000111828	D830035M03	0	0	0	0	0	0	0	0	0	0	1	0
ENSMUSG00000111847	GM8899	1	0	0	1	2	1	3	2	2	4	1	2
ENSMUSG00000111868	GM47398	0	0	0	0	0	0	0	0	0	1	1	0
ENSMUSG00000111875	GM5425	0	0	0	0	0	0	0	0	1	0	0	0
ENSMUSG00000111877	GM6477	2	0	3	3	2	2	14	11	9	17	8	5
ENSMUSG00000111880	GM5040	0	0	0	0	1	0	4	2	0	0	0	0
ENSMUSG00000111897	GM19810	0	1	0	0	0	1	2	0	0	0	3	1
ENSMUSG00000111905	GM48045	0	0	0	0	1	0	1	2	0	3	1	1
ENSMUSG00000111916	4921501I09R	0	0	0	0	0	0	0	0	1	0	0	0
ENSMUSG00000111919	GM46192	0	0	0	0	0	0	0	1	0	0	0	0
ENSMUSG00000111922	GM47760	0	0	0	0	0	0	1	0	0	0	0	0
ENSMUSG00000111927	GM47770	0	0	2	0	0	0	1	1	0	0	0	0
ENSMUSG00000111929	GM48780	0	0	0	0	0	0	1	0	0	0	0	0
ENSMUSG00000111932	GM18899	0	0	0	0	0	0	0	0	0	1	0	0
ENSMUSG00000111937	GM46203	1	0	0	0	0	0	0	0	0	0	0	1
ENSMUSG00000111939	GM48177	0	0	1	0	0	0	0	0	1	1	0	1
ENSMUSG00000111942	GM5182	1	0	0	0	0	0	0	0	0	0	0	0
ENSMUSG00000111947	GM47438	1	0	0	0	1	0	0	0	0	0	0	0
ENSMUSG00000111957	GM47582	0	0	0	0	0	0	0	1	0	0	0	0

ENSMUSG00000111958	GM47844	0	0	0	0	0	0	0	0	0	0	1	0
ENSMUSG00000111964	GM8942	0	0	0	0	0	0	2	0	0	0	0	0
ENSMUSG00000111966	GM5781	25	33	39	41	43	42	122	130	106	115	70	116
ENSMUSG00000111968	GM8873	0	0	0	0	0	0	0	2	0	0	1	0
ENSMUSG00000111971	GM48678	0	0	0	1	0	2	3	4	5	2	2	1
ENSMUSG00000111974	GM4803	0	0	0	0	0	0	0	0	0	0	1	0
ENSMUSG00000111997	GM5176	1	4	3	4	0	2	13	15	9	16	16	10
ENSMUSG00000112008	GM47218	0	0	0	0	0	0	0	0	0	1	1	0
ENSMUSG00000112009	GM48591	0	0	0	0	0	0	0	0	0	1	0	0
ENSMUSG00000112015	GM47592	0	0	0	0	0	0	0	0	1	0	0	0
ENSMUSG00000112021	GM30346	0	0	0	0	0	0	0	0	1	1	0	0
ENSMUSG00000112023	LILR4B	1	0	0	0	0	0	0	0	0	0	0	0
ENSMUSG00000112026	GM6653	0	0	0	0	2	0	0	2	3	0	3	1
ENSMUSG00000112033	GM48808	0	1	0	0	0	1	0	0	0	0	0	0
ENSMUSG00000112046	GM5175	0	0	0	0	0	0	0	0	0	0	1	0
ENSMUSG00000112054	GM33680	0	0	0	0	1	0	0	0	0	0	0	0
ENSMUSG00000112056	GM48181	0	0	0	0	0	0	0	0	0	1	0	0
ENSMUSG00000112068	GM9182	0	0	0	0	0	0	1	0	0	0	0	1
ENSMUSG00000112082	GM35035	0	0	1	0	0	0	0	0	1	1	1	1
ENSMUSG00000112090	GM48231	0	0	0	0	1	1	1	2	2	3	0	0
ENSMUSG00000112093	GM47941	0	0	0	0	0	0	0	1	0	0	0	0
ENSMUSG00000112095	A130077B15	0	0	2	0	0	2	3	4	2	3	1	3
ENSMUSG00000112096	A430103D13	0	0	1	2	1	0	9	6	2	11	4	6
ENSMUSG00000112099	GM8960	39	36	34	53	49	35	117	118	101	109	99	88
ENSMUSG00000112110	AC158605.1	0	0	0	0	0	0	1	1	0	0	0	0
ENSMUSG00000112116	GM46234	0	0	0	1	0	0	0	0	0	0	0	0
ENSMUSG00000112120	GM32255	0	0	0	0	0	0	0	0	0	0	1	0
ENSMUSG00000112129	PBLD1	0	0	0	0	0	0	0	0	1	0	0	0
ENSMUSG00000112141	4930563J15R	0	1	0	0	0	0	0	0	0	0	0	0
ENSMUSG00000112144	GM47861	0	0	0	0	0	0	1	1	2	0	0	1
ENSMUSG00000112145	GM6218	0	1	0	0	0	1	0	0	1	1	0	1
ENSMUSG00000112150	9530018F02	0	0	0	0	0	0	1	0	0	0	2	0
ENSMUSG00000112155	4930477N07	0	0	0	0	0	0	0	1	0	0	0	1
ENSMUSG00000112160	BC024063	2	2	2	1	2	0	5	4	3	5	2	4
ENSMUSG00000112195	GM35405	0	0	1	0	0	0	2	0	0	0	0	1
ENSMUSG00000112203	AC153506.1	0	0	1	1	0	0	0	2	0	2	0	0
ENSMUSG00000112214	CYP3A61-PS1	0	0	1	0	0	0	0	1	0	0	1	0
ENSMUSG00000112220	GM36208	0	1	0	0	0	0	1	0	0	0	3	0
ENSMUSG00000112222	TYMS-PS	0	0	0	0	0	0	0	1	0	0	0	0
ENSMUSG00000112229	GM48086	1	1	0	0	1	1	1	0	1	2	1	2
ENSMUSG00000112237	AC153911.1	1	0	0	0	1	1	2	4	0	1	1	0
ENSMUSG00000112249	GM30262	0	0	0	0	0	0	0	0	0	0	1	0
ENSMUSG00000112254	GM47690	0	0	0	0	0	0	0	0	0	0	1	0
ENSMUSG00000112255	GM47594	0	0	2	0	0	0	1	1	0	0	0	0
ENSMUSG00000112259	GM17980	0	0	0	0	0	0	0	0	0	0	0	1
ENSMUSG00000112265	GM34983	0	0	0	0	0	0	0	0	0	0	1	0
ENSMUSG00000112276	5033421B08	0	0	0	1	0	0	0	0	0	0	0	0
ENSMUSG00000112278	GM30025	0	2	0	0	3	1	3	3	3	0	2	0
ENSMUSG00000112280	A830082N09	0	0	0	2	0	0	3	3	0	6	5	3
ENSMUSG00000112294	GM4129	0	0	0	0	3	0	0	0	0	1	0	0
ENSMUSG00000112298	GM48088	0	0	0	0	0	0	0	0	0	1	2	1
ENSMUSG00000112300	GM47976	0	0	0	0	0	0	1	1	0	0	0	0
ENSMUSG00000112302	GM48226	0	1	0	1	3	1	8	4	2	6	6	4
ENSMUSG00000112308	GM48512	0	0	0	0	0	0	0	0	0	1	0	0
ENSMUSG00000112309	GM48353	0	0	0	0	0	0	0	0	1	0	1	0
ENSMUSG00000112314	AC155712.1	0	0	0	0	0	1	4	2	2	2	1	0
ENSMUSG00000112317	GM47580	0	0	0	0	0	0	1	0	0	1	0	0
ENSMUSG00000112324	GM47939	0	0	0	0	0	0	0	0	0	0	0	1

ENSMUSG00000112332	4930466K18F	0	0	0	3	0	0	5	6	10	4	1	7
ENSMUSG00000112341	GM4796	0	0	0	0	0	0	0	0	0	0	0	1
ENSMUSG00000112346	GM48768	2	0	3	3	0	1	5	1	1	2	2	5
ENSMUSG00000112347	GM47257	0	0	0	0	0	1	0	0	0	0	0	0
ENSMUSG00000112348	GM36634	0	0	0	1	0	0	0	0	0	0	0	0
ENSMUSG00000112351	GM48326	0	0	0	0	0	0	0	0	0	0	1	0
ENSMUSG00000112368	AC166323.1	0	0	1	0	0	1	0	3	1	2	1	2
ENSMUSG00000112375	GM38560	0	0	0	0	0	0	0	0	0	1	0	0
ENSMUSG00000112381	GM8170	0	1	2	2	0	0	0	0	0	0	1	0
ENSMUSG00000112388	AI463170	1	0	0	0	0	0	0	0	0	1	0	0
ENSMUSG00000112391	GM48230	0	0	0	0	0	0	1	0	0	3	0	1
ENSMUSG00000112393	GM48655	0	0	0	0	0	0	1	0	0	2	1	0
ENSMUSG00000112398	9230102K24F	0	0	0	0	0	0	0	0	0	1	0	0
ENSMUSG00000112406	4921515L22R	0	0	0	0	0	0	0	0	0	0	1	0
ENSMUSG00000112411	GM19337	0	0	0	0	0	0	3	0	0	1	0	0
ENSMUSG00000112413	GM6867	0	0	1	0	1	0	2	1	0	0	0	1
ENSMUSG00000112417	A430028G04I	0	0	0	0	0	0	0	2	0	0	0	0
ENSMUSG00000112426	GM47200	0	1	0	0	0	0	0	0	0	0	0	0
ENSMUSG00000112429	GM47093	0	0	0	0	0	0	0	0	1	0	0	1
ENSMUSG00000112430	GM30624	0	0	0	0	0	0	0	1	0	0	0	0
ENSMUSG00000112442	GM47992	0	1	0	0	0	0	1	0	0	0	0	1
ENSMUSG00000112458	GM40773	0	0	0	0	0	0	0	0	1	0	0	1
ENSMUSG00000112459	GM47689	1	1	1	0	0	0	0	0	1	1	1	3
ENSMUSG00000112462	GM48772	0	0	0	0	0	0	0	1	0	0	1	0
ENSMUSG00000112471	GM5779	0	0	0	0	0	0	0	0	0	0	1	0
ENSMUSG00000112474	GM46209	0	0	0	0	1	0	0	2	3	2	0	2
ENSMUSG00000112478	GM47761	0	0	0	0	0	0	0	0	1	0	0	0
ENSMUSG00000112489	AC165249.1	0	0	0	0	0	0	0	0	0	0	0	1
ENSMUSG00000112490	GM48068	0	0	0	0	1	0	0	1	0	2	0	1
ENSMUSG00000112505	GM48610	0	0	0	0	0	0	1	0	0	0	0	0
ENSMUSG00000112510	GM47566	1	4	2	0	1	3	15	1	5	9	4	10
ENSMUSG00000112515	GM4928	0	0	0	0	0	0	1	0	1	0	0	1
ENSMUSG00000112533	GM20758	0	0	0	0	0	0	0	0	0	1	0	0
ENSMUSG00000112534	GM47940	0	0	0	0	0	0	0	0	1	0	0	0
ENSMUSG00000112545	1300014J16R	0	0	0	0	0	0	0	1	0	0	1	0
ENSMUSG00000112550	GM6627	0	0	0	0	0	0	0	1	0	0	0	1
ENSMUSG00000112553	GM4556	0	0	0	0	0	0	0	0	1	0	1	0
ENSMUSG00000112567	GM32899	5	3	7	9	8	15	24	26	34	33	18	26
ENSMUSG00000112572	GM7530	0	0	0	0	0	0	0	0	1	0	0	0
ENSMUSG00000112574	GM5750	0	1	0	1	0	0	1	2	4	1	3	0
ENSMUSG00000112575	GM31849	0	0	0	0	0	0	0	0	0	0	0	1
ENSMUSG00000112582	RPL19-PS2	0	0	0	0	0	0	0	1	0	0	0	0
ENSMUSG00000112587	GM47769	1	0	0	0	0	1	0	1	1	2	3	3
ENSMUSG00000112593	GM48882	0	0	0	0	1	1	0	0	0	1	0	1
ENSMUSG00000112594	GM48333	0	0	0	0	0	0	0	0	0	1	0	0
ENSMUSG00000112605	GM7476	0	0	0	0	0	0	1	0	0	0	0	1
ENSMUSG00000112611	GLIPR1L3	0	0	0	0	0	0	0	1	0	0	0	0
ENSMUSG00000112613	GM48175	0	0	0	0	0	1	0	0	0	0	0	0
ENSMUSG00000112616	GM47434	0	0	0	0	0	0	1	0	0	0	0	0
ENSMUSG00000112621	GM5173	0	0	0	0	0	1	0	0	1	0	0	0
ENSMUSG00000112627	4933412E12F	1	1	0	0	0	2	4	3	1	4	2	1
ENSMUSG00000112637	GM48225	0	1	0	1	0	1	2	4	1	2	2	2
ENSMUSG00000112639	AC134329.1	1	2	0	0	1	1	4	1	4	3	3	5
ENSMUSG00000112640	GM32687	0	0	0	0	0	0	1	0	0	0	1	0
ENSMUSG00000112643	GM19688	0	0	0	0	0	0	0	0	1	0	0	0
ENSMUSG00000112646	GM5654	0	0	0	0	0	1	0	0	0	0	0	1
ENSMUSG00000112649	GM47647	0	0	0	0	0	0	0	0	1	0	1	0
ENSMUSG00000112652	AC160411.1	0	0	0	0	0	1	2	4	1	4	2	2

ENSMUSG00000112654	4930455C13F	0	0	0	0	0	0	1	0	1	0	0	0
ENSMUSG00000112669	GM48689	0	0	0	0	0	0	0	0	1	0	0	0
ENSMUSG00000112673	GM7413	0	0	0	0	0	0	0	1	0	1	0	1
ENSMUSG00000112684	GM47958	0	0	0	0	0	0	0	0	0	0	1	0
ENSMUSG00000112691	GM8681	0	0	1	0	0	0	1	0	2	1	1	2
ENSMUSG00000112693	GM5512	0	0	0	0	0	0	0	0	0	1	0	0
ENSMUSG00000112698	GM7525	0	0	0	0	0	0	0	0	0	1	0	0
ENSMUSG00000112708	GM8255	0	0	0	0	0	0	0	1	0	0	0	0
ENSMUSG00000112716	4930471E19F	0	0	0	0	1	0	0	0	0	0	0	0
ENSMUSG00000112727	GM17794	0	0	0	0	0	0	1	0	0	0	0	1
ENSMUSG00000112728	GM48903	0	0	0	0	0	0	0	0	0	1	0	0
ENSMUSG00000112734	GM47644	0	0	0	1	0	0	0	0	0	0	0	0
ENSMUSG00000112736	GM48718	0	0	0	0	0	0	0	0	0	0	0	1
ENSMUSG00000112742	GM31793	0	1	1	1	1	0	1	0	0	1	0	1
ENSMUSG00000112744	GM48880	0	0	0	0	0	0	0	0	0	0	2	1
ENSMUSG00000112758	GM36172	0	0	0	0	1	0	0	0	0	0	0	0
ENSMUSG00000112759	GM47615	0	0	0	1	0	0	1	2	2	1	2	0
ENSMUSG00000112765	9430078K24F	0	0	0	0	0	0	0	0	0	1	0	0
ENSMUSG00000112779	GM18029	0	0	0	0	0	0	2	0	0	0	0	0
ENSMUSG00000112782	GM7940	0	0	0	0	0	0	1	0	0	0	0	0
ENSMUSG00000112784	GM34868	0	0	0	0	0	0	0	0	1	0	0	0
ENSMUSG00000112790	GM33378	1	0	1	0	0	0	1	0	2	5	1	0
ENSMUSG00000112794	GM48878	0	0	0	1	0	0	0	1	0	0	0	0
ENSMUSG00000112801	GM48034	0	0	0	0	0	0	1	0	0	0	0	0
ENSMUSG00000112805	GM34574	0	0	0	0	0	1	0	0	0	0	0	0
ENSMUSG00000112806	GM48146	1	0	2	1	0	0	3	2	1	4	5	4
ENSMUSG00000112808	GM4739	0	0	0	0	0	0	4	0	0	2	1	1
ENSMUSG00000112816	GM48798	0	0	0	0	0	0	1	0	0	0	0	0
ENSMUSG00000112819	GM46332	0	0	0	0	0	0	0	1	1	0	0	0
ENSMUSG00000112820	GM48298	0	0	0	0	0	0	1	0	0	0	0	0
ENSMUSG00000112822	A130012E19F	0	1	0	2	1	1	2	6	6	3	3	3
ENSMUSG00000112825	GM9118	0	0	0	0	0	0	0	2	0	0	1	2
ENSMUSG00000112841	GM48752	0	0	0	0	0	0	0	0	0	1	0	0
ENSMUSG00000112847	GM47573	0	0	0	0	1	0	1	0	0	0	0	1
ENSMUSG00000112848	GM32023	1	0	1	1	1	0	6	4	3	2	2	0
ENSMUSG00000112873	GM48877	0	0	0	0	0	0	0	1	0	0	0	0
ENSMUSG00000112874	GM34297	0	0	0	0	0	0	0	0	0	0	1	0
ENSMUSG00000112879	GM47726	1	0	0	0	0	0	0	1	0	0	1	0
ENSMUSG00000112880	GM20337	0	0	0	0	0	0	0	0	0	0	0	1
ENSMUSG00000112881	AC139376.1	0	1	0	0	0	0	0	0	1	0	1	0
ENSMUSG00000112891	GM47391	0	0	0	0	0	0	0	0	0	1	0	0
ENSMUSG00000112895	GM47567	0	2	1	1	1	1	2	3	1	4	0	2
ENSMUSG00000112907	GM47015	4	4	12	5	7	3	44	35	29	40	32	39
ENSMUSG00000112908	GM7392	0	0	0	0	0	0	0	0	0	0	2	0
ENSMUSG00000112909	GM10120	0	0	0	0	0	0	0	0	0	1	0	1
ENSMUSG00000112922	GM47572	0	1	0	0	0	0	0	1	0	3	1	0
ENSMUSG00000112925	GM31763	0	0	0	0	1	0	0	0	0	2	0	1
ENSMUSG00000112926	GM7172	0	0	0	0	0	0	1	0	0	0	0	0
ENSMUSG00000112927	GM48332	0	0	0	0	0	0	0	0	0	1	1	1
ENSMUSG00000112928	GM47710	0	0	0	0	0	0	1	0	0	0	0	0
ENSMUSG00000112932	GM48308	0	0	0	0	0	0	0	0	1	0	0	0
ENSMUSG00000112944	GM48885	0	0	0	2	0	0	2	0	1	1	0	3
ENSMUSG00000112946	GM9280	0	0	0	0	0	0	0	0	0	0	1	0
ENSMUSG00000112947	GM47493	0	0	0	0	0	0	0	0	0	0	0	1
ENSMUSG00000112948	GM48871	0	1	0	0	0	0	0	0	0	0	0	0
ENSMUSG00000112952	GM48336	0	0	0	0	0	0	1	0	0	0	0	0
ENSMUSG00000112955	GM48889	0	0	0	0	0	0	1	3	2	1	3	0
ENSMUSG00000112963	GM6093	0	0	0	0	0	0	1	0	0	0	0	0

ENSMUSG00000112964	E430024I08R	2	8	1	3	0	4	15	13	5	41	6	9
ENSMUSG00000112980	D430020J02R	1	0	1	1	1	2	3	0	1	4	6	3
ENSMUSG00000112990	GM47372	0	0	1	0	0	0	1	0	0	0	0	0
ENSMUSG00000112999	9530050K03R	0	0	0	0	0	0	0	0	2	2	0	0
ENSMUSG00000113002	GM48620	0	0	0	0	0	0	0	0	1	0	0	3
ENSMUSG00000113003	GM47138	0	0	1	0	0	0	1	0	1	1	0	1
ENSMUSG00000113012	GM47196	0	0	0	0	0	0	0	0	1	1	0	0
ENSMUSG00000113018	GM9318	0	0	0	0	0	0	0	0	0	0	1	0
ENSMUSG00000113019	GM47467	0	0	0	0	0	0	0	0	1	1	0	2
ENSMUSG00000113035	5830428M24	0	0	0	1	0	0	3	5	1	2	1	2
ENSMUSG00000113041	GM48302	0	0	0	1	0	1	0	0	0	0	0	0
ENSMUSG00000113043	GM7753	0	0	0	0	0	0	0	1	0	0	0	0
ENSMUSG00000113045	GM48621	0	0	0	1	0	0	0	0	0	0	0	0
ENSMUSG00000113047	GM47469	1	1	1	2	1	3	3	3	2	9	0	5
ENSMUSG00000113052	GM31513	0	0	0	0	0	0	2	1	0	0	0	1
ENSMUSG00000113059	GM46344	3	1	2	2	2	0	23	10	14	10	20	9
ENSMUSG00000113061	GM11361	2	2	3	1	1	0	0	6	2	3	4	4
ENSMUSG00000113065	GM48870	0	1	0	0	0	1	0	1	1	1	2	1
ENSMUSG00000113067	GM47166	0	0	0	0	0	0	1	0	0	0	0	0
ENSMUSG00000113068	GM48133	0	0	0	0	0	0	0	0	0	0	0	1
ENSMUSG00000113070	GM48420	0	0	0	0	0	0	0	1	2	1	0	1
ENSMUSG00000113071	GM6353	0	0	1	0	0	0	0	0	0	0	0	0
ENSMUSG00000113072	GM47820	0	0	1	0	0	0	0	1	0	1	1	0
ENSMUSG00000113075	RPL7A-PS1	0	0	0	0	0	0	0	0	0	1	0	0
ENSMUSG00000113076	GM47088	0	0	0	0	0	0	1	0	0	0	0	0
ENSMUSG00000113078	GM48734	0	0	0	0	0	0	1	0	0	0	0	1
ENSMUSG00000113086	GM47376	0	0	0	0	0	0	0	0	0	1	1	0
ENSMUSG00000113090	GM48190	0	0	0	0	0	0	0	1	0	0	0	0
ENSMUSG00000113102	A930040O22I	0	0	0	0	0	0	1	0	0	0	0	0
ENSMUSG00000113104	GM47806	0	0	0	0	0	0	0	0	2	0	0	0
ENSMUSG00000113106	GM47139	0	0	0	0	0	1	0	0	1	0	0	2
ENSMUSG00000113113	GM2614	1	0	0	0	1	0	2	0	0	2	1	1
ENSMUSG00000113119	GM48883	0	0	0	0	0	0	1	0	0	1	0	0
ENSMUSG00000113126	GM47588	0	0	0	1	0	0	1	2	0	2	1	0
ENSMUSG00000113128	GM47813	0	0	1	0	0	0	2	0	3	2	1	2
ENSMUSG00000113136	GM19951	0	0	0	0	0	0	1	0	0	0	0	1
ENSMUSG00000113137	AC157515.1	0	0	0	0	1	0	1	0	0	1	0	0
ENSMUSG00000113140	GM48624	0	0	0	0	0	0	1	1	0	0	0	0
ENSMUSG00000113149	AC099934.1	0	0	1	0	1	1	2	5	3	4	3	3
ENSMUSG00000113151	GM48690	0	0	0	0	1	0	5	2	3	2	7	4
ENSMUSG00000113154	GM48713	0	0	0	0	1	0	0	0	0	0	0	0
ENSMUSG00000113168	GM7614	0	0	0	0	0	0	1	0	0	1	0	1
ENSMUSG00000113183	GM47664	0	1	1	2	0	0	4	2	2	4	3	3
ENSMUSG00000113190	GM47545	0	0	0	0	0	0	0	0	1	0	0	0
ENSMUSG00000113195	GM48586	0	0	0	0	0	0	0	0	0	1	0	1
ENSMUSG00000113196	AC159193.1	0	0	0	1	0	2	1	5	0	3	0	1
ENSMUSG00000113198	GM6988	29	26	34	30	34	34	77	102	85	98	95	101
ENSMUSG00000113204	GM46430	0	0	1	3	0	2	7	7	2	17	2	7
ENSMUSG00000113209	1700081N11I	0	0	0	0	0	0	0	2	1	1	1	2
ENSMUSG00000113210	4930474N09I	0	0	0	0	0	0	0	0	1	0	0	0
ENSMUSG00000113211	4921525O09I	1	0	0	0	0	0	5	2	5	1	2	5
ENSMUSG00000113222	GM48557	0	0	0	0	0	0	1	1	0	0	0	1
ENSMUSG00000113237	GM6109	0	0	0	0	0	0	0	1	0	0	0	0
ENSMUSG00000113238	GM47251	0	0	0	0	0	0	1	1	0	0	0	1
ENSMUSG00000113249	GM33312	1	2	1	1	3	2	10	10	7	14	4	11
ENSMUSG00000113250	GM48585	1	1	1	1	0	0	3	1	1	1	2	0
ENSMUSG00000113255	TES3-PS	0	0	0	0	2	1	4	4	4	5	5	1
ENSMUSG00000113262	GM48551	0	0	0	0	0	0	2	0	0	1	0	0

ENSMUSG00000113263	GM4811	0	0	0	0	0	0	0	0	0	0	1	0
ENSMUSG00000113268	4930477G07I	0	1	0	1	0	0	0	0	1	0	1	0
ENSMUSG00000113277	GM18116	0	0	0	0	0	1	0	0	0	0	0	0
ENSMUSG00000113286	AC211878.2	0	0	0	0	0	0	1	0	0	0	0	0
ENSMUSG00000113309	GM48408	0	0	0	0	0	1	0	0	0	0	0	0
ENSMUSG00000113310	GM47884	0	0	0	0	1	0	0	0	0	0	0	0
ENSMUSG00000113311	GM47428	0	0	0	0	0	0	1	0	0	0	0	0
ENSMUSG00000113313	GM32036	6	8	4	2	8	5	11	9	4	9	6	8
ENSMUSG00000113318	GM18113	1	0	0	0	0	0	0	0	0	1	0	1
ENSMUSG00000113326	GM47586	0	0	0	0	0	0	0	1	0	0	0	1
ENSMUSG00000113328	GM47260	1	4	1	1	2	2	10	5	4	9	7	6
ENSMUSG00000113341	9430031K09F	0	0	0	0	0	0	0	0	0	0	2	0
ENSMUSG00000113349	GM48369	2	0	0	0	1	1	4	3	6	2	2	3
ENSMUSG00000113352	GM34639	0	1	0	0	0	0	0	0	0	0	0	0
ENSMUSG00000113355	GM48888	0	0	0	0	0	0	0	0	0	0	0	1
ENSMUSG00000113357	GM19195	0	0	1	1	1	0	1	0	2	0	0	2
ENSMUSG00000113361	AC152827.1	0	0	0	0	0	0	1	0	2	0	0	0
ENSMUSG00000113362	GM40557	0	0	1	0	0	0	0	0	0	0	0	0
ENSMUSG00000113368	GM10054	0	0	0	0	0	0	1	1	0	1	0	2
ENSMUSG00000113379	GM46436	0	0	0	0	0	0	1	0	0	0	1	0
ENSMUSG00000113385	GM48405	0	0	0	0	1	0	0	1	0	0	0	0
ENSMUSG00000113387	AC162181.1	0	0	0	0	0	0	0	0	0	0	1	0
ENSMUSG00000113389	GM9512	0	0	0	1	0	0	1	2	0	1	1	1
ENSMUSG00000113392	GM36262	0	0	0	0	0	0	0	0	0	0	0	1
ENSMUSG00000113405	GM4935	0	0	0	0	0	0	0	0	0	0	1	0
ENSMUSG00000113423	GM3791	0	0	0	0	0	0	0	0	1	0	0	0
ENSMUSG00000113424	GM48161	0	1	0	0	0	0	0	0	1	0	0	0
ENSMUSG00000113425	GM48653	1	0	1	0	0	0	2	0	4	0	1	1
ENSMUSG00000113427	AC163040.1	0	0	0	0	0	0	1	0	3	0	1	1
ENSMUSG00000113430	GM47086	0	0	0	0	0	1	0	0	2	0	0	0
ENSMUSG00000113432	AC154828.1	0	0	0	0	0	0	0	1	0	0	0	0
ENSMUSG00000113440	GM46404	0	0	0	0	0	0	0	0	0	0	0	1
ENSMUSG00000113450	ZFP935	0	1	1	1	2	2	7	11	6	4	6	4
ENSMUSG00000113455	GM48825	0	0	0	0	0	0	0	0	0	0	1	0
ENSMUSG00000113459	GM30655	0	0	0	0	0	0	0	0	0	1	0	0
ENSMUSG00000113467	AC138767.1	0	0	0	0	0	0	0	0	0	2	1	2
ENSMUSG00000113469	GM7065	0	0	0	0	0	0	0	0	0	1	1	0
ENSMUSG00000113473	GM7045	1	0	0	0	1	1	7	4	0	11	1	1
ENSMUSG00000113511	GM19046	0	0	0	0	0	0	0	1	0	0	0	0
ENSMUSG00000113514	GM18960	0	0	0	0	0	0	0	0	1	0	0	1
ENSMUSG00000113515	AL645745.1	1	0	0	0	0	0	0	0	0	0	0	0
ENSMUSG00000113519	GM47541	0	0	0	0	0	0	0	0	0	0	0	1
ENSMUSG00000113527	GM48079	0	0	0	0	0	0	0	2	0	1	0	0
ENSMUSG00000113528	GM32699	0	0	0	0	0	0	0	1	0	0	1	0
ENSMUSG00000113529	GM47484	2	0	2	1	2	3	4	3	4	4	4	4
ENSMUSG00000113542	GM19154	0	0	0	0	1	0	0	0	0	0	0	0
ENSMUSG00000113555	GM10095	1	0	1	1	7	3	17	15	15	21	9	16
ENSMUSG00000113557	GM47657	0	0	0	0	0	0	1	0	0	0	0	0
ENSMUSG00000113568	GM46369	0	0	0	0	0	0	0	0	0	0	0	1
ENSMUSG00000113571	GM48137	0	0	0	0	0	0	1	0	0	0	0	0
ENSMUSG00000113576	GM47658	0	0	0	0	0	0	1	0	0	0	0	0
ENSMUSG00000113577	GM19238	0	0	0	0	0	0	0	0	0	1	1	0
ENSMUSG00000113585	GM19605	0	1	1	0	0	1	2	2	1	3	3	2
ENSMUSG00000113587	GM36287	0	0	0	0	0	0	1	0	0	0	0	0
ENSMUSG00000113596	GM48452	0	0	1	0	0	0	0	2	0	0	0	2
ENSMUSG00000113601	GM48735	0	0	0	0	0	0	0	1	0	0	2	1
ENSMUSG00000113603	GM47004	0	0	0	0	0	0	2	0	0	1	1	0
ENSMUSG00000113617	GM8655	1	0	0	0	0	0	0	0	0	0	0	0

ENSMUSG00000113623	GM48691	0	0	0	0	0	0	0	0	0	1	0	1
ENSMUSG00000113625	GM3379	1	1	0	0	0	0	0	0	0	2	4	4
ENSMUSG00000113626	GM7240	0	2	0	1	1	1	5	7	2	3	3	2
ENSMUSG00000113643	GM40598	0	0	0	0	0	0	0	0	1	0	0	0
ENSMUSG00000113647	GM47210	0	0	0	0	0	0	0	1	0	0	0	0
ENSMUSG00000113649	GM47665	0	0	0	0	0	0	0	0	0	0	1	0
ENSMUSG00000113650	GM47826	0	0	0	0	0	1	1	0	0	1	0	0
ENSMUSG00000113656	GM47370	0	0	0	0	0	0	0	0	0	1	0	0
ENSMUSG00000113662	GM35558	0	0	0	0	0	0	1	0	0	1	0	0
ENSMUSG00000113666	GM35725	0	0	0	0	0	0	0	0	1	0	0	0
ENSMUSG00000113669	AC163354.1	0	0	0	0	0	0	0	1	1	1	0	0
ENSMUSG00000113679	GM10432	2	3	0	0	4	4	19	15	13	12	9	4
ENSMUSG00000113684	GM48418	0	0	0	0	0	0	0	0	0	1	0	0
ENSMUSG00000113687	AC123705.1	0	0	0	0	0	0	1	0	0	1	0	0
ENSMUSG00000113688	A930027P06F	0	0	0	0	0	0	0	0	0	0	1	0
ENSMUSG00000113690	GM36501	0	0	0	0	0	0	0	0	0	1	0	0
ENSMUSG00000113701	B230303A05F	0	1	0	0	0	0	0	1	0	1	0	0
ENSMUSG00000113707	GM10457	0	0	0	0	0	0	1	1	0	1	1	0
ENSMUSG00000113708	GM47584	0	0	0	0	0	1	0	0	1	0	0	0
ENSMUSG00000113716	GM47518	0	1	0	0	0	0	0	0	0	0	0	0
ENSMUSG00000113722	SNHG10	3	4	8	6	4	6	19	27	24	21	18	23
ENSMUSG00000113725	GM48358	1	6	1	4	3	3	25	5	8	15	14	9
ENSMUSG00000113730	GM47805	0	0	0	1	0	0	0	0	0	0	0	0
ENSMUSG00000113737	BB123696	0	0	1	0	0	0	0	0	0	0	0	0
ENSMUSG00000113741	GM48236	1	0	0	0	0	0	1	1	0	1	0	1
ENSMUSG00000113746	GM48242	0	0	0	0	0	0	0	0	1	0	0	0
ENSMUSG00000113748	GM18628	0	0	0	0	0	0	0	1	0	0	0	0
ENSMUSG00000113750	GM5628	0	0	0	0	0	0	1	0	0	0	0	0
ENSMUSG00000113752	GM47817	1	0	0	0	1	1	4	1	1	3	2	1
ENSMUSG00000113756	GM18025	0	0	0	0	0	0	0	0	0	0	2	0
ENSMUSG00000113771	GM36529	0	0	0	0	1	0	0	0	0	0	0	0
ENSMUSG00000113773	GM8278	0	0	0	0	0	0	0	0	0	1	0	0
ENSMUSG00000113774	GM36236	0	0	0	0	0	0	0	0	0	0	0	1
ENSMUSG00000113775	GM48027	0	1	0	0	0	0	3	4	0	2	2	1
ENSMUSG00000113776	GM19327	1	0	2	0	2	2	1	1	1	0	1	1
ENSMUSG00000113780	GM33195	0	1	0	0	0	0	1	4	3	1	2	3
ENSMUSG00000113789	GM47322	0	0	0	0	0	0	2	2	1	1	0	1
ENSMUSG00000113790	GM17872	0	0	0	0	0	0	0	0	2	1	0	0
ENSMUSG00000113792	GM10933	0	0	0	0	0	0	0	0	2	0	0	0
ENSMUSG00000113811	GM47882	0	1	0	0	0	0	0	0	0	0	0	0
ENSMUSG00000113812	GM48699	0	0	0	1	0	0	3	1	0	0	1	0
ENSMUSG00000113816	GM47003	1	2	0	0	0	1	2	1	0	3	2	2
ENSMUSG00000113831	AC163032.1	0	1	4	0	2	2	4	8	3	11	8	9
ENSMUSG00000113836	AC124426.1	1	2	0	2	1	0	0	4	1	3	0	2
ENSMUSG00000113841	GM48701	0	0	0	0	0	0	0	1	0	0	1	2
ENSMUSG00000113842	GM48138	0	0	0	0	0	0	0	0	0	0	0	1
ENSMUSG00000113843	GM6129	0	0	0	0	0	1	1	0	0	1	0	0
ENSMUSG00000113845	GM39473	0	1	0	1	0	0	0	0	0	0	0	0
ENSMUSG00000113848	GM16419	0	0	0	0	0	0	2	3	0	0	0	0
ENSMUSG00000113852	GM48062	0	0	0	0	0	0	1	3	0	4	0	1
ENSMUSG00000113853	GM47583	0	0	0	0	0	0	0	0	0	0	0	1
ENSMUSG00000113854	GM2099	0	0	1	0	0	0	0	0	2	1	0	0
ENSMUSG00000113859	GM7979	0	0	0	0	0	0	0	1	0	0	0	0
ENSMUSG00000113869	GM48869	0	0	0	0	0	1	0	0	0	0	0	1
ENSMUSG00000113877	GM47208	0	0	0	0	0	0	0	1	0	1	0	1
ENSMUSG00000113878	GM19045	0	0	0	0	0	0	0	0	0	1	0	0
ENSMUSG00000113880	A030005L19F	0	1	0	1	0	0	0	0	1	0	0	1
ENSMUSG00000113882	GM48550	1	1	0	1	0	0	6	8	1	4	4	5

ENSMUSG00000113884	GM2270	0	0	0	0	0	0	0	0	1	0	0	0
ENSMUSG00000113885	GM47802	0	0	0	0	1	0	0	0	0	0	0	0
ENSMUSG00000113890	GM48368	0	0	0	0	0	0	0	0	0	0	1	0
ENSMUSG00000113894	GM48838	1	3	0	2	1	1	9	3	4	7	5	3
ENSMUSG00000113902	AC121965.1	18	12	27	25	31	26	162	182	115	154	126	131
ENSMUSG00000113905	GM48872	0	0	0	0	0	0	2	0	0	0	2	1
ENSMUSG00000113907	GM47167	0	0	1	0	0	0	0	0	0	0	0	1
ENSMUSG00000113910	GM48286	1	0	0	0	0	0	3	1	1	0	0	0
ENSMUSG00000113917	GM48382	0	0	0	0	0	0	0	0	2	1	2	0
ENSMUSG00000113918	GM6566	0	1	0	0	0	0	1	1	0	1	0	2
ENSMUSG00000113922	GM48887	0	0	0	1	0	1	1	3	1	3	1	1
ENSMUSG00000113931	GM5928	0	0	0	0	0	0	1	0	2	3	1	0
ENSMUSG00000113941	GM35755	0	0	0	0	0	0	0	0	0	0	1	0
ENSMUSG00000113948	RPL17-PS3	6	10	10	12	13	8	16	48	34	40	28	31
ENSMUSG00000113949	SCAMP4	0	0	0	0	0	0	1	0	0	0	0	0
ENSMUSG00000113960	4933412O06I	0	0	0	0	0	0	0	0	0	0	0	1
ENSMUSG00000113961	GM48498	0	0	0	1	0	0	0	0	0	0	0	0
ENSMUSG00000113965	A730091E23F	0	0	0	0	0	0	1	0	1	2	0	0
ENSMUSG00000113975	GM36372	0	0	0	0	0	0	0	1	0	0	0	0
ENSMUSG00000113984	GM48890	0	0	0	1	0	0	1	1	1	0	1	0
ENSMUSG00000113985	9130015A21F	0	0	0	0	0	0	0	0	0	1	0	0
ENSMUSG00000114003	GM9616	0	0	1	1	0	0	1	4	1	0	2	4
ENSMUSG00000114016	GM47258	0	1	0	0	0	0	1	3	1	2	1	1
ENSMUSG00000114019	GM47155	0	0	0	0	0	0	1	1	0	1	2	0
ENSMUSG00000114033	GM47705	0	0	0	0	0	0	0	0	0	0	0	1
ENSMUSG00000114034	GM9267	0	0	0	0	0	1	0	1	0	0	0	0
ENSMUSG00000114045	GM34220	3	4	0	0	2	1	5	6	5	10	7	7
ENSMUSG00000114062	GM48313	0	0	0	1	0	0	0	0	0	0	0	0
ENSMUSG00000114063	GM47555	0	0	0	0	0	0	0	0	0	1	0	0
ENSMUSG00000114070	GM47727	0	0	0	0	0	0	0	0	0	3	2	0
ENSMUSG00000114081	GM48904	0	0	0	0	1	0	0	0	0	1	1	0
ENSMUSG00000114094	GM48375	0	0	0	1	0	0	0	0	0	0	0	0
ENSMUSG00000114095	GM47424	0	0	0	0	0	0	0	1	0	0	0	0
ENSMUSG00000114096	GM48641	0	0	0	0	0	0	0	0	1	0	1	0
ENSMUSG00000114103	GM8557	2	4	1	1	2	3	12	11	5	15	8	6
ENSMUSG00000114104	GM47585	0	0	1	0	1	0	0	0	0	1	0	0
ENSMUSG00000114110	GM46379	0	0	0	0	0	0	1	0	2	0	0	0
ENSMUSG00000114113	GM47639	0	0	0	0	0	0	1	1	1	0	1	2
ENSMUSG00000114133	GM20075	1	1	0	0	0	0	4	0	1	2	0	0
ENSMUSG00000114135	GM7252	0	0	0	0	0	0	0	0	0	0	1	0
ENSMUSG00000114138	GM36423	1	2	3	4	2	4	23	25	8	29	16	16
ENSMUSG00000114150	GM46367	0	0	0	0	0	0	0	1	0	0	0	0
ENSMUSG00000114152	GM5374	0	1	0	0	0	0	0	0	0	0	0	1
ENSMUSG00000114155	GM7986	0	0	0	0	0	0	0	0	0	0	1	0
ENSMUSG00000114169	GM47075	0	0	0	1	0	0	0	0	0	0	0	0
ENSMUSG00000114172	GM36298	1	0	0	0	1	1	0	4	3	3	0	1
ENSMUSG00000114179	EIF1-PS2	0	0	0	0	0	0	0	0	0	0	0	1
ENSMUSG00000114184	GM7143	1	1	0	1	1	1	0	1	0	0	0	0
ENSMUSG00000114190	4930556H04F	0	0	0	0	0	0	0	0	0	0	0	1
ENSMUSG00000114193	GM48139	0	0	0	0	1	0	0	1	0	1	0	1
ENSMUSG00000114194	GM3800	0	0	0	0	0	0	1	0	1	1	0	1
ENSMUSG00000114197	GM47743	4	4	3	5	5	3	10	10	18	13	8	5
ENSMUSG00000114202	GM18078	0	0	0	0	0	1	1	0	0	1	1	0
ENSMUSG00000114206	GM30551	0	0	0	0	0	0	1	0	0	1	0	0
ENSMUSG00000114210	A330084C13F	0	1	0	0	0	0	1	0	0	0	1	0
ENSMUSG00000114213	A430090L17F	0	0	0	0	0	0	0	0	0	0	1	0
ENSMUSG00000114220	GM10353	0	0	0	1	0	0	1	1	1	3	0	0
ENSMUSG00000114241	GM30108	1	0	0	2	0	0	2	3	5	5	9	7

ENSMUSG00000114242	GM31219	0	1	0	0	0	0	0	0	0	2	1	0
ENSMUSG00000114243	GM48163	0	0	0	0	0	0	1	0	0	0	0	0
ENSMUSG00000114251	TMED10-PS	0	0	0	0	0	0	0	0	0	1	0	0
ENSMUSG00000114254	GM48067	1	0	0	0	0	0	3	0	1	1	2	0
ENSMUSG00000114255	GM10734	0	0	0	0	0	0	0	1	0	0	1	0
ENSMUSG00000114264	GM48095	0	0	0	0	0	0	0	0	0	0	0	1
ENSMUSG00000114270	GM48746	0	0	0	0	0	0	0	0	0	0	1	0
ENSMUSG00000114279	HIST1H2BM	1	0	0	0	0	0	2	0	1	1	1	1
ENSMUSG00000114283	GM47057	0	0	0	0	0	0	1	0	0	0	1	0
ENSMUSG00000114285	GM47773	0	0	0	0	0	0	0	0	0	0	0	1
ENSMUSG00000114295	GM2534	0	0	0	0	0	0	0	1	0	0	0	0
ENSMUSG00000114302	GM48683	0	0	0	0	0	0	0	1	0	0	0	0
ENSMUSG00000114309	GM48295	0	0	0	0	0	0	0	0	0	1	0	0
ENSMUSG00000114312	GM18077	1	1	0	0	0	0	1	0	0	1	1	0
ENSMUSG00000114321	GM8971	0	0	1	0	0	0	0	0	1	0	0	1
ENSMUSG00000114354	GM34961	0	0	0	0	0	0	0	0	1	0	0	0
ENSMUSG00000114359	GM47816	0	2	0	1	1	2	5	1	3	1	2	2
ENSMUSG00000114362	4933433G19I	1	0	0	1	1	1	0	0	0	0	1	0
ENSMUSG00000114369	GM41077	0	0	0	0	0	0	1	0	0	0	0	0
ENSMUSG00000114384	4930455J16R	0	0	0	0	0	0	0	0	0	1	0	0
ENSMUSG00000114390	1700062C10F	0	0	0	0	0	0	1	0	0	0	0	0
ENSMUSG00000114391	GM48439	0	0	0	0	0	0	0	0	0	1	0	0
ENSMUSG00000114398	GM20763	0	1	0	0	0	0	0	0	0	0	0	0
ENSMUSG00000114400	GM47533	1	0	0	0	0	0	7	6	3	10	8	6
ENSMUSG00000114401	GM38604	1	0	0	1	3	0	0	0	1	0	0	5
ENSMUSG00000114409	GM9042	0	0	0	0	0	0	0	2	0	0	0	1
ENSMUSG00000114419	5430414B19F	1	0	0	0	0	0	1	1	0	0	3	0
ENSMUSG00000114420	AC154361.1	0	0	0	1	0	0	1	0	1	0	0	1
ENSMUSG00000114424	GM47414	0	0	0	0	0	0	0	1	1	0	0	1
ENSMUSG00000114430	GM40264	0	0	0	0	1	0	0	0	0	0	0	0
ENSMUSG00000114436	GM48283	6	17	9	9	8	12	68	39	22	61	31	37
ENSMUSG00000114440	GM47505	0	0	0	0	0	0	0	1	0	0	0	0
ENSMUSG00000114447	GM47636	0	0	0	0	0	0	0	1	0	0	1	0
ENSMUSG00000114453	GM17805	0	0	0	0	0	0	0	2	0	0	1	0
ENSMUSG00000114456	HIST1H2BH	0	0	1	1	0	0	7	4	2	3	0	3
ENSMUSG00000114458	GM47551	1	0	0	0	0	0	0	0	0	0	0	0
ENSMUSG00000114477	AC165265.1	0	0	0	0	0	0	2	1	0	0	0	1
ENSMUSG00000114493	GM47071	1	2	0	4	2	0	4	5	6	8	1	3
ENSMUSG00000114516	GM46440	0	0	0	0	0	0	1	1	0	0	1	0
ENSMUSG00000114519	GM32376	0	0	0	1	0	0	0	0	0	0	0	0
ENSMUSG00000114529	GM4939	0	0	0	0	1	0	0	0	0	0	0	0
ENSMUSG00000114535	GM47246	0	0	0	0	0	0	3	0	1	0	0	0
ENSMUSG00000114540	GM6421	0	0	0	0	0	0	0	0	1	0	0	0
ENSMUSG00000114547	AC154767.1	0	0	0	0	1	0	0	1	0	2	0	2
ENSMUSG00000114551	GM6035	1	0	0	0	0	0	1	1	0	1	0	1
ENSMUSG00000114552	GM48570	0	0	0	0	0	0	0	0	0	1	0	0
ENSMUSG00000114555	GM48706	0	0	0	0	0	0	2	1	2	0	1	0
ENSMUSG00000114575	GM47814	0	0	0	0	0	0	1	0	0	0	0	0
ENSMUSG00000114579	GM4130	0	0	0	0	0	0	0	1	0	0	1	0
ENSMUSG00000114584	GM47694	0	0	0	1	0	0	1	1	0	2	2	2
ENSMUSG00000114585	GM32401	1	2	2	2	2	0	8	6	5	5	2	4
ENSMUSG00000114586	GM31804	0	0	1	0	0	0	0	0	0	0	0	1
ENSMUSG00000114593	GM19716	0	0	0	0	0	0	0	0	0	0	0	1
ENSMUSG00000114598	D130062J10F	0	0	0	0	0	0	1	0	1	0	0	1
ENSMUSG00000114602	AC154335.1	5	7	2	0	6	5	7	7	12	18	8	5
ENSMUSG00000114605	C130051F05F	1	1	1	3	2	2	5	6	2	8	2	2
ENSMUSG00000114610	CYP2C52-PS	0	0	0	0	0	0	0	2	0	1	0	1
ENSMUSG00000114617	GM48766	1	0	0	0	0	0	0	2	1	2	0	0

ENSMUSG00000114627	B230220B15F	0	1	0	0	0	0	0	0	0	1	0	1
ENSMUSG00000114633	GM46416	0	0	0	0	1	0	1	1	0	1	0	0
ENSMUSG00000114639	GM31946	0	0	0	0	1	0	0	0	0	0	0	0
ENSMUSG00000114649	3110006O06I	0	0	1	0	0	0	0	0	0	1	1	0
ENSMUSG00000114650	GM47570	0	1	1	0	0	0	1	2	0	1	0	1
ENSMUSG00000114652	GM41043	1	0	0	2	0	0	3	1	0	4	2	1
ENSMUSG00000114661	GM48707	0	0	0	0	1	0	0	0	0	0	0	0
ENSMUSG00000114662	GM31683	0	0	0	0	0	0	0	0	1	0	0	0
ENSMUSG00000114664	GM48639	0	0	0	1	0	0	0	1	0	0	0	0
ENSMUSG00000114689	GM9465	0	0	0	0	2	0	0	1	0	1	1	0
ENSMUSG00000114691	GM48438	0	0	0	0	0	0	0	0	0	0	1	0
ENSMUSG00000114696	GM47479	1	0	1	2	0	0	1	1	0	2	0	1
ENSMUSG00000114698	RPL31-PS3	0	1	0	1	0	0	3	6	1	5	4	3
ENSMUSG00000114707	GM47558	1	2	1	1	2	0	0	5	0	3	2	1
ENSMUSG00000114709	GM47920	0	0	0	0	0	0	0	1	0	0	1	1
ENSMUSG00000114726	GM48900	0	0	0	0	0	0	0	2	0	0	0	0
ENSMUSG00000114748	GM6734	0	0	0	0	0	0	0	1	0	0	0	0
ENSMUSG00000114749	GM48442	0	1	0	0	0	0	0	1	0	1	0	0
ENSMUSG00000114754	GM8996	0	0	0	0	0	0	0	0	0	0	0	1
ENSMUSG00000114755	GALR3	0	0	0	0	0	0	0	0	0	1	0	0
ENSMUSG00000114756	GM47273	0	0	0	0	0	0	0	0	0	1	0	0
ENSMUSG00000114761	GM47242	0	0	0	0	0	0	0	0	0	0	2	0
ENSMUSG00000114771	AC163667.1	0	0	0	1	0	0	0	1	1	2	1	1
ENSMUSG00000114788	GM48899	0	0	1	0	0	0	2	1	0	1	1	2
ENSMUSG00000114794	GM47782	0	1	0	0	0	0	0	0	0	1	0	0
ENSMUSG00000114796	A930028N01I	0	0	0	0	1	1	1	0	0	0	1	0
ENSMUSG00000114797	NUPL1	4	5	9	11	12	14	48	62	45	65	45	53
ENSMUSG00000114809	ATP5C1-PS	0	0	0	0	1	0	1	1	0	0	2	0
ENSMUSG00000114812	GM48254	0	0	0	2	0	0	1	0	0	1	0	0
ENSMUSG00000114823	GM31630	0	0	0	0	0	0	0	0	1	1	0	0
ENSMUSG00000114830	GM47400	0	0	0	0	0	0	0	0	0	0	0	1
ENSMUSG00000114835	GM48194	0	0	0	0	0	0	1	3	1	1	0	0
ENSMUSG00000114836	GM18517	0	0	1	0	0	0	0	1	0	0	0	1
ENSMUSG00000114842	GM47797	0	1	0	0	0	0	2	0	1	2	0	0
ENSMUSG00000114851	AC156802.1	0	0	0	0	0	1	0	1	1	0	0	0
ENSMUSG00000114863	GM48824	0	0	0	0	0	0	2	1	2	0	0	0
ENSMUSG00000114869	GM48876	0	0	0	0	0	0	0	0	0	0	1	0
ENSMUSG00000114872	GM18759	0	0	0	0	0	0	0	0	0	1	0	0
ENSMUSG00000114888	AC140462.1	0	0	0	0	0	0	0	1	0	0	0	0
ENSMUSG00000114893	GM48684	0	0	0	0	0	0	0	0	1	0	3	5
ENSMUSG00000114907	GM47794	0	0	0	0	0	0	2	1	0	2	1	2
ENSMUSG00000114918	GM9012	0	0	0	0	0	0	0	0	0	0	0	1
ENSMUSG00000114931	GM32256	0	0	0	1	0	0	0	1	0	1	1	0
ENSMUSG00000114932	GM4936	0	0	0	0	0	0	1	0	0	0	1	1
ENSMUSG00000114934	GM48342	0	0	0	0	0	0	0	0	0	2	0	0
ENSMUSG00000114937	GM30054	0	0	1	0	0	0	0	1	4	1	2	0
ENSMUSG00000114940	GM21378	0	0	0	0	0	0	0	0	0	0	0	1
ENSMUSG00000114951	GM20784	0	0	0	1	0	0	1	0	2	1	1	0
ENSMUSG00000114952	GM29717	0	0	0	0	0	0	0	0	0	1	0	0
ENSMUSG00000114968	A630019I02R	1	0	0	0	0	0	0	1	0	1	0	0
ENSMUSG00000114980	AC102815.1	0	0	0	0	0	0	0	1	0	0	0	0
ENSMUSG00000114981	AC115304.1	0	0	0	0	0	0	0	0	0	1	1	0
ENSMUSG00000114995	AC137842.1	0	0	0	0	0	0	0	0	0	0	1	0
ENSMUSG00000115002	AC135083.1	0	0	0	0	0	0	1	0	0	1	0	0
ENSMUSG00000115003	AC093043.1	0	1	0	0	0	0	0	0	0	0	0	0
ENSMUSG00000115005	AC152940.1	0	1	1	1	0	1	5	3	2	2	2	3
ENSMUSG00000115007	AC154276.1	0	0	0	0	0	0	2	1	2	1	1	3
ENSMUSG00000115025	AC139294.1	0	0	0	0	0	0	1	0	0	0	0	0

ENSMUSG00000115026	AC155729.1	0	0	0	0	0	1	3	1	0	1	1	1
ENSMUSG00000115037	AC137871.1	0	0	0	0	0	0	0	0	0	1	1	0
ENSMUSG00000115039	AC122350.1	0	0	0	0	1	0	0	2	0	0	1	0
ENSMUSG00000115040	AC121598.1	0	1	1	0	0	0	0	0	1	0	0	0
ENSMUSG00000115044	CT573086.2	0	0	0	0	0	0	0	4	1	0	1	0
ENSMUSG00000115049	AC113178.1	0	0	0	0	0	0	2	0	0	1	0	0
ENSMUSG00000115057	AC122459.1	0	0	0	0	0	0	0	0	0	0	1	0
ENSMUSG00000115062	AC158777.1	0	0	0	1	0	0	0	0	0	1	0	0
ENSMUSG00000115087	AC126251.1	0	0	0	0	0	0	0	0	0	0	0	1
ENSMUSG00000115090	AC155167.1	0	0	0	0	0	0	0	0	1	0	0	1
ENSMUSG00000115097	AC156016.3	0	0	0	0	0	0	1	0	0	0	0	0
ENSMUSG00000115102	AC154616.1	0	0	0	0	0	0	1	1	1	1	0	0
ENSMUSG00000115106	AC133283.1	0	1	1	0	0	0	0	0	0	1	1	0
ENSMUSG00000115119	AC135292.1	0	0	0	0	0	0	0	0	0	0	0	1
ENSMUSG00000115124	AC154738.2	1	1	1	0	1	1	1	3	2	1	3	3
ENSMUSG00000115137	AC107645.1	0	0	0	2	1	0	1	1	1	0	0	0
ENSMUSG00000115144	AC121598.2	0	0	0	0	0	0	1	0	0	0	0	0
ENSMUSG00000115145	AC123665.1	1	0	0	0	0	0	1	1	0	0	0	0
ENSMUSG00000115148	AC107711.1	0	0	0	0	0	0	0	1	0	1	1	0
ENSMUSG00000115149	AC112142.1	1	2	0	0	0	0	4	3	2	2	1	1
ENSMUSG00000115161	AC130219.1	0	0	0	0	0	0	1	0	0	0	0	0
ENSMUSG00000115170	VMN1R38	0	0	0	0	0	0	1	0	0	0	0	0
ENSMUSG00000115184	CT010453.1	0	0	1	0	0	0	0	0	2	1	0	1
ENSMUSG00000115186	AC151836.1	0	0	0	0	0	0	0	1	0	1	0	0
ENSMUSG00000115188	AC137842.2	0	0	0	0	0	0	0	1	0	1	0	0
ENSMUSG00000115193	AC121357.1	0	0	0	0	1	0	0	0	0	0	1	1
ENSMUSG00000115194	AC166574.2	0	0	0	0	0	0	0	1	1	3	0	0
ENSMUSG00000115210	AC120375.1	0	0	0	0	0	0	0	0	0	1	0	1
ENSMUSG00000115219	EEF1AKMT4	0	1	1	3	1	3	7	12	12	12	9	9
ENSMUSG00000115220	AC154760.1	0	0	0	1	0	0	0	0	0	2	0	0
ENSMUSG00000115222	AC174678.1	0	0	1	0	1	0	0	1	0	5	0	1
ENSMUSG00000115224	AC115735.1	0	0	1	0	1	0	1	3	0	1	0	0
ENSMUSG00000115226	AC123692.2	0	0	0	0	0	0	1	0	0	0	0	0
ENSMUSG00000115239	AC164100.1	0	0	0	0	1	1	0	0	0	0	0	0
ENSMUSG00000115249	AC122291.1	1	1	1	4	0	0	3	2	4	3	0	3
ENSMUSG00000115264	AC169511.2	0	0	0	0	0	0	1	0	3	1	1	2
ENSMUSG00000115276	AC149588.1	6	13	10	15	6	9	18	18	17	28	15	17
ENSMUSG00000115284	AC160550.2	0	0	0	0	1	0	1	0	2	0	1	1
ENSMUSG00000115294	AC156790.2	0	0	0	0	0	0	0	0	0	0	0	1
ENSMUSG00000115300	AC126028.1	0	0	0	0	0	0	0	0	0	1	0	0
ENSMUSG00000115301	AC102847.1	0	1	0	0	0	0	0	0	0	0	0	1
ENSMUSG00000115306	AC158988.1	0	1	0	0	1	0	0	2	1	0	0	1
ENSMUSG00000115314	AC121598.3	0	2	0	0	0	4	1	0	0	3	0	0
ENSMUSG00000115324	AC107711.2	0	0	0	0	0	0	0	0	0	0	1	0
ENSMUSG00000115328	AC122034.1	0	0	0	0	0	0	0	0	0	1	0	0
ENSMUSG00000115338	PNP	39	41	56	48	43	49	247	258	204	232	190	193
ENSMUSG00000115340	AC144772.1	0	0	0	0	0	0	0	0	0	2	0	1
ENSMUSG00000115356	AC150560.2	0	0	0	0	0	0	0	1	0	0	0	0
ENSMUSG00000115357	AC115735.2	0	0	0	0	0	0	0	1	0	0	0	0
ENSMUSG00000115358	AC127578.1	9	10	22	23	17	29	62	79	58	60	51	93
ENSMUSG00000115372	AC163295.2	0	0	0	0	0	0	0	0	0	1	0	0
ENSMUSG00000115373	AC154575.1	0	0	0	0	0	0	0	0	0	1	0	0
ENSMUSG00000115383	AC115735.3	0	1	0	0	0	0	0	0	0	0	0	0
ENSMUSG00000115384	AC121997.1	0	0	0	1	0	0	0	0	0	0	0	0
ENSMUSG00000115386	AC138716.1	0	0	0	0	0	0	0	0	0	1	0	0
ENSMUSG00000115401	AC183096.1	0	0	0	0	0	0	0	0	0	1	0	0
ENSMUSG00000115417	AC131586.4	0	0	0	0	0	0	2	0	0	0	0	0
ENSMUSG00000115424	AC121997.2	0	0	0	1	0	0	0	0	0	1	1	0

ENSMUSG00000115426	AC129931.1	0	0	0	0	0	0	1	2	1	0	1	1
ENSMUSG00000115431	AC160930.2	0	0	1	0	0	0	0	0	1	0	0	0
ENSMUSG00000115439	AC154270.1	0	0	0	0	0	0	0	0	0	1	0	0
ENSMUSG00000115450	AC161383.1	0	0	0	0	0	0	0	0	1	0	0	0
ENSMUSG00000115451	AC154276.2	0	0	0	0	0	0	0	0	0	1	1	0
ENSMUSG00000115457	AC022775.2	0	0	0	0	0	0	0	0	0	1	0	0
ENSMUSG00000115469	CT010505.2	0	0	0	0	0	0	1	0	0	0	0	0
ENSMUSG00000115483	AC166344.3	0	0	0	0	0	0	1	0	0	0	0	0
ENSMUSG00000115493	AC140799.1	0	0	0	0	0	0	0	0	0	0	1	0
ENSMUSG00000115497	AC131033.1	2	3	2	7	1	3	5	11	8	12	9	3
ENSMUSG00000115499	CT025525.4	0	0	0	0	1	0	0	0	0	0	0	0
ENSMUSG00000115503	AC122937.3	0	0	0	0	0	0	1	1	2	1	0	0
ENSMUSG00000115505	AC123061.1	0	0	0	0	0	0	0	0	0	1	0	0
ENSMUSG00000115508	AC164883.6	0	0	0	1	0	0	1	0	0	1	0	0
ENSMUSG00000115512	AC102815.2	0	0	0	0	0	0	0	3	0	0	0	1
ENSMUSG00000115541	AC157572.3	1	1	1	2	1	0	1	1	1	3	2	0
ENSMUSG00000115543	AC130671.2	0	0	0	0	0	0	0	6	0	0	0	0
ENSMUSG00000115545	BX649293.1	0	0	0	0	1	0	1	2	2	0	1	1
ENSMUSG00000115548	AC138716.2	0	0	0	0	0	0	0	0	1	0	1	0
ENSMUSG00000115568	AC130671.3	0	0	0	0	1	0	0	0	0	0	0	0
ENSMUSG00000115569	AC107757.2	0	1	2	0	2	0	4	2	0	4	3	2
ENSMUSG00000115571	AC123665.4	0	0	0	0	0	0	0	0	1	0	0	0
ENSMUSG00000115584	AC154477.1	0	0	0	0	0	0	0	1	0	0	0	0
ENSMUSG00000115591	AC132833.3	0	0	0	0	0	0	2	0	0	0	0	0
ENSMUSG00000115593	AC131178.1	0	0	0	0	0	0	1	0	0	0	0	2
ENSMUSG00000115595	CT009550.1	0	1	1	0	0	2	2	8	1	3	3	2
ENSMUSG00000115598	AC144819.1	0	0	0	0	0	0	0	0	0	0	0	1
ENSMUSG00000115604	AC112945.1	0	1	1	0	0	1	4	3	4	1	7	2
ENSMUSG00000115617	AC123665.5	0	0	0	0	0	0	0	1	0	0	0	0
ENSMUSG00000115624	AC154640.4	0	0	0	0	0	0	0	1	0	2	0	0
ENSMUSG00000115630	AC125405.1	0	0	0	0	0	0	1	0	0	1	0	0
ENSMUSG00000115635	AC116656.1	0	0	0	2	0	0	0	0	0	2	0	0
ENSMUSG00000115644	VMN1R17	0	0	0	0	0	0	0	1	0	0	0	0
ENSMUSG00000115645	AC139294.2	0	0	0	0	0	0	0	0	1	0	0	0
ENSMUSG00000115662	AC125311.2	0	0	0	0	0	2	0	0	0	5	1	0
ENSMUSG00000115664	CT010577.1	0	0	0	0	0	0	0	1	0	3	0	0
ENSMUSG00000115667	AC158921.1	0	1	0	0	0	0	0	0	0	0	0	0
ENSMUSG00000115681	AC140209.1	0	0	0	0	0	0	1	1	0	3	3	3
ENSMUSG00000115685	AC126028.2	3	7	2	1	5	2	11	8	7	8	8	9
ENSMUSG00000115691	AC167565.1	1	0	0	0	0	0	0	0	0	0	0	0
ENSMUSG00000115692	AC122821.1	0	0	0	0	0	0	0	0	0	1	0	0
ENSMUSG00000115699	AC107757.3	0	0	0	0	0	0	3	0	1	3	0	1
ENSMUSG00000115701	VMN1R6	0	0	0	0	0	0	0	1	0	0	1	0
ENSMUSG00000115708	AC166159.1	0	0	1	0	0	0	0	0	0	0	0	0
ENSMUSG00000115710	AC115735.5	0	0	0	0	0	0	0	0	1	0	0	1
ENSMUSG00000115718	AC154377.1	12	14	12	16	8	16	34	35	23	41	27	28
ENSMUSG00000115728	AC156016.4	0	1	0	0	0	0	0	0	0	0	0	0
ENSMUSG00000115730	AC106830.2	0	0	0	0	0	0	0	0	0	0	0	1
ENSMUSG00000115736	AC157572.4	0	0	0	0	0	0	2	1	1	0	0	1
ENSMUSG00000115737	CT025618.1	0	0	1	0	0	0	3	2	1	2	1	2
ENSMUSG00000115743	AC154855.1	0	0	0	0	0	0	0	0	0	1	1	0
ENSMUSG00000115745	AC154649.2	1	0	0	0	2	0	4	7	4	8	2	2
ENSMUSG00000115754	AC154270.2	0	0	0	0	0	0	1	0	0	0	0	0
ENSMUSG00000115756	AC164437.5	0	0	0	0	0	0	0	0	0	1	0	0
ENSMUSG00000115759	AC133156.2	0	1	0	0	0	0	0	0	0	0	0	0
ENSMUSG00000115762	AC114585.2	0	0	0	0	0	0	0	0	0	1	0	0
ENSMUSG00000115763	CT025602.2	0	0	0	0	0	1	0	0	1	0	0	0
ENSMUSG00000115764	AC159006.3	0	0	0	0	0	0	0	0	0	0	1	0

ENSMUSG00000115767	AC164433.2	0	0	0	0	0	0	0	1	0	0	0	0
ENSMUSG00000115771	AC122035.2	0	0	0	0	0	0	1	1	1	0	1	0
ENSMUSG00000115775	AC101831.1	0	0	1	0	0	0	0	1	0	1	0	0
ENSMUSG00000115778	AC159002.2	0	0	0	0	0	0	1	0	0	0	0	1
ENSMUSG00000115779	AC158954.1	0	0	0	0	0	0	0	3	0	0	0	0
ENSMUSG00000115783	AC161763.1	5	5	2	2	2	6	9	9	7	14	9	10
ENSMUSG00000115789	AC161877.2	0	0	0	0	0	0	0	0	0	2	0	0
ENSMUSG00000115791	VMN1R217	0	0	0	0	1	0	0	0	0	0	0	0
ENSMUSG00000115793	AC144772.2	0	1	0	0	0	0	3	0	0	1	1	0
ENSMUSG00000115801	AC160336.1	0	0	0	0	0	0	1	1	0	0	0	0
ENSMUSG00000115802	CT009550.4	1	0	0	0	0	0	1	0	0	1	1	0
ENSMUSG00000115811	AC121577.1	0	0	0	0	0	0	1	0	1	1	0	0
ENSMUSG00000115812	AC120375.2	2	0	0	0	1	0	1	2	0	3	0	1
ENSMUSG00000115813	AC167565.2	0	0	0	0	0	0	0	0	1	1	0	0
ENSMUSG00000115829	AC166356.1	0	2	0	0	1	0	4	0	1	4	0	1
ENSMUSG00000115837	AC144772.3	0	1	1	0	1	1	5	6	4	2	3	2
ENSMUSG00000115843	AC137902.2	0	0	0	0	0	0	0	2	1	0	0	2
ENSMUSG00000115849	AC156016.5	0	0	0	0	1	0	5	1	0	1	2	0
ENSMUSG00000115855	AC102815.3	0	0	0	0	1	0	0	0	0	0	0	0
ENSMUSG00000115896	AC161172.2	0	0	0	0	0	0	0	1	0	0	0	0
ENSMUSG00000115902	AC113595.1	0	0	1	0	0	0	1	1	0	1	0	2
ENSMUSG00000115918	AC146911.1	0	2	0	0	0	2	1	2	2	2	1	2
ENSMUSG00000115923	AC112943.2	1	0	0	0	0	0	2	3	3	1	2	0
ENSMUSG00000115938	AC134548.1	1	1	0	0	0	0	2	0	4	1	1	0
ENSMUSG00000115955	AL731707.1	1	3	0	3	2	4	2	4	4	0	6	4
ENSMUSG00000115961	AC139347.1	1	0	0	0	0	0	2	0	0	2	1	0
ENSMUSG00000115976	AC141880.1	0	0	0	0	0	0	0	2	0	0	1	1
ENSMUSG00000115980	AC161165.1	0	0	0	0	0	0	1	0	0	0	0	0
ENSMUSG00000115986	AC136372.1	0	0	0	0	0	0	1	0	0	0	0	1
ENSMUSG00000115987	VPS28	3	8	8	9	5	4	39	60	52	60	32	39
ENSMUSG00000116000	AC140304.1	0	0	0	0	0	0	0	0	0	0	1	0
ENSMUSG00000116004	AC162302.1	0	0	0	0	0	0	0	0	0	1	0	0
ENSMUSG00000116009	AC104325.1	0	0	0	0	0	0	0	0	0	1	0	0
ENSMUSG00000116021	AC133868.1	0	0	0	0	0	0	0	1	0	1	1	0
ENSMUSG00000116083	AC138715.1	0	0	0	0	1	0	0	0	0	0	0	0
ENSMUSG00000116089	AC125541.1	0	0	0	0	0	1	2	0	0	0	0	0
ENSMUSG00000116090	AC123611.1	1	1	0	1	4	1	3	3	1	2	1	5
ENSMUSG00000116095	AC115005.1	0	0	0	0	0	0	0	0	0	1	0	0
ENSMUSG00000116097	AL590144.2	0	0	0	1	0	0	3	1	1	0	0	1
ENSMUSG00000116121	PICK1	0	0	0	0	0	0	1	0	1	0	0	1
ENSMUSG00000116127	AC140267.3	0	0	0	0	0	0	0	1	0	0	0	0
ENSMUSG00000116138	C030006K11F	0	0	0	0	1	0	7	6	4	5	2	5
ENSMUSG00000116164	AC123724.1	0	0	0	0	0	0	0	0	1	0	0	0
ENSMUSG00000116165	PDXP	4	4	7	11	7	5	41	38	30	40	19	32
ENSMUSG00000116173	AC169509.4	0	0	0	0	0	0	0	0	0	0	1	0
ENSMUSG00000116174	AC132863.4	2	0	1	0	0	0	0	5	1	0	2	1
ENSMUSG00000116183	AC163291.2	0	0	0	1	0	0	0	0	0	0	0	0
ENSMUSG00000116213	AC173707.1	0	0	0	0	0	0	0	1	0	0	0	0
ENSMUSG00000116220	AC147041.1	0	0	0	0	0	0	3	2	2	6	4	2
ENSMUSG00000116226	AC105358.1	0	1	1	0	2	0	3	2	2	7	1	3
ENSMUSG00000116238	AC157566.3	0	0	2	3	0	1	3	4	3	1	0	3
ENSMUSG00000116249	AC117831.2	0	1	0	0	1	0	0	0	0	0	0	0
ENSMUSG00000116250	AC134548.2	0	0	0	0	0	0	0	1	0	0	0	0
ENSMUSG00000116262	AL589670.3	0	0	0	0	0	0	1	1	0	0	0	0
ENSMUSG00000116280	AC087902.3	0	0	0	0	0	0	1	0	0	0	0	0
ENSMUSG00000116287	AL589670.4	0	1	0	0	0	0	0	0	1	0	0	1
ENSMUSG00000116289	AC087116.1	0	0	0	0	0	0	0	0	0	0	1	0
ENSMUSG00000116305	AC162302.2	0	0	0	0	0	0	0	1	0	0	0	1

ENSMUSG00000116321	AC102198.1	0	0	0	0	0	0	0	0	1	0	0	1
ENSMUSG00000116348	AC165274.1	0	0	1	0	0	0	0	1	0	0	0	0
ENSMUSG00000116366	AC125526.1	0	0	0	0	0	0	0	0	0	0	1	0
ENSMUSG00000116378	GCAT	0	0	0	0	0	0	1	0	0	0	0	1
ENSMUSG00000116380	AC139671.1	1	0	0	0	0	0	0	0	0	0	0	0
ENSMUSG00000116386	CT010522.2	0	0	1	0	0	0	0	0	0	1	0	0
ENSMUSG00000116414	CT033751.1	0	0	0	0	0	0	0	0	0	0	0	1
ENSMUSG00000116433	AC147041.2	0	0	0	1	1	0	0	1	0	1	1	0
ENSMUSG00000116438	AL592187.7	0	0	0	0	0	0	0	0	0	0	1	0
ENSMUSG00000116475	AC110211.2	0	0	0	0	0	0	0	0	0	0	1	0
ENSMUSG00000116480	AC126682.1	0	0	0	0	0	0	0	0	0	0	1	0
ENSMUSG00000116504	AC123724.3	0	0	0	0	0	0	0	1	1	0	0	0
ENSMUSG00000116506	AC161165.6	0	0	0	0	0	0	0	0	0	1	0	0
ENSMUSG00000116518	AC138177.5	1	0	0	0	0	0	0	0	0	0	0	1

Normalized count matrix

ID	GeneID	C1498S_rep1	C1498S_rep2	C1498S_rep3	C1498S_rep4	C1498S_rep5	C1498S_rep6	C1498R_rep1	C1498R_rep2	C1498R_rep3	C1498R_rep4	C1498R_rep5	C1498R_rep6
EGFP	EGFP	26.2189633	6.621771059	0	11.93566676	0	12.87166946	13.18532368	11.60032996	17.55143448	21.62788267	17.51678546	12.35456362
ENSMUSG00000000001	GNAI3	122.3551621	72.83948165	43.72444853	47.74266702	128.6231535	83.66585146	130.5347044	158.5378429	124.6151848	118.9533547	157.6510691	114.2797135
ENSMUSG00000000028	CDC45	78.65688991	86.08302377	43.72444853	77.58183392	57.88041905	70.79418201	72.51928024	70.89090534	71.96088136	52.86815763	77.07385602	72.58306128
ENSMUSG00000000056	NARF	8.739654434	13.24354212	21.86222426	11.93566676	25.72463069	12.87166946	18.45945315	21.91173438	24.57200827	15.62013748	36.78524947	23.16480679
ENSMUSG00000000058	CAV2	0	0	21.86222426	0	0	12.87166946	7.911194208	2.577851103	1.755143448	7.209294222	5.255035638	6.177281811
ENSMUSG00000000078	KLF6	87.39654434	39.73062635	109.3111213	101.4531674	45.01810371	83.66585146	54.05982709	45.11239431	64.94030757	46.86041244	54.30203492	47.87393403
ENSMUSG00000000085	SCMH1	69.91723547	33.10885529	109.3111213	107.4210008	70.7427344	70.79418201	43.51156814	65.73520313	63.18516412	61.27900089	54.30203492	40.15233177
ENSMUSG00000000088	COX5A	209.7517064	185.4095896	225.9096507	161.1315012	212.2282032	148.0241987	291.3956533	278.4079192	252.7406565	219.8834738	282.0202459	262.534477
ENSMUSG00000000093	TBX2	0	0	0	0	0	0	0	0	3.510286896	0	0	0
ENSMUSG00000000127	FER	0	6.621771059	0	0	0	0	0	0	0	0	0	0
ENSMUSG00000000131	XPO6	69.91723547	92.70479482	72.87408088	59.67833378	77.17389207	77.23001673	68.56368314	74.757682	77.22631171	84.10843259	78.82553457	78.76034309
ENSMUSG00000000134	TFE3	0	19.86531318	7.287408088	5.967833378	12.86231535	25.74333891	25.05211499	19.33388327	12.28600414	12.01549037	7.006714184	18.53184543
ENSMUSG00000000142	AXIN2	52.4379266	39.73062635	72.87408088	23.87133351	45.01810371	32.17917364	51.42276235	43.82346876	33.34772551	48.06196148	63.06042765	50.96257494
ENSMUSG00000000148	BRAT1	17.47930887	0	21.86222426	5.967833378	12.86231535	12.87166946	14.50385605	15.46710662	21.06172137	18.02323556	19.26846401	21.62048634
ENSMUSG00000000149	GNA12	8.739654434	6.621771059	29.14963235	17.90350013	12.86231535	6.435834728	21.09651789	28.35636214	21.06172137	18.02323556	29.77853528	29.3420886
ENSMUSG00000000157	ITGB2L	0	0	0	0	0	6.435834728	0	0	0	0	0	0
ENSMUSG00000000159	IGSF5	8.739654434	13.24354212	36.43704044	23.87133351	6.431157673	19.30750418	13.18532368	19.33388327	19.30657793	10.81394133	19.26846401	12.35456362
ENSMUSG00000000167	PIH1D2	0	0	0	0	0	0	0	3.866776655	0	2.403098074	0	0
ENSMUSG00000000168	DLAT	43.69827217	66.21771059	29.14963235	59.67833378	45.01810371	12.87166946	69.8822155	63.15735203	68.45059447	67.28674607	54.30203492	71.03874082
ENSMUSG00000000171	SDHD	148.5741254	132.4354212	138.4607537	113.3888342	160.7789418	90.10168619	188.5501286	224.273046	201.8414965	204.2633363	213.7047826	196.1286975
ENSMUSG00000000183	FGF6	0	0	0	0	0	0	0	0	0	0	0	1.544320453
ENSMUSG00000000184	CCND2	1083.71715	1562.73797	1209.709743	1181.631009	1144.746066	1242.116102	926.9282547	879.0472262	803.8556991	922.7896604	830.2956308	849.376249
ENSMUSG00000000194	GPR107	17.47930887	33.10885529	21.86222426	11.93566676	38.58694604	12.87166946	17.14092078	19.33388327	17.55143448	25.23252978	14.01342837	15.44320453
ENSMUSG00000000215	INS2	0	0	0	0	0	0	0	2.577851103	0	0	0	0
ENSMUSG00000000223	DRP2	0	0	0	5.967833378	0	0	0	6.444627758	1.755143448	2.403098074	0	3.088640905
ENSMUSG00000000244	TSPAN32	113.6155076	119.1918791	94.73630514	65.64616716	115.7608381	96.53752092	59.33395656	85.06908641	52.65430344	67.28674607	71.81882038	91.11490671
ENSMUSG00000000253	GMPR	0	0	0	0	0	0	1.318532368	0	0	0	0	0
ENSMUSG00000000263	GLRA1	0	0	7.287408088	0	0	0	0	0	0	0	1.751678546	0
ENSMUSG00000000266	MID2	0	0	0	0	0	0	0	1.288925552	0	0	0	0
ENSMUSG00000000275	TRIM25	34.95861774	46.35239741	21.86222426	5.967833378	45.01810371	12.87166946	22.41505026	36.08991545	36.85801241	40.85266726	29.77853528	35.51937041
ENSMUSG00000000276	DGKE	8.739654434	0	29.14963235	35.80700027	25.72463069	19.30750418	11.86679131	18.04495772	12.28600414	13.21703941	14.01342837	16.98752498
ENSMUSG00000000278	SCPEP1	34.95861774	19.86531318	21.86222426	17.90350013	64.31157673	25.74333891	42.19303578	42.5345432	45.63372964	36.04647111	35.03357092	41.69665222
ENSMUSG00000000282	MNT	34.95861774	13.24354212	29.14963235	23.87133351	38.58694604	0	27.68917973	20.62280883	26.32715172	26.43407881	19.26846401	26.2534477
ENSMUSG00000000290	ITGB2	96.13619877	66.21771059	43.72444853	59.67833378	77.17389207	90.10168619	59.33395656	54.13487317	68.45059447	64.883648	66.56378475	63.31713856
ENSMUSG00000000295	HDDC2	61.17758104	52.97416847	29.14963235	5.967833378	38.58694604	57.92251255	54.05982709	64.44627758	59.67487723	67.28674607	75.32217748	78.76034309
ENSMUSG00000000301	PEMT	8.739654434	0	0	0	0	0	3.955597104	1.288925552	1.755143448	1.201549037	1.751678546	1.544320453
ENSMUSG00000000308	CKMT1	26.2189633	19.86531318	21.86222426	0	6.431157673	6.435834728	3.955597104	6.444627758	8.775717239	3.604647111	8.75839273	1.544320453
ENSMUSG00000000325	ARVCF	0	13.24354212	29.14963235	23.87133351	32.15578836	45.05084309	32.9633092	32.22313879	24.57200827	37.24802015	36.78524947	33.97504996
ENSMUSG00000000326	COMT	900.1844067	960.1568035	976.5126837	1008.563841	951.8113356	1049.041061	444.345408	447.2571664	524.7878909	486.62736	504.4834212	540.5121584
ENSMUSG00000000339	RTCA	69.91723547	59.59593953	65.58667279	83.54966729	51.44926138	102.9733556	109.4381865	125.0257785	112.3291807	100.9301191	129.6242124	128.1785976
ENSMUSG00000000340	DBT	113.6155076	178.7878186	102.0237132	173.067168	83.60504975	122.2808598	110.7567189	143.0707362	170.2489144	142.9843354	113.8591055	134.3558794

ENSMUSG00000000346	DAZAP2	52.4379266	33.10885529	182.1852022	59.67833378	128.6231535	57.92251255	125.260575	110.8475974	103.5534634	98.52702104	122.6174982	131.2672385
ENSMUSG00000000355	MCTS1	61.17758104	46.35239741	51.01185661	65.64616716	64.31157673	77.23001673	100.20846	121.1590019	136.9011889	129.767296	106.8523913	83.39330444
ENSMUSG00000000374	TRAPPC10	17.47930887	13.24354212	36.43704044	29.83916689	19.29347302	12.87166946	25.05211499	27.06743658	31.59258206	36.04647111	31.53021383	37.06369086
ENSMUSG00000000378	CCM2	61.17758104	86.08302377	65.58667279	41.77483365	51.44926138	45.05084309	87.02313629	73.46875644	100.0431765	68.48829511	87.5839273	72.58306128
ENSMUSG00000000384	TBRG4	78.65688991	46.35239741	72.87408088	65.64616716	115.7608381	70.79418201	59.33395656	77.3355331	70.20573791	69.68984415	61.30874911	67.95009992
ENSMUSG00000000386	MX1	0	0	0	5.967833378	0	0	0	0	0	0	0	0
ENSMUSG00000000399	NDUFA9	52.4379266	92.70479482	109.3111213	71.61400054	70.7427344	45.05084309	73.83781261	95.38049082	119.3497545	86.51153067	84.08057021	98.83650897
ENSMUSG00000000409	LCK	34.95861774	26.48708424	21.86222426	11.93566676	38.58694604	25.74333891	15.82238842	29.64528769	26.32715172	24.03098074	19.26846401	12.35456362
ENSMUSG00000000416	CTTNBP2	0	6.621771059	7.287408088	0	0	0	1.318532368	1.288925552	3.510286896	1.201549037	0	3.088640905
ENSMUSG00000000420	GALNT1	358.3258318	304.6014687	429.9570772	566.9441709	340.8513567	302.4842322	234.6987615	241.0290782	222.9032179	217.4803757	259.2484248	180.685493
ENSMUSG00000000439	MKRN2	34.95861774	39.73062635	80.16148896	53.7105004	19.29347302	19.30750418	34.28184157	36.08991545	36.85801241	30.03872593	42.0402851	46.32961358
ENSMUSG00000000441	RAF1	61.17758104	59.59593953	80.16148896	65.64616716	122.1919958	64.35834728	81.74900682	69.60197979	78.98145515	80.50378548	70.06714184	71.03874082
ENSMUSG00000000486	37135	34.95861774	6.621771059	29.14963235	17.90350013	12.86231535	6.435834728	26.37064736	23.20065993	19.30657793	15.62013748	28.02685674	23.16480679
ENSMUSG00000000532	ACVR1B	0	13.24354212	7.287408088	0	0	0	1.318532368	1.288925552	0	1.201549037	0	1.544320453
ENSMUSG00000000538	TOM1L2	8.739654434	6.621771059	21.86222426	23.87133351	19.29347302	12.87166946	14.50385605	9.022478862	8.775717239	24.03098074	19.26846401	21.62048634
ENSMUSG00000000552	ZFP385A	17.47930887	0	0	0	0	6.435834728	3.955597104	2.577851103	3.510286896	2.403098074	0	4.632961358
ENSMUSG00000000560	GABRA2	0	0	0	0	0	0	0	0	0	1.201549037	0	0
ENSMUSG00000000561	WDR77	52.4379266	59.59593953	36.43704044	65.64616716	83.60504975	38.61500837	94.9343305	95.38049082	93.02260274	112.9456095	96.34232003	108.1024317
ENSMUSG00000000562	ADORA3	0	0	0	0	0	0	1.318532368	0	0	0	0	0
ENSMUSG00000000563	ATP5F1	410.7637584	403.9280346	298.7837316	399.8448363	469.4745101	411.8934226	718.6001406	812.0230975	705.567666	684.8829511	818.033881	656.3361924
ENSMUSG00000000568	HNRNPD	235.9706697	258.2490713	269.6340992	256.6168353	282.9709376	296.0483975	361.2778688	382.8108888	382.6212716	361.6662601	315.3021383	387.6244336
ENSMUSG00000000579	DYNLT1C	0	0	0	11.93566676	0	0	0	2.577851103	0	1.201549037	0	0
ENSMUSG00000000581	C1D	52.4379266	66.21771059	51.01185661	77.58183392	64.31157673	45.05084309	51.42276235	78.62445865	57.91973378	85.30998163	45.5436422	52.50689539
ENSMUSG00000000594	GM2A	0	0	0	5.967833378	32.15578836	12.87166946	0	1.288925552	1.755143448	0	0	1.544320453
ENSMUSG00000000600	KRIT1	218.4913609	79.46125271	131.1733456	113.3888342	109.3296804	135.1525293	114.712316	143.0707362	122.8600414	129.767296	120.8658197	118.9126749
ENSMUSG00000000605	CLCN4	34.95861774	46.35239741	36.43704044	11.93566676	19.29347302	19.30750418	23.73358262	25.77851103	24.57200827	19.22478459	21.02014255	24.70912724
ENSMUSG00000000627	SEMA4F	26.2189633	19.86531318	29.14963235	5.967833378	6.431157673	25.74333891	7.911194208	16.75603217	14.04114758	10.81394133	5.255035638	12.35456362
ENSMUSG00000000628	HK2	104.8758532	112.570108	123.8859375	101.4531674	57.88041905	115.8450251	90.97873339	97.95834193	98.28803308	94.92237392	64.8121062	94.20354761
ENSMUSG00000000631	MYO18A	52.4379266	33.10885529	14.57481618	23.87133351	19.29347302	32.17917364	15.82238842	16.75603217	28.08229517	20.42633363	8.75839273	27.79776815
ENSMUSG00000000673	HAAO	0	0	0	0	0	0	0	1.288925552	0	0	0	0
ENSMUSG00000000682	CD52	0	0	0	0	0	0	0	1.288925552	0	1.201549037	0	0
ENSMUSG00000000686	ABHD15	0	6.621771059	7.287408088	11.93566676	6.431157673	0	5.274129472	7.73355331	8.775717239	1.201549037	7.006714184	6.177281811
ENSMUSG00000000693	LOXL3	26.2189633	19.86531318	21.86222426	0	38.58694604	12.87166946	15.82238842	18.04495772	14.04114758	16.82168652	17.51678546	20.07616588
ENSMUSG00000000706	BTN1A1	0	6.621771059	0	0	0	0	0	0	3.510286896	0	0	0
ENSMUSG00000000708	KAT2B	52.4379266	6.621771059	43.72444853	17.90350013	25.72463069	57.92251255	30.32624446	20.62280883	19.30657793	36.04647111	29.77853528	21.62048634
ENSMUSG00000000711	RAB5B	0	0	0	0	6.431157673	6.435834728	5.274129472	15.46710662	8.775717239	4.806196148	10.51007128	10.81024317
ENSMUSG00000000738	SPG7	52.4379266	33.10885529	36.43704044	35.80700027	64.31157673	45.05084309	36.9189063	33.51206434	50.89915999	30.03872593	54.30203492	40.15233177
ENSMUSG00000000739	SULT5A1	0	0	0	0	0	0	0	0	1.755143448	0	1.751678546	0
ENSMUSG00000000740	RPL13	1887.765358	1390.571922	1712.540901	1474.054844	1530.615526	1602.522847	1202.50152	1259.280264	1246.151848	1086.200329	1303.248838	1190.671069
ENSMUSG00000000743	CHMP1A	8.739654434	0	29.14963235	5.967833378	19.29347302	25.74333891	18.45945315	24.48958548	36.85801241	22.8294317	22.7718211	40.15233177
ENSMUSG00000000751	RPA1	122.3551621	139.0571922	87.44889705	47.74266702	102.8985228	57.92251255	113.3937836	116.0032996	128.1254717	100.9301191	127.8725339	122.0013158
ENSMUSG00000000759	TUBGCP3	61.17758104	26.48708424	21.86222426	35.80700027	45.01810371	38.61500837	48.78569762	38.66776655	28.08229517	27.63562785	42.0402851	38.60801132
ENSMUSG00000000766	OPRM1	17.47930887	6.621771059	7.287408088	11.93566676	0	0	3.955597104	0	1.755143448	6.007745185	0	3.088640905
ENSMUSG00000000776	POLR3D	8.739654434	13.24354212	21.86222426	11.93566676	12.86231535	25.74333891	19.77798552	16.75603217	15.79629103	18.02323556	5.255035638	20.07616588
ENSMUSG00000000782	TCF7	445.7223761	350.9538661	342.5081801	405.8126697	379.4383027	379.7142489	425.8859549	456.2796453	442.2961489	438.5653985	446.6780292	450.9415722

ENSMUSG00000000787	DDX3X	253.4499786	178.7878186	218.6222426	196.9385015	218.6593609	212.382546	328.3145596	326.0981646	303.6398165	368.8755544	304.792067	281.0663224
ENSMUSG00000000791	IL12RB1	0	6.621771059	7.287408088	0	0	0	0	0	3.510286896	1.201549037	0	1.544320453
ENSMUSG00000000792	SLC5A5	0	0	0	5.967833378	6.431157673	0	0	1.288925552	1.755143448	0	0	1.544320453
ENSMUSG00000000804	USP32	0	39.73062635	43.72444853	41.77483365	19.29347302	19.30750418	27.68917973	34.80098989	26.32715172	43.25576533	36.78524947	33.97504996
ENSMUSG00000000811	TXNRD3	0	0	0	0	0	6.435834728	0	0	0	0	0	0
ENSMUSG00000000817	FASL	0	6.621771059	0	0	0	0	1.318532368	0	0	0	0	0
ENSMUSG00000000823	ZFP512B	69.91723547	66.21771059	109.3111213	89.51750067	51.44926138	102.9733556	100.20846	79.9133842	70.20573791	60.07745185	64.8121062	84.9376249
ENSMUSG00000000826	DNAJC5	131.0948165	119.1918791	80.16148896	113.3888342	141.4854688	115.8450251	112.0752513	97.95834193	105.3086069	98.52702104	126.1208553	92.65922716
ENSMUSG00000000827	TPD52L2	52.4379266	92.70479482	72.87408088	53.7105004	96.46736509	38.61500837	89.66020102	82.49123531	91.26745929	84.10843259	78.82553457	91.11490671
ENSMUSG00000000838	FMR1	52.4379266	72.83948165	94.73630514	113.3888342	96.46736509	45.05084309	98.8899276	87.64693751	107.0637503	99.72857007	94.59064148	91.11490671
ENSMUSG00000000869	IL4	0	0	0	0	6.431157673	6.435834728	0	0	0	0	1.751678546	0
ENSMUSG00000000876	PXMP4	34.95861774	26.48708424	87.44889705	23.87133351	38.58694604	19.30750418	32.9633092	41.24561765	42.12344275	32.441824	50.79867783	54.05121584
ENSMUSG00000000881	DLG3	0	13.24354212	0	5.967833378	0	6.435834728	10.54825894	5.155702207	12.28600414	2.403098074	5.255035638	4.632961358
ENSMUSG00000000884	GNB1L	17.47930887	6.621771059	21.86222426	0	6.431157673	0	5.274129472	10.31140441	7.020573791	12.01549037	7.006714184	12.35456362
ENSMUSG00000000889	DBH	0	0	7.287408088	0	6.431157673	0	0	1.288925552	1.755143448	0	0	3.088640905
ENSMUSG00000000902	SMARCB1	26.2189633	46.35239741	72.87408088	65.64616716	51.44926138	38.61500837	58.01542419	51.55702207	56.16459033	61.27900089	63.06042765	57.13985675
ENSMUSG00000000903	VPREB3	0	0	0	0	0	0	0	0	0	1.201549037	0	0
ENSMUSG00000000915	HIP1R	34.95861774	33.10885529	80.16148896	53.7105004	57.88041905	45.05084309	59.33395656	64.44627758	64.94030757	39.65111822	49.04699929	58.6841772
ENSMUSG00000000916	NSUN5	69.91723547	39.73062635	51.01185661	71.61400054	90.03620742	64.35834728	65.9266184	78.62445865	70.20573791	73.29449126	47.29532074	61.77281811
ENSMUSG00000000948	GM38393	0	0	0	0	0	6.435834728	1.318532368	1.288925552	3.510286896	1.201549037	0	0
ENSMUSG00000000958	SLC7A7	0	0	0	0	0	0	2.637064736	1.288925552	5.265430344	0	1.751678546	0
ENSMUSG00000000959	OXA1L	43.69827217	33.10885529	36.43704044	47.74266702	57.88041905	38.61500837	63.28955366	48.97917096	80.7365986	58.87590281	80.57721311	61.77281811
ENSMUSG00000000976	HEATR6	34.95861774	33.10885529	21.86222426	23.87133351	38.58694604	38.61500837	55.37835946	39.9566921	36.85801241	43.25576533	45.5436422	46.32961358
ENSMUSG00000001014	ICAM4	0	6.621771059	7.287408088	0	0	6.435834728	3.955597104	0	0	1.201549037	7.006714184	0
ENSMUSG00000001016	ILF2	419.5034128	297.9796976	400.8074448	262.5846686	289.4020953	238.1258849	378.4187896	344.1431223	391.3969889	366.4724563	395.8793514	375.26987
ENSMUSG00000001017	CHTOP	78.65688991	112.570108	87.44889705	89.51750067	77.17389207	96.53752092	84.38607155	69.60197979	94.77774619	75.69758933	99.84567712	84.9376249
ENSMUSG00000001018	SNAPIN	227.2310153	72.83948165	247.771875	190.9706681	205.7970455	109.4091904	138.4458986	153.3821406	231.6789351	123.7595508	203.1947113	134.3558794
ENSMUSG00000001020	S100A4	43.69827217	39.73062635	51.01185661	11.93566676	19.29347302	32.17917364	14.50385605	21.91173438	22.81686482	12.01549037	24.52349964	13.89888407
ENSMUSG00000001021	S100A3	0	0	0	0	0	0	0	0	0	1.201549037	0	0
ENSMUSG00000001025	S100A6	8.739654434	0	0	0	0	0	1.318532368	2.577851103	0	2.403098074	1.751678546	0
ENSMUSG00000001027	SCN4A	0	0	0	0	0	0	0	0	1.755143448	0	0	0
ENSMUSG00000001029	ICAM2	52.4379266	6.621771059	43.72444853	23.87133351	25.72463069	32.17917364	23.73358262	24.48958548	29.83743861	24.03098074	19.26846401	30.88640905
ENSMUSG00000001052	SEC24B	52.4379266	52.97416847	36.43704044	53.7105004	128.6231535	32.17917364	84.38607155	85.06908641	73.71602481	91.31772681	98.09399857	63.31713856
ENSMUSG00000001053	N4BP3	0	6.621771059	7.287408088	5.967833378	0	6.435834728	5.274129472	1.288925552	1.755143448	1.201549037	10.51007128	1.544320453
ENSMUSG00000001054	RMND5B	8.739654434	19.86531318	14.57481618	17.90350013	25.72463069	6.435834728	11.86679131	10.31140441	14.04114758	22.8294317	5.255035638	20.07616588
ENSMUSG00000001056	NHP2	201.012052	132.4354212	116.5985294	179.0350013	154.3477841	199.5108766	210.9651789	202.3613116	217.6377875	173.0230613	171.6644975	225.4707861
ENSMUSG00000001062	VPS9D1	17.47930887	13.24354212	14.57481618	11.93566676	6.431157673	6.435834728	13.18532368	11.60032996	5.265430344	6.007745185	7.006714184	6.177281811
ENSMUSG00000001065	ZFP276	0	0	29.14963235	17.90350013	51.44926138	12.87166946	13.18532368	15.46710662	15.79629103	19.22478459	19.26846401	27.79776815
ENSMUSG00000001082	MFSD10	26.2189633	6.621771059	43.72444853	23.87133351	12.86231535	0	3.955597104	12.88925552	12.28600414	3.604647111	8.75839273	1.544320453
ENSMUSG00000001089	LUZP1	0	6.621771059	0	0	0	12.87166946	1.318532368	3.866776655	5.265430344	3.604647111	10.51007128	7.721602263
ENSMUSG00000001098	KCTD10	43.69827217	39.73062635	21.86222426	11.93566676	6.431157673	25.74333891	34.28184157	25.77851103	21.06172137	16.82168652	22.7718211	21.62048634
ENSMUSG00000001100	POLDIP2	26.2189633	26.48708424	14.57481618	35.80700027	25.72463069	6.435834728	27.68917973	21.91173438	26.32715172	16.82168652	31.53021383	32.43072951
ENSMUSG00000001105	IFT20	61.17758104	66.21771059	109.3111213	65.64616716	51.44926138	70.79418201	60.65248893	74.757682	70.20573791	81.70533452	84.08057021	75.67170218
ENSMUSG00000001123	LGALS9	26.2189633	6.621771059	14.57481618	11.93566676	12.86231535	0	3.955597104	7.73355331	12.28600414	6.007745185	17.51678546	18.53184543
ENSMUSG00000001127	ARAF	61.17758104	79.46125271	43.72444853	53.7105004	102.8985228	57.92251255	73.83781261	68.31305424	78.98145515	70.89139318	87.5839273	75.67170218

ENSMUSG00000001128	CFP	0	6.621771059	21.86222426	23.87133351	6.431157673	12.87166946	9.229726576	18.04495772	10.53086069	2.403098074	8.75839273	0
ENSMUSG00000001131	TIMP1	0	0	0	0	0	0	0	1.288925552	1.755143448	1.201549037	0	0
ENSMUSG00000001134	UXT	8.739654434	0	0	5.967833378	12.86231535	0	7.911194208	5.155702207	7.020573791	15.62013748	5.255035638	13.89888407
ENSMUSG00000001138	CNNM3	26.2189633	52.97416847	29.14963235	47.74266702	12.86231535	45.05084309	35.60037394	43.82346876	29.83743861	25.23252978	40.28860656	41.69665222
ENSMUSG00000001143	LMAN2L	8.739654434	0	14.57481618	11.93566676	0	6.435834728	9.229726576	19.33388327	14.04114758	9.612392296	8.75839273	7.721602263
ENSMUSG00000001150	MCM3AP	26.2189633	19.86531318	21.86222426	11.93566676	19.29347302	32.17917364	19.77798552	25.77851103	22.81686482	38.44956918	24.52349964	37.06369086
ENSMUSG00000001151	PCNT	148.5741254	158.9225054	116.5985294	83.54966729	147.9166265	122.2808598	93.61579813	96.66941637	107.0637503	115.3487076	119.1141411	83.39330444
ENSMUSG00000001156	MXD1	0	19.86531318	14.57481618	11.93566676	0	0	6.59266184	16.75603217	12.28600414	7.209294222	7.006714184	9.265922716
ENSMUSG00000001157	GMCL1	78.65688991	33.10885529	14.57481618	35.80700027	38.58694604	64.35834728	31.64477683	28.35636214	42.12344275	40.85266726	19.26846401	29.3420886
ENSMUSG00000001158	SNRNP27	113.6155076	92.70479482	58.2992647	95.48533405	64.31157673	109.4091904	102.8455247	91.51371417	86.00202895	57.67435378	85.83224875	72.58306128
ENSMUSG00000001166	OAS1C	0	6.621771059	7.287408088	5.967833378	12.86231535	0	1.318532368	5.155702207	3.510286896	3.604647111	3.503357092	0
ENSMUSG00000001173	OCRL	17.47930887	6.621771059	14.57481618	23.87133351	12.86231535	0	7.911194208	6.444627758	7.020573791	3.604647111	5.255035638	10.81024317
ENSMUSG00000001175	CALM1	1678.013651	1350.841296	1574.080147	1336.794677	1569.202472	1518.856996	1119.43398	1203.856465	1079.41322	1167.905664	1142.094412	1210.747235
ENSMUSG00000001211	AGPAT3	61.17758104	52.97416847	43.72444853	35.80700027	83.60504975	51.48667782	46.14863288	61.86842648	52.65430344	52.86815763	54.30203492	64.86145901
ENSMUSG00000001225	SLC26A3	0	0	0	0	0	0	0	1.288925552	1.755143448	1.201549037	0	0
ENSMUSG00000001227	SEMA6B	0	6.621771059	0	11.93566676	0	0	0	3.866776655	0	3.604647111	1.751678546	3.088640905
ENSMUSG00000001228	UHRF1	297.1482508	119.1918791	153.0355698	149.1958345	212.2282032	276.7408933	210.9651789	241.0290782	236.9443655	228.294317	213.7047826	239.3696702
ENSMUSG00000001229	DPP9	61.17758104	52.97416847	36.43704044	29.83916689	19.29347302	51.48667782	38.23743867	51.55702207	42.12344275	46.86041244	35.03357092	32.43072951
ENSMUSG00000001240	RAMP2	0	0	0	0	0	0	0	0	1.755143448	0	0	0
ENSMUSG00000001247	LSR	0	0	0	5.967833378	0	0	1.318532368	0	0	2.403098074	0	0
ENSMUSG00000001248	GRAMD1A	122.3551621	119.1918791	123.8859375	83.54966729	109.3296804	141.588364	110.7567189	110.8475974	112.3291807	103.3332172	122.6174982	95.74786807
ENSMUSG00000001249	HPN	0	0	0	0	0	0	0	1.288925552	0	0	0	0
ENSMUSG00000001280	SP1	8.739654434	19.86531318	21.86222426	29.83916689	19.29347302	25.74333891	25.05211499	29.64528769	26.32715172	30.03872593	31.53021383	27.79776815
ENSMUSG00000001281	ITGB7	87.39654434	112.570108	145.7481618	137.2601677	90.03620742	90.10168619	102.8455247	146.9375129	136.9011889	114.1471585	126.1208553	137.4445203
ENSMUSG00000001285	MYG1	69.91723547	19.86531318	65.58667279	23.87133351	64.31157673	51.48667782	61.9710213	65.73520313	71.96088136	60.07745185	42.0402851	57.13985675
ENSMUSG00000001288	RARG	17.47930887	0	14.57481618	11.93566676	12.86231535	0	11.86679131	5.155702207	14.04114758	10.81394133	8.75839273	1.544320453
ENSMUSG00000001289	PFDN5	148.5741254	198.6531318	233.1970588	137.2601677	180.0724148	102.9733556	137.1273663	140.4928851	157.9629103	154.9998258	190.9329615	157.5206862
ENSMUSG00000001300	EFNB2	0	0	0	0	0	0	0	1.288925552	0	0	0	0
ENSMUSG00000001305	RRP15	34.95861774	39.73062635	14.57481618	41.77483365	19.29347302	19.30750418	48.78569762	52.84594762	47.38887309	38.44956918	40.28860656	63.31713856
ENSMUSG00000001313	RND2	0	0	0	0	0	0	1.318532368	0	1.755143448	0	3.503357092	0
ENSMUSG00000001323	SRR	26.2189633	39.73062635	14.57481618	0	6.431157673	25.74333891	13.18532368	12.88925552	14.04114758	21.62788267	14.01342837	18.53184543
ENSMUSG00000001333	SYNC	8.739654434	19.86531318	14.57481618	17.90350013	12.86231535	12.87166946	6.59266184	12.88925552	10.53086069	9.612392296	19.26846401	13.89888407
ENSMUSG00000001334	FNDC5	0	6.621771059	0	0	0	6.435834728	0	1.288925552	0	1.201549037	3.503357092	0
ENSMUSG00000001349	CNN1	0	0	0	0	0	0	0	0	0	0	1.751678546	0
ENSMUSG00000001366	FBXO9	26.2189633	39.73062635	29.14963235	17.90350013	19.29347302	12.87166946	29.0077121	38.66776655	33.34772551	28.83717689	40.28860656	38.60801132
ENSMUSG00000001376	VPS50	61.17758104	39.73062635	51.01185661	53.7105004	38.58694604	57.92251255	51.42276235	37.378841	43.8785862	40.85266726	38.53692801	52.50689539
ENSMUSG00000001380	HARS	148.5741254	99.32656588	196.7600184	143.2280011	109.3296804	77.23001673	127.8976397	162.4046195	154.4526234	142.9843354	168.1611404	129.722918
ENSMUSG00000001383	ZMAT2	148.5741254	125.8136501	145.7481618	125.3245009	147.9166265	160.8958682	127.8976397	194.6277583	164.9834841	120.1549037	161.1544262	193.0400566
ENSMUSG00000001403	UBE2C	43.69827217	86.08302377	51.01185661	59.67833378	45.01810371	64.35834728	106.8011218	67.02412869	87.75717239	62.48054992	94.59064148	89.57058625
ENSMUSG00000001415	SMG5	104.8758532	172.1660475	131.1733456	143.2280011	154.3477841	102.9733556	98.8899276	139.2039596	114.0843241	106.9378643	115.610784	132.8115589
ENSMUSG00000001416	CCT3	305.8879052	364.1974082	400.8074448	393.877003	360.1448297	302.4842322	404.789437	474.324603	530.0533213	445.7746927	530.7585994	461.7518153
ENSMUSG00000001418	GLMP	26.2189633	13.24354212	36.43704044	29.83916689	25.72463069	32.17917364	30.32624446	34.80098989	40.3682993	39.65111822	28.02685674	40.15233177
ENSMUSG00000001419	MEF2D	96.13619877	39.73062635	43.72444853	65.64616716	45.01810371	77.23001673	60.65248893	83.78016086	89.51231584	84.10843259	85.83224875	80.30466354
ENSMUSG00000001420	TMEM79	17.47930887	6.621771059	0	11.93566676	0	0	2.637064736	2.577851103	1.755143448	2.403098074	7.006714184	1.544320453
ENSMUSG00000001424	SND1	1345.906783	1059.483369	1333.59568	1074.210008	1189.764169	1209.936929	1176.130872	1313.415137	1286.520147	1116.239055	1017.725235	1289.507578

ENSMUSG00000001436	SLC19A1	26.2189633	13.24354212	14.57481618	17.90350013	0	6.435834728	26.37064736	19.33388327	7.020573791	19.22478459	19.26846401	18.53184543
ENSMUSG00000001440	KPNB1	288.4085963	317.8450108	298.7837316	328.2308358	270.1086223	347.5350753	307.2180417	358.3213034	365.0698372	370.0771034	336.3222808	367.5482677
ENSMUSG00000001441	NPEPPS	166.0534342	165.5442765	58.2992647	137.2601677	141.4854688	96.53752092	117.3493808	101.8251186	140.4114758	130.968845	129.6242124	106.5581112
ENSMUSG00000001445	MRPL10	0	13.24354212	21.86222426	35.80700027	38.58694604	25.74333891	44.83010051	37.378841	43.8785862	51.66660859	35.03357092	43.24097267
ENSMUSG00000001467	CYP51	87.39654434	72.83948165	102.0237132	101.4531674	141.4854688	64.35834728	126.5791073	131.4704063	142.1666193	127.3641979	129.6242124	137.4445203
ENSMUSG00000001472	TCF25	166.0534342	258.2490713	204.0474265	137.2601677	257.2463069	218.8183807	188.5501286	201.0723861	186.0452055	182.6354536	225.9665324	196.1286975
ENSMUSG00000001482	DEF8	8.739654434	13.24354212	0	23.87133351	6.431157673	12.87166946	17.14092078	11.60032996	19.30657793	12.01549037	12.26174982	16.98752498
ENSMUSG00000001494	SOST	8.739654434	0	0	0	0	0	0	0	0	0	0	0
ENSMUSG00000001517	FOXM1	17.47930887	33.10885529	43.72444853	53.7105004	57.88041905	77.23001673	39.55597104	42.5345432	31.59258206	34.84492207	50.79867783	40.15233177
ENSMUSG00000001518	ITFG2	43.69827217	46.35239741	65.58667279	11.93566676	25.72463069	12.87166946	27.68917973	28.35636214	22.81686482	22.8294317	28.02685674	30.88640905
ENSMUSG00000001520	NRIP2	0	0	0	0	6.431157673	0	0	0	0	0	0	1.544320453
ENSMUSG00000001521	TULP3	8.739654434	39.73062635	0	35.80700027	51.44926138	25.74333891	15.82238842	9.022478862	21.06172137	12.01549037	8.75839273	6.177281811
ENSMUSG00000001524	GTF2H4	26.2189633	26.48708424	65.58667279	29.83916689	57.88041905	32.17917364	43.51156814	38.66776655	43.8785862	48.06196148	33.28189237	46.32961358
ENSMUSG00000001525	TUBB5	830.2671712	1105.835767	1005.662316	1104.049175	1228.351115	1267.859441	882.0981542	987.3169726	974.1046136	872.3246009	853.0674519	1030.061742
ENSMUSG00000001542	ELL2	26.2189633	13.24354212	21.86222426	23.87133351	25.72463069	45.05084309	48.78569762	42.5345432	56.16459033	32.441824	49.04699929	23.16480679
ENSMUSG00000001552	JUP	0	0	7.287408088	0	0	0	0	0	0	0	0	0
ENSMUSG00000001558	KLHL10	0	0	7.287408088	0	0	0	0	0	0	0	0	0
ENSMUSG00000001569	NOM1	87.39654434	59.59593953	43.72444853	59.67833378	51.44926138	64.35834728	75.15634498	76.04660755	61.43002068	69.68984415	105.1007128	84.9376249
ENSMUSG00000001576	ERGIC1	43.69827217	72.83948165	87.44889705	71.61400054	96.46736509	51.48667782	80.43047445	104.4029697	119.3497545	94.92237392	91.08728439	100.3808294
ENSMUSG00000001583	TNK1	0	0	0	0	0	0	0	1.288925552	0	0	1.751678546	0
ENSMUSG00000001588	ACAP1	131.0948165	132.4354212	94.73630514	125.3245009	141.4854688	90.10168619	94.9343305	104.4029697	100.0431765	68.48829511	103.3490342	88.0262658
ENSMUSG00000001604	TCEA3	0	0	0	0	0	0	0	1.288925552	0	0	0	0
ENSMUSG00000001627	IFRD1	61.17758104	92.70479482	94.73630514	113.3888342	83.60504975	90.10168619	141.0829634	148.2264384	129.8806151	140.5812373	143.6376408	95.74786807
ENSMUSG00000001630	STK38L	17.47930887	26.48708424	14.57481618	23.87133351	12.86231535	0	10.54825894	18.04495772	10.53086069	14.41858844	29.77853528	21.62048634
ENSMUSG00000001632	BRPF1	131.0948165	112.570108	80.16148896	173.067168	96.46736509	109.4091904	80.43047445	103.1140441	100.0431765	117.7518056	96.34232003	95.74786807
ENSMUSG00000001642	AKR1B3	26.2189633	0	7.287408088	0	19.29347302	12.87166946	3.955597104	14.17818107	8.775717239	12.01549037	10.51007128	13.89888407
ENSMUSG00000001657	HOXC8	17.47930887	0	14.57481618	11.93566676	19.29347302	19.30750418	9.229726576	5.155702207	1.755143448	7.209294222	7.006714184	9.265922716
ENSMUSG00000001661	HOXC6	17.47930887	13.24354212	14.57481618	17.90350013	19.29347302	25.74333891	17.14092078	6.444627758	12.28600414	26.43407881	17.51678546	15.44320453
ENSMUSG00000001663	GSTT1	0	0	0	0	0	0	0	1.288925552	0	1.201549037	0	0
ENSMUSG00000001666	DDT	26.2189633	19.86531318	7.287408088	41.77483365	12.86231535	32.17917364	34.28184157	30.93421324	26.32715172	22.8294317	10.51007128	38.60801132
ENSMUSG00000001674	DDX18	96.13619877	79.46125271	80.16148896	149.1958345	122.1919958	102.9733556	59.33395656	64.44627758	80.7365986	82.90688355	78.82553457	84.9376249
ENSMUSG00000001687	UBL3	218.4913609	211.8966739	240.4844669	196.9385015	225.0905185	154.4600335	205.6910494	264.2297381	261.5163737	269.1469843	261.0001033	245.546952
ENSMUSG00000001707	EEF1E1	122.3551621	152.3007344	94.73630514	95.48533405	167.2100995	135.1525293	148.9941576	188.1831305	154.4526234	157.4029238	140.1342837	146.710443
ENSMUSG00000001729	AKT1	122.3551621	79.46125271	94.73630514	83.54966729	102.8985228	109.4091904	93.61579813	104.4029697	86.00202895	103.3332172	94.59064148	122.0013158
ENSMUSG00000001741	IL16	201.012052	172.1660475	160.3229779	167.0993346	205.7970455	173.7675376	200.4169199	207.5170138	173.7592013	175.4261594	229.4698895	166.7866089
ENSMUSG00000001750	TCIRG1	43.69827217	26.48708424	43.72444853	23.87133351	45.01810371	32.17917364	39.55597104	38.66776655	59.67487723	48.06196148	29.77853528	33.97504996
ENSMUSG00000001751	NAGLU	0	0	0	0	0	0	0	1.288925552	1.755143448	0	0	0
ENSMUSG00000001755	PPAT	0	19.86531318	0	0	19.29347302	6.435834728	14.50385605	7.73355331	17.55143448	9.612392296	14.01342837	9.265922716
ENSMUSG00000001763	TSPAN33	0	0	7.287408088	5.967833378	0	0	3.955597104	1.288925552	1.755143448	1.201549037	0	1.544320453
ENSMUSG00000001767	CRNKL1	78.65688991	33.10885529	14.57481618	17.90350013	57.88041905	38.61500837	60.65248893	52.84594762	75.47116826	49.26351052	43.79196365	47.87393403
ENSMUSG00000001774	CHORDC1	52.4379266	59.59593953	65.58667279	65.64616716	90.03620742	83.66585146	60.65248893	74.757682	57.91973378	67.28674607	80.57721311	81.84898399
ENSMUSG00000001783	RTCB	61.17758104	72.83948165	94.73630514	101.4531674	102.8985228	25.74333891	93.61579813	77.3355331	57.91973378	61.27900089	61.30874911	80.30466354
ENSMUSG00000001785	PWP1	26.2189633	26.48708424	51.01185661	11.93566676	38.58694604	25.74333891	40.87450341	41.24561765	52.65430344	30.03872593	40.28860656	57.13985675
ENSMUSG00000001786	FBXO7	17.47930887	13.24354212	29.14963235	17.90350013	12.86231535	6.435834728	11.86679131	25.77851103	22.81686482	19.22478459	29.77853528	18.53184543
ENSMUSG00000001794	CAPNS1	17.47930887	59.59593953	51.01185661	59.67833378	64.31157673	57.92251255	59.33395656	52.84594762	57.91973378	60.07745185	71.81882038	86.48194535

ENSMUSG00000001819	HOXD13	0	0	0	0	0	0	0	0	0	0	0	1.544320453
ENSMUSG00000001829	CLPB	52.4379266	13.24354212	29.14963235	29.83916689	38.58694604	32.17917364	26.37064736	20.62280883	33.34772551	38.44956918	24.52349964	41.69665222
ENSMUSG00000001833	39326	201.012052	350.9538661	255.0592831	292.4238355	282.9709376	283.176728	362.5964012	329.9649412	386.1315585	442.1700456	362.597459	353.6493837
ENSMUSG00000001844	ZDHHC4	17.47930887	13.24354212	7.287408088	23.87133351	12.86231535	12.87166946	11.86679131	11.60032996	12.28600414	14.41858844	14.01342837	13.89888407
ENSMUSG00000001847	RAC1	192.2723975	139.0571922	138.4607537	161.1315012	173.6412572	141.588364	159.5424165	159.8267684	212.3723572	130.968845	145.3893193	197.6730179
ENSMUSG00000001855	NUP214	34.95861774	52.97416847	58.2992647	53.7105004	70.7427344	38.61500837	44.83010051	54.13487317	47.38887309	48.06196148	47.29532074	44.78529313
ENSMUSG00000001864	AIF1L	0	0	0	5.967833378	0	0	0	0	0	0	0	1.544320453
ENSMUSG00000001865	CPA3	332.1068685	370.8191793	291.4963235	316.295169	360.1448297	289.6125627	383.6929191	357.0323778	401.9278496	346.0461227	418.6511725	458.6631744
ENSMUSG00000001891	UGP2	52.4379266	66.21771059	65.58667279	89.51750067	38.58694604	45.05084309	87.02313629	70.89090534	77.22631171	76.89913837	68.31546329	60.22849765
ENSMUSG00000001909	TRMT1	122.3551621	152.3007344	160.3229779	137.2601677	173.6412572	109.4091904	113.3937836	118.5811508	124.6151848	118.9533547	119.1141411	129.722918
ENSMUSG00000001910	NACC1	26.2189633	39.73062635	36.43704044	35.80700027	38.58694604	32.17917364	48.78569762	42.5345432	35.10286896	36.04647111	21.02014255	46.32961358
ENSMUSG00000001911	NFIX	8.739654434	13.24354212	7.287408088	0	6.431157673	0	3.955597104	6.444627758	3.510286896	7.209294222	5.255035638	6.177281811
ENSMUSG00000001918	SLC1A5	297.1482508	278.1143845	364.3704044	304.3595023	257.2463069	186.6392071	268.9806031	293.8750258	263.2715172	242.7129055	283.7719244	270.2560792
ENSMUSG00000001924	UBA1	113.6155076	99.32656588	145.7481618	113.3888342	77.17389207	70.79418201	110.7567189	125.0257785	115.8394676	126.1626489	147.1409979	137.4445203
ENSMUSG00000001942	SIAE	17.47930887	26.48708424	29.14963235	17.90350013	6.431157673	19.30750418	23.73358262	18.04495772	28.08229517	20.42633363	26.27517819	20.07616588
ENSMUSG00000001946	ESAM	8.739654434	0	0	0	0	0	0	0	0	0	0	1.544320453
ENSMUSG00000001948	SPA17	8.739654434	6.621771059	21.86222426	0	6.431157673	19.30750418	10.54825894	19.33388327	7.020573791	14.41858844	19.26846401	23.16480679
ENSMUSG00000001962	FAM50A	34.95861774	46.35239741	21.86222426	17.90350013	12.86231535	32.17917364	35.60037394	43.82346876	31.59258206	32.441824	36.78524947	26.2534477
ENSMUSG00000001964	EMD	43.69827217	46.35239741	14.57481618	11.93566676	6.431157673	19.30750418	27.68917973	55.42379872	59.67487723	45.65886341	49.04699929	47.87393403
ENSMUSG00000001983	TACO1	0	0	0	0	6.431157673	0	1.318532368	3.866776655	3.510286896	1.201549037	1.751678546	0
ENSMUSG00000001986	GRIA3	8.739654434	0	0	0	0	0	0	0	0	0	0	0
ENSMUSG00000001988	NPAS1	0	0	0	0	0	0	0	0	0	0	1.751678546	0
ENSMUSG00000001998	AP4E1	0	13.24354212	7.287408088	0	19.29347302	25.74333891	3.955597104	6.444627758	5.265430344	10.81394133	5.255035638	4.632961358
ENSMUSG00000001999	BLVRA	17.47930887	6.621771059	72.87408088	65.64616716	57.88041905	25.74333891	58.01542419	56.71272427	70.20573791	36.04647111	43.79196365	55.5955363
ENSMUSG00000002006	PDZD4	0	0	0	5.967833378	6.431157673	0	2.637064736	1.288925552	3.510286896	0	0	4.632961358
ENSMUSG00000002007	SRPK3	0	0	7.287408088	0	0	0	1.318532368	0	0	2.403098074	3.503357092	0
ENSMUSG00000002010	IDH3G	96.13619877	19.86531318	87.44889705	29.83916689	45.01810371	70.79418201	59.33395656	68.31305424	66.69545102	69.68984415	77.07385602	83.39330444
ENSMUSG00000002014	SSR4	122.3551621	178.7878186	189.4726103	173.067168	180.0724148	186.6392071	207.0095818	221.6951949	208.8620703	211.4726305	185.6779259	225.4707861
ENSMUSG00000002015	BCAP31	87.39654434	59.59593953	36.43704044	89.51750067	70.7427344	51.48667782	80.43047445	92.80263972	98.28803308	99.72857007	110.3557484	128.1785976
ENSMUSG00000002017	FAM98A	61.17758104	19.86531318	14.57481618	23.87133351	6.431157673	25.74333891	29.0077121	37.378841	28.08229517	27.63562785	19.26846401	30.88640905
ENSMUSG00000002028	KMT2A	419.5034128	403.9280346	262.3466912	364.0378361	366.5759873	373.2784142	208.3281141	211.3837905	222.9032179	270.3485333	206.6980684	220.8378247
ENSMUSG00000002031	IFT46	26.2189633	13.24354212	58.2992647	17.90350013	19.29347302	19.30750418	25.05211499	18.04495772	10.53086069	21.62788267	22.7718211	16.98752498
ENSMUSG00000002032	TMEM25	0	0	0	0	0	12.87166946	1.318532368	0	0	2.403098074	5.255035638	0
ENSMUSG00000002033	CD3G	900.1844067	761.5036718	845.3393382	716.1400054	610.9599789	598.5326297	829.3568595	826.2012786	879.3268674	702.9061867	765.4835246	886.4399398
ENSMUSG00000002043	TRAPPC6A	8.739654434	6.621771059	7.287408088	5.967833378	25.72463069	12.87166946	10.54825894	9.022478862	12.28600414	9.612392296	17.51678546	15.44320453
ENSMUSG00000002052	SUPT6	52.4379266	59.59593953	102.0237132	125.3245009	109.3296804	83.66585146	63.28955366	82.49123531	100.0431765	93.72082489	103.3490342	81.84898399
ENSMUSG00000002055	SPAG5	96.13619877	79.46125271	94.73630514	101.4531674	122.1919958	90.10168619	90.97873339	104.4029697	122.8600414	85.30998163	87.5839273	91.11490671
ENSMUSG00000002058	UNC119	0	13.24354212	7.287408088	17.90350013	0	0	11.86679131	6.444627758	8.775717239	10.81394133	3.503357092	4.632961358
ENSMUSG00000002059	RAB34	0	0	0	0	0	0	0	0	0	1.201549037	0	0
ENSMUSG00000002064	SDF2	0	19.86531318	7.287408088	5.967833378	19.29347302	19.30750418	22.41505026	21.91173438	19.30657793	21.62788267	28.02685674	20.07616588
ENSMUSG00000002068	CCNE1	52.4379266	26.48708424	43.72444853	41.77483365	25.72463069	25.74333891	43.51156814	32.22313879	21.06172137	27.63562785	22.7718211	26.2534477
ENSMUSG00000002083	BBC3	122.3551621	59.59593953	58.2992647	71.61400054	57.88041905	51.48667782	65.9266184	69.60197979	63.18516412	50.46505955	70.06714184	72.58306128
ENSMUSG00000002102	PSMC3	183.5327431	152.3007344	153.0355698	131.2923343	128.6231535	109.4091904	142.4014957	122.4479274	150.9423365	147.7905316	145.3893193	185.3184543
ENSMUSG00000002103	ACP2	8.739654434	19.86531318	7.287408088	23.87133351	38.58694604	45.05084309	26.37064736	18.04495772	21.06172137	55.2712557	8.75839273	21.62048634
ENSMUSG00000002104	RAPSN	34.95861774	13.24354212	14.57481618	53.7105004	19.29347302	32.17917364	21.09651789	11.60032996	7.020573791	12.01549037	17.51678546	26.2534477

ENSMUSG00000002105	SLC39A13	0	0	0	5.967833378	0	12.87166946	3.955597104	6.444627758	3.510286896	2.403098074	1.751678546	6.177281811
ENSMUSG00000002107	CELF2	908.9240611	913.8044061	1042.099357	787.7540059	829.6193398	817.3510104	698.822155	621.2621159	691.5265185	700.5030886	697.1680613	647.0702697
ENSMUSG00000002108	NR1H3	0	0	0	0	0	0	0	0	0	0	3.503357092	1.544320453
ENSMUSG00000002109	DDB2	96.13619877	46.35239741	65.58667279	59.67833378	70.7427344	102.9733556	60.65248893	60.57950093	66.69545102	92.51927585	56.05371347	63.31713856
ENSMUSG00000002111	SPI1	0	0	0	0	0	0	0	0	1.755143448	0	0	0
ENSMUSG00000002129	SF3A1	87.39654434	99.32656588	94.73630514	71.61400054	102.8985228	64.35834728	117.3493808	105.6918952	138.6563324	99.72857007	106.8523913	94.20354761
ENSMUSG00000002147	STAT6	69.91723547	52.97416847	36.43704044	65.64616716	45.01810371	45.05084309	44.83010051	52.84594762	43.8785862	31.24027496	35.03357092	55.5955363
ENSMUSG00000002204	NAPSA	0	0	7.287408088	0	0	0	2.637064736	0	3.510286896	0	3.503357092	0
ENSMUSG00000002205	VRK3	34.95861774	19.86531318	21.86222426	17.90350013	25.72463069	19.30750418	21.09651789	24.48958548	22.81686482	22.8294317	17.51678546	24.70912724
ENSMUSG00000002210	SMG9	0	26.48708424	21.86222426	5.967833378	25.72463069	12.87166946	14.50385605	9.022478862	15.79629103	12.01549037	12.26174982	15.44320453
ENSMUSG00000002221	PAXIP1	104.8758532	59.59593953	65.58667279	59.67833378	57.88041905	57.92251255	68.56368314	58.00164982	49.14401654	69.68984415	64.8121062	58.6841772
ENSMUSG00000002222	RMND5A	227.2310153	139.0571922	138.4607537	137.2601677	122.1919958	173.7675376	162.1794813	128.8925552	121.1048979	126.1626489	134.879248	112.735393
ENSMUSG00000002227	MOV10	122.3551621	99.32656588	123.8859375	59.67833378	70.7427344	135.1525293	58.01542419	81.20230975	89.51231584	58.87590281	71.81882038	60.22849765
ENSMUSG00000002228	PPM1J	0	0	0	5.967833378	0	0	0	0	0	0	0	1.544320453
ENSMUSG00000002233	RHOC	0	0	0	5.967833378	0	0	2.637064736	6.444627758	0	1.201549037	0	3.088640905
ENSMUSG00000002250	PPARD	26.2189633	26.48708424	58.2992647	35.80700027	45.01810371	19.30750418	36.9189063	43.82346876	21.06172137	15.62013748	22.7718211	37.06369086
ENSMUSG00000002257	DEF6	78.65688991	66.21771059	123.8859375	125.3245009	90.03620742	115.8450251	93.61579813	97.95834193	96.53288963	86.51153067	124.3691768	98.83650897
ENSMUSG00000002274	METRN	139.8344709	86.08302377	160.3229779	125.3245009	90.03620742	96.53752092	123.9420426	116.0032996	121.1048979	108.1394133	119.1141411	109.6467521
ENSMUSG00000002279	LMF1	17.47930887	19.86531318	36.43704044	41.77483365	19.29347302	6.435834728	15.82238842	19.33388327	21.06172137	9.612392296	14.01342837	21.62048634
ENSMUSG00000002280	NARFL	34.95861774	13.24354212	36.43704044	11.93566676	12.86231535	32.17917364	22.41505026	19.33388327	26.32715172	18.02323556	33.28189237	18.53184543
ENSMUSG00000002289	ANGPTL4	43.69827217	19.86531318	29.14963235	17.90350013	25.72463069	6.435834728	27.68917973	23.20065993	35.10286896	19.22478459	31.53021383	26.2534477
ENSMUSG00000002297	DBF4	192.2723975	165.5442765	160.3229779	179.0350013	167.2100995	141.588364	148.9941576	118.5811508	154.4526234	148.9920806	169.912819	132.8115589
ENSMUSG00000002307	DAXX	61.17758104	99.32656588	58.2992647	47.74266702	57.88041905	51.48667782	54.05982709	70.89090534	49.14401654	66.08519704	57.80539202	75.67170218
ENSMUSG00000002308	CD320	8.739654434	19.86531318	65.58667279	35.80700027	45.01810371	25.74333891	31.64477683	25.77851103	22.81686482	31.24027496	28.02685674	30.88640905
ENSMUSG00000002319	IPO4	113.6155076	39.73062635	36.43704044	119.3566676	128.6231535	96.53752092	67.24515077	82.49123531	94.77774619	70.89139318	68.31546329	74.12738173
ENSMUSG00000002320	TM9SF1	8.739654434	0	7.287408088	0	6.431157673	19.30750418	5.274129472	9.022478862	3.510286896	7.209294222	7.006714184	4.632961358
ENSMUSG00000002325	IRF9	8.739654434	19.86531318	7.287408088	5.967833378	25.72463069	0	3.955597104	6.444627758	5.265430344	7.209294222	8.75839273	3.088640905
ENSMUSG00000002326	GMPR2	17.47930887	33.10885529	14.57481618	17.90350013	32.15578836	32.17917364	26.37064736	25.77851103	26.32715172	32.441824	21.02014255	18.53184543
ENSMUSG00000002329	MDP1	34.95861774	26.48708424	29.14963235	35.80700027	38.58694604	32.17917364	35.60037394	42.5345432	31.59258206	25.23252978	52.55035638	33.97504996
ENSMUSG00000002332	DHRS1	8.739654434	13.24354212	14.57481618	23.87133351	19.29347302	0	15.82238842	16.75603217	7.020573791	18.02323556	15.76510691	10.81024317
ENSMUSG00000002342	TMEM161A	52.4379266	39.73062635	43.72444853	65.64616716	96.46736509	32.17917364	59.33395656	69.60197979	84.2468855	51.66660859	70.06714184	78.76034309
ENSMUSG00000002343	ARMC6	34.95861774	19.86531318	21.86222426	17.90350013	6.431157673	6.435834728	11.86679131	11.60032996	21.06172137	12.01549037	14.01342837	9.265922716
ENSMUSG00000002345	BORCS8	26.2189633	6.621771059	14.57481618	11.93566676	12.86231535	12.87166946	9.229726576	15.46710662	15.79629103	6.007745185	15.76510691	13.89888407
ENSMUSG00000002346	SLC25A42	0	6.621771059	0	0	6.431157673	6.435834728	3.955597104	7.73355331	14.04114758	8.410843259	0	6.177281811
ENSMUSG00000002365	SNX9	26.2189633	19.86531318	14.57481618	41.77483365	12.86231535	32.17917364	15.82238842	37.378841	26.32715172	22.8294317	17.51678546	30.88640905
ENSMUSG00000002372	RANBP3	166.0534342	192.0313607	160.3229779	161.1315012	199.3658879	180.2033724	138.4458986	163.6935451	180.7797751	175.4261594	176.9195331	163.697968
ENSMUSG00000002379	NDUFA11	122.3551621	92.70479482	109.3111213	77.58183392	160.7789418	122.2808598	133.1717692	148.2264384	177.2694882	133.3719431	150.644355	149.7990839
ENSMUSG00000002393	NR2F6	8.739654434	13.24354212	36.43704044	29.83916689	25.72463069	12.87166946	13.18532368	12.88925552	22.81686482	21.62788267	22.7718211	21.62048634
ENSMUSG00000002395	USE1	52.4379266	33.10885529	14.57481618	35.80700027	25.72463069	70.79418201	39.55597104	54.13487317	47.38887309	44.45731437	56.05371347	60.22849765
ENSMUSG00000002396	OCEL1	0	13.24354212	14.57481618	17.90350013	6.431157673	6.435834728	5.274129472	2.577851103	7.020573791	4.806196148	10.51007128	4.632961358
ENSMUSG00000002409	DYRK1B	8.739654434	0	0	11.93566676	0	6.435834728	2.637064736	2.577851103	1.755143448	4.806196148	0	3.088640905
ENSMUSG00000002413	BRAF	131.0948165	105.9483369	196.7600184	143.2280011	83.60504975	77.23001673	79.11194208	91.51371417	112.3291807	55.2712557	113.8591055	97.29218852
ENSMUSG00000002416	NDUFB2	148.5741254	125.8136501	138.4607537	107.4210008	154.3477841	57.92251255	114.712316	116.0032996	140.4114758	117.7518056	99.84567712	162.1536475
ENSMUSG00000002428	HLTF	52.4379266	99.32656588	80.16148896	107.4210008	122.1919958	70.79418201	96.25286286	95.38049082	96.53288963	112.9456095	98.09399857	94.20354761
ENSMUSG00000002455	PRPF6	61.17758104	52.97416847	72.87408088	47.74266702	64.31157673	32.17917364	68.56368314	74.757682	78.98145515	62.48054992	66.56378475	88.0262658

ENSMUSG00000002458	RGS19	0	6.621771059	14.57481618	29.83916689	12.86231535	0	25.05211499	37.378841	42.12344275	32.441824	21.02014255	18.53184543
ENSMUSG00000002459	RGS20	0	0	0	0	0	0	0	1.288925552	0	0	0	0
ENSMUSG00000002477	SNRPD1	253.4499786	258.2490713	218.6222426	244.6811685	315.126726	218.8183807	354.685207	355.7434523	361.5595503	317.2089458	374.8592088	443.2199699
ENSMUSG00000002486	TCHP	0	26.48708424	14.57481618	17.90350013	25.72463069	12.87166946	23.73358262	28.35636214	19.30657793	20.42633363	22.7718211	12.35456362
ENSMUSG00000002489	TIAM1	17.47930887	59.59593953	36.43704044	53.7105004	57.88041905	45.05084309	43.51156814	25.77851103	40.3682993	28.83717689	31.53021383	43.24097267
ENSMUSG00000002496	TSC2	8.739654434	39.73062635	43.72444853	35.80700027	57.88041905	45.05084309	34.28184157	37.378841	43.8785862	32.441824	38.53692801	38.60801132
ENSMUSG00000002524	PUF60	104.8758532	99.32656588	65.58667279	107.4210008	141.4854688	128.7166946	114.712316	161.115694	136.9011889	132.1703941	169.912819	134.3558794
ENSMUSG00000002546	GOLGA2	78.65688991	52.97416847	72.87408088	95.48533405	102.8985228	70.79418201	64.60808603	48.97917096	56.16459033	54.06970667	80.57721311	64.86145901
ENSMUSG00000002550	UCK1	26.2189633	13.24354212	29.14963235	17.90350013	6.431157673	12.87166946	25.05211499	20.62280883	24.57200827	25.23252978	24.52349964	32.43072951
ENSMUSG00000002578	IKZF4	0	13.24354212	0	5.967833378	0	0	0	0	0	0	0	0
ENSMUSG00000002580	MIEN1	0	0	21.86222426	11.93566676	0	6.435834728	0	0	0	0	1.751678546	0
ENSMUSG00000002602	AXL	8.739654434	0	14.57481618	5.967833378	19.29347302	12.87166946	6.59266184	6.444627758	3.510286896	3.604647111	5.255035638	4.632961358
ENSMUSG00000002603	TGFB1	8.739654434	6.621771059	21.86222426	23.87133351	12.86231535	12.87166946	11.86679131	10.31140441	22.81686482	10.81394133	24.52349964	15.44320453
ENSMUSG00000002608	CCDC97	43.69827217	19.86531318	43.72444853	29.83916689	32.15578836	32.17917364	26.37064736	39.9566921	40.3682993	26.43407881	26.27517819	40.15233177
ENSMUSG00000002617	ZFP40	26.2189633	33.10885529	36.43704044	41.77483365	25.72463069	25.74333891	26.37064736	25.77851103	24.57200827	39.65111822	24.52349964	38.60801132
ENSMUSG00000002625	AKAP8L	0	6.621771059	7.287408088	0	0	19.30750418	1.318532368	3.866776655	5.265430344	4.806196148	5.255035638	13.89888407
ENSMUSG00000002635	PDCD2L	78.65688991	13.24354212	80.16148896	23.87133351	38.58694604	51.48667782	51.42276235	76.04660755	66.69545102	46.86041244	57.80539202	38.60801132
ENSMUSG00000002658	GTF2F1	166.0534342	231.7619871	196.7600184	208.8741682	173.6412572	186.6392071	156.9053518	170.1381728	187.8003489	183.8370027	206.6980684	203.8502998
ENSMUSG00000002660	CLPP	113.6155076	66.21771059	72.87408088	53.7105004	115.7608381	57.92251255	77.79340971	87.64693751	100.0431765	82.90688355	89.33560584	64.86145901
ENSMUSG00000002661	ALKBH7	78.65688991	39.73062635	87.44889705	101.4531674	102.8985228	45.05084309	65.9266184	63.15735203	82.49174205	44.45731437	71.81882038	54.05121584
ENSMUSG00000002668	DENND1C	131.0948165	105.9483369	153.0355698	125.3245009	147.9166265	64.35834728	106.8011218	136.6261085	126.3703282	112.9456095	129.6242124	137.4445203
ENSMUSG00000002679	MED6	26.2189633	13.24354212	65.58667279	29.83916689	32.15578836	64.35834728	43.51156814	46.40131986	45.63372964	37.24802015	42.0402851	29.3420886
ENSMUSG00000002688	PRKD1	0	0	0	0	0	0	0	0	0	0	1.751678546	0
ENSMUSG00000002699	LCP2	87.39654434	92.70479482	72.87408088	65.64616716	45.01810371	57.92251255	83.06753918	76.04660755	66.69545102	97.325472	70.06714184	84.9376249
ENSMUSG00000002718	CSE1L	43.69827217	139.0571922	72.87408088	59.67833378	38.58694604	32.17917364	89.66020102	87.64693751	89.51231584	98.52702104	84.08057021	72.58306128
ENSMUSG00000002728	NAA20	61.17758104	33.10885529	51.01185661	59.67833378	45.01810371	25.74333891	44.83010051	74.757682	68.45059447	50.46505955	52.55035638	78.76034309
ENSMUSG00000002731	PRKRA	8.739654434	13.24354212	14.57481618	5.967833378	6.431157673	6.435834728	19.77798552	12.88925552	22.81686482	8.410843259	10.51007128	9.265922716
ENSMUSG00000002732	FKBP7	8.739654434	6.621771059	7.287408088	0	0	0	11.86679131	10.31140441	12.28600414	12.01549037	3.503357092	9.265922716
ENSMUSG00000002733	PLEKHA3	166.0534342	139.0571922	145.7481618	190.9706681	160.7789418	83.66585146	191.1871934	226.8508971	243.9649393	186.2401007	185.6779259	220.8378247
ENSMUSG00000002741	YKT6	43.69827217	59.59593953	21.86222426	47.74266702	38.58694604	12.87166946	25.05211499	45.11239431	43.8785862	39.65111822	35.03357092	49.41825449
ENSMUSG00000002748	BAZ1B	769.0895902	609.2029374	808.9022977	811.6253394	829.6193398	720.8134895	655.3105869	604.5060837	605.5244895	629.6116954	679.6512758	596.1076947
ENSMUSG00000002763	PEX6	113.6155076	46.35239741	58.2992647	59.67833378	45.01810371	64.35834728	40.87450341	28.35636214	66.69545102	31.24027496	54.30203492	44.78529313
ENSMUSG00000002767	MRPL2	8.739654434	46.35239741	58.2992647	29.83916689	90.03620742	70.79418201	71.20074787	91.51371417	64.94030757	62.48054992	75.32217748	71.03874082
ENSMUSG00000002768	MEA1	17.47930887	19.86531318	36.43704044	5.967833378	12.86231535	19.30750418	34.28184157	36.08991545	29.83743861	26.43407881	31.53021383	20.07616588
ENSMUSG00000002769	GNMT	0	0	0	0	0	0	0	0	3.510286896	2.403098074	0	3.088640905
ENSMUSG00000002778	KDELR1	96.13619877	105.9483369	116.5985294	101.4531674	115.7608381	96.53752092	141.0829634	135.3371829	145.6769062	104.5347662	105.1007128	125.0899567
ENSMUSG00000002781	TMEM143	8.739654434	0	0	0	6.431157673	0	2.637064736	6.444627758	1.755143448	1.201549037	0	1.544320453
ENSMUSG00000002797	GGCT	43.69827217	26.48708424	21.86222426	35.80700027	19.29347302	12.87166946	27.68917973	23.20065993	33.34772551	21.62788267	31.53021383	44.78529313
ENSMUSG00000002799	JAG2	0	0	0	0	0	6.435834728	2.637064736	1.288925552	0	1.201549037	0	3.088640905
ENSMUSG00000002803	BTBD6	8.739654434	6.621771059	7.287408088	0	12.86231535	6.435834728	2.637064736	6.444627758	3.510286896	6.007745185	5.255035638	1.544320453
ENSMUSG00000002804	NUDT14	8.739654434	0	7.287408088	5.967833378	12.86231535	0	6.59266184	2.577851103	5.265430344	1.201549037	7.006714184	4.632961358
ENSMUSG00000002812	FLII	87.39654434	59.59593953	102.0237132	107.4210008	96.46736509	57.92251255	56.69689182	73.46875644	68.45059447	56.47280474	85.83224875	84.9376249
ENSMUSG00000002814	TOP3A	8.739654434	6.621771059	7.287408088	11.93566676	25.72463069	6.435834728	13.18532368	10.31140441	10.53086069	13.21703941	12.26174982	18.53184543
ENSMUSG00000002820	ATG4D	61.17758104	66.21771059	43.72444853	47.74266702	70.7427344	45.05084309	40.87450341	25.77851103	47.38887309	38.44956918	50.79867783	46.32961358
ENSMUSG00000002825	QTRT1	43.69827217	46.35239741	58.2992647	65.64616716	19.29347302	45.05084309	51.42276235	50.26809651	56.16459033	46.86041244	56.05371347	60.22849765

ENSMUSG00000002831	PLIN4	0	0	14.57481618	0	19.29347302	12.87166946	6.59266184	3.866776655	5.265430344	2.403098074	3.503357092	0
ENSMUSG00000002833	HDGFL2	113.6155076	145.6789633	109.3111213	149.1958345	173.6412572	141.588364	89.66020102	135.3371829	131.6357586	102.1316681	140.1342837	126.6342771
ENSMUSG00000002835	CHAF1A	139.8344709	139.0571922	138.4607537	149.1958345	167.2100995	83.66585146	131.8532368	159.8267684	157.9629103	134.5734921	152.3960335	135.9001998
ENSMUSG00000002844	ADPRH	26.2189633	19.86531318	14.57481618	17.90350013	19.29347302	12.87166946	17.14092078	18.04495772	24.57200827	21.62788267	17.51678546	26.2534477
ENSMUSG00000002845	TMEM39A	17.47930887	46.35239741	51.01185661	41.77483365	19.29347302	38.61500837	50.10422998	52.84594762	47.38887309	45.65886341	40.28860656	50.96257494
ENSMUSG00000002846	TIMMDC1	104.8758532	46.35239741	72.87408088	77.58183392	70.7427344	83.66585146	51.42276235	46.40131986	50.89915999	42.0542163	43.79196365	37.06369086
ENSMUSG00000002847	PLA1A	0	6.621771059	0	0	0	0	5.274129472	2.577851103	0	2.403098074	5.255035638	1.544320453
ENSMUSG00000002870	MCM2	174.7930887	311.2232398	255.0592831	250.6490019	192.9347302	238.1258849	309.8551065	313.2089091	287.8435255	446.9762418	339.8256379	284.1549633
ENSMUSG00000002871	TPRA1	26.2189633	33.10885529	29.14963235	17.90350013	25.72463069	25.74333891	19.77798552	25.77851103	26.32715172	30.03872593	40.28860656	26.2534477
ENSMUSG00000002881	NAB1	96.13619877	72.83948165	123.8859375	95.48533405	109.3296804	109.4091904	77.79340971	68.31305424	84.2468855	110.5425114	82.32889166	88.0262658
ENSMUSG00000002885	ADGRE5	69.91723547	46.35239741	65.58667279	41.77483365	77.17389207	83.66585146	63.28955366	54.13487317	89.51231584	51.66660859	59.55707056	81.84898399
ENSMUSG00000002897	IL17RA	104.8758532	52.97416847	94.73630514	143.2280011	77.17389207	96.53752092	77.79340971	90.22478862	75.47116826	72.09294222	70.06714184	88.0262658
ENSMUSG00000002900	LAMB1	0	0	0	0	0	0	0	0	1.755143448	0	0	0
ENSMUSG00000002908	KCNN1	0	0	0	0	0	0	0	0	0	1.201549037	0	0
ENSMUSG00000002910	ARRDC2	0	6.621771059	7.287408088	35.80700027	25.72463069	0	7.911194208	6.444627758	12.28600414	4.806196148	7.006714184	7.721602263
ENSMUSG00000002944	CD36	0	0	0	5.967833378	0	0	0	0	0	0	0	0
ENSMUSG00000002948	MAP2K7	96.13619877	26.48708424	29.14963235	29.83916689	25.72463069	45.05084309	30.32624446	41.24561765	43.8785862	37.24802015	47.29532074	43.24097267
ENSMUSG00000002949	TIMM44	69.91723547	105.9483369	116.5985294	77.58183392	77.17389207	83.66585146	87.02313629	79.9133842	89.51231584	58.87590281	101.5973557	57.13985675
ENSMUSG00000002957	AP2A2	78.65688991	46.35239741	116.5985294	35.80700027	90.03620742	57.92251255	77.79340971	95.38049082	98.28803308	78.10068741	75.32217748	89.57058625
ENSMUSG00000002963	PNKP	26.2189633	6.621771059	36.43704044	11.93566676	6.431157673	19.30750418	14.50385605	19.33388327	17.55143448	14.41858844	21.02014255	13.89888407
ENSMUSG00000002968	MED25	87.39654434	79.46125271	72.87408088	47.74266702	45.01810371	57.92251255	55.37835946	51.55702207	56.16459033	67.28674607	49.04699929	57.13985675
ENSMUSG00000002980	BCAM	0	0	0	0	0	0	0	0	1.755143448	0	0	0
ENSMUSG00000002981	CLPTM1	61.17758104	59.59593953	29.14963235	41.77483365	57.88041905	102.9733556	75.15634498	96.66941637	101.79832	86.51153067	80.57721311	101.9251499
ENSMUSG00000002983	RELB	43.69827217	13.24354212	0	0	12.86231535	6.435834728	3.955597104	6.444627758	8.775717239	1.201549037	3.503357092	4.632961358
ENSMUSG00000002984	TOMM40	166.0534342	125.8136501	116.5985294	131.2923343	122.1919958	238.1258849	148.9941576	149.515364	161.4731972	108.1394133	164.6577833	152.8877248
ENSMUSG00000002985	APOE	34.95861774	19.86531318	29.14963235	35.80700027	25.72463069	32.17917364	21.09651789	23.20065993	35.10286896	9.612392296	19.26846401	21.62048634
ENSMUSG00000002996	HBP1	26.2189633	52.97416847	36.43704044	29.83916689	25.72463069	51.48667782	38.23743867	28.35636214	36.85801241	34.84492207	54.30203492	33.97504996
ENSMUSG00000002997	PRKAR2B	0	6.621771059	0	11.93566676	0	12.87166946	10.54825894	3.866776655	10.53086069	9.612392296	14.01342837	6.177281811
ENSMUSG00000003031	CDKN1B	314.6275596	350.9538661	298.7837316	399.8448363	321.5578836	360.4067448	431.1600843	398.2779955	449.3167227	430.1545553	404.6377441	420.0551631
ENSMUSG00000003033	AP1M1	34.95861774	46.35239741	36.43704044	41.77483365	45.01810371	45.05084309	30.32624446	29.64528769	24.57200827	21.62788267	24.52349964	27.79776815
ENSMUSG00000003037	RAB8A	78.65688991	132.4354212	65.58667279	83.54966729	122.1919958	70.79418201	96.25286286	113.4254485	112.3291807	86.51153067	92.83896294	109.6467521
ENSMUSG00000003038	HMGN2	26.2189633	33.10885529	14.57481618	29.83916689	32.15578836	45.05084309	44.83010051	39.9566921	36.85801241	42.0542163	43.79196365	57.13985675
ENSMUSG00000003039	FAM32A	26.2189633	66.21771059	72.87408088	83.54966729	115.7608381	38.61500837	94.9343305	94.09156527	68.45059447	75.69758933	103.3490342	83.39330444
ENSMUSG00000003051	ELF3	0	0	0	0	0	0	0	0	0	0	1.751678546	0
ENSMUSG00000003062	STARD3NL	34.95861774	33.10885529	51.01185661	53.7105004	51.44926138	38.61500837	38.23743867	33.51206434	36.85801241	45.65886341	35.03357092	50.96257494
ENSMUSG00000003068	STK11	104.8758532	86.08302377	58.2992647	155.1636678	141.4854688	90.10168619	88.34166866	105.6918952	96.53288963	111.7440604	122.6174982	92.65922716
ENSMUSG00000003070	EFNA2	0	0	0	0	0	0	0	1.288925552	1.755143448	1.201549037	1.751678546	0
ENSMUSG00000003072	ATP5D	262.189633	205.2749028	255.0592831	256.6168353	199.3658879	173.7675376	208.3281141	244.8958548	261.5163737	215.0772776	217.2081397	279.5220019
ENSMUSG00000003099	PPP5C	262.189633	245.0055292	174.8977941	244.6811685	250.8151492	218.8183807	185.9130639	184.3163539	200.0863531	204.2633363	183.9262473	220.8378247
ENSMUSG00000003119	CDK12	148.5741254	112.570108	145.7481618	131.2923343	173.6412572	154.4600335	117.3493808	104.4029697	100.0431765	144.1858844	129.6242124	138.9888407
ENSMUSG00000003123	LIPE	26.2189633	13.24354212	21.86222426	11.93566676	12.86231535	12.87166946	11.86679131	14.17818107	8.775717239	10.81394133	5.255035638	12.35456362
ENSMUSG00000003131	PAFAH1B2	113.6155076	46.35239741	94.73630514	101.4531674	135.0543111	122.2808598	117.3493808	121.1590019	145.6769062	115.3487076	82.32889166	131.2672385
ENSMUSG00000003134	TBC1D8	26.2189633	13.24354212	14.57481618	11.93566676	6.431157673	12.87166946	19.77798552	15.46710662	14.04114758	8.410843259	15.76510691	13.89888407
ENSMUSG00000003135	CNOT11	43.69827217	33.10885529	87.44889705	77.58183392	57.88041905	96.53752092	71.20074787	68.31305424	78.98145515	99.72857007	91.08728439	72.58306128
ENSMUSG00000003153	SLC2A3	69.91723547	46.35239741	87.44889705	95.48533405	38.58694604	77.23001673	60.65248893	76.04660755	61.43002068	60.07745185	87.5839273	72.58306128

ENSMUSG00000003154	FOXJ2	52.4379266	13.24354212	21.86222426	17.90350013	38.58694604	45.05084309	11.86679131	15.46710662	17.55143448	19.22478459	7.006714184	18.53184543
ENSMUSG00000003161	SRI	78.65688991	125.8136501	80.16148896	101.4531674	102.8985228	154.4600335	129.2161721	132.7593318	126.3703282	110.5425114	152.3960335	122.0013158
ENSMUSG00000003166	DGCR2	34.95861774	26.48708424	58.2992647	41.77483365	51.44926138	51.48667782	48.78569762	34.80098989	36.85801241	44.45731437	47.29532074	26.2534477
ENSMUSG00000003184	IRF3	34.95861774	33.10885529	36.43704044	23.87133351	38.58694604	45.05084309	32.9633092	51.55702207	43.8785862	31.24027496	43.79196365	43.24097267
ENSMUSG00000003190	BCL2L12	26.2189633	33.10885529	21.86222426	17.90350013	25.72463069	51.48667782	27.68917973	28.35636214	49.14401654	27.63562785	29.77853528	26.2534477
ENSMUSG00000003198	ZFP959	8.739654434	6.621771059	0	17.90350013	19.29347302	12.87166946	6.59266184	12.88925552	15.79629103	14.41858844	3.503357092	7.721602263
ENSMUSG00000003199	MPND	43.69827217	39.73062635	43.72444853	59.67833378	77.17389207	45.05084309	51.42276235	60.57950093	50.89915999	36.04647111	50.79867783	71.03874082
ENSMUSG00000003200	SH3GL1	26.2189633	46.35239741	43.72444853	41.77483365	45.01810371	51.48667782	40.87450341	50.26809651	36.85801241	37.24802015	43.79196365	60.22849765
ENSMUSG00000003206	EBI3	0	0	0	0	0	0	0	0	1.755143448	0	0	0
ENSMUSG00000003208	CCDC94	17.47930887	0	7.287408088	11.93566676	38.58694604	19.30750418	19.77798552	18.04495772	22.81686482	8.410843259	17.51678546	20.07616588
ENSMUSG00000003226	RANBP2	174.7930887	205.2749028	160.3229779	155.1636678	167.2100995	109.4091904	129.2161721	149.515364	168.493771	148.9920806	152.3960335	146.710443
ENSMUSG00000003228	GRK5	0	6.621771059	21.86222426	5.967833378	0	0	13.18532368	5.155702207	7.020573791	8.410843259	19.26846401	3.088640905
ENSMUSG00000003233	DVL3	43.69827217	26.48708424	51.01185661	47.74266702	38.58694604	38.61500837	27.68917973	39.9566921	26.32715172	30.03872593	38.53692801	40.15233177
ENSMUSG00000003234	ABCF3	26.2189633	19.86531318	21.86222426	35.80700027	19.29347302	12.87166946	22.41505026	14.17818107	10.53086069	18.02323556	15.76510691	23.16480679
ENSMUSG00000003235	EIF2B5	52.4379266	13.24354212	29.14963235	17.90350013	64.31157673	38.61500837	51.42276235	47.69024541	36.85801241	37.24802015	40.28860656	54.05121584
ENSMUSG00000003269	CYTH2	8.739654434	33.10885529	51.01185661	17.90350013	32.15578836	12.87166946	30.32624446	30.93421324	47.38887309	38.44956918	31.53021383	38.60801132
ENSMUSG00000003271	SULT2B1	0	0	0	0	0	0	2.637064736	9.022478862	3.510286896	3.604647111	1.751678546	6.177281811
ENSMUSG00000003279	DLGAP1	0	0	7.287408088	0	0	0	0	1.288925552	0	0	0	0
ENSMUSG00000003282	PLAG1	0	0	0	0	0	0	0	0	0	2.403098074	0	0
ENSMUSG00000003299	MRPL4	34.95861774	39.73062635	14.57481618	11.93566676	64.31157673	25.74333891	35.60037394	36.08991545	35.10286896	34.84492207	42.0402851	29.3420886
ENSMUSG00000003308	KEAP1	34.95861774	52.97416847	36.43704044	59.67833378	64.31157673	83.66585146	55.37835946	54.13487317	86.00202895	57.67435378	75.32217748	64.86145901
ENSMUSG00000003309	AP1M2	8.739654434	6.621771059	7.287408088	0	6.431157673	0	6.59266184	9.022478862	12.28600414	9.612392296	7.006714184	7.721602263
ENSMUSG00000003316	GLG1	122.3551621	112.570108	87.44889705	143.2280011	115.7608381	109.4091904	94.9343305	94.09156527	93.02260274	114.1471585	94.59064148	108.1024317
ENSMUSG00000003341	ATP8B3	0	6.621771059	0	0	0	0	0	0	0	0	0	0
ENSMUSG00000003344	BTBD2	0	0	0	5.967833378	6.431157673	6.435834728	3.955597104	3.866776655	1.755143448	0	1.751678546	1.544320453
ENSMUSG00000003345	CSNK1G2	69.91723547	33.10885529	65.58667279	35.80700027	77.17389207	32.17917364	50.10422998	51.55702207	61.43002068	64.883648	52.55035638	49.41825449
ENSMUSG00000003346	ABHD17A	69.91723547	46.35239741	80.16148896	83.54966729	57.88041905	45.05084309	72.51928024	81.20230975	78.98145515	69.68984415	71.81882038	81.84898399
ENSMUSG00000003348	MOB3A	17.47930887	33.10885529	65.58667279	53.7105004	90.03620742	32.17917364	59.33395656	55.42379872	63.18516412	42.0542163	49.04699929	66.40577946
ENSMUSG00000003354	CCDC65	0	0	0	0	0	0	0	0	0	1.201549037	0	0
ENSMUSG00000003355	FKBP11	0	0	0	0	0	0	2.637064736	0	0	2.403098074	0	4.632961358
ENSMUSG00000003360	DDX23	358.3258318	278.1143845	276.9215073	346.1343359	353.713672	308.9200669	230.7431644	199.7834605	317.6809641	217.4803757	225.9665324	265.6231179
ENSMUSG00000003363	PLD3	8.739654434	6.621771059	29.14963235	35.80700027	12.86231535	6.435834728	10.54825894	19.33388327	12.28600414	19.22478459	10.51007128	12.35456362
ENSMUSG00000003378	GRIK5	17.47930887	6.621771059	0	11.93566676	19.29347302	12.87166946	7.911194208	9.022478862	3.510286896	6.007745185	7.006714184	1.544320453
ENSMUSG00000003379	CD79A	0	0	0	0	0	0	1.318532368	0	0	1.201549037	1.751678546	1.544320453
ENSMUSG00000003380	RABAC1	0	46.35239741	36.43704044	29.83916689	32.15578836	25.74333891	44.83010051	47.69024541	28.08229517	36.04647111	47.29532074	35.51937041
ENSMUSG00000003382	ETV3	87.39654434	112.570108	131.1733456	137.2601677	109.3296804	135.1525293	76.47487734	68.31305424	68.45059447	90.11617778	99.84567712	58.6841772
ENSMUSG00000003402	PRKCSH	87.39654434	46.35239741	65.58667279	59.67833378	19.29347302	64.35834728	68.56368314	88.93586306	70.20573791	56.47280474	84.08057021	97.29218852
ENSMUSG00000003410	ELAVL3	0	0	0	5.967833378	0	6.435834728	1.318532368	1.288925552	0	1.201549037	3.503357092	3.088640905
ENSMUSG00000003421	NOSIP	26.2189633	33.10885529	43.72444853	53.7105004	25.72463069	25.74333891	67.24515077	48.97917096	22.81686482	34.84492207	40.28860656	49.41825449
ENSMUSG00000003423	PIH1D1	26.2189633	0	7.287408088	11.93566676	25.72463069	19.30750418	7.911194208	12.88925552	12.28600414	16.82168652	15.76510691	15.44320453
ENSMUSG00000003429	RPS11	917.6637156	668.7988769	852.6267462	704.2043386	771.7389207	868.8376882	845.1792479	924.1596205	944.267175	844.688973	977.4366286	982.1878079
ENSMUSG00000003435	SUPT5	104.8758532	66.21771059	138.4607537	101.4531674	90.03620742	122.2808598	87.02313629	112.136523	86.00202895	92.51927585	129.6242124	81.84898399
ENSMUSG00000003436	DLL3	0	0	0	0	0	0	0	2.577851103	0	0	0	0
ENSMUSG00000003437	PAF1	78.65688991	59.59593953	65.58667279	77.58183392	102.8985228	45.05084309	68.56368314	64.44627758	84.2468855	82.90688355	73.57049893	84.9376249
ENSMUSG00000003438	TIMM50	69.91723547	66.21771059	36.43704044	35.80700027	32.15578836	45.05084309	46.14863288	50.26809651	50.89915999	50.46505955	47.29532074	47.87393403

ENSMUSG00000003444	MED29	0	6.621771059	7.287408088	0	0	0	9.229726576	1.288925552	1.755143448	9.612392296	0	7.721602263
ENSMUSG00000003452	BICD1	17.47930887	33.10885529	7.287408088	41.77483365	25.72463069	19.30750418	39.55597104	29.64528769	19.30657793	44.45731437	29.77853528	29.3420886
ENSMUSG00000003458	NCSTN	26.2189633	39.73062635	58.2992647	35.80700027	25.72463069	51.48667782	38.23743867	38.66776655	47.38887309	37.24802015	36.78524947	38.60801132
ENSMUSG00000003464	PEX19	17.47930887	72.83948165	65.58667279	47.74266702	38.58694604	83.66585146	81.74900682	88.93586306	86.00202895	85.30998163	89.33560584	81.84898399
ENSMUSG00000003500	IMPDH1	52.4379266	72.83948165	72.87408088	41.77483365	32.15578836	25.74333891	34.28184157	33.51206434	59.67487723	45.65886341	38.53692801	40.15233177
ENSMUSG00000003526	PRODH	0	0	7.287408088	0	0	0	0	0	0	0	0	0
ENSMUSG00000003527	DGCR14	17.47930887	0	14.57481618	5.967833378	12.86231535	0	11.86679131	11.60032996	15.79629103	6.007745185	10.51007128	13.89888407
ENSMUSG00000003528	SLC25A1	17.47930887	39.73062635	43.72444853	35.80700027	45.01810371	25.74333891	35.60037394	21.91173438	28.08229517	24.03098074	29.77853528	40.15233177
ENSMUSG00000003531	DGCR6	0	13.24354212	7.287408088	0	12.86231535	19.30750418	35.60037394	15.46710662	19.30657793	19.22478459	15.76510691	18.53184543
ENSMUSG00000003541	IER3	0	0	0	0	0	0	0	0	0	1.201549037	0	0
ENSMUSG00000003545	FOSB	0	0	0	0	0	0	1.318532368	2.577851103	1.755143448	0	0	1.544320453
ENSMUSG00000003546	KLC4	8.739654434	6.621771059	7.287408088	11.93566676	12.86231535	6.435834728	10.54825894	6.444627758	5.265430344	13.21703941	14.01342837	21.62048634
ENSMUSG00000003549	ERCC1	8.739654434	6.621771059	7.287408088	5.967833378	12.86231535	19.30750418	9.229726576	12.88925552	14.04114758	12.01549037	14.01342837	10.81024317
ENSMUSG00000003559	AS3MT	0	0	0	0	0	0	0	0	1.755143448	0	1.751678546	0
ENSMUSG00000003573	HOMER3	0	6.621771059	7.287408088	5.967833378	0	6.435834728	2.637064736	7.73355331	3.510286896	8.410843259	7.006714184	7.721602263
ENSMUSG00000003575	CRTC1	8.739654434	0	7.287408088	5.967833378	6.431157673	6.435834728	13.18532368	9.022478862	8.775717239	13.21703941	15.76510691	16.98752498
ENSMUSG00000003581	RNF215	8.739654434	6.621771059	14.57481618	5.967833378	0	25.74333891	6.59266184	9.022478862	19.30657793	7.209294222	7.006714184	10.81024317
ENSMUSG00000003604	AVEN	52.4379266	26.48708424	14.57481618	11.93566676	12.86231535	25.74333891	27.68917973	20.62280883	14.04114758	38.44956918	24.52349964	18.53184543
ENSMUSG00000003617	CP	0	0	0	5.967833378	0	0	2.637064736	1.288925552	0	2.403098074	1.751678546	1.544320453
ENSMUSG00000003623	CROT	104.8758532	86.08302377	80.16148896	83.54966729	38.58694604	57.92251255	72.51928024	85.06908641	56.16459033	57.67435378	70.06714184	58.6841772
ENSMUSG00000003644	RPS6KA1	0	0	7.287408088	0	0	6.435834728	0	0	1.755143448	1.201549037	0	0
ENSMUSG00000003660	SNRNP200	69.91723547	72.83948165	72.87408088	59.67833378	70.7427344	96.53752092	100.20846	109.5586719	73.71602481	80.50378548	77.07385602	83.39330444
ENSMUSG00000003662	CIAO1	43.69827217	19.86531318	21.86222426	65.64616716	57.88041905	19.30750418	42.19303578	41.24561765	66.69545102	45.65886341	42.0402851	44.78529313
ENSMUSG00000003665	HAS1	0	0	0	0	0	0	0	0	0	0	0	1.544320453
ENSMUSG00000003680	TAF6L	8.739654434	13.24354212	14.57481618	0	0	19.30750418	9.229726576	15.46710662	19.30657793	4.806196148	14.01342837	16.98752498
ENSMUSG00000003721	INSIG2	34.95861774	46.35239741	51.01185661	71.61400054	51.44926138	57.92251255	47.46716525	38.66776655	40.3682993	28.83717689	52.55035638	46.32961358
ENSMUSG00000003731	KPNA6	139.8344709	33.10885529	36.43704044	41.77483365	70.7427344	70.79418201	85.70460392	91.51371417	77.22631171	63.68209896	92.83896294	71.03874082
ENSMUSG00000003746	MAN1A	17.47930887	19.86531318	14.57481618	0	38.58694604	6.435834728	6.59266184	11.60032996	12.28600414	18.02323556	19.26846401	16.98752498
ENSMUSG00000003752	ITPKC	8.739654434	0	7.287408088	5.967833378	12.86231535	6.435834728	6.59266184	3.866776655	3.510286896	1.201549037	3.503357092	1.544320453
ENSMUSG00000003762	COQ8B	0	0	0	0	6.431157673	0	5.274129472	5.155702207	5.265430344	4.806196148	3.503357092	3.088640905
ENSMUSG00000003778	BRD8	122.3551621	218.5184449	189.4726103	244.6811685	225.0905185	186.6392071	175.3648049	159.8267684	172.0040579	171.8215123	201.4430328	177.5968521
ENSMUSG00000003779	KIF20A	8.739654434	19.86531318	36.43704044	23.87133351	51.44926138	32.17917364	35.60037394	38.66776655	42.12344275	44.45731437	35.03357092	37.06369086
ENSMUSG00000003808	FARSA	69.91723547	59.59593953	65.58667279	95.48533405	45.01810371	57.92251255	63.28955366	73.46875644	77.22631171	76.89913837	84.08057021	92.65922716
ENSMUSG00000003809	GCDH	26.2189633	13.24354212	29.14963235	17.90350013	12.86231535	25.74333891	23.73358262	20.62280883	10.53086069	22.8294317	31.53021383	16.98752498
ENSMUSG00000003810	MAST2	69.91723547	52.97416847	80.16148896	65.64616716	102.8985228	51.48667782	60.65248893	45.11239431	68.45059447	56.47280474	59.55707056	60.22849765
ENSMUSG00000003812	DNASE2A	26.2189633	19.86531318	43.72444853	47.74266702	19.29347302	25.74333891	18.45945315	15.46710662	24.57200827	14.41858844	21.02014255	24.70912724
ENSMUSG00000003813	RAD23A	96.13619877	86.08302377	72.87408088	125.3245009	83.60504975	38.61500837	55.37835946	65.73520313	64.94030757	70.89139318	63.06042765	89.57058625
ENSMUSG00000003814	CALR	637.9947737	728.3948165	677.7289521	698.2365052	681.7027133	630.7118033	770.0229029	765.6217777	821.4071336	716.1232261	760.2284889	818.4898399
ENSMUSG00000003824	SYCE2	8.739654434	6.621771059	0	0	0	0	0	1.288925552	3.510286896	0	1.751678546	0
ENSMUSG00000003847	NFAT5	69.91723547	79.46125271	51.01185661	65.64616716	77.17389207	70.79418201	63.28955366	43.82346876	54.40944688	63.68209896	45.5436422	47.87393403
ENSMUSG00000003848	NOB1	61.17758104	86.08302377	36.43704044	35.80700027	70.7427344	45.05084309	59.33395656	46.40131986	49.14401654	61.27900089	47.29532074	69.49442037
ENSMUSG00000003849	NQO1	0	6.621771059	0	0	12.86231535	6.435834728	1.318532368	1.288925552	7.020573791	4.806196148	0	6.177281811
ENSMUSG00000003863	PPFIA3	0	0	0	0	0	0	0	0	0	1.201549037	0	0
ENSMUSG00000003865	GYS1	69.91723547	6.621771059	7.287408088	41.77483365	32.15578836	12.87166946	11.86679131	9.022478862	10.53086069	12.01549037	12.26174982	24.70912724
ENSMUSG00000003868	RUVBL2	104.8758532	92.70479482	65.58667279	89.51750067	64.31157673	96.53752092	96.25286286	105.6918952	96.53288963	106.9378643	103.3490342	126.6342771

ENSMUSG00000003872	LIN7B	0	6.621771059	0	0	0	0	0	0	1.755143448	0	0	0
ENSMUSG00000003873	BAX	87.39654434	66.21771059	58.2992647	95.48533405	109.3296804	70.79418201	72.51928024	74.757682	86.00202895	62.48054992	99.84567712	88.0262658
ENSMUSG00000003882	IL7R	0	0	7.287408088	5.967833378	0	0	0	0	1.755143448	1.201549037	0	0
ENSMUSG00000003923	TFAM	87.39654434	92.70479482	72.87408088	41.77483365	115.7608381	70.79418201	112.0752513	117.2922252	84.2468855	110.5425114	85.83224875	63.31713856
ENSMUSG00000003929	ZFP81	43.69827217	52.97416847	43.72444853	95.48533405	32.15578836	25.74333891	36.9189063	37.378841	43.8785862	37.24802015	28.02685674	41.69665222
ENSMUSG00000003934	EFNB3	8.739654434	6.621771059	0	11.93566676	6.431157673	0	1.318532368	1.288925552	1.755143448	6.007745185	0	7.721602263
ENSMUSG00000003948	MMD	8.739654434	46.35239741	29.14963235	41.77483365	25.72463069	32.17917364	34.28184157	28.35636214	33.34772551	22.8294317	22.7718211	33.97504996
ENSMUSG00000003955	FAM162A	122.3551621	52.97416847	123.8859375	101.4531674	115.7608381	70.79418201	168.7721431	137.915034	184.290062	122.5580018	131.3758909	114.2797135
ENSMUSG00000003970	RPL8	1922.723975	1787.878186	1727.115717	1796.317847	2000.090036	1686.188699	1454.341202	1509.331821	1576.118816	1375.773647	1530.967049	1718.828664
ENSMUSG00000003974	GRM3	0	0	0	0	0	0	0	1.288925552	0	0	0	0
ENSMUSG00000004018	FANCL	34.95861774	19.86531318	29.14963235	35.80700027	57.88041905	45.05084309	32.9633092	32.22313879	45.63372964	30.03872593	28.02685674	40.15233177
ENSMUSG00000004040	STAT3	122.3551621	59.59593953	94.73630514	83.54966729	64.31157673	45.05084309	65.9266184	72.17983089	91.26745929	94.92237392	94.59064148	80.30466354
ENSMUSG00000004043	STAT5A	8.739654434	33.10885529	0	23.87133351	25.72463069	19.30750418	18.45945315	23.20065993	22.81686482	15.62013748	17.51678546	20.07616588
ENSMUSG00000004044	CAVIN1	0	0	0	0	0	0	0	0	1.755143448	0	0	0
ENSMUSG00000004054	MAP3K11	34.95861774	26.48708424	21.86222426	29.83916689	12.86231535	6.435834728	18.45945315	27.06743658	15.79629103	16.82168652	17.51678546	13.89888407
ENSMUSG00000004056	AKT2	17.47930887	13.24354212	14.57481618	23.87133351	6.431157673	6.435834728	21.09651789	23.20065993	12.28600414	24.03098074	15.76510691	26.2534477
ENSMUSG00000004069	DNAJA3	122.3551621	99.32656588	51.01185661	89.51750067	38.58694604	70.79418201	77.79340971	78.62445865	101.79832	70.89139318	61.30874911	74.12738173
ENSMUSG00000004070	HMOX2	61.17758104	52.97416847	80.16148896	77.58183392	83.60504975	51.48667782	76.47487734	88.93586306	82.49174205	79.30223644	99.84567712	98.83650897
ENSMUSG00000004071	CDIP1	34.95861774	79.46125271	87.44889705	53.7105004	70.7427344	32.17917364	63.28955366	78.62445865	49.14401654	55.2712557	59.55707056	54.05121584
ENSMUSG00000004096	CWC15	148.5741254	172.1660475	131.1733456	155.1636678	122.1919958	135.1525293	222.8319702	216.5394927	180.7797751	179.0308065	199.6913542	217.7491838
ENSMUSG00000004099	DNMT1	349.5861774	377.4409504	371.6578125	370.0056694	340.8513567	270.3050586	287.4400562	278.4079192	307.1501034	313.6042987	364.3491376	339.7504996
ENSMUSG00000004100	PPAN	174.7930887	99.32656588	145.7481618	155.1636678	96.46736509	154.4600335	178.0018697	181.7385028	175.5143448	171.8215123	201.4430328	166.7866089
ENSMUSG00000004105	ANGPTL2	0	0	14.57481618	0	12.86231535	0	1.318532368	2.577851103	3.510286896	1.201549037	0	1.544320453
ENSMUSG00000004113	CACNA1B	0	0	0	0	6.431157673	0	0	0	7.020573791	0	1.751678546	0
ENSMUSG00000004127	TRMT10A	34.95861774	66.21771059	80.16148896	59.67833378	57.88041905	90.10168619	72.51928024	70.89090534	64.94030757	63.68209896	70.06714184	54.05121584
ENSMUSG00000004187	KIFC2	8.739654434	0	0	0	6.431157673	0	0	0	3.510286896	0	0	0
ENSMUSG00000004207	PSAP	8.739654434	0	0	0	6.431157673	6.435834728	17.14092078	21.91173438	15.79629103	26.43407881	22.7718211	16.98752498
ENSMUSG00000004221	IKBKG	17.47930887	13.24354212	0	0	0	19.30750418	9.229726576	20.62280883	12.28600414	14.41858844	15.76510691	18.53184543
ENSMUSG00000004233	WARS2	17.47930887	19.86531318	21.86222426	41.77483365	6.431157673	0	6.59266184	10.31140441	10.53086069	7.209294222	10.51007128	7.721602263
ENSMUSG00000004263	ATN1	0	26.48708424	36.43704044	47.74266702	45.01810371	6.435834728	17.14092078	15.46710662	19.30657793	19.22478459	24.52349964	18.53184543
ENSMUSG00000004264	PHB2	323.3672141	251.6273002	255.0592831	268.552502	392.300618	321.7917364	309.8551065	341.5652712	370.3352675	254.7283958	311.7987812	313.4970519
ENSMUSG00000004266	PTPN6	17.47930887	6.621771059	0	23.87133351	12.86231535	0	17.14092078	14.17818107	19.30657793	18.02323556	14.01342837	24.70912724
ENSMUSG00000004267	ENO2	0	6.621771059	0	5.967833378	0	0	0	5.155702207	1.755143448	3.604647111	1.751678546	3.088640905
ENSMUSG00000004268	EMG1	113.6155076	185.4095896	204.0474265	143.2280011	231.5216762	193.0750418	218.8763731	232.0065993	263.2715172	245.1160036	224.2148539	213.1162225
ENSMUSG00000004270	LPCAT3	43.69827217	46.35239741	29.14963235	47.74266702	25.72463069	19.30750418	36.9189063	20.62280883	43.8785862	28.83717689	42.0402851	38.60801132
ENSMUSG00000004285	ATP6V1F	34.95861774	13.24354212	29.14963235	29.83916689	57.88041905	57.92251255	55.37835946	54.13487317	43.8785862	60.07745185	66.56378475	57.13985675
ENSMUSG00000004317	CLCN5	26.2189633	19.86531318	21.86222426	35.80700027	19.29347302	32.17917364	10.54825894	11.60032996	17.55143448	20.42633363	8.75839273	18.53184543
ENSMUSG00000004319	CLCN3	332.1068685	357.5756372	218.6222426	340.1665026	340.8513567	341.0992406	304.580977	302.8975046	310.6603903	299.1857102	322.3088525	302.6868087
ENSMUSG00000004347	PDE1C	0	0	0	0	0	0	1.318532368	0	0	1.201549037	0	1.544320453
ENSMUSG00000004356	UTP20	78.65688991	52.97416847	138.4607537	77.58183392	77.17389207	51.48667782	73.83781261	67.02412869	82.49174205	78.10068741	96.34232003	80.30466354
ENSMUSG00000004364	CUL3	227.2310153	165.5442765	196.7600184	190.9706681	225.0905185	218.8183807	230.7431644	189.4720561	191.3106358	199.4571401	176.9195331	185.3184543
ENSMUSG00000004371	IL11	26.2189633	26.48708424	36.43704044	11.93566676	19.29347302	12.87166946	30.32624446	18.04495772	22.81686482	24.03098074	36.78524947	30.88640905
ENSMUSG00000004393	DDX56	43.69827217	52.97416847	43.72444853	11.93566676	38.58694604	25.74333891	52.74129472	54.13487317	59.67487723	55.2712557	77.07385602	49.41825449
ENSMUSG00000004394	TMED4	43.69827217	46.35239741	51.01185661	29.83916689	51.44926138	64.35834728	40.87450341	42.5345432	59.67487723	51.66660859	56.05371347	50.96257494
ENSMUSG00000004446	BID	8.739654434	0	0	5.967833378	19.29347302	0	11.86679131	9.022478862	3.510286896	6.007745185	14.01342837	10.81024317

ENSMUSG00000004451	RALB	0	0	0	0	0	0	1.318532368	0	0	1.201549037	0	0
ENSMUSG00000004455	PPP1CC	8.739654434	0	0	5.967833378	0	0	0	0	3.510286896	4.806196148	5.255035638	6.177281811
ENSMUSG00000004460	DNAJB11	61.17758104	52.97416847	116.5985294	95.48533405	160.7789418	83.66585146	61.9710213	104.4029697	80.7365986	74.4960403	103.3490342	80.30466354
ENSMUSG00000004462	TBCCD1	43.69827217	6.621771059	21.86222426	23.87133351	19.29347302	6.435834728	40.87450341	23.20065993	19.30657793	26.43407881	29.77853528	18.53184543
ENSMUSG00000004500	ZFP324	0	6.621771059	7.287408088	11.93566676	0	12.87166946	6.59266184	14.17818107	8.775717239	9.612392296	10.51007128	7.721602263
ENSMUSG00000004508	GAB2	0	6.621771059	7.287408088	23.87133351	6.431157673	6.435834728	6.59266184	5.155702207	14.04114758	3.604647111	3.503357092	6.177281811
ENSMUSG00000004530	CORO1C	87.39654434	66.21771059	109.3111213	77.58183392	70.7427344	96.53752092	85.70460392	79.9133842	70.20573791	62.48054992	73.57049893	77.21602263
ENSMUSG00000004535	TAX1BP1	349.5861774	331.0885529	357.0829963	328.2308358	295.8332529	321.7917364	363.9149336	371.2105589	317.6809641	347.2476717	341.5773165	336.6618587
ENSMUSG00000004558	NDRG2	0	0	0	5.967833378	0	0	0	0	0	0	0	0
ENSMUSG00000004561	METTL17	8.739654434	33.10885529	29.14963235	11.93566676	38.58694604	12.87166946	18.45945315	23.20065993	24.57200827	20.42633363	31.53021383	12.35456362
ENSMUSG00000004562	ARHGEF40	8.739654434	0	0	0	6.431157673	6.435834728	1.318532368	2.577851103	3.510286896	2.403098074	3.503357092	3.088640905
ENSMUSG00000004565	PNPLA6	0	39.73062635	0	23.87133351	38.58694604	19.30750418	15.82238842	23.20065993	17.55143448	26.43407881	19.26846401	13.89888407
ENSMUSG00000004567	MCOLN1	17.47930887	6.621771059	7.287408088	5.967833378	12.86231535	6.435834728	9.229726576	10.31140441	10.53086069	8.410843259	8.75839273	7.721602263
ENSMUSG00000004568	ARHGEF18	34.95861774	19.86531318	29.14963235	35.80700027	25.72463069	6.435834728	40.87450341	14.17818107	45.63372964	32.441824	24.52349964	23.16480679
ENSMUSG00000004591	PKN2	201.012052	165.5442765	131.1733456	185.0028347	250.8151492	180.2033724	148.9941576	184.3163539	170.2489144	134.5734921	157.6510691	131.2672385
ENSMUSG00000004610	ETFB	0	19.86531318	21.86222426	5.967833378	6.431157673	6.435834728	0	0	0	1.201549037	0	1.544320453
ENSMUSG00000004626	STXBP2	17.47930887	0	0	17.90350013	12.86231535	25.74333891	0	1.288925552	0	1.201549037	1.751678546	0
ENSMUSG00000004630	PCP2	0	0	0	0	0	0	0	0	0	1.201549037	0	1.544320453
ENSMUSG00000004633	CHN2	78.65688991	86.08302377	87.44889705	83.54966729	135.0543111	51.48667782	65.9266184	81.20230975	93.02260274	86.51153067	68.31546329	72.58306128
ENSMUSG00000004637	WWOX	0	19.86531318	0	0	19.29347302	0	2.637064736	2.577851103	5.265430344	3.604647111	1.751678546	4.632961358
ENSMUSG00000004642	SLBP	113.6155076	139.0571922	123.8859375	77.58183392	141.4854688	115.8450251	58.01542419	65.73520313	71.96088136	78.10068741	70.06714184	64.86145901
ENSMUSG00000004661	ARID3B	8.739654434	6.621771059	7.287408088	0	6.431157673	0	5.274129472	3.866776655	1.755143448	3.604647111	8.75839273	4.632961358
ENSMUSG00000004665	CNN2	183.5327431	192.0313607	160.3229779	167.0993346	192.9347302	199.5108766	148.9941576	172.7160239	133.390902	144.1858844	150.644355	165.2422884
ENSMUSG00000004667	POLR2E	69.91723547	72.83948165	80.16148896	47.74266702	51.44926138	57.92251255	96.25286286	116.0032996	96.53288963	84.10843259	131.3758909	118.9126749
ENSMUSG00000004677	MYO9B	96.13619877	46.35239741	51.01185661	41.77483365	32.15578836	32.17917364	35.60037394	43.82346876	38.61315585	46.86041244	50.79867783	35.51937041
ENSMUSG00000004698	HDAC9	17.47930887	13.24354212	0	17.90350013	6.431157673	0	6.59266184	3.866776655	15.79629103	13.21703941	12.26174982	1.544320453
ENSMUSG00000004707	LY9	87.39654434	39.73062635	109.3111213	101.4531674	90.03620742	83.66585146	76.47487734	70.89090534	91.26745929	58.87590281	70.06714184	55.5955363
ENSMUSG00000004709	CD244	8.739654434	6.621771059	14.57481618	41.77483365	6.431157673	0	13.18532368	16.75603217	15.79629103	16.82168652	10.51007128	7.721602263
ENSMUSG00000004730	ADGRE1	0	0	0	0	0	0	0	0	0	2.403098074	0	0
ENSMUSG00000004748	MTFP1	17.47930887	19.86531318	21.86222426	5.967833378	6.431157673	0	19.77798552	15.46710662	17.55143448	10.81394133	19.26846401	20.07616588
ENSMUSG00000004768	RAB23	34.95861774	19.86531318	14.57481618	17.90350013	12.86231535	19.30750418	29.0077121	18.04495772	19.30657793	22.8294317	21.02014255	26.2534477
ENSMUSG00000004771	RAB11A	52.4379266	46.35239741	36.43704044	59.67833378	45.01810371	70.79418201	55.37835946	63.15735203	66.69545102	69.68984415	59.55707056	52.50689539
ENSMUSG00000004788	EIF2B2	34.95861774	26.48708424	29.14963235	17.90350013	6.431157673	32.17917364	30.32624446	32.22313879	33.34772551	25.23252978	17.51678546	20.07616588
ENSMUSG00000004789	DLST	104.8758532	92.70479482	58.2992647	53.7105004	77.17389207	51.48667782	105.4825894	105.6918952	89.51231584	81.70533452	99.84567712	100.3808294
ENSMUSG00000004798	ULK2	0	0	21.86222426	0	12.86231535	6.435834728	7.911194208	9.022478862	12.28600414	8.410843259	7.006714184	3.088640905
ENSMUSG00000004815	DGKQ	17.47930887	6.621771059	36.43704044	11.93566676	12.86231535	6.435834728	15.82238842	19.33388327	10.53086069	19.22478459	24.52349964	20.07616588
ENSMUSG00000004837	GRAP	8.739654434	0	0	0	0	6.435834728	1.318532368	3.866776655	3.510286896	2.403098074	3.503357092	0
ENSMUSG00000004843	CHMP2B	17.47930887	19.86531318	14.57481618	17.90350013	12.86231535	25.74333891	25.05211499	23.20065993	33.34772551	26.43407881	33.28189237	20.07616588
ENSMUSG00000004846	PLOD3	26.2189633	46.35239741	29.14963235	17.90350013	45.01810371	25.74333891	29.0077121	36.08991545	33.34772551	24.03098074	31.53021383	30.88640905
ENSMUSG00000004849	AP1S1	43.69827217	112.570108	51.01185661	71.61400054	102.8985228	90.10168619	80.43047445	92.80263972	101.79832	98.52702104	80.57721311	103.4694703
ENSMUSG00000004865	SRPK1	218.4913609	271.4926134	276.9215073	298.3916689	315.126726	186.6392071	257.1138118	273.252217	272.0472344	246.3175526	315.3021383	242.4583111
ENSMUSG00000004880	LBR	288.4085963	251.6273002	255.0592831	226.7776684	237.9528339	218.8183807	280.8473944	232.0065993	242.2097958	266.7438862	241.7316393	253.2685542
ENSMUSG00000004885	CRABP2	0	0	0	5.967833378	0	0	1.318532368	3.866776655	1.755143448	2.403098074	7.006714184	1.544320453
ENSMUSG00000004891	NES	78.65688991	105.9483369	102.0237132	77.58183392	70.7427344	57.92251255	69.8822155	68.31305424	89.51231584	67.28674607	75.32217748	55.5955363
ENSMUSG00000004892	BCAN	8.739654434	0	7.287408088	0	0	0	0	0	0	0	0	0

ENSMUSG00000004895	PRCC	69.91723547	105.9483369	116.5985294	83.54966729	90.03620742	45.05084309	60.65248893	82.49123531	78.98145515	73.29449126	77.07385602	81.84898399
ENSMUSG00000004896	RRNAD1	209.7517064	192.0313607	65.58667279	101.4531674	109.3296804	160.8958682	104.1640571	99.24726748	145.6769062	97.325472	99.84567712	122.0013158
ENSMUSG00000004897	HDGF	332.1068685	390.6844925	313.3585478	370.0056694	443.7498794	315.3559017	385.0114515	418.9008043	356.2941199	360.4647111	373.1075303	381.4471518
ENSMUSG00000004929	THOP1	8.739654434	33.10885529	29.14963235	29.83916689	77.17389207	32.17917364	34.28184157	38.66776655	42.12344275	45.65886341	40.28860656	54.05121584
ENSMUSG00000004931	APBA3	0	0	0	0	0	0	3.955597104	1.288925552	1.755143448	0	1.751678546	0
ENSMUSG00000004933	MATK	0	0	0	0	0	6.435834728	0	2.577851103	0	0	0	0
ENSMUSG00000004934	PIAS4	96.13619877	99.32656588	145.7481618	83.54966729	90.03620742	109.4091904	64.60808603	86.35801196	89.51231584	74.4960403	87.5839273	78.76034309
ENSMUSG00000004936	MAP2K1	69.91723547	26.48708424	65.58667279	53.7105004	57.88041905	57.92251255	80.43047445	50.26809651	61.43002068	62.48054992	54.30203492	61.77281811
ENSMUSG00000004937	SGTA	34.95861774	13.24354212	43.72444853	53.7105004	51.44926138	38.61500837	52.74129472	33.51206434	49.14401654	46.86041244	59.55707056	58.6841772
ENSMUSG00000004939	NMRK2	0	0	0	0	0	0	0	1.288925552	0	0	0	0
ENSMUSG00000004945	TMEM242	34.95861774	13.24354212	29.14963235	35.80700027	38.58694604	51.48667782	30.32624446	41.24561765	42.12344275	50.46505955	54.30203492	37.06369086
ENSMUSG00000004947	DTX2	87.39654434	33.10885529	65.58667279	47.74266702	19.29347302	38.61500837	50.10422998	61.86842648	68.45059447	55.2712557	56.05371347	61.77281811
ENSMUSG00000004952	RASA4	0	0	0	0	0	0	1.318532368	0	0	1.201549037	0	1.544320453
ENSMUSG00000004980	HNRNPA2B1	1275.989547	1225.027646	1289.871231	1312.923343	1202.626485	1254.987772	1869.678898	1822.54073	1658.610558	1611.277259	1541.47712	1493.357878
ENSMUSG00000004988	FXYD4	17.47930887	19.86531318	29.14963235	17.90350013	0	6.435834728	14.50385605	5.155702207	19.30657793	4.806196148	8.75839273	13.89888407
ENSMUSG00000004994	CCDC130	0	6.621771059	7.287408088	17.90350013	19.29347302	0	3.955597104	7.73355331	7.020573791	7.209294222	1.751678546	4.632961358
ENSMUSG00000004996	MRI1	104.8758532	33.10885529	58.2992647	47.74266702	70.7427344	83.66585146	79.11194208	69.60197979	93.02260274	78.10068741	87.5839273	67.95009992
ENSMUSG00000005034	PRKACB	655.4740826	715.1512744	524.6933823	572.9120043	520.9237715	592.096795	719.9186729	613.5285626	663.4442233	869.9215028	697.1680613	619.2725015
ENSMUSG00000005043	SGSH	8.739654434	6.621771059	14.57481618	5.967833378	19.29347302	0	7.911194208	3.866776655	3.510286896	0	3.503357092	9.265922716
ENSMUSG00000005045	CHD5	8.739654434	0	7.287408088	0	0	0	2.637064736	2.577851103	5.265430344	0	0	4.632961358
ENSMUSG00000005054	CSTB	43.69827217	86.08302377	65.58667279	53.7105004	38.58694604	51.48667782	94.9343305	90.22478862	78.98145515	75.69758933	82.32889166	81.84898399
ENSMUSG00000005069	PEX5	0	46.35239741	58.2992647	41.77483365	57.88041905	38.61500837	23.73358262	51.55702207	45.63372964	31.24027496	52.55035638	33.97504996
ENSMUSG00000005078	JKAMP	17.47930887	26.48708424	51.01185661	53.7105004	51.44926138	32.17917364	30.32624446	27.06743658	31.59258206	40.85266726	43.79196365	33.97504996
ENSMUSG00000005087	CD44	192.2723975	152.3007344	189.4726103	119.3566676	141.4854688	128.7166946	93.61579813	106.9808208	145.6769062	120.1549037	124.3691768	152.8877248
ENSMUSG00000005102	EIF2AK4	34.95861774	26.48708424	21.86222426	17.90350013	12.86231535	25.74333891	34.28184157	43.82346876	43.8785862	43.25576533	47.29532074	37.06369086
ENSMUSG00000005103	WDR1	707.9120092	562.85054	612.1422794	447.5875034	514.4926138	566.353456	440.3898109	464.0131986	491.4401654	464.9994773	457.1881005	492.6382244
ENSMUSG00000005107	SLC2A9	0	0	0	0	6.431157673	0	1.318532368	1.288925552	0	1.201549037	3.503357092	0
ENSMUSG00000005125	NDRG1	0	6.621771059	0	0	0	0	0	0	1.755143448	0	0	0
ENSMUSG00000005131	4930550C14F	0	6.621771059	0	0	0	0	0	5.155702207	5.265430344	3.604647111	1.751678546	3.088640905
ENSMUSG00000005142	MAN2B1	122.3551621	238.3837581	131.1733456	185.0028347	96.46736509	141.588364	129.2161721	121.1590019	157.9629103	116.5502566	105.1007128	131.2672385
ENSMUSG00000005148	KLF5	0	0	0	0	0	0	0	1.288925552	0	0	0	0
ENSMUSG00000005150	WDR83	0	19.86531318	43.72444853	5.967833378	6.431157673	19.30750418	13.18532368	12.88925552	12.28600414	13.21703941	7.006714184	10.81024317
ENSMUSG00000005161	PRDX2	148.5741254	112.570108	58.2992647	125.3245009	83.60504975	96.53752092	130.5347044	149.515364	196.5760662	129.767296	154.147712	166.7866089
ENSMUSG00000005198	POLR2A	61.17758104	52.97416847	145.7481618	125.3245009	96.46736509	83.66585146	87.02313629	78.62445865	103.5534634	76.89913837	101.5973557	118.9126749
ENSMUSG00000005204	SENP3	34.95861774	19.86531318	29.14963235	17.90350013	32.15578836	32.17917364	39.55597104	43.82346876	31.59258206	37.24802015	35.03357092	43.24097267
ENSMUSG00000005225	PLEKHA8	43.69827217	52.97416847	21.86222426	11.93566676	19.29347302	6.435834728	18.45945315	18.04495772	22.81686482	19.22478459	33.28189237	18.53184543
ENSMUSG00000005233	SPC25	17.47930887	6.621771059	14.57481618	29.83916689	32.15578836	25.74333891	26.37064736	30.93421324	26.32715172	30.03872593	33.28189237	32.43072951
ENSMUSG00000005237	DNAH2	0	0	7.287408088	0	0	0	0	0	0	1.201549037	0	0
ENSMUSG00000005262	UFD1	78.65688991	33.10885529	72.87408088	41.77483365	45.01810371	32.17917364	68.56368314	55.42379872	80.7365986	67.28674607	75.32217748	54.05121584
ENSMUSG00000005267	ZFP287	0	6.621771059	0	5.967833378	6.431157673	0	0	0	0	0	0	0
ENSMUSG00000005268	PRLR	0	0	0	0	0	6.435834728	0	0	0	0	0	0
ENSMUSG00000005299	LETM1	122.3551621	132.4354212	167.610386	149.1958345	109.3296804	148.0241987	148.9941576	159.8267684	138.6563324	138.1781393	155.8993906	128.1785976
ENSMUSG00000005312	UBQLN1	96.13619877	152.3007344	94.73630514	119.3566676	141.4854688	154.4600335	119.9864455	172.7160239	108.8188938	121.3564527	131.3758909	120.4569953
ENSMUSG00000005320	FGFR4	0	0	0	0	0	0	1.318532368	0	0	1.201549037	0	1.544320453
ENSMUSG00000005338	CADM3	0	6.621771059	14.57481618	5.967833378	6.431157673	0	5.274129472	14.17818107	3.510286896	3.604647111	7.006714184	18.53184543

ENSMUSG00000005354	TXN2	52.4379266	66.21771059	51.01185661	35.80700027	77.17389207	77.23001673	63.28955366	87.64693751	64.94030757	74.4960403	64.8121062	74.12738173
ENSMUSG00000005360	SLC1A3	0	0	0	0	0	0	0	1.288925552	0	0	0	0
ENSMUSG00000005362	CRBN	17.47930887	59.59593953	58.2992647	41.77483365	45.01810371	57.92251255	79.11194208	70.89090534	108.8188938	72.09294222	56.05371347	61.77281811
ENSMUSG00000005364	IL5RA	0	0	0	0	0	0	0	0	0	0	1.751678546	0
ENSMUSG00000005370	MSH6	43.69827217	46.35239741	21.86222426	83.54966729	96.46736509	32.17917364	73.83781261	45.11239431	66.69545102	58.87590281	61.30874911	64.86145901
ENSMUSG00000005371	FBXO11	34.95861774	39.73062635	58.2992647	95.48533405	57.88041905	25.74333891	72.51928024	34.80098989	54.40944688	57.67435378	47.29532074	44.78529313
ENSMUSG00000005373	MLXIPL	0	0	0	0	0	0	1.318532368	0	0	1.201549037	1.751678546	0
ENSMUSG00000005374	TBL2	43.69827217	26.48708424	36.43704044	53.7105004	51.44926138	51.48667782	38.23743867	46.40131986	49.14401654	48.06196148	42.0402851	35.51937041
ENSMUSG00000005378	BUD23	131.0948165	86.08302377	153.0355698	125.3245009	141.4854688	160.8958682	145.0385605	172.7160239	186.0452055	130.968845	147.1409979	177.5968521
ENSMUSG00000005410	MCM5	166.0534342	185.4095896	167.610386	143.2280011	244.3839916	141.588364	147.6756252	177.8717261	215.8826441	174.2246104	168.1611404	186.8627748
ENSMUSG00000005413	HMOX1	8.739654434	0	0	0	6.431157673	12.87166946	1.318532368	3.866776655	5.265430344	6.007745185	1.751678546	6.177281811
ENSMUSG00000005417	MPRIP	26.2189633	66.21771059	65.58667279	77.58183392	83.60504975	45.05084309	50.10422998	56.71272427	52.65430344	48.06196148	43.79196365	47.87393403
ENSMUSG00000005442	CIC	17.47930887	33.10885529	21.86222426	41.77483365	19.29347302	25.74333891	30.32624446	48.97917096	43.8785862	34.84492207	42.0402851	29.3420886
ENSMUSG00000005447	PAFAH1B3	34.95861774	39.73062635	36.43704044	53.7105004	70.7427344	25.74333891	34.28184157	30.93421324	52.65430344	46.86041244	31.53021383	38.60801132
ENSMUSG00000005465	IL27RA	26.2189633	26.48708424	21.86222426	29.83916689	19.29347302	6.435834728	18.45945315	16.75603217	35.10286896	8.410843259	33.28189237	20.07616588
ENSMUSG00000005469	PRKACA	104.8758532	99.32656588	87.44889705	53.7105004	38.58694604	77.23001673	98.8899276	79.9133842	108.8188938	76.89913837	77.07385602	92.65922716
ENSMUSG00000005470	ASF1B	34.95861774	72.83948165	58.2992647	41.77483365	45.01810371	32.17917364	55.37835946	51.55702207	43.8785862	52.86815763	57.80539202	74.12738173
ENSMUSG00000005481	DDX39	139.8344709	125.8136501	109.3111213	83.54966729	128.6231535	122.2808598	143.7200281	132.7593318	115.8394676	121.3564527	171.6644975	134.3558794
ENSMUSG00000005483	DNAJB1	17.47930887	39.73062635	14.57481618	23.87133351	6.431157673	25.74333891	47.46716525	33.51206434	43.8785862	54.06970667	45.5436422	55.5955363
ENSMUSG00000005493	MSH4	8.739654434	0	0	0	6.431157673	0	0	0	1.755143448	0	0	1.544320453
ENSMUSG00000005501	USP40	17.47930887	39.73062635	51.01185661	41.77483365	45.01810371	19.30750418	19.77798552	21.91173438	19.30657793	13.21703941	17.51678546	21.62048634
ENSMUSG00000005505	KBTBD4	17.47930887	19.86531318	29.14963235	17.90350013	19.29347302	12.87166946	23.73358262	25.77851103	21.06172137	22.8294317	17.51678546	18.53184543
ENSMUSG00000005506	CELF1	253.4499786	258.2490713	364.3704044	250.6490019	289.4020953	328.2275711	283.4844591	266.8075892	247.4752262	285.9686708	236.4766037	223.9264656
ENSMUSG00000005510	NDUFS3	17.47930887	19.86531318	0	11.93566676	12.86231535	38.61500837	18.45945315	19.33388327	15.79629103	26.43407881	12.26174982	18.53184543
ENSMUSG00000005514	POR	87.39654434	52.97416847	43.72444853	71.61400054	77.17389207	83.66585146	42.19303578	51.55702207	57.91973378	48.06196148	56.05371347	54.05121584
ENSMUSG00000005533	IGF1R	0	0	0	0	0	0	0	0	0	0	1.751678546	0
ENSMUSG00000005534	INSR	61.17758104	72.83948165	102.0237132	35.80700027	38.58694604	25.74333891	65.9266184	55.42379872	73.71602481	55.2712557	56.05371347	44.78529313
ENSMUSG00000005540	FCER2A	0	0	0	0	0	0	0	0	0	0	1.751678546	0
ENSMUSG00000005566	TRIM28	113.6155076	119.1918791	123.8859375	113.3888342	186.5035725	135.1525293	148.9941576	140.4928851	198.3312096	164.6122181	166.4094619	171.4195702
ENSMUSG00000005575	UBE2M	192.2723975	165.5442765	174.8977941	149.1958345	167.2100995	109.4091904	160.8609489	154.6710662	205.3517834	134.5734921	164.6577833	211.571902
ENSMUSG00000005580	ADCY9	0	0	0	5.967833378	0	0	0	0	0	0	0	0
ENSMUSG00000005583	MEF2C	8.739654434	0	0	0	0	0	0	1.288925552	0	0	1.751678546	0
ENSMUSG00000005609	CTR9	69.91723547	125.8136501	123.8859375	119.3566676	102.8985228	90.10168619	100.20846	92.80263972	98.28803308	112.9456095	106.8523913	100.3808294
ENSMUSG00000005610	EIF4G2	288.4085963	331.0885529	422.6696691	346.1343359	353.713672	366.8425795	522.1388177	556.8158383	508.9915999	543.1001647	532.510278	471.0177381
ENSMUSG00000005611	MRVI1	0	0	0	0	0	6.435834728	2.637064736	3.866776655	1.755143448	3.604647111	5.255035638	1.544320453
ENSMUSG00000005615	PCYT1A	26.2189633	6.621771059	21.86222426	5.967833378	12.86231535	6.435834728	21.09651789	21.91173438	22.81686482	16.82168652	14.01342837	15.44320453
ENSMUSG00000005621	ZFP592	8.739654434	19.86531318	29.14963235	23.87133351	6.431157673	51.48667782	23.73358262	23.20065993	17.55143448	22.8294317	19.26846401	29.3420886
ENSMUSG00000005625	PSMD4	209.7517064	145.6789633	138.4607537	179.0350013	212.2282032	128.7166946	224.1505026	221.6951949	191.3106358	233.1005132	262.7517819	259.445836
ENSMUSG00000005628	TMOD4	0	0	0	0	0	0	2.637064736	1.288925552	0	1.201549037	0	0
ENSMUSG00000005640	INSRR	0	0	0	5.967833378	0	0	0	0	0	0	0	0
ENSMUSG00000005649	CABP5	0	0	0	0	0	0	0	0	3.510286896	0	0	1.544320453
ENSMUSG00000005656	SNX6	61.17758104	46.35239741	51.01185661	29.83916689	83.60504975	38.61500837	68.56368314	59.29057538	52.65430344	54.06970667	78.82553457	72.58306128
ENSMUSG00000005667	MTHFD2	174.7930887	172.1660475	153.0355698	137.2601677	180.0724148	109.4091904	168.7721431	140.4928851	138.6563324	144.1858844	112.1074269	176.0525316
ENSMUSG00000005674	TOMM40L	26.2189633	13.24354212	21.86222426	11.93566676	19.29347302	19.30750418	18.45945315	19.33388327	8.775717239	24.03098074	24.52349964	32.43072951
ENSMUSG00000005677	NR1I3	0	0	0	5.967833378	6.431157673	0	6.59266184	1.288925552	3.510286896	3.604647111	1.751678546	4.632961358

ENSMUSG00000005681	APOA2	0	19.86531318	7.287408088	0	12.86231535	12.87166946	5.274129472	0	1.755143448	6.007745185	5.255035638	9.265922716
ENSMUSG00000005682	PAN2	8.739654434	19.86531318	43.72444853	17.90350013	32.15578836	25.74333891	21.09651789	15.46710662	17.55143448	15.62013748	26.27517819	12.35456362
ENSMUSG00000005683	CS	122.3551621	198.6531318	167.610386	190.9706681	154.3477841	122.2808598	170.0906755	174.0049495	207.1069268	186.2401007	176.9195331	211.571902
ENSMUSG00000005686	AMPD3	0	0	0	0	0	0	1.318532368	0	0	0	0	0
ENSMUSG00000005687	BCAS2	183.5327431	251.6273002	233.1970588	196.9385015	180.0724148	115.8450251	294.0327181	306.7642813	314.1706772	320.8135929	287.2752815	248.6355929
ENSMUSG00000005696	SH2D1A	26.2189633	6.621771059	14.57481618	11.93566676	6.431157673	0	3.955597104	5.155702207	8.775717239	4.806196148	3.503357092	9.265922716
ENSMUSG00000005698	CTCF	201.012052	152.3007344	153.0355698	196.9385015	205.7970455	257.4333891	207.0095818	189.4720561	222.9032179	223.4881209	217.2081397	177.5968521
ENSMUSG00000005699	PARD6A	8.739654434	13.24354212	7.287408088	0	6.431157673	6.435834728	5.274129472	9.022478862	5.265430344	6.007745185	5.255035638	10.81024317
ENSMUSG00000005705	AGRP	0	0	0	0	0	0	0	1.288925552	1.755143448	2.403098074	0	1.544320453
ENSMUSG00000005718	TFAP4	122.3551621	125.8136501	58.2992647	107.4210008	109.3296804	45.05084309	80.43047445	68.31305424	73.71602481	84.10843259	71.81882038	86.48194535
ENSMUSG00000005732	RANBP1	550.5982293	576.0940821	663.154136	560.9763375	688.133871	534.1742824	867.5942981	797.8449165	865.2857198	812.247149	833.7989879	745.9067786
ENSMUSG00000005763	CD247	0	0	0	0	6.431157673	0	0	3.866776655	0	0	0	0
ENSMUSG00000005774	RFX5	0	6.621771059	7.287408088	11.93566676	19.29347302	12.87166946	19.77798552	12.88925552	19.30657793	22.8294317	28.02685674	13.89888407
ENSMUSG00000005779	PSMB4	270.9292875	357.5756372	408.0948529	298.3916689	411.5940911	347.5350753	432.4786167	458.8574964	401.9278496	356.860064	462.4431361	407.7005995
ENSMUSG00000005802	SLC30A4	8.739654434	13.24354212	21.86222426	17.90350013	6.431157673	25.74333891	13.18532368	7.73355331	10.53086069	9.612392296	8.75839273	16.98752498
ENSMUSG00000005803	SQOR	0	0	0	0	6.431157673	0	3.955597104	1.288925552	5.265430344	4.806196148	3.503357092	3.088640905
ENSMUSG00000005804	BLOC1S6	34.95861774	39.73062635	29.14963235	29.83916689	83.60504975	19.30750418	58.01542419	59.29057538	33.34772551	46.86041244	61.30874911	67.95009992
ENSMUSG00000005813	METAP1	43.69827217	26.48708424	94.73630514	35.80700027	96.46736509	77.23001673	42.19303578	63.15735203	29.83743861	56.47280474	61.30874911	63.31713856
ENSMUSG00000005823	GPR108	43.69827217	39.73062635	14.57481618	47.74266702	32.15578836	6.435834728	21.09651789	32.22313879	24.57200827	16.82168652	31.53021383	18.53184543
ENSMUSG00000005824	TNFSF14	0	13.24354212	7.287408088	0	6.431157673	12.87166946	0	1.288925552	0	1.201549037	1.751678546	1.544320453
ENSMUSG00000005846	RSL1D1	262.189633	350.9538661	306.0711397	268.552502	295.8332529	205.9467113	416.6562283	413.7451021	400.1727061	433.7592024	341.5773165	321.2186542
ENSMUSG00000005871	APC	61.17758104	46.35239741	51.01185661	59.67833378	19.29347302	51.48667782	54.05982709	33.51206434	49.14401654	40.85266726	45.5436422	35.51937041
ENSMUSG00000005873	REEP5	131.0948165	125.8136501	102.0237132	83.54966729	51.44926138	109.4091904	106.8011218	101.8251186	93.02260274	87.7130797	96.34232003	106.5581112
ENSMUSG00000005881	ERGIC3	87.39654434	33.10885529	58.2992647	59.67833378	45.01810371	51.48667782	43.51156814	58.00164982	63.18516412	73.29449126	68.31546329	35.51937041
ENSMUSG00000005882	UQCC1	52.4379266	26.48708424	51.01185661	11.93566676	25.72463069	51.48667782	48.78569762	27.06743658	45.63372964	28.83717689	31.53021383	43.24097267
ENSMUSG00000005883	SPO11	0	33.10885529	14.57481618	17.90350013	0	0	13.18532368	18.04495772	19.30657793	19.22478459	17.51678546	18.53184543
ENSMUSG00000005886	NCOA2	61.17758104	13.24354212	80.16148896	41.77483365	38.58694604	38.61500837	36.9189063	48.97917096	52.65430344	39.65111822	24.52349964	30.88640905
ENSMUSG00000005893	NR2C2	26.2189633	99.32656588	72.87408088	29.83916689	32.15578836	57.92251255	48.78569762	56.71272427	50.89915999	52.86815763	59.55707056	43.24097267
ENSMUSG00000005897	NR2C1	17.47930887	0	0	17.90350013	6.431157673	6.435834728	6.59266184	19.33388327	21.06172137	13.21703941	15.76510691	9.265922716
ENSMUSG00000005899	SMPD4	52.4379266	52.97416847	102.0237132	23.87133351	45.01810371	57.92251255	47.46716525	65.73520313	66.69545102	52.86815763	49.04699929	58.6841772
ENSMUSG00000005907	PEX1	17.47930887	19.86531318	7.287408088	23.87133351	64.31157673	19.30750418	25.05211499	28.35636214	26.32715172	21.62788267	15.76510691	18.53184543
ENSMUSG00000005917	OTX1	0	0	0	0	0	0	1.318532368	0	0	0	0	0
ENSMUSG00000005936	KCTD20	26.2189633	39.73062635	21.86222426	35.80700027	45.01810371	45.05084309	42.19303578	32.22313879	40.3682993	26.43407881	49.04699929	38.60801132
ENSMUSG00000005947	ITGAE	8.739654434	6.621771059	29.14963235	0	12.86231535	0	1.318532368	3.866776655	0	1.201549037	0	0
ENSMUSG00000005949	CTNS	17.47930887	6.621771059	7.287408088	5.967833378	6.431157673	12.87166946	10.54825894	10.31140441	7.020573791	8.410843259	5.255035638	4.632961358
ENSMUSG00000005950	P2RX5	0	0	0	0	0	0	0	1.288925552	0	0	0	0
ENSMUSG00000005951	SHPK	0	0	0	5.967833378	0	0	1.318532368	0	5.265430344	1.201549037	5.255035638	0
ENSMUSG00000005952	TRPV1	0	0	0	0	0	0	0	0	0	1.201549037	0	1.544320453
ENSMUSG00000005968	TUFT1	0	6.621771059	29.14963235	23.87133351	6.431157673	6.435834728	3.955597104	10.31140441	5.265430344	8.410843259	1.751678546	4.632961358
ENSMUSG00000005973	RCN1	52.4379266	79.46125271	7.287408088	11.93566676	51.44926138	32.17917364	46.14863288	37.378841	52.65430344	46.86041244	38.53692801	46.32961358
ENSMUSG00000005980	DNASE1	0	6.621771059	0	0	0	0	10.54825894	7.73355331	8.775717239	4.806196148	3.503357092	4.632961358
ENSMUSG00000005981	TRAP1	131.0948165	178.7878186	116.5985294	137.2601677	147.9166265	154.4600335	196.4613228	181.7385028	177.2694882	197.0540421	189.181283	185.3184543
ENSMUSG00000005982	NAA60	17.47930887	33.10885529	36.43704044	5.967833378	12.86231535	25.74333891	35.60037394	27.06743658	33.34772551	25.23252978	24.52349964	24.70912724
ENSMUSG00000005983	1700037C18F	8.739654434	6.621771059	0	0	12.86231535	12.87166946	7.911194208	7.73355331	14.04114758	8.410843259	10.51007128	3.088640905
ENSMUSG00000005986	ANKRD13D	17.47930887	0	0	0	0	0	0	0	0	1.201549037	1.751678546	0

ENSMUSG00000006005	TPR	655.4740826	668.7988769	626.7170955	650.4938382	585.2353482	624.2759686	578.8357095	598.061456	552.8701861	580.3481849	595.5707056	628.5384242
ENSMUSG00000006010	BC003331	52.4379266	59.59593953	102.0237132	53.7105004	83.60504975	90.10168619	50.10422998	56.71272427	71.96088136	45.65886341	57.80539202	49.41825449
ENSMUSG00000006014	PRG4	26.2189633	26.48708424	43.72444853	41.77483365	12.86231535	19.30750418	21.09651789	19.33388327	22.81686482	36.04647111	31.53021383	35.51937041
ENSMUSG00000006019	DHX34	0	13.24354212	14.57481618	11.93566676	12.86231535	12.87166946	5.274129472	7.73355331	5.265430344	7.209294222	3.503357092	7.721602263
ENSMUSG00000006021	KPTN	17.47930887	6.621771059	7.287408088	17.90350013	19.29347302	6.435834728	25.05211499	15.46710662	24.57200827	13.21703941	12.26174982	7.721602263
ENSMUSG00000006024	NAPA	34.95861774	13.24354212	43.72444853	53.7105004	64.31157673	51.48667782	43.51156814	59.29057538	61.43002068	36.04647111	63.06042765	71.03874082
ENSMUSG00000006050	SRA1	52.4379266	86.08302377	43.72444853	53.7105004	51.44926138	19.30750418	50.10422998	54.13487317	54.40944688	50.46505955	40.28860656	44.78529313
ENSMUSG00000006057	ATP5G1	43.69827217	72.83948165	116.5985294	65.64616716	102.8985228	135.1525293	126.5791073	117.2922252	133.390902	134.5734921	110.3557484	151.3434044
ENSMUSG00000006058	SNF8	131.0948165	52.97416847	51.01185661	47.74266702	45.01810371	25.74333891	42.19303578	60.57950093	68.45059447	40.85266726	54.30203492	74.12738173
ENSMUSG00000006095	TBCB	61.17758104	59.59593953	65.58667279	71.61400054	64.31157673	25.74333891	54.05982709	59.29057538	71.96088136	76.89913837	64.8121062	60.22849765
ENSMUSG00000006127	INPP5K	17.47930887	19.86531318	7.287408088	11.93566676	12.86231535	0	14.50385605	14.17818107	10.53086069	9.612392296	17.51678546	16.98752498
ENSMUSG00000006134	CRKL	52.4379266	72.83948165	87.44889705	83.54966729	32.15578836	38.61500837	64.60808603	52.84594762	50.89915999	39.65111822	47.29532074	38.60801132
ENSMUSG00000006154	EPS8L1	0	0	0	5.967833378	0	12.87166946	7.911194208	7.73355331	10.53086069	12.01549037	3.503357092	10.81024317
ENSMUSG00000006169	CLINT1	139.8344709	92.70479482	116.5985294	77.58183392	173.6412572	83.66585146	178.0018697	192.0499072	172.0040579	158.6044729	199.6913542	188.4070952
ENSMUSG00000006179	PRSS16	0	6.621771059	21.86222426	0	0	6.435834728	9.229726576	5.155702207	3.510286896	4.806196148	3.503357092	7.721602263
ENSMUSG00000006191	CDKAL1	69.91723547	79.46125271	116.5985294	83.54966729	77.17389207	45.05084309	118.6679131	86.35801196	94.77774619	80.50378548	85.83224875	88.0262658
ENSMUSG00000006215	ZBTB17	17.47930887	19.86531318	36.43704044	11.93566676	38.58694604	45.05084309	25.05211499	21.91173438	29.83743861	20.42633363	28.02685674	30.88640905
ENSMUSG00000006262	MOB1B	17.47930887	33.10885529	14.57481618	29.83916689	32.15578836	19.30750418	23.73358262	25.77851103	47.38887309	36.04647111	36.78524947	35.51937041
ENSMUSG00000006273	ATP6V1B2	96.13619877	86.08302377	94.73630514	119.3566676	102.8985228	83.66585146	100.20846	113.4254485	78.98145515	91.31772681	84.08057021	95.74786807
ENSMUSG00000006276	EPS15L1	26.2189633	39.73062635	29.14963235	41.77483365	25.72463069	19.30750418	40.87450341	39.9566921	52.65430344	43.25576533	50.79867783	44.78529313
ENSMUSG00000006281	TEP1	8.739654434	19.86531318	14.57481618	5.967833378	19.29347302	12.87166946	11.86679131	15.46710662	12.28600414	16.82168652	8.75839273	7.721602263
ENSMUSG00000006288	TTC5	26.2189633	105.9483369	65.58667279	47.74266702	122.1919958	64.35834728	50.10422998	54.13487317	50.89915999	40.85266726	49.04699929	71.03874082
ENSMUSG00000006289	OSGEP	8.739654434	13.24354212	29.14963235	17.90350013	32.15578836	12.87166946	36.9189063	29.64528769	38.61315585	28.83717689	28.02685674	23.16480679
ENSMUSG00000006299	AAMP	157.3137798	211.8966739	160.3229779	179.0350013	154.3477841	122.2808598	222.8319702	199.7834605	210.6172137	207.8679834	236.4766037	211.571902
ENSMUSG00000006301	TMBIM1	17.47930887	13.24354212	14.57481618	35.80700027	45.01810371	19.30750418	35.60037394	24.48958548	21.06172137	25.23252978	42.0402851	40.15233177
ENSMUSG00000006304	ARPC2	157.3137798	284.7361555	225.9096507	399.8448363	257.2463069	218.8183807	255.7952794	244.8958548	261.5163737	258.333043	250.4900321	253.2685542
ENSMUSG00000006307	KMT2B	43.69827217	39.73062635	29.14963235	29.83916689	12.86231535	51.48667782	25.05211499	36.08991545	29.83743861	38.44956918	42.0402851	44.78529313
ENSMUSG00000006310	ZBTB32	0	0	0	0	0	0	0	1.288925552	0	0	0	3.088640905
ENSMUSG00000006313	UPK1A	0	0	0	0	6.431157673	0	0	2.577851103	1.755143448	0	1.751678546	1.544320453
ENSMUSG00000006315	TMEM147	17.47930887	72.83948165	51.01185661	29.83916689	57.88041905	45.05084309	67.24515077	59.29057538	57.91973378	44.45731437	54.30203492	75.67170218
ENSMUSG00000006333	RPS9	970.1016422	887.3173219	976.5126837	1068.242175	1022.55407	1074.7844	1290.843188	1315.992988	1511.178509	1166.704115	1434.624729	1391.432728
ENSMUSG00000006335	TFPT	8.739654434	26.48708424	21.86222426	17.90350013	19.29347302	12.87166946	25.05211499	25.77851103	17.55143448	12.01549037	24.52349964	35.51937041
ENSMUSG00000006342	SUSD2	0	0	0	0	6.431157673	0	0	0	0	0	0	0
ENSMUSG00000006344	GGT5	0	0	0	0	0	0	1.318532368	0	0	0	0	0
ENSMUSG00000006345	GGT1	0	0	0	5.967833378	6.431157673	0	1.318532368	1.288925552	0	0	0	3.088640905
ENSMUSG00000006356	CRIP2	0	0	0	0	6.431157673	0	1.318532368	1.288925552	1.755143448	0	0	0
ENSMUSG00000006360	CRIP1	305.8879052	291.3579266	313.3585478	328.2308358	360.1448297	218.8183807	396.8782428	407.3004743	473.8887309	349.6507698	434.4162794	469.4734176
ENSMUSG00000006362	CBFA2T3	69.91723547	66.21771059	51.01185661	107.4210008	64.31157673	77.23001673	76.47487734	48.97917096	54.40944688	67.28674607	59.55707056	71.03874082
ENSMUSG00000006369	FBLN1	0	0	0	0	0	0	0	0	0	0	1.751678546	0
ENSMUSG00000006373	PGRMC1	61.17758104	52.97416847	80.16148896	83.54966729	38.58694604	51.48667782	97.57139523	109.5586719	143.9217627	98.52702104	110.3557484	81.84898399
ENSMUSG00000006378	GCAT	43.69827217	33.10885529	21.86222426	23.87133351	51.44926138	19.30750418	42.19303578	58.00164982	50.89915999	56.47280474	68.31546329	54.05121584
ENSMUSG00000006386	TEK	0	0	0	0	0	0	1.318532368	0	0	0	0	0
ENSMUSG00000006389	MPL	0	0	0	5.967833378	0	0	1.318532368	0	0	0	0	1.544320453
ENSMUSG00000006390	ELOVL1	52.4379266	52.97416847	80.16148896	59.67833378	83.60504975	57.92251255	71.20074787	76.04660755	82.49174205	79.30223644	70.06714184	98.83650897
ENSMUSG00000006392	MED8	61.17758104	72.83948165	72.87408088	59.67833378	77.17389207	77.23001673	100.20846	103.1140441	108.8188938	103.3332172	101.5973557	91.11490671

ENSMUSG00000006395	HYI	17.47930887	33.10885529	21.86222426	11.93566676	32.15578836	45.05084309	31.64477683	33.51206434	42.12344275	25.23252978	28.02685674	32.43072951
ENSMUSG00000006398	CDC20	218.4913609	185.4095896	262.3466912	286.4560021	289.4020953	250.9975544	251.8396823	232.0065993	275.5575213	194.650944	239.9799608	271.8003997
ENSMUSG00000006411	NECTIN4	0	0	0	0	0	0	0	1.288925552	1.755143448	4.806196148	0	0
ENSMUSG00000006412	PFDN2	568.0775382	576.0940821	765.1778492	590.8155044	649.5469249	695.0701506	456.2121993	522.0148484	565.1561902	434.9607514	483.4632787	521.980313
ENSMUSG00000006418	RNF114	34.95861774	39.73062635	58.2992647	77.58183392	32.15578836	70.79418201	52.74129472	52.84594762	50.89915999	49.26351052	54.30203492	44.78529313
ENSMUSG00000006423	C330007P06F	8.739654434	33.10885529	21.86222426	23.87133351	12.86231535	6.435834728	17.14092078	10.31140441	7.020573791	8.410843259	10.51007128	12.35456362
ENSMUSG00000006442	SRM	113.6155076	152.3007344	102.0237132	95.48533405	135.0543111	90.10168619	137.1273663	146.9375129	140.4114758	130.968845	159.4027477	152.8877248
ENSMUSG00000006445	EPHA2	8.739654434	0	7.287408088	0	0	12.87166946	1.318532368	0	0	0	0	0
ENSMUSG00000006456	RBM14	0	13.24354212	29.14963235	35.80700027	25.72463069	19.30750418	34.28184157	43.82346876	49.14401654	50.46505955	29.77853528	46.32961358
ENSMUSG00000006457	ACTN3	0	0	0	5.967833378	0	0	1.318532368	0	0	0	1.751678546	0
ENSMUSG00000006463	ZDHHC24	17.47930887	6.621771059	7.287408088	0	32.15578836	6.435834728	6.59266184	6.444627758	12.28600414	6.007745185	8.75839273	6.177281811
ENSMUSG00000006464	BBS1	0	0	0	5.967833378	6.431157673	0	2.637064736	1.288925552	3.510286896	1.201549037	0	4.632961358
ENSMUSG00000006471	NDOR1	26.2189633	6.621771059	7.287408088	17.90350013	12.86231535	12.87166946	7.911194208	6.444627758	12.28600414	12.01549037	24.52349964	12.35456362
ENSMUSG00000006476	NSMF	34.95861774	46.35239741	29.14963235	35.80700027	57.88041905	51.48667782	34.28184157	45.11239431	43.8785862	37.24802015	19.26846401	55.5955363
ENSMUSG00000006488	PRL7A1	0	0	0	0	0	0	0	1.288925552	0	1.201549037	0	0
ENSMUSG00000006494	PDK1	244.7103242	211.8966739	153.0355698	137.2601677	225.0905185	154.4600335	250.5211499	262.9408125	221.1480744	209.0695324	259.2484248	206.9389407
ENSMUSG00000006498	PTBP1	157.3137798	105.9483369	153.0355698	95.48533405	122.1919958	96.53752092	154.2682871	183.0274283	170.2489144	161.007571	196.1879971	180.685493
ENSMUSG00000006517	MVD	34.95861774	33.10885529	36.43704044	35.80700027	70.7427344	32.17917364	56.69689182	54.13487317	47.38887309	58.87590281	43.79196365	55.5955363
ENSMUSG00000006519	CYBA	122.3551621	172.1660475	233.1970588	179.0350013	109.3296804	83.66585146	261.0694089	229.4287482	210.6172137	194.650944	255.7450677	245.546952
ENSMUSG00000006526	TMEM110	8.739654434	13.24354212	0	5.967833378	6.431157673	19.30750418	10.54825894	6.444627758	12.28600414	15.62013748	24.52349964	15.44320453
ENSMUSG00000006527	SFMBT1	61.17758104	59.59593953	72.87408088	65.64616716	64.31157673	51.48667782	50.10422998	46.40131986	35.10286896	44.45731437	45.5436422	44.78529313
ENSMUSG00000006567	ATP7B	0	0	0	0	0	0	0	0	0	0	0	3.088640905
ENSMUSG00000006575	RUNDC3A	8.739654434	6.621771059	21.86222426	5.967833378	19.29347302	0	2.637064736	2.577851103	8.775717239	7.209294222	8.75839273	10.81024317
ENSMUSG00000006585	CDT1	157.3137798	145.6789633	102.0237132	125.3245009	147.9166265	109.4091904	181.9574668	167.5603217	172.0040579	153.7982767	166.4094619	177.5968521
ENSMUSG00000006589	APRT	104.8758532	99.32656588	65.58667279	89.51750067	64.31157673	64.35834728	126.5791073	103.1140441	117.594611	102.1316681	108.6040698	132.8115589
ENSMUSG00000006599	GTF2H1	104.8758532	72.83948165	102.0237132	119.3566676	160.7789418	102.9733556	129.2161721	131.4704063	142.1666193	132.1703941	134.879248	143.6218021
ENSMUSG00000006638	ABHD1	0	13.24354212	7.287408088	5.967833378	12.86231535	19.30750418	5.274129472	6.444627758	14.04114758	8.410843259	12.26174982	6.177281811
ENSMUSG00000006641	SLC5A6	8.739654434	6.621771059	21.86222426	0	0	0	9.229726576	3.866776655	8.775717239	7.209294222	5.255035638	3.088640905
ENSMUSG00000006649	NPHS1	0	0	7.287408088	0	0	0	0	1.288925552	1.755143448	1.201549037	0	0
ENSMUSG00000006673	QRICH1	384.5447951	297.9796976	371.6578125	190.9706681	295.8332529	148.0241987	213.6022436	250.051557	247.4752262	240.3098074	243.4833179	227.0151065
ENSMUSG00000006675	P4HTM	0	6.621771059	0	0	6.431157673	6.435834728	6.59266184	6.444627758	8.775717239	2.403098074	5.255035638	3.088640905
ENSMUSG00000006676	USP19	52.4379266	33.10885529	51.01185661	35.80700027	38.58694604	45.05084309	51.42276235	52.84594762	49.14401654	58.87590281	43.79196365	61.77281811
ENSMUSG00000006678	POLA1	61.17758104	72.83948165	43.72444853	83.54966729	70.7427344	90.10168619	68.56368314	68.31305424	75.47116826	79.30223644	70.06714184	71.03874082
ENSMUSG00000006699	CDC42	235.9706697	251.6273002	211.3348345	381.9413362	244.3839916	180.2033724	295.3512504	292.5861002	326.4566813	257.1314939	276.7652103	281.0663224
ENSMUSG00000006705	PKNOX1	26.2189633	39.73062635	21.86222426	35.80700027	45.01810371	45.05084309	25.05211499	16.75603217	19.30657793	34.84492207	14.01342837	23.16480679
ENSMUSG00000006715	GMNN	218.4913609	172.1660475	204.0474265	268.552502	244.3839916	231.6900502	295.3512504	324.809239	314.1706772	326.8213381	359.0941019	290.3322451
ENSMUSG00000006717	ACOT13	87.39654434	46.35239741	72.87408088	53.7105004	90.03620742	70.79418201	85.70460392	110.8475974	82.49174205	121.3564527	106.8523913	105.0137908
ENSMUSG00000006720	ZFP184	0	0	7.287408088	0	0	0	1.318532368	2.577851103	3.510286896	4.806196148	0	1.544320453
ENSMUSG00000006724	CYP27B1	0	0	0	0	0	0	0	0	1.755143448	0	0	0
ENSMUSG00000006728	CDK4	218.4913609	185.4095896	233.1970588	262.5846686	186.5035725	167.3317029	272.9362002	332.5427923	356.2941199	228.294317	296.0336743	296.5095269
ENSMUSG00000006731	B4GALNT1	34.95861774	6.621771059	0	17.90350013	12.86231535	0	22.41505026	25.77851103	28.08229517	18.02323556	12.26174982	20.07616588
ENSMUSG00000006732	METTL1	87.39654434	46.35239741	58.2992647	41.77483365	90.03620742	45.05084309	47.46716525	72.17983089	61.43002068	44.45731437	40.28860656	64.86145901
ENSMUSG00000006736	TSPAN31	34.95861774	26.48708424	43.72444853	29.83916689	57.88041905	32.17917364	48.78569762	61.86842648	47.38887309	45.65886341	57.80539202	57.13985675
ENSMUSG00000006740	KIF5B	297.1482508	311.2232398	306.0711397	364.0378361	398.7317757	334.6634058	287.4400562	246.1847804	256.2509434	291.976416	275.0135317	274.8890406
ENSMUSG00000006763	SAAL1	8.739654434	19.86531318	21.86222426	23.87133351	19.29347302	25.74333891	25.05211499	19.33388327	19.30657793	22.8294317	28.02685674	30.88640905

ENSMUSG00000006782	CNP	8.739654434	19.86531318	14.57481618	23.87133351	12.86231535	0	6.59266184	10.31140441	10.53086069	7.209294222	22.7718211	15.44320453
ENSMUSG00000006784	TTC25	0	0	0	5.967833378	0	0	0	0	0	2.403098074	0	0
ENSMUSG00000006800	SULF2	0	19.86531318	0	0	0	6.435834728	3.955597104	3.866776655	1.755143448	8.410843259	1.751678546	3.088640905
ENSMUSG00000006818	SOD2	131.0948165	132.4354212	131.1733456	137.2601677	83.60504975	64.35834728	168.7721431	175.293875	196.5760662	158.6044729	203.1947113	176.0525316
ENSMUSG00000006850	TMCO6	0	0	14.57481618	5.967833378	19.29347302	6.435834728	7.911194208	7.73355331	12.28600414	6.007745185	5.255035638	4.632961358
ENSMUSG00000006906	STAMBP	17.47930887	6.621771059	14.57481618	35.80700027	25.72463069	12.87166946	25.05211499	14.17818107	21.06172137	20.42633363	33.28189237	12.35456362
ENSMUSG00000006920	EZH1	8.739654434	6.621771059	36.43704044	23.87133351	6.431157673	19.30750418	30.32624446	28.35636214	29.83743861	21.62788267	38.53692801	33.97504996
ENSMUSG00000006932	CTNNB1	104.8758532	112.570108	123.8859375	113.3888342	90.03620742	122.2808598	221.5134378	192.0499072	179.0246317	197.0540421	190.9329615	157.5206862
ENSMUSG00000006941	EIF1B	61.17758104	59.59593953	58.2992647	89.51750067	122.1919958	77.23001673	79.11194208	77.3355331	56.16459033	66.08519704	70.06714184	84.9376249
ENSMUSG00000006998	PSMD2	69.91723547	86.08302377	116.5985294	143.2280011	122.1919958	102.9733556	114.712316	114.7143741	122.8600414	102.1316681	122.6174982	109.6467521
ENSMUSG00000007029	VARS	637.9947737	615.8247085	604.8548713	560.9763375	655.9780826	489.1234393	464.1233935	541.3487317	540.5841819	493.8366542	532.510278	497.2711858
ENSMUSG00000007030	VWA7	0	0	0	0	6.431157673	0	0	0	1.755143448	3.604647111	0	0
ENSMUSG00000007033	HSPA1L	0	6.621771059	0	5.967833378	0	0	0	1.288925552	0	1.201549037	1.751678546	1.544320453
ENSMUSG00000007035	MSH5	0	0	7.287408088	0	6.431157673	0	6.59266184	7.73355331	1.755143448	3.604647111	3.503357092	1.544320453
ENSMUSG00000007036	ABHD16A	52.4379266	33.10885529	21.86222426	41.77483365	38.58694604	19.30750418	29.0077121	42.5345432	35.10286896	34.84492207	47.29532074	37.06369086
ENSMUSG00000007038	NEU1	17.47930887	79.46125271	58.2992647	23.87133351	57.88041905	32.17917364	21.09651789	33.51206434	43.8785862	40.85266726	42.0402851	54.05121584
ENSMUSG00000007039	DDAH2	8.739654434	6.621771059	29.14963235	35.80700027	6.431157673	0	11.86679131	6.444627758	24.57200827	8.410843259	14.01342837	12.35456362
ENSMUSG00000007041	CLIC1	253.4499786	331.0885529	393.5200367	405.8126697	392.300618	257.4333891	286.1215239	327.3870901	287.8435255	245.1160036	266.255139	335.1175382
ENSMUSG00000007050	LSM2	227.2310153	297.9796976	225.9096507	334.1986692	212.2282032	263.8692238	357.3222717	295.1639513	317.6809641	377.2863976	339.8256379	356.7380246
ENSMUSG00000007080	POLE	52.4379266	72.83948165	87.44889705	47.74266702	32.15578836	45.05084309	59.33395656	67.02412869	59.67487723	61.27900089	52.55035638	60.22849765
ENSMUSG00000007122	CASQ1	0	0	0	0	0	0	0	0	0	0	1.751678546	0
ENSMUSG00000007207	STX1A	43.69827217	59.59593953	109.3111213	89.51750067	70.7427344	51.48667782	51.42276235	39.9566921	50.89915999	30.03872593	40.28860656	58.6841772
ENSMUSG00000007216	ZFP775	0	0	0	0	0	0	1.318532368	2.577851103	1.755143448	2.403098074	0	3.088640905
ENSMUSG00000007338	MRPL49	52.4379266	46.35239741	65.58667279	35.80700027	64.31157673	51.48667782	72.51928024	55.42379872	49.14401654	51.66660859	54.30203492	58.6841772
ENSMUSG00000007379	DENND2C	0	0	0	0	0	0	0	1.288925552	0	0	0	0
ENSMUSG00000007411	MARK3	157.3137798	99.32656588	160.3229779	113.3888342	109.3296804	70.79418201	94.9343305	99.24726748	108.8188938	109.3409624	113.8591055	112.735393
ENSMUSG00000007415	GATAD1	183.5327431	158.9225054	204.0474265	143.2280011	141.4854688	141.588364	134.4903015	141.7818107	147.4320496	132.1703941	133.1275695	109.6467521
ENSMUSG00000007458	M6PR	166.0534342	92.70479482	167.610386	101.4531674	237.9528339	128.7166946	102.8455247	136.6261085	128.1254717	117.7518056	115.610784	103.4694703
ENSMUSG00000007476	LRRC8A	61.17758104	59.59593953	80.16148896	65.64616716	45.01810371	90.10168619	35.60037394	29.64528769	26.32715172	51.66660859	33.28189237	46.32961358
ENSMUSG00000007480	MC5R	8.739654434	13.24354212	7.287408088	5.967833378	0	6.435834728	2.637064736	1.288925552	0	1.201549037	1.751678546	1.544320453
ENSMUSG00000007564	PPP2R1A	183.5327431	145.6789633	160.3229779	155.1636678	212.2282032	135.1525293	189.868661	212.672716	231.6789351	183.8370027	222.4631753	228.559427
ENSMUSG00000007570	FANCE	17.47930887	6.621771059	0	11.93566676	25.72463069	12.87166946	18.45945315	18.04495772	17.55143448	14.41858844	19.26846401	13.89888407
ENSMUSG00000007589	TINF2	26.2189633	13.24354212	0	23.87133351	25.72463069	38.61500837	22.41505026	20.62280883	26.32715172	20.42633363	26.27517819	32.43072951
ENSMUSG00000007594	HAPLN4	8.739654434	0	0	0	0	0	0	0	0	0	0	0
ENSMUSG00000007603	DUS3L	69.91723547	72.83948165	80.16148896	59.67833378	32.15578836	51.48667782	35.60037394	69.60197979	64.94030757	44.45731437	64.8121062	66.40577946
ENSMUSG00000007610	GTPBP3	17.47930887	0	21.86222426	29.83916689	12.86231535	25.74333891	21.09651789	27.06743658	28.08229517	15.62013748	19.26846401	27.79776815
ENSMUSG00000007613	TGFBR1	69.91723547	39.73062635	58.2992647	65.64616716	12.86231535	38.61500837	38.23743867	32.22313879	21.06172137	43.25576533	21.02014255	33.97504996
ENSMUSG00000007617	HOMER1	43.69827217	39.73062635	43.72444853	29.83916689	38.58694604	12.87166946	36.9189063	39.9566921	40.3682993	27.63562785	26.27517819	27.79776815
ENSMUSG00000007646	RAD51C	0	6.621771059	7.287408088	0	6.431157673	0	2.637064736	3.866776655	5.265430344	8.410843259	7.006714184	7.721602263
ENSMUSG00000007656	ARPP19	314.6275596	463.5239741	393.5200367	411.7805031	360.1448297	353.97091	553.7835946	538.7708806	577.4421944	526.2784782	597.3223842	593.0190538
ENSMUSG00000007659	BCL2L1	270.9292875	271.4926134	211.3348345	292.4238355	282.9709376	296.0483975	258.4323441	286.1414725	329.9669682	264.3407881	287.2752815	307.3197701
ENSMUSG00000007670	KHSRP	2307.268771	2238.158618	2164.360202	2094.709516	2148.006663	2065.902948	1654.758122	1706.53743	1648.079698	1536.781218	1627.309369	1816.120852
ENSMUSG00000007682	DIO2	0	0	0	0	0	0	0	0	0	0	0	1.544320453
ENSMUSG00000007721	CCDC124	52.4379266	19.86531318	21.86222426	23.87133351	51.44926138	64.35834728	55.37835946	64.44627758	70.20573791	56.47280474	56.05371347	91.11490671
ENSMUSG00000007739	CCT4	96.13619877	112.570108	138.4607537	137.2601677	115.7608381	128.7166946	179.320402	207.5170138	207.1069268	175.4261594	192.6846401	210.0275816

ENSMUSG00000007777	0610009B22F	0	0	7.287408088	5.967833378	0	6.435834728	7.911194208	6.444627758	5.265430344	7.209294222	7.006714184	10.81024317
ENSMUSG00000007812	ZFP655	17.47930887	26.48708424	0	29.83916689	38.58694604	38.61500837	29.0077121	21.91173438	28.08229517	22.8294317	21.02014255	21.62048634
ENSMUSG00000007815	RHOA	454.4620306	450.280432	539.2681985	429.6840032	424.4564064	450.5084309	448.3010051	418.9008043	398.4175627	386.8987899	404.6377441	430.8654063
ENSMUSG00000007817	ZMIZ1	26.2189633	79.46125271	58.2992647	71.61400054	77.17389207	77.23001673	93.61579813	118.5811508	100.0431765	124.9610998	99.84567712	122.0013158
ENSMUSG00000007827	ANKRD26	375.8051407	417.1715767	371.6578125	399.8448363	302.2644106	341.0992406	359.9593365	297.7418024	303.6398165	326.8213381	324.060531	313.4970519
ENSMUSG00000007833	ALDH16A1	8.739654434	13.24354212	65.58667279	29.83916689	19.29347302	19.30750418	47.46716525	27.06743658	28.08229517	34.84492207	49.04699929	47.87393403
ENSMUSG00000007836	HNRNPA0	804.0482079	787.990756	1012.949724	728.0756721	971.1048086	907.4526966	941.4321107	915.1371417	903.8988757	907.1695229	954.6648076	897.250183
ENSMUSG00000007837	PRRG2	0	0	7.287408088	0	6.431157673	6.435834728	1.318532368	10.31140441	8.775717239	4.806196148	1.751678546	7.721602263
ENSMUSG00000007850	HNRNPH1	192.2723975	178.7878186	131.1733456	226.7776684	295.8332529	238.1258849	325.6774949	319.6535368	280.8229517	365.2709073	301.2887099	305.7754496
ENSMUSG00000007867	IFT43	8.739654434	0	0	0	6.431157673	0	2.637064736	0	0	0	0	0
ENSMUSG00000007872	ID3	8.739654434	0	7.287408088	0	6.431157673	6.435834728	1.318532368	7.73355331	7.020573791	1.201549037	3.503357092	3.088640905
ENSMUSG00000007880	ARID1A	297.1482508	317.8450108	218.6222426	220.809835	302.2644106	283.176728	142.4014957	154.6710662	191.3106358	162.20912	168.1611404	213.1162225
ENSMUSG00000007891	CTSD	43.69827217	66.21771059	43.72444853	59.67833378	64.31157673	32.17917364	52.74129472	48.97917096	61.43002068	27.63562785	52.55035638	60.22849765
ENSMUSG00000007892	RPLP1	1092.456804	1145.566393	1268.009007	1462.119178	1299.09385	1415.88364	1495.215705	1501.598268	1472.565353	1243.603253	1728.906725	1467.10443
ENSMUSG00000007944	TTC9B	0	6.621771059	0	0	0	0	1.318532368	0	0	2.403098074	1.751678546	1.544320453
ENSMUSG00000007946	PHOX2A	8.739654434	0	51.01185661	0	6.431157673	6.435834728	2.637064736	7.73355331	1.755143448	1.201549037	0	4.632961358
ENSMUSG00000007950	ABHD8	87.39654434	66.21771059	94.73630514	89.51750067	109.3296804	102.9733556	71.20074787	77.3355331	80.7365986	81.70533452	85.83224875	101.9251499
ENSMUSG00000008036	AP2S1	87.39654434	66.21771059	109.3111213	53.7105004	77.17389207	57.92251255	84.38607155	90.22478862	105.3086069	85.30998163	101.5973557	100.3808294
ENSMUSG00000008090	FGFRL1	0	0	0	0	0	6.435834728	0	5.155702207	0	3.604647111	3.503357092	1.544320453
ENSMUSG00000008129	4930432K21F	0	0	14.57481618	11.93566676	0	6.435834728	2.637064736	6.444627758	7.020573791	1.201549037	1.751678546	3.088640905
ENSMUSG00000008136	FHL2	0	6.621771059	7.287408088	11.93566676	6.431157673	12.87166946	25.05211499	15.46710662	14.04114758	14.41858844	19.26846401	13.89888407
ENSMUSG00000008140	EMC10	43.69827217	19.86531318	36.43704044	11.93566676	25.72463069	12.87166946	22.41505026	25.77851103	17.55143448	21.62788267	22.7718211	20.07616588
ENSMUSG00000008153	CLSTN3	0	0	0	5.967833378	0	0	0	0	0	0	0	0
ENSMUSG00000008167	FBXW9	8.739654434	0	14.57481618	5.967833378	19.29347302	0	5.274129472	9.022478862	3.510286896	3.604647111	5.255035638	1.544320453
ENSMUSG00000008193	SPIB	0	0	0	0	0	0	0	1.288925552	1.755143448	0	1.751678546	0
ENSMUSG00000008200	FNBP4	201.012052	231.7619871	189.4726103	214.8420016	212.2282032	193.0750418	222.8319702	229.4287482	222.9032179	213.8757286	213.7047826	230.1037474
ENSMUSG00000008206	CERS4	201.012052	125.8136501	123.8859375	155.1636678	135.0543111	167.3317029	145.0385605	131.4704063	149.1871931	133.3719431	131.3758909	129.722918
ENSMUSG00000008226	SCRN3	0	6.621771059	21.86222426	0	6.431157673	12.87166946	14.50385605	18.04495772	19.30657793	8.410843259	10.51007128	7.721602263
ENSMUSG00000008301	PHAX	78.65688991	59.59593953	58.2992647	77.58183392	83.60504975	96.53752092	73.83781261	76.04660755	77.22631171	79.30223644	91.08728439	92.65922716
ENSMUSG00000008307	1700109H08F	8.739654434	0	14.57481618	11.93566676	6.431157673	32.17917364	9.229726576	11.60032996	5.265430344	7.209294222	7.006714184	7.721602263
ENSMUSG00000008318	RELT	0	13.24354212	0	0	0	0	6.59266184	3.866776655	7.020573791	10.81394133	12.26174982	7.721602263
ENSMUSG00000008333	SNRPB2	218.4913609	304.6014687	247.771875	226.7776684	231.5216762	225.2542155	228.1060997	242.3180037	219.392931	242.7129055	217.2081397	223.9264656
ENSMUSG00000008348	UBC	297.1482508	437.0368899	349.7955882	262.5846686	437.3187217	353.97091	333.5886891	340.2763456	365.0698372	358.061613	387.1209587	375.26987
ENSMUSG00000008373	PRPF31	87.39654434	59.59593953	94.73630514	71.61400054	90.03620742	83.66585146	77.79340971	81.20230975	75.47116826	99.72857007	75.32217748	94.20354761
ENSMUSG00000008384	SERTAD1	0	6.621771059	7.287408088	5.967833378	6.431157673	6.435834728	3.955597104	11.60032996	1.755143448	4.806196148	10.51007128	4.632961358
ENSMUSG00000008393	CARHSP1	26.2189633	13.24354212	0	0	25.72463069	6.435834728	6.59266184	14.17818107	8.775717239	9.612392296	3.503357092	10.81024317
ENSMUSG00000008398	ELK3	43.69827217	92.70479482	43.72444853	47.74266702	38.58694604	38.61500837	42.19303578	45.11239431	47.38887309	33.64337304	57.80539202	40.15233177
ENSMUSG00000008429	HERPUD2	43.69827217	52.97416847	43.72444853	47.74266702	25.72463069	45.05084309	38.23743867	42.5345432	61.43002068	54.06970667	52.55035638	35.51937041
ENSMUSG00000008435	RDH13	34.95861774	6.621771059	29.14963235	29.83916689	38.58694604	12.87166946	30.32624446	20.62280883	24.57200827	37.24802015	24.52349964	27.79776815
ENSMUSG00000008450	NUTF2	0	0	0	0	0	0	1.318532368	1.288925552	3.510286896	0	1.751678546	3.088640905
ENSMUSG00000008475	ARPC5	279.6689419	311.2232398	182.1852022	226.7776684	199.3658879	405.4575878	212.2837112	216.5394927	242.2097958	207.8679834	220.7114968	199.2173384
ENSMUSG00000008482	RNF151	0	0	14.57481618	5.967833378	0	0	0	0	1.755143448	1.201549037	0	1.544320453
ENSMUSG00000008489	ELAVL2	0	0	0	0	0	6.435834728	0	0	0	0	0	0
ENSMUSG00000008496	POU2F2	78.65688991	132.4354212	211.3348345	149.1958345	141.4854688	141.588364	85.70460392	78.62445865	59.67487723	100.9301191	85.83224875	69.49442037
ENSMUSG00000008590	HTR3B	0	0	0	0	0	0	0	1.288925552	0	0	0	0

ENSMUSG00000008604	UBQLN4	61.17758104	79.46125271	94.73630514	83.54966729	57.88041905	45.05084309	65.9266184	81.20230975	80.7365986	66.08519704	80.57721311	74.12738173
ENSMUSG00000008668	RPS18	3810.489333	3728.057106	4139.247794	3843.284695	3794.383027	4015.96087	3412.361768	4013.714168	3868.336159	3163.678614	3951.7868	3885.510259
ENSMUSG00000008682	RPL10	436.9827217	377.4409504	342.5081801	405.8126697	398.7317757	418.3292573	576.1986448	599.3503815	519.5224606	559.9218513	628.852598	594.5633743
ENSMUSG00000008683	RPS15A	1136.155076	1271.380043	1231.571967	1110.017008	1086.865647	1197.065259	1339.628886	1304.392658	1365.501602	1217.169174	1240.188411	1241.633644
ENSMUSG00000008690	NCAPH2	69.91723547	66.21771059	80.16148896	119.3566676	96.46736509	83.66585146	77.79340971	78.62445865	68.45059447	64.883648	94.59064148	81.84898399
ENSMUSG00000008730	HIPK1	96.13619877	59.59593953	109.3111213	59.67833378	96.46736509	64.35834728	100.20846	74.757682	43.8785862	87.7130797	82.32889166	77.21602263
ENSMUSG00000008763	MAN1A2	227.2310153	225.140216	269.6340992	274.5203354	250.8151492	186.6392071	216.2393084	181.7385028	198.3312096	260.736141	206.6980684	200.7616588
ENSMUSG00000008822	ACYP1	26.2189633	19.86531318	21.86222426	17.90350013	25.72463069	12.87166946	34.28184157	33.51206434	47.38887309	37.24802015	35.03357092	40.15233177
ENSMUSG00000008855	HDAC5	34.95861774	46.35239741	36.43704044	29.83916689	51.44926138	102.9733556	68.56368314	52.84594762	66.69545102	46.86041244	64.8121062	64.86145901
ENSMUSG00000008859	RALA	113.6155076	39.73062635	109.3111213	77.58183392	102.8985228	25.74333891	61.9710213	74.757682	66.69545102	40.85266726	57.80539202	55.5955363
ENSMUSG00000008892	VDAC3	17.47930887	39.73062635	21.86222426	29.83916689	6.431157673	25.74333891	40.87450341	29.64528769	24.57200827	32.441824	28.02685674	33.97504996
ENSMUSG00000008958	VPS72	26.2189633	26.48708424	29.14963235	11.93566676	12.86231535	25.74333891	32.9633092	46.40131986	40.3682993	48.06196148	36.78524947	41.69665222
ENSMUSG00000008976	GABPA	8.739654434	26.48708424	65.58667279	53.7105004	32.15578836	45.05084309	46.14863288	56.71272427	70.20573791	37.24802015	70.06714184	60.22849765
ENSMUSG00000008999	BMP7	43.69827217	19.86531318	43.72444853	11.93566676	25.72463069	32.17917364	25.05211499	21.91173438	17.55143448	26.43407881	28.02685674	29.3420886
ENSMUSG00000009013	DYNLL1	166.0534342	158.9225054	225.9096507	208.8741682	147.9166265	109.4091904	164.816546	181.7385028	189.5554924	199.4571401	182.1745688	213.1162225
ENSMUSG00000009030	PDCL	34.95861774	52.97416847	43.72444853	29.83916689	77.17389207	38.61500837	46.14863288	47.69024541	52.65430344	54.06970667	43.79196365	52.50689539
ENSMUSG00000009035	TMEM184B	8.739654434	46.35239741	14.57481618	5.967833378	6.431157673	6.435834728	19.77798552	11.60032996	24.57200827	19.22478459	17.51678546	21.62048634
ENSMUSG00000009073	NF2	87.39654434	39.73062635	36.43704044	59.67833378	12.86231535	64.35834728	38.23743867	51.55702207	42.12344275	38.44956918	54.30203492	60.22849765
ENSMUSG00000009075	CABP7	0	0	7.287408088	0	0	0	1.318532368	0	7.020573791	2.403098074	1.751678546	10.81024317
ENSMUSG00000009076	ZMAT5	8.739654434	6.621771059	36.43704044	23.87133351	32.15578836	32.17917364	34.28184157	38.66776655	50.89915999	32.441824	40.28860656	54.05121584
ENSMUSG00000009079	EWSR1	506.8999572	463.5239741	473.6815257	656.4616716	475.9056678	566.353456	494.449638	551.6601361	586.2179116	543.1001647	534.2619565	480.2836608
ENSMUSG00000009090	AP1B1	52.4379266	33.10885529	36.43704044	41.77483365	64.31157673	25.74333891	47.46716525	47.69024541	38.61315585	30.03872593	57.80539202	64.86145901
ENSMUSG00000009092	DERL3	0	0	7.287408088	0	0	0	2.637064736	1.288925552	1.755143448	3.604647111	1.751678546	0
ENSMUSG00000009093	GSTT4	0	0	0	0	0	6.435834728	3.955597104	5.155702207	3.510286896	3.604647111	7.006714184	3.088640905
ENSMUSG00000009108	GNAT2	0	6.621771059	0	0	0	0	1.318532368	1.288925552	1.755143448	1.201549037	1.751678546	1.544320453
ENSMUSG00000009112	BCL2L13	61.17758104	39.73062635	51.01185661	107.4210008	57.88041905	45.05084309	61.9710213	76.04660755	59.67487723	49.26351052	68.31546329	61.77281811
ENSMUSG00000009115	SPATC1L	0	0	7.287408088	0	0	0	1.318532368	0	1.755143448	0	0	1.544320453
ENSMUSG00000009145	DQX1	17.47930887	6.621771059	0	23.87133351	19.29347302	6.435834728	10.54825894	10.31140441	12.28600414	6.007745185	7.006714184	6.177281811
ENSMUSG00000009207	LNPK	166.0534342	165.5442765	174.8977941	161.1315012	192.9347302	122.2808598	129.2161721	113.4254485	108.8188938	108.1394133	99.84567712	117.3683544
ENSMUSG00000009246	TRPM5	0	0	0	0	0	0	0	0	0	1.201549037	0	0
ENSMUSG00000009248	ASCL2	0	0	0	0	0	0	0	1.288925552	1.755143448	0	0	0
ENSMUSG00000009281	RARRES2	0	0	0	0	0	0	0	1.288925552	1.755143448	0	0	0
ENSMUSG00000009291	PTTG1IP	34.95861774	6.621771059	21.86222426	11.93566676	19.29347302	19.30750418	27.68917973	23.20065993	21.06172137	19.22478459	33.28189237	23.16480679
ENSMUSG00000009292	TRPM2	34.95861774	6.621771059	21.86222426	29.83916689	19.29347302	0	17.14092078	11.60032996	5.265430344	7.209294222	12.26174982	18.53184543
ENSMUSG00000009293	UBE2G2	34.95861774	26.48708424	43.72444853	41.77483365	32.15578836	38.61500837	59.33395656	56.71272427	70.20573791	60.07745185	56.05371347	71.03874082
ENSMUSG00000009350	MPO	0	0	0	0	0	0	0	0	1.755143448	0	0	0
ENSMUSG00000009406	ELK1	8.739654434	26.48708424	29.14963235	11.93566676	6.431157673	6.435834728	15.82238842	15.46710662	8.775717239	8.410843259	17.51678546	21.62048634
ENSMUSG00000009418	NAV1	113.6155076	92.70479482	58.2992647	53.7105004	77.17389207	70.79418201	48.78569762	67.02412869	52.65430344	42.0542163	68.31546329	49.41825449
ENSMUSG00000009470	TNPO1	104.8758532	112.570108	167.610386	83.54966729	77.17389207	90.10168619	121.3049779	95.38049082	96.53288963	118.9533547	92.83896294	123.5456362
ENSMUSG00000009535	RNMT	96.13619877	52.97416847	43.72444853	41.77483365	64.31157673	70.79418201	89.66020102	82.49123531	61.43002068	85.30998163	91.08728439	40.15233177
ENSMUSG00000009549	SRP14	209.7517064	165.5442765	196.7600184	179.0350013	289.4020953	173.7675376	286.1215239	253.9183337	303.6398165	263.1392391	280.2685674	251.7242338
ENSMUSG00000009555	CDK9	148.5741254	112.570108	182.1852022	137.2601677	192.9347302	148.0241987	201.7354523	220.4062693	247.4752262	194.650944	234.7249252	251.7242338
ENSMUSG00000009563	TOR2A	61.17758104	39.73062635	51.01185661	59.67833378	57.88041905	32.17917364	55.37835946	68.31305424	91.26745929	44.45731437	92.83896294	75.67170218
ENSMUSG00000009566	FPGS	96.13619877	99.32656588	138.4607537	125.3245009	160.7789418	115.8450251	126.5791073	123.736853	129.8806151	126.1626489	105.1007128	131.2672385
ENSMUSG00000009569	MKL2	8.739654434	6.621771059	7.287408088	17.90350013	6.431157673	0	11.86679131	7.73355331	21.06172137	13.21703941	8.75839273	15.44320453

ENSMUSG00000009575	CBX5	305.8879052	311.2232398	335.220772	214.8420016	263.6774646	244.5617197	243.9284881	271.9632914	203.59664	258.333043	299.5370314	227.0151065
ENSMUSG00000009585	APOBEC3	122.3551621	185.4095896	131.1733456	95.48533405	154.3477841	115.8450251	109.4381865	116.0032996	115.8394676	104.5347662	99.84567712	106.5581112
ENSMUSG00000009596	TAF7L	0	0	0	0	0	0	0	1.288925552	0	0	0	0
ENSMUSG00000009614	SARDH	0	0	0	0	0	0	1.318532368	0	0	1.201549037	3.503357092	1.544320453
ENSMUSG00000009621	VAV2	17.47930887	59.59593953	7.287408088	41.77483365	32.15578836	51.48667782	52.74129472	34.80098989	52.65430344	33.64337304	33.28189237	55.5955363
ENSMUSG00000009630	PPP2CB	34.95861774	13.24354212	0	11.93566676	0	6.435834728	13.18532368	14.17818107	22.81686482	12.01549037	12.26174982	21.62048634
ENSMUSG00000009640	DMAP1	96.13619877	66.21771059	51.01185661	23.87133351	51.44926138	57.92251255	44.83010051	45.11239431	61.43002068	54.06970667	49.04699929	37.06369086
ENSMUSG00000009647	MCU	0	0	7.287408088	11.93566676	19.29347302	32.17917364	6.59266184	15.46710662	17.55143448	14.41858844	8.75839273	13.89888407
ENSMUSG00000009681	BCR	0	0	0	0	0	0	0	0	0	0	1.751678546	0
ENSMUSG00000009687	FXYD5	131.0948165	66.21771059	94.73630514	107.4210008	83.60504975	90.10168619	112.0752513	103.1140441	66.69545102	105.7363153	91.08728439	109.6467521
ENSMUSG00000009731	KCND1	8.739654434	6.621771059	0	5.967833378	0	0	0	0	0	1.201549037	0	0
ENSMUSG00000009733	TFCP2	17.47930887	13.24354212	0	5.967833378	6.431157673	19.30750418	15.82238842	16.75603217	21.06172137	22.8294317	15.76510691	20.07616588
ENSMUSG00000009734	POU6F2	0	0	0	0	0	0	0	0	1.755143448	2.403098074	0	0
ENSMUSG00000009739	POU6F1	0	6.621771059	0	0	0	0	0	0	0	0	0	0
ENSMUSG00000009741	UBP1	253.4499786	370.8191793	211.3348345	370.0056694	263.6774646	250.9975544	259.7508765	274.5411425	326.4566813	326.8213381	311.7987812	293.420886
ENSMUSG00000009828	ICK	17.47930887	13.24354212	14.57481618	5.967833378	19.29347302	12.87166946	15.82238842	10.31140441	10.53086069	13.21703941	8.75839273	3.088640905
ENSMUSG00000009863	SDHB	166.0534342	198.6531318	174.8977941	107.4210008	141.4854688	122.2808598	208.3281141	188.1831305	214.1275006	170.6199633	208.449747	183.7741339
ENSMUSG00000009894	SNAP47	26.2189633	13.24354212	14.57481618	17.90350013	19.29347302	12.87166946	14.50385605	11.60032996	14.04114758	25.23252978	7.006714184	12.35456362
ENSMUSG00000009905	KDSR	157.3137798	185.4095896	211.3348345	196.9385015	199.3658879	167.3317029	114.712316	96.66941637	117.594611	118.9533547	127.8725339	137.4445203
ENSMUSG00000009907	VPS4B	78.65688991	52.97416847	43.72444853	41.77483365	45.01810371	38.61500837	29.0077121	38.66776655	45.63372964	57.67435378	66.56378475	38.60801132
ENSMUSG00000009927	RPS25	1660.534342	1476.654946	1872.863879	1575.508012	1736.412572	1615.394517	1972.524423	2008.146009	2042.986973	1828.757634	2010.926971	2044.680279
ENSMUSG00000009995	TAZ	26.2189633	26.48708424	21.86222426	5.967833378	38.58694604	25.74333891	23.73358262	25.77851103	31.59258206	21.62788267	24.52349964	29.3420886
ENSMUSG00000010025	ALDH3A2	17.47930887	6.621771059	21.86222426	23.87133351	12.86231535	12.87166946	9.229726576	9.022478862	21.06172137	4.806196148	8.75839273	12.35456362
ENSMUSG00000010044	ZMYND10	0	0	0	0	0	0	1.318532368	0	0	0	1.751678546	1.544320453
ENSMUSG00000010045	TMEM115	0	6.621771059	7.287408088	17.90350013	12.86231535	0	18.45945315	16.75603217	15.79629103	25.23252978	12.26174982	27.79776815
ENSMUSG00000010047	HYAL2	0	6.621771059	0	5.967833378	6.431157673	0	6.59266184	7.73355331	8.775717239	6.007745185	12.26174982	10.81024317
ENSMUSG00000010048	IFRD2	34.95861774	59.59593953	43.72444853	35.80700027	45.01810371	64.35834728	73.83781261	64.44627758	78.98145515	73.29449126	70.06714184	67.95009992
ENSMUSG00000010051	HYAL1	8.739654434	0	0	5.967833378	0	0	0	3.866776655	0	1.201549037	1.751678546	3.088640905
ENSMUSG00000010054	TUSC2	17.47930887	26.48708424	29.14963235	5.967833378	38.58694604	32.17917364	25.05211499	25.77851103	21.06172137	19.22478459	26.27517819	26.2534477
ENSMUSG00000010057	NPRL2	34.95861774	0	0	5.967833378	12.86231535	12.87166946	11.86679131	9.022478862	10.53086069	13.21703941	22.7718211	13.89888407
ENSMUSG00000010066	CACNA2D2	2106.256719	1860.717668	2164.360202	2130.516516	2276.629816	2336.208006	1538.727273	1932.099402	1767.429452	1704.998084	1734.161761	1714.195702
ENSMUSG00000010067	RASSF1	43.69827217	26.48708424	14.57481618	17.90350013	57.88041905	12.87166946	29.0077121	25.77851103	29.83743861	22.8294317	21.02014255	35.51937041
ENSMUSG00000010080	EPN3	0	0	0	0	0	0	1.318532368	1.288925552	0	0	0	0
ENSMUSG00000010095	SLC3A2	61.17758104	72.83948165	87.44889705	107.4210008	90.03620742	77.23001673	84.38607155	112.136523	115.8394676	85.30998163	113.8591055	125.0899567
ENSMUSG00000010097	NXF1	131.0948165	86.08302377	87.44889705	47.74266702	77.17389207	90.10168619	83.06753918	92.80263972	117.594611	79.30223644	113.8591055	103.4694703
ENSMUSG00000010110	STX5A	78.65688991	26.48708424	36.43704044	47.74266702	45.01810371	77.23001673	29.0077121	50.26809651	47.38887309	34.84492207	40.28860656	50.96257494
ENSMUSG00000010136	PIFO	8.739654434	0	0	0	6.431157673	0	0	0	0	0	0	0
ENSMUSG00000010154	SPIRE2	0	0	0	0	0	6.435834728	1.318532368	2.577851103	3.510286896	2.403098074	5.255035638	4.632961358
ENSMUSG00000010277	2610507B11F	113.6155076	79.46125271	94.73630514	89.51750067	96.46736509	90.10168619	102.8455247	105.6918952	101.79832	109.3409624	117.3624626	142.0774816
ENSMUSG00000010290	AI597479	17.47930887	19.86531318	14.57481618	17.90350013	12.86231535	32.17917364	19.77798552	27.06743658	28.08229517	21.62788267	28.02685674	21.62048634
ENSMUSG00000010342	TEX14	17.47930887	39.73062635	0	23.87133351	19.29347302	12.87166946	15.82238842	27.06743658	22.81686482	15.62013748	15.76510691	13.89888407
ENSMUSG00000010358	IFI35	17.47930887	0	7.287408088	11.93566676	25.72463069	6.435834728	22.41505026	23.20065993	14.04114758	9.612392296	26.27517819	16.98752498
ENSMUSG00000010362	RDM1	17.47930887	6.621771059	0	0	0	6.435834728	3.955597104	1.288925552	0	1.201549037	3.503357092	4.632961358
ENSMUSG00000010376	NEDD8	131.0948165	119.1918791	116.5985294	59.67833378	135.0543111	128.7166946	180.6389344	141.7818107	166.7386275	150.1936296	183.9262473	179.1411725
ENSMUSG00000010392	GOSR1	43.69827217	26.48708424	21.86222426	41.77483365	12.86231535	45.05084309	25.05211499	28.35636214	29.83743861	28.83717689	22.7718211	35.51937041

ENSMUSG00000010406	MRPL52	122.3551621	46.35239741	87.44889705	71.61400054	90.03620742	57.92251255	97.57139523	83.78016086	75.47116826	96.12392296	87.5839273	94.20354761
ENSMUSG00000010453	KANSL3	166.0534342	92.70479482	80.16148896	101.4531674	122.1919958	90.10168619	96.25286286	101.8251186	108.8188938	80.50378548	91.08728439	98.83650897
ENSMUSG00000010492	UCKL1OS	0	0	0	11.93566676	6.431157673	12.87166946	5.274129472	6.444627758	7.020573791	4.806196148	8.75839273	3.088640905
ENSMUSG00000010517	FAF1	52.4379266	52.97416847	36.43704044	53.7105004	6.431157673	38.61500837	51.42276235	59.29057538	66.69545102	51.66660859	47.29532074	54.05121584
ENSMUSG00000010529	GM266	0	0	0	0	0	0	0	1.288925552	0	0	0	0
ENSMUSG00000010538	TSACC	17.47930887	0	0	11.93566676	0	0	2.637064736	1.288925552	3.510286896	0	3.503357092	1.544320453
ENSMUSG00000010554	METTL16	43.69827217	59.59593953	29.14963235	29.83916689	45.01810371	45.05084309	48.78569762	70.89090534	64.94030757	46.86041244	84.08057021	58.6841772
ENSMUSG00000010592	DAZL	0	0	0	0	0	0	0	0	0	0	1.751678546	0
ENSMUSG00000010607	PIGYL	34.95861774	6.621771059	21.86222426	17.90350013	19.29347302	25.74333891	23.73358262	18.04495772	35.10286896	14.41858844	26.27517819	16.98752498
ENSMUSG00000010608	RBM25	1756.670541	1827.608812	1836.426838	1891.803181	1858.604567	1563.907839	1315.895303	1446.174469	1439.217627	1340.928725	1616.799298	1266.342771
ENSMUSG00000010609	PSEN2	218.4913609	192.0313607	218.6222426	125.3245009	115.7608381	135.1525293	152.9497547	127.6036296	101.79832	98.52702104	143.6376408	149.7990839
ENSMUSG00000010663	FADS1	0	13.24354212	21.86222426	11.93566676	6.431157673	12.87166946	15.82238842	15.46710662	19.30657793	16.82168652	10.51007128	16.98752498
ENSMUSG00000010751	TNFRSF22	0	0	0	0	0	0	0	1.288925552	0	0	0	0
ENSMUSG00000010755	CARS	192.2723975	112.570108	196.7600184	226.7776684	141.4854688	128.7166946	184.5945315	170.1381728	201.8414965	171.8215123	166.4094619	180.685493
ENSMUSG00000010825	GRID2IP	0	0	0	0	6.431157673	0	0	0	0	0	0	0
ENSMUSG00000010830	KDELR3	0	0	7.287408088	5.967833378	0	0	0	2.577851103	0	0	1.751678546	1.544320453
ENSMUSG00000010911	APIP	0	52.97416847	29.14963235	17.90350013	0	19.30750418	29.0077121	29.64528769	26.32715172	30.03872593	21.02014255	32.43072951
ENSMUSG00000010914	PDHX	26.2189633	26.48708424	58.2992647	23.87133351	25.72463069	51.48667782	51.42276235	45.11239431	61.43002068	36.04647111	56.05371347	52.50689539
ENSMUSG00000010936	VAC14	43.69827217	39.73062635	36.43704044	11.93566676	25.72463069	19.30750418	31.64477683	41.24561765	33.34772551	37.24802015	35.03357092	35.51937041
ENSMUSG00000011008	MCOLN2	61.17758104	59.59593953	72.87408088	95.48533405	70.7427344	83.66585146	93.61579813	83.78016086	96.53288963	70.89139318	89.33560584	74.12738173
ENSMUSG00000011096	AKT1S1	17.47930887	26.48708424	14.57481618	35.80700027	32.15578836	25.74333891	25.05211499	18.04495772	17.55143448	20.42633363	28.02685674	33.97504996
ENSMUSG00000011114	TBRG1	113.6155076	59.59593953	58.2992647	143.2280011	77.17389207	83.66585146	76.47487734	79.9133842	71.96088136	73.29449126	98.09399857	77.21602263
ENSMUSG00000011118	PANX3	0	0	7.287408088	0	0	0	0	0	0	0	0	0
ENSMUSG00000011148	ADSSL1	0	0	0	0	0	0	1.318532368	0	0	2.403098074	0	0
ENSMUSG00000011158	BRF1	69.91723547	59.59593953	51.01185661	35.80700027	57.88041905	57.92251255	68.56368314	63.15735203	59.67487723	46.86041244	54.30203492	43.24097267
ENSMUSG00000011171	VIPR2	0	0	0	0	0	6.435834728	0	0	1.755143448	0	1.751678546	0
ENSMUSG00000011179	ODC1	26.2189633	6.621771059	21.86222426	11.93566676	12.86231535	6.435834728	15.82238842	20.62280883	28.08229517	22.8294317	28.02685674	16.98752498
ENSMUSG00000011254	THG1L	0	26.48708424	29.14963235	17.90350013	32.15578836	25.74333891	17.14092078	15.46710662	28.08229517	9.612392296	19.26846401	10.81024317
ENSMUSG00000011256	ADAM19	0	0	0	0	0	0	0	0	1.755143448	0	0	0
ENSMUSG00000011257	PABPC4	148.5741254	178.7878186	131.1733456	155.1636678	122.1919958	154.4600335	112.0752513	136.6261085	152.69748	132.1703941	138.3826051	152.8877248
ENSMUSG00000011267	ZFP296	0	6.621771059	0	0	0	0	0	0	0	0	0	0
ENSMUSG00000011306	SUGP1	87.39654434	26.48708424	21.86222426	35.80700027	32.15578836	25.74333891	31.64477683	56.71272427	43.8785862	20.42633363	52.55035638	49.41825449
ENSMUSG00000011350	GM5893	0	0	7.287408088	0	0	0	0	0	0	0	0	0
ENSMUSG00000011382	DHDH	0	0	0	0	0	0	1.318532368	0	0	0	0	0
ENSMUSG00000011427	ZFP790	0	6.621771059	0	5.967833378	6.431157673	6.435834728	6.59266184	3.866776655	1.755143448	2.403098074	3.503357092	7.721602263
ENSMUSG00000011463	CPB1	8.739654434	13.24354212	7.287408088	5.967833378	6.431157673	6.435834728	3.955597104	11.60032996	10.53086069	6.007745185	8.75839273	12.35456362
ENSMUSG00000011486	SLC25A41	0	0	0	0	0	0	2.637064736	0	0	0	0	0
ENSMUSG00000011658	FUZ	26.2189633	6.621771059	0	17.90350013	19.29347302	6.435834728	14.50385605	23.20065993	10.53086069	10.81394133	7.006714184	4.632961358
ENSMUSG00000011751	SPTBN4	0	0	0	0	0	0	0	0	1.755143448	1.201549037	0	0
ENSMUSG00000011752	PGAM1	0	0	7.287408088	0	0	0	5.274129472	2.577851103	3.510286896	2.403098074	1.751678546	0
ENSMUSG00000011831	EVI5	17.47930887	39.73062635	21.86222426	53.7105004	57.88041905	32.17917364	29.0077121	27.06743658	49.14401654	28.83717689	28.02685674	20.07616588
ENSMUSG00000011832	EVI5L	8.739654434	6.621771059	14.57481618	5.967833378	6.431157673	0	6.59266184	9.022478862	10.53086069	4.806196148	5.255035638	6.177281811
ENSMUSG00000011837	SNAPC2	0	13.24354212	7.287408088	0	6.431157673	12.87166946	7.911194208	7.73355331	3.510286896	4.806196148	8.75839273	12.35456362
ENSMUSG00000011877	GIT1	87.39654434	66.21771059	94.73630514	53.7105004	64.31157673	64.35834728	81.74900682	72.17983089	80.7365986	78.10068741	87.5839273	84.9376249
ENSMUSG00000011884	GLTP	26.2189633	66.21771059	51.01185661	41.77483365	45.01810371	32.17917364	59.33395656	46.40131986	35.10286896	42.0542163	38.53692801	32.43072951

ENSMUSG00000011958	BNIP2	174.7930887	105.9483369	123.8859375	137.2601677	160.7789418	167.3317029	133.1717692	131.4704063	117.594611	116.5502566	140.1342837	112.735393
ENSMUSG00000011960	CCNT1	297.1482508	291.3579266	204.0474265	232.7455017	135.0543111	218.8183807	138.4458986	148.2264384	157.9629103	145.3874335	147.1409979	162.1536475
ENSMUSG00000012017	SCARF2	0	0	0	0	0	0	1.318532368	0	0	0	0	0
ENSMUSG00000012076	BRMS1L	17.47930887	0	21.86222426	23.87133351	32.15578836	12.87166946	14.50385605	24.48958548	21.06172137	25.23252978	17.51678546	21.62048634
ENSMUSG00000012114	MED15	69.91723547	39.73062635	21.86222426	47.74266702	19.29347302	57.92251255	36.9189063	29.64528769	33.34772551	20.42633363	33.28189237	40.15233177
ENSMUSG00000012117	DHDDS	26.2189633	13.24354212	21.86222426	23.87133351	12.86231535	25.74333891	11.86679131	11.60032996	14.04114758	24.03098074	5.255035638	16.98752498
ENSMUSG00000012126	UBXN11	0	0	7.287408088	5.967833378	6.431157673	0	5.274129472	0	0	1.201549037	0	4.632961358
ENSMUSG00000012211	TEX22	0	0	0	0	0	0	0	0	0	1.201549037	0	0
ENSMUSG00000012296	TJAP1	52.4379266	33.10885529	36.43704044	53.7105004	45.01810371	57.92251255	36.9189063	32.22313879	49.14401654	37.24802015	49.04699929	49.41825449
ENSMUSG00000012405	RPL15	131.0948165	72.83948165	94.73630514	89.51750067	70.7427344	115.8450251	93.61579813	81.20230975	70.20573791	86.51153067	82.32889166	100.3808294
ENSMUSG00000012422	TMEM167	174.7930887	86.08302377	80.16148896	190.9706681	96.46736509	128.7166946	84.38607155	110.8475974	89.51231584	92.51927585	89.33560584	100.3808294
ENSMUSG00000012429	MPLKIP	43.69827217	33.10885529	51.01185661	47.74266702	6.431157673	57.92251255	65.9266184	48.97917096	43.8785862	46.86041244	31.53021383	43.24097267
ENSMUSG00000012443	KIF11	113.6155076	105.9483369	109.3111213	95.48533405	70.7427344	70.79418201	109.4381865	104.4029697	93.02260274	99.72857007	119.1141411	114.2797135
ENSMUSG00000012483	RPA3	349.5861774	264.8708424	291.4963235	244.6811685	340.8513567	250.9975544	458.8492641	462.724273	447.5615792	338.8368284	499.2283856	381.4471518
ENSMUSG00000012535	TNPO3	358.3258318	344.3320951	262.3466912	381.9413362	398.7317757	334.6634058	329.633092	360.8991545	359.8044068	379.6894957	364.3491376	369.0925882
ENSMUSG00000012609	TTLL5	0	19.86531318	21.86222426	23.87133351	6.431157673	6.435834728	3.955597104	12.88925552	8.775717239	8.410843259	19.26846401	12.35456362
ENSMUSG00000012640	ZFP715	0	13.24354212	0	5.967833378	45.01810371	6.435834728	18.45945315	14.17818107	21.06172137	12.01549037	14.01342837	16.98752498
ENSMUSG00000012777	ACP4	8.739654434	0	7.287408088	17.90350013	12.86231535	0	10.54825894	10.31140441	19.30657793	15.62013748	15.76510691	6.177281811
ENSMUSG00000012819	CDH23	0	0	0	0	0	0	0	1.288925552	0	0	0	1.544320453
ENSMUSG00000012848	RPS5	1188.593003	1032.996285	1165.985294	1008.563841	964.6736509	939.6318703	1296.117318	1367.55001	1311.092156	1165.502566	1429.369694	1397.61001
ENSMUSG00000012889	PODNL1	17.47930887	6.621771059	0	35.80700027	12.86231535	19.30750418	7.911194208	12.88925552	14.04114758	4.806196148	7.006714184	6.177281811
ENSMUSG00000013033	ADGRL1	131.0948165	99.32656588	123.8859375	143.2280011	90.03620742	102.9733556	64.60808603	74.757682	71.96088136	82.90688355	57.80539202	83.39330444
ENSMUSG00000013076	AMOTL1	0	6.621771059	0	0	0	0	0	0	0	0	0	0
ENSMUSG00000013089	ETV5	34.95861774	13.24354212	94.73630514	53.7105004	51.44926138	38.61500837	48.78569762	58.00164982	49.14401654	44.45731437	33.28189237	55.5955363
ENSMUSG00000013091	TMEM190	0	0	0	0	0	0	1.318532368	2.577851103	0	0	0	1.544320453
ENSMUSG00000013150	GFOD2	8.739654434	26.48708424	7.287408088	5.967833378	12.86231535	0	11.86679131	9.022478862	7.020573791	2.403098074	8.75839273	10.81024317
ENSMUSG00000013155	ENKD1	113.6155076	105.9483369	94.73630514	59.67833378	38.58694604	90.10168619	50.10422998	67.02412869	61.43002068	44.45731437	80.57721311	61.77281811
ENSMUSG00000013158	4933405L10R	0	0	0	0	0	0	1.318532368	1.288925552	0	0	0	0
ENSMUSG00000013160	ATP6V0D1	26.2189633	26.48708424	21.86222426	5.967833378	38.58694604	0	34.28184157	23.20065993	10.53086069	27.63562785	21.02014255	35.51937041
ENSMUSG00000013236	PTPRS	244.7103242	271.4926134	349.7955882	298.3916689	321.5578836	302.4842322	230.7431644	261.651887	300.1295296	279.9609256	289.0269601	267.1674383
ENSMUSG00000013275	SLC41A1	0	13.24354212	7.287408088	5.967833378	19.29347302	6.435834728	14.50385605	9.022478862	8.775717239	14.41858844	10.51007128	26.2534477
ENSMUSG00000013415	IGF2BP1	0	0	0	0	0	0	0	1.288925552	0	0	0	0
ENSMUSG00000013418	B4GALNT2	0	0	0	5.967833378	0	0	0	0	0	0	0	0
ENSMUSG00000013419	ZFP651	0	0	14.57481618	0	6.431157673	0	1.318532368	0	3.510286896	2.403098074	1.751678546	3.088640905
ENSMUSG00000013465	NELFB	78.65688991	99.32656588	58.2992647	71.61400054	45.01810371	57.92251255	94.9343305	99.24726748	89.51231584	70.89139318	91.08728439	89.57058625
ENSMUSG00000013495	TMEM175	0	0	21.86222426	23.87133351	12.86231535	0	10.54825894	10.31140441	15.79629103	14.41858844	17.51678546	16.98752498
ENSMUSG00000013523	BCAS1	17.47930887	6.621771059	14.57481618	11.93566676	6.431157673	0	2.637064736	0	3.510286896	0	1.751678546	1.544320453
ENSMUSG00000013539	TANGO2	17.47930887	19.86531318	14.57481618	17.90350013	6.431157673	19.30750418	14.50385605	15.46710662	17.55143448	12.01549037	17.51678546	15.44320453
ENSMUSG00000013593	NDUFS2	166.0534342	125.8136501	65.58667279	137.2601677	167.2100995	90.10168619	125.260575	153.3821406	156.2077669	164.6122181	129.6242124	172.9638907
ENSMUSG00000013611	SNX31	0	0	7.287408088	0	0	0	0	2.577851103	0	0	1.751678546	1.544320453
ENSMUSG00000013622	ATRAID	26.2189633	0	7.287408088	17.90350013	25.72463069	19.30750418	10.54825894	14.17818107	24.57200827	12.01549037	15.76510691	12.35456362
ENSMUSG00000013629	CAD	78.65688991	125.8136501	116.5985294	149.1958345	141.4854688	109.4091904	121.3049779	103.1140441	119.3497545	87.7130797	136.6309266	112.735393
ENSMUSG00000013646	SH3BP5L	8.739654434	13.24354212	21.86222426	11.93566676	0	25.74333891	14.50385605	16.75603217	21.06172137	13.21703941	17.51678546	16.98752498
ENSMUSG00000013662	ATAD1	61.17758104	66.21771059	72.87408088	101.4531674	51.44926138	109.4091904	71.20074787	65.73520313	75.47116826	94.92237392	52.55035638	84.9376249
ENSMUSG00000013663	PTEN	445.7223761	423.7933478	488.2563419	495.3301704	366.5759873	347.5350753	479.9457819	482.0581563	472.1335875	443.3715947	514.9934925	455.5745335

ENSMUSG00000013698	PEA15A	0	6.621771059	0	17.90350013	6.431157673	0	7.911194208	7.73355331	5.265430344	6.007745185	5.255035638	6.177281811
ENSMUSG00000013701	TIMM23	0	6.621771059	7.287408088	0	0	6.435834728	3.955597104	3.866776655	5.265430344	3.604647111	5.255035638	4.632961358
ENSMUSG00000013707	TNFAIP8L2	34.95861774	19.86531318	43.72444853	11.93566676	25.72463069	19.30750418	26.37064736	37.378841	26.32715172	7.209294222	29.77853528	30.88640905
ENSMUSG00000013736	TRNT1	87.39654434	86.08302377	102.0237132	95.48533405	115.7608381	57.92251255	123.9420426	154.6710662	163.2283407	185.0385517	171.6644975	157.5206862
ENSMUSG00000013787	EHMT2	122.3551621	112.570108	87.44889705	143.2280011	122.1919958	96.53752092	116.0308484	153.3821406	103.5534634	111.7440604	134.879248	143.6218021
ENSMUSG00000013822	ELOF1	34.95861774	13.24354212	29.14963235	23.87133351	25.72463069	38.61500837	36.9189063	36.08991545	47.38887309	32.441824	36.78524947	40.15233177
ENSMUSG00000013833	MED16	8.739654434	6.621771059	0	5.967833378	0	12.87166946	9.229726576	10.31140441	15.79629103	9.612392296	12.26174982	9.265922716
ENSMUSG00000013846	ST3GAL1	8.739654434	13.24354212	14.57481618	11.93566676	0	32.17917364	9.229726576	18.04495772	15.79629103	13.21703941	21.02014255	12.35456362
ENSMUSG00000013858	TMEM259	113.6155076	86.08302377	204.0474265	101.4531674	96.46736509	135.1525293	98.8899276	110.8475974	117.594611	93.72082489	113.8591055	123.5456362
ENSMUSG00000013878	RNF170	0	6.621771059	14.57481618	0	0	12.87166946	6.59266184	2.577851103	5.265430344	1.201549037	7.006714184	4.632961358
ENSMUSG00000013973	DEDD	69.91723547	52.97416847	51.01185661	89.51750067	70.7427344	96.53752092	75.15634498	76.04660755	63.18516412	70.89139318	59.55707056	66.40577946
ENSMUSG00000013997	NIT1	192.2723975	112.570108	167.610386	131.2923343	128.6231535	238.1258849	93.61579813	85.06908641	94.77774619	96.12392296	87.5839273	89.57058625
ENSMUSG00000014030	PAX5	0	0	0	0	0	6.435834728	0	1.288925552	0	0	1.751678546	1.544320453
ENSMUSG00000014039	PRDM15	26.2189633	26.48708424	21.86222426	17.90350013	6.431157673	6.435834728	10.54825894	25.77851103	19.30657793	14.41858844	21.02014255	21.62048634
ENSMUSG00000014074	RNF168	78.65688991	79.46125271	65.58667279	59.67833378	57.88041905	45.05084309	58.01542419	74.757682	75.47116826	74.4960403	50.79867783	38.60801132
ENSMUSG00000014077	CHP1	69.91723547	59.59593953	29.14963235	35.80700027	57.88041905	32.17917364	64.60808603	72.17983089	70.20573791	57.67435378	70.06714184	88.0262658
ENSMUSG00000014158	TRPV4	0	0	0	0	0	0	0	0	0	1.201549037	0	0
ENSMUSG00000014177	TVP23B	61.17758104	86.08302377	21.86222426	77.58183392	77.17389207	25.74333891	101.5269923	79.9133842	84.2468855	114.1471585	101.5973557	118.9126749
ENSMUSG00000014195	DNAJC7	148.5741254	139.0571922	131.1733456	113.3888342	154.3477841	135.1525293	118.6679131	135.3371829	138.6563324	152.5967277	169.912819	135.9001998
ENSMUSG00000014198	ZFP385C	0	0	0	0	0	6.435834728	1.318532368	1.288925552	0	1.201549037	0	0
ENSMUSG00000014226	CACYBP	166.0534342	205.2749028	204.0474265	280.4881688	160.7789418	205.9467113	315.129236	323.5203135	319.4361075	325.619789	294.2819957	271.8003997
ENSMUSG00000014232	CLUAP1	17.47930887	13.24354212	58.2992647	53.7105004	38.58694604	25.74333891	47.46716525	42.5345432	38.61315585	30.03872593	38.53692801	26.2534477
ENSMUSG00000014243	ZSWIM7	0	6.621771059	7.287408088	0	6.431157673	0	10.54825894	5.155702207	7.020573791	7.209294222	0	12.35456362
ENSMUSG00000014245	PIGL	26.2189633	19.86531318	51.01185661	41.77483365	38.58694604	57.92251255	29.0077121	29.64528769	40.3682993	42.0542163	21.02014255	43.24097267
ENSMUSG00000014294	NDUFA2	218.4913609	218.5184449	182.1852022	173.067168	180.0724148	135.1525293	257.1138118	244.8958548	259.7612303	223.4881209	276.7652103	260.9901565
ENSMUSG00000014301	PAM16	43.69827217	52.97416847	29.14963235	53.7105004	51.44926138	45.05084309	50.10422998	60.57950093	70.20573791	48.06196148	36.78524947	75.67170218
ENSMUSG00000014303	GLIS2	8.739654434	19.86531318	51.01185661	5.967833378	25.72463069	0	34.28184157	20.62280883	17.55143448	13.21703941	15.76510691	13.89888407
ENSMUSG00000014313	COX6C	568.0775382	390.6844925	510.1185661	340.1665026	469.4745101	431.2009268	627.6214072	689.5751701	603.7693461	569.5342435	686.65799	693.3998832
ENSMUSG00000014329	BICC1	0	6.621771059	7.287408088	0	0	0	0	0	0	0	0	0
ENSMUSG00000014349	UBE2Z	78.65688991	26.48708424	43.72444853	77.58183392	83.60504975	38.61500837	52.74129472	47.69024541	71.96088136	50.46505955	57.80539202	35.51937041
ENSMUSG00000014351	GIP	0	0	0	0	0	0	0	1.288925552	0	0	0	0
ENSMUSG00000014353	TMEM87B	17.47930887	26.48708424	29.14963235	5.967833378	51.44926138	19.30750418	21.09651789	21.91173438	28.08229517	18.02323556	14.01342837	15.44320453
ENSMUSG00000014355	ANAPC1	122.3551621	92.70479482	116.5985294	149.1958345	147.9166265	77.23001673	93.61579813	94.09156527	100.0431765	106.9378643	96.34232003	100.3808294
ENSMUSG00000014402	TSG101	26.2189633	19.86531318	14.57481618	17.90350013	45.01810371	32.17917364	27.68917973	37.378841	35.10286896	39.65111822	31.53021383	16.98752498
ENSMUSG00000014418	HPS5	8.739654434	26.48708424	7.287408088	35.80700027	19.29347302	19.30750418	30.32624446	25.77851103	28.08229517	22.8294317	35.03357092	30.88640905
ENSMUSG00000014426	MAP3K4	104.8758532	52.97416847	58.2992647	65.64616716	51.44926138	38.61500837	81.74900682	77.3355331	86.00202895	70.89139318	84.08057021	75.67170218
ENSMUSG00000014444	PIEZO1	78.65688991	86.08302377	72.87408088	71.61400054	90.03620742	51.48667782	68.56368314	67.02412869	64.94030757	58.87590281	68.31546329	66.40577946
ENSMUSG00000014470	RNF166	8.739654434	72.83948165	43.72444853	47.74266702	70.7427344	25.74333891	43.51156814	60.57950093	64.94030757	48.06196148	56.05371347	57.13985675
ENSMUSG00000014496	ANKRD28	52.4379266	33.10885529	7.287408088	29.83916689	25.72463069	19.30750418	21.09651789	33.51206434	17.55143448	21.62788267	28.02685674	21.62048634
ENSMUSG00000014498	ANKRD52	17.47930887	0	14.57481618	5.967833378	12.86231535	25.74333891	7.911194208	7.73355331	14.04114758	13.21703941	7.006714184	9.265922716
ENSMUSG00000014503	PKD2L2	0	6.621771059	7.287408088	29.83916689	12.86231535	32.17917364	3.955597104	6.444627758	1.755143448	6.007745185	3.503357092	3.088640905
ENSMUSG00000014504	SRP19	332.1068685	238.3837581	269.6340992	220.809835	205.7970455	321.7917364	410.0635664	457.5685708	477.3990178	410.9297707	430.9129223	416.9665222
ENSMUSG00000014547	WDFY2	26.2189633	39.73062635	21.86222426	47.74266702	19.29347302	12.87166946	27.68917973	23.20065993	21.06172137	38.44956918	26.27517819	18.53184543
ENSMUSG00000014550	RBSN	26.2189633	39.73062635	21.86222426	29.83916689	12.86231535	32.17917364	29.0077121	27.06743658	26.32715172	27.63562785	38.53692801	30.88640905
ENSMUSG00000014551	MRPS25	87.39654434	52.97416847	116.5985294	107.4210008	96.46736509	102.9733556	90.97873339	114.7143741	86.00202895	111.7440604	138.3826051	95.74786807

ENSMUSG00000014554	DGUOK	34.95861774	33.10885529	14.57481618	5.967833378	19.29347302	12.87166946	9.229726576	16.75603217	26.32715172	19.22478459	12.26174982	21.62048634
ENSMUSG00000014592	CAMTA1	34.95861774	13.24354212	29.14963235	17.90350013	45.01810371	12.87166946	42.19303578	28.35636214	29.83743861	19.22478459	33.28189237	24.70912724
ENSMUSG00000014601	STRIP1	17.47930887	13.24354212	43.72444853	23.87133351	19.29347302	38.61500837	19.77798552	19.33388327	26.32715172	19.22478459	26.27517819	35.51937041
ENSMUSG00000014606	SLC25A11	34.95861774	26.48708424	51.01185661	47.74266702	51.44926138	51.48667782	40.87450341	48.97917096	35.10286896	38.44956918	45.5436422	41.69665222
ENSMUSG00000014609	CHRNE	0	0	0	0	0	0	2.637064736	0	3.510286896	1.201549037	0	1.544320453
ENSMUSG00000014633	CMC2	43.69827217	26.48708424	80.16148896	47.74266702	32.15578836	51.48667782	35.60037394	56.71272427	38.61315585	42.0542163	29.77853528	29.3420886
ENSMUSG00000014668	CHFR	34.95861774	33.10885529	65.58667279	89.51750067	51.44926138	38.61500837	67.24515077	54.13487317	54.40944688	60.07745185	64.8121062	50.96257494
ENSMUSG00000014747	ANKRD53	0	0	0	0	0	0	1.318532368	0	0	0	0	0
ENSMUSG00000014748	TEX261	69.91723547	66.21771059	94.73630514	59.67833378	102.8985228	102.9733556	64.60808603	52.84594762	49.14401654	61.27900089	61.30874911	54.05121584
ENSMUSG00000014763	FAM120B	69.91723547	86.08302377	36.43704044	83.54966729	83.60504975	96.53752092	64.60808603	79.9133842	66.69545102	64.883648	63.06042765	60.22849765
ENSMUSG00000014767	TBP	87.39654434	72.83948165	58.2992647	35.80700027	83.60504975	115.8450251	106.8011218	85.06908641	100.0431765	104.5347662	91.08728439	86.48194535
ENSMUSG00000014769	PSMB1	541.8585749	629.0682506	670.4415441	560.9763375	681.7027133	707.9418201	725.1928024	817.1787998	789.8145515	701.7046376	739.2083464	812.3125581
ENSMUSG00000014771	PDCD2	78.65688991	92.70479482	109.3111213	113.3888342	102.8985228	83.66585146	105.4825894	112.136523	122.8600414	102.1316681	129.6242124	92.65922716
ENSMUSG00000014773	DLL1	61.17758104	39.73062635	58.2992647	59.67833378	38.58694604	96.53752092	46.14863288	45.11239431	33.34772551	56.47280474	33.28189237	49.41825449
ENSMUSG00000014776	NOL3	0	0	0	5.967833378	0	0	1.318532368	0	0	1.201549037	3.503357092	1.544320453
ENSMUSG00000014778	FHOD1	0	13.24354212	7.287408088	11.93566676	12.86231535	25.74333891	13.18532368	12.88925552	14.04114758	10.81394133	8.75839273	13.89888407
ENSMUSG00000014782	PLEKHG4	0	6.621771059	0	0	0	0	0	0	0	0	0	0
ENSMUSG00000014786	SLC9A5	8.739654434	6.621771059	14.57481618	5.967833378	19.29347302	0	15.82238842	18.04495772	19.30657793	21.62788267	14.01342837	20.07616588
ENSMUSG00000014791	ELMO3	0	6.621771059	0	0	6.431157673	0	3.955597104	3.866776655	5.265430344	1.201549037	1.751678546	3.088640905
ENSMUSG00000014813	STC1	26.2189633	0	14.57481618	5.967833378	6.431157673	6.435834728	0	0	0	0	0	0
ENSMUSG00000014837	4931428F04R	0	0	0	0	0	0	10.54825894	3.866776655	7.020573791	3.604647111	7.006714184	1.544320453
ENSMUSG00000014846	TPPP3	0	0	0	0	0	6.435834728	1.318532368	1.288925552	0	1.201549037	1.751678546	3.088640905
ENSMUSG00000014850	MSH3	61.17758104	66.21771059	131.1733456	89.51750067	70.7427344	90.10168619	72.51928024	79.9133842	56.16459033	75.69758933	73.57049893	55.5955363
ENSMUSG00000014852	ADAMTS13	0	0	0	0	0	6.435834728	1.318532368	1.288925552	0	1.201549037	1.751678546	0
ENSMUSG00000014856	TMEM208	69.91723547	33.10885529	36.43704044	35.80700027	51.44926138	32.17917364	71.20074787	68.31305424	64.94030757	66.08519704	71.81882038	71.03874082
ENSMUSG00000014859	E2F4	69.91723547	59.59593953	72.87408088	41.77483365	77.17389207	102.9733556	89.66020102	83.78016086	89.51231584	74.4960403	94.59064148	84.9376249
ENSMUSG00000014867	SURF4	174.7930887	192.0313607	145.7481618	137.2601677	167.2100995	173.7675376	204.372517	190.7609816	215.8826441	201.8602382	206.6980684	234.7367088
ENSMUSG00000014873	SURF2	87.39654434	79.46125271	131.1733456	71.61400054	64.31157673	64.35834728	80.43047445	76.04660755	93.02260274	90.11617778	115.610784	81.84898399
ENSMUSG00000014907	NAF1	69.91723547	119.1918791	72.87408088	71.61400054	115.7608381	70.79418201	108.1196542	139.2039596	103.5534634	128.565747	122.6174982	105.0137908
ENSMUSG00000014956	PPP1CB	235.9706697	192.0313607	204.0474265	208.8741682	205.7970455	199.5108766	373.1446601	302.8975046	312.4155337	342.4414756	297.7853528	298.0538474
ENSMUSG00000014959	GORASP2	218.4913609	158.9225054	247.771875	256.6168353	244.3839916	193.0750418	176.6833373	154.6710662	236.9443655	171.8215123	208.449747	228.559427
ENSMUSG00000014980	TSEN15	34.95861774	13.24354212	36.43704044	11.93566676	57.88041905	19.30750418	30.32624446	25.77851103	35.10286896	28.83717689	42.0402851	21.62048634
ENSMUSG00000015002	EFR3A	131.0948165	66.21771059	189.4726103	214.8420016	154.3477841	186.6392071	90.97873339	72.17983089	77.22631171	88.91462874	98.09399857	95.74786807
ENSMUSG00000015013	TRAPPC2L	8.739654434	19.86531318	14.57481618	35.80700027	12.86231535	19.30750418	26.37064736	19.33388327	28.08229517	24.03098074	14.01342837	26.2534477
ENSMUSG00000015016	ACSF3	17.47930887	0	14.57481618	5.967833378	6.431157673	6.435834728	3.955597104	2.577851103	3.510286896	3.604647111	1.751678546	1.544320453
ENSMUSG00000015023	DDX19A	61.17758104	92.70479482	65.58667279	41.77483365	25.72463069	109.4091904	81.74900682	59.29057538	61.43002068	87.7130797	82.32889166	57.13985675
ENSMUSG00000015027	GALNS	0	6.621771059	0	11.93566676	0	0	3.955597104	10.31140441	3.510286896	3.604647111	1.751678546	4.632961358
ENSMUSG00000015083	C8G	0	0	0	0	0	0	2.637064736	0	1.755143448	0	0	0
ENSMUSG00000015087	RABL6	393.2844495	297.9796976	349.7955882	328.2308358	418.0252487	270.3050586	313.8107036	324.809239	312.4155337	285.9686708	345.0806736	299.5981678
ENSMUSG00000015090	PTGDS	0	0	0	0	0	0	0	0	0	1.201549037	0	0
ENSMUSG00000015092	EDF1	270.9292875	211.8966739	240.4844669	274.5203354	270.1086223	238.1258849	303.2624446	373.78841	349.2735461	320.8135929	322.3088525	352.1050632
ENSMUSG00000015095	FBXW5	26.2189633	19.86531318	29.14963235	11.93566676	32.15578836	6.435834728	13.18532368	21.91173438	22.81686482	24.03098074	17.51678546	16.98752498
ENSMUSG00000015112	SLC25A13	61.17758104	46.35239741	58.2992647	35.80700027	45.01810371	25.74333891	55.37835946	39.9566921	54.40944688	63.68209896	43.79196365	54.05121584
ENSMUSG00000015120	UBE2I	52.4379266	6.621771059	29.14963235	11.93566676	25.72463069	45.05084309	27.68917973	19.33388327	29.83743861	19.22478459	26.27517819	12.35456362
ENSMUSG00000015126	TSR3	87.39654434	72.83948165	102.0237132	35.80700027	57.88041905	45.05084309	56.69689182	54.13487317	66.69545102	46.86041244	52.55035638	66.40577946

ENSMUSG00000015127	UNKL	43.69827217	52.97416847	58.2992647	47.74266702	45.01810371	38.61500837	31.64477683	43.82346876	35.10286896	34.84492207	52.55035638	40.15233177
ENSMUSG00000015143	ACTN1	17.47930887	6.621771059	7.287408088	0	0	6.435834728	0	0	3.510286896	1.201549037	0	3.088640905
ENSMUSG00000015149	SIRT2	17.47930887	19.86531318	43.72444853	35.80700027	19.29347302	6.435834728	15.82238842	23.20065993	31.59258206	24.03098074	36.78524947	23.16480679
ENSMUSG00000015165	HNRNPL	4046.460003	3648.595853	4124.672978	3544.893027	3620.74177	3758.527481	2928.460389	2940.039183	3161.01335	2800.810805	2984.860242	3218.363823
ENSMUSG00000015176	NOLC1	253.4499786	403.9280346	473.6815257	483.3945036	488.7679831	366.8425795	522.1388177	528.4594762	519.5224606	527.4800273	518.4968496	528.1575948
ENSMUSG00000015189	CASD1	17.47930887	19.86531318	36.43704044	17.90350013	25.72463069	45.05084309	22.41505026	28.35636214	15.79629103	27.63562785	17.51678546	20.07616588
ENSMUSG00000015202	MAGI1	17.47930887	13.24354212	0	5.967833378	6.431157673	0	11.86679131	15.46710662	10.53086069	7.209294222	7.006714184	4.632961358
ENSMUSG00000015214	MTMR1	26.2189633	39.73062635	7.287408088	29.83916689	6.431157673	45.05084309	30.32624446	33.51206434	28.08229517	43.25576533	29.77853528	37.06369086
ENSMUSG00000015217	HMGB3	17.47930887	79.46125271	43.72444853	29.83916689	45.01810371	45.05084309	48.78569762	48.97917096	52.65430344	40.85266726	57.80539202	44.78529313
ENSMUSG00000015222	MAP2	26.2189633	13.24354212	21.86222426	17.90350013	6.431157673	6.435834728	22.41505026	30.93421324	8.775717239	21.62788267	22.7718211	21.62048634
ENSMUSG00000015243	ABCA1	0	0	0	0	0	0	0	0	0	0	1.751678546	0
ENSMUSG00000015247	NIPSNAP3B	0	6.621771059	0	0	0	0	0	0	0	0	0	0
ENSMUSG00000015289	LAGE3	0	13.24354212	14.57481618	23.87133351	0	19.30750418	22.41505026	28.35636214	21.06172137	25.23252978	22.7718211	24.70912724
ENSMUSG00000015290	UBL4A	69.91723547	72.83948165	80.16148896	83.54966729	70.7427344	102.9733556	83.06753918	97.95834193	82.49174205	85.30998163	89.33560584	108.1024317
ENSMUSG00000015291	GDI1	52.4379266	59.59593953	72.87408088	59.67833378	32.15578836	90.10168619	75.15634498	76.04660755	71.96088136	61.27900089	78.82553457	63.31713856
ENSMUSG00000015312	GADD45B	8.739654434	13.24354212	21.86222426	11.93566676	12.86231535	6.435834728	17.14092078	12.88925552	26.32715172	9.612392296	15.76510691	15.44320453
ENSMUSG00000015316	SLAMF1	0	0	0	0	0	0	1.318532368	0	0	0	0	0
ENSMUSG00000015335	ZDHHC12	52.4379266	13.24354212	36.43704044	41.77483365	45.01810371	32.17917364	40.87450341	32.22313879	29.83743861	43.25576533	36.78524947	38.60801132
ENSMUSG00000015337	ENDOG	8.739654434	6.621771059	14.57481618	5.967833378	32.15578836	38.61500837	15.82238842	20.62280883	28.08229517	22.8294317	21.02014255	21.62048634
ENSMUSG00000015340	CYBB	0	6.621771059	0	0	0	0	0	0	0	0	1.751678546	0
ENSMUSG00000015341	GOLGA7	17.47930887	6.621771059	7.287408088	23.87133351	12.86231535	12.87166946	19.77798552	11.60032996	15.79629103	20.42633363	21.02014255	23.16480679
ENSMUSG00000015354	PCOLCE2	0	6.621771059	0	0	0	6.435834728	2.637064736	2.577851103	1.755143448	6.007745185	1.751678546	3.088640905
ENSMUSG00000015355	CD48	0	0	0	0	6.431157673	0	0	0	0	0	0	0
ENSMUSG00000015357	CLPX	26.2189633	33.10885529	51.01185661	41.77483365	19.29347302	19.30750418	34.28184157	41.24561765	43.8785862	64.883648	40.28860656	44.78529313
ENSMUSG00000015363	TRABD	96.13619877	86.08302377	43.72444853	35.80700027	83.60504975	77.23001673	52.74129472	45.11239431	49.14401654	48.06196148	40.28860656	55.5955363
ENSMUSG00000015377	DENND6B	43.69827217	26.48708424	14.57481618	11.93566676	19.29347302	32.17917364	27.68917973	23.20065993	21.06172137	26.43407881	26.27517819	37.06369086
ENSMUSG00000015451	C4A	0	0	0	0	0	0	0	0	0	1.755143448	0	0
ENSMUSG00000015452	AGER	0	6.621771059	0	5.967833378	0	0	3.955597104	1.288925552	1.755143448	2.403098074	0	1.544320453
ENSMUSG00000015461	ATF6B	34.95861774	39.73062635	36.43704044	59.67833378	64.31157673	6.435834728	44.83010051	32.22313879	47.38887309	50.46505955	47.29532074	32.43072951
ENSMUSG00000015467	EGFL8	288.4085963	218.5184449	218.6222426	214.8420016	167.2100995	167.3317029	100.20846	76.04660755	103.5534634	82.90688355	101.5973557	95.74786807
ENSMUSG00000015468	NOTCH4	0	0	0	0	0	0	0	0	1.755143448	1.201549037	0	0
ENSMUSG00000015474	PPT2	8.739654434	0	0	5.967833378	0	0	0	0	0	0	0	0
ENSMUSG00000015478	RNF5	61.17758104	72.83948165	51.01185661	65.64616716	70.7427344	57.92251255	56.69689182	60.57950093	61.43002068	56.47280474	64.8121062	83.39330444
ENSMUSG00000015484	FAM163A	0	0	0	0	12.86231535	6.435834728	3.955597104	1.288925552	0	1.201549037	3.503357092	4.632961358
ENSMUSG00000015488	CACFD1	26.2189633	26.48708424	65.58667279	17.90350013	32.15578836	12.87166946	31.64477683	29.64528769	40.3682993	21.62788267	26.27517819	29.3420886
ENSMUSG00000015501	HIVEP2	17.47930887	26.48708424	7.287408088	65.64616716	19.29347302	38.61500837	13.18532368	12.88925552	24.57200827	16.82168652	15.76510691	21.62048634
ENSMUSG00000015522	ARNT	61.17758104	39.73062635	72.87408088	71.61400054	83.60504975	64.35834728	89.66020102	76.04660755	70.20573791	72.09294222	49.04699929	100.3808294
ENSMUSG00000015533	ITGA2	43.69827217	86.08302377	36.43704044	71.61400054	32.15578836	51.48667782	63.28955366	54.13487317	38.61315585	42.0542163	45.5436422	49.41825449
ENSMUSG00000015536	MOCS2	26.2189633	33.10885529	58.2992647	29.83916689	45.01810371	51.48667782	54.05982709	56.71272427	28.08229517	51.66660859	54.30203492	57.13985675
ENSMUSG00000015542	NAT9	0	0	0	0	6.431157673	6.435834728	2.637064736	2.577851103	5.265430344	1.201549037	0	1.544320453
ENSMUSG00000015575	ATP6V0E	87.39654434	79.46125271	72.87408088	113.3888342	32.15578836	51.48667782	83.06753918	92.80263972	117.594611	85.30998163	106.8523913	84.9376249
ENSMUSG00000015597	ZFP318	43.69827217	119.1918791	87.44889705	77.58183392	90.03620742	90.10168619	80.43047445	83.78016086	89.51231584	87.7130797	105.1007128	81.84898399
ENSMUSG00000015605	SRF	26.2189633	46.35239741	0	17.90350013	12.86231535	19.30750418	21.09651789	28.35636214	28.08229517	16.82168652	21.02014255	29.3420886
ENSMUSG00000015619	GATA3	882.7050978	847.5866955	801.6148896	829.5288396	958.2424932	695.0701506	615.7546159	555.5269128	610.7899199	589.9605772	548.2753849	619.2725015
ENSMUSG00000015656	HSPA8	1083.71715	874.0737798	1027.52454	1074.210008	1022.55407	1068.348565	864.9572334	996.3394514	961.8186094	874.7276989	952.913129	962.111642

ENSMUSG00000015659	SERAC1	0	6.621771059	0	0	0	0	2.637064736	0	1.755143448	2.403098074	1.751678546	3.088640905
ENSMUSG00000015668	PDZD11	26.2189633	0	7.287408088	11.93566676	25.72463069	25.74333891	30.32624446	37.378841	28.08229517	26.43407881	22.7718211	27.79776815
ENSMUSG00000015671	PSMA2	262.189633	132.4354212	153.0355698	179.0350013	250.8151492	154.4600335	288.7585886	279.6968447	280.8229517	263.1392391	313.5504597	290.3322451
ENSMUSG00000015672	MRPL32	69.91723547	99.32656588	80.16148896	101.4531674	96.46736509	102.9733556	142.4014957	121.1590019	136.9011889	126.1626489	117.3624626	129.722918
ENSMUSG00000015697	SETDB1	78.65688991	66.21771059	65.58667279	95.48533405	64.31157673	64.35834728	83.06753918	90.22478862	112.3291807	99.72857007	113.8591055	100.3808294
ENSMUSG00000015702	ANXA9	0	0	0	0	12.86231535	6.435834728	1.318532368	3.866776655	0	1.201549037	0	3.088640905
ENSMUSG00000015709	ARNT2	0	0	7.287408088	0	0	12.87166946	3.955597104	3.866776655	5.265430344	4.806196148	7.006714184	4.632961358
ENSMUSG00000015711	PRUNE1	0	6.621771059	7.287408088	0	0	0	5.274129472	9.022478862	10.53086069	2.403098074	0	4.632961358
ENSMUSG00000015714	CERS2	87.39654434	172.1660475	65.58667279	113.3888342	147.9166265	96.53752092	100.20846	112.136523	133.390902	115.3487076	105.1007128	125.0899567
ENSMUSG00000015733	CAPZA2	61.17758104	86.08302377	58.2992647	113.3888342	96.46736509	64.35834728	119.9864455	134.0482574	140.4114758	121.3564527	138.3826051	125.0899567
ENSMUSG00000015745	PLEKHO1	17.47930887	0	36.43704044	0	19.29347302	38.61500837	5.274129472	14.17818107	7.020573791	7.209294222	3.503357092	6.177281811
ENSMUSG00000015747	VPS45	26.2189633	26.48708424	14.57481618	11.93566676	12.86231535	25.74333891	15.82238842	15.46710662	14.04114758	8.410843259	14.01342837	18.53184543
ENSMUSG00000015748	PRPF3	52.4379266	92.70479482	72.87408088	65.64616716	45.01810371	90.10168619	90.97873339	74.757682	50.89915999	88.91462874	82.32889166	100.3808294
ENSMUSG00000015749	ANP32E	235.9706697	271.4926134	233.1970588	286.4560021	231.5216762	205.9467113	303.2624446	304.1864302	263.2715172	296.7826121	303.0403885	282.6106428
ENSMUSG00000015750	APH1A	8.739654434	6.621771059	14.57481618	5.967833378	0	19.30750418	6.59266184	7.73355331	10.53086069	8.410843259	10.51007128	6.177281811
ENSMUSG00000015755	TAB2	139.8344709	125.8136501	102.0237132	214.8420016	115.7608381	180.2033724	137.1273663	167.5603217	138.6563324	132.1703941	140.1342837	120.4569953
ENSMUSG00000015757	PPIL4	131.0948165	185.4095896	174.8977941	149.1958345	180.0724148	173.7675376	180.6389344	198.494535	187.8003489	191.0462969	180.4228902	185.3184543
ENSMUSG00000015759	CNIH1	104.8758532	66.21771059	58.2992647	83.54966729	64.31157673	51.48667782	69.8822155	82.49123531	82.49174205	66.08519704	68.31546329	81.84898399
ENSMUSG00000015776	MED22	34.95861774	26.48708424	36.43704044	41.77483365	32.15578836	32.17917364	46.14863288	45.11239431	49.14401654	43.25576533	38.53692801	49.41825449
ENSMUSG00000015790	SURF1	52.4379266	66.21771059	72.87408088	59.67833378	64.31157673	57.92251255	48.78569762	76.04660755	66.69545102	48.06196148	68.31546329	91.11490671
ENSMUSG00000015804	MED28	52.4379266	26.48708424	14.57481618	35.80700027	12.86231535	38.61500837	44.83010051	43.82346876	28.08229517	33.64337304	42.0402851	49.41825449
ENSMUSG00000015806	QDPR	69.91723547	79.46125271	36.43704044	11.93566676	70.7427344	57.92251255	61.9710213	41.24561765	64.94030757	57.67435378	63.06042765	58.6841772
ENSMUSG00000015812	GNRH1	0	0	0	0	0	0	0	1.288925552	1.755143448	3.604647111	0	1.544320453
ENSMUSG00000015837	SQSTM1	87.39654434	86.08302377	87.44889705	53.7105004	32.15578836	64.35834728	60.65248893	76.04660755	93.02260274	64.883648	75.32217748	71.03874082
ENSMUSG00000015839	NFE2L2	61.17758104	33.10885529	131.1733456	65.64616716	109.3296804	96.53752092	117.3493808	132.7593318	133.390902	121.3564527	108.6040698	105.0137908
ENSMUSG00000015846	RXRA	0	6.621771059	0	0	6.431157673	0	0	0	0	0	0	0
ENSMUSG00000015850	ADAMTSL4	0	0	0	0	0	0	0	0	0	0	0	3.088640905
ENSMUSG00000015869	PRPSAP1	34.95861774	66.21771059	21.86222426	41.77483365	19.29347302	64.35834728	34.28184157	19.33388327	26.32715172	25.23252978	24.52349964	23.16480679
ENSMUSG00000015879	FAM184B	0	0	0	0	0	0	1.318532368	0	0	0	1.751678546	0
ENSMUSG00000015880	NCAPG	87.39654434	132.4354212	153.0355698	167.0993346	147.9166265	109.4091904	147.6756252	184.3163539	124.6151848	174.2246104	162.9061048	122.0013158
ENSMUSG00000015882	LCORL	78.65688991	86.08302377	58.2992647	77.58183392	102.8985228	51.48667782	94.9343305	96.66941637	115.8394676	94.92237392	70.06714184	95.74786807
ENSMUSG00000015889	LTA4H	43.69827217	52.97416847	80.16148896	113.3888342	90.03620742	96.53752092	88.34166866	88.93586306	84.2468855	82.90688355	92.83896294	74.12738173
ENSMUSG00000015890	AMDHD1	0	0	0	0	0	0	1.318532368	0	1.755143448	0	0	0
ENSMUSG00000015932	DSTN	52.4379266	26.48708424	43.72444853	29.83916689	38.58694604	12.87166946	40.87450341	28.35636214	24.57200827	25.23252978	29.77853528	23.16480679
ENSMUSG00000015937	H2AFY	830.2671712	821.0996113	903.6386029	859.3680064	874.6374435	669.3268117	722.5557377	768.1996288	716.0985267	677.6736569	742.7117035	718.1090105
ENSMUSG00000015942	GTF2IRD2	8.739654434	13.24354212	21.86222426	17.90350013	12.86231535	6.435834728	10.54825894	9.022478862	7.020573791	10.81394133	10.51007128	10.81024317
ENSMUSG00000015943	BOLA1	87.39654434	26.48708424	58.2992647	89.51750067	102.8985228	32.17917364	69.8822155	77.3355331	82.49174205	64.883648	82.32889166	77.21602263
ENSMUSG00000015947	FCGR1	34.95861774	46.35239741	7.287408088	23.87133351	38.58694604	19.30750418	17.14092078	21.91173438	17.55143448	15.62013748	26.27517819	23.16480679
ENSMUSG00000015950	NCF1	0	0	0	0	0	6.435834728	0	0	0	0	3.503357092	0
ENSMUSG00000015957	WNT11	0	0	0	0	0	0	1.318532368	0	0	0	1.751678546	0
ENSMUSG00000015961	ADSS	104.8758532	112.570108	131.1733456	143.2280011	102.8985228	96.53752092	145.0385605	171.4270984	147.4320496	164.6122181	140.1342837	145.1661226
ENSMUSG00000015968	CACNA1D	8.739654434	26.48708424	29.14963235	23.87133351	19.29347302	12.87166946	21.09651789	41.24561765	40.3682993	39.65111822	21.02014255	30.88640905
ENSMUSG00000015970	CHDH	8.739654434	13.24354212	14.57481618	17.90350013	6.431157673	12.87166946	6.59266184	9.022478862	7.020573791	8.410843259	8.75839273	4.632961358
ENSMUSG00000015971	ACTR8	34.95861774	39.73062635	36.43704044	29.83916689	19.29347302	19.30750418	50.10422998	41.24561765	38.61315585	26.43407881	38.53692801	54.05121584
ENSMUSG00000015994	FNTA	26.2189633	46.35239741	21.86222426	29.83916689	45.01810371	38.61500837	26.37064736	34.80098989	38.61315585	43.25576533	40.28860656	35.51937041

ENSMUSG00000016018	SKIV2L2	227.2310153	158.9225054	218.6222426	202.9063349	205.7970455	205.9467113	224.1505026	211.3837905	221.1480744	216.2788267	282.0202459	236.2810293
ENSMUSG00000016087	FLI1	174.7930887	218.5184449	196.7600184	143.2280011	135.0543111	270.3050586	147.6756252	144.3596618	157.9629103	147.7905316	175.1678546	160.6093271
ENSMUSG00000016181	DIEXF	43.69827217	46.35239741	21.86222426	71.61400054	64.31157673	19.30750418	39.55597104	46.40131986	45.63372964	24.03098074	47.29532074	40.15233177
ENSMUSG00000016194	HSD11B1	0	0	7.287408088	0	0	6.435834728	5.274129472	6.444627758	3.510286896	3.604647111	5.255035638	3.088640905
ENSMUSG00000016206	H2-M3	69.91723547	66.21771059	87.44889705	95.48533405	115.7608381	70.79418201	87.02313629	82.49123531	101.79832	105.7363153	87.5839273	89.57058625
ENSMUSG00000016252	ATP5E	174.7930887	238.3837581	276.9215073	232.7455017	115.7608381	154.4600335	258.4323441	226.8508971	282.5780951	207.8679834	225.9665324	244.0026315
ENSMUSG00000016253	NELFCD	17.47930887	26.48708424	7.287408088	5.967833378	6.431157673	19.30750418	25.05211499	6.444627758	12.28600414	28.83717689	29.77853528	20.07616588
ENSMUSG00000016256	CTSZ	8.739654434	6.621771059	14.57481618	0	38.58694604	12.87166946	15.82238842	15.46710662	15.79629103	14.41858844	12.26174982	20.07616588
ENSMUSG00000016257	PRELID3B	69.91723547	86.08302377	80.16148896	107.4210008	83.60504975	109.4091904	175.3648049	132.7593318	152.69748	187.4416498	173.4161761	182.2298134
ENSMUSG00000016308	UBE2A	96.13619877	26.48708424	29.14963235	59.67833378	70.7427344	57.92251255	65.9266184	73.46875644	68.45059447	81.70533452	73.57049893	67.95009992
ENSMUSG00000016319	SLC25A5	201.012052	211.8966739	138.4607537	238.7133351	244.3839916	218.8183807	315.129236	350.5877501	344.0081158	302.7903573	292.5303172	372.1812291
ENSMUSG00000016344	PPDPF	0	0	14.57481618	17.90350013	25.72463069	0	10.54825894	16.75603217	12.28600414	15.62013748	22.7718211	18.53184543
ENSMUSG00000016356	COL20A1	0	6.621771059	0	5.967833378	0	0	2.637064736	1.288925552	10.53086069	1.201549037	8.75839273	4.632961358
ENSMUSG00000016386	MPPED2	0	0	0	11.93566676	6.431157673	0	1.318532368	1.288925552	5.265430344	2.403098074	0	3.088640905
ENSMUSG00000016409	NKAP	87.39654434	158.9225054	65.58667279	89.51750067	83.60504975	38.61500837	58.01542419	63.15735203	50.89915999	58.87590281	50.79867783	52.50689539
ENSMUSG00000016427	NDUFA1	279.6689419	251.6273002	189.4726103	190.9706681	173.6412572	167.3317029	272.9362002	315.7867602	326.4566813	230.6974151	306.5437455	318.1300133
ENSMUSG00000016477	E2F3	87.39654434	172.1660475	131.1733456	101.4531674	128.6231535	173.7675376	109.4381865	125.0257785	136.9011889	116.5502566	147.1409979	109.6467521
ENSMUSG00000016481	CR1L	52.4379266	26.48708424	43.72444853	41.77483365	32.15578836	25.74333891	18.45945315	30.93421324	31.59258206	36.04647111	26.27517819	33.97504996
ENSMUSG00000016487	PPFIBP1	17.47930887	6.621771059	21.86222426	23.87133351	12.86231535	6.435834728	19.77798552	18.04495772	17.55143448	24.03098074	38.53692801	13.89888407
ENSMUSG00000016493	CD46	8.739654434	6.621771059	14.57481618	5.967833378	0	12.87166946	7.911194208	3.866776655	3.510286896	6.007745185	8.75839273	13.89888407
ENSMUSG00000016495	PLGRKT	17.47930887	6.621771059	43.72444853	11.93566676	19.29347302	32.17917364	34.28184157	42.5345432	40.3682993	40.85266726	49.04699929	46.32961358
ENSMUSG00000016496	CD274	0	0	14.57481618	5.967833378	6.431157673	6.435834728	2.637064736	3.866776655	7.020573791	4.806196148	1.751678546	1.544320453
ENSMUSG00000016498	PDCD1LG2	0	0	14.57481618	0	19.29347302	6.435834728	7.911194208	6.444627758	8.775717239	4.806196148	5.255035638	10.81024317
ENSMUSG00000016503	GTF3A	69.91723547	26.48708424	36.43704044	41.77483365	57.88041905	57.92251255	61.9710213	61.86842648	77.22631171	61.27900089	59.55707056	60.22849765
ENSMUSG00000016510	MTIF3	8.739654434	13.24354212	14.57481618	11.93566676	12.86231535	19.30750418	17.14092078	23.20065993	24.57200827	26.43407881	17.51678546	26.2534477
ENSMUSG00000016520	LNX2	8.739654434	6.621771059	0	0	12.86231535	6.435834728	13.18532368	14.17818107	14.04114758	10.81394133	5.255035638	7.721602263
ENSMUSG00000016526	DYRK3	0	0	29.14963235	0	6.431157673	6.435834728	11.86679131	5.155702207	7.020573791	3.604647111	5.255035638	7.721602263
ENSMUSG00000016528	MAPKAPK2	104.8758532	52.97416847	65.58667279	65.64616716	77.17389207	109.4091904	89.66020102	78.62445865	100.0431765	78.10068741	105.1007128	115.824034
ENSMUSG00000016534	LAMP2	34.95861774	19.86531318	36.43704044	5.967833378	25.72463069	25.74333891	42.19303578	32.22313879	36.85801241	28.83717689	49.04699929	27.79776815
ENSMUSG00000016541	ATXN10	34.95861774	46.35239741	87.44889705	53.7105004	45.01810371	64.35834728	59.33395656	83.78016086	71.96088136	61.27900089	71.81882038	72.58306128
ENSMUSG00000016552	FOXRED2	0	0	0	0	6.431157673	0	2.637064736	9.022478862	5.265430344	6.007745185	10.51007128	4.632961358
ENSMUSG00000016554	EIF3D	104.8758532	178.7878186	189.4726103	125.3245009	212.2282032	128.7166946	187.2315963	194.6277583	222.9032179	174.2246104	185.6779259	205.3946202
ENSMUSG00000016559	H3F3B	349.5861774	384.0627214	371.6578125	334.1986692	302.2644106	450.5084309	545.8724003	533.6151784	577.4421944	538.2939686	579.8055987	542.0564789
ENSMUSG00000016619	NUP50	34.95861774	33.10885529	87.44889705	77.58183392	77.17389207	64.35834728	76.47487734	69.60197979	107.0637503	86.51153067	92.83896294	101.9251499
ENSMUSG00000016624	PHF21B	8.739654434	13.24354212	43.72444853	23.87133351	32.15578836	19.30750418	14.50385605	15.46710662	17.55143448	21.62788267	14.01342837	21.62048634
ENSMUSG00000016626	NLRP14	0	0	0	0	0	0	1.318532368	0	0	2.403098074	0	0
ENSMUSG00000016637	IFT27	61.17758104	26.48708424	58.2992647	53.7105004	51.44926138	25.74333891	59.33395656	81.20230975	75.47116826	69.68984415	66.56378475	74.12738173
ENSMUSG00000016664	PACSIN2	17.47930887	19.86531318	36.43704044	23.87133351	38.58694604	25.74333891	40.87450341	28.35636214	43.8785862	22.8294317	31.53021383	41.69665222
ENSMUSG00000016756	CMAH	690.4327003	523.1199137	386.2326286	555.0085042	456.6121948	547.0459519	548.5094651	538.7708806	568.6664771	526.2784782	506.2350998	579.1201698
ENSMUSG00000016757	TTLL12	26.2189633	33.10885529	21.86222426	23.87133351	25.72463069	38.61500837	36.9189063	46.40131986	52.65430344	39.65111822	47.29532074	46.32961358
ENSMUSG00000016831	TOX4	52.4379266	59.59593953	51.01185661	83.54966729	57.88041905	45.05084309	67.24515077	58.00164982	63.18516412	56.47280474	87.5839273	61.77281811
ENSMUSG00000016833	MRPS18C	104.8758532	99.32656588	94.73630514	65.64616716	128.6231535	77.23001673	126.5791073	146.9375129	114.0843241	104.5347662	108.6040698	105.0137908
ENSMUSG00000016918	SULF1	0	6.621771059	0	0	0	0	7.911194208	2.577851103	3.510286896	3.604647111	1.751678546	4.632961358
ENSMUSG00000016921	SRSF6	192.2723975	192.0313607	182.1852022	167.0993346	135.0543111	180.2033724	204.372517	202.3613116	212.3723572	206.6664344	178.6712117	197.6730179
ENSMUSG00000016933	PLCG1	17.47930887	0	21.86222426	23.87133351	32.15578836	32.17917364	47.46716525	42.5345432	56.16459033	40.85266726	35.03357092	35.51937041

ENSMUSG00000016940	KCTD2	0	13.24354212	0	0	6.431157673	12.87166946	5.274129472	6.444627758	10.53086069	4.806196148	7.006714184	7.721602263
ENSMUSG00000016946	KCTD5	34.95861774	52.97416847	58.2992647	65.64616716	32.15578836	51.48667782	64.60808603	45.11239431	54.40944688	58.87590281	61.30874911	47.87393403
ENSMUSG00000016984	ETAA1	17.47930887	39.73062635	65.58667279	53.7105004	38.58694604	77.23001673	67.24515077	74.757682	54.40944688	39.65111822	71.81882038	44.78529313
ENSMUSG00000017009	SDC4	0	0	7.287408088	0	0	6.435834728	0	0	0	0	0	0
ENSMUSG00000017119	NBR1	26.2189633	6.621771059	43.72444853	23.87133351	19.29347302	25.74333891	34.28184157	45.11239431	38.61315585	33.64337304	45.5436422	35.51937041
ENSMUSG00000017132	CYTH1	87.39654434	66.21771059	65.58667279	53.7105004	38.58694604	51.48667782	51.42276235	42.5345432	61.43002068	39.65111822	54.30203492	44.78529313
ENSMUSG00000017146	BRCA1	43.69827217	46.35239741	51.01185661	41.77483365	38.58694604	51.48667782	44.83010051	48.97917096	57.91973378	45.65886341	35.03357092	50.96257494
ENSMUSG00000017167	CNTNAP1	0	0	0	0	0	0	0	0	2.577851103	3.510286896	0	0	0
ENSMUSG00000017176	NT5C3B	157.3137798	125.8136501	145.7481618	113.3888342	102.8985228	173.7675376	93.61579813	94.09156527	117.594611	84.10843259	85.83224875	111.1910726
ENSMUSG00000017188	COA3	26.2189633	6.621771059	58.2992647	17.90350013	38.58694604	25.74333891	43.51156814	32.22313879	36.85801241	38.44956918	40.28860656	32.43072951
ENSMUSG00000017210	MED24	69.91723547	66.21771059	80.16148896	89.51750067	57.88041905	70.79418201	76.47487734	79.9133842	93.02260274	84.10843259	71.81882038	67.95009992
ENSMUSG00000017221	PSMD3	183.5327431	132.4354212	138.4607537	137.2601677	96.46736509	122.2808598	139.764431	172.7160239	138.6563324	127.3641979	159.4027477	169.8752498
ENSMUSG00000017264	EXOSC10	96.13619877	119.1918791	145.7481618	167.0993346	115.7608381	96.53752092	108.1196542	118.5811508	115.8394676	98.52702104	110.3557484	125.0899567
ENSMUSG00000017286	GLOD4	17.47930887	19.86531318	14.57481618	17.90350013	0	12.87166946	36.9189063	30.93421324	15.79629103	39.65111822	28.02685674	29.3420886
ENSMUSG00000017288	VPS53	26.2189633	26.48708424	51.01185661	17.90350013	51.44926138	64.35834728	42.19303578	48.97917096	31.59258206	34.84492207	36.78524947	40.15233177
ENSMUSG00000017291	TAOK1	131.0948165	139.0571922	167.610386	101.4531674	70.7427344	90.10168619	88.34166866	85.06908641	89.51231584	84.10843259	99.84567712	105.0137908
ENSMUSG00000017299	DNTTIP1	8.739654434	0	14.57481618	5.967833378	6.431157673	0	10.54825894	12.88925552	10.53086069	12.01549037	12.26174982	10.81024317
ENSMUSG00000017300	TNNC2	0	0	0	0	0	0	2.637064736	0	0	1.201549037	0	0
ENSMUSG00000017307	ACOT8	8.739654434	13.24354212	14.57481618	23.87133351	6.431157673	6.435834728	10.54825894	14.17818107	12.28600414	9.612392296	8.75839273	6.177281811
ENSMUSG00000017376	NLK	139.8344709	105.9483369	87.44889705	107.4210008	122.1919958	109.4091904	102.8455247	108.2697463	82.49174205	111.7440604	96.34232003	106.5581112
ENSMUSG00000017386	TRAF4	8.739654434	0	7.287408088	0	0	0	2.637064736	2.577851103	3.510286896	1.201549037	3.503357092	1.544320453
ENSMUSG00000017390	ALDOC	0	0	0	0	0	0	0	0	0	0	0	3.088640905
ENSMUSG00000017404	RPL19	882.7050978	754.8819007	743.3156249	889.2071733	810.3258668	778.7360021	1050.870297	1087.853166	1232.1107	881.9369932	1156.10784	1145.885776
ENSMUSG00000017405	NEK8	0	6.621771059	7.287408088	5.967833378	6.431157673	6.435834728	11.86679131	10.31140441	8.775717239	1.201549037	3.503357092	7.721602263
ENSMUSG00000017412	CACNB4	0	0	7.287408088	0	0	0	2.637064736	0	0	0	3.503357092	0
ENSMUSG00000017417	PLXDC1	0	6.621771059	0	0	0	0	0	0	0	0	0	0
ENSMUSG00000017418	ARL5B	26.2189633	26.48708424	29.14963235	47.74266702	19.29347302	12.87166946	40.87450341	30.93421324	28.08229517	42.0542163	38.53692801	26.2534477
ENSMUSG00000017421	ZFP207	235.9706697	172.1660475	218.6222426	196.9385015	154.3477841	205.9467113	247.8840852	225.5619715	173.7592013	318.4104948	217.2081397	200.7616588
ENSMUSG00000017428	PSMD11	244.7103242	238.3837581	247.771875	161.1315012	96.46736509	141.588364	129.2161721	130.1814807	121.1048979	133.3719431	131.3758909	135.9001998
ENSMUSG00000017453	PIPOX	0	0	0	0	0	0	0	0	1.755143448	0	1.751678546	1.544320453
ENSMUSG00000017478	ZC3H18	235.9706697	205.2749028	153.0355698	226.7776684	250.8151492	218.8183807	239.972891	215.2505671	208.8620703	203.0617873	220.7114968	259.445836
ENSMUSG00000017485	TOP2B	349.5861774	364.1974082	357.0829963	316.295169	282.9709376	334.6634058	499.7237675	489.7917096	419.479284	551.511008	474.704886	401.5233177
ENSMUSG00000017491	RARB	0	0	0	0	0	0	1.318532368	0	0	1.201549037	0	0
ENSMUSG00000017493	IGFBP4	0	0	0	0	0	0	0	0	1.755143448	0	0	0
ENSMUSG00000017499	CDC6	96.13619877	72.83948165	109.3111213	89.51750067	96.46736509	90.10168619	135.8088339	132.7593318	110.5740372	116.5502566	131.3758909	97.29218852
ENSMUSG00000017548	SUZ12	314.6275596	337.710324	276.9215073	435.6518366	276.5397799	296.0483975	437.7527462	360.8991545	333.4772551	416.9375158	395.8793514	330.4845769
ENSMUSG00000017550	ATAD5	139.8344709	172.1660475	145.7481618	161.1315012	122.1919958	167.3317029	159.5424165	125.0257785	143.9217627	156.2013748	108.6040698	123.5456362
ENSMUSG00000017561	CRLF3	104.8758532	105.9483369	116.5985294	95.48533405	115.7608381	70.79418201	108.1196542	143.0707362	115.8394676	94.92237392	164.6577833	137.4445203
ENSMUSG00000017607	TNS4	0	0	0	5.967833378	0	0	0	0	1.755143448	0	0	0
ENSMUSG00000017615	TNFAIP1	87.39654434	52.97416847	51.01185661	41.77483365	45.01810371	77.23001673	35.60037394	33.51206434	26.32715172	32.441824	19.26846401	38.60801132
ENSMUSG00000017631	ABR	34.95861774	26.48708424	36.43704044	29.83916689	32.15578836	38.61500837	43.51156814	23.20065993	33.34772551	22.8294317	22.7718211	32.43072951
ENSMUSG00000017639	RAB11FIP4	0	6.621771059	0	0	0	0	0	0	0	0	0	0
ENSMUSG00000017664	SLC35C2	26.2189633	26.48708424	7.287408088	17.90350013	25.72463069	19.30750418	15.82238842	15.46710662	14.04114758	12.01549037	15.76510691	16.98752498
ENSMUSG00000017670	ELMO2	26.2189633	6.621771059	29.14963235	17.90350013	12.86231535	12.87166946	21.09651789	19.33388327	21.06172137	8.410843259	21.02014255	21.62048634
ENSMUSG00000017677	WSB1	78.65688991	99.32656588	138.4607537	173.067168	45.01810371	70.79418201	121.3049779	121.1590019	159.7180538	122.5580018	154.147712	115.824034

ENSMUSG00000017679	TTPAL	0	13.24354212	14.57481618	5.967833378	0	6.435834728	10.54825894	2.577851103	12.28600414	4.806196148	5.255035638	13.89888407
ENSMUSG00000017686	RHOT1	69.91723547	79.46125271	14.57481618	23.87133351	32.15578836	83.66585146	56.69689182	63.15735203	66.69545102	73.29449126	56.05371347	46.32961358
ENSMUSG00000017692	RHBDL3	17.47930887	26.48708424	14.57481618	17.90350013	12.86231535	6.435834728	29.0077121	24.48958548	21.06172137	12.01549037	19.26846401	23.16480679
ENSMUSG00000017697	ADA	0	6.621771059	0	5.967833378	0	0	3.955597104	1.288925552	0	0	0	0
ENSMUSG00000017707	SERINC3	148.5741254	165.5442765	174.8977941	185.0028347	147.9166265	148.0241987	213.6022436	217.8284182	187.8003489	234.3020622	201.4430328	188.4070952
ENSMUSG00000017713	THA1	8.739654434	6.621771059	14.57481618	0	0	6.435834728	5.274129472	14.17818107	7.020573791	4.806196148	10.51007128	12.35456362
ENSMUSG00000017715	PGS1	17.47930887	19.86531318	29.14963235	11.93566676	19.29347302	83.66585146	25.05211499	28.35636214	33.34772551	15.62013748	31.53021383	26.2534477
ENSMUSG00000017716	BIRC5	113.6155076	125.8136501	131.1733456	71.61400054	64.31157673	90.10168619	94.9343305	103.1140441	75.47116826	103.3332172	96.34232003	122.0013158
ENSMUSG00000017718	AFMID	0	0	0	0	0	0	0	1.288925552	0	0	1.751678546	0
ENSMUSG00000017721	PIGT	0	13.24354212	7.287408088	17.90350013	25.72463069	12.87166946	10.54825894	3.866776655	5.265430344	4.806196148	14.01342837	7.721602263
ENSMUSG00000017723	WFDC2	0	0	0	0	0	0	0	0	0	2.403098074	0	1.544320453
ENSMUSG00000017724	ETV4	0	0	0	0	0	0	0	1.288925552	0	0	0	1.544320453
ENSMUSG00000017734	DBNDD2	0	0	0	0	0	0	0	3.866776655	1.755143448	0	0	0
ENSMUSG00000017737	MMP9	0	0	7.287408088	5.967833378	0	0	0	0	3.510286896	1.201549037	1.751678546	1.544320453
ENSMUSG00000017747	GHDC	0	13.24354212	0	11.93566676	0	6.435834728	1.318532368	3.866776655	1.755143448	4.806196148	3.503357092	3.088640905
ENSMUSG00000017754	PLTP	8.739654434	0	7.287408088	0	6.431157673	0	1.318532368	3.866776655	5.265430344	4.806196148	1.751678546	4.632961358
ENSMUSG00000017756	SLC12A7	8.739654434	0	14.57481618	17.90350013	12.86231535	12.87166946	18.45945315	15.46710662	17.55143448	13.21703941	17.51678546	23.16480679
ENSMUSG00000017760	CTSA	61.17758104	6.621771059	36.43704044	29.83916689	25.72463069	25.74333891	38.23743867	30.93421324	22.81686482	37.24802015	33.28189237	30.88640905
ENSMUSG00000017764	ZSWIM1	0	0	7.287408088	5.967833378	0	0	1.318532368	3.866776655	0	1.201549037	0	3.088640905
ENSMUSG00000017765	SLC12A4	8.739654434	0	29.14963235	11.93566676	32.15578836	6.435834728	3.955597104	5.155702207	3.510286896	2.403098074	7.006714184	6.177281811
ENSMUSG00000017774	MYO1C	34.95861774	0	14.57481618	11.93566676	6.431157673	0	15.82238842	14.17818107	21.06172137	13.21703941	8.75839273	13.89888407
ENSMUSG00000017776	CRK	148.5741254	86.08302377	72.87408088	65.64616716	45.01810371	45.05084309	100.20846	94.09156527	80.7365986	84.10843259	77.07385602	66.40577946
ENSMUSG00000017778	COX7C	367.0654862	331.0885529	313.3585478	304.3595023	411.5940911	289.6125627	483.9013791	512.9923696	475.6438744	440.9684966	481.7116001	481.8279812
ENSMUSG00000017781	PITPNA	34.95861774	52.97416847	58.2992647	65.64616716	64.31157673	19.30750418	48.78569762	52.84594762	42.12344275	52.86815763	54.30203492	46.32961358
ENSMUSG00000017801	MLX	61.17758104	46.35239741	51.01185661	47.74266702	25.72463069	25.74333891	46.14863288	41.24561765	36.85801241	28.83717689	43.79196365	52.50689539
ENSMUSG00000017802	RETREG3	26.2189633	13.24354212	36.43704044	5.967833378	12.86231535	0	10.54825894	19.33388327	17.55143448	16.82168652	15.76510691	16.98752498
ENSMUSG00000017830	DHX58	0	0	0	5.967833378	0	0	1.318532368	0	3.510286896	1.201549037	1.751678546	0
ENSMUSG00000017831	RAB5A	17.47930887	13.24354212	0	23.87133351	6.431157673	6.435834728	14.50385605	18.04495772	10.53086069	14.41858844	10.51007128	18.53184543
ENSMUSG00000017837	NKIRAS2	17.47930887	19.86531318	21.86222426	17.90350013	6.431157673	12.87166946	11.86679131	5.155702207	22.81686482	16.82168652	17.51678546	13.89888407
ENSMUSG00000017843	PPP2R5C	157.3137798	119.1918791	182.1852022	155.1636678	102.8985228	289.6125627	160.8609489	130.1814807	128.1254717	179.0308065	133.1275695	131.2672385
ENSMUSG00000017858	IFT52	0	6.621771059	14.57481618	5.967833378	0	0	3.955597104	9.022478862	7.020573791	2.403098074	7.006714184	9.265922716
ENSMUSG00000017861	MYBL2	8.739654434	0	7.287408088	0	6.431157673	6.435834728	6.59266184	6.444627758	3.510286896	6.007745185	3.503357092	0
ENSMUSG00000017897	EYA2	26.2189633	26.48708424	21.86222426	47.74266702	19.29347302	12.87166946	13.18532368	32.22313879	12.28600414	26.43407881	31.53021383	32.43072951
ENSMUSG00000017929	B4GALT5	26.2189633	79.46125271	36.43704044	17.90350013	45.01810371	57.92251255	42.19303578	28.35636214	57.91973378	46.86041244	38.53692801	88.0262658
ENSMUSG00000017999	DDX27	227.2310153	158.9225054	182.1852022	244.6811685	102.8985228	186.6392071	167.4536107	216.5394927	205.3517834	197.0540421	204.9463899	200.7616588
ENSMUSG00000018001	CYTH3	0	0	0	0	0	0	0	0	1.755143448	0	0	3.088640905
ENSMUSG00000018008	CYTH4	43.69827217	13.24354212	29.14963235	29.83916689	12.86231535	45.05084309	17.14092078	39.9566921	24.57200827	21.62788267	42.0402851	24.70912724
ENSMUSG00000018012	RAC3	0	6.621771059	7.287408088	23.87133351	6.431157673	12.87166946	6.59266184	3.866776655	5.265430344	4.806196148	5.255035638	3.088640905
ENSMUSG00000018040	RRP7A	26.2189633	33.10885529	43.72444853	23.87133351	12.86231535	19.30750418	26.37064736	45.11239431	43.8785862	24.03098074	24.52349964	35.51937041
ENSMUSG00000018042	CYB5R3	8.739654434	13.24354212	14.57481618	41.77483365	19.29347302	12.87166946	22.41505026	39.9566921	24.57200827	22.8294317	31.53021383	50.96257494
ENSMUSG00000018068	INTS2	69.91723547	72.83948165	87.44889705	71.61400054	70.7427344	51.48667782	58.01542419	100.536193	94.77774619	66.08519704	70.06714184	54.05121584
ENSMUSG00000018076	MED13L	436.9827217	450.280432	546.5556066	387.9091696	456.6121948	469.8159351	305.8995094	340.2763456	319.4361075	373.6817505	308.2954241	369.0925882
ENSMUSG00000018102	HIST1H2BC	157.3137798	139.0571922	138.4607537	131.2923343	109.3296804	193.0750418	296.6697828	295.1639513	277.3126648	285.9686708	322.3088525	290.3322451
ENSMUSG00000018143	MAFK	17.47930887	13.24354212	29.14963235	11.93566676	19.29347302	6.435834728	22.41505026	25.77851103	24.57200827	20.42633363	17.51678546	33.97504996
ENSMUSG00000018160	MED1	131.0948165	112.570108	87.44889705	71.61400054	128.6231535	90.10168619	102.8455247	114.7143741	84.2468855	102.1316681	84.08057021	106.5581112

ENSMUSG00000018166	ERBB3	0	13.24354212	14.57481618	29.83916689	6.431157673	12.87166946	0	0	1.755143448	0	0	0
ENSMUSG00000018167	STARD3	34.95861774	52.97416847	51.01185661	47.74266702	32.15578836	0	17.14092078	29.64528769	43.8785862	19.22478459	36.78524947	35.51937041
ENSMUSG00000018169	MFNG	34.95861774	46.35239741	29.14963235	35.80700027	64.31157673	38.61500837	51.42276235	69.60197979	71.96088136	51.66660859	85.83224875	47.87393403
ENSMUSG00000018171	VMP1	17.47930887	19.86531318	43.72444853	59.67833378	77.17389207	32.17917364	42.19303578	51.55702207	57.91973378	37.24802015	49.04699929	47.87393403
ENSMUSG00000018189	UCHL5	104.8758532	125.8136501	131.1733456	119.3566676	64.31157673	70.79418201	151.6312223	177.8717261	172.0040579	144.1858844	152.3960335	151.3434044
ENSMUSG00000018196	GLRX2	43.69827217	66.21771059	65.58667279	71.61400054	70.7427344	38.61500837	69.8822155	64.44627758	56.16459033	66.08519704	84.08057021	72.58306128
ENSMUSG00000018199	TROVE2	17.47930887	26.48708424	29.14963235	17.90350013	32.15578836	25.74333891	23.73358262	16.75603217	17.55143448	13.21703941	29.77853528	21.62048634
ENSMUSG00000018209	STK4	87.39654434	66.21771059	109.3111213	131.2923343	135.0543111	141.588364	123.9420426	105.6918952	114.0843241	114.1471585	103.3490342	106.5581112
ENSMUSG00000018238	GDF9	0	0	7.287408088	0	0	0	0	0	1.755143448	0	0	0
ENSMUSG00000018239	ZCCHC10	69.91723547	86.08302377	65.58667279	77.58183392	57.88041905	51.48667782	92.29726576	70.89090534	91.26745929	92.51927585	73.57049893	105.0137908
ENSMUSG00000018286	PSMB6	288.4085963	198.6531318	189.4726103	214.8420016	205.7970455	160.8958682	229.424632	264.2297381	250.985513	223.4881209	273.2618532	265.6231179
ENSMUSG00000018287	SPAG7	17.47930887	26.48708424	43.72444853	23.87133351	32.15578836	45.05084309	47.46716525	76.04660755	66.69545102	56.47280474	49.04699929	71.03874082
ENSMUSG00000018293	PFN1	1005.06026	807.8560692	1005.662316	859.3680064	842.4816551	604.9684644	1075.922412	1053.052176	1096.964655	960.0376806	937.1480221	1142.797135
ENSMUSG00000018322	TOMM34	34.95861774	19.86531318	29.14963235	23.87133351	64.31157673	45.05084309	29.0077121	42.5345432	31.59258206	39.65111822	40.28860656	35.51937041
ENSMUSG00000018326	YWHAB	244.7103242	251.6273002	204.0474265	298.3916689	231.5216762	231.6900502	266.3435383	246.1847804	242.2097958	240.3098074	243.4833179	248.6355929
ENSMUSG00000018334	KSR1	0	0	14.57481618	17.90350013	0	12.87166946	15.82238842	9.022478862	10.53086069	20.42633363	8.75839273	18.53184543
ENSMUSG00000018340	ANXA6	96.13619877	119.1918791	80.16148896	179.0350013	96.46736509	96.53752092	71.20074787	83.78016086	78.98145515	81.70533452	92.83896294	100.3808294
ENSMUSG00000018341	IL12RB2	8.739654434	0	0	0	0	0	0	2.577851103	0	2.403098074	1.751678546	0
ENSMUSG00000018347	ZKSCAN6	8.739654434	19.86531318	0	0	6.431157673	6.435834728	2.637064736	3.866776655	5.265430344	7.209294222	8.75839273	10.81024317
ENSMUSG00000018362	KPNA2	0	0	0	5.967833378	0	0	6.59266184	5.155702207	3.510286896	8.410843259	3.503357092	6.177281811
ENSMUSG00000018363	SMURF2	34.95861774	26.48708424	29.14963235	23.87133351	25.72463069	6.435834728	18.45945315	20.62280883	19.30657793	16.82168652	24.52349964	26.2534477
ENSMUSG00000018372	CEP95	61.17758104	26.48708424	72.87408088	47.74266702	57.88041905	19.30750418	59.33395656	47.69024541	63.18516412	60.07745185	42.0402851	58.6841772
ENSMUSG00000018377	VEZF1	131.0948165	99.32656588	94.73630514	77.58183392	57.88041905	45.05084309	79.11194208	59.29057538	73.71602481	76.89913837	73.57049893	75.67170218
ENSMUSG00000018378	CUEDC1	17.47930887	13.24354212	36.43704044	23.87133351	12.86231535	25.74333891	25.05211499	18.04495772	14.04114758	20.42633363	21.02014255	24.70912724
ENSMUSG00000018379	SRSF1	375.8051407	377.4409504	364.3704044	381.9413362	398.7317757	386.1500837	495.7681704	469.1689008	526.5430344	526.2784782	425.6578867	483.3723017
ENSMUSG00000018387	SHROOM1	0	0	0	0	0	0	0	0	0	0	0	1.544320453
ENSMUSG00000018395	KIF3A	26.2189633	6.621771059	21.86222426	29.83916689	19.29347302	19.30750418	22.41505026	24.48958548	22.81686482	34.84492207	33.28189237	20.07616588
ENSMUSG00000018398	39692	26.2189633	19.86531318	14.57481618	5.967833378	12.86231535	25.74333891	19.77798552	6.444627758	12.28600414	16.82168652	14.01342837	12.35456362
ENSMUSG00000018401	MTMR4	17.47930887	6.621771059	7.287408088	5.967833378	6.431157673	6.435834728	13.18532368	15.46710662	22.81686482	18.02323556	12.26174982	13.89888407
ENSMUSG00000018405	MRM1	26.2189633	13.24354212	0	0	12.86231535	6.435834728	3.955597104	6.444627758	5.265430344	9.612392296	10.51007128	4.632961358
ENSMUSG00000018411	MAPT	0	0	0	0	0	0	0	0	0	1.201549037	0	1.544320453
ENSMUSG00000018412	KANSL1	69.91723547	92.70479482	72.87408088	77.58183392	102.8985228	96.53752092	102.8455247	97.95834193	50.89915999	98.52702104	103.3490342	75.67170218
ENSMUSG00000018415	GID4	17.47930887	26.48708424	29.14963235	17.90350013	45.01810371	19.30750418	13.18532368	16.75603217	17.55143448	18.02323556	19.26846401	23.16480679
ENSMUSG00000018425	DHX40	34.95861774	26.48708424	43.72444853	47.74266702	45.01810371	38.61500837	54.05982709	38.66776655	33.34772551	50.46505955	33.28189237	23.16480679
ENSMUSG00000018427	YPEL2	8.739654434	0	14.57481618	5.967833378	6.431157673	6.435834728	11.86679131	9.022478862	7.020573791	6.007745185	7.006714184	13.89888407
ENSMUSG00000018428	AKAP1	52.4379266	39.73062635	36.43704044	83.54966729	32.15578836	51.48667782	61.9710213	52.84594762	57.91973378	46.86041244	49.04699929	50.96257494
ENSMUSG00000018433	NOL11	78.65688991	92.70479482	87.44889705	71.61400054	57.88041905	38.61500837	79.11194208	81.20230975	84.2468855	63.68209896	75.32217748	75.67170218
ENSMUSG00000018442	DERL2	43.69827217	33.10885529	36.43704044	29.83916689	25.72463069	45.05084309	44.83010051	29.64528769	21.06172137	31.24027496	38.53692801	30.88640905
ENSMUSG00000018446	C1QBP	87.39654434	72.83948165	153.0355698	119.3566676	102.8985228	122.2808598	212.2837112	184.3163539	172.0040579	191.0462969	197.9396757	216.2048634
ENSMUSG00000018449	RPAIN	26.2189633	13.24354212	14.57481618	5.967833378	19.29347302	12.87166946	11.86679131	21.91173438	17.55143448	12.01549037	10.51007128	20.07616588
ENSMUSG00000018451	6330403K07F	0	0	0	0	0	0	1.318532368	2.577851103	0	2.403098074	1.751678546	0
ENSMUSG00000018470	KCNAB3	0	0	0	0	0	0	0	1.288925552	0	0	0	0
ENSMUSG00000018474	CHD3	270.9292875	304.6014687	218.6222426	232.7455017	237.9528339	238.1258849	172.7277402	203.6502372	175.5143448	162.20912	206.6980684	200.7616588
ENSMUSG00000018476	KDM6B	201.012052	152.3007344	116.5985294	143.2280011	83.60504975	115.8450251	90.97873339	118.5811508	87.75717239	98.52702104	77.07385602	131.2672385
ENSMUSG00000018479	1700125H20F	0	0	0	0	0	0	0	1.288925552	0	0	0	0

ENSMUSG00000018481	APPBP2	113.6155076	99.32656588	51.01185661	83.54966729	57.88041905	51.48667782	77.79340971	90.22478862	64.94030757	84.10843259	66.56378475	81.84898399
ENSMUSG00000018500	ADORA2B	0	0	0	0	0	0	0	0	0	2.403098074	0	0
ENSMUSG00000018501	NCOR1	568.0775382	569.4723111	546.5556066	614.6868379	533.7860868	469.8159351	352.0481423	413.7451021	440.5410054	413.3328687	385.3692801	381.4471518
ENSMUSG00000018507	TRPV2	122.3551621	66.21771059	58.2992647	65.64616716	77.17389207	77.23001673	76.47487734	99.24726748	77.22631171	92.51927585	89.33560584	94.20354761
ENSMUSG00000018509	CENPV	61.17758104	52.97416847	80.16148896	101.4531674	51.44926138	45.05084309	71.20074787	67.02412869	80.7365986	67.28674607	84.08057021	100.3808294
ENSMUSG00000018537	PCGF2	17.47930887	33.10885529	36.43704044	5.967833378	38.58694604	19.30750418	13.18532368	11.60032996	12.28600414	21.62788267	12.26174982	29.3420886
ENSMUSG00000018541	CWC25	43.69827217	39.73062635	14.57481618	23.87133351	32.15578836	25.74333891	27.68917973	27.06743658	42.12344275	20.42633363	33.28189237	24.70912724
ENSMUSG00000018543	1700001P01F	0	0	0	0	0	0	0	0	0	1.201549037	0	1.544320453
ENSMUSG00000018547	PIP4K2B	34.95861774	33.10885529	36.43704044	35.80700027	19.29347302	6.435834728	22.41505026	32.22313879	36.85801241	31.24027496	38.53692801	29.3420886
ENSMUSG00000018548	TRIM37	8.739654434	52.97416847	36.43704044	41.77483365	38.58694604	25.74333891	32.9633092	29.64528769	17.55143448	18.02323556	19.26846401	29.3420886
ENSMUSG00000018559	CTDNEP1	78.65688991	86.08302377	123.8859375	53.7105004	70.7427344	64.35834728	48.78569762	74.757682	87.75717239	72.09294222	61.30874911	63.31713856
ENSMUSG00000018565	ELP5	78.65688991	39.73062635	72.87408088	59.67833378	38.58694604	12.87166946	47.46716525	51.55702207	57.91973378	43.25576533	54.30203492	60.22849765
ENSMUSG00000018567	GABARAP	96.13619877	26.48708424	43.72444853	41.77483365	38.58694604	45.05084309	52.74129472	56.71272427	66.69545102	42.0542163	56.05371347	64.86145901
ENSMUSG00000018572	PHF23	34.95861774	6.621771059	36.43704044	41.77483365	25.72463069	57.92251255	46.14863288	37.378841	68.45059447	38.44956918	36.78524947	35.51937041
ENSMUSG00000018574	ACADVL	26.2189633	26.48708424	65.58667279	29.83916689	25.72463069	38.61500837	58.01542419	50.26809651	36.85801241	34.84492207	49.04699929	58.6841772
ENSMUSG00000018581	DNAH11	34.95861774	13.24354212	7.287408088	35.80700027	6.431157673	25.74333891	17.14092078	18.04495772	12.28600414	25.23252978	21.02014255	12.35456362
ENSMUSG00000018583	G3BP1	1634.315379	1443.546091	1523.06829	1342.76251	1569.202472	1280.731111	1024.49965	1166.477624	1089.944081	1182.324252	1126.329305	999.1753329
ENSMUSG00000018585	ATOX1	43.69827217	59.59593953	43.72444853	53.7105004	70.7427344	32.17917364	101.5269923	78.62445865	80.7365986	78.10068741	89.33560584	100.3808294
ENSMUSG00000018599	MIEF2	8.739654434	19.86531318	7.287408088	0	6.431157673	19.30750418	10.54825894	7.73355331	7.020573791	10.81394133	10.51007128	9.265922716
ENSMUSG00000018648	DUSP14	0	0	0	0	0	0	1.318532368	3.866776655	5.265430344	1.201549037	0	1.544320453
ENSMUSG00000018651	TADA2A	34.95861774	33.10885529	36.43704044	29.83916689	38.58694604	25.74333891	35.60037394	30.93421324	24.57200827	39.65111822	42.0402851	38.60801132
ENSMUSG00000018654	IKZF1	332.1068685	251.6273002	291.4963235	274.5203354	270.1086223	263.8692238	250.5211499	244.8958548	249.2303696	288.3717689	253.9933892	282.6106428
ENSMUSG00000018659	PNPO	8.739654434	6.621771059	43.72444853	29.83916689	19.29347302	19.30750418	22.41505026	21.91173438	22.81686482	14.41858844	21.02014255	12.35456362
ENSMUSG00000018661	COG1	34.95861774	6.621771059	21.86222426	5.967833378	12.86231535	0	9.229726576	2.577851103	8.775717239	13.21703941	15.76510691	15.44320453
ENSMUSG00000018666	CBX1	384.5447951	350.9538661	371.6578125	453.5553367	302.2644106	527.7384477	553.7835946	600.6393071	637.1170716	541.8986157	581.5572773	626.9941038
ENSMUSG00000018669	CDK5RAP3	26.2189633	33.10885529	0	35.80700027	32.15578836	19.30750418	25.05211499	37.378841	36.85801241	24.03098074	15.76510691	27.79776815
ENSMUSG00000018672	COPZ2	0	0	0	0	6.431157673	6.435834728	0	0	0	0	0	0
ENSMUSG00000018677	SLC25A39	52.4379266	92.70479482	51.01185661	83.54966729	77.17389207	70.79418201	92.29726576	64.44627758	91.26745929	90.11617778	70.06714184	71.03874082
ENSMUSG00000018678	SP2	43.69827217	39.73062635	51.01185661	41.77483365	38.58694604	25.74333891	22.41505026	38.66776655	33.34772551	34.84492207	29.77853528	26.2534477
ENSMUSG00000018697	AATF	69.91723547	39.73062635	51.01185661	59.67833378	51.44926138	57.92251255	40.87450341	33.51206434	56.16459033	33.64337304	43.79196365	58.6841772
ENSMUSG00000018707	DYNC1H1	61.17758104	46.35239741	72.87408088	83.54966729	109.3296804	83.66585146	59.33395656	76.04660755	73.71602481	66.08519704	85.83224875	84.9376249
ENSMUSG00000018733	PEX12	8.739654434	13.24354212	14.57481618	5.967833378	12.86231535	25.74333891	7.911194208	14.17818107	21.06172137	14.41858844	28.02685674	10.81024317
ENSMUSG00000018736	NDEL1	26.2189633	19.86531318	14.57481618	17.90350013	6.431157673	19.30750418	19.77798552	25.77851103	29.83743861	15.62013748	29.77853528	15.44320453
ENSMUSG00000018740	SLC25A35	0	0	0	0	0	0	1.318532368	0	1.755143448	0	0	0
ENSMUSG00000018750	ZBTB4	34.95861774	6.621771059	14.57481618	5.967833378	6.431157673	6.435834728	2.637064736	5.155702207	5.265430344	1.201549037	1.751678546	6.177281811
ENSMUSG00000018761	MPDU1	17.47930887	33.10885529	14.57481618	11.93566676	19.29347302	38.61500837	27.68917973	28.35636214	24.57200827	26.43407881	33.28189237	23.16480679
ENSMUSG00000018765	FXR2	61.17758104	59.59593953	72.87408088	83.54966729	32.15578836	64.35834728	68.56368314	73.46875644	80.7365986	40.85266726	84.08057021	64.86145901
ENSMUSG00000018770	ATP5G3	655.4740826	483.3892873	699.5911764	560.9763375	610.9599789	469.8159351	948.0247726	934.471025	989.9009046	987.6733084	973.9332716	942.0354761
ENSMUSG00000018774	CD68	0	0	0	0	0	0	3.955597104	0	1.755143448	2.403098074	0	0
ENSMUSG00000018796	ACSL1	0	6.621771059	7.287408088	23.87133351	12.86231535	19.30750418	17.14092078	10.31140441	8.775717239	13.21703941	8.75839273	12.35456362
ENSMUSG00000018800	ABCA5	0	0	7.287408088	0	6.431157673	0	6.59266184	1.288925552	8.775717239	1.201549037	5.255035638	4.632961358
ENSMUSG00000018809	SMYD4	0	19.86531318	21.86222426	17.90350013	12.86231535	12.87166946	15.82238842	12.88925552	12.28600414	3.604647111	8.75839273	20.07616588
ENSMUSG00000018819	LSP1	0	0	0	0	0	0	0	0	0	1.201549037	0	0
ENSMUSG00000018820	ZFYVE27	26.2189633	26.48708424	7.287408088	11.93566676	19.29347302	25.74333891	11.86679131	20.62280883	21.06172137	20.42633363	28.02685674	29.3420886
ENSMUSG00000018821	AVPI1	8.739654434	0	0	0	0	6.435834728	1.318532368	0	1.755143448	3.604647111	7.006714184	3.088640905

ENSMUSG00000018822	SFRP5	0	0	0	0	6.431157673	0	0	0	0	0	0	0
ENSMUSG00000018830	MYH11	0	0	0	5.967833378	0	0	1.318532368	1.288925552	0	1.201549037	0	0
ENSMUSG00000018841	RAD51D	34.95861774	6.621771059	14.57481618	5.967833378	12.86231535	12.87166946	22.41505026	18.04495772	21.06172137	9.612392296	24.52349964	27.79776815
ENSMUSG00000018844	FNDC8	0	0	0	5.967833378	19.29347302	0	2.637064736	2.577851103	3.510286896	2.403098074	1.751678546	7.721602263
ENSMUSG00000018846	PANK3	17.47930887	33.10885529	51.01185661	23.87133351	51.44926138	45.05084309	61.9710213	59.29057538	61.43002068	62.48054992	59.55707056	54.05121584
ENSMUSG00000018848	RARS	96.13619877	79.46125271	58.2992647	113.3888342	77.17389207	45.05084309	135.8088339	139.2039596	124.6151848	106.9378643	110.3557484	111.1910726
ENSMUSG00000018849	WWC1	0	0	0	0	0	0	0	1.318532368	0	0	1.201549037	1.751678546	0
ENSMUSG00000018858	MRPL58	26.2189633	19.86531318	29.14963235	41.77483365	51.44926138	38.61500837	27.68917973	25.77851103	24.57200827	36.04647111	26.27517819	26.2534477
ENSMUSG00000018861	FDXR	26.2189633	46.35239741	36.43704044	17.90350013	19.29347302	38.61500837	25.05211499	37.378841	22.81686482	33.64337304	24.52349964	29.3420886
ENSMUSG00000018882	MRPL45	69.91723547	39.73062635	58.2992647	71.61400054	102.8985228	83.66585146	87.02313629	74.757682	84.2468855	69.68984415	68.31546329	88.0262658
ENSMUSG00000018899	IRF1	26.2189633	52.97416847	58.2992647	17.90350013	64.31157673	57.92251255	39.55597104	38.66776655	40.3682993	30.03872593	49.04699929	47.87393403
ENSMUSG00000018900	SLC22A5	0	0	0	0	0	6.435834728	0	3.866776655	1.755143448	1.201549037	0	1.544320453
ENSMUSG00000018906	P4HA2	0	0	0	0	0	0	2.637064736	0	0	0	0	0
ENSMUSG00000018909	ARRB1	0	0	0	0	0	0	3.955597104	6.444627758	0	1.201549037	1.751678546	0
ENSMUSG00000018916	CSF2	17.47930887	0	0	23.87133351	6.431157673	12.87166946	1.318532368	5.155702207	3.510286896	6.007745185	5.255035638	10.81024317
ENSMUSG00000018920	CXCL16	8.739654434	0	0	0	0	0	0	0	0	0	1.751678546	0
ENSMUSG00000018921	PELP1	139.8344709	46.35239741	94.73630514	101.4531674	90.03620742	77.23001673	73.83781261	105.6918952	107.0637503	102.1316681	119.1141411	109.6467521
ENSMUSG00000018923	MED11	26.2189633	6.621771059	14.57481618	23.87133351	6.431157673	12.87166946	21.09651789	24.48958548	10.53086069	15.62013748	26.27517819	27.79776815
ENSMUSG00000018924	ALOX15	0	0	0	5.967833378	0	0	0	0	0	0	0	0
ENSMUSG00000018925	HEATR9	0	0	0	0	0	0	0	1.288925552	0	0	0	0
ENSMUSG00000018931	NATD1	0	0	0	0	0	0	2.637064736	2.577851103	0	1.201549037	0	0
ENSMUSG00000018932	MAP2K3	43.69827217	39.73062635	14.57481618	41.77483365	38.58694604	38.61500837	43.51156814	36.08991545	42.12344275	36.04647111	40.28860656	24.70912724
ENSMUSG00000018965	YWHAH	61.17758104	39.73062635	36.43704044	35.80700027	25.72463069	25.74333891	42.19303578	32.22313879	36.85801241	49.26351052	50.79867783	57.13985675
ENSMUSG00000018974	SART3	113.6155076	72.83948165	87.44889705	89.51750067	128.6231535	64.35834728	52.74129472	64.44627758	73.71602481	70.89139318	75.32217748	80.30466354
ENSMUSG00000018983	E2F2	69.91723547	19.86531318	43.72444853	53.7105004	45.01810371	38.61500837	35.60037394	25.77851103	43.8785862	34.84492207	33.28189237	30.88640905
ENSMUSG00000018986	SLFN3	8.739654434	39.73062635	21.86222426	23.87133351	6.431157673	19.30750418	13.18532368	18.04495772	14.04114758	16.82168652	28.02685674	9.265922716
ENSMUSG00000018995	NARS2	8.739654434	13.24354212	7.287408088	17.90350013	0	0	10.54825894	7.73355331	15.79629103	9.612392296	10.51007128	4.632961358
ENSMUSG00000018999	SLC35B4	104.8758532	46.35239741	80.16148896	41.77483365	83.60504975	64.35834728	60.65248893	43.82346876	43.8785862	54.06970667	43.79196365	67.95009992
ENSMUSG00000019039	DALRD3	43.69827217	59.59593953	36.43704044	89.51750067	45.01810371	51.48667782	55.37835946	61.86842648	50.89915999	32.441824	94.59064148	63.31713856
ENSMUSG00000019054	FIS1	69.91723547	125.8136501	94.73630514	77.58183392	83.60504975	32.17917364	125.260575	113.4254485	80.7365986	88.91462874	108.6040698	109.6467521
ENSMUSG00000019055	PLOD1	0	6.621771059	0	0	0	0	5.274129472	2.577851103	5.265430344	7.209294222	3.503357092	4.632961358
ENSMUSG00000019066	RAB3D	0	0	0	0	0	0	1.318532368	0	0	0	3.503357092	0
ENSMUSG00000019080	MFSD3	17.47930887	13.24354212	14.57481618	5.967833378	12.86231535	12.87166946	10.54825894	7.73355331	5.265430344	4.806196148	1.751678546	15.44320453
ENSMUSG00000019082	SLC25A22	0	39.73062635	36.43704044	41.77483365	45.01810371	19.30750418	32.9633092	27.06743658	28.08229517	27.63562785	29.77853528	44.78529313
ENSMUSG00000019087	ATP6AP1	34.95861774	26.48708424	7.287408088	23.87133351	12.86231535	45.05084309	44.83010051	32.22313879	22.81686482	34.84492207	31.53021383	33.97504996
ENSMUSG00000019088	DNASE1L1	0	0	0	0	0	0	5.274129472	5.155702207	3.510286896	2.403098074	0	0
ENSMUSG00000019132	BC005537	550.5982293	615.8247085	604.8548713	543.0728374	463.0433524	656.4551422	440.3898109	430.5011343	501.9710261	462.5963793	409.8927798	362.9153064
ENSMUSG00000019139	ISYNA1	61.17758104	19.86531318	51.01185661	83.54966729	77.17389207	32.17917364	60.65248893	58.00164982	42.12344275	39.65111822	43.79196365	58.6841772
ENSMUSG00000019143	HARS2	17.47930887	13.24354212	21.86222426	23.87133351	32.15578836	19.30750418	25.05211499	28.35636214	38.61315585	25.23252978	36.78524947	32.43072951
ENSMUSG00000019158	TMEM160	43.69827217	52.97416847	87.44889705	83.54966729	64.31157673	83.66585146	65.9266184	96.66941637	66.69545102	70.89139318	82.32889166	71.03874082
ENSMUSG00000019173	RAB5C	52.4379266	105.9483369	51.01185661	107.4210008	90.03620742	64.35834728	68.56368314	94.09156527	82.49174205	70.89139318	85.83224875	83.39330444
ENSMUSG00000019179	MDH2	166.0534342	198.6531318	211.3348345	179.0350013	180.0724148	193.0750418	304.580977	320.9424624	324.7015379	296.7826121	348.5840306	308.8640905
ENSMUSG00000019188	H13	139.8344709	139.0571922	116.5985294	131.2923343	167.2100995	148.0241987	168.7721431	185.6052794	193.0657793	163.410669	147.1409979	208.4832611
ENSMUSG00000019189	RNF145	34.95861774	72.83948165	87.44889705	47.74266702	45.01810371	57.92251255	88.34166866	95.38049082	75.47116826	103.3332172	103.3490342	81.84898399
ENSMUSG00000019210	ATP6V1E1	61.17758104	13.24354212	51.01185661	35.80700027	19.29347302	57.92251255	43.51156814	59.29057538	42.12344275	55.2712557	63.06042765	55.5955363

ENSMUSG00000019214	CHTF18	148.5741254	59.59593953	58.2992647	77.58183392	90.03620742	77.23001673	75.15634498	78.62445865	73.71602481	70.89139318	78.82553457	92.65922716
ENSMUSG00000019230	LHX9	0	6.621771059	0	0	6.431157673	0	2.637064736	0	0	0	0	0
ENSMUSG00000019235	RPS6KL1	17.47930887	19.86531318	21.86222426	5.967833378	6.431157673	6.435834728	7.911194208	9.022478862	8.775717239	9.612392296	17.51678546	12.35456362
ENSMUSG00000019254	PPP1R12C	157.3137798	86.08302377	131.1733456	137.2601677	102.8985228	57.92251255	88.34166866	94.09156527	78.98145515	82.90688355	115.610784	97.29218852
ENSMUSG00000019261	MAP1S	34.95861774	33.10885529	29.14963235	17.90350013	45.01810371	0	18.45945315	20.62280883	31.59258206	25.23252978	22.7718211	29.3420886
ENSMUSG00000019295	TMEM129	0	39.73062635	14.57481618	11.93566676	25.72463069	25.74333891	15.82238842	19.33388327	24.57200827	13.21703941	31.53021383	15.44320453
ENSMUSG00000019297	NOP9	17.47930887	26.48708424	29.14963235	11.93566676	19.29347302	12.87166946	36.9189063	34.80098989	17.55143448	19.22478459	15.76510691	30.88640905
ENSMUSG00000019302	ATP6V0A1	0	13.24354212	0	5.967833378	0	0	0	5.155702207	1.755143448	6.007745185	1.751678546	3.088640905
ENSMUSG00000019303	PSMC3IP	17.47930887	19.86531318	14.57481618	29.83916689	38.58694604	32.17917364	44.83010051	43.82346876	45.63372964	38.44956918	31.53021383	47.87393403
ENSMUSG00000019320	NOXO1	8.739654434	0	0	0	0	0	0	0	0	1.201549037	0	1.544320453
ENSMUSG00000019326	AOC3	8.739654434	0	0	0	0	0	0	0	0	0	3.503357092	0
ENSMUSG00000019338	ZFP687	174.7930887	165.5442765	218.6222426	167.0993346	154.3477841	173.7675376	113.3937836	104.4029697	121.1048979	99.72857007	99.84567712	128.1785976
ENSMUSG00000019359	GDPD2	0	0	0	0	0	6.435834728	0	0	0	0	0	0
ENSMUSG00000019362	D8ERTD738E	174.7930887	158.9225054	123.8859375	107.4210008	135.0543111	135.1525293	168.7721431	162.4046195	191.3106358	169.4184142	171.6644975	210.0275816
ENSMUSG00000019370	CALM3	646.7344281	496.6328294	685.0163602	614.6868379	623.8222943	566.353456	359.9593365	390.5444422	422.9895709	311.2012006	413.3961368	466.3847767
ENSMUSG00000019373	COPS3	87.39654434	79.46125271	80.16148896	77.58183392	77.17389207	64.35834728	113.3937836	122.4479274	133.390902	90.11617778	99.84567712	95.74786807
ENSMUSG00000019428	FKBP8	157.3137798	165.5442765	94.73630514	125.3245009	83.60504975	64.35834728	150.31269	141.7818107	110.5740372	91.31772681	127.8725339	157.5206862
ENSMUSG00000019432	DDX39B	340.8465229	403.9280346	284.2089154	220.809835	392.300618	276.7408933	276.8917973	260.3629614	301.884673	248.7206507	327.5638881	330.4845769
ENSMUSG00000019433	GIPC1	8.739654434	26.48708424	7.287408088	17.90350013	19.29347302	0	7.911194208	18.04495772	5.265430344	14.41858844	1.751678546	12.35456362
ENSMUSG00000019437	TLCD1	253.4499786	238.3837581	189.4726103	202.9063349	308.6955683	238.1258849	284.8029915	284.8525469	305.3949599	322.0151419	304.792067	267.1674383
ENSMUSG00000019461	PLSCR3	0	0	0	0	12.86231535	0	2.637064736	2.577851103	0	0	1.751678546	3.088640905
ENSMUSG00000019464	PTGER1	0	6.621771059	7.287408088	0	0	6.435834728	5.274129472	5.155702207	1.755143448	3.604647111	3.503357092	9.265922716
ENSMUSG00000019467	ARHGEF25	26.2189633	0	7.287408088	29.83916689	25.72463069	51.48667782	6.59266184	7.73355331	12.28600414	4.806196148	7.006714184	9.265922716
ENSMUSG00000019470	XAB2	61.17758104	86.08302377	65.58667279	71.61400054	45.01810371	90.10168619	48.78569762	54.13487317	45.63372964	43.25576533	78.82553457	49.41825449
ENSMUSG00000019471	CDC37	69.91723547	105.9483369	87.44889705	101.4531674	109.3296804	70.79418201	105.4825894	105.6918952	114.0843241	94.92237392	119.1141411	146.710443
ENSMUSG00000019478	RAB4A	26.2189633	6.621771059	36.43704044	35.80700027	32.15578836	25.74333891	44.83010051	48.97917096	64.94030757	62.48054992	47.29532074	52.50689539
ENSMUSG00000019487	TRIP10	8.739654434	33.10885529	43.72444853	23.87133351	25.72463069	25.74333891	22.41505026	25.77851103	28.08229517	24.03098074	28.02685674	23.16480679
ENSMUSG00000019494	COPS6	122.3551621	125.8136501	109.3111213	95.48533405	96.46736509	128.7166946	105.4825894	136.6261085	108.8188938	127.3641979	145.3893193	103.4694703
ENSMUSG00000019505	UBB	270.9292875	264.8708424	225.9096507	214.8420016	250.8151492	186.6392071	247.8840852	277.1189936	273.8023779	235.5036113	289.0269601	296.5095269
ENSMUSG00000019518	AP4M1	174.7930887	158.9225054	160.3229779	107.4210008	135.0543111	167.3317029	117.3493808	188.1831305	142.1666193	117.7518056	150.644355	177.5968521
ENSMUSG00000019528	GYG	157.3137798	178.7878186	80.16148896	77.58183392	128.6231535	90.10168619	138.4458986	155.9599918	168.493771	138.1781393	131.3758909	117.3683544
ENSMUSG00000019539	RCN3	0	0	7.287408088	0	0	0	0	0	0	0	0	0
ENSMUSG00000019558	SLC6A8	0	0	0	0	0	0	0	2.577851103	0	0	1.751678546	0
ENSMUSG00000019564	ARID3A	69.91723547	46.35239741	123.8859375	83.54966729	122.1919958	128.7166946	84.38607155	64.44627758	101.79832	87.7130797	112.1074269	89.57058625
ENSMUSG00000019578	UBXN6	52.4379266	79.46125271	43.72444853	35.80700027	64.31157673	115.8450251	76.47487734	77.3355331	63.18516412	81.70533452	71.81882038	74.12738173
ENSMUSG00000019579	MYDGF	122.3551621	92.70479482	109.3111213	119.3566676	96.46736509	115.8450251	135.8088339	130.1814807	140.4114758	111.7440604	148.8926764	134.3558794
ENSMUSG00000019659	CCDC12	8.739654434	6.621771059	14.57481618	11.93566676	6.431157673	25.74333891	22.41505026	10.31140441	33.34772551	19.22478459	14.01342837	20.07616588
ENSMUSG00000019689	FMC1	139.8344709	92.70479482	153.0355698	179.0350013	180.0724148	109.4091904	172.7277402	174.0049495	214.1275006	177.8292575	185.6779259	182.2298134
ENSMUSG00000019699	AKT3	0	0	0	0	0	0	0	0	0	0	0	1.544320453
ENSMUSG00000019710	MRPL24	183.5327431	145.6789633	109.3111213	131.2923343	270.1086223	109.4091904	193.8242581	183.0274283	210.6172137	159.8060219	187.4296044	152.8877248
ENSMUSG00000019715	GLE1	61.17758104	52.97416847	87.44889705	47.74266702	70.7427344	70.79418201	60.65248893	97.95834193	93.02260274	76.89913837	92.83896294	112.735393
ENSMUSG00000019726	LYST	0	0	7.287408088	0	0	6.435834728	7.911194208	14.17818107	8.775717239	8.410843259	10.51007128	13.89888407
ENSMUSG00000019731	SLC35E1	61.17758104	72.83948165	65.58667279	17.90350013	57.88041905	32.17917364	27.68917973	24.48958548	33.34772551	26.43407881	31.53021383	24.70912724
ENSMUSG00000019732	CALR3	0	0	0	5.967833378	0	0	1.318532368	1.288925552	0	1.201549037	0	3.088640905
ENSMUSG00000019734	TMC4	8.739654434	6.621771059	7.287408088	0	6.431157673	6.435834728	0	1.288925552	0	1.201549037	1.751678546	1.544320453

ENSMUSG00000019737	SYNE4	8.739654434	0	0	5.967833378	0	6.435834728	0	2.577851103	3.510286896	1.201549037	0	0
ENSMUSG00000019738	POLR2I	34.95861774	26.48708424	43.72444853	35.80700027	25.72463069	64.35834728	47.46716525	38.66776655	43.8785862	38.44956918	71.81882038	46.32961358
ENSMUSG00000019763	RMND1	43.69827217	6.621771059	43.72444853	17.90350013	51.44926138	6.435834728	25.05211499	20.62280883	17.55143448	26.43407881	19.26846401	18.53184543
ENSMUSG00000019767	CCDC170	0	6.621771059	0	0	0	0	0	0	0	0	0	0
ENSMUSG00000019768	ESR1	26.2189633	13.24354212	29.14963235	0	12.86231535	0	9.229726576	15.46710662	8.775717239	8.410843259	5.255035638	10.81024317
ENSMUSG00000019773	FBXO5	96.13619877	205.2749028	72.87408088	89.51750067	70.7427344	90.10168619	106.8011218	122.4479274	121.1048979	121.3564527	117.3624626	118.9126749
ENSMUSG00000019774	MTRF1L	26.2189633	46.35239741	29.14963235	59.67833378	19.29347302	51.48667782	36.9189063	50.26809651	22.81686482	22.8294317	43.79196365	30.88640905
ENSMUSG00000019775	RGS17	0	0	0	0	0	0	0	1.288925552	0	0	0	0
ENSMUSG00000019777	HDAC2	148.5741254	165.5442765	182.1852022	214.8420016	173.6412572	135.1525293	195.1427905	186.894205	180.7797751	200.6586892	182.1745688	160.6093271
ENSMUSG00000019782	RWDD1	113.6155076	112.570108	65.58667279	95.48533405	45.01810371	25.74333891	87.02313629	54.13487317	75.47116826	48.06196148	70.06714184	64.86145901
ENSMUSG00000019787	TRDN	0	0	0	0	0	0	0	0	1.755143448	0	0	0
ENSMUSG00000019790	STXBP5	52.4379266	92.70479482	51.01185661	71.61400054	70.7427344	57.92251255	35.60037394	47.69024541	50.89915999	39.65111822	49.04699929	49.41825449
ENSMUSG00000019791	HINT3	0	13.24354212	14.57481618	11.93566676	6.431157673	6.435834728	1.318532368	6.444627758	7.020573791	1.201549037	5.255035638	9.265922716
ENSMUSG00000019792	TRMT11	17.47930887	33.10885529	7.287408088	11.93566676	25.72463069	12.87166946	26.37064736	29.64528769	21.06172137	36.04647111	40.28860656	29.3420886
ENSMUSG00000019794	KATNA1	43.69827217	39.73062635	21.86222426	29.83916689	25.72463069	51.48667782	36.9189063	41.24561765	43.8785862	39.65111822	52.55035638	32.43072951
ENSMUSG00000019795	PCMT1	43.69827217	26.48708424	87.44889705	77.58183392	45.01810371	25.74333891	56.69689182	60.57950093	59.67487723	67.28674607	75.32217748	52.50689539
ENSMUSG00000019796	LRP11	0	0	29.14963235	5.967833378	32.15578836	12.87166946	6.59266184	7.73355331	8.775717239	4.806196148	8.75839273	12.35456362
ENSMUSG00000019797	1700021F05R	17.47930887	46.35239741	21.86222426	41.77483365	38.58694604	12.87166946	19.77798552	29.64528769	26.32715172	24.03098074	28.02685674	43.24097267
ENSMUSG00000019802	SEC63	52.4379266	92.70479482	94.73630514	113.3888342	96.46736509	51.48667782	68.56368314	74.757682	43.8785862	68.48829511	47.29532074	74.12738173
ENSMUSG00000019804	SNX3	122.3551621	99.32656588	72.87408088	53.7105004	96.46736509	83.66585146	126.5791073	113.4254485	117.594611	121.3564527	136.6309266	131.2672385
ENSMUSG00000019806	AIG1	8.739654434	13.24354212	21.86222426	29.83916689	25.72463069	25.74333891	19.77798552	19.33388327	21.06172137	14.41858844	12.26174982	21.62048634
ENSMUSG00000019808	ADAT2	8.739654434	0	0	0	6.431157673	0	5.274129472	1.288925552	1.755143448	2.403098074	1.751678546	0
ENSMUSG00000019809	PEX3	8.739654434	19.86531318	14.57481618	5.967833378	51.44926138	25.74333891	21.09651789	23.20065993	26.32715172	19.22478459	35.03357092	10.81024317
ENSMUSG00000019810	FUCA2	26.2189633	26.48708424	14.57481618	11.93566676	12.86231535	25.74333891	11.86679131	10.31140441	19.30657793	7.209294222	12.26174982	12.35456362
ENSMUSG00000019813	CEP57L1	43.69827217	13.24354212	0	47.74266702	38.58694604	64.35834728	36.9189063	24.48958548	35.10286896	40.85266726	42.0402851	33.97504996
ENSMUSG00000019814	LTV1	34.95861774	52.97416847	29.14963235	23.87133351	45.01810371	83.66585146	54.05982709	51.55702207	57.91973378	51.66660859	66.56378475	47.87393403
ENSMUSG00000019817	PLAGL1	690.4327003	622.4464795	896.3511948	942.9176737	771.7389207	592.096795	660.5847164	746.2878944	865.2857198	782.2084231	709.4298111	878.7183376
ENSMUSG00000019818	CD164	69.91723547	86.08302377	116.5985294	47.74266702	83.60504975	102.9733556	75.15634498	94.09156527	71.96088136	81.70533452	71.81882038	92.65922716
ENSMUSG00000019820	UTRN	61.17758104	119.1918791	116.5985294	59.67833378	77.17389207	77.23001673	81.74900682	64.44627758	82.49174205	86.51153067	80.57721311	71.03874082
ENSMUSG00000019822	SMPD2	26.2189633	46.35239741	29.14963235	47.74266702	25.72463069	12.87166946	19.77798552	33.51206434	15.79629103	15.62013748	28.02685674	18.53184543
ENSMUSG00000019823	MICAL1	34.95861774	19.86531318	36.43704044	17.90350013	38.58694604	45.05084309	15.82238842	37.378841	22.81686482	20.42633363	24.52349964	21.62048634
ENSMUSG00000019826	ZBTB24	0	33.10885529	14.57481618	11.93566676	6.431157673	0	14.50385605	14.17818107	19.30657793	6.007745185	1.751678546	4.632961358
ENSMUSG00000019837	GTF3C6	104.8758532	52.97416847	58.2992647	65.64616716	12.86231535	45.05084309	46.14863288	64.44627758	71.96088136	56.47280474	75.32217748	57.13985675
ENSMUSG00000019838	SLC16A10	43.69827217	39.73062635	43.72444853	65.64616716	51.44926138	51.48667782	35.60037394	47.69024541	36.85801241	46.86041244	64.8121062	44.78529313
ENSMUSG00000019841	REV3L	113.6155076	79.46125271	72.87408088	71.61400054	77.17389207	45.05084309	88.34166866	70.89090534	59.67487723	75.69758933	45.5436422	50.96257494
ENSMUSG00000019842	TRAF3IP2	34.95861774	19.86531318	21.86222426	17.90350013	6.431157673	19.30750418	10.54825894	30.93421324	29.83743861	18.02323556	26.27517819	26.2534477
ENSMUSG00000019843	FYN	78.65688991	125.8136501	80.16148896	173.067168	160.7789418	115.8450251	97.57139523	117.2922252	114.0843241	99.72857007	126.1208553	106.5581112
ENSMUSG00000019845	TUBE1	43.69827217	13.24354212	29.14963235	29.83916689	32.15578836	12.87166946	22.41505026	20.62280883	38.61315585	26.43407881	21.02014255	27.79776815
ENSMUSG00000019849	PREP	61.17758104	92.70479482	80.16148896	131.2923343	57.88041905	115.8450251	116.0308484	143.0707362	129.8806151	108.1394133	110.3557484	117.3683544
ENSMUSG00000019850	TNFAIP3	17.47930887	6.621771059	7.287408088	0	6.431157673	0	5.274129472	5.155702207	12.28600414	7.209294222	12.26174982	10.81024317
ENSMUSG00000019851	PERP	0	0	0	0	0	0	0	0	0	0	0	1.544320453
ENSMUSG00000019852	ARFGEF3	131.0948165	86.08302377	72.87408088	65.64616716	83.60504975	83.66585146	60.65248893	56.71272427	40.3682993	49.26351052	43.79196365	57.13985675
ENSMUSG00000019854	REPS1	43.69827217	33.10885529	58.2992647	41.77483365	38.58694604	64.35834728	42.19303578	42.5345432	50.89915999	39.65111822	43.79196365	50.96257494
ENSMUSG00000019857	ASF1A	122.3551621	105.9483369	102.0237132	35.80700027	45.01810371	51.48667782	79.11194208	76.04660755	82.49174205	91.31772681	96.34232003	81.84898399
ENSMUSG00000019861	GOPC	34.95861774	33.10885529	7.287408088	23.87133351	51.44926138	19.30750418	36.9189063	54.13487317	50.89915999	57.67435378	28.02685674	49.41825449

ENSMUSG00000019863	QRSL1	0	13.24354212	21.86222426	17.90350013	38.58694604	0	11.86679131	20.62280883	12.28600414	6.007745185	12.26174982	6.177281811
ENSMUSG00000019864	RTN4IP1	8.739654434	6.621771059	7.287408088	0	6.431157673	0	1.318532368	2.577851103	8.775717239	4.806196148	3.503357092	6.177281811
ENSMUSG00000019866	CRYBG1	0	0	0	0	0	6.435834728	0	0	1.755143448	0	0	0
ENSMUSG00000019868	VTA1	34.95861774	26.48708424	29.14963235	29.83916689	45.01810371	12.87166946	36.9189063	42.5345432	49.14401654	48.06196148	57.80539202	38.60801132
ENSMUSG00000019872	SMPDL3A	26.2189633	33.10885529	7.287408088	5.967833378	6.431157673	12.87166946	22.41505026	30.93421324	19.30657793	30.03872593	28.02685674	29.3420886
ENSMUSG00000019873	REEP3	26.2189633	19.86531318	14.57481618	47.74266702	12.86231535	25.74333891	27.68917973	19.33388327	19.30657793	20.42633363	7.006714184	16.98752498
ENSMUSG00000019877	SERINC1	43.69827217	112.570108	80.16148896	53.7105004	38.58694604	38.61500837	93.61579813	118.5811508	107.0637503	106.9378643	70.06714184	100.3808294
ENSMUSG00000019878	HSF2	305.8879052	245.0055292	204.0474265	280.4881688	270.1086223	283.176728	324.3589625	296.4528769	305.3949599	295.5810631	276.7652103	304.2311292
ENSMUSG00000019883	ECHDC1	34.95861774	13.24354212	36.43704044	11.93566676	19.29347302	12.87166946	40.87450341	24.48958548	35.10286896	33.64337304	26.27517819	32.43072951
ENSMUSG00000019889	PTPRK	0	0	0	0	0	0	1.318532368	0	1.755143448	2.403098074	1.751678546	4.632961358
ENSMUSG00000019897	CCDC59	122.3551621	218.5184449	138.4607537	137.2601677	154.3477841	160.8958682	170.0906755	154.6710662	140.4114758	154.9998258	136.6309266	163.697968
ENSMUSG00000019907	PPP1R12A	174.7930887	470.1457452	262.3466912	334.1986692	276.5397799	231.6900502	181.9574668	188.1831305	189.5554924	199.4571401	161.1544262	154.4320453
ENSMUSG00000019916	P4HA1	34.95861774	13.24354212	0	23.87133351	0	25.74333891	42.19303578	38.66776655	40.3682993	32.441824	31.53021383	43.24097267
ENSMUSG00000019917	40422	87.39654434	79.46125271	43.72444853	23.87133351	25.72463069	70.79418201	52.74129472	52.84594762	56.16459033	49.26351052	63.06042765	49.41825449
ENSMUSG00000019920	LIMS1	26.2189633	46.35239741	72.87408088	65.64616716	19.29347302	90.10168619	77.79340971	72.17983089	43.8785862	90.11617778	52.55035638	55.5955363
ENSMUSG00000019923	ZWINT	26.2189633	66.21771059	36.43704044	59.67833378	45.01810371	45.05084309	76.47487734	58.00164982	68.45059447	54.06970667	87.5839273	57.13985675
ENSMUSG00000019927	UBE2D1	26.2189633	19.86531318	36.43704044	35.80700027	25.72463069	57.92251255	30.32624446	37.378841	17.55143448	36.04647111	26.27517819	26.2534477
ENSMUSG00000019929	DCN	0	0	0	0	0	0	0	0	0	1.201549037	0	0
ENSMUSG00000019933	MRLN	0	0	0	0	0	0	0	1.288925552	0	0	0	0
ENSMUSG00000019936	EPYC	0	0	0	0	0	0	0	1.288925552	0	0	0	0
ENSMUSG00000019942	CDK1	61.17758104	59.59593953	80.16148896	107.4210008	154.3477841	128.7166946	134.4903015	127.6036296	154.4526234	135.7750412	122.6174982	132.8115589
ENSMUSG00000019943	ATP2B1	288.4085963	357.5756372	218.6222426	280.4881688	308.6955683	341.0992406	213.6022436	217.8284182	226.4135048	210.2710815	150.644355	177.5968521
ENSMUSG00000019945	CABCOCO1	0	0	0	0	0	0	0	1.288925552	0	1.201549037	1.751678546	1.544320453
ENSMUSG00000019947	ARID5B	34.95861774	19.86531318	36.43704044	35.80700027	25.72463069	38.61500837	29.0077121	47.69024541	28.08229517	38.44956918	52.55035638	44.78529313
ENSMUSG00000019948	ACTR6	8.739654434	19.86531318	21.86222426	0	25.72463069	0	29.0077121	27.06743658	21.06172137	20.42633363	24.52349964	43.24097267
ENSMUSG00000019951	UHRF1BP1L	78.65688991	79.46125271	36.43704044	89.51750067	51.44926138	25.74333891	60.65248893	70.89090534	70.20573791	51.66660859	82.32889166	61.77281811
ENSMUSG00000019952	POC1B	104.8758532	72.83948165	102.0237132	95.48533405	70.7427344	64.35834728	64.60808603	68.31305424	80.7365986	67.28674607	84.08057021	86.48194535
ENSMUSG00000019960	DUSP6	821.5275168	708.5295033	772.4652573	692.2686719	752.4454477	643.5834728	747.6078527	748.8657455	765.2425433	674.0690098	707.6781326	732.0078946
ENSMUSG00000019961	TMPO	244.7103242	291.3579266	306.0711397	298.3916689	289.4020953	205.9467113	378.4187896	327.3870901	384.3764151	385.6972409	406.3894227	362.9153064
ENSMUSG00000019969	PSEN1	43.69827217	13.24354212	29.14963235	5.967833378	19.29347302	6.435834728	25.05211499	21.91173438	17.55143448	12.01549037	19.26846401	18.53184543
ENSMUSG00000019970	SGK1	17.47930887	6.621771059	14.57481618	11.93566676	6.431157673	6.435834728	10.54825894	9.022478862	14.04114758	18.02323556	17.51678546	21.62048634
ENSMUSG00000019971	CEP290	0	0	0	0	0	0	0	0	1.755143448	2.403098074	0	0
ENSMUSG00000019975	IKBIP	52.4379266	79.46125271	94.73630514	53.7105004	77.17389207	57.92251255	71.20074787	72.17983089	78.98145515	73.29449126	66.56378475	81.84898399
ENSMUSG00000019977	HBS1L	17.47930887	59.59593953	29.14963235	41.77483365	51.44926138	25.74333891	55.37835946	38.66776655	49.14401654	43.25576533	42.0402851	44.78529313
ENSMUSG00000019978	EPB41L2	52.4379266	39.73062635	58.2992647	29.83916689	57.88041905	19.30750418	39.55597104	43.82346876	52.65430344	48.06196148	45.5436422	35.51937041
ENSMUSG00000019979	APAF1	26.2189633	39.73062635	65.58667279	29.83916689	32.15578836	32.17917364	46.14863288	54.13487317	42.12344275	45.65886341	57.80539202	52.50689539
ENSMUSG00000019982	MYB	69.91723547	66.21771059	102.0237132	41.77483365	45.01810371	38.61500837	55.37835946	82.49123531	93.02260274	50.46505955	49.04699929	67.95009992
ENSMUSG00000019984	MED23	34.95861774	52.97416847	21.86222426	41.77483365	38.58694604	6.435834728	18.45945315	27.06743658	22.81686482	26.43407881	19.26846401	26.2534477
ENSMUSG00000019986	AHI1	26.2189633	33.10885529	29.14963235	41.77483365	12.86231535	25.74333891	22.41505026	27.06743658	28.08229517	16.82168652	15.76510691	23.16480679
ENSMUSG00000019987	ARG1	26.2189633	26.48708424	58.2992647	23.87133351	77.17389207	38.61500837	30.32624446	16.75603217	28.08229517	22.8294317	8.75839273	18.53184543
ENSMUSG00000019988	NEDD1	0	19.86531318	14.57481618	35.80700027	38.58694604	19.30750418	23.73358262	20.62280883	21.06172137	36.04647111	28.02685674	26.2534477
ENSMUSG00000019989	ENPP3	0	0	0	5.967833378	0	0	0	0	0	0	0	0
ENSMUSG00000019992	MTFR2	17.47930887	13.24354212	14.57481618	5.967833378	6.431157673	0	2.637064736	6.444627758	3.510286896	6.007745185	5.255035638	9.265922716
ENSMUSG00000019998	STX7	0	0	0	0	0	0	0	0	1.755143448	3.604647111	0	1.544320453
ENSMUSG00000020000	MOXD1	0	0	0	0	0	0	0	1.288925552	0	0	0	0

ENSMUSG00000020003	PEX7	26.2189633	26.48708424	36.43704044	35.80700027	25.72463069	38.61500837	39.55597104	38.66776655	43.8785862	34.84492207	57.80539202	47.87393403
ENSMUSG00000020007	IL20RA	26.2189633	13.24354212	0	29.83916689	6.431157673	0	17.14092078	11.60032996	12.28600414	15.62013748	8.75839273	21.62048634
ENSMUSG00000020009	IFNGR1	192.2723975	79.46125271	174.8977941	161.1315012	154.3477841	173.7675376	93.61579813	157.2489173	115.8394676	126.1626489	112.1074269	129.722918
ENSMUSG00000020014	CFAP54	0	0	7.287408088	0	0	12.87166946	0	1.288925552	1.755143448	0	1.751678546	0
ENSMUSG00000020015	CDK17	96.13619877	99.32656588	51.01185661	47.74266702	32.15578836	77.23001673	105.4825894	74.757682	93.02260274	118.9533547	75.32217748	81.84898399
ENSMUSG00000020017	HAL	0	0	0	0	0	0	0	0	0	3.510286896	2.403098074	0	1.544320453
ENSMUSG00000020018	SNRPF	375.8051407	536.3634558	510.1185661	543.0728374	540.2172445	592.096795	474.6716525	487.2138585	533.5636082	462.5963793	476.4565645	537.4235175
ENSMUSG00000020021	FGD6	0	19.86531318	21.86222426	5.967833378	12.86231535	19.30750418	11.86679131	14.17818107	5.265430344	15.62013748	19.26846401	13.89888407
ENSMUSG00000020022	NDUFA12	192.2723975	145.6789633	160.3229779	190.9706681	115.7608381	115.8450251	151.6312223	208.8059394	222.9032179	148.9920806	196.1879971	180.685493
ENSMUSG00000020023	TMCC3	17.47930887	6.621771059	0	0	6.431157673	0	2.637064736	1.288925552	0	0	1.751678546	3.088640905
ENSMUSG00000020024	CEP83	174.7930887	331.0885529	174.8977941	238.7133351	295.8332529	148.0241987	181.9574668	190.7609816	154.4526234	195.852493	215.4564612	199.2173384
ENSMUSG00000020027	SOCS2	0	6.621771059	0	0	0	0	0	2.577851103	1.755143448	0	0	0
ENSMUSG00000020029	NUDT4	122.3551621	92.70479482	51.01185661	71.61400054	57.88041905	57.92251255	102.8455247	81.20230975	71.96088136	73.29449126	85.83224875	86.48194535
ENSMUSG00000020034	TCP11L2	8.739654434	13.24354212	21.86222426	23.87133351	25.72463069	0	21.09651789	24.48958548	35.10286896	28.83717689	29.77853528	20.07616588
ENSMUSG00000020037	RFX4	0	0	7.287408088	0	0	0	1.318532368	2.577851103	0	1.201549037	1.751678546	1.544320453
ENSMUSG00000020038	CRY1	26.2189633	0	7.287408088	11.93566676	0	12.87166946	11.86679131	15.46710662	12.28600414	25.23252978	17.51678546	9.265922716
ENSMUSG00000020048	HSP90B1	830.2671712	787.990756	852.6267462	781.7861725	932.5178625	933.1960355	1033.729377	1203.856465	1037.289778	1143.874683	1073.778949	989.9094102
ENSMUSG00000020056	WASHC3	17.47930887	6.621771059	43.72444853	35.80700027	19.29347302	6.435834728	26.37064736	21.91173438	24.57200827	21.62788267	38.53692801	23.16480679
ENSMUSG00000020059	SYCP3	0	0	0	0	0	0	1.318532368	2.577851103	1.755143448	3.604647111	3.503357092	0
ENSMUSG00000020061	MYBPC1	0	0	0	0	0	0	0	1.288925552	0	0	0	0
ENSMUSG00000020063	SIRT1	8.739654434	33.10885529	21.86222426	41.77483365	32.15578836	12.87166946	22.41505026	43.82346876	54.40944688	34.84492207	35.03357092	43.24097267
ENSMUSG00000020064	HERC4	113.6155076	86.08302377	94.73630514	77.58183392	96.46736509	51.48667782	60.65248893	34.80098989	61.43002068	46.86041244	64.8121062	57.13985675
ENSMUSG00000020069	HNRNPH3	52.4379266	132.4354212	58.2992647	113.3888342	122.1919958	83.66585146	65.9266184	72.17983089	89.51231584	75.69758933	78.82553457	89.57058625
ENSMUSG00000020070	RUFY2	34.95861774	26.48708424	43.72444853	17.90350013	12.86231535	12.87166946	27.68917973	24.48958548	35.10286896	21.62788267	49.04699929	26.2534477
ENSMUSG00000020072	PBLD2	0	0	0	0	0	0	0	1.288925552	0	0	0	0
ENSMUSG00000020074	CCAR1	340.8465229	357.5756372	393.5200367	501.2980038	450.1810371	437.6367615	383.6929191	377.6551866	410.7035668	359.2631621	425.6578867	378.3585109
ENSMUSG00000020075	DDX21	244.7103242	245.0055292	233.1970588	435.6518366	321.5578836	212.382546	340.1813509	309.3421324	287.8435255	270.3485333	287.2752815	299.5981678
ENSMUSG00000020076	DDX50	43.69827217	79.46125271	109.3111213	107.4210008	115.7608381	57.92251255	77.79340971	67.02412869	73.71602481	80.50378548	92.83896294	75.67170218
ENSMUSG00000020077	SRGN	17.47930887	52.97416847	14.57481618	35.80700027	38.58694604	19.30750418	30.32624446	37.378841	26.32715172	50.46505955	33.28189237	23.16480679
ENSMUSG00000020078	VPS26A	69.91723547	52.97416847	72.87408088	71.61400054	83.60504975	45.05084309	76.47487734	76.04660755	82.49174205	60.07745185	70.06714184	83.39330444
ENSMUSG00000020079	SUPV3L1	61.17758104	33.10885529	36.43704044	23.87133351	6.431157673	38.61500837	30.32624446	30.93421324	38.61315585	32.441824	40.28860656	32.43072951
ENSMUSG00000020085	AIFM2	17.47930887	0	0	0	0	0	10.54825894	10.31140441	7.020573791	8.410843259	8.75839273	6.177281811
ENSMUSG00000020086	H2AFY2	0	0	0	0	0	0	1.318532368	0	1.755143448	3.604647111	0	0
ENSMUSG00000020087	TYSND1	17.47930887	0	0	5.967833378	0	25.74333891	11.86679131	10.31140441	14.04114758	19.22478459	15.76510691	15.44320453
ENSMUSG00000020088	SAR1A	104.8758532	99.32656588	131.1733456	143.2280011	64.31157673	96.53752092	94.9343305	94.09156527	115.8394676	90.11617778	96.34232003	100.3808294
ENSMUSG00000020089	PPA1	69.91723547	59.59593953	51.01185661	71.61400054	64.31157673	90.10168619	83.06753918	81.20230975	87.75717239	81.70533452	63.06042765	74.12738173
ENSMUSG00000020090	NPFFR1	0	0	0	0	6.431157673	0	0	0	0	0	0	0
ENSMUSG00000020091	EIF4EBP2	61.17758104	92.70479482	65.58667279	47.74266702	115.7608381	70.79418201	84.38607155	78.62445865	82.49174205	61.27900089	68.31546329	89.57058625
ENSMUSG00000020092	PALD1	0	0	0	0	0	0	0	0	0	1.201549037	0	0
ENSMUSG00000020096	TBATA	0	6.621771059	7.287408088	0	6.431157673	0	0	1.288925552	0	0	1.751678546	0
ENSMUSG00000020097	SGPL1	17.47930887	46.35239741	43.72444853	23.87133351	32.15578836	25.74333891	22.41505026	16.75603217	15.79629103	26.43407881	21.02014255	26.2534477
ENSMUSG00000020100	SLC29A3	8.739654434	0	0	0	0	6.435834728	0	1.288925552	3.510286896	0	0	1.544320453
ENSMUSG00000020101	VSIR	17.47930887	6.621771059	7.287408088	17.90350013	19.29347302	19.30750418	13.18532368	6.444627758	12.28600414	16.82168652	10.51007128	16.98752498
ENSMUSG00000020105	LRIG3	0	0	0	0	0	0	0	0	0	0	1.751678546	0
ENSMUSG00000020107	ANAPC16	69.91723547	52.97416847	43.72444853	23.87133351	38.58694604	45.05084309	52.74129472	45.11239431	22.81686482	38.44956918	42.0402851	37.06369086

ENSMUSG00000020108	DDIT4	78.65688991	52.97416847	51.01185661	47.74266702	64.31157673	57.92251255	81.74900682	78.62445865	96.53288963	72.09294222	87.5839273	83.39330444
ENSMUSG00000020109	DNAJB12	87.39654434	105.9483369	65.58667279	107.4210008	102.8985228	90.10168619	85.70460392	99.24726748	80.7365986	76.89913837	80.57721311	95.74786807
ENSMUSG00000020111	MICU1	0	13.24354212	21.86222426	5.967833378	0	0	6.59266184	10.31140441	12.28600414	3.604647111	3.503357092	13.89888407
ENSMUSG00000020114	CAND1	61.17758104	79.46125271	131.1733456	77.58183392	64.31157673	83.66585146	100.20846	106.9808208	94.77774619	79.30223644	115.610784	89.57058625
ENSMUSG00000020115	TBK1	34.95861774	46.35239741	21.86222426	29.83916689	32.15578836	32.17917364	44.83010051	59.29057538	52.65430344	61.27900089	47.29532074	49.41825449
ENSMUSG00000020116	PNO1	104.8758532	99.32656588	80.16148896	107.4210008	102.8985228	90.10168619	118.6679131	125.0257785	126.3703282	88.91462874	82.32889166	120.4569953
ENSMUSG00000020120	PLEK	0	0	0	0	0	0	1.318532368	1.288925552	0	1.201549037	0	0
ENSMUSG00000020123	AVPR1A	0	0	0	0	0	0	1.318532368	0	0	2.403098074	0	1.544320453
ENSMUSG00000020124	USP15	78.65688991	72.83948165	72.87408088	131.2923343	122.1919958	90.10168619	108.1196542	117.2922252	112.3291807	123.7595508	112.1074269	89.57058625
ENSMUSG00000020128	VPS54	122.3551621	105.9483369	145.7481618	143.2280011	109.3296804	109.4091904	87.02313629	114.7143741	71.96088136	73.29449126	84.08057021	89.57058625
ENSMUSG00000020130	TBC1D15	69.91723547	39.73062635	80.16148896	47.74266702	70.7427344	90.10168619	63.28955366	63.15735203	66.69545102	63.68209896	68.31546329	64.86145901
ENSMUSG00000020131	PCSK4	0	13.24354212	0	5.967833378	0	0	3.955597104	7.73355331	10.53086069	4.806196148	3.503357092	6.177281811
ENSMUSG00000020132	RAB21	78.65688991	66.21771059	65.58667279	53.7105004	96.46736509	90.10168619	75.15634498	82.49123531	70.20573791	78.10068741	71.81882038	63.31713856
ENSMUSG00000020133	2310011J03R	43.69827217	19.86531318	43.72444853	11.93566676	12.86231535	25.74333891	11.86679131	20.62280883	12.28600414	14.41858844	12.26174982	18.53184543
ENSMUSG00000020134	PELI1	113.6155076	92.70479482	116.5985294	89.51750067	64.31157673	77.23001673	80.43047445	85.06908641	86.00202895	115.3487076	59.55707056	72.58306128
ENSMUSG00000020135	APC2	0	0	7.287408088	0	0	0	1.318532368	0	0	0	0	0
ENSMUSG00000020137	THAP2	17.47930887	6.621771059	29.14963235	23.87133351	32.15578836	12.87166946	32.9633092	25.77851103	36.85801241	27.63562785	17.51678546	27.79776815
ENSMUSG00000020140	LGR5	166.0534342	59.59593953	65.58667279	53.7105004	77.17389207	57.92251255	31.64477683	36.08991545	56.16459033	43.25576533	31.53021383	32.43072951
ENSMUSG00000020142	SLC1A4	8.739654434	13.24354212	14.57481618	59.67833378	25.72463069	51.48667782	30.32624446	37.378841	28.08229517	49.26351052	31.53021383	43.24097267
ENSMUSG00000020143	DOCK2	69.91723547	66.21771059	109.3111213	113.3888342	90.03620742	102.9733556	105.4825894	109.5586719	80.7365986	94.92237392	91.08728439	95.74786807
ENSMUSG00000020149	RAB1	131.0948165	178.7878186	174.8977941	119.3566676	154.3477841	173.7675376	259.7508765	246.1847804	266.7818041	270.3485333	203.1947113	219.2935043
ENSMUSG00000020150	GAMT	0	0	0	0	0	0	0	0	0	0	0	1.544320453
ENSMUSG00000020152	ACTR2	489.4206483	490.0110584	437.2444853	537.105004	469.4745101	456.9442657	464.1233935	488.5027841	400.1727061	477.0149677	383.6176016	429.3210858
ENSMUSG00000020153	NDUFS7	113.6155076	86.08302377	87.44889705	107.4210008	77.17389207	102.9733556	94.9343305	94.09156527	86.00202895	100.9301191	108.6040698	123.5456362
ENSMUSG00000020154	PTPRB	0	0	0	0	0	0	0	0	0	0	1.751678546	0
ENSMUSG00000020155	KCNMB1	0	26.48708424	14.57481618	29.83916689	19.29347302	0	23.73358262	12.88925552	28.08229517	26.43407881	22.7718211	10.81024317
ENSMUSG00000020156	MUM1	43.69827217	26.48708424	94.73630514	29.83916689	19.29347302	64.35834728	30.32624446	39.9566921	61.43002068	49.26351052	40.28860656	44.78529313
ENSMUSG00000020163	UQCR11	157.3137798	125.8136501	131.1733456	155.1636678	115.7608381	115.8450251	189.868661	179.1606517	194.8209227	141.7827864	199.6913542	183.7741339
ENSMUSG00000020166	CNOT2	34.95861774	46.35239741	43.72444853	53.7105004	38.58694604	51.48667782	71.20074787	79.9133842	68.45059447	74.4960403	64.8121062	58.6841772
ENSMUSG00000020167	TCF3	157.3137798	218.5184449	211.3348345	167.0993346	154.3477841	128.7166946	150.31269	164.9824706	163.2283407	150.1936296	141.8859622	151.3434044
ENSMUSG00000020170	FRS2	43.69827217	19.86531318	21.86222426	35.80700027	12.86231535	45.05084309	22.41505026	12.88925552	8.775717239	21.62788267	22.7718211	27.79776815
ENSMUSG00000020171	YEATS4	43.69827217	66.21771059	43.72444853	107.4210008	38.58694604	96.53752092	80.43047445	82.49123531	70.20573791	86.51153067	73.57049893	81.84898399
ENSMUSG00000020175	RAB36	0	6.621771059	0	0	0	0	1.318532368	1.288925552	0	0	0	1.544320453
ENSMUSG00000020176	GRB10	8.739654434	0	7.287408088	11.93566676	0	0	5.274129472	1.288925552	0	4.806196148	3.503357092	0
ENSMUSG00000020178	ADORA2A	17.47930887	6.621771059	21.86222426	5.967833378	0	12.87166946	10.54825894	9.022478862	12.28600414	6.007745185	21.02014255	10.81024317
ENSMUSG00000020180	SNRPD3	550.5982293	509.8763715	480.9689338	417.7483365	488.7679831	424.765092	650.0364574	674.1080635	645.8927888	609.1853618	681.4029544	687.2226014
ENSMUSG00000020182	DDC	78.65688991	46.35239741	7.287408088	23.87133351	12.86231535	25.74333891	40.87450341	42.5345432	42.12344275	45.65886341	52.55035638	55.5955363
ENSMUSG00000020183	CPM	0	0	7.287408088	0	0	0	0	0	0	0	0	0
ENSMUSG00000020184	MDM2	61.17758104	66.21771059	36.43704044	65.64616716	77.17389207	90.10168619	73.83781261	72.17983089	89.51231584	79.30223644	85.83224875	77.21602263
ENSMUSG00000020185	E2F7	8.739654434	6.621771059	7.287408088	0	0	6.435834728	0	3.866776655	1.755143448	3.604647111	0	1.544320453
ENSMUSG00000020186	CSRP2	0	0	0	0	6.431157673	0	0	0	0	0	0	0
ENSMUSG00000020189	OSBPL8	61.17758104	66.21771059	131.1733456	65.64616716	57.88041905	51.48667782	63.28955366	60.57950093	57.91973378	64.883648	63.06042765	55.5955363
ENSMUSG00000020190	MKNK2	157.3137798	158.9225054	167.610386	190.9706681	186.5035725	263.8692238	167.4536107	180.4495772	193.0657793	183.8370027	227.718211	214.6605429
ENSMUSG00000020191	SPATA48	0	0	0	0	0	0	0	1.288925552	0	1.201549037	0	3.088640905
ENSMUSG00000020193	ZPBP	8.739654434	0	0	0	0	0	0	1.288925552	1.755143448	1.201549037	0	0

ENSMUSG00000020196	CABIN1	113.6155076	59.59593953	87.44889705	65.64616716	109.3296804	57.92251255	90.97873339	97.95834193	89.51231584	98.52702104	75.32217748	66.40577946
ENSMUSG00000020198	AP3D1	96.13619877	119.1918791	65.58667279	161.1315012	180.0724148	141.588364	85.70460392	82.49123531	122.8600414	98.52702104	126.1208553	97.29218852
ENSMUSG00000020205	PHLDA1	218.4913609	245.0055292	211.3348345	173.067168	289.4020953	128.7166946	192.5057257	184.3163539	224.6583613	177.8292575	196.1879971	191.4957361
ENSMUSG00000020211	SF3A2	113.6155076	79.46125271	58.2992647	83.54966729	70.7427344	90.10168619	71.20074787	92.80263972	100.0431765	67.28674607	106.8523913	83.39330444
ENSMUSG00000020212	MDM1	17.47930887	19.86531318	7.287408088	17.90350013	38.58694604	38.61500837	25.05211499	29.64528769	26.32715172	40.85266726	19.26846401	29.3420886
ENSMUSG00000020216	JSRP1	0	0	0	0	0	0	0	1.288925552	0	0	0	0
ENSMUSG00000020219	TIMM13	61.17758104	105.9483369	116.5985294	77.58183392	128.6231535	77.23001673	127.8976397	106.9808208	129.8806151	121.3564527	112.1074269	140.5331612
ENSMUSG00000020220	VPS13D	0	19.86531318	21.86222426	5.967833378	19.29347302	0	21.09651789	29.64528769	21.06172137	19.22478459	22.7718211	13.89888407
ENSMUSG00000020224	LLPH	43.69827217	46.35239741	43.72444853	77.58183392	38.58694604	19.30750418	54.05982709	48.97917096	63.18516412	72.09294222	54.30203492	44.78529313
ENSMUSG00000020225	TMBIM4	17.47930887	26.48708424	21.86222426	23.87133351	25.72463069	25.74333891	35.60037394	29.64528769	24.57200827	26.43407881	14.01342837	24.70912724
ENSMUSG00000020228	HELB	34.95861774	33.10885529	72.87408088	59.67833378	19.29347302	57.92251255	46.14863288	48.97917096	42.12344275	57.67435378	57.80539202	30.88640905
ENSMUSG00000020230	PRMT2	0	0	0	0	0	6.435834728	6.59266184	2.577851103	0	2.403098074	1.751678546	0
ENSMUSG00000020231	DIP2A	8.739654434	0	14.57481618	11.93566676	6.431157673	12.87166946	7.911194208	14.17818107	17.55143448	9.612392296	5.255035638	12.35456362
ENSMUSG00000020232	HMG20B	78.65688991	66.21771059	21.86222426	59.67833378	64.31157673	70.79418201	60.65248893	51.55702207	47.38887309	56.47280474	71.81882038	54.05121584
ENSMUSG00000020234	4930404N11R	8.739654434	0	0	0	6.431157673	12.87166946	1.318532368	0	1.755143448	1.201549037	0	0
ENSMUSG00000020235	FZR1	34.95861774	26.48708424	7.287408088	35.80700027	51.44926138	45.05084309	17.14092078	19.33388327	21.06172137	28.83717689	19.26846401	20.07616588
ENSMUSG00000020238	NCLN	78.65688991	86.08302377	36.43704044	53.7105004	70.7427344	32.17917364	47.46716525	52.84594762	36.85801241	49.26351052	56.05371347	44.78529313
ENSMUSG00000020246	HCFC2	8.739654434	26.48708424	0	23.87133351	19.29347302	32.17917364	15.82238842	18.04495772	21.06172137	28.83717689	12.26174982	10.81024317
ENSMUSG00000020248	NFYB	61.17758104	13.24354212	58.2992647	35.80700027	19.29347302	38.61500837	39.55597104	51.55702207	36.85801241	39.65111822	45.5436422	44.78529313
ENSMUSG00000020250	TXNRD1	61.17758104	112.570108	153.0355698	137.2601677	102.8985228	135.1525293	146.3570928	149.515364	154.4526234	147.7905316	94.59064148	154.4320453
ENSMUSG00000020251	GLT8D2	0	0	0	0	6.431157673	0	0	0	1.755143448	0	0	0
ENSMUSG00000020253	PPM1M	26.2189633	26.48708424	14.57481618	11.93566676	12.86231535	32.17917364	31.64477683	29.64528769	33.34772551	22.8294317	17.51678546	15.44320453
ENSMUSG00000020255	D10WSU102E	113.6155076	59.59593953	80.16148896	71.61400054	57.88041905	45.05084309	72.51928024	59.29057538	66.69545102	73.29449126	80.57721311	63.31713856
ENSMUSG00000020256	ALDH1L2	0	0	0	5.967833378	0	6.435834728	0	1.288925552	0	0	0	1.544320453
ENSMUSG00000020257	WDR82	52.4379266	86.08302377	65.58667279	47.74266702	64.31157673	25.74333891	65.9266184	63.15735203	54.40944688	72.09294222	54.30203492	91.11490671
ENSMUSG00000020258	GLYCTK	8.739654434	0	0	5.967833378	0	0	2.637064736	0	0	1.201549037	1.751678546	3.088640905
ENSMUSG00000020260	POFUT2	43.69827217	59.59593953	72.87408088	41.77483365	25.72463069	32.17917364	25.05211499	18.04495772	38.61315585	24.03098074	40.28860656	35.51937041
ENSMUSG00000020261	SLC36A1	26.2189633	19.86531318	0	47.74266702	19.29347302	6.435834728	9.229726576	3.866776655	14.04114758	8.410843259	21.02014255	9.265922716
ENSMUSG00000020262	ADARB1	0	19.86531318	7.287408088	17.90350013	12.86231535	32.17917364	27.68917973	18.04495772	22.81686482	15.62013748	19.26846401	13.89888407
ENSMUSG00000020263	APPL2	69.91723547	46.35239741	29.14963235	41.77483365	25.72463069	51.48667782	46.14863288	43.82346876	36.85801241	54.06970667	47.29532074	37.06369086
ENSMUSG00000020265	SUMO3	113.6155076	19.86531318	94.73630514	83.54966729	70.7427344	64.35834728	58.01542419	68.31305424	70.20573791	60.07745185	57.80539202	50.96257494
ENSMUSG00000020267	HINT1	576.8171926	569.4723111	575.7052389	471.4588369	578.8041905	534.1742824	573.5615801	653.4852547	572.176764	543.1001647	639.3626693	662.5134742
ENSMUSG00000020268	LYRM7	0	6.621771059	0	11.93566676	0	12.87166946	6.59266184	6.444627758	10.53086069	0	5.255035638	12.35456362
ENSMUSG00000020271	FBXW11	8.739654434	13.24354212	7.287408088	47.74266702	6.431157673	32.17917364	34.28184157	30.93421324	29.83743861	32.441824	31.53021383	21.62048634
ENSMUSG00000020272	STK10	78.65688991	119.1918791	72.87408088	77.58183392	115.7608381	64.35834728	64.60808603	76.04660755	86.00202895	98.52702104	77.07385602	100.3808294
ENSMUSG00000020273	PAPOLG	8.739654434	6.621771059	14.57481618	0	25.72463069	25.74333891	19.77798552	14.17818107	19.30657793	16.82168652	12.26174982	13.89888407
ENSMUSG00000020275	REL	0	19.86531318	7.287408088	11.93566676	12.86231535	12.87166946	15.82238842	18.04495772	10.53086069	15.62013748	15.76510691	15.44320453
ENSMUSG00000020277	PFKL	61.17758104	26.48708424	109.3111213	65.64616716	51.44926138	77.23001673	79.11194208	70.89090534	47.38887309	56.47280474	50.79867783	58.6841772
ENSMUSG00000020279	IL9R	104.8758532	105.9483369	87.44889705	83.54966729	77.17389207	90.10168619	97.57139523	122.4479274	107.0637503	105.7363153	77.07385602	100.3808294
ENSMUSG00000020280	PUS10	34.95861774	72.83948165	29.14963235	35.80700027	51.44926138	19.30750418	48.78569762	39.9566921	36.85801241	50.46505955	50.79867783	30.88640905
ENSMUSG00000020283	PEX13	34.95861774	66.21771059	29.14963235	35.80700027	6.431157673	12.87166946	26.37064736	24.48958548	17.55143448	26.43407881	22.7718211	37.06369086
ENSMUSG00000020284	1810043G02R	8.739654434	6.621771059	14.57481618	11.93566676	0	6.435834728	5.274129472	9.022478862	7.020573791	7.209294222	10.51007128	10.81024317
ENSMUSG00000020286	1700093K21R	0	0	7.287408088	0	0	0	0	0	0	1.201549037	1.751678546	1.544320453
ENSMUSG00000020287	MPG	61.17758104	13.24354212	21.86222426	11.93566676	25.72463069	25.74333891	19.77798552	18.04495772	19.30657793	21.62788267	24.52349964	20.07616588
ENSMUSG00000020288	AHSA2	61.17758104	86.08302377	94.73630514	119.3566676	83.60504975	83.66585146	134.4903015	114.7143741	98.28803308	122.5580018	136.6309266	122.0013158

ENSMUSG00000020289	NPRL3	8.739654434	6.621771059	7.287408088	17.90350013	19.29347302	12.87166946	17.14092078	5.155702207	7.020573791	9.612392296	7.006714184	10.81024317
ENSMUSG00000020290	XPO1	288.4085963	271.4926134	298.7837316	262.5846686	237.9528339	315.3559017	338.8628186	308.0532068	305.3949599	295.5810631	327.5638881	333.5732178
ENSMUSG00000020297	NSG2	0	0	0	0	0	0	1.318532368	0	0	0	0	0
ENSMUSG00000020300	CPEB4	17.47930887	13.24354212	29.14963235	35.80700027	45.01810371	12.87166946	26.37064736	25.77851103	15.79629103	26.43407881	26.27517819	30.88640905
ENSMUSG00000020303	STC2	8.739654434	19.86531318	7.287408088	11.93566676	0	19.30750418	6.59266184	10.31140441	22.81686482	13.21703941	10.51007128	4.632961358
ENSMUSG00000020305	ASB3	17.47930887	6.621771059	0	11.93566676	6.431157673	12.87166946	14.50385605	14.17818107	14.04114758	28.83717689	14.01342837	3.088640905
ENSMUSG00000020307	CDC34	104.8758532	92.70479482	153.0355698	101.4531674	180.0724148	141.588364	129.2161721	134.0482574	143.9217627	157.4029238	164.6577833	146.710443
ENSMUSG00000020308	TPGS1	17.47930887	13.24354212	7.287408088	11.93566676	19.29347302	12.87166946	10.54825894	12.88925552	15.79629103	10.81394133	12.26174982	4.632961358
ENSMUSG00000020309	CHAC2	8.739654434	0	0	5.967833378	6.431157673	19.30750418	11.86679131	7.73355331	5.265430344	10.81394133	5.255035638	20.07616588
ENSMUSG00000020311	ERLEC1	17.47930887	39.73062635	43.72444853	23.87133351	32.15578836	19.30750418	29.0077121	34.80098989	26.32715172	30.03872593	38.53692801	38.60801132
ENSMUSG00000020315	SPTBN1	183.5327431	152.3007344	240.4844669	185.0028347	205.7970455	135.1525293	168.7721431	177.8717261	159.7180538	197.0540421	168.1611404	186.8627748
ENSMUSG00000020319	WDPCP	0	6.621771059	0	5.967833378	0	0	0	3.866776655	0	1.201549037	1.751678546	0
ENSMUSG00000020321	MDH1	227.2310153	264.8708424	255.0592831	179.0350013	270.1086223	167.3317029	315.129236	369.9216333	365.0698372	308.7981025	313.5504597	277.9776815
ENSMUSG00000020323	PRSS57	0	0	0	0	0	0	5.274129472	7.73355331	1.755143448	2.403098074	1.751678546	1.544320453
ENSMUSG00000020325	FSTL3	0	0	0	5.967833378	0	0	0	2.577851103	3.510286896	1.201549037	1.751678546	0
ENSMUSG00000020326	CCNG1	43.69827217	33.10885529	43.72444853	23.87133351	32.15578836	51.48667782	26.37064736	28.35636214	38.61315585	37.24802015	43.79196365	33.97504996
ENSMUSG00000020327	FGF22	17.47930887	19.86531318	14.57481618	0	6.431157673	6.435834728	6.59266184	7.73355331	7.020573791	9.612392296	8.75839273	6.177281811
ENSMUSG00000020328	NUDCD2	367.0654862	218.5184449	233.1970588	316.295169	424.4564064	263.8692238	341.4998833	393.1222933	379.1109847	331.6275342	341.5773165	271.8003997
ENSMUSG00000020329	POLRMT	8.739654434	19.86531318	7.287408088	11.93566676	38.58694604	12.87166946	29.0077121	29.64528769	33.34772551	18.02323556	24.52349964	26.2534477
ENSMUSG00000020330	HMMR	104.8758532	112.570108	167.610386	101.4531674	141.4854688	154.4600335	119.9864455	137.915034	126.3703282	115.3487076	134.879248	106.5581112
ENSMUSG00000020331	HCN2	0	0	14.57481618	5.967833378	0	0	1.318532368	6.444627758	7.020573791	2.403098074	5.255035638	6.177281811
ENSMUSG00000020333	ACSL6	0	0	0	0	6.431157673	6.435834728	0	0	0	0	0	0
ENSMUSG00000020340	CYFIP2	131.0948165	119.1918791	167.610386	125.3245009	199.3658879	90.10168619	156.9053518	145.6485873	186.0452055	156.2013748	126.1208553	134.3558794
ENSMUSG00000020346	MGAT1	26.2189633	52.97416847	14.57481618	23.87133351	19.29347302	25.74333891	30.32624446	38.66776655	22.81686482	27.63562785	43.79196365	37.06369086
ENSMUSG00000020349	PPP2CA	113.6155076	112.570108	109.3111213	113.3888342	77.17389207	122.2808598	116.0308484	137.915034	126.3703282	174.2246104	115.610784	117.3683544
ENSMUSG00000020354	SGCD	0	0	0	0	0	0	0	0	0	0	0	1.544320453
ENSMUSG00000020358	HNRNPAB	454.4620306	549.6069979	415.382261	405.8126697	411.5940911	469.8159351	618.3916806	603.2171582	563.4010468	607.9838127	627.1009195	593.0190538
ENSMUSG00000020359	PHYKPL	104.8758532	158.9225054	123.8859375	137.2601677	109.3296804	199.5108766	121.3049779	106.9808208	119.3497545	145.3874335	136.6309266	118.9126749
ENSMUSG00000020361	HSPA4	235.9706697	297.9796976	225.9096507	298.3916689	282.9709376	257.4333891	258.4323441	326.0981646	317.6809641	326.8213381	324.060531	308.8640905
ENSMUSG00000020362	CNOT6	139.8344709	192.0313607	116.5985294	95.48533405	180.0724148	238.1258849	183.2759992	145.6485873	152.69748	161.007571	154.147712	148.2547635
ENSMUSG00000020366	MAPK9	8.739654434	39.73062635	21.86222426	23.87133351	32.15578836	12.87166946	23.73358262	38.66776655	26.32715172	30.03872593	36.78524947	21.62048634
ENSMUSG00000020368	CANX	454.4620306	496.6328294	561.1304227	513.2336705	501.6302985	534.1742824	603.8878245	589.0389771	623.075924	623.6039502	565.7921703	552.8667221
ENSMUSG00000020372	RACK1	1503.220563	1648.820994	1712.540901	1641.154179	1633.514049	1441.626979	1984.391214	2118.993607	2213.235888	1884.02889	2072.23572	2151.238391
ENSMUSG00000020375	RUFY1	0	6.621771059	0	5.967833378	0	0	0	5.155702207	0	2.403098074	0	6.177281811
ENSMUSG00000020380	RAD50	157.3137798	158.9225054	233.1970588	292.4238355	270.1086223	225.2542155	156.9053518	175.293875	145.6769062	157.4029238	157.6510691	143.6218021
ENSMUSG00000020381	MRNIP	8.739654434	6.621771059	7.287408088	5.967833378	12.86231535	0	3.955597104	11.60032996	12.28600414	10.81394133	10.51007128	1.544320453
ENSMUSG00000020385	CLK4	122.3551621	59.59593953	94.73630514	71.61400054	38.58694604	96.53752092	52.74129472	79.9133842	114.0843241	97.325472	80.57721311	66.40577946
ENSMUSG00000020386	SAR1B	26.2189633	26.48708424	72.87408088	35.80700027	38.58694604	38.61500837	79.11194208	69.60197979	80.7365986	48.06196148	56.05371347	60.22849765
ENSMUSG00000020387	JADE2	34.95861774	0	14.57481618	23.87133351	6.431157673	6.435834728	7.911194208	2.577851103	14.04114758	9.612392296	10.51007128	15.44320453
ENSMUSG00000020388	PDLIM4	0	0	0	0	0	0	0	0	0	0	1.751678546	0
ENSMUSG00000020389	CDKL3	0	6.621771059	7.287408088	0	6.431157673	6.435834728	2.637064736	6.444627758	0	7.209294222	10.51007128	4.632961358
ENSMUSG00000020390	UBE2B	227.2310153	231.7619871	160.3229779	202.9063349	173.6412572	122.2808598	250.5211499	230.7176737	217.6377875	272.7516314	262.7517819	208.4832611
ENSMUSG00000020392	CDKN2AIPNL	52.4379266	66.21771059	58.2992647	47.74266702	77.17389207	70.79418201	80.43047445	92.80263972	71.96088136	74.4960403	80.57721311	66.40577946
ENSMUSG00000020393	KREMEN1	69.91723547	52.97416847	36.43704044	35.80700027	57.88041905	25.74333891	31.64477683	34.80098989	36.85801241	22.8294317	22.7718211	32.43072951
ENSMUSG00000020395	ITK	113.6155076	72.83948165	94.73630514	125.3245009	167.2100995	115.8450251	85.70460392	99.24726748	103.5534634	94.92237392	96.34232003	60.22849765

ENSMUSG00000020397	MED7	69.91723547	26.48708424	7.287408088	71.61400054	32.15578836	19.30750418	55.37835946	48.97917096	42.12344275	43.25576533	42.0402851	33.97504996
ENSMUSG00000020399	HAVCR2	0	0	0	0	6.431157673	0	0	0	1.755143448	1.201549037	0	0
ENSMUSG00000020400	TNIP1	8.739654434	39.73062635	21.86222426	23.87133351	19.29347302	12.87166946	23.73358262	25.77851103	19.30657793	32.441824	19.26846401	23.16480679
ENSMUSG00000020401	FAM71B	17.47930887	6.621771059	21.86222426	5.967833378	19.29347302	25.74333891	9.229726576	12.88925552	8.775717239	16.82168652	24.52349964	9.265922716
ENSMUSG00000020402	VDAC1	279.6689419	324.4667819	262.3466912	262.5846686	218.6593609	225.2542155	319.0848331	332.5427923	394.9072758	316.0073967	360.8457805	355.1937041
ENSMUSG00000020409	SLU7	17.47930887	52.97416847	72.87408088	47.74266702	51.44926138	57.92251255	63.28955366	51.55702207	52.65430344	74.4960403	54.30203492	52.50689539
ENSMUSG00000020412	ASCC2	17.47930887	19.86531318	21.86222426	17.90350013	45.01810371	25.74333891	26.37064736	20.62280883	19.30657793	21.62788267	21.02014255	23.16480679
ENSMUSG00000020413	HUS1	17.47930887	13.24354212	7.287408088	11.93566676	19.29347302	19.30750418	18.45945315	18.04495772	24.57200827	21.62788267	15.76510691	16.98752498
ENSMUSG00000020415	PTTG1	0	6.621771059	7.287408088	0	6.431157673	12.87166946	17.14092078	15.46710662	8.775717239	13.21703941	17.51678546	9.265922716
ENSMUSG00000020420	ZFP607A	17.47930887	6.621771059	0	11.93566676	12.86231535	19.30750418	3.955597104	2.577851103	7.020573791	10.81394133	5.255035638	4.632961358
ENSMUSG00000020423	BTG2	17.47930887	0	14.57481618	0	19.29347302	6.435834728	0	0	0	0	0	0
ENSMUSG00000020424	GATSL3	0	0	0	0	0	6.435834728	1.318532368	1.288925552	1.755143448	0	1.751678546	1.544320453
ENSMUSG00000020430	PES1	17.47930887	26.48708424	29.14963235	29.83916689	38.58694604	6.435834728	19.77798552	32.22313879	31.59258206	25.23252978	38.53692801	26.2534477
ENSMUSG00000020431	ADCY1	0	19.86531318	0	5.967833378	6.431157673	6.435834728	11.86679131	3.866776655	5.265430344	4.806196148	7.006714184	1.544320453
ENSMUSG00000020432	TCN2	0	0	0	0	6.431157673	0	0	0	0	1.201549037	0	0
ENSMUSG00000020437	MYO1G	166.0534342	198.6531318	174.8977941	167.0993346	154.3477841	122.2808598	172.7277402	170.1381728	168.493771	170.6199633	182.1745688	165.2422884
ENSMUSG00000020440	ARF5	559.3378838	529.7416847	459.1067095	459.5231701	495.1991408	540.6101171	345.4554804	400.8558466	417.7241406	386.8987899	404.6377441	469.4734176
ENSMUSG00000020441	2310033P09F	104.8758532	119.1918791	109.3111213	53.7105004	102.8985228	289.6125627	93.61579813	94.09156527	47.38887309	147.7905316	89.33560584	117.3683544
ENSMUSG00000020444	GUK1	34.95861774	33.10885529	21.86222426	17.90350013	25.72463069	12.87166946	26.37064736	32.22313879	26.32715172	22.8294317	35.03357092	37.06369086
ENSMUSG00000020447	NPC1L1	0	0	14.57481618	5.967833378	0	0	1.318532368	3.866776655	5.265430344	2.403098074	0	3.088640905
ENSMUSG00000020448	RNF185	8.739654434	6.621771059	7.287408088	17.90350013	12.86231535	6.435834728	11.86679131	15.46710662	24.57200827	10.81394133	15.76510691	15.44320453
ENSMUSG00000020451	LIMK2	8.739654434	19.86531318	29.14963235	29.83916689	32.15578836	38.61500837	27.68917973	25.77851103	35.10286896	32.441824	43.79196365	38.60801132
ENSMUSG00000020453	PATZ1	8.739654434	33.10885529	21.86222426	29.83916689	25.72463069	25.74333891	48.78569762	50.26809651	38.61315585	54.06970667	43.79196365	37.06369086
ENSMUSG00000020454	EIF4ENIF1	96.13619877	66.21771059	51.01185661	59.67833378	96.46736509	64.35834728	68.56368314	82.49123531	66.69545102	57.67435378	85.83224875	46.32961358
ENSMUSG00000020455	TRIM11	52.4379266	0	7.287408088	29.83916689	25.72463069	12.87166946	15.82238842	16.75603217	12.28600414	25.23252978	24.52349964	13.89888407
ENSMUSG00000020456	OGDH	148.5741254	66.21771059	109.3111213	83.54966729	83.60504975	70.79418201	89.66020102	90.22478862	80.7365986	86.51153067	68.31546329	78.76034309
ENSMUSG00000020457	DRG1	43.69827217	59.59593953	80.16148896	29.83916689	64.31157673	25.74333891	39.55597104	45.11239431	47.38887309	37.24802015	59.55707056	49.41825449
ENSMUSG00000020458	RTN4	78.65688991	59.59593953	65.58667279	77.58183392	77.17389207	90.10168619	89.66020102	94.09156527	94.77774619	98.52702104	78.82553457	77.21602263
ENSMUSG00000020459	MTIF2	43.69827217	52.97416847	65.58667279	71.61400054	57.88041905	38.61500837	50.10422998	69.60197979	68.45059447	62.48054992	54.30203492	72.58306128
ENSMUSG00000020460	RPS27A	1581.877453	1456.789633	1639.66682	1993.256348	1729.981414	1705.496203	1202.50152	1255.413487	1295.295865	1094.611173	1334.779052	1317.305346
ENSMUSG00000020461	CLHC1	0	0	0	5.967833378	0	6.435834728	0	0	0	2.403098074	0	0
ENSMUSG00000020462	CFAP36	52.4379266	92.70479482	72.87408088	41.77483365	51.44926138	45.05084309	68.56368314	58.00164982	63.18516412	57.67435378	59.55707056	64.86145901
ENSMUSG00000020463	PPP4R3B	52.4379266	92.70479482	80.16148896	77.58183392	109.3296804	45.05084309	110.7567189	77.3355331	70.20573791	97.325472	92.83896294	66.40577946
ENSMUSG00000020464	PNPT1	96.13619877	79.46125271	102.0237132	77.58183392	77.17389207	122.2808598	101.5269923	119.8700763	91.26745929	96.12392296	141.8859622	100.3808294
ENSMUSG00000020471	POLD2	69.91723547	99.32656588	80.16148896	125.3245009	115.7608381	77.23001673	65.9266184	105.6918952	87.75717239	75.69758933	96.34232003	89.57058625
ENSMUSG00000020472	ZKSCAN17	17.47930887	13.24354212	7.287408088	0	0	0	9.229726576	20.62280883	22.81686482	9.612392296	10.51007128	9.265922716
ENSMUSG00000020473	AEBP1	0	0	0	0	0	0	0	1.288925552	0	0	0	0
ENSMUSG00000020474	POLM	8.739654434	19.86531318	29.14963235	0	19.29347302	12.87166946	9.229726576	9.022478862	12.28600414	6.007745185	14.01342837	10.81024317
ENSMUSG00000020476	DBNL	43.69827217	52.97416847	14.57481618	53.7105004	45.01810371	6.435834728	47.46716525	43.82346876	63.18516412	42.0542163	40.28860656	55.5955363
ENSMUSG00000020477	MRPS24	61.17758104	52.97416847	43.72444853	53.7105004	64.31157673	83.66585146	63.28955366	64.44627758	82.49174205	46.86041244	77.07385602	80.30466354
ENSMUSG00000020482	CCDC117	26.2189633	19.86531318	21.86222426	35.80700027	25.72463069	19.30750418	44.83010051	51.55702207	47.38887309	36.04647111	36.78524947	32.43072951
ENSMUSG00000020483	DYNLL2	113.6155076	112.570108	58.2992647	119.3566676	141.4854688	83.66585146	152.9497547	141.7818107	138.6563324	141.7827864	171.6644975	177.5968521
ENSMUSG00000020484	XBP1	34.95861774	66.21771059	29.14963235	35.80700027	51.44926138	38.61500837	42.19303578	48.97917096	42.12344275	36.04647111	54.30203492	32.43072951
ENSMUSG00000020485	SUPT4A	8.739654434	13.24354212	29.14963235	5.967833378	6.431157673	6.435834728	35.60037394	24.48958548	31.59258206	24.03098074	29.77853528	37.06369086
ENSMUSG00000020486	38231	0	0	0	5.967833378	0	0	1.318532368	1.288925552	3.510286896	1.201549037	0	4.632961358

ENSMUSG00000020491	2810021J22R	0	0	0	5.967833378	6.431157673	12.87166946	2.637064736	5.155702207	8.775717239	3.604647111	3.503357092	3.088640905
ENSMUSG00000020492	SKA2	43.69827217	105.9483369	94.73630514	83.54966729	57.88041905	77.23001673	72.51928024	78.62445865	101.79832	75.69758933	94.59064148	72.58306128
ENSMUSG00000020493	PRR11	43.69827217	26.48708424	21.86222426	47.74266702	19.29347302	6.435834728	17.14092078	20.62280883	28.08229517	25.23252978	15.76510691	20.07616588
ENSMUSG00000020495	SMG8	8.739654434	13.24354212	14.57481618	5.967833378	6.431157673	12.87166946	10.54825894	12.88925552	12.28600414	20.42633363	17.51678546	30.88640905
ENSMUSG00000020496	RNF187	26.2189633	59.59593953	65.58667279	47.74266702	51.44926138	64.35834728	63.28955366	59.29057538	80.7365986	64.883648	78.82553457	67.95009992
ENSMUSG00000020513	TUBD1	0	6.621771059	14.57481618	5.967833378	0	6.435834728	3.955597104	9.022478862	7.020573791	6.007745185	3.503357092	3.088640905
ENSMUSG00000020514	MRPL22	17.47930887	26.48708424	14.57481618	23.87133351	45.01810371	12.87166946	19.77798552	28.35636214	36.85801241	31.24027496	29.77853528	32.43072951
ENSMUSG00000020515	CNOT8	69.91723547	66.21771059	29.14963235	47.74266702	64.31157673	45.05084309	58.01542419	50.26809651	63.18516412	48.06196148	43.79196365	46.32961358
ENSMUSG00000020516	RPS6KB1	148.5741254	178.7878186	153.0355698	131.2923343	147.9166265	173.7675376	183.2759992	164.9824706	157.9629103	175.4261594	169.912819	185.3184543
ENSMUSG00000020519	SAP30L	52.4379266	39.73062635	43.72444853	41.77483365	51.44926138	25.74333891	48.78569762	46.40131986	56.16459033	40.85266726	40.28860656	44.78529313
ENSMUSG00000020520	GALNT10	0	0	0	0	0	0	0	0	0	0	1.751678546	0
ENSMUSG00000020521	RNFT1	61.17758104	33.10885529	36.43704044	53.7105004	64.31157673	38.61500837	80.43047445	51.55702207	73.71602481	78.10068741	80.57721311	66.40577946
ENSMUSG00000020522	MFAP3	17.47930887	26.48708424	58.2992647	59.67833378	32.15578836	12.87166946	23.73358262	37.378841	22.81686482	33.64337304	29.77853528	30.88640905
ENSMUSG00000020523	FAM114A2	87.39654434	59.59593953	58.2992647	53.7105004	51.44926138	45.05084309	44.83010051	47.69024541	38.61315585	62.48054992	50.79867783	49.41825449
ENSMUSG00000020525	PPM1D	43.69827217	6.621771059	14.57481618	23.87133351	32.15578836	19.30750418	21.09651789	21.91173438	21.06172137	25.23252978	19.26846401	16.98752498
ENSMUSG00000020526	ZNHIT3	26.2189633	33.10885529	43.72444853	35.80700027	45.01810371	19.30750418	43.51156814	59.29057538	36.85801241	54.06970667	47.29532074	30.88640905
ENSMUSG00000020527	MYO19	17.47930887	26.48708424	14.57481618	17.90350013	25.72463069	12.87166946	23.73358262	23.20065993	19.30657793	24.03098074	14.01342837	20.07616588
ENSMUSG00000020528	PRPSAP2	17.47930887	0	14.57481618	11.93566676	6.431157673	32.17917364	29.0077121	28.35636214	22.81686482	33.64337304	19.26846401	24.70912724
ENSMUSG00000020530	GGNBP2	279.6689419	198.6531318	174.8977941	137.2601677	225.0905185	199.5108766	187.2315963	163.6935451	163.2283407	192.2478459	178.6712117	186.8627748
ENSMUSG00000020532	ACACA	43.69827217	46.35239741	65.58667279	53.7105004	45.01810371	38.61500837	40.87450341	51.55702207	66.69545102	38.44956918	38.53692801	29.3420886
ENSMUSG00000020534	SHMT1	78.65688991	72.83948165	58.2992647	59.67833378	109.3296804	57.92251255	85.70460392	101.8251186	117.594611	78.10068741	92.83896294	95.74786807
ENSMUSG00000020536	LLGL1	26.2189633	33.10885529	14.57481618	11.93566676	51.44926138	6.435834728	18.45945315	21.91173438	15.79629103	24.03098074	22.7718211	18.53184543
ENSMUSG00000020537	DRG2	26.2189633	13.24354212	43.72444853	11.93566676	25.72463069	25.74333891	36.9189063	36.08991545	50.89915999	28.83717689	29.77853528	29.3420886
ENSMUSG00000020538	SREBF1	52.4379266	79.46125271	109.3111213	89.51750067	70.7427344	77.23001673	101.5269923	109.5586719	121.1048979	102.1316681	87.5839273	137.4445203
ENSMUSG00000020541	TOM1L1	0	0	7.287408088	0	6.431157673	6.435834728	3.955597104	7.73355331	1.755143448	1.201549037	5.255035638	3.088640905
ENSMUSG00000020544	COX11	26.2189633	13.24354212	7.287408088	11.93566676	6.431157673	12.87166946	13.18532368	19.33388327	14.04114758	12.01549037	15.76510691	18.53184543
ENSMUSG00000020545	LRRC72	0	0	0	0	0	0	0	1.288925552	0	0	0	0
ENSMUSG00000020546	STXBP4	0	0	7.287408088	11.93566676	12.86231535	12.87166946	3.955597104	9.022478862	12.28600414	4.806196148	8.75839273	6.177281811
ENSMUSG00000020547	BZW2	323.3672141	245.0055292	400.8074448	280.4881688	308.6955683	302.4842322	326.9960273	324.809239	317.6809641	324.41824	366.1008161	335.1175382
ENSMUSG00000020549	ELAC2	26.2189633	6.621771059	7.287408088	11.93566676	19.29347302	6.435834728	15.82238842	15.46710662	14.04114758	21.62788267	19.26846401	16.98752498
ENSMUSG00000020561	TWISTNB	183.5327431	119.1918791	131.1733456	220.809835	167.2100995	135.1525293	146.3570928	155.9599918	164.9834841	145.3874335	145.3893193	154.4320453
ENSMUSG00000020564	ATXN7L1	0	0	0	5.967833378	0	0	3.955597104	3.866776655	3.510286896	1.201549037	5.255035638	6.177281811
ENSMUSG00000020566	ATP6V1C2	0	0	0	0	0	0	0	0	0	1.201549037	0	0
ENSMUSG00000020570	SYPL	183.5327431	165.5442765	138.4607537	107.4210008	186.5035725	128.7166946	114.712316	114.7143741	100.0431765	112.9456095	124.3691768	106.5581112
ENSMUSG00000020571	PDIA6	253.4499786	165.5442765	145.7481618	137.2601677	192.9347302	180.2033724	238.6543586	239.7401526	221.1480744	230.6974151	220.7114968	213.1162225
ENSMUSG00000020572	NAMPT	8.739654434	39.73062635	14.57481618	23.87133351	77.17389207	32.17917364	55.37835946	54.13487317	61.43002068	69.68984415	61.30874911	47.87393403
ENSMUSG00000020573	PIK3CG	26.2189633	33.10885529	7.287408088	17.90350013	25.72463069	38.61500837	7.911194208	16.75603217	28.08229517	27.63562785	26.27517819	13.89888407
ENSMUSG00000020576	NBAS	26.2189633	19.86531318	29.14963235	17.90350013	25.72463069	38.61500837	30.32624446	20.62280883	26.32715172	20.42633363	14.01342837	20.07616588
ENSMUSG00000020577	TSPAN13	209.7517064	211.8966739	225.9096507	226.7776684	250.8151492	231.6900502	247.8840852	300.3196535	245.7200827	314.8058477	257.4967463	274.8890406
ENSMUSG00000020580	ROCK2	218.4913609	145.6789633	80.16148896	220.809835	180.0724148	193.0750418	123.9420426	132.7593318	129.8806151	162.20912	145.3893193	95.74786807
ENSMUSG00000020585	LAPTM4A	78.65688991	185.4095896	131.1733456	107.4210008	102.8985228	109.4091904	109.4381865	127.6036296	135.1460455	142.9843354	122.6174982	115.824034
ENSMUSG00000020590	SNX13	26.2189633	6.621771059	14.57481618	47.74266702	32.15578836	12.87166946	34.28184157	20.62280883	22.81686482	28.83717689	26.27517819	26.2534477
ENSMUSG00000020592	SDC1	0	6.621771059	0	0	0	0	1.318532368	0	0	0	0	0
ENSMUSG00000020593	LPIN1	17.47930887	19.86531318	36.43704044	11.93566676	45.01810371	45.05084309	11.86679131	28.35636214	29.83743861	25.23252978	15.76510691	30.88640905
ENSMUSG00000020594	PUM2	402.024104	403.9280346	371.6578125	453.5553367	520.9237715	399.0217531	320.4033654	372.4994844	294.8640992	288.3717689	306.5437455	284.1549633

ENSMUSG00000020598	NRCAM	0	0	0	0	0	6.435834728	0	0	0	0	0	0
ENSMUSG00000020599	RGS9	0	0	0	0	0	0	0	1.288925552	0	0	0	1.544320453
ENSMUSG00000020601	TRIB2	0	6.621771059	0	0	0	0	0	1.288925552	0	1.201549037	1.751678546	0
ENSMUSG00000020604	ARSG	0	0	0	5.967833378	0	0	0	1.288925552	0	0	0	3.088640905
ENSMUSG00000020605	HS1BP3	0	0	0	0	0	0	0	0	1.755143448	1.201549037	3.503357092	1.544320453
ENSMUSG00000020608	SMC6	270.9292875	211.8966739	255.0592831	286.4560021	231.5216762	250.9975544	201.7354523	185.6052794	180.7797751	212.6741796	231.2215681	194.584377
ENSMUSG00000020610	AMZ2	17.47930887	39.73062635	51.01185661	71.61400054	38.58694604	25.74333891	40.87450341	32.22313879	26.32715172	30.03872593	42.0402851	27.79776815
ENSMUSG00000020611	GNA13	69.91723547	33.10885529	116.5985294	29.83916689	64.31157673	83.66585146	79.11194208	68.31305424	78.98145515	55.2712557	49.04699929	58.6841772
ENSMUSG00000020612	PRKAR1A	96.13619877	105.9483369	94.73630514	113.3888342	135.0543111	96.53752092	152.9497547	128.8925552	177.2694882	136.9765902	147.1409979	117.3683544
ENSMUSG00000020617	1700012B07F	0	0	0	0	0	0	0	0	1.755143448	0	0	0
ENSMUSG00000020621	RDH14	0	19.86531318	0	11.93566676	6.431157673	19.30750418	13.18532368	12.88925552	15.79629103	10.81394133	7.006714184	7.721602263
ENSMUSG00000020623	MAP2K6	8.739654434	39.73062635	7.287408088	11.93566676	19.29347302	32.17917364	9.229726576	27.06743658	19.30657793	13.21703941	12.26174982	23.16480679
ENSMUSG00000020628	TRAPPC12	43.69827217	6.621771059	29.14963235	17.90350013	6.431157673	32.17917364	10.54825894	18.04495772	15.79629103	12.01549037	14.01342837	26.2534477
ENSMUSG00000020629	ADI1	26.2189633	6.621771059	0	23.87133351	0	32.17917364	25.05211499	23.20065993	17.55143448	25.23252978	15.76510691	16.98752498
ENSMUSG00000020630	RNASEH1	34.95861774	26.48708424	36.43704044	5.967833378	12.86231535	25.74333891	5.274129472	14.17818107	12.28600414	10.81394133	15.76510691	20.07616588
ENSMUSG00000020634	UBXN2A	61.17758104	66.21771059	14.57481618	41.77483365	57.88041905	64.35834728	31.64477683	28.35636214	40.3682993	34.84492207	33.28189237	33.97504996
ENSMUSG00000020640	ITSN2	209.7517064	92.70479482	131.1733456	113.3888342	128.6231535	141.588364	105.4825894	97.95834193	107.0637503	133.3719431	108.6040698	97.29218852
ENSMUSG00000020642	RNF144A	34.95861774	13.24354212	7.287408088	41.77483365	19.29347302	19.30750418	15.82238842	24.48958548	22.81686482	24.03098074	19.26846401	20.07616588
ENSMUSG00000020644	ID2	26.2189633	19.86531318	65.58667279	35.80700027	19.29347302	32.17917364	39.55597104	34.80098989	42.12344275	22.8294317	43.79196365	24.70912724
ENSMUSG00000020647	NCOA1	34.95861774	59.59593953	51.01185661	35.80700027	12.86231535	32.17917364	27.68917973	9.022478862	35.10286896	26.43407881	33.28189237	13.89888407
ENSMUSG00000020648	DUS4L	17.47930887	19.86531318	21.86222426	29.83916689	12.86231535	19.30750418	29.0077121	34.80098989	38.61315585	38.44956918	40.28860656	29.3420886
ENSMUSG00000020649	RRM2	742.8706269	774.7472139	765.1778492	835.4966729	913.2243895	862.4018535	1093.063333	1051.76325	1184.721827	996.0841517	1061.517199	1164.417621
ENSMUSG00000020650	BCAP29	218.4913609	205.2749028	204.0474265	238.7133351	109.3296804	148.0241987	137.1273663	132.7593318	149.1871931	179.0308065	155.8993906	148.2547635
ENSMUSG00000020652	CENPO	8.739654434	13.24354212	14.57481618	11.93566676	19.29347302	12.87166946	23.73358262	16.75603217	21.06172137	12.01549037	36.78524947	16.98752498
ENSMUSG00000020653	KLF11	0	0	0	0	0	0	0	3.866776655	5.265430344	2.403098074	1.751678546	4.632961358
ENSMUSG00000020654	ADCY3	17.47930887	33.10885529	21.86222426	29.83916689	25.72463069	77.23001673	38.23743867	32.22313879	36.85801241	34.84492207	31.53021383	46.32961358
ENSMUSG00000020657	DNAJC27	17.47930887	13.24354212	7.287408088	5.967833378	19.29347302	0	5.274129472	6.444627758	15.79629103	10.81394133	12.26174982	12.35456362
ENSMUSG00000020658	EFR3B	17.47930887	19.86531318	0	5.967833378	12.86231535	6.435834728	3.955597104	6.444627758	8.775717239	6.007745185	5.255035638	3.088640905
ENSMUSG00000020659	CBLL1	52.4379266	33.10885529	43.72444853	35.80700027	64.31157673	38.61500837	52.74129472	45.11239431	42.12344275	40.85266726	50.79867783	38.60801132
ENSMUSG00000020660	POMC	0	6.621771059	7.287408088	5.967833378	0	6.435834728	3.955597104	7.73355331	12.28600414	4.806196148	1.751678546	4.632961358
ENSMUSG00000020661	DNMT3A	201.012052	258.2490713	189.4726103	220.809835	192.9347302	270.3050586	156.9053518	166.2713962	140.4114758	182.6354536	150.644355	160.6093271
ENSMUSG00000020664	DLD	122.3551621	79.46125271	65.58667279	101.4531674	77.17389207	90.10168619	127.8976397	140.4928851	126.3703282	86.51153067	126.1208553	97.29218852
ENSMUSG00000020668	KIF3C	17.47930887	19.86531318	58.2992647	0	12.86231535	6.435834728	14.50385605	12.88925552	12.28600414	15.62013748	10.51007128	13.89888407
ENSMUSG00000020669	SH3YL1	0	0	0	0	0	0	1.318532368	5.155702207	1.755143448	1.201549037	5.255035638	3.088640905
ENSMUSG00000020671	RAB10	61.17758104	72.83948165	43.72444853	167.0993346	38.58694604	51.48667782	69.8822155	51.55702207	50.89915999	86.51153067	56.05371347	67.95009992
ENSMUSG00000020677	DDX52	218.4913609	178.7878186	131.1733456	119.3566676	135.0543111	148.0241987	158.2238842	163.6935451	150.9423365	124.9610998	150.644355	179.1411725
ENSMUSG00000020680	TAF15	122.3551621	125.8136501	138.4607537	161.1315012	115.7608381	148.0241987	137.1273663	176.5828006	184.290062	169.4184142	211.9531041	168.3309293
ENSMUSG00000020687	CDC27	43.69827217	59.59593953	72.87408088	35.80700027	77.17389207	51.48667782	43.51156814	58.00164982	64.94030757	44.45731437	66.56378475	55.5955363
ENSMUSG00000020689	ITGB3	0	0	0	0	0	0	0	0	0	0	0	1.544320453
ENSMUSG00000020691	METTL2	8.739654434	13.24354212	51.01185661	17.90350013	6.431157673	12.87166946	15.82238842	16.75603217	21.06172137	19.22478459	26.27517819	13.89888407
ENSMUSG00000020692	NLE1	43.69827217	52.97416847	65.58667279	41.77483365	32.15578836	51.48667782	44.83010051	63.15735203	33.34772551	46.86041244	33.28189237	50.96257494
ENSMUSG00000020694	TLK2	87.39654434	139.0571922	109.3111213	185.0028347	186.5035725	141.588364	141.0829634	141.7818107	98.28803308	120.1549037	112.1074269	134.3558794
ENSMUSG00000020696	RFFL	8.739654434	19.86531318	14.57481618	35.80700027	45.01810371	25.74333891	30.32624446	33.51206434	14.04114758	26.43407881	29.77853528	24.70912724
ENSMUSG00000020697	LIG3	61.17758104	59.59593953	145.7481618	83.54966729	32.15578836	45.05084309	76.47487734	68.31305424	78.98145515	72.09294222	78.82553457	47.87393403
ENSMUSG00000020698	CCT6B	0	0	0	0	0	6.435834728	0	0	0	0	0	0

ENSMUSG00000020700	MAP3K3	17.47930887	13.24354212	14.57481618	23.87133351	38.58694604	32.17917364	15.82238842	12.88925552	24.57200827	20.42633363	19.26846401	23.16480679
ENSMUSG00000020705	DDX42	166.0534342	145.6789633	145.7481618	137.2601677	173.6412572	173.7675376	162.1794813	126.3147041	142.1666193	181.4339046	171.6644975	152.8877248
ENSMUSG00000020706	FTSJ3	104.8758532	99.32656588	123.8859375	53.7105004	147.9166265	90.10168619	117.3493808	87.64693751	89.51231584	98.52702104	117.3624626	72.58306128
ENSMUSG00000020707	RNF135	0	0	0	0	0	6.435834728	0	0	0	0	0	0
ENSMUSG00000020708	PSMC5	209.7517064	178.7878186	240.4844669	179.0350013	147.9166265	167.3317029	195.1427905	181.7385028	205.3517834	170.6199633	215.4564612	197.6730179
ENSMUSG00000020709	ADAP2	0	6.621771059	0	0	0	0	0	0	0	0	0	0
ENSMUSG00000020712	TCAM1	0	0	0	0	6.431157673	0	0	0	0	0	0	0
ENSMUSG00000020715	ERN1	8.739654434	13.24354212	14.57481618	11.93566676	32.15578836	19.30750418	10.54825894	7.73355331	12.28600414	15.62013748	10.51007128	16.98752498
ENSMUSG00000020716	NF1	131.0948165	86.08302377	109.3111213	137.2601677	51.44926138	109.4091904	67.24515077	86.35801196	59.67487723	103.3332172	82.32889166	111.1910726
ENSMUSG00000020717	PECAM1	61.17758104	72.83948165	51.01185661	53.7105004	51.44926138	51.48667782	38.23743867	58.00164982	56.16459033	37.24802015	52.55035638	55.5955363
ENSMUSG00000020718	POLG2	17.47930887	0	29.14963235	5.967833378	32.15578836	19.30750418	14.50385605	15.46710662	17.55143448	13.21703941	22.7718211	24.70912724
ENSMUSG00000020719	DDX5	358.3258318	410.5498057	408.0948529	364.0378361	373.007145	386.1500837	682.9997666	654.7741802	624.8310674	783.4099721	569.2955274	579.1201698
ENSMUSG00000020720	PSMD12	166.0534342	79.46125271	131.1733456	119.3566676	199.3658879	128.7166946	184.5945315	164.9824706	198.3312096	185.0385517	213.7047826	182.2298134
ENSMUSG00000020721	HELZ	174.7930887	132.4354212	116.5985294	185.0028347	115.7608381	148.0241987	97.57139523	81.20230975	84.2468855	82.90688355	105.1007128	94.20354761
ENSMUSG00000020728	CEP112	8.739654434	13.24354212	0	0	0	6.435834728	10.54825894	10.31140441	3.510286896	9.612392296	15.76510691	7.721602263
ENSMUSG00000020732	RAB37	26.2189633	46.35239741	21.86222426	29.83916689	25.72463069	25.74333891	32.9633092	33.51206434	29.83743861	28.83717689	31.53021383	46.32961358
ENSMUSG00000020733	SLC9A3R1	104.8758532	125.8136501	87.44889705	173.067168	115.7608381	160.8958682	156.9053518	150.8042895	177.2694882	165.8137671	157.6510691	189.9514157
ENSMUSG00000020736	NT5C	104.8758532	46.35239741	65.58667279	65.64616716	64.31157673	45.05084309	64.60808603	88.93586306	91.26745929	76.89913837	73.57049893	81.84898399
ENSMUSG00000020737	HN1	78.65688991	66.21771059	58.2992647	83.54966729	45.01810371	83.66585146	108.1196542	92.80263972	122.8600414	86.51153067	106.8523913	80.30466354
ENSMUSG00000020738	SUMO2	8.739654434	6.621771059	0	0	6.431157673	0	2.637064736	0	0	0	0	0
ENSMUSG00000020739	NUP85	166.0534342	192.0313607	196.7600184	149.1958345	270.1086223	173.7675376	163.4980136	211.3837905	219.392931	162.20912	213.7047826	242.4583111
ENSMUSG00000020740	GGA3	26.2189633	26.48708424	29.14963235	41.77483365	19.29347302	25.74333891	19.77798552	21.91173438	22.81686482	14.41858844	15.76510691	18.53184543
ENSMUSG00000020741	CLUH	139.8344709	86.08302377	116.5985294	59.67833378	90.03620742	96.53752092	94.9343305	87.64693751	100.0431765	85.30998163	110.3557484	80.30466354
ENSMUSG00000020743	MIF4GD	0	6.621771059	14.57481618	0	12.86231535	0	7.911194208	9.022478862	12.28600414	8.410843259	15.76510691	9.265922716
ENSMUSG00000020744	SLC25A19	8.739654434	6.621771059	0	23.87133351	19.29347302	6.435834728	17.14092078	14.17818107	21.06172137	12.01549037	10.51007128	10.81024317
ENSMUSG00000020745	PAFAH1B1	87.39654434	178.7878186	123.8859375	155.1636678	141.4854688	96.53752092	160.8609489	135.3371829	128.1254717	145.3874335	148.8926764	132.8115589
ENSMUSG00000020747	TMEM94	34.95861774	26.48708424	21.86222426	47.74266702	38.58694604	51.48667782	26.37064736	24.48958548	38.61315585	22.8294317	26.27517819	24.70912724
ENSMUSG00000020752	RECQL5	0	6.621771059	0	23.87133351	25.72463069	6.435834728	6.59266184	7.73355331	14.04114758	3.604647111	1.751678546	6.177281811
ENSMUSG00000020755	SAP30BP	17.47930887	19.86531318	14.57481618	35.80700027	12.86231535	6.435834728	30.32624446	28.35636214	26.32715172	34.84492207	17.51678546	30.88640905
ENSMUSG00000020758	ITGB4	0	13.24354212	0	0	0	19.30750418	1.318532368	2.577851103	3.510286896	6.007745185	5.255035638	3.088640905
ENSMUSG00000020766	GALK1	87.39654434	92.70479482	145.7481618	77.58183392	167.2100995	83.66585146	100.20846	136.6261085	150.9423365	115.3487076	154.147712	117.3683544
ENSMUSG00000020770	UNK	43.69827217	26.48708424	36.43704044	41.77483365	38.58694604	32.17917364	22.41505026	43.82346876	29.83743861	20.42633363	38.53692801	26.2534477
ENSMUSG00000020773	TRIM47	0	0	0	0	0	0	0	0	1.755143448	0	0	3.088640905
ENSMUSG00000020774	ASPA	0	0	0	0	0	0	0	0	0	1.201549037	0	0
ENSMUSG00000020775	MRPL38	26.2189633	66.21771059	43.72444853	71.61400054	64.31157673	51.48667782	60.65248893	52.84594762	73.71602481	55.2712557	64.8121062	67.95009992
ENSMUSG00000020776	FBF1	17.47930887	6.621771059	0	11.93566676	12.86231535	12.87166946	3.955597104	3.866776655	5.265430344	7.209294222	3.503357092	12.35456362
ENSMUSG00000020777	ACOX1	26.2189633	13.24354212	21.86222426	17.90350013	6.431157673	19.30750418	23.73358262	19.33388327	26.32715172	22.8294317	17.51678546	18.53184543
ENSMUSG00000020778	TEN1	78.65688991	39.73062635	29.14963235	59.67833378	38.58694604	38.61500837	48.78569762	54.13487317	38.61315585	45.65886341	38.53692801	60.22849765
ENSMUSG00000020780	SRP68	104.8758532	46.35239741	72.87408088	47.74266702	96.46736509	51.48667782	72.51928024	52.84594762	78.98145515	75.69758933	75.32217748	61.77281811
ENSMUSG00000020781	TSEN54	0	13.24354212	0	17.90350013	12.86231535	0	6.59266184	11.60032996	12.28600414	16.82168652	17.51678546	15.44320453
ENSMUSG00000020782	LLGL2	0	0	0	0	0	0	0	0	1.755143448	0	0	0
ENSMUSG00000020783	NCBP3	34.95861774	19.86531318	58.2992647	35.80700027	25.72463069	45.05084309	39.55597104	37.378841	28.08229517	33.64337304	50.79867783	41.69665222
ENSMUSG00000020785	CAMKK1	0	0	0	0	0	12.87166946	0	0	3.510286896	1.201549037	1.751678546	1.544320453
ENSMUSG00000020787	P2RX1	34.95861774	6.621771059	14.57481618	35.80700027	12.86231535	6.435834728	19.77798552	23.20065993	15.79629103	21.62788267	14.01342837	21.62048634
ENSMUSG00000020788	ATP2A3	43.69827217	39.73062635	43.72444853	77.58183392	57.88041905	45.05084309	2.637064736	0	3.510286896	1.201549037	1.751678546	1.544320453

ENSMUSG00000020790	ANKFY1	17.47930887	19.86531318	58.2992647	35.80700027	12.86231535	12.87166946	34.28184157	28.35636214	26.32715172	32.441824	38.53692801	27.79776815
ENSMUSG00000020792	EXOC7	0	0	14.57481618	0	0	25.74333891	7.911194208	19.33388327	22.81686482	12.01549037	14.01342837	16.98752498
ENSMUSG00000020794	UBE2G1	69.91723547	13.24354212	29.14963235	29.83916689	51.44926138	38.61500837	56.69689182	39.9566921	47.38887309	44.45731437	42.0402851	29.3420886
ENSMUSG00000020798	SPNS3	0	0	7.287408088	0	6.431157673	6.435834728	1.318532368	1.288925552	1.755143448	2.403098074	3.503357092	0
ENSMUSG00000020801	MED31	0	6.621771059	29.14963235	17.90350013	38.58694604	12.87166946	21.09651789	18.04495772	26.32715172	13.21703941	28.02685674	32.43072951
ENSMUSG00000020802	UBE2O	43.69827217	39.73062635	29.14963235	47.74266702	38.58694604	32.17917364	46.14863288	61.86842648	43.8785862	49.26351052	40.28860656	47.87393403
ENSMUSG00000020803	TXNDC17	87.39654434	33.10885529	87.44889705	53.7105004	32.15578836	96.53752092	105.4825894	79.9133842	133.390902	98.52702104	113.8591055	91.11490671
ENSMUSG00000020807	4933427D14I	0	0	0	0	0	0	1.318532368	0	0	0	0	0
ENSMUSG00000020812	1810032O08I	8.739654434	13.24354212	7.287408088	17.90350013	0	6.435834728	10.54825894	27.06743658	26.32715172	20.42633363	29.77853528	20.07616588
ENSMUSG00000020814	MXRA7	0	0	0	0	0	0	0	0	1.755143448	0	0	0
ENSMUSG00000020817	RABEP1	157.3137798	139.0571922	123.8859375	119.3566676	180.0724148	83.66585146	123.9420426	126.3147041	108.8188938	151.3951787	131.3758909	120.4569953
ENSMUSG00000020818	MFSD11	17.47930887	19.86531318	7.287408088	5.967833378	0	0	2.637064736	1.288925552	12.28600414	10.81394133	12.26174982	4.632961358
ENSMUSG00000020821	KIF1C	17.47930887	26.48708424	14.57481618	11.93566676	38.58694604	6.435834728	22.41505026	6.444627758	12.28600414	13.21703941	10.51007128	18.53184543
ENSMUSG00000020823	SEC14L1	8.739654434	6.621771059	7.287408088	5.967833378	12.86231535	6.435834728	17.14092078	12.88925552	10.53086069	12.01549037	15.76510691	10.81024317
ENSMUSG00000020826	NOS2	0	6.621771059	0	0	0	0	0	0	0	0	0	0
ENSMUSG00000020827	MINK1	17.47930887	6.621771059	21.86222426	17.90350013	19.29347302	25.74333891	21.09651789	23.20065993	12.28600414	18.02323556	21.02014255	23.16480679
ENSMUSG00000020828	PLD2	0	0	0	5.967833378	0	0	0	0	0	0	0	0
ENSMUSG00000020829	SLC46A1	0	0	0	0	0	0	0	0	1.755143448	2.403098074	0	3.088640905
ENSMUSG00000020831	0610010K14I	0	0	0	0	0	0	0	1.288925552	0	0	0	0
ENSMUSG00000020832	ERAL1	0	6.621771059	29.14963235	17.90350013	12.86231535	32.17917364	22.41505026	14.17818107	29.83743861	21.62788267	22.7718211	29.3420886
ENSMUSG00000020834	DHRS13	8.739654434	13.24354212	7.287408088	0	12.86231535	19.30750418	13.18532368	11.60032996	5.265430344	4.806196148	8.75839273	9.265922716
ENSMUSG00000020843	TIMM22	8.739654434	46.35239741	7.287408088	17.90350013	19.29347302	0	31.64477683	18.04495772	31.59258206	16.82168652	26.27517819	30.88640905
ENSMUSG00000020844	NXN	17.47930887	13.24354212	21.86222426	17.90350013	19.29347302	6.435834728	26.37064736	21.91173438	26.32715172	27.63562785	19.26846401	13.89888407
ENSMUSG00000020846	RFLNB	0	6.621771059	0	17.90350013	32.15578836	19.30750418	13.18532368	11.60032996	3.510286896	4.806196148	5.255035638	12.35456362
ENSMUSG00000020847	RPH3AL	0	0	0	0	0	0	2.637064736	1.288925552	1.755143448	6.007745185	15.76510691	4.632961358
ENSMUSG00000020849	YWHAE	506.8999572	529.7416847	422.6696691	417.7483365	495.1991408	437.6367615	515.5461559	592.9057538	498.4607392	561.1234003	548.2753849	534.3348766
ENSMUSG00000020850	PRPF8	96.13619877	125.8136501	196.7600184	71.61400054	64.31157673	122.2808598	147.6756252	157.2489173	173.7592013	142.9843354	136.6309266	159.0650066
ENSMUSG00000020859	SPAG9	43.69827217	33.10885529	36.43704044	35.80700027	45.01810371	51.48667782	35.60037394	38.66776655	45.63372964	43.25576533	40.28860656	55.5955363
ENSMUSG00000020863	LUC7L3	550.5982293	609.2029374	634.0045036	763.8826724	636.6846096	630.7118033	696.1850903	761.755001	772.2631171	758.1774424	984.4433428	782.9704695
ENSMUSG00000020864	ANKRD40	17.47930887	59.59593953	51.01185661	0	12.86231535	12.87166946	39.55597104	27.06743658	54.40944688	36.04647111	24.52349964	44.78529313
ENSMUSG00000020868	XYLT2	34.95861774	19.86531318	21.86222426	17.90350013	32.15578836	12.87166946	22.41505026	16.75603217	24.57200827	15.62013748	26.27517819	35.51937041
ENSMUSG00000020869	LRRC59	78.65688991	86.08302377	116.5985294	77.58183392	96.46736509	77.23001673	142.4014957	114.7143741	112.3291807	126.1626489	141.8859622	106.5581112
ENSMUSG00000020870	CDC34B	0	0	7.287408088	0	0	0	0	0	0	0	0	0
ENSMUSG00000020872	TAC4	0	0	0	0	0	0	0	1.288925552	1.755143448	0	0	1.544320453
ENSMUSG00000020873	SLC35B1	96.13619877	66.21771059	58.2992647	65.64616716	122.1919958	38.61500837	73.83781261	58.00164982	59.67487723	67.28674607	54.30203492	77.21602263
ENSMUSG00000020876	SNX11	8.739654434	0	14.57481618	17.90350013	0	6.435834728	7.911194208	5.155702207	7.020573791	4.806196148	3.503357092	9.265922716
ENSMUSG00000020877	SCRN2	8.739654434	6.621771059	0	5.967833378	6.431157673	12.87166946	7.911194208	7.73355331	5.265430344	9.612392296	14.01342837	1.544320453
ENSMUSG00000020878	LRRC46	0	0	0	0	6.431157673	0	0	0	0	0	1.751678546	1.544320453
ENSMUSG00000020882	CACNB1	0	0	0	0	6.431157673	0	0	1.288925552	0	0	0	0
ENSMUSG00000020883	FBXL20	52.4379266	59.59593953	58.2992647	47.74266702	64.31157673	51.48667782	27.68917973	36.08991545	40.3682993	24.03098074	36.78524947	41.69665222
ENSMUSG00000020884	ASGR1	0	0	0	0	0	0	0	0	1.288925552	0	0	0
ENSMUSG00000020886	DLG4	0	13.24354212	0	17.90350013	6.431157673	25.74333891	3.955597104	9.022478862	8.775717239	2.403098074	1.751678546	6.177281811
ENSMUSG00000020888	DVL2	34.95861774	33.10885529	94.73630514	23.87133351	6.431157673	32.17917364	40.87450341	42.5345432	29.83743861	32.441824	36.78524947	40.15233177
ENSMUSG00000020889	NR1D1	0	0	0	5.967833378	0	0	2.637064736	1.288925552	5.265430344	7.209294222	0	4.632961358
ENSMUSG00000020891	ALOX8	0	0	0	0	6.431157673	0	1.318532368	0	0	0	0	1.544320453

ENSMUSG00000020893	PER1	26.2189633	13.24354212	29.14963235	53.7105004	12.86231535	6.435834728	38.23743867	33.51206434	28.08229517	25.23252978	26.27517819	29.3420886
ENSMUSG00000020894	VAMP2	34.95861774	6.621771059	0	35.80700027	19.29347302	6.435834728	15.82238842	12.88925552	19.30657793	19.22478459	15.76510691	21.62048634
ENSMUSG00000020895	TMEM107	0	6.621771059	0	5.967833378	6.431157673	6.435834728	5.274129472	5.155702207	5.265430344	1.201549037	5.255035638	7.721602263
ENSMUSG00000020897	AURKB	87.39654434	86.08302377	29.14963235	89.51750067	115.7608381	109.4091904	108.1196542	116.0032996	108.8188938	96.12392296	82.32889166	114.2797135
ENSMUSG00000020898	CTC1	26.2189633	13.24354212	21.86222426	17.90350013	12.86231535	25.74333891	25.05211499	25.77851103	22.81686482	20.42633363	28.02685674	21.62048634
ENSMUSG00000020899	PFAS	113.6155076	165.5442765	138.4607537	113.3888342	180.0724148	96.53752092	154.2682871	164.9824706	166.7386275	156.2013748	138.3826051	176.0525316
ENSMUSG00000020900	MYH10	0	0	7.287408088	0	0	0	1.318532368	0	0	0	0	0
ENSMUSG00000020901	PIK3R5	8.739654434	0	7.287408088	5.967833378	0	0	1.318532368	1.288925552	3.510286896	6.007745185	3.503357092	4.632961358
ENSMUSG00000020903	STX8	17.47930887	13.24354212	7.287408088	5.967833378	6.431157673	6.435834728	15.82238842	15.46710662	17.55143448	14.41858844	17.51678546	20.07616588
ENSMUSG00000020910	ADPRM	8.739654434	19.86531318	7.287408088	17.90350013	32.15578836	38.61500837	14.50385605	20.62280883	24.57200827	16.82168652	10.51007128	12.35456362
ENSMUSG00000020914	TOP2A	515.6396116	490.0110584	466.3941176	698.2365052	501.6302985	669.3268117	465.4419259	523.303774	510.7467433	483.0227129	516.7451711	437.0426881
ENSMUSG00000020917	ACLY	201.012052	172.1660475	94.73630514	250.6490019	147.9166265	135.1525293	205.6910494	219.1173438	240.4546524	254.7283958	218.9598182	234.7367088
ENSMUSG00000020918	KAT2A	34.95861774	13.24354212	36.43704044	17.90350013	25.72463069	57.92251255	38.23743867	48.97917096	49.14401654	43.25576533	54.30203492	63.31713856
ENSMUSG00000020919	STAT5B	69.91723547	39.73062635	58.2992647	29.83916689	19.29347302	32.17917364	58.01542419	52.84594762	52.65430344	36.04647111	26.27517819	50.96257494
ENSMUSG00000020921	TMEM101	0	19.86531318	7.287408088	0	6.431157673	12.87166946	3.955597104	3.866776655	1.755143448	0	5.255035638	3.088640905
ENSMUSG00000020922	LSM12	367.0654862	384.0627214	561.1304227	340.1665026	373.007145	405.4575878	229.424632	241.0290782	229.9237917	191.0462969	225.9665324	256.3571951
ENSMUSG00000020923	UBTF	262.189633	139.0571922	335.220772	196.9385015	250.8151492	263.8692238	253.1582147	288.7193236	275.5575213	267.9454353	236.4766037	228.559427
ENSMUSG00000020925	CCDC43	43.69827217	66.21771059	65.58667279	23.87133351	57.88041905	51.48667782	22.41505026	37.378841	36.85801241	50.46505955	31.53021383	29.3420886
ENSMUSG00000020928	HIGD1B	0	0	0	0	0	0	0	0	0	0	1.751678546	0
ENSMUSG00000020929	EFTUD2	113.6155076	86.08302377	43.72444853	101.4531674	90.03620742	38.61500837	101.5269923	118.5811508	101.79832	96.12392296	124.3691768	105.0137908
ENSMUSG00000020930	CCDC103	0	0	0	0	0	0	0	0	0	0	0	1.544320453
ENSMUSG00000020932	GFAP	0	0	0	5.967833378	0	0	0	0	0	0	0	0
ENSMUSG00000020935	DCAKD	43.69827217	6.621771059	29.14963235	17.90350013	32.15578836	25.74333891	19.77798552	23.20065993	15.79629103	20.42633363	8.75839273	18.53184543
ENSMUSG00000020936	NMT1	26.2189633	46.35239741	58.2992647	17.90350013	51.44926138	45.05084309	43.51156814	50.26809651	56.16459033	38.44956918	45.5436422	44.78529313
ENSMUSG00000020937	PLCD3	0	6.621771059	0	0	0	0	1.318532368	0	0	3.604647111	0	0
ENSMUSG00000020940	1700023F06F	0	0	0	0	0	0	1.318532368	0	0	0	0	0
ENSMUSG00000020941	MAP3K14	0	0	0	0	6.431157673	19.30750418	3.955597104	6.444627758	0	7.209294222	3.503357092	1.544320453
ENSMUSG00000020946	GOSR2	8.739654434	13.24354212	21.86222426	5.967833378	32.15578836	0	11.86679131	10.31140441	14.04114758	13.21703941	7.006714184	13.89888407
ENSMUSG00000020948	KLHL28	52.4379266	19.86531318	43.72444853	17.90350013	45.01810371	25.74333891	32.9633092	16.75603217	15.79629103	24.03098074	8.75839273	15.44320453
ENSMUSG00000020949	FKBP3	279.6689419	238.3837581	298.7837316	196.9385015	244.3839916	276.7408933	394.241178	358.3213034	391.3969889	392.9065351	338.0739594	316.5856928
ENSMUSG00000020952	SCFD1	61.17758104	79.46125271	72.87408088	83.54966729	109.3296804	109.4091904	77.79340971	116.0032996	93.02260274	111.7440604	94.59064148	84.9376249
ENSMUSG00000020954	STRN3	113.6155076	119.1918791	167.610386	161.1315012	109.3296804	122.2808598	98.8899276	65.73520313	112.3291807	86.51153067	85.83224875	86.48194535
ENSMUSG00000020955	AP4S1	26.2189633	6.621771059	14.57481618	11.93566676	32.15578836	12.87166946	21.09651789	18.04495772	15.79629103	25.23252978	17.51678546	16.98752498
ENSMUSG00000020956	DTD2	17.47930887	52.97416847	36.43704044	65.64616716	38.58694604	12.87166946	32.9633092	43.82346876	45.63372964	27.63562785	22.7718211	50.96257494
ENSMUSG00000020961	STON2	0	0	0	5.967833378	0	0	1.318532368	0	0	0	0	0
ENSMUSG00000020962	GTF2A1	17.47930887	52.97416847	58.2992647	95.48533405	38.58694604	19.30750418	46.14863288	47.69024541	24.57200827	24.03098074	42.0402851	38.60801132
ENSMUSG00000020964	SEL1L	113.6155076	72.83948165	80.16148896	83.54966729	83.60504975	77.23001673	44.83010051	51.55702207	45.63372964	50.46505955	54.30203492	71.03874082
ENSMUSG00000020973	DNAAF2	8.739654434	13.24354212	7.287408088	0	12.86231535	6.435834728	10.54825894	10.31140441	12.28600414	15.62013748	14.01342837	9.265922716
ENSMUSG00000020974	POLE2	17.47930887	19.86531318	21.86222426	17.90350013	12.86231535	25.74333891	23.73358262	19.33388327	22.81686482	13.21703941	24.52349964	12.35456362
ENSMUSG00000020978	KLHDC2	8.739654434	46.35239741	51.01185661	17.90350013	19.29347302	12.87166946	34.28184157	25.77851103	49.14401654	34.84492207	42.0402851	41.69665222
ENSMUSG00000020982	NEMF	262.189633	264.8708424	196.7600184	238.7133351	147.9166265	148.0241987	195.1427905	188.1831305	196.5760662	164.6122181	232.9732466	208.4832611
ENSMUSG00000020986	SEC23A	17.47930887	66.21771059	21.86222426	59.67833378	38.58694604	6.435834728	50.10422998	68.31305424	45.63372964	48.06196148	40.28860656	55.5955363
ENSMUSG00000020988	L2HGDH	8.739654434	33.10885529	14.57481618	47.74266702	38.58694604	38.61500837	59.33395656	45.11239431	54.40944688	62.48054992	47.29532074	35.51937041
ENSMUSG00000020993	TRAPPC6B	34.95861774	19.86531318	36.43704044	65.64616716	32.15578836	25.74333891	50.10422998	52.84594762	38.61315585	64.883648	42.0402851	33.97504996
ENSMUSG00000020994	PNN	270.9292875	304.6014687	247.771875	304.3595023	373.007145	302.4842322	382.3743867	331.2538668	398.4175627	396.5111822	464.1948147	364.4596268

ENSMUSG00000021000	MIA2	297.1482508	251.6273002	298.7837316	340.1665026	289.4020953	238.1258849	304.580977	266.8075892	222.9032179	295.5810631	304.792067	223.9264656
ENSMUSG00000021003	GALC	34.95861774	19.86531318	21.86222426	17.90350013	32.15578836	25.74333891	11.86679131	15.46710662	17.55143448	21.62788267	17.51678546	20.07616588
ENSMUSG00000021007	SPATA7	8.739654434	0	29.14963235	17.90350013	12.86231535	32.17917364	14.50385605	16.75603217	12.28600414	10.81394133	8.75839273	16.98752498
ENSMUSG00000021009	PTPN21	17.47930887	6.621771059	21.86222426	5.967833378	6.431157673	25.74333891	7.911194208	14.17818107	12.28600414	13.21703941	14.01342837	7.721602263
ENSMUSG00000021010	NPAS3	0	6.621771059	0	0	0	0	0	0	0	0	0	0
ENSMUSG00000021012	ZC3H14	174.7930887	72.83948165	87.44889705	89.51750067	102.8985228	70.79418201	80.43047445	94.09156527	105.3086069	94.92237392	85.83224875	100.3808294
ENSMUSG00000021018	POLR2H	69.91723547	46.35239741	51.01185661	47.74266702	45.01810371	64.35834728	61.9710213	68.31305424	70.20573791	52.86815763	52.55035638	63.31713856
ENSMUSG00000021022	PPP2R3C	17.47930887	46.35239741	21.86222426	29.83916689	19.29347302	0	15.82238842	23.20065993	22.81686482	19.22478459	14.01342837	21.62048634
ENSMUSG00000021023	1110008L16R	8.739654434	33.10885529	0	5.967833378	19.29347302	6.435834728	15.82238842	19.33388327	22.81686482	10.81394133	21.02014255	10.81024317
ENSMUSG00000021024	PSMA6	131.0948165	192.0313607	204.0474265	196.9385015	205.7970455	193.0750418	224.1505026	228.1398226	229.9237917	217.4803757	261.0001033	211.571902
ENSMUSG00000021025	NFKBIA	96.13619877	86.08302377	123.8859375	95.48533405	122.1919958	122.2808598	87.02313629	68.31305424	86.00202895	75.69758933	91.08728439	86.48194535
ENSMUSG00000021027	RALGAPA1	52.4379266	66.21771059	138.4607537	101.4531674	51.44926138	83.66585146	76.47487734	55.42379872	87.75717239	70.89139318	68.31546329	97.29218852
ENSMUSG00000021028	MBIP	34.95861774	52.97416847	43.72444853	65.64616716	51.44926138	19.30750418	44.83010051	48.97917096	49.14401654	48.06196148	42.0402851	43.24097267
ENSMUSG00000021032	NGB	0	0	0	0	0	0	0	0	0	1.201549037	0	0
ENSMUSG00000021033	GSTZ1	17.47930887	6.621771059	29.14963235	11.93566676	12.86231535	6.435834728	11.86679131	14.17818107	7.020573791	10.81394133	10.51007128	10.81024317
ENSMUSG00000021036	SPTLC2	61.17758104	39.73062635	51.01185661	35.80700027	57.88041905	45.05084309	46.14863288	28.35636214	49.14401654	45.65886341	35.03357092	46.32961358
ENSMUSG00000021037	AHSA1	43.69827217	13.24354212	7.287408088	23.87133351	32.15578836	19.30750418	38.23743867	58.00164982	36.85801241	44.45731437	36.78524947	40.15233177
ENSMUSG00000021038	VIPAS39	17.47930887	0	7.287408088	0	6.431157673	19.30750418	3.955597104	11.60032996	8.775717239	6.007745185	0	10.81024317
ENSMUSG00000021039	SNW1	139.8344709	139.0571922	138.4607537	131.2923343	147.9166265	148.0241987	168.7721431	170.1381728	186.0452055	138.1781393	168.1611404	151.3434044
ENSMUSG00000021040	SLIRP	192.2723975	119.1918791	204.0474265	143.2280011	167.2100995	148.0241987	189.868661	167.5603217	207.1069268	168.2168652	187.4296044	240.9139906
ENSMUSG00000021044	ADCK1	17.47930887	0	0	17.90350013	0	12.87166946	3.955597104	7.73355331	5.265430344	4.806196148	12.26174982	12.35456362
ENSMUSG00000021048	MTHFD1	96.13619877	99.32656588	94.73630514	77.58183392	115.7608381	115.8450251	127.8976397	113.4254485	117.594611	97.325472	122.6174982	94.20354761
ENSMUSG00000021051	PPP2R5E	8.739654434	26.48708424	29.14963235	35.80700027	45.01810371	12.87166946	34.28184157	30.93421324	33.34772551	24.03098074	36.78524947	32.43072951
ENSMUSG00000021054	SGPP1	26.2189633	26.48708424	14.57481618	5.967833378	12.86231535	19.30750418	0	0	0	0	0	0
ENSMUSG00000021057	AKAP5	0	6.621771059	0	5.967833378	0	0	1.318532368	2.577851103	0	3.604647111	0	3.088640905
ENSMUSG00000021065	FUT8	26.2189633	0	29.14963235	17.90350013	38.58694604	12.87166946	7.911194208	15.46710662	15.79629103	12.01549037	21.02014255	9.265922716
ENSMUSG00000021068	NIN	78.65688991	46.35239741	21.86222426	23.87133351	12.86231535	32.17917364	50.10422998	42.5345432	54.40944688	45.65886341	36.78524947	50.96257494
ENSMUSG00000021071	TRIM9	0	0	0	0	0	0	0	0	1.755143448	0	1.751678546	0
ENSMUSG00000021072	TMX1	139.8344709	105.9483369	80.16148896	59.67833378	57.88041905	83.66585146	69.8822155	70.89090534	63.18516412	68.48829511	82.32889166	77.21602263
ENSMUSG00000021076	ACTR10	26.2189633	59.59593953	80.16148896	83.54966729	45.01810371	70.79418201	58.01542419	55.42379872	61.43002068	55.2712557	66.56378475	63.31713856
ENSMUSG00000021078	TOMM20L	8.739654434	6.621771059	7.287408088	5.967833378	0	0	3.955597104	6.444627758	1.755143448	2.403098074	10.51007128	4.632961358
ENSMUSG00000021079	TIMM9	69.91723547	72.83948165	94.73630514	125.3245009	57.88041905	64.35834728	92.29726576	85.06908641	64.94030757	92.51927585	91.08728439	67.95009992
ENSMUSG00000021086	CCDC175	0	0	7.287408088	5.967833378	6.431157673	0	9.229726576	3.866776655	12.28600414	7.209294222	7.006714184	4.632961358
ENSMUSG00000021094	DHRS7	34.95861774	13.24354212	43.72444853	17.90350013	57.88041905	38.61500837	35.60037394	36.08991545	45.63372964	28.83717689	26.27517819	46.32961358
ENSMUSG00000021096	PPM1A	87.39654434	52.97416847	109.3111213	77.58183392	45.01810371	70.79418201	59.33395656	61.86842648	61.43002068	44.45731437	45.5436422	54.05121584
ENSMUSG00000021102	GLRX5	61.17758104	19.86531318	43.72444853	53.7105004	6.431157673	45.05084309	67.24515077	63.15735203	52.65430344	43.25576533	77.07385602	63.31713856
ENSMUSG00000021103	MNAT1	43.69827217	39.73062635	43.72444853	71.61400054	45.01810371	70.79418201	48.78569762	50.26809651	63.18516412	43.25576533	59.55707056	46.32961358
ENSMUSG00000021108	PRKCH	61.17758104	46.35239741	72.87408088	29.83916689	51.44926138	32.17917364	32.9633092	39.9566921	35.10286896	38.44956918	43.79196365	32.43072951
ENSMUSG00000021109	HIF1A	52.4379266	39.73062635	51.01185661	71.61400054	51.44926138	38.61500837	48.78569762	50.26809651	63.18516412	80.50378548	68.31546329	49.41825449
ENSMUSG00000021111	PAPOLA	96.13619877	105.9483369	145.7481618	149.1958345	102.8985228	77.23001673	145.0385605	161.115694	179.0246317	159.8060219	180.4228902	152.8877248
ENSMUSG00000021112	MPP5	26.2189633	52.97416847	14.57481618	35.80700027	25.72463069	64.35834728	39.55597104	41.24561765	42.12344275	42.0542163	35.03357092	37.06369086
ENSMUSG00000021113	SNAPC1	17.47930887	19.86531318	29.14963235	11.93566676	25.72463069	19.30750418	32.9633092	37.378841	17.55143448	38.44956918	24.52349964	26.2534477
ENSMUSG00000021114	ATP6V1D	8.739654434	26.48708424	14.57481618	17.90350013	51.44926138	45.05084309	40.87450341	29.64528769	33.34772551	37.24802015	61.30874911	37.06369086
ENSMUSG00000021115	VRK1	52.4379266	72.83948165	102.0237132	59.67833378	102.8985228	102.9733556	94.9343305	108.2697463	91.26745929	74.4960403	78.82553457	106.5581112
ENSMUSG00000021116	EIF2S1	78.65688991	92.70479482	123.8859375	119.3566676	115.7608381	102.9733556	109.4381865	113.4254485	156.2077669	109.3409624	127.8725339	146.710443

ENSMUSG00000021120	PIGH	8.739654434	13.24354212	0	0	12.86231535	6.435834728	14.50385605	7.73355331	7.020573791	21.62788267	14.01342837	6.177281811
ENSMUSG00000021123	RDH12	0	0	0	5.967833378	6.431157673	0	0	0	0	0	0	0
ENSMUSG00000021124	VTI1B	43.69827217	39.73062635	14.57481618	59.67833378	45.01810371	12.87166946	42.19303578	32.22313879	33.34772551	30.03872593	26.27517819	30.88640905
ENSMUSG00000021125	ARG2	8.739654434	6.621771059	0	0	0	0	1.318532368	1.288925552	3.510286896	1.201549037	3.503357092	3.088640905
ENSMUSG00000021127	ZFP36L1	104.8758532	165.5442765	123.8859375	119.3566676	96.46736509	148.0241987	152.9497547	140.4928851	107.0637503	151.3951787	150.644355	163.697968
ENSMUSG00000021130	GALNT16	8.739654434	0	0	0	0	0	0	0	1.288925552	1.755143448	1.201549037	0	0
ENSMUSG00000021131	ERH	0	6.621771059	14.57481618	17.90350013	12.86231535	6.435834728	9.229726576	11.60032996	7.020573791	20.42633363	24.52349964	23.16480679
ENSMUSG00000021133	SUSD6	52.4379266	26.48708424	21.86222426	47.74266702	45.01810371	32.17917364	34.28184157	42.5345432	45.63372964	45.65886341	35.03357092	30.88640905
ENSMUSG00000021134	SRSF5	183.5327431	185.4095896	233.1970588	220.809835	270.1086223	238.1258849	271.6176678	286.1414725	284.3332386	289.5733179	245.2349964	262.534477
ENSMUSG00000021135	SLC10A1	0	0	0	11.93566676	0	0	0	0	0	0	1.751678546	0
ENSMUSG00000021139	GM20498	8.739654434	0	7.287408088	0	6.431157673	0	2.637064736	2.577851103	1.755143448	3.604647111	1.751678546	0
ENSMUSG00000021140	PCNX	8.739654434	13.24354212	21.86222426	11.93566676	19.29347302	25.74333891	22.41505026	19.33388327	21.06172137	19.22478459	12.26174982	23.16480679
ENSMUSG00000021143	PACS2	8.739654434	13.24354212	36.43704044	11.93566676	45.01810371	12.87166946	17.14092078	19.33388327	26.32715172	13.21703941	24.52349964	15.44320453
ENSMUSG00000021144	MTA1	166.0534342	218.5184449	225.9096507	190.9706681	173.6412572	128.7166946	142.4014957	146.9375129	154.4526234	163.410669	161.1544262	194.584377
ENSMUSG00000021147	WDR37	34.95861774	26.48708424	36.43704044	29.83916689	25.72463069	12.87166946	36.9189063	23.20065993	31.59258206	30.03872593	33.28189237	35.51937041
ENSMUSG00000021149	GTPBP4	183.5327431	105.9483369	174.8977941	190.9706681	205.7970455	115.8450251	162.1794813	136.6261085	119.3497545	146.5889825	155.8993906	135.9001998
ENSMUSG00000021156	ZMYND11	87.39654434	99.32656588	196.7600184	161.1315012	186.5035725	154.4600335	142.4014957	140.4928851	154.4526234	118.9533547	145.3893193	154.4320453
ENSMUSG00000021171	ESYT2	340.8465229	178.7878186	196.7600184	185.0028347	289.4020953	199.5108766	145.0385605	136.6261085	164.9834841	162.20912	127.8725339	138.9888407
ENSMUSG00000021175	CDCA7L	52.4379266	79.46125271	43.72444853	47.74266702	57.88041905	45.05084309	69.8822155	59.29057538	42.12344275	44.45731437	75.32217748	69.49442037
ENSMUSG00000021176	EFCAB11	17.47930887	13.24354212	7.287408088	5.967833378	0	0	2.637064736	1.288925552	3.510286896	6.007745185	1.751678546	0
ENSMUSG00000021177	TDP1	34.95861774	19.86531318	29.14963235	11.93566676	25.72463069	38.61500837	19.77798552	34.80098989	28.08229517	33.64337304	40.28860656	33.97504996
ENSMUSG00000021178	PSMC1	139.8344709	105.9483369	80.16148896	119.3566676	77.17389207	77.23001673	147.6756252	127.6036296	91.26745929	115.3487076	115.610784	109.6467521
ENSMUSG00000021179	NRDE2	8.739654434	33.10885529	7.287408088	41.77483365	6.431157673	6.435834728	11.86679131	14.17818107	14.04114758	14.41858844	24.52349964	16.98752498
ENSMUSG00000021180	RPS6KA5	17.47930887	6.621771059	36.43704044	17.90350013	6.431157673	12.87166946	22.41505026	20.62280883	21.06172137	15.62013748	19.26846401	21.62048634
ENSMUSG00000021182	CCDC88C	43.69827217	52.97416847	51.01185661	41.77483365	64.31157673	57.92251255	73.83781261	54.13487317	71.96088136	61.27900089	50.79867783	69.49442037
ENSMUSG00000021185	DGLUCY	0	0	0	0	0	0	2.637064736	0	0	6.007745185	0	3.088640905
ENSMUSG00000021187	TC2N	0	6.621771059	0	0	0	0	0	0	0	0	0	0
ENSMUSG00000021188	TRIP11	122.3551621	192.0313607	102.0237132	149.1958345	70.7427344	115.8450251	92.29726576	91.51371417	87.75717239	117.7518056	77.07385602	80.30466354
ENSMUSG00000021189	ATXN3	61.17758104	26.48708424	43.72444853	41.77483365	32.15578836	25.74333891	35.60037394	21.91173438	31.59258206	24.03098074	31.53021383	20.07616588
ENSMUSG00000021190	LGMN	0	0	0	5.967833378	0	0	9.229726576	7.73355331	14.04114758	12.01549037	5.255035638	4.632961358
ENSMUSG00000021192	GOLGA5	8.739654434	6.621771059	14.57481618	11.93566676	6.431157673	19.30750418	9.229726576	15.46710662	17.55143448	10.81394133	15.76510691	9.265922716
ENSMUSG00000021193	PITRM1	17.47930887	52.97416847	51.01185661	65.64616716	70.7427344	83.66585146	69.8822155	65.73520313	82.49174205	57.67435378	57.80539202	64.86145901
ENSMUSG00000021196	PFKP	131.0948165	105.9483369	109.3111213	107.4210008	186.5035725	115.8450251	155.5868194	122.4479274	124.6151848	144.1858844	168.1611404	123.5456362
ENSMUSG00000021198	UNC79	0	13.24354212	7.287408088	5.967833378	0	6.435834728	10.54825894	3.866776655	1.755143448	8.410843259	3.503357092	6.177281811
ENSMUSG00000021200	ASB2	34.95861774	6.621771059	21.86222426	0	0	6.435834728	7.911194208	7.73355331	8.775717239	8.410843259	12.26174982	6.177281811
ENSMUSG00000021203	OTUB2	8.739654434	13.24354212	7.287408088	5.967833378	12.86231535	19.30750418	10.54825894	9.022478862	5.265430344	6.007745185	8.75839273	4.632961358
ENSMUSG00000021211	AKR1C12	0	0	0	0	0	0	0	1.288925552	0	0	0	0
ENSMUSG00000021213	AKR1C13	0	0	0	0	0	0	1.318532368	0	0	0	0	0
ENSMUSG00000021214	AKR1C18	0	0	7.287408088	0	0	0	1.318532368	0	0	0	0	0
ENSMUSG00000021215	NET1	96.13619877	86.08302377	94.73630514	59.67833378	122.1919958	96.53752092	105.4825894	90.22478862	70.20573791	86.51153067	92.83896294	72.58306128
ENSMUSG00000021216	TUBAL3	0	0	0	0	0	0	0	0	0	0	0	1.544320453
ENSMUSG00000021218	GDI2	131.0948165	172.1660475	196.7600184	137.2601677	218.6593609	180.2033724	225.4690349	179.1606517	205.3517834	191.0462969	213.7047826	180.685493
ENSMUSG00000021219	RGS6	0	0	0	0	0	0	0	0	0	0	1.751678546	0
ENSMUSG00000021221	DPF3	0	0	0	0	0	0	1.318532368	1.288925552	0	1.201549037	3.503357092	1.544320453
ENSMUSG00000021222	DCAF4	61.17758104	19.86531318	36.43704044	41.77483365	19.29347302	38.61500837	22.41505026	42.5345432	22.81686482	28.83717689	28.02685674	29.3420886

ENSMUSG00000021224	NUMB	0	19.86531318	14.57481618	11.93566676	32.15578836	19.30750418	17.14092078	19.33388327	14.04114758	22.8294317	10.51007128	18.53184543
ENSMUSG00000021226	ACOT2	139.8344709	165.5442765	131.1733456	107.4210008	122.1919958	141.588364	90.97873339	97.95834193	84.2468855	84.10843259	82.32889166	81.84898399
ENSMUSG00000021234	FAM161B	0	0	0	5.967833378	0	0	0	3.866776655	0	1.201549037	5.255035638	3.088640905
ENSMUSG00000021235	COQ6	8.739654434	26.48708424	14.57481618	11.93566676	6.431157673	25.74333891	19.77798552	16.75603217	12.28600414	19.22478459	7.006714184	18.53184543
ENSMUSG00000021236	ENTPD5	0	33.10885529	43.72444853	35.80700027	19.29347302	38.61500837	25.05211499	28.35636214	12.28600414	24.03098074	29.77853528	37.06369086
ENSMUSG00000021238	ALDH6A1	0	26.48708424	29.14963235	0	19.29347302	19.30750418	14.50385605	11.60032996	21.06172137	9.612392296	10.51007128	20.07616588
ENSMUSG00000021240	ABCD4	8.739654434	6.621771059	7.287408088	17.90350013	6.431157673	25.74333891	17.14092078	11.60032996	12.28600414	4.806196148	8.75839273	10.81024317
ENSMUSG00000021241	ISCA2	52.4379266	13.24354212	51.01185661	23.87133351	25.72463069	51.48667782	39.55597104	33.51206434	43.8785862	37.24802015	45.5436422	30.88640905
ENSMUSG00000021242	NPC2	52.4379266	59.59593953	65.58667279	89.51750067	51.44926138	57.92251255	68.56368314	83.78016086	64.94030757	74.4960403	66.56378475	64.86145901
ENSMUSG00000021243	FCF1	87.39654434	119.1918791	87.44889705	95.48533405	96.46736509	57.92251255	127.8976397	139.2039596	117.594611	120.1549037	162.9061048	131.2672385
ENSMUSG00000021244	YLPM1	87.39654434	112.570108	116.5985294	155.1636678	96.46736509	77.23001673	94.9343305	95.38049082	105.3086069	96.12392296	96.34232003	115.824034
ENSMUSG00000021245	MLH3	0	6.621771059	0	0	12.86231535	6.435834728	1.318532368	5.155702207	1.755143448	8.410843259	5.255035638	3.088640905
ENSMUSG00000021248	TMED10	113.6155076	79.46125271	65.58667279	143.2280011	64.31157673	45.05084309	47.46716525	61.86842648	68.45059447	61.27900089	73.57049893	61.77281811
ENSMUSG00000021250	FOS	8.739654434	6.621771059	0	0	0	6.435834728	3.955597104	0	1.755143448	0	0	0
ENSMUSG00000021252	ERG28	78.65688991	66.21771059	87.44889705	41.77483365	57.88041905	57.92251255	94.9343305	82.49123531	91.26745929	82.90688355	103.3490342	101.9251499
ENSMUSG00000021254	GPATCH2L	0	13.24354212	14.57481618	0	12.86231535	0	5.274129472	11.60032996	5.265430344	9.612392296	3.503357092	20.07616588
ENSMUSG00000021257	ANGEL1	0	0	0	0	0	0	2.637064736	1.288925552	0	0	1.751678546	0
ENSMUSG00000021258	CCNK	26.2189633	26.48708424	58.2992647	29.83916689	57.88041905	45.05084309	31.64477683	41.24561765	36.85801241	31.24027496	38.53692801	40.15233177
ENSMUSG00000021259	CYP46A1	0	0	0	0	0	0	0	0	0	0	0	1.544320453
ENSMUSG00000021262	EVL	113.6155076	105.9483369	174.8977941	119.3566676	90.03620742	96.53752092	117.3493808	136.6261085	140.4114758	102.1316681	119.1141411	137.4445203
ENSMUSG00000021263	DEGS2	52.4379266	119.1918791	21.86222426	23.87133351	38.58694604	45.05084309	42.19303578	30.93421324	36.85801241	32.441824	22.7718211	52.50689539
ENSMUSG00000021264	YY1	148.5741254	99.32656588	138.4607537	125.3245009	154.3477841	167.3317029	167.4536107	208.8059394	168.493771	201.8602382	194.4363186	165.2422884
ENSMUSG00000021265	SLC25A29	8.739654434	0	0	0	6.431157673	0	3.955597104	1.288925552	1.755143448	2.403098074	3.503357092	3.088640905
ENSMUSG00000021266	WARS	17.47930887	46.35239741	21.86222426	23.87133351	45.01810371	57.92251255	34.28184157	29.64528769	29.83743861	39.65111822	49.04699929	41.69665222
ENSMUSG00000021270	HSP90AA1	550.5982293	490.0110584	459.1067095	513.2336705	488.7679831	624.2759686	448.3010051	411.167251	470.378444	489.0304581	462.4431361	421.5994836
ENSMUSG00000021271	ZFP839	8.739654434	0	0	11.93566676	6.431157673	12.87166946	14.50385605	7.73355331	10.53086069	6.007745185	5.255035638	3.088640905
ENSMUSG00000021273	FDFT1	17.47930887	19.86531318	58.2992647	17.90350013	6.431157673	19.30750418	35.60037394	39.9566921	38.61315585	38.44956918	40.28860656	50.96257494
ENSMUSG00000021275	TECPR2	8.739654434	0	0	11.93566676	12.86231535	19.30750418	10.54825894	9.022478862	10.53086069	8.410843259	15.76510691	13.89888407
ENSMUSG00000021276	CINP	26.2189633	39.73062635	14.57481618	53.7105004	6.431157673	32.17917364	42.19303578	48.97917096	43.8785862	44.45731437	68.31546329	41.69665222
ENSMUSG00000021277	TRAF3	69.91723547	52.97416847	36.43704044	29.83916689	38.58694604	38.61500837	25.05211499	25.77851103	21.06172137	25.23252978	17.51678546	40.15233177
ENSMUSG00000021278	AMN	0	6.621771059	0	0	0	0	0	0	1.755143448	0	0	0
ENSMUSG00000021282	EIF5	323.3672141	344.3320951	400.8074448	334.1986692	353.713672	302.4842322	395.5597104	415.0340276	391.3969889	406.1235745	380.1142445	406.1562791
ENSMUSG00000021285	PPP1R13B	0	13.24354212	7.287408088	17.90350013	19.29347302	12.87166946	13.18532368	15.46710662	5.265430344	12.01549037	17.51678546	12.35456362
ENSMUSG00000021286	ZFYVE21	8.739654434	13.24354212	7.287408088	0	0	6.435834728	11.86679131	15.46710662	8.775717239	10.81394133	10.51007128	7.721602263
ENSMUSG00000021287	XRCC3	17.47930887	6.621771059	14.57481618	11.93566676	12.86231535	12.87166946	17.14092078	18.04495772	21.06172137	8.410843259	10.51007128	12.35456362
ENSMUSG00000021288	KLC1	69.91723547	72.83948165	21.86222426	47.74266702	32.15578836	38.61500837	32.9633092	38.66776655	38.61315585	43.25576533	54.30203492	35.51937041
ENSMUSG00000021290	2010107E04F	209.7517064	198.6531318	218.6222426	173.067168	167.2100995	218.8183807	301.9439123	288.7193236	321.191251	246.3175526	339.8256379	301.1424883
ENSMUSG00000021298	GPR132	8.739654434	33.10885529	7.287408088	23.87133351	6.431157673	19.30750418	9.229726576	11.60032996	8.775717239	12.01549037	8.75839273	10.81024317
ENSMUSG00000021301	HECW1	0	0	0	5.967833378	0	0	0	0	0	0	0	0
ENSMUSG00000021302	GGPS1	26.2189633	6.621771059	29.14963235	53.7105004	12.86231535	32.17917364	22.41505026	29.64528769	36.85801241	19.22478459	21.02014255	38.60801132
ENSMUSG00000021311	MTR	8.739654434	26.48708424	29.14963235	11.93566676	25.72463069	25.74333891	23.73358262	42.5345432	21.06172137	27.63562785	24.52349964	40.15233177
ENSMUSG00000021313	RYR2	0	0	0	5.967833378	0	0	0	0	0	0	0	0
ENSMUSG00000021314	AMPH	8.739654434	0	7.287408088	5.967833378	6.431157673	12.87166946	0	3.866776655	5.265430344	6.007745185	5.255035638	4.632961358
ENSMUSG00000021322	AOAH	26.2189633	6.621771059	0	0	0	12.87166946	2.637064736	7.73355331	5.265430344	2.403098074	7.006714184	3.088640905
ENSMUSG00000021326	TRIM27	113.6155076	86.08302377	72.87408088	65.64616716	57.88041905	51.48667782	84.38607155	82.49123531	96.53288963	84.10843259	84.08057021	77.21602263

ENSMUSG00000021327	ZKSCAN3	87.39654434	86.08302377	58.2992647	77.58183392	102.8985228	77.23001673	89.66020102	112.136523	129.8806151	91.31772681	110.3557484	114.2797135
ENSMUSG00000021338	CARMIL1	8.739654434	52.97416847	36.43704044	11.93566676	25.72463069	12.87166946	10.54825894	19.33388327	8.775717239	16.82168652	14.01342837	9.265922716
ENSMUSG00000021339	MRS2	96.13619877	86.08302377	51.01185661	77.58183392	77.17389207	57.92251255	46.14863288	38.66776655	52.65430344	39.65111822	61.30874911	37.06369086
ENSMUSG00000021340	GPLD1	0	6.621771059	0	0	6.431157673	0	1.318532368	0	1.755143448	0	1.751678546	1.544320453
ENSMUSG00000021356	IRF4	8.739654434	0	0	0	0	0	0	0	0	0	1.751678546	0
ENSMUSG00000021357	EXOC2	8.739654434	6.621771059	36.43704044	29.83916689	19.29347302	32.17917364	26.37064736	19.33388327	28.08229517	33.64337304	21.02014255	29.3420886
ENSMUSG00000021359	TFAP2A	0	0	7.287408088	5.967833378	0	0	2.637064736	1.288925552	0	0	0	0
ENSMUSG00000021360	GCNT2	8.739654434	6.621771059	29.14963235	11.93566676	19.29347302	0	17.14092078	19.33388327	10.53086069	16.82168652	24.52349964	13.89888407
ENSMUSG00000021361	TMEM14C	104.8758532	66.21771059	43.72444853	59.67833378	51.44926138	64.35834728	73.83781261	67.02412869	73.71602481	46.86041244	59.55707056	60.22849765
ENSMUSG00000021365	NEDD9	69.91723547	39.73062635	21.86222426	11.93566676	38.58694604	51.48667782	25.05211499	32.22313879	36.85801241	32.441824	24.52349964	29.3420886
ENSMUSG00000021366	HIVEP1	43.69827217	33.10885529	58.2992647	29.83916689	19.29347302	19.30750418	17.14092078	16.75603217	26.32715172	26.43407881	26.27517819	24.70912724
ENSMUSG00000021368	TBC1D7	0	0	0	5.967833378	12.86231535	12.87166946	5.274129472	7.73355331	3.510286896	6.007745185	1.751678546	10.81024317
ENSMUSG00000021371	MCUR1	17.47930887	6.621771059	14.57481618	11.93566676	19.29347302	25.74333891	15.82238842	14.17818107	12.28600414	8.410843259	19.26846401	15.44320453
ENSMUSG00000021373	CAP2	0	0	0	5.967833378	0	0	3.955597104	6.444627758	3.510286896	1.201549037	3.503357092	6.177281811
ENSMUSG00000021374	NUP153	8.739654434	33.10885529	29.14963235	41.77483365	51.44926138	32.17917364	32.9633092	54.13487317	59.67487723	40.85266726	42.0402851	41.69665222
ENSMUSG00000021375	KIF13A	0	13.24354212	0	5.967833378	12.86231535	6.435834728	18.45945315	21.91173438	12.28600414	9.612392296	7.006714184	12.35456362
ENSMUSG00000021376	TPMT	8.739654434	6.621771059	7.287408088	5.967833378	0	6.435834728	10.54825894	3.866776655	3.510286896	4.806196148	5.255035638	12.35456362
ENSMUSG00000021377	DEK	681.6930459	940.2914904	823.4771139	1044.370841	694.5650287	759.4284979	610.4804864	564.5493916	587.973055	607.9838127	635.8593122	572.9428879
ENSMUSG00000021384	SUSD3	8.739654434	0	0	5.967833378	0	0	0	1.288925552	0	0	0	1.544320453
ENSMUSG00000021385	IPPK	43.69827217	46.35239741	29.14963235	29.83916689	25.72463069	32.17917364	61.9710213	51.55702207	42.12344275	39.65111822	35.03357092	43.24097267
ENSMUSG00000021388	ASPN	0	0	7.287408088	5.967833378	0	6.435834728	1.318532368	1.288925552	0	0	0	0
ENSMUSG00000021390	OGN	0	0	7.287408088	0	0	0	0	0	0	0	0	0
ENSMUSG00000021391	CENPP	34.95861774	39.73062635	14.57481618	53.7105004	19.29347302	19.30750418	36.9189063	34.80098989	36.85801241	39.65111822	28.02685674	26.2534477
ENSMUSG00000021392	NOL8	122.3551621	132.4354212	123.8859375	173.067168	122.1919958	167.3317029	130.5347044	126.3147041	142.1666193	139.3796883	143.6376408	125.0899567
ENSMUSG00000021395	SPIN1	34.95861774	86.08302377	65.58667279	47.74266702	57.88041905	70.79418201	69.8822155	48.97917096	78.98145515	62.48054992	73.57049893	54.05121584
ENSMUSG00000021396	NXNL2	0	0	0	5.967833378	0	0	0	0	1.755143448	0	0	0
ENSMUSG00000021400	WRNIP1	17.47930887	39.73062635	29.14963235	53.7105004	51.44926138	57.92251255	69.8822155	74.757682	64.94030757	56.47280474	36.78524947	49.41825449
ENSMUSG00000021408	RIPK1	61.17758104	39.73062635	65.58667279	47.74266702	12.86231535	25.74333891	19.77798552	30.93421324	40.3682993	33.64337304	29.77853528	50.96257494
ENSMUSG00000021413	PRPF4B	332.1068685	443.6586609	306.0711397	358.0700027	398.7317757	431.2009268	452.2566022	406.0115488	368.5801241	511.8598898	460.6914576	418.5108427
ENSMUSG00000021414	FAM217A	8.739654434	0	0	0	0	0	0	0	0	1.201549037	0	0
ENSMUSG00000021417	ECI2	34.95861774	46.35239741	51.01185661	47.74266702	12.86231535	64.35834728	34.28184157	39.9566921	47.38887309	42.0542163	43.79196365	32.43072951
ENSMUSG00000021418	RPP40	26.2189633	33.10885529	29.14963235	29.83916689	25.72463069	32.17917364	26.37064736	25.77851103	33.34772551	30.03872593	31.53021383	29.3420886
ENSMUSG00000021420	FARS2	17.47930887	6.621771059	7.287408088	5.967833378	6.431157673	0	11.86679131	10.31140441	8.775717239	8.410843259	8.75839273	12.35456362
ENSMUSG00000021427	SSR1	480.6809939	470.1457452	451.8193014	346.1343359	347.2825143	450.5084309	319.0848331	326.0981646	284.3332386	252.3252978	296.0336743	281.0663224
ENSMUSG00000021428	RIOK1	139.8344709	99.32656588	109.3111213	113.3888342	128.6231535	90.10168619	106.8011218	101.8251186	87.75717239	104.5347662	96.34232003	118.9126749
ENSMUSG00000021431	SNRNP48	297.1482508	245.0055292	378.9452206	304.3595023	360.1448297	238.1258849	224.1505026	219.1173438	272.0472344	300.3872593	248.7383535	233.1923884
ENSMUSG00000021432	SLC35B3	8.739654434	26.48708424	29.14963235	11.93566676	12.86231535	6.435834728	25.05211499	38.66776655	22.81686482	33.64337304	24.52349964	26.2534477
ENSMUSG00000021448	SHC3	0	0	0	0	0	0	0	0	0	1.201549037	0	0
ENSMUSG00000021451	SEMA4D	52.4379266	66.21771059	58.2992647	71.61400054	57.88041905	25.74333891	39.55597104	60.57950093	64.94030757	75.69758933	63.06042765	57.13985675
ENSMUSG00000021453	GADD45G	69.91723547	0	21.86222426	11.93566676	64.31157673	25.74333891	31.64477683	24.48958548	24.57200827	27.63562785	29.77853528	40.15233177
ENSMUSG00000021458	2010111I01R	0	13.24354212	21.86222426	5.967833378	0	6.435834728	6.59266184	9.022478862	3.510286896	6.007745185	7.006714184	4.632961358
ENSMUSG00000021460	AUH	139.8344709	59.59593953	109.3111213	17.90350013	77.17389207	70.79418201	64.60808603	54.13487317	61.43002068	62.48054992	66.56378475	63.31713856
ENSMUSG00000021461	FANCC	17.47930887	0	7.287408088	11.93566676	12.86231535	6.435834728	7.911194208	14.17818107	14.04114758	12.01549037	10.51007128	10.81024317
ENSMUSG00000021466	PTCH1	61.17758104	66.21771059	72.87408088	131.2923343	96.46736509	70.79418201	83.06753918	81.20230975	94.77774619	66.08519704	94.59064148	89.57058625
ENSMUSG00000021468	SPTLC1	8.739654434	13.24354212	14.57481618	0	25.72463069	6.435834728	7.911194208	6.444627758	3.510286896	6.007745185	5.255035638	4.632961358

ENSMUSG00000021470	ERCC6L2	26.2189633	33.10885529	21.86222426	41.77483365	83.60504975	64.35834728	32.9633092	45.11239431	24.57200827	38.44956918	33.28189237	30.88640905
ENSMUSG00000021474	SFXN1	262.189633	291.3579266	313.3585478	238.7133351	250.8151492	263.8692238	261.0694089	211.3837905	238.6995089	229.4958661	231.2215681	259.445836
ENSMUSG00000021476	HABP4	8.739654434	6.621771059	14.57481618	0	25.72463069	0	10.54825894	9.022478862	14.04114758	15.62013748	7.006714184	9.265922716
ENSMUSG00000021481	ZFP346	61.17758104	39.73062635	29.14963235	35.80700027	12.86231535	25.74333891	27.68917973	29.64528769	24.57200827	20.42633363	24.52349964	32.43072951
ENSMUSG00000021482	AAED1	43.69827217	66.21771059	36.43704044	29.83916689	96.46736509	45.05084309	27.68917973	51.55702207	43.8785862	32.441824	38.53692801	33.97504996
ENSMUSG00000021484	LMAN2	192.2723975	211.8966739	196.7600184	173.067168	225.0905185	238.1258849	191.1871934	189.4720561	222.9032179	204.2633363	231.2215681	188.4070952
ENSMUSG00000021485	MXD3	17.47930887	26.48708424	36.43704044	41.77483365	51.44926138	45.05084309	26.37064736	32.22313879	26.32715172	20.42633363	22.7718211	30.88640905
ENSMUSG00000021486	PRELID1	122.3551621	66.21771059	131.1733456	149.1958345	128.6231535	102.9733556	125.260575	131.4704063	112.3291807	116.5502566	117.3624626	118.9126749
ENSMUSG00000021488	NSD1	166.0534342	211.8966739	109.3111213	208.8741682	173.6412572	135.1525293	97.57139523	78.62445865	49.14401654	106.9378643	82.32889166	97.29218852
ENSMUSG00000021493	PDLIM7	17.47930887	26.48708424	29.14963235	11.93566676	32.15578836	32.17917364	19.77798552	15.46710662	14.04114758	22.8294317	21.02014255	18.53184543
ENSMUSG00000021494	DDX41	52.4379266	59.59593953	58.2992647	53.7105004	64.31157673	25.74333891	56.69689182	70.89090534	54.40944688	81.70533452	45.5436422	49.41825449
ENSMUSG00000021495	FAM193B	113.6155076	139.0571922	153.0355698	83.54966729	83.60504975	135.1525293	60.65248893	82.49123531	80.7365986	78.10068741	87.5839273	74.12738173
ENSMUSG00000021496	PCBD2	8.739654434	6.621771059	0	5.967833378	32.15578836	25.74333891	29.0077121	25.77851103	22.81686482	20.42633363	14.01342837	26.2534477
ENSMUSG00000021497	TXNDC15	52.4379266	26.48708424	65.58667279	29.83916689	45.01810371	32.17917364	42.19303578	30.93421324	40.3682993	28.83717689	24.52349964	40.15233177
ENSMUSG00000021499	CATSPER3	0	0	0	0	0	0	1.318532368	0	0	0	0	0
ENSMUSG00000021500	DDX46	358.3258318	390.6844925	218.6222426	262.5846686	257.2463069	244.5617197	296.6697828	265.5186636	312.4155337	240.3098074	297.7853528	281.0663224
ENSMUSG00000021501	CAML	26.2189633	13.24354212	0	65.64616716	25.72463069	6.435834728	21.09651789	21.91173438	26.32715172	18.02323556	19.26846401	21.62048634
ENSMUSG00000021504	B4GALT7	17.47930887	66.21771059	21.86222426	0	19.29347302	32.17917364	35.60037394	41.24561765	47.38887309	26.43407881	42.0402851	35.51937041
ENSMUSG00000021510	ZFP729A	26.2189633	0	0	35.80700027	6.431157673	6.435834728	10.54825894	10.31140441	1.755143448	13.21703941	15.76510691	15.44320453
ENSMUSG00000021514	ZFP369	26.2189633	19.86531318	21.86222426	23.87133351	19.29347302	12.87166946	26.37064736	20.62280883	24.57200827	18.02323556	21.02014255	26.2534477
ENSMUSG00000021518	PTDSS1	34.95861774	19.86531318	21.86222426	29.83916689	38.58694604	0	7.911194208	16.75603217	21.06172137	20.42633363	19.26846401	29.3420886
ENSMUSG00000021519	MTERF3	17.47930887	6.621771059	58.2992647	5.967833378	25.72463069	6.435834728	9.229726576	23.20065993	26.32715172	21.62788267	42.0402851	16.98752498
ENSMUSG00000021520	UQCRB	192.2723975	158.9225054	153.0355698	161.1315012	231.5216762	102.9733556	283.4844591	284.8525469	259.7612303	227.092768	308.2954241	284.1549633
ENSMUSG00000021532	FASTKD3	8.739654434	13.24354212	14.57481618	5.967833378	25.72463069	19.30750418	31.64477683	27.06743658	29.83743861	31.24027496	43.79196365	27.79776815
ENSMUSG00000021537	CETN3	183.5327431	125.8136501	204.0474265	167.0993346	135.0543111	173.7675376	245.2470204	215.2505671	210.6172137	209.0695324	229.4698895	188.4070952
ENSMUSG00000021540	SMAD5	26.2189633	33.10885529	36.43704044	35.80700027	38.58694604	51.48667782	27.68917973	24.48958548	26.32715172	20.42633363	24.52349964	29.3420886
ENSMUSG00000021546	HNRNPK	996.3206055	1105.835767	947.3630514	1056.306508	1247.644589	907.4526966	1416.103763	1540.266034	1484.851357	1254.417195	1431.121372	1460.927148
ENSMUSG00000021548	CCNH	52.4379266	46.35239741	14.57481618	89.51750067	57.88041905	32.17917364	67.24515077	74.757682	75.47116826	67.28674607	59.55707056	46.32961358
ENSMUSG00000021549	RASA1	43.69827217	59.59593953	87.44889705	89.51750067	102.8985228	102.9733556	80.43047445	63.15735203	89.51231584	76.89913837	56.05371347	67.95009992
ENSMUSG00000021550	2210016F16R	34.95861774	13.24354212	21.86222426	41.77483365	19.29347302	32.17917364	26.37064736	41.24561765	24.57200827	27.63562785	38.53692801	37.06369086
ENSMUSG00000021555	NAA35	78.65688991	86.08302377	65.58667279	101.4531674	90.03620742	90.10168619	50.10422998	81.20230975	70.20573791	84.10843259	98.09399857	84.9376249
ENSMUSG00000021556	GOLM1	122.3551621	79.46125271	87.44889705	77.58183392	64.31157673	51.48667782	72.51928024	72.17983089	94.77774619	69.68984415	61.30874911	67.95009992
ENSMUSG00000021557	AGTPBP1	34.95861774	79.46125271	51.01185661	53.7105004	57.88041905	45.05084309	64.60808603	47.69024541	35.10286896	49.26351052	68.31546329	40.15233177
ENSMUSG00000021565	SLC6A19	78.65688991	26.48708424	123.8859375	83.54966729	90.03620742	83.66585146	90.97873339	144.3596618	114.0843241	117.7518056	103.3490342	109.6467521
ENSMUSG00000021567	NKD2	0	0	7.287408088	0	0	0	0	0	0	0	0	0
ENSMUSG00000021569	TRIP13	69.91723547	46.35239741	36.43704044	11.93566676	32.15578836	45.05084309	27.68917973	34.80098989	49.14401654	32.441824	54.30203492	38.60801132
ENSMUSG00000021572	CEP72	0	13.24354212	7.287408088	0	0	12.87166946	14.50385605	6.444627758	17.55143448	14.41858844	10.51007128	7.721602263
ENSMUSG00000021573	TPPP	0	0	0	0	0	0	0	1.288925552	0	0	3.503357092	0
ENSMUSG00000021575	AHRR	0	0	0	0	0	0	0	1.288925552	0	0	0	1.544320453
ENSMUSG00000021576	PDCD6	78.65688991	86.08302377	116.5985294	59.67833378	96.46736509	83.66585146	81.74900682	110.8475974	93.02260274	109.3409624	115.610784	94.20354761
ENSMUSG00000021577	SDHA	52.4379266	99.32656588	58.2992647	83.54966729	83.60504975	115.8450251	109.4381865	90.22478862	103.5534634	84.10843259	85.83224875	109.6467521
ENSMUSG00000021578	CCDC127	87.39654434	119.1918791	72.87408088	83.54966729	70.7427344	96.53752092	84.38607155	103.1140441	70.20573791	62.48054992	91.08728439	108.1024317
ENSMUSG00000021579	LRRC14B	0	6.621771059	7.287408088	5.967833378	0	6.435834728	5.274129472	3.866776655	5.265430344	1.201549037	7.006714184	3.088640905
ENSMUSG00000021583	ERAP1	17.47930887	52.97416847	58.2992647	83.54966729	19.29347302	83.66585146	84.38607155	64.44627758	93.02260274	78.10068741	77.07385602	77.21602263
ENSMUSG00000021585	CAST	148.5741254	112.570108	102.0237132	113.3888342	109.3296804	186.6392071	123.9420426	106.9808208	131.6357586	140.5812373	124.3691768	98.83650897

ENSMUSG00000021589	RHOBTB3	0	6.621771059	7.287408088	0	0	0	3.955597104	5.155702207	8.775717239	3.604647111	12.26174982	4.632961358
ENSMUSG00000021591	GLRX	8.739654434	0	0	0	6.431157673	0	2.637064736	7.73355331	5.265430344	7.209294222	10.51007128	6.177281811
ENSMUSG00000021592	ARSK	8.739654434	52.97416847	7.287408088	29.83916689	6.431157673	6.435834728	11.86679131	11.60032996	12.28600414	13.21703941	8.75839273	4.632961358
ENSMUSG00000021594	SRD5A1	17.47930887	13.24354212	0	17.90350013	0	12.87166946	3.955597104	3.866776655	3.510286896	4.806196148	5.255035638	7.721602263
ENSMUSG00000021595	NSUN2	131.0948165	105.9483369	145.7481618	137.2601677	115.7608381	102.9733556	171.4092078	140.4928851	184.290062	133.3719431	164.6577833	151.3434044
ENSMUSG00000021596	MCTP1	52.4379266	33.10885529	80.16148896	17.90350013	12.86231535	45.05084309	23.73358262	41.24561765	40.3682993	45.65886341	31.53021383	44.78529313
ENSMUSG00000021597	SLF1	61.17758104	86.08302377	58.2992647	71.61400054	64.31157673	83.66585146	44.83010051	45.11239431	40.3682993	54.06970667	52.55035638	44.78529313
ENSMUSG00000021598	MED10	26.2189633	33.10885529	14.57481618	29.83916689	32.15578836	45.05084309	51.42276235	47.69024541	56.16459033	50.46505955	50.79867783	40.15233177
ENSMUSG00000021606	NDUFS6	192.2723975	132.4354212	138.4607537	125.3245009	147.9166265	135.1525293	208.3281141	244.8958548	235.189222	194.650944	255.7450677	217.7491838
ENSMUSG00000021607	MRPL36	78.65688991	19.86531318	43.72444853	23.87133351	25.72463069	38.61500837	51.42276235	58.00164982	47.38887309	52.86815763	63.06042765	52.50689539
ENSMUSG00000021608	LPCAT1	43.69827217	52.97416847	72.87408088	59.67833378	38.58694604	38.61500837	50.10422998	74.757682	57.91973378	49.26351052	70.06714184	46.32961358
ENSMUSG00000021610	CLPTM1L	96.13619877	59.59593953	94.73630514	77.58183392	83.60504975	45.05084309	63.28955366	68.31305424	73.71602481	75.69758933	87.5839273	74.12738173
ENSMUSG00000021611	TERT	17.47930887	26.48708424	7.287408088	23.87133351	12.86231535	6.435834728	18.45945315	12.88925552	5.265430344	13.21703941	10.51007128	16.98752498
ENSMUSG00000021614	VCAN	0	0	0	0	6.431157673	0	1.318532368	2.577851103	1.755143448	0	1.751678546	7.721602263
ENSMUSG00000021615	XRCC4	8.739654434	0	7.287408088	5.967833378	6.431157673	6.435834728	7.911194208	6.444627758	5.265430344	9.612392296	5.255035638	9.265922716
ENSMUSG00000021619	ATG10	17.47930887	6.621771059	7.287408088	11.93566676	0	6.435834728	3.955597104	5.155702207	8.775717239	2.403098074	1.751678546	1.544320453
ENSMUSG00000021621	ZCCHC9	69.91723547	6.621771059	43.72444853	41.77483365	25.72463069	32.17917364	35.60037394	32.22313879	43.8785862	50.46505955	42.0402851	44.78529313
ENSMUSG00000021629	SLC30A5	26.2189633	46.35239741	43.72444853	41.77483365	64.31157673	32.17917364	54.05982709	68.31305424	75.47116826	70.89139318	61.30874911	61.77281811
ENSMUSG00000021635	RAD17	8.739654434	46.35239741	29.14963235	35.80700027	32.15578836	38.61500837	38.23743867	52.84594762	42.12344275	32.441824	64.8121062	46.32961358
ENSMUSG00000021636	MARVELD2	17.47930887	13.24354212	7.287408088	11.93566676	6.431157673	0	11.86679131	7.73355331	14.04114758	8.410843259	15.76510691	15.44320453
ENSMUSG00000021638	OCLN	0	0	0	0	12.86231535	0	0	0	0	0	0	1.544320453
ENSMUSG00000021639	GTF2H2	43.69827217	19.86531318	51.01185661	65.64616716	32.15578836	70.79418201	42.19303578	50.26809651	59.67487723	55.2712557	42.0402851	38.60801132
ENSMUSG00000021643	SERF1	69.91723547	46.35239741	36.43704044	71.61400054	77.17389207	57.92251255	131.8532368	81.20230975	82.49174205	91.31772681	94.59064148	108.1024317
ENSMUSG00000021645	SMN1	96.13619877	52.97416847	58.2992647	65.64616716	38.58694604	45.05084309	69.8822155	94.09156527	64.94030757	73.29449126	85.83224875	64.86145901
ENSMUSG00000021646	MCCC2	26.2189633	19.86531318	21.86222426	5.967833378	25.72463069	19.30750418	31.64477683	32.22313879	26.32715172	24.03098074	26.27517819	21.62048634
ENSMUSG00000021647	CARTPT	8.739654434	0	0	0	0	0	0	0	3.510286896	0	1.751678546	0
ENSMUSG00000021650	PTCD2	0	13.24354212	36.43704044	17.90350013	19.29347302	32.17917364	15.82238842	23.20065993	26.32715172	25.23252978	21.02014255	16.98752498
ENSMUSG00000021660	BTF3	8.739654434	6.621771059	7.287408088	5.967833378	6.431157673	6.435834728	38.23743867	38.66776655	31.59258206	28.83717689	29.77853528	44.78529313
ENSMUSG00000021661	ANKRA2	8.739654434	19.86531318	7.287408088	5.967833378	32.15578836	12.87166946	23.73358262	18.04495772	15.79629103	16.82168652	17.51678546	16.98752498
ENSMUSG00000021665	HEXB	8.739654434	19.86531318	7.287408088	29.83916689	45.01810371	19.30750418	17.14092078	16.75603217	17.55143448	16.82168652	21.02014255	12.35456362
ENSMUSG00000021666	GFM2	43.69827217	26.48708424	14.57481618	17.90350013	38.58694604	19.30750418	22.41505026	33.51206434	24.57200827	20.42633363	22.7718211	26.2534477
ENSMUSG00000021668	POLK	26.2189633	19.86531318	0	11.93566676	19.29347302	25.74333891	29.0077121	19.33388327	40.3682993	27.63562785	26.27517819	21.62048634
ENSMUSG00000021669	COL4A3BP	26.2189633	33.10885529	58.2992647	35.80700027	64.31157673	32.17917364	32.9633092	46.40131986	38.61315585	38.44956918	33.28189237	38.60801132
ENSMUSG00000021670	HMGCR	375.8051407	364.1974082	386.2326286	364.0378361	379.4383027	308.9200669	429.841552	448.546092	473.8887309	440.9684966	425.6578867	393.8017154
ENSMUSG00000021671	POC5	0	13.24354212	21.86222426	11.93566676	12.86231535	6.435834728	22.41505026	19.33388327	26.32715172	22.8294317	31.53021383	24.70912724
ENSMUSG00000021675	F2RL2	0	0	0	0	0	0	0	0	1.755143448	0	0	0
ENSMUSG00000021676	IQGAP2	122.3551621	112.570108	80.16148896	131.2923343	135.0543111	115.8450251	112.0752513	126.3147041	73.71602481	108.1394133	115.610784	89.57058625
ENSMUSG00000021680	CRHBP	0	0	0	0	6.431157673	0	2.637064736	3.866776655	1.755143448	1.201549037	0	0
ENSMUSG00000021681	AGGF1	209.7517064	99.32656588	182.1852022	173.067168	128.6231535	115.8450251	97.57139523	108.2697463	98.28803308	112.9456095	106.8523913	117.3683544
ENSMUSG00000021684	PDE8B	0	0	0	5.967833378	0	0	1.318532368	2.577851103	0	1.201549037	0	0
ENSMUSG00000021686	AP3B1	52.4379266	66.21771059	72.87408088	89.51750067	83.60504975	70.79418201	79.11194208	72.17983089	73.71602481	87.7130797	82.32889166	74.12738173
ENSMUSG00000021687	SCAMP1	8.739654434	19.86531318	51.01185661	47.74266702	45.01810371	38.61500837	36.9189063	32.22313879	43.8785862	39.65111822	31.53021383	33.97504996
ENSMUSG00000021690	JMY	61.17758104	33.10885529	43.72444853	83.54966729	70.7427344	83.66585146	46.14863288	50.26809651	54.40944688	40.85266726	52.55035638	40.15233177
ENSMUSG00000021692	DIMT1	17.47930887	33.10885529	21.86222426	5.967833378	19.29347302	12.87166946	23.73358262	25.77851103	35.10286896	25.23252978	21.02014255	16.98752498
ENSMUSG00000021693	KIF2A	148.5741254	139.0571922	174.8977941	143.2280011	102.8985228	115.8450251	138.4458986	145.6485873	126.3703282	110.5425114	161.1544262	120.4569953

ENSMUSG00000021694	ERCC8	0	13.24354212	0	11.93566676	19.29347302	6.435834728	10.54825894	11.60032996	8.775717239	2.403098074	8.75839273	16.98752498
ENSMUSG00000021696	ELOVL7	0	0	0	0	6.431157673	0	0	0	1.755143448	1.201549037	0	1.544320453
ENSMUSG00000021697	DEPDC1B	8.739654434	0	0	0	0	0	2.637064736	3.866776655	0	3.604647111	1.751678546	1.544320453
ENSMUSG00000021699	PDE4D	8.739654434	13.24354212	14.57481618	35.80700027	0	32.17917364	10.54825894	11.60032996	12.28600414	13.21703941	8.75839273	7.721602263
ENSMUSG00000021702	THBS4	0	0	0	0	0	0	0	1.288925552	0	0	0	0
ENSMUSG00000021703	SERINC5	0	0	0	0	0	0	1.318532368	0	0	0	0	0
ENSMUSG00000021704	MTX3	26.2189633	0	0	11.93566676	19.29347302	6.435834728	6.59266184	11.60032996	14.04114758	7.209294222	10.51007128	10.81024317
ENSMUSG00000021706	ZFYVE16	0	0	14.57481618	0	0	25.74333891	0	12.88925552	8.775717239	19.22478459	17.51678546	4.632961358
ENSMUSG00000021707	DHFR	209.7517064	165.5442765	211.3348345	196.9385015	173.6412572	193.0750418	193.8242581	235.873376	215.8826441	194.650944	196.1879971	208.4832611
ENSMUSG00000021709	ERBIN	122.3551621	99.32656588	72.87408088	59.67833378	90.03620742	64.35834728	108.1196542	87.64693751	66.69545102	99.72857007	92.83896294	75.67170218
ENSMUSG00000021710	NLN	17.47930887	26.48708424	14.57481618	17.90350013	25.72463069	38.61500837	30.32624446	39.9566921	22.81686482	30.03872593	36.78524947	30.88640905
ENSMUSG00000021711	TRAPPC13	69.91723547	33.10885529	43.72444853	65.64616716	51.44926138	32.17917364	92.29726576	86.35801196	71.96088136	91.31772681	91.08728439	69.49442037
ENSMUSG00000021712	TRIM23	26.2189633	33.10885529	58.2992647	41.77483365	19.29347302	25.74333891	43.51156814	42.5345432	38.61315585	32.441824	43.79196365	33.97504996
ENSMUSG00000021713	PPWD1	375.8051407	245.0055292	298.7837316	226.7776684	199.3658879	270.3050586	230.7431644	244.8958548	261.5163737	245.1160036	262.7517819	305.7754496
ENSMUSG00000021714	CENPK	139.8344709	86.08302377	102.0237132	131.2923343	109.3296804	148.0241987	116.0308484	119.8700763	112.3291807	97.325472	105.1007128	83.39330444
ENSMUSG00000021715	CWC27	26.2189633	52.97416847	43.72444853	29.83916689	12.86231535	32.17917364	50.10422998	46.40131986	52.65430344	42.0542163	33.28189237	46.32961358
ENSMUSG00000021716	SREK1IP1	157.3137798	218.5184449	153.0355698	202.9063349	167.2100995	199.5108766	121.3049779	85.06908641	87.75717239	116.5502566	101.5973557	94.20354761
ENSMUSG00000021718	4933425L06R	0	0	0	0	0	6.435834728	2.637064736	1.288925552	0	2.403098074	1.751678546	1.544320453
ENSMUSG00000021725	PARP8	8.739654434	26.48708424	36.43704044	23.87133351	45.01810371	38.61500837	19.77798552	30.93421324	21.06172137	14.41858844	17.51678546	9.265922716
ENSMUSG00000021728	EMB	139.8344709	99.32656588	153.0355698	190.9706681	205.7970455	128.7166946	121.3049779	164.9824706	154.4526234	147.7905316	155.8993906	111.1910726
ENSMUSG00000021730	HCN1	0	0	0	0	0	0	0	1.288925552	1.755143448	0	0	0
ENSMUSG00000021731	MRPS30	34.95861774	46.35239741	29.14963235	53.7105004	32.15578836	45.05084309	46.14863288	52.84594762	57.91973378	50.46505955	54.30203492	41.69665222
ENSMUSG00000021733	SLC4A7	52.4379266	46.35239741	36.43704044	71.61400054	77.17389207	51.48667782	50.10422998	55.42379872	59.67487723	44.45731437	47.29532074	20.07616588
ENSMUSG00000021737	PSMD6	52.4379266	125.8136501	109.3111213	71.61400054	122.1919958	102.9733556	129.2161721	127.6036296	138.6563324	123.7595508	113.8591055	123.5456362
ENSMUSG00000021738	ATXN7	34.95861774	26.48708424	14.57481618	65.64616716	12.86231535	0	22.41505026	21.91173438	47.38887309	26.43407881	19.26846401	20.07616588
ENSMUSG00000021745	PTPRG	0	0	0	0	0	0	0	0	1.755143448	0	0	0
ENSMUSG00000021748	PDHB	157.3137798	66.21771059	123.8859375	107.4210008	109.3296804	109.4091904	133.1717692	125.0257785	136.9011889	122.5580018	124.3691768	128.1785976
ENSMUSG00000021752	KCTD6	8.739654434	13.24354212	0	5.967833378	6.431157673	0	9.229726576	9.022478862	14.04114758	8.410843259	7.006714184	7.721602263
ENSMUSG00000021754	MAP3K1	253.4499786	112.570108	182.1852022	214.8420016	192.9347302	193.0750418	112.0752513	108.2697463	136.9011889	128.565747	140.1342837	134.3558794
ENSMUSG00000021756	IL6ST	17.47930887	39.73062635	21.86222426	17.90350013	45.01810371	32.17917364	25.05211499	28.35636214	22.81686482	33.64337304	19.26846401	20.07616588
ENSMUSG00000021759	PLPP1	43.69827217	92.70479482	65.58667279	47.74266702	57.88041905	38.61500837	44.83010051	52.84594762	49.14401654	38.44956918	49.04699929	47.87393403
ENSMUSG00000021764	NDUFS4	61.17758104	125.8136501	87.44889705	101.4531674	109.3296804	109.4091904	154.2682871	137.915034	129.8806151	128.565747	129.6242124	122.0013158
ENSMUSG00000021767	KAT6B	279.6689419	264.8708424	298.7837316	310.3273357	289.4020953	321.7917364	323.0404302	313.2089091	275.5575213	332.8290833	255.7450677	327.395936
ENSMUSG00000021770	SAMD8	174.7930887	132.4354212	174.8977941	155.1636678	154.3477841	90.10168619	83.06753918	83.78016086	80.7365986	87.7130797	113.8591055	94.20354761
ENSMUSG00000021771	VDAC2	235.9706697	225.140216	153.0355698	196.9385015	231.5216762	186.6392071	311.1736388	333.8317179	328.2118248	336.4337304	360.8457805	285.6992837
ENSMUSG00000021772	NKIRAS1	8.739654434	46.35239741	29.14963235	35.80700027	38.58694604	19.30750418	39.55597104	34.80098989	40.3682993	27.63562785	54.30203492	35.51937041
ENSMUSG00000021773	COMTD1	69.91723547	6.621771059	29.14963235	41.77483365	45.01810371	19.30750418	52.74129472	51.55702207	50.89915999	52.86815763	78.82553457	63.31713856
ENSMUSG00000021774	UBE2E1	131.0948165	66.21771059	131.1733456	83.54966729	64.31157673	25.74333891	147.6756252	134.0482574	122.8600414	129.767296	138.3826051	131.2672385
ENSMUSG00000021775	NR1D2	8.739654434	6.621771059	7.287408088	17.90350013	12.86231535	6.435834728	6.59266184	19.33388327	5.265430344	6.007745185	14.01342837	16.98752498
ENSMUSG00000021779	THRB	0	6.621771059	0	0	0	0	1.318532368	0	1.755143448	2.403098074	0	1.544320453
ENSMUSG00000021785	NGLY1	34.95861774	26.48708424	21.86222426	17.90350013	32.15578836	6.435834728	10.54825894	16.75603217	17.55143448	18.02323556	15.76510691	20.07616588
ENSMUSG00000021786	OXSM	8.739654434	26.48708424	7.287408088	17.90350013	6.431157673	12.87166946	14.50385605	6.444627758	7.020573791	10.81394133	12.26174982	15.44320453
ENSMUSG00000021792	FAM213A	8.739654434	0	0	0	0	0	0	0	0	0	0	0
ENSMUSG00000021794	GLUD1	69.91723547	79.46125271	80.16148896	95.48533405	96.46736509	45.05084309	97.57139523	103.1140441	103.5534634	87.7130797	96.34232003	100.3808294
ENSMUSG00000021806	NID2	0	0	0	0	0	0	2.637064736	1.288925552	1.755143448	1.201549037	0	1.544320453

ENSMUSG00000021807	RTRAF	131.0948165	139.0571922	87.44889705	143.2280011	70.7427344	102.9733556	137.1273663	143.0707362	112.3291807	110.5425114	155.8993906	137.4445203
ENSMUSG00000021809	NUDT13	17.47930887	6.621771059	14.57481618	29.83916689	12.86231535	6.435834728	15.82238842	23.20065993	19.30657793	13.21703941	14.01342837	30.88640905
ENSMUSG00000021810	ECD	34.95861774	59.59593953	51.01185661	59.67833378	51.44926138	38.61500837	80.43047445	61.86842648	80.7365986	85.30998163	61.30874911	69.49442037
ENSMUSG00000021811	DNAJC9	104.8758532	145.6789633	65.58667279	131.2923343	90.03620742	102.9733556	156.9053518	158.5378429	159.7180538	141.7827864	176.9195331	157.5206862
ENSMUSG00000021814	ANXA7	157.3137798	119.1918791	145.7481618	89.51750067	154.3477841	128.7166946	98.8899276	101.8251186	135.1460455	112.9456095	127.8725339	134.3558794
ENSMUSG00000021815	MSS51	0	6.621771059	7.287408088	17.90350013	6.431157673	0	5.274129472	9.022478862	12.28600414	9.612392296	1.751678546	16.98752498
ENSMUSG00000021816	PPP3CB	113.6155076	79.46125271	65.58667279	89.51750067	102.8985228	96.53752092	72.51928024	94.09156527	96.53288963	91.31772681	82.32889166	80.30466354
ENSMUSG00000021819	ZSWIM8	43.69827217	33.10885529	29.14963235	41.77483365	19.29347302	25.74333891	29.0077121	34.80098989	22.81686482	37.24802015	47.29532074	49.41825449
ENSMUSG00000021820	CAMK2G	227.2310153	218.5184449	211.3348345	220.809835	109.3296804	148.0241987	148.9941576	177.8717261	156.2077669	163.410669	134.879248	129.722918
ENSMUSG00000021823	VCL	8.739654434	6.621771059	14.57481618	11.93566676	32.15578836	0	13.18532368	20.62280883	28.08229517	13.21703941	15.76510691	21.62048634
ENSMUSG00000021824	AP3M1	43.69827217	6.621771059	36.43704044	47.74266702	32.15578836	25.74333891	30.32624446	54.13487317	38.61315585	33.64337304	43.79196365	26.2534477
ENSMUSG00000021830	TXNDC16	34.95861774	33.10885529	29.14963235	11.93566676	51.44926138	25.74333891	46.14863288	32.22313879	38.61315585	33.64337304	38.53692801	30.88640905
ENSMUSG00000021831	ERO1L	113.6155076	92.70479482	131.1733456	125.3245009	122.1919958	77.23001673	96.25286286	100.536193	77.22631171	108.1394133	98.09399857	103.4694703
ENSMUSG00000021832	PSMC6	305.8879052	231.7619871	233.1970588	310.3273357	282.9709376	283.176728	398.1967751	429.2122087	407.1932799	396.5111822	404.6377441	318.1300133
ENSMUSG00000021840	MAPK1IP1L	78.65688991	79.46125271	102.0237132	95.48533405	51.44926138	83.66585146	73.83781261	79.9133842	91.26745929	86.51153067	84.08057021	71.03874082
ENSMUSG00000021843	KTN1	358.3258318	430.4151188	429.9570772	358.0700027	347.2825143	334.6634058	259.7508765	256.4961848	296.6192427	317.2089458	268.0068175	225.4707861
ENSMUSG00000021846	PELI2	0	0	0	0	0	0	0	1.288925552	0	0	0	0
ENSMUSG00000021852	SLC35F4	0	6.621771059	0	0	0	0	0	0	0	0	0	0
ENSMUSG00000021868	PPIF	61.17758104	52.97416847	80.16148896	53.7105004	64.31157673	32.17917364	55.37835946	64.44627758	68.45059447	43.25576533	50.79867783	58.6841772
ENSMUSG00000021870	SLMAP	69.91723547	86.08302377	116.5985294	119.3566676	77.17389207	102.9733556	85.70460392	106.9808208	93.02260274	96.12392296	64.8121062	67.95009992
ENSMUSG00000021876	RNASE4	17.47930887	13.24354212	7.287408088	0	0	0	1.318532368	0	0	1.201549037	0	1.544320453
ENSMUSG00000021877	ARF4	104.8758532	132.4354212	80.16148896	196.9385015	135.0543111	109.4091904	168.7721431	163.6935451	168.493771	159.8060219	148.8926764	174.5082112
ENSMUSG00000021879	DNAH12	0	0	0	5.967833378	0	0	0	1.288925552	0	0	0	0
ENSMUSG00000021884	HACL1	0	6.621771059	7.287408088	0	0	0	1.318532368	1.288925552	3.510286896	1.201549037	1.751678546	1.544320453
ENSMUSG00000021886	GPR65	148.5741254	132.4354212	116.5985294	125.3245009	141.4854688	115.8450251	72.51928024	88.93586306	77.22631171	74.4960403	84.08057021	83.39330444
ENSMUSG00000021890	EAF1	17.47930887	26.48708424	14.57481618	17.90350013	38.58694604	32.17917364	46.14863288	43.82346876	49.14401654	55.2712557	36.78524947	40.15233177
ENSMUSG00000021891	METTL6	61.17758104	26.48708424	80.16148896	29.83916689	70.7427344	32.17917364	48.78569762	65.73520313	61.43002068	55.2712557	64.8121062	67.95009992
ENSMUSG00000021892	SH3BP5	0	0	0	0	0	0	5.274129472	3.866776655	5.265430344	3.604647111	1.751678546	6.177281811
[truncated: 2,411,448 more chars]
